# Transcriptome of rhesus macaque (*Macaca mulatta*) exposed to total-body irradiation

**DOI:** 10.1038/s41598-021-85669-6

**Published:** 2021-03-18

**Authors:** Yaoxiang Li, Jatinder Singh, Rency Varghese, Yubo Zhang, Oluseyi O. Fatanmi, Amrita K. Cheema, Vijay K. Singh

**Affiliations:** 1 Department of Oncology, Lombardi Comprehensive Cancer Center, Georgetown University Medical Center, Washington, DC USA; 2 Division of Radioprotectants, Department of Pharmacology and Molecular Therapeutics, F. Edward Hébert School of Medicine “America’s Medical School”, Uniformed Serices University of the Health Sciences, 4301 Jones Bridge Road, Bethesda, MD USA; 3 Armed Forces Radiobiology Research Institute, Uniformed Services University of the Health Sciences, Bethesda, MD USA; 4 Department of Biochemistry and Molecular Medicine, George Washington University, Washington, DC USA; 5 Department of Biochemistry, Molecular and Cellular Biology, Georgetown University Medical Center, Washington, DC USA

**Keywords:** Transcriptomics, Diagnostic markers

## Abstract

The field of biodosimetry has seen a paradigm shift towards an increased use of molecular phenotyping technologies including omics and miRNA, in addition to conventional cytogenetic techniques. Here, we have used a nonhuman primate (NHP) model to study the impact of gamma-irradiation on alterations in blood-based gene expression. With a goal to delineate radiation induced changes in gene expression, we followed eight NHPs for 60 days after exposure to 6.5 Gy gamma-radiation for survival outcomes. Analysis of differential gene expression in response to radiation exposure yielded 26,944 dysregulated genes that were not significantly impacted by sex. Further analysis showed an increased association of several pathways including IL-3 signaling, ephrin receptor signaling, ErbB signaling, nitric oxide signaling in the cardiovascular system, Wnt/β-catenin signaling, and inflammasome pathway, which were associated with positive survival outcomes in NHPs after acute exposure to radiation. This study provides novel insights into major pathways and networks involved in radiation-induced injuries that may identify biomarkers for radiation injury.

## Introduction

The possible detonation of a radiological dispersal device or improvised nuclear device, accidental exposure to a radiation source, or nuclear accidents have led to the urgent need to develop essential analytic tools to assess such radiation exposures, especially radiation doses to exposed individuals. Biomarkers are important to assess the absorbed dose of radiation after a radiological or nuclear accident, or after the deliberate use of a radiation source to expose individuals^1–4^. Identification of radiation injury biomarkers, or the objective features that can be precisely assessed to distinguish a specific biological, pathological, or therapeutic development of the host, is an important area of investigation in radiation biology^4,5^. The nuclear reactor accidents of Fukushima-Daiichi and Chernobyl are reminders that natural disasters or human error can cause lasting effects on human health and the environment^6^. The bombings of Hiroshima and Nagasaki demonstrated the catastrophic effects of nuclear weapons used in times of war. In recent years, a large number of studies have implemented different strategies to identify and validate biomarkers for both total- and partial-body irradiation using various animal models^3–5^.

In addition to identifying radiation injury, biomarkers are also needed for radiation countermeasure development since these agents are developed following the United States Food and Drug Administration (US FDA) ‘Animal Rule’, where efficacy testing is conducted in well-controlled large animal models rather than in Phase II and Phase III clinical trials due to ethical reasons^7^. Specifically, the human dose of a radiation countermeasure needs to be obtained from animal studies based on biomarkers, as biomarkers are needed for drug dose conversion from animals to humans. For the last few years, various omic platforms are being used for the identification of such biomarkers. Furthermore, biomarkers are advantageous in understanding the mechanism of countermeasure efficacy. Several biomarkers have been approved for various injuries by regulatory bodies around the world, but none of these approved biomarkers are for radiation-induced injuries. Multiple molecular pathways and potential biomarkers for radiation injury are being identified and validated by a large number of investigators^4,8–13^.

In this study, we used a transcriptomic approach to evaluate whole blood samples collected from nonhuman primates (NHPs) exposed to a single dose of ionizing radiation (cobalt-60 gamma, 6.5 Gy, 0.6 Gy/min) leading to acute radiation syndrome. Samples were collected 7 days before radiation exposure (C) in addition to 1 (SD1), 2 (SD2) 3 (SD3), 35 (SD35), and 60 (SD60) days post-irradiation. The samples collected during the first few days post-exposure were compared with the baseline pre-irradiation samples (C) for an understanding of the injuries induced by ionizing radiation.

## Results

In an effort to understand the impact of ionizing radiation on gene expression and mRNA diversity, a total of 8 NHPs (5 males and 3 females) were exposed to a bilateral midline dose of 6.5 Gy ^60^Co γ-radiation at a dose rate of 0.6 Gy/min. All eight NHPs developed radiation-induced stress, weakness, and other symptoms of acute radiation syndrome (ARS) around day 10 post-irradiation^14,15^. Clinical observations of moribundity included animals having in appetence, slow to respond to stimuli, lethargy, and inability to obtain feed or water. Respiratory distress also occurred in some of the animals; the skin appeared to be pale, redness was noted in some parts, and non-healing wounds resulting in cyanosis in some parts of the skin. Mouth ulcers from bleeding gums also showed up in some animals. Most of the animals presented with sustained vomiting and diarrhea. All above-listed deteriorating conditions and distress resulted in significant weight loss in most of the animals. Complete blood profiles in moribund animals revealed severe anemia (low hemoglobin and hematocrit), thrombocytopenia, and neutropenia. Four animals out of eight survived at 60 days’ post-irradiation, which is considered the endpoint for ARS survival studies for NHPs. The death of these animals was due to ARS, which leads to multi-organ failure and sepsis.

Anatomical observations during necropsy of euthanized moribund animals showed, in general, petechiae, redness, bruising, and hemorrhage in most of the organs. Adhesions between the lobes of the lungs, pericardial fluid in copious amounts in the pericardium of the heart, pale and shriveled kidneys, enlarged liver with atypical gall bladders, shrunken or wrinkled spleens, and/or distended stomachs were noticed in most of the euthanized animals. Overall, the gastrointestinal tract of the four euthanized NHPs presented with redness, intussusceptions, and hemorrhage. Histopathological examination of various organs demonstrated typical features of damage as a result of irradiation.

**Figure 1:**
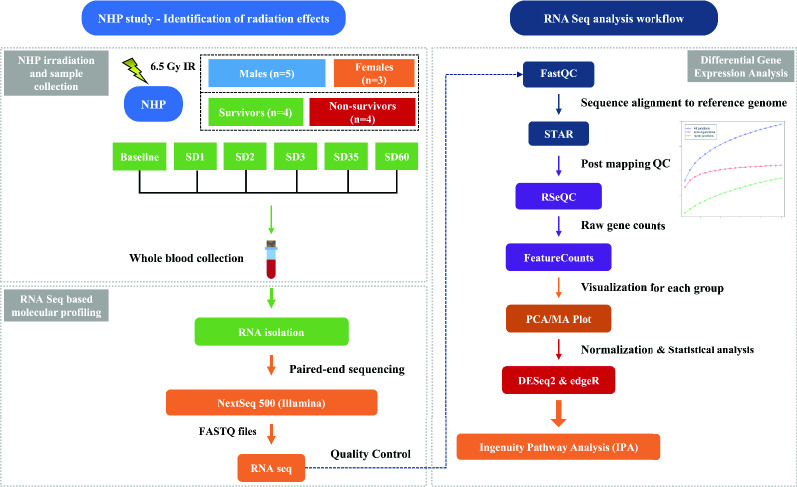
Experimental and data analysis workflow. Longitudinally collected blood samples including: C (pre-irradiation), SD1 (day 1 post-irradiation), SD2 (day 2), SD3 (day 3), SD35 (day 35), SD60 (day 60) were collected from 7 NHPs for NextSeq 500 paired-end sequencing analyses and downstream bioinformatics analyses. Differential gene expression and functional pathway analysis were performed for gaining insight into radiation response in the survivors and non-survivors. Figure is created by the author using Microsoft PowerPoint (https://www.microsoft.com/en-us/microsoft-365/powerpoint).

Whole blood samples were collected from the eight NHPs at 7 days prior to irradiation (baseline) and subsequently at 1, 2, 3, 35 and 60 days’ post-irradiation, as shown in Supplementary Table S1. Total RNA was isolated from the samples and was subjected to whole transcriptome sequencing (paired-end sequencing) using NextSeq 500 platform (Illumina). The overall experimental design is shown in Fig. 1. Sequencing reads from all the samples were subjected to quality checks pre- and post-mapping. The number of reads per sample ranged from 50 to 78 million, underscoring the high quality of data. The average data quality based on reads/sample yielded a Phred score of 35 indicating the likelihood of an error in base calling of < 1/2500. This is much above a minimal requirement of > 1/1000 for accurate base calling, thus underscoring the excellent quality of base calls. The globin and ribosome comprised an insignificant percentage of the total reads. The reads were then aligned to the macaque genome *Macaca mulatta* Mmul_10.98, using the STAR spliced read aligner. The percent of uniquely mapped reads (mapping efficiency) of samples to the reference genome ranged from 43 to 86%, suggesting a high degree of matching^16^ (Supplementary Figure S1).

### Whole blood transcriptome profiling and identification of radiation-response mRNAs in macaques

**Figure 2:**
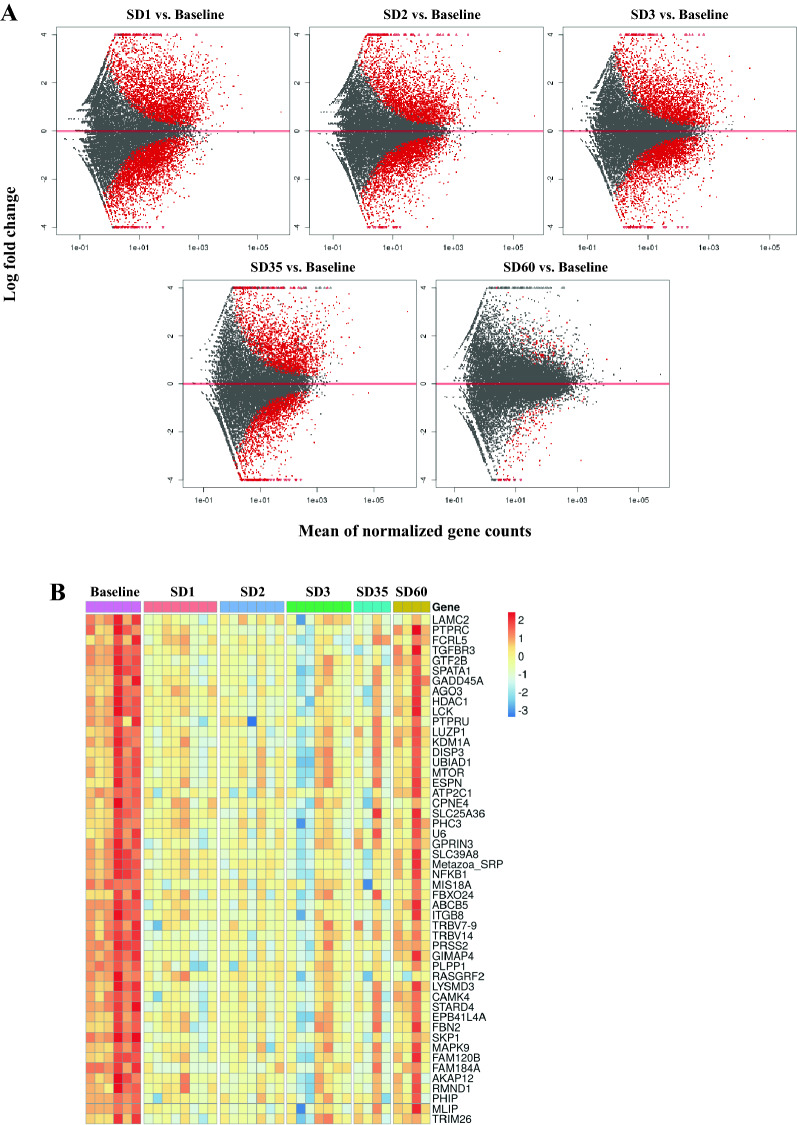
(**A**) MA plot showing differences in gene expression between irradiated and baseline samples at different time points post-irradiation. The red dots indicate significantly changed genes and grey dots indicate non-significant genes. The X-axis represents mean of normalized gene counts and the Y-axis represents the Log transformed fold change. (**B**) Heat map showing top 50 genes expression across baseline, SD1 (day 1 post-irradiation), SD2 (day 2), SD3 (day 3), SD35 (day 35), SD60 (day 60). Heat map is created by open source software R (version 4.0.3, https://cran.r-project.org/).

**Figure 3:**
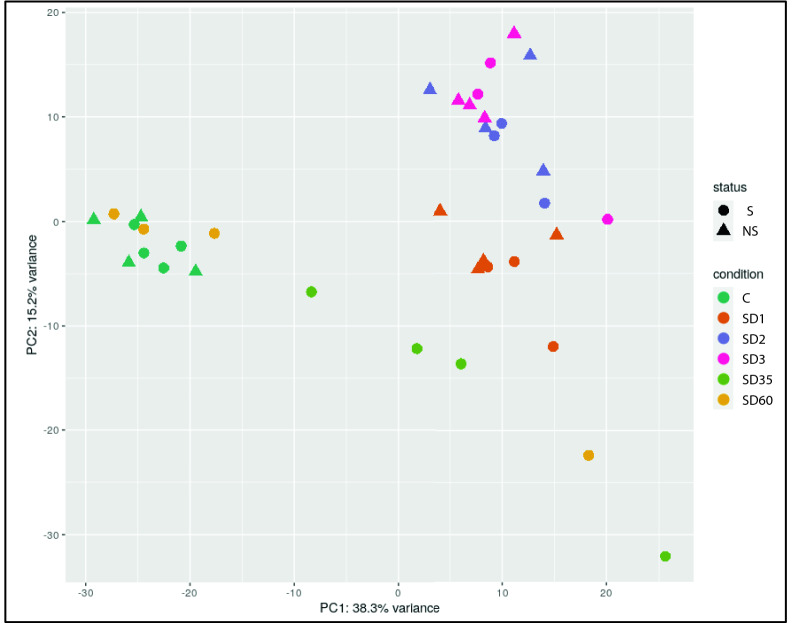
2D-PCA plot of overall gene counts showing a tight clustering within group and a clear separation among different comparative groups. Survivors are marked as S, while non-survivors are marked as NS. Figure is created by open source software R (version 4.0.3, https://cran.r-project.org/).

Genes were mapped in the macaque genome database resulting in 27,109 annotated genes using the R Package “Rsubread”^17^. We performed differential gene expression analysis through DESeq2 comparing irradiated NHP samples to samples collected prior to irradiation (baseline) from the same NHP cohort, and also for sex-based gene expression differences at each time point after irradiation (SD1, SD2, SD3). In order to delineate the gene expression signature of radiation injury in the NHPs, we used the 7-day pre-irradiation RNA samples as the baseline for following the overall time-dependent and mortality-specific changes. Genes with significant alteration in expression were filtered with a cut-off of FDR < 0.05 (see “Materials and methods”). Radiation-induced robust changes in gene expression for each comparison at SD1, SD2, and SD3 (Table 1, Supplementary Table S2). Overall radiation effects were visualized using MA plot which shows the mean of normalized gene counts along with log-transformed fold change comparing post-radiation versus baseline at different time points (Fig. 2, panel A). The heat map (Fig. 2, panel B) for top 50 dysregulated genes clearly shows the overall down-regulation trend in SD 1, SD2, SD3, SD35, and SD60. Interestingly, the percentage of differential gene expression in the survivor group was highest at SD1 and subsequently stabilized and decreased over time, suggesting homeostasis. On the other hand, the non-survivor group showed a sustained increase in the percentage of differentially expressed genes over time which could help explain high mortality in this group (Table 1). Since there was 50% mortality by SD35, we did not have enough statistical power to determine radiation response at SD35 and SD60. A score plot for principal component analysis (PCA) was used to visualize group differences (Fig. 3) based on overall gene expression in different comparative groups. We observed maximum separation between groups (X-axis) and clustering within groups (Y-axis), providing good support to the model.

**Table 1:** Differentially expressed genes at each time point for each outcome group.

Days post irradiation	Survivor	Non-survivor	Overall
1	2890 (10.67%)	3771 (13.91%)	6104 (22.52%)
2	1515 (5.59%)	3003 (11.08%)	4931 (18.19%)
3	1620 (5.98%)	3076 (11.35%)	4775 (17.61%)
35	3363 (12.41%)	–	3363 (12.41%)
60	175 (0.65%)	–	175 (0.65%)

The numbers in parenthesis represent the percentage of those differentially expressed genes that were identified in the database matching.

Previous work has shown that female NHPs are more sensitive to radiation than males. However, we did not identify a sex effect; this could be attributed to the small cohort size used in this study compared to the earlier study. The number of differentially expressed genes (fold change ≥ 2 and FDR < 0.05) in female as compared to male NHPs at SD1 (N = 22), SD2 (N = 24), and SD3 (N = 141) were found to be modest (Supplementary Table S2). There were no statistically significant differences in gene expression between animals that succumbed to radiation injury and those that survived after adjustment for multiple hypothesis testing for sex-specific groups, although the small sample size does not allow for an independent subset analysis.

### Radiation exposure triggers robust changes in gene expression

A comparison of survivors at SD1 vs. pre-irradiation samples yielded a total of 2404 differentially expressed genes (fold change ≥ 2 and FDR < 0.05) including 1407 genes that were upregulated and 997 downregulated after irradiation. A similar comparison of the non-survivors yielded a total of 2996 differentially expressed genes including 1618 genes that were upregulated and 1378 that were downregulated. Similar comparisons were performed for SD2 and SD3; an analysis was performed for the survivors and non-survivors separately for which detailed test statistics are shown in Supplementary Table S2. Similarly, at SD2, the survivor group showed 998 upregulated genes and 517 downregulated genes, while in the non-survivor group, 1692 genes were upregulated and 1311 genes were downregulated. At SD3, the survivor group has an overall decrease in the number of dysregulated genes (970 up and 650 down), while the non-survivor group showed 1744 upregulated genes and 1332 downregulated genes, respectively.

We used a 6-way Venn diagram to visualize common and unique gene expression changes for upregulated genes in the survivor and non-survivor groups at days 1, 2 and 3 post-irradiation (Supplementary Figure S2 Panel A). The Venn diagram illustrates that there were 151 unique and significant genes at SD1 (survivors at day 1) and 471 overlapping, differentially expressed genes among survivors and non-survivors at SD1, SD2 and SD3. The downregulated genes for similar comparisons are illustrated in (Supplementary Figure S2, Panel B). The detailed gene names for each group are listed in Supplementary Table S3. Those unique genes which show up/down regulation in survivors group only are of our most interest and will be discussed in the later section.

### Pathway analysis

**Figure 4:**
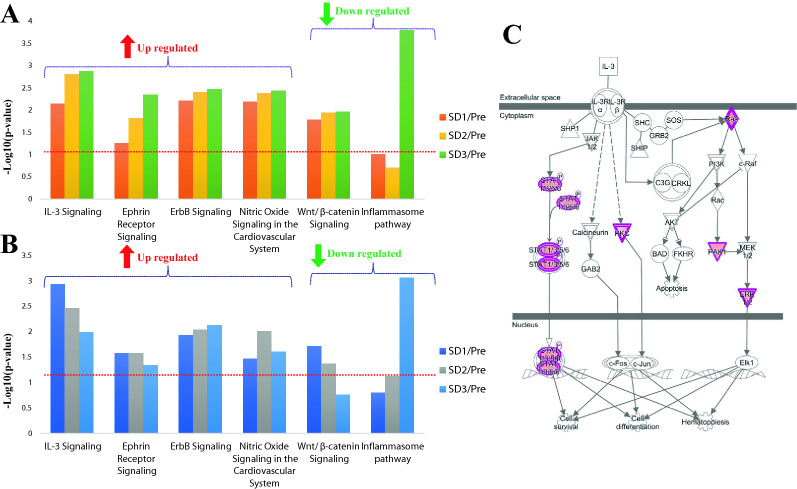
Ingenuity pathway analysis (IPA) showing trend of radiation related significantly dysregulated pathways in survivors (**A**) and non-survivors (**B**). The red line denotes the threshold of significance (p < 0.05). (**C**) IL-3 Signaling pathway was upregulated in the survivors at day 1. The regulatory gene nodes (in purple) were found to be upregulated in our dataset. The pathway figure is generated by Ingenuity Pathway Analysis (IPA, QIAGEN Inc. https://www.qiagen.com/).

**Figure 5:**
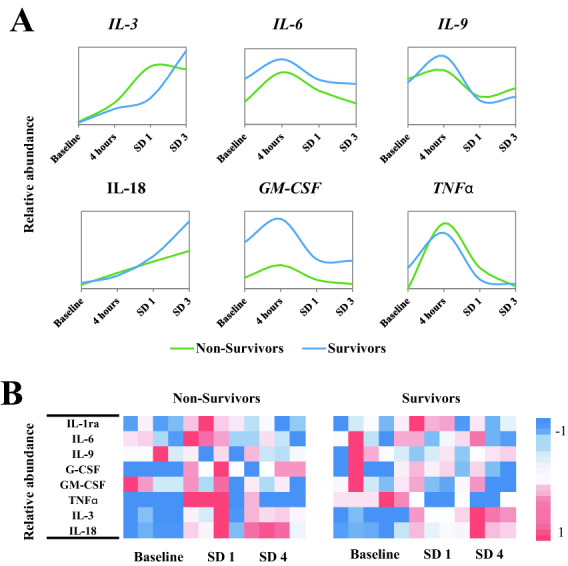
(**A**) Line plots represent the relative abundance of cytokine levels in validation study performed with samples at baseline, 4 h, day 1 and day 3 post-irradiation time points. (**B**) Heat map of per-mortality group per sample cytokine levels across time. Heat map is created by open source software R (version 4.0.3, https://cran.r-project.org/).

The canonical pathway analysis results for upregulated and downregulated genes correlated with post-exposure time and mortality are shown in Supplementary Table S4. Overall, the IPA based analyses showed different time-dependent trends in the survivor and non-survivors that are detailed in Supplementary Table S5. For example, in the survivor group, multiple pathways were found to be significantly enriched at SD1, SD2, and SD3 (p value < 0.05). Pertinently, some of these pathways including ephrin receptor signaling, paxillin signaling, CCR3 signaling in eosinophils, ErbB signaling, nitric Oxide signaling in the cardiovascular system, integrin signaling, and IL-3 signaling, etc. are well known to mediate radiation response. Interestingly, some of these pathways showed a divergent trend in the survivors and non-survivors, suggesting that pathway perturbations in response to acute radiation exposure at early time points can be highly predictive of survival outcomes that occur weeks later (Fig. 4, panel A, panel B). The dysregulation of the IL-3 signaling pathway (Fig. 4, panel C) was validated using a cytokine array (Fig. 5, panel A) and was visualized as a heat map (Fig. 5, panel B).

## Discussion

Exposure to ionizing radiation triggers a systemic cascade of changes in gene expression that ultimately dictates the individual’s response to radiation exposure that manifests as delayed effects that determine survival outcomes. There are several reports of gene regulation in NHPs in response to radiation exposure^18–21^. For example, radiation induced acute effects manifesting as alterations in gene expression in peripheral blood cells have been reported by Ghandhi et al.^22^. Dr. Cline’s group has shown histopathological changes in brains of NHPs that obtained fractionated doses of radiation, after a 14 month follow up period^23^. Additionally, Michalson et al.^24^ have reported differential gene expression of monocytes isolated from peripheral blood mononuclear cells of NHPs to study delayed effects of acute radiation exposure (DEARE). Several genes have been identified for early prediction of late-occurring hematopoietic ARS (H-ARS) in baboons^20^. Six genes were identified in baboons (WNT3, POU2AF1, CCR7, ARG2, CD177, and WLS) and validated in human leukemia patients exposed to radiotherapy^19^. There are studies for gene expression changes after radiation exposure conducted on ex vivo whole blood or lymphocyte cultures^25–27^. Previously, a transcriptomic study performed with partial-/total-body irradiation of baboons has reported robust gene expression changes.

The purpose of this study was to examine the effects of whole-body exposure to ionizing radiation over time in NHPs. We used a transcriptomic approach to explore pathway alterations in the survivors and non-survivors of male and female NHPs that received an acute exposure to 6.5 Gy of γ-radiation at a dose rate of 0.6 Gy/min. We followed the animals up to 60 days’ post-irradiation with blood collection at the baseline (pre-irradiation), SD1, SD2, SD3, SD35, and SD60 for NextSeq 500 paired-end sequencing. During this time, four animals succumbed to radiation injury that included 2 females and 2 male NHPs, indicating minimal effect of sex-based sensitivity to radiation related mortality. The principal component analysis in Fig. 3 illustrates that NHP samples cluster very well for each time point before/after radiation and survivor/non-survivors group separates clearly within each time group. We then used a combination of t statistics bioinformatics analytical pipelines for pair-wise comparison of radiation and baseline samples for each time point. Our goal was twofold; firstly, to understand the overall changes in gene expression as a function of time after radiation response and secondly, to determine if there were specific changes that help predict survival or non-survival at early time points after irradiation. Our findings strongly suggest that pathway perturbations observed at SD2 and SD3 can be predictive of survival outcomes due to radiation injury. One of the striking observations of the study was the dysregulation of Interleukin (IL)-3 signaling that is known to modulate immune system response. IL-3 is a cytokine produced by activated T-lymphocytes which stimulates the production and function of hematopoietic cell types and cells involved in immune response^28–30^. We found that IL-3 signaling was upregulated in both survivors and non-survivors at 24 h’ post-irradiation. However, while the pathway remained upregulated in survivors at SD3, there was a progressive decrease in the gene expression of this signaling pathway in the non-survivors. In the survivors, MAPK3, PAK1, PRKCE, PRKCH, PRKD3, RALB, RRAS, and STAT5B that are involved in the IL-3 signaling pathway were significantly upregulated, while in the non-survivors, genes including JAK1, MAPK3, PAK1, PRKCE, PRKCH, PRKD3, PTPN6, RALB, RRAS, and STAT5B were significantly upregulated comparing SD1 with baseline, and showed decreased expression at SD2 and SD3. These trends were further confirmed by cytokine measurements using multiplex Luminex platform.

In addition, IL-3 plays an important role in angiogenesis^31^ and central nervous system development^32,33^. IL-3, granulocyte–macrophage colony-stimulating factor (GM-CSF), and IL-5 are members of the β common (βc) cytokine family. It has been noted in several studies that G-CSF and GM-CSF given prior to lethal irradiation enhance survival in several preclinical animal models, and both agents have been approved by the US FDA as radiomitigators for human use^34–36^. Progressive increase in IL-3 signaling in the survivors, therefore, may contribute to recovery from radiation injury since it has been shown to elevate IL1 levels that is protective of the hematopoietic system against ionizing radiation^37^. Furthermore, it has been used as a radiation countermeasure for the treatment of various radiation exposed accident victims^38,39^.

We also observed significant dysregulation of the ephrin receptor signaling in the survivors and the non-survivors with divergent trends. The survivors showed an increase in expression of participating genes of this pathway in a time-dependent manner while in the non-survivors, the upregulated ephrin receptor signaling pathway stayed constant on SD1 and SD2, and slightly decreased on SD3. The upregulated genes of the ephrin receptor signaling pathway showed significant increase over time in the survivors including EFNB3, EGF, EPHA2, MAPK3, PAK1, PAK6, RALB, ROCK2, RRAS, VEGFB, and WIPF1. Radiation is known to upregulate chronic inflammation that leads to fibrosis and organ injury^40^. Thus, sustained upregulation in the survivors may contribute to higher survival rate since it is a principal pathway that modulates the inflammatory response to radiation that may, in part, contribute towards longevity in the survivor group.

Another significantly dysregulated pathway was nitric oxide signaling that is related to vascular and cardiac function/disease^41^. The upregulation of nitric oxide signaling in both survivors and non-survivors included significantly upregulated genes: ARG2, GUCY1A1, MAPK3, PRKACB, PRKCE, PRKCH, PRKD3, RYR2, and VEGFB. The trend was similar in both groups with a significantly increased expression of pathway. However, the level of upregulation was higher in the survivors. The contribution of this pathway to radiation response and alleviation of radiation injury is well documented.

Finally, we found the ErbB signaling pathway significantly increased for both survivors and non-survivors, including genes: EFNB3, EGF, EPHA2, MAPK3, PAK1, PAK6, RALB, ROCK2, RRAS, VEGFB, and WIPF1. Specifically, in non-survivors, ErbB4 signaling increase was larger. For non-survivors, at SD1 versus pre-irradiation, EGF was the distinct gene that was upregulated while on SD2 and SD3 compared to pre-irradiation, NRG3 was uniquely over expressed. This is a strong and common indicator of tumor progression pathways^42^. For survivors, EGF was also a uniquely expressed gene on SD1 versus pre-irradiation, while NRG3 was not significantly regulated in survivors. The ErbB receptors signal through Akt, MAPK, and many other pathways to regulate cell proliferation, migration, differentiation, apoptosis, and cell motility. The common existence and upregulation trends in both survivors and non-survivors indicate that radiation is a common risk factor of cancer^43^.

There are certain limitations with NHP use in research; firstly, a minimum number of animals are used in any study and secondly, any unnecessary stress, pain, and suffering to the animals must be avoided. Thus, it is not always possible to have appropriate control to answer all questions. Our study is with medical management (except for the use of blood products), and it is not possible to have another group without medical management to know the effects of medical management on the transcriptome. Similarly, pre-irradiation samples are compared with post-irradiation samples from the same animals to delineate the effects of irradiation, as a sham irradiated group is not easy to have.

This study outlines a gene expression analysis approach not only for delineating molecular signatures of radiation injury but also to predict pathway perturbations that may help predict long term survival after acute exposure to high doses of radiation in NHPs. Death post-irradiation occurs due to radiation injury which leads to infection and multi-organ failure. IL3 signaling and ErbB family of proteins were elevated in both survivors and non-survivors, which may trigger organ injury. Finally, both groups showed upregulation in the gene expression of the nitric oxide signaling pathway, indicative of increased oxidative and nitrosative stress upon irradiation. Findings of this pilot study need to be validated in independent experiments before the usefulness of these genes/pathways for predicting the outcome of H-ARS can be fully established. Further studies are also needed to dissect these pathways that were impacted by ionizing radiation and those that were triggered in the survivors leading to alleviation of radiation injury. In future investigations, metabolomics and proteomics analysis can provide a deeper understanding of radiation induced alleviation of radiation injury.

## Material and methods

### Animals and animal care

Eight naïve rhesus macaques (*Macaca mulatta*, Chinese sub-strain, 5 males and 3 females) 49–54 months of age (young adult), weighing 4.05–5.45 kg, were procured from the National Institutes of Health Animal Center (Poolsville, MD, USA) and quarantined for 6 weeks prior to the experiment. Animals were fed primate diet (Teklad T.2050 diet, Harlan Laboratories Inc. Madison, WI), health monitoring was carried out, appropriate enrichment was provided, and animals received water ad libitum^44^. Due to study-specific reasons, paired housing was not possible during this study. The animals were housed individually, but they were able to see and touch conspecifics through the cage divider. Irradiated animals are more prone to infection as their natural immunity is suppressed. Individual housing eliminates the chance of conflict injuries that could be caused by paired housing, leading to serious health consequences for the animals. This animal study was conducted in a facility accredited by the Association for Assessment and Accreditation of Laboratory Animal Care-International. All procedures involving animals were approved (Protocol # P-2015-01-001 approved on 30th March 2015) by the Institutional Animal Care and Use Committee of Armed Forces Radiobiology Research Institute/Uniformed Services University of the Health Sciences and Department of Defense second tier Animal Care and Use Review Office (ACURO). All animal procedures in this study were carried out in strict accordance with the recommendations in the *Guide for the Care and Use of Laboratory Animals* of the National Academy of Sciences^45^. This study was carried out in compliance with the ARRIVE guidelines.

### Radiation exposure

For irradiation, two NHPs were placed on the irradiation platform facing away from each other and were exposed to a midline dose of 6.5 Gy [lethal dose to 25–50% of the population within 60 days (LD_25–50/60_) without full supportive care (blood products)] ^60^Co γ-radiation at a dose rate of 0.6 Gy/min from both sides (bilateral, simultaneous exposure). To minimize the occurrence of radiation-induced vomiting, food was withheld from each animal approximately 12–18 h prior to irradiation. Approximately 30–45 min prior to irradiation, NHPs were administered 10–15 mg/kg of ketamine hydrochloride intramuscularly for sedation, then placed in custom-made Plexiglas irradiation boxes and secured in a seated position. To deliver the precise radiation dose, NHP abdominal widths were measured with digital calipers. Animals were observed throughout the irradiation procedure via in-room cameras. Following irradiation, the animals were returned to the transport cart and their cages in the housing area, and were monitored for recovery from the procedure. The radiation field in the area of the NHP location was uniform within ± 1.5%. The dosimetry for photons was based on the alanine/EPR (electron paramagnetic resonance) dosimetry system^46^. This is one of the most precise dosimetry techniques at present which is used by national standards laboratories for the most critical measurements and calibrations. Thus, it is one of the very few methods that are used in regular worldwide inter-comparisons of the national standards of Gray. This study was performed with minimal supportive care, all supportive care provided except blood product transfusion. This model is used to depict a large scale event where blood product transfusion is not possible.

### Blood sample collection

One ml of whole blood was collected into PAXgene blood RNA tubes (PreAnalytiX, a Qiagen/Becton, Dickinson and Company, Franklin Lakes, NJ) by venipuncture from the saphenous vein of the lower leg 7 days before irradiation and on days 1, 2, 3, 35, and 60 post-irradiation. The blood was mixed immediately by inverting the tube 10 times. The tubes were left at room temperature on the bench overnight and subsequently stored at – 80 °C until use.

### Euthanasia

In this study, all animals were not expected to survive the study duration of 60 days, as the radiation doses delivered were approximately LD_25–50/60_ (6.5 Gy). Moribundity instead of mortality was used to relieve the animal from unnecessary pain and distress. Euthanasia was carried out per the American Veterinary Medical Association (AVMA) guidelines when animals reached a point of no return. When an animal reached a state of moribundity parameters described elsewhere^44^, the animal was euthanized. Moribundity status of the animal was determined by a joint effort between the institutional veterinarian, principal investigator, research staff, veterinary technicians, and husbandry staff based on the combination of criteria described.

### Cytokines analysis using Luminex platform

Luminex 200 (Luminex Corporation, Austin, TX, USA) was used to detect cytokines in NHP serum samples using custom ordered Bio-Plex human cytokine assay kits (Bio-Rad Inc., Hercules, CA, USA) as described earlier^47^. Cytokine quantification was performed using Bio-Plex Manager software, version 6.1 (Bio-Rad Inc.).

### RNA isolation

Total RNA was isolated from whole blood following the manufacturer’s protocol for the PAXgene Blood RNA Kit (PreAnalytiX, Switzerland) and quantified by fluorometry using Qubit 4 fluorometer (Invitrogen, Carlsbad, CA, USA). The quality of RNA was analyzed on a Bioanalyzer Eukaryote Total RNA Pico Chip (Agilent 2100, Agilent, CA). The average RNA Integrity Number (RIN) score across all samples was above the recommended minimum RIN of 7. Total RNA samples were stored at − 80 °C until use.

### Library preparation and sequencing

The library for RNA-Seq was prepared with 600 ng of total RNA input using TrueSeq stranded Total RNA with Ribo-Zero Globin kit (Illumina, San Diego, CA, USA) with barcoded adapters. Library size distribution was determined using a Bioanalyzer DNA 1000 kit (Agilent 2100), and the library yield and concentration was determined using the KAPA Library Quantification Kit for Illumina (Kapa Biosystems, Inc. Wilmington, MA). Clustering and sequencing were performed on the NextSeq 500 (Illumina) with paired-end reads of 75 bp in length. The gene body coverage was calculated using RSeQC, an RNA-Seq quality control package and using a set of housekeeping genes^48^. Gene body coverage curves indicate no 5′-3′ bias on coverage. The gene body coverage compares very well with the RIN values, above the recommended minimum of 7, obtained for total RNA used for library preparation and sequencing. The 5′/3′ bias RNA degradation could result in reads enriched towards the 3′ end of the gene. The data does not show any 5′ or 3′ bias, suggesting excellent RNA quality. All transcripts were scaled to 100 nucleotides, the number of reads covering each nucleotide position was calculated and a plot was generated illustrating the coverage profile along the gene body.

### Data processing and analysis

Sequencing data were demultiplexed and FASTQ files were generated using bcl2fastq2 software (Illumina, version 2.20.0). Sequencing quality control was performed using FastQC tool^49^. The reads were aligned to the macaque genome *Macaca mulatta* Mmul_10.98 using the STAR spliced read aligner^50^ and the latest Ensembl gene transfer format (GTF) file. The percent alignment of samples to the reference genome ranged from 42 to 86%. Post mapping quality control was performed using RSeQC^51^.

The read-count for each gene was obtained using the featureCounts from Rsubread^52^ along with the Ensembl GTF file. Principal Component Analysis (PCA) was performed to visualize inter- and intra-treatment differences. Differential gene expression analysis was performed using DESeq2^53^ and edgeR^54^ with an FDR cutoff of ≤ 0.05 using the Benjamini–Hochberg procedure for multiple testing correction. We used the DESeq2 multi-factor design to analyze the paired samples in this study, which includes the sample information as a term in the design formula and accounts for differences between the samples while estimating the effect due to the condition. In addition, we have applied multiple testing correction so as to minimize/eliminate false positive results.

Differentially expressed mRNAs with more than twofold change at FDR adjusted p value < 0.05 in different comparisons were used to perform pathway analysis using Ingenuity Pathway Analysis (IPA, QIAGEN Inc.)^55^. Pathway analysis of up- and down-regulated genes in the comparisons were performed separately to reveal the different radiation effect among all groups separated by time and mortality. The significant pathways were compared to identify those that increased or decreased across time. Specific pathways based on increased or decreased activity were then identified.

## Supplementary Information

Supplementary Information

## Data Availability

All raw data generated and analyzed during this study are openly available on the Dryad Digital Repository (https://datadryad.org/stash/share/UUkZcMyt8kT7-004BqRMHsZXQRuhUfRs6ToanSU8kqE).

## References

[1] Pannkuk EL, Fornace AJ, Laiakis EC (2017). Metabolomic applications in radiation biodosimetry: exploring radiation effects through small molecules. Int. J. Radiat. Biol..

[2] Straume, T. *et al.* in *NASA Radiation Biomarker Workshop.* (ed T. Straume). 10.1667/RR1382.1 18763867

[3] Singh VK, Simas M, Pollard H (2018). Biomarkers for acute radiation syndrome: Challenges for developing radiation countermeasures following animal rule. Expert Rev. Mol. Diagn..

[4] Singh VK, Newman VL, Romaine PL, Hauer-Jensen M, Pollard HB (2016). Use of biomarkers for assessing radiation injury and efficacy of countermeasures. Expert Rev. Mol. Diagn..

[5] Sproull M, Camphausen K (2016). State-of-the-art advances in radiation biodosimetry for mass casualty events involving radiation exposure. Radiat. Res..

[6] Coeytaux K (2015). Reported radiation overexposure accidents worldwide, 1980–2013: a systematic review. PLoS One.

[7] U.S. Food and Drug Administration. *Guidance Document: Product Development Under the Animal Rule*. http://www.fda.gov/downloads/drugs/guidancecomplianceregulatoryinformation/guidances/ucm399217.pdf (2015).

[8] U.S. Food and Drug Administration. *Table of Pharmacogenomic Biomarkers in Drug Labeling*. http://www.fda.gov/drugs/scienceresearch/researchareas/pharmacogenetics/ucm083378.htm (2015).

[9] European Medicines Agency. *Qualification of Novel Methodologies for Medicine Development*. http://www.ema.europa.eu/ema/index.jsp?curl=pages/regulation/document_listing/document_listing_000319.jsp&mid=WC0b01ac0580022bb0 (2015).

[10] Pharmaceutical and Medical Devices Agency. *Record of Consultations on Pharmacogenomics/Biomarkers*. https://www.pmda.go.jp/english/review-services/consultations/0001.html (2010).

[11] Hinzman CP (2018). Exposure to ionizing radiation causes endoplasmic reticulum stress in the mouse hippocampus. Radiat. Res..

[12] Hinzman CP (2019). Plasma-derived extracellular vesicles yield predictive markers of cranial irradiation exposure in mice. Sci. Rep..

[13] Cheema AK (2018). Plasma derived exosomal biomarkers of exposure to ionizing radiation in nonhuman primates. Int. J. Mol. Sci..

[14] Singh VK, Seed TM (2017). A review of radiation countermeasures focusing on injury-specific medicinals and regulatory approval status: part I. Radiation sub-syndromes, animal models and FDA-approved countermeasures. Int. J. Radiat. Biol..

[15] Hall EJ, Giaccia AJ (2012). Radiobiology for the Radiobiologist.

[16] Dobin A, Gingeras TR (2015). Mapping RNA-seq Reads with STAR. Curr. Protoc. Bioinform..

[17] Liao Y, Smyth GK, Shi W (2019). The R package Rsubread is easier, faster, cheaper and better for alignment and quantification of RNA sequencing reads. Nucleic Acids Res..

[18] Port M (2017). Pre-exposure gene expression in baboons with and without pancytopenia after radiation exposure. Int. J. Mol. Sci..

[19] Port M (2018). Validating baboon ex vivo and in vivo radiation-related gene expression with corresponding human data. Radiat. Res..

[20] Port M (2016). First generation gene expression signature for early prediction of late occurring hematological acute radiation syndrome in baboons. Radiat. Res..

[21] Port M (2017). Gene expression signature for early prediction of late occurring pancytopenia in irradiated baboons. Ann. Hematol..

[22] Ghandhi SA (2018). Whole thorax irradiation of non-human primates induces persistent nuclear damage and gene expression changes in peripheral blood cells. PLoS One.

[23] Hanbury DB (2015). Pathology of fractionated whole-brain irradiation in rhesus monkeys (*Macaca mulatta*). Radiat. Res..

[24] Michalson KT (2019). Monocyte polarization is altered by total-body irradiation in male rhesus macaques: implications for delayed effects of acute radiation exposure. Radiat. Res..

[25] Tilton SC, Markillie LM, Hays S, Taylor RC, Stenoien DL (2016). Identification of differential gene expression patterns after acute exposure to high and low doses of ow-LET ionizing radiation in a reconstituted human skin tissue. Radiat. Res..

[26] Manning G, Kabacik S, Finnon P, Bouffler S, Badie C (2013). High and low dose responses of transcriptional biomarkers in ex vivo X-irradiated human blood. Int. J. Radiat. Biol..

[27] Paul S, Amundson SA (2008). Development of gene expression signatures for practical radiation biodosimetry. Int. J. Radiat. Oncol. Biol. Phys..

[28] Broughton SE (2012). The GM-CSF/IL-3/IL-5 cytokine receptor family: from ligand recognition to initiation of signaling. Immunol. Rev..

[29] Hercus TR (2013). Signalling by the betac family of cytokines. Cytokine Growth Factor Rev..

[30] Fendler W (2017). Evolutionarily conserved serum microRNAs predict radiation-induced fatality in nonhuman primates. Sci. Transl. Med..

[31] Dentelli P, Rosso A, Olgasi C, Camussi G, Brizzi MF (2011). IL-3 is a novel target to interfere with tumor vasculature. Oncogene.

[32] Li M (2016). Adaptive evolution of interleukin-3 (IL3), a gene associated with brain volume variation in general human populations. Hum. Genet..

[33] Luo XJ (2012). The interleukin 3 gene (IL3) contributes to human brain volume variation by regulating proliferation and survival of neural progenitors. PLoS One.

[34] Neta R, Oppenheim JJ, Douches SD (1988). Interdependence of the radioprotective effects of human recombinant interleukin 1 alpha, tumor necrosis factor alpha, granulocyte colony-stimulating factor, and murine recombinant granulocyte-macrophage colony-stimulating factor. J. Immunol..

[35] Farese AM, MacVittie TJ (2015). Filgrastim for the treatment of hematopoietic acute radiation syndrome. Drugs Today (Barc.).

[36] Singh VK, Seed TM (2018). An update on sargramostim for treatment of acute radiation syndrome. Drugs Today (Barc.).

[37] Neta R, Douches S, Oppenheim JJ (1986). Interleukin 1 is a radioprotector. J. Immunol..

[38] International Atomic Energy Agency. *The Radiological Accident in Soreq, IAEA*. http://www-pub.iaea.org/books/IAEABooks/3798/The-Radiological-Accident-in-Soreq. (1993).

[39] Nesterenko VB, Nesterenko AV, Babenko VI, Yerkovich TV, Babenko IV (2004). Reducing the 137Cs-load in the organism of "Chernobyl" children with apple-pectin. Swiss Med. Wkly..

[40] Straub JM (2015). Radiation-induced fibrosis: mechanisms and implications for therapy. J. Cancer Res. Clin. Oncol..

[41] Liu VW, Huang PL (2008). Cardiovascular roles of nitric oxide: a review of insights from nitric oxide synthase gene disrupted mice. Cardiovasc. Res..

[42] Wang Z (2017). ErbB receptors and cancer. Methods Mol. Biol..

[43] Gilbert ES (2009). Ionising radiation and cancer risks: what have we learned from epidemiology?. Int. J. Radiat. Biol..

[44] Singh VK (2016). Radioprotective efficacy of gamma-tocotrienol in nonhuman primates. Radiat. Res..

[45] National Research Council of the National Academy of Sciences (2011). Guide for the Care and Use of Laboratory Animals.

[46] Nagy V (2000). Accuracy considerations in EPR dosimetry. Appl. Radiat. Isot..

[47] Singh VK (2012). Myeloid progenitors: A radiation countermeasure that is effective when initiated days after irradiation. Radiat. Res..

[48] Caracausi M (2017). Systematic identification of human housekeeping genes possibly useful as references in gene expression studies. Mol. Med. Rep..

[49] Babraham Institute. *FastQC: A Quality Control Tool For high Throughput Sequence Data*. https://www.bioinformatics.babraham.ac.uk/projects/fastqc/ (2019).

[50] Dobin A (2013). STAR: ultrafast universal RNA-seq aligner. Bioinformatics.

[51] Wang L, Wang S, Li W (2012). RSeQC: quality control of RNA-seq experiments. Bioinformatics.

[52] Liao Y, Smyth GK, Shi W (2014). featureCounts: an efficient general purpose program for assigning sequence reads to genomic features. Bioinformatics.

[53] Love MI, Huber W, Anders S (2014). Moderated estimation of fold change and dispersion for RNA-seq data with DESeq2. Genome Biol..

[54] Robinson MD, McCarthy DJ, Smyth GK (2010). edgeR: a Bioconductor package for differential expression analysis of digital gene expression data. Bioinformatics.

[55] Kramer A, Green J, Pollard J, Tugendreich S (2014). Causal analysis approaches in ingenuity pathway analysis. Bioinformatics.

