# Supplementary material for: Transcriptome of rhesus macaque (Macaca mulatta) exposed to total-body irradiation
Source: Sci Rep. 2021 Mar 18;11:6295. doi: 10.1038/s41598-021-85669-6 (PMC7973550; doi:10.1038/s41598-021-85669-6)
Supplement: Supplementary file 1 — Supplementary Information [file 41598_2021_85669_MOESM1_ESM.pdf]

Transcriptome of rhesus macaque (*Macaca mulatta*) exposed to total-body irradiation

$Yaoxiang Li,[1] $Jatinder Singh,[2,3] Rency Varghese,[1] Yubo Zhang,[4] Oluseyi O. Fatanmi,[2,3] Amrita K Cheema,[1,5] Vijay K Singh[2,3,*]

$YL and JS have made equal contributions to this study.

[1]*Department of Oncology, Lombardi Comprehensive Cancer Center, Georgetown University Medical Center, Washington, DC, USA;* [2]*Division of Radioprotectants, Department of Pharmacology and Molecular Therapeutics, F. Edward Hébert School of Medicine, Uniformed Serices University of the Health Sciences, Bethesda, MD, USA;* [3]*Armed Forces Radiobiology Research Institute, Uniformed Services University of the Health Sciences, Bethesda, MD, USA;*[4]*Department of Biochemistry and Molecular Medicine, George Washington University, Washington DC, USA;* [5]*Department of Biochemistry, Molecular and Cellular Biology, Georgetown University Medical Center, Washington DC, USA*

Supplementary Figure 1. The percent of uniquely mapped reads (mapping efficiency) of samples to the reference genome ranged from 43% to 86%. Figure is created by the author using Microsoft Excel (URL: https://www.microsoft.com/en-us/microsoft-365/excel).

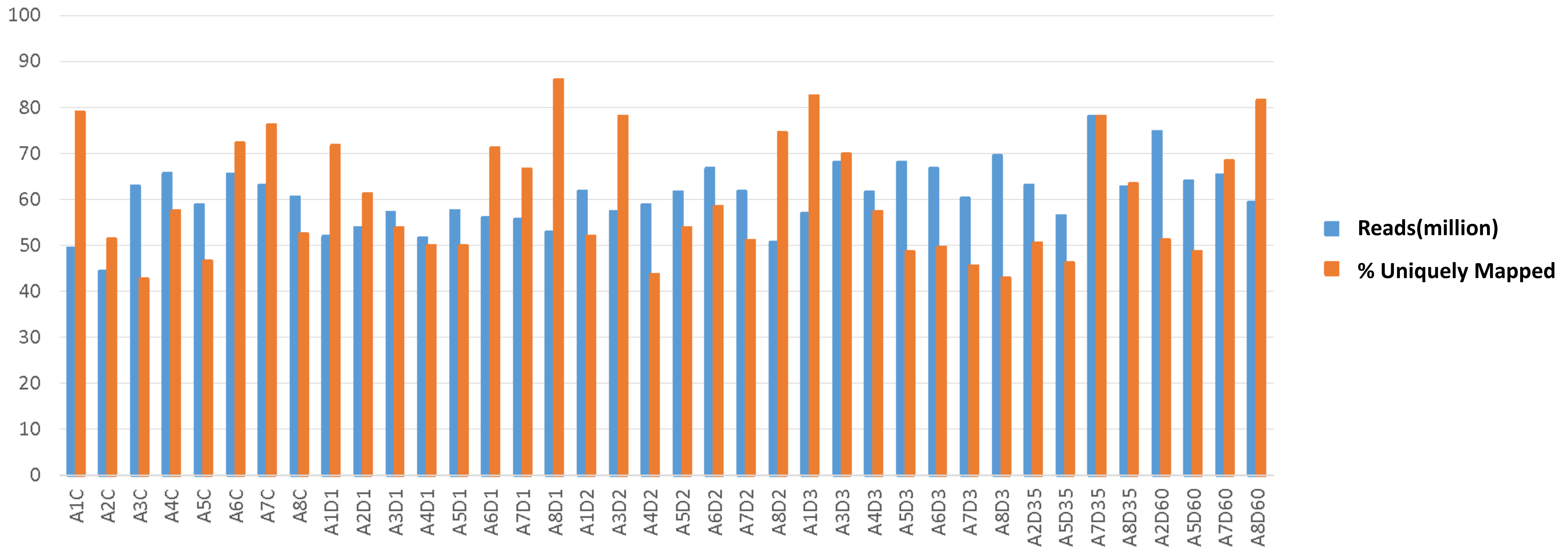

Supplementary Figure 2. 6-way Venn diagram of upregulated (**Panel A**)/ downregulated (**Panel B**) differentially expressed genes (DEGs) with a fold change ≥ 2, and FDR cut-off of 0.05, in the survivor and non-survivor groups, for days 1, 2, 3 versus baseline. The six groups are color coded to denote significantly expressed genes within each group and overlap across groups. Venn diagram data was processed using R (version 4.0.3, URL: https://cran.r-project.org/) and generated using an online tool (http://bioinformatics.psb.ugent.be/webtools/Venn/)

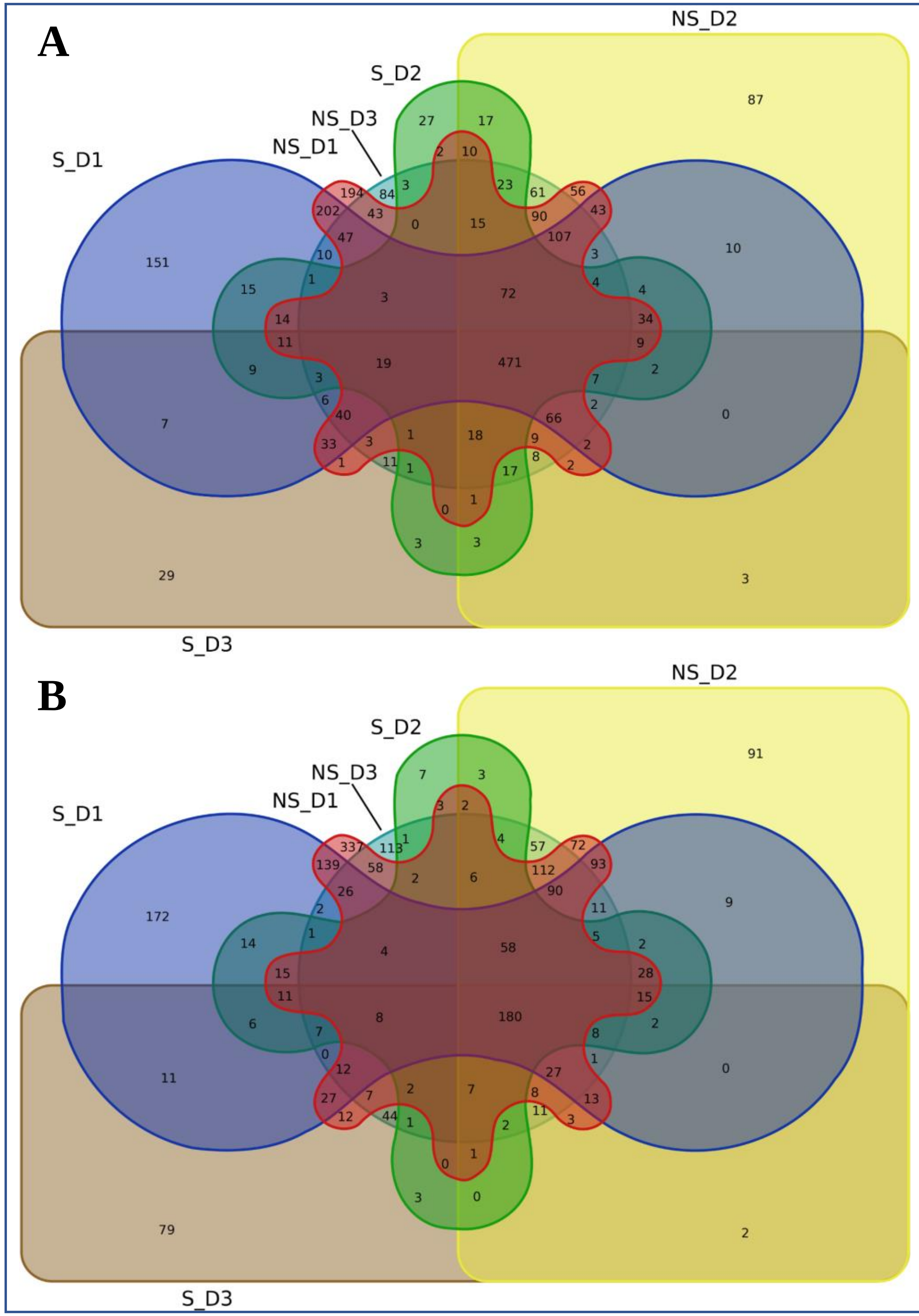

S_D1: Survivor D1 vs. Baseline
S_D2: Survivor D2 vs. Baseline
S_D3: Survivor D3 vs. Baseline
NS_D1: Non-Survivor D1 vs. Baseline
NS_D2: Non-Survivor D2 vs. Baseline
NS_D3: Non-Survivor D3 vs. Baseline

Supp. Table 1. NHP Cohort sampling details and survival outcomes

				Sampling Time Post-Irradiation - Days					
**Animal#**	**NHP#**	**Sex**	**Group**	**SD (-7)[a]**	**SD1**	**SD2**	**SD3**	**SD35**	**SD60**
A1	RA2688M	Male	Veh	X[b]	X	X	X	Dead	-
A2	RA2708M	Male	Veh	X	X	Low RNA	Low RNA	X	X
A3	RA2270F	Female	Veh	X	X	X	X	Dead	-
A4	RA2384F	Female	Veh	X	X	X	X	Dead	-
A5	RA2796M	Male	Veh	X	X	X	X	X	X
A6	RA2693M	Male	Veh	X	X	X	X	Dead	-
A7	RA2698M	Male	Veh	X	X	X	X	X	X
A8	RA2654F	Female	Veh	X	X	X	X	X	X

a. SD (-7)= Sampled 7 days prior to Irradiation

b. X= Sequenced

Supplementary Table 2

A. Overall radiation effects

SD 1 vs. Baseline

Ensembl Gene ID	Description	Log2(FC)	p-value	FDR
ENSMMUG00000049101	olfactory receptor 2T10 [Source:NCBI gene;Acc:100431025]	0.498201968	0.018785099	0.046307017
ENSMMUG00000048289	olfactory receptor 2T5-like [Source:NCBI gene;Acc:707109]	0.683801844	0.00463043	0.013910785
ENSMMUG00000017368	olfactory receptor 2G6 [Source:NCBI gene;Acc:694380]	-0.776701367	0.000754872	0.0027938
ENSMMUG00000038524	olfactory receptor 2M4 [Source:NCBI gene;Acc:100424541]	2.59194334	2.13E-41	7.02E-39
ENSMMUG00000049432	olfactory receptor 2T33 [Source:NCBI gene;Acc:706331]	1.249206954	1.36E-17	3.77E-16
ENSMMUG00000050812		1.175793013	0.001410394	0.004888091
ENSMMUG00000064763		0.944209014	0.000204876	0.000871575
ENSMMUG00000050041	olfactory receptor 2L5 [Source:NCBI gene;Acc:697549]	0.908775468	0.00859237	0.023751136
ENSMMUG00000006214	olfactory receptor family 11 subfamily L member 1 [Source:HGNC Symbol;Acc:HGNC:14998]	-3.030623468	0.000136522	0.000602926
ENSMMUG00000005955	germinal center associated signaling and motility like [Source:VGNC Symbol;Acc:VGNC:72806]	-0.563037253	0.011466013	0.030462374
ENSMMUG00000005949	olfactory receptor 2B11 [Source:NCBI gene;Acc:709256]	-2.168963174	4.39E-05	0.000215432
ENSMMUG00000004566	zinc finger protein 124 [Source:VGNC Symbol;Acc:VGNC:78836]	-3.467873667	0.000866997	0.003161336
ENSMMUG00000000021	catsper channel auxiliary subunit epsilon [Source:VGNC Symbol;Acc:VGNC:70657]	-3.419497424	0.000546971	0.002108546
ENSMMUG00000063991		-2.427877095	0.00301095	0.009516706
ENSMMUG00000049847		3.078744825	0.005187842	0.015310586
ENSMMUG00000019994	serologically defined colon cancer antigen 8 [Source:NCBI gene;Acc:705545]	-1.069939492	0.005212745	0.015362681
ENSMMUG00000059561		-2.325621318	0.000360686	0.001450371
ENSMMUG00000004265	phospholipase D family member 5 [Source:VGNC Symbol;Acc:VGNC:76120]	-1.700719786	0.002194349	0.007199601
ENSMMUG00000005668	kynurenine 3-monooxygenase [Source:VGNC Symbol;Acc:VGNC:74197]	1.040220131	1.91E-07	1.47E-06
ENSMMUG00000005667	fumarate hydratase [Source:VGNC Symbol;Acc:VGNC:72659]	0.687718876	0.000433542	0.001711776
ENSMMUG00000054974		-0.881425983	0.001175644	0.00416602
ENSMMUG00000064824	gremlin 2, DAN family BMP antagonist [Source:NCBI gene;Acc:708280]	2.345141191	1.08E-12	1.77E-11
ENSMMUG00000059198		-1.903796177	0.011917265	0.031480768
ENSMMUG00000054726		-0.667884254	0.01833837	0.045371709
ENSMMUG00000061845		2.350903974	5.66E-10	6.43E-09
ENSMMUG00000001060	ryanodine receptor 2 [Source:VGNC Symbol;Acc:VGNC:76954]	1.548552956	1.99E-28	1.71E-26
ENSMMUG00000015132	galectin 8 [Source:VGNC Symbol;Acc:VGNC:74258]	-1.212015316	0.001148895	0.004080985
ENSMMUG00000049832	interferon regulatory factor 2 binding protein 2 [Source:NCBI gene;Acc:718705]	-0.638231733	0.014531361	0.03724509
ENSMMUG00000046785		-2.436050369	6.75E-06	3.91E-05
ENSMMUG00000009226	mitogen-activated protein kinase kinase kinase 21 [Source:NCBI gene;Acc:712755]	-0.869824685	4.73E-05	0.000231229
ENSMMUG00000063981	nucleoside-triphosphatase, cancer-related [Source:NCBI gene;Acc:712959]	3.6018504	1.11E-24	6.47E-23

ENSMMUG00000040055		-0.683426784	0.000876068	0.003189401
ENSMMUG00000061796		-1.156977916	0.000234567	0.000986286
ENSMMUG00000012068	signal induced proliferation associated 1 like 2 [Source:NCBI gene;Acc:100425123]	2.531881213	5.01E-12	7.51E-11
ENSMMUG00000052961		2.462399181	6.68E-07	4.68E-06
ENSMMUG00000064226		2.115958719	1.97E-06	1.27E-05
ENSMMUG00000016204	translin associated factor X [Source:NCBI gene;Acc:713347]	-3.412761944	0.00125738	0.004416616
ENSMMUG00000050564		-1.727577446	0.01092665	0.029186278
ENSMMUG00000005801	glyceronephosphate O-acyltransferase [Source:VGNC Symbol;Acc:VGNC:73100]	-1.973626384	1.13E-15	2.55E-14
ENSMMUG00000005888	tetratricopeptide repeat domain 13 [Source:VGNC Symbol;Acc:VGNC:79588]	-0.964054571	1.12E-08	1.04E-07
ENSMMUG00000005886	chromosome 1 C1orf198 homolog [Source:VGNC Symbol;Acc:VGNC:70389]	0.601053007	0.008821616	0.024316834
ENSMMUG00000009508	TATA-box binding protein associated factor 5 like [Source:VGNC Symbol;Acc:VGNC:78148]	1.942057133	4.29E-07	3.10E-06
ENSMMUG00000022094	ATP binding cassette subfamily B member 10 [Source:NCBI gene;Acc:574117]	0.573220389	3.50E-06	2.14E-05
ENSMMUG00000055996		0.722751206	0.003261394	0.010232491
ENSMMUG00000017720	RAB4A, member RAS oncogene family [Source:VGNC Symbol;Acc:VGNC:81545]	0.408570552	0.014329518	0.036772235
ENSMMUG00000061914		0.573343598	0.015597098	0.039525798
ENSMMUG00000061675	5S ribosomal RNA [Source:RFAM;Acc:RF00001]	3.079160772	0.01855547	0.04579699
ENSMMUG00000005185	regulator of G protein signaling 4 [Source:VGNC Symbol;Acc:VGNC:76690]	2.717486612	0.006325443	0.018207747
ENSMMUG00000005186	regulator of G protein signaling 5 [Source:NCBI gene;Acc:693510]	0.800922809	0.009360306	0.025582996
ENSMMUG00000001152	NUF2 component of NDC80 kinetochore complex [Source:VGNC Symbol;Acc:VGNC:75405]	0.359450371	0.00397621	0.012165709
ENSMMUG00000039738	U6 spliceosomal RNA [Source:RFAM;Acc:RF00026]	-1.732908322	0.005097665	0.015086508
ENSMMUG00000005585	transmembrane and coiled-coil domains 1 [Source:VGNC Symbol;Acc:VGNC:79118]	-0.98195721	0.000815985	0.002997535
ENSMMUG00000017789	pogo transposable element derived with KRAB domain [Source:VGNC Symbol;Acc:VGNC:76066]	0.617155699	0.003497011	0.010854054
ENSMMUG00000023463	maelstrom spermatogenic transposon silencer [Source:VGNC Symbol;Acc:VGNC:74362]	-1.723218878	0.008082937	0.022536182
ENSMMUG00000051788	dual specificity phosphatase 27, atypical [Source:NCBI gene;Acc:697299]	-1.555551462	0.011313269	0.030132144
ENSMMUG00000008155	POU class 2 homeobox 1 [Source:VGNC Symbol;Acc:VGNC:76223]	-0.890526417	0.000862155	0.003145327
ENSMMUG00000063120	cellular repressor of E1A stimulated genes 1 [Source:NCBI gene;Acc:701021]	-0.690737017	0.001756248	0.005934189
ENSMMUG00000009184	myelin protein zero like 1 [Source:NCBI gene;Acc:698437]	2.227489544	1.17E-08	1.09E-07
ENSMMUG00000010389	DDB1 and CUL4 associated factor 6 [Source:NCBI gene;Acc:698686]	0.507638899	0.016694469	0.041948319
ENSMMUG00000060729	T-box transcription factor 19 [Source:NCBI gene;Acc:699312]	0.501631477	0.008893861	0.024475034
ENSMMUG00000056707		2.536684968	3.90E-06	2.36E-05
ENSMMUG00000059777		1.111908874	2.33E-10	2.81E-09
ENSMMUG00000021078	ATPase Na+/K+ transporting subunit beta 1 [Source:VGNC Symbol;Acc:VGNC:70176]	-1.150226485	0.000568288	0.002175795
ENSMMUG00000021079	NME/NM23 family member 7 [Source:VGNC Symbol;Acc:VGNC:75221]	0.935527157	0.001014743	0.003648155
ENSMMUG00000021080	basic leucine zipper nuclear factor 1 [Source:VGNC Symbol;Acc:VGNC:70258]	1.207146093	3.18E-27	2.48E-25
ENSMMUG00000000065	coiled-coil domain containing 181 [Source:VGNC Symbol;Acc:VGNC:70884]	-0.973692833	2.12E-10	2.57E-09
ENSMMUG00000042510	coagulation factor V [Source:NCBI gene;Acc:704695]	1.495706278	6.34E-26	4.13E-24
ENSMMUG00000013044	selectin P [Source:VGNC Symbol;Acc:VGNC:77157]	1.250248938	1.92E-11	2.64E-10
ENSMMUG00000054881		2.933333517	0.018489293	0.045676531
ENSMMUG00000016579	selectin L [Source:NCBI gene;Acc:701419]	2.855179029	2.10E-37	4.33E-35
ENSMMUG00000016580	selectin E [Source:VGNC Symbol;Acc:VGNC:77156]	2.868716123	3.30E-47	1.96E-44

ENSMMUG00000016581	methyltransferase like 18 [Source:VGNC Symbol;Acc:VGNC:74606]	-0.994518328	0.014088583	0.036279509
ENSMMUG00000016582	chromosome 1 C1orf112 homolog [Source:VGNC Symbol;Acc:VGNC:70385]	1.658041922	1.79E-14	3.63E-13
ENSMMUG00000059773	methyltransferase like 11B [Source:NCBI gene;Acc:705503]	-1.499234137	1.14E-06	7.67E-06
ENSMMUG00000063298		-1.103610012	0.00038912	0.001554176
ENSMMUG00000014674	golgin, RAB6 interacting [Source:VGNC Symbol;Acc:VGNC:73012]	1.238384313	1.42E-09	1.52E-08
ENSMMUG00000007279	phosphatidylinositol glycan anchor biosynthesis class C [Source:VGNC Symbol;Acc:VGNC:75986]	0.997446031	9.11E-06	5.16E-05
ENSMMUG00000054261		1.185589217	0.000639208	0.002418847
ENSMMUG00000022798	aspartyl-tRNA synthetase 2, mitochondrial [Source:VGNC Symbol;Acc:VGNC:71708]	0.935957239	2.10E-07	1.60E-06
ENSMMUG00000056971	Metazoan signal recognition particle RNA [Source:RFAM;Acc:RF00017]	1.584625438	1.20E-07	9.59E-07
ENSMMUG00000060229		1.084755067	0.000664615	0.002500313
ENSMMUG00000025832		2.410320957	0.020346544	0.049562951
ENSMMUG00000022801	zinc finger and BTB domain containing 37 [Source:VGNC Symbol;Acc:VGNC:79702]	1.280765367	0.000235329	0.00098865
ENSMMUG00000000629	serpin family C member 1 [Source:VGNC Symbol;Acc:VGNC:77360]	1.164723172	5.00E-23	2.47E-21
ENSMMUG00000000630	ring finger and CCCH-type domains 1 [Source:VGNC Symbol;Acc:VGNC:76861]	-0.580746936	2.21E-05	0.000116402
ENSMMUG00000064770		-1.140140357	0.002154517	0.007086157
ENSMMUG00000000546	KIAA0040 ortholog [Source:NCBI gene;Acc:100423509]	-1.094044474	2.45E-05	0.000127808
ENSMMUG00000023216	astrotactin 1 [Source:VGNC Symbol;Acc:VGNC:70167]	-0.558846206	0.004515024	0.01360378
ENSMMUG00000022935	BMP/retinoic acid inducible neural specific 2 [Source:VGNC Symbol;Acc:VGNC:70303]	-0.762111977	2.10E-05	0.00011103
ENSMMUG00000020320	protein transport protein Sec16B [Source:NCBI gene;Acc:709635]	0.884664137	1.86E-06	1.20E-05
ENSMMUG00000021073	RAS protein activator like 2 [Source:VGNC Symbol;Acc:VGNC:76670]	3.004918591	3.33E-34	5.21E-32
ENSMMUG00000059117	Metazoan signal recognition particle RNA [Source:RFAM;Acc:RF00017]	1.211241452	0.000790378	0.002915748
ENSMMUG00000056058	C-type lectin domain containing 20A [Source:HGNC Symbol;Acc:HGNC:34521]	3.316062848	2.07E-07	1.58E-06
ENSMMUG00000054486	testis expressed 35 [Source:NCBI gene;Acc:717255]	2.552038198	5.48E-06	3.24E-05
ENSMMUG00000055256	family with sequence similarity 163 member A [Source:NCBI gene;Acc:718704]	1.496974607	1.73E-14	3.51E-13
ENSMMUG00000031279	torsin A interacting protein 2 [Source:NCBI gene;Acc:716654]	1.532528861	1.35E-26	9.71E-25
ENSMMUG00000053617	torsin 1A interacting protein 2 [Source:NCBI gene;Acc:716691]	0.801545086	9.22E-07	6.30E-06
ENSMMUG00000014873	torsin 1A interacting protein 1 [Source:VGNC Symbol;Acc:VGNC:79810]	0.678483974	1.58E-05	8.57E-05
ENSMMUG00000046557	Metazoan signal recognition particle RNA [Source:RFAM;Acc:RF00017]	2.770181931	0.015196856	0.038670106
ENSMMUG00000054568		1.530000202	5.67E-08	4.73E-07
ENSMMUG00000061785		1.516605866	2.70E-26	1.84E-24
ENSMMUG00000052988	Metazoan signal recognition particle RNA [Source:RFAM;Acc:RF00017]	1.220831856	0.00289687	0.009220989
ENSMMUG00000022884	glutamate-ammonia ligase [Source:NCBI gene;Acc:715952]	3.867648668	1.24E-13	2.28E-12
ENSMMUG00000031291	regulator of G protein signaling like 1 [Source:NCBI gene;Acc:715693]	1.720048617	2.17E-22	1.02E-20
ENSMMUG00000005634	ribonuclease L [Source:VGNC Symbol;Acc:VGNC:82223]	-1.186849456	9.60E-07	6.53E-06
ENSMMUG00000055481	regulator of G protein signaling 16 [Source:NCBI gene;Acc:715599]	-0.960087414	5.45E-05	0.000262731
ENSMMUG00000008990	regulator of G protein signaling 8 [Source:NCBI gene;Acc:720353]	2.27893476	0.008705046	0.024035716
ENSMMUG00000008992	N-acetylneuraminate pyruvate lyase [Source:VGNC Symbol;Acc:VGNC:75191]	0.365313313	0.002380027	0.007742005
ENSMMUG00000015084	laminin subunit gamma 1 [Source:VGNC Symbol;Acc:VGNC:74232]	-1.596206061	0.016538494	0.041570487
ENSMMUG00000001479	laminin subunit gamma 2 [Source:VGNC Symbol;Acc:VGNC:74233]	-3.054677654	3.20E-16	7.59E-15
ENSMMUG00000001481	nicotinamide nucleotide adenylyltransferase 2 [Source:VGNC Symbol;Acc:VGNC:75193]	-3.487285632	1.59E-08	1.44E-07

ENSMMUG00000016399	ral guanine nucleotide dissociation stimulator like 1 [Source:VGNC Symbol;Acc:VGNC:76868]	-1.442432899	0.000121949	0.000544895
ENSMMUG00000016405	collagen beta(1-O)galactosyltransferase 2 [Source:NCBI gene;Acc:717508]	-1.493500983	0.009102057	0.024964585
ENSMMUG00000064206	tRNA splicing endonuclease subunit 15 [Source:NCBI gene;Acc:717481]	-1.135875584	0.017121243	0.042795893
ENSMMUG00000054157		-2.379094788	0.005453476	0.015989531
ENSMMUG00000016857	prostaglandin-endoperoxide synthase 2 [Source:VGNC Symbol;Acc:VGNC:76589]	-1.525707172	5.84E-10	6.63E-09
ENSMMUG00000059986		-1.147404752	5.40E-08	4.53E-07
ENSMMUG00000062021		-1.772230013	3.73E-06	2.27E-05
ENSMMUG00000049590	regulator of G protein signaling 2 [Source:NCBI gene;Acc:712617]	-1.904003305	0.006010457	0.017412594
ENSMMUG00000052479		-1.434042091	0.000269407	0.001115511
ENSMMUG00000022641	ubiquitin C-terminal hydrolase L5 [Source:VGNC Symbol;Acc:VGNC:79197]	-1.190555296	8.78E-05	0.000405853
ENSMMUG00000022643	Ro60, Y RNA binding protein [Source:NCBI gene;Acc:715194]	-1.173921781	0.001300549	0.004554224
ENSMMUG00000022647	cell division cycle 73 [Source:VGNC Symbol;Acc:VGNC:70766]	-1.318990509	9.35E-09	8.79E-08
ENSMMUG00000022648	beta-1,3-galactosyltransferase 2 [Source:VGNC Symbol;Acc:VGNC:70205]	-1.549386447	6.83E-07	4.78E-06
ENSMMUG00000021082	potassium sodium-activated channel subfamily T member 2 [Source:VGNC Symbol;Acc:VGNC:73857]	0.988803412	3.60E-11	4.82E-10
ENSMMUG00000045497	complement factor H [Source:NCBI gene;Acc:717346]	-1.296173401	2.57E-05	0.000133109
ENSMMUG00000000244	coagulation factor XIII B chain [Source:VGNC Symbol;Acc:VGNC:72463]	-0.844876139	3.31E-05	0.000167407
ENSMMUG00000000009	protein tyrosine phosphatase receptor type C [Source:VGNC Symbol;Acc:VGNC:76612]	-2.116340629	3.02E-08	2.63E-07
ENSMMUG00000017414	calcium voltage-gated channel subunit alpha1 S [Source:VGNC Symbol;Acc:VGNC:70507]	-0.989050521	2.28E-06	1.45E-05
ENSMMUG00000064915	Metazoan signal recognition particle RNA [Source:RFAM;Acc:RF00017]	1.7354998	0.004636848	0.013919931
ENSMMUG00000050920	Metazoan signal recognition particle RNA [Source:RFAM;Acc:RF00017]	1.613528175	7.21E-05	0.00033846
ENSMMUG00000021642	fibromodulin [Source:VGNC Symbol;Acc:VGNC:72686]	1.460476979	0.000859716	0.003138745
ENSMMUG00000021116	ATPase plasma membrane Ca2+ transporting 4 [Source:VGNC Symbol;Acc:VGNC:70183]	0.855063537	0.009219719	0.02524062
ENSMMUG00000055783		-2.961498849	2.90E-08	2.54E-07
ENSMMUG00000008460	SRY-box transcription factor 13 [Source:VGNC Symbol;Acc:VGNC:77839]	-3.089400278	0.001862819	0.006257497
ENSMMUG00000064841	golgi transport 1A [Source:NCBI gene;Acc:700305]	-3.112953459	0.00190762	0.006391778
ENSMMUG00000017114	pleckstrin homology domain containing A6 [Source:VGNC Symbol;Acc:VGNC:76040]	-1.676943103	2.97E-05	0.000152172
ENSMMUG00000063658	leucine rich repeat neuronal 2 [Source:NCBI gene;Acc:100429709]	1.582663606	2.41E-05	0.000125609
ENSMMUG00000018797	contactin 2 [Source:VGNC Symbol;Acc:VGNC:71284]	-1.526037751	0.005510787	0.016141611
ENSMMUG00000004113	major facilitator superfamily domain containing 4A [Source:VGNC Symbol;Acc:VGNC:74575]	-0.928087651	2.28E-06	1.45E-05
ENSMMUG00000001024	ETS transcription factor ELK4 [Source:VGNC Symbol;Acc:VGNC:72211]	-1.310662217	0.009699215	0.02634903
ENSMMUG00000004116	solute carrier family 45 member 3 [Source:VGNC Symbol;Acc:VGNC:77817]	-1.538440561	0.00103523	0.003712361

ENSMMUG00000055835	nuclear casein kinase and cyclin dependent kinase substrate 1 [Source:NCBI gene;Acc:697298]	-1.138925832	1.18E-05	6.55E-05
ENSMMUG00000021426	solute carrier family 41 member 1 [Source:VGNC Symbol;Acc:VGNC:77515]	0.473011279	0.008157603	0.022721286
ENSMMUG00000023570	interleukin 19 [Source:NCBI gene;Acc:694806]	3.014085815	0.008332328	0.023161842
ENSMMUG00000022170	6-phosphofructo-2-kinase/fructose-2,6-biphosphatase 2 [Source:VGNC Symbol;Acc:VGNC:75958]	-0.394506368	0.007247914	0.020458045
ENSMMUG00000013738	complement component 4 binding protein alpha [Source:VGNC Symbol;Acc:VGNC:70474]	-0.345819585	0.004882922	0.014562365
ENSMMUG00000013596	complement C3b/C4b receptor 1 (Knops blood group) [Source:NCBI gene;Acc:718120]	1.402717118	0.001381748	0.00480453
ENSMMUG00000040888	complement C3b/C4b receptor 1 like [Source:HGNC Symbol;Acc:HGNC:2335]	0.52954324	0.002273214	0.00742223
ENSMMUG00000051230		0.832404681	0.000119716	0.000535401
ENSMMUG00000057560		0.902127075	1.16E-10	1.45E-09
ENSMMUG00000064202		-1.553494829	2.65E-06	1.66E-05
ENSMMUG00000003369	calcium/calmodulin dependent protein kinase IG [Source:VGNC Symbol;Acc:VGNC:70521]	-0.965186292	0.013936527	0.035975403
ENSMMUG00000016925	laminin subunit beta 3 [Source:VGNC Symbol;Acc:VGNC:74230]	-1.19333862	0.000644092	0.002434222
ENSMMUG00000045482	G0/G1 switch 2 [Source:NCBI gene;Acc:717581]	-0.490232982	0.000155955	0.000678642
ENSMMUG00000016099	hedgehog acyltransferase [Source:NCBI gene;Acc:712333]	2.345126402	3.74E-08	3.19E-07
ENSMMUG00000058133		-3.011079896	4.92E-07	3.52E-06
ENSMMUG00000017396	3'(2'), 5'-bisphosphate nucleotidase 1 [Source:NCBI gene;Acc:721277]	-1.82580545	9.16E-07	6.25E-06
ENSMMUG00000007783	isoleucyl-tRNA synthetase 2, mitochondrial [Source:VGNC Symbol;Acc:VGNC:73458]	-1.603303106	4.88E-20	1.71E-18
ENSMMUG00000026897	mml-mir-215 [Source:miRBase;Acc:MI0003025]	-1.98410926	0.000117224	0.000525049
ENSMMUG00000027053	mml-mir-194-1 [Source:miRBase;Acc:MI0003024]	-2.020054001	0.002012009	0.006690156
ENSMMUG00000018525	presenilin 2 [Source:VGNC Symbol;Acc:VGNC:76436]	-1.487393241	2.41E-16	5.82E-15
ENSMMUG00000008638	CDC42 binding protein kinase alpha [Source:VGNC Symbol;Acc:VGNC:70799]	0.378343623	0.002185926	0.007175141
ENSMMUG00000031497		-1.080874594	0.008585876	0.023737609
ENSMMUG00000008338	Wnt family member 9A [Source:VGNC Symbol;Acc:VGNC:78796]	0.698394211	0.000897513	0.003257742
ENSMMUG00000023096	obscurin, cytoskeletal calmodulin and titin-interacting RhoGEF [Source:NCBI gene;Acc:694413]	-1.094470778	3.01E-16	7.19E-15
ENSMMUG00000019513	U2AF homology motif kinase 1 [Source:VGNC Symbol;Acc:VGNC:79198]	2.241810666	0.004985142	0.014821502
ENSMMUG00000004049	activating transcription factor 6 [Source:VGNC Symbol;Acc:VGNC:70171]	0.331411247	0.004966086	0.014773733
ENSMMUG00000004042	Fc receptor like A [Source:VGNC Symbol;Acc:VGNC:72526]	1.531802144	0.000492663	0.001919147
ENSMMUG00000003775	Fc fragment of IgG receptor IIb [Source:NCBI gene;Acc:720009]	-0.934447971	0.007644878	0.021465446
ENSMMUG00000054686		2.023132292	4.14E-09	4.13E-08
ENSMMUG00000008251	cilia and flagella associated protein 126 [Source:VGNC Symbol;Acc:VGNC:71150]	2.103296453	0.000394474	0.001574709
ENSMMUG00000004516	apolipoprotein A2 [Source:NCBI gene;Acc:719946]	1.262627802	0.003278171	0.010269386
ENSMMUG00000004512	Fc fragment of IgE receptor Ig [Source:VGNC Symbol;Acc:VGNC:72629]	1.224380902	0.000112426	0.00050662
ENSMMUG00000004520	NADH:ubiquinone oxidoreductase core subunit S2 [Source:NCBI gene;Acc:719935]	0.620933142	0.001251477	0.00440005
ENSMMUG00000050419	ADAM metallopeptidase with thrombospondin type 1 motif 4 [Source:NCBI gene;Acc:719927]	1.041775779	5.55E-06	3.27E-05
ENSMMUG00000060555		0.805278262	0.000793053	0.00292416
ENSMMUG00000022630	protoporphyrinogen oxidase [Source:VGNC Symbol;Acc:VGNC:76365]	-1.717586485	1.38E-08	1.27E-07
ENSMMUG00000022631	ubiquitin specific peptidase 21 [Source:VGNC Symbol;Acc:VGNC:78741]	-1.451003113	0.000141392	0.000621654
ENSMMUG00000022632	ubiquitin-fold modifier conjugating enzyme 1 [Source:VGNC Symbol;Acc:VGNC:79643]	-1.406433487	9.98E-08	8.04E-07

ENSMMUG00000002010	death effector domain containing [Source:VGNC Symbol;Acc:VGNC:71768]	-1.273060814	3.56E-09	3.58E-08
ENSMMUG00000002009	nitrilase 1 [Source:VGNC Symbol;Acc:VGNC:75351]	-1.161029536	8.16E-07	5.63E-06
ENSMMUG00000060871	prefoldin subunit 2 [Source:NCBI gene;Acc:719886]	-1.218733616	3.92E-05	0.000194052
ENSMMUG00000002007	kelch domain containing 9 [Source:NCBI gene;Acc:719863]	-1.073108198	0.001602535	0.005459591
ENSMMUG00000064337	nectin cell adhesion molecule 4 [Source:NCBI gene;Acc:719843]	-1.192916159	7.83E-08	6.41E-07
ENSMMUG00000002005	Rho GTPase activating protein 30 [Source:NCBI gene;Acc:718236]	-0.881301835	0.00043396	0.001712514
ENSMMUG00000002003	upstream transcription factor 1 [Source:NCBI gene;Acc:719835]	-0.806800233	0.00596383	0.017291026
ENSMMUG00000013391	intelectin 1 [Source:NCBI gene;Acc:719807]	-0.75980563	0.003057929	0.009649276
ENSMMUG00000017333	CD244 molecule [Source:VGNC Symbol;Acc:VGNC:70821]	-0.780105279	0.001386076	0.004813935
ENSMMUG00000015812	CD48 molecule [Source:VGNC Symbol;Acc:VGNC:70769]	-0.600322088	0.000263128	0.001091637
ENSMMUG00000011338	SLAM family member 8 [Source:VGNC Symbol;Acc:VGNC:77545]	-1.085954493	7.60E-06	4.37E-05
ENSMMUG00000011335	Fc receptor like 6 [Source:VGNC Symbol;Acc:VGNC:72602]	-1.037976515	5.89E-15	1.26E-13
ENSMMUG00000018444	dual specificity phosphatase 23 [Source:VGNC Symbol;Acc:VGNC:71880]	-1.294877533	0.002336632	0.00761588
ENSMMUG00000060051	olfactory receptor 6N1-like [Source:NCBI gene;Acc:719970]	-0.647582741	0.012804136	0.033465868
ENSMMUG00000010626	CD1c molecule [Source:VGNC Symbol;Acc:VGNC:70756]	2.950264395	5.84E-09	5.67E-08
ENSMMUG00000062253	CD1a molecule [Source:NCBI gene;Acc:698438]	2.184077276	3.74E-05	0.000186376
ENSMMUG00000020156	CD1d molecule [Source:VGNC Symbol;Acc:VGNC:70757]	3.016555191	4.29E-07	3.10E-06
ENSMMUG00000020155		3.173950334	0.004601116	0.013843488
ENSMMUG00000003191	kirre like nephrin family adhesion molecule 1 [Source:VGNC Symbol;Acc:VGNC:73837]	-1.679728282	1.56E-13	2.83E-12
ENSMMUG00000050541		-1.197289054	0.005626408	0.01643483
ENSMMUG00000019835	CD5 molecule like [Source:VGNC Symbol;Acc:VGNC:70774]	1.117013182	8.37E-08	6.82E-07
ENSMMUG00000019833	Fc receptor like 1 [Source:VGNC Symbol;Acc:VGNC:72528]	1.53468351	1.13E-24	6.58E-23
ENSMMUG00000019830	Fc receptor like 2 [Source:VGNC Symbol;Acc:VGNC:72509]	1.748135489	8.61E-09	8.13E-08
ENSMMUG00000046969	Fc receptor like 3 [Source:NCBI gene;Acc:718801]	1.280796383	6.24E-11	8.11E-10
ENSMMUG00000008973	Fc receptor like 4 [Source:VGNC Symbol;Acc:VGNC:72527]	1.165309598	0.008295137	0.023067106
ENSMMUG00000008974	Fc receptor like 5 [Source:NCBI gene;Acc:718771]	-1.374909874	2.05E-05	0.000108794
ENSMMUG00000065257		-2.378811813	0.000380325	0.001523561
ENSMMUG00000042886	ETS variant transcription factor 3 [Source:NCBI gene;Acc:719741]	1.235717742	4.71E-06	2.82E-05
ENSMMUG00000012436	Rho guanine nucleotide exchange factor 11 [Source:VGNC Symbol;Acc:VGNC:69829]	0.33327486	5.10E-06	3.02E-05
ENSMMUG00000064007	leucine rich repeat containing 71 [Source:NCBI gene;Acc:718714]	0.282683499	0.013681657	0.035427442
ENSMMUG00000040660	heparin binding growth factor [Source:NCBI gene;Acc:716742]	0.927848569	0.002925381	0.009297772
ENSMMUG00000000039	mitochondrial ribosomal protein L24 [Source:VGNC Symbol;Acc:VGNC:74739]	1.459317448	0.000468144	0.001832936
ENSMMUG00000017093	brevican [Source:VGNC Symbol;Acc:VGNC:70112]	-1.843922317	0.018328041	0.04536886
ENSMMUG00000060011	hyaluronan and proteoglycan link protein 2 [Source:NCBI gene;Acc:718500]	-1.670478248	0.016491575	0.04147364
ENSMMUG00000017966	chromosome 1 C1orf61 homolog [Source:NCBI gene;Acc:106992893]	0.666771582	0.001076281	0.003846085

ENSMMUG00000019034	chaperonin containing TCP1 subunit 3 [Source:VGNC Symbol;Acc:VGNC:70746]	-1.592727357	0.000675763	0.002537748
ENSMMUG00000016096	lamin A/C [Source:VGNC Symbol;Acc:VGNC:74427]	1.811408374	1.77E-05	9.56E-05
ENSMMUG00000004648	KH domain containing 4, pre-mRNA splicing factor [Source:VGNC Symbol;Acc:VGNC:73931]	-1.166897404	5.95E-14	1.14E-12
ENSMMUG00000045167		-1.531029745	0.020508925	0.049876577
ENSMMUG00000053454	Ras like without CAAX 1 [Source:NCBI gene;Acc:717985]	-1.059572746	4.77E-05	0.000232906
ENSMMUG00000062397		1.39176744	5.77E-12	8.56E-11
ENSMMUG00000023145	RUN and SH3 domain containing 1 [Source:VGNC Symbol;Acc:VGNC:77036]	-1.349809266	0.0002415	0.001011424
ENSMMUG00000061381	hyperpolarization activated cyclic nucleotide gated potassium channel 3 [Source:NCBI gene;Acc:717719]	-1.573114469	0.00538604	0.015826227
ENSMMUG00000023141	CDC like kinase 2 [Source:VGNC Symbol;Acc:VGNC:71259]	-1.005230355	0.000381172	0.001525306
ENSMMUG00000053423		1.600553183	0.003344128	0.010445117
ENSMMUG00000003902	thrombospondin 3 [Source:VGNC Symbol;Acc:VGNC:78345]	1.858553022	7.38E-08	6.06E-07
ENSMMUG00000010978	S100 calcium binding protein A2 [Source:VGNC Symbol;Acc:VGNC:76956]	-1.089221668	1.34E-07	1.06E-06
ENSMMUG00000010979	S100 calcium binding protein A4 [Source:VGNC Symbol;Acc:VGNC:76958]	-0.745926086	1.98E-05	0.000105216
ENSMMUG00000010980	S100 calcium binding protein A5 [Source:VGNC Symbol;Acc:VGNC:76959]	-0.940694459	0.005132667	0.015171907
ENSMMUG00000008917	S100 calcium binding protein A6 [Source:VGNC Symbol;Acc:VGNC:76960]	-1.105733419	9.88E-07	6.70E-06
ENSMMUG00000040678	Metazoan signal recognition particle RNA [Source:RFAM;Acc:RF00017]	-1.910720746	0.003150966	0.009919089
ENSMMUG00000051645		2.814602746	4.79E-06	2.86E-05
ENSMMUG00000061474		1.729539874	9.01E-05	0.000415128
ENSMMUG00000042618	involucrin [Source:NCBI gene;Acc:713728]	1.359988317	0.000557771	0.002141276
ENSMMUG00000017740	sperm mitochondria associated cysteine rich protein [Source:VGNC Symbol;Acc:VGNC:77816]	2.049444447	0.000471207	0.001843312
ENSMMUG00000055038		2.026257952	2.82E-05	0.00014497
ENSMMUG00000054634	late cornified envelope protein 1C-like [Source:NCBI gene;Acc:713600]	2.365730087	0.002760672	0.008827245
ENSMMUG00000051719	late cornified envelope 1C [Source:HGNC Symbol;Acc:HGNC:29464]	1.747178452	0.004083095	0.012456758
ENSMMUG00000058472	late cornified envelope protein 1C [Source:NCBI gene;Acc:107000798]	1.421321398	0.00028646	0.00117451
ENSMMUG00000056940	late cornified envelope protein 1C [Source:NCBI gene;Acc:713408]	2.161109716	0.000849167	0.003105586
ENSMMUG00000055266	late cornified envelope protein 1C-like [Source:NCBI gene;Acc:107000794]	2.199659511	2.69E-05	0.000138868
ENSMMUG00000051816	keratinocyte proline rich protein [Source:NCBI gene;Acc:100424312]	1.963774445	3.20E-08	2.77E-07
ENSMMUG00000001966	chromosome 1 C1orf68 homolog [Source:VGNC Symbol;Acc:VGNC:70436]	1.737566908	0.014206535	0.036507176
ENSMMUG00000049843	late cornified envelope protein 2D [Source:NCBI gene;Acc:100424118]	2.702212282	0.005580171	0.016319056
ENSMMUG00000062996		2.127465209	1.59E-06	1.04E-05
ENSMMUG00000009040	cornulin [Source:VGNC Symbol;Acc:VGNC:71397]	2.376485894	1.93E-06	1.24E-05
ENSMMUG00000056511		1.843004079	1.48E-06	9.74E-06
ENSMMUG00000059472	filaggrin family member 2 [Source:HGNC Symbol;Acc:HGNC:33276]	1.819580901	4.11E-11	5.48E-10
ENSMMUG00000010067	MINDY lysine 48 deubiquitinase 1 [Source:VGNC Symbol;Acc:VGNC:74828]	-4.667042887	9.17E-06	5.19E-05
ENSMMUG00000023234	ceramide synthase 2 [Source:VGNC Symbol;Acc:VGNC:70946]	-2.934569883	0.000914239	0.003313334
ENSMMUG00000023235	SET domain bifurcated histone lysine methyltransferase 1 [Source:NCBI gene;Acc:716141]	-2.220693922	0.002093544	0.006922466
ENSMMUG00000012814	endosulfine alpha [Source:NCBI gene;Acc:715857]	-2.788247218	0.005935426	0.017222141
ENSMMUG00000008218	ADAMTS like 4 [Source:VGNC Symbol;Acc:VGNC:69480]	0.882060608	0.001885817	0.00632587

ENSMMUG00000021388	circadian associated repressor of transcription [Source:VGNC Symbol;Acc:VGNC:71345]	-1.65135635	2.53E-10	3.04E-09
ENSMMUG00000021386	aph-1 homolog A, gamma-secretase subunit [Source:VGNC Symbol;Acc:VGNC:69924]	-0.74182794	0.001197999	0.004231863
ENSMMUG00000021385	carbonic anhydrase 14 [Source:VGNC Symbol;Acc:VGNC:70489]	-1.015101631	1.01E-08	9.42E-08
ENSMMUG00000007244	OTU deubiquitinase 7B [Source:VGNC Symbol;Acc:VGNC:75634]	-1.271928246	1.08E-11	1.54E-10
ENSMMUG00000052282		-1.305891958	1.90E-09	2.00E-08
ENSMMUG00000007246	myotubularin related protein 11 [Source:VGNC Symbol;Acc:VGNC:75016]	-1.143548146	2.34E-06	1.48E-05
ENSMMUG00000007256	splicing factor 3b subunit 4 [Source:VGNC Symbol;Acc:VGNC:77182]	-1.442458768	2.16E-11	2.96E-10
ENSMMUG00000007257	synaptic vesicle glycoprotein 2A [Source:VGNC Symbol;Acc:VGNC:78181]	-1.285294192	1.65E-14	3.36E-13
ENSMMUG00000007258	bolA family member 1 [Source:VGNC Symbol;Acc:VGNC:70373]	-1.595019077	0.000519022	0.002009161
ENSMMUG00000061036	histone cluster 2, H2ab [Source:NCBI gene;Acc:705170]	-2.010238252	8.87E-05	0.00040974
ENSMMUG00000037540	histone cluster 2, H2ac [Source:NCBI gene;Acc:705054]	-1.571788646	0.000696493	0.002605041
ENSMMUG00000063990	Metazoan signal recognition particle RNA [Source:RFAM;Acc:RF00017]	-1.349772547	0.019758956	0.048344629
ENSMMUG00000052382	histone H4 [Source:NCBI gene;Acc:107000818]	-1.470136169	1.67E-10	2.05E-09
ENSMMUG00000054615		-1.256116461	2.77E-05	0.000142693
ENSMMUG00000051523	Metazoan signal recognition particle RNA [Source:RFAM;Acc:RF00017]	-1.675729476	2.28E-06	1.45E-05
ENSMMUG00000054078	histone H3 [Source:NCBI gene;Acc:114669830]	-1.267140719	0.000117369	0.000525539
ENSMMUG00000040506	H2B clustered histone 18 [Source:HGNC Symbol;Acc:HGNC:24700]	-0.809169873	0.019507809	0.047810413
ENSMMUG00000004362	Fc fragment of IgG receptor Ia [Source:NCBI gene;Acc:714664]	-1.220523119	3.11E-08	2.70E-07
ENSMMUG00000062140		-1.422895558	3.47E-08	2.97E-07
ENSMMUG00000024186	U1 spliceosomal RNA [Source:RFAM;Acc:RF00003]	-2.18575206	5.75E-07	4.07E-06
ENSMMUG00000036857	U1 spliceosomal RNA [Source:RFAM;Acc:RF00003]	-1.603282761	0.001682456	0.00570957
ENSMMUG00000061239		-0.627967456	0.011132818	0.029683511
ENSMMUG00000054590		-0.632387108	0.011363579	0.030233562
ENSMMUG00000056076	U1 spliceosomal RNA [Source:RFAM;Acc:RF00003]	-0.93394788	0.015435355	0.039149361
ENSMMUG00000055754	U1 spliceosomal RNA [Source:RFAM;Acc:RF00003]	-1.522581034	0.005007279	0.014866455
ENSMMUG00000056325	NBPF member 20 [Source:NCBI gene;Acc:708768]	-1.181983416	0.009504037	0.025899462
ENSMMUG00000018375	notch receptor 2 [Source:NCBI gene;Acc:713798]	-0.441950056	0.000103035	0.000469579
ENSMMUG00000017482	ADAM metallopeptidase domain 30 [Source:NCBI gene;Acc:713727]	-0.41946477	0.001438853	0.004972776
ENSMMUG00000008970	calsequestrin 2 [Source:VGNC Symbol;Acc:VGNC:70549]	0.687212212	2.69E-06	1.68E-05
ENSMMUG00000008967	VANGL planar cell polarity protein 1 [Source:VGNC Symbol;Acc:VGNC:78976]	0.797100734	2.50E-09	2.57E-08
ENSMMUG00000001973	suppressor of IKBKE 1 [Source:VGNC Symbol;Acc:VGNC:77314]	-2.872408647	0.001795572	0.006056023

ENSMMUG00000049105	NRAS proto-oncogene, GTPase [Source:NCBI gene;Acc:709089]	-1.521689058	0.000672378	0.002526314
ENSMMUG00000001978	adenosine monophosphate deaminase 1 [Source:VGNC Symbol;Acc:VGNC:69806]	-0.899822659	3.24E-08	2.80E-07
ENSMMUG00000014620	tripartite motif containing 33 [Source:VGNC Symbol;Acc:VGNC:79028]	1.128093924	0.003696814	0.011390765
ENSMMUG00000019528	adaptor related protein complex 4 subunit beta 1 [Source:VGNC Symbol;Acc:VGNC:69972]	2.7396611	1.19E-48	8.43E-46
ENSMMUG00000019527	DNA cross-link repair 1B [Source:VGNC Symbol;Acc:VGNC:71647]	1.78614995	2.39E-07	1.80E-06
ENSMMUG00000006787	BCL2 like 15 [Source:VGNC Symbol;Acc:VGNC:70162]	2.540951282	0.001311107	0.004586865
ENSMMUG00000006786	protein tyrosine phosphatase non-receptor type 22 [Source:VGNC Symbol;Acc:VGNC:76603]	1.565084738	1.44E-10	1.78E-09
ENSMMUG00000017823	round spermatid basic protein 1 [Source:VGNC Symbol;Acc:VGNC:77105]	-0.906772462	0.004408637	0.013318412
ENSMMUG00000017819	putative homeodomain transcription factor 1 [Source:VGNC Symbol;Acc:VGNC:75982]	-1.074449104	7.09E-06	4.10E-05
ENSMMUG00000017817	membrane associated guanylate kinase, WW and PDZ domain containing 3 [Source:VGNC Symbol;Acc:VGNC:74400]	0.665879151	8.21E-07	5.66E-06
ENSMMUG00000059489	Mov10 RISC complex RNA helicase [Source:NCBI gene;Acc:705910]	-4.11929694	0.001834378	0.006172866
ENSMMUG00000007766	suppression of tumorigenicity 7 like [Source:VGNC Symbol;Acc:VGNC:77954]	-1.991537556	2.47E-18	7.34E-17
ENSMMUG00000007765	Wnt family member 2B [Source:VGNC Symbol;Acc:VGNC:78797]	-1.85915237	4.19E-28	3.43E-26
ENSMMUG00000037458	endoplasmic reticulum resident protein 29 [Source:NCBI gene;Acc:720396]	-2.555147235	3.70E-05	0.000184687
ENSMMUG00000050825	potassium voltage-gated channel subfamily D member 3 [Source:NCBI gene;Acc:641437]	-0.485579731	0.000764338	0.002826717
ENSMMUG00000014489	DEAD-box helicase 20 [Source:NCBI gene;Acc:704731]	0.808108777	7.79E-10	8.70E-09
ENSMMUG00000052184	inka box actin regulator 2 [Source:NCBI gene;Acc:704844]	1.292385238	0.000687986	0.002579077
ENSMMUG00000003581	transmembrane and immunoglobulin domain containing 3 [Source:NCBI gene;Acc:104564310]	0.55737006	0.005669305	0.016534079
ENSMMUG00000065322	ATP synthase peripheral stalk-membrane subunit b [Source:NCBI gene;Acc:704072]	-0.840415021	8.56E-07	5.88E-06
ENSMMUG00000054017	potassium voltage-gated channel subfamily A member 2 [Source:NCBI gene;Acc:702126]	0.881844941	8.84E-05	0.000408288
ENSMMUG00000016056	potassium voltage-gated channel subfamily A member 10 [Source:VGNC Symbol;Acc:VGNC:73963]	0.835893729	8.58E-06	4.88E-05
ENSMMUG00000062177		0.681237996	3.21E-08	2.78E-07
ENSMMUG00000004614	potassium voltage-gated channel subfamily C member 4 [Source:VGNC Symbol;Acc:VGNC:73974]	0.963139795	0.015960713	0.040323232
ENSMMUG00000022715	adenosylhomocysteinase like 1 [Source:VGNC Symbol;Acc:VGNC:81350]	-0.954706779	2.86E-07	2.13E-06
ENSMMUG00000005820	adenosine monophosphate deaminase 2 [Source:VGNC Symbol;Acc:VGNC:69807]	-0.935532198	0.010678489	0.028590359
ENSMMUG00000051186	proteasome 20S subunit alpha 5 [Source:NCBI gene;Acc:698435]	0.934577585	1.41E-12	2.28E-11
ENSMMUG00000008713	myosin binding protein H like [Source:NCBI gene;Acc:698183]	-0.860702296	1.59E-08	1.44E-07
ENSMMUG00000021837	seryl-tRNA synthetase 1 [Source:NCBI gene;Acc:695046]	-0.839014353	8.57E-08	6.96E-07
ENSMMUG00000017266	UPF0577 protein KIAA1324 [Source:NCBI gene;Acc:697670]	1.73470575	3.04E-08	2.65E-07
ENSMMUG00000009431	HEN methyltransferase 1 [Source:VGNC Symbol;Acc:VGNC:73241]	-2.288823673	0.00786733	0.022005377
ENSMMUG00000042232	NBPF member 6 [Source:NCBI gene;Acc:698524]	0.439706248	0.000270075	0.001116688
ENSMMUG00000002789	solute carrier family 25 (mitochondrial carrier; phosphate carrier), member 24 [Source:NCBI gene;Acc:694803]	2.838584265	3.87E-23	1.94E-21
ENSMMUG00000019714	vav guanine nucleotide exchange factor 3 [Source:VGNC Symbol;Acc:VGNC:78759]	-2.123553192	0.000123554	0.000551567
ENSMMUG00000008197	netrin G1 [Source:VGNC Symbol;Acc:VGNC:75450]	2.085414931	1.52E-05	8.27E-05
ENSMMUG00000060795		1.984797578	3.06E-12	4.75E-11
ENSMMUG00000004103	collagen type XI alpha 1 chain [Source:VGNC Symbol;Acc:VGNC:71289]	2.392138129	1.87E-69	6.93E-66
ENSMMUG00000060790		-3.724945356	0.000170165	0.000734443
ENSMMUG00000022906	vascular cell adhesion molecule 1 [Source:VGNC Symbol;Acc:VGNC:78761]	-0.727025	2.49E-07	1.87E-06

ENSMMUG00000062643		-2.139953798	0.002902809	0.00923791
ENSMMUG00000055443	G protein-coupled receptor 88 [Source:NCBI gene;Acc:716520]	0.398932625	0.007849951	0.021965051
ENSMMUG00000019106	cell division cycle 14A [Source:VGNC Symbol;Acc:VGNC:70791]	-0.417369344	0.007900114	0.022080418
ENSMMUG00000019105	RNA 3'-terminal phosphate cyclase [Source:VGNC Symbol;Acc:VGNC:77043]	-2.039187607	0.000111816	0.000504327
ENSMMUG00000007706	dihydrolipoamide branched chain transacylase E2 [Source:VGNC Symbol;Acc:VGNC:71744]	-2.730176814	0.001215227	0.004289887
ENSMMUG00000000931	solute carrier family 35 member A3 [Source:VGNC Symbol;Acc:VGNC:77461]	1.804901273	2.77E-08	2.43E-07
ENSMMUG00000021441	amylo-alpha-1, 6-glucosidase, 4-alpha-glucanotransferase [Source:VGNC Symbol;Acc:VGNC:69744]	-0.579032616	0.00011273	0.000507527
ENSMMUG00000016335	palmdelphin [Source:VGNC Symbol;Acc:VGNC:75613]	-1.157214069	0.008374179	0.023256381
ENSMMUG00000038667		-1.572782155	9.35E-05	0.000429589
ENSMMUG00000008518	phospholipid phosphatase related 5 [Source:NCBI gene;Acc:710549]	1.634269329	5.58E-19	1.73E-17
ENSMMUG00000008515	sorting nexin 7 [Source:NCBI gene;Acc:716015]	1.045910292	3.22E-12	4.98E-11
ENSMMUG00000049701		0.416016121	0.00487907	0.014558675
ENSMMUG00000049494		1.651632933	1.73E-06	1.12E-05
ENSMMUG00000020726	dihydropyrimidine dehydrogenase [Source:VGNC Symbol;Acc:VGNC:71982]	2.347221136	3.32E-30	3.31E-28
ENSMMUG00000049297		1.462069084	4.74E-18	1.37E-16
ENSMMUG00000050661		1.166321282	2.06E-10	2.50E-09
ENSMMUG00000001690	calponin 3 [Source:VGNC Symbol;Acc:VGNC:71278]	1.050381637	1.08E-09	1.18E-08
ENSMMUG00000056548	coagulation factor III, tissue factor [Source:NCBI gene;Acc:714456]	0.984555433	1.05E-12	1.72E-11
ENSMMUG00000014013	ATP binding cassette subfamily D member 3 [Source:VGNC Symbol;Acc:VGNC:69579]	0.580955529	3.17E-07	2.33E-06
ENSMMUG00000013858	ATP binding cassette subfamily A member 4 [Source:VGNC Symbol;Acc:VGNC:69563]	0.411330075	1.38E-07	1.09E-06
ENSMMUG00000032677	microRNA 760 [Source:HGNC Symbol;Acc:HGNC:33666]	0.693202351	0.001415739	0.004904324
ENSMMUG00000045959		1.349903741	2.94E-07	2.18E-06
ENSMMUG00000015924	BCAR3 adaptor protein, NSP family member [Source:VGNC Symbol;Acc:VGNC:70235]	1.328772261	8.31E-13	1.39E-11
ENSMMUG00000015922	formin binding protein 1 like [Source:VGNC Symbol;Acc:VGNC:72543]	1.431068905	1.76E-14	3.57E-13
ENSMMUG00000016812	coiled-coil domain containing 18 [Source:VGNC Symbol;Acc:VGNC:70913]	-1.012861231	7.22E-05	0.000339174
ENSMMUG00000005070	ecotropic viral integration site 5 [Source:NCBI gene;Acc:706592]	2.304982691	2.66E-22	1.22E-20
ENSMMUG00000014018	bromodomain testis associated [Source:VGNC Symbol;Acc:VGNC:70282]	-1.693844558	2.56E-05	0.000132791
ENSMMUG00000001742	transforming growth factor beta receptor 3 [Source:VGNC Symbol;Acc:VGNC:78339]	-2.590656924	2.22E-26	1.53E-24
ENSMMUG00000051155	U2 spliceosomal RNA [Source:RFAM;Acc:RF00004]	-3.304647581	0.000191174	0.000818923
ENSMMUG00000006819	helicase for meiosis 1 [Source:NCBI gene;Acc:704516]	0.922257592	6.49E-05	0.000308074
ENSMMUG00000049678		2.489790285	1.04E-13	1.93E-12
ENSMMUG00000060600		-1.691050167	1.83E-05	9.79E-05
ENSMMUG00000053737	BarH like homeobox 2 [Source:NCBI gene;Acc:696831]	-0.561297015	0.011450229	0.030431344
ENSMMUG00000000663	guanylate-binding protein 6-like [Source:NCBI gene;Acc:695158]	-1.711467329	2.95E-09	3.02E-08
ENSMMUG00000000662	guanylate-binding protein 1 [Source:NCBI gene;Acc:694538]	-1.023073297	5.01E-09	4.92E-08
ENSMMUG00000050170		1.008290257	0.00955491	0.026004647
ENSMMUG00000018795	kynurenine aminotransferase 3 [Source:VGNC Symbol;Acc:VGNC:74177]	-1.006415292	0.010389659	0.027953309
ENSMMUG00000037887	RBMX like 1 [Source:NCBI gene;Acc:100424767]	0.979945054	3.30E-09	3.35E-08

ENSMMUG00000002794	general transcription factor IIB [Source:VGNC Symbol;Acc:VGNC:73306]	-2.775236215	9.00E-33	1.18E-30
ENSMMUG00000020588	protein kinase N2 [Source:VGNC Symbol;Acc:VGNC:76015]	0.980825952	1.52E-17	4.19E-16
ENSMMUG00000065295		2.791188488	1.94E-31	2.06E-29
ENSMMUG00000010306	calcium-activated chloride channel regulator 4 [Source:NCBI gene;Acc:712123]	-1.234083756	0.000475332	0.001858471
ENSMMUG00000006836	chloride channel accessory 1 [Source:NCBI gene;Acc:574295]	2.080479344	3.89E-12	5.90E-11
ENSMMUG00000001380	outer dense fiber of sperm tails 2 like [Source:VGNC Symbol;Acc:VGNC:75482]	1.723838961	0.000827983	0.003037099
ENSMMUG00000013203	zinc finger HIT-type containing 6 [Source:VGNC Symbol;Acc:VGNC:79576]	-0.697208406	0.011693295	0.030982949
ENSMMUG00000014573	dimethylarginine dimethylaminohydrolase 1 [Source:VGNC Symbol;Acc:VGNC:71753]	1.049928346	4.82E-16	1.13E-14
ENSMMUG00000005361	mucolipin 3 [Source:VGNC Symbol;Acc:VGNC:74505]	0.826756794	0.004388893	0.013266873
ENSMMUG00000015402	lysophosphatidic acid receptor 3 [Source:VGNC Symbol;Acc:VGNC:74287]	1.014685494	0.001228607	0.004328877
ENSMMUG00000010127	chitobiase [Source:VGNC Symbol;Acc:VGNC:71540]	-1.922607292	1.37E-08	1.26E-07
ENSMMUG00000022183	spermatogenesis associated 1 [Source:NCBI gene;Acc:710462]	-1.984742895	1.83E-23	9.66E-22
ENSMMUG00000059938		-2.18240135	4.33E-09	4.30E-08
ENSMMUG00000000404	uricase [Source:NCBI gene;Acc:714004]	-1.251199423	0.005609656	0.016392354
ENSMMUG00000003457	protein kinase cAMP-activated catalytic subunit beta [Source:NCBI gene;Acc:709728]	1.453457072	1.74E-25	1.07E-23
ENSMMUG00000057763		-1.661154682	1.06E-05	5.94E-05
ENSMMUG00000050832		3.523089113	2.74E-05	0.000141169
ENSMMUG00000023345	adhesion G protein-coupled receptor L4 [Source:VGNC Symbol;Acc:VGNC:69652]	1.638661313	9.27E-07	6.32E-06
ENSMMUG00000049326	GIPC PDZ domain containing family member 2 [Source:NCBI gene;Acc:707432]	-0.636836948	0.001057265	0.003782689
ENSMMUG00000050425	DnaJ heat shock protein family (Hsp40) member B4 [Source:NCBI gene;Acc:707206]	-0.503647595	0.001254254	0.004406676
ENSMMUG00000013853	ankyrin repeat domain 13C [Source:VGNC Symbol;Acc:VGNC:69695]	0.326418824	0.001715572	0.005809988
ENSMMUG00000021917	leucine rich repeat containing 7 [Source:NCBI gene;Acc:702347]	-2.317209291	0.000613551	0.002328887
ENSMMUG00000041837	Metazoan signal recognition particle RNA [Source:RFAM;Acc:RF00017]	-2.31610749	7.37E-06	4.25E-05
ENSMMUG00000056103	growth arrest and DNA damage inducible alpha [Source:NCBI gene;Acc:701054]	-1.637137749	5.36E-18	1.54E-16
ENSMMUG00000003265	leptin receptor [Source:VGNC Symbol;Acc:VGNC:81429]	0.913731485	6.34E-12	9.33E-11
ENSMMUG00000053254	adenylate kinase 4 [Source:NCBI gene;Acc:698560]	1.291548867	1.86E-16	4.53E-15
ENSMMUG00000053072		1.192444373	6.40E-08	5.32E-07
ENSMMUG00000018566	Janus kinase 1 [Source:VGNC Symbol;Acc:VGNC:73810]	0.892831128	0.006522462	0.018680594
ENSMMUG00000018565	ribonucleoprotein, PTB binding 2 [Source:VGNC Symbol;Acc:VGNC:76536]	-3.814580019	4.59E-09	4.53E-08
ENSMMUG00000065340		-2.826496671	0.000653067	0.002463043
ENSMMUG00000012538	ubiquitin conjugating enzyme E2 U [Source:VGNC Symbol;Acc:VGNC:79726]	0.930953478	9.47E-06	5.34E-05
ENSMMUG00000020049	phosphoglucomutase 1 [Source:NCBI gene;Acc:699401]	-0.860683436	6.94E-08	5.72E-07
ENSMMUG00000020047	integrin subunit beta 3 binding protein [Source:VGNC Symbol;Acc:VGNC:73787]	-1.021656522	5.97E-09	5.80E-08
ENSMMUG00000010351	KN motif and ankyrin repeat domains 4 [Source:VGNC Symbol;Acc:VGNC:73916]	-0.595600278	0.000506757	0.001969394
ENSMMUG00000049952		0.998010257	8.53E-13	1.42E-11
ENSMMUG00000055834		1.631741389	0.000305478	0.001246969
ENSMMUG00000022762	OMA1 zinc metallopeptidase [Source:VGNC Symbol;Acc:VGNC:75485]	-1.189888471	9.20E-06	5.20E-05

ENSMMUG00000010406	protein kinase AMP-activated catalytic subunit alpha 2 [Source:VGNC Symbol;Acc:VGNC:76400]	-4.179744406	3.89E-05	0.000192813
ENSMMUG00000002200	phospholipid phosphatase 3 [Source:NCBI gene;Acc:715993]	-2.034053739	1.84E-30	1.85E-28
ENSMMUG00000059612		0.485138913	0.000346974	0.001400551
ENSMMUG00000005740	ubiquitin specific peptidase 24 [Source:VGNC Symbol;Acc:VGNC:78743]	-0.538476435	0.00335753	0.010480355
ENSMMUG00000006754	tetratricopeptide repeat domain 4 [Source:VGNC Symbol;Acc:VGNC:78668]	-0.942952411	6.33E-07	4.46E-06
ENSMMUG00000019056	Fas associated factor 1 [Source:VGNC Symbol;Acc:VGNC:72474]	-0.52153773	0.004972035	0.014785499
ENSMMUG00000008828	BEN domain containing 5 [Source:NCBI gene;Acc:711150]	2.070788882	1.31E-13	2.40E-12
ENSMMUG00000015371	microtubule associated serine/threonine kinase 2 [Source:VGNC Symbol;Acc:VGNC:74532]	-1.368453693	3.02E-07	2.23E-06
ENSMMUG00000028192	Y RNA [Source:RFAM;Acc:RF00019]	2.707435278	0.008134759	0.022672144
ENSMMUG00000005986	Kruppel like factor 17 [Source:NCBI gene;Acc:702810]	2.168141916	1.24E-07	9.86E-07
ENSMMUG00000015555	ST3 beta-galactoside alpha-2,3-sialyltransferase 3 [Source:VGNC Symbol;Acc:VGNC:77828]	3.155057041	1.58E-21	6.74E-20
ENSMMUG00000053498		1.885331083	0.000437314	0.001724374
ENSMMUG00000012477	protein tyrosine phosphatase receptor type F [Source:VGNC Symbol;Acc:VGNC:76615]	-0.339239592	0.006618238	0.018925659
ENSMMUG00000016111	chromosome 1 C1orf50 homolog [Source:VGNC Symbol;Acc:VGNC:70432]	2.271218018	0.000411834	0.001636961
ENSMMUG00000022381	prolyl 3-hydroxylase 1 [Source:VGNC Symbol;Acc:VGNC:75648]	2.360609737	0.000861228	0.003143492
ENSMMUG00000057076	claudin 19 [Source:NCBI gene;Acc:697807]	4.163500932	0.000250088	0.001044145
ENSMMUG00000013129	peptidylprolyl isomerase H [Source:NCBI gene;Acc:697669]	1.533976089	0.001223857	0.00431419
ENSMMUG00000013128	coiled-coil domain containing 30 [Source:VGNC Symbol;Acc:VGNC:70866]	0.669083166	9.17E-05	0.00042199
ENSMMUG00000014138	forkhead box J3 [Source:VGNC Symbol;Acc:VGNC:72596]	2.418663843	5.68E-13	9.69E-12
ENSMMUG00000064973		0.685732342	3.28E-08	2.82E-07
ENSMMUG00000052046		1.022291041	1.96E-12	3.11E-11
ENSMMUG00000052537		-1.955998287	0.000206661	0.000878918
ENSMMUG00000044175	forkhead box O6 [Source:NCBI gene;Acc:697505]	-1.608951118	1.00E-15	2.28E-14
ENSMMUG00000043675	Cbp/p300 interacting transactivator with Glu/Asp rich carboxy-terminal domain 4 [Source:NCBI gene;Acc:114672840]	2.557371309	2.01E-34	3.20E-32
ENSMMUG00000019536	potassium voltage-gated channel subfamily Q member 4 [Source:VGNC Symbol;Acc:VGNC:74007]	1.303384184	1.88E-15	4.16E-14
ENSMMUG00000018664	collagen type IX alpha 2 chain [Source:VGNC Symbol;Acc:VGNC:71307]	2.56433402	2.96E-29	2.74E-27
ENSMMUG00000005992	zinc metallopeptidase STE24 [Source:VGNC Symbol;Acc:VGNC:78830]	2.45624796	6.14E-17	1.57E-15
ENSMMUG00000052510	transmembrane and coiled-coil domains 2 [Source:NCBI gene;Acc:693977]	2.364205195	0.013565202	0.035163772
ENSMMUG00000032180	RLF zinc finger [Source:VGNC Symbol;Acc:VGNC:76699]	1.840811741	1.36E-12	2.20E-11
ENSMMUG00000009189	palmitoyl-protein thioesterase 1 [Source:VGNC Symbol;Acc:VGNC:76386]	-0.379683424	0.001082146	0.003865179
ENSMMUG00000063318		-1.388351021	0.007751608	0.021730877
ENSMMUG00000009562	microtubule actin crosslinking factor 1 [Source:NCBI gene;Acc:718651]	1.211614116	0.006496575	0.018617237
ENSMMUG00000044406	POU class 3 homeobox 1 [Source:NCBI gene;Acc:718015]	-0.843772785	0.010470652	0.02814059
ENSMMUG00000061825	U6 spliceosomal RNA [Source:RFAM;Acc:RF00026]	0.894177615	2.50E-06	1.57E-05
ENSMMUG00000023277	inositol polyphosphate-5-phosphatase B [Source:VGNC Symbol;Acc:VGNC:73750]	-0.515451312	0.002116093	0.006984984
ENSMMUG00000022040	mannosidase endo-alpha like [Source:NCBI gene;Acc:716710]	-0.418224129	0.006797033	0.019377162
ENSMMUG00000008419	G protein nucleolar 2 [Source:VGNC Symbol;Acc:VGNC:73037]	-1.817756313	1.53E-05	8.35E-05
ENSMMUG00000008418	dynein axonemal light intermediate chain 1 [Source:VGNC Symbol;Acc:VGNC:71836]	-1.555425279	0.010819462	0.028926019

ENSMMUG00000059855	Smad nuclear interacting protein 1 [Source:NCBI gene;Acc:713733]	-1.906943063	1.03E-09	1.13E-08
ENSMMUG00000010328	MYST/Esa1 associated factor 6 [Source:VGNC Symbol;Acc:VGNC:74580]	-1.654408045	3.53E-05	0.000177391
ENSMMUG00000010323	zinc finger CCCH-type containing 12A [Source:VGNC Symbol;Acc:VGNC:79595]	-1.244828079	4.22E-05	0.000208046
ENSMMUG00000050060		0.496923945	0.000708612	0.002643704
ENSMMUG00000020432	organic solute carrier partner 1 [Source:VGNC Symbol;Acc:VGNC:75696]	0.896105372	0.003634737	0.011229823
ENSMMUG00000017338	LSM10, U7 small nuclear RNA associated [Source:VGNC Symbol;Acc:VGNC:74307]	0.994832896	0.006573181	0.018814953
ENSMMUG00000006252	argonaute RISC catalytic component 3 [Source:NCBI gene;Acc:712543]	-1.150708103	7.48E-10	8.38E-09
ENSMMUG00000006251	argonaute RISC component 1 [Source:VGNC Symbol;Acc:VGNC:81346]	-0.799030548	6.95E-05	0.000327474
ENSMMUG00000019815	transcription factor AP-2 epsilon [Source:VGNC Symbol;Acc:VGNC:79878]	-2.070777563	3.29E-08	2.83E-07
ENSMMUG00000002741	CUB and Sushi multiple domains 2 [Source:VGNC Symbol;Acc:VGNC:71441]	3.518296111	0.000399384	0.001590457
ENSMMUG00000003269	polyhomeotic homolog 2 [Source:VGNC Symbol;Acc:VGNC:75903]	2.684398922	0.000139128	0.000613596
ENSMMUG00000003268	alpha 1,3-galactosyltransferase 2 [Source:VGNC Symbol;Acc:VGNC:69550]	1.149395183	0.012376028	0.032530396
ENSMMUG00000003267	zinc finger protein 362 [Source:NCBI gene;Acc:710013]	1.213640693	3.31E-11	4.44E-10
ENSMMUG00000015097	tripartite motif containing 62 [Source:VGNC Symbol;Acc:VGNC:79513]	1.030544731	2.99E-07	2.22E-06
ENSMMUG00000006285	antizyme inhibitor 2 [Source:VGNC Symbol;Acc:VGNC:70153]	1.10064186	6.55E-15	1.39E-13
ENSMMUG00000006284	adenylate kinase 2 [Source:VGNC Symbol;Acc:VGNC:69761]	1.36659225	8.76E-14	1.64E-12
ENSMMUG00000044265	Metazoan signal recognition particle RNA [Source:RFAM;Acc:RF00017]	1.02241794	2.51E-06	1.58E-05
ENSMMUG00000054846	Metazoan signal recognition particle RNA [Source:RFAM;Acc:RF00017]	1.435302253	2.47E-05	0.00012879
ENSMMUG00000001264	BSD domain containing 1 [Source:VGNC Symbol;Acc:VGNC:70384]	-3.528275103	9.45E-06	5.33E-05
ENSMMUG00000009948	histone deacetylase 1 [Source:NCBI gene;Acc:708441]	-2.050877688	9.79E-20	3.32E-18
ENSMMUG00000040694	LCK proto-oncogene, Src family tyrosine kinase [Source:NCBI gene;Acc:717704]	-1.776941213	4.20E-23	2.10E-21
ENSMMUG00000015650	transmembrane protein 39B [Source:VGNC Symbol;Acc:VGNC:79507]	-0.447280141	0.016154149	0.040763319
ENSMMUG00000060894	Metazoan signal recognition particle RNA [Source:RFAM;Acc:RF00017]	-1.116435621	3.22E-06	1.98E-05
ENSMMUG00000005466	SPOC domain containing 1 [Source:VGNC Symbol;Acc:VGNC:77997]	-1.190941361	1.65E-08	1.50E-07
ENSMMUG00000023401	adhesion G protein-coupled receptor B2 [Source:VGNC Symbol;Acc:VGNC:69598]	-1.301127603	1.49E-11	2.08E-10
ENSMMUG00000023398	collagen type XVI alpha 1 chain [Source:VGNC Symbol;Acc:VGNC:71293]	-1.227220258	6.11E-13	1.04E-11
ENSMMUG00000023397	penta-EF-hand domain containing 1 [Source:NCBI gene;Acc:706038]	-0.997577027	2.74E-09	2.80E-08
ENSMMUG00000023396	hypocretin receptor 1 [Source:VGNC Symbol;Acc:VGNC:73356]	-0.986891092	9.55E-06	5.38E-05
ENSMMUG00000060319	Metazoan signal recognition particle RNA [Source:RFAM;Acc:RF00017]	-1.111659647	4.75E-05	0.000232131
ENSMMUG00000013188	serine incorporator 2 [Source:VGNC Symbol;Acc:VGNC:77294]	-0.982194348	7.88E-10	8.79E-09
ENSMMUG00000049574	antizyme inhibitor 2-like [Source:NCBI gene;Acc:705256]	-0.696604148	3.98E-07	2.89E-06
ENSMMUG00000057282	Y RNA [Source:RFAM;Acc:RF00019]	-1.031458489	0.000431949	0.001707759
ENSMMUG00000064353	fatty acid binding protein 3 [Source:NCBI gene;Acc:717237]	-0.511122912	0.000725466	0.00269708

ENSMMUG00000019406	small nuclear ribonucleoprotein U5 subunit 40 [Source:NCBI gene;Acc:705059]	-0.527576278	0.009931236	0.026875954
ENSMMUG00000013520	pumilio RNA binding family member 1 [Source:VGNC Symbol;Acc:VGNC:76479]	0.862615918	1.45E-11	2.04E-10
ENSMMUG00000063979		-0.621390733	3.11E-09	3.17E-08
ENSMMUG00000025533		-2.389664405	0.0046481	0.013948063
ENSMMUG00000008744	lysosomal protein transmembrane 5 [Source:VGNC Symbol;Acc:VGNC:74162]	1.388153658	0.019075617	0.046882898
ENSMMUG00000057452		1.181284581	2.30E-07	1.74E-06
ENSMMUG00000059136		1.088831086	0.015334106	0.038959191
ENSMMUG00000051008		-3.467745536	0.000196323	0.000838801
ENSMMUG00000057173		-3.48494634	7.16E-05	0.000336224
ENSMMUG00000015661	protein tyrosine phosphatase receptor type U [Source:VGNC Symbol;Acc:VGNC:76624]	-2.901119592	2.88E-11	3.89E-10
ENSMMUG00000015659	mitochondrial trans-2-enoyl-CoA reductase [Source:VGNC Symbol;Acc:VGNC:74536]	-3.690640726	0.000706136	0.00263579
ENSMMUG00000007110	serine and arginine rich splicing factor 4 [Source:NCBI gene;Acc:717401]	-2.564158309	0.000135479	0.000599566
ENSMMUG00000016893	solute carrier family 9 member A1 [Source:VGNC Symbol;Acc:VGNC:82241]	-1.555150869	3.28E-08	2.83E-07
ENSMMUG00000064416	Metazoan signal recognition particle RNA [Source:RFAM;Acc:RF00017]	0.826573466	0.003862624	0.011852413
ENSMMUG00000015115	terminal nucleotidyltransferase 5B [Source:VGNC Symbol;Acc:VGNC:79692]	1.142206648	1.00E-11	1.43E-10
ENSMMUG00000021031	TMF1 regulated nuclear protein 1 [Source:VGNC Symbol;Acc:VGNC:79946]	0.945627822	6.39E-10	7.23E-09
ENSMMUG00000050286		0.65564302	0.001946926	0.006502908
ENSMMUG00000058432	zinc finger DHHC-type palmitoyltransferase 18 [Source:NCBI gene;Acc:719959]	2.323504982	9.12E-25	5.37E-23
ENSMMUG00000012697	AT-rich interaction domain 1A [Source:VGNC Symbol;Acc:VGNC:69822]	1.727953008	8.28E-11	1.06E-09
ENSMMUG00000056266	Metazoan signal recognition particle RNA [Source:RFAM;Acc:RF00017]	1.894546043	1.07E-07	8.63E-07
ENSMMUG00000000649	UBX domain protein 11 [Source:VGNC Symbol;Acc:VGNC:79859]	1.671399447	1.68E-06	1.10E-05
ENSMMUG00000000644	centrosomal protein 85 [Source:VGNC Symbol;Acc:VGNC:71096]	1.893202459	4.09E-09	4.08E-08
ENSMMUG00000011156	Rh blood group, CcEe antigens [Source:NCBI gene;Acc:613023]	0.595007728	0.010561835	0.028349686
ENSMMUG00000057953		1.293417012	0.000666528	0.00250624
ENSMMUG00000002420	RUNX family transcription factor 3 [Source:VGNC Symbol;Acc:VGNC:76948]	-1.022140148	0.004201256	0.012772576
ENSMMUG00000057675	non-compact myelin associated protein [Source:NCBI gene;Acc:712192]	1.199722934	0.000220068	0.000930068
ENSMMUG00000058954		2.660824884	0.002942376	0.009341776
ENSMMUG00000053036		-1.433463015	3.74E-10	4.35E-09
ENSMMUG00000049609		-1.557606179	0.000116189	0.000521198
ENSMMUG00000022927	RCAN family member 3 [Source:VGNC Symbol;Acc:VGNC:76875]	-2.021380054	1.04E-17	2.91E-16
ENSMMUG00000050770		-0.80160018	0.000356656	0.001436179
ENSMMUG00000059931		-0.641085525	0.000245222	0.001025853
ENSMMUG00000004217	cannabinoid receptor 2 [Source:VGNC Symbol;Acc:VGNC:71281]	-0.727514151	0.012669273	0.033165987
ENSMMUG00000004216	alpha-L-fucosidase 1 [Source:VGNC Symbol;Acc:VGNC:72826]	-0.701254235	0.020322464	0.049528701
ENSMMUG00000019549	UDP-galactose-4-epimerase [Source:VGNC Symbol;Acc:VGNC:72868]	-3.170061424	0.008959786	0.02462976
ENSMMUG00000010970	heterogeneous nuclear ribonucleoprotein R [Source:VGNC Symbol;Acc:VGNC:73499]	-2.712965568	0.007629687	0.021429593
ENSMMUG00000045584	leucine zipper protein 1 [Source:NCBI gene;Acc:718626]	-1.803997807	1.06E-14	2.20E-13

ENSMMUG00000009773	lysine demethylase 1A [Source:VGNC Symbol;Acc:VGNC:73928]	-1.90892778	2.06E-09	2.16E-08
ENSMMUG00000058332	testis expressed 46 [Source:NCBI gene;Acc:709002]	-2.143724394	7.56E-07	5.25E-06
ENSMMUG00000041482	lactamase beta like 1 [Source:NCBI gene;Acc:718590]	-1.271376962	0.000280624	0.00115443
ENSMMUG00000023382	EPH receptor B2 [Source:NCBI gene;Acc:718570]	0.344882826	0.003451843	0.010727332
ENSMMUG00000002217	alkaline phosphatase, biomineralization associated [Source:VGNC Symbol;Acc:VGNC:69801]	1.590023358	1.85E-25	1.14E-23
ENSMMUG00000004149	eukaryotic translation initiation factor 4 gamma 3 [Source:NCBI gene;Acc:717555]	1.690562764	5.95E-27	4.51E-25
ENSMMUG00000006891	aflatoxin B1 aldehyde reductase member 3 [Source:NCBI gene;Acc:715623]	-0.63504482	0.017553343	0.043713866
ENSMMUG00000064473	MRT4 homolog, ribosome maturation factor [Source:NCBI gene;Acc:702926]	-0.928913184	0.000154762	0.000674616
ENSMMUG00000006688	ER membrane protein complex subunit 1 [Source:VGNC Symbol;Acc:VGNC:72134]	-1.447522691	2.13E-13	3.79E-12
ENSMMUG00000050018		0.552789078	0.020558154	0.049970012
ENSMMUG00000004754	intermediate filament family orphan 2 [Source:NCBI gene;Acc:106995162]	-1.174501338	2.10E-08	1.88E-07
ENSMMUG00000023517	immunoglobin superfamily member 21 [Source:VGNC Symbol;Acc:VGNC:73668]	0.614473114	0.010916463	0.029164321
ENSMMUG00000032238	peptidyl arginine deiminase 4 [Source:NCBI gene;Acc:700806]	1.248827639	0.006398438	0.018385747
ENSMMUG00000051619	peptidyl arginine deiminase 3 [Source:NCBI gene;Acc:700560]	0.950212051	2.10E-11	2.89E-10
ENSMMUG00000050983	peptidyl arginine deiminase 1 [Source:NCBI gene;Acc:700444]	0.666754648	1.21E-05	6.71E-05
ENSMMUG00000001616	peptidyl arginine deiminase 2 [Source:VGNC Symbol;Acc:VGNC:75644]	1.15845387	2.85E-05	0.000146299
ENSMMUG00000018245	EPH receptor A2 [Source:VGNC Symbol;Acc:VGNC:72235]	1.326639823	7.37E-10	8.28E-09
ENSMMUG00000058587	family with sequence similarity 131 member C [Source:NCBI gene;Acc:703604]	1.376682322	1.95E-15	4.31E-14
ENSMMUG00000018235	chloride voltage-gated channel Kb [Source:NCBI gene;Acc:703476]	0.925517	0.000795389	0.002931318
ENSMMUG00000001910	heat shock protein family B (small) member 7 [Source:NCBI gene;Acc:696704]	1.347166613	8.64E-05	0.000399943
ENSMMUG00000001911	steroid receptor associated and regulated protein [Source:VGNC Symbol;Acc:VGNC:78008]	0.701561161	0.001237622	0.004356241
ENSMMUG00000008031	zinc finger and BTB domain containing 17 [Source:VGNC Symbol;Acc:VGNC:78849]	1.253222586	7.82E-09	7.45E-08
ENSMMUG00000002802	agmatinase [Source:NCBI gene;Acc:713190]	-1.568184554	8.93E-05	0.000412081
ENSMMUG00000032244	DnaJ heat shock protein family (Hsp40) member C16 [Source:NCBI gene;Acc:700894]	-1.850350992	1.86E-05	9.96E-05
ENSMMUG00000018163	caspase 9 [Source:NCBI gene;Acc:694544]	-4.580020937	1.65E-06	1.08E-05
ENSMMUG00000031821	chymotrypsin-like elastase family member 2B [Source:NCBI gene;Acc:106992406]	-2.347803025	3.83E-05	0.000190243
ENSMMUG00000053550		-4.6462774	2.16E-07	1.64E-06
ENSMMUG00000060541		-3.621349909	1.92E-06	1.23E-05
ENSMMUG00000058742		-3.178570834	0.019996718	0.048846513
ENSMMUG00000050246	EF-hand domain family member D2 [Source:NCBI gene;Acc:694155]	-2.192523883	0.000412178	0.001637559
ENSMMUG00000053235		-2.706440324	1.95E-06	1.26E-05
ENSMMUG00000003987	forkhead associated phosphopeptide binding domain 1 [Source:NCBI gene;Acc:699900]	-2.382379897	4.16E-08	3.52E-07
ENSMMUG00000055233	PRAME family member 17-like [Source:NCBI gene;Acc:100427847]	-1.094568471	3.74E-08	3.19E-07
ENSMMUG00000054309	PRAME family member 5 [Source:NCBI gene;Acc:722781]	-1.439944784	0.000738429	0.002739782
ENSMMUG00000011137	arylacetamide deacetylase like 4 [Source:VGNC Symbol;Acc:VGNC:81338]	-1.625195709	0.000144026	0.000632297
ENSMMUG00000021449	vacuolar protein sorting 13 homolog D [Source:NCBI gene;Acc:722775]	1.370332283	1.58E-35	2.76E-33
ENSMMUG00000037284	small nucleolar RNA, H/ACA box 59A [Source:HGNC Symbol;Acc:HGNC:32653]	1.602013104	0.001004372	0.003615253

ENSMMUG00000064242		1.324200824	1.30E-10	1.62E-09
ENSMMUG00000063313	Small nucleolar RNA U3 [Source:RFAM;Acc:RF00012]	0.803889031	0.008842142	0.024354766
ENSMMUG00000021447	TNF receptor superfamily member 1B [Source:VGNC Symbol;Acc:VGNC:78590]	5.053901054	5.72E-07	4.05E-06
ENSMMUG00000023449	mitofusin 2 [Source:VGNC Symbol;Acc:VGNC:74522]	2.066286006	1.58E-09	1.69E-08
ENSMMUG00000032249	procollagen-lysine,2-oxoglutarate 5-dioxygenase 1 [Source:NCBI gene;Acc:722763]	1.812108121	1.11E-31	1.21E-29
ENSMMUG00000012469	KIAA2013 [Source:VGNC Symbol;Acc:VGNC:73918]	1.222650456	1.56E-19	5.10E-18
ENSMMUG00000062316	Metazoan signal recognition particle RNA [Source:RFAM;Acc:RF00017]	1.322937466	9.64E-14	1.80E-12
ENSMMUG00000037413	U5 spliceosomal RNA [Source:RFAM;Acc:RF00020]	0.831833973	5.88E-06	3.45E-05
ENSMMUG00000051669		0.792578154	1.58E-07	1.24E-06
ENSMMUG00000023668	F-box protein 6 [Source:VGNC Symbol;Acc:VGNC:72490]	-2.12426171	0.015410235	0.039099023
ENSMMUG00000002261	F-box protein 2 [Source:VGNC Symbol;Acc:VGNC:72486]	-1.909238565	0.004971387	0.014785499
ENSMMUG00000049196	dispatched RND transporter family member 3 [Source:NCBI gene;Acc:722727]	-1.280404001	2.58E-22	1.19E-20
ENSMMUG00000032252	UbiA prenyltransferase domain containing 1 [Source:VGNC Symbol;Acc:VGNC:79881]	-1.554197902	3.42E-22	1.55E-20
ENSMMUG00000019325	mechanistic target of rapamycin kinase [Source:VGNC Symbol;Acc:VGNC:75062]	-1.920685541	5.18E-19	1.61E-17
ENSMMUG00000053062	U6 spliceosomal RNA [Source:RFAM;Acc:RF00026]	-1.542885633	0.007328901	0.020655189
ENSMMUG00000006187	angiopoietin like 7 [Source:VGNC Symbol;Acc:VGNC:69683]	-1.781938175	7.89E-11	1.01E-09
ENSMMUG00000058771	PHD finger protein 13 [Source:NCBI gene;Acc:704846]	-1.49383345	8.02E-05	0.000373115
ENSMMUG00000032281	zinc finger and BTB domain containing 48 [Source:VGNC Symbol;Acc:VGNC:79959]	-1.181629677	6.33E-07	4.46E-06
ENSMMUG00000023676	espin [Source:NCBI gene;Acc:722532]	-1.258004306	2.54E-10	3.04E-09
ENSMMUG00000055126		1.605392864	2.22E-06	1.41E-05
ENSMMUG00000054719		-2.086591197	1.63E-16	4.01E-15
ENSMMUG00000032284	leucine rich repeat containing 47 [Source:VGNC Symbol;Acc:VGNC:74388]	2.838042888	0.009523796	0.025943771
ENSMMUG00000003786	WD repeat containing, antisense to TP73 [Source:VGNC Symbol;Acc:VGNC:78791]	-1.070920269	0.000554301	0.002131263
ENSMMUG00000009358	multiple epidermal growth factor-like domains protein 6 [Source:NCBI gene;Acc:697150]	-0.658596902	0.01900811	0.046755726
ENSMMUG00000050239		-1.169792963	0.000241395	0.00101127
ENSMMUG00000010280	SKI proto-oncogene [Source:VGNC Symbol;Acc:VGNC:77374]	-3.306496888	0.000769611	0.002844799
ENSMMUG00000018911	protein kinase C zeta [Source:VGNC Symbol;Acc:VGNC:82212]	-2.466233322	1.10E-05	6.16E-05
ENSMMUG00000019086	gamma-aminobutyric acid type A receptor delta subunit [Source:VGNC Symbol;Acc:VGNC:72854]	-1.842654006	9.39E-05	0.000431169
ENSMMUG00000014075	cilia and flagella associated protein 74 [Source:NCBI gene;Acc:709689]	-1.723230499	0.000108831	0.000492364
ENSMMUG00000001896	G protein subunit beta 1 [Source:VGNC Symbol;Acc:VGNC:73095]	-1.389507827	0.003962743	0.012132014
ENSMMUG00000010879	NAD kinase [Source:VGNC Symbol;Acc:VGNC:75047]	-1.55731768	1.61E-05	8.74E-05
ENSMMUG00000005578	solute carrier family 35 member E2 [Source:VGNC Symbol;Acc:VGNC:77590]	-1.290189585	6.40E-06	3.73E-05
ENSMMUG00000001894	cyclin dependent kinase 11B [Source:NCBI gene;Acc:703046]	-1.680255406	6.87E-07	4.80E-06
ENSMMUG00000012961	mindbomb E3 ubiquitin protein ligase 2 [Source:VGNC Symbol;Acc:VGNC:74627]	-1.304735795	5.35E-10	6.12E-09

ENSMMUG00000048805		-1.596652519	4.65E-11	6.14E-10
ENSMMUG00000040063	fibronectin type III domain containing 10 [Source:NCBI gene;Acc:106996973]	-1.389296607	0.000884616	0.00321462
ENSMMUG00000060167		-1.847766957	1.37E-10	1.69E-09
ENSMMUG00000001551	SSU72 homolog, RNA polymerase II CTD phosphatase [Source:VGNC Symbol;Acc:VGNC:77986]	-1.758737891	2.99E-14	5.85E-13
ENSMMUG00000051354		-1.27269903	0.009533092	0.025954792
ENSMMUG00000001549	ATPase family AAA domain-containing protein 3A-like [Source:NCBI gene;Acc:114674453]	-1.503782036	7.08E-26	4.54E-24
ENSMMUG00000058426		-1.232705388	0.007648241	0.021465446
ENSMMUG00000064596	ATPase family AAA domain-containing protein 3A-like [Source:NCBI gene;Acc:114674454]	-1.41204744	1.84E-05	9.87E-05
ENSMMUG00000050086		-1.394325004	0.010324072	0.027822277
ENSMMUG00000006904	cyclin L2 [Source:NCBI gene;Acc:114669744]	-1.306027051	0.000277758	0.001144843
ENSMMUG00000059208	aurora kinase A interacting protein 1 [Source:NCBI gene;Acc:706329]	-1.877759651	3.30E-07	2.42E-06
ENSMMUG00000007109	dishevelled segment polarity protein 1 [Source:NCBI gene;Acc:705864]	-1.078875462	0.002129788	0.007020396
ENSMMUG00000013862	ArfGAP with coiled-coil, ankyrin repeat and PH domains 3 [Source:VGNC Symbol;Acc:VGNC:69434]	-0.645936184	0.008170544	0.022737692
ENSMMUG00000032292	sodium channel epithelial 1 delta subunit [Source:VGNC Symbol;Acc:VGNC:76993]	-1.381704777	0.000455958	0.001789796
ENSMMUG00000010030	ubiquitin conjugating enzyme E2 J2 [Source:VGNC Symbol;Acc:VGNC:79189]	-0.547655182	0.013472304	0.034959626
ENSMMUG00000019518	stromal cell derived factor 4 [Source:VGNC Symbol;Acc:VGNC:77092]	-0.60636051	0.015448672	0.039176437
ENSMMUG00000056613		0.664264278	2.37E-05	0.000123678
ENSMMUG00000018699	chromosome 1 C1orf159 homolog [Source:VGNC Symbol;Acc:VGNC:70393]	-0.75365268	0.000691302	0.002588891
ENSMMUG00000065097		-1.743309821	0.00028636	0.001174424
ENSMMUG00000005947	sterile alpha motif domain containing 11 [Source:NCBI gene;Acc:100430343]	0.753120958	0.010015934	0.027083968
ENSMMUG00000022562	interleukin 1 receptor accessory protein [Source:VGNC Symbol;Acc:VGNC:73718]	-2.156841659	0.000284018	0.001166434
ENSMMUG00000006596	CCR4-NOT transcription complex subunit 10 [Source:VGNC Symbol;Acc:VGNC:71327]	2.305186304	5.45E-13	9.32E-12
ENSMMUG00000012675	CKLF like MARVEL transmembrane domain containing 8 [Source:VGNC Symbol;Acc:VGNC:71219]	-3.583076121	0.003783018	0.011625371
ENSMMUG00000049551		1.416097279	7.67E-06	4.40E-05
ENSMMUG00000031449	zinc finger CW-type and PWWP domain containing 2 [Source:NCBI gene;Acc:702617]	-1.421923932	0.005946569	0.017244352
ENSMMUG00000055585		-2.510754179	2.73E-10	3.25E-09
ENSMMUG00000000056	5-azacytidine induced 2 [Source:VGNC Symbol;Acc:VGNC:70115]	-1.380472397	0.000170134	0.000734443
ENSMMUG00000051896		-2.393340253	0.011060423	0.029495788
ENSMMUG00000060958	leucine rich repeat containing 3B [Source:NCBI gene;Acc:701323]	-2.138348998	0.001032366	0.003703431
ENSMMUG00000051809		-2.431730159	0.005198978	0.015334298
ENSMMUG00000051975		1.828272386	1.00E-08	9.40E-08
ENSMMUG00000005370	potassium voltage-gated channel subfamily H member 8 [Source:VGNC Symbol;Acc:VGNC:73855]	-3.032644109	0.014563109	0.037320011

ENSMMUG00000054204		-0.694618894	2.50E-05	0.000129981
ENSMMUG00000019009	SATB homeobox 1 [Source:VGNC Symbol;Acc:VGNC:84086]	0.522999674	0.006110876	0.017672445
ENSMMUG00000020377	collagen type VI alpha 5 chain [Source:NCBI gene;Acc:719331]	-1.463236853	4.30E-11	5.71E-10
ENSMMUG00000050595		-2.275274292	0.005127288	0.015161189
ENSMMUG00000020294	phosphoinositide-3-kinase regulatory subunit 4 [Source:VGNC Symbol;Acc:VGNC:84060]	-0.948196138	0.000479548	0.001873471
ENSMMUG00000005137	ATPase secretory pathway Ca2+ transporting 1 [Source:VGNC Symbol;Acc:VGNC:70054]	-3.089074801	2.03E-16	4.95E-15
ENSMMUG00000001154	U8 snoRNA-decapping enzyme-like [Source:NCBI gene;Acc:717946]	0.839884028	3.41E-06	2.08E-05
ENSMMUG00000024125	small nucleolar RNA, H/ACA box 58 [Source:HGNC Symbol;Acc:HGNC:32652]	-2.508120456	0.01862023	0.04593878
ENSMMUG00000003277	copine 4 [Source:VGNC Symbol;Acc:VGNC:71466]	-1.2226976	1.36E-07	1.08E-06
ENSMMUG00000056585		-1.24100831	0.005714064	0.016658064
ENSMMUG00000016004	acid phosphatase 3 [Source:VGNC Symbol;Acc:VGNC:69385]	-1.992500262	6.29E-07	4.43E-06
ENSMMUG00000054158	acyl-CoA dehydrogenase family member 11 [Source:NCBI gene;Acc:106997118]	2.339085043	2.21E-21	9.16E-20
ENSMMUG00000019854	transmembrane protein 108 [Source:VGNC Symbol;Acc:VGNC:79714]	-2.529969109	4.92E-15	1.06E-13
ENSMMUG00000019857	beaded filament structural protein 2 [Source:VGNC Symbol;Acc:VGNC:70245]	-2.02906079	2.54E-12	3.98E-11
ENSMMUG00000057769		-2.356172897	0.00054319	0.002094516
ENSMMUG00000000058	CDV3 homolog [Source:VGNC Symbol;Acc:VGNC:71114]	-2.347746267	1.19E-11	1.69E-10
ENSMMUG00000000059	DNA topoisomerase II binding protein 1 [Source:VGNC Symbol;Acc:VGNC:79061]	-2.138019565	8.00E-32	9.05E-30
ENSMMUG00000053660	Metazoan signal recognition particle RNA [Source:RFAM;Acc:RF00017]	-1.689626167	1.73E-06	1.13E-05
ENSMMUG00000035834	U6 spliceosomal RNA [Source:RFAM;Acc:RF00026]	-1.963319408	0.007232975	0.020423653
ENSMMUG00000008916	transferrin [Source:VGNC Symbol;Acc:VGNC:82716]	-1.989687017	6.89E-23	3.37E-21
ENSMMUG00000008918	SRP receptor subunit beta [Source:VGNC Symbol;Acc:VGNC:77980]	-1.395392871	7.35E-09	7.05E-08
ENSMMUG00000008919	RAB6B, member RAS oncogene family [Source:VGNC Symbol;Acc:VGNC:81547]	-1.43324236	9.79E-08	7.90E-07
ENSMMUG00000012605	solute carrier organic anion transporter family member 2A1 [Source:VGNC Symbol;Acc:VGNC:77600]	-0.594776216	8.04E-06	4.60E-05
ENSMMUG00000055637		-1.688378886	0.006919697	0.019685231
ENSMMUG00000003263	stromal antigen 1 [Source:VGNC Symbol;Acc:VGNC:77901]	-0.718601684	1.72E-06	1.12E-05
ENSMMUG00000041198	muscle RAS oncogene homolog [Source:NCBI gene;Acc:716352]	0.928991752	0.000589467	0.002247248
ENSMMUG00000007757	extended synaptotagmin 3 [Source:VGNC Symbol;Acc:VGNC:84182]	-0.514005456	0.013038016	0.033963424
ENSMMUG00000007760	centrosomal protein 70 [Source:VGNC Symbol;Acc:VGNC:71140]	1.134666912	9.66E-15	2.02E-13
ENSMMUG00000053063	forkhead box L2 [Source:NCBI gene;Acc:717906]	0.81626955	7.53E-07	5.23E-06
ENSMMUG00000003051	COPI coat complex subunit beta 2 [Source:VGNC Symbol;Acc:VGNC:71313]	-1.043089725	2.02E-10	2.45E-09
ENSMMUG00000003169	calsyntenin 2 [Source:VGNC Symbol;Acc:VGNC:71197]	-3.000661202	0.001385274	0.004813404
ENSMMUG00000062527		-1.13009361	3.17E-07	2.33E-06
ENSMMUG00000022153	solute carrier family 25 member 36 [Source:VGNC Symbol;Acc:VGNC:84079]	-1.377390948	5.93E-08	4.94E-07

ENSMMUG00000053344	splA/ryanodine receptor domain and SOCS box containing 4 [Source:NCBI gene;Acc:715278]	-<br>1.942420142	3.65E-05	0.000182693
ENSMMUG00000057297		-1.30208872	0.000876335	0.003189401
ENSMMUG00000056024		1.323358111	4.64E-17	1.21E-15
ENSMMUG00000007387	G protein-coupled receptor kinase 7 [Source:VGNC Symbol;Acc:VGNC:73182]	1.231707622	1.89E-10	2.30E-09
ENSMMUG00000052474		1.172464789	8.42E-07	5.79E-06
ENSMMUG00000050932		0.782727346	0.001719998	0.005822333
ENSMMUG00000060612	ATPase Na+/K+ transporting subunit beta 3 [Source:NCBI gene;Acc:716682]	0.801463665	0.01170127	0.030998542
ENSMMUG00000053154	U6 spliceosomal RNA [Source:RFAM;Acc:RF00026]	2.037993987	0.008164172	0.022724226
ENSMMUG00000011134	transcription factor Dp-2 [Source:VGNC Symbol;Acc:VGNC:78324]	0.717510937	7.66E-07	5.30E-06
ENSMMUG00000012151	glycerol kinase 5 [Source:VGNC Symbol;Acc:VGNC:73022]	-2.43455373	1.44E-07	1.14E-06
ENSMMUG00000055990	plastin 1 [Source:NCBI gene;Acc:714391]	-<br>0.866622276	0.009672735	0.026286724
ENSMMUG00000012405	transient receptor potential cation channel subfamily C member 1 [Source:VGNC Symbol;Acc:VGNC:78651]	3.326357513	2.86E-12	4.46E-11
ENSMMUG00000009694	solute carrier family 9 member A9 [Source:VGNC Symbol;Acc:VGNC:77747]	-<br>2.849184163	2.43E-05	0.000126887
ENSMMUG00000062595		0.847967213	0.000364995	0.001466904
ENSMMUG00000021755	phospholipid scramblase 2 [Source:NCBI gene;Acc:713298]	-1.42430213	0.019721306	0.048277808
ENSMMUG00000049859		-<br>1.803244317	8.64E-12	1.25E-10
ENSMMUG00000062175	carboxypeptidase B1 [Source:NCBI gene;Acc:712639]	1.090131992	2.84E-08	2.49E-07
ENSMMUG00000005519	carboxypeptidase A3 [Source:VGNC Symbol;Acc:VGNC:71456]	0.825196928	2.05E-05	0.000108813
ENSMMUG00000059895		0.882996638	0.000200263	0.000854157
ENSMMUG00000005521	glycogenin 1 [Source:VGNC Symbol;Acc:VGNC:73332]	0.920151755	2.30E-08	2.05E-07
ENSMMUG00000005526	helicase like transcription factor [Source:VGNC Symbol;Acc:VGNC:73383]	0.601453366	0.001409219	0.00488516
ENSMMUG00000005529	ceruloplasmin [Source:VGNC Symbol;Acc:VGNC:71454]	0.468126275	0.00013945	0.000614576
ENSMMUG00000059534		0.698084369	0.002234306	0.007310329
ENSMMUG00000047621	transmembrane 4 L six family member 18 [Source:VGNC Symbol;Acc:VGNC:82713]	0.883287145	7.78E-14	1.47E-12
ENSMMUG00000031534	ankyrin repeat and ubiquitin domain containing 1 [Source:VGNC Symbol;Acc:VGNC:84239]	2.357629398	5.11E-36	9.46E-34
ENSMMUG00000035943	U6 spliceosomal RNA [Source:RFAM;Acc:RF00026]	2.741393946	1.52E-14	3.11E-13
ENSMMUG00000023335	ring finger protein 13 [Source:VGNC Symbol;Acc:VGNC:81557]	1.827112769	1.19E-13	2.20E-12
ENSMMUG00000059788		0.710445704	4.41E-05	0.000216386
ENSMMUG00000048232	MINDY family member 4B [Source:VGNC Symbol;Acc:VGNC:84153]	-<br>1.317379048	3.61E-11	4.83E-10
ENSMMUG00000017239	mediator complex subunit 12L [Source:VGNC Symbol;Acc:VGNC:74515]	0.458074333	0.000622995	0.00236292
ENSMMUG00000038998	purinergic receptor P2Y13 [Source:VGNC Symbol;Acc:VGNC:83950]	1.368476618	9.21E-08	7.46E-07
ENSMMUG00000001105	purinergic receptor P2Y12 [Source:VGNC Symbol;Acc:VGNC:75642]	1.116999409	1.57E-08	1.42E-07
ENSMMUG00000057554	Metazoan signal recognition particle RNA [Source:RFAM;Acc:RF00017]	1.676557526	9.17E-05	0.00042199
ENSMMUG00000057501		-<br>1.976017447	4.65E-09	4.58E-08
ENSMMUG00000031537		-<br>1.835466061	0.013954965	0.036010462
ENSMMUG00000053217		3.992811803	0.000532017	0.002054638
ENSMMUG00000001664	arylacetamide deacetylase [Source:VGNC Symbol;Acc:VGNC:81172]	-<br>1.801862343	0.004260611	0.012934465
ENSMMUG00000061194		1.594721387	9.10E-08	7.37E-07
ENSMMUG00000061684		1.620490856	2.10E-06	1.34E-05
ENSMMUG00000053540		1.675603032	0.002744899	0.008784389
ENSMMUG00000031540	purinergic receptor P2Y1 [Source:VGNC Symbol;Acc:VGNC:75737]	4.933464966	2.25E-06	1.43E-05
ENSMMUG00000011347	DEAH-box helicase 36 [Source:VGNC Symbol;Acc:VGNC:71623]	1.266382597	2.75E-24	1.55E-22
ENSMMUG00000051847		1.520621073	0.000331635	0.001344859
ENSMMUG00000056924	G protein-coupled receptor 149 [Source:NCBI gene;Acc:106997054]	1.206997304	0.000179862	0.000774266
ENSMMUG00000060572		0.988694295	3.96E-05	0.000195792

ENSMMUG00000023081	schwannomin interacting protein 1 [Source:NCBI gene;Acc:703321]	-0.55933379	0.010729828	0.028722627
ENSMMUG00000056116		-1.503948708	5.62E-09	5.48E-08
ENSMMUG00000009564	NMD3 ribosome export adaptor [Source:VGNC Symbol;Acc:VGNC:75242]	1.724349333	0.000489788	0.001909452
ENSMMUG00000063228		-4.436392698	1.24E-05	6.84E-05
ENSMMUG00000042979		-2.771588456	0.000975266	0.003521103
ENSMMUG00000054039		-2.685555379	0.009766294	0.026511832
ENSMMUG00000055530	Metazoan signal recognition particle RNA [Source:RFAM;Acc:RF00017]	-1.524107049	0.001794412	0.006053488
ENSMMUG00000001302	sucrase-isomaltase [Source:VGNC Symbol;Acc:VGNC:77213]	0.760010666	1.53E-08	1.39E-07
ENSMMUG00000003742	butyrylcholinesterase [Source:VGNC Symbol;Acc:VGNC:70238]	0.564430787	0.000413539	0.001641977
ENSMMUG00000023045	zinc finger B-box domain containing [Source:VGNC Symbol;Acc:VGNC:79592]	1.922479003	4.67E-06	2.80E-05
ENSMMUG00000005939	serpin family I member 1 [Source:VGNC Symbol;Acc:VGNC:77366]	1.456727027	2.15E-05	0.000113174
ENSMMUG00000051641		-1.915485098	7.59E-12	1.10E-10
ENSMMUG00000063303	U6 spliceosomal RNA [Source:RFAM;Acc:RF00026]	-3.347293918	0.004032165	0.012321657
ENSMMUG00000021875	myoneurin [Source:VGNC Symbol;Acc:VGNC:74958]	-2.716529507	0.016430665	0.041348508
ENSMMUG00000022199	polyhomeotic homolog 3 [Source:VGNC Symbol;Acc:VGNC:75804]	-1.253644007	1.21E-06	8.06E-06
ENSMMUG00000022880	eukaryotic translation initiation factor 5A2 [Source:VGNC Symbol;Acc:VGNC:72198]	-1.400908762	2.62E-08	2.31E-07
ENSMMUG00000040869	spermatogenesis associated 16 [Source:VGNC Symbol;Acc:VGNC:84073]	0.633866106	0.006200762	0.017897489
ENSMMUG00000023078	zinc finger protein 639 [Source:VGNC Symbol;Acc:VGNC:82561]	0.781463286	2.26E-06	1.44E-05
ENSMMUG00000003594	ubiquitin specific peptidase 13 [Source:VGNC Symbol;Acc:VGNC:78735]	-0.814849459	2.55E-08	2.25E-07
ENSMMUG00000064088	U2 spliceosomal RNA [Source:RFAM;Acc:RF00004]	-0.723883856	0.000331823	0.001345252
ENSMMUG00000003595	peroxisomal biogenesis factor 5 like [Source:VGNC Symbol;Acc:VGNC:75889]	-2.490107182	0.000729155	0.002709242
ENSMMUG00000055097		-1.436514523	1.41E-05	7.75E-05
ENSMMUG00000008070	YEATS domain containing 2 [Source:VGNC Symbol;Acc:VGNC:79502]	2.141317238	2.11E-49	1.74E-46
ENSMMUG00000013661	presenilin associated rhomboid like [Source:NCBI gene;Acc:704652]	-1.998867039	0.000408367	0.00162405
ENSMMUG00000005515	ATP binding cassette subfamily C member 5 [Source:VGNC Symbol;Acc:VGNC:69574]	-1.394594378	6.52E-13	1.11E-11
ENSMMUG00000006553	dishevelled segment polarity protein 3 [Source:VGNC Symbol;Acc:VGNC:72014]	-1.680145871	1.69E-09	1.79E-08
ENSMMUG00000000800	proteasome 26S subunit, non-ATPase 2 [Source:VGNC Symbol;Acc:VGNC:84144]	-0.362862457	0.010584186	0.028389126
ENSMMUG00000007503	family with sequence similarity 131 member A [Source:VGNC Symbol;Acc:VGNC:72385]	-0.516546203	0.0003394	0.001373719
ENSMMUG00000007524	RNA polymerase II subunit H [Source:VGNC Symbol;Acc:VGNC:76216]	-0.896725227	5.43E-06	3.21E-05
ENSMMUG00000007528	chordin [Source:VGNC Symbol;Acc:VGNC:71042]	3.064205884	5.70E-18	1.62E-16
ENSMMUG00000060581		2.662888325	4.27E-13	7.38E-12
ENSMMUG00000002953	MAGE family member F1 [Source:VGNC Symbol;Acc:VGNC:74399]	1.235454023	2.15E-06	1.37E-05
ENSMMUG00000023814	mitogen-activated protein kinase kinase kinase 13 [Source:VGNC Symbol;Acc:VGNC:74494]	-1.095225947	0.000713977	0.002660373

ENSMMUG00000064748	Metazoan signal recognition particle RNA [Source:RFAM;Acc:RF00017]	-1.128980342	0.002600836	0.008368495
ENSMMUG00000021972	diacylglycerol kinase gamma [Source:VGNC Symbol;Acc:VGNC:71785]	1.163153288	5.24E-11	6.88E-10
ENSMMUG00000052418		-0.700110187	1.95E-07	1.50E-06
ENSMMUG00000018909	fetuin B [Source:VGNC Symbol;Acc:VGNC:72638]	0.734848086	3.51E-07	2.56E-06
ENSMMUG00000018910	histidine rich glycoprotein [Source:VGNC Symbol;Acc:VGNC:73521]	1.857082178	5.81E-10	6.60E-09
ENSMMUG00000022532	EPM2A interacting protein 1 [Source:VGNC Symbol;Acc:VGNC:84184]	-1.254874333	2.85E-06	1.78E-05
ENSMMUG00000049937	Y RNA [Source:RFAM;Acc:RF00019]	-0.649814541	0.014220191	0.036533048
ENSMMUG00000054175		1.042264347	2.01E-09	2.11E-08
ENSMMUG00000021927	integrin subunit alpha 9 [Source:VGNC Symbol;Acc:VGNC:82165]	0.377940019	0.014136706	0.036378163
ENSMMUG00000000987	DLEC1 cilia and flagella associated protein [Source:VGNC Symbol;Acc:VGNC:71808]	-0.533313188	0.002175518	0.007144138
ENSMMUG00000004847	xylulokinase [Source:VGNC Symbol;Acc:VGNC:78815]	0.984558224	6.11E-12	9.03E-11
ENSMMUG00000018790	activin A receptor type 2B [Source:VGNC Symbol;Acc:VGNC:69450]	3.195498985	1.40E-49	1.22E-46
ENSMMUG00000020150	xin actin binding repeat containing 1 [Source:VGNC Symbol;Acc:VGNC:78952]	-0.752602167	2.92E-10	3.45E-09
ENSMMUG00000051408	myelin associated oligodendrocyte basic protein [Source:NCBI gene;Acc:717265]	1.925759671	2.55E-19	8.19E-18
ENSMMUG00000009197	myelin associated oligodendrocyte basic protein [Source:VGNC Symbol;Acc:VGNC:74908]	2.071019288	8.31E-33	1.10E-30
ENSMMUG00000017701	myosin VIIA and Rab interacting protein [Source:VGNC Symbol;Acc:VGNC:74992]	2.657009325	1.05E-17	2.95E-16
ENSMMUG00000013330	ectonucleoside triphosphate diphosphohydrolase 3 [Source:VGNC Symbol;Acc:VGNC:72226]	2.84411191	6.99E-23	3.41E-21
ENSMMUG00000009437	zinc finger protein 619 [Source:VGNC Symbol;Acc:VGNC:82562]	2.903726486	1.50E-07	1.18E-06
ENSMMUG00000024178	U5 spliceosomal RNA [Source:RFAM;Acc:RF00020]	2.959372832	0.001569623	0.005359323
ENSMMUG00000050991		-0.970925852	6.01E-05	0.000287109
ENSMMUG00000001074	unc-51 like kinase 4 [Source:VGNC Symbol;Acc:VGNC:82565]	-0.617348867	0.010157241	0.027427509
ENSMMUG00000052672		4.032477065	0.004684752	0.01404952
ENSMMUG00000001075	trafficking kinesin protein 1 [Source:VGNC Symbol;Acc:VGNC:79830]	0.676963333	0.001869259	0.006277427
ENSMMUG00000035160	U4 spliceosomal RNA [Source:RFAM;Acc:RF00015]	2.3291133	0.003400198	0.010593475
ENSMMUG00000001077	cholecystokinin [Source:VGNC Symbol;Acc:VGNC:70716]	2.864802989	0.018174923	0.045034938
ENSMMUG00000050275		-0.725166299	0.020560824	0.049970012
ENSMMUG00000021242	natural killer cell triggering receptor [Source:VGNC Symbol;Acc:VGNC:81486]	-1.636182966	0.013931841	0.035969568
ENSMMUG00000021244	kelch like family member 40 [Source:VGNC Symbol;Acc:VGNC:74165]	-2.249281648	0.015771245	0.039912553
ENSMMUG00000031684	KRAB box domain containing 1 [Source:NCBI gene;Acc:717189]	2.883997338	1.12E-06	7.54E-06
ENSMMUG00000009745	protein O-linked mannose N-acetylglucosaminyltransferase 2 (beta 1,4-) [Source:VGNC Symbol;Acc:VGNC:84062]	1.523078712	0.002007296	0.006677479
ENSMMUG00000056798		2.290262248	5.33E-07	3.79E-06
ENSMMUG00000054286		1.561540271	2.94E-05	0.000150356
ENSMMUG00000001028	SNF related kinase [Source:VGNC Symbol;Acc:VGNC:84074]	0.797232642	5.94E-14	1.14E-12
ENSMMUG00000040696	Metazoan signal recognition particle RNA [Source:RFAM;Acc:RF00017]	2.201582938	0.002073628	0.006868482
ENSMMUG00000017585	zinc finger protein 197 [Source:VGNC Symbol;Acc:VGNC:78864]	0.731737136	0.017065939	0.042700839
ENSMMUG00000051589		-0.771016233	0.000412287	0.001637559
ENSMMUG00000019740	zinc finger DHHC-type palmitoyltransferase 3 [Source:VGNC Symbol;Acc:VGNC:79143]	-1.297443603	0.000525731	0.002032881
ENSMMUG00000019130	exosome component 7 [Source:VGNC Symbol;Acc:VGNC:72383]	-1.056732381	0.009507013	0.025902813
ENSMMUG00000005742	CUB domain containing protein 1 [Source:VGNC Symbol;Acc:VGNC:70849]	-1.398171847	0.000839299	0.003072957

ENSMMUG00000018020	leucyl-tRNA synthetase 2, mitochondrial [Source:VGNC Symbol;Acc:VGNC:74063]	0.288025934	0.004608999	0.01385877
ENSMMUG00000056769	C-C motif chemokine receptor 1 [Source:VGNC Symbol;Acc:VGNC:70737]	-0.994924095	5.84E-05	0.000279641
ENSMMUG00000004576	C-C motif chemokine receptor 3 [Source:VGNC Symbol;Acc:VGNC:70738]	3.571917061	3.38E-31	3.56E-29
ENSMMUG00000062394	C-C motif chemokine receptor 2 [Source:NCBI gene;Acc:574098]	0.987104977	3.03E-05	0.000154458
ENSMMUG00000006454	kelch like family member 18 [Source:VGNC Symbol;Acc:VGNC:74154]	-0.848181741	0.003389839	0.010567862
ENSMMUG00000014045	protein tyrosine phosphatase non-receptor type 23 [Source:VGNC Symbol;Acc:VGNC:76477]	0.92523749	0.000214193	0.000907855
ENSMMUG00000014046	SREBF chaperone [Source:VGNC Symbol;Acc:VGNC:77140]	1.603531047	6.63E-20	2.31E-18
ENSMMUG00000013385	elongator acetyltransferase complex subunit 6 [Source:VGNC Symbol;Acc:VGNC:72113]	1.426864196	8.06E-11	1.03E-09
ENSMMUG00000041434	Metazoan signal recognition particle RNA [Source:RFAM;Acc:RF00017]	-0.458941561	0.013979522	0.036067556
ENSMMUG00000020954	cell division cycle 25A [Source:VGNC Symbol;Acc:VGNC:70794]	-0.964721255	0.0007139	0.002660373
ENSMMUG00000064458		-1.698314559	1.79E-07	1.38E-06
ENSMMUG00000003074	plexin B1 [Source:VGNC Symbol;Acc:VGNC:76187]	3.03665145	0.000321111	0.001306468
ENSMMUG00000050584	coiled-coil domain containing 51 [Source:NCBI gene;Acc:710311]	2.506220137	0.000702018	0.002623469
ENSMMUG00000010058	ATR interacting protein [Source:VGNC Symbol;Acc:VGNC:84235]	1.098940583	2.07E-26	1.46E-24
ENSMMUG00000043955	three prime repair exonuclease 1 [Source:VGNC Symbol;Acc:VGNC:82708]	0.936868231	7.62E-07	5.28E-06
ENSMMUG00000010062	shisa family member 5 [Source:VGNC Symbol;Acc:VGNC:84082]	0.921573161	4.84E-19	1.51E-17
ENSMMUG00000000591	6-phosphofructo-2-kinase/fructose-2,6-biphosphatase 4 [Source:VGNC Symbol;Acc:VGNC:75960]	1.102389028	7.97E-19	2.44E-17
ENSMMUG00000059318	urocortin 2 [Source:NCBI gene;Acc:106996925]	2.670853531	5.06E-47	2.89E-44
ENSMMUG00000014699		2.118923686	1.46E-39	3.80E-37
ENSMMUG00000032659	mml-mir-711 [Source:miRBase;Acc:MI0012553]	1.983338407	8.12E-06	4.64E-05
ENSMMUG00000014707	cadherin EGF LAG seven-pass G-type receptor 3 [Source:VGNC Symbol;Acc:VGNC:71148]	1.532399523	0.001937992	0.006476092
ENSMMUG00000064174		1.574730256	0.00049626	0.001931637
ENSMMUG00000014720	NCK interacting protein with SH3 domain [Source:VGNC Symbol;Acc:VGNC:74960]	1.663617731	7.82E-09	7.45E-08
ENSMMUG00000015418	solute carrier family 25 member 20 [Source:VGNC Symbol;Acc:VGNC:77473]	1.887326883	0.000987514	0.003562355
ENSMMUG00000039597	ariadne RBR E3 ubiquitin protein ligase 2 [Source:NCBI gene;Acc:708356]	1.929583249	0.002049398	0.006799234
ENSMMUG00000006130	prolyl 4-hydroxylase, transmembrane [Source:VGNC Symbol;Acc:VGNC:75693]	2.458219892	1.56E-05	8.49E-05
ENSMMUG00000005660	glutamine rich 1 [Source:VGNC Symbol;Acc:VGNC:76530]	1.808491779	0.010412728	0.028000137
ENSMMUG00000002943	glutaminyl-tRNA synthetase 1 [Source:VGNC Symbol;Acc:VGNC:76634]	1.271531524	0.001536002	0.005264365
ENSMMUG00000002948	ubiquitin specific peptidase 19 [Source:VGNC Symbol;Acc:VGNC:78739]	1.38285457	0.003692942	0.011381198
ENSMMUG00000002952	laminin subunit beta 2 [Source:VGNC Symbol;Acc:VGNC:74229]	1.556666976	7.40E-05	0.000346908
ENSMMUG00000002836	coiled-coil domain containing 71 [Source:VGNC Symbol;Acc:VGNC:70862]	2.036382743	0.000230486	0.000970632
ENSMMUG00000002833	kelch domain containing 8B [Source:VGNC Symbol;Acc:VGNC:74142]	1.613690524	0.001794253	0.006053488
ENSMMUG00000052238		1.257912841	2.56E-12	4.00E-11
ENSMMUG00000014537	bassoon presynaptic cytomatrix protein [Source:VGNC Symbol;Acc:VGNC:70284]	0.828496224	4.94E-06	2.94E-05
ENSMMUG00000014540	acylaminoacyl-peptide hydrolase [Source:VGNC Symbol;Acc:VGNC:69982]	-0.980044697	6.21E-05	0.000296082
ENSMMUG00000014564	ring finger protein 123 [Source:VGNC Symbol;Acc:VGNC:76790]	-0.738361148	7.12E-10	8.01E-09
ENSMMUG00000020979	inka box actin regulator 1 [Source:VGNC Symbol;Acc:VGNC:73692]	0.558435079	0.020286982	0.049450352
ENSMMUG00000016476	TRAF interacting protein [Source:VGNC Symbol;Acc:VGNC:82261]	0.509392989	0.002972295	0.009426674
ENSMMUG00000010342	solute carrier family 38 member 3 [Source:VGNC Symbol;Acc:VGNC:77502]	-1.102006554	0.008641668	0.023882956
ENSMMUG00000056280	G protein subunit alpha i2 [Source:NCBI gene;Acc:703780]	-1.079684183	9.02E-06	5.12E-05
ENSMMUG00000020117	semaphorin 3B [Source:VGNC Symbol;Acc:VGNC:77160]	-1.3753562	2.80E-06	1.75E-05
ENSMMUG00000007022	calcium voltage-gated channel auxiliary subunit alpha2delta 2 [Source:VGNC Symbol;Acc:VGNC:70508]	1.210647281	0.009539317	0.025966973
ENSMMUG00000010499	chromosome 2 C3orf18 homolog [Source:VGNC Symbol;Acc:VGNC:70622]	0.801241167	0.015342866	0.038961422
ENSMMUG00000004273	dedicator of cytokinesis 3 [Source:VGNC Symbol;Acc:VGNC:71967]	1.804173869	1.61E-25	9.98E-24

ENSMMUG00000004280	DDB1 and CUL4 associated factor 1 [Source:VGNC Symbol;Acc:VGNC:71819]	1.45080839	0.000318871	0.001298779
ENSMMUG00000017351	stabilin 1 [Source:VGNC Symbol;Acc:VGNC:77956]	-0.945338549	4.31E-05	0.000212089
ENSMMUG00000050505	small integral membrane protein 4 [Source:NCBI gene;Acc:696553]	1.23268678	3.91E-10	4.55E-09
ENSMMUG00000033553		0.472032203	0.019470852	0.047743507
ENSMMUG00000012511	G protein nucleolar 3 [Source:VGNC Symbol;Acc:VGNC:73038]	0.40123506	0.000354465	0.001428454
ENSMMUG00000037073	small nucleolar RNA, C/D box 19B [Source:HGNC Symbol;Acc:HGNC:33570]	1.033619642	0.002205659	0.007230312
ENSMMUG00000012526	glycosyltransferase 8 domain containing 1 [Source:VGNC Symbol;Acc:VGNC:73001]	0.891426563	2.21E-10	2.66E-09
ENSMMUG00000017370	signal peptidase complex subunit 1 [Source:VGNC Symbol;Acc:VGNC:84072]	0.865952918	0.003889616	0.011927833
ENSMMUG00000014527	Scm like with four mbt domains 1 [Source:VGNC Symbol;Acc:VGNC:77279]	-0.93960453	0.00506658	0.015021468
ENSMMUG00000019262	calcium voltage-gated channel subunit alpha1 D [Source:VGNC Symbol;Acc:VGNC:70501]	-0.998019981	0.002739263	0.008773923
ENSMMUG00000019266	choline dehydrogenase [Source:VGNC Symbol;Acc:VGNC:71086]	-1.115328196	1.54E-07	1.20E-06
ENSMMUG00000019267	interleukin 17 receptor B [Source:VGNC Symbol;Acc:VGNC:73618]	-1.198274839	1.03E-06	6.97E-06
ENSMMUG00000019269	actin related protein 8 [Source:VGNC Symbol;Acc:VGNC:69406]	-0.977733094	0.01055791	0.028344282
ENSMMUG00000051778		0.477256841	0.01901555	0.046758515
ENSMMUG00000021620	ELKS/RAB6-interacting/CAST family member 2 [Source:VGNC Symbol;Acc:VGNC:72164]	-0.644054591	0.002043021	0.006779594
ENSMMUG00000039085	adaptor protein, phosphotyrosine interacting with PH domain and leucine zipper 1 [Source:NCBI gene;Acc:100425753]	-2.776269857	5.25E-05	0.000254095
ENSMMUG00000021008	ankyrin repeat and SOCS box containing 14 [Source:VGNC Symbol;Acc:VGNC:84237]	-2.738632247	0.000481045	0.001878691
ENSMMUG00000055244	U6 spliceosomal RNA [Source:RFAM;Acc:RF00026]	-1.50154846	6.62E-11	8.58E-10
ENSMMUG00000004052	phosphodiesterase 12 [Source:VGNC Symbol;Acc:VGNC:75863]	-1.247990312	0.006017272	0.017428934
ENSMMUG00000018619	DENN domain containing 6A [Source:VGNC Symbol;Acc:VGNC:71726]	1.902367288	2.37E-06	1.50E-05
ENSMMUG00000000706	filamin B [Source:VGNC Symbol;Acc:VGNC:72672]	1.188108845	7.39E-11	9.52E-10
ENSMMUG00000017510	membrane associated guanylate kinase, WW and PDZ domain containing 1 [Source:VGNC Symbol;Acc:VGNC:74499]	2.800897854	2.41E-46	1.32E-43
ENSMMUG00000057539		3.044429243	3.98E-18	1.15E-16
ENSMMUG00000001529	solute carrier family 25 member 26 [Source:VGNC Symbol;Acc:VGNC:77530]	-1.666540606	3.63E-16	8.55E-15
ENSMMUG00000001531	leucine rich repeats and immunoglobulin like domains 1 [Source:VGNC Symbol;Acc:VGNC:84156]	-1.665881274	2.72E-18	8.02E-17
ENSMMUG00000062338		-1.292749512	8.21E-06	4.68E-05
ENSMMUG00000054412		-1.480823779	6.08E-07	4.29E-06
ENSMMUG00000057486		1.14059008	2.07E-07	1.58E-06
ENSMMUG00000054043		-2.265173152	0.002609325	0.008392351
ENSMMUG00000057543	MyoD family inhibitor domain containing 2 [Source:HGNC Symbol;Acc:HGNC:53442]	0.905060781	0.000212518	0.000901759
ENSMMUG00000009425	actin, cytoplasmic 2-like [Source:NCBI gene;Acc:705671]	-0.713728134	0.000153897	0.000671461
ENSMMUG00000054107		-1.175043973	5.76E-06	3.39E-05
ENSMMUG00000001849	interleukin 5 receptor subunit alpha [Source:VGNC Symbol;Acc:VGNC:73731]	-1.065102828	0.001859247	0.006249464
ENSMMUG00000017983	tRNA nucleotidyl transferase 1 [Source:VGNC Symbol;Acc:VGNC:79073]	2.049303088	6.84E-05	0.000322676
ENSMMUG00000060798		-1.252425818	1.23E-06	8.22E-06
ENSMMUG00000012574	inositol 1,4,5-trisphosphate receptor type 1 [Source:VGNC Symbol;Acc:VGNC:73804]	-0.431828739	0.0167136	0.041989274

ENSMMUG00000043287		-0.413120148	0.015926376	0.040270785
ENSMMUG00000057502		3.268367717	0.013494071	0.035009984
ENSMMUG00000018281	interleukin 17 receptor E [Source:VGNC Symbol;Acc:VGNC:73623]	-0.82621344	2.13E-07	1.62E-06
ENSMMUG00000018282	interleukin 17 receptor C [Source:VGNC Symbol;Acc:VGNC:84164]	-0.949133834	7.58E-07	5.26E-06
ENSMMUG00000018285	cysteine rich with EGF like domains 1 [Source:VGNC Symbol;Acc:VGNC:71407]	-1.204659107	5.96E-11	7.77E-10
ENSMMUG00000018289	proline rich transmembrane protein 3 [Source:VGNC Symbol;Acc:VGNC:76324]	-0.839082386	3.63E-05	0.00018195
ENSMMUG00000018290	ER membrane protein complex subunit 3 [Source:VGNC Symbol;Acc:VGNC:72091]	-1.169318061	3.31E-05	0.000167466
ENSMMUG00000019933	TatD DNase domain containing 2 [Source:NCBI gene;Acc:701390]	1.078257949	0.00281701	0.008982211
ENSMMUG00000019938	ATPase plasma membrane Ca2+ transporting 2 [Source:VGNC Symbol;Acc:VGNC:70181]	-2.518212692	0.00490975	0.014629614
ENSMMUG00000021806	vestigial like family member 4 [Source:VGNC Symbol;Acc:VGNC:79755]	-1.101466898	6.13E-05	0.000292442
ENSMMUG00000054916	Metazoan signal recognition particle RNA [Source:RFAM;Acc:RF00017]	-1.112783267	0.003419314	0.010641853
ENSMMUG00000007195	tRNA splicing endonuclease subunit 2 [Source:VGNC Symbol;Acc:VGNC:82707]	1.152422758	4.45E-05	0.00021834
ENSMMUG00000009536	makorin ring finger protein 2 [Source:VGNC Symbol;Acc:VGNC:74878]	-1.456516531	8.91E-08	7.23E-07
ENSMMUG00000023125	XPC complex subunit, DNA damage recognition and repair factor [Source:VGNC Symbol;Acc:VGNC:78809]	-1.372434046	3.28E-06	2.02E-05
ENSMMUG00000023124	transmembrane protein 43 [Source:VGNC Symbol;Acc:VGNC:82711]	-1.630778124	8.99E-16	2.05E-14
ENSMMUG00000057449		-1.54812556	0.004806683	0.014371593
ENSMMUG00000006340	solute carrier family 6 member 6 [Source:VGNC Symbol;Acc:VGNC:77622]	-0.917102326	2.53E-09	2.60E-08
ENSMMUG00000044822	coiled-coil domain containing 174 [Source:VGNC Symbol;Acc:VGNC:84219]	1.107719555	4.86E-13	8.34E-12
ENSMMUG00000012727	intraflagellar transport 122 [Source:VGNC Symbol;Acc:VGNC:84167]	2.803190068	0.007204974	0.020363973
ENSMMUG00000018663	eukaryotic elongation factor, selenocysteine-tRNA specific [Source:VGNC Symbol;Acc:VGNC:72097]	-2.033785372	0.006351783	0.018276474
ENSMMUG00000000158	minichromosome maintenance complex component 2 [Source:VGNC Symbol;Acc:VGNC:74680]	-1.391746302	1.51E-13	2.75E-12
ENSMMUG00000000756	transmembrane protein adipocyte associated 1 [Source:VGNC Symbol;Acc:VGNC:79848]	0.8375393	0.000280289	0.001153674
ENSMMUG00000016719	coiled-coil-helix-coiled-coil-helix domain containing 6 [Source:VGNC Symbol;Acc:VGNC:71119]	-0.459200979	0.01435862	0.036840541
ENSMMUG00000049548		1.112214037	3.27E-07	2.40E-06
ENSMMUG00000017125	oxysterol binding protein like 11 [Source:VGNC Symbol;Acc:VGNC:75631]	-1.052668152	6.29E-07	4.43E-06
ENSMMUG00000032031		-1.462326083	0.00053902	0.002079516
ENSMMUG00000061615		-1.80827779	0.009408165	0.025694858
ENSMMUG00000049323		1.154446602	0.011453321	0.030434108
ENSMMUG00000001949	coiled-coil domain containing 191 [Source:VGNC Symbol;Acc:VGNC:70901]	1.332570556	0.000250798	0.001046817
ENSMMUG00000001946	zinc finger DHHC-type palmitoyltransferase 23 [Source:VGNC Symbol;Acc:VGNC:79731]	1.258412901	0.000258335	0.001074341
ENSMMUG00000001943	GRAM domain containing 1C [Source:VGNC Symbol;Acc:VGNC:73007]	0.984017605	3.02E-05	0.000154458
ENSMMUG00000052581	upstream transcription factor family member 3 [Source:NCBI gene;Acc:709660]	-0.953178506	7.32E-08	6.01E-07
ENSMMUG00000057363		1.486639424	1.31E-05	7.20E-05
ENSMMUG00000014874	GTP binding protein 8 (putative) [Source:VGNC Symbol;Acc:VGNC:84180]	1.973633635	4.62E-21	1.85E-19
ENSMMUG00000014876	nucleolus and neural progenitor protein [Source:VGNC Symbol;Acc:VGNC:75244]	1.900900718	2.47E-12	3.89E-11
ENSMMUG00000003199	CD200 receptor 1 [Source:VGNC Symbol;Acc:VGNC:70845]	1.901751419	3.79E-07	2.75E-06
ENSMMUG00000050558		0.856074983	0.000665124	0.002501597

ENSMMUG00000000894	cellular inhibitor of PP2A [Source:VGNC Symbol;Acc:VGNC:71371]	2.467998013	0.003119923	0.009827625
ENSMMUG00000019977	myosin heavy chain 15 [Source:VGNC Symbol;Acc:VGNC:84148]	-1.617344951	4.35E-08	3.67E-07
ENSMMUG00000008791	CD47 molecule [Source:VGNC Symbol;Acc:VGNC:70767]	1.970280689	2.96E-09	3.02E-08
ENSMMUG00000008787	BBX high mobility group box domain containing [Source:VGNC Symbol;Acc:VGNC:82110]	0.637211167	1.42E-06	9.37E-06
ENSMMUG00000058765		-1.467643756	7.62E-06	4.38E-05
ENSMMUG00000055345	Y RNA [Source:RFAM;Acc:RF00019]	0.955766872	1.10E-07	8.86E-07
ENSMMUG00000063790		2.063413146	0.00109532	0.003907528
ENSMMUG00000007565	Cbl proto-oncogene B [Source:VGNC Symbol;Acc:VGNC:70555]	-1.020836129	5.24E-06	3.10E-05
ENSMMUG00000026103	U1 spliceosomal RNA [Source:RFAM;Acc:RF00003]	-1.843242543	8.45E-05	0.000392178
ENSMMUG00000060744		-2.128757882	1.69E-10	2.07E-09
ENSMMUG00000021859	zona pellucida like domain containing 1 [Source:VGNC Symbol;Acc:VGNC:79650]	0.515021593	0.000378611	0.001517514
ENSMMUG00000063181		0.410272919	0.010738002	0.028739321
ENSMMUG00000059753		-0.951955049	2.45E-08	2.17E-07
ENSMMUG00000021567	neurexophilin and PC-esterase domain family member 3 [Source:VGNC Symbol;Acc:VGNC:75476]	-0.725470231	1.53E-07	1.20E-06
ENSMMUG00000058605	Metazoan signal recognition particle RNA [Source:RFAM;Acc:RF00017]	-0.847225946	0.000165244	0.00071654
ENSMMUG00000057768		-1.445279772	5.52E-15	1.18E-13
ENSMMUG00000024598	U6 spliceosomal RNA [Source:RFAM;Acc:RF00026]	-3.956039781	3.04E-05	0.000155162
ENSMMUG00000038349	crystallin beta-gamma domain containing 3 [Source:NCBI gene;Acc:698282]	1.091194055	3.21E-19	1.02E-17
ENSMMUG00000049786		-1.181779028	0.018920317	0.04660089
ENSMMUG00000043727	zinc finger protein 654 [Source:VGNC Symbol;Acc:VGNC:82560]	-2.023214941	5.63E-05	0.000270409
ENSMMUG00000047994	CGG triplet repeat binding protein 1 [Source:VGNC Symbol;Acc:VGNC:84203]	-1.946558354	8.72E-08	7.09E-07
ENSMMUG00000033738	RNA, U6atac small nuclear 6, pseudogene [Source:HGNC Symbol;Acc:HGNC:46905]	-0.945121763	1.27E-06	8.45E-06
ENSMMUG00000000519	vestigial like family member 3 [Source:VGNC Symbol;Acc:VGNC:79430]	-1.115253198	0.00796022	0.022227464
ENSMMUG00000050994		-1.587587503	0.000512267	0.001988728
ENSMMUG00000015877	1,4-alpha-glucan branching enzyme 1 [Source:VGNC Symbol;Acc:VGNC:72894]	0.698694013	7.91E-05	0.000368403
ENSMMUG00000055096		0.560853622	0.003002174	0.009495039
ENSMMUG00000049913		1.254220101	0.000672723	0.002526973
ENSMMUG00000056687		-1.246807631	2.47E-05	0.000128795
ENSMMUG00000052831		0.450410068	0.017193916	0.042941357
ENSMMUG00000032295	zinc finger protein 595 [Source:VGNC Symbol;Acc:VGNC:79655]	-0.84544874	0.000491425	0.001914827
ENSMMUG00000047256	zinc finger protein 732 [Source:NCBI gene;Acc:716016]	-1.353171289	0.009858141	0.026717148
ENSMMUG00000051381	Metazoan signal recognition particle RNA [Source:RFAM;Acc:RF00017]	-1.734654591	1.12E-10	1.41E-09
ENSMMUG00000039020	zinc finger protein 141 [Source:NCBI gene;Acc:714265]	-1.717889697	2.12E-12	3.35E-11
ENSMMUG00000017457	phosphatidylinositol glycan anchor biosynthesis class G [Source:VGNC Symbol;Acc:VGNC:75865]	-0.600301104	0.0176093	0.043809078

ENSMMUG00000017221	transmembrane protein 175 [Source:VGNC Symbol;Acc:VGNC:79736]	-0.488374792	0.018153148	0.044996019
ENSMMUG00000065084		-0.34779509	0.000272646	0.00112628
ENSMMUG00000008323	C-terminal binding protein 1 [Source:VGNC Symbol;Acc:VGNC:71539]	-0.579396566	5.03E-06	2.99E-05
ENSMMUG00000019945	fibroblast growth factor receptor 3 [Source:VGNC Symbol;Acc:VGNC:72653]	1.092048838	4.55E-06	2.73E-05
ENSMMUG00000032306	zinc finger FYVE-type containing 28 [Source:NCBI gene;Acc:720600]	-1.157911178	1.53E-05	8.36E-05
ENSMMUG00000008379	adducin 1 [Source:VGNC Symbol;Acc:VGNC:69528]	1.604915504	5.29E-14	1.02E-12
ENSMMUG00000008383	major facilitator superfamily domain containing 10 [Source:NCBI gene;Acc:720098]	1.916043812	1.33E-22	6.35E-21
ENSMMUG00000008385	NOP14 nucleolar protein [Source:VGNC Symbol;Acc:VGNC:75194]	1.546475632	1.83E-18	5.49E-17
ENSMMUG00000008392	huntingtin [Source:VGNC Symbol;Acc:VGNC:73468]	-0.871253599	3.47E-09	3.50E-08
ENSMMUG00000006234	acyl-CoA oxidase 3, pristanoyl [Source:VGNC Symbol;Acc:VGNC:69382]	0.481718334	0.000110735	0.000499907
ENSMMUG00000030400	SH3 domain and tetratricopeptide repeats 1 [Source:NCBI gene;Acc:722649]	-0.747132284	4.21E-06	2.54E-05
ENSMMUG00000053541		-1.417346976	0.000712094	0.002655356
ENSMMUG00000015099	janus kinase and microtubule interacting protein 1 [Source:VGNC Symbol;Acc:VGNC:73662]	-0.518929919	0.002557565	0.008245368
ENSMMUG00000023152		0.949737299	0.010016978	0.027083968
ENSMMUG00000019563	collapsin response mediator protein 1 [Source:VGNC Symbol;Acc:VGNC:71494]	1.590188038	7.65E-20	2.64E-18
ENSMMUG00000057703		1.460754271	3.64E-08	3.11E-07
ENSMMUG00000020861	EvC ciliary complex subunit 1 [Source:VGNC Symbol;Acc:VGNC:72445]	1.514371038	1.41E-12	2.28E-11
ENSMMUG00000020860	EvC ciliary complex subunit 2 [Source:VGNC Symbol;Acc:VGNC:72301]	0.554588152	0.002010898	0.006687962
ENSMMUG00000016922	serine/threonine kinase 32B [Source:VGNC Symbol;Acc:VGNC:78114]	0.864136483	0.001314365	0.004595008
ENSMMUG00000060830		-3.558343724	0.000877553	0.003192863
ENSMMUG00000023242	transmembrane protein 128 [Source:VGNC Symbol;Acc:VGNC:82257]	-0.695686128	0.001748907	0.005911223
ENSMMUG00000017761	zinc finger protein 518B [Source:VGNC Symbol;Acc:VGNC:79802]	1.271912805	0.012039448	0.031769576
ENSMMUG00000049640		-0.9049966	4.77E-05	0.000233059
ENSMMUG00000056982		0.535912734	0.0002142	0.000907855
ENSMMUG00000061061		3.335161494	0.007109199	0.020137523
ENSMMUG00000053853	C1q and TNF related 7 [Source:NCBI gene;Acc:714049]	0.478140124	0.007943949	0.022194567
ENSMMUG00000009388	F-box and leucine rich repeat protein 5 [Source:VGNC Symbol;Acc:VGNC:81408]	3.693120621	0.000412316	0.001637559
ENSMMUG00000061298		3.805620323	7.57E-05	0.000354141
ENSMMUG00000015298	CD38 molecule [Source:VGNC Symbol;Acc:VGNC:70762]	1.276067189	0.00223212	0.00730736
ENSMMUG00000008162	fibroblast growth factor binding protein 1 [Source:VGNC Symbol;Acc:VGNC:72536]	2.139274278	4.87E-27	3.72E-25
ENSMMUG00000008163	fibroblast growth factor binding protein 2 [Source:VGNC Symbol;Acc:VGNC:72599]	1.54922462	0.008445294	0.023417845
ENSMMUG00000044289		-1.846896779	0.012935469	0.033761528
ENSMMUG00000023116	LIM domain binding 2 [Source:VGNC Symbol;Acc:VGNC:74246]	-1.084121682	6.42E-08	5.33E-07
ENSMMUG00000018070	non-SMC condensin I complex subunit G [Source:VGNC Symbol;Acc:VGNC:75014]	-2.298821612	0.019999732	0.048846513
ENSMMUG00000018073	ligand dependent nuclear receptor corepressor like [Source:NCBI gene;Acc:715285]	-1.233362441	6.50E-08	5.39E-07
ENSMMUG00000057338		-0.597181929	0.01633241	0.041150127
ENSMMUG00000060440	leucine rich repeat LGI family member 2 [Source:NCBI gene;Acc:693308]	-0.657318787	1.86E-10	2.27E-09
ENSMMUG00000003629	phosphatidylinositol 4-kinase type 2 beta [Source:NCBI gene;Acc:693561]	2.007368466	2.33E-05	0.000122099

ENSMMUG00000053638		-0.732710321	0.005928706	0.017209377
ENSMMUG00000049298	cholecystokinin A receptor [Source:NCBI gene;Acc:694742]	7.193475326	3.28E-13	5.72E-12
ENSMMUG00000057711		0.80892162	0.003016837	0.009529736
ENSMMUG00000055119		-2.631333553	0.011781892	0.031189835
ENSMMUG00000051768		-2.793078326	3.65E-12	5.57E-11
ENSMMUG00000060404		1.658662022	0.00869044	0.024004326
ENSMMUG00000049637		0.73673111	0.005318347	0.015649025
ENSMMUG00000057377		-1.894822333	0.012228492	0.032190001
ENSMMUG00000000237	TBC1 domain family member 1 [Source:VGNC Symbol;Acc:VGNC:78103]	0.565078536	0.001404178	0.004871103
ENSMMUG00000050580		0.51227272	0.011053543	0.029488045
ENSMMUG00000055079		0.848302232	5.91E-10	6.70E-09
ENSMMUG00000003363	Kruppel like factor 3 [Source:VGNC Symbol;Acc:VGNC:73840]	2.433638801	0.000591412	0.002253505
ENSMMUG00000004471	family with sequence similarity 114 member A1 [Source:VGNC Symbol;Acc:VGNC:72342]	1.098886578	0.004048159	0.012357803
ENSMMUG00000002755	kelch like family member 5 [Source:VGNC Symbol;Acc:VGNC:74188]	-2.044933107	0.000154867	0.000674698
ENSMMUG00000050388	Metazoan signal recognition particle RNA [Source:RFAM;Acc:RF00017]	-0.736678343	0.015951627	0.040307144
ENSMMUG00000009324	replication factor C subunit 1 [Source:NCBI gene;Acc:698987]	0.864996257	0.000257474	0.001071666
ENSMMUG00000008834	klotho beta [Source:VGNC Symbol;Acc:VGNC:73835]	1.932257109	0.001462065	0.005043598
ENSMMUG00000001892	UDP-glucose 6-dehydrogenase [Source:VGNC Symbol;Acc:VGNC:78711]	-1.349185294	1.55E-07	1.21E-06
ENSMMUG00000063857	ras homolog family member H [Source:NCBI gene;Acc:700499]	0.354244847	0.015203728	0.03867432
ENSMMUG00000012036	RNA binding motif protein 47 [Source:VGNC Symbol;Acc:VGNC:76576]	-0.640495315	2.29E-05	0.000120034
ENSMMUG00000012561	NOP2/Sun RNA methyltransferase family member 7 [Source:VGNC Symbol;Acc:VGNC:75463]	-1.424865824	2.95E-13	5.15E-12
ENSMMUG00000055441		2.160995871	6.71E-08	5.55E-07
ENSMMUG00000007805	LIM and calponin homology domains 1 [Source:VGNC Symbol;Acc:VGNC:74155]	-1.14759481	0.002479946	0.008017648
ENSMMUG00000051296	paired like homeobox 2B [Source:NCBI gene;Acc:701940]	-1.361371641	0.000137309	0.000606218
ENSMMUG00000053719	Metazoan signal recognition particle RNA [Source:RFAM;Acc:RF00017]	-2.739706564	0.00606431	0.017544618
ENSMMUG00000030349	glutaredoxin and cysteine rich domain containing 1 [Source:VGNC Symbol;Acc:VGNC:73262]	-1.276489539	0.016863757	0.042302014
ENSMMUG00000018625	NIPA like domain containing 1 [Source:NCBI gene;Acc:704998]	-0.97335879	6.24E-06	3.64E-05
ENSMMUG00000055221	Metazoan signal recognition particle RNA [Source:RFAM;Acc:RF00017]	-1.659509044	0.000870851	0.003173932
ENSMMUG00000006703	solute carrier family 10 member 4 [Source:VGNC Symbol;Acc:VGNC:77553]	-1.482205007	8.89E-09	8.38E-08
ENSMMUG00000006704	zygote arrest 1 [Source:VGNC Symbol;Acc:VGNC:79251]	-1.063055109	6.61E-06	3.85E-05
ENSMMUG00000006705	FRY like transcription coactivator [Source:VGNC Symbol;Acc:VGNC:72803]	-0.871622868	0.001873394	0.006289421
ENSMMUG00000056196		-1.153828258	0.000563763	0.002162041
ENSMMUG00000052435	Metazoan signal recognition particle RNA [Source:RFAM;Acc:RF00017]	-1.877591938	2.29E-06	1.45E-05
ENSMMUG00000014868	OCIA domain containing 1 [Source:VGNC Symbol;Acc:VGNC:75409]	1.436847001	4.76E-11	6.27E-10
ENSMMUG00000058229	U6 spliceosomal RNA [Source:RFAM;Acc:RF00026]	3.396065351	5.34E-07	3.80E-06
ENSMMUG00000008514	lin-54 DREAM MuvB core complex component [Source:VGNC Symbol;Acc:VGNC:74123]	1.687360163	4.80E-05	0.000234131
ENSMMUG00000008510	THAP domain containing 9 [Source:VGNC Symbol;Acc:VGNC:79379]	1.291458135	1.98E-05	0.000105405

ENSMMUG00000052743	stearoyl-CoA desaturase 5 [Source:NCBI gene;Acc:694079]	-0.868087192	0.000837882	0.00307037
ENSMMUG00000059119		-2.334146691	0.015029086	0.038315513
ENSMMUG00000001485	transmembrane protein 150C [Source:NCBI gene;Acc:694136]	1.619874062	0.010230359	0.027599824
ENSMMUG00000048621	enolase-phosphatase 1 [Source:NCBI gene;Acc:106998330]	2.395680542	3.33E-12	5.13E-11
ENSMMUG00000060484	heterogeneous nuclear ribonucleoprotein D [Source:NCBI gene;Acc:694867]	1.172414746	2.28E-05	0.000119548
ENSMMUG00000052348		1.606140018	5.12E-07	3.65E-06
ENSMMUG00000021988	N-alpha-acetyltransferase 11, NatA catalytic subunit [Source:NCBI gene;Acc:696758]	1.316712585	2.14E-12	3.38E-11
ENSMMUG00000007366		1.0880028	1.53E-07	1.20E-06
ENSMMUG00000002564	Fraser extracellular matrix complex subunit 1 [Source:VGNC Symbol;Acc:VGNC:72743]	2.009739034	7.01E-23	3.41E-21
ENSMMUG00000045609	CCR4-NOT transcription complex subunit 6 like [Source:NCBI gene;Acc:697981]	3.140102799	1.04E-38	2.34E-36
ENSMMUG00000019901	septin 11 [Source:VGNC Symbol;Acc:VGNC:77310]	-0.538609649	0.002090244	0.006914639
ENSMMUG00000019904	cyclin I [Source:VGNC Symbol;Acc:VGNC:70733]	-1.539204714	0.003081393	0.00971451
ENSMMUG00000018186	sosondowah ankyrin repeat domain family member B [Source:VGNC Symbol;Acc:VGNC:78033]	-0.89175768	2.23E-11	3.05E-10
ENSMMUG00000020919	shroom family member 3 [Source:VGNC Symbol;Acc:VGNC:77352]	2.156890749	2.69E-08	2.36E-07
ENSMMUG00000020916	coiled-coil domain containing 158 [Source:NCBI gene;Acc:699735]	1.230069546	3.86E-17	1.02E-15
ENSMMUG00000020913	scavenger receptor class B member 2 [Source:VGNC Symbol;Acc:VGNC:77026]	-1.117083308	0.000168009	0.00072662
ENSMMUG00000020910	nucleoporin 54 [Source:VGNC Symbol;Acc:VGNC:75420]	-1.843218131	5.53E-08	4.63E-07
ENSMMUG00000002153	G3BP stress granule assembly factor 2 [Source:NCBI gene;Acc:701343]	-3.158018862	0.008367303	0.023243527
ENSMMUG00000003833	Ras association domain family member 6 [Source:VGNC Symbol;Acc:VGNC:76515]	1.495574613	3.93E-05	0.000194482
ENSMMUG00000003830	alpha fetoprotein [Source:VGNC Symbol;Acc:VGNC:69741]	1.421059612	0.005036072	0.014942967
ENSMMUG00000003827	albumin [Source:VGNC Symbol;Acc:VGNC:69782]	1.591697365	1.61E-09	1.71E-08
ENSMMUG00000000790	ankyrin repeat domain 17 [Source:VGNC Symbol;Acc:VGNC:84240]	2.301261813	4.30E-13	7.42E-12
ENSMMUG00000052832		1.222801763	0.002064603	0.006843554
ENSMMUG00000006031	casein kappa [Source:VGNC Symbol;Acc:VGNC:71522]	-1.858124124	0.007895882	0.02207691
ENSMMUG00000062248	follicular dendritic cell secreted protein [Source:NCBI gene;Acc:707784]	0.993051615	3.45E-05	0.000173845
ENSMMUG00000006029	odontogenic, ameloblast associated [Source:NCBI gene;Acc:707906]	1.001297054	0.000328411	0.001332879
ENSMMUG00000048823		-1.149759708	3.33E-09	3.37E-08
ENSMMUG00000004849	casein beta [Source:VGNC Symbol;Acc:VGNC:71523]	-1.360891696	6.84E-09	6.58E-08
ENSMMUG00000047324	casein alpha s1 [Source:HGNC Symbol;Acc:HGNC:2445]	-1.768859048	0.000432314	0.001708581
ENSMMUG00000004667	sulfotransferase family 1E member 1 [Source:NCBI gene;Acc:708519]	-1.120687412	0.017216037	0.042960428
ENSMMUG00000009390	centromere protein C [Source:VGNC Symbol;Acc:VGNC:71007]	-0.34980243	0.014478482	0.037128806
ENSMMUG00000001919	clock circadian regulator [Source:VGNC Symbol;Acc:VGNC:71262]	-1.0360796	5.28E-09	5.17E-08
ENSMMUG00000001918	transmembrane protein 165 [Source:VGNC Symbol;Acc:VGNC:79875]	-1.966065565	4.94E-09	4.85E-08
ENSMMUG00000010113	steroid 5 alpha-reductase 3 [Source:VGNC Symbol;Acc:VGNC:82246]	-1.399446828	2.30E-07	1.74E-06
ENSMMUG00000057204	Metazoan signal recognition particle RNA [Source:RFAM;Acc:RF00017]	1.645890293	0.006358248	0.018291525
ENSMMUG00000054500		-1.845912901	0.004765877	0.014261089
ENSMMUG00000060048		-0.677329477	0.015313601	0.0389271

ENSMMUG00000059659		-0.967094992	0.000471697	0.001844743
ENSMMUG00000052882	RAS like family 11 member B [Source:NCBI gene;Acc:701397]	2.318974789	0.00326742	0.010244246
ENSMMUG00000018943	spermatogenesis associated 18 [Source:VGNC Symbol;Acc:VGNC:78013]	-1.808252808	6.99E-07	4.88E-06
ENSMMUG00000018939	leucine rich repeat containing 66 [Source:VGNC Symbol;Acc:VGNC:74415]	-1.798929169	1.35E-13	2.46E-12
ENSMMUG00000008579	defective in cullin neddylation 1 domain containing 4 [Source:VGNC Symbol;Acc:VGNC:71809]	-1.752918107	4.46E-07	3.21E-06
ENSMMUG00000003726	protein tyrosine phosphatase non-receptor type 13 [Source:VGNC Symbol;Acc:VGNC:76599]	-1.188422965	6.04E-05	0.000288704
ENSMMUG00000059657	chromosome 5 C4orf36 homolog [Source:NCBI gene;Acc:700500]	-1.194060104	0.001182767	0.004186262
ENSMMUG00000014076	AF4/FMR2 family member 1 [Source:VGNC Symbol;Acc:VGNC:69817]	2.259196747	9.13E-06	5.16E-05
ENSMMUG00000051677		1.339535416	2.42E-11	3.30E-10
ENSMMUG00000014080	kelch like family member 8 [Source:VGNC Symbol;Acc:VGNC:74076]	-1.334282688	4.29E-05	0.000211203
ENSMMUG00000019040	hydroxysteroid 17-beta dehydrogenase 13 [Source:VGNC Symbol;Acc:VGNC:73417]	1.30304659	6.28E-12	9.25E-11
ENSMMUG00000019041	hydroxysteroid 17-beta dehydrogenase 11 [Source:VGNC Symbol;Acc:VGNC:73431]	0.994822681	2.20E-05	0.000115948
ENSMMUG00000008795	polycystin 2, transient receptor potential cation channel [Source:NCBI gene;Acc:702179]	-1.167848353	1.24E-06	8.26E-06
ENSMMUG00000008797	ATP binding cassette subfamily G member 2 (Junior blood group) [Source:NCBI gene;Acc:574307]	-1.221549968	1.19E-06	8.00E-06
ENSMMUG00000028039	U6 spliceosomal RNA [Source:RFAM;Acc:RF00026]	-2.121513522	0.003701039	0.011401415
ENSMMUG00000058698		-1.00439793	0.011805842	0.031242084
ENSMMUG00000010138	HECT and RLD domain containing E3 ubiquitin protein ligase 3 [Source:VGNC Symbol;Acc:VGNC:73365]	0.659941474	2.35E-05	0.00012301
ENSMMUG00000058544	nucleosome assembly protein 1 like 5 [Source:NCBI gene;Acc:703223]	0.870154608	2.61E-07	1.96E-06
ENSMMUG00000010141	family with sequence similarity 13 member A [Source:VGNC Symbol;Acc:VGNC:72299]	0.914283404	3.78E-07	2.75E-06
ENSMMUG00000032793	U6 spliceosomal RNA [Source:RFAM;Acc:RF00026]	-2.70922892	0.000479313	0.001873048
ENSMMUG00000057606	GPRIN family member 3 [Source:NCBI gene;Acc:703567]	-2.6738693	3.15E-19	1.00E-17
ENSMMUG00000049396	Metazoan signal recognition particle RNA [Source:RFAM;Acc:RF00017]	-2.738920183	2.87E-06	1.79E-05
ENSMMUG00000022003	multimerin 1 [Source:VGNC Symbol;Acc:VGNC:74904]	1.677189008	1.02E-06	6.91E-06
ENSMMUG00000056021		-1.139453881	0.020529936	0.049919486
ENSMMUG00000022246	PDZ and LIM domain 5 [Source:VGNC Symbol;Acc:VGNC:75816]	1.04478326	5.57E-06	3.28E-05
ENSMMUG00000060760		1.430559254	2.14E-08	1.91E-07
ENSMMUG00000006702	bone morphogenetic protein receptor type 1B [Source:VGNC Symbol;Acc:VGNC:70267]	-1.753143761	3.33E-05	0.000168126
ENSMMUG00000006309	unc-5 netrin receptor C [Source:VGNC Symbol;Acc:VGNC:78723]	-3.239279278	2.91E-06	1.81E-05
ENSMMUG00000011702	tetraspanin 5 [Source:VGNC Symbol;Acc:VGNC:79086]	-1.905054142	7.00E-09	6.73E-08
ENSMMUG00000063972		-1.158123304	3.73E-06	2.27E-05
ENSMMUG00000011066	eukaryotic translation initiation factor 4E [Source:NCBI gene;Acc:706751]	-2.117982883	0.0019523	0.006517922
ENSMMUG00000040764	alcohol dehydrogenase 1A [Source:NCBI gene;Acc:100424103]	-1.350298686	4.37E-07	3.15E-06
ENSMMUG00000018738	alcohol dehydrogenase 1B (class I), beta polypeptide [Source:NCBI gene;Acc:707908]	-0.906213116	8.02E-08	6.54E-07
ENSMMUG00000061366		0.604949008	0.001240145	0.00436331
ENSMMUG00000016880	solute carrier family 39 member 8 [Source:VGNC Symbol;Acc:VGNC:77541]	-2.403900596	3.12E-10	3.68E-09

ENSMMUG00000037551	Metazoan signal recognition particle RNA [Source:RFAM;Acc:RF00017]	-2.564027706	1.53E-11	2.13E-10
ENSMMUG00000003736	nuclear factor kappa B subunit 1 [Source:VGNC Symbol;Acc:VGNC:75339]	-2.553960537	3.10E-10	3.66E-09
ENSMMUG00000003739	mannosidase beta [Source:VGNC Symbol;Acc:VGNC:74486]	2.43834061	3.53E-23	1.79E-21
ENSMMUG00000042419		2.142382859	0.000935958	0.003386255
ENSMMUG00000011206	solute carrier family 9 member B1 [Source:NCBI gene;Acc:710949]	0.961201095	1.34E-09	1.44E-08
ENSMMUG00000011207		0.862105487	0.001015289	0.003648347
ENSMMUG00000019083	solute carrier family 9 member B2 [Source:VGNC Symbol;Acc:VGNC:77773]	1.025821369	4.12E-06	2.49E-05
ENSMMUG00000019085	3-hydroxybutyrate dehydrogenase 2 [Source:VGNC Symbol;Acc:VGNC:70161]	1.239538644	3.55E-12	5.43E-11
ENSMMUG00000019089	centromere protein E [Source:VGNC Symbol;Acc:VGNC:71008]	1.19192897	2.86E-10	3.40E-09
ENSMMUG00000049998	tachykinin receptor 3 [Source:NCBI gene;Acc:653013]	1.441007761	0.000496111	0.001931563
ENSMMUG00000010941	Rho guanine nucleotide exchange factor 38 [Source:VGNC Symbol;Acc:VGNC:82087]	-0.506466574	0.000182915	0.00078627
ENSMMUG00000011722	integrator complex subunit 12 [Source:VGNC Symbol;Acc:VGNC:73647]	-0.509786635	0.002370547	0.007714552
ENSMMUG00000018811	glutathione S-transferase C-terminal domain containing [Source:VGNC Symbol;Acc:VGNC:73234]	-0.749037552	0.005741904	0.016729359
ENSMMUG00000023428	nephronectin [Source:VGNC Symbol;Acc:VGNC:75266]	-2.255970383	0.015337842	0.038961422
ENSMMUG00000023256	lymphoid enhancer binding factor 1 [Source:VGNC Symbol;Acc:VGNC:74251]	0.448872014	0.009474942	0.025848673
ENSMMUG00000009066	60S ribosomal protein L26 [Source:NCBI gene;Acc:696134]	2.291906157	0.001675507	0.005688591
ENSMMUG00000060432		-1.272973578	0.001405364	0.004874077
ENSMMUG00000052609	ribosomal protein L34 [Source:NCBI gene;Acc:696636]	-3.492844739	7.13E-15	1.51E-13
ENSMMUG00000011902	oligosaccharyltransferase complex non-catalytic subunit [Source:NCBI gene;Acc:697283]	-3.659965308	2.29E-32	2.80E-30
ENSMMUG00000055759	Metazoan signal recognition particle RNA [Source:RFAM;Acc:RF00017]	-2.857076863	2.00E-14	4.01E-13
ENSMMUG00000010043	epidermal growth factor [Source:VGNC Symbol;Acc:VGNC:72171]	1.080331627	1.60E-07	1.25E-06
ENSMMUG00000050694	ELOVL fatty acid elongase 6 [Source:NCBI gene;Acc:698870]	0.390198907	0.011056669	0.029491081
ENSMMUG00000062794		3.214104802	2.16E-21	8.96E-20
ENSMMUG00000008705	ubiquitin specific peptidase 53 [Source:VGNC Symbol;Acc:VGNC:79793]	-2.620248285	0.000567425	0.002173835
ENSMMUG00000007305	phosphodiesterase 5A [Source:VGNC Symbol;Acc:VGNC:75918]	-2.107224686	0.015078879	0.038429245
ENSMMUG00000054168	mitotic arrest deficient 2 like 1 [Source:NCBI gene;Acc:708574]	-2.211670562	0.012341558	0.032457049
ENSMMUG00000009816	exosome component 9 [Source:VGNC Symbol;Acc:VGNC:72477]	1.247702686	0.003300721	0.010322583
ENSMMUG00000061098		1.8533901	4.29E-26	2.86E-24
ENSMMUG00000007422	spermatogenesis associated 5 [Source:VGNC Symbol;Acc:VGNC:77952]	1.477814817	3.08E-12	4.78E-11
ENSMMUG00000001848	sprouty RTK signaling antagonist 1 [Source:VGNC Symbol;Acc:VGNC:77867]	1.348600609	4.56E-13	7.86E-12
ENSMMUG00000056171		-1.247500425	0.001453272	0.005017932
ENSMMUG00000054312		-1.916314005	0.002029241	0.006738389
ENSMMUG00000011810	FAT atypical cadherin 4 [Source:VGNC Symbol;Acc:VGNC:72552]	-2.831593914	1.92E-11	2.64E-10
ENSMMUG00000057895		-3.234926824	0.000192897	0.000825112
ENSMMUG00000051329		1.759395012	6.62E-07	4.65E-06
ENSMMUG00000049109		1.882545791	0.002702221	0.008660889
ENSMMUG00000010444	ring finger protein 150 [Source:VGNC Symbol;Acc:VGNC:76805]	2.935987419	0.001370858	0.004769257

ENSMMUG00000060157	zinc finger protein 330 [Source:NCBI gene;Acc:699364]	0.712572931	7.42E-06	4.27E-05
ENSMMUG00000003697	inositol polyphosphate-4-phosphatase type II B [Source:VGNC Symbol;Acc:VGNC:73748]	1.672782698	1.60E-11	2.23E-10
ENSMMUG00000044429	glycophorin A (MNS blood group) [Source:NCBI gene;Acc:574101]	0.890037798	0.011819087	0.031265976
ENSMMUG00000014173		-1.96405214	1.22E-07	9.75E-07
ENSMMUG00000007968	hedgehog interacting protein [Source:VGNC Symbol;Acc:VGNC:73169]	-2.159348634	4.90E-16	1.14E-14
ENSMMUG00000004432	anaphase promoting complex subunit 10 [Source:VGNC Symbol;Acc:VGNC:69682]	-1.125864615	0.008331602	0.023161842
ENSMMUG00000004433	ATP binding cassette subfamily E member 1 [Source:VGNC Symbol;Acc:VGNC:69581]	-1.038507799	0.004108688	0.012529683
ENSMMUG00000004437	OTU deubiquitinase 4 [Source:VGNC Symbol;Acc:VGNC:75675]	-1.160571435	0.001299805	0.004552695
ENSMMUG00000005380	zinc finger protein 827 [Source:NCBI gene;Acc:706018]	0.747075832	0.000716751	0.002669367
ENSMMUG00000014953	Rho GTPase activating protein 10 [Source:VGNC Symbol;Acc:VGNC:69914]	-1.043069675	0.005108265	0.015111822
ENSMMUG00000014955	nuclear receptor subfamily 3 group C member 2 [Source:VGNC Symbol;Acc:VGNC:75516]	-2.707973929	5.84E-11	7.62E-10
ENSMMUG00000002211	doublecortin like kinase 2 [Source:VGNC Symbol;Acc:VGNC:71670]	0.784733784	0.001220365	0.004302902
ENSMMUG00000002212	LPS responsive beige-like anchor protein [Source:NCBI gene;Acc:693823]	-1.215782443	0.000155616	0.000677566
ENSMMUG00000039441	mab-21 like 2 [Source:NCBI gene;Acc:693948]	-2.379208425	1.57E-05	8.52E-05
ENSMMUG00000059101		-2.714793188	2.63E-08	2.31E-07
ENSMMUG00000020330	F-box and WD repeat domain containing 7 [Source:VGNC Symbol;Acc:VGNC:72516]	-1.946864296	0.002234506	0.007310329
ENSMMUG00000064587		-2.065807151	0.000857112	0.003130781
ENSMMUG00000063665		-1.77138673	0.000220418	0.000931018
ENSMMUG00000052327		-1.852854807	7.24E-06	4.18E-05
ENSMMUG00000018490	ADP ribosylation factor interacting protein 1 [Source:VGNC Symbol;Acc:VGNC:69878]	-2.112434506	1.06E-26	7.83E-25
ENSMMUG00000018493	FH2 domain containing 1 [Source:VGNC Symbol;Acc:VGNC:72598]	-2.027340096	2.17E-09	2.26E-08
ENSMMUG00000065105		-1.668297712	0.000998244	0.003597561
ENSMMUG00000039869		-0.948418339	0.000587404	0.00223996
ENSMMUG00000009001	transmembrane 131 like [Source:VGNC Symbol;Acc:VGNC:79781]	-1.361722194	2.75E-05	0.000141764
ENSMMUG00000057060		-1.057109156	0.003754619	0.011552074
ENSMMUG00000057643		0.815446486	1.82E-07	1.40E-06
ENSMMUG00000052999	lecithin retinol acyltransferase [Source:NCBI gene;Acc:698871]	0.600078325	1.62E-07	1.26E-06
ENSMMUG00000064074		0.828784204	6.76E-05	0.00031934
ENSMMUG00000021147	RNA binding motif protein 46 [Source:VGNC Symbol;Acc:VGNC:76555]	0.653109568	0.000113754	0.000511724
ENSMMUG00000023244	guanylate cyclase 1 soluble subunit alpha 1 [Source:VGNC Symbol;Acc:VGNC:73327]	1.546305376	0.005020761	0.0149035
ENSMMUG00000022019	cathepsin O [Source:VGNC Symbol;Acc:VGNC:71561]	0.712572665	1.78E-07	1.38E-06
ENSMMUG00000064707		2.519127864	2.44E-14	4.84E-13
ENSMMUG00000053790		2.004340004	9.39E-14	1.76E-12
ENSMMUG00000010581	Rap guanine nucleotide exchange factor 2 [Source:VGNC Symbol;Acc:VGNC:76493]	2.05097277	2.12E-05	0.000111762
ENSMMUG00000053650		-3.053311332	0.003779244	0.011622172
ENSMMUG00000015799	tolloid like 1 [Source:VGNC Symbol;Acc:VGNC:78369]	-1.1699449	1.44E-05	7.87E-05

ENSMMUG00000045101		0.677565622	0.000593721	0.002261721
ENSMMUG00000006851	SPARC (osteonectin), cwcv and kazal like domains proteoglycan 3 [Source:NCBI gene;Acc:705964]	3.956170642	1.97E-23	1.04E-21
ENSMMUG00000001879	annexin A10 [Source:VGNC Symbol;Acc:VGNC:69945]	1.224497212	5.11E-08	4.29E-07
ENSMMUG00000001880	DExD/H-box helicase 60 [Source:VGNC Symbol;Acc:VGNC:71711]	1.366260358	8.42E-23	4.07E-21
ENSMMUG00000003634	DExD/H-box 60 like [Source:VGNC Symbol;Acc:VGNC:71720]	-1.34592002	6.19E-06	3.61E-05
ENSMMUG00000013730	WD repeat domain 17 [Source:NCBI gene;Acc:700546]	0.626402551	4.66E-07	3.34E-06
ENSMMUG00000064304		-0.601586124	5.14E-05	0.000249041
ENSMMUG00000049670		-4.067583233	0.001216322	0.004292732
ENSMMUG00000015733	sorbin and SH3 domain containing 2 [Source:VGNC Symbol;Acc:VGNC:77767]	-1.221434795	0.012495278	0.032768342
ENSMMUG00000011633	kallikrein B1 [Source:VGNC Symbol;Acc:VGNC:74195]	1.865288995	0.002138114	0.007045863
ENSMMUG00000011634	coagulation factor XI [Source:VGNC Symbol;Acc:VGNC:72460]	1.610522344	0.012411907	0.032613144
ENSMMUG00000018051	FAT atypical cadherin 1 [Source:VGNC Symbol;Acc:VGNC:72617]	-0.901922821	8.80E-15	1.85E-13
ENSMMUG00000050600		2.326158663	0.006298225	0.018139964
ENSMMUG00000058448		0.622772483	0.018569641	0.045821594
ENSMMUG00000050465	ZFP42 zinc finger protein [Source:NCBI gene;Acc:707621]	2.803868801	4.76E-26	3.14E-24
ENSMMUG00000010967	tripartite motif family like 2 [Source:VGNC Symbol;Acc:VGNC:79603]	3.423798528	0.000384956	0.001537956
ENSMMUG00000054799		-2.084057895	0.000156103	0.000679087
ENSMMUG00000064721		-1.948148329	0.000263211	0.001091637
ENSMMUG00000052287		-2.911046796	3.71E-07	2.70E-06
ENSMMUG00000044628		-2.677168598	2.79E-07	2.08E-06
ENSMMUG00000063077	S100 calcium binding protein B [Source:NCBI gene;Acc:708117]	1.538834345	0.000795132	0.002931099
ENSMMUG00000015797	disco interacting protein 2 homolog A [Source:VGNC Symbol;Acc:VGNC:71641]	0.973056317	3.64E-07	2.66E-06
ENSMMUG00000062496		3.650044665	0.001040256	0.003726435
ENSMMUG00000000544	collagen type VI alpha 1 chain [Source:VGNC Symbol;Acc:VGNC:82084]	2.941314781	0.009040015	0.024821962
ENSMMUG00000055589		2.285706042	7.45E-11	9.60E-10
ENSMMUG00000052777		2.43275464	1.08E-09	1.18E-08
ENSMMUG00000004477	solute carrier family 19 member 1 [Source:VGNC Symbol;Acc:VGNC:77408]	2.090796258	3.04E-08	2.65E-07
ENSMMUG00000004472	collagen type XVIII alpha 1 chain [Source:VGNC Symbol;Acc:VGNC:81291]	2.678674234	2.63E-05	0.000136278
ENSMMUG00000010695	adenosine deaminase RNA specific B1 [Source:VGNC Symbol;Acc:VGNC:69503]	-1.872456002	1.57E-09	1.67E-08
ENSMMUG00000000953	family with sequence similarity 207 member A [Source:VGNC Symbol;Acc:VGNC:72273]	1.076382414	1.79E-07	1.39E-06
ENSMMUG00000000951	integrin subunit beta 2 [Source:VGNC Symbol;Acc:VGNC:73786]	2.258696184	6.44E-45	3.29E-42
ENSMMUG00000048545	small ubiquitin like modifier 3 [Source:NCBI gene;Acc:710764]	-0.459279158	0.014712452	0.037650676
ENSMMUG00000049755	leucine rich repeat containing 3 [Source:NCBI gene;Acc:712418]	3.464153409	3.50E-10	4.08E-09
ENSMMUG00000004175	transient receptor potential cation channel subfamily M member 2 [Source:VGNC Symbol;Acc:VGNC:78655]	1.730311363	3.53E-09	3.56E-08
ENSMMUG00000004173	phosphofructokinase, liver type [Source:VGNC Symbol;Acc:VGNC:81737]	-0.841781752	1.75E-06	1.14E-05
ENSMMUG00000010648	DNA methyltransferase 3 like [Source:NCBI gene;Acc:722184]	-1.837996441	7.74E-13	1.30E-11
ENSMMUG00000005271	PWP2 small subunit processome component [Source:NCBI gene;Acc:722216]	-0.515412698	0.000826258	0.003031521
ENSMMUG00000005269	trafficking protein particle complex 10 [Source:NCBI gene;Acc:713177]	-0.343447984	0.000644292	0.002434358
ENSMMUG00000045179	1-acylglycerol-3-phosphate O-acyltransferase 3 [Source:NCBI gene;Acc:713301]	0.687770401	2.75E-05	0.000141773

ENSMMUG00000012304	ribosomal RNA processing 1 [Source:VGNC Symbol;Acc:VGNC:77050]	-0.655406866	0.003567275	0.011046709
ENSMMUG00000000104	ribosomal RNA processing 1B [Source:VGNC Symbol;Acc:VGNC:77089]	-1.498321044	3.08E-12	4.77E-11
ENSMMUG00000022791	heat shock transcription factor 2 binding protein [Source:VGNC Symbol;Acc:VGNC:73532]	-1.809936024	1.28E-06	8.51E-06
ENSMMUG00000020050	salt inducible kinase 1 [Source:NCBI gene;Acc:722303]	-1.497646934	0.000576555	0.002205393
ENSMMUG00000022107	phosphodiesterase 9A [Source:VGNC Symbol;Acc:VGNC:75925]	-3.66606496	0.000138518	0.000611374
ENSMMUG00000018081	ubiquitin associated and SH3 domain containing A [Source:VGNC Symbol;Acc:VGNC:78687]	-1.638492487	3.25E-08	2.81E-07
ENSMMUG00000057951	U6 spliceosomal RNA [Source:RFAM;Acc:RF00026]	-2.769388502	0.000610177	0.002318452
ENSMMUG00000018079	transmembrane serine protease 3 [Source:VGNC Symbol;Acc:VGNC:78576]	-1.355702114	0.000148294	0.000649692
ENSMMUG00000018058	ATP binding cassette subfamily G member 1 [Source:VGNC Symbol;Acc:VGNC:69584]	-2.12971798	2.43E-11	3.31E-10
ENSMMUG00000018057	uromodulin like 1 [Source:VGNC Symbol;Acc:VGNC:78717]	-1.354594087	1.43E-07	1.13E-06
ENSMMUG00000018053	zinc finger and BTB domain containing 21 [Source:VGNC Symbol;Acc:VGNC:78892]	-0.517287894	0.004321207	0.013094299
ENSMMUG00000015329	MX dynamin like GTPase 1 [Source:VGNC Symbol;Acc:VGNC:75086]	1.181427131	0.001829183	0.00615958
ENSMMUG00000044257	MX dynamin like GTPase 2 [Source:VGNC Symbol;Acc:VGNC:81723]	1.775021374	3.16E-08	2.74E-07
ENSMMUG00000004257	beta-secretase 2 [Source:VGNC Symbol;Acc:VGNC:70216]	1.665752679	1.34E-08	1.23E-07
ENSMMUG00000058004		1.286873368	0.001474912	0.005080823
ENSMMUG00000010296	ETS transcription factor ERG [Source:VGNC Symbol;Acc:VGNC:72427]	2.67359813	0.018751861	0.046232767
ENSMMUG00000056593		3.588011286	3.45E-07	2.53E-06
ENSMMUG00000049784		0.708287039	0.004883253	0.014562365
ENSMMUG00000006962	VPS26 endosomal protein sorting factor C [Source:VGNC Symbol;Acc:VGNC:79850]	0.532057831	0.006263045	0.01804565
ENSMMUG00000017505	tetratricopeptide repeat domain 3 [Source:VGNC Symbol;Acc:VGNC:81785]	0.795644078	7.93E-13	1.33E-11
ENSMMUG00000062048		1.039759143	8.58E-18	2.42E-16
ENSMMUG00000055002	U2 spliceosomal RNA [Source:RFAM;Acc:RF00004]	1.625137354	0.009929858	0.026875954
ENSMMUG00000015566	holocarboxylase synthetase [Source:VGNC Symbol;Acc:VGNC:73477]	-1.019269645	0.001479267	0.005092278
ENSMMUG00000010336	DOP1 leucine zipper like protein B [Source:VGNC Symbol;Acc:VGNC:71832]	1.146778376	1.34E-19	4.45E-18
ENSMMUG00000064897		-0.860564015	0.012957492	0.033801162
ENSMMUG00000053087	mitochondrial ribosomal protein S6 [Source:NCBI gene;Acc:697852]	2.345735527	0.004277967	0.012976529
ENSMMUG00000065271		2.282880304	0.002141745	0.007051971
ENSMMUG00000054417		-0.808670946	2.06E-05	0.000108908
ENSMMUG00000005511	SON DNA binding protein [Source:VGNC Symbol;Acc:VGNC:77689]	1.855536574	6.93E-10	7.81E-09
ENSMMUG00000022702	phosphoribosylglycinamide formyltransferase, phosphoribosylglycinamide synthetase, phosphoribosylaminoimidazole synthetase [Source:VGNC Symbol;Acc:VGNC:72882]	1.315466903	1.03E-06	6.95E-06
ENSMMUG00000003956	interferon alpha and beta receptor subunit 2 [Source:VGNC Symbol;Acc:VGNC:81330]	0.566741603	1.00E-05	5.64E-05
ENSMMUG00000065005	Metazoan signal recognition particle RNA [Source:RFAM;Acc:RF00017]	2.156734875	8.69E-13	1.44E-11
ENSMMUG00000052780		1.97325524	1.65E-24	9.46E-23
ENSMMUG00000051823		4.003723253	0.006490425	0.018617237
ENSMMUG00000004624	synaptojanin 1 [Source:VGNC Symbol;Acc:VGNC:78071]	-0.976032758	5.75E-07	4.07E-06
ENSMMUG00000010304	cilia and flagella associated protein 298 [Source:NCBI gene;Acc:700721]	-0.606265679	4.66E-06	2.79E-05
ENSMMUG00000048824	eva-1 homolog C [Source:NCBI gene;Acc:708681]	1.442711239	1.42E-25	8.84E-24
ENSMMUG00000010295	URB1 ribosome biogenesis homolog [Source:VGNC Symbol;Acc:VGNC:79074]	-2.271752402	0.000895709	0.003253336

ENSMMUG00000059677	melanocortin 2 receptor accessory protein [Source:NCBI gene;Acc:701330]	-1.547848003	0.008142324	0.022688965
ENSMMUG00000059707	MIS18 kinetochore protein A [Source:NCBI gene;Acc:709275]	-3.052352976	1.18E-11	1.67E-10
ENSMMUG00000049457		-0.698043195	0.000766604	0.002834389
ENSMMUG00000019571	TIAM Rac1 associated GEF 1 [Source:VGNC Symbol;Acc:VGNC:81886]	1.733443152	0.008825983	0.02432435
ENSMMUG00000064916		-2.219974179	0.008760942	0.02417205
ENSMMUG00000056363		-1.899015642	4.45E-05	0.000218468
ENSMMUG00000033200	Small nucleolar RNA U3 [Source:RFAM;Acc:RF00012]	2.590060347	0.00472493	0.0141557
ENSMMUG00000061208	MAP3K7 C-terminal like [Source:NCBI gene;Acc:713717]	2.154349392	1.02E-50	1.16E-47
ENSMMUG00000064638	Y RNA [Source:RFAM;Acc:RF00019]	2.695291358	0.001927151	0.006449933
ENSMMUG00000003023	chaperonin containing TCP1 subunit 8 [Source:VGNC Symbol;Acc:VGNC:70749]	1.565850487	0.009028252	0.024794256
ENSMMUG00000015728	listerin E3 ubiquitin protein ligase 1 [Source:VGNC Symbol;Acc:VGNC:74275]	1.029558603	0.012073431	0.031842253
ENSMMUG00000017605	mitochondrial ribosomal protein L39 [Source:VGNC Symbol;Acc:VGNC:74737]	0.960376524	1.24E-07	9.85E-07
ENSMMUG00000018743	ubiquitin specific peptidase 25 [Source:VGNC Symbol;Acc:VGNC:78744]	4.838025534	1.84E-10	2.25E-09
ENSMMUG00000052195		3.752426532	2.35E-08	2.09E-07
ENSMMUG00000060808		3.574240915	2.43E-09	2.52E-08
ENSMMUG00000020090	ATP-binding cassette transporter 13 [Source:NCBI gene;Acc:574326]	-0.495951535	0.005607109	0.016388142
ENSMMUG00000060576		2.050963023	2.77E-32	3.34E-30
ENSMMUG00000057702		2.217479812	6.41E-38	1.36E-35
ENSMMUG00000060168		-0.743151939	0.000705839	0.002635384
ENSMMUG00000065108		-1.398017803	0.001290992	0.004522893
ENSMMUG00000017056	Sad1 and UNC84 domain containing 1 [Source:VGNC Symbol;Acc:VGNC:78161]	3.485589769	3.44E-14	6.68E-13
ENSMMUG00000007204	cytochrome P450 family 2 subfamily W member 1 [Source:VGNC Symbol;Acc:VGNC:81300]	1.508437374	0.001803838	0.006079755
ENSMMUG00000007206	chromosome 3 C7orf50 homolog [Source:VGNC Symbol;Acc:VGNC:70609]	2.672019525	4.60E-05	0.000225352
ENSMMUG00000007208	G protein-coupled receptor 146 [Source:VGNC Symbol;Acc:VGNC:72979]	2.030823174	7.47E-08	6.13E-07
ENSMMUG00000054497		-1.426184792	0.00774524	0.021717133
ENSMMUG00000003283	extracellular leucine rich repeat and fibronectin type III domain containing 1 [Source:VGNC Symbol;Acc:VGNC:72204]	1.489382049	3.67E-18	1.07E-16
ENSMMUG00000015529	eukaryotic translation initiation factor 3 subunit B [Source:VGNC Symbol;Acc:VGNC:72193]	-0.646594606	0.000176271	0.000759472
ENSMMUG00000055562	Metazoan signal recognition particle RNA [Source:RFAM;Acc:RF00017]	-1.301594759	0.008461871	0.023445154
ENSMMUG00000022624	putative speedy protein E16 [Source:NCBI gene;Acc:718058]	-1.102254974	4.46E-07	3.21E-06
ENSMMUG00000015546	chromosome 3 C7orf26 homolog [Source:VGNC Symbol;Acc:VGNC:70604]	0.884685685	0.003058098	0.009649276
ENSMMUG00000015545	zinc finger DHHC-type palmitoyltransferase 4 [Source:VGNC Symbol;Acc:VGNC:79144]	0.583237205	0.013344727	0.034658897
ENSMMUG00000010292	diacylglycerol lipase beta [Source:VGNC Symbol;Acc:VGNC:71803]	1.768120186	0.019951653	0.048769246
ENSMMUG00000021075	Rac family small GTPase 1 [Source:NCBI gene;Acc:717755]	1.023981455	3.07E-09	3.13E-08
ENSMMUG00000003167	cytohesin 3 [Source:VGNC Symbol;Acc:VGNC:71829]	1.928630165	9.72E-19	2.97E-17
ENSMMUG00000030422	radial spoke head 10 homolog B [Source:NCBI gene;Acc:100424218]	-0.460923202	0.000549693	0.002117389
ENSMMUG00000007970	CCZ1 homolog, vacuolar protein trafficking and biogenesis associated [Source:NCBI gene;Acc:718128]	1.841195472	3.97E-14	7.68E-13
ENSMMUG00000015477	lemur tyrosine kinase 2 [Source:VGNC Symbol;Acc:VGNC:74309]	1.539989563	1.43E-13	2.61E-12
ENSMMUG00000015479	tectonin beta-propeller repeat containing 1 [Source:VGNC Symbol;Acc:VGNC:79348]	1.596625463	8.37E-39	1.94E-36
ENSMMUG00000013276	BAR/IMD domain containing adaptor protein 2 like 1 [Source:VGNC Symbol;Acc:VGNC:70109]	-0.557745731	0.009169422	0.025130758

ENSMMUG00000059873		-0.693971778	0.019381856	0.047563491
ENSMMUG00000023318	transformation/transcription domain associated protein [Source:VGNC Symbol;Acc:VGNC:78657]	0.822016611	1.62E-19	5.27E-18
ENSMMUG00000040393	zinc finger protein 655 [Source:NCBI gene;Acc:718811]	3.418225303	2.73E-26	1.85E-24
ENSMMUG00000055483	transmembrane protein 225B [Source:NCBI gene;Acc:718838]	2.130412406	0.00246942	0.007991334
ENSMMUG00000022728	zinc finger and SCAN domain containing 25 [Source:VGNC Symbol;Acc:VGNC:79344]	2.550932597	1.66E-24	9.47E-23
ENSMMUG00000022730	cytochrome P450, family 3, subfamily A, polypeptide 5 [Source:NCBI gene;Acc:678692]	1.974977326	5.89E-29	5.29E-27
ENSMMUG00000050824		-1.835132429	0.000800846	0.002947764
ENSMMUG00000022502	tripartite motif containing 4 [Source:VGNC Symbol;Acc:VGNC:79021]	-0.775393695	0.005084647	0.015059978
ENSMMUG00000022504	alpha-2-glycoprotein 1, zinc-binding [Source:VGNC Symbol;Acc:VGNC:70201]	1.092733939	4.60E-08	3.88E-07
ENSMMUG00000004021	zinc finger protein 3 [Source:VGNC Symbol;Acc:VGNC:81889]	-1.428561265	0.013786336	0.035662195
ENSMMUG00000013450	TATA-box binding protein associated factor 6 [Source:VGNC Symbol;Acc:VGNC:78093]	1.0193723	0.009719616	0.026399617
ENSMMUG00000056451	Metazoan signal recognition particle RNA [Source:RFAM;Acc:RF00017]	-2.359383403	0.019957068	0.048774444
ENSMMUG00000046663	Metazoan signal recognition particle RNA [Source:RFAM;Acc:RF00017]	-1.108300907	1.82E-05	9.78E-05
ENSMMUG00000048302	ArfGAP with FG repeats 2 [Source:VGNC Symbol;Acc:VGNC:80329]	-0.920131929	6.01E-07	4.25E-06
ENSMMUG00000015311	leucine rich repeats and calponin homology domain containing 4 [Source:VGNC Symbol;Acc:VGNC:74441]	-0.980781939	1.26E-08	1.16E-07
ENSMMUG00000015309	F-box protein 24 [Source:VGNC Symbol;Acc:VGNC:72495]	-1.033399641	1.58E-09	1.68E-08
ENSMMUG00000002947	procollagen C-endopeptidase enhancer [Source:VGNC Symbol;Acc:VGNC:75782]	-0.935340503	5.00E-11	6.57E-10
ENSMMUG00000002949	motile sperm domain containing 3 [Source:VGNC Symbol;Acc:VGNC:74823]	2.427017868	1.06E-16	2.67E-15
ENSMMUG00000002950	transferrin receptor 2 [Source:VGNC Symbol;Acc:VGNC:81770]	2.478546128	3.38E-29	3.12E-27
ENSMMUG00000004080	G protein subunit beta 2 [Source:VGNC Symbol;Acc:VGNC:81318]	-2.194191048	0.000633222	0.002397418
ENSMMUG00000004082	GRB10 interacting GYF protein 1 [Source:VGNC Symbol;Acc:VGNC:73158]	-1.339389226	9.62E-05	0.000440957
ENSMMUG00000022840	zonadhesin [Source:VGNC Symbol;Acc:VGNC:81797]	-1.142836857	8.25E-05	0.000383476
ENSMMUG00000060459		0.728077868	3.35E-06	2.05E-05
ENSMMUG00000063570	Metazoan signal recognition particle RNA [Source:RFAM;Acc:RF00017]	-4.614056882	2.28E-05	0.000119676
ENSMMUG00000030377	MLX interacting protein like [Source:VGNC Symbol;Acc:VGNC:81722]	-2.995586843	0.002750433	0.008798302
ENSMMUG00000011367	BUD23 rRNA methyltransferase and ribosome maturation factor [Source:VGNC Symbol;Acc:VGNC:70325]	-3.74557861	0.000192836	0.00082509
ENSMMUG00000056841		1.349099906	0.000139819	0.000615468
ENSMMUG00000050132		1.556699989	1.91E-08	1.72E-07
ENSMMUG00000061790		-0.773343204	0.010883557	0.029081649
ENSMMUG00000056980		2.816973354	1.48E-06	9.73E-06
ENSMMUG00000047155	zinc finger protein 679 [Source:NCBI gene;Acc:106995568]	3.742049873	0.000737973	0.002739459
ENSMMUG00000059100		-0.607271203	0.001339239	0.004672059
ENSMMUG00000015272	chaperonin containing TCP1 subunit 6A [Source:NCBI gene;Acc:703326]	0.465560848	0.00095166	0.003440546
ENSMMUG00000051107		-1.4441051	0.000169681	0.000732994
ENSMMUG00000058499		3.682979297	0.00048568	0.001894933
ENSMMUG00000060184	SEC61 translocon subunit gamma [Source:NCBI gene;Acc:716898]	4.570903562	7.29E-11	9.41E-10
ENSMMUG00000001308	V-set and transmembrane domain containing 2A [Source:VGNC Symbol;Acc:VGNC:79720]	2.920351361	0.004193253	0.012750858

ENSMMUG00000056368		0.474992834	9.93E-05	0.000454654
ENSMMUG00000064560		-0.920720612	0.001418816	0.004913242
ENSMMUG00000019992	growth factor receptor bound protein 10 [Source:VGNC Symbol;Acc:VGNC:73157]	-1.540344068	0.007975303	0.022261197
ENSMMUG00000019991	dopa decarboxylase [Source:VGNC Symbol;Acc:VGNC:71755]	0.972826514	1.11E-11	1.57E-10
ENSMMUG00000049620		-0.881347417	0.014184142	0.036487569
ENSMMUG00000044759		-1.42114583	4.12E-12	6.23E-11
ENSMMUG00000043922	spermatogenesis associated 48 [Source:VGNC Symbol;Acc:VGNC:81762]	-1.185129816	1.34E-10	1.66E-09
ENSMMUG00000016191	ATP binding cassette subfamily A member 13 [Source:VGNC Symbol;Acc:VGNC:69402]	2.267180806	1.95E-05	0.000104155
ENSMMUG00000019393	tensin 3 [Source:VGNC Symbol;Acc:VGNC:79324]	-2.250593761	0.006168726	0.017815426
ENSMMUG00000050300		-1.281108624	0.000367213	0.001474621
ENSMMUG00000052540		-1.518194742	4.92E-06	2.93E-05
ENSMMUG00000000962	H2A.Z variant histone 2 [Source:VGNC Symbol;Acc:VGNC:81324]	-1.582805804	2.50E-06	1.57E-05
ENSMMUG00000002433	calcium/calmodulin dependent protein kinase II beta [Source:VGNC Symbol;Acc:VGNC:70524]	1.409222466	0.001104802	0.003938513
ENSMMUG00000002430	YKT6 v-SNARE homolog [Source:VGNC Symbol;Acc:VGNC:81796]	1.344061733	0.00306882	0.009678987
ENSMMUG00000002427	glucokinase [Source:VGNC Symbol;Acc:VGNC:72901]	1.683665126	4.72E-20	1.66E-18
ENSMMUG00000002411	drebrin like [Source:NCBI gene;Acc:720621]	2.033206508	1.97E-38	4.29E-36
ENSMMUG00000002426	myosin light chain 7 [Source:VGNC Symbol;Acc:VGNC:75002]	1.67368644	7.86E-19	2.41E-17
ENSMMUG00000002423	DNA polymerase delta 2, accessory subunit [Source:VGNC Symbol;Acc:VGNC:76203]	1.619254457	5.21E-16	1.21E-14
ENSMMUG00000002422	AE binding protein 1 [Source:VGNC Symbol;Acc:VGNC:80328]	1.686981427	5.82E-12	8.63E-11
ENSMMUG00000002421	DNA polymerase mu [Source:VGNC Symbol;Acc:VGNC:81742]	4.311570649	0.001930204	0.006458692
ENSMMUG00000064860	phosphoglycerate mutase 2 [Source:NCBI gene;Acc:720615]	4.076184601	0.002767728	0.008845991
ENSMMUG00000019023	cytochrome c oxidase assembly factor 1 homolog [Source:VGNC Symbol;Acc:VGNC:81290]	0.733971688	0.000323841	0.001317216
ENSMMUG00000017382	biliverdin reductase A [Source:VGNC Symbol;Acc:VGNC:70256]	0.747512654	2.34E-10	2.81E-09
ENSMMUG00000019022	serine/threonine kinase 17a [Source:VGNC Symbol;Acc:VGNC:77913]	0.792137281	5.05E-09	4.95E-08
ENSMMUG00000019015	HECT, C2 and WW domain containing E3 ubiquitin protein ligase 1 [Source:VGNC Symbol;Acc:VGNC:73217]	0.65329296	6.70E-06	3.89E-05
ENSMMUG00000057956		0.566669349	0.004919767	0.014656439
ENSMMUG00000035562	U6 spliceosomal RNA [Source:RFAM;Acc:RF00026]	-2.994305083	0.002934456	0.009320621
ENSMMUG00000024428	Y RNA [Source:RFAM;Acc:RF00019]	1.997156736	2.87E-05	0.000147249
ENSMMUG00000053696	Metazoan signal recognition particle RNA [Source:RFAM;Acc:RF00017]	1.911110414	3.63E-05	0.000181996
ENSMMUG00000060273	mitochondrial ribosomal protein L32 [Source:NCBI gene;Acc:701820]	2.231967637	6.06E-17	1.56E-15
ENSMMUG00000022451	proteasome 20S subunit alpha 2 [Source:NCBI gene;Acc:701683]	1.927685705	4.93E-07	3.53E-06
ENSMMUG00000052530		2.075720079	7.69E-08	6.30E-07
ENSMMUG00000001702	succinyl-CoA:glutarate-CoA transferase [Source:VGNC Symbol;Acc:VGNC:81767]	1.822288767	8.14E-09	7.73E-08
ENSMMUG00000065307		-1.311702867	0.000729285	0.002709242
ENSMMUG00000050335		3.602500268	0.000202773	0.000863869
ENSMMUG00000021186	anillin actin binding protein [Source:VGNC Symbol;Acc:VGNC:69610]	-1.064332868	0.000650092	0.002453146
ENSMMUG00000010703	KIAA0895 [Source:VGNC Symbol;Acc:VGNC:73884]	-1.989321367	3.11E-05	0.000158456
ENSMMUG00000010702	endonuclease/exonuclease/phosphatase family domain containing 1 [Source:VGNC Symbol;Acc:VGNC:72085]	-1.399026446	0.003254096	0.010213386
ENSMMUG00000006980	septin 7 [Source:VGNC Symbol;Acc:VGNC:81759]	1.646001673	0.01188291	0.031406801
ENSMMUG00000001350	HERPUD family member 2 [Source:VGNC Symbol;Acc:VGNC:73216]	2.870745611	1.63E-30	1.64E-28
ENSMMUG00000058423		3.434423456	3.02E-20	1.09E-18

ENSMMUG00000002886	Bardet-Biedl syndrome 9 [Source:NCBI gene;Acc:708412]	2.134378194	1.08E-24	6.32E-23
ENSMMUG00000050858	Metazoan signal recognition particle RNA [Source:RFAM;Acc:RF00017]	-1.23108546	3.74E-05	0.000186376
ENSMMUG00000011695	5'-nucleotidase, cytosolic IIIA [Source:VGNC Symbol;Acc:VGNC:75423]	-0.484287021	0.005177292	0.015288497
ENSMMUG00000001625	phosphodiesterase 1C [Source:VGNC Symbol;Acc:VGNC:75911]	-0.659018981	0.001162354	0.004122866
ENSMMUG00000022485	protein phosphatase 1 regulatory subunit 17 [Source:VGNC Symbol;Acc:VGNC:76270]	-0.611731802	0.004244405	0.012895827
ENSMMUG00000022484	ITPR interacting domain containing 1 [Source:VGNC Symbol;Acc:VGNC:73667]	-2.969777818	5.84E-06	3.43E-05
ENSMMUG00000055809		-2.601490714	3.84E-12	5.85E-11
ENSMMUG00000060878		0.764293976	0.013986577	0.036073205
ENSMMUG00000062454		2.112283168	8.67E-18	2.44E-16
ENSMMUG00000039009		1.976169907	1.28E-10	1.59E-09
ENSMMUG00000012529	pleckstrin homology domain containing A8 [Source:VGNC Symbol;Acc:VGNC:81741]	1.714535984	2.27E-09	2.36E-08
ENSMMUG00000050459		-0.43272137	0.009315905	0.025475729
ENSMMUG00000032973	mml-mir-550 [Source:miRBase;Acc:MI0007826]	-1.169590542	0.000253547	0.001056209
ENSMMUG00000020184	carboxypeptidase vitellogenic like [Source:VGNC Symbol;Acc:VGNC:71406]	-1.281012499	5.59E-05	0.000268457
ENSMMUG00000039317	cAMP responsive element binding protein 5 [Source:VGNC Symbol;Acc:VGNC:81295]	-1.993257055	8.00E-06	4.58E-05
ENSMMUG00000055193		-2.651639549	0.015346659	0.038964382
ENSMMUG00000000517	family with sequence similarity 126 member A [Source:VGNC Symbol;Acc:VGNC:72327]	-1.149259324	0.005271589	0.01552068
ENSMMUG00000042472	Metazoan signal recognition particle RNA [Source:RFAM;Acc:RF00017]	0.7994617	0.001803009	0.006078342
ENSMMUG00000060787		1.550548756	0.020549911	0.049959866
ENSMMUG00000007821	ATP binding cassette subfamily B member 5 [Source:VGNC Symbol;Acc:VGNC:80321]	-2.61845033	1.64E-20	6.18E-19
ENSMMUG00000006898	integrin subunit beta 8 [Source:VGNC Symbol;Acc:VGNC:73791]	-2.159967251	1.17E-15	2.63E-14
ENSMMUG00000011170	histone deacetylase 9 [Source:VGNC Symbol;Acc:VGNC:73165]	-1.983882415	0.000384355	0.001536383
ENSMMUG00000052574		-1.648216014	5.96E-20	2.08E-18
ENSMMUG00000020418	diacylglycerol kinase beta [Source:VGNC Symbol;Acc:VGNC:71782]	-1.377601667	3.14E-07	2.31E-06
ENSMMUG00000008632	myosin, light chain 6, alkali, smooth muscle and non-muscle-like [Source:NCBI gene;Acc:710137]	-1.519079435	9.68E-05	0.00044338
ENSMMUG00000005159	transmembrane protein 106B [Source:VGNC Symbol;Acc:VGNC:79355]	0.81362549	0.011581068	0.030729511
ENSMMUG00000060770		0.940800519	8.60E-05	0.000398644
ENSMMUG00000012694	thrombospondin type 1 domain containing 7A [Source:VGNC Symbol;Acc:VGNC:79347]	-1.766476793	0.001361108	0.004740542
ENSMMUG00000045513		2.360324855	1.01E-13	1.88E-12
ENSMMUG00000008698	cyclin dependent kinase 6 [Source:VGNC Symbol;Acc:VGNC:70983]	3.256583619	0.01380264	0.035698142
ENSMMUG00000019802	family with sequence similarity 133 member B [Source:NCBI gene;Acc:702054]	2.796458742	1.74E-09	1.84E-08
ENSMMUG00000040007	peroxisomal biogenesis factor 1 [Source:VGNC Symbol;Acc:VGNC:81736]	4.365781379	0.000216579	0.000916369
ENSMMUG00000015685	zinc finger protein 804B [Source:VGNC Symbol;Acc:VGNC:81890]	2.08281431	2.40E-49	1.84E-46
ENSMMUG00000022697	STEAP4 metalloreductase [Source:VGNC Symbol;Acc:VGNC:77974]	-1.216802258	9.97E-09	9.34E-08
ENSMMUG00000022695	ADAM metallopeptidase domain 22 [Source:VGNC Symbol;Acc:VGNC:69483]	-0.59553865	0.000559275	0.002145382
ENSMMUG00000006766	RUN domain containing 3B [Source:VGNC Symbol;Acc:VGNC:77129]	1.220821448	2.16E-06	1.38E-05
ENSMMUG00000010788	ATP binding cassette subfamily B member 1 [Source:NCBI gene;Acc:574235]	-0.833063962	1.69E-06	1.10E-05

ENSMMUG00000000480	KIAA1324 like [Source:VGNC Symbol;Acc:VGNC:73882]	3.37808	0.000257985	0.001073187
ENSMMUG00000007555	semaphorin 3A [Source:VGNC Symbol;Acc:VGNC:77159]	-1.945364256	0.006534235	0.018710698
ENSMMUG00000018652	hepatocyte growth factor [Source:VGNC Symbol;Acc:VGNC:73373]	-0.397032484	0.001921551	0.006434095
ENSMMUG00000053516		1.818646369	1.24E-07	9.89E-07
ENSMMUG00000042946	CD36 molecule [Source:VGNC Symbol;Acc:VGNC:80857]	0.583879311	0.017560023	0.043719007
ENSMMUG00000049939		-1.034446213	2.22E-13	3.94E-12
ENSMMUG00000009006	round spermatid basic protein 1 like [Source:VGNC Symbol;Acc:VGNC:77097]	-0.97861434	1.51E-06	9.94E-06
ENSMMUG00000012427	F-box and leucine rich repeat protein 13 [Source:VGNC Symbol;Acc:VGNC:81312]	-0.599732702	0.003506234	0.010880405
ENSMMUG00000054290		-1.167125572	6.35E-06	3.71E-05
ENSMMUG00000012066	origin recognition complex subunit 5 [Source:VGNC Symbol;Acc:VGNC:75717]	-0.458603648	0.003211979	0.010091876
ENSMMUG00000062666		-0.608641489	0.00020226	0.000862177
ENSMMUG00000058302		-1.030149063	0.000808051	0.002970598
ENSMMUG00000022727	lysine methyltransferase 2E [Source:VGNC Symbol;Acc:VGNC:74073]	-2.587976146	0.000382628	0.001530305
ENSMMUG00000008189	cadherin related family member 3 [Source:HGNC Symbol;Acc:HGNC:26308]	1.534869871	2.94E-24	1.65E-22
ENSMMUG00000003572	nicotinamide phosphoribosyltransferase [Source:VGNC Symbol;Acc:VGNC:75056]	1.403718595	1.11E-07	8.92E-07
ENSMMUG00000011385	phosphatidylinositol-4,5-bisphosphate 3-kinase catalytic subunit gamma [Source:VGNC Symbol;Acc:VGNC:75998]	-0.499386505	0.000686873	0.002576208
ENSMMUG00000062092		2.856566853	0.001017043	0.003653765
ENSMMUG00000058817		2.676361201	4.35E-21	1.75E-19
ENSMMUG00000002894	dedicator of cytokinesis 4 [Source:VGNC Symbol;Acc:VGNC:71866]	-1.414790416	2.41E-09	2.50E-08
ENSMMUG00000008005	forkhead box P2 [Source:VGNC Symbol;Acc:VGNC:72720]	-1.933618635	0.001198219	0.004231863
ENSMMUG00000007097	testin LIM domain protein [Source:VGNC Symbol;Acc:VGNC:78383]	0.817507318	1.76E-07	1.36E-06
ENSMMUG00000011269	CF transmembrane conductance regulator [Source:VGNC Symbol;Acc:VGNC:71020]	-1.129890481	2.80E-07	2.09E-06
ENSMMUG00000002434	inhibitor of growth family member 3 [Source:VGNC Symbol;Acc:VGNC:73595]	-1.504170922	5.46E-05	0.000263226
ENSMMUG00000054565		-1.026338012	0.013263338	0.034477705
ENSMMUG00000015240	glutamate metabotropic receptor 8 [Source:VGNC Symbol;Acc:VGNC:73292]	-0.640859453	0.003368878	0.010511351
ENSMMUG00000002428	zinc finger protein 800 [Source:VGNC Symbol;Acc:VGNC:79658]	1.404243581	6.34E-14	1.21E-12
ENSMMUG00000046381	ADP ribosylation factor 5 [Source:VGNC Symbol;Acc:VGNC:80792]	-0.420579304	0.012723952	0.033286426
ENSMMUG00000015432	staphylococcal nuclease and tudor domain containing 1 [Source:VGNC Symbol;Acc:VGNC:77798]	-0.742794999	0.000231475	0.000974389
ENSMMUG00000007250	RNA binding motif protein 28 [Source:VGNC Symbol;Acc:VGNC:76519]	-1.228234409	1.22E-05	6.77E-05
ENSMMUG00000037001	U7 small nuclear RNA [Source:RFAM;Acc:RF00066]	-2.457482579	0.003005459	0.009503402
ENSMMUG00000009625	coiled-coil domain containing 136 [Source:VGNC Symbol;Acc:VGNC:70836]	0.307714241	0.004552494	0.013709919
ENSMMUG00000018478	interferon regulatory factor 5 [Source:VGNC Symbol;Acc:VGNC:73763]	1.288037399	4.23E-06	2.55E-05
ENSMMUG00000004311	transportin 3 [Source:VGNC Symbol;Acc:VGNC:79063]	0.833039456	0.020403976	0.049678371
ENSMMUG00000053821		-2.754647652	0.005817234	0.016922241

ENSMMUG00000012110	smoothened, frizzled class receptor [Source:VGNC Symbol;Acc:VGNC:77658]	-1.778828703	2.26E-13	4.00E-12
ENSMMUG00000019358	adenosylhomocysteinase like 2 [Source:VGNC Symbol;Acc:VGNC:80771]	-1.956970048	0.014711105	0.037650676
ENSMMUG00000019359	striatin interacting protein 2 [Source:VGNC Symbol;Acc:VGNC:78194]	-0.34420391	0.0024073	0.007820429
ENSMMUG00000041919	nuclear respiratory factor 1 [Source:VGNC Symbol;Acc:VGNC:81731]	1.041623068	2.07E-09	2.17E-08
ENSMMUG00000017542	ubiquitin conjugating enzyme E2 H [Source:VGNC Symbol;Acc:VGNC:78696]	-0.608994336	8.07E-05	0.000375449
ENSMMUG00000006155	zinc finger C3HC-type containing 1 [Source:VGNC Symbol;Acc:VGNC:79827]	-1.662801794	0.000482791	0.001884156
ENSMMUG00000061007		-1.16358381	0.003709854	0.011422106
ENSMMUG00000010060	serine rich single-pass membrane protein 1 [Source:VGNC Symbol;Acc:VGNC:78014]	-1.247678638	0.000918719	0.003327132
ENSMMUG00000009494	carboxypeptidase A5 [Source:VGNC Symbol;Acc:VGNC:71410]	1.08911495	6.64E-06	3.86E-05
ENSMMUG00000009495	carboxypeptidase A1 [Source:VGNC Symbol;Acc:VGNC:71455]	0.869301193	0.002450673	0.00794393
ENSMMUG00000009499	mesoderm specific transcript [Source:VGNC Symbol;Acc:VGNC:74707]	3.356894211	4.42E-28	3.61E-26
ENSMMUG00000004801	COPI coat complex subunit gamma 2 [Source:VGNC Symbol;Acc:VGNC:71315]	3.308782299	1.62E-27	1.30E-25
ENSMMUG00000001671	coiled-coil-helix-coiled-coil-helix domain containing 3 [Source:VGNC Symbol;Acc:VGNC:80860]	-1.317209823	0.000526406	0.002034559
ENSMMUG00000000916	solute carrier family 35 member B4 [Source:VGNC Symbol;Acc:VGNC:77528]	-1.373153921	0.005214642	0.015365217
ENSMMUG00000013098	aldo-keto reductase family 1 member B10 [Source:NCBI gene;Acc:713150]	1.281255017	0.008946209	0.024600832
ENSMMUG00000004598	bisphosphoglycerate mutase [Source:VGNC Symbol;Acc:VGNC:70275]	-4.134664058	4.68E-05	0.000229381
ENSMMUG00000037485		-5.054477537	4.61E-07	3.31E-06
ENSMMUG00000054821		-3.024358707	0.000107145	0.000486068
ENSMMUG00000006530	ATP/GTP binding protein like 3 [Source:VGNC Symbol;Acc:VGNC:69723]	1.159070239	2.19E-13	3.89E-12
ENSMMUG00000050339	Metazoan signal recognition particle RNA [Source:RFAM;Acc:RF00017]	2.203970241	2.99E-14	5.85E-13
ENSMMUG00000062419		2.341995368	0.00040881	0.001625377
ENSMMUG00000002318	stimulated by retinoic acid 8 [Source:VGNC Symbol;Acc:VGNC:78250]	-1.042229506	0.008200365	0.02281212
ENSMMUG00000016181	CCR4-NOT transcription complex subunit 4 [Source:VGNC Symbol;Acc:VGNC:71389]	-0.642137416	7.38E-06	4.26E-05
ENSMMUG00000016384	nucleoporin 205 [Source:VGNC Symbol;Acc:VGNC:75432]	0.746865111	0.001189256	0.004205212
ENSMMUG00000002995	tripartite motif containing 24 [Source:VGNC Symbol;Acc:VGNC:78562]	1.041342867	1.16E-10	1.46E-09
ENSMMUG00000050262		1.434416881	0.002160465	0.007099424
ENSMMUG00000021676	SVOP like [Source:VGNC Symbol;Acc:VGNC:78224]	0.419497469	0.005401097	0.015861042
ENSMMUG00000052458		0.592786731	0.000114136	0.000513077
ENSMMUG00000006547	ATPase H+ transporting V0 subunit a4 [Source:VGNC Symbol;Acc:VGNC:70189]	-3.490161642	6.62E-05	0.00031345
ENSMMUG00000059192	transmembrane protein 213 [Source:NCBI gene;Acc:710580]	-3.105977567	0.016724643	0.042009902
ENSMMUG00000006548	KIAA1549 [Source:VGNC Symbol;Acc:VGNC:73885]	-3.808575385	8.46E-09	8.01E-08
ENSMMUG00000006770	putative RNA-binding protein Luc7-like 2 [Source:NCBI gene;Acc:711104]	-0.713947039	0.01096803	0.029286259
ENSMMUG00000052228	Metazoan signal recognition particle RNA [Source:RFAM;Acc:RF00017]	2.463484838	2.80E-06	1.74E-05
ENSMMUG00000004224	poly(ADP-ribose) polymerase family member 12 [Source:VGNC Symbol;Acc:VGNC:81734]	1.008740541	1.45E-11	2.04E-10
ENSMMUG00000056175		0.836283136	1.82E-07	1.40E-06
ENSMMUG00000008126	lysine demethylase 7A [Source:VGNC Symbol;Acc:VGNC:73886]	1.233416594	2.52E-21	1.03E-19
ENSMMUG00000010420	makorin ring finger protein 1 [Source:VGNC Symbol;Acc:VGNC:81476]	0.885975338	0.000105419	0.000478825

ENSMMUG00000042299	DENN domain containing 2A [Source:VGNC Symbol;Acc:VGNC:81302]	-1.738584765	0.009106805	0.024972988
ENSMMUG00000015282	aarF domain containing kinase 2 [Source:VGNC Symbol;Acc:VGNC:69473]	-0.580399265	9.31E-05	0.00042791
ENSMMUG00000015284	NADH:ubiquinone oxidoreductase subunit B2 [Source:VGNC Symbol;Acc:VGNC:75156]	0.726121014	8.92E-10	9.83E-09
ENSMMUG00000042793	B-Raf proto-oncogene, serine/threonine kinase [Source:NCBI gene;Acc:693554]	0.618994913	0.000261517	0.001086052
ENSMMUG00000060893		-2.316700084	1.45E-07	1.15E-06
ENSMMUG00000064529		-1.279349189	2.86E-05	0.000146542
ENSMMUG00000055369		0.495477077	0.007389674	0.020798798
ENSMMUG00000021017	acylglycerol kinase [Source:VGNC Symbol;Acc:VGNC:69669]	1.56538975	3.49E-22	1.58E-20
ENSMMUG00000042443	olfactory receptor 9A4 [Source:NCBI gene;Acc:695321]	2.110508517	0.001141513	0.004056706
ENSMMUG00000055710	taste 2 receptor member 38 [Source:NCBI gene;Acc:695549]	2.663952004	5.70E-12	8.46E-11
ENSMMUG00000016273	maltase-glucoamylase [Source:VGNC Symbol;Acc:VGNC:74713]	3.267990782	7.65E-43	3.07E-40
ENSMMUG00000019937	maltase-glucoamylase 2 (putative) [Source:VGNC Symbol;Acc:VGNC:81720]	2.449734132	1.41E-05	7.73E-05
ENSMMUG00000056515		-1.186562485	0.000539738	0.002081747
ENSMMUG00000049575	T cell receptor beta variable 7-2 [Source:HGNC Symbol;Acc:HGNC:12236]	-1.566752373	1.32E-07	1.05E-06
ENSMMUG00000051385		-2.443899031	1.26E-09	1.36E-08
ENSMMUG00000049767		-3.158942937	0.0011937	0.004217911
ENSMMUG00000048246		-3.312225797	0.00024722	0.001033337
ENSMMUG00000057494	T cell receptor beta variable 7-9 [Source:HGNC Symbol;Acc:HGNC:12243]	-2.713401106	2.02E-09	2.12E-08
ENSMMUG00000055612	T cell receptor beta variable 12-5 [Source:HGNC Symbol;Acc:HGNC:12187]	-3.176525627	0.01500055	0.038262497
ENSMMUG00000050269	T cell receptor beta variable 14 [Source:HGNC Symbol;Acc:HGNC:12189]	-2.855975342	3.60E-26	2.42E-24
ENSMMUG00000016286	protease, serine, 1 (trypsin 1) [Source:NCBI gene;Acc:698729]	-1.969104442	1.03E-06	6.99E-06
ENSMMUG00000051032	serine protease 2 [Source:NCBI gene;Acc:698352]	-2.704892791	1.03E-27	8.31E-26
ENSMMUG00000052682		-2.904836671	3.31E-12	5.11E-11
ENSMMUG00000059019		-3.090777764	1.02E-19	3.44E-18
ENSMMUG00000058459		-3.234198744	0.00013969	0.000615085
ENSMMUG00000012628	glutathione S-transferase kappa 1 [Source:VGNC Symbol;Acc:VGNC:73180]	-0.315620176	0.009849266	0.026707736
ENSMMUG00000043809	caspase 2 [Source:VGNC Symbol;Acc:VGNC:80804]	-1.186255553	1.65E-11	2.29E-10
ENSMMUG00000012635	zyxin [Source:VGNC Symbol;Acc:VGNC:78917]	2.203436357	4.29E-32	5.04E-30
ENSMMUG00000055322	EPH receptor A1 [Source:NCBI gene;Acc:706295]	2.177915194	6.03E-43	2.48E-40
ENSMMUG00000058370	taste receptor type 2 member 62 [Source:NCBI gene;Acc:704388]	3.483128502	8.51E-05	0.000394778
ENSMMUG00000042798		-1.561522232	1.63E-11	2.27E-10
ENSMMUG00000063823	U6 spliceosomal RNA [Source:RFAM;Acc:RF00026]	-1.810280928	0.002917837	0.00927777
ENSMMUG00000005136	TRPM8 channel associated factor 1 [Source:VGNC Symbol;Acc:VGNC:79346]	-0.794663596	3.42E-06	2.09E-05

ENSMMUG00000049419		-1.747593014	0.010606244	0.028432862
ENSMMUG00000019856	olfactory receptor 2F2-like [Source:NCBI gene;Acc:100428924]	-1.271811591	1.38E-05	7.56E-05
ENSMMUG00000059211		1.34307846	0.01956055	0.047915915
ENSMMUG00000017541	cullin 1 [Source:VGNC Symbol;Acc:VGNC:71565]	-3.656301707	0.00565168	0.016495653
ENSMMUG00000057973		-1.808366755	2.25E-05	0.000118018
ENSMMUG00000042048	protein disulfide isomerase family A member 4 [Source:NCBI gene;Acc:709716]	0.909555239	0.001508945	0.005186014
ENSMMUG00000018549	zinc finger protein 786 [Source:VGNC Symbol;Acc:VGNC:79338]	2.678115881	1.76E-07	1.36E-06
ENSMMUG00000002497	zinc finger protein 777 [Source:HGNC Symbol;Acc:HGNC:22213]	-1.208154958	2.49E-06	1.57E-05
ENSMMUG00000060626	U6 spliceosomal RNA [Source:RFAM;Acc:RF00026]	1.453159754	0.000464801	0.001822579
ENSMMUG00000051020	zinc finger protein 467 [Source:NCBI gene;Acc:712106]	-1.70731275	2.50E-08	2.20E-07
ENSMMUG00000048892	SCO-spondin [Source:VGNC Symbol;Acc:VGNC:81765]	-1.247892943	2.08E-09	2.18E-08
ENSMMUG00000062384		-1.090244162	0.013851637	0.035812375
ENSMMUG00000022633	replication initiator 1 [Source:VGNC Symbol;Acc:VGNC:76758]	2.702410994	4.47E-21	1.79E-19
ENSMMUG00000022636	GTPase, IMAP family member 8 [Source:VGNC Symbol;Acc:VGNC:72981]	-1.538682991	1.79E-06	1.16E-05
ENSMMUG00000011386	GTPase, IMAP family member 4 [Source:VGNC Symbol;Acc:VGNC:72982]	-2.427431855	1.69E-34	2.79E-32
ENSMMUG00000052830		-2.638059992	4.24E-18	1.23E-16
ENSMMUG00000047276	GTPase, IMAP family member 6 [Source:VGNC Symbol;Acc:VGNC:81315]	-1.846957108	8.61E-13	1.43E-11
ENSMMUG00000017008	GTPase, IMAP family member 2 [Source:VGNC Symbol;Acc:VGNC:72980]	-3.62833975	5.15E-06	3.05E-05
ENSMMUG00000049295	GTPase, IMAP family member 5 [Source:NCBI gene;Acc:713835]	-1.458103467	2.14E-07	1.62E-06
ENSMMUG00000008596	transmembrane protein 176B [Source:VGNC Symbol;Acc:VGNC:79817]	-0.979618184	0.001805152	0.0060828
ENSMMUG00000020435	amine oxidase copper containing 1 [Source:VGNC Symbol;Acc:VGNC:70033]	-1.782190646	8.26E-11	1.05E-09
ENSMMUG00000020436	potassium voltage-gated channel subfamily H member 2 [Source:VGNC Symbol;Acc:VGNC:73980]	-1.499463808	3.66E-08	3.12E-07
ENSMMUG00000054070	IQ motif containing with AAA domain 1 like [Source:NCBI gene;Acc:714415]	-3.265634292	0.006204926	0.017902539
ENSMMUG00000006682	chondroitin polymerizing factor 2 [Source:VGNC Symbol;Acc:VGNC:71131]	-2.052843282	3.20E-05	0.000162339
ENSMMUG00000038898	Metazoan signal recognition particle RNA [Source:RFAM;Acc:RF00017]	0.748247474	0.00017475	0.000753354
ENSMMUG00000012238	polypeptide N-acetylgalactosaminyltransferase 11 [Source:VGNC Symbol;Acc:VGNC:72749]	-1.02991095	0.000309848	0.001264111
ENSMMUG00000007212	lysine methyltransferase 2C [Source:VGNC Symbol;Acc:VGNC:74043]	0.54053632	0.002461731	0.007972806
ENSMMUG00000062591	insulin induced gene 1 [Source:NCBI gene;Acc:718513]	-0.74833205	0.000343123	0.001386894
ENSMMUG00000007770	canopy FGF signaling regulator 1 [Source:VGNC Symbol;Acc:VGNC:71363]	-1.751570351	0.011434734	0.030413954
ENSMMUG00000054296		-1.990145086	0.009081793	0.024918221
ENSMMUG00000039033	nucleolar protein with MIF4G domain 1 [Source:VGNC Symbol;Acc:VGNC:81729]	-3.272591741	4.13E-05	0.000203899
ENSMMUG00000008733	ubiquitin protein ligase E3C [Source:VGNC Symbol;Acc:VGNC:79049]	-1.454117574	1.68E-10	2.06E-09
ENSMMUG00000009611	vasoactive intestinal peptide receptor 2 [Source:VGNC Symbol;Acc:VGNC:78772]	-0.780981845	0.002627904	0.008445207

ENSMMUG00000000752	programmed cell death 6 [Source:NCBI gene;Acc:723043]	0.366255032	0.016957187	0.0424789
ENSMMUG00000003791	centrosomal protein 72 [Source:VGNC Symbol;Acc:VGNC:71099]	-1.608218031	0.001937784	0.006476092
ENSMMUG00000052291		-1.970930048	6.71E-05	0.000317361
ENSMMUG00000061096	tubulin polymerization promoting protein [Source:NCBI gene;Acc:708834]	-1.604523753	0.000899597	0.003264259
ENSMMUG00000010940	telomerase reverse transcriptase [Source:VGNC Symbol;Acc:VGNC:78313]	0.791930163	1.90E-09	2.00E-08
ENSMMUG00000005198	solute carrier family 6 member 3 [Source:VGNC Symbol;Acc:VGNC:77619]	0.580416726	0.007897878	0.022078329
ENSMMUG00000019205	ADAM metallopeptidase with thrombospondin type 1 motif 16 [Source:VGNC Symbol;Acc:VGNC:69439]	-1.220665745	0.001050144	0.003759025
ENSMMUG00000061176		-0.764953282	2.04E-07	1.56E-06
ENSMMUG00000002561	NOP2/Sun RNA methyltransferase 2 [Source:VGNC Symbol;Acc:VGNC:75466]	-2.394938139	0.001584128	0.005400602
ENSMMUG00000018745	chromosome 6 C5orf49 homolog [Source:VGNC Symbol;Acc:VGNC:70646]	-1.254812037	0.014127231	0.036366402
ENSMMUG00000018746	FAST kinase domains 3 [Source:VGNC Symbol;Acc:VGNC:72588]	-1.995336967	9.19E-15	1.93E-13
ENSMMUG00000018747	5-methyltetrahydrofolate-homocysteine methyltransferase reductase [Source:VGNC Symbol;Acc:VGNC:75079]	-1.915497704	2.64E-13	4.64E-12
ENSMMUG00000061922		0.470057465	1.98E-07	1.52E-06
ENSMMUG00000001130	ankyrin repeat domain 33B [Source:VGNC Symbol;Acc:VGNC:69758]	-1.67068306	0.008547942	0.023654782
ENSMMUG00000021942	ANKH inorganic pyrophosphate transport regulator [Source:VGNC Symbol;Acc:VGNC:69612]	-0.92024564	3.03E-06	1.87E-05
ENSMMUG00000065128	Metazoan signal recognition particle RNA [Source:RFAM;Acc:RF00017]	-0.963742945	1.52E-05	8.29E-05
ENSMMUG00000059412		-0.755628637	0.001152581	0.004092118
ENSMMUG00000062083		2.370262923	3.53E-09	3.56E-08
ENSMMUG00000059036		2.588663737	1.29E-05	7.14E-05
ENSMMUG00000064602		1.945350987	0.00034417	0.001390746
ENSMMUG00000059710		1.440194672	0.000873946	0.003184428
ENSMMUG00000049919		1.575557745	0.000867183	0.003161336
ENSMMUG00000060891	histone H3.Y [Source:NCBI gene;Acc:718280]	1.918310016	0.00010832	0.0004902
ENSMMUG00000013954	PDZ domain containing 2 [Source:VGNC Symbol;Acc:VGNC:75829]	-0.39448173	0.005860063	0.017026789
ENSMMUG00000055028		-2.165297262	0.004099459	0.012504109
ENSMMUG00000009574	solute carrier family 45 member 2 [Source:VGNC Symbol;Acc:VGNC:82240]	-0.681959449	0.01517256	0.038628166
ENSMMUG00000018739	sperm flagellar 2 [Source:VGNC Symbol;Acc:VGNC:82243]	-2.773174711	0.000364083	0.001463635
ENSMMUG00000015225	S-phase kinase associated protein 2 [Source:VGNC Symbol;Acc:VGNC:77376]	-2.430165252	0.015734791	0.039833895
ENSMMUG00000006202	solute carrier family 1 member 3 [Source:VGNC Symbol;Acc:VGNC:77411]	-0.537148334	5.93E-07	4.19E-06
ENSMMUG00000052836		0.410043405	0.012477482	0.032729158
ENSMMUG00000056658	Metazoan signal recognition particle RNA [Source:RFAM;Acc:RF00017]	0.714718985	0.01276867	0.033390827
ENSMMUG00000052974		-0.570345599	0.011692549	0.030982949
ENSMMUG00000017163	EGF like, fibronectin type III and laminin G domains [Source:VGNC Symbol;Acc:VGNC:72122]	-0.803128188	1.10E-06	7.44E-06
ENSMMUG00000064324		2.755070488	0.000667196	0.002508115
ENSMMUG00000004774	maestro heat like repeat family member 2B [Source:VGNC Symbol;Acc:VGNC:74837]	2.114313639	1.58E-07	1.23E-06
ENSMMUG00000011878	poly(A) binding protein interacting protein 1 [Source:VGNC Symbol;Acc:VGNC:84476]	2.370124905	1.18E-10	1.48E-09
ENSMMUG00000055022	Metazoan signal recognition particle RNA [Source:RFAM;Acc:RF00017]	2.694694138	0.004632213	0.013911647

ENSMMUG00000047162	fibroblast growth factor 10 [Source:VGNC Symbol;Acc:VGNC:84378]	-1.48624633	1.34E-08	1.23E-07
ENSMMUG00000059786	hyperpolarization activated cyclic nucleotide gated potassium channel 1 [Source:NCBI gene;Acc:704286]	-2.760209266	0.004729244	0.014165763
ENSMMUG00000016149	embigin [Source:VGNC Symbol;Acc:VGNC:72144]	-2.200883157	0.008890059	0.024469112
ENSMMUG00000057857		1.253862376	0.008341424	0.02318278
ENSMMUG00000001753	integrin subunit alpha 1 [Source:VGNC Symbol;Acc:VGNC:73772]	2.096111526	2.14E-26	1.49E-24
ENSMMUG00000039101	pelota mRNA surveillance and ribosome rescue factor [Source:VGNC Symbol;Acc:VGNC:84478]	2.311624608	4.88E-23	2.42E-21
ENSMMUG00000010379	Mtr4 exosome RNA helicase [Source:VGNC Symbol;Acc:VGNC:74989]	-1.908745404	5.11E-10	5.86E-09
ENSMMUG00000030099	phospholipid phosphatase 1 [Source:VGNC Symbol;Acc:VGNC:76226]	-3.499974153	2.12E-12	3.35E-11
ENSMMUG00000061973		-3.15607049	1.12E-35	2.00E-33
ENSMMUG00000010466	solute carrier family 38 member 9 [Source:VGNC Symbol;Acc:VGNC:77574]	1.561783379	1.18E-05	6.55E-05
ENSMMUG00000049885		-1.226718409	8.97E-06	5.10E-05
ENSMMUG00000051661		-1.574505279	4.76E-06	2.84E-05
ENSMMUG00000005181	RAB3C, member RAS oncogene family [Source:VGNC Symbol;Acc:VGNC:81541]	-1.31600016	0.000551032	0.002120345
ENSMMUG00000018379	DEP domain containing 1B [Source:VGNC Symbol;Acc:VGNC:71705]	-0.833924209	0.00073521	0.002729887
ENSMMUG00000021812	ERCC excision repair 8, CSA ubiquitin ligase complex subunit [Source:VGNC Symbol;Acc:VGNC:72425]	0.882772183	5.28E-13	9.04E-12
ENSMMUG00000054780		0.665600227	0.002456474	0.007960995
ENSMMUG00000060505		-1.211699525	0.000192272	0.000822915
ENSMMUG00000021131	regulator of G protein signaling 7 binding protein [Source:VGNC Symbol;Acc:VGNC:76808]	-1.473749648	0.004188131	0.012739252
ENSMMUG00000015041	CWC27 spliceosome associated cyclophilin [Source:VGNC Symbol;Acc:VGNC:71396]	-3.444750767	5.64E-08	4.71E-07
ENSMMUG00000051169		-2.210462582	0.000287849	0.001179551
ENSMMUG00000016514	tripartite motif containing 23 [Source:VGNC Symbol;Acc:VGNC:79962]	0.432186088	2.55E-05	0.000132281
ENSMMUG00000053925	shieldin complex subunit 3 [Source:NCBI gene;Acc:114678803]	0.54672531	0.005471958	0.01604055
ENSMMUG00000016519	trafficking protein particle complex 13 [Source:VGNC Symbol;Acc:VGNC:79549]	0.402167866	0.001385259	0.004813404
ENSMMUG00000016521	small glutamine rich tetratricopeptide repeat containing beta [Source:VGNC Symbol;Acc:VGNC:77296]	0.479491416	2.35E-06	1.48E-05
ENSMMUG00000016522	neurolysin [Source:VGNC Symbol;Acc:VGNC:75183]	-0.824646393	2.25E-10	2.72E-09
ENSMMUG00000038355		-3.128494506	0.006462574	0.018552066
ENSMMUG00000049935		1.639090982	3.44E-12	5.27E-11
ENSMMUG00000046550	coiled-coil domain containing 125 [Source:NCBI gene;Acc:704391]	1.57916914	1.10E-32	1.41E-30
ENSMMUG00000006217	general transcription factor IIH subunit 2 [Source:NCBI gene;Acc:700737]	-2.527204046	1.65E-09	1.75E-08
ENSMMUG00000054237	Y RNA [Source:RFAM;Acc:RF00019]	1.440319921	0.004636044	0.013919931
ENSMMUG00000059671		1.341482624	1.61E-16	3.96E-15
ENSMMUG00000019996	ectodermal-neural cortex 1 [Source:VGNC Symbol;Acc:VGNC:72217]	-3.391831727	0.00343008	0.010670882
ENSMMUG00000064344	Metazoan signal recognition particle RNA [Source:RFAM;Acc:RF00017]	-3.51967974	0.000725704	0.002697291
ENSMMUG00000011061	ankyrin repeat domain 31 [Source:VGNC Symbol;Acc:VGNC:84277]	1.228831444	1.30E-25	8.20E-24
ENSMMUG00000006139	3-hydroxy-3-methylglutaryl-CoA reductase [Source:VGNC Symbol;Acc:VGNC:73484]	0.744488467	3.16E-08	2.74E-07
ENSMMUG00000006141	ceramide transporter 1 [Source:VGNC Symbol;Acc:VGNC:71298]	0.797993048	5.40E-07	3.83E-06
ENSMMUG00000019887	synaptic vesicle glycoprotein 2C [Source:VGNC Symbol;Acc:VGNC:78252]	-0.887327339	3.72E-05	0.000185746

ENSMMUG00000022982	IQ motif containing GTPase activating protein 2 [Source:VGNC Symbol;Acc:VGNC:73756]	-1.314685613	0.001937633	0.006476092
ENSMMUG00000021357	zinc finger BED-type containing 3 [Source:VGNC Symbol;Acc:VGNC:79271]	1.564244743	0.004445951	0.013412012
ENSMMUG00000006792	arylsulfatase B [Source:VGNC Symbol;Acc:VGNC:70023]	1.049911312	0.012861554	0.033592259
ENSMMUG00000006796	betaine--homocysteine S-methyltransferase 2 [Source:VGNC Symbol;Acc:VGNC:70248]	-1.374199433	3.56E-05	0.000178713
ENSMMUG00000046325	junction mediating and regulatory protein, p53 cofactor [Source:VGNC Symbol;Acc:VGNC:84397]	-3.018649378	0.002140506	0.007049456
ENSMMUG00000020731	terminal nucleotidyltransferase 2 [Source:VGNC Symbol;Acc:VGNC:79637]	-1.976871639	0.005649156	0.016491533
ENSMMUG00000036820	U7 small nuclear RNA [Source:RFAM;Acc:RF00066]	1.510182611	9.39E-06	5.30E-05
ENSMMUG00000051429		-2.93263792	2.36E-07	1.78E-06
ENSMMUG00000018283	dihydrofolate reductase [Source:NCBI gene;Acc:711268]	-1.978362159	0.00034279	0.001386125
ENSMMUG00000018284	mutS homolog 3 [Source:VGNC Symbol;Acc:VGNC:74932]	-0.912704304	2.07E-05	0.000109405
ENSMMUG00000054808	Metazoan signal recognition particle RNA [Source:RFAM;Acc:RF00017]	-1.638311763	0.012906996	0.033705009
ENSMMUG00000039944	Ras protein specific guanine nucleotide releasing factor 2 [Source:VGNC Symbol;Acc:VGNC:84486]	-1.80164829	1.65E-07	1.28E-06
ENSMMUG00000006932	creatine kinase, mitochondrial 2 [Source:VGNC Symbol;Acc:VGNC:71231]	-1.129515893	0.005294953	0.015586374
ENSMMUG00000017583	single stranded DNA binding protein 2 [Source:NCBI gene;Acc:711752]	-1.411892454	2.51E-14	4.96E-13
ENSMMUG00000003578	ribosomal protein S23 [Source:NCBI gene;Acc:711914]	-1.620912308	0.013992502	0.036077569
ENSMMUG00000046560	transmembrane protein 167A [Source:VGNC Symbol;Acc:VGNC:84513]	0.972191629	0.000142571	0.000626654
ENSMMUG00000035653	small Cajal body-specific RNA 18 [Source:HGNC Symbol;Acc:HGNC:32559]	2.329167947	0.013928659	0.035969568
ENSMMUG00000064265	X-ray repair cross complementing 4 [Source:NCBI gene;Acc:712291]	0.559842964	0.010278997	0.027715915
ENSMMUG00000011513	versican [Source:VGNC Symbol;Acc:VGNC:79445]	3.505384997	4.04E-69	1.20E-65
ENSMMUG00000061282	hyaluronan and proteoglycan link protein 1 [Source:NCBI gene;Acc:712501]	4.499460004	0.00013173	0.000585072
ENSMMUG00000009319	transmembrane protein 161B [Source:VGNC Symbol;Acc:VGNC:84508]	-1.363082563	1.97E-05	0.000105026
ENSMMUG00000019836	myocyte enhancer factor 2C [Source:VGNC Symbol;Acc:VGNC:84438]	-1.514848511	4.10E-08	3.48E-07
ENSMMUG00000055392		-1.498836401	2.78E-07	2.08E-06
ENSMMUG00000056790		-2.781680955	0.00028231	0.001160061
ENSMMUG00000018972	RNA polymerase III subunit G [Source:VGNC Symbol;Acc:VGNC:84480]	-1.719861406	1.81E-05	9.71E-05
ENSMMUG00000018974	LysM domain containing 3 [Source:VGNC Symbol;Acc:VGNC:74373]	-2.205599995	2.49E-42	9.01E-40
ENSMMUG00000055271		-1.960198354	3.42E-09	3.46E-08
ENSMMUG00000018976	adhesion G protein-coupled receptor V1 [Source:VGNC Symbol;Acc:VGNC:69625]	-1.608149998	1.15E-06	7.74E-06
ENSMMUG00000001722	family with sequence similarity 172 member A [Source:VGNC Symbol;Acc:VGNC:72348]	-0.519128053	0.013735464	0.035543734
ENSMMUG00000001397	multiple C2 and transmembrane domain containing 1 [Source:VGNC Symbol;Acc:VGNC:74589]	1.759847719	1.01E-08	9.43E-08
ENSMMUG00000010632	family with sequence similarity 81 member B [Source:VGNC Symbol;Acc:VGNC:72365]	2.743561889	0.002209183	0.00723998
ENSMMUG00000001209	arylsulfatase family member K [Source:VGNC Symbol;Acc:VGNC:84280]	-1.468797506	2.18E-06	1.39E-05
ENSMMUG00000001211	G protein-coupled receptor 150 [Source:VGNC Symbol;Acc:VGNC:72988]	-1.196016396	0.00582832	0.016951164

ENSMMUG00000001212	Rieske Fe-S domain containing [Source:VGNC Symbol;Acc:VGNC:84487]	-0.956975108	9.32E-07	6.36E-06
ENSMMUG00000001213	spermatogenesis associated 9 [Source:VGNC Symbol;Acc:VGNC:77976]	-0.993775994	6.62E-05	0.00031345
ENSMMUG00000032858	mml-mir-583 [Source:miRBase;Acc:MI0007850]	1.40935926	0.000432387	0.001708581
ENSMMUG00000002063	endoplasmic reticulum aminopeptidase 1 [Source:VGNC Symbol;Acc:VGNC:72066]	0.978532087	2.01E-11	2.77E-10
ENSMMUG00000011043	endoplasmic reticulum aminopeptidase 2 [Source:VGNC Symbol;Acc:VGNC:72141]	0.951507337	2.14E-20	7.83E-19
ENSMMUG00000065221		0.782052356	2.58E-07	1.94E-06
ENSMMUG00000018542	leucyl and cystinyl aminopeptidase [Source:VGNC Symbol;Acc:VGNC:74438]	-0.774661942	1.02E-06	6.90E-06
ENSMMUG00000000307	RIO kinase 2 [Source:VGNC Symbol;Acc:VGNC:76774]	0.506860337	2.15E-05	0.000113379
ENSMMUG00000054789	family with sequence similarity 174 member A [Source:NCBI gene;Acc:702300]	0.728821034	8.54E-06	4.86E-05
ENSMMUG00000007773	solute carrier organic anion transporter family member 4C1 [Source:VGNC Symbol;Acc:VGNC:77764]	3.214044707	6.59E-10	7.44E-09
ENSMMUG00000007844	peptidylglycine alpha-amidating monooxygenase [Source:VGNC Symbol;Acc:VGNC:75752]	-0.667547419	0.014795298	0.037823524
ENSMMUG00000007786	gypsy retrotransposon integrase 1 [Source:VGNC Symbol;Acc:VGNC:73015]	-0.298831963	0.014023619	0.036143598
ENSMMUG00000007787	diphosphoinositol pentakisphosphate kinase 2 [Source:VGNC Symbol;Acc:VGNC:76128]	-0.7135779	0.001314029	0.004594918
ENSMMUG00000059227		3.44757698	0.016028572	0.040467097
ENSMMUG00000054898		-1.877786522	0.002684877	0.008609021
ENSMMUG00000013183	F-box and leucine rich repeat protein 17 [Source:VGNC Symbol;Acc:VGNC:82142]	-1.170150777	2.47E-10	2.97E-09
ENSMMUG00000003316	mannosidase alpha class 2A member 1 [Source:VGNC Symbol;Acc:VGNC:81449]	-0.89024199	1.75E-06	1.13E-05
ENSMMUG00000038051	uncharacterized LOC106998885 [Source:NCBI gene;Acc:106998885]	-1.512475833	3.79E-11	5.06E-10
ENSMMUG00000042638		-2.907870979	0.007772034	0.021775791
ENSMMUG00000011974	calcium/calmodulin dependent protein kinase IV [Source:VGNC Symbol;Acc:VGNC:70526]	-3.126623692	7.43E-24	4.02E-22
ENSMMUG00000063177	StAR related lipid transfer domain containing 4 [Source:NCBI gene;Acc:706654]	-2.710404229	1.67E-24	9.49E-23
ENSMMUG00000002768	neuronal regeneration related protein [Source:VGNC Symbol;Acc:VGNC:84454]	-3.028937863	0.002428359	0.007879469
ENSMMUG00000012192	erythrocyte membrane protein band 4.1 like 4A [Source:VGNC Symbol;Acc:VGNC:72039]	-1.36015545	1.26E-09	1.36E-08
ENSMMUG00000055720		-1.119999588	0.019392548	0.047567233
ENSMMUG00000056959		0.590438909	0.00021578	0.000913767
ENSMMUG00000000330	APC regulator of WNT signaling pathway [Source:VGNC Symbol;Acc:VGNC:84282]	0.55125838	0.008394455	0.0232996
ENSMMUG00000042951	signal recognition particle 19 [Source:NCBI gene;Acc:693829]	1.122361118	3.68E-08	3.14E-07
ENSMMUG00000020488	receptor accessory protein 5 [Source:VGNC Symbol;Acc:VGNC:76864]	1.04731898	1.83E-15	4.06E-14
ENSMMUG00000011130	tripartite motif containing 36 [Source:VGNC Symbol;Acc:VGNC:79023]	-0.272274394	0.020242869	0.049367168
ENSMMUG00000005018	protein geranylgeranyltransferase type I subunit beta [Source:VGNC Symbol;Acc:VGNC:75965]	3.047508443	0.001565572	0.00535038
ENSMMUG00000015114	toll like receptor adaptor molecule 2 [Source:NCBI gene;Acc:100426353]	0.546068171	2.17E-10	2.63E-09
ENSMMUG00000061758	cysteine dioxygenase type 1 [Source:NCBI gene;Acc:696387]	0.473884409	0.000645117	0.002436852
ENSMMUG00000060812	autophagy related 12 [Source:NCBI gene;Acc:696765]	1.344954945	1.96E-12	3.11E-11
ENSMMUG00000015288	adaptor related protein complex 3 subunit sigma 1 [Source:VGNC Symbol;Acc:VGNC:69867]	1.224493028	1.27E-06	8.48E-06
ENSMMUG00000022192		-1.108213369	0.000252777	0.001053888
ENSMMUG00000008738	COMM domain containing 10 [Source:VGNC Symbol;Acc:VGNC:71374]	-1.565143439	3.26E-06	2.01E-05
ENSMMUG00000037127	U6 spliceosomal RNA [Source:RFAM;Acc:RF00026]	-1.206624068	0.005945309	0.017244352

ENSMMUG00000018640	Dmx like 1 [Source:VGNC Symbol;Acc:VGNC:71935]	-1.021632759	0.002555232	0.008239636
ENSMMUG00000063474		-0.759802703	7.61E-07	5.28E-06
ENSMMUG00000059145		-1.248857598	0.001851202	0.006223832
ENSMMUG00000063816	Metazoan signal recognition particle RNA [Source:RFAM;Acc:RF00017]	-2.162171403	0.005128019	0.015161189
ENSMMUG00000018764		-0.863169717	0.008585862	0.023737609
ENSMMUG00000063354		-1.194229261	0.004942532	0.014715469
ENSMMUG00000053794		-1.5042525	1.22E-09	1.32E-08
ENSMMUG00000021006	zinc finger protein 474 [Source:VGNC Symbol;Acc:VGNC:81617]	-1.230557235	0.000441537	0.001737789
ENSMMUG00000046428		-0.774099407	0.01297366	0.033831437
ENSMMUG00000023598	sorting nexin 2 [Source:VGNC Symbol;Acc:VGNC:77682]	1.949648208	0.001343901	0.004686116
ENSMMUG00000006646	casein kinase 1 gamma 3 [Source:VGNC Symbol;Acc:VGNC:81384]	-0.542405597	0.010167779	0.027450968
ENSMMUG00000016683	phosphorylated adaptor for RNA export [Source:NCBI gene;Acc:702980]	-1.001083563	0.018213767	0.045101047
ENSMMUG00000021547	membrane associated ring-CH-type finger 3 [Source:VGNC Symbol;Acc:VGNC:74605]	-2.258197542	0.006133593	0.01772777
ENSMMUG00000037806	chromosome 6 C5orf63 homolog [Source:NCBI gene;Acc:100423212]	-1.707114642	0.004871883	0.014543081
ENSMMUG00000018827	multiple EGF like domains 10 [Source:VGNC Symbol;Acc:VGNC:74617]	-4.233937945	0.001583645	0.005400195
ENSMMUG00000063396		-4.35040039	0.008380505	0.023266822
ENSMMUG00000011214	solute carrier family 12 member 2 [Source:VGNC Symbol;Acc:VGNC:77386]	-2.007415239	8.47E-13	1.41E-11
ENSMMUG00000010682	fibrillin 2 [Source:VGNC Symbol;Acc:VGNC:72624]	-1.625625699	2.80E-10	3.33E-09
ENSMMUG00000003312	chondroitin sulfate synthase 3 [Source:VGNC Symbol;Acc:VGNC:71089]	-2.020913725	0.007226785	0.020410059
ENSMMUG00000058082		0.380293496	0.001634127	0.005555912
ENSMMUG00000016909	meiotic kinetochore factor [Source:NCBI gene;Acc:709179]	0.937602406	1.17E-10	1.47E-09
ENSMMUG00000051989	interleukin 5 [Source:NCBI gene;Acc:710622]	3.247860237	5.43E-05	0.000262176
ENSMMUG00000003131	interleukin 13 [Source:VGNC Symbol;Acc:VGNC:84393]	1.358581968	8.36E-09	7.92E-08
ENSMMUG00000003133	interleukin 4 [Source:VGNC Symbol;Acc:VGNC:73729]	1.465184296	3.86E-05	0.000191667
ENSMMUG00000010313	kinesin family member 3A [Source:VGNC Symbol;Acc:VGNC:74017]	1.853782308	2.19E-26	1.52E-24
ENSMMUG00000010308	cyclin I family member 2 [Source:VGNC Symbol;Acc:VGNC:70923]	1.705776481	7.48E-08	6.14E-07
ENSMMUG00000010309	septin 8 [Source:VGNC Symbol;Acc:VGNC:77248]	1.748127948	1.40E-05	7.68E-05
ENSMMUG00000039781	sosondowah ankyrin repeat domain family member A [Source:VGNC Symbol;Acc:VGNC:84498]	-1.225356557	0.000191784	0.000821299
ENSMMUG00000059080		-1.619621042	6.69E-06	3.89E-05
ENSMMUG00000015640	AF4/FMR2 family member 4 [Source:VGNC Symbol;Acc:VGNC:69629]	-1.479023119	9.07E-08	7.35E-07
ENSMMUG00000026029	Y RNA [Source:RFAM;Acc:RF00019]	-1.307250673	0.004757594	0.014247802
ENSMMUG00000052685		0.563993148	0.006112899	0.017674848
ENSMMUG00000013047	S-phase kinase associated protein 1 [Source:NCBI gene;Acc:100423634]	-3.026084455	6.08E-52	7.51E-49
ENSMMUG00000016641	ubiquitin conjugating enzyme E2 B [Source:VGNC Symbol;Acc:VGNC:78692]	0.874021888	0.012085554	0.031868559

ENSMMUG00000053384		-2.527602802	9.39E-48	6.05E-45
ENSMMUG00000058096	CDKN2A interacting protein N-terminal like [Source:NCBI gene;Acc:710774]	1.174682276	0.002113094	0.006977773
ENSMMUG00000004417	secretion associated Ras related GTPase 1B [Source:VGNC Symbol;Acc:VGNC:76970]	1.074814673	1.34E-13	2.45E-12
ENSMMUG00000012463	DEAD-box helicase 46 [Source:VGNC Symbol;Acc:VGNC:71663]	-1.591866498	1.18E-12	1.93E-11
ENSMMUG00000054798		-0.730976622	0.002974264	0.009426871
ENSMMUG00000060605		1.055217699	3.85E-09	3.85E-08
ENSMMUG00000016357	polycystin 2 like 2, transient receptor potential cation channel [Source:VGNC Symbol;Acc:VGNC:76009]	-2.090785352	8.99E-05	0.000414254
ENSMMUG00000008009	family with sequence similarity 13 member B [Source:VGNC Symbol;Acc:VGNC:72268]	-1.985934546	3.11E-10	3.67E-09
ENSMMUG00000001507	bromodomain containing 8 [Source:VGNC Symbol;Acc:VGNC:70350]	-1.97737799	0.017363454	0.04328459
ENSMMUG00000009025	cell division cycle 25C [Source:VGNC Symbol;Acc:VGNC:70796]	-0.351062122	0.005205237	0.015343604
ENSMMUG00000005871	eukaryotic translation termination factor 1 [Source:VGNC Symbol;Acc:VGNC:84370]	-0.561829181	0.007777581	0.021787216
ENSMMUG00000017985	catenin alpha 1 [Source:VGNC Symbol;Acc:VGNC:84291]	0.783663953	9.43E-08	7.62E-07
ENSMMUG00000001941	CXXC finger protein 5 [Source:VGNC Symbol;Acc:VGNC:71594]	-0.789375354	3.61E-11	4.83E-10
ENSMMUG00000010301	neuregulin 2 [Source:VGNC Symbol;Acc:VGNC:81487]	1.00294627	0.01684026	0.04225737
ENSMMUG00000028372	Y RNA [Source:RFAM;Acc:RF00019]	-2.764437781	0.000298516	0.001220231
ENSMMUG00000019582	ankyrin repeat and KH domain-containing protein 1 [Source:NCBI gene;Acc:698045]	-0.574581905	2.24E-05	0.00011756
ENSMMUG00000001707	amyloid beta precursor protein binding family B member 3 [Source:VGNC Symbol;Acc:VGNC:69869]	2.764009555	2.03E-59	5.01E-56
ENSMMUG00000001725	solute carrier family 35 member A4 [Source:VGNC Symbol;Acc:VGNC:77532]	2.620058528	5.41E-30	5.25E-28
ENSMMUG00000062326		2.720177225	1.19E-30	1.21E-28
ENSMMUG00000024537		3.500097511	6.03E-09	5.85E-08
ENSMMUG00000010007	CD14 molecule [Source:VGNC Symbol;Acc:VGNC:70751]	2.971633883	7.21E-17	1.83E-15
ENSMMUG00000060632		2.62271541	2.81E-09	2.88E-08
ENSMMUG00000018246	transmembrane and coiled-coil domains 6 [Source:VGNC Symbol;Acc:VGNC:84507]	2.17662103	3.16E-39	7.80E-37
ENSMMUG00000056796	NADH:ubiquinone oxidoreductase subunit A2 [Source:NCBI gene;Acc:697736]	2.753004233	1.16E-07	9.29E-07
ENSMMUG00000010008	IK cytokine [Source:VGNC Symbol;Acc:VGNC:73703]	0.592810461	0.008448955	0.023419593
ENSMMUG00000004481	histidyl-tRNA synthetase 1 [Source:VGNC Symbol;Acc:VGNC:84385]	-0.753918388	0.018064329	0.044798328
ENSMMUG00000004483	histidyl-tRNA synthetase 2, mitochondrial [Source:VGNC Symbol;Acc:VGNC:73346]	-0.717697771	7.02E-05	0.000330797
ENSMMUG00000014571	protocadherin alpha-C2 [Source:NCBI gene;Acc:698874]	0.901115734	1.31E-15	2.93E-14
ENSMMUG00000008710	protocadherin 1 [Source:VGNC Symbol;Acc:VGNC:75775]	-1.190112607	0.001090471	0.003891165
ENSMMUG00000006287	DAP3 binding cell death enhancer 1 [Source:VGNC Symbol;Acc:VGNC:71806]	0.634208199	7.53E-06	4.34E-05
ENSMMUG00000006291	protocadherin 12 [Source:VGNC Symbol;Acc:VGNC:75776]	1.012126999	1.27E-06	8.45E-06
ENSMMUG00000009768	ring finger protein 14 [Source:VGNC Symbol;Acc:VGNC:84488]	0.731814854	3.62E-09	3.64E-08
ENSMMUG00000014175	sprouty RTK signaling antagonist 4 [Source:VGNC Symbol;Acc:VGNC:77925]	1.144207941	0.000550874	0.002120287
ENSMMUG00000060402		0.666691253	0.003628019	0.011216077
ENSMMUG00000000419	Rho GTPase activating protein 26 [Source:VGNC Symbol;Acc:VGNC:69846]	2.07334627	2.92E-22	1.34E-20
ENSMMUG00000050405		2.284383604	1.54E-18	4.66E-17
ENSMMUG00000000421	nuclear receptor subfamily 3 group C member 1 [Source:VGNC Symbol;Acc:VGNC:75515]	2.227531594	2.32E-41	7.47E-39
ENSMMUG00000035921	U7 small nuclear RNA [Source:RFAM;Acc:RF00066]	1.717802861	0.004959825	0.014758067
ENSMMUG00000055068		-2.82099922	0.011023542	0.029418595
ENSMMUG00000063348		-0.835625936	3.62E-06	2.21E-05

ENSMMUG00000019291	dihydropyrimidinase like 3 [Source:VGNC Symbol;Acc:VGNC:71985]	-1.081697614	0.002552464	0.008232931
ENSMMUG00000019820	SH3 domain and tetratricopeptide repeats 2 [Source:VGNC Symbol;Acc:VGNC:77337]	1.009560234	2.21E-11	3.02E-10
ENSMMUG00000056140	U6 spliceosomal RNA [Source:RFAM;Acc:RF00026]	1.17395549	0.007109917	0.020137523
ENSMMUG00000064432		-2.090602559	0.016966517	0.042487916
ENSMMUG00000062594		-0.860861861	0.003411771	0.010622834
ENSMMUG00000001758	casein kinase 1 alpha 1 [Source:VGNC Symbol;Acc:VGNC:84290]	-1.349665912	0.010385354	0.027946798
ENSMMUG00000004749	PPARG coactivator 1 beta [Source:VGNC Symbol;Acc:VGNC:76134]	2.302354704	0.000167637	0.000725222
ENSMMUG00000011557	platelet derived growth factor receptor beta [Source:VGNC Symbol;Acc:VGNC:75929]	0.571852724	1.11E-16	2.79E-15
ENSMMUG00000011563	calcium/calmodulin dependent protein kinase II alpha [Source:VGNC Symbol;Acc:VGNC:70523]	2.586090192	0.020142813	0.049171674
ENSMMUG00000005414	N-deacetylase and N-sulfotransferase 1 [Source:VGNC Symbol;Acc:VGNC:75151]	-1.069781669	2.05E-10	2.49E-09
ENSMMUG00000051062		-0.845375452	0.000780195	0.002882485
ENSMMUG00000020679	myozenin 3 [Source:VGNC Symbol;Acc:VGNC:74988]	1.532104395	1.77E-09	1.87E-08
ENSMMUG00000020683	RNA binding motif protein 22 [Source:VGNC Symbol;Acc:VGNC:76535]	2.175961666	8.24E-13	1.38E-11
ENSMMUG00000019764	dynactin subunit 4 [Source:VGNC Symbol;Acc:VGNC:71625]	1.820730293	1.50E-08	1.37E-07
ENSMMUG00000021051	TNFAIP3 interacting protein 1 [Source:VGNC Symbol;Acc:VGNC:79057]	-1.170711129	1.02E-07	8.23E-07
ENSMMUG00000021053	annexin A6 [Source:VGNC Symbol;Acc:VGNC:69951]	-1.135515883	1.80E-06	1.16E-05
ENSMMUG00000053656		0.988681734	7.16E-08	5.89E-07
ENSMMUG00000003499	solute carrier family 36 member 1 [Source:VGNC Symbol;Acc:VGNC:77509]	1.735887095	6.19E-14	1.18E-12
ENSMMUG00000003497	FAT atypical cadherin 2 [Source:VGNC Symbol;Acc:VGNC:72618]	-1.21642151	5.09E-06	3.02E-05
ENSMMUG00000053367	secreted protein acidic and cysteine rich [Source:NCBI gene;Acc:712388]	1.650060118	2.14E-18	6.41E-17
ENSMMUG00000054302	antioxidant 1 copper chaperone [Source:NCBI gene;Acc:712464]	-1.254788537	9.42E-06	5.31E-05
ENSMMUG00000054082		-0.400860418	0.020338112	0.049552219
ENSMMUG00000021228	gem nuclear organelle associated protein 5 [Source:VGNC Symbol;Acc:VGNC:72751]	-0.501191529	0.001279255	0.004488137
ENSMMUG00000011463	kinesin family member 4B [Source:NCBI gene;Acc:715082]	-1.206962476	0.016517444	0.041531653
ENSMMUG00000035086	U2 spliceosomal RNA [Source:RFAM;Acc:RF00004]	-2.73230685	7.31E-05	0.000342891
ENSMMUG00000062861		-2.040934664	4.37E-09	4.33E-08
ENSMMUG00000051081		0.730363884	0.005657389	0.01650582
ENSMMUG00000059241		-2.75978212	6.52E-10	7.37E-09
ENSMMUG00000011471	tetratricopeptide repeat domain 1 [Source:VGNC Symbol;Acc:VGNC:78667]	0.60366505	0.013534378	0.035090004
ENSMMUG00000011472	PWWP domain containing 2A [Source:VGNC Symbol;Acc:VGNC:76564]	1.935395607	2.73E-07	2.04E-06
ENSMMUG00000018044	gamma-aminobutyric acid type A receptor beta2 subunit [Source:VGNC Symbol;Acc:VGNC:72852]	-1.513083902	0.001252008	0.004400162
ENSMMUG00000009171	potassium voltage-gated channel interacting protein 1 [Source:VGNC Symbol;Acc:VGNC:73832]	0.569999624	0.007995088	0.022312223
ENSMMUG00000000570	gamma-aminobutyric acid type A receptor pi subunit [Source:VGNC Symbol;Acc:VGNC:72859]	0.615614203	0.0026485	0.00850341
ENSMMUG00000015840	RAN binding protein 17 [Source:VGNC Symbol;Acc:VGNC:84485]	0.714906585	1.12E-07	8.97E-07
ENSMMUG00000046970	Metazoan signal recognition particle RNA [Source:RFAM;Acc:RF00017]	0.666517866	0.001333061	0.004652694
ENSMMUG00000059105		0.61313347	0.000193984	0.000829524
ENSMMUG00000033319		-3.049165405	0.00695622	0.019770169
ENSMMUG00000003067	endoplasmic reticulum-golgi intermediate compartment 1 [Source:VGNC Symbol;Acc:VGNC:82141]	1.382626135	1.93E-17	5.24E-16
ENSMMUG00000059290	ATPase H+ transporting V0 subunit e1 [Source:NCBI gene;Acc:703342]	0.848634089	0.018984891	0.046717257

ENSMMUG00000019926	cytoplasmic polyadenylation element binding protein 4 [Source:VGNC Symbol;Acc:VGNC:71450]	0.715577335	4.96E-07	3.55E-06
ENSMMUG00000053691	Metazoan signal recognition particle RNA [Source:RFAM;Acc:RF00017]	3.002345603	2.46E-06	1.55E-05
ENSMMUG00000009058	clathrin light chain B [Source:VGNC Symbol;Acc:VGNC:71267]	-2.068542544	0.003356657	0.010480355
ENSMMUG00000009059	Fas associated factor family member 2 [Source:VGNC Symbol;Acc:VGNC:72332]	-2.179433553	0.000109467	0.00049509
ENSMMUG00000013283	cadherin related family member 2 [Source:NCBI gene;Acc:697249]	2.174421308	9.78E-08	7.90E-07
ENSMMUG00000000137	synuclein beta [Source:VGNC Symbol;Acc:VGNC:77670]	2.780065728	0.009727236	0.026415476
ENSMMUG00000002649	unc-5 netrin receptor A [Source:VGNC Symbol;Acc:VGNC:78721]	-2.137523965	9.52E-05	0.000436894
ENSMMUG00000002653	hexokinase 3 [Source:VGNC Symbol;Acc:VGNC:73476]	-1.347229726	2.80E-14	5.52E-13
ENSMMUG00000009041	nuclear receptor binding SET domain protein 1 [Source:VGNC Symbol;Acc:VGNC:75401]	-1.344098135	2.51E-05	0.00013075
ENSMMUG00000009045	RAB24, member RAS oncogene family [Source:VGNC Symbol;Acc:VGNC:81524]	-1.240722577	0.009352842	0.025572023
ENSMMUG00000016192	lectin, mannose binding 2 [Source:VGNC Symbol;Acc:VGNC:74302]	-0.533772836	0.002759409	0.008825109
ENSMMUG00000019807	regulator of G protein signaling 14 [Source:VGNC Symbol;Acc:VGNC:76918]	0.780787861	3.54E-09	3.57E-08
ENSMMUG00000016911	DEAD-box helicase 41 [Source:VGNC Symbol;Acc:VGNC:71660]	-1.165637761	5.16E-05	0.000249845
ENSMMUG00000063462		1.132146252	0.000425949	0.001685835
ENSMMUG00000017607	NEDD4 binding protein 3 [Source:VGNC Symbol;Acc:VGNC:75053]	1.246891623	4.80E-05	0.000234131
ENSMMUG00000004000	collagen type XXIII alpha 1 chain [Source:HGNC Symbol;Acc:HGNC:22990]	0.822952316	0.000160384	0.000696891
ENSMMUG00000004002	CDC like kinase 4 [Source:VGNC Symbol;Acc:VGNC:71173]	1.720596095	3.20E-41	1.01E-38
ENSMMUG00000041519	zinc finger protein 354A [Source:VGNC Symbol;Acc:VGNC:84517]	2.143276453	1.71E-05	9.22E-05
ENSMMUG00000012370	calnexin [Source:VGNC Symbol;Acc:VGNC:70527]	0.320661653	0.003001953	0.009495039
ENSMMUG00000049557		-1.696110764	0.000353891	0.001426531
ENSMMUG00000001728	mitogen-activated protein kinase 9 [Source:VGNC Symbol;Acc:VGNC:74652]	-1.578581968	1.66E-11	2.31E-10
ENSMMUG00000047880	alpha-1,3-mannosyl-glycoprotein 2-beta-N-acetylglucosaminyltransferase [Source:VGNC Symbol;Acc:VGNC:84439]	0.761760079	6.51E-09	6.29E-08
ENSMMUG00000042832	ZFP62 zinc finger protein [Source:VGNC Symbol;Acc:VGNC:84512]	0.745385923	0.000694993	0.002600745
ENSMMUG00000054010		0.385723044	0.013886805	0.035896553
ENSMMUG00000031833	butyrophilin like 9 [Source:NCBI gene;Acc:106996463]	0.809218428	0.000832543	0.003051562
ENSMMUG00000062522		2.039821798	2.50E-18	7.42E-17
ENSMMUG00000059428	olfactory receptor 2V2 [Source:NCBI gene;Acc:707790]	1.200547622	0.00111549	0.003974705
ENSMMUG00000061151		2.112449552	9.04E-10	9.96E-09
ENSMMUG00000054414		2.134461242	8.45E-13	1.41E-11
ENSMMUG00000003598	tripartite motif containing 7 [Source:VGNC Symbol;Acc:VGNC:79022]	2.151720533	7.11E-20	2.47E-18
ENSMMUG00000062960		-2.033165829	0.002072952	0.006868158
ENSMMUG00000021145	family with sequence similarity 120B [Source:VGNC Symbol;Acc:VGNC:72311]	-1.546015518	6.57E-16	1.51E-14
ENSMMUG00000039829		-1.362458818	2.06E-05	0.000109195
ENSMMUG00000014474	SPARC related modular calcium binding 2 [Source:VGNC Symbol;Acc:VGNC:83470]	-1.661316464	8.94E-19	2.74E-17
ENSMMUG00000021044	afadin, adherens junction formation factor [Source:VGNC Symbol;Acc:VGNC:69818]	1.310280804	5.67E-21	2.25E-19
ENSMMUG00000053990		1.361940868	0.000121533	0.000543201
ENSMMUG00000062443		-1.466511538	2.89E-10	3.43E-09
ENSMMUG00000057859		-3.68261893	2.48E-13	4.37E-12

ENSMMUG00000029352	T-complex protein 10A homolog 2 [Source:NCBI gene;Acc:693619]	-2.886519058	0.000734458	0.002727778
ENSMMUG00000008228	unc-93 homolog A [Source:VGNC Symbol;Acc:VGNC:78724]	-2.766274828	1.14E-07	9.14E-07
ENSMMUG00000057983		2.648045725	0.00208224	0.006889696
ENSMMUG00000054834		0.556744628	6.92E-07	4.83E-06
ENSMMUG00000020412	parkin coregulated [Source:VGNC Symbol;Acc:VGNC:75646]	-0.634695034	0.019474623	0.047744861
ENSMMUG00000057544		-0.925404117	0.000477146	0.001865071
ENSMMUG00000000500	fibronectin type III domain containing 1 [Source:VGNC Symbol;Acc:VGNC:72546]	0.488671835	0.001543758	0.005287283
ENSMMUG00000011994	T cell activation RhoGTPase activating protein [Source:VGNC Symbol;Acc:VGNC:78126]	0.624551017	0.002189699	0.007185933
ENSMMUG00000022247	osteoclastogenesis associated transmembrane protein 1 [Source:VGNC Symbol;Acc:VGNC:75664]	2.351341226	1.60E-21	6.79E-20
ENSMMUG00000008184	methyltransferase like 24 [Source:VGNC Symbol;Acc:VGNC:74552]	1.410090855	1.77E-05	9.54E-05
ENSMMUG00000021534	solute carrier family 16 member 10 [Source:NCBI gene;Acc:696132]	-1.860708399	0.000356672	0.001436179
ENSMMUG00000058763		-1.005945665	0.01243781	0.032646501
ENSMMUG00000013782	laminin subunit alpha 4 [Source:VGNC Symbol;Acc:VGNC:74227]	-0.543425756	4.76E-05	0.0002329
ENSMMUG00000061031		1.786339234	8.23E-07	5.67E-06
ENSMMUG00000001200		1.414007973	0.018629035	0.04595286
ENSMMUG00000060903		0.819389951	5.68E-05	0.000272751
ENSMMUG00000058557		1.982839704	7.59E-14	1.44E-12
ENSMMUG00000017189	regulatory factor X6 [Source:VGNC Symbol;Acc:VGNC:76800]	2.443717348	2.00E-14	4.01E-13
ENSMMUG00000017558	discoidin, CUB and LCCL domain containing 1 [Source:VGNC Symbol;Acc:VGNC:71689]	-1.254259467	2.75E-05	0.000141788
ENSMMUG00000059327	NUS1 dehydrodolichyl diphosphate synthase subunit [Source:NCBI gene;Acc:715015]	-1.652135188	1.00E-12	1.65E-11
ENSMMUG00000053187		-1.825460537	0.017404365	0.043379284
ENSMMUG00000060554		-1.493517828	0.009360306	0.025582996
ENSMMUG00000022354	solute carrier family 35 member F1 [Source:VGNC Symbol;Acc:VGNC:77548]	-1.85911603	0.006622186	0.018933299
ENSMMUG00000008525	minichromosome maintenance 9 homologous recombination repair factor [Source:VGNC Symbol;Acc:VGNC:83432]	-3.213569929	2.71E-06	1.69E-05
ENSMMUG00000013302	family with sequence similarity 184 member A [Source:VGNC Symbol;Acc:VGNC:72308]	-5.034940905	9.69E-11	1.23E-09
ENSMMUG00000005225	gap junction protein alpha 1 [Source:VGNC Symbol;Acc:VGNC:73055]	-1.327069867	1.91E-10	2.33E-09
ENSMMUG00000024966	U4 spliceosomal RNA [Source:RFAM;Acc:RF00015]	-2.777001719	0.000202773	0.000863869
ENSMMUG00000061165		1.501321632	5.60E-18	1.60E-16
ENSMMUG00000051170		1.765032121	1.44E-10	1.78E-09
ENSMMUG00000060822		-1.358723715	7.40E-19	2.28E-17
ENSMMUG00000007910	heat shock transcription factor 2 [Source:VGNC Symbol;Acc:VGNC:81077]	-0.774783171	0.006665868	0.019044429
ENSMMUG00000052494		0.704339722	0.004041973	0.012343999
ENSMMUG00000009725	triadin [Source:HGNC Symbol;Acc:HGNC:12261]	0.922058919	9.52E-12	1.37E-10
ENSMMUG00000065276		0.810761806	5.95E-10	6.75E-09
ENSMMUG00000003134	ring finger protein 217 [Source:VGNC Symbol;Acc:VGNC:76801]	-1.258474263	2.88E-06	1.79E-05
ENSMMUG00000062110	TPD52 like 1 [Source:NCBI gene;Acc:716568]	-1.522745849	0.00042295	0.001674411

ENSMMUG00000060901		1.388852998	2.52E-16	6.07E-15
ENSMMUG00000015355	R-spondin 3 [Source:VGNC Symbol;Acc:VGNC:77066]	-1.932072584	0.000466376	0.001827309
ENSMMUG00000048882	KIAA0408 ortholog [Source:NCBI gene;Acc:100429459]	-0.720429981	3.16E-11	4.26E-10
ENSMMUG00000029421	protein tyrosine phosphatase receptor type K [Source:VGNC Symbol;Acc:VGNC:83453]	1.553951475	5.98E-05	0.000286143
ENSMMUG00000022136	laminin subunit alpha 2 [Source:VGNC Symbol;Acc:VGNC:74225]	0.636154021	7.15E-06	4.13E-05
ENSMMUG00000021498	L3MBTL histone methyl-lysine binding protein 3 [Source:VGNC Symbol;Acc:VGNC:74106]	0.839667636	4.51E-07	3.24E-06
ENSMMUG00000021499	sterile alpha motif domain containing 3 [Source:VGNC Symbol;Acc:VGNC:77079]	-0.932271309	3.75E-05	0.000186995
ENSMMUG00000059338	Y RNA [Source:RFAM;Acc:RF00019]	1.328941277	0.002160052	0.007099424
ENSMMUG00000065212		1.199923912	1.11E-06	7.50E-06
ENSMMUG00000059491		1.561149471	4.57E-15	9.84E-14
ENSMMUG00000017544	mediator complex subunit 23 [Source:VGNC Symbol;Acc:VGNC:74562]	0.567490781	7.93E-07	5.48E-06
ENSMMUG00000017549	ectonucleotide pyrophosphatase/phosphodiesterase 3 [Source:VGNC Symbol;Acc:VGNC:72224]	-0.821918129	0.000908266	0.003294103
ENSMMUG00000004996	monooxygenase DBH like 1 [Source:VGNC Symbol;Acc:VGNC:74800]	0.928459064	3.78E-08	3.22E-07
ENSMMUG00000004988	trace amine-associated receptor 3 [Source:NCBI gene;Acc:709130]	-0.613324059	0.018796228	0.04632675
ENSMMUG00000004987	trace amine associated receptor 2 [Source:VGNC Symbol;Acc:VGNC:83475]	-0.992231568	0.002766527	0.008844062
ENSMMUG00000064191	Metazoan signal recognition particle RNA [Source:RFAM;Acc:RF00017]	-1.556463911	0.00282468	0.009003558
ENSMMUG00000015089	vanin 1 [Source:VGNC Symbol;Acc:VGNC:83967]	0.840620171	9.52E-07	6.47E-06
ENSMMUG00000054573		0.851880246	4.72E-06	2.82E-05
ENSMMUG00000005745	EYA transcriptional coactivator and phosphatase 4 [Source:VGNC Symbol;Acc:VGNC:72456]	1.82039005	1.41E-31	1.52E-29
ENSMMUG00000022920	TATA-box binding protein like 1 [Source:VGNC Symbol;Acc:VGNC:83491]	0.838640869	0.001910184	0.006398922
ENSMMUG00000022921	solute carrier family 2 member 12 [Source:VGNC Symbol;Acc:VGNC:77503]	2.917212034	2.52E-28	2.14E-26
ENSMMUG00000061787		2.521922073	0.00071274	0.002657098
ENSMMUG00000008140	serum/glucocorticoid regulated kinase 1 [Source:VGNC Symbol;Acc:VGNC:77195]	0.607539826	0.004987802	0.01482644
ENSMMUG00000027839	5S ribosomal RNA [Source:RFAM;Acc:RF00001]	1.160101129	0.006909248	0.019663049
ENSMMUG00000018166	MYB proto-oncogene, transcription factor [Source:VGNC Symbol;Acc:VGNC:75089]	0.986029979	2.71E-08	2.37E-07
ENSMMUG00000009368	Abelson helper integration site 1 [Source:VGNC Symbol;Acc:VGNC:81010]	1.111075949	1.69E-16	4.13E-15
ENSMMUG00000044284	40S ribosomal protein S11-like [Source:NCBI gene;Acc:706532]	0.796612678	0.000874471	0.003185556
ENSMMUG00000053936		0.685061205	0.001176821	0.004169197
ENSMMUG00000016376	mitogen-activated protein kinase kinase kinase 5 [Source:VGNC Symbol;Acc:VGNC:74495]	1.402306137	3.16E-07	2.33E-06
ENSMMUG00000000122	heme binding protein 2 [Source:VGNC Symbol;Acc:VGNC:73172]	0.388804432	0.013616851	0.035285321
ENSMMUG00000000123	NHS like 1 [Source:VGNC Symbol;Acc:VGNC:75225]	0.741327094	4.56E-08	3.85E-07
ENSMMUG00000048283	mml-mir-3145 [Source:miRBase;Acc:MI0020897]	1.652329182	5.15E-05	0.00024982
ENSMMUG00000061250	ABRA C-terminal like [Source:NCBI gene;Acc:708266]	1.076768777	0.000358279	0.001441476
ENSMMUG00000019136	taxilin beta [Source:VGNC Symbol;Acc:VGNC:79325]	0.650198752	2.82E-11	3.82E-10
ENSMMUG00000046765		-1.136355592	0.000220153	0.000930162
ENSMMUG00000059794		-1.342211034	0.002997919	0.009486089
ENSMMUG00000058187		1.419584901	1.80E-23	9.52E-22
ENSMMUG00000023012	vesicle trafficking 1 [Source:VGNC Symbol;Acc:VGNC:79296]	-2.224354897	1.49E-08	1.36E-07
ENSMMUG00000044958	adhesion G protein-coupled receptor G6 [Source:VGNC Symbol;Acc:VGNC:81009]	-1.743369088	4.48E-09	4.43E-08
ENSMMUG00000064366		-1.462963243	0.00013065	0.000580969
ENSMMUG00000061117		-2.037024359	6.96E-15	1.48E-13

ENSMMUG00000007998	HIVEP zinc finger 2 [Source:VGNC Symbol;Acc:VGNC:83427]	-1.006506437	4.20E-09	4.17E-08
ENSMMUG00000006430	androgen induced 1 [Source:VGNC Symbol;Acc:VGNC:69667]	-1.002856752	0.008938255	0.024588077
ENSMMUG00000005366	alpha-L-fucosidase 2 [Source:VGNC Symbol;Acc:VGNC:72827]	-1.478619451	0.003274565	0.010262424
ENSMMUG00000003750	PLAG1 like zinc finger 1 [Source:VGNC Symbol;Acc:VGNC:83444]	1.47689261	0.000180431	0.000776491
ENSMMUG00000019443	syntaxin 11 [Source:VGNC Symbol;Acc:VGNC:78041]	3.913652586	5.16E-05	0.000249845
ENSMMUG00000019438	utrophin [Source:VGNC Symbol;Acc:VGNC:78749]	1.030167115	2.93E-06	1.82E-05
ENSMMUG00000051314		-1.956098758	0.003261563	0.010232491
ENSMMUG00000023265	A-kinase anchoring protein 12 [Source:VGNC Symbol;Acc:VGNC:69766]	-1.691621965	1.96E-07	1.50E-06
ENSMMUG00000059202	Y RNA [Source:RFAM;Acc:RF00019]	-2.431224846	0.00626086	0.018043063
ENSMMUG00000060839		-3.539750633	0.003448216	0.010720553
ENSMMUG00000029475	zinc finger and BTB domain containing 2 [Source:VGNC Symbol;Acc:VGNC:79291]	-2.202007906	9.52E-07	6.47E-06
ENSMMUG00000050174		-3.069590979	0.004366387	0.013212307
ENSMMUG00000018342	required for meiotic nuclear division 1 homolog [Source:VGNC Symbol;Acc:VGNC:76797]	-1.677062312	1.16E-05	6.47E-05
ENSMMUG00000060940	acidic residue methyltransferase 1 [Source:NCBI gene;Acc:704570]	-1.933201133	0.000864419	0.003152813
ENSMMUG00000018346	coiled-coil domain containing 170 [Source:VGNC Symbol;Acc:VGNC:70832]	-2.254482251	1.39E-06	9.20E-06
ENSMMUG00000025949	U6 spliceosomal RNA [Source:RFAM;Acc:RF00026]	-2.265815197	0.017659903	0.043920236
ENSMMUG00000019045	estrogen receptor 1 [Source:VGNC Symbol;Acc:VGNC:72432]	-1.65470496	4.07E-08	3.45E-07
ENSMMUG00000011171	transcription factor B1, mitochondrial [Source:VGNC Symbol;Acc:VGNC:78398]	-0.473625067	0.001980142	0.006597507
ENSMMUG00000011172	claudin 20 [Source:VGNC Symbol;Acc:VGNC:71249]	-0.504527506	9.98E-05	0.000456781
ENSMMUG00000013483	prolyl endopeptidase [Source:VGNC Symbol;Acc:VGNC:76396]	0.755598191	0.000264487	0.001096546
ENSMMUG00000055978		0.704706563	1.08E-05	6.01E-05
ENSMMUG00000057697		-1.802076681	4.18E-11	5.57E-10
ENSMMUG00000051522	Metazoan signal recognition particle RNA [Source:RFAM;Acc:RF00017]	-3.175190031	6.62E-05	0.00031345
ENSMMUG00000051271		-3.216285232	1.12E-22	5.39E-21
ENSMMUG00000000808	melanin concentrating hormone receptor 2 [Source:VGNC Symbol;Acc:VGNC:74550]	1.105173101	2.74E-19	8.79E-18
ENSMMUG00000004061	cyclin C [Source:VGNC Symbol;Acc:VGNC:70722]	0.621746075	0.010662032	0.028556823
ENSMMUG00000050740	coenzyme Q3, methyltransferase [Source:NCBI gene;Acc:694215]	-1.518172532	0.00256702	0.008274052
ENSMMUG00000005923	failed axon connections homolog, metaxin like GST domain containing [Source:VGNC Symbol;Acc:VGNC:72541]	-2.023296127	1.38E-10	1.71E-09
ENSMMUG00000037890		1.475783613	3.97E-05	0.00019622
ENSMMUG00000009182	caspase 8 associated protein 2 [Source:VGNC Symbol;Acc:VGNC:70548]	3.65045527	8.31E-05	0.000385643
ENSMMUG00000003462	ankyrin repeat domain 6 [Source:VGNC Symbol;Acc:VGNC:81023]	-0.712949972	8.05E-10	8.96E-09
ENSMMUG00000064235		0.571883723	0.003795605	0.011656415
ENSMMUG00000060841		-1.50898516	0.000107939	0.000488776

ENSMMUG00000002824	akirin 2 [Source:VGNC Symbol;Acc:VGNC:69665]	-1.204324751	0.003010288	0.009516642
ENSMMUG00000002821	origin recognition complex subunit 3 [Source:VGNC Symbol;Acc:VGNC:75715]	-1.376954016	9.36E-07	6.38E-06
ENSMMUG00000017039	arginyl-tRNA synthetase 2, mitochondrial [Source:VGNC Symbol;Acc:VGNC:76516]	0.806074285	7.52E-07	5.23E-06
ENSMMUG00000064804		-1.664697286	5.79E-09	5.64E-08
ENSMMUG00000005442	5'-nucleotidase ecto [Source:VGNC Symbol;Acc:VGNC:75537]	-1.476630894	4.89E-09	4.81E-08
ENSMMUG00000061590		-2.62763035	1.20E-06	8.01E-06
ENSMMUG00000004829	branched chain keto acid dehydrogenase E1 subunit beta [Source:VGNC Symbol;Acc:VGNC:70239]	3.173366072	0.002458035	0.007964312
ENSMMUG00000017186	pleckstrin homology domain interacting protein [Source:VGNC Symbol;Acc:VGNC:75803]	-2.598459761	7.99E-14	1.51E-12
ENSMMUG00000004491	SUMO specific peptidase 6 [Source:VGNC Symbol;Acc:VGNC:77287]	-0.614241921	8.03E-10	8.94E-09
ENSMMUG00000046092	adhesion G protein-coupled receptor B3 [Source:VGNC Symbol;Acc:VGNC:81006]	0.467343065	0.001891694	0.00634025
ENSMMUG00000041338	eyes shut homolog [Source:HGNC Symbol;Acc:HGNC:21555]	-0.673900722	1.61E-10	1.98E-09
ENSMMUG00000017160	PHD finger protein 3 [Source:VGNC Symbol;Acc:VGNC:75975]	-0.973503536	3.22E-07	2.37E-06
ENSMMUG00000062339	endogenous retrovirus group K member 25 Env polyprotein-like [Source:NCBI gene;Acc:114677579]	-0.999649669	9.08E-06	5.15E-05
ENSMMUG00000002777	protein tyrosine phosphatase 4A1 [Source:VGNC Symbol;Acc:VGNC:83452]	-0.979421472	3.64E-09	3.65E-08
ENSMMUG00000000777	dystonin [Source:VGNC Symbol;Acc:VGNC:81061]	-1.671975478	0.011346869	0.03019452
ENSMMUG00000011858	bone morphogenetic protein 5 [Source:VGNC Symbol;Acc:VGNC:70264]	0.9977071	5.37E-06	3.18E-05
ENSMMUG00000001395	hypocretin receptor 2 [Source:VGNC Symbol;Acc:VGNC:73357]	-2.628359727	0.004218837	0.012823397
ENSMMUG00000036060	U6 spliceosomal RNA [Source:RFAM;Acc:RF00026]	2.59687451	0.004606256	0.013853695
ENSMMUG00000012784	tubulointerstitial nephritis antigen [Source:VGNC Symbol;Acc:VGNC:78382]	-1.669115046	0.001873679	0.006289421
ENSMMUG00000012781	muscular LMNA interacting protein [Source:VGNC Symbol;Acc:VGNC:83434]	-1.784921474	2.20E-09	2.29E-08
ENSMMUG00000011049	leucine rich repeat containing 1 [Source:VGNC Symbol;Acc:VGNC:81087]	-1.322434426	0.003902812	0.011960881
ENSMMUG00000013718	translocation associated membrane protein 2 [Source:VGNC Symbol;Acc:VGNC:83515]	-1.454472528	4.10E-18	1.19E-16
ENSMMUG00000052626	progestin and adipoQ receptor family member 8 [Source:NCBI gene;Acc:708418]	0.637338236	1.49E-05	8.11E-05
ENSMMUG00000007403	interleukin 17A [Source:VGNC Symbol;Acc:VGNC:81081]	-1.561378068	0.004846545	0.01447327
ENSMMUG00000026975	mml-mir-206 [Source:miRBase;Acc:MI0007667]	4.46903577	0.000314948	0.001283857
ENSMMUG00000016993	PKHD1 ciliary IPT domain containing fibrocystin/polyductin [Source:VGNC Symbol;Acc:VGNC:76010]	-2.31820807	6.71E-14	1.28E-12
ENSMMUG00000038096	Metazoan signal recognition particle RNA [Source:RFAM;Acc:RF00017]	-2.843861783	0.014361726	0.036842138
ENSMMUG00000053984		2.872722588	6.28E-08	5.22E-07
ENSMMUG00000056168		3.867570014	0.000326683	0.001326956
ENSMMUG00000022983	adhesion G protein-coupled receptor F1 [Source:VGNC Symbol;Acc:VGNC:81007]	-1.113486882	0.000747301	0.002767159
ENSMMUG00000023002	adhesion G protein-coupled receptor F5 [Source:VGNC Symbol;Acc:VGNC:69471]	-0.787071284	6.02E-09	5.85E-08
ENSMMUG00000023000	meprin A subunit alpha [Source:VGNC Symbol;Acc:VGNC:74704]	-0.981165057	1.25E-05	6.90E-05
ENSMMUG00000042154	ankyrin repeat domain 66 [Source:VGNC Symbol;Acc:VGNC:81024]	-0.924778921	1.40E-07	1.11E-06

ENSMMUG00000019254	phospholipase A2 group VII [Source:VGNC Symbol;Acc:VGNC:76158]	-1.3877263	2.47E-11	3.36E-10
ENSMMUG00000019252	tudor domain containing 6 [Source:VGNC Symbol;Acc:VGNC:79319]	-1.476927784	1.78E-15	3.95E-14
ENSMMUG00000019250	solute carrier family 25 member 27 [Source:VGNC Symbol;Acc:VGNC:77543]	-1.322801299	1.65E-13	2.99E-12
ENSMMUG00000055199	cytochrome P450, family 39, subfamily A, polypeptide 1 [Source:NCBI gene;Acc:704242]	1.278169758	0.002986943	0.009463013
ENSMMUG00000019246	regulator of calcineurin 2 [Source:VGNC Symbol;Acc:VGNC:82092]	2.060991552	1.03E-11	1.47E-10
ENSMMUG00000042260	ectonucleotide pyrophosphatase/phosphodiesterase 4 [Source:VGNC Symbol;Acc:VGNC:81066]	-1.935044991	0.012435808	0.032646501
ENSMMUG00000003511	chloride intracellular channel 5 [Source:VGNC Symbol;Acc:VGNC:71171]	-1.300067179	2.49E-08	2.20E-07
ENSMMUG00000052523		-2.904197849	0.000139592	0.000614836
ENSMMUG00000021377	SPT3 homolog, SAGA and STAGA complex component [Source:VGNC Symbol;Acc:VGNC:78055]	-2.088430272	4.73E-19	1.48E-17
ENSMMUG00000014389	cell division cycle 5 like [Source:VGNC Symbol;Acc:VGNC:70801]	-0.766811094	0.000441411	0.001737757
ENSMMUG00000014388	spermatogenesis associated serine rich 1 [Source:VGNC Symbol;Acc:VGNC:77975]	-0.796103411	2.59E-06	1.63E-05
ENSMMUG00000054860		-1.45849831	0.013450311	0.034914775
ENSMMUG00000060503	Metazoan signal recognition particle RNA [Source:RFAM;Acc:RF00017]	-1.20126193	3.92E-05	0.000194196
ENSMMUG00000063288		-1.043353409	6.45E-08	5.34E-07
ENSMMUG00000011670	protein tyrosine kinase 7 (inactive) [Source:VGNC Symbol;Acc:VGNC:83451]	-2.39293402	0.004188577	0.012739252
ENSMMUG00000013920	triggering receptor expressed on myeloid cells 2 [Source:VGNC Symbol;Acc:VGNC:79089]	-0.941396589	0.000550045	0.002118196
ENSMMUG00000062538	adenylate cyclase type 10 [Source:NCBI gene;Acc:719662]	-1.735198629	2.48E-09	2.55E-08
ENSMMUG00000021173	nuclear transcription factor Y subunit alpha [Source:VGNC Symbol;Acc:VGNC:75344]	-1.500355109	1.49E-24	8.58E-23
ENSMMUG00000021171	apolipoprotein B mRNA editing enzyme catalytic subunit 2 [Source:VGNC Symbol;Acc:VGNC:69986]	-1.144152914	4.13E-10	4.79E-09
ENSMMUG00000021169	translocator protein 2 [Source:VGNC Symbol;Acc:VGNC:83960]	-1.073533341	3.51E-05	0.000176509
ENSMMUG00000021168	unc-5 family C-terminal like [Source:VGNC Symbol;Acc:VGNC:79314]	-1.491134019	8.61E-18	2.43E-16
ENSMMUG00000013651	kinesin family member 6 [Source:VGNC Symbol;Acc:VGNC:73873]	3.872443296	0.000501729	0.001951387
ENSMMUG00000013648	potassium two pore domain channel subfamily K member 16 [Source:VGNC Symbol;Acc:VGNC:73827]	3.747356749	5.89E-06	3.45E-05
ENSMMUG00000013644	potassium two pore domain channel subfamily K member 17 [Source:VGNC Symbol;Acc:VGNC:73828]	3.109448361	3.45E-26	2.33E-24
ENSMMUG00000023381	dynein axonemal heavy chain 8 [Source:VGNC Symbol;Acc:VGNC:71943]	0.671443918	1.10E-09	1.20E-08
ENSMMUG00000048502	Metazoan signal recognition particle RNA [Source:RFAM;Acc:RF00017]	1.073314452	0.00010439	0.000474734
ENSMMUG00000062055	glyoxalase I [Source:NCBI gene;Acc:719518]	1.201366259	2.44E-23	1.26E-21
ENSMMUG00000001634	BTB domain containing 9 [Source:VGNC Symbol;Acc:VGNC:70360]	2.743208285	0.01368364	0.035427442
ENSMMUG00000011575	coiled-coil domain containing 167 [Source:VGNC Symbol;Acc:VGNC:70833]	-3.399247291	8.99E-09	8.47E-08
ENSMMUG00000011573	cap methyltransferase 1 [Source:VGNC Symbol;Acc:VGNC:71271]	-2.225343142	2.04E-08	1.83E-07
ENSMMUG00000022008	TBC1 domain family member 22B [Source:VGNC Symbol;Acc:VGNC:78192]	-1.447835237	0.002459076	0.007965945
ENSMMUG00000063198		4.31470259	2.90E-12	4.51E-11
ENSMMUG00000059553		0.66191895	4.49E-05	0.000219954
ENSMMUG00000020558	serine/threonine kinase 38 [Source:VGNC Symbol;Acc:VGNC:78155]	1.219613431	0.003041681	0.009603604
ENSMMUG00000014508	potassium channel tetramerization domain containing 20 [Source:VGNC Symbol;Acc:VGNC:73872]	1.285587646	3.07E-05	0.000156437
ENSMMUG00000055914		2.060227129	7.34E-12	1.07E-10
ENSMMUG00000015416	bromodomain and PHD finger containing 3 [Source:VGNC Symbol;Acc:VGNC:70309]	2.274373403	2.83E-15	6.14E-14

ENSMMUG00000000869	small nuclear ribonucleoprotein polypeptide C [Source:NCBI gene;Acc:718654]	4.241174956	1.56E-18	4.68E-17
ENSMMUG00000007811	protein kinase C and casein kinase substrate in neurons 1 [Source:VGNC Symbol;Acc:VGNC:75744]	0.573974245	0.000281579	0.001157699
ENSMMUG00000004287	death domain associated protein [Source:VGNC Symbol;Acc:VGNC:71737]	-1.788229366	1.81E-05	9.70E-05
ENSMMUG00000003875	TAP binding protein [Source:VGNC Symbol;Acc:VGNC:78098]	-1.141188993	0.000517376	0.002003311
ENSMMUG00000003872	ral guanine nucleotide dissociation stimulator like 2 [Source:VGNC Symbol;Acc:VGNC:76886]	-1.17330447	7.08E-05	0.000332982
ENSMMUG00000003871	prefoldin subunit 6 [Source:VGNC Symbol;Acc:VGNC:75906]	-1.12553549	0.014116745	0.036345718
ENSMMUG00000003869	WD repeat domain 46 [Source:VGNC Symbol;Acc:VGNC:78940]	-1.221196718	4.31E-05	0.000212089
ENSMMUG00000064507	beta-1,3-galactosyltransferase 4 [Source:NCBI gene;Acc:718178]	-1.053834992	0.000702127	0.002623469
ENSMMUG00000003864	VPS52 subunit of GARP complex [Source:VGNC Symbol;Acc:VGNC:78551]	-1.457183111	1.57E-08	1.43E-07
ENSMMUG00000003867	ribosomal protein S18 [Source:NCBI gene;Acc:706414]	-1.508551607	0.000799906	0.002945037
ENSMMUG00000003863	ring finger protein 1 [Source:VGNC Symbol;Acc:VGNC:83457]	-1.008854456	0.002110178	0.006972804
ENSMMUG00000003859	solute carrier family 39 member 7 [Source:VGNC Symbol;Acc:VGNC:77592]	-1.532894332	1.76E-05	9.47E-05
ENSMMUG00000003858	retinoid X receptor beta [Source:VGNC Symbol;Acc:VGNC:76952]	-1.467297861	1.46E-06	9.64E-06
ENSMMUG00000003850	collagen type XI alpha 2 chain [Source:VGNC Symbol;Acc:VGNC:71290]	-1.307880914	3.77E-07	2.74E-06
ENSMMUG00000019371	major histocompatibility complex, class II, DP alpha [Source:NCBI gene;Acc:717992]	-1.336999449	0.006494682	0.018617237
ENSMMUG00000023138	bromodomain containing 2 [Source:VGNC Symbol;Acc:VGNC:83422]	-2.147424228	0.000791844	0.002920428
ENSMMUG00000022626	major histocompatibility complex, class II, DM alpha [Source:NCBI gene;Acc:717870]	-1.989956655	0.000101811	0.000464595
ENSMMUG00000018461	notch receptor 4 [Source:VGNC Symbol;Acc:VGNC:75372]	-1.870317079	0.00055505	0.002133036
ENSMMUG00000018459	G protein signaling modulator 3 [Source:VGNC Symbol;Acc:VGNC:72938]	-1.522253555	0.002912185	0.009263772
ENSMMUG00000018456	PBX homeobox 2 [Source:VGNC Symbol;Acc:VGNC:75771]	-1.402933307	0.000421748	0.001670098
ENSMMUG00000018434	1-acylglycerol-3-phosphate O-acyltransferase 1 [Source:VGNC Symbol;Acc:VGNC:69745]	-0.90351721	0.016933834	0.042434733
ENSMMUG00000006828	valyl-tRNA synthetase 1 [Source:VGNC Symbol;Acc:VGNC:78755]	-2.054348981	0.003768847	0.011593447
ENSMMUG00000006827	von Willebrand factor A domain containing 7 [Source:VGNC Symbol;Acc:VGNC:78941]	-2.422930877	0.000171657	0.000740667
ENSMMUG00000009702	mutS homolog 5 [Source:NCBI gene;Acc:715494]	-2.348151785	0.000378106	0.001515901
ENSMMUG00000058534		3.796995815	9.48E-14	1.77E-12
ENSMMUG00000015693	lymphocyte antigen 6 family member G6C [Source:VGNC Symbol;Acc:VGNC:74285]	3.592750451	4.99E-17	1.30E-15
ENSMMUG00000053324	lymphocyte antigen 6 complex locus protein G6d [Source:NCBI gene;Acc:715304]	2.954192505	3.39E-22	1.54E-20
ENSMMUG00000039268	lymphocyte antigen 6G6e-like [Source:NCBI gene;Acc:100427419]	3.733766808	1.36E-05	7.50E-05
ENSMMUG00000015686	abhydrolase domain containing 16A [Source:VGNC Symbol;Acc:VGNC:69393]	2.966156084	5.88E-34	8.90E-32
ENSMMUG00000054159	lymphocyte antigen 6 family member G5C [Source:VGNC Symbol;Acc:VGNC:83431]	3.302939398	7.37E-07	5.12E-06
ENSMMUG00000005294	casein kinase 2 beta [Source:NCBI gene;Acc:715807]	3.055203882	7.57E-13	1.27E-11
ENSMMUG00000005293	G-patch domain and ankyrin repeats 1 [Source:VGNC Symbol;Acc:VGNC:81073]	3.401774391	2.16E-17	5.86E-16
ENSMMUG00000005302	BAG cochaperone 6 [Source:VGNC Symbol;Acc:VGNC:81044]	3.023213244	5.85E-44	2.71E-41
ENSMMUG00000005303	apolipoprotein M [Source:VGNC Symbol;Acc:VGNC:69825]	3.391822357	1.33E-14	2.74E-13
ENSMMUG00000005297	proline rich coiled-coil 2A [Source:VGNC Symbol;Acc:VGNC:83448]	2.76901654	3.70E-33	5.13E-31
ENSMMUG00000025918	small nucleolar RNA, H/ACA box 38 [Source:HGNC Symbol;Acc:HGNC:32631]	3.341157544	3.95E-09	3.94E-08

ENSMMUG00000049735	allograft inflammatory factor 1 [Source:NCBI gene;Acc:574115]	2.747946979	8.59E-34	1.26E-31
ENSMMUG00000008854	natural cytotoxicity triggering receptor 3 [Source:VGNC Symbol;Acc:VGNC:74990]	2.971614028	2.38E-32	2.90E-30
ENSMMUG00000008853	leukocyte specific transcript 1 [Source:HGNC Symbol;Acc:HGNC:14189]	2.714324451	1.33E-09	1.44E-08
ENSMMUG00000008849	lymphotoxin beta [Source:VGNC Symbol;Acc:VGNC:74454]	2.316556832	3.72E-05	0.000185841
ENSMMUG00000045654	tumor necrosis factor [Source:VGNC Symbol;Acc:VGNC:83501]	3.009296061	1.97E-10	2.40E-09
ENSMMUG00000008843	lymphotoxin alpha [Source:VGNC Symbol;Acc:VGNC:74452]	2.456878485	1.46E-08	1.33E-07
ENSMMUG00000062024	NFKB inhibitor like 1 [Source:NCBI gene;Acc:715283]	2.27933411	0.00051615	0.002000653
ENSMMUG00000052422	ATPase H+ transporting V1 subunit G2 [Source:NCBI gene;Acc:715331]	4.873701961	6.12E-06	3.58E-05
ENSMMUG00000008839	DExD-box helicase 39B [Source:VGNC Symbol;Acc:VGNC:71621]	2.623999861	3.67E-13	6.38E-12
ENSMMUG00000026474	Small nucleolar RNA SNORD83 [Source:RFAM;Acc:RF00137]	4.325567813	1.35E-05	7.44E-05
ENSMMUG00000008838	mitochondrial coiled-coil domain 1 [Source:VGNC Symbol;Acc:VGNC:74548]	2.332413179	0.002091443	0.00691706
ENSMMUG00000006609	MHC class I polypeptide-related sequence B [Source:NCBI gene;Acc:715141]	2.104588616	0.000452166	0.001775855
ENSMMUG00000019961	protein phosphatase 1 regulatory subunit 18 [Source:NCBI gene;Acc:712176]	2.771783532	0.005151815	0.015225471
ENSMMUG00000053627		1.693560227	0.018191838	0.045063964
ENSMMUG00000011682	ribonuclease P/MRP subunit p21 [Source:NCBI gene;Acc:713056]	3.003612729	6.13E-05	0.000292442
ENSMMUG00000060527		-2.615353236	1.42E-12	2.29E-11
ENSMMUG00000023679	tripartite motif containing 26 [Source:VGNC Symbol;Acc:VGNC:78854]	-2.270484267	1.36E-12	2.21E-11
ENSMMUG00000002890	tripartite motif containing 15 [Source:VGNC Symbol;Acc:VGNC:83958]	-2.546349844	1.19E-17	3.32E-16
ENSMMUG00000012129	tripartite motif containing 10 [Source:VGNC Symbol;Acc:VGNC:78550]	-2.488950773	3.35E-22	1.52E-20
ENSMMUG00000058580	tripartite motif containing 40 [Source:NCBI gene;Acc:100427925]	-2.466437328	2.05E-09	2.14E-08
ENSMMUG00000062264		-2.123887742	1.61E-05	8.76E-05
ENSMMUG00000012124	ring finger protein 39 [Source:VGNC Symbol;Acc:VGNC:83458]	-2.676927631	3.91E-09	3.91E-08
ENSMMUG00000012122	protein phosphatase 1 regulatory inhibitor subunit 11 [Source:VGNC Symbol;Acc:VGNC:82209]	-2.531372294	6.31E-08	5.25E-07
ENSMMUG00000012120	zinc ribbon domain containing 1 [Source:VGNC Symbol;Acc:VGNC:78912]	-2.774937394	5.85E-16	1.36E-14
ENSMMUG00000055272	putative uncharacterized protein ZNRD1-AS1 [Source:NCBI gene;Acc:712069]	-2.881892079	8.85E-17	2.23E-15
ENSMMUG00000056544		-3.572994557	1.15E-08	1.07E-07
ENSMMUG00000037625		-2.489280206	5.30E-09	5.18E-08
ENSMMUG00000050761		-1.763812479	0.000841138	0.0030785
ENSMMUG00000053561		3.666036004	0.000116615	0.000522792
ENSMMUG00000029883	olfactory receptor, family 2, subfamily J, member 3 [Source:NCBI gene;Acc:708943]	3.806807367	1.40E-05	7.67E-05
ENSMMUG00000059442		2.869383458	1.80E-07	1.39E-06
ENSMMUG00000005415	olfactory receptor, family 2, subfamily B, member 3 [Source:NCBI gene;Acc:708747]	2.792543678	5.82E-06	3.42E-05
ENSMMUG00000004377	olfactory receptor, family 2, subfamily W, member 1 [Source:NCBI gene;Acc:708515]	3.483478112	0.009913622	0.026847885
ENSMMUG00000004619	zinc finger BED-type containing 9 [Source:VGNC Symbol;Acc:VGNC:81608]	0.807778733	1.21E-05	6.73E-05
ENSMMUG00000004617	glutathione peroxidase 6 [Source:VGNC Symbol;Acc:VGNC:73154]	-1.6267169	5.55E-06	3.27E-05
ENSMMUG00000060483		0.59195759	8.11E-05	0.000377363
ENSMMUG00000012396	zinc finger and SCAN domain containing 23 [Source:VGNC Symbol;Acc:VGNC:79313]	-1.830571473	1.09E-12	1.78E-11
ENSMMUG00000011675	zinc finger and SCAN domain containing 12 [Source:VGNC Symbol;Acc:VGNC:78910]	-1.364177219	2.31E-13	4.09E-12

ENSMMUG00000017495	geminin DNA replication inhibitor [Source:VGNC Symbol;Acc:VGNC:72954]	-0.846390064	0.018805532	0.046341981
ENSMMUG00000058151	chromosome 4 C6orf62 homolog [Source:NCBI gene;Acc:708218]	-1.934919637	5.04E-26	3.31E-24
ENSMMUG00000029939	acyl-CoA thioesterase 13 [Source:VGNC Symbol;Acc:VGNC:69487]	-2.15099011	0.00807782	0.022530386
ENSMMUG00000051037		5.005467366	1.20E-20	4.61E-19
ENSMMUG00000063635		-3.065641365	0.012333536	0.032441707
ENSMMUG00000054532		-1.508535702	4.75E-09	4.68E-08
ENSMMUG00000052442		-1.603747251	1.24E-10	1.54E-09
ENSMMUG00000059595	Metazoan signal recognition particle RNA [Source:RFAM;Acc:RF00017]	-2.776189244	0.004244399	0.012895827
ENSMMUG00000014691	inhibitor of DNA binding 4, HLH protein [Source:VGNC Symbol;Acc:VGNC:73557]	-0.676591853	3.31E-05	0.000167698
ENSMMUG00000047127		-0.858390878	0.00288142	0.009175748
ENSMMUG00000063576	Metazoan signal recognition particle RNA [Source:RFAM;Acc:RF00017]	-1.878296966	3.11E-05	0.000158404
ENSMMUG00000062089	ring finger protein 144B [Source:NCBI gene;Acc:705099]	-0.927417108	0.001979835	0.006597507
ENSMMUG00000052891		2.413768367	3.93E-19	1.24E-17
ENSMMUG00000020289	CD83 molecule [Source:VGNC Symbol;Acc:VGNC:70783]	-2.213382144	3.88E-17	1.02E-15
ENSMMUG00000044473		-1.796153993	3.40E-12	5.22E-11
ENSMMUG00000009922	mitochondrial calcium uniporter regulator 1 [Source:VGNC Symbol;Acc:VGNC:81471]	-1.157644786	1.31E-09	1.41E-08
ENSMMUG00000020122	RAN binding protein 9 [Source:VGNC Symbol;Acc:VGNC:76475]	-1.233056912	1.02E-08	9.54E-08
ENSMMUG00000008681	nucleolar protein 7 [Source:VGNC Symbol;Acc:VGNC:75226]	-1.016685437	0.00764643	0.021465446
ENSMMUG00000061899	glucose-fructose oxidoreductase domain containing 1 [Source:NCBI gene;Acc:700607]	-0.453085628	0.007036035	0.019958761
ENSMMUG00000005406	TBC1 domain family member 7 [Source:NCBI gene;Acc:706015]	-0.775793968	0.01534045	0.038961422
ENSMMUG00000004347	androgen dependent TFPI regulating protein [Source:VGNC Symbol;Acc:VGNC:69661]	2.065097659	0.002737382	0.008769794
ENSMMUG00000058800		1.663811744	2.00E-13	3.59E-12
ENSMMUG00000016135	neural precursor cell expressed, developmentally down-regulated 9 [Source:VGNC Symbol;Acc:VGNC:75304]	-1.502571261	8.28E-09	7.85E-08
ENSMMUG00000046520	small integral membrane protein 13 [Source:NCBI gene;Acc:703387]	-1.13490541	2.86E-05	0.000146904
ENSMMUG00000043161	endogenous retrovirus group FRD member 1, envelope [Source:VGNC Symbol;Acc:VGNC:81068]	-1.554000596	3.47E-08	2.97E-07
ENSMMUG00000005532	male germ cell associated kinase [Source:VGNC Symbol;Acc:VGNC:74480]	1.183103826	7.76E-10	8.68E-09
ENSMMUG00000018788	transmembrane protein 14B [Source:NCBI gene;Acc:698237]	1.064285888	1.04E-06	7.04E-06
ENSMMUG00000018787	PAK1 interacting protein 1 [Source:VGNC Symbol;Acc:VGNC:75661]	1.025039878	1.91E-06	1.23E-05
ENSMMUG00000061586	chromosome 4 C6orf52 homolog [Source:NCBI gene;Acc:702114]	0.756345138	0.000116487	0.000522378
ENSMMUG00000006186	transcription factor AP-2 alpha [Source:VGNC Symbol;Acc:VGNC:78320]	-1.143146429	0.005050207	0.014978915
ENSMMUG00000062812	Metazoan signal recognition particle RNA [Source:RFAM;Acc:RF00017]	1.042288661	0.00296375	0.009401585
ENSMMUG00000038560	eukaryotic translation elongation factor 1 epsilon 1 [Source:NCBI gene;Acc:695426]	-1.515151004	1.01E-15	2.28E-14
ENSMMUG00000044859		-1.48200121	0.000487797	0.001902692

ENSMMUG00000021600	bone morphogenetic protein 6 [Source:VGNC Symbol;Acc:VGNC:70265]	-0.811433152	0.007339451	0.020677062
ENSMMUG00000015577	RIO kinase 1 [Source:VGNC Symbol;Acc:VGNC:76767]	-0.830458553	0.016918133	0.042402553
ENSMMUG00000007852	ras responsive element binding protein 1 [Source:VGNC Symbol;Acc:VGNC:76939]	-2.525669456	0.005475401	0.0160443
ENSMMUG00000001248	coagulation factor XIII A chain [Source:VGNC Symbol;Acc:VGNC:72462]	-1.962602388	0.000661822	0.002492334
ENSMMUG00000060973		0.674259494	0.001413551	0.00489789
ENSMMUG00000055919		-1.538570351	0.018415001	0.045523331
ENSMMUG00000023558	exocyst complex component 2 [Source:VGNC Symbol;Acc:VGNC:72338]	-1.621409242	3.89E-05	0.000192813
ENSMMUG00000058002	zinc finger protein 669-like [Source:NCBI gene;Acc:696282]	1.426449158	4.88E-22	2.19E-20
ENSMMUG00000003013	NIPA magnesium transporter 1 [Source:VGNC Symbol;Acc:VGNC:75196]	-4.231513972	0.005447636	0.015975567
ENSMMUG00000052887		-2.643460259	0.00095836	0.003463922
ENSMMUG00000051793	gamma-aminobutyric acid type A receptor beta3 subunit [Source:NCBI gene;Acc:711754]	-0.814752115	1.62E-05	8.80E-05
ENSMMUG00000062676		-1.309456107	7.11E-10	8.01E-09
ENSMMUG00000054713		-1.801730872	5.19E-07	3.70E-06
ENSMMUG00000065031		-1.827788104	2.25E-23	1.18E-21
ENSMMUG00000046479		-0.564430924	0.006611931	0.01891127
ENSMMUG00000058972		-2.603225203	0.007150849	0.020230274
ENSMMUG00000019483	putative golgin subfamily A member 8D [Source:NCBI gene;Acc:100430266]	-3.175738529	0.000577915	0.002210024
ENSMMUG00000021656	golgin subfamily A member 8S [Source:NCBI gene;Acc:722977]	-2.839670045	0.001533217	0.005256035
ENSMMUG00000054315		-3.47430429	1.85E-09	1.95E-08
ENSMMUG00000055117		-3.71583141	1.14E-10	1.42E-09
ENSMMUG00000063268		-3.512294662	3.45E-13	6.00E-12
ENSMMUG00000012838	OTU deubiquitinase 7A [Source:VGNC Symbol;Acc:VGNC:75660]	-1.387032292	0.013129216	0.034158996
ENSMMUG00000009430	transient receptor potential cation channel subfamily M member 1 [Source:VGNC Symbol;Acc:VGNC:79964]	0.822994418	4.95E-07	3.54E-06
ENSMMUG00000009426	FANCD2 and FANCI associated nuclease 1 [Source:VGNC Symbol;Acc:VGNC:72595]	-2.392374456	0.013192561	0.034311764
ENSMMUG00000019339	amyloid beta precursor protein binding family A member 2 [Source:VGNC Symbol;Acc:VGNC:69977]	-1.20483536	0.001862903	0.006257497
ENSMMUG00000005270	ryanodine receptor 3 [Source:NCBI gene;Acc:694996]	1.078294571	1.96E-20	7.25E-19
ENSMMUG00000064964		0.408001708	0.004500252	0.01356754
ENSMMUG00000002337	aquarius intron-binding spliceosomal factor [Source:VGNC Symbol;Acc:VGNC:69925]	-0.721277799	0.00588254	0.017082058
ENSMMUG00000046486	zinc finger protein 770 [Source:NCBI gene;Acc:697740]	-0.868928534	0.000141022	0.000620397
ENSMMUG00000064307		-0.747054224	0.014322471	0.036760514
ENSMMUG00000061601		-0.710684494	0.01918242	0.047106356
ENSMMUG00000063659		-1.722687176	4.72E-06	2.82E-05

ENSMMUG00000051611		-1.639053122	0.005805072	0.016890174
ENSMMUG00000049006	Metazoan signal recognition particle RNA [Source:RFAM;Acc:RF00017]	-2.205563831	0.000610858	0.002319851
ENSMMUG00000060667		1.420654182	0.000818668	0.003006649
ENSMMUG00000018332	family with sequence similarity 98 member B [Source:VGNC Symbol;Acc:VGNC:72583]	-1.838748666	3.31E-10	3.89E-09
ENSMMUG00000018333	RAS guanyl releasing protein 1 [Source:VGNC Symbol;Acc:VGNC:76671]	-1.712758174	0.016309317	0.041107583
ENSMMUG00000055491		-1.657672016	0.001388746	0.004820348
ENSMMUG00000016686	BUB1 mitotic checkpoint serine/threonine kinase B [Source:VGNC Symbol;Acc:VGNC:70293]	1.026691913	1.53E-08	1.39E-07
ENSMMUG00000020534	p21 (RAC1) activated kinase 6 [Source:NCBI gene;Acc:701589]	1.809390415	9.55E-16	2.17E-14
ENSMMUG00000020539	coiled-coil domain containing 9B [Source:VGNC Symbol;Acc:VGNC:70920]	-0.754809292	0.003291954	0.010297339
ENSMMUG00000012408	dispatched RND transporter family member 2 [Source:VGNC Symbol;Acc:VGNC:71676]	-1.029755796	6.12E-06	3.58E-05
ENSMMUG00000058981	kinetochore localized astrin (SPAG5) binding protein [Source:NCBI gene;Acc:702753]	-0.862072436	1.41E-05	7.74E-05
ENSMMUG00000001482	coiled-coil domain containing 32 [Source:VGNC Symbol;Acc:VGNC:70891]	1.598986977	0.005612611	0.016397758
ENSMMUG00000001483	RNA pseudouridine synthase domain containing 2 [Source:VGNC Symbol;Acc:VGNC:77091]	1.459511856	0.002002903	0.006667353
ENSMMUG00000048910	kinetochore scaffold 1 [Source:VGNC Symbol;Acc:VGNC:84366]	-0.7001206	0.000839408	0.003072957
ENSMMUG00000004579	RAD51 recombinase [Source:VGNC Symbol;Acc:VGNC:76645]	-0.849134502	0.001363349	0.004746118
ENSMMUG00000000306	regulator of microtubule dynamics 3 [Source:VGNC Symbol;Acc:VGNC:76821]	-0.77453383	0.009424289	0.025734158
ENSMMUG00000062039	chromosome 7 C15orf62 homolog [Source:NCBI gene;Acc:704353]	1.895100072	0.006707139	0.019146654
ENSMMUG00000013160	DnaJ heat shock protein family (Hsp40) member C17 [Source:VGNC Symbol;Acc:VGNC:84335]	1.825414314	4.35E-06	2.62E-05
ENSMMUG00000044445	NADH:ubiquinone oxidoreductase complex assembly factor 1 [Source:VGNC Symbol;Acc:VGNC:84406]	0.451531947	0.002803534	0.008946934
ENSMMUG00000015482	RTF1 homolog, Paf1/RNA polymerase II complex component [Source:VGNC Symbol;Acc:VGNC:77119]	-2.913705077	0.002931861	0.009316371
ENSMMUG00000010168	MAX dimerization protein MGA [Source:VGNC Symbol;Acc:VGNC:84390]	0.424363229	0.003783145	0.011625371
ENSMMUG00000003839	mitogen-activated protein kinase binding protein 1 [Source:VGNC Symbol;Acc:VGNC:74613]	1.050114789	1.98E-07	1.52E-06
ENSMMUG00000003844	spectrin beta, non-erythrocytic 5 [Source:VGNC Symbol;Acc:VGNC:77927]	0.474766995	0.00678163	0.019340688
ENSMMUG00000015449	phospholipase A2 group IVF [Source:VGNC Symbol;Acc:VGNC:76123]	0.813787007	4.31E-12	6.50E-11
ENSMMUG00000014511	VPS39 subunit of HOPS complex [Source:VGNC Symbol;Acc:VGNC:79257]	0.490171476	0.000566538	0.002170996
ENSMMUG00000001532	glucosidase alpha, neutral C [Source:NCBI gene;Acc:708126]	1.253849114	4.47E-32	5.22E-30
ENSMMUG00000001534	calpain 3 [Source:VGNC Symbol;Acc:VGNC:70533]	1.478468494	8.74E-13	1.45E-11
ENSMMUG00000063886	Metazoan signal recognition particle RNA [Source:RFAM;Acc:RF00017]	2.210952056	0.00337502	0.010524036
ENSMMUG00000001547	zinc finger protein 106 [Source:VGNC Symbol;Acc:VGNC:78831]	1.224789385	4.30E-19	1.35E-17
ENSMMUG00000064341	U6 spliceosomal RNA [Source:RFAM;Acc:RF00026]	3.578410576	4.37E-05	0.000214456
ENSMMUG00000008093	StAR related lipid transfer domain containing 9 [Source:NCBI gene;Acc:708953]	-1.129611242	4.99E-11	6.57E-10
ENSMMUG00000015123	codanin 1 [Source:VGNC Symbol;Acc:VGNC:70788]	-0.802771056	1.36E-05	7.49E-05
ENSMMUG00000010036	tau tubulin kinase 2 [Source:VGNC Symbol;Acc:VGNC:81598]	-0.510753016	0.000521466	0.002017834
ENSMMUG00000006498	ubiquitin protein ligase E3 component n-recognin 1 [Source:VGNC Symbol;Acc:VGNC:79051]	0.433292828	0.012971691	0.033831437
ENSMMUG00000022251	transglutaminase 7 [Source:VGNC Symbol;Acc:VGNC:79880]	-0.64668284	5.10E-05	0.000247778
ENSMMUG00000017067	adenosine deaminase like [Source:VGNC Symbol;Acc:VGNC:69555]	-0.60973153	3.60E-05	0.000180595
ENSMMUG00000017070	zinc finger and SCAN domain containing 29 [Source:VGNC Symbol;Acc:VGNC:79633]	-0.665715026	1.67E-05	9.04E-05
ENSMMUG00000017076	tubulin gamma complex associated protein 4 [Source:VGNC Symbol;Acc:VGNC:79045]	-0.941426174	2.94E-07	2.18E-06
ENSMMUG00000007255	microtubule associated protein 1A [Source:VGNC Symbol;Acc:VGNC:74489]	-1.69507711	1.14E-05	6.36E-05

ENSMMUG00000007860	diphosphoinositol pentakisphosphate kinase 1 [Source:VGNC Symbol;Acc:VGNC:76127]	-0.85774307	0.001637366	0.005565468
ENSMMUG00000007870	creatine kinase U-type, mitochondrial [Source:NCBI gene;Acc:114669858]	-0.357672836	0.003634106	0.011229823
ENSMMUG00000021840	stereocilin [Source:NCBI gene;Acc:106996176]	0.300355939	0.011439994	0.030420497
ENSMMUG00000001682	protein disulfide isomerase family A member 3 [Source:VGNC Symbol;Acc:VGNC:75905]	-1.690743577	0.001217257	0.004294296
ENSMMUG00000057376	inositol hexakisphosphate and diphosphoinositol-pentakisphosphate kinase 1-like [Source:NCBI gene;Acc:717098]	0.351712979	0.01220448	0.032147858
ENSMMUG00000016742	CTD small phosphatase like 2 [Source:VGNC Symbol;Acc:VGNC:71593]	0.588310037	6.91E-08	5.71E-07
ENSMMUG00000014288	eukaryotic translation initiation factor 3 subunit J [Source:VGNC Symbol;Acc:VGNC:72188]	0.782542394	7.91E-05	0.000368403
ENSMMUG00000014291	SPG11 vesicle trafficking associated, spatacsin [Source:VGNC Symbol;Acc:VGNC:77856]	1.506502717	3.08E-11	4.16E-10
ENSMMUG00000001284	solute carrier family 28 member 2 [Source:VGNC Symbol;Acc:VGNC:77445]	-0.584260056	0.003287219	0.010289037
ENSMMUG00000005856	glycine amidinotransferase [Source:VGNC Symbol;Acc:VGNC:72892]	-1.704029035	6.17E-15	1.31E-13
ENSMMUG00000049265	Metazoan signal recognition particle RNA [Source:RFAM;Acc:RF00017]	2.451359391	6.62E-09	6.38E-08
ENSMMUG00000062254		2.288880489	6.13E-12	9.05E-11
ENSMMUG00000016896	solute carrier family 12 member 1 [Source:VGNC Symbol;Acc:VGNC:77385]	-1.105470844	1.07E-08	9.96E-08
ENSMMUG00000009833	SHC adaptor protein 4 [Source:VGNC Symbol;Acc:VGNC:77249]	-1.804942612	0.008569005	0.023703614
ENSMMUG00000043893	Metazoan signal recognition particle RNA [Source:RFAM;Acc:RF00017]	-1.299209694	0.019014063	0.046758515
ENSMMUG00000009838	COP9 signalosome subunit 2 [Source:VGNC Symbol;Acc:VGNC:71376]	-1.040577399	7.71E-05	0.000359807
ENSMMUG00000009839	galactokinase 2 [Source:VGNC Symbol;Acc:VGNC:72870]	-1.171563688	2.69E-05	0.000139012
ENSMMUG00000013496	DTW domain containing 1 [Source:VGNC Symbol;Acc:VGNC:72017]	2.918787784	0.010229459	0.027599824
ENSMMUG00000056522		2.225445942	1.03E-09	1.13E-08
ENSMMUG00000050608		2.675276838	1.05E-33	1.52E-31
ENSMMUG00000003684	ATPase phospholipid transporting 8B4 (putative) [Source:VGNC Symbol;Acc:VGNC:84311]	2.252107158	2.17E-25	1.33E-23
ENSMMUG00000057283		2.018446291	8.46E-10	9.38E-09
ENSMMUG00000002876	solute carrier family 27 member 2 [Source:VGNC Symbol;Acc:VGNC:77439]	-1.108472105	0.000705849	0.002635384
ENSMMUG00000002877	histidine decarboxylase [Source:VGNC Symbol;Acc:VGNC:73360]	-1.530777056	0.006105767	0.017661112
ENSMMUG00000002879	GA binding protein transcription factor subunit beta 1 [Source:VGNC Symbol;Acc:VGNC:72845]	-1.101956893	5.41E-10	6.18E-09
ENSMMUG00000060278	Metazoan signal recognition particle RNA [Source:RFAM;Acc:RF00017]	-2.161923191	2.67E-06	1.67E-05
ENSMMUG00000015159	adaptor related protein complex 4 subunit epsilon 1 [Source:VGNC Symbol;Acc:VGNC:69973]	0.378437392	0.008837187	0.02434618
ENSMMUG00000000754	Dmx like 2 [Source:VGNC Symbol;Acc:VGNC:71936]	2.766051845	2.07E-20	7.61E-19
ENSMMUG00000017437	LysM domain containing 2 [Source:NCBI gene;Acc:694916]	-1.678522728	3.15E-05	0.000159811
ENSMMUG00000017432	tropomodulin 2 [Source:VGNC Symbol;Acc:VGNC:78571]	-1.526211346	3.50E-21	1.42E-19
ENSMMUG00000002345	tropomodulin 3 [Source:VGNC Symbol;Acc:VGNC:78572]	1.00445848	2.66E-22	1.22E-20
ENSMMUG00000017374	G protein subunit beta 5 [Source:VGNC Symbol;Acc:VGNC:73097]	-2.813366875	1.08E-06	7.27E-06
ENSMMUG00000007504	myosin VC [Source:VGNC Symbol;Acc:VGNC:75117]	-4.223289524	8.83E-08	7.17E-07
ENSMMUG00000007509	myosin VA [Source:VGNC Symbol;Acc:VGNC:75116]	-0.659910346	0.000797017	0.002935858
ENSMMUG00000048644		-1.612043378	0.000157421	0.000684421

ENSMMUG00000001423	family with sequence similarity 214 member A [Source:VGNC Symbol;Acc:VGNC:72354]	-0.857433396	1.55E-09	1.65E-08
ENSMMUG00000020694	RAB27A, member RAS oncogene family [Source:VGNC Symbol;Acc:VGNC:81527]	0.605059073	0.003211836	0.010091876
ENSMMUG00000010798	cell cycle progression 1 [Source:VGNC Symbol;Acc:VGNC:70844]	0.779946841	2.74E-05	0.000141169
ENSMMUG00000010797	phosphatidylinositol glycan anchor biosynthesis class B [Source:VGNC Symbol;Acc:VGNC:75985]	0.635125388	0.000115385	0.000517905
ENSMMUG00000029638	chromosome 7 C15orf65 homolog [Source:VGNC Symbol;Acc:VGNC:70689]	1.438788694	1.83E-06	1.18E-05
ENSMMUG00000000863	dynein axonemal assembly factor 4 [Source:VGNC Symbol;Acc:VGNC:71881]	2.030042462	9.53E-26	6.01E-24
ENSMMUG00000024054	U6 spliceosomal RNA [Source:RFAM;Acc:RF00026]	-2.266608275	0.013993134	0.036077569
ENSMMUG00000008718	zinc finger protein 280D [Source:NCBI gene;Acc:699509]	-2.049139988	5.83E-21	2.30E-19
ENSMMUG00000004091	transcription factor 12 [Source:VGNC Symbol;Acc:VGNC:78283]	-1.483399615	5.50E-18	1.57E-16
ENSMMUG00000063795		-1.540536432	0.000283752	0.001165661
ENSMMUG00000012690	myocardial zonula adherens protein [Source:NCBI gene;Acc:700247]	-2.269853261	0.000107288	0.00048657
ENSMMUG00000050482		2.117431546	0.000226116	0.000952915
ENSMMUG00000063524		-0.380306551	0.002270329	0.007416076
ENSMMUG00000012933	myosin IE [Source:VGNC Symbol;Acc:VGNC:75114]	0.661809689	5.07E-08	4.27E-07
ENSMMUG00000060242	lactate dehydrogenase A like 6B [Source:NCBI gene;Acc:702403]	1.036341395	0.000270485	0.001117662
ENSMMUG00000057663	U4 spliceosomal RNA [Source:RFAM;Acc:RF00015]	1.183302582	0.002255384	0.007370504
ENSMMUG00000018426	glucosaminyl (N-acetyl) transferase 3, mucin type [Source:VGNC Symbol;Acc:VGNC:84342]	-2.375251523	0.011878878	0.03140174
ENSMMUG00000020348	BCL2 interacting protein 2 [Source:VGNC Symbol;Acc:VGNC:70272]	-1.742300253	3.79E-06	2.30E-05
ENSMMUG00000049749		-2.16946857	5.77E-19	1.78E-17
ENSMMUG00000050503		-1.672305112	0.00014656	0.000642663
ENSMMUG00000001362	vacuolar protein sorting 13 homolog C [Source:VGNC Symbol;Acc:VGNC:79399]	-0.625865442	0.000628496	0.002380741
ENSMMUG00000060717	C2 calcium dependent domain containing 4A [Source:NCBI gene;Acc:704354]	0.507615524	0.001449855	0.005007302
ENSMMUG00000063231	Metazoan signal recognition particle RNA [Source:RFAM;Acc:RF00017]	-0.475495343	0.003666701	0.011307649
ENSMMUG00000060671		-0.489554669	0.006994885	0.019868635
ENSMMUG00000013460	RAB8B, member RAS oncogene family [Source:VGNC Symbol;Acc:VGNC:81550]	1.219435465	1.59E-17	4.38E-16
ENSMMUG00000059755	aph-1 homolog B, gamma-secretase subunit [Source:NCBI gene;Acc:705718]	1.061831802	0.001563941	0.005346529
ENSMMUG00000012117	HECT and RLD domain containing E3 ubiquitin protein ligase family member 1 [Source:VGNC Symbol;Acc:VGNC:73364]	0.661923867	8.99E-12	1.30E-10
ENSMMUG00000049375		0.607017361	1.60E-09	1.70E-08
ENSMMUG00000001247	zinc finger protein 609 [Source:VGNC Symbol;Acc:VGNC:79764]	0.471432029	0.012478129	0.032729158
ENSMMUG00000013270	RNA binding protein, mRNA processing factor 2 [Source:VGNC Symbol;Acc:VGNC:76511]	1.45065422	0.005074807	0.015039846
ENSMMUG00000010949	SPG21 abhydrolase domain containing, maspardin [Source:VGNC Symbol;Acc:VGNC:77970]	1.444504925	3.91E-16	9.21E-15
ENSMMUG00000004028	kelch repeat and BTB domain containing 13 [Source:VGNC Symbol;Acc:VGNC:73953]	1.827873354	0.000103629	0.000471562
ENSMMUG00000006297	programmed cell death 7 [Source:NCBI gene;Acc:708758]	0.607945974	7.43E-05	0.000348231
ENSMMUG00000006714	caseinolytic mitochondrial matrix peptidase chaperone subunit X [Source:VGNC Symbol;Acc:VGNC:84326]	-0.828065193	0.005977971	0.017328637
ENSMMUG00000021788	immunoglobulin superfamily DCC subclass member 4 [Source:VGNC Symbol;Acc:VGNC:73388]	-1.38326795	6.79E-06	3.94E-05
ENSMMUG00000015450	3-hydroxyacyl-CoA dehydratase 3 [Source:VGNC Symbol;Acc:VGNC:73225]	-0.692333079	0.001540961	0.005278921
ENSMMUG00000022056	DENN domain containing 4A [Source:VGNC Symbol;Acc:VGNC:71697]	-1.209785895	1.09E-06	7.35E-06
ENSMMUG00000054184	Metazoan signal recognition particle RNA [Source:RFAM;Acc:RF00017]	1.435914174	8.24E-18	2.33E-16

ENSMMUG00000035748	U6 spliceosomal RNA [Source:RFAM;Acc:RF00026]	1.488038168	2.04E-05	0.000108467
ENSMMUG00000008006	RAB11A, member RAS oncogene family [Source:VGNC Symbol;Acc:VGNC:81517]	1.55131563	2.70E-20	9.80E-19
ENSMMUG00000008007	multiple EGF like domains 11 [Source:VGNC Symbol;Acc:VGNC:84376]	1.287631195	1.30E-11	1.84E-10
ENSMMUG00000052828		1.538399017	1.80E-09	1.90E-08
ENSMMUG00000003693	DIS3 like exosome 3'-5' exoribonuclease [Source:VGNC Symbol;Acc:VGNC:71796]	-4.011947419	5.69E-05	0.000272764
ENSMMUG00000063378		0.560479156	0.00012726	0.000566918
ENSMMUG00000041415	alpha and gamma adaptin binding protein [Source:NCBI gene;Acc:711436]	-2.47147486	4.17E-05	0.000205755
ENSMMUG00000015458	IQ motif containing H [Source:VGNC Symbol;Acc:VGNC:73655]	-0.583562258	0.002645634	0.008496048
ENSMMUG00000004026	integrin subunit alpha 11 [Source:VGNC Symbol;Acc:VGNC:73774]	0.519374972	1.73E-05	9.34E-05
ENSMMUG00000006230	NADPH oxidase 5 [Source:VGNC Symbol;Acc:VGNC:82198]	0.871960089	3.88E-09	3.88E-08
ENSMMUG00000007570	uveal autoantigen with coiled-coil domains and ankyrin repeats [Source:VGNC Symbol;Acc:VGNC:78995]	-0.799559869	2.81E-06	1.75E-05
ENSMMUG00000006490	ariadne RBR E3 ubiquitin protein ligase 1 [Source:VGNC Symbol;Acc:VGNC:84308]	-0.498781066	0.012724258	0.033286426
ENSMMUG00000011309	ADP dependent glucokinase [Source:VGNC Symbol;Acc:VGNC:69692]	0.517798234	8.31E-08	6.77E-07
ENSMMUG00000009584	neogenin 1 [Source:VGNC Symbol;Acc:VGNC:75314]	-2.780529578	0.010319561	0.027815176
ENSMMUG00000057906		0.790252641	1.16E-09	1.26E-08
ENSMMUG00000059627		0.650867484	0.003125621	0.009841392
ENSMMUG00000063495		1.241745309	3.06E-22	1.40E-20
ENSMMUG00000001568	AT-rich interaction domain 3B [Source:VGNC Symbol;Acc:VGNC:69827]	2.128666407	1.50E-19	4.93E-18
ENSMMUG00000011069	CDC like kinase 3 [Source:VGNC Symbol;Acc:VGNC:71260]	1.039851456	0.000253117	0.00105501
ENSMMUG00000022481	cytochrome P450, family 1, subfamily A, polypeptide 1 [Source:NCBI gene;Acc:678694]	0.963110534	1.78E-10	2.18E-09
ENSMMUG00000015267	C-terminal Src kinase [Source:VGNC Symbol;Acc:VGNC:81380]	0.914076727	2.14E-15	4.69E-14
ENSMMUG00000018800	secretory carrier membrane protein 5 [Source:VGNC Symbol;Acc:VGNC:77136]	0.401860733	0.006494247	0.018617237
ENSMMUG00000008529	chromosome 7 C15orf39 homolog [Source:VGNC Symbol;Acc:VGNC:70620]	1.985091902	8.85E-09	8.34E-08
ENSMMUG00000061079	golgin subfamily A member 6C [Source:NCBI gene;Acc:715898]	1.711039489	5.79E-16	1.34E-14
ENSMMUG00000016104	chondroitin sulfate proteoglycan 4 [Source:VGNC Symbol;Acc:VGNC:71525]	0.98697726	8.48E-11	1.08E-09
ENSMMUG00000061431	outer dense fiber of sperm tails 3 like 1 [Source:NCBI gene;Acc:706545]	1.009676664	6.41E-05	0.000304318
ENSMMUG00000053989		1.136086529	7.23E-14	1.37E-12
ENSMMUG00000058835		0.901322276	0.000743673	0.002756794
ENSMMUG00000011047	S-phase cyclin A associated protein in the ER [Source:VGNC Symbol;Acc:VGNC:77006]	-1.155150597	5.06E-05	0.00024581
ENSMMUG00000053250		-1.379645064	3.33E-06	2.04E-05
ENSMMUG00000008213	reticulocalbin 2 [Source:VGNC Symbol;Acc:VGNC:76887]	-0.843596091	0.001425398	0.004932023
ENSMMUG00000010171	proline-serine-threonine phosphatase interacting protein 1 [Source:VGNC Symbol;Acc:VGNC:76459]	-1.067449883	1.83E-09	1.93E-08
ENSMMUG00000042378	pseudopodium enriched atypical kinase 1 [Source:VGNC Symbol;Acc:VGNC:84419]	1.26804401	1.90E-20	7.04E-19
ENSMMUG00000014207	WD repeat domain 61 [Source:VGNC Symbol;Acc:VGNC:79843]	-1.063263181	0.000439143	0.001730663
ENSMMUG00000045680	iron responsive element binding protein 2 [Source:VGNC Symbol;Acc:VGNC:84360]	-1.535845022	5.53E-06	3.26E-05
ENSMMUG00000059973		-0.658734257	0.002951696	0.009367354
ENSMMUG00000007541	cholinergic receptor nicotinic beta 4 subunit [Source:NCBI gene;Acc:106996685]	0.58386306	3.71E-06	2.26E-05
ENSMMUG00000064497		-1.851640475	0.017906741	0.044459563
ENSMMUG00000059589		2.396479933	6.39E-09	6.18E-08
ENSMMUG00000056992	zinc finger AN1-type containing 6 [Source:NCBI gene;Acc:717543]	2.136059528	1.81E-14	3.67E-13
ENSMMUG00000009361	fumarylacetoacetate hydrolase [Source:NCBI gene;Acc:712716]	3.073526513	2.45E-33	3.43E-31

ENSMMUG00000054825		-1.001560615	0.000383247	0.001532369
ENSMMUG00000002038	cell migration inducing hyaluronidase 1 [Source:VGNC Symbol;Acc:VGNC:71130]	-1.125883542	9.68E-07	6.57E-06
ENSMMUG00000009409	mesoderm development LRP chaperone [Source:VGNC Symbol;Acc:VGNC:74507]	-1.268979775	3.33E-05	0.000168239
ENSMMUG00000011564	transmembrane channel like 3 [Source:VGNC Symbol;Acc:VGNC:79360]	3.568912988	0.0002154	0.000912418
ENSMMUG00000057295		0.441008831	0.003373267	0.010522834
ENSMMUG00000003002		-0.775679978	0.000525835	0.002032881
ENSMMUG00000009087	HDGF like 3 [Source:VGNC Symbol;Acc:VGNC:84346]	-1.15811071	0.000105939	0.000481041
ENSMMUG00000009084	transmembrane 6 superfamily member 1 [Source:VGNC Symbol;Acc:VGNC:78566]	-1.598801416	1.04E-11	1.49E-10
ENSMMUG00000062559		-1.296135516	0.003661638	0.011301164
ENSMMUG00000008297	homer scaffold protein 2 [Source:VGNC Symbol;Acc:VGNC:73406]	0.416052172	0.008535442	0.023633422
ENSMMUG00000011328	cytoplasmic polyadenylation element binding protein 1 [Source:NCBI gene;Acc:694357]	1.095804597	1.37E-05	7.52E-05
ENSMMUG00000019838	phosphodiesterase 8A [Source:VGNC Symbol;Acc:VGNC:75924]	-1.293370515	1.82E-10	2.22E-09
ENSMMUG00000056737		-1.75945307	2.54E-05	0.00013216
ENSMMUG00000049563		-1.668338824	6.34E-09	6.14E-08
ENSMMUG00000016772	solute carrier family 28 member 1 [Source:VGNC Symbol;Acc:VGNC:77444]	1.152312328	2.70E-23	1.40E-21
ENSMMUG00000010432	zinc finger protein 592 [Source:VGNC Symbol;Acc:VGNC:79762]	0.64446806	3.33E-09	3.37E-08
ENSMMUG00000063195		0.545432026	7.14E-05	0.000335736
ENSMMUG00000001386	neurotrophic receptor tyrosine kinase 3 [Source:VGNC Symbol;Acc:VGNC:75542]	-1.5129793	0.000110978	0.000500853
ENSMMUG00000057531	mitochondrial ribosomal protein S11 [Source:NCBI gene;Acc:697743]	-0.818939271	0.020235138	0.049357548
ENSMMUG00000043001	milk fat globule EGF and factor V/VIII domain containing [Source:NCBI gene;Acc:703501]	-1.379991291	0.002428662	0.007879469
ENSMMUG00000014414	abhydrolase domain containing 2 [Source:VGNC Symbol;Acc:VGNC:69463]	0.814358799	0.008870331	0.024419345
ENSMMUG00000011155	FA complementation group I [Source:VGNC Symbol;Acc:VGNC:72575]	1.537466567	2.78E-19	8.87E-18
ENSMMUG00000011160	DNA polymerase gamma, catalytic subunit [Source:VGNC Symbol;Acc:VGNC:76205]	0.745929524	0.004370935	0.013223372
ENSMMUG00000056869		-0.284457142	0.017346716	0.043250133
ENSMMUG00000001254	TOPBP1 interacting checkpoint and replication regulator [Source:NCBI gene;Acc:700248]	-0.853361683	0.010784546	0.02884307
ENSMMUG00000015394	WD repeat domain 93 [Source:VGNC Symbol;Acc:VGNC:79642]	1.647524088	1.32E-49	1.22E-46
ENSMMUG00000010586	mesoderm posterior bHLH transcription factor 1 [Source:VGNC Symbol;Acc:VGNC:74618]	1.880926214	4.11E-07	2.97E-06
ENSMMUG00000003584	mesoderm posterior bHLH transcription factor 2 [Source:VGNC Symbol;Acc:VGNC:74619]	1.684601972	3.92E-40	1.10E-37
ENSMMUG00000014983	alanyl aminopeptidase, membrane [Source:VGNC Symbol;Acc:VGNC:69943]	1.711662804	3.79E-39	9.07E-37
ENSMMUG00000029507	adaptor related protein complex 3 subunit sigma 2 [Source:NCBI gene;Acc:701217]	1.536325245	4.86E-41	1.47E-38
ENSMMUG00000023816	semaphorin 4B [Source:VGNC Symbol;Acc:VGNC:77165]	2.610002942	9.67E-44	4.35E-41
ENSMMUG00000004376	calcium and integrin binding 1 [Source:VGNC Symbol;Acc:VGNC:71189]	2.731744554	1.38E-40	4.00E-38
ENSMMUG00000050794	zinc finger protein 774 [Source:NCBI gene;Acc:702985]	-1.295203502	0.000115	0.000516336
ENSMMUG00000019526	BLM RecQ like helicase [Source:VGNC Symbol;Acc:VGNC:70254]	1.539739335	0.000197164	0.000841424
ENSMMUG00000062217	HD domain containing 3 [Source:NCBI gene;Acc:715621]	1.804948265	6.82E-07	4.77E-06
ENSMMUG00000005108	RCC1 domain containing 1 [Source:VGNC Symbol;Acc:VGNC:76876]	1.57948416	0.000216682	0.00091654
ENSMMUG00000063322		1.454965539	1.20E-16	2.97E-15
ENSMMUG00000061958		2.132900488	0.001156672	0.004103696
ENSMMUG00000005220	synemin [Source:VGNC Symbol;Acc:VGNC:78201]	0.536683399	0.000110082	0.000497417
ENSMMUG00000005224	tetratricopeptide repeat domain 23 [Source:VGNC Symbol;Acc:VGNC:79534]	0.38324477	0.000260309	0.001081944

ENSMMUG00000018122	myocyte enhancer factor 2A [Source:VGNC Symbol;Acc:VGNC:74698]	-1.833953176	0.005556118	0.016258332
ENSMMUG00000009934	golgin subfamily A member 8B-like [Source:NCBI gene;Acc:100430473]	-2.56299942	0.011330833	0.030169165
ENSMMUG00000016504	ankyrin repeat and SOCS box containing 7 [Source:VGNC Symbol;Acc:VGNC:69851]	0.929412772	0.019631433	0.048073672
ENSMMUG00000060063		1.018980945	2.75E-07	2.05E-06
ENSMMUG00000006659	aldehyde dehydrogenase 1 family member A3 [Source:VGNC Symbol;Acc:VGNC:81353]	1.404807343	6.24E-05	0.000297299
ENSMMUG00000001863	olfactory receptor, family 4, subfamily K, member 17 [Source:NCBI gene;Acc:697616]	0.869902783	0.013926547	0.035969568
ENSMMUG00000029455	olfactory receptor 4Q2 [Source:NCBI gene;Acc:114679271]	2.739415515	6.72E-29	5.97E-27
ENSMMUG00000053848		1.763829504	0.011266967	0.030014213
ENSMMUG00000045269		3.022501303	0.007225197	0.020409462
ENSMMUG00000058162		3.239128892	0.000853489	0.003119854
ENSMMUG00000016251	cyclin B1 interacting protein 1 [Source:VGNC Symbol;Acc:VGNC:70827]	2.863543216	0.00193632	0.006476092
ENSMMUG00000016255	telomerase associated protein 1 [Source:VGNC Symbol;Acc:VGNC:78310]	1.201507191	3.68E-07	2.68E-06
ENSMMUG00000048039	apurinic/apyrimidinic endodeoxyribonuclease 1 [Source:VGNC Symbol;Acc:VGNC:84305]	1.519312478	3.12E-05	0.000158814
ENSMMUG00000019126	purine nucleoside phosphorylase [Source:NCBI gene;Acc:704183]	1.295902639	3.44E-05	0.000173236
ENSMMUG00000056465	epididymal protein 3A [Source:NCBI gene;Acc:704355]	0.400822858	0.001154226	0.004095997
ENSMMUG00000001656	epididymal protein 3B [Source:VGNC Symbol;Acc:VGNC:84336]	0.338910464	0.011648952	0.030893054
ENSMMUG00000056509	ribonuclease, RNase A family, 3 [Source:NCBI gene;Acc:100425327]	0.592450456	1.34E-05	7.38E-05
ENSMMUG00000020747	NDRG family member 2 [Source:VGNC Symbol;Acc:VGNC:74953]	-2.616439937	0.002996643	0.009485639
ENSMMUG00000050445	ribonuclease A family member 8 [Source:NCBI gene;Acc:716333]	-3.512814542	0.000397619	0.001585132
ENSMMUG00000006457	Rho guanine nucleotide exchange factor 40 [Source:VGNC Symbol;Acc:VGNC:69907]	-4.037803488	4.52E-09	4.47E-08
ENSMMUG00000014809	heterogeneous nuclear ribonucleoprotein C [Source:VGNC Symbol;Acc:VGNC:84348]	-3.338837089	1.46E-05	7.98E-05
ENSMMUG00000021552	RPGR interacting protein 1 [Source:VGNC Symbol;Acc:VGNC:77007]	-4.286048258	1.45E-20	5.49E-19
ENSMMUG00000055229		-3.587303646	3.97E-28	3.30E-26
ENSMMUG00000015000	SPT16 homolog, facilitates chromatin remodeling subunit [Source:VGNC Symbol;Acc:VGNC:78054]	-3.06372006	9.04E-21	3.48E-19
ENSMMUG00000015002	chromodomain helicase DNA binding protein 8 [Source:VGNC Symbol;Acc:VGNC:71060]	-2.418430406	5.97E-11	7.78E-10
ENSMMUG00000014992	RAB2B, member RAS oncogene family [Source:VGNC Symbol;Acc:VGNC:81530]	-1.997924479	0.006989581	0.019857373
ENSMMUG00000015004	TOX high mobility group box family member 4 [Source:NCBI gene;Acc:706950]	-1.873136966	0.000353319	0.00142461
ENSMMUG00000014997	spalt like transcription factor 2 [Source:NCBI gene;Acc:708367]	-2.024234925	1.53E-07	1.20E-06
ENSMMUG00000047342	T cell receptor alpha variable 12-2 [Source:HGNC Symbol;Acc:HGNC:12106]	-1.378570291	0.004413508	0.013330413
ENSMMUG00000062974		-1.912975386	5.63E-07	4.00E-06
ENSMMUG00000040154	T cell receptor alpha variable 9-2 [Source:HGNC Symbol;Acc:HGNC:12154]	-2.569496293	8.34E-16	1.91E-14
ENSMMUG00000038070	T cell receptor alpha variable 12-3 [Source:HGNC Symbol;Acc:HGNC:12107]	-2.683099638	1.34E-27	1.08E-25
ENSMMUG00000054116	T cell receptor alpha variable 8-6 [Source:HGNC Symbol;Acc:HGNC:12151]	-2.795034124	2.36E-25	1.44E-23
ENSMMUG00000056910		-1.476495414	3.12E-07	2.30E-06
ENSMMUG00000056431		0.40384208	0.000708428	0.00264368
ENSMMUG00000057791		1.230722632	7.60E-05	0.000355032
ENSMMUG00000053686		3.353433195	0.004034009	0.012322216

ENSMMUG00000000413	solute carrier family 7 member 7 [Source:VGNC Symbol;Acc:VGNC:77631]	-0.568216095	0.018058346	0.044790981
ENSMMUG00000014748	solute carrier family 22 member 17 [Source:VGNC Symbol;Acc:VGNC:77560]	-2.173066899	0.000152896	0.000667749
ENSMMUG00000014762	myosin heavy chain 6 [Source:NCBI gene;Acc:713471]	-1.243517001	8.24E-09	7.83E-08
ENSMMUG00000029420		1.209068492	0.000251442	0.001049207
ENSMMUG00000014766	neuroguidin [Source:VGNC Symbol;Acc:VGNC:75220]	0.776054465	0.000443089	0.001742975
ENSMMUG00000045970	thiamine triphosphatase [Source:NCBI gene;Acc:714403]	0.751299848	3.40E-07	2.49E-06
ENSMMUG00000014771	zinc finger homeobox 2 [Source:NCBI gene;Acc:100429448]	1.148213876	2.34E-14	4.64E-13
ENSMMUG00000004144	adaptor related protein complex 1 subunit gamma 2 [Source:VGNC Symbol;Acc:VGNC:69959]	0.648989833	5.01E-06	2.98E-05
ENSMMUG00000041108	junctophilin 4 [Source:VGNC Symbol;Acc:VGNC:84363]	0.343047764	0.011914089	0.031477986
ENSMMUG00000059196		0.999367028	6.18E-13	1.05E-11
ENSMMUG00000050597		2.637928694	0.00787462	0.022021613
ENSMMUG00000012462	proteasome activator subunit 1 [Source:VGNC Symbol;Acc:VGNC:76455]	-2.148897118	6.19E-09	6.00E-08
ENSMMUG00000012473	proteasome activator subunit 2 [Source:VGNC Symbol;Acc:VGNC:76456]	-3.062878624	1.43E-07	1.13E-06
ENSMMUG00000012474	ring finger protein 31 [Source:NCBI gene;Acc:715239]	-2.367988266	1.63E-05	8.82E-05
ENSMMUG00000040817	interferon regulatory factor 9 [Source:VGNC Symbol;Acc:VGNC:84361]	-2.2252276	5.47E-05	0.000263559
ENSMMUG00000012480	REC8 meiotic recombination protein [Source:VGNC Symbol;Acc:VGNC:76746]	-2.913504756	7.47E-13	1.26E-11
ENSMMUG00000012481	importin 4 [Source:VGNC Symbol;Acc:VGNC:73630]	-2.420550143	1.24E-12	2.01E-11
ENSMMUG00000012493	transmembrane 9 superfamily member 1 [Source:NCBI gene;Acc:715472]	-2.978648234	0.002079344	0.006883186
ENSMMUG00000012452	transglutaminase 1 [Source:NCBI gene;Acc:715854]	-2.0983613	0.007306245	0.020600487
ENSMMUG00000010049	NOP9 nucleolar protein [Source:VGNC Symbol;Acc:VGNC:75216]	-2.016352405	0.001035356	0.003712361
ENSMMUG00000019692	sec1 family domain containing 1 [Source:VGNC Symbol;Acc:VGNC:77065]	0.777808339	1.91E-05	0.000102039
ENSMMUG00000011515	striatin 3 [Source:VGNC Symbol;Acc:VGNC:78127]	-1.466646063	4.08E-05	0.000201903
ENSMMUG00000003123	adaptor related protein complex 4 subunit sigma 1 [Source:VGNC Symbol;Acc:VGNC:84304]	-1.7709731	0.016238187	0.040947511
ENSMMUG00000003120	HECT domain E3 ubiquitin protein ligase 1 [Source:VGNC Symbol;Acc:VGNC:73200]	-1.777791464	1.94E-08	1.75E-07
ENSMMUG00000056906		1.14112466	3.41E-14	6.63E-13
ENSMMUG00000012576	sorting nexin 6 [Source:NCBI gene;Acc:693626]	-1.051491748	0.000433379	0.001711587
ENSMMUG00000015073	signal recognition particle 54 [Source:VGNC Symbol;Acc:VGNC:82247]	-0.558854351	0.001781948	0.006015544
ENSMMUG00000003530	protein phosphatase 2 regulatory subunit B''gamma [Source:VGNC Symbol;Acc:VGNC:76279]	-0.651787894	6.40E-06	3.73E-05
ENSMMUG00000016487	Ral GTPase activating protein catalytic alpha subunit 1 [Source:VGNC Symbol;Acc:VGNC:76504]	-1.063384355	0.0021424	0.007052561
ENSMMUG00000009648	paired box 9 [Source:VGNC Symbol;Acc:VGNC:75768]	1.806884771	0.018335219	0.045371709
ENSMMUG00000056013	forkhead box A1 [Source:NCBI gene;Acc:697909]	2.620762651	0.000238237	0.000998883
ENSMMUG00000052465		-1.253055289	0.003063133	0.009663105
ENSMMUG00000052781		-0.585127307	3.96E-05	0.000195792
ENSMMUG00000017677	chromosome 7 C14orf28 homolog [Source:VGNC Symbol;Acc:VGNC:70581]	-0.76652536	8.67E-10	9.59E-09
ENSMMUG00000016513	FA complementation group M [Source:VGNC Symbol;Acc:VGNC:72554]	3.230774526	0.003184767	0.010012741
ENSMMUG00000003913	DNA polymerase epsilon 2, accessory subunit [Source:VGNC Symbol;Acc:VGNC:76204]	0.796782865	0.000235576	0.000989404

ENSMMUG00000008214	nuclear export mediator factor [Source:VGNC Symbol;Acc:VGNC:84407]	-1.278489193	5.08E-05	0.00024683
ENSMMUG00000055903	Metazoan signal recognition particle RNA [Source:RFAM;Acc:RF00017]	-1.663609573	0.004335988	0.013135532
ENSMMUG00000049543		1.390428066	5.86E-06	3.44E-05
ENSMMUG00000055012		1.980101051	9.28E-06	5.24E-05
ENSMMUG00000059134		1.875424611	2.09E-07	1.59E-06
ENSMMUG00000007464	SOS Ras/Rho guanine nucleotide exchange factor 2 [Source:VGNC Symbol;Acc:VGNC:77838]	-0.610699799	0.015469012	0.039214606
ENSMMUG00000009577	atlastin GTPase 1 [Source:VGNC Symbol;Acc:VGNC:70049]	1.113517504	3.00E-16	7.18E-15
ENSMMUG00000014658	ninein [Source:VGNC Symbol;Acc:VGNC:75177]	4.051661295	2.00E-44	9.59E-42
ENSMMUG00000056844		2.042354419	5.74E-06	3.38E-05
ENSMMUG00000018274	FERM domain containing 6 [Source:VGNC Symbol;Acc:VGNC:72747]	0.991188947	8.10E-10	9.01E-09
ENSMMUG00000018200	RNA transcription, translation and transport factor [Source:VGNC Symbol;Acc:VGNC:77087]	2.432763856	5.65E-05	0.000271132
ENSMMUG00000000023	prostaglandin E receptor 2 [Source:VGNC Symbol;Acc:VGNC:76468]	1.091507924	2.63E-05	0.000136178
ENSMMUG00000006997	thioredoxin domain containing 16 [Source:VGNC Symbol;Acc:VGNC:79214]	1.705807935	1.70E-43	7.43E-41
ENSMMUG00000010689	fermitin family member 2 [Source:VGNC Symbol;Acc:VGNC:82144]	-0.848460121	0.00058391	0.002228353
ENSMMUG00000004084	GTP cyclohydrolase 1 [Source:VGNC Symbol;Acc:VGNC:72900]	-0.803364006	0.001574461	0.005373421
ENSMMUG00000000302	WD repeat and HMG-box DNA binding protein 1 [Source:VGNC Symbol;Acc:VGNC:79376]	2.573351018	3.74E-46	1.98E-43
ENSMMUG00000054112		-0.874805169	1.69E-09	1.79E-08
ENSMMUG00000003567	DLG associated protein 5 [Source:VGNC Symbol;Acc:VGNC:71850]	-0.764598017	1.07E-05	6.00E-05
ENSMMUG00000023041	TATA-box binding protein like 2 [Source:VGNC Symbol;Acc:VGNC:78175]	-0.948317855	0.000266605	0.00110471
ENSMMUG00000061576		1.928859754	1.04E-05	5.81E-05
ENSMMUG00000053489		1.32066157	6.92E-08	5.71E-07
ENSMMUG00000059984	pellino E3 ubiquitin protein ligase family member 2 [Source:NCBI gene;Acc:699169]	1.505999971	0.001711182	0.005797768
ENSMMUG00000018278	exocyst complex component 5 [Source:VGNC Symbol;Acc:VGNC:72260]	-1.612057667	0.000580909	0.00222033
ENSMMUG00000003508	actin related protein 10 [Source:VGNC Symbol;Acc:VGNC:69449]	0.59354683	2.04E-07	1.56E-06
ENSMMUG00000003509	proteasome 20S subunit alpha 3 [Source:NCBI gene;Acc:700251]	0.44301678	0.012231332	0.032190001
ENSMMUG00000055678	translocase of outer mitochondrial membrane 20 like [Source:NCBI gene;Acc:700628]	-1.467481057	0.000280628	0.00115443
ENSMMUG00000004854	leucine rich repeat containing 9 [Source:VGNC Symbol;Acc:VGNC:81443]	1.854026754	4.18E-30	4.08E-28
ENSMMUG00000002540	dehydrogenase/reductase 7 [Source:VGNC Symbol;Acc:VGNC:71690]	0.619530449	7.23E-07	5.03E-06
ENSMMUG00000058852	SIX homeobox 1 [Source:NCBI gene;Acc:703696]	-0.98607231	0.005848075	0.017003563
ENSMMUG00000058450		-1.871114495	3.32E-05	0.000167812
ENSMMUG00000001640	SIX homeobox 4 [Source:NCBI gene;Acc:703798]	-2.049121609	7.21E-11	9.31E-10
ENSMMUG00000001642	MNAT1 component of CDK activating kinase [Source:VGNC Symbol;Acc:VGNC:74905]	-1.442597609	9.92E-09	9.30E-08
ENSMMUG00000050297		-1.502935239	1.33E-06	8.80E-06
ENSMMUG00000051446		-1.227950463	0.001186969	0.004199411
ENSMMUG00000007264	transmembrane protein 30B [Source:VGNC Symbol;Acc:VGNC:79656]	-1.633483408	7.87E-07	5.45E-06
ENSMMUG00000002260	protein kinase C eta [Source:VGNC Symbol;Acc:VGNC:84424]	1.511798249	1.54E-29	1.47E-27
ENSMMUG00000008203	ras homolog family member J [Source:VGNC Symbol;Acc:VGNC:76885]	-0.757126025	0.000521535	0.002017834

ENSMMUG00000054689		-1.837124293	3.83E-07	2.77E-06
ENSMMUG00000001008	spectrin repeat containing nuclear envelope protein 2 [Source:NCBI gene;Acc:706759]	-1.21898011	3.26E-06	2.01E-05
ENSMMUG00000001319	methylenetetrahydrofolate dehydrogenase, cyclohydrolase and formyltetrahydrofolate synthetase 1 [Source:VGNC Symbol;Acc:VGNC:84399]	-1.085397451	1.45E-09	1.55E-08
ENSMMUG00000004748	zinc finger and BTB domain containing 1 [Source:VGNC Symbol;Acc:VGNC:79238]	1.477579369	2.49E-19	8.02E-18
ENSMMUG00000056063		3.153791295	6.41E-10	7.25E-09
ENSMMUG00000053575	Metazoan signal recognition particle RNA [Source:RFAM;Acc:RF00017]	1.298190037	0.004817308	0.014397557
ENSMMUG00000019172	spectrin beta, erythrocytic [Source:VGNC Symbol;Acc:VGNC:77870]	1.025465509	4.69E-05	0.000229825
ENSMMUG00000004340	churchill domain containing 1 [Source:NCBI gene;Acc:100427906]	0.5982655	9.59E-05	0.000439612
ENSMMUG00000021987	glutathione peroxidase 2 [Source:VGNC Symbol;Acc:VGNC:73152]	2.061974242	0.007968762	0.022247127
ENSMMUG00000004339	RAB15, member RAS oncogene family [Source:VGNC Symbol;Acc:VGNC:81519]	0.827524602	0.000284281	0.001167188
ENSMMUG00000053774		-1.629467886	0.008494611	0.023524759
ENSMMUG00000013848	fucosyltransferase 8 [Source:VGNC Symbol;Acc:VGNC:72829]	-2.191970213	7.74E-11	9.92E-10
ENSMMUG00000013907	ribosomal protein S27a [Source:NCBI gene;Acc:718909]	-1.814704684	0.000162186	0.000704515
ENSMMUG00000033490	mml-mir-625 [Source:miRBase;Acc:MI0007874]	-2.890405232	0.004762364	0.014256329
ENSMMUG00000006768	coiled-coil domain containing 196 [Source:VGNC Symbol;Acc:VGNC:70828]	-2.780369571	7.36E-10	8.27E-09
ENSMMUG00000009520	gephyrin [Source:VGNC Symbol;Acc:VGNC:72948]	-2.008200947	3.81E-08	3.24E-07
ENSMMUG00000021418	ATPase H+ transporting V1 subunit D [Source:VGNC Symbol;Acc:VGNC:70060]	1.055334937	4.57E-06	2.74E-05
ENSMMUG00000021419	eukaryotic translation initiation factor 2 subunit alpha [Source:VGNC Symbol;Acc:VGNC:72187]	-0.588089791	0.004718724	0.01414282
ENSMMUG00000021327	phosphatidylinositol glycan anchor biosynthesis class H [Source:VGNC Symbol;Acc:VGNC:75987]	-1.421063201	8.95E-05	0.000412809
ENSMMUG00000015865	arginase 2 [Source:VGNC Symbol;Acc:VGNC:70005]	2.76982968	1.77E-34	2.87E-32
ENSMMUG00000015866	vesicle transport through interaction with t-SNAREs 1B [Source:NCBI gene;Acc:711113]	2.237071205	6.20E-34	9.28E-32
ENSMMUG00000015868	retinol dehydrogenase 11 [Source:NCBI gene;Acc:711195]	1.006907409	1.88E-11	2.60E-10
ENSMMUG00000015869	retinol dehydrogenase 12 [Source:VGNC Symbol;Acc:VGNC:76781]	-1.014517104	0.003608279	0.011157375
ENSMMUG00000015871	zinc finger FYVE-type containing 26 [Source:VGNC Symbol;Acc:VGNC:79283]	1.370637455	8.74E-05	0.000404333
ENSMMUG00000050511		1.223349876	0.001488444	0.00512268
ENSMMUG00000015873	RAD51 paralog B [Source:VGNC Symbol;Acc:VGNC:76647]	1.054900587	0.009392479	0.025666198
ENSMMUG00000064908		1.014280377	0.016229459	0.040932461
ENSMMUG00000054163		-2.701470746	0.011017374	0.029407426
ENSMMUG00000021572	solute carrier family 39 member 9 [Source:VGNC Symbol;Acc:VGNC:77523]	-0.609463558	0.00563311	0.016451165
ENSMMUG00000055018	sushi domain containing 6 [Source:NCBI gene;Acc:712719]	1.788584567	7.80E-17	1.98E-15
ENSMMUG00000022407	serine and arginine rich splicing factor 5 [Source:NCBI gene;Acc:712867]	1.478145793	9.19E-09	8.64E-08
ENSMMUG00000022409	solute carrier family 10 member 1 [Source:VGNC Symbol;Acc:VGNC:77380]	1.952998426	1.06E-28	9.26E-27
ENSMMUG00000045443		1.508623245	0.012687481	0.033207791
ENSMMUG00000011084	ADAM metallopeptidase domain 21 [Source:NCBI gene;Acc:715640]	-1.006474619	0.00294531	0.009349089
ENSMMUG00000059282		-1.33583777	0.004850666	0.01448266
ENSMMUG00000052580	disintegrin and metalloproteinase domain-containing protein 20-like [Source:NCBI gene;Acc:713632]	-1.061101026	0.001252103	0.004400162
ENSMMUG00000019470	pecanex 1 [Source:VGNC Symbol;Acc:VGNC:75655]	-0.762832696	4.34E-06	2.61E-05

ENSMMUG00000060340		-0.778504015	0.005582848	0.016323666
ENSMMUG00000001820	regulator of G protein signaling 6 [Source:VGNC Symbol;Acc:VGNC:76691]	3.1777234	0.000181715	0.000781566
ENSMMUG00000002332	RNA binding motif protein 25 [Source:VGNC Symbol;Acc:VGNC:76521]	-1.281494822	0.000138988	0.000613266
ENSMMUG00000010173	presenilin 1 [Source:VGNC Symbol;Acc:VGNC:76435]	1.879448453	1.83E-17	4.98E-16
ENSMMUG00000037015	U7 small nuclear RNA [Source:RFAM;Acc:RF00066]	1.799106844	0.013929102	0.035969568
ENSMMUG00000056580	Metazoan signal recognition particle RNA [Source:RFAM;Acc:RF00017]	2.88928679	6.33E-05	0.000301111
ENSMMUG00000000313	HEAT repeat containing 4 [Source:VGNC Symbol;Acc:VGNC:73176]	2.030251492	2.23E-32	2.78E-30
ENSMMUG00000053481		1.967107021	1.79E-12	2.86E-11
ENSMMUG00000049894		2.120140166	2.25E-07	1.70E-06
ENSMMUG00000006855	acyl-CoA thioesterase 2 [Source:NCBI gene;Acc:697011]	1.961661785	5.83E-20	2.04E-18
ENSMMUG00000004910	dynein axonemal light chain 1 [Source:VGNC Symbol;Acc:VGNC:71885]	1.692376846	3.24E-09	3.29E-08
ENSMMUG00000034824	U6 spliceosomal RNA [Source:RFAM;Acc:RF00026]	2.071632474	0.009807578	0.026604423
ENSMMUG00000004911	PNMA family member 1 [Source:VGNC Symbol;Acc:VGNC:76199]	1.129521791	0.005220975	0.015380823
ENSMMUG00000004913	mitotic deacetylase associated SANT domain protein [Source:VGNC Symbol;Acc:VGNC:72073]	0.878483186	4.63E-09	4.56E-08
ENSMMUG00000011148	coenzyme Q6, monooxygenase [Source:VGNC Symbol;Acc:VGNC:71239]	0.352898958	0.006380325	0.018347919
ENSMMUG00000056376		2.326081145	1.22E-14	2.52E-13
ENSMMUG00000004255	YLP motif containing 1 [Source:VGNC Symbol;Acc:VGNC:79092]	1.423494663	8.76E-06	4.98E-05
ENSMMUG00000002909	placental growth factor [Source:VGNC Symbol;Acc:VGNC:75964]	0.618150785	0.005875114	0.017063834
ENSMMUG00000002911	mutL homolog 3 [Source:VGNC Symbol;Acc:VGNC:82187]	-1.299697302	0.000260609	0.001082891
ENSMMUG00000015165	zinc finger C2HC-type containing 1C [Source:VGNC Symbol;Acc:VGNC:79247]	-1.003663229	0.00487465	0.014548413
ENSMMUG00000015166	NIMA related kinase 9 [Source:VGNC Symbol;Acc:VGNC:75209]	-1.129761882	9.40E-20	3.21E-18
ENSMMUG00000015168	transmembrane p24 trafficking protein 10 [Source:NCBI gene;Acc:701522]	-0.928626283	0.001031396	0.003700848
ENSMMUG00000040100	Fos proto-oncogene, AP-1 transcription factor subunit [Source:VGNC Symbol;Acc:VGNC:84340]	-0.70307676	2.11E-05	0.000111291
ENSMMUG00000003834	basic leucine zipper ATF-like transcription factor [Source:VGNC Symbol;Acc:VGNC:70229]	1.403332891	2.47E-07	1.86E-06
ENSMMUG00000012065	tubulin tyrosine ligase like 5 [Source:NCBI gene;Acc:703116]	2.171729301	3.33E-24	1.86E-22
ENSMMUG00000012069	transforming growth factor beta 3 [Source:VGNC Symbol;Acc:VGNC:78335]	-1.087719009	6.78E-05	0.00032028
ENSMMUG00000008792	G-patch domain containing 2 like [Source:VGNC Symbol;Acc:VGNC:72973]	-1.293547707	2.28E-06	1.45E-05
ENSMMUG00000016773	estrogen related receptor beta [Source:VGNC Symbol;Acc:VGNC:72434]	0.553706666	0.002588483	0.008335982
ENSMMUG00000057021	CLOCK interacting pacemaker [Source:NCBI gene;Acc:704679]	-1.374823542	1.91E-07	1.47E-06
ENSMMUG00000007561	zinc finger DHHC-type palmitoyltransferase 22 [Source:NCBI gene;Acc:706468]	-1.689924775	3.84E-15	8.27E-14
ENSMMUG00000001097	glutathione S-transferase zeta 1 [Source:VGNC Symbol;Acc:VGNC:73304]	0.417647549	0.015140678	0.038553616
ENSMMUG00000013551	VPS33B interacting protein, apical-basolateral polarity regulator, spe-39 homolog [Source:VGNC Symbol;Acc:VGNC:79246]	-0.823371875	0.002127147	0.007013249
ENSMMUG00000023627	aarF domain containing kinase 1 [Source:VGNC Symbol;Acc:VGNC:69472]	1.015775066	0.005195967	0.015328464
ENSMMUG00000019710	centrosomal protein 128 [Source:VGNC Symbol;Acc:VGNC:71062]	-0.833687348	0.005006855	0.014866455
ENSMMUG00000019713	thyroid stimulating hormone receptor [Source:VGNC Symbol;Acc:VGNC:78662]	-1.218068299	2.07E-05	0.000109304
ENSMMUG00000022124	stonin 2 [Source:VGNC Symbol;Acc:VGNC:78249]	-1.479969038	7.32E-05	0.000343385
ENSMMUG00000017261	galactosylceramidase [Source:VGNC Symbol;Acc:VGNC:72867]	1.384783658	6.91E-26	4.46E-24
ENSMMUG00000017260	G protein-coupled receptor 65 [Source:VGNC Symbol;Acc:VGNC:73140]	1.575573007	1.66E-17	4.57E-16
ENSMMUG00000021011	spermatogenesis associated 7 [Source:VGNC Symbol;Acc:VGNC:77971]	-1.465944841	0.007603915	0.0213653

ENSMMUG00000004603	zinc finger CCCH-type containing 14 [Source:VGNC Symbol;Acc:VGNC:79255]	-0.521169707	0.000626675	0.00237627
ENSMMUG00000004606	EMAP like 5 [Source:VGNC Symbol;Acc:VGNC:72067]	-1.033288276	2.76E-08	2.42E-07
ENSMMUG00000004460	tyrosyl-DNA phosphodiesterase 1 [Source:VGNC Symbol;Acc:VGNC:79164]	-1.185258966	2.88E-05	0.000147512
ENSMMUG00000020687	coiled-coil domain containing 88C [Source:VGNC Symbol;Acc:VGNC:84321]	-0.473893482	0.000420249	0.001665055
ENSMMUG00000008447	fibulin 5 [Source:VGNC Symbol;Acc:VGNC:72622]	-2.160952715	3.28E-06	2.02E-05
ENSMMUG00000020751	ataxin 3 [Source:NCBI gene;Acc:720515]	-0.922268239	0.000168238	0.000727395
ENSMMUG00000000699	cleavage and polyadenylation specific factor 2 [Source:VGNC Symbol;Acc:VGNC:71473]	0.868331638	0.002472433	0.00799873
ENSMMUG00000013966	legumain [Source:VGNC Symbol;Acc:VGNC:74274]	1.159482981	6.33E-11	8.23E-10
ENSMMUG00000059579		0.658148622	0.008547413	0.023654782
ENSMMUG00000009974	inositol-tetrakisphosphate 1-kinase [Source:VGNC Symbol;Acc:VGNC:73802]	1.422547991	0.001052197	0.003765465
ENSMMUG00000044581	modulator of apoptosis 1 [Source:VGNC Symbol;Acc:VGNC:84392]	1.358163054	3.59E-06	2.19E-05
ENSMMUG00000061833	transmembrane protein 251 [Source:NCBI gene;Acc:708131]	1.560852782	7.69E-06	4.41E-05
ENSMMUG00000053729		-0.645190778	0.000221388	0.000934581
ENSMMUG00000062301		-0.795293338	0.001975705	0.006585683
ENSMMUG00000052453	serpin family A member 11 [Source:NCBI gene;Acc:701483]	3.438808714	1.31E-10	1.63E-09
ENSMMUG00000012356	serpin family A member 9 [Source:VGNC Symbol;Acc:VGNC:77241]	3.016023684	7.37E-20	2.55E-18
ENSMMUG00000020586	serpin family A member 12 [Source:VGNC Symbol;Acc:VGNC:77266]	4.173462552	0.000180823	0.000777954
ENSMMUG00000064358		2.882263469	0.012546237	0.032878723
ENSMMUG00000000681	calmin [Source:VGNC Symbol;Acc:VGNC:71232]	-1.250668619	2.58E-12	4.03E-11
ENSMMUG00000054267	glutaredoxin 5 [Source:NCBI gene;Acc:702989]	0.551231939	0.00066378	0.002498442
ENSMMUG00000003658	autophagy related 2B [Source:VGNC Symbol;Acc:VGNC:84310]	1.209223437	0.002149458	0.007072657
ENSMMUG00000051277		-2.405682858	0.001891814	0.00634025
ENSMMUG00000003261	BAF chromatin remodeling complex subunit BCL11B [Source:VGNC Symbol;Acc:VGNC:70056]	-2.592637487	2.28E-42	8.67E-40
ENSMMUG00000051552		-2.173703934	4.13E-10	4.79E-09
ENSMMUG00000061892		-2.092369755	2.06E-19	6.66E-18
ENSMMUG00000049437	Metazoan signal recognition particle RNA [Source:RFAM;Acc:RF00017]	-1.925997106	1.10E-11	1.56E-10
ENSMMUG00000029197	YY1 transcription factor [Source:NCBI gene;Acc:717370]	-2.233649203	1.19E-16	2.97E-15
ENSMMUG00000051100	Metazoan signal recognition particle RNA [Source:RFAM;Acc:RF00017]	-3.072275426	1.02E-05	5.72E-05
ENSMMUG00000013871	WD repeat domain 25 [Source:VGNC Symbol;Acc:VGNC:78420]	2.203500767	2.81E-15	6.12E-14
ENSMMUG00000005780	dynein cytoplasmic 1 heavy chain 1 [Source:VGNC Symbol;Acc:VGNC:71951]	0.36499602	0.000134892	0.000597148
ENSMMUG00000054990	heat shock protein 90 alpha family class A member 1 [Source:NCBI gene;Acc:708431]	-0.468080661	0.018951572	0.046647605
ENSMMUG00000060616	Metazoan signal recognition particle RNA [Source:RFAM;Acc:RF00017]	0.435458616	0.009406155	0.025694858
ENSMMUG00000007135	WD repeat domain 20 [Source:VGNC Symbol;Acc:VGNC:79196]	0.522160627	6.14E-06	3.59E-05
ENSMMUG00000007140	MOK protein kinase [Source:VGNC Symbol;Acc:VGNC:84394]	1.879283611	1.65E-06	1.08E-05
ENSMMUG00000003483	cyclin dependent kinase 2 interacting protein [Source:VGNC Symbol;Acc:VGNC:71326]	-1.39491588	7.49E-11	9.64E-10
ENSMMUG00000003484	tectonin beta-propeller repeat containing 2 [Source:NCBI gene;Acc:708959]	2.169689865	1.48E-16	3.66E-15
ENSMMUG00000057818		2.120430529	0.001453733	0.005018358

ENSMMUG00000005845	REST corepressor 1 [Source:VGNC Symbol;Acc:VGNC:76752]	1.298210953	2.03E-07	1.55E-06
ENSMMUG00000008380	CDC42 binding protein kinase beta [Source:VGNC Symbol;Acc:VGNC:81369]	0.635332704	0.008424929	0.023375435
ENSMMUG00000029186	exocyst complex component 3 like 4 [Source:VGNC Symbol;Acc:VGNC:72305]	2.825685744	0.005654028	0.016499259
ENSMMUG00000050495		2.399648044	8.14E-06	4.65E-05
ENSMMUG00000021634	TNF alpha induced protein 2 [Source:VGNC Symbol;Acc:VGNC:78579]	2.528129411	3.53E-39	8.59E-37
ENSMMUG00000049145	creatine kinase B [Source:NCBI gene;Acc:711167]	0.848628398	2.27E-06	1.44E-05
ENSMMUG00000007673	tRNA methyltransferase 61A [Source:VGNC Symbol;Acc:VGNC:79412]	0.602984263	0.017856634	0.044357433
ENSMMUG00000029181	X-ray repair cross complementing 3 [Source:NCBI gene;Acc:695788]	0.459973224	0.00037657	0.001510556
ENSMMUG00000018121	zinc finger FYVE-type containing 21 [Source:VGNC Symbol;Acc:VGNC:79282]	0.653802404	8.55E-09	8.08E-08
ENSMMUG00000016552	inverted formin, FH2 and WH2 domain containing [Source:VGNC Symbol;Acc:VGNC:84357]	2.087839319	0.000516999	0.002002897
ENSMMUG00000052518	SIVA1 apoptosis inducing factor [Source:NCBI gene;Acc:706688]	-2.010925903	0.000203662	0.000867159
ENSMMUG00000001044	AKT serine/threonine kinase 1 [Source:VGNC Symbol;Acc:VGNC:69776]	-0.844603275	0.001798475	0.006064434
ENSMMUG00000064694	AHNAK nucleoprotein 2 [Source:NCBI gene;Acc:707626]	3.217219093	0.000173635	0.000748766
ENSMMUG00000007517	G protein-coupled receptor 132 [Source:VGNC Symbol;Acc:VGNC:84343]	-1.570797714	0.010587137	0.028391906
ENSMMUG00000065019	Metazoan signal recognition particle RNA [Source:RFAM;Acc:RF00017]	-2.272978641	2.76E-06	1.72E-05
ENSMMUG00000022939	phosphofurin acidic cluster sorting protein 2 [Source:VGNC Symbol;Acc:VGNC:75671]	-1.068314804	0.0021191	0.006991379
ENSMMUG00000007159	tubulin epsilon and delta complex 1 [Source:VGNC Symbol;Acc:VGNC:79235]	-1.527981847	2.93E-05	0.000150339
ENSMMUG00000015202		-3.314870836	6.42E-05	0.000304972
ENSMMUG00000040771		-1.872190315	1.96E-05	0.000104705
ENSMMUG00000045298	immunoglobulin heavy constant mu [Source:HGNC Symbol;Acc:HGNC:5541]	-0.487993774	0.010479567	0.028159447
ENSMMUG00000059525		-1.87551475	2.14E-06	1.37E-05
ENSMMUG00000055207		-5.235582452	8.07E-11	1.03E-09
ENSMMUG00000060892		-1.791273496	2.65E-07	1.98E-06
ENSMMUG00000064429	immunoglobulin heavy variable 1-45 [Source:HGNC Symbol;Acc:HGNC:5553]	-3.342884788	0.00213894	0.007045863
ENSMMUG00000052929		-4.102802409	2.44E-08	2.16E-07
ENSMMUG00000052535		-4.439949115	3.69E-07	2.69E-06
ENSMMUG00000060524		-1.952318137	0.003649254	0.011265288
ENSMMUG00000063346		3.571979427	0.000132677	0.000588749
ENSMMUG00000053333	acetylserotonin O-methyltransferase like [Source:NCBI gene;Acc:106995142]	4.769041757	6.88E-05	0.000324217
ENSMMUG00000007514	zinc finger BED-type containing 1 [Source:VGNC Symbol;Acc:VGNC:79956]	-1.094693987	0.009292031	0.025424506
ENSMMUG00000004985	arylsulfatase L [Source:VGNC Symbol;Acc:VGNC:81361]	-2.422731729	6.28E-05	0.000299118
ENSMMUG00000038289	Metazoan signal recognition particle RNA [Source:RFAM;Acc:RF00017]	-1.304446413	0.009013482	0.024758277
ENSMMUG00000000443	arylsulfatase F [Source:VGNC Symbol;Acc:VGNC:81362]	-1.358275255	1.37E-09	1.47E-08
ENSMMUG00000017378	steroid sulfatase [Source:VGNC Symbol;Acc:VGNC:81590]	-1.909327739	0.000273252	0.001128469
ENSMMUG00000008256	amelogenin X-linked [Source:NCBI gene;Acc:710057]	0.4333083	0.016013831	0.040436764

ENSMMUG00000010252	FERM and PDZ domain containing 4 [Source:VGNC Symbol;Acc:VGNC:72802]	-0.663208466	0.00434693	0.013158803
ENSMMUG00000003482	glycine receptor alpha 2 [Source:VGNC Symbol;Acc:VGNC:73075]	1.591752869	3.50E-08	3.00E-07
ENSMMUG00000015737	phosphatidylinositol glycan anchor biosynthesis class A [Source:VGNC Symbol;Acc:VGNC:75984]	-1.035832363	2.50E-08	2.21E-07
ENSMMUG00000015738	vascular endothelial growth factor D [Source:VGNC Symbol;Acc:VGNC:79953]	-0.990547767	0.017158552	0.042867474
ENSMMUG00000015740	pirin [Source:VGNC Symbol;Acc:VGNC:75893]	-0.903892563	0.000200638	0.000855508
ENSMMUG00000004797	CTP synthase 2 [Source:VGNC Symbol;Acc:VGNC:71550]	2.426543449	6.34E-16	1.47E-14
ENSMMUG00000004798	S100 calcium binding protein G [Source:VGNC Symbol;Acc:VGNC:77016]	3.388934325	0.000163004	0.000707655
ENSMMUG00000058700	Metazoan signal recognition particle RNA [Source:RFAM;Acc:RF00017]	4.141688239	8.07E-07	5.57E-06
ENSMMUG00000019363	synapse associated protein 1 [Source:VGNC Symbol;Acc:VGNC:78135]	2.754562912	3.78E-20	1.34E-18
ENSMMUG00000028363	U7 small nuclear RNA [Source:RFAM;Acc:RF00066]	2.87442512	0.006829834	0.019455712
ENSMMUG00000016237	taxilin gamma [Source:NCBI gene;Acc:713769]	3.396831547	2.06E-06	1.32E-05
ENSMMUG00000016238	RB binding protein 7, chromatin remodeling factor [Source:VGNC Symbol;Acc:VGNC:76678]	2.567328525	0.008021114	0.022380644
ENSMMUG00000012894	NHS actin remodeling regulator [Source:VGNC Symbol;Acc:VGNC:75348]	-1.840829973	4.17E-05	0.000205794
ENSMMUG00000020515	adhesion G protein-coupled receptor G2 [Source:VGNC Symbol;Acc:VGNC:69802]	1.864647066	1.61E-06	1.05E-05
ENSMMUG00000004744	SH3 domain containing kinase binding protein 1 [Source:VGNC Symbol;Acc:VGNC:77227]	-0.276216418	0.014245954	0.036576784
ENSMMUG00000002134	ribosomal protein S6 kinase A3 [Source:VGNC Symbol;Acc:VGNC:76929]	-1.037736174	0.002774701	0.008864457
ENSMMUG00000007500	connector enhancer of kinase suppressor of Ras 2 [Source:VGNC Symbol;Acc:VGNC:71223]	-0.824661528	0.000336097	0.001361091
ENSMMUG00000001088	patched domain containing 1 [Source:VGNC Symbol;Acc:VGNC:76322]	1.610582815	8.51E-17	2.15E-15
ENSMMUG00000061261	Metazoan signal recognition particle RNA [Source:RFAM;Acc:RF00017]	-1.425101209	8.77E-05	0.000405623
ENSMMUG00000042317	putative transcription factor SPT20 homolog-like 2 [Source:NCBI gene;Acc:100429227]	-1.139409792	0.008172296	0.022738303
ENSMMUG00000042416		-1.898299113	0.003645783	0.011256915
ENSMMUG00000012819	pyruvate dehydrogenase kinase 3 [Source:VGNC Symbol;Acc:VGNC:75934]	-1.294118112	0.002081721	0.006889517
ENSMMUG00000012821	phosphate cytidylyltransferase 1, choline, beta [Source:VGNC Symbol;Acc:VGNC:75790]	-2.378928577	0.002106665	0.006964297
ENSMMUG00000005959	glycerol kinase [Source:NCBI gene;Acc:707531]	0.844943344	1.55E-08	1.41E-07
ENSMMUG00000005263	chromosome X CXorf38 homolog [Source:VGNC Symbol;Acc:VGNC:71600]	1.402490888	0.003580195	0.011084403
ENSMMUG00000005264	mediator complex subunit 14 [Source:VGNC Symbol;Acc:VGNC:74561]	0.476577802	0.008347601	0.023195601
ENSMMUG00000063721		-0.888297403	8.26E-09	7.83E-08
ENSMMUG00000055907		0.93228238	8.51E-08	6.92E-07
ENSMMUG00000057909		2.149798329	1.18E-08	1.10E-07
ENSMMUG00000057804		-1.965433152	0.000457599	0.001794816
ENSMMUG00000016391	EF-hand domain containing 2 [Source:VGNC Symbol;Acc:VGNC:72116]	3.152766834	0.019192954	0.047124422
ENSMMUG00000003505	FUN14 domain containing 1 [Source:NCBI gene;Acc:709370]	2.438194984	0.000448791	0.001764
ENSMMUG00000003897	solute carrier family 9 member A7 [Source:NCBI gene;Acc:710906]	-0.75496538	1.38E-05	7.57E-05
ENSMMUG00000049666		1.065932116	1.14E-08	1.06E-07
ENSMMUG00000023018	RP2 activator of ARL3 GTPase [Source:VGNC Symbol;Acc:VGNC:76720]	0.911035084	0.000688176	0.00257914
ENSMMUG00000023016	jade family PHD finger 3 [Source:VGNC Symbol;Acc:VGNC:73640]	1.850470637	5.06E-27	3.85E-25
ENSMMUG00000019546	regucalcin [Source:VGNC Symbol;Acc:VGNC:76912]	2.149339247	6.04E-07	4.27E-06
ENSMMUG00000017423	RNA binding motif protein 10 [Source:VGNC Symbol;Acc:VGNC:76680]	-1.374744573	0.016908064	0.042391646

ENSMMUG00000014002	cyclin dependent kinase 16 [Source:VGNC Symbol;Acc:VGNC:71160]	-1.35536201	0.000384501	0.001536553
ENSMMUG00000016608	ubiquitin specific peptidase 11 [Source:VGNC Symbol;Acc:VGNC:78734]	-1.41340256	0.011780365	0.031189835
ENSMMUG00000008566	zinc finger protein 157 [Source:VGNC Symbol;Acc:VGNC:78850]	1.229631131	2.35E-12	3.70E-11
ENSMMUG00000010404	zinc finger protein 41 [Source:VGNC Symbol;Acc:VGNC:78900]	-1.035874633	2.63E-05	0.000135998
ENSMMUG00000014066	zinc finger protein 81 [Source:VGNC Symbol;Acc:VGNC:78908]	-1.028178465	0.007478455	0.021036701
ENSMMUG00000056352	putative protein SSX9 [Source:NCBI gene;Acc:709251]	-1.990686966	0.012330258	0.032438838
ENSMMUG00000018894	FtsJ RNA 2'-O-methyltransferase 1 [Source:VGNC Symbol;Acc:VGNC:72716]	-1.787353956	0.009623823	0.026172986
ENSMMUG00000019768	suppressor of variegation 3-9 homolog 1 [Source:VGNC Symbol;Acc:VGNC:78061]	-0.912153893	6.86E-05	0.000323485
ENSMMUG00000063882	U6 spliceosomal RNA [Source:RFAM;Acc:RF00026]	-2.644375971	0.019684434	0.048195503
ENSMMUG00000016465	histone deacetylase 6 [Source:VGNC Symbol;Acc:VGNC:73164]	1.055922332	8.87E-21	3.43E-19
ENSMMUG00000060865	Metazoan signal recognition particle RNA [Source:RFAM;Acc:RF00017]	4.216804244	0.000114834	0.000515899
ENSMMUG00000016469	proprotein convertase subtilisin/kexin type 1 inhibitor [Source:VGNC Symbol;Acc:VGNC:75639]	2.560170682	7.74E-06	4.44E-05
ENSMMUG00000053345	Pim-2 proto-oncogene, serine/threonine kinase [Source:NCBI gene;Acc:711844]	1.048982342	0.000627832	0.00237883
ENSMMUG00000004558	OTU deubiquitinase 5 [Source:VGNC Symbol;Acc:VGNC:75677]	1.531373193	2.50E-22	1.16E-20
ENSMMUG00000010617	coiled-coil domain containing 120 [Source:VGNC Symbol;Acc:VGNC:70664]	0.610953444	0.000414656	0.001645971
ENSMMUG00000055688	PRA1 domain family member 2 [Source:HGNC Symbol;Acc:HGNC:28911]	0.561152438	0.014519239	0.037220451
ENSMMUG00000023024	prickle planar cell polarity protein 3 [Source:VGNC Symbol;Acc:VGNC:76361]	0.700719311	0.001124368	0.004002493
ENSMMUG00000023025	synaptophysin [Source:VGNC Symbol;Acc:VGNC:78072]	0.968671997	6.86E-10	7.74E-09
ENSMMUG00000008618	calcium voltage-gated channel subunit alpha1 F [Source:VGNC Symbol;Acc:VGNC:70503]	1.164417631	1.70E-21	7.13E-20
ENSMMUG00000008623	coiled-coil domain containing 22 [Source:VGNC Symbol;Acc:VGNC:70899]	1.501864862	2.72E-23	1.40E-21
ENSMMUG00000008624	forkhead box P3 [Source:VGNC Symbol;Acc:VGNC:72926]	1.705996208	0.000500004	0.001945189
ENSMMUG00000052252	protein phosphatase 1 regulatory subunit 3F [Source:NCBI gene;Acc:715950]	2.202267896	1.94E-31	2.06E-29
ENSMMUG00000060311	G antigen 10-like [Source:NCBI gene;Acc:114674954]	0.524767017	0.00174085	0.005886185
ENSMMUG00000041376	G antigen 10 [Source:NCBI gene;Acc:693330]	1.626613503	1.23E-15	2.75E-14
ENSMMUG00000026584	Y RNA [Source:RFAM;Acc:RF00019]	2.387313289	0.000194704	0.00083212
ENSMMUG00000056042	Y RNA [Source:RFAM;Acc:RF00019]	2.79178904	0.017415277	0.043399186
ENSMMUG00000023060	A-kinase anchoring protein 4 [Source:VGNC Symbol;Acc:VGNC:69770]	2.356642619	1.21E-09	1.31E-08
ENSMMUG00000045093	cyclin B3 [Source:NCBI gene;Acc:694534]	2.342114338	6.25E-24	3.42E-22
ENSMMUG00000049267		3.565611725	1.13E-16	2.82E-15
ENSMMUG00000050420	centromere protein V-like protein 1 [Source:NCBI gene;Acc:717718]	-1.235973137	0.007375871	0.02076783
ENSMMUG00000020202	melanoma-associated antigen D4 [Source:NCBI gene;Acc:106996411]	-1.95598819	2.03E-06	1.30E-05
ENSMMUG00000062319		-2.763752502	0.015706789	0.039769793
ENSMMUG00000012073	X antigen family member 1-like [Source:NCBI gene;Acc:106996044]	-0.821044286	7.40E-06	4.27E-05
ENSMMUG00000046218	X antigen family member 5 [Source:NCBI gene;Acc:114669729]	-1.828861991	3.49E-06	2.13E-05
ENSMMUG00000003251	X antigen family member 3 [Source:NCBI gene;Acc:698267]	0.67249885	0.003311499	0.010354106
ENSMMUG00000053262		-0.471859634	0.014758741	0.037743081
ENSMMUG00000020553	lysine demethylase 5C [Source:VGNC Symbol;Acc:VGNC:73816]	-0.409384877	0.013668507	0.035410065
ENSMMUG00000020555	IQ motif and Sec7 domain ArfGEF 2 [Source:VGNC Symbol;Acc:VGNC:73676]	-0.471542387	0.000611548	0.002321878
ENSMMUG00000050146		-0.966619131	0.017863143	0.044366172

ENSMMUG00000047741	Metazoan signal recognition particle RNA [Source:RFAM;Acc:RF00017]	-1.051317223	2.69E-07	2.01E-06
ENSMMUG00000005433	WNK lysine deficient protein kinase 3 [Source:VGNC Symbol;Acc:VGNC:78962]	-0.512487105	0.003486795	0.010825068
ENSMMUG00000008422	trophinin [Source:NCBI gene;Acc:704512]	-0.635999323	0.000745359	0.002761347
ENSMMUG00000059922	family with sequence similarity 104 member B [Source:NCBI gene;Acc:705418]	-2.579683512	2.74E-05	0.000141456
ENSMMUG00000051670		-2.515824419	3.78E-12	5.77E-11
ENSMMUG00000054377		1.313699932	3.21E-23	1.64E-21
ENSMMUG00000063913	endogenous retrovirus group K member 7 Env polyprotein-like [Source:NCBI gene;Acc:114674933]	4.257924905	3.83E-09	3.84E-08
ENSMMUG00000059335		4.823618504	6.07E-07	4.29E-06
ENSMMUG00000004309	APC membrane recruitment protein 1 [Source:VGNC Symbol;Acc:VGNC:69716]	5.200083771	1.93E-07	1.48E-06
ENSMMUG00000065109	ankyrin repeat and SOCS box containing 12 [Source:NCBI gene;Acc:707931]	3.658302181	0.000808568	0.002971763
ENSMMUG00000065001		-0.362726284	0.007289326	0.020563192
ENSMMUG00000061393	OTU deubiquitinase 6A [Source:NCBI gene;Acc:694498]	-1.543983078	0.013983147	0.036070633
ENSMMUG00000054817	immunoglobulin binding protein 1 [Source:NCBI gene;Acc:694632]	-1.371655528	0.001500853	0.005161791
ENSMMUG00000013934	pyrimidinergic receptor P2Y4 [Source:VGNC Symbol;Acc:VGNC:75739]	-1.651783147	0.001518991	0.005213292
ENSMMUG00000013935	arrestin 3 [Source:VGNC Symbol;Acc:VGNC:70020]	-1.354010329	0.005431329	0.0159372
ENSMMUG00000013937	RAB41, member RAS oncogene family [Source:VGNC Symbol;Acc:VGNC:81544]	-2.1418887	2.95E-07	2.18E-06
ENSMMUG00000061295	PDZ domain containing 11 [Source:NCBI gene;Acc:695458]	-1.389390558	1.50E-07	1.18E-06
ENSMMUG00000013940	kinesin family member 4A [Source:NCBI gene;Acc:695574]	-1.619831689	3.36E-05	0.000169761
ENSMMUG00000013943	glycerophosphodiester phosphodiesterase domain containing 2 [Source:VGNC Symbol;Acc:VGNC:72787]	-2.267224104	1.17E-07	9.36E-07
ENSMMUG00000003049	testis expressed 11 [Source:VGNC Symbol;Acc:VGNC:78316]	-3.35608044	3.73E-09	3.74E-08
ENSMMUG00000032429	forkhead box O4 [Source:VGNC Symbol;Acc:VGNC:72712]	1.96555259	3.28E-21	1.34E-19
ENSMMUG00000032427	neuroligin 3 [Source:NCBI gene;Acc:574216]	-1.937208462	3.59E-14	6.96E-13
ENSMMUG00000000669	gap junction protein beta 1 [Source:VGNC Symbol;Acc:VGNC:73059]	-2.497605949	6.08E-06	3.56E-05
ENSMMUG00000000667	zinc finger MYM-type containing 3 [Source:VGNC Symbol;Acc:VGNC:78886]	-2.241133731	1.03E-10	1.30E-09
ENSMMUG00000056450		-1.735622955	0.017105985	0.042773913
ENSMMUG00000015238	O-linked N-acetylglucosamine (GlcNAc) transferase [Source:NCBI gene;Acc:698268]	-2.166191131	6.31E-29	5.64E-27
ENSMMUG00000011469	germ cell nuclear acidic peptidase [Source:VGNC Symbol;Acc:VGNC:72724]	-2.794269414	2.28E-05	0.00011974
ENSMMUG00000021924	NHS like 2 [Source:NCBI gene;Acc:702808]	0.737073415	8.71E-12	1.26E-10
ENSMMUG00000055786	peptidylprolyl cis/trans isomerase, NIMA-interacting 4 [Source:NCBI gene;Acc:699273]	-3.295448432	0.004337471	0.013135532
ENSMMUG00000057710	DMRT like family C1B [Source:NCBI gene;Acc:704619]	-1.55475554	0.010333478	0.027842565
ENSMMUG00000054132		-2.014622239	0.014060991	0.036221036
ENSMMUG00000063858		-0.391349239	0.005202726	0.01533925
ENSMMUG00000009249	ring finger protein, LIM domain interacting [Source:NCBI gene;Acc:703471]	0.791618972	1.13E-10	1.42E-09

ENSMMUG00000053505	U6 spliceosomal RNA [Source:RFAM;Acc:RF00026]	1.256246425	0.000789416	0.002912924
ENSMMUG00000017660		1.900746347	1.25E-20	4.76E-19
ENSMMUG00000011440	neurite extension and migration factor [Source:VGNC Symbol;Acc:VGNC:75274]	1.896736451	3.97E-10	4.61E-09
ENSMMUG00000015619	ATP binding cassette subfamily B member 7 [Source:VGNC Symbol;Acc:VGNC:81340]	-1.628163822	3.37E-11	4.52E-10
ENSMMUG00000049351	MAGE family member E1 [Source:NCBI gene;Acc:705379]	-2.010754376	2.18E-14	4.33E-13
ENSMMUG00000014407	magnesium transporter 1 [Source:VGNC Symbol;Acc:VGNC:74386]	1.167777072	4.48E-12	6.75E-11
ENSMMUG00000027544	U6 spliceosomal RNA [Source:RFAM;Acc:RF00026]	0.989378556	0.004997175	0.014845373
ENSMMUG00000015861	cytochrome c oxidase subunit VIIb [Source:NCBI gene;Acc:706099]	1.340433876	5.38E-13	9.20E-12
ENSMMUG00000013387	cysteinyl leukotriene receptor 1 [Source:VGNC Symbol;Acc:VGNC:71646]	1.704322402	8.08E-14	1.52E-12
ENSMMUG00000052073		1.535689663	2.58E-13	4.53E-12
ENSMMUG00000054447	T-box transcription factor 22 [Source:NCBI gene;Acc:708338]	-1.15658465	0.003316286	0.010366445
ENSMMUG00000008212	tenomodulin [Source:VGNC Symbol;Acc:VGNC:79087]	-1.372069831	0.000235325	0.00098865
ENSMMUG00000008210	sushi repeat containing protein X-linked 2 [Source:NCBI gene;Acc:700756]	-0.614966919	0.01494221	0.038146491
ENSMMUG00000013008	cleavage stimulation factor subunit 2 [Source:VGNC Symbol;Acc:VGNC:71536]	-1.926237053	5.24E-07	3.73E-06
ENSMMUG00000004178	NADPH oxidase 1 [Source:VGNC Symbol;Acc:VGNC:75374]	-2.414045542	3.53E-11	4.74E-10
ENSMMUG00000065018	transmembrane protein 35A [Source:NCBI gene;Acc:702205]	-1.245717187	5.74E-12	8.52E-11
ENSMMUG00000007601	centromere protein I [Source:NCBI gene;Acc:702316]	-0.679201801	0.005422294	0.015913837
ENSMMUG00000022281	family with sequence similarity 199, X-linked [Source:NCBI gene;Acc:699523]	2.177869335	0.000897582	0.003257742
ENSMMUG00000006684	testis expressed 13A [Source:VGNC Symbol;Acc:VGNC:78317]	2.226163201	0.007955615	0.02221879
ENSMMUG00000013150	MORC family CW-type zinc finger 4 [Source:VGNC Symbol;Acc:VGNC:74808]	1.666698707	1.64E-23	8.76E-22
ENSMMUG00000013146	claudin 2 [Source:HGNC Symbol;Acc:HGNC:2041]	1.718138443	8.47E-22	3.73E-20
ENSMMUG00000022293	FERM and PDZ domain containing 3 [Source:VGNC Symbol;Acc:VGNC:72805]	-0.918203458	1.42E-09	1.53E-08
ENSMMUG00000004674	phosphoribosyl pyrophosphate synthetase 1 [Source:NCBI gene;Acc:702529]	-1.340124411	7.26E-19	2.24E-17
ENSMMUG00000007688	angiomotin [Source:VGNC Symbol;Acc:VGNC:69627]	4.486819724	7.78E-06	4.45E-05
ENSMMUG00000006377	plastin 3 [Source:VGNC Symbol;Acc:VGNC:76181]	0.902128869	0.000776918	0.002871092
ENSMMUG00000055906	angiotensin II receptor type 2 [Source:NCBI gene;Acc:709513]	2.157222867	8.25E-07	5.68E-06
ENSMMUG00000026243	U6 spliceosomal RNA [Source:RFAM;Acc:RF00026]	2.650604455	0.000468307	0.001832936
ENSMMUG00000057063	solute carrier family 6 member 14 [Source:NCBI gene;Acc:709598]	2.189764382	0.000286329	0.001174424
ENSMMUG00000008818	SWI/SNF related, matrix associated, actin dependent regulator of chromatin, subfamily a, member 1 [Source:VGNC Symbol;Acc:VGNC:77646]	-0.983997875	0.009119372	0.025002825
ENSMMUG00000059153		-0.814057646	0.001320086	0.004612836
ENSMMUG00000020700	UTP14A small subunit processome component [Source:NCBI gene;Acc:703510]	0.859887539	0.00815852	0.022721286
ENSMMUG00000008781	BCL6 corepressor like 1 [Source:VGNC Symbol;Acc:VGNC:70131]	-1.070373957	1.44E-06	9.51E-06
ENSMMUG00000063900	Metazoan signal recognition particle RNA [Source:RFAM;Acc:RF00017]	-3.197923633	0.002933698	0.009320211
ENSMMUG00000004954	apoptosis inducing factor mitochondria associated 1 [Source:VGNC Symbol;Acc:VGNC:69820]	-1.243844433	1.58E-09	1.68E-08
ENSMMUG00000049309	RAB33A, member RAS oncogene family [Source:NCBI gene;Acc:703002]	-1.412053249	0.002116219	0.006984984
ENSMMUG00000003479	FERM domain containing 7 [Source:VGNC Symbol;Acc:VGNC:72927]	-0.89988654	0.006666192	0.019044429
ENSMMUG00000025616		-2.33338387	0.006926091	0.019699643

ENSMMUG00000003456	glypican 4 [Source:VGNC Symbol;Acc:VGNC:73118]	-0.424353019	0.01147689	0.030480352
ENSMMUG00000032341	coiled-coil domain containing 160 [Source:VGNC Symbol;Acc:VGNC:70926]	0.632029926	3.43E-07	2.51E-06
ENSMMUG00000059560		0.771743874	0.002363715	0.007695697
ENSMMUG00000000466	AF4/FMR2 family member 2 [Source:VGNC Symbol;Acc:VGNC:69773]	-0.582952591	1.76E-05	9.50E-05
ENSMMUG00000022371	myotubularin 1 [Source:VGNC Symbol;Acc:VGNC:75071]	-2.39389413	0.014413181	0.03696774
ENSMMUG00000016610	CD99 molecule like 2 [Source:VGNC Symbol;Acc:VGNC:70823]	-1.939368744	8.59E-10	9.50E-09
ENSMMUG00000016929	proline rich and Gla domain 3 [Source:VGNC Symbol;Acc:VGNC:76350]	2.256459409	1.47E-08	1.34E-07
ENSMMUG00000063694	cyclic nucleotide gated channel subunit alpha 2 [Source:NCBI gene;Acc:704486]	1.21623626	1.28E-18	3.89E-17
ENSMMUG00000062628		1.483123193	1.96E-08	1.76E-07
ENSMMUG00000057473		0.782218645	0.002004602	0.006671509
ENSMMUG00000040274	MAGE family member A4 [Source:NCBI gene;Acc:710825]	1.489533838	5.59E-19	1.73E-17
ENSMMUG00000039292		1.959455501	2.37E-17	6.41E-16
ENSMMUG00000062063	melanoma-associated antigen 3 [Source:NCBI gene;Acc:711544]	2.207126861	1.16E-35	2.04E-33
ENSMMUG00000037641	chondrosarcoma-associated gene 2/3 protein [Source:NCBI gene;Acc:106992305]	2.278508067	0.000549134	0.002115786
ENSMMUG00000058013	melanoma-associated antigen 3 [Source:NCBI gene;Acc:706103]	2.081526023	1.45E-14	2.97E-13
ENSMMUG00000040071		2.230997416	3.51E-06	2.14E-05
ENSMMUG00000020743	centrin 2 [Source:NCBI gene;Acc:693376]	1.479263735	0.01425633	0.036597086
ENSMMUG00000013176	NAD(P) dependent steroid dehydrogenase-like [Source:NCBI gene;Acc:693333]	2.468756761	5.97E-12	8.83E-11
ENSMMUG00000010897	PNMA family member 3 [Source:VGNC Symbol;Acc:VGNC:76063]	0.835810716	6.67E-07	4.68E-06
ENSMMUG00000064249		-1.283037297	0.015903895	0.04022766
ENSMMUG00000041072		-1.160991431	0.00359381	0.011117271
ENSMMUG00000061278		-1.321451237	1.12E-06	7.54E-06
ENSMMUG00000010137	microcephalin 1 [Source:VGNC Symbol;Acc:VGNC:74687]	2.545606303	1.42E-06	9.37E-06
ENSMMUG00000010139	angiopoietin 2 [Source:VGNC Symbol;Acc:VGNC:69811]	3.328935636	0.005443228	0.015965796
ENSMMUG00000053229	defensin beta 1 [Source:NCBI gene;Acc:574187]	-2.829665234	0.013014921	0.033921137
ENSMMUG00000058176	b-defensin2-like [Source:NCBI gene;Acc:100169969]	2.914260578	6.76E-29	5.97E-27
ENSMMUG00000063525		2.884966585	1.58E-16	3.88E-15
ENSMMUG00000061302		2.370376847	0.006853574	0.019512095
ENSMMUG00000054960		2.229506364	7.73E-13	1.30E-11
ENSMMUG00000027220	microRNA 598 [Source:HGNC Symbol;Acc:HGNC:32854]	2.577124616	0.007116394	0.02015202
ENSMMUG00000010180	PIN2 (TERF1) interacting telomerase inhibitor 1 [Source:NCBI gene;Acc:701523]	2.413329962	9.70E-14	1.81E-12
ENSMMUG00000008867	exoribonuclease 1 [Source:VGNC Symbol;Acc:VGNC:72324]	-2.47219715	2.33E-05	0.000121864
ENSMMUG00000055078	Small nucleolar RNA U3 [Source:RFAM;Acc:RF00012]	-2.802239545	0.014033382	0.036156194
ENSMMUG00000007068	tumor suppressor candidate 3 [Source:VGNC Symbol;Acc:VGNC:84132]	3.802132223	3.51E-07	2.56E-06
ENSMMUG00000001873	CCR4-NOT transcription complex subunit 7 [Source:VGNC Symbol;Acc:VGNC:83990]	2.521453715	0.016220941	0.040917938
ENSMMUG00000022303	solute carrier family 7 member 2 [Source:VGNC Symbol;Acc:VGNC:77629]	-0.762062462	4.25E-09	4.22E-08
ENSMMUG00000001151	microtubule associated scaffold protein 1 [Source:VGNC Symbol;Acc:VGNC:84018]	2.03632505	4.11E-28	3.38E-26
ENSMMUG00000005920	N-acetyltransferase 1 [Source:VGNC Symbol;Acc:VGNC:75133]	3.029765914	5.57E-06	3.28E-05
ENSMMUG00000011774	integrator complex subunit 10 [Source:VGNC Symbol;Acc:VGNC:73653]	1.440869402	5.84E-34	8.90E-32
ENSMMUG00000023361	GDNF family receptor alpha 2 [Source:VGNC Symbol;Acc:VGNC:72918]	0.862634016	0.00045174	0.001774652
ENSMMUG00000002032	exportin 7 [Source:VGNC Symbol;Acc:VGNC:78946]	-1.213808068	8.51E-06	4.84E-05
ENSMMUG00000063764		-1.831270723	0.000457315	0.001794176

ENSMMUG00000013028	leucine rich repeat LGI family member 3 [Source:VGNC Symbol;Acc:VGNC:74079]	-1.261838021	0.018193753	0.045063964
ENSMMUG00000013029	surfactant protein C [Source:VGNC Symbol;Acc:VGNC:84113]	-2.415544552	2.30E-11	3.14E-10
ENSMMUG00000013031	bone morphogenetic protein 1 [Source:VGNC Symbol;Acc:VGNC:70259]	-1.115662245	1.35E-08	1.24E-07
ENSMMUG00000013032	phytanoyl-CoA 2-hydroxylase interacting protein [Source:VGNC Symbol;Acc:VGNC:75812]	-1.328676588	2.32E-09	2.40E-08
ENSMMUG00000023226	RNA polymerase III subunit D [Source:VGNC Symbol;Acc:VGNC:76017]	-1.119632292	3.81E-12	5.80E-11
ENSMMUG00000052651	Metazoan signal recognition particle RNA [Source:RFAM;Acc:RF00017]	-1.319919042	1.51E-08	1.37E-07
ENSMMUG00000014438	piwi like RNA-mediated gene silencing 2 [Source:VGNC Symbol;Acc:VGNC:81502]	-1.169667145	5.89E-14	1.13E-12
ENSMMUG00000017713	solute carrier family 39 member 14 [Source:VGNC Symbol;Acc:VGNC:77537]	0.696216251	0.019629711	0.048073672
ENSMMUG00000018345	phosphatidylethanolamine binding protein 4 [Source:VGNC Symbol;Acc:VGNC:82204]	2.457886641	2.68E-26	1.84E-24
ENSMMUG00000050868		1.568297221	0.001422538	0.004924345
ENSMMUG00000007540	Rho related BTB domain containing 2 [Source:VGNC Symbol;Acc:VGNC:76772]	1.265134949	2.51E-07	1.89E-06
ENSMMUG00000048076	TNF receptor superfamily member 10b [Source:NCBI gene;Acc:716650]	1.509806681	1.47E-14	3.01E-13
ENSMMUG00000016655	R3H domain and coiled-coil containing 1 [Source:VGNC Symbol;Acc:VGNC:76551]	1.526574689	1.28E-07	1.02E-06
ENSMMUG00000056749	Metazoan signal recognition particle RNA [Source:RFAM;Acc:RF00017]	3.445296967	1.76E-06	1.15E-05
ENSMMUG00000050549	lysyl oxidase like 2 [Source:NCBI gene;Acc:710151]	2.633165623	3.28E-27	2.55E-25
ENSMMUG00000046186	stanniocalcin 1 [Source:VGNC Symbol;Acc:VGNC:84120]	2.948967302	0.000160048	0.000695637
ENSMMUG00000003348	neurofilament light [Source:VGNC Symbol;Acc:VGNC:75307]	2.122213072	1.73E-23	9.18E-22
ENSMMUG00000056605	Metazoan signal recognition particle RNA [Source:RFAM;Acc:RF00017]	2.46873202	1.81E-05	9.70E-05
ENSMMUG00000008028	dedicator of cytokinesis 5 [Source:VGNC Symbol;Acc:VGNC:83995]	0.725653372	0.00146784	0.005058812
ENSMMUG00000059181		1.163211778	3.44E-05	0.000173236
ENSMMUG00000016019	PDZ binding kinase [Source:VGNC Symbol;Acc:VGNC:75643]	-2.306239829	0.005585978	0.016329599
ENSMMUG00000022044	scavenger receptor class A member 5 [Source:VGNC Symbol;Acc:VGNC:77117]	-1.130650258	8.86E-14	1.66E-12
ENSMMUG00000005067	elongator acetyltransferase complex subunit 3 [Source:VGNC Symbol;Acc:VGNC:72080]	-2.034170302	2.01E-10	2.45E-09
ENSMMUG00000007480	F-box protein 16 [Source:NCBI gene;Acc:106999728]	0.924638084	7.59E-05	0.00035471
ENSMMUG00000017700	exostosin like glycosyltransferase 3 [Source:VGNC Symbol;Acc:VGNC:72452]	-0.780406482	0.00066074	0.002489526
ENSMMUG00000014981	homeobox containing 1 [Source:VGNC Symbol;Acc:VGNC:73443]	0.915050198	5.48E-07	3.89E-06
ENSMMUG00000039515	kinesin family member 13B [Source:NCBI gene;Acc:100429368]	-1.746615272	0.000324452	0.001318615
ENSMMUG00000000458	RNA binding protein, mRNA processing factor [Source:VGNC Symbol;Acc:VGNC:76510]	0.811024236	0.00023604	0.000991075
ENSMMUG00000064702		-1.784235741	0.000839417	0.003072957
ENSMMUG00000020819	protein phosphatase 2 catalytic subunit beta [Source:VGNC Symbol;Acc:VGNC:76373]	-1.557958483	0.000530271	0.002048427
ENSMMUG00000061408		-2.850041195	0.000101775	0.000464595
ENSMMUG00000063345		-3.228311217	8.71E-07	5.97E-06
ENSMMUG00000010638	unc-5 netrin receptor D [Source:VGNC Symbol;Acc:VGNC:79158]	2.183316922	0.018450681	0.045603932
ENSMMUG00000034978	U6 spliceosomal RNA [Source:RFAM;Acc:RF00026]	2.340952609	0.003517572	0.01090874
ENSMMUG00000001635	zinc finger protein 703 [Source:VGNC Symbol;Acc:VGNC:79552]	1.00643866	5.06E-05	0.00024581
ENSMMUG00000051162		-1.946199032	0.018006792	0.044696488
ENSMMUG00000003591	pleckstrin homology domain containing A2 [Source:VGNC Symbol;Acc:VGNC:76026]	2.116374316	9.27E-10	1.02E-08

ENSMMUG00000006037	HtrA serine peptidase 4 [Source:VGNC Symbol;Acc:VGNC:73447]	3.14995069	0.006952006	0.01976198
ENSMMUG00000019304	indoleamine 2,3-dioxygenase 1 [Source:VGNC Symbol;Acc:VGNC:73580]	3.113225682	6.58E-21	2.56E-19
ENSMMUG00000000346	succinate dehydrogenase cytochrome b560 subunit, mitochondrial [Source:NCBI gene;Acc:702309]	2.63972011	0.005473215	0.016041063
ENSMMUG00000019306	indoleamine 2,3-dioxygenase 2 [Source:VGNC Symbol;Acc:VGNC:84005]	3.349937185	4.83E-07	3.46E-06
ENSMMUG00000000776	zinc finger matrin-type 4 [Source:VGNC Symbol;Acc:VGNC:78424]	1.182248663	6.43E-08	5.34E-07
ENSMMUG00000019082	secreted frizzled related protein 1 [Source:VGNC Symbol;Acc:VGNC:77185]	-0.325957988	0.018531162	0.045749482
ENSMMUG00000014721	glycerol-3-phosphate acyltransferase 4 [Source:VGNC Symbol;Acc:VGNC:84002]	-1.113687713	9.36E-05	0.000429893
ENSMMUG00000010891	lysine acetyltransferase 6A [Source:VGNC Symbol;Acc:VGNC:73821]	-1.284748174	1.91E-06	1.23E-05
ENSMMUG00000014431	adaptor related protein complex 3 subunit mu 2 [Source:VGNC Symbol;Acc:VGNC:69971]	0.525646228	5.63E-05	0.000270409
ENSMMUG00000014432	plasminogen activator, tissue type [Source:VGNC Symbol;Acc:VGNC:76161]	0.722313199	8.14E-09	7.73E-08
ENSMMUG00000020185	voltage dependent anion channel 3 [Source:VGNC Symbol;Acc:VGNC:84135]	1.912323561	5.09E-06	3.02E-05
ENSMMUG00000020186	solute carrier family 20 member 2 [Source:VGNC Symbol;Acc:VGNC:77416]	1.335218714	0.000427403	0.001690237
ENSMMUG00000053488	small integral membrane protein 19 [Source:NCBI gene;Acc:705371]	0.930581885	5.29E-09	5.17E-08
ENSMMUG00000053422		0.523044333	0.000153124	0.000668481
ENSMMUG00000062518		1.724961188	0.00038931	0.001554515
ENSMMUG00000002781	peroxidasin like [Source:VGNC Symbol;Acc:VGNC:84107]	-0.400466959	0.006234778	0.017978175
ENSMMUG00000065247	opioid receptor kappa 1 [Source:NCBI gene;Acc:710783]	2.199896339	8.56E-26	5.45E-24
ENSMMUG00000025039	Small nucleolar RNA SNORA73 family [Source:RFAM;Acc:RF00045]	3.649110687	0.001164069	0.004127965
ENSMMUG00000005478	trimethylguanosine synthase 1 [Source:VGNC Symbol;Acc:VGNC:78404]	1.393445485	2.20E-07	1.67E-06
ENSMMUG00000014149	LYN proto-oncogene, Src family tyrosine kinase [Source:VGNC Symbol;Acc:VGNC:74464]	1.340334778	1.37E-24	7.91E-23
ENSMMUG00000055121	Metazoan signal recognition particle RNA [Source:RFAM;Acc:RF00017]	1.761486832	3.65E-18	1.07E-16
ENSMMUG00000039862	Metazoan signal recognition particle RNA [Source:RFAM;Acc:RF00017]	-1.788471508	0.001131854	0.00402624
ENSMMUG00000061268	PLAG1 zinc finger [Source:NCBI gene;Acc:696149]	-2.135135795	3.39E-12	5.22E-11
ENSMMUG00000046464	coiled-coil-helix-coiled-coil-helix domain containing 7 [Source:VGNC Symbol;Acc:VGNC:83989]	-2.394198805	2.04E-15	4.49E-14
ENSMMUG00000061068		-1.743390501	6.41E-09	6.19E-08
ENSMMUG00000014810	short chain dehydrogenase/reductase family 16C member 5 [Source:VGNC Symbol;Acc:VGNC:77343]	-1.899942992	1.49E-19	4.92E-18
ENSMMUG00000052791		-2.476864214	6.29E-21	2.47E-19
ENSMMUG00000006775	proenkephalin [Source:VGNC Symbol;Acc:VGNC:75941]	-2.222454651	1.50E-14	3.08E-13
ENSMMUG00000006543	cytochrome P450, family 7, subfamily A, polypeptide 1 [Source:NCBI gene;Acc:698127]	-3.569834334	0.003399746	0.010593475
ENSMMUG00000017038	RAB2A, member RAS oncogene family [Source:VGNC Symbol;Acc:VGNC:81529]	0.547594668	0.000636096	0.002407684
ENSMMUG00000006729	YTH N6-methyladenosine RNA binding protein 3 [Source:VGNC Symbol;Acc:VGNC:79614]	-1.208221884	0.001963054	0.0065494
ENSMMUG00000059575		-2.465484538	1.65E-26	1.18E-24
ENSMMUG00000038661		-2.779353832	2.42E-23	1.26E-21
ENSMMUG00000049866		0.801725021	1.89E-05	0.000100903
ENSMMUG00000014424	ADP ribosylation factor guanine nucleotide exchange factor 1 [Source:VGNC Symbol;Acc:VGNC:69834]	-1.604566552	0.004698878	0.014089033
ENSMMUG00000008783	solute carrier organic anion transporter family member 5A1 [Source:VGNC Symbol;Acc:VGNC:77736]	-2.121111982	0.017106681	0.042773913
ENSMMUG00000012513	lactamase beta 2 [Source:VGNC Symbol;Acc:VGNC:74072]	-1.242748664	0.000303515	0.001239296

ENSMMUG00000045333	Metazoan signal recognition particle RNA [Source:RFAM;Acc:RF00017]	-0.620442427	0.001888772	0.006332917
ENSMMUG00000007576	EYA transcriptional coactivator and phosphatase 1 [Source:VGNC Symbol;Acc:VGNC:72453]	-0.741682462	3.92E-11	5.23E-10
ENSMMUG00000010748	transient receptor potential cation channel subfamily A member 1 [Source:VGNC Symbol;Acc:VGNC:78429]	-1.04812775	0.000218441	0.000923457
ENSMMUG00000053156		-1.339266553	0.000396149	0.001580122
ENSMMUG00000019414	zinc finger homeobox 4 [Source:VGNC Symbol;Acc:VGNC:79902]	-0.836426591	0.012777265	0.033407412
ENSMMUG00000060062		-2.073620025	0.019499822	0.047798738
ENSMMUG00000064054		2.303012556	0.000781819	0.002887765
ENSMMUG00000000507	stathmin 2 [Source:VGNC Symbol;Acc:VGNC:78038]	-1.030138721	0.003165418	0.00995613
ENSMMUG00000007115	RALY RNA binding protein like [Source:VGNC Symbol;Acc:VGNC:82217]	-1.097673735	0.001974577	0.006583404
ENSMMUG00000028699		-1.555585512	5.51E-05	0.00026523
ENSMMUG00000063555		-1.994370865	8.23E-05	0.00038228
ENSMMUG00000028701		-1.557626047	1.81E-08	1.63E-07
ENSMMUG00000013193	matrix metallopeptidase 16 [Source:VGNC Symbol;Acc:VGNC:74894]	0.898961533	3.15E-08	2.73E-07
ENSMMUG00000020010	cadherin 17 [Source:VGNC Symbol;Acc:VGNC:70810]	3.589905208	0.016110971	0.040661282
ENSMMUG00000021112	epithelial splicing regulatory protein 1 [Source:VGNC Symbol;Acc:VGNC:72358]	-1.066515329	4.72E-08	3.98E-07
ENSMMUG00000005356	integrator complex subunit 8 [Source:VGNC Symbol;Acc:VGNC:73657]	2.47025133	7.57E-06	4.36E-05
ENSMMUG00000057219	tumor protein p53 inducible nuclear protein 1 [Source:NCBI gene;Acc:700632]	3.861751662	7.39E-11	9.52E-10
ENSMMUG00000003878	carboxypeptidase Q [Source:VGNC Symbol;Acc:VGNC:71426]	1.187700143	0.002814456	0.008975996
ENSMMUG00000055454		0.634598104	3.77E-07	2.74E-06
ENSMMUG00000060329		0.65390074	0.000106512	0.000483493
ENSMMUG00000061917		0.679880055	0.000148796	0.000651698
ENSMMUG00000014210	metadherin [Source:VGNC Symbol;Acc:VGNC:74859]	0.618971173	9.25E-05	0.000425634
ENSMMUG00000008926	lysosomal protein transmembrane 4 beta [Source:VGNC Symbol;Acc:VGNC:84010]	0.585988408	6.11E-05	0.000291701
ENSMMUG00000060771		0.533947991	0.006399768	0.018386006
ENSMMUG00000020072	matrilin 2 [Source:VGNC Symbol;Acc:VGNC:74665]	0.435462822	0.002973697	0.009426871
ENSMMUG00000053292	POP1 homolog, ribonuclease P/MRP subunit [Source:NCBI gene;Acc:705643]	0.364185026	0.006053915	0.017521383
ENSMMUG00000022185	odd-skipped related transciption factor 2 [Source:VGNC Symbol;Acc:VGNC:84097]	-1.543549804	4.53E-10	5.23E-09
ENSMMUG00000022186	vacuolar protein sorting 13 homolog B [Source:VGNC Symbol;Acc:VGNC:79339]	-0.472204017	0.000585873	0.002234696
ENSMMUG00000046389	Metazoan signal recognition particle RNA [Source:RFAM;Acc:RF00017]	-2.997468694	0.011234992	0.029934588
ENSMMUG00000013742	regulator of G protein signaling 22 [Source:VGNC Symbol;Acc:VGNC:82221]	-1.862071895	0.002033464	0.006749856
ENSMMUG00000064531	RNA polymerase II subunit K [Source:NCBI gene;Acc:708464]	-3.075915434	0.005120066	0.015143713
ENSMMUG00000004386	sperm associated antigen 1 [Source:VGNC Symbol;Acc:VGNC:77849]	-2.608718736	0.0001282	0.000570601
ENSMMUG00000061687		-2.349461508	1.80E-05	9.65E-05
ENSMMUG00000004387	ring finger protein 19A, RBR E3 ubiquitin protein ligase [Source:VGNC Symbol;Acc:VGNC:82224]	-1.786960202	1.76E-07	1.36E-06
ENSMMUG00000051088		-2.21874124	1.38E-06	9.17E-06

ENSMMUG00000009958	ankyrin repeat domain 46 [Source:VGNC Symbol;Acc:VGNC:69719]	-2.448160772	5.19E-10	5.94E-09
ENSMMUG00000034608	U6 spliceosomal RNA [Source:RFAM;Acc:RF00026]	1.079200843	6.38E-05	0.000303268
ENSMMUG00000017786	poly(A) binding protein cytoplasmic 1 [Source:VGNC Symbol;Acc:VGNC:84098]	0.313155696	0.006206852	0.017904614
ENSMMUG00000051999		-1.360959244	8.92E-05	0.000411529
ENSMMUG00000001338	antizyme inhibitor 1 [Source:VGNC Symbol;Acc:VGNC:70080]	4.033712213	2.03E-06	1.30E-05
ENSMMUG00000040361	ATPase H+ transporting V1 subunit C1 [Source:NCBI gene;Acc:693840]	-2.111926316	9.19E-05	0.000422659
ENSMMUG00000052440		-4.652006116	2.77E-05	0.00014269
ENSMMUG00000054357		-2.960209342	0.000610146	0.002318452
ENSMMUG00000056520		-2.760160261	0.006699665	0.019129001
ENSMMUG00000010744		-1.881451082	0.00215865	0.00709798
ENSMMUG00000011359	eukaryotic translation initiation factor 3 subunit E [Source:VGNC Symbol;Acc:VGNC:83997]	-1.053018791	5.13E-05	0.000248559
ENSMMUG00000003435	transcriptional repressor GATA binding 1 [Source:VGNC Symbol;Acc:VGNC:78656]	-0.704349233	0.001517276	0.005208613
ENSMMUG00000001087		-0.870301403	0.013669765	0.035410065
ENSMMUG00000056417		-1.653186257	0.006035891	0.017476036
ENSMMUG00000002068	mediator complex subunit 30 [Source:VGNC Symbol;Acc:VGNC:74556]	-3.080167824	0.010405971	0.027987042
ENSMMUG00000004846	sterile alpha motif domain containing 12 [Source:VGNC Symbol;Acc:VGNC:77145]	-0.883184421	0.013735748	0.035543734
ENSMMUG00000053962		-1.172935125	0.003250314	0.010203673
ENSMMUG00000024448		-2.138119328	0.002915331	0.009271791
ENSMMUG00000057685	Small nucleolar RNA SNORD55/SNORD39 [Source:RFAM;Acc:RF00157]	-1.865961563	0.010855024	0.029010634
ENSMMUG00000014702	TATA-box binding protein associated factor 2 [Source:VGNC Symbol;Acc:VGNC:78090]	-0.935673573	0.000960568	0.003471057
ENSMMUG00000058797		0.909519213	1.69E-08	1.53E-07
ENSMMUG00000052557		0.381705647	0.000237672	0.000997298
ENSMMUG00000021157	TBC1 domain family member 31 [Source:VGNC Symbol;Acc:VGNC:84121]	2.24884845	0.003339787	0.010433757
ENSMMUG00000020467	ATPase family AAA domain containing 2 [Source:VGNC Symbol;Acc:VGNC:70154]	-0.324886373	0.015766589	0.03990758
ENSMMUG00000028923	WDYHV motif containing 1 [Source:VGNC Symbol;Acc:VGNC:79503]	0.38335169	0.002159069	0.00709798
ENSMMUG00000047542	U4 spliceosomal RNA [Source:RFAM;Acc:RF00015]	-1.592438217	0.001280952	0.004493025
ENSMMUG00000059929	Small nucleolar RNA U3 [Source:RFAM;Acc:RF00012]	-2.058123067	0.002937707	0.00932895
ENSMMUG00000016375	fer-1 like family member 6 [Source:VGNC Symbol;Acc:VGNC:72585]	-0.47411819	0.000165415	0.00071707
ENSMMUG00000054755	transmembrane protein 65 [Source:NCBI gene;Acc:705727]	2.694151081	2.80E-16	6.71E-15
ENSMMUG00000053001		-2.618498198	0.000991372	0.003575402
ENSMMUG00000060708		0.85579658	0.001648289	0.005598748
ENSMMUG00000010440	adenylate cyclase 8 [Source:VGNC Symbol;Acc:VGNC:69516]	-0.949672643	1.17E-05	6.48E-05
ENSMMUG00000058106	otoconin 90 [Source:NCBI gene;Acc:100429699]	1.532624994	2.13E-24	1.20E-22

ENSMMUG00000012560	HERV-H LTR-associating 1 [Source:NCBI gene;Acc:100430297]	1.911490324	6.25E-22	2.78E-20
ENSMMUG00000055934		0.64022294	0.001515267	0.005202918
ENSMMUG00000048066	trafficking protein particle complex 9 [Source:NCBI gene;Acc:711466]	1.364154551	1.73E-09	1.83E-08
ENSMMUG00000062257		-1.506388984	0.004819643	0.014401634
ENSMMUG00000052421		-0.952087665	0.002422744	0.007868878
ENSMMUG00000059617		-1.243790527	1.93E-08	1.74E-07
ENSMMUG00000041901	Metazoan signal recognition particle RNA [Source:RFAM;Acc:RF00017]	-2.383242038	0.010397868	0.027970321
ENSMMUG00000001432	t-SNARE domain containing 1 [Source:NCBI gene;Acc:701269]	-0.844561214	2.32E-07	1.75E-06
ENSMMUG00000062642		-1.345280034	4.53E-09	4.47E-08
ENSMMUG00000049486		-0.725453082	0.014226306	0.036538988
ENSMMUG00000000959	cytochrome P450, family 11, subfamily B, polypeptide 2 [Source:NCBI gene;Acc:695914]	0.65825002	0.000105089	0.000477618
ENSMMUG00000018500	ZFP41 zinc finger protein [Source:NCBI gene;Acc:106999981]	-0.631280274	0.017149869	0.042853002
ENSMMUG00000022933	DNA topoisomerase I mitochondrial [Source:VGNC Symbol;Acc:VGNC:79821]	1.275611712	3.84E-17	1.01E-15
ENSMMUG00000059932	Metazoan signal recognition particle RNA [Source:RFAM;Acc:RF00017]	1.346133351	0.000222103	0.000937066
ENSMMUG00000010939	rhophilin Rho GTPase binding protein 1 [Source:VGNC Symbol;Acc:VGNC:76779]	-0.757329507	6.80E-07	4.76E-06
ENSMMUG00000001187	mitogen-activated protein kinase 15 [Source:VGNC Symbol;Acc:VGNC:74570]	1.169077432	0.011334099	0.030171368
ENSMMUG00000057609		-0.553097386	0.018392997	0.045476517
ENSMMUG00000013552	scribble planar cell polarity protein [Source:HGNC Symbol;Acc:HGNC:30377]	-1.187332914	2.21E-18	6.59E-17
ENSMMUG00000013558	poly(U) binding splicing factor 60 [Source:VGNC Symbol;Acc:VGNC:84106]	-0.802735696	0.003208512	0.010085254
ENSMMUG00000028891	nuclear receptor binding protein 2 [Source:VGNC Symbol;Acc:VGNC:75437]	-1.336959947	0.00873515	0.024105373
ENSMMUG00000063093	epiplakin 1 [Source:NCBI gene;Acc:699265]	0.457545635	2.02E-06	1.30E-05
ENSMMUG00000000388	plectin [Source:VGNC Symbol;Acc:VGNC:76173]	-0.627735949	6.08E-05	0.00029016
ENSMMUG00000060194	mml-mir-661 [Source:miRBase;Acc:MI0007902]	-2.076913412	0.001478425	0.005090559
ENSMMUG00000061025		-3.453015588	0.002682736	0.008604018
ENSMMUG00000052917		0.451572328	0.004443254	0.013406604
ENSMMUG00000021279	zinc finger MYND-type containing 11 [Source:VGNC Symbol;Acc:VGNC:79060]	0.571566427	2.24E-06	1.43E-05
ENSMMUG00000050907		-1.177399919	3.52E-10	4.10E-09
ENSMMUG00000049588		-1.269424907	0.012001385	0.03168041
ENSMMUG00000049879		3.390618872	0.017867267	0.044368984
ENSMMUG00000053106		2.871898159	2.32E-22	1.08E-20
ENSMMUG00000023282	aldo-keto reductase family 1, member C1 (dihydrodiol dehydrogenase 1; 20-alpha (3-alpha)-hydroxysteroid dehydrogenase) [Source:NCBI gene;Acc:711198]	2.442805423	2.14E-20	7.83E-19
ENSMMUG00000000958	aldo-keto reductase family 1, member C2 [Source:NCBI gene;Acc:711360]	2.734714473	3.79E-32	4.50E-30
ENSMMUG00000063267	aldo-keto reductase family 1 member C15 [Source:NCBI gene;Acc:722496]	-1.451446533	8.56E-05	0.000396751
ENSMMUG00000001290	ankyrin repeat and SOCS box containing 13 [Source:VGNC Symbol;Acc:VGNC:69865]	-3.76542129	7.62E-05	0.000355776
ENSMMUG00000015141	ankyrin repeat domain 16 [Source:NCBI gene;Acc:720787]	-2.358808067	2.99E-07	2.22E-06

ENSMMUG00000015143	F-box DNA helicase 1 [Source:NCBI gene;Acc:720797]	-2.159281401	1.38E-08	1.27E-07
ENSMMUG00000056045		-2.53568365	1.63E-09	1.73E-08
ENSMMUG00000015149	interleukin 15 receptor subunit alpha [Source:VGNC Symbol;Acc:VGNC:73711]	-2.148078488	0.000146119	0.000640918
ENSMMUG00000015151	interleukin 2 receptor subunit alpha [Source:NCBI gene;Acc:574300]	-2.285317556	4.77E-10	5.49E-09
ENSMMUG00000054646		-3.954959026	0.000103377	0.000470848
ENSMMUG00000061189		0.654035261	5.96E-05	0.000285226
ENSMMUG00000052242		0.88271479	6.50E-05	0.000308291
ENSMMUG00000064980		-2.104383893	2.23E-06	1.42E-05
ENSMMUG00000037449	CUGBP Elav-like family member 2 [Source:NCBI gene;Acc:714291]	1.032978154	1.52E-05	8.28E-05
ENSMMUG00000017145	cell division cycle 123 [Source:VGNC Symbol;Acc:VGNC:70768]	-1.07391001	6.75E-07	4.73E-06
ENSMMUG00000017164	FERM domain containing 4A [Source:VGNC Symbol;Acc:VGNC:72786]	-0.494632321	0.018013038	0.044701029
ENSMMUG00000017170	cerebral dopamine neurotrophic factor [Source:VGNC Symbol;Acc:VGNC:71094]	-0.652306862	7.87E-08	6.43E-07
ENSMMUG00000017173	heat shock protein family A (Hsp70) member 14 [Source:NCBI gene;Acc:697257]	-2.239927589	2.30E-07	1.74E-06
ENSMMUG00000017174	suppressor of variegation 3-9 homolog 2 [Source:NCBI gene;Acc:697378]	-3.457275622	0.001689508	0.005729567
ENSMMUG00000017176	DNA cross-link repair 1C [Source:NCBI gene;Acc:697493]	-3.65654635	0.001371923	0.004771485
ENSMMUG00000011661	integrin subunit alpha 8 [Source:VGNC Symbol;Acc:VGNC:73782]	0.981114345	2.02E-07	1.55E-06
ENSMMUG00000024234	U6 spliceosomal RNA [Source:RFAM;Acc:RF00026]	2.120834085	2.86E-06	1.78E-05
ENSMMUG00000016069	cubilin [Source:VGNC Symbol;Acc:VGNC:82132]	3.779810171	1.34E-21	5.71E-20
ENSMMUG00000010589	nebulette [Source:VGNC Symbol;Acc:VGNC:75195]	-0.782852529	2.63E-06	1.65E-05
ENSMMUG00000038216	chromosome 9 C10orf113 homolog [Source:NCBI gene;Acc:702996]	-1.008852251	0.003316843	0.010366445
ENSMMUG00000023648	protein AF-10 [Source:NCBI gene;Acc:709083]	0.344327441	0.018505609	0.045709224
ENSMMUG00000002930	G protein-coupled receptor 158 [Source:NCBI gene;Acc:707065]	0.974456782	1.10E-06	7.45E-06
ENSMMUG00000058873		1.022614367	1.20E-05	6.68E-05
ENSMMUG00000037243	U6 spliceosomal RNA [Source:RFAM;Acc:RF00026]	1.518366332	5.09E-06	3.02E-05
ENSMMUG00000016407	microtubule associated serine/threonine kinase like [Source:NCBI gene;Acc:708134]	1.177644529	0.00658557	0.018839507
ENSMMUG00000016410	acyl-CoA binding domain containing 5 [Source:VGNC Symbol;Acc:VGNC:69509]	0.829050098	0.00011193	0.000504687
ENSMMUG00000051106		0.922723523	0.007062392	0.020022036
ENSMMUG00000020657	patched domain containing 3 [Source:VGNC Symbol;Acc:VGNC:76310]	0.896223466	6.37E-05	0.000303045
ENSMMUG00000058108	BMP and activin membrane bound inhibitor [Source:NCBI gene;Acc:710152]	1.656314001	3.34E-05	0.000168926
ENSMMUG00000059681		1.536475861	0.000435162	0.001716799
ENSMMUG00000024540	5S ribosomal RNA [Source:RFAM;Acc:RF00001]	1.363257479	0.014199062	0.036500436
ENSMMUG00000001374	supervillin [Source:NCBI gene;Acc:695267]	-1.577781876	9.92E-15	2.07E-13
ENSMMUG00000050047	Metazoan signal recognition particle RNA [Source:RFAM;Acc:RF00017]	1.058459054	1.10E-07	8.84E-07
ENSMMUG00000021559	junctional cadherin 5 associated [Source:VGNC Symbol;Acc:VGNC:73937]	-1.860008548	0.014649235	0.037508315
ENSMMUG00000041067	Metazoan signal recognition particle RNA [Source:RFAM;Acc:RF00017]	-0.995785822	0.014130304	0.036367997
ENSMMUG00000040958	lysozyme-like protein 1 [Source:NCBI gene;Acc:698176]	-0.928667604	2.45E-05	0.000127493
ENSMMUG00000044214		-1.148634902	3.48E-10	4.07E-09

ENSMMUG00000007864	zinc finger protein 438 [Source:NCBI gene;Acc:700170]	-1.726417068	0.004398232	0.013292396
ENSMMUG00000043648		1.344341784	0.003717818	0.011443595
ENSMMUG00000057199		-0.984037604	0.000862092	0.003145327
ENSMMUG00000006713	cyclin Y [Source:VGNC Symbol;Acc:VGNC:70840]	-0.835368264	0.000275871	0.0011377
ENSMMUG00000007426	BMS1 ribosome biogenesis factor [Source:HGNC Symbol;Acc:HGNC:23505]	-0.60819095	0.00841115	0.023341572
ENSMMUG00000054392	zinc finger protein 239 [Source:NCBI gene;Acc:717737]	-0.948433203	0.008540569	0.023643204
ENSMMUG00000056797		2.513434958	0.009646102	0.026219152
ENSMMUG00000053760		-0.933936339	3.44E-05	0.000173236
ENSMMUG00000060110	pleckstrin homology domain-containing family B member 2 [Source:NCBI gene;Acc:720588]	-1.491554796	0.011331237	0.030169165
ENSMMUG00000057407	dorsal root ganglia homeobox [Source:NCBI gene;Acc:719355]	-1.40061021	0.00180994	0.006096163
ENSMMUG00000021308	chromosome 9 C10orf71 homolog [Source:NCBI gene;Acc:710332]	-1.460535808	7.57E-07	5.26E-06
ENSMMUG00000033001	U6 spliceosomal RNA [Source:RFAM;Acc:RF00026]	-1.07993269	1.29E-06	8.59E-06
ENSMMUG00000055918		2.552739385	0.000585579	0.00223415
ENSMMUG00000058251		-1.853530308	0.003904758	0.011961899
ENSMMUG00000018634	methionine adenosyltransferase 1A [Source:VGNC Symbol;Acc:VGNC:74663]	-0.922679502	0.000143343	0.000629673
ENSMMUG00000063666		-0.726454211	0.01312471	0.034153265
ENSMMUG00000019780	annexin A11 [Source:VGNC Symbol;Acc:VGNC:69946]	-0.649567366	0.0130877	0.034072153
ENSMMUG00000057196	pulmonary surfactant-associated protein A [Source:NCBI gene;Acc:701715]	2.350549398	3.99E-36	7.49E-34
ENSMMUG00000051972	zinc finger CCHC-type containing 24 [Source:NCBI gene;Acc:702083]	3.867687442	5.23E-12	7.82E-11
ENSMMUG00000049862		1.182224795	2.02E-05	0.00010726
ENSMMUG00000008790	zinc finger MIZ-type containing 1 [Source:VGNC Symbol;Acc:VGNC:79036]	0.867929039	3.12E-05	0.000158814
ENSMMUG00000055601		1.114400077	9.70E-24	5.23E-22
ENSMMUG00000005514	RNA polymerase III subunit A [Source:VGNC Symbol;Acc:VGNC:76138]	0.720159607	2.20E-06	1.40E-05
ENSMMUG00000009128	discs large MAGUK scaffold protein 5 [Source:VGNC Symbol;Acc:VGNC:71921]	0.643887658	9.01E-06	5.12E-05
ENSMMUG00000050271		-0.600261483	0.014990412	0.038243216
ENSMMUG00000053916		0.973036593	4.63E-11	6.12E-10
ENSMMUG00000050841		0.625498121	0.003860068	0.011847023
ENSMMUG00000021393	calcium/calmodulin dependent protein kinase II gamma [Source:VGNC Symbol;Acc:VGNC:70525]	-1.932465407	3.47E-08	2.97E-07
ENSMMUG00000004604	zinc finger SWIM-type containing 8 [Source:VGNC Symbol;Acc:VGNC:79396]	-1.594911872	0.004421223	0.013348276
ENSMMUG00000061526	synaptopodin 2 like [Source:NCBI gene;Acc:714736]	1.384827255	1.18E-10	1.47E-09
ENSMMUG00000013696	myozenin 1 [Source:VGNC Symbol;Acc:VGNC:74951]	1.437593969	1.43E-09	1.53E-08
ENSMMUG00000020402	mitochondrial calcium uniporter [Source:VGNC Symbol;Acc:VGNC:74560]	-0.984938775	0.006577789	0.018824509
ENSMMUG00000010431	DnaJ heat shock protein family (Hsp40) member B12 [Source:NCBI gene;Acc:721891]	-1.197464068	0.010597525	0.028414626
ENSMMUG00000055226	DNA damage inducible transcript 4 [Source:NCBI gene;Acc:715307]	-1.786244805	0.000801227	0.002948435
ENSMMUG00000009479	activating signal cointegrator 1 complex subunit 1 [Source:VGNC Symbol;Acc:VGNC:69896]	-1.497589191	0.020193845	0.049288137
ENSMMUG00000063113		3.102504989	0.015908051	0.040231309
ENSMMUG00000016652	cell division cycle and apoptosis regulator 1 [Source:NCBI gene;Acc:693585]	0.87904492	1.26E-08	1.16E-07

ENSMMUG00000023198	tet methylcytosine dioxygenase 1 [Source:VGNC Symbol;Acc:VGNC:79811]	0.526178077	1.68E-05	9.10E-05
ENSMMUG00000030532	solute carrier family 25 member 16 [Source:NCBI gene;Acc:694145]	1.004657067	1.42E-08	1.30E-07
ENSMMUG00000019703	RUN and FYVE domain containing 2 [Source:VGNC Symbol;Acc:VGNC:77057]	1.188073095	1.24E-20	4.74E-19
ENSMMUG00000050104	Metazoan signal recognition particle RNA [Source:RFAM;Acc:RF00017]	1.266667683	1.71E-08	1.54E-07
ENSMMUG00000009792	heterogeneous nuclear ribonucleoprotein H3 [Source:VGNC Symbol;Acc:VGNC:73497]	0.715904477	0.000101816	0.000464595
ENSMMUG00000016902	catenin alpha 3 [Source:VGNC Symbol;Acc:VGNC:82131]	0.546314929	0.004265852	0.012946629
ENSMMUG00000053199		0.576825665	0.004137544	0.0126099
ENSMMUG00000059754		0.836061012	0.000596296	0.002270365
ENSMMUG00000009794	protocadherin related 15 [Source:VGNC Symbol;Acc:VGNC:75608]	1.017487058	1.34E-13	2.46E-12
ENSMMUG00000010844	protein kinase cGMP-dependent 1 [Source:VGNC Symbol;Acc:VGNC:76419]	-1.713780697	0.003864545	0.011855855
ENSMMUG00000007078	N-acylsphingosine amidohydrolase 2 [Source:NCBI gene;Acc:703913]	-0.7162777	3.08E-10	3.64E-09
ENSMMUG00000007081	sphingomyelin synthase 1 [Source:VGNC Symbol;Acc:VGNC:77341]	0.716976098	0.002364963	0.007698067
ENSMMUG00000006414	ATPase family AAA domain containing 1 [Source:NCBI gene;Acc:704809]	0.979516987	0.006469325	0.018564258
ENSMMUG00000038485	Metazoan signal recognition particle RNA [Source:RFAM;Acc:RF00017]	1.284642176	0.003016193	0.009529736
ENSMMUG00000057042		1.004199289	0.007055015	0.020004947
ENSMMUG00000014343	lipase family member J [Source:VGNC Symbol;Acc:VGNC:74333]	-1.428301866	5.45E-05	0.000262754
ENSMMUG00000000470	ankyrin repeat domain 22 [Source:VGNC Symbol;Acc:VGNC:69728]	-1.962192719	0.000579325	0.002214845
ENSMMUG00000061761	interferon induced protein with tetratricopeptide repeats 2 [Source:NCBI gene;Acc:695452]	-1.031086448	0.002074439	0.006868482
ENSMMUG00000058942		-1.263661919	0.000783459	0.002893102
ENSMMUG00000017533	interferon induced protein with tetratricopeptide repeats 1 [Source:NCBI gene;Acc:695683]	0.832550657	0.000419415	0.001662637
ENSMMUG00000059038	5-hydroxytryptamine receptor 7 [Source:NCBI gene;Acc:696317]	-1.427333065	9.10E-06	5.16E-05
ENSMMUG00000006628	ribonuclease P/MRP subunit p30 [Source:VGNC Symbol;Acc:VGNC:77040]	-2.204910144	0.000269253	0.001115369
ENSMMUG00000038018	polycomb group ring finger 5 [Source:NCBI gene;Acc:697020]	2.505076049	7.32E-05	0.000343385
ENSMMUG00000000238	HECT domain E3 ubiquitin protein ligase 2 [Source:VGNC Symbol;Acc:VGNC:73246]	-2.229433185	7.35E-15	1.55E-13
ENSMMUG00000058603		-2.937449982	2.11E-08	1.89E-07
ENSMMUG00000059608	fibroblast growth factor binding protein 3 [Source:NCBI gene;Acc:697881]	1.336535936	2.22E-11	3.04E-10
ENSMMUG00000017627	B-TFIID TATA-box binding protein associated factor 1 [Source:VGNC Symbol;Acc:VGNC:70336]	1.680531246	1.78E-34	2.87E-32
ENSMMUG00000004816	membrane associated ring-CH-type finger 5 [Source:VGNC Symbol;Acc:VGNC:74588]	1.325465087	2.62E-10	3.13E-09
ENSMMUG00000023266	kinesin family member 11 [Source:VGNC Symbol;Acc:VGNC:74030]	1.014241263	0.002173297	0.007138426
ENSMMUG00000058935	Metazoan signal recognition particle RNA [Source:RFAM;Acc:RF00017]	1.774144279	0.00031022	0.001265282
ENSMMUG00000049455	Metazoan signal recognition particle RNA [Source:RFAM;Acc:RF00017]	2.221642816	0.009427248	0.025737498
ENSMMUG00000011789	hematopoietically expressed homeobox [Source:VGNC Symbol;Acc:VGNC:73376]	3.087640744	9.16E-34	1.33E-31
ENSMMUG00000049508		3.263381247	9.86E-13	1.63E-11
ENSMMUG00000028000	Y RNA [Source:RFAM;Acc:RF00019]	3.650305426	1.26E-05	6.95E-05
ENSMMUG00000052637	Metazoan signal recognition particle RNA [Source:RFAM;Acc:RF00017]	3.423802466	3.56E-18	1.04E-16
ENSMMUG00000010881	cytochrome P450, family 26, subfamily A, polypeptide 1 [Source:NCBI gene;Acc:699519]	1.286688577	0.002990686	0.009472849
ENSMMUG00000006841	myoferlin [Source:VGNC Symbol;Acc:VGNC:75061]	2.515670136	0.002584603	0.008325296
ENSMMUG00000014715	fragile site, folic acid type, rare, fra(10)(q23.3) or fra(10)(q24.2) candidate 1 [Source:NCBI gene;Acc:700389]	3.129691413	3.28E-15	7.11E-14
ENSMMUG00000050975		-1.578359598	4.32E-08	3.65E-07
ENSMMUG00000008687	B cell linker [Source:VGNC Symbol;Acc:VGNC:70306]	-0.662112355	9.36E-08	7.57E-07
ENSMMUG00000001876	phosphoinositide-3-kinase adaptor protein 1 [Source:VGNC Symbol;Acc:VGNC:75891]	-1.083358	5.02E-07	3.59E-06

ENSMMUG00000010427	ligand dependent nuclear receptor corepressor [Source:NCBI gene;Acc:705646]	-0.661810116	0.000102581	0.000467797
ENSMMUG00000010428	slit guidance ligand 1 [Source:VGNC Symbol;Acc:VGNC:77643]	0.777911647	2.00E-18	6.00E-17
ENSMMUG00000019634	ribosomal RNA processing 12 homolog [Source:VGNC Symbol;Acc:VGNC:77122]	-0.408680618	0.002021481	0.006717677
ENSMMUG00000052662	Y RNA [Source:RFAM;Acc:RF00019]	-1.406813474	0.001037412	0.003717936
ENSMMUG00000015503	zinc finger FYVE-type containing 27 [Source:VGNC Symbol;Acc:VGNC:79623]	0.785865422	1.45E-12	2.34E-11
ENSMMUG00000060995		-2.199767123	0.013035592	0.033963076
ENSMMUG00000020603	pyridine nucleotide-disulphide oxidoreductase domain 2 [Source:NCBI gene;Acc:707797]	1.074231367	4.57E-20	1.62E-18
ENSMMUG00000054904	Metazoan signal recognition particle RNA [Source:RFAM;Acc:RF00017]	1.288353109	4.81E-15	1.03E-13
ENSMMUG00000020604	HPS1 biogenesis of lysosomal organelles complex 3 subunit 1 [Source:VGNC Symbol;Acc:VGNC:73515]	1.031317495	3.48E-10	4.07E-09
ENSMMUG00000049770	Metazoan signal recognition particle RNA [Source:RFAM;Acc:RF00017]	1.002644393	0.001555468	0.005323698
ENSMMUG00000020605	heparanase 2 (inactive) [Source:NCBI gene;Acc:708795]	0.73354885	0.001085981	0.003876208
ENSMMUG00000060230	Metazoan signal recognition particle RNA [Source:RFAM;Acc:RF00017]	0.92001164	0.000640719	0.002423945
ENSMMUG00000010357	cyclin and CBS domain divalent metal cation transport mediator 1 [Source:NCBI gene;Acc:708335]	1.369437922	1.92E-12	3.07E-11
ENSMMUG00000023761	ectonucleoside triphosphate diphosphohydrolase 7 [Source:VGNC Symbol;Acc:VGNC:72071]	1.169510582	2.21E-08	1.97E-07
ENSMMUG00000063121		0.934427189	0.001229769	0.004331942
ENSMMUG00000015117	X-prolyl aminopeptidase 1 [Source:VGNC Symbol;Acc:VGNC:78810]	-2.082476146	1.37E-09	1.47E-08
ENSMMUG00000006233	adducin 3 [Source:VGNC Symbol;Acc:VGNC:69531]	1.944304705	7.37E-09	7.07E-08
ENSMMUG00000063986		1.531614358	0.000568377	0.002175795
ENSMMUG00000051414	Metazoan signal recognition particle RNA [Source:RFAM;Acc:RF00017]	1.150366112	0.001237842	0.004356241
ENSMMUG00000014350	MAX interactor 1, dimerization protein [Source:VGNC Symbol;Acc:VGNC:75087]	-1.174115572	2.15E-09	2.24E-08
ENSMMUG00000007775	survival motor neuron domain containing 1 [Source:NCBI gene;Acc:694761]	-1.642578704	2.99E-07	2.21E-06
ENSMMUG00000057793	dual specificity phosphatase 5 [Source:NCBI gene;Acc:698178]	-2.413177769	0.00499184	0.014835468
ENSMMUG00000007982	structural maintenance of chromosomes 3 [Source:VGNC Symbol;Acc:VGNC:77769]	-1.258367419	0.00404664	0.012355707
ENSMMUG00000017204	programmed cell death 4 [Source:VGNC Symbol;Acc:VGNC:75794]	-2.74901709	0.000139573	0.000614836
ENSMMUG00000056056		-3.08385595	0.001466404	0.005055037
ENSMMUG00000017206	SHOC2 leucine rich repeat scaffold protein [Source:VGNC Symbol;Acc:VGNC:81569]	-3.036496865	0.000139145	0.000613596
ENSMMUG00000003548	tectorin beta [Source:VGNC Symbol;Acc:VGNC:78306]	1.287083142	0.001040286	0.003726435
ENSMMUG00000054123	vesicle transport through interaction with t-SNAREs 1A [Source:NCBI gene;Acc:696654]	0.957491159	2.84E-07	2.11E-06
ENSMMUG00000053789		-1.181144192	7.81E-07	5.41E-06
ENSMMUG00000001217	caspase 7 [Source:VGNC Symbol;Acc:VGNC:70546]	-0.981602825	5.53E-05	0.000266035
ENSMMUG00000055239		1.016091738	2.75E-12	4.30E-11
ENSMMUG00000022514	coiled-coil domain containing 186 [Source:VGNC Symbol;Acc:VGNC:70847]	0.837937334	2.59E-08	2.28E-07
ENSMMUG00000022524	actin binding LIM protein 1 [Source:VGNC Symbol;Acc:VGNC:69587]	-0.406578949	0.004621645	0.013891163
ENSMMUG00000058240		-1.954981396	3.08E-08	2.68E-07
ENSMMUG00000015414	family with sequence similarity 160 member B1 [Source:VGNC Symbol;Acc:VGNC:72403]	-1.351195644	3.81E-07	2.77E-06
ENSMMUG00000036145	U6 spliceosomal RNA [Source:RFAM;Acc:RF00026]	-2.530523867	0.020059604	0.048984675
ENSMMUG00000016663	coiled-coil domain containing 172 [Source:VGNC Symbol;Acc:VGNC:70916]	-1.037546492	0.005669103	0.016534079

ENSMMUG00000053348	PDZ domain containing 8 [Source:NCBI gene;Acc:709084]	-0.972983213	0.000189336	0.00081152
ENSMMUG00000059847		-0.719656033	0.017215146	0.042960428
ENSMMUG00000021610	regulator of G protein signaling 10 [Source:VGNC Symbol;Acc:VGNC:76915]	2.547762222	1.83E-38	4.05E-36
ENSMMUG00000054942		2.268826967	1.10E-10	1.39E-09
ENSMMUG00000001472	TIA1 cytotoxic granule associated RNA binding protein like 1 [Source:VGNC Symbol;Acc:VGNC:78354]	2.344128804	2.64E-18	7.84E-17
ENSMMUG00000009559	minichromosome maintenance complex binding protein [Source:VGNC Symbol;Acc:VGNC:74583]	2.151374021	1.22E-11	1.73E-10
ENSMMUG00000064321		1.960739809	3.25E-14	6.33E-13
ENSMMUG00000064557		2.111482015	3.47E-24	1.93E-22
ENSMMUG00000052711		-0.861303526	6.96E-06	4.03E-05
ENSMMUG00000021594	arginyltransferase 1 [Source:VGNC Symbol;Acc:VGNC:70168]	-2.802968393	0.002619701	0.008421887
ENSMMUG00000023480	IKAROS family zinc finger 5 [Source:VGNC Symbol;Acc:VGNC:73590]	-1.797174388	4.12E-06	2.49E-05
ENSMMUG00000000684	acyl-CoA dehydrogenase short/branched chain [Source:VGNC Symbol;Acc:VGNC:81343]	-1.573248948	0.000787823	0.00290777
ENSMMUG00000020346	carboxypeptidase X, M14 family member 2 [Source:VGNC Symbol;Acc:VGNC:71596]	1.526272799	2.88E-20	1.05E-18
ENSMMUG00000058933		-2.514798959	8.02E-07	5.54E-06
ENSMMUG00000008539	uroporphyrinogen III synthase [Source:VGNC Symbol;Acc:VGNC:78731]	-1.225307664	8.11E-09	7.71E-08
ENSMMUG00000008541	BRCA2 and CDKN1A interacting protein [Source:NCBI gene;Acc:699437]	-1.049123856	7.79E-08	6.38E-07
ENSMMUG00000008542	DEAH-box helicase 32 (putative) [Source:NCBI gene;Acc:695919]	-0.905043682	0.00013348	0.000591959
ENSMMUG00000035166	U2 spliceosomal RNA [Source:RFAM;Acc:RF00004]	-1.43372377	0.000749159	0.002773347
ENSMMUG00000044121	marker of proliferation Ki-67 [Source:NCBI gene;Acc:697883]	-0.6767984	0.000320429	0.001304049
ENSMMUG00000064746		-3.42611819	0.014188618	0.036492751
ENSMMUG00000059108		-1.974577034	0.002153549	0.007084547
ENSMMUG00000059161		-2.021573834	0.003157402	0.00993513
ENSMMUG00000013653	lysine demethylase 5A [Source:VGNC Symbol;Acc:VGNC:82169]	0.674577815	1.27E-11	1.80E-10
ENSMMUG00000013894	beta-1,4-N-acetyl-galactosaminyltransferase 3 [Source:VGNC Symbol;Acc:VGNC:70121]	-1.24515079	0.00163418	0.005555912
ENSMMUG00000018650	WNK lysine deficient protein kinase 1 [Source:VGNC Symbol;Acc:VGNC:78960]	0.520176513	0.003289345	0.010291346
ENSMMUG00000007405	FKBP prolyl isomerase 4 [Source:VGNC Symbol;Acc:VGNC:72664]	-1.35192371	0.007694402	0.021586829
ENSMMUG00000007411	integrin alpha FG-GAP repeat containing 2 [Source:VGNC Symbol;Acc:VGNC:73690]	-1.807797823	1.10E-13	2.04E-12
ENSMMUG00000007424	nuclear receptor interacting protein 2 [Source:VGNC Symbol;Acc:VGNC:75448]	-1.657847124	2.86E-14	5.62E-13
ENSMMUG00000037675	testis expressed 52 [Source:NCBI gene;Acc:107000723]	-1.431608005	1.00E-10	1.27E-09
ENSMMUG00000061935		2.43476304	6.83E-13	1.16E-11
ENSMMUG00000023583	calcium release activated channel regulator 2A [Source:NCBI gene;Acc:709929]	-1.133200347	0.003691273	0.01137842
ENSMMUG00000018273	poly(ADP-ribose) polymerase family member 11 [Source:NCBI gene;Acc:721828]	-0.820076288	0.015019453	0.038297539
ENSMMUG00000057580		-1.015392044	0.001605794	0.005469435
ENSMMUG00000008546	cyclin D2 [Source:NCBI gene;Acc:710379]	-1.065529556	3.86E-05	0.000191667
ENSMMUG00000064833	fibroblast growth factor 23 [Source:NCBI gene;Acc:710643]	-0.804368628	0.016481613	0.041455618

ENSMMUG00000015189	RAD51 associated protein 1 [Source:VGNC Symbol;Acc:VGNC:76497]	-1.17714961	8.69E-05	0.000402513
ENSMMUG00000020811	dual specificity tyrosine phosphorylation regulated kinase 4 [Source:NCBI gene;Acc:710999]	-0.773145792	0.0006071	0.002307947
ENSMMUG00000020812	A-kinase anchoring protein 3 [Source:VGNC Symbol;Acc:VGNC:69769]	-1.43659842	9.27E-05	0.000426175
ENSMMUG00000020813	NADH:ubiquinone oxidoreductase subunit A9 [Source:VGNC Symbol;Acc:VGNC:81480]	-0.93218495	1.59E-05	8.62E-05
ENSMMUG00000022893	von Willebrand factor [Source:VGNC Symbol;Acc:VGNC:78778]	2.967769405	4.41E-09	4.37E-08
ENSMMUG00000047465	Metazoan signal recognition particle RNA [Source:RFAM;Acc:RF00017]	4.950593274	2.13E-08	1.91E-07
ENSMMUG00000004470	CD9 molecule [Source:VGNC Symbol;Acc:VGNC:70787]	3.662878216	2.14E-08	1.91E-07
ENSMMUG00000000375	pleckstrin homology and RhoGEF domain containing G6 [Source:VGNC Symbol;Acc:VGNC:76110]	2.491834176	1.83E-36	3.52E-34
ENSMMUG00000000377	TNF receptor superfamily member 1A [Source:VGNC Symbol;Acc:VGNC:78589]	1.143701334	0.01775442	0.044133097
ENSMMUG00000053013		2.059305705	1.75E-13	3.16E-12
ENSMMUG00000014265	TAP binding protein like [Source:NCBI gene;Acc:712626]	2.648526038	7.42E-21	2.88E-19
ENSMMUG00000018679	glyceraldehyde-3-phosphate dehydrogenase [Source:NCBI gene;Acc:574353]	-1.760483344	1.56E-07	1.22E-06
ENSMMUG00000017619	lysophosphatidic acid receptor 5 [Source:VGNC Symbol;Acc:VGNC:74277]	1.273754495	5.79E-15	1.24E-13
ENSMMUG00000016983	acrosin binding protein [Source:VGNC Symbol;Acc:VGNC:69445]	-1.391330464	1.96E-13	3.52E-12
ENSMMUG00000050979	PILR alpha associated neural protein [Source:NCBI gene;Acc:713548]	-1.511100396	0.004761075	0.014255349
ENSMMUG00000009583	COP9 signalosome subunit 7A [Source:VGNC Symbol;Acc:VGNC:71185]	1.65673147	6.14E-05	0.000292861
ENSMMUG00000013202	CD4 molecule [Source:NCBI gene;Acc:713807]	0.493672224	0.003510006	0.01088983
ENSMMUG00000005189	prolyl 3-hydroxylase 3 [Source:VGNC Symbol;Acc:VGNC:75650]	0.401209609	0.019846002	0.048534994
ENSMMUG00000005192	ubiquitin specific peptidase 5 [Source:VGNC Symbol;Acc:VGNC:78747]	0.31646786	0.015105956	0.038471807
ENSMMUG00000009443	enolase 2 [Source:VGNC Symbol;Acc:VGNC:72219]	-1.902921434	0.004177193	0.012713634
ENSMMUG00000010201	atrophin 1 [Source:VGNC Symbol;Acc:VGNC:70157]	0.966568853	6.66E-05	0.000315211
ENSMMUG00000010203	protein tyrosine phosphatase non-receptor type 6 [Source:VGNC Symbol;Acc:VGNC:76607]	1.004360998	3.63E-05	0.00018171
ENSMMUG00000012786	complement C1s [Source:VGNC Symbol;Acc:VGNC:70297]	-0.747023059	1.41E-06	9.30E-06
ENSMMUG00000006470	complement C1r subcomponent [Source:NCBI gene;Acc:722133]	1.372850956	4.38E-17	1.14E-15
ENSMMUG00000022109	CD163 molecule like 1 [Source:VGNC Symbol;Acc:VGNC:70914]	1.886176972	2.26E-21	9.35E-20
ENSMMUG00000005150	CD163 molecule [Source:VGNC Symbol;Acc:VGNC:70752]	2.130823917	3.72E-06	2.26E-05
ENSMMUG00000057570		3.537832699	1.17E-52	1.57E-49
ENSMMUG00000048744		1.794565629	5.78E-05	0.000276927
ENSMMUG00000057785		7.413054822	9.66E-22	4.20E-20
ENSMMUG00000061774	taste 2 receptor member 10 [Source:NCBI gene;Acc:717960]	-1.895789438	0.009499509	0.02589188
ENSMMUG00000050862		-1.411048645	1.90E-09	2.00E-08
ENSMMUG00000018298	killer cell lectin like receptor D1 [Source:NCBI gene;Acc:574145]	3.342446733	4.47E-06	2.69E-05
ENSMMUG00000015305	C-type lectin domain family 2 member A [Source:VGNC Symbol;Acc:VGNC:71329]	-1.873181181	0.000101491	0.000463537
ENSMMUG00000045446		-3.404992271	1.17E-05	6.51E-05
ENSMMUG00000032892	U6 spliceosomal RNA [Source:RFAM;Acc:RF00026]	-1.993384908	1.22E-06	8.12E-06
ENSMMUG00000044047	killer cell lectin like receptor B1 [Source:NCBI gene;Acc:100430783]	-1.216526806	2.21E-05	0.000116429
ENSMMUG00000056741	ovostatin-like [Source:NCBI gene;Acc:722294]	2.884395506	8.82E-07	6.04E-06
ENSMMUG00000003238	pregnancy zone protein [Source:NCBI gene;Acc:100430319]	-1.35348341	0.005349909	0.015726293
ENSMMUG00000022680	alpha-2-macroglobulin like 1 [Source:VGNC Symbol;Acc:VGNC:69507]	-1.762148233	1.01E-08	9.43E-08
ENSMMUG00000022676	ribosomal modification protein rimK like family member B [Source:NCBI gene;Acc:722261]	-2.911075682	0.001638428	0.005567801

ENSMMUG00000013698	C-type lectin domain containing 6A [Source:VGNC Symbol;Acc:VGNC:71179]	-1.11861071	0.017728453	0.044075935
ENSMMUG00000052134		1.359759265	0.000468781	0.001834308
ENSMMUG00000060675		1.291572963	1.39E-09	1.49E-08
ENSMMUG00000021005	taste receptor type 2 member 50 [Source:NCBI gene;Acc:693513]	1.200840472	0.000123483	0.000551415
ENSMMUG00000056428	taste receptor type 2 member 20 [Source:NCBI gene;Acc:693643]	1.176730616	0.002122497	0.007001031
ENSMMUG00000050696	taste receptor type 2 member 31-like [Source:NCBI gene;Acc:693892]	1.227980879	6.62E-09	6.38E-08
ENSMMUG00000054225	taste receptor type 2 member 43 [Source:NCBI gene;Acc:694161]	1.811519819	3.92E-06	2.38E-05
ENSMMUG00000042870	Macaca mulatta taste receptor, type 2, member 46 (TAS2R46), mRNA. [Source:RefSeq mRNA;Acc:NM_001080757]	1.134086928	0.001626159	0.005532448
ENSMMUG00000023317	LDL receptor related protein 6 [Source:VGNC Symbol;Acc:VGNC:74446]	-0.902318929	0.00378256	0.011625371
ENSMMUG00000019112	glutamate ionotropic receptor NMDA type subunit 2B [Source:VGNC Symbol;Acc:VGNC:73283]	-0.495154788	3.52E-05	0.000176815
ENSMMUG00000047247	matrix Gla protein [Source:NCBI gene;Acc:574116]	2.965641313	0.000206835	0.000879405
ENSMMUG00000018030	endoplasmic reticulum protein 27 [Source:VGNC Symbol;Acc:VGNC:72369]	1.964592933	1.48E-07	1.16E-06
ENSMMUG00000053781	Rho GDP dissociation inhibitor beta [Source:NCBI gene;Acc:702320]	2.4700581	6.26E-07	4.41E-06
ENSMMUG00000050529	phosphodiesterase 6H [Source:NCBI gene;Acc:700447]	1.808019691	0.009603859	0.026126428
ENSMMUG00000056014	potassium inwardly rectifying channel subfamily J member 8 [Source:NCBI gene;Acc:706369]	-1.360208331	0.011995567	0.031670689
ENSMMUG00000049682	Metazoan signal recognition particle RNA [Source:RFAM;Acc:RF00017]	-2.33794212	0.001429001	0.00494103
ENSMMUG00000048030	ferritin, heavy polypeptide 1-like [Source:NCBI gene;Acc:707011]	-2.135170246	0.017843692	0.04434014
ENSMMUG00000020370	SRY-box transcription factor 5 [Source:VGNC Symbol;Acc:VGNC:77843]	-2.602289296	2.40E-05	0.000125048
ENSMMUG00000057276		-1.972716388	0.00155234	0.005314222
ENSMMUG00000049874		-2.24854008	0.005000547	0.014852415
ENSMMUG00000038076	Metazoan signal recognition particle RNA [Source:RFAM;Acc:RF00017]	0.913540247	0.005644476	0.016481113
ENSMMUG00000060493		0.711205345	6.55E-06	3.81E-05
ENSMMUG00000012223	branched chain amino acid transaminase 1 [Source:VGNC Symbol;Acc:VGNC:70236]	1.028217366	4.14E-14	8.00E-13
ENSMMUG00000053657		-2.288580072	0.001180586	0.004180536
ENSMMUG00000039562	basic helix-loop-helix family member e41 [Source:NCBI gene;Acc:709786]	0.285063822	0.009306248	0.025458707
ENSMMUG00000018630	sarcospan [Source:VGNC Symbol;Acc:VGNC:77887]	0.404945593	0.001612847	0.005492198
ENSMMUG00000050627		0.376164129	0.000288885	0.001183473
ENSMMUG00000023128	inositol 1,4,5-trisphosphate receptor type 2 [Source:VGNC Symbol;Acc:VGNC:73805]	0.46179482	0.000103534	0.000471419
ENSMMUG00000006468	FGFR1 oncogene partner 2 [Source:VGNC Symbol;Acc:VGNC:72551]	1.493173048	2.46E-09	2.54E-08
ENSMMUG00000003217	transmembrane 7 superfamily member 3 [Source:VGNC Symbol;Acc:VGNC:79371]	1.613281691	1.45E-19	4.79E-18
ENSMMUG00000023248	serine/threonine kinase 38 like [Source:VGNC Symbol;Acc:VGNC:78156]	-1.39640294	1.92E-06	1.23E-05
ENSMMUG00000023249	aryl hydrocarbon receptor nuclear translocator like 2 [Source:NCBI gene;Acc:709547]	-0.983885287	2.64E-08	2.32E-07
ENSMMUG00000021736	single-pass membrane protein with coiled-coil domains 2 [Source:VGNC Symbol;Acc:VGNC:77808]	1.293552839	3.40E-12	5.23E-11
ENSMMUG00000054181		-1.420131848	0.012942704	0.033768525
ENSMMUG00000053437		-0.441119303	0.004809969	0.014378519
ENSMMUG00000001361	importin 8 [Source:VGNC Symbol;Acc:VGNC:73815]	-0.774292211	0.016892404	0.042366709
ENSMMUG00000001359	caprin family member 2 [Source:VGNC Symbol;Acc:VGNC:70599]	-0.848010642	0.00099802	0.003597561
ENSMMUG00000001357	tetraspanin 11 [Source:VGNC Symbol;Acc:VGNC:79882]	-1.103863244	1.55E-13	2.82E-12
ENSMMUG00000009155	SIN3-HDAC complex associated factor [Source:NCBI gene;Acc:694162]	-2.207431186	9.37E-07	6.39E-06

ENSMMUG00000039300		-1.527978695	0.005387232	0.015826594
ENSMMUG00000003221	DENN domain containing 5B [Source:VGNC Symbol;Acc:VGNC:71772]	-0.968109957	0.000277037	0.001142192
ENSMMUG00000063112		1.849432139	3.16E-06	1.95E-05
ENSMMUG00000013655	dynamin 1 like [Source:VGNC Symbol;Acc:VGNC:71956]	-1.515999295	0.001591384	0.005424091
ENSMMUG00000060125		-2.742723827	0.007416712	0.020870937
ENSMMUG00000021163	contactin 1 [Source:VGNC Symbol;Acc:VGNC:71283]	-0.454971028	2.40E-05	0.000125317
ENSMMUG00000056303		-2.47229055	7.61E-06	4.37E-05
ENSMMUG00000000205	transmembrane protein 117 [Source:VGNC Symbol;Acc:VGNC:79488]	-1.223211354	5.12E-17	1.33E-15
ENSMMUG00000015962	AT-rich interaction domain 2 [Source:VGNC Symbol;Acc:VGNC:69858]	-2.191432201	1.03E-17	2.88E-16
ENSMMUG00000037874	Metazoan signal recognition particle RNA [Source:RFAM;Acc:RF00017]	-1.511384335	0.000237723	0.000997298
ENSMMUG00000023791	SR-related CTD associated factor 11 [Source:VGNC Symbol;Acc:VGNC:77002]	-2.926818835	4.13E-06	2.49E-05
ENSMMUG00000022359	solute carrier family 38 member 2 [Source:VGNC Symbol;Acc:VGNC:77469]	-0.796649636	0.010349855	0.027871499
ENSMMUG00000064730		2.845827604	3.48E-26	2.35E-24
ENSMMUG00000012331	solute carrier family 38 member 4 [Source:VGNC Symbol;Acc:VGNC:77484]	-0.822807761	7.66E-05	0.000357686
ENSMMUG00000049514	solute carrier family 48 member 1 [Source:NCBI gene;Acc:704304]	1.249417439	7.28E-09	6.99E-08
ENSMMUG00000021655	histone deacetylase 7 [Source:NCBI gene;Acc:718225]	1.063600284	2.61E-13	4.58E-12
ENSMMUG00000006727	vitamin D receptor [Source:VGNC Symbol;Acc:VGNC:78764]	0.66223989	6.75E-05	0.000318892
ENSMMUG00000011441	transmembrane protein 106C [Source:VGNC Symbol;Acc:VGNC:79741]	2.706526215	0.000126887	0.000565423
ENSMMUG00000020125	SUMO specific peptidase 1 [Source:NCBI gene;Acc:707603]	-1.575175195	0.002161028	0.007099701
ENSMMUG00000014484	zinc finger protein 641 [Source:NCBI gene;Acc:708246]	-2.279038202	4.07E-16	9.54E-15
ENSMMUG00000003877	KAT8 regulatory NSL complex subunit 2 [Source:VGNC Symbol;Acc:VGNC:73910]	-1.074996688	0.001726832	0.005842782
ENSMMUG00000054025	testis expressed 49 [Source:NCBI gene;Acc:709603]	-0.460213122	0.008803108	0.024270326
ENSMMUG00000012769	limb development membrane protein 1 like [Source:VGNC Symbol;Acc:VGNC:74310]	-0.96996561	1.58E-06	1.03E-05
ENSMMUG00000021081	tubulin alpha 1b [Source:NCBI gene;Acc:709643]	-0.900131904	0.000128393	0.00057128
ENSMMUG00000028047	Y RNA [Source:RFAM;Acc:RF00019]	-1.557600221	0.005328651	0.015673124
ENSMMUG00000009788	tubulin alpha-1C chain [Source:NCBI gene;Acc:710110]	0.863060538	0.002294568	0.007488653
ENSMMUG00000031976	peripherin [Source:NCBI gene;Acc:709827]	0.765810851	0.013297949	0.034555562
ENSMMUG00000007544	trophinin associated protein [Source:VGNC Symbol;Acc:VGNC:78650]	0.912714388	0.000213295	0.000904796
ENSMMUG00000007546	complement C1q like 4 [Source:VGNC Symbol;Acc:VGNC:70450]	1.284632433	5.95E-05	0.000284644
ENSMMUG00000007548	DnaJ heat shock protein family (Hsp40) member C22 [Source:NCBI gene;Acc:710205]	1.109558489	0.003084905	0.009723517
ENSMMUG00000064430		0.941074154	4.22E-05	0.000207987
ENSMMUG00000049374		0.988892777	4.87E-08	4.10E-07
ENSMMUG00000038256	aquaporin 2 [Source:NCBI gene;Acc:711719]	1.043057252	2.37E-14	4.69E-13
ENSMMUG00000021688	aquaporin 6 [Source:VGNC Symbol;Acc:VGNC:69994]	-1.946269939	0.011824335	0.031274279
ENSMMUG00000021689	Rac GTPase activating protein 1 [Source:NCBI gene;Acc:711887]	0.720524974	2.56E-05	0.000132941
ENSMMUG00000006529	acid sensing ion channel subunit 1 [Source:VGNC Symbol;Acc:VGNC:70035]	-1.099327797	0.006716535	0.0191661

ENSMMUG00000006531	SWI/SNF related, matrix associated, actin dependent regulator of chromatin, subfamily d, member 1 [Source:VGNC Symbol;Acc:VGNC:77653]	-0.981241412	0.000149212	0.000653134
ENSMMUG00000018485	methyltransferase like 7A [Source:VGNC Symbol;Acc:VGNC:74567]	1.577644709	0.000395361	0.001577401
ENSMMUG00000027375	Y RNA [Source:RFAM;Acc:RF00019]	1.264985072	0.000231603	0.00097465
ENSMMUG00000022961	solute carrier family 11 member 2 [Source:VGNC Symbol;Acc:VGNC:77383]	0.989648778	0.001957214	0.006531387
ENSMMUG00000012453	LETM1 domain containing 1 [Source:VGNC Symbol;Acc:VGNC:74109]	0.484864869	0.000285314	0.001171105
ENSMMUG00000000152	transcription factor CP2 [Source:VGNC Symbol;Acc:VGNC:78323]	1.800645684	0.000262463	0.001089371
ENSMMUG00000004982	activin A receptor like type 1 [Source:VGNC Symbol;Acc:VGNC:69451]	2.456973644	0.003590411	0.011109075
ENSMMUG00000022922	nuclear receptor subfamily 4 group A member 1 [Source:VGNC Symbol;Acc:VGNC:75517]	-0.301373557	0.005945441	0.017244352
ENSMMUG00000010000	keratin 7 [Source:VGNC Symbol;Acc:VGNC:74214]	-0.67994275	3.18E-09	3.23E-08
ENSMMUG00000064514	keratin, type II cuticular Hb1 [Source:NCBI gene;Acc:697511]	-0.76140753	4.63E-06	2.77E-05
ENSMMUG00000057841	keratin 86 [Source:NCBI gene;Acc:697770]	0.923517386	1.81E-05	9.71E-05
ENSMMUG00000018217	keratin 84 [Source:VGNC Symbol;Acc:VGNC:74220]	-1.155962578	0.000583428	0.002227086
ENSMMUG00000018219	keratin 75 [Source:VGNC Symbol;Acc:VGNC:74113]	1.159027477	0.011802189	0.031237992
ENSMMUG00000057134	keratin, type II cytoskeletal 6C [Source:NCBI gene;Acc:100424162]	1.174964748	5.70E-06	3.36E-05
ENSMMUG00000055016	keratin, type II cytoskeletal 6A [Source:NCBI gene;Acc:719283]	0.793235938	1.40E-12	2.27E-11
ENSMMUG00000014400	keratin 71 [Source:VGNC Symbol;Acc:VGNC:74147]	0.431114249	0.001431067	0.004947023
ENSMMUG00000002304	keratin 73 [Source:VGNC Symbol;Acc:VGNC:74148]	1.305509661	3.14E-05	0.000159288
ENSMMUG00000002305	keratin 2 [Source:VGNC Symbol;Acc:VGNC:74212]	0.893574519	0.013464238	0.03494481
ENSMMUG00000016962	keratin 1 [Source:VGNC Symbol;Acc:VGNC:74206]	0.77378229	0.000185465	0.000796076
ENSMMUG00000045353	keratin, type II cytoskeletal 1b [Source:NCBI gene;Acc:718983]	0.742117487	0.007589592	0.021329098
ENSMMUG00000014980	keratin 76 [Source:VGNC Symbol;Acc:VGNC:74112]	-0.997951335	0.000574348	0.00219752
ENSMMUG00000005246	zinc finger protein 740 [Source:VGNC Symbol;Acc:VGNC:79665]	-2.423545168	1.75E-11	2.43E-10
ENSMMUG00000052817	Sp7 transcription factor [Source:NCBI gene;Acc:702575]	-1.621434926	0.001136123	0.004040457
ENSMMUG00000020036	Sp1 transcription factor [Source:VGNC Symbol;Acc:VGNC:77844]	-1.69436394	3.49E-10	4.07E-09
ENSMMUG00000018492	TARBP2 subunit of RISC loading complex [Source:VGNC Symbol;Acc:VGNC:78099]	2.145975925	1.39E-20	5.28E-19
ENSMMUG00000022313	activating transcription factor 7 [Source:NCBI gene;Acc:700403]	2.216343225	2.66E-20	9.70E-19
ENSMMUG00000055610	homeobox C4 [Source:NCBI gene;Acc:100430434]	-2.732844926	1.26E-21	5.43E-20
ENSMMUG00000000108	NCK associated protein 1 like [Source:VGNC Symbol;Acc:VGNC:75063]	3.512417843	0.000208095	0.000883747
ENSMMUG00000052854	mucin like 1 [Source:NCBI gene;Acc:100423942]	1.750962671	1.75E-17	4.79E-16
ENSMMUG00000016203	thymocyte expressed, positive selection associated 1 [Source:VGNC Symbol;Acc:VGNC:79775]	0.501259438	0.001048693	0.003754734
ENSMMUG00000055590		-1.12539132	2.14E-09	2.23E-08
ENSMMUG00000006293	SAP domain containing ribonucleoprotein [Source:NCBI gene;Acc:709932]	1.109676682	3.26E-13	5.68E-12
ENSMMUG00000053458	ORMDL sphingolipid biosynthesis regulator 2 [Source:NCBI gene;Acc:710296]	0.953237341	1.43E-13	2.60E-12
ENSMMUG00000051937	uncharacterized LOC707807 [Source:NCBI gene;Acc:707807]	0.808231075	0.001464219	0.005049853
ENSMMUG00000006295	matrix metallopeptidase 19 [Source:VGNC Symbol;Acc:VGNC:74896]	0.911436973	2.04E-06	1.31E-05
ENSMMUG00000064364	sulfite oxidase [Source:NCBI gene;Acc:711164]	-0.721868833	0.020433992	0.049727615
ENSMMUG00000008273	SWI/SNF related, matrix associated, actin dependent regulator of chromatin subfamily c member 2 [Source:VGNC Symbol;Acc:VGNC:77652]	-1.391006342	0.020285894	0.049450352
ENSMMUG00000064075	ring finger protein 41 [Source:NCBI gene;Acc:711969]	-1.14954407	1.54E-05	8.39E-05
ENSMMUG00000054020	U6 spliceosomal RNA [Source:RFAM;Acc:RF00026]	-1.187076873	0.000332963	0.001349138
ENSMMUG00000059663		-1.254392665	4.81E-10	5.53E-09
ENSMMUG00000031834	coenzyme Q10A [Source:VGNC Symbol;Acc:VGNC:71356]	-1.271276328	0.000113868	0.000512026

ENSMMUG00000002483	citrate synthase [Source:VGNC Symbol;Acc:VGNC:71509]	1.672498753	8.74E-07	5.99E-06
ENSMMUG00000013482	poly(A) specific ribonuclease subunit PAN2 [Source:NCBI gene;Acc:712478]	2.387071623	0.00018987	0.000813571
ENSMMUG00000000803	signal transducer and activator of transcription 2 [Source:VGNC Symbol;Acc:VGNC:77905]	2.454713759	6.03E-20	2.10E-18
ENSMMUG00000063898	apolipoprotein F [Source:NCBI gene;Acc:712761]	2.807126941	9.52E-20	3.24E-18
ENSMMUG00000053460		2.508059284	5.36E-17	1.38E-15
ENSMMUG00000021878	timeless circadian regulator [Source:VGNC Symbol;Acc:VGNC:78356]	-1.116003436	1.08E-07	8.68E-07
ENSMMUG00000013766	prostaglandin E synthase 3 [Source:NCBI gene;Acc:713353]	1.421787934	8.26E-16	1.89E-14
ENSMMUG00000004522	DNA primase subunit 1 [Source:NCBI gene;Acc:710253]	0.699005261	5.52E-10	6.29E-09
ENSMMUG00000057617	hydroxysteroid 17-beta dehydrogenase 6 [Source:NCBI gene;Acc:713549]	0.891928717	0.015099613	0.038468864
ENSMMUG00000043411	zinc finger and BTB domain containing 39 [Source:NCBI gene;Acc:713914]	-1.688417103	8.88E-08	7.21E-07
ENSMMUG00000021114	G protein-coupled receptor 182 [Source:VGNC Symbol;Acc:VGNC:72936]	-1.1911484	1.90E-08	1.71E-07
ENSMMUG00000013518	myosin IA [Source:VGNC Symbol;Acc:VGNC:75110]	-0.622707898	5.57E-06	3.28E-05
ENSMMUG00000021782	LDL receptor related protein 1 [Source:VGNC Symbol;Acc:VGNC:74442]	-0.785071544	2.74E-06	1.71E-05
ENSMMUG00000004233	leucine rich repeats and immunoglobulin like domains 3 [Source:NCBI gene;Acc:716344]	-2.424497608	7.45E-23	3.61E-21
ENSMMUG00000055477		-2.290643628	1.85E-11	2.55E-10
ENSMMUG00000062139		1.270134846	2.21E-05	0.000116402
ENSMMUG00000011579	TAFA chemokine like family member 2 [Source:VGNC Symbol;Acc:VGNC:78189]	0.603839498	7.16E-08	5.89E-07
ENSMMUG00000020533	chromosome 11 C12orf56 homolog [Source:VGNC Symbol;Acc:VGNC:70409]	-4.093629281	3.21E-17	8.51E-16
ENSMMUG00000016887	WNT inhibitory factor 1 [Source:VGNC Symbol;Acc:VGNC:79110]	-2.966595098	0.0124703	0.032720196
ENSMMUG00000003669	methionine sulfoxide reductase B3 [Source:VGNC Symbol;Acc:VGNC:74842]	0.842218812	3.19E-08	2.76E-07
ENSMMUG00000041729		1.591893845	5.61E-08	4.69E-07
ENSMMUG00000052321		2.281538238	0.013931451	0.035969568
ENSMMUG00000016621	transmembrane BAX inhibitor motif containing 4 [Source:NCBI gene;Acc:717825]	4.212583039	0.000301263	0.001230443
ENSMMUG00000054816	interleukin 1 receptor associated kinase 3 [Source:NCBI gene;Acc:717845]	3.613607853	0.000100193	0.000458174
ENSMMUG00000016631	glutamate receptor interacting protein 1 [Source:VGNC Symbol;Acc:VGNC:73195]	-1.140158265	0.001830798	0.006163618
ENSMMUG00000011650	dual specificity tyrosine phosphorylation regulated kinase 2 [Source:VGNC Symbol;Acc:VGNC:72018]	2.359354453	3.96E-16	9.32E-15
ENSMMUG00000059548		2.153906848	0.000356187	0.001435005
ENSMMUG00000014186	nucleoporin 107 [Source:VGNC Symbol;Acc:VGNC:75488]	-0.554058573	0.008830806	0.02433312
ENSMMUG00000014187	solute carrier family 35 member E3 [Source:VGNC Symbol;Acc:VGNC:77542]	-0.643051424	0.012385234	0.032548826
ENSMMUG00000055447	MDM2 proto-oncogene [Source:NCBI gene;Acc:718284]	-1.027487441	0.000111039	0.000500977
ENSMMUG00000053722		3.470985062	1.16E-12	1.90E-11
ENSMMUG00000010819	fibroblast growth factor receptor substrate 2 [Source:VGNC Symbol;Acc:VGNC:72732]	-1.301988716	1.03E-12	1.69E-11
ENSMMUG00000010820	chaperonin containing TCP1 subunit 2 [Source:VGNC Symbol;Acc:VGNC:70745]	-2.582517348	9.18E-15	1.93E-13
ENSMMUG00000054953	leucine rich repeat containing 10 [Source:NCBI gene;Acc:114669753]	-4.226512381	2.90E-07	2.15E-06
ENSMMUG00000012289	GLIPR1 like 1 [Source:VGNC Symbol;Acc:VGNC:81412]	-0.912602304	0.000135635	0.000599956
ENSMMUG00000012290	GLIPR1 like 2 [Source:VGNC Symbol;Acc:VGNC:81413]	-1.57004101	1.93E-06	1.24E-05
ENSMMUG00000051874		0.709908164	8.24E-07	5.67E-06
ENSMMUG00000015583	nucleosome assembly protein 1 like 1 [Source:NCBI gene;Acc:719047]	1.416655527	4.79E-05	0.000233683

ENSMMUG00000017864	oxysterol binding protein like 8 [Source:VGNC Symbol;Acc:VGNC:75630]	1.037452157	0.009679123	0.026299265
ENSMMUG00000059985		2.672583673	0.011435483	0.030413954
ENSMMUG00000005530	synaptotagmin 1 [Source:VGNC Symbol;Acc:VGNC:78074]	1.11813261	0.00020456	0.00087073
ENSMMUG00000050266		-1.501249008	0.014567674	0.037325259
ENSMMUG00000063005	coiled-coil domain containing 59 [Source:NCBI gene;Acc:698443]	-0.958958724	0.008570383	0.023703614
ENSMMUG00000002514	methyltransferase like 25 [Source:VGNC Symbol;Acc:VGNC:74591]	-1.680201084	0.011492552	0.03051648
ENSMMUG00000059068		-1.084208707	0.006252409	0.01802551
ENSMMUG00000056953		-2.233041247	0.002442566	0.007922847
ENSMMUG00000038410		-1.873146803	2.58E-05	0.000133694
ENSMMUG00000063190		0.688454532	0.000514835	0.0019966
ENSMMUG00000003574	POC1 centriolar protein B [Source:NCBI gene;Acc:709011]	0.507459759	0.000415501	0.001648885
ENSMMUG00000023378	ATPase plasma membrane Ca2+ transporting 1 [Source:VGNC Symbol;Acc:VGNC:70180]	1.208307361	0.00988679	0.026785002
ENSMMUG00000055284		-2.057965882	0.000298847	0.001221245
ENSMMUG00000064177		-2.1155203	0.002655288	0.008523358
ENSMMUG00000049117	CLLU1 antisense RNA 1 [Source:NCBI gene;Acc:100424000]	-4.095953088	9.26E-05	0.000425954
ENSMMUG00000002243	pleckstrin homology and RhoGEF domain containing G7 [Source:VGNC Symbol;Acc:VGNC:76149]	-1.027933213	2.64E-08	2.32E-07
ENSMMUG00000002244	early endosome antigen 1 [Source:VGNC Symbol;Acc:VGNC:72161]	-1.18277455	3.41E-08	2.93E-07
ENSMMUG00000052496		-0.978789704	4.13E-08	3.50E-07
ENSMMUG00000055071	Y RNA [Source:RFAM;Acc:RF00019]	-2.208359223	6.05E-05	0.000288966
ENSMMUG00000018237	plexin C1 [Source:VGNC Symbol;Acc:VGNC:76190]	2.470342626	7.13E-55	1.06E-51
ENSMMUG00000018239	centrosomal protein 83 [Source:VGNC Symbol;Acc:VGNC:70992]	2.469077915	1.72E-32	2.18E-30
ENSMMUG00000056553		-1.020119317	0.002982534	0.009451065
ENSMMUG00000060793		-0.447135879	0.020202162	0.049300324
ENSMMUG00000018898	nuclear receptor subfamily 2 group C member 1 [Source:VGNC Symbol;Acc:VGNC:75508]	-1.172995475	0.000234268	0.000985307
ENSMMUG00000009303	coiled-coil domain containing 38 [Source:VGNC Symbol;Acc:VGNC:70880]	-1.529145923	5.42E-09	5.29E-08
ENSMMUG00000009308	histidine ammonia-lyase [Source:VGNC Symbol;Acc:VGNC:73344]	1.879877801	0.001340214	0.004674358
ENSMMUG00000009312	leukotriene A4 hydrolase [Source:VGNC Symbol;Acc:VGNC:74453]	2.498150682	4.76E-18	1.37E-16
ENSMMUG00000053376		1.376705689	7.56E-08	6.20E-07
ENSMMUG00000050585		1.086414678	0.000223374	0.000942162
ENSMMUG00000018145	ETS transcription factor ELK3 [Source:VGNC Symbol;Acc:VGNC:72210]	-0.466962928	0.01160384	0.030784428
ENSMMUG00000053956	Y RNA [Source:RFAM;Acc:RF00019]	-0.648665561	0.009857771	0.026717148
ENSMMUG00000023719	thymopoietin [Source:VGNC Symbol;Acc:VGNC:78574]	1.156861513	3.98E-06	2.41E-05
ENSMMUG00000051674	IKBKB interacting protein [Source:NCBI gene;Acc:693896]	0.398456267	0.006383847	0.018354488
ENSMMUG00000022404	apoptotic peptidase activating factor 1 [Source:VGNC Symbol;Acc:VGNC:69975]	0.726819811	1.18E-10	1.47E-09
ENSMMUG00000022406	ankyrin repeat and sterile alpha motif domain containing 1B [Source:VGNC Symbol;Acc:VGNC:69689]	1.591568843	3.25E-20	1.16E-18
ENSMMUG00000023601	UHRF1 binding protein 1 like [Source:VGNC Symbol;Acc:VGNC:79774]	0.564803501	0.005088586	0.015068635
ENSMMUG00000063774		0.759156293	0.012785374	0.033422722

ENSMMUG00000061260	Spi-C transcription factor [Source:NCBI gene;Acc:695809]	-1.33428214	5.63E-12	8.39E-11
ENSMMUG00000042077	DNA damage regulated autophagy modulator 1 [Source:NCBI gene;Acc:697427]	0.899102756	2.10E-14	4.20E-13
ENSMMUG00000046478	WASH complex subunit 3 [Source:NCBI gene;Acc:697824]	2.912783899	3.67E-50	3.63E-47
ENSMMUG00000061923		2.389544245	2.92E-09	2.99E-08
ENSMMUG00000005504	nucleoporin 37 [Source:VGNC Symbol;Acc:VGNC:75491]	2.559302748	2.14E-14	4.28E-13
ENSMMUG00000005506	PARP1 binding protein [Source:VGNC Symbol;Acc:VGNC:75682]	0.701622577	0.018923167	0.04660089
ENSMMUG00000038958	Metazoan signal recognition particle RNA [Source:RFAM;Acc:RF00017]	-2.220173267	0.004993694	0.014838006
ENSMMUG00000023469	heat shock protein 90 beta family member 1 [Source:NCBI gene;Acc:699568]	-1.313068038	0.000466072	0.001826598
ENSMMUG00000021291	thymine DNA glycosylase [Source:VGNC Symbol;Acc:VGNC:78297]	-1.372803326	0.000239329	0.001002895
ENSMMUG00000021292	glycosyltransferase 8 domain containing 2 [Source:VGNC Symbol;Acc:VGNC:73004]	-0.959963866	0.007757784	0.021744081
ENSMMUG00000057796	host cell factor C2 [Source:NCBI gene;Acc:700316]	-0.372804006	0.012218671	0.032179523
ENSMMUG00000022570	nuclear transcription factor Y subunit beta [Source:NCBI gene;Acc:700451]	-0.938041014	0.000131273	0.000583309
ENSMMUG00000012091	thioredoxin reductase 1 [Source:VGNC Symbol;Acc:VGNC:78679]	-1.036820089	2.37E-09	2.45E-08
ENSMMUG00000017127	carbohydrate sulfotransferase 11 [Source:VGNC Symbol;Acc:VGNC:82123]	0.907205792	0.002311485	0.007537231
ENSMMUG00000017129	solute carrier family 41 member 2 [Source:VGNC Symbol;Acc:VGNC:77585]	1.342385838	4.31E-13	7.44E-12
ENSMMUG00000009519	WASH complex subunit 4 [Source:VGNC Symbol;Acc:VGNC:79783]	-1.232723273	1.21E-05	6.71E-05
ENSMMUG00000009524	adaptor protein, phosphotyrosine interacting with PH domain and leucine zipper 2 [Source:VGNC Symbol;Acc:VGNC:69861]	-1.262585345	1.19E-05	6.62E-05
ENSMMUG00000005208	RNA polymerase III subunit B [Source:VGNC Symbol;Acc:VGNC:76143]	2.580267861	7.48E-17	1.90E-15
ENSMMUG00000022118	regulatory factor X4 [Source:VGNC Symbol;Acc:VGNC:76909]	-1.411317748	2.60E-05	0.000134511
ENSMMUG00000022278	mitochondrial transcription termination factor 2 [Source:VGNC Symbol;Acc:VGNC:74868]	-0.857660756	0.017001865	0.042569248
ENSMMUG00000021808	cryptochrome circadian regulator 1 [Source:VGNC Symbol;Acc:VGNC:71499]	-1.114691599	3.64E-09	3.65E-08
ENSMMUG00000015540	BTB domain containing 11 [Source:VGNC Symbol;Acc:VGNC:70367]	-1.578359209	0.001465528	0.005053192
ENSMMUG00000015544	PWP1 homolog, endonuclein [Source:NCBI gene;Acc:705262]	-2.551902908	0.001258171	0.004418345
ENSMMUG00000015550	PR/SET domain 4 [Source:VGNC Symbol;Acc:VGNC:76393]	-2.384679114	7.95E-09	7.57E-08
ENSMMUG00000021049	ret finger protein-like 3 [Source:NCBI gene;Acc:705507]	-3.4658309	1.36E-05	7.49E-05
ENSMMUG00000017124	spliceosome associated factor 3, U4/U6 recycling protein [Source:VGNC Symbol;Acc:VGNC:77029]	2.71775654	1.03E-19	3.46E-18
ENSMMUG00000017126	iron-sulfur cluster assembly enzyme [Source:VGNC Symbol;Acc:VGNC:73686]	2.407482863	0.012931223	0.033756387
ENSMMUG00000054621		3.318090527	3.09E-07	2.28E-06
ENSMMUG00000006893	D-amino acid oxidase [Source:VGNC Symbol;Acc:VGNC:71729]	1.543232624	1.86E-13	3.34E-12
ENSMMUG00000015108	SV2 related protein [Source:VGNC Symbol;Acc:VGNC:78208]	1.248922832	8.86E-10	9.78E-09
ENSMMUG00000056729	U6 spliceosomal RNA [Source:RFAM;Acc:RF00026]	1.38228274	2.03E-13	3.63E-12
ENSMMUG00000058267	glycolipid transfer protein [Source:NCBI gene;Acc:701777]	-1.337879586	0.002391132	0.007769606
ENSMMUG00000023032	intraflagellar transport 81 [Source:VGNC Symbol;Acc:VGNC:73389]	1.194967368	1.04E-15	2.35E-14
ENSMMUG00000001739	ATPase sarcoplasmic/endoplasmic reticulum Ca2+ transporting 2 [Source:VGNC Symbol;Acc:VGNC:70178]	1.341570946	4.86E-27	3.72E-25
ENSMMUG00000003297	coiled-coil domain containing 63 [Source:VGNC Symbol;Acc:VGNC:70879]	0.839783385	0.000155294	0.000676361
ENSMMUG00000002347	myosin light chain 2 [Source:VGNC Symbol;Acc:VGNC:75103]	0.551077569	0.013244795	0.034435539
ENSMMUG00000008336	acyl-CoA dehydrogenase family member 10 [Source:NCBI gene;Acc:711080]	0.999149575	2.08E-16	5.06E-15
ENSMMUG00000053496		0.812507903	0.004266364	0.012946629

ENSMMUG00000060754	Metazoan signal recognition particle RNA [Source:RFAM;Acc:RF00017]	1.148968686	3.79E-15	8.20E-14
ENSMMUG00000060917		1.008251048	1.79E-05	9.65E-05
ENSMMUG00000043209	transmembrane protein 116 [Source:NCBI gene;Acc:107001019]	0.751245696	2.82E-05	0.000144887
ENSMMUG00000005255	TRAF-type zinc finger domain containing 1 [Source:VGNC Symbol;Acc:VGNC:79459]	1.510911272	3.35E-12	5.16E-11
ENSMMUG00000013253	protein tyrosine phosphatase non-receptor type 11 [Source:NCBI gene;Acc:712044]	0.723162191	0.00327533	0.010262655
ENSMMUG00000012778	rabphilin 3A [Source:VGNC Symbol;Acc:VGNC:77033]	-1.012270039	0.000162856	0.000707219
ENSMMUG00000004435	two pore segment channel 1 [Source:VGNC Symbol;Acc:VGNC:79117]	0.810191615	0.003449687	0.01072288
ENSMMUG00000004205	phospholipase B domain containing 2 [Source:VGNC Symbol;Acc:VGNC:76122]	-2.909077746	3.31E-06	2.03E-05
ENSMMUG00000004208	serine dehydratase [Source:VGNC Symbol;Acc:VGNC:77148]	-2.284501147	1.72E-05	9.29E-05
ENSMMUG00000061944	LIM homeobox 5 [Source:NCBI gene;Acc:713417]	-1.414224048	0.000121482	0.000543135
ENSMMUG00000051363		1.837073575	4.95E-13	8.49E-12
ENSMMUG00000054768		0.959312957	1.37E-08	1.26E-07
ENSMMUG00000057506		0.819019091	1.77E-06	1.15E-05
ENSMMUG00000052680		0.843637655	2.62E-11	3.55E-10
ENSMMUG00000017478	tescalcin [Source:VGNC Symbol;Acc:VGNC:79571]	-0.740873961	8.81E-05	0.000407428
ENSMMUG00000014027	nitric oxide synthase 1 [Source:VGNC Symbol;Acc:VGNC:75368]	-1.598809873	2.05E-07	1.57E-06
ENSMMUG00000062615		-1.702539655	2.12E-06	1.36E-05
ENSMMUG00000005421	kinase suppressor of ras 2 [Source:VGNC Symbol;Acc:VGNC:74075]	-0.976400729	0.004613622	0.013869858
ENSMMUG00000020629	SDS3 homolog, SIN3A corepressor complex component [Source:NCBI gene;Acc:696166]	1.110854141	2.00E-12	3.17E-11
ENSMMUG00000057515		0.846461517	0.019384608	0.047563491
ENSMMUG00000053710		0.986958139	7.69E-13	1.29E-11
ENSMMUG00000011209	BICD family like cargo adaptor 1 [Source:HGNC Symbol;Acc:HGNC:28095]	1.579786003	2.12E-15	4.67E-14
ENSMMUG00000055209		1.749808829	4.32E-17	1.13E-15
ENSMMUG00000063609	cytochrome c oxidase subunit 6A1, mitochondrial [Source:NCBI gene;Acc:696956]	-2.636023325	0.005351743	0.015728568
ENSMMUG00000046665	glutamyl-tRNA amidotransferase subunit C [Source:NCBI gene;Acc:702258]	-2.219800187	2.76E-07	2.06E-06
ENSMMUG00000052631		-2.976457248	0.010635661	0.02850142
ENSMMUG00000049873		-3.124569703	0.010571566	0.028365536
ENSMMUG00000046646	Y RNA [Source:RFAM;Acc:RF00019]	0.5482755	0.008381076	0.023266822
ENSMMUG00000004223	ring finger protein 10 [Source:VGNC Symbol;Acc:VGNC:76702]	-0.764651085	1.94E-06	1.25E-05
ENSMMUG00000049342	POP5 homolog, ribonuclease P/MRP subunit [Source:NCBI gene;Acc:697559]	-1.828291519	0.006802451	0.01938888
ENSMMUG00000055513		-1.524217532	0.006140348	0.017740377
ENSMMUG00000058536		2.613061808	3.88E-28	3.25E-26
ENSMMUG00000002536	calcium binding protein 1 [Source:VGNC Symbol;Acc:VGNC:70495]	2.233455791	3.50E-19	1.10E-17
ENSMMUG00000065224	unc-119 lipid binding chaperone B [Source:NCBI gene;Acc:698066]	-1.360795198	0.009640681	0.02620922
ENSMMUG00000006435	HNF1 homeobox A [Source:NCBI gene;Acc:107001057]	0.898459942	0.000176078	0.000758859
ENSMMUG00000039520	chromosome 11 C12orf43 homolog [Source:NCBI gene;Acc:698821]	1.239679523	6.81E-11	8.81E-10
ENSMMUG00000060614		-0.480305383	0.008843588	0.024354766

ENSMMUG00000021564	lysine demethylase 2B [Source:VGNC Symbol;Acc:VGNC:81425]	2.281593959	6.35E-12	9.33E-11
ENSMMUG00000059782		3.002394334	1.83E-14	3.69E-13
ENSMMUG00000038707	U6 spliceosomal RNA [Source:RFAM;Acc:RF00026]	2.979892913	0.00877256	0.0241986
ENSMMUG00000041124	MORN repeat containing 3 [Source:NCBI gene;Acc:700317]	0.739312942	0.017568712	0.04372288
ENSMMUG00000063813	Metazoan signal recognition particle RNA [Source:RFAM;Acc:RF00017]	1.290016608	0.008432768	0.023392809
ENSMMUG00000012842	SET domain containing 1B, histone lysine methyltransferase [Source:NCBI gene;Acc:703717]	2.428089651	7.07E-05	0.00033275
ENSMMUG00000016454	4-hydroxyphenylpyruvate dioxygenase [Source:VGNC Symbol;Acc:VGNC:73511]	1.257255337	2.84E-23	1.46E-21
ENSMMUG00000046409	VPS33A core subunit of CORVET and HOPS complexes [Source:NCBI gene;Acc:702257]	-0.398316913	0.010637788	0.028501969
ENSMMUG00000013643	zinc finger CCHC-type containing 8 [Source:VGNC Symbol;Acc:VGNC:79487]	-1.309970584	0.006277903	0.018084947
ENSMMUG00000004412	arginine and serine rich coiled-coil 2 [Source:VGNC Symbol;Acc:VGNC:77139]	-1.929277764	0.000182177	0.000783323
ENSMMUG00000007556	kinetochore associated 1 [Source:VGNC Symbol;Acc:VGNC:74064]	0.62997266	0.000655193	0.002469881
ENSMMUG00000017102	coiled-coil domain containing 62 [Source:NCBI gene;Acc:707179]	0.899702177	7.50E-05	0.000350854
ENSMMUG00000015221	huntingtin interacting protein 1 related [Source:VGNC Symbol;Acc:VGNC:73412]	0.996593892	2.65E-05	0.000137202
ENSMMUG00000039447	VPS37B subunit of ESCRT-I [Source:NCBI gene;Acc:703415]	1.200009079	0.009044321	0.024829188
ENSMMUG00000005301	ATP binding cassette subfamily B member 9 [Source:VGNC Symbol;Acc:VGNC:81341]	-1.465463677	7.37E-13	1.24E-11
ENSMMUG00000015926	2-oxoglutarate and iron dependent oxygenase domain containing 2 [Source:VGNC Symbol;Acc:VGNC:75462]	1.885014114	2.09E-10	2.53E-09
ENSMMUG00000015930	ADP ribosylation factor like GTPase 6 interacting protein 4 [Source:VGNC Symbol;Acc:VGNC:69860]	1.702084819	2.84E-05	0.000145897
ENSMMUG00000015931	phosphatidylinositol transfer protein membrane associated 2 [Source:VGNC Symbol;Acc:VGNC:75843]	1.614846864	8.69E-14	1.64E-12
ENSMMUG00000019678	M-phase phosphoprotein 9 [Source:VGNC Symbol;Acc:VGNC:74918]	-0.661403609	0.00154799	0.005300551
ENSMMUG00000031495	chromosome 11 C12orf65 homolog [Source:NCBI gene;Acc:708974]	-1.476803063	0.007676144	0.02153968
ENSMMUG00000000434	cyclin dependent kinase 2 associated protein 1 [Source:VGNC Symbol;Acc:VGNC:70944]	-1.689636101	1.02E-06	6.90E-06
ENSMMUG00000013514	lysine methyltransferase 5A [Source:VGNC Symbol;Acc:VGNC:74159]	-0.415699205	0.006174237	0.01782787
ENSMMUG00000023048	cytochrome b-c1 complex subunit Rieske, mitochondrial [Source:NCBI gene;Acc:705178]	1.853355607	3.59E-09	3.61E-08
ENSMMUG00000058836	transmembrane p24 trafficking protein 2 [Source:NCBI gene;Acc:106992483]	-1.217407016	3.68E-07	2.68E-06
ENSMMUG00000004592	general transcription factor IIH subunit 3 [Source:NCBI gene;Acc:106992484]	-3.070630648	0.013323396	0.03461562
ENSMMUG00000064225	tectonic family member 2 [Source:NCBI gene;Acc:721415]	0.583337255	1.99E-05	0.000106031
ENSMMUG00000006546	ATPase H+ transporting V0 subunit a2 [Source:VGNC Symbol;Acc:VGNC:70096]	-0.403914746	0.000567749	0.002174513
ENSMMUG00000054430		-1.025661562	0.014230074	0.036542337
ENSMMUG00000006861	dynein axonemal heavy chain 10 [Source:NCBI gene;Acc:710343]	-0.745311191	0.000109896	0.000496725
ENSMMUG00000046631	refilin A [Source:NCBI gene;Acc:706153]	-2.15654871	0.012665479	0.033161909
ENSMMUG00000017902	DEAH-box helicase 37 [Source:VGNC Symbol;Acc:VGNC:71639]	2.691963446	0.012520413	0.032828451
ENSMMUG00000020804	transmembrane protein 132B [Source:NCBI gene;Acc:707118]	-0.769651133	0.014961705	0.038189689
ENSMMUG00000050145	Metazoan signal recognition particle RNA [Source:RFAM;Acc:RF00017]	-3.693576927	0.001086018	0.003876208
ENSMMUG00000020926	syntaxin 2 [Source:VGNC Symbol;Acc:VGNC:78259]	-4.361178827	7.93E-07	5.48E-06
ENSMMUG00000058674		-3.595666006	0.004406523	0.013314738
ENSMMUG00000003735	polypeptide N-acetylgalactosaminyltransferase 9 [Source:NCBI gene;Acc:710382]	-0.872188518	0.011654429	0.030902053
ENSMMUG00000017026	fibrosin like 1 [Source:NCBI gene;Acc:696295]	0.869947273	0.000332387	0.00134717

ENSMMUG00000015463	DNA polymerase epsilon, catalytic subunit [Source:NCBI gene;Acc:698023]	-0.746180468	7.24E-06	4.18E-05
ENSMMUG00000015468	checkpoint with forkhead and ring finger domains [Source:VGNC Symbol;Acc:VGNC:71064]	2.059309298	0.00036877	0.001480469
ENSMMUG00000013867	zinc finger protein 84 [Source:VGNC Symbol;Acc:VGNC:78909]	-2.472563399	0.018457425	0.045612999
ENSMMUG00000009615	IMP U3 small nucleolar ribonucleoprotein 4 [Source:VGNC Symbol;Acc:VGNC:73689]	-0.946850871	7.00E-06	4.05E-05
ENSMMUG00000021159	tubulin alpha 3e [Source:NCBI gene;Acc:718034]	-0.966410389	0.000395299	0.001577401
ENSMMUG00000061826	family with sequence similarity 168 member B [Source:NCBI gene;Acc:703758]	0.854695243	0.000797508	0.002936938
ENSMMUG00000023321	UDP-glucose glycoprotein glucosyltransferase 1 [Source:VGNC Symbol;Acc:VGNC:78981]	0.614276438	0.002534972	0.008181422
ENSMMUG00000049232	AMMECR1 like [Source:VGNC Symbol;Acc:VGNC:84208]	4.443617047	0.000819606	0.003009349
ENSMMUG00000001989	RNA polymerase II subunit D [Source:VGNC Symbol;Acc:VGNC:76213]	4.836963104	1.98E-05	0.000105489
ENSMMUG00000027543	Y RNA [Source:RFAM;Acc:RF00019]	4.615892424	0.000262456	0.001089371
ENSMMUG00000004179	LIM zinc finger domain containing 2 [Source:VGNC Symbol;Acc:VGNC:74054]	0.495134382	0.002232794	0.007307952
ENSMMUG00000004177	myosin VIIB [Source:NCBI gene;Acc:714563]	0.754927406	4.76E-17	1.24E-15
ENSMMUG00000056151		2.261527186	0.000167256	0.000724206
ENSMMUG00000000376	bridging integrator 1 [Source:VGNC Symbol;Acc:VGNC:70251]	2.475777202	1.05E-05	5.86E-05
ENSMMUG00000021119	translin [Source:VGNC Symbol;Acc:VGNC:78663]	-0.550996192	0.002973126	0.009426871
ENSMMUG00000020928	nucleolar protein interacting with the FHA domain of MKI67 [Source:VGNC Symbol;Acc:VGNC:75201]	-1.092414772	2.60E-05	0.000134511
ENSMMUG00000046774	RAS like proto-oncogene B [Source:NCBI gene;Acc:695852]	2.174968701	5.38E-18	1.54E-16
ENSMMUG00000059182		2.258299756	1.89E-22	8.94E-21
ENSMMUG00000063759	transmembrane protein 185B [Source:NCBI gene;Acc:695744]	2.218285714	1.60E-20	6.05E-19
ENSMMUG00000059504		2.740905142	3.07E-16	7.30E-15
ENSMMUG00000051751		0.516785762	0.015218386	0.038704967
ENSMMUG00000005610	protein tyrosine phosphatase non-receptor type 4 [Source:VGNC Symbol;Acc:VGNC:76605]	-1.403274758	6.10E-06	3.57E-05
ENSMMUG00000006446	transmembrane protein 177 [Source:VGNC Symbol;Acc:VGNC:79686]	-3.367674749	1.32E-05	7.25E-05
ENSMMUG00000011129	cilia and flagella associated protein 221 [Source:VGNC Symbol;Acc:VGNC:71153]	-2.934199176	0.001749052	0.005911223
ENSMMUG00000008875	solute carrier family 35 member F5 [Source:VGNC Symbol;Acc:VGNC:77567]	1.375657055	1.82E-11	2.52E-10
ENSMMUG00000043114	U6 spliceosomal RNA [Source:RFAM;Acc:RF00026]	0.82703525	0.002381538	0.007743522
ENSMMUG00000012248	UBX domain protein 4 [Source:VGNC Symbol;Acc:VGNC:78968]	-1.31390356	0.007028994	0.019950237
ENSMMUG00000012250	lactase [Source:VGNC Symbol;Acc:VGNC:74244]	-1.148235012	1.48E-05	8.07E-05
ENSMMUG00000017486	minichromosome maintenance complex component 6 [Source:VGNC Symbol;Acc:VGNC:74685]	-1.542396423	2.65E-09	2.72E-08
ENSMMUG00000040854	aspartyl-tRNA synthetase 1 [Source:VGNC Symbol;Acc:VGNC:84226]	-1.153736632	1.88E-05	0.000100407
ENSMMUG00000006528	Rho GTPase activating protein 15 [Source:VGNC Symbol;Acc:VGNC:69874]	0.614847027	7.14E-08	5.88E-07
ENSMMUG00000016845	zinc finger E-box binding homeobox 2 [Source:VGNC Symbol;Acc:VGNC:78969]	-1.907592692	0.006425293	0.018452188
ENSMMUG00000051691		1.798389217	4.17E-05	0.000205758
ENSMMUG00000050074		-1.094050503	0.002468458	0.007991102
ENSMMUG00000008615	nebulin [Source:NCBI gene;Acc:100430324]	0.868739469	7.62E-09	7.29E-08
ENSMMUG00000010526	calcium voltage-gated channel auxiliary subunit beta 4 [Source:VGNC Symbol;Acc:VGNC:82116]	0.908998985	0.00400936	0.012259548
ENSMMUG00000014176	cytohesin 1 interacting protein [Source:VGNC Symbol;Acc:VGNC:71831]	-1.209909875	3.66E-05	0.000183102
ENSMMUG00000011638	plakophilin 4 [Source:VGNC Symbol;Acc:VGNC:76153]	-1.730514724	0.001288685	0.00451588

ENSMMUG00000006443		-2.490587728	0.016481197	0.041455618
ENSMMUG00000052818	death associated protein like 1 [Source:NCBI gene;Acc:699071]	-2.805155134	0.000100095	0.00045787
ENSMMUG00000057379		-1.220731387	0.002790147	0.008909964
ENSMMUG00000006180	tetratricopeptide repeat, ankyrin repeat and coiled-coil containing 1 [Source:VGNC Symbol;Acc:VGNC:78238]	2.123041239	4.12E-27	3.18E-25
ENSMMUG00000055201	U6 spliceosomal RNA [Source:RFAM;Acc:RF00026]	2.982754806	0.000910655	0.003301152
ENSMMUG00000061606	Metazoan signal recognition particle RNA [Source:RFAM;Acc:RF00017]	2.079983118	6.39E-07	4.50E-06
ENSMMUG00000006181	WD repeat, sterile alpha motif and U-box domain containing 1 [Source:VGNC Symbol;Acc:VGNC:79634]	1.684093407	1.64E-16	4.02E-15
ENSMMUG00000063868	Metazoan signal recognition particle RNA [Source:RFAM;Acc:RF00017]	1.767268829	1.74E-07	1.35E-06
ENSMMUG00000006182	bromodomain adjacent to zinc finger domain 2B [Source:NCBI gene;Acc:702780]	0.779007117	2.95E-08	2.58E-07
ENSMMUG00000051450	phospholipase A2 receptor 1 [Source:NCBI gene;Acc:700191]	0.961188903	3.83E-14	7.41E-13
ENSMMUG00000002678	integrin subunit beta 6 [Source:VGNC Symbol;Acc:VGNC:73789]	-1.55319569	3.60E-08	3.08E-07
ENSMMUG00000055970		-0.620957385	0.014892736	0.038033283
ENSMMUG00000008384	solute carrier family 4 member 10 [Source:VGNC Symbol;Acc:VGNC:77695]	1.522716052	9.52E-16	2.17E-14
ENSMMUG00000008387	dipeptidyl peptidase 4 [Source:VGNC Symbol;Acc:VGNC:71979]	3.093738815	5.04E-41	1.49E-38
ENSMMUG00000018510	sodium voltage-gated channel alpha subunit 9 [Source:VGNC Symbol;Acc:VGNC:76990]	-2.301375718	0.001883222	0.006318595
ENSMMUG00000013251	xin actin binding repeat containing 2 [Source:VGNC Symbol;Acc:VGNC:78953]	2.449503259	4.81E-05	0.000234438
ENSMMUG00000063463		-2.193574995	9.37E-13	1.55E-11
ENSMMUG00000021775	beta-1,3-galactosyltransferase 1 [Source:VGNC Symbol;Acc:VGNC:70204]	-1.644742393	1.53E-07	1.19E-06
ENSMMUG00000006215	serine/threonine kinase 39 [Source:VGNC Symbol;Acc:VGNC:81587]	-1.628037586	2.37E-08	2.11E-07
ENSMMUG00000013830	glucose-6-phosphatase catalytic subunit 2 [Source:NCBI gene;Acc:709062]	1.989095979	2.11E-13	3.77E-12
ENSMMUG00000013832	ATP binding cassette subfamily B member 11 [Source:VGNC Symbol;Acc:VGNC:81339]	1.964782223	6.78E-08	5.61E-07
ENSMMUG00000020510	kelch like family member 41 [Source:VGNC Symbol;Acc:VGNC:74062]	-0.521080433	0.006854973	0.019512332
ENSMMUG00000020511	FAST kinase domains 1 [Source:VGNC Symbol;Acc:VGNC:72580]	-1.144653444	0.005029871	0.014927554
ENSMMUG00000031398	coiled-coil domain containing 173 [Source:NCBI gene;Acc:709886]	-1.047052906	0.002824919	0.009003558
ENSMMUG00000016256	myosin IIIB [Source:VGNC Symbol;Acc:VGNC:74961]	2.321120136	0.014729645	0.03768817
ENSMMUG00000015198	glutamate decarboxylase 1 [Source:VGNC Symbol;Acc:VGNC:72862]	-0.861777774	5.30E-05	0.000256428
ENSMMUG00000015203	golgi reassembly stacking protein 2 [Source:VGNC Symbol;Acc:VGNC:72957]	-0.845880613	0.001619408	0.005512008
ENSMMUG00000011189	tousled like kinase 1 [Source:VGNC Symbol;Acc:VGNC:78367]	-1.103999507	1.32E-06	8.75E-06
ENSMMUG00000055482		-2.201939013	0.002598113	0.008361547
ENSMMUG00000054176	methyltransferase like 8 [Source:NCBI gene;Acc:695361]	-1.520329916	3.10E-06	1.92E-05
ENSMMUG00000023370	DDB1 and CUL4 associated factor 17 [Source:VGNC Symbol;Acc:VGNC:71709]	2.145424105	5.65E-25	3.39E-23
ENSMMUG00000007301	dynein cytoplasmic 1 intermediate chain 2 [Source:VGNC Symbol;Acc:VGNC:71953]	-0.691553459	6.74E-05	0.000318665
ENSMMUG00000007310	solute carrier family 25 member 12 [Source:VGNC Symbol;Acc:VGNC:77430]	-0.842989671	3.56E-05	0.000178713
ENSMMUG00000007313	histone acetyltransferase 1 [Source:VGNC Symbol;Acc:VGNC:73349]	-2.327413188	3.07E-07	2.26E-06
ENSMMUG00000002370	integrin subunit alpha 6 [Source:VGNC Symbol;Acc:VGNC:73780]	-0.976367909	1.59E-06	1.04E-05

ENSMMUG00000054462		-1.104830423	1.00E-05	5.64E-05
ENSMMUG00000020792	Rap guanine nucleotide exchange factor 4 [Source:VGNC Symbol;Acc:VGNC:76490]	-0.847453824	0.001964251	0.006551922
ENSMMUG00000049482		-0.767668757	0.004553024	0.013709919
ENSMMUG00000023005	Sp9 transcription factor [Source:VGNC Symbol;Acc:VGNC:78027]	-0.725885844	0.010076772	0.027225092
ENSMMUG00000023006	corepressor interacting with RBPJ, 1 [Source:VGNC Symbol;Acc:VGNC:71331]	-1.631179985	6.98E-05	0.00032881
ENSMMUG00000021012	G protein-coupled receptor 155 [Source:VGNC Symbol;Acc:VGNC:72985]	0.585078736	1.05E-05	5.87E-05
ENSMMUG00000053004		0.641485815	0.002033605	0.006749856
ENSMMUG00000009593	WAS/WASL interacting protein family member 1 [Source:VGNC Symbol;Acc:VGNC:78784]	1.851420638	1.10E-17	3.06E-16
ENSMMUG00000033245	U6 spliceosomal RNA [Source:RFAM;Acc:RF00026]	-0.920558849	0.007647698	0.021465446
ENSMMUG00000052177		-2.410742533	0.002698944	0.008652257
ENSMMUG00000065344	homeobox D1 [Source:NCBI gene;Acc:701064]	-1.7410707	1.06E-11	1.51E-10
ENSMMUG00000008502	alkylglycerone phosphate synthase [Source:VGNC Symbol;Acc:VGNC:69747]	-0.383391301	0.011656732	0.030902634
ENSMMUG00000002183	oxysterol binding protein like 6 [Source:VGNC Symbol;Acc:VGNC:75627]	-1.235787431	2.41E-06	1.52E-05
ENSMMUG00000059250	U6 spliceosomal RNA [Source:RFAM;Acc:RF00026]	-3.161597886	0.006022471	0.017440586
ENSMMUG00000002185	protein activator of interferon induced protein kinase EIF2AK2 [Source:VGNC Symbol;Acc:VGNC:76421]	-0.753775849	2.20E-06	1.40E-05
ENSMMUG00000054264	pejvakin [Source:NCBI gene;Acc:708668]	-0.836122083	1.21E-05	6.71E-05
ENSMMUG00000009038	titin [Source:NCBI gene;Acc:703527]	-1.367529491	0.000550619	0.002119856
ENSMMUG00000019690	SEC14 and spectrin domain containing 1 [Source:VGNC Symbol;Acc:VGNC:77267]	3.078967048	4.15E-19	1.30E-17
ENSMMUG00000053406		4.112301632	1.64E-07	1.28E-06
ENSMMUG00000052229		-1.226127834	0.006202194	0.017898138
ENSMMUG00000014277	ITPR interacting domain containing 2 [Source:VGNC Symbol;Acc:VGNC:73584]	1.796018045	1.54E-42	6.00E-40
ENSMMUG00000014394	DnaJ heat shock protein family (Hsp40) member C10 [Source:VGNC Symbol;Acc:VGNC:84229]	-0.860028571	3.02E-05	0.000154458
ENSMMUG00000014397	NCK associated protein 1 [Source:VGNC Symbol;Acc:VGNC:75143]	2.572459686	6.12E-13	1.04E-11
ENSMMUG00000019462	integrin subunit alpha V [Source:VGNC Symbol;Acc:VGNC:73783]	-1.256930429	2.36E-13	4.16E-12
ENSMMUG00000004022	zinc finger SWIM-type containing 2 [Source:VGNC Symbol;Acc:VGNC:79908]	2.631567268	2.36E-19	7.62E-18
ENSMMUG00000014314	ankyrin and armadillo repeat containing [Source:VGNC Symbol;Acc:VGNC:69706]	-0.754392567	0.005870888	0.017054902
ENSMMUG00000014318	PMS1 homolog 1, mismatch repair system component [Source:VGNC Symbol;Acc:VGNC:81506]	1.780491574	0.006788706	0.019357144
ENSMMUG00000061305		-1.424228255	2.22E-07	1.69E-06
ENSMMUG00000003260		-1.893978955	1.35E-19	4.47E-18
ENSMMUG00000051366		-1.142711562	0.000107794	0.000488265
ENSMMUG00000059380		-0.310549019	0.012093434	0.031883666
ENSMMUG00000005612	glutaminase [Source:NCBI gene;Acc:693520]	-1.975594256	0.000216019	0.000914258
ENSMMUG00000005613	signal transducer and activator of transcription 1 [Source:VGNC Symbol;Acc:VGNC:77904]	-0.404927907	0.013059998	0.034008738

ENSMMUG00000001761	myosin IB [Source:VGNC Symbol;Acc:VGNC:75111]	0.519635978	0.01974744	0.048325832
ENSMMUG00000037175	U6 spliceosomal RNA [Source:RFAM;Acc:RF00026]	1.103887346	0.015299395	0.038897656
ENSMMUG00000001475	transmembrane protein with EGF like and two follistatin like domains 2 [Source:VGNC Symbol;Acc:VGNC:78569]	1.506868965	0.005416196	0.015899088
ENSMMUG00000061797	U6 spliceosomal RNA [Source:RFAM;Acc:RF00026]	1.856729488	3.13E-05	0.000158946
ENSMMUG00000061123	Metazoan signal recognition particle RNA [Source:RFAM;Acc:RF00017]	2.018360382	1.45E-06	9.56E-06
ENSMMUG00000005622	dynein axonemal heavy chain 7 [Source:VGNC Symbol;Acc:VGNC:71859]	0.741177568	0.006832254	0.019458867
ENSMMUG00000057432	serine/threonine kinase 17b [Source:NCBI gene;Acc:696908]	-0.735161114	0.017117441	0.042793602
ENSMMUG00000015970	coiled-coil domain containing 150 [Source:VGNC Symbol;Acc:VGNC:84222]	-1.359105487	3.55E-10	4.14E-09
ENSMMUG00000008643	general transcription factor IIIC subunit 3 [Source:VGNC Symbol;Acc:VGNC:73317]	-1.810246811	1.20E-17	3.35E-16
ENSMMUG00000043369	post-GPI attachment to proteins inositol deacylase 1 [Source:NCBI gene;Acc:696475]	-1.130265531	0.003283395	0.010281407
ENSMMUG00000016547	heat shock protein family E (Hsp10) member 1 [Source:NCBI gene;Acc:100425423]	-1.691944369	0.009834462	0.026672472
ENSMMUG00000023809	raftlin family member 2 [Source:VGNC Symbol;Acc:VGNC:76837]	-0.889887549	0.006911159	0.019664715
ENSMMUG00000065120	glutamate dehydrogenase 1, mitochondrial-like [Source:NCBI gene;Acc:697678]	-1.075319421	0.009210809	0.025224423
ENSMMUG00000023810	methionyl-tRNA synthetase 2, mitochondrial [Source:VGNC Symbol;Acc:VGNC:74572]	-1.18194967	1.47E-07	1.16E-06
ENSMMUG00000023811	boule homolog, RNA binding protein [Source:VGNC Symbol;Acc:VGNC:70310]	-1.108743884	0.000152911	0.000667749
ENSMMUG00000051936		-3.346226898	1.99E-08	1.78E-07
ENSMMUG00000050082		-0.539747775	0.019802184	0.048435823
ENSMMUG00000047974	aldehyde oxidase 4 [Source:NCBI gene;Acc:701502]	-0.758149072	3.79E-06	2.30E-05
ENSMMUG00000005835	aldehyde oxidase 2 [Source:NCBI gene;Acc:701613]	-1.36796616	0.010340683	0.027856918
ENSMMUG00000022202	basic leucine zipper and W2 domains 1 [Source:VGNC Symbol;Acc:VGNC:84213]	-2.043178324	9.60E-15	2.01E-13
ENSMMUG00000006639	NGG1 interacting factor 3 like 1 [Source:VGNC Symbol;Acc:VGNC:75166]	-1.858053664	0.002427657	0.007879469
ENSMMUG00000014460	origin recognition complex subunit 2 [Source:VGNC Symbol;Acc:VGNC:75714]	-2.194881384	0.006184875	0.017855109
ENSMMUG00000053805	Metazoan signal recognition particle RNA [Source:RFAM;Acc:RF00017]	-3.104413121	0.000248163	0.001036986
ENSMMUG00000012224	family with sequence similarity 126 member B [Source:VGNC Symbol;Acc:VGNC:72393]	-1.846925524	5.69E-07	4.03E-06
ENSMMUG00000012310	CASP8 and FADD like apoptosis regulator [Source:VGNC Symbol;Acc:VGNC:71019]	-2.223821461	0.000246618	0.001031111
ENSMMUG00000031296	caspase 8 [Source:VGNC Symbol;Acc:VGNC:70547]	-1.156300243	1.99E-06	1.28E-05
ENSMMUG00000018356	flagellum associated containing coiled-coil domains 1 [Source:VGNC Symbol;Acc:VGNC:72508]	-1.877489217	6.70E-06	3.89E-05
ENSMMUG00000018144	C2 calcium-dependent domain-containing protein 6 [Source:NCBI gene;Acc:702140]	2.211807449	8.26E-27	6.16E-25
ENSMMUG00000018151	membrane palmitoylated protein 4 [Source:VGNC Symbol;Acc:VGNC:74734]	1.983194212	0.009769871	0.026516689
ENSMMUG00000000582	NOP58 ribonucleoprotein [Source:VGNC Symbol;Acc:VGNC:75284]	1.302023306	4.70E-11	6.20E-10
ENSMMUG00000035551	small nucleolar RNA, C/D box 11B [Source:HGNC Symbol;Acc:HGNC:33571]	1.418011844	0.000759938	0.002811146
ENSMMUG00000061021		1.512176667	3.13E-06	1.93E-05
ENSMMUG00000038427	Metazoan signal recognition particle RNA [Source:RFAM;Acc:RF00017]	2.386463885	0.016311301	0.041107583
ENSMMUG00000000314	Ras association (RalGDS/AF-6) and pleckstrin homology domains 1 [Source:NCBI gene;Acc:705065]	-1.791439938	0.016911014	0.042391875

ENSMMUG00000014814	par-3 family cell polarity regulator beta [Source:VGNC Symbol;Acc:VGNC:75602]	-2.728825251	0.018164939	0.04501772
ENSMMUG00000059518		-1.916178671	0.007118279	0.020153508
ENSMMUG00000056824		-2.910378179	1.41E-06	9.31E-06
ENSMMUG00000022601	cAMP responsive element binding protein 1 [Source:VGNC Symbol;Acc:VGNC:71485]	-1.761732887	2.87E-08	2.51E-07
ENSMMUG00000022602	methyltransferase like 21A [Source:VGNC Symbol;Acc:VGNC:74625]	-1.752279645	1.16E-06	7.75E-06
ENSMMUG00000020211	cyclin Y like 1 [Source:VGNC Symbol;Acc:VGNC:84224]	-2.203602044	2.70E-16	6.48E-15
ENSMMUG00000057651	frizzled class receptor 5 [Source:NCBI gene;Acc:710796]	-3.88702474	1.81E-07	1.40E-06
ENSMMUG00000008113	pleckstrin homology domain containing M3 [Source:NCBI gene;Acc:711125]	-2.182667402	6.48E-07	4.56E-06
ENSMMUG00000056938	crystallin, gamma C [Source:NCBI gene;Acc:709553]	-3.338312223	0.010076082	0.027225092
ENSMMUG00000014299	chromosome 12 C2orf80 homolog [Source:VGNC Symbol;Acc:VGNC:70459]	-2.224768457	0.003156003	0.009932837
ENSMMUG00000016077	isocitrate dehydrogenase (NADP(+)) 1 [Source:NCBI gene;Acc:710019]	-1.483605614	0.00227704	0.007433084
ENSMMUG00000017524	phosphoinositide kinase, FYVE-type zinc finger containing [Source:VGNC Symbol;Acc:VGNC:75854]	-1.917484429	1.63E-06	1.07E-05
ENSMMUG00000005295	parathyroid hormone 2 receptor [Source:VGNC Symbol;Acc:VGNC:76592]	-1.514962289	0.017323583	0.043199721
ENSMMUG00000002809	unc-80 homolog, NALCN channel complex subunit [Source:NCBI gene;Acc:713007]	-1.369630562	4.21E-08	3.57E-07
ENSMMUG00000010513	ribulose-5-phosphate-3-epimerase [Source:NCBI gene;Acc:711322]	1.986526583	5.29E-09	5.17E-08
ENSMMUG00000010515	KAT8 regulatory NSL complex subunit 1 like [Source:VGNC Symbol;Acc:VGNC:73913]	2.182916173	2.72E-09	2.79E-08
ENSMMUG00000010508	carbamoyl-phosphate synthase 1 [Source:VGNC Symbol;Acc:VGNC:71471]	-1.975894663	0.00040786	0.001622469
ENSMMUG00000059604		0.416315049	0.016475439	0.041454151
ENSMMUG00000061342		2.427379213	2.45E-27	1.95E-25
ENSMMUG00000000381	IKAROS family zinc finger 2 [Source:VGNC Symbol;Acc:VGNC:73586]	1.456628776	7.27E-25	4.33E-23
ENSMMUG00000000382	sperm associated antigen 16 [Source:VGNC Symbol;Acc:VGNC:77977]	0.602436686	0.000107632	0.000487832
ENSMMUG00000064880		-0.782129483	1.77E-05	9.56E-05
ENSMMUG00000053721		-1.944882321	1.20E-09	1.30E-08
ENSMMUG00000006744	serine/threonine kinase 36 [Source:VGNC Symbol;Acc:VGNC:78145]	-3.193186793	0.006665194	0.019044429
ENSMMUG00000006755	tubulin tyrosine ligase like 4 [Source:VGNC Symbol;Acc:VGNC:79758]	-1.201724922	9.31E-07	6.35E-06
ENSMMUG00000006758	cytochrome P450, family 27, subfamily A, polypeptide 1 [Source:NCBI gene;Acc:700192]	2.142799296	2.21E-05	0.000116282
ENSMMUG00000021877	protein kinase AMP-activated non-catalytic subunit gamma 3 [Source:VGNC Symbol;Acc:VGNC:76406]	2.274939239	3.50E-05	0.000176284
ENSMMUG00000052954	Wnt family member 6 [Source:NCBI gene;Acc:700573]	2.461051361	0.000116775	0.000523196
ENSMMUG00000002519	serine/threonine kinase 11 interacting protein [Source:VGNC Symbol;Acc:VGNC:77963]	-1.000023075	5.45E-12	8.13E-11
ENSMMUG00000002520	solute carrier family 4 member 3 [Source:VGNC Symbol;Acc:VGNC:77604]	-1.202946053	1.37E-11	1.93E-10
ENSMMUG00000061834		-1.332690172	6.37E-12	9.35E-11
ENSMMUG00000046282		-1.158119278	1.22E-13	2.25E-12
ENSMMUG00000051349		2.750448623	0.009856411	0.026717148

ENSMMUG00000018139	dedicator of cytokinesis 10 [Source:VGNC Symbol;Acc:VGNC:71888]	0.667061478	1.14E-12	1.86E-11
ENSMMUG00000051482		-2.130775663	1.26E-08	1.16E-07
ENSMMUG00000054072		0.842679553	7.81E-12	1.13E-10
ENSMMUG00000045544	neuronal tyrosine-phosphorylated phosphoinositide-3-kinase adaptor 2 [Source:NCBI gene;Acc:711209]	0.760161707	3.43E-07	2.51E-06
ENSMMUG00000000722	rhomboid domain containing 1 [Source:VGNC Symbol;Acc:VGNC:76803]	-0.526501722	0.015950143	0.040307144
ENSMMUG00000013233	collagen type IV alpha 4 chain [Source:VGNC Symbol;Acc:VGNC:71299]	-1.121810593	7.15E-05	0.000335874
ENSMMUG00000013235	collagen type IV alpha 3 chain [Source:VGNC Symbol;Acc:VGNC:81375]	-1.07917352	1.49E-07	1.17E-06
ENSMMUG00000002103	delta/notch like EGF repeat containing [Source:VGNC Symbol;Acc:VGNC:71895]	-1.914498654	4.15E-09	4.13E-08
ENSMMUG00000022831	COP9 signalosome subunit 7B [Source:VGNC Symbol;Acc:VGNC:71186]	-2.898094889	0.010384315	0.027946798
ENSMMUG00000015167	DIS3 like 3'-5' exoribonuclease 2 [Source:VGNC Symbol;Acc:VGNC:71793]	-1.378810736	0.000104651	0.000475772
ENSMMUG00000016871	alkaline phosphatase, intestinal [Source:VGNC Symbol;Acc:VGNC:69800]	-2.449461786	3.96E-06	2.40E-05
ENSMMUG00000051779		0.747173595	2.82E-05	0.000144877
ENSMMUG00000023359	S-antigen visual arrestin [Source:VGNC Symbol;Acc:VGNC:76965]	-0.799213136	0.000498945	0.001941578
ENSMMUG00000005650	ubiquitin specific peptidase 40 [Source:VGNC Symbol;Acc:VGNC:79226]	-0.444590383	0.011475965	0.030480352
ENSMMUG00000021216	UDP glucuronosyltransferase 1 family, polypeptide A1 [Source:NCBI gene;Acc:574210]	-0.985116159	9.07E-07	6.20E-06
ENSMMUG00000057588		1.846562905	0.014821621	0.03787776
ENSMMUG00000064179	Metazoan signal recognition particle RNA [Source:RFAM;Acc:RF00017]	2.282414138	0.01419278	0.036494376
ENSMMUG00000051807		1.750089516	1.93E-13	3.46E-12
ENSMMUG00000061244		1.813307928	3.03E-05	0.000154458
ENSMMUG00000011516	ADP ribosylation factor like GTPase 4C [Source:VGNC Symbol;Acc:VGNC:70018]	2.317302464	9.08E-06	5.14E-05
ENSMMUG00000054449	Metazoan signal recognition particle RNA [Source:RFAM;Acc:RF00017]	1.404149433	0.000269932	0.001116688
ENSMMUG00000017404	IQ motif containing with AAA domain 1 [Source:VGNC Symbol;Acc:VGNC:73660]	-2.48192491	0.0022624	0.007391803
ENSMMUG00000060487	atypical chemokine receptor 3 [Source:NCBI gene;Acc:694428]	-1.884705542	5.21E-06	3.09E-05
ENSMMUG00000061673		0.530495468	0.000522346	0.002020448
ENSMMUG00000014049	collagen type VI alpha 3 chain [Source:VGNC Symbol;Acc:VGNC:71303]	-1.673564291	5.10E-06	3.02E-05
ENSMMUG00000031293	anoctamin-9 [Source:NCBI gene;Acc:100425888]	0.694219973	3.33E-08	2.87E-07
ENSMMUG00000059353	phosphatidylserine synthase 2 [Source:NCBI gene;Acc:698582]	-0.680428474	6.70E-07	4.69E-06
ENSMMUG00000056711	ribonuclease inhibitor-like [Source:NCBI gene;Acc:114672186]	-0.516427694	3.66E-05	0.000183134
ENSMMUG00000062863		2.205273241	3.24E-14	6.33E-13
ENSMMUG00000004622	NLR family pyrin domain containing 6 [Source:VGNC Symbol;Acc:VGNC:75234]	-1.737883177	0.000858124	0.003133704
ENSMMUG00000004625	protein-glucosylgalactosylhydroxylysine glucosidase [Source:VGNC Symbol;Acc:VGNC:75870]	-2.311307226	0.017197667	0.042943494
ENSMMUG00000057640	plakophilin-3-like [Source:NCBI gene;Acc:114669833]	2.10680162	5.70E-22	2.55E-20
ENSMMUG00000002917	single Ig IL-1-related receptor [Source:NCBI gene;Acc:100425060]	3.223290088	5.78E-15	1.24E-13
ENSMMUG00000010783	ribonuclease inhibitor [Source:NCBI gene;Acc:720791]	0.407729015	0.019539573	0.047872438
ENSMMUG00000021661	leucine rich repeat containing 56 [Source:VGNC Symbol;Acc:VGNC:74356]	1.951580894	1.79E-13	3.22E-12
ENSMMUG00000021664	lamin tail domain containing 2 [Source:VGNC Symbol;Acc:VGNC:74380]	2.004497245	1.85E-26	1.31E-24
ENSMMUG00000004014	interferon regulatory factor 7 [Source:VGNC Symbol;Acc:VGNC:73765]	1.111458063	0.000100304	0.000458539
ENSMMUG00000004024	cadherin related family member 5 [Source:VGNC Symbol;Acc:VGNC:71158]	0.862229659	0.001026856	0.003686341

ENSMMUG00000007510	transaldolase 1 [Source:VGNC Symbol;Acc:VGNC:78096]	0.772038633	3.79E-06	2.30E-05
ENSMMUG00000007511	glutamine amidotransferase like class 1 domain containing 1 [Source:VGNC Symbol;Acc:VGNC:82149]	0.715716121	0.000148274	0.000649692
ENSMMUG00000042839		0.895578369	2.06E-06	1.32E-05
ENSMMUG00000016715	p53-induced death domain protein 1 [Source:VGNC Symbol;Acc:VGNC:81498]	0.859411441	2.32E-09	2.40E-08
ENSMMUG00000043846		0.697025967	8.37E-07	5.75E-06
ENSMMUG00000010544	mucin 5B, oligomeric mucus/gel-forming [Source:VGNC Symbol;Acc:VGNC:75081]	-0.683843479	0.000725119	0.00269647
ENSMMUG00000017450	MOB kinase activator 2 [Source:NCBI gene;Acc:720951]	1.931455803	8.74E-07	5.99E-06
ENSMMUG00000003874	interferon induced transmembrane protein 10 [Source:NCBI gene;Acc:721010]	3.654607373	2.10E-20	7.70E-19
ENSMMUG00000031267	insulin [Source:NCBI gene;Acc:704534]	-0.999491476	0.001384298	0.004812267
ENSMMUG00000001324	tetraspanin 32 [Source:VGNC Symbol;Acc:VGNC:78920]	1.225829431	0.017677754	0.043957259
ENSMMUG00000004773	transient receptor potential cation channel subfamily M member 5 [Source:VGNC Symbol;Acc:VGNC:78955]	-0.844850579	0.00107521	0.003843181
ENSMMUG00000007742	potassium voltage-gated channel subfamily Q member 1 [Source:VGNC Symbol;Acc:VGNC:82168]	-1.055654424	4.13E-05	0.000203841
ENSMMUG00000000511	cysteinyl-tRNA synthetase 1 [Source:VGNC Symbol;Acc:VGNC:70540]	-1.12010599	3.81E-05	0.000189382
ENSMMUG00000051131	MAS related GPR family member G [Source:NCBI gene;Acc:721253]	0.79159156	0.000359843	0.001447376
ENSMMUG00000052168		2.507863546	1.05E-14	2.18E-13
ENSMMUG00000044640		2.87015272	0.008468618	0.023457155
ENSMMUG00000060517		2.420050777	6.48E-16	1.50E-14
ENSMMUG00000001777	testis expressed metallothionein like protein [Source:VGNC Symbol;Acc:VGNC:79965]	0.932523712	0.000252135	0.00105151
ENSMMUG00000042838	NADH dehydrogenase [ubiquinone] iron-sulfur protein 2, mitochondrial-like [Source:NCBI gene;Acc:721639]	-1.579885053	0.000553601	0.002129125
ENSMMUG00000014237	lysine methyltransferase 5B [Source:VGNC Symbol;Acc:VGNC:74110]	2.291278688	1.09E-37	2.28E-35
ENSMMUG00000056342	Metazoan signal recognition particle RNA [Source:RFAM;Acc:RF00017]	3.288201475	1.66E-05	8.99E-05
ENSMMUG00000023192	choline kinase alpha [Source:VGNC Symbol;Acc:VGNC:71039]	1.372020834	4.42E-06	2.66E-05
ENSMMUG00000039824	aminoacylase 3 [Source:NCBI gene;Acc:711083]	-0.57306801	0.000163512	0.000709651
ENSMMUG00000056105	T-box transcription factor 10 [Source:NCBI gene;Acc:721689]	-0.731922105	1.69E-05	9.14E-05
ENSMMUG00000042611	nudix hydrolase 8 [Source:NCBI gene;Acc:721695]	-2.595640868	0.009927022	0.026875954
ENSMMUG00000062171		-1.18842276	0.020572831	0.049982811
ENSMMUG00000011821	phosphatidylinositol transfer protein membrane associated 1 [Source:VGNC Symbol;Acc:VGNC:76007]	-1.329072432	0.012229336	0.032190001
ENSMMUG00000011817	aryl hydrocarbon receptor interacting protein [Source:VGNC Symbol;Acc:VGNC:69759]	1.171875609	3.16E-11	4.26E-10
ENSMMUG00000005489	G protein-coupled receptor 152 [Source:VGNC Symbol;Acc:VGNC:72986]	0.963077911	0.003054736	0.009642769
ENSMMUG00000053378		1.323960037	2.64E-09	2.71E-08
ENSMMUG00000005488	coronin 1B [Source:VGNC Symbol;Acc:VGNC:71320]	1.82831656	1.87E-24	1.06E-22
ENSMMUG00000005486	protein tyrosine phosphatase receptor type C associated protein [Source:VGNC Symbol;Acc:VGNC:76613]	1.166315349	8.18E-11	1.05E-09
ENSMMUG00000005483	ribosomal protein S6 kinase B2 [Source:VGNC Symbol;Acc:VGNC:76934]	1.166626252	1.76E-13	3.18E-12
ENSMMUG00000005480	carnosine synthase 1 [Source:VGNC Symbol;Acc:VGNC:81367]	1.106938277	3.71E-18	1.08E-16
ENSMMUG00000017280	TBC1 domain family member 10C [Source:VGNC Symbol;Acc:VGNC:78203]	1.176598076	1.13E-06	7.60E-06
ENSMMUG00000017278	protein phosphatase 1 catalytic subunit alpha [Source:VGNC Symbol;Acc:VGNC:76366]	0.726049244	0.000348826	0.001406876
ENSMMUG00000017275	RAD9 checkpoint clamp component A [Source:VGNC Symbol;Acc:VGNC:76648]	0.6576904	0.003584779	0.01109628
ENSMMUG00000004523	DNA polymerase delta 4, accessory subunit [Source:NCBI gene;Acc:712632]	1.133396379	3.57E-05	0.000178949
ENSMMUG00000064122		1.08166944	1.52E-07	1.19E-06
ENSMMUG00000015932	slingshot protein phosphatase 3 [Source:VGNC Symbol;Acc:VGNC:78019]	0.892995758	0.000345727	0.001396277
ENSMMUG00000015928	ankyrin repeat domain 13D [Source:VGNC Symbol;Acc:VGNC:69722]	1.343329017	6.76E-05	0.000319321
ENSMMUG00000015925	G protein-coupled receptor kinase 2 [Source:VGNC Symbol;Acc:VGNC:73253]	0.934217163	1.34E-06	8.88E-06
ENSMMUG00000008771	lysine demethylase 2A [Source:VGNC Symbol;Acc:VGNC:73824]	0.941904211	1.08E-09	1.18E-08
ENSMMUG00000004529	chromosome 14 C11orf80 homolog [Source:NCBI gene;Acc:721782]	-0.286605011	0.003232424	0.010149661

ENSMMUG00000062635		-0.747382295	3.07E-07	2.26E-06
ENSMMUG00000020363	RNA-binding protein 4 [Source:NCBI gene;Acc:713490]	-0.52114961	0.009307995	0.025458791
ENSMMUG00000003387	pellino E3 ubiquitin protein ligase family member 3 [Source:VGNC Symbol;Acc:VGNC:75886]	-0.504893015	0.020442002	0.049738292
ENSMMUG00000015984	solute carrier family 29 member 2 [Source:VGNC Symbol;Acc:VGNC:77446]	-0.5511699	0.010438554	0.028064497
ENSMMUG00000015987	Ras and Rab interactor 1 [Source:VGNC Symbol;Acc:VGNC:76769]	-1.012495095	2.08E-07	1.58E-06
ENSMMUG00000006950	cornichon family AMPA receptor auxiliary protein 2 [Source:VGNC Symbol;Acc:VGNC:71366]	-1.55392328	3.36E-06	2.06E-05
ENSMMUG00000031202	RAB1B, member RAS oncogene family [Source:NCBI gene;Acc:721852]	-1.157213097	1.64E-07	1.28E-06
ENSMMUG00000022520	kinesin light chain 2 [Source:VGNC Symbol;Acc:VGNC:73869]	-0.930708073	6.36E-05	0.000302731
ENSMMUG00000022518	phosphofurin acidic cluster sorting protein 1 [Source:VGNC Symbol;Acc:VGNC:75697]	-0.650036555	0.002590013	0.008339101
ENSMMUG00000057179	cation channel sperm associated 1 [Source:NCBI gene;Acc:721869]	1.613132598	0.002028148	0.006736269
ENSMMUG00000056794	cystatin E/M [Source:NCBI gene;Acc:714999]	2.556158955	0.010358564	0.027889889
ENSMMUG00000019115	eukaryotic translation initiation factor 1A domain containing [Source:VGNC Symbol;Acc:VGNC:72127]	0.590900592	4.86E-05	0.000236719
ENSMMUG00000008652	coiled-coil domain containing 85B [Source:VGNC Symbol;Acc:VGNC:70853]	1.216964652	1.55E-09	1.66E-08
ENSMMUG00000008651	FGF1 intracellular binding protein [Source:VGNC Symbol;Acc:VGNC:72663]	1.323304554	1.45E-10	1.79E-09
ENSMMUG00000008649	cathepsin W [Source:VGNC Symbol;Acc:VGNC:71563]	1.654095168	0.00106872	0.003820905
ENSMMUG00000038363	adaptor related protein complex 5 subunit beta 1 [Source:NCBI gene;Acc:721904]	1.837869598	2.09E-21	8.71E-20
ENSMMUG00000050028		1.783335827	1.79E-13	3.22E-12
ENSMMUG00000064005		1.467477731	5.31E-07	3.78E-06
ENSMMUG00000060437	Metazoan signal recognition particle RNA [Source:RFAM;Acc:RF00017]	1.645919344	8.14E-06	4.65E-05
ENSMMUG00000019837	ribonuclease H2 subunit C [Source:NCBI gene;Acc:700330]	1.393494061	3.81E-13	6.62E-12
ENSMMUG00000019839	lysine acetyltransferase 5 [Source:VGNC Symbol;Acc:VGNC:73955]	1.548936668	1.84E-20	6.80E-19
ENSMMUG00000061174		0.757142982	0.00073856	0.002739782
ENSMMUG00000007418	RELA proto-oncogene, NF-kB subunit [Source:VGNC Symbol;Acc:VGNC:76893]	0.328866472	0.007244029	0.02045097
ENSMMUG00000007416	signal-induced proliferation-associated 1 [Source:VGNC Symbol;Acc:VGNC:77218]	0.669709582	0.000275338	0.001135817
ENSMMUG00000007402	potassium two pore domain channel subfamily K member 7 [Source:VGNC Symbol;Acc:VGNC:73996]	2.07812951	2.42E-08	2.14E-07
ENSMMUG00000023479	latent transforming growth factor beta binding protein 3 [Source:VGNC Symbol;Acc:VGNC:74458]	2.45521734	6.27E-24	3.42E-22
ENSMMUG00000023477		1.853746389	1.28E-26	9.29E-25
ENSMMUG00000052424		1.047468297	2.32E-06	1.46E-05
ENSMMUG00000023300	FERM domain containing 8 [Source:NCBI gene;Acc:716379]	1.686407614	2.37E-08	2.11E-07
ENSMMUG00000023297	solute carrier family 25 member 45 [Source:VGNC Symbol;Acc:VGNC:77576]	1.122177875	0.004429064	0.013369229
ENSMMUG00000056343		1.235110199	9.02E-07	6.17E-06
ENSMMUG00000012316	CDC42 effector protein 2 [Source:VGNC Symbol;Acc:VGNC:70750]	2.498757129	5.36E-05	0.000258846
ENSMMUG00000022031	DNA polymerase alpha 2, accessory subunit [Source:NCBI gene;Acc:716541]	1.361715115	7.02E-14	1.33E-12
ENSMMUG00000018781	synoviolin 1 [Source:VGNC Symbol;Acc:VGNC:78183]	-0.690327942	0.010662113	0.028556823
ENSMMUG00000006568	basic leucine zipper ATF-like transcription factor 2 [Source:VGNC Symbol;Acc:VGNC:70127]	1.285866499	2.72E-10	3.25E-09
ENSMMUG00000031163	membrane anchored junction protein [Source:VGNC Symbol;Acc:VGNC:74375]	2.123776558	3.36E-30	3.31E-28
ENSMMUG00000003976	glycoprotein hormone subunit alpha 2 [Source:VGNC Symbol;Acc:VGNC:72961]	1.919822995	6.17E-21	2.43E-19
ENSMMUG00000041101	protein phosphatase 2 regulatory subunit B'beta [Source:NCBI gene;Acc:722026]	2.14914923	1.73E-50	1.83E-47
ENSMMUG00000003973	autophagy related 2A [Source:VGNC Symbol;Acc:VGNC:70145]	-1.73443839	6.25E-07	4.41E-06
ENSMMUG00000003972	EH domain containing 1 [Source:VGNC Symbol;Acc:VGNC:72175]	0.538518926	7.84E-05	0.000365092
ENSMMUG00000003971	CDC42 binding protein kinase gamma [Source:VGNC Symbol;Acc:VGNC:70909]	0.492972836	4.98E-05	0.000242201
ENSMMUG00000031160	menin 1 [Source:NCBI gene;Acc:106995582]	0.620738114	2.14E-08	1.91E-07
ENSMMUG00000030597	mitogen-activated protein kinase kinase kinase kinase 2 [Source:VGNC Symbol;Acc:VGNC:81465]	0.608028308	1.32E-09	1.43E-08
ENSMMUG00000007610	ribosomal protein S6 kinase A4 [Source:VGNC Symbol;Acc:VGNC:76930]	1.622633708	1.02E-21	4.41E-20
ENSMMUG00000007603	coiled-coil domain containing 88B [Source:NCBI gene;Acc:722080]	0.751939847	0.001044752	0.003741528

ENSMMUG00000013397	peroxiredoxin 5 [Source:NCBI gene;Acc:719764]	1.087394253	1.63E-12	2.62E-11
ENSMMUG00000060596	BCL2 associated agonist of cell death [Source:NCBI gene;Acc:717740]	0.761739152	0.000565087	0.002165997
ENSMMUG00000012948	phospholipase C beta 3 [Source:VGNC Symbol;Acc:VGNC:76165]	-0.459584142	0.007818871	0.021882216
ENSMMUG00000037977	protein phosphatase 1 regulatory inhibitor subunit 14B [Source:NCBI gene;Acc:722103]	1.12476303	0.000315399	0.001285345
ENSMMUG00000048072	FKBP prolyl isomerase 2 [Source:NCBI gene;Acc:717805]	0.567392241	0.004249339	0.012908171
ENSMMUG00000012944	vascular endothelial growth factor B [Source:VGNC Symbol;Acc:VGNC:78765]	1.571880694	1.54E-22	7.34E-21
ENSMMUG00000012942	DnaJ heat shock protein family (Hsp40) member C4 [Source:NCBI gene;Acc:717847]	1.394878871	1.07E-05	5.97E-05
ENSMMUG00000012941	nudix hydrolase 22 [Source:VGNC Symbol;Acc:VGNC:75475]	1.57995276	3.65E-23	1.85E-21
ENSMMUG00000012940	tRNA phosphotransferase 1 [Source:VGNC Symbol;Acc:VGNC:79242]	1.055324848	1.98E-06	1.27E-05
ENSMMUG00000012939	fermitin family member 3 [Source:VGNC Symbol;Acc:VGNC:72553]	-0.555299743	0.000152767	0.000667512
ENSMMUG00000012953	stress induced phosphoprotein 1 [Source:NCBI gene;Acc:717926]	-1.074378403	2.54E-12	3.98E-11
ENSMMUG00000056349		-2.234652564	0.004140629	0.012616712
ENSMMUG00000000478	OTU deubiquitinase, ubiquitin aldehyde binding 1 [Source:NCBI gene;Acc:717989]	0.764954688	1.72E-10	2.11E-09
ENSMMUG00000018632	N-alpha-acetyltransferase 40, NatD catalytic subunit [Source:NCBI gene;Acc:722126]	-2.232721193	0.001574706	0.005373421
ENSMMUG00000052569		3.498538279	4.82E-12	7.23E-11
ENSMMUG00000014131	microtubule affinity regulating kinase 2 [Source:VGNC Symbol;Acc:VGNC:74634]	1.09784863	3.83E-05	0.000190243
ENSMMUG00000059969		0.977996982	0.000405107	0.001612383
ENSMMUG00000018152	spindlin interactor and repressor of chromatin binding [Source:VGNC Symbol;Acc:VGNC:77987]	0.604489625	0.001706059	0.005783051
ENSMMUG00000005776	atlastin GTPase 3 [Source:VGNC Symbol;Acc:VGNC:70125]	1.098578575	6.51E-12	9.54E-11
ENSMMUG00000005775	phospholipase A and acyltransferase 3 [Source:VGNC Symbol;Acc:VGNC:76055]	0.422542833	0.000253407	0.00105592
ENSMMUG00000045105	phospholipase A and acyltransferase 4 [Source:NCBI gene;Acc:722189]	-1.702271986	9.49E-07	6.46E-06
ENSMMUG00000056054	zinc finger and BTB domain containing 3 [Source:NCBI gene;Acc:722240]	-1.024922472	8.98E-11	1.14E-09
ENSMMUG00000059077	Metazoan signal recognition particle RNA [Source:RFAM;Acc:RF00017]	-0.90340085	0.019091604	0.046906641
ENSMMUG00000005761	UBX domain protein 1 [Source:VGNC Symbol;Acc:VGNC:79135]	-0.949944591	4.81E-05	0.000234566
ENSMMUG00000013949	chromosome 14 C11orf98 homolog [Source:NCBI gene;Acc:100429527]	-0.64587979	0.008367983	0.023243527
ENSMMUG00000000327	fatty acid desaturase 2 [Source:VGNC Symbol;Acc:VGNC:72472]	1.794993321	6.91E-15	1.47E-13
ENSMMUG00000013798	fatty acid desaturase 1 [Source:VGNC Symbol;Acc:VGNC:72471]	2.864318843	2.77E-15	6.04E-14
ENSMMUG00000006655	myelin regulatory factor [Source:VGNC Symbol;Acc:VGNC:74949]	2.889440398	3.11E-06	1.92E-05
ENSMMUG00000049301	diacylglycerol lipase alpha [Source:NCBI gene;Acc:722350]	-1.568972917	0.017835249	0.044326586
ENSMMUG00000015280	cleavage and polyadenylation specific factor 7 [Source:VGNC Symbol;Acc:VGNC:71606]	2.205870922	0.000806626	0.002966097
ENSMMUG00000042208	pepsin A-4 [Source:NCBI gene;Acc:694646]	-1.066075275	0.000106605	0.000483767
ENSMMUG00000037611	pepsinogen 4, group I (pepsinogen A) [Source:NCBI gene;Acc:694562]	0.343187148	0.014644195	0.037501886
ENSMMUG00000015019	VPS37C subunit of ESCRT-I [Source:VGNC Symbol;Acc:VGNC:79578]	3.303869898	0.000320204	0.001303852
ENSMMUG00000044417	CD6 molecule [Source:NCBI gene;Acc:695183]	-2.491919584	0.00014907	0.000652705
ENSMMUG00000014535	solute carrier family 15 member 3 [Source:VGNC Symbol;Acc:VGNC:77504]	-1.828220599	0.000447564	0.001759643
ENSMMUG00000052420	transmembrane protein 132A [Source:NCBI gene;Acc:695291]	-1.627144631	9.85E-06	5.54E-05
ENSMMUG00000014531	transmembrane protein 109 [Source:VGNC Symbol;Acc:VGNC:79740]	-1.929599584	3.06E-12	4.75E-11
ENSMMUG00000014530	pre-mRNA processing factor 19 [Source:VGNC Symbol;Acc:VGNC:76281]	1.775951471	6.40E-17	1.64E-15
ENSMMUG00000052454	prostaglandin D2 receptor 2 [Source:NCBI gene;Acc:695853]	-1.160305502	3.17E-06	1.95E-05

ENSMMUG00000000019	coiled-coil domain containing 86 [Source:VGNC Symbol;Acc:VGNC:70893]	-0.531743669	0.001515021	0.005202918
ENSMMUG00000031100	membrane-spanning 4-domains subfamily A member 4A-like [Source:NCBI gene;Acc:698152]	4.306988416	2.79E-17	7.47E-16
ENSMMUG00000004821	membrane spanning 4-domains A14 [Source:NCBI gene;Acc:697083]	-2.713887798	1.58E-11	2.20E-10
ENSMMUG00000061603		3.884097967	8.66E-06	4.92E-05
ENSMMUG00000059299	membrane spanning 4-domains A2 [Source:NCBI gene;Acc:697830]	2.540012112	3.85E-07	2.79E-06
ENSMMUG00000053140	putative oocyte-secreted protein 1 homolog [Source:NCBI gene;Acc:698403]	1.971098148	0.001568614	0.005357566
ENSMMUG00000017287	PAT1 homolog 1, processing body mRNA decay factor [Source:VGNC Symbol;Acc:VGNC:75684]	2.802391907	1.49E-15	3.33E-14
ENSMMUG00000001745	oxysterol binding protein [Source:VGNC Symbol;Acc:VGNC:75718]	1.79233505	4.99E-18	1.43E-16
ENSMMUG00000060712		1.35670438	1.35E-11	1.91E-10
ENSMMUG00000001083	glycine N-acyltransferase-like protein 1 [Source:NCBI gene;Acc:701437]	0.859239588	8.68E-07	5.96E-06
ENSMMUG00000011713	leupaxin [Source:VGNC Symbol;Acc:VGNC:74286]	-0.529727758	3.38E-09	3.42E-08
ENSMMUG00000011712	olfactory receptor 5B21 [Source:NCBI gene;Acc:702145]	-1.130125709	0.000194395	0.000831041
ENSMMUG00000054737		-0.99372745	1.22E-13	2.25E-12
ENSMMUG00000062697	olfactory receptor 5B12 [Source:NCBI gene;Acc:106993236]	-1.509346895	0.000263229	0.001091637
ENSMMUG00000059848	olfactory receptor 5B2 [Source:NCBI gene;Acc:100429465]	-1.115076712	0.001849046	0.006217994
ENSMMUG00000060679	olfactory receptor 5B3 [Source:NCBI gene;Acc:701735]	-2.516232769	0.005180818	0.0152929
ENSMMUG00000050844	BTB domain containing 18 [Source:NCBI gene;Acc:703264]	2.557629726	9.65E-10	1.06E-08
ENSMMUG00000056184		3.888553029	1.54E-15	3.45E-14
ENSMMUG00000002566	selenoprotein H [Source:VGNC Symbol;Acc:VGNC:82234]	3.352692878	5.21E-08	4.38E-07
ENSMMUG00000040741	thioredoxin related transmembrane protein 2 [Source:NCBI gene;Acc:100169970]	2.083844109	1.47E-16	3.63E-15
ENSMMUG00000002562	mediator complex subunit 19 [Source:VGNC Symbol;Acc:VGNC:74615]	1.662697206	0.002444071	0.007925993
ENSMMUG00000002563	zinc finger DHHC-type palmitoyltransferase 5 [Source:VGNC Symbol;Acc:VGNC:79145]	2.199114216	2.17E-06	1.38E-05
ENSMMUG00000058453	cleavage factor polyribonucleotide kinase subunit 1 [Source:NCBI gene;Acc:705071]	-0.922785973	0.000438459	0.001728429
ENSMMUG00000012618	serpin family G member 1 [Source:VGNC Symbol;Acc:VGNC:77223]	0.66780859	0.000140527	0.000618402
ENSMMUG00000017810	structure specific recognition protein 1 [Source:VGNC Symbol;Acc:VGNC:77891]	1.687582151	2.84E-14	5.59E-13
ENSMMUG00000010627	apelin receptor [Source:VGNC Symbol;Acc:VGNC:69939]	-1.373115126	6.88E-13	1.17E-11
ENSMMUG00000010869	nucleoporin 160 [Source:VGNC Symbol;Acc:VGNC:75429]	1.274620528	0.00115363	0.004094862
ENSMMUG00000040099	Y RNA [Source:RFAM;Acc:RF00019]	1.678202675	0.000328023	0.001331666
ENSMMUG00000058885	formin binding protein 4 [Source:NCBI gene;Acc:712633]	1.48216239	3.57E-16	8.43E-15
ENSMMUG00000043682	mitochondrial carrier 2 [Source:NCBI gene;Acc:712767]	-2.249159572	3.78E-05	0.000188243
ENSMMUG00000004286	protein tyrosine phosphatase mitochondrial 1 [Source:NCBI gene;Acc:713104]	-1.327985761	0.003282786	0.010281407
ENSMMUG00000011657	CUGBP Elav-like family member 1 [Source:VGNC Symbol;Acc:VGNC:71098]	-0.415695941	0.002462947	0.007975001
ENSMMUG00000015611	proteasome 26S subunit, ATPase 3 [Source:NCBI gene;Acc:713356]	1.484545222	2.52E-05	0.000131064
ENSMMUG00000015607	solute carrier family 39 member 13 [Source:VGNC Symbol;Acc:VGNC:77538]	0.78056885	0.001923751	0.006440005
ENSMMUG00000015606	Spi-1 proto-oncogene [Source:NCBI gene;Acc:712010]	2.106885181	4.06E-12	6.15E-11
ENSMMUG00000062208	Metazoan signal recognition particle RNA [Source:RFAM;Acc:RF00017]	1.807051581	1.30E-06	8.63E-06
ENSMMUG00000015604	myosin binding protein C3 [Source:VGNC Symbol;Acc:VGNC:75094]	-0.528844686	0.007108635	0.020137523
ENSMMUG00000015602	MAP kinase activating death domain [Source:VGNC Symbol;Acc:VGNC:74473]	-0.374848545	0.000347526	0.001402398
ENSMMUG00000060750	Metazoan signal recognition particle RNA [Source:RFAM;Acc:RF00017]	2.557230561	3.55E-05	0.000178485
ENSMMUG00000003631	cytoskeleton associated protein 5 [Source:VGNC Symbol;Acc:VGNC:71368]	-2.07781469	1.77E-07	1.37E-06

ENSMMUG00000019674	coagulation factor II, thrombin [Source:VGNC Symbol;Acc:VGNC:72464]	-1.853687156	0.001565789	0.00535038
ENSMMUG00000019672	autophagy related 13 [Source:VGNC Symbol;Acc:VGNC:70148]	-2.057930495	4.50E-06	2.70E-05
ENSMMUG00000021446	diacylglycerol kinase zeta [Source:VGNC Symbol;Acc:VGNC:71789]	0.567760324	4.81E-05	0.000234438
ENSMMUG00000002452	solute carrier family 35 member C1 [Source:VGNC Symbol;Acc:VGNC:77524]	1.764575476	1.17E-24	6.78E-23
ENSMMUG00000049305	Metazoan signal recognition particle RNA [Source:RFAM;Acc:RF00017]	1.693064659	2.41E-08	2.14E-07
ENSMMUG00000047219		1.586728252	6.14E-13	1.04E-11
ENSMMUG00000000874	carbohydrate sulfotransferase 1 [Source:VGNC Symbol;Acc:VGNC:71055]	1.95384925	9.31E-22	4.06E-20
ENSMMUG00000050952		-1.186308368	0.001171355	0.004151817
ENSMMUG00000059728	tumor protein p53 inducible protein 11 [Source:NCBI gene;Acc:715295]	-1.477331257	0.000795724	0.002931822
ENSMMUG00000000368	tetraspanin 18 [Source:VGNC Symbol;Acc:VGNC:79265]	-1.927041948	0.004157902	0.012661538
ENSMMUG00000064903	Metazoan signal recognition particle RNA [Source:RFAM;Acc:RF00017]	2.374135079	0.018336118	0.045371709
ENSMMUG00000049355	ALX homeobox 4 [Source:NCBI gene;Acc:715652]	1.29720914	0.002340732	0.007627567
ENSMMUG00000017294	alkB homolog 3, alpha-ketoglutaratedependent dioxygenase [Source:VGNC Symbol;Acc:VGNC:69736]	-0.956319624	0.005547243	0.016235567
ENSMMUG00000056754		-1.123616149	0.014194171	0.036494376
ENSMMUG00000008827	apoptosis inhibitor 5 [Source:NCBI gene;Acc:716346]	-0.952713649	0.004347893	0.013159032
ENSMMUG00000049945		-0.515500703	0.002006898	0.006677479
ENSMMUG00000053160		-0.396701834	0.002510715	0.00810843
ENSMMUG00000004332	tripartite motif containing 44 [Source:VGNC Symbol;Acc:VGNC:79168]	2.587677544	2.20E-14	4.37E-13
ENSMMUG00000004331	four-jointed box kinase 1 [Source:NCBI gene;Acc:717833]	2.135510365	0.010130718	0.027360872
ENSMMUG00000004330	peptidase domain containing associated with muscle regeneration 1 [Source:VGNC Symbol;Acc:VGNC:75673]	3.256998163	2.05E-08	1.84E-07
ENSMMUG00000004329	solute carrier family 1 member 2 [Source:VGNC Symbol;Acc:VGNC:77410]	-1.250364021	7.73E-09	7.38E-08
ENSMMUG00000004327	CD44 molecule (Indian blood group) [Source:NCBI gene;Acc:717204]	-1.106444992	0.000852618	0.003117437
ENSMMUG00000002432	E74 like ETS transcription factor 5 [Source:VGNC Symbol;Acc:VGNC:72209]	-0.867309832	0.000481422	0.001879307
ENSMMUG00000002429	catalase [Source:VGNC Symbol;Acc:VGNC:70552]	-0.940435199	3.54E-09	3.57E-08
ENSMMUG00000017218	KIAA1549 like [Source:VGNC Symbol;Acc:VGNC:73902]	-0.529088569	0.004901405	0.014607683
ENSMMUG00000019988	homeodomain interacting protein kinase 3 [Source:VGNC Symbol;Acc:VGNC:73472]	1.549206469	3.36E-10	3.94E-09
ENSMMUG00000001536	glutamine and serine rich 1 [Source:VGNC Symbol;Acc:VGNC:76544]	1.33124002	3.85E-23	1.94E-21
ENSMMUG00000055352		1.631878377	2.55E-39	6.41E-37
ENSMMUG00000049850	proline rich and Gla domain 4 [Source:NCBI gene;Acc:694707]	1.051482932	2.02E-09	2.11E-08
ENSMMUG00000009026	eukaryotic translation initiation factor 3 subunit M [Source:VGNC Symbol;Acc:VGNC:72095]	0.439009776	0.006130967	0.017723634
ENSMMUG00000060261		0.859231926	3.75E-05	0.000186995
ENSMMUG00000065337		-0.411674982	0.000238052	0.000998391
ENSMMUG00000049710	doublecortin domain containing 1 [Source:NCBI gene;Acc:696721]	-1.331500639	0.013759784	0.035599721
ENSMMUG00000006842	doublecortin domain containing 1 [Source:NCBI gene;Acc:696721]	-1.60615169	2.71E-18	8.02E-17
ENSMMUG00000023119	lin-7 homolog C, crumbs cell polarity complex component [Source:VGNC Symbol;Acc:VGNC:74070]	-1.841638549	0.000515783	0.001999755
ENSMMUG00000063557		-1.662670896	0.0051595	0.015245146

ENSMMUG00000014614	lactate dehydrogenase A like 6A [Source:VGNC Symbol;Acc:VGNC:74141]	-3.020105262	0.00845032	0.023419593
ENSMMUG00000014615	lactate dehydrogenase C [Source:VGNC Symbol;Acc:VGNC:74247]	0.940997207	0.001338684	0.00467122
ENSMMUG00000031024	lactate dehydrogenase A [Source:NCBI gene;Acc:694309]	1.05426169	1.27E-06	8.46E-06
ENSMMUG00000006405	secretion regulating guanine nucleotide exchange factor [Source:VGNC Symbol;Acc:VGNC:77258]	1.202676462	5.44E-06	3.21E-05
ENSMMUG00000006403	potassium voltage-gated channel subfamily C member 1 [Source:VGNC Symbol;Acc:VGNC:73971]	0.736868048	2.66E-09	2.73E-08
ENSMMUG00000005895	nucleobindin 2 [Source:VGNC Symbol;Acc:VGNC:75549]	-1.470958968	1.95E-05	0.000104155
ENSMMUG00000064558		-0.978264244	0.000916578	0.003321001
ENSMMUG00000022548	phosphatidylinositol-4-phosphate 3-kinase catalytic subunit type 2 alpha [Source:VGNC Symbol;Acc:VGNC:75992]	-1.682838517	4.31E-10	4.99E-09
ENSMMUG00000022547	ribosomal protein S13 [Source:NCBI gene;Acc:697513]	-1.507205438	0.003881398	0.011905096
ENSMMUG00000017628	pleckstrin homology domain containing A7 [Source:VGNC Symbol;Acc:VGNC:76121]	-3.958104587	2.41E-08	2.13E-07
ENSMMUG00000060216		-3.871690388	0.001581511	0.00539416
ENSMMUG00000064582		-2.828397259	0.001680574	0.00570449
ENSMMUG00000055212		-3.790733981	7.88E-05	0.00036715
ENSMMUG00000020246	INSC spindle orientation adaptor protein [Source:VGNC Symbol;Acc:VGNC:73693]	2.356774732	0.017215068	0.042960428
ENSMMUG00000021914	spondin 1 [Source:VGNC Symbol;Acc:VGNC:77862]	-1.182554254	1.16E-08	1.07E-07
ENSMMUG00000062000		-1.069354703	0.000747196	0.002767159
ENSMMUG00000011990	fatty acyl-CoA reductase 1 [Source:NCBI gene;Acc:699947]	-0.85551093	0.00646462	0.018554349
ENSMMUG00000054213	parathyroid hormone [Source:NCBI gene;Acc:700195]	-2.039076598	9.52E-06	5.37E-05
ENSMMUG00000009695	BTB domain containing 10 [Source:VGNC Symbol;Acc:VGNC:70361]	-1.641981449	1.78E-05	9.57E-05
ENSMMUG00000010875	TEA domain transcription factor 1 [Source:VGNC Symbol;Acc:VGNC:78301]	0.809039053	1.12E-10	1.40E-09
ENSMMUG00000020956	parvin alpha [Source:VGNC Symbol;Acc:VGNC:75605]	0.800852333	0.000516587	0.002001825
ENSMMUG00000043504		-1.062087802	1.16E-08	1.07E-07
ENSMMUG00000037426	U7 small nuclear RNA [Source:RFAM;Acc:RF00066]	4.296608054	0.00023267	0.000978865
ENSMMUG00000009204	switching B cell complex subunit SWAP70 [Source:VGNC Symbol;Acc:VGNC:78142]	0.624880416	0.00224728	0.007347258
ENSMMUG00000008758	zinc finger protein 143 [Source:VGNC Symbol;Acc:VGNC:81612]	1.733895322	4.67E-08	3.94E-07
ENSMMUG00000023636	importin 7 [Source:NCBI gene;Acc:704213]	1.089436284	0.000165504	0.00071725
ENSMMUG00000005639	transmembrane protein 41B [Source:VGNC Symbol;Acc:VGNC:79754]	0.929985578	0.01707596	0.042718706
ENSMMUG00000058029	Metazoan signal recognition particle RNA [Source:RFAM;Acc:RF00017]	3.111398915	0.020567364	0.049977718
ENSMMUG00000021142	DENN domain containing 5A [Source:VGNC Symbol;Acc:VGNC:71674]	0.701587011	0.000855959	0.003127339
ENSMMUG00000006692	signal peptide, CUB domain and EGF like domain containing 2 [Source:VGNC Symbol;Acc:VGNC:77138]	1.249669967	1.07E-07	8.59E-07
ENSMMUG00000006689	nuclear receptor interacting protein 3 [Source:VGNC Symbol;Acc:VGNC:75397]	1.713548623	4.67E-13	8.03E-12
ENSMMUG00000021830	DENN domain containing 2B [Source:VGNC Symbol;Acc:VGNC:77898]	0.641675808	1.28E-06	8.54E-06
ENSMMUG00000021828	ribosomal protein L27a [Source:NCBI gene;Acc:708863]	0.465424398	0.000128203	0.000570601
ENSMMUG00000018471	serine/threonine kinase 33 [Source:VGNC Symbol;Acc:VGNC:78118]	1.65863807	2.00E-05	0.000106412
ENSMMUG00000050922		-0.977447702	0.015082697	0.038432369
ENSMMUG00000035632	U6 spliceosomal RNA [Source:RFAM;Acc:RF00026]	-0.869095444	0.001629306	0.005541881
ENSMMUG00000050864		-1.453406304	0.000149958	0.000656204

ENSMMUG00000011068		-2.520437795	0.01640072	0.041308199
ENSMMUG00000041831	hemoglobin subunit epsilon 1 [Source:NCBI gene;Acc:715414]	0.949044685	0.000345915	0.001396656
ENSMMUG00000058081	olfactory receptor 52D1-like [Source:NCBI gene;Acc:106992372]	-1.164066956	0.013044502	0.033974352
ENSMMUG00000001065	olfactory receptor, family 51, subfamily A, member 4 [Source:NCBI gene;Acc:100425539]	-0.594746569	0.009787033	0.026553548
ENSMMUG00000049802	olfactory receptor 51G1 [Source:NCBI gene;Acc:716561]	1.36989514	6.02E-08	5.01E-07
ENSMMUG00000053270	olfactory receptor, family 51, subfamily S, member 1 [Source:NCBI gene;Acc:717205]	0.992064363	0.010346756	0.027868216
ENSMMUG00000062162	olfactory receptor 51F1 [Source:NCBI gene;Acc:106993363]	-1.225286177	0.010838804	0.028972506
ENSMMUG00000030974	olfactory receptor, family 51, subfamily E, member 2 [Source:NCBI gene;Acc:717504]	-0.564434716	0.006211268	0.017913866
ENSMMUG00000057860	olfactory receptor 51E1 [Source:NCBI gene;Acc:717598]	-1.286375809	0.000179806	0.000774254
ENSMMUG00000022682	tripartite motif containing 68 [Source:NCBI gene;Acc:717644]	0.614264585	1.78E-06	1.15E-05
ENSMMUG00000063820	olfactory receptor, family 52, subfamily M, member 1 [Source:NCBI gene;Acc:717723]	4.651434633	1.94E-09	2.04E-08
ENSMMUG00000003962	ribonucleotide reductase catalytic subunit M1 [Source:VGNC Symbol;Acc:VGNC:76941]	-0.915150386	0.004419163	0.013344773
ENSMMUG00000003958	stromal interaction molecule 1 [Source:VGNC Symbol;Acc:VGNC:77911]	-1.067666201	0.000687594	0.00257826
ENSMMUG00000003955	post-GPI attachment to proteins 2 [Source:VGNC Symbol;Acc:VGNC:75823]	1.890681256	2.43E-28	2.07E-26
ENSMMUG00000003950	nucleoporin 98 and 96 precursor [Source:VGNC Symbol;Acc:VGNC:75563]	1.610504205	2.10E-26	1.47E-24
ENSMMUG00000037170	U7 small nuclear RNA [Source:RFAM;Acc:RF00066]	1.823353075	0.014832221	0.037898318
ENSMMUG00000003949	cholinergic receptor nicotinic alpha 10 subunit [Source:NCBI gene;Acc:718133]	-0.967016262	3.72E-05	0.000185841
ENSMMUG00000003948	ADP-ribosyltransferase 1 [Source:VGNC Symbol;Acc:VGNC:70024]	-1.54057833	0.000513968	0.001993761
ENSMMUG00000003946	ADP-ribosyltransferase 5 [Source:VGNC Symbol;Acc:VGNC:69890]	-1.53523663	0.00025779	0.001072678
ENSMMUG00000007325	Xrcc1 N-terminal domain containing 1 [Source:NCBI gene;Acc:115945140]	-2.112502837	1.10E-09	1.20E-08
ENSMMUG00000059115		-1.743353701	3.65E-07	2.66E-06
ENSMMUG00000007326	ring finger protein 121 [Source:VGNC Symbol;Acc:VGNC:76788]	-2.473510588	1.66E-21	7.01E-20
ENSMMUG00000007330	nuclear mitotic apparatus protein 1 [Source:VGNC Symbol;Acc:VGNC:75556]	-1.577850686	3.45E-12	5.27E-11
ENSMMUG00000021280	leucine rich transmembrane and O-methyltransferase domain containing [Source:NCBI gene;Acc:718286]	-2.504887022	9.25E-20	3.17E-18
ENSMMUG00000021282	late endosomal/lysosomal adaptor, MAPK and MTOR activator 1 [Source:VGNC Symbol;Acc:VGNC:74129]	-1.940211419	7.05E-08	5.81E-07
ENSMMUG00000010453	folate receptor beta [Source:VGNC Symbol;Acc:VGNC:72690]	-2.566445511	3.14E-27	2.46E-25
ENSMMUG00000010451	inositol polyphosphate phosphatase like 1 [Source:VGNC Symbol;Acc:VGNC:73752]	3.124938185	1.60E-21	6.80E-20
ENSMMUG00000056905		4.795406415	2.68E-06	1.68E-05
ENSMMUG00000005423	caseinolytic mitochondrial matrix peptidase chaperone subunit B [Source:VGNC Symbol;Acc:VGNC:71375]	1.117667784	2.53E-05	0.000131467
ENSMMUG00000015054	ArfGAP with RhoGAP domain, ankyrin repeat and PH domain 1 [Source:VGNC Symbol;Acc:VGNC:69844]	1.04271925	6.91E-08	5.71E-07
ENSMMUG00000039301	StAR related lipid transfer domain containing 10 [Source:NCBI gene;Acc:718503]	1.231495053	0.000103707	0.000471769
ENSMMUG00000001294	FCH and double SH3 domains 2 [Source:VGNC Symbol;Acc:VGNC:72593]	-1.333766725	1.25E-06	8.31E-06
ENSMMUG00000003139	Rho guanine nucleotide exchange factor 17 [Source:VGNC Symbol;Acc:VGNC:69880]	1.029449195	4.35E-07	3.14E-06
ENSMMUG00000003140	RELT TNF receptor [Source:VGNC Symbol;Acc:VGNC:76728]	1.131037191	7.00E-15	1.48E-13
ENSMMUG00000003142	family with sequence similarity 168 member A [Source:VGNC Symbol;Acc:VGNC:72398]	0.881296826	0.001499095	0.005156941
ENSMMUG00000004791	pleckstrin homology domain containing B1 [Source:VGNC Symbol;Acc:VGNC:76069]	-1.644659802	6.58E-07	4.62E-06
ENSMMUG00000030964	RAB6A, member RAS oncogene family [Source:NCBI gene;Acc:718717]	1.566024289	8.99E-21	3.47E-19

ENSMMUG00000007382	proteasomal ATPase associated factor 1 [Source:NCBI gene;Acc:718755]	0.532369173	3.17E-06	1.95E-05
ENSMMUG00000002605	uncoupling protein 2 [Source:VGNC Symbol;Acc:VGNC:78709]	-1.89803189	0.015320342	0.038937561
ENSMMUG00000010321	C2 domain containing 3 centriole elongation regulator [Source:VGNC Symbol;Acc:VGNC:70375]	-0.670782829	7.60E-05	0.000355056
ENSMMUG00000017398	protein phosphatase methylesterase 1 [Source:VGNC Symbol;Acc:VGNC:76345]	3.300371549	0.005747018	0.016740969
ENSMMUG00000017402	phosphoglucomutase 2 like 1 [Source:VGNC Symbol;Acc:VGNC:75839]	0.77846333	7.69E-05	0.000358839
ENSMMUG00000013094	lipoyl(octanoyl) transferase 2 [Source:NCBI gene;Acc:718425]	-0.72623748	6.96E-06	4.03E-05
ENSMMUG00000013093	uncharacterized LOC106993385 [Source:NCBI gene;Acc:106993385]	-0.850588076	7.95E-09	7.57E-08
ENSMMUG00000021039	X-ray radiation resistance associated 1 [Source:NCBI gene;Acc:693788]	3.113207029	8.18E-25	4.84E-23
ENSMMUG00000010686	neuraminidase 3 [Source:VGNC Symbol;Acc:VGNC:75318]	-0.602242225	0.012817697	0.033489509
ENSMMUG00000064190		-0.873859852	0.004274391	0.012968335
ENSMMUG00000009126	solute carrier organic anion transporter family member 2B1 [Source:VGNC Symbol;Acc:VGNC:77603]	-1.017749112	1.78E-06	1.15E-05
ENSMMUG00000058539		-1.249143647	0.001766291	0.005966765
ENSMMUG00000010688	olfactory receptor, family 2, subfamily AT, member 4 [Source:NCBI gene;Acc:694711]	-1.166066471	0.00202578	0.006729911
ENSMMUG00000045580	trophoblast glycoprotein like [Source:NCBI gene;Acc:695023]	-1.091518826	0.001119517	0.003988094
ENSMMUG00000017529	arrestin beta 1 [Source:VGNC Symbol;Acc:VGNC:70021]	-0.762413132	0.000116163	0.000521198
ENSMMUG00000007676	microtubule associated protein 6 [Source:VGNC Symbol;Acc:VGNC:74640]	0.423604454	0.002124806	0.007007088
ENSMMUG00000000187	UV radiation resistance associated [Source:VGNC Symbol;Acc:VGNC:78751]	1.085001028	1.23E-05	6.78E-05
ENSMMUG00000022401	EMSY transcriptional repressor, BRCA2 interacting [Source:VGNC Symbol;Acc:VGNC:72065]	2.75790389	1.04E-26	7.73E-25
ENSMMUG00000062043		1.866074228	0.002199245	0.007212473
ENSMMUG00000018168	alkaline ceramidase 3 [Source:VGNC Symbol;Acc:VGNC:69415]	-0.546173969	0.000610853	0.002319851
ENSMMUG00000050318	UDP-GlcNAc:betaGal beta-1,3-N-acetylglucosaminyltransferase 6 [Source:NCBI gene;Acc:697946]	-0.526766054	0.00430705	0.013054068
ENSMMUG00000018174	calpain 5 [Source:VGNC Symbol;Acc:VGNC:70534]	-1.001841625	0.003232413	0.010149661
ENSMMUG00000055575	olfactory marker protein [Source:HGNC Symbol;Acc:HGNC:8136]	-1.372957854	3.03E-05	0.000154684
ENSMMUG00000001387	p21 (RAC1) activated kinase 1 [Source:NCBI gene;Acc:698585]	2.026773379	2.88E-23	1.47E-21
ENSMMUG00000018944	aquaporin 11 [Source:VGNC Symbol;Acc:VGNC:69866]	-2.078314917	0.000669024	0.002514352
ENSMMUG00000018945	chloride nucleotide-sensitive channel 1A [Source:VGNC Symbol;Acc:VGNC:71261]	-1.638166716	2.30E-05	0.000120684
ENSMMUG00000017355	remodeling and spacing factor 1 [Source:VGNC Symbol;Acc:VGNC:77044]	-2.075746695	1.31E-16	3.26E-15
ENSMMUG00000063047		2.800824806	0.010251156	0.027650898
ENSMMUG00000007921	discs large MAGUK scaffold protein 2 [Source:VGNC Symbol;Acc:VGNC:81388]	-1.185885351	0.002242199	0.007333878
ENSMMUG00000051202		-4.347045258	3.77E-06	2.29E-05
ENSMMUG00000060971		-1.057983397	3.42E-05	0.0001724
ENSMMUG00000051769		-0.80653737	0.002479544	0.008017648
ENSMMUG00000050245		-1.52988873	1.49E-05	8.15E-05
ENSMMUG00000050119		1.409182628	3.12E-18	9.18E-17
ENSMMUG00000054765	U6 spliceosomal RNA [Source:RFAM;Acc:RF00026]	3.005877026	0.005315442	0.015643582
ENSMMUG00000016540	transmembrane protein 126B [Source:VGNC Symbol;Acc:VGNC:79893]	-0.46261257	0.013094785	0.034081373

ENSMMUG00000016543	CREB/ATF bZIP transcription factor [Source:VGNC Symbol;Acc:VGNC:71538]	-1.463706852	1.98E-07	1.52E-06
ENSMMUG00000063415	coiled-coil domain containing 89 [Source:NCBI gene;Acc:703071]	-1.708049919	0.002496155	0.008066675
ENSMMUG00000012449	synaptotagmin like 2 [Source:VGNC Symbol;Acc:VGNC:78123]	-1.683820913	3.66E-05	0.000182944
ENSMMUG00000013045	coiled-coil domain containing 81 [Source:VGNC Symbol;Acc:VGNC:70868]	-1.548040257	0.000167572	0.000725151
ENSMMUG00000013055	malic enzyme 3 [Source:VGNC Symbol;Acc:VGNC:74695]	-1.928464001	1.18E-06	7.93E-06
ENSMMUG00000011116	NADPH oxidase 4 [Source:VGNC Symbol;Acc:VGNC:75376]	-1.422207116	8.88E-12	1.28E-10
ENSMMUG00000057627		-0.886260558	5.09E-05	0.000247227
ENSMMUG00000011646	TATA-box binding protein associated factor, RNA polymerase I subunit D [Source:NCBI gene;Acc:696602]	-1.619603835	0.005542079	0.016223654
ENSMMUG00000013601	chromosome 14 C11orf54 homolog [Source:NCBI gene;Acc:696483]	-0.916948661	4.97E-06	2.96E-05
ENSMMUG00000061600	V-set and transmembrane domain containing 5 [Source:NCBI gene;Acc:697779]	-0.73018586	0.013379864	0.034744071
ENSMMUG00000004112	MRE11 homolog, double strand break repair nuclease [Source:VGNC Symbol;Acc:VGNC:74926]	-0.641531306	1.02E-06	6.90E-06
ENSMMUG00000062781	chromosome 14 C11orf97 homolog [Source:NCBI gene;Acc:106993442]	1.87584743	1.95E-15	4.30E-14
ENSMMUG00000009394	sestrin 3 [Source:VGNC Symbol;Acc:VGNC:77291]	-0.872720204	8.54E-09	8.07E-08
ENSMMUG00000061815		-2.309136295	6.79E-27	5.08E-25
ENSMMUG00000025977	Y RNA [Source:RFAM;Acc:RF00019]	-2.740483443	0.019962192	0.048778928
ENSMMUG00000016737	contactin 5 [Source:VGNC Symbol;Acc:VGNC:71287]	3.714341028	3.45E-06	2.11E-05
ENSMMUG00000064295		2.726821333	0.000802999	0.002953492
ENSMMUG00000020784	centrosomal protein 126 [Source:NCBI gene;Acc:701911]	-0.766556588	7.11E-05	0.000334638
ENSMMUG00000003394	cilia and flagella associated protein 300 [Source:VGNC Symbol;Acc:VGNC:71120]	-2.236338928	1.38E-48	9.31E-46
ENSMMUG00000050759	Metazoan signal recognition particle RNA [Source:RFAM;Acc:RF00017]	-2.322444586	0.000135648	0.000599956
ENSMMUG00000052815		-1.96420097	0.000337562	0.00136665
ENSMMUG00000003400	baculoviral IAP repeat containing 2 [Source:NCBI gene;Acc:702592]	4.201432377	4.79E-23	2.38E-21
ENSMMUG00000011015	matrix metallopeptidase 27 [Source:VGNC Symbol;Acc:VGNC:74749]	-0.995554149	1.92E-07	1.47E-06
ENSMMUG00000018308	dynein cytoplasmic 2 heavy chain 1 [Source:NCBI gene;Acc:704215]	-1.347052489	0.00721201	0.020376093
ENSMMUG00000057934		2.018859253	9.75E-07	6.62E-06
ENSMMUG00000013419	guanylate cyclase 1 soluble subunit alpha 2 [Source:VGNC Symbol;Acc:VGNC:82154]	-1.197370346	0.000565055	0.002165997
ENSMMUG00000052457	Metazoan signal recognition particle RNA [Source:RFAM;Acc:RF00017]	-2.24138674	0.009482596	0.025860656
ENSMMUG00000005440	ELMO domain containing 1 [Source:VGNC Symbol;Acc:VGNC:72109]	0.996092431	0.005097673	0.015086508
ENSMMUG00000010358	solute carrier family 35 member F2 [Source:VGNC Symbol;Acc:VGNC:77565]	-0.965218859	0.018556582	0.04579699
ENSMMUG00000052526	RAB39A, member RAS oncogene family [Source:NCBI gene;Acc:707451]	-1.931340817	1.16E-26	8.49E-25
ENSMMUG00000001800	cullin 5 [Source:VGNC Symbol;Acc:VGNC:71570]	-2.161837108	3.32E-09	3.36E-08
ENSMMUG00000004157	nuclear protein, coactivator of histone transcription [Source:VGNC Symbol;Acc:VGNC:75379]	-2.235662853	1.08E-19	3.65E-18

ENSMMUG00000004153	ATM serine/threonine kinase [Source:VGNC Symbol;Acc:VGNC:70173]	-1.811434467	9.23E-06	5.22E-05
ENSMMUG00000000797	exophilin 5 [Source:VGNC Symbol;Acc:VGNC:72412]	-1.633438726	8.80E-09	8.30E-08
ENSMMUG00000052460		-1.612861072	0.000220544	0.000931282
ENSMMUG00000062793		-2.244977921	4.81E-10	5.53E-09
ENSMMUG00000035318	U2 spliceosomal RNA [Source:RFAM;Acc:RF00004]	-1.932513141	0.010288795	0.027737289
ENSMMUG00000065306		-0.945050974	8.46E-06	4.82E-05
ENSMMUG00000014001	protein phosphatase 2 scaffold subunit Abeta [Source:VGNC Symbol;Acc:VGNC:76375]	-1.027460368	0.000517232	0.002003277
ENSMMUG00000014003	ALG9 alpha-1,2-mannosyltransferase [Source:NCBI gene;Acc:710567]	-0.601324604	0.014739967	0.037708074
ENSMMUG00000007080	5-hydroxytryptamine receptor 3A [Source:VGNC Symbol;Acc:VGNC:73542]	-1.260221929	0.015105846	0.038471807
ENSMMUG00000007085	zinc finger and BTB domain containing 16 [Source:VGNC Symbol;Acc:VGNC:78845]	-1.979033032	0.00164462	0.005587563
ENSMMUG00000016128	SIK family kinase 3 [Source:VGNC Symbol;Acc:VGNC:77330]	0.575640707	0.000405907	0.001615133
ENSMMUG00000002053	SID1 transmembrane family member 2 [Source:VGNC Symbol;Acc:VGNC:77300]	-0.703411958	0.001345896	0.004691968
ENSMMUG00000007785	beta-secretase 1 [Source:VGNC Symbol;Acc:VGNC:70215]	-2.289361882	0.018008194	0.044696488
ENSMMUG00000059436	interleukin 10 receptor subunit alpha [Source:NCBI gene;Acc:704383]	2.406694841	1.07E-34	1.78E-32
ENSMMUG00000053399	small integral membrane protein 35 [Source:HGNC Symbol;Acc:HGNC:44179]	0.75864191	0.010753132	0.02877462
ENSMMUG00000007620	transmembrane serine protease 4 [Source:VGNC Symbol;Acc:VGNC:78577]	-2.187867915	4.88E-14	9.41E-13
ENSMMUG00000063655	sodium voltage-gated channel beta subunit 4 [Source:NCBI gene;Acc:100430940]	-1.716593085	5.63E-09	5.49E-08
ENSMMUG00000061384	sodium voltage-gated channel beta subunit 2 [Source:NCBI gene;Acc:678698]	0.59948288	1.37E-07	1.08E-06
ENSMMUG00000006268	junction adhesion molecule like [Source:VGNC Symbol;Acc:VGNC:73860]	-1.324320474	6.85E-12	1.00E-10
ENSMMUG00000017593	CD3e molecule [Source:VGNC Symbol;Acc:VGNC:70764]	-1.308190939	3.12E-14	6.10E-13
ENSMMUG00000065091	myelin protein zero like 2 [Source:NCBI gene;Acc:699330]	-1.21615963	5.07E-11	6.66E-10
ENSMMUG00000049268	CD3d molecule [Source:NCBI gene;Acc:699582]	-0.736910784	0.017526414	0.043654134
ENSMMUG00000045160	ubiquitination factor E4A [Source:NCBI gene;Acc:699836]	0.521433509	0.000169117	0.000730769
ENSMMUG00000005482	lysine methyltransferase 2A [Source:NCBI gene;Acc:705511]	2.673197386	2.76E-10	3.29E-09
ENSMMUG00000046655	pleckstrin homology like domain family B member 1 [Source:NCBI gene;Acc:706336]	-2.763992935	1.31E-21	5.63E-20
ENSMMUG00000002549	trehalase [Source:NCBI gene;Acc:706449]	-2.424672012	3.59E-13	6.24E-12
ENSMMUG00000009318	BCL9 like [Source:VGNC Symbol;Acc:VGNC:70113]	1.19133912	5.57E-11	7.28E-10
ENSMMUG00000005982	coiled-coil domain containing 84 [Source:NCBI gene;Acc:707400]	1.385924219	6.92E-08	5.71E-07
ENSMMUG00000056381	forkhead box R1 [Source:NCBI gene;Acc:707285]	2.606888268	8.85E-10	9.78E-09
ENSMMUG00000016894	ribosomal protein S25 [Source:NCBI gene;Acc:702593]	2.139338707	2.89E-08	2.53E-07
ENSMMUG00000016946	trafficking protein particle complex 4 [Source:VGNC Symbol;Acc:VGNC:79211]	1.308314492	9.06E-06	5.14E-05
ENSMMUG00000016890	solute carrier family 37 member 4 [Source:VGNC Symbol;Acc:VGNC:77591]	1.961761279	3.49E-29	3.19E-27
ENSMMUG00000030891	hypoxia up-regulated 1 [Source:VGNC Symbol;Acc:VGNC:73399]	1.518805332	7.79E-20	2.68E-18
ENSMMUG00000014864	VPS11 core subunit of CORVET and HOPS complexes [Source:VGNC Symbol;Acc:VGNC:78965]	1.554745228	3.38E-16	8.00E-15
ENSMMUG00000059341	Metazoan signal recognition particle RNA [Source:RFAM;Acc:RF00017]	2.068022294	7.65E-33	1.02E-30
ENSMMUG00000014866	hydroxymethylbilane synthase [Source:VGNC Symbol;Acc:VGNC:73479]	1.696504335	2.74E-07	2.05E-06

ENSMMUG00000044496	H2A histone family, member X [Source:NCBI gene;Acc:703073]	1.623083306	5.27E-12	7.87E-11
ENSMMUG00000014872	dolichyl-phosphate N-acetylglucosaminephosphotransferase 1 [Source:VGNC Symbol;Acc:VGNC:71971]	2.074758174	3.99E-37	7.99E-35
ENSMMUG00000014875	C2CD2 like [Source:VGNC Symbol;Acc:VGNC:70435]	1.947213843	6.01E-05	0.000287108
ENSMMUG00000014877	histone H4 transcription factor [Source:VGNC Symbol;Acc:VGNC:73408]	-0.735741432	0.011444727	0.030422173
ENSMMUG00000013799	ATP binding cassette subfamily G member 4 [Source:VGNC Symbol;Acc:VGNC:69390]	-3.890088861	3.30E-10	3.88E-09
ENSMMUG00000012190	PDZ domain containing 3 [Source:VGNC Symbol;Acc:VGNC:75832]	-2.428994786	0.001021862	0.003669301
ENSMMUG00000012191	coiled-coil domain containing 153 [Source:NCBI gene;Acc:704216]	-2.126094474	0.000215189	0.000911785
ENSMMUG00000008923	Thy-1 cell surface antigen [Source:VGNC Symbol;Acc:VGNC:78352]	3.019805909	2.56E-10	3.06E-09
ENSMMUG00000039397	tubulin folding cofactor E like [Source:NCBI gene;Acc:706506]	1.685837726	1.61E-19	5.24E-18
ENSMMUG00000053625	sterol-C5-desaturase [Source:NCBI gene;Acc:706827]	1.787806841	7.49E-16	1.72E-14
ENSMMUG00000014880	sortilin related receptor 1 [Source:NCBI gene;Acc:713011]	1.750554192	1.08E-16	2.71E-15
ENSMMUG00000054956		-3.154820986	0.003286072	0.010287617
ENSMMUG00000015522	GRAM domain containing 1B [Source:VGNC Symbol;Acc:VGNC:73028]	-1.532929938	4.57E-10	5.27E-09
ENSMMUG00000042169	olfactory receptor 6T1 [Source:NCBI gene;Acc:709477]	-0.615557005	0.009488393	0.025866337
ENSMMUG00000058858	olfactory receptor 10G9-like [Source:NCBI gene;Acc:709651]	-1.995508622	0.014575223	0.03733815
ENSMMUG00000008481	neurogranin [Source:VGNC Symbol;Acc:VGNC:75528]	-0.833323459	0.010454205	0.028101478
ENSMMUG00000057600	V-set and immunoglobulin domain containing 2 [Source:NCBI gene;Acc:712047]	-1.02213994	0.001562198	0.005341802
ENSMMUG00000008486	endothelial cell adhesion molecule [Source:VGNC Symbol;Acc:VGNC:72290]	-1.238839956	1.65E-07	1.28E-06
ENSMMUG00000065277	hepatic and glial cell adhesion molecule [Source:NCBI gene;Acc:716321]	1.647963948	0.002428493	0.007879469
ENSMMUG00000006375	coiled-coil domain containing 15 [Source:NCBI gene;Acc:716362]	1.687823049	2.88E-08	2.51E-07
ENSMMUG00000057529		-0.7813701	0.000204756	0.000871316
ENSMMUG00000010009	EI24 autophagy associated transmembrane protein [Source:VGNC Symbol;Acc:VGNC:72038]	-0.624993691	0.001846341	0.006210304
ENSMMUG00000058923	Metazoan signal recognition particle RNA [Source:RFAM;Acc:RF00017]	-1.573402083	0.010017233	0.027083968
ENSMMUG00000060925		-3.436593019	0.001121297	0.003993476
ENSMMUG00000038587	prostate and testis expressed 2 [Source:NCBI gene;Acc:717085]	-4.206278578	7.05E-07	4.92E-06
ENSMMUG00000060291	prostate and testis expressed 3 [Source:NCBI gene;Acc:100379154]	-2.677165904	0.001622454	0.005521109
ENSMMUG00000014120	sc328 [Source:NCBI gene;Acc:574167]	-3.78237525	0.000129519	0.000576116
ENSMMUG00000060101		-3.273270941	0.000127436	0.000567531
ENSMMUG00000065066	V-set and immunoglobulin domain containing 10 like 2 [Source:NCBI gene;Acc:717387]	-0.70846326	3.00E-06	1.86E-05
ENSMMUG00000007628	cell adhesion associated, oncogene regulated [Source:VGNC Symbol;Acc:VGNC:70961]	-4.066093136	4.42E-11	5.86E-10
ENSMMUG00000007933	RNA pseudouridine synthase D4 [Source:VGNC Symbol;Acc:VGNC:77108]	-2.302096085	0.020235596	0.049357548
ENSMMUG00000007935	family with sequence similarity 118 member B [Source:VGNC Symbol;Acc:VGNC:72360]	-0.683312939	0.003124307	0.009839346
ENSMMUG00000006929	ST3 beta-galactoside alpha-2,3-sialyltransferase 4 [Source:VGNC Symbol;Acc:VGNC:77827]	2.088338467	6.54E-22	2.91E-20
ENSMMUG00000006931	kirre like nephrin family adhesion molecule 3 [Source:NCBI gene;Acc:714534]	2.379246545	1.02E-31	1.12E-29
ENSMMUG00000020706	ETS proto-oncogene 1, transcription factor [Source:VGNC Symbol;Acc:VGNC:72439]	-2.374857166	4.68E-26	3.10E-24

ENSMMUG00000060836		-2.851364928	9.33E-06	5.26E-05
ENSMMUG00000003141	Fli-1 proto-oncogene, ETS transcription factor [Source:VGNC Symbol;Acc:VGNC:72669]	0.539228357	0.001689192	0.005729567
ENSMMUG00000003144	potassium inwardly rectifying channel subfamily J member 1 [Source:VGNC Symbol;Acc:VGNC:73984]	0.478329254	9.19E-05	0.000422659
ENSMMUG00000017555	PR/SET domain 10 [Source:VGNC Symbol;Acc:VGNC:76250]	-0.599629181	8.99E-07	6.15E-06
ENSMMUG00000018029	amyloid beta precursor like protein 2 [Source:VGNC Symbol;Acc:VGNC:69984]	1.291123696	2.52E-17	6.78E-16
ENSMMUG00000018034	suppression of tumorigenicity 14 [Source:VGNC Symbol;Acc:VGNC:77897]	2.317485691	1.42E-56	2.63E-53
ENSMMUG00000007107	acyl-CoA dehydrogenase family member 8 [Source:VGNC Symbol;Acc:VGNC:69593]	-2.567272003	0.002856233	0.009101406
ENSMMUG00000002250	beta-1,3-glucuronyltransferase 1 [Source:VGNC Symbol;Acc:VGNC:70207]	-2.432555867	0.000203312	0.000865914
ENSMMUG00000009187	calcium voltage-gated channel subunit alpha1 B [Source:VGNC Symbol;Acc:VGNC:70499]	2.020275294	0.01989604	0.048641325
ENSMMUG00000019611	arrestin domain containing 1 [Source:VGNC Symbol;Acc:VGNC:69920]	-1.18672188	0.003521891	0.010917567
ENSMMUG00000019607	patatin like phospholipase domain containing 7 [Source:VGNC Symbol;Acc:VGNC:76104]	0.695940196	0.002301315	0.007507368
ENSMMUG00000046202	transmembrane protein 203 [Source:NCBI gene;Acc:706164]	-0.57747091	4.72E-05	0.000231058
ENSMMUG00000001677	taperin [Source:NCBI gene;Acc:721581]	-0.462358703	0.007761586	0.021750626
ENSMMUG00000047902	SS nuclear autoantigen 1 [Source:NCBI gene;Acc:706507]	-0.43515787	0.017060417	0.042694227
ENSMMUG00000001669	anaphase promoting complex subunit 2 [Source:VGNC Symbol;Acc:VGNC:69641]	-0.541628431	0.000997178	0.003595465
ENSMMUG00000001667	transmembrane protein 210 [Source:VGNC Symbol;Acc:VGNC:79945]	0.76520952	1.63E-06	1.07E-05
ENSMMUG00000063598	leucine rich repeat containing 26 [Source:NCBI gene;Acc:706616]	-1.231693236	0.004630988	0.013910785
ENSMMUG00000022750	dipeptidyl peptidase 7 [Source:NCBI gene;Acc:721595]	-0.41564617	0.00429728	0.013032447
ENSMMUG00000021428	lipocalin 12 [Source:VGNC Symbol;Acc:VGNC:74143]	2.02093368	1.74E-20	6.51E-19
ENSMMUG00000032142	complement C8 gamma chain [Source:VGNC Symbol;Acc:VGNC:70481]	2.273640864	2.50E-08	2.21E-07
ENSMMUG00000011528	F-box and WD repeat domain containing 5 [Source:VGNC Symbol;Acc:VGNC:72503]	1.558652008	1.53E-08	1.39E-07
ENSMMUG00000011527	TNF receptor associated factor 2 [Source:VGNC Symbol;Acc:VGNC:78641]	1.335935034	2.97E-08	2.59E-07
ENSMMUG00000005048	MAM domain containing 4 [Source:VGNC Symbol;Acc:VGNC:74345]	-1.228904586	5.66E-12	8.41E-11
ENSMMUG00000005047	phosphohistidine phosphatase 1 [Source:NCBI gene;Acc:721674]	-1.953802158	0.00267533	0.008583977
ENSMMUG00000005046	apical junction component 1 homolog [Source:VGNC Symbol;Acc:VGNC:69768]	-1.357255405	0.011442068	0.030420558
ENSMMUG00000005042	RAB, member RAS oncogene family like 6 [Source:VGNC Symbol;Acc:VGNC:76529]	-1.26203369	5.67E-08	4.73E-07
ENSMMUG00000005040	coiled-coil domain containing 183 [Source:NCBI gene;Acc:709019]	-1.382602512	4.35E-05	0.000213732
ENSMMUG00000026981	EGF like domain multiple 7 [Source:VGNC Symbol;Acc:VGNC:72079]	-1.23028957	3.81E-07	2.77E-06
ENSMMUG00000056009		1.300011327	3.59E-07	2.62E-06
ENSMMUG00000003106	SEC16 homolog A, endoplasmic reticulum export factor [Source:VGNC Symbol;Acc:VGNC:77326]	1.784642013	5.72E-14	1.10E-12
ENSMMUG00000003105	inositol polyphosphate-5-phosphatase E [Source:VGNC Symbol;Acc:VGNC:73637]	1.764026963	1.48E-22	7.05E-21
ENSMMUG00000003098	peptidase, mitochondrial processing alpha subunit [Source:VGNC Symbol;Acc:VGNC:76062]	2.248194128	1.92E-29	1.81E-27
ENSMMUG00000003081	G protein signaling modulator 1 [Source:VGNC Symbol;Acc:VGNC:72960]	-0.734298708	0.001512284	0.005196286
ENSMMUG00000001409	coiled-coil domain containing 187 [Source:NCBI gene;Acc:710749]	1.839023293	3.83E-15	8.27E-14
ENSMMUG00000004885	quiescin sulfhydryl oxidase 2 [Source:VGNC Symbol;Acc:VGNC:76568]	-1.807063252	0.003408487	0.010614839
ENSMMUG00000045749		-1.651124172	0.001347578	0.004696729
ENSMMUG00000000276	NACC family member 2 [Source:VGNC Symbol;Acc:VGNC:75013]	2.952181789	0.001724895	0.005837562
ENSMMUG00000016827	UBA domain containing 1 [Source:VGNC Symbol;Acc:VGNC:79837]	2.477554599	4.97E-07	3.55E-06
ENSMMUG00000055942	spermatogenesis and oogenesis specific basic helix-loop-helix 1 [Source:NCBI gene;Acc:721833]	-1.367108008	0.003899926	0.011954506

ENSMMUG00000012147	collagen type V alpha 1 chain [Source:NCBI gene;Acc:722014]	2.859911385	9.61E-20	3.27E-18
ENSMMUG00000008183	retinoid X receptor alpha [Source:VGNC Symbol;Acc:VGNC:76951]	3.591992738	0.000113741	0.000511724
ENSMMUG00000051925		2.844197766	1.30E-19	4.33E-18
ENSMMUG00000064229		2.058179474	5.67E-08	4.73E-07
ENSMMUG00000005463	bromodomain containing 3 [Source:VGNC Symbol;Acc:VGNC:70281]	-1.001775855	1.13E-06	7.63E-06
ENSMMUG00000017214	vav guanine nucleotide exchange factor 2 [Source:VGNC Symbol;Acc:VGNC:78758]	-1.137725434	0.016727977	0.042011159
ENSMMUG00000017210	sarcosine dehydrogenase [Source:VGNC Symbol;Acc:VGNC:76971]	-2.191678333	7.66E-11	9.83E-10
ENSMMUG00000017207	dopamine beta-hydroxylase [Source:VGNC Symbol;Acc:VGNC:71740]	-2.049243004	2.11E-06	1.34E-05
ENSMMUG00000022348	surfeit 4 [Source:VGNC Symbol;Acc:VGNC:78060]	1.030860505	1.44E-05	7.87E-05
ENSMMUG00000057105	Metazoan signal recognition particle RNA [Source:RFAM;Acc:RF00017]	0.810873119	0.008563924	0.023694589
ENSMMUG00000022346	surfeit 2 [Source:VGNC Symbol;Acc:VGNC:78059]	0.859708894	0.004487081	0.013533335
ENSMMUG00000018468	senataxin [Source:VGNC Symbol;Acc:VGNC:77356]	-0.830136547	1.30E-05	7.19E-05
ENSMMUG00000006479	Rap guanine nucleotide exchange factor 1 [Source:VGNC Symbol;Acc:VGNC:76582]	-0.469347491	1.27E-05	7.03E-05
ENSMMUG00000050752	Metazoan signal recognition particle RNA [Source:RFAM;Acc:RF00017]	-1.718206119	0.006628681	0.018948214
ENSMMUG00000051984		-1.426940868	0.013570888	0.035172363
ENSMMUG00000009976	uridine-cytidine kinase 1 [Source:VGNC Symbol;Acc:VGNC:78967]	0.309826282	0.007469479	0.021015437
ENSMMUG00000009973	proline rich coiled-coil 2B [Source:VGNC Symbol;Acc:VGNC:76327]	1.469195908	0.004374936	0.013230079
ENSMMUG00000059098	Small nucleolar RNA SNORD62 [Source:RFAM;Acc:RF00153]	2.239093183	0.010576811	0.028374477
ENSMMUG00000021724	nucleoporin 214 [Source:VGNC Symbol;Acc:VGNC:75560]	0.960153549	0.016948803	0.04246507
ENSMMUG00000002267	laminin subunit gamma 3 [Source:NCBI gene;Acc:722430]	3.601371618	1.03E-11	1.48E-10
ENSMMUG00000036223		3.351473904	2.69E-06	1.68E-05
ENSMMUG00000059085	PR/SET domain 12 [Source:NCBI gene;Acc:716040]	-0.95015572	0.000324186	0.001317894
ENSMMUG00000008249	far upstream element binding protein 3 [Source:NCBI gene;Acc:722470]	-0.774299242	0.000255992	0.001065796
ENSMMUG00000001522	argininosuccinate synthase 1 [Source:NCBI gene;Acc:716168]	-0.75249359	0.001708296	0.005789313
ENSMMUG00000049177	neuronal calcium sensor 1 [Source:NCBI gene;Acc:722507]	-0.446546085	0.008940343	0.02458926
ENSMMUG00000003797	ubiquitin specific peptidase 20 [Source:VGNC Symbol;Acc:VGNC:78740]	2.269403198	5.92E-08	4.93E-07
ENSMMUG00000003793	torsin family 1 member B [Source:VGNC Symbol;Acc:VGNC:78624]	-1.026550845	0.011017158	0.029407426
ENSMMUG00000050493	paired related homeobox 2 [Source:NCBI gene;Acc:716699]	-1.606681629	0.007322193	0.020640204
ENSMMUG00000009965	chromosome 15 C9orf50 homolog [Source:VGNC Symbol;Acc:VGNC:70365]	-3.234742519	2.25E-32	2.78E-30
ENSMMUG00000049483		-2.4266237	0.00893683	0.024588077
ENSMMUG00000055560		-1.497449648	0.008719926	0.02407232
ENSMMUG00000051852	Metazoan signal recognition particle RNA [Source:RFAM;Acc:RF00017]	1.222479825	0.000292481	0.001196882
ENSMMUG00000002515	protein phosphatase 2 phosphatase activator [Source:VGNC Symbol;Acc:VGNC:76583]	1.979901106	1.13E-14	2.35E-13
ENSMMUG00000011767	carnitine O-acetyltransferase [Source:VGNC Symbol;Acc:VGNC:71483]	1.236345978	1.87E-09	1.97E-08
ENSMMUG00000032030	dolichyldiphosphatase 1 [Source:VGNC Symbol;Acc:VGNC:71947]	0.992748957	1.37E-06	9.08E-06
ENSMMUG00000005989	GLE1 RNA export mediator [Source:VGNC Symbol;Acc:VGNC:73067]	1.557731914	1.83E-17	4.98E-16
ENSMMUG00000005987	outer dense fiber of sperm tails 2 [Source:VGNC Symbol;Acc:VGNC:75580]	-0.654107178	0.001187049	0.004199411
ENSMMUG00000009961	niban apoptosis regulator 2 [Source:VGNC Symbol;Acc:VGNC:75254]	-1.006407138	0.002446871	0.00793334

ENSMMUG00000009956	leucine rich repeat and sterile alpha motif containing 1 [Source:VGNC Symbol;Acc:VGNC:74353]	-0.896277451	0.000786711	0.002904389
ENSMMUG00000060992	Metazoan signal recognition particle RNA [Source:RFAM;Acc:RF00017]	-1.365506767	0.001532718	0.00525554
ENSMMUG00000021760	zinc finger protein 79 [Source:NCBI gene;Acc:700961]	-1.203912408	1.98E-08	1.77E-07
ENSMMUG00000012167	solute carrier family 2 member 8 [Source:VGNC Symbol;Acc:VGNC:77470]	-1.633839886	3.07E-05	0.000156224
ENSMMUG00000012163	GTPase activating Rap/RanGAP domain like 3 [Source:VGNC Symbol;Acc:VGNC:72785]	-0.888993089	0.0001077	0.000487989
ENSMMUG00000023495	Ral GEF with PH domain and SH3 binding motif 1 [Source:VGNC Symbol;Acc:VGNC:76492]	0.681142031	1.39E-05	7.60E-05
ENSMMUG00000054544	Metazoan signal recognition particle RNA [Source:RFAM;Acc:RF00017]	1.838722229	0.003468431	0.010774366
ENSMMUG00000050175	zinc finger and BTB domain containing 34 [Source:NCBI gene;Acc:701665]	1.001787613	0.000251804	0.001050422
ENSMMUG00000064247		0.562537256	0.018380832	0.045454016
ENSMMUG00000052476	zinc finger and BTB domain containing 43 [Source:NCBI gene;Acc:701797]	0.945017889	3.85E-09	3.85E-08
ENSMMUG00000016826	MAPK associated protein 1 [Source:VGNC Symbol;Acc:VGNC:74531]	0.780011573	0.002799269	0.00893717
ENSMMUG00000003668	GTPase activating protein and VPS9 domains 1 [Source:VGNC Symbol;Acc:VGNC:72767]	0.80251403	2.29E-07	1.73E-06
ENSMMUG00000005215	Rab9 effector protein with kelch motifs [Source:VGNC Symbol;Acc:VGNC:76495]	0.948270914	3.00E-05	0.000153284
ENSMMUG00000051739	Metazoan signal recognition particle RNA [Source:RFAM;Acc:RF00017]	1.888318461	0.010960271	0.029270813
ENSMMUG00000061871		-1.991142104	0.002629712	0.008448578
ENSMMUG00000011183	olfactomedin like 2A [Source:VGNC Symbol;Acc:VGNC:75472]	-2.28453279	7.98E-08	6.52E-07
ENSMMUG00000011185	nuclear receptor subfamily 6 group A member 1 [Source:VGNC Symbol;Acc:VGNC:75522]	-0.687689827	0.020469033	0.049795893
ENSMMUG00000033102	mml-mir-181b-2 [Source:miRBase;Acc:MI0007646]	-3.226405411	9.03E-06	5.12E-05
ENSMMUG00000026876	mml-mir-181a-2 [Source:miRBase;Acc:MI0002808]	-3.774029511	2.65E-07	1.98E-06
ENSMMUG00000014111	nuclear receptor subfamily 5 group A member 1 [Source:VGNC Symbol;Acc:VGNC:75520]	-0.373781103	0.002203132	0.007223624
ENSMMUG00000014110	adhesion G protein-coupled receptor D2 [Source:VGNC Symbol;Acc:VGNC:69462]	0.868220423	1.05E-13	1.95E-12
ENSMMUG00000054736		2.865562992	2.05E-05	0.000108788
ENSMMUG00000063639		1.729485022	3.00E-07	2.22E-06
ENSMMUG00000038944		2.618877472	1.39E-36	2.72E-34
ENSMMUG00000058731	olfactory receptor 1B1 [Source:NCBI gene;Acc:699905]	-0.355520824	0.001204208	0.004252003
ENSMMUG00000043396	Metazoan signal recognition particle RNA [Source:RFAM;Acc:RF00017]	3.904282006	2.81E-07	2.09E-06
ENSMMUG00000005595	RAB14, member RAS oncogene family [Source:NCBI gene;Acc:699837]	2.857276373	1.95E-12	3.11E-11
ENSMMUG00000012668	centriolin [Source:VGNC Symbol;Acc:VGNC:71204]	2.652185494	4.17E-40	1.14E-37
ENSMMUG00000058237		1.233198868	5.42E-10	6.18E-09
ENSMMUG00000013105	astrotactin 2 [Source:VGNC Symbol;Acc:VGNC:70087]	0.694314212	0.000439473	0.001731503
ENSMMUG00000065273		-2.445761573	5.83E-05	0.000279132
ENSMMUG00000013107	tripartite motif containing 32 [Source:VGNC Symbol;Acc:VGNC:79032]	0.854820608	6.85E-14	1.30E-12
ENSMMUG00000013099	pappalysin 1 [Source:VGNC Symbol;Acc:VGNC:75756]	1.247892185	0.0002871	0.001176808
ENSMMUG00000003356	tenascin C [Source:VGNC Symbol;Acc:VGNC:79960]	1.236264229	4.39E-10	5.07E-09
ENSMMUG00000041301	TNF superfamily member 8 [Source:NCBI gene;Acc:702944]	-1.677198928	1.28E-05	7.07E-05
ENSMMUG00000064892		2.118708308	2.34E-13	4.13E-12
ENSMMUG00000062461	chromosome 15 C9orf43 homolog [Source:NCBI gene;Acc:704968]	-2.970850733	0.004673113	0.014017449
ENSMMUG00000044537	DNA polymerase epsilon 3, accessory subunit [Source:NCBI gene;Acc:705074]	-2.84916952	2.85E-06	1.78E-05
ENSMMUG00000031965	aminolevulinate dehydratase [Source:VGNC Symbol;Acc:VGNC:69779]	-1.453362862	0.010129127	0.027360872

ENSMMUG00000057031		-2.432054281	1.14E-05	6.35E-05
ENSMMUG00000005851	B-box and SPRY domain containing [Source:VGNC Symbol;Acc:VGNC:70342]	-2.905449136	2.88E-10	3.42E-09
ENSMMUG00000011654	hydroxysteroid dehydrogenase like 2 [Source:VGNC Symbol;Acc:VGNC:73414]	-2.696818103	0.013889037	0.035896553
ENSMMUG00000000464	polypyrimidine tract binding protein 3 [Source:VGNC Symbol;Acc:VGNC:76244]	-0.678432152	0.000371148	0.001489209
ENSMMUG00000021097	sushi domain containing 1 [Source:VGNC Symbol;Acc:VGNC:78207]	-1.630601374	6.45E-06	3.76E-05
ENSMMUG00000000695	transmembrane protein 245 [Source:VGNC Symbol;Acc:VGNC:78933]	0.887094522	5.39E-05	0.000260082
ENSMMUG00000060448	Y RNA [Source:RFAM;Acc:RF00019]	1.205052606	0.002998065	0.009486089
ENSMMUG00000000690	catenin alpha like 1 [Source:VGNC Symbol;Acc:VGNC:71544]	-1.484176733	3.18E-06	1.96E-05
ENSMMUG00000053648	actin binding transcription modulator [Source:NCBI gene;Acc:710486]	-1.64900215	2.35E-11	3.20E-10
ENSMMUG00000064066		-2.002066133	7.78E-06	4.45E-05
ENSMMUG00000019357	elongator complex protein 1 [Source:VGNC Symbol;Acc:VGNC:72257]	0.813944327	1.06E-05	5.93E-05
ENSMMUG00000019355	actin like 7A [Source:VGNC Symbol;Acc:VGNC:69417]	1.286433921	0.000889603	0.00323195
ENSMMUG00000019354	actin like 7B [Source:VGNC Symbol;Acc:VGNC:69421]	1.808118661	1.50E-05	8.20E-05
ENSMMUG00000023797	post-GPI attachment to proteins GalNAc transferase 4 [Source:VGNC Symbol;Acc:VGNC:79688]	1.717531307	1.52E-06	9.96E-06
ENSMMUG00000009775	caveolae associated protein 4 [Source:NCBI gene;Acc:714379]	-0.938793996	5.40E-08	4.53E-07
ENSMMUG00000057137		-0.942592781	0.004501391	0.013568218
ENSMMUG00000011410	nuclear receptor subfamily 4 group A member 3 [Source:VGNC Symbol;Acc:VGNC:75519]	-0.982767816	0.009877567	0.026764903
ENSMMUG00000010661	SEC61 translocon subunit beta [Source:NCBI gene;Acc:717815]	-0.457408292	0.010772027	0.028814784
ENSMMUG00000001304	transforming growth factor beta receptor 1 [Source:VGNC Symbol;Acc:VGNC:78337]	0.639063862	0.000269455	0.001115511
ENSMMUG00000009831	TBC1 domain family member 2 [Source:VGNC Symbol;Acc:VGNC:78157]	-1.850750744	1.02E-05	5.74E-05
ENSMMUG00000008670	SH2 domain containing adaptor protein B [Source:VGNC Symbol;Acc:VGNC:82236]	0.664939733	0.012347274	0.03246057
ENSMMUG00000047381	DDB1 and CUL4 associated factor 10 [Source:NCBI gene;Acc:716256]	-0.439507638	0.014798523	0.037825249
ENSMMUG00000017886	F-box protein 10 [Source:NCBI gene;Acc:718443]	-1.057530034	6.50E-06	3.79E-05
ENSMMUG00000017892	RNA polymerase I subunit E [Source:VGNC Symbol;Acc:VGNC:76047]	-1.021372967	2.29E-06	1.45E-05
ENSMMUG00000007421	zinc finger CCHC-type containing 7 [Source:VGNC Symbol;Acc:VGNC:82088]	-1.228305603	2.88E-13	5.03E-12
ENSMMUG00000019639	paired box 5 [Source:VGNC Symbol;Acc:VGNC:75764]	-2.617297439	1.05E-08	9.82E-08
ENSMMUG00000006135	glucosamine (UDP-N-acetyl)-2-epimerase/N-acetylmannosamine kinase [Source:NCBI gene;Acc:693525]	-0.901924754	0.001121585	0.003993545
ENSMMUG00000054523	Metazoan signal recognition particle RNA [Source:RFAM;Acc:RF00017]	0.776085665	3.43E-09	3.47E-08
ENSMMUG00000010213	GLI pathogenesis related 2 [Source:VGNC Symbol;Acc:VGNC:81414]	4.340308316	2.22E-05	0.000116769
ENSMMUG00000022613	reversion inducing cysteine rich protein with kazal motifs [Source:VGNC Symbol;Acc:VGNC:76722]	2.236075827	2.38E-43	1.01E-40
ENSMMUG00000059406	RGP1 homolog, RAB6A GEF complex partner 1 [Source:NCBI gene;Acc:695936]	-4.355132371	1.74E-09	1.85E-08
ENSMMUG00000016331	glucosylceramidase beta 2 [Source:VGNC Symbol;Acc:VGNC:72741]	-3.00348933	0.000301146	0.0012303
ENSMMUG00000016328	cAMP responsive element binding protein 3 [Source:VGNC Symbol;Acc:VGNC:71486]	-3.137781612	0.009526381	0.025946049
ENSMMUG00000031856	talin 1 [Source:VGNC Symbol;Acc:VGNC:78371]	-0.808239673	1.37E-06	9.08E-06

ENSMMUG00000016321	tropomyosin 2 [Source:NCBI gene;Acc:696604]	0.408396485	0.002111462	0.006975493
ENSMMUG00000050822	carbonic anhydrase 9 [Source:NCBI gene;Acc:696729]	1.780400036	1.26E-21	5.43E-20
ENSMMUG00000048375	Metazoan signal recognition particle RNA [Source:RFAM;Acc:RF00017]	1.937011942	3.58E-41	1.11E-38
ENSMMUG00000016323	Rho guanine nucleotide exchange factor 39 [Source:VGNC Symbol;Acc:VGNC:69912]	1.964386024	2.49E-29	2.34E-27
ENSMMUG00000016327	coiled-coil domain containing 107 [Source:VGNC Symbol;Acc:VGNC:70656]	2.04227652	2.63E-27	2.08E-25
ENSMMUG00000038988	RNase MRP [Source:RFAM;Acc:RF00030]	2.614131969	0.00221569	0.007256775
ENSMMUG00000016316	testis associated actin remodelling kinase 1 [Source:VGNC Symbol;Acc:VGNC:78314]	- 1.667042275	1.59E-07	1.24E-06
ENSMMUG00000049863		- 3.338875435	0.003663595	0.01130485
ENSMMUG00000016314	family with sequence similarity 166 member B [Source:VGNC Symbol;Acc:VGNC:72421]	- 2.883061008	6.30E-27	4.74E-25
ENSMMUG00000016313	RUN and SH3 domain containing 2 [Source:VGNC Symbol;Acc:VGNC:82230]	- 1.345323873	6.52E-11	8.47E-10
ENSMMUG00000059191	stomatin like 2 [Source:NCBI gene;Acc:698458]	1.314907983	0.005238811	0.0154303
ENSMMUG00000011866	phosphatidylinositol glycan anchor biosynthesis class O [Source:VGNC Symbol;Acc:VGNC:75851]	1.967511197	0.000151637	0.000662964
ENSMMUG00000020190	valosin containing protein [Source:VGNC Symbol;Acc:VGNC:78762]	2.376826027	7.51E-20	2.59E-18
ENSMMUG00000061896		2.635873084	4.04E-28	3.35E-26
ENSMMUG00000044048	Metazoan signal recognition particle RNA [Source:RFAM;Acc:RF00017]	2.30831761	2.02E-12	3.19E-11
ENSMMUG00000020188	chromosome 15 C9orf131 homolog [Source:VGNC Symbol;Acc:VGNC:70451]	0.689864553	4.07E-06	2.46E-05
ENSMMUG00000022895	DnaJ heat shock protein family (Hsp40) member B5 [Source:NCBI gene;Acc:699081]	0.733557676	1.08E-12	1.77E-11
ENSMMUG00000006927	PHD finger protein 24 [Source:NCBI gene;Acc:698904]	0.719568535	0.000717604	0.002671821
ENSMMUG00000020780	ciliary neurotrophic factor receptor [Source:VGNC Symbol;Acc:VGNC:71282]	- 0.546431607	0.006562014	0.018786615
ENSMMUG00000031826	ubiquitin associated protein 1 [Source:VGNC Symbol;Acc:VGNC:78686]	1.085046527	0.020358253	0.04958333
ENSMMUG00000058149		0.952181788	0.002580206	0.008314743
ENSMMUG00000017458	DDB1 and CUL4 associated factor 12 [Source:VGNC Symbol;Acc:VGNC:71678]	1.353938342	0.000342839	0.001386125
ENSMMUG00000012597	ubiquitin associated protein 2 [Source:VGNC Symbol;Acc:VGNC:78949]	1.883457427	0.000432985	0.001710487
ENSMMUG00000056939		1.647182998	0.001301243	0.004555581
ENSMMUG00000035888	small nucleolar RNA, C/D box 121A [Source:HGNC Symbol;Acc:HGNC:33560]	2.226502205	1.74E-05	9.37E-05
ENSMMUG00000053200	ubiquitin conjugating enzyme E2 R2 [Source:NCBI gene;Acc:701507]	1.296090637	9.77E-06	5.49E-05
ENSMMUG00000000389	nucleolar protein 6 [Source:VGNC Symbol;Acc:VGNC:75218]	1.055214925	5.65E-10	6.43E-09
ENSMMUG00000003314	aquaporin 3 (Gill blood group) [Source:NCBI gene;Acc:702721]	1.136363969	3.90E-05	0.000193227
ENSMMUG00000001545	aquaporin 7 [Source:NCBI gene;Acc:702836]	1.169408321	0.002789354	0.008909353
ENSMMUG00000043096	nuclear transcription factor, X-box binding 1 [Source:NCBI gene;Acc:702945]	- 0.834528943	0.001981259	0.006599746
ENSMMUG00000020149	charged multivesicular body protein 5 [Source:NCBI gene;Acc:703191]	- 0.805239711	0.000908796	0.003295218
ENSMMUG00000020148	BAG cochaperone 1 [Source:NCBI gene;Acc:702101]	-1.22642889	0.001386658	0.004814831
ENSMMUG00000060647	Metazoan signal recognition particle RNA [Source:RFAM;Acc:RF00017]	- 1.156288613	0.001187413	0.0041997
ENSMMUG00000012442	DExD/H-box helicase 58 [Source:VGNC Symbol;Acc:VGNC:71672]	0.435025713	2.89E-06	1.80E-05
ENSMMUG00000008196	aconitase 1 [Source:VGNC Symbol;Acc:VGNC:69378]	0.875693509	2.02E-06	1.30E-05
ENSMMUG00000061225		2.023127225	0.004644632	0.013940475
ENSMMUG00000062843	MOB kinase activator 3B [Source:NCBI gene;Acc:706919]	0.706237867	0.000243321	0.001018187
ENSMMUG00000006076	intraflagellar transport 74 [Source:VGNC Symbol;Acc:VGNC:73430]	1.717769694	0.001859946	0.006250395
ENSMMUG00000006079	leucine rich repeat containing 19 [Source:VGNC Symbol;Acc:VGNC:74337]	2.585055532	3.16E-07	2.33E-06
ENSMMUG00000064752	phospholipase A2 activating protein [Source:NCBI gene;Acc:707876]	- 2.786601784	5.08E-06	3.02E-05
ENSMMUG00000006073	caspase activity and apoptosis inhibitor 1 [Source:VGNC Symbol;Acc:VGNC:70634]	- 3.092621103	0.000344664	0.001392365
ENSMMUG00000050918		0.709254184	7.09E-05	0.000333522

ENSMMUG00000054514	interferon alpha 6 [Source:NCBI gene;Acc:106995851]	-3.119922729	0.005991939	0.017365734
ENSMMUG00000010959	kelch like family member 9 [Source:VGNC Symbol;Acc:VGNC:74080]	-4.191602307	4.23E-05	0.000208372
ENSMMUG00000019070	focadhesin [Source:VGNC Symbol;Acc:VGNC:72556]	1.337414308	9.63E-14	1.80E-12
ENSMMUG00000051114		-1.276320907	0.009992061	0.027035625
ENSMMUG00000018396	HAUS augmin like complex subunit 6 [Source:VGNC Symbol;Acc:VGNC:73237]	2.088696029	3.03E-09	3.09E-08
ENSMMUG00000002224	PC4 and SFRS1 interacting protein 1 [Source:VGNC Symbol;Acc:VGNC:76437]	-2.522713407	0.004033311	0.012322216
ENSMMUG00000013466	tetratricopeptide repeat domain 39B [Source:VGNC Symbol;Acc:VGNC:79402]	-0.80421599	3.93E-06	2.38E-05
ENSMMUG00000055400	U6 spliceosomal RNA [Source:RFAM;Acc:RF00026]	-1.128972219	1.42E-06	9.36E-06
ENSMMUG00000002176	ubiquitin like with PHD and ring finger domains 2 [Source:VGNC Symbol;Acc:VGNC:78714]	-1.115569258	1.24E-18	3.76E-17
ENSMMUG00000050923		-1.423966773	8.40E-10	9.31E-09
ENSMMUG00000002175	TPD52 like 3 [Source:HGNC Symbol;Acc:HGNC:23382]	-0.79485197	0.002498731	0.008073243
ENSMMUG00000021344	interleukin 33 [Source:VGNC Symbol;Acc:VGNC:73606]	-0.888569467	7.74E-05	0.000360809
ENSMMUG00000064013		-1.135495636	0.001441906	0.004982169
ENSMMUG00000005469	endoplasmic reticulum metallopeptidase 1 [Source:VGNC Symbol;Acc:VGNC:72322]	-0.628867689	9.64E-09	9.05E-08
ENSMMUG00000057739		0.456132253	0.002746032	0.008786119
ENSMMUG00000052972		0.399369665	0.006063137	0.017544618
ENSMMUG00000060228		0.541610603	0.000297911	0.001218093
ENSMMUG00000065127	insulin like 6 [Source:NCBI gene;Acc:693735]	-0.543550513	0.000535669	0.002068206
ENSMMUG00000057331	RNA terminal phosphate cyclase like 1 [Source:NCBI gene;Acc:694568]	3.184102702	4.90E-42	1.73E-39
ENSMMUG00000026950	microRNA 101-2 [Source:HGNC Symbol;Acc:HGNC:31489]	3.322693657	5.25E-11	6.89E-10
ENSMMUG00000026024	U6 spliceosomal RNA [Source:RFAM;Acc:RF00026]	1.34278863	0.000936773	0.003388375
ENSMMUG00000057520		2.656326253	0.006515082	0.01866306
ENSMMUG00000056693		-0.905536946	1.94E-19	6.31E-18
ENSMMUG00000015093	very low density lipoprotein receptor [Source:VGNC Symbol;Acc:VGNC:78774]	-0.400935847	0.004836727	0.01444686
ENSMMUG00000057073		-4.709611135	5.11E-09	5.01E-08
ENSMMUG00000015279	SWI/SNF related, matrix associated, actin dependent regulator of chromatin, subfamily a, member 2 [Source:VGNC Symbol;Acc:VGNC:77647]	-1.420390099	4.98E-06	2.96E-05
ENSMMUG00000055563		-0.530250559	0.005801621	0.016883445
ENSMMUG00000012472	phosphatidylinositol-4-phosphate 5-kinase type 1 beta [Source:VGNC Symbol;Acc:VGNC:76001]	1.008052845	9.94E-12	1.43E-10
ENSMMUG00000027986	U6 spliceosomal RNA [Source:RFAM;Acc:RF00026]	2.599185393	1.93E-14	3.88E-13
ENSMMUG00000003780	tight junction protein 2 [Source:NCBI gene;Acc:699586]	2.78079975	0.018484829	0.045673111
ENSMMUG00000011401	amyloid beta precursor protein binding family A member 1 [Source:VGNC Symbol;Acc:VGNC:69976]	-1.677029666	0.000119214	0.000533475
ENSMMUG00000015844	chromosome 15 C9orf135 homolog [Source:VGNC Symbol;Acc:VGNC:70452]	-2.406646328	0.005740525	0.016728628
ENSMMUG00000063917		-1.235069592	0.015798545	0.039974821
ENSMMUG00000012667	annexin A1 [Source:VGNC Symbol;Acc:VGNC:69944]	0.621185882	3.82E-09	3.83E-08
ENSMMUG00000022023	proprotein convertase subtilisin/kexin type 5 [Source:VGNC Symbol;Acc:VGNC:75787]	1.09196817	0.000917246	0.003322607
ENSMMUG00000056840		1.825167475	3.66E-18	1.07E-16

ENSMMUG00000064984		-2.504246369	0.007339083	0.020677062
ENSMMUG00000008685	solute carrier family 28 member 3 [Source:VGNC Symbol;Acc:VGNC:77493]	-3.410024475	0.007565125	0.021264368
ENSMMUG00000053862		-3.572569739	0.00396807	0.012145815
ENSMMUG00000023117	neurotrophic receptor tyrosine kinase 2 [Source:VGNC Symbol;Acc:VGNC:75541]	-0.724493262	7.79E-09	7.44E-08
ENSMMUG00000061650		3.135223754	0.004507694	0.013584454
ENSMMUG00000054687		2.576966385	0.01935235	0.047500058
ENSMMUG00000060761		2.137600499	1.98E-32	2.49E-30
ENSMMUG00000017053	death associated protein kinase 1 [Source:VGNC Symbol;Acc:VGNC:71732]	3.162739335	0.00378943	0.011639862
ENSMMUG00000054211		2.583476995	2.66E-08	2.33E-07
ENSMMUG00000057499		2.604112153	6.56E-12	9.62E-11
ENSMMUG00000033550		3.216047707	0.000141375	0.000621654
ENSMMUG00000049479		3.643244544	1.10E-05	6.14E-05
ENSMMUG00000053778		-1.46265711	7.03E-07	4.90E-06
ENSMMUG00000001748	spleen associated tyrosine kinase [Source:VGNC Symbol;Acc:VGNC:78063]	2.639629025	7.93E-10	8.84E-09
ENSMMUG00000020009	receptor tyrosine kinase like orphan receptor 2 [Source:VGNC Symbol;Acc:VGNC:76716]	1.582147538	4.58E-11	6.07E-10
ENSMMUG00000060533		1.377839881	1.38E-16	3.43E-15
ENSMMUG00000003006	serine palmitoyltransferase long chain base subunit 1 [Source:VGNC Symbol;Acc:VGNC:77873]	1.702728194	4.11E-11	5.47E-10
ENSMMUG00000004629	nucleolar protein 8 [Source:VGNC Symbol;Acc:VGNC:75237]	-0.551629519	3.03E-05	0.000154458
ENSMMUG00000004630	centromere protein P [Source:NCBI gene;Acc:100427110]	-0.626220133	0.014985299	0.038236748
ENSMMUG00000061517	osteomodulin [Source:NCBI gene;Acc:706272]	3.140461193	0.004439548	0.013398147
ENSMMUG00000009493	family with sequence similarity 120A [Source:NCBI gene;Acc:717459]	-1.788798035	1.79E-06	1.16E-05
ENSMMUG00000002517	protein tyrosine phosphatase domain containing 1 [Source:VGNC Symbol;Acc:VGNC:76567]	-1.52155221	1.80E-06	1.16E-05
ENSMMUG00000016712	contactin associated protein like 3 [Source:NCBI gene;Acc:711939]	-1.443594442	0.003587709	0.011103032
ENSMMUG00000060049		-1.388916123	0.000143242	0.000629415
ENSMMUG00000050773		-0.867903373	0.000979354	0.003534638
ENSMMUG00000005997	cell division cycle 14B [Source:NCBI gene;Acc:710300]	2.908723623	7.08E-06	4.10E-05
ENSMMUG00000019423	zinc finger protein 2 [Source:NCBI gene;Acc:703864]	-2.692051955	0.002480443	0.008017648
ENSMMUG00000060932	zinc finger protein 383-like [Source:NCBI gene;Acc:106993074]	-1.741982163	1.47E-07	1.16E-06
ENSMMUG00000049314	dual specificity phosphatase 2 [Source:NCBI gene;Acc:718442]	-3.498351327	6.40E-05	0.000303876
ENSMMUG00000057143	transmembrane protein 127 [Source:NCBI gene;Acc:705069]	-0.984909166	0.003935992	0.012052604
ENSMMUG00000015515	REV1 DNA directed polymerase [Source:NCBI gene;Acc:709475]	0.709873363	0.000695794	0.002603085
ENSMMUG00000058615		-1.422362505	0.000164793	0.000714795
ENSMMUG00000057336		0.564858033	1.69E-07	1.31E-06
ENSMMUG00000019682	mitochondrial ribosomal protein S9 [Source:VGNC Symbol;Acc:VGNC:74762]	1.700461898	0.000245314	0.001025949
ENSMMUG00000001390	NCK adaptor protein 2 [Source:VGNC Symbol;Acc:VGNC:75142]	-1.289812792	0.000289164	0.001184286
ENSMMUG00000050861		-1.092241921	0.013949893	0.036003641
ENSMMUG00000002589	sulfotransferase 1C3-like [Source:NCBI gene;Acc:114671897]	-1.301148447	8.47E-10	9.38E-09

ENSMMUG00000060346		-3.813161647	7.79E-16	1.78E-14
ENSMMUG00000010066	MER proto-oncogene, tyrosine kinase [Source:VGNC Symbol;Acc:VGNC:74706]	-0.662483635	0.000269513	0.001115511
ENSMMUG00000002231	transmembrane protein 87B [Source:VGNC Symbol;Acc:VGNC:79557]	-1.221525353	2.57E-07	1.93E-06
ENSMMUG00000044163	zinc finger CCCH-type containing 8 [Source:NCBI gene;Acc:698828]	-0.867614174	2.08E-05	0.000109854
ENSMMUG00000016430	zinc finger CCCH-type containing 6 [Source:VGNC Symbol;Acc:VGNC:79458]	-1.274342921	0.002581644	0.008317571
ENSMMUG00000059850	immunoglobulin kappa variable 1-9 [Source:NCBI gene;Acc:106993071]	-1.921604417	0.000167335	0.000724338
ENSMMUG00000050630		-1.728177099	0.001662601	0.005646066
ENSMMUG00000051042		-0.999696638	0.002804165	0.008947025
ENSMMUG00000050256		-1.328481367	0.01898617	0.046717257
ENSMMUG00000019116	dynein axonemal heavy chain 6 [Source:NCBI gene;Acc:694115]	0.295259586	0.012428539	0.032645276
ENSMMUG00000000908	succinate-CoA ligase alpha subunit [Source:VGNC Symbol;Acc:VGNC:78052]	0.816442383	0.005200756	0.01533649
ENSMMUG00000046169		2.469391706	2.49E-11	3.38E-10
ENSMMUG00000000293	catenin alpha 2 [Source:VGNC Symbol;Acc:VGNC:71543]	1.804043562	2.61E-15	5.70E-14
ENSMMUG00000062818	regenerating islet-derived protein 3-alpha [Source:NCBI gene;Acc:716890]	-2.010068176	5.20E-05	0.000251857
ENSMMUG00000040046	lithostathine-1-alpha [Source:NCBI gene;Acc:711810]	-1.896403515	0.00017361	0.000748766
ENSMMUG00000049292	regenerating islet-derived 1 alpha [Source:NCBI gene;Acc:711892]	-3.482011501	0.003017	0.009529736
ENSMMUG00000054863		-1.72463108	0.000831376	0.003048036
ENSMMUG00000056785		-0.760705134	0.000188512	0.000808689
ENSMMUG00000056765		-0.559849871	0.00022208	0.000937066
ENSMMUG00000062747		0.384810181	0.000188859	0.000809942
ENSMMUG00000018512	GC-rich sequence DNA-binding factor 2 [Source:VGNC Symbol;Acc:VGNC:72715]	-0.492499137	0.002138804	0.007045863
ENSMMUG00000023758	mitochondrial ribosomal protein L19 [Source:VGNC Symbol;Acc:VGNC:74743]	-0.385961454	0.00219773	0.0072091
ENSMMUG00000048322	eva-1 homolog A, regulator of programmed cell death [Source:NCBI gene;Acc:710843]	-1.501584436	9.61E-05	0.000440542
ENSMMUG00000063035		-2.964495544	0.013030661	0.033956196
ENSMMUG00000051843		-1.112248745	2.20E-05	0.000115691
ENSMMUG00000053587		2.294515828	0.008160352	0.022722123
ENSMMUG00000001845	DNA polymerase epsilon 4, accessory subunit [Source:VGNC Symbol;Acc:VGNC:76065]	2.676369204	2.75E-13	4.82E-12
ENSMMUG00000058044		-0.920027033	3.74E-08	3.18E-07
ENSMMUG00000010864	STAM binding protein [Source:VGNC Symbol;Acc:VGNC:77935]	3.3816004	0.006422738	0.018448422
ENSMMUG00000030805	TP53RK binding protein [Source:VGNC Symbol;Acc:VGNC:79426]	3.240840216	0.019091331	0.046906641
ENSMMUG00000008396	methylmalonyl-CoA epimerase [Source:NCBI gene;Acc:704094]	-1.069423448	1.03E-09	1.13E-08
ENSMMUG00000002093	testis expressed 261 [Source:NCBI gene;Acc:703863]	-1.337387445	1.52E-12	2.45E-11
ENSMMUG00000055845	ankyrin repeat domain 53 [Source:NCBI gene;Acc:703757]	-1.86117898	1.51E-11	2.11E-10

ENSMMUG00000041775	Metazoan signal recognition particle RNA [Source:RFAM;Acc:RF00017]	-1.139539782	0.010546639	0.028319149
ENSMMUG00000002090	ATPase H+ transporting V1 subunit B1 [Source:VGNC Symbol;Acc:VGNC:70184]	-1.556172561	3.77E-23	1.90E-21
ENSMMUG00000061424	ventral anterior homeobox 2 [Source:NCBI gene;Acc:703531]	3.505198842	6.52E-32	7.44E-30
ENSMMUG00000059700		3.582445105	3.25E-08	2.81E-07
ENSMMUG00000020855	CD207 molecule [Source:VGNC Symbol;Acc:VGNC:70818]	1.79732221	0.009095055	0.024949994
ENSMMUG00000020854	C-type lectin domain family 4 member F [Source:VGNC Symbol;Acc:VGNC:71349]	4.460283725	3.55E-05	0.000178485
ENSMMUG00000010592	adducin 2 [Source:VGNC Symbol;Acc:VGNC:69529]	2.016147223	0.000442238	0.001740087
ENSMMUG00000063699		2.829159457	0.008960051	0.02462976
ENSMMUG00000064749		-2.093211144	7.68E-06	4.40E-05
ENSMMUG00000007868	AP2 associated kinase 1 [Source:VGNC Symbol;Acc:VGNC:82094]	-0.997209612	0.000207364	0.000880897
ENSMMUG00000049539		-1.236091588	5.42E-05	0.000261862
ENSMMUG00000062212	Metazoan signal recognition particle RNA [Source:RFAM;Acc:RF00017]	-2.828366039	0.008863185	0.024404203
ENSMMUG00000058336		-0.926991703	0.002993022	0.009478222
ENSMMUG00000007552	aprataxin and PNKP like factor [Source:NCBI gene;Acc:705875]	-0.847297222	0.009212102	0.025224423
ENSMMUG00000017209	C1D nuclear receptor corepressor [Source:NCBI gene;Acc:698450]	1.593644119	0.000968199	0.003497779
ENSMMUG00000057729		3.589910611	0.016773464	0.042111134
ENSMMUG00000047067	SERTA domain containing 2 [Source:NCBI gene;Acc:696216]	-0.831113245	4.33E-05	0.000213049
ENSMMUG00000006503	aftiphilin [Source:VGNC Symbol;Acc:VGNC:69701]	-0.734755911	0.001236218	0.004352592
ENSMMUG00000059267	pellino E3 ubiquitin protein ligase 1 [Source:NCBI gene;Acc:696416]	-1.008872141	3.06E-07	2.26E-06
ENSMMUG00000008772	VPS54 subunit of GARP complex [Source:VGNC Symbol;Acc:VGNC:79159]	0.424915355	0.001448224	0.005002832
ENSMMUG00000008770	UDP-glucose pyrophosphorylase 2 [Source:VGNC Symbol;Acc:VGNC:78712]	0.569129383	0.000903463	0.003277486
ENSMMUG00000060943		0.54385881	7.70E-06	4.41E-05
ENSMMUG00000006163	malate dehydrogenase 1 [Source:VGNC Symbol;Acc:VGNC:74690]	-1.188956293	1.11E-13	2.05E-12
ENSMMUG00000003979	WD repeat containing planar cell polarity effector [Source:VGNC Symbol;Acc:VGNC:79677]	-1.315520059	1.82E-05	9.74E-05
ENSMMUG00000057986	transmembrane protein 17 [Source:NCBI gene;Acc:694384]	-1.429334571	0.000927146	0.003355193
ENSMMUG00000063238		2.313946264	3.16E-06	1.95E-05
ENSMMUG00000006941	exportin 1 [Source:VGNC Symbol;Acc:VGNC:78812]	1.066311423	6.46E-07	4.54E-06
ENSMMUG00000008098	ubiquitin specific peptidase 34 [Source:VGNC Symbol;Acc:VGNC:79223]	-0.626389163	3.03E-05	0.000154501
ENSMMUG00000005685	pseudouridine synthase 10 [Source:VGNC Symbol;Acc:VGNC:76547]	1.339928671	1.05E-08	9.77E-08
ENSMMUG00000055963		1.794572892	1.05E-11	1.50E-10
ENSMMUG00000010834	REL proto-oncogene, NF-kB subunit [Source:VGNC Symbol;Acc:VGNC:76892]	0.620486512	9.35E-09	8.79E-08
ENSMMUG00000049993		-0.478564788	2.71E-07	2.03E-06
ENSMMUG00000015888	coiled-coil domain containing 85A [Source:VGNC Symbol;Acc:VGNC:70905]	-2.906006426	0.016376883	0.041255167
ENSMMUG00000007003	polyribonucleotide nucleotidyltransferase 1 [Source:VGNC Symbol;Acc:VGNC:76095]	-3.197670448	2.29E-05	0.00011997
ENSMMUG00000007001	protein phosphatase 4 regulatory subunit 3B [Source:VGNC Symbol;Acc:VGNC:76342]	-3.382284591	0.010811312	0.028909442
ENSMMUG00000008104	proteasome activator subunit 4 [Source:VGNC Symbol;Acc:VGNC:76295]	0.382808349	0.005778754	0.016826807

ENSMMUG00000008105	G protein-coupled receptor 75 [Source:VGNC Symbol;Acc:VGNC:73143]	0.325005319	0.01422153	0.036533048
ENSMMUG00000062091		3.07281211	4.99E-09	4.90E-08
ENSMMUG00000065301		-0.740162865	0.003479602	0.010806807
ENSMMUG00000061344		-1.436422393	0.000919072	0.003327599
ENSMMUG00000052169	potassium two pore domain channel subfamily K member 12 [Source:NCBI gene;Acc:715556]	-1.99291284	0.003268073	0.010244246
ENSMMUG00000053577		-2.31660112	6.79E-11	8.79E-10
ENSMMUG00000000974	mutS homolog 2 [Source:VGNC Symbol;Acc:VGNC:74931]	-2.378241459	2.44E-10	2.94E-09
ENSMMUG00000059300		0.946802405	1.80E-05	9.65E-05
ENSMMUG00000058455	sperm-tail PG-rich repeat containing 4 [Source:NCBI gene;Acc:717951]	0.649888585	0.013509072	0.035036644
ENSMMUG00000003310	tetratricopeptide repeat domain 7A [Source:VGNC Symbol;Acc:VGNC:79202]	0.302203648	0.006581898	0.018832636
ENSMMUG00000048973	CXXC repeat containing interactor of PDZ3 domain [Source:NCBI gene;Acc:714949]	0.326158875	0.002209585	0.00723998
ENSMMUG00000038319	Metazoan signal recognition particle RNA [Source:RFAM;Acc:RF00017]	-2.231381112	0.005395028	0.015846359
ENSMMUG00000014425	protein kinase C epsilon [Source:VGNC Symbol;Acc:VGNC:76412]	1.15011877	2.63E-06	1.65E-05
ENSMMUG00000054734		-0.946756883	6.55E-05	0.000310662
ENSMMUG00000023752	SIX homeobox 2 [Source:VGNC Symbol;Acc:VGNC:81570]	-1.301281332	2.17E-06	1.38E-05
ENSMMUG00000053683		-0.98627905	0.000558582	0.002143833
ENSMMUG00000014824	SIX homeobox 3 [Source:HGNC Symbol;Acc:HGNC:10889]	-1.253274531	6.54E-17	1.67E-15
ENSMMUG00000006312	calmodulin-lysine N-methyltransferase [Source:VGNC Symbol;Acc:VGNC:70637]	-1.812649683	0.000642384	0.002429006
ENSMMUG00000006311	prolyl endopeptidase like [Source:VGNC Symbol;Acc:VGNC:76346]	-0.538134062	0.00705238	0.0200013
ENSMMUG00000051072		0.9693386	7.70E-09	7.36E-08
ENSMMUG00000019841	leucine rich pentatricopeptide repeat containing [Source:VGNC Symbol;Acc:VGNC:82177]	-0.944930375	2.67E-05	0.000137978
ENSMMUG00000008081	ATP binding cassette subfamily G member 8 [Source:VGNC Symbol;Acc:VGNC:69392]	1.665099946	1.07E-11	1.52E-10
ENSMMUG00000008080	ATP binding cassette subfamily G member 5 [Source:VGNC Symbol;Acc:VGNC:69391]	1.38660698	3.07E-21	1.25E-19
ENSMMUG00000062474	Metazoan signal recognition particle RNA [Source:RFAM;Acc:RF00017]	1.781896949	9.09E-12	1.31E-10
ENSMMUG00000008077	dynein cytoplasmic 2 light intermediate chain 1 [Source:VGNC Symbol;Acc:VGNC:71899]	1.34621114	1.49E-19	4.91E-18
ENSMMUG00000025150	Y RNA [Source:RFAM;Acc:RF00019]	3.689680804	0.000213431	0.000905113
ENSMMUG00000000642	THADA armadillo repeat containing [Source:VGNC Symbol;Acc:VGNC:79185]	0.40039888	0.003820623	0.011730816
ENSMMUG00000064735	3-hydroxyanthranilate 3,4-dioxygenase [Source:NCBI gene;Acc:713101]	0.741373383	1.45E-07	1.14E-06
ENSMMUG00000063947	oxoeicosanoid receptor 1 [Source:NCBI gene;Acc:713039]	1.279703945	5.27E-11	6.90E-10
ENSMMUG00000020744	metastasis associated 1 family member 3 [Source:VGNC Symbol;Acc:VGNC:74814]	1.073431186	9.79E-08	7.90E-07
ENSMMUG00000056008		0.914180405	1.39E-13	2.53E-12
ENSMMUG00000055395	Y RNA [Source:RFAM;Acc:RF00019]	0.850080261	0.005733008	0.016710007
ENSMMUG00000054310	potassium voltage-gated channel modifier subfamily G member 3 [Source:NCBI gene;Acc:712838]	3.323006902	0.009406558	0.025694858
ENSMMUG00000064710		-2.070495462	0.002425672	0.007876663
ENSMMUG00000043458	transmembrane protein 178A [Source:NCBI gene;Acc:714010]	-0.807953922	0.000143504	0.000630195
ENSMMUG00000056330		1.898475258	2.78E-06	1.73E-05
ENSMMUG00000013901	mitogen-activated protein kinase kinase kinase kinase 3 [Source:VGNC Symbol;Acc:VGNC:81466]	1.333220824	2.41E-09	2.50E-08
ENSMMUG00000020575	SOS Ras/Rac guanine nucleotide exchange factor 1 [Source:VGNC Symbol;Acc:VGNC:77837]	0.614501534	0.000998511	0.003597649
ENSMMUG00000060562		1.121500642	0.00012456	0.000555391
ENSMMUG00000002338	heterogeneous nuclear ribonucleoprotein L like [Source:VGNC Symbol;Acc:VGNC:73441]	-0.69955164	8.58E-07	5.89E-06
ENSMMUG00000013944	regulator of microtubule dynamics 2 [Source:VGNC Symbol;Acc:VGNC:76839]	1.867910055	1.63E-28	1.41E-26

ENSMMUG00000054398		1.491177577	7.85E-10	8.76E-09
ENSMMUG00000016442	glutaminyl-peptide cyclotransferase [Source:VGNC Symbol;Acc:VGNC:76636]	3.066750396	2.77E-10	3.30E-09
ENSMMUG00000016438	protein kinase D3 [Source:VGNC Symbol;Acc:VGNC:76415]	2.464842659	3.03E-20	1.09E-18
ENSMMUG00000016434	NADH:ubiquinone oxidoreductase complex assembly factor 7 [Source:VGNC Symbol;Acc:VGNC:75035]	3.017765125	3.53E-18	1.04E-16
ENSMMUG00000016433	CCAAT enhancer binding protein zeta [Source:VGNC Symbol;Acc:VGNC:71082]	3.611419952	0.00321315	0.010093415
ENSMMUG00000001278	vitrin [Source:VGNC Symbol;Acc:VGNC:78773]	0.5477897	0.004764731	0.014260536
ENSMMUG00000049720		-2.715624451	0.000489654	0.001909433
ENSMMUG00000053593		-0.797119421	0.000435878	0.001719166
ENSMMUG00000021436	latent transforming growth factor beta binding protein 1 [Source:VGNC Symbol;Acc:VGNC:74456]	1.837961896	2.50E-14	4.94E-13
ENSMMUG00000009372	tetratricopeptide repeat domain 27 [Source:VGNC Symbol;Acc:VGNC:79565]	2.007791255	0.02020714	0.049304357
ENSMMUG00000021691	spastin [Source:VGNC Symbol;Acc:VGNC:77857]	-1.817958932	5.61E-08	4.69E-07
ENSMMUG00000008288	sorting nexin 17 [Source:VGNC Symbol;Acc:VGNC:77709]	3.272607089	4.61E-06	2.76E-05
ENSMMUG00000058351		0.410567312	0.008669562	0.023955582
ENSMMUG00000048513	tudor domain containing 15 [Source:NCBI gene;Acc:703066]	-0.761856513	0.000999584	0.003600639
ENSMMUG00000007692	apolipoprotein B [Source:VGNC Symbol;Acc:VGNC:69985]	-0.990135726	9.78E-07	6.64E-06
ENSMMUG00000003355	lipid droplet associated hydrolase [Source:VGNC Symbol;Acc:VGNC:74130]	1.492236302	1.24E-18	3.78E-17
ENSMMUG00000050446	growth differentiation factor 7 [Source:NCBI gene;Acc:702462]	0.68799141	0.001231906	0.004338438
ENSMMUG00000022996	HCLS1 binding protein 3 [Source:VGNC Symbol;Acc:VGNC:82158]	2.067943674	1.35E-33	1.92E-31
ENSMMUG00000056023	succinate dehydrogenase complex, subunit D, integral membrane protein [Source:NCBI gene;Acc:711326]	-2.876985186	0.013243599	0.034435539
ENSMMUG00000064216		-1.799365954	0.00137096	0.004769257
ENSMMUG00000012058	GEN1 Holliday junction 5' flap endonuclease [Source:VGNC Symbol;Acc:VGNC:72790]	-2.598996861	8.22E-08	6.70E-07
ENSMMUG00000012056	structural maintenance of chromosomes 6 [Source:VGNC Symbol;Acc:VGNC:77746]	-3.451113346	5.06E-12	7.58E-11
ENSMMUG00000040529	visinin like 1 [Source:NCBI gene;Acc:699459]	-4.424654704	4.09E-06	2.47E-05
ENSMMUG00000052363	MYCN proto-oncogene, bHLH transcription factor [Source:NCBI gene;Acc:698699]	0.635143136	1.86E-05	9.94E-05
ENSMMUG00000064240		0.541514364	3.21E-05	0.000162728
ENSMMUG00000056755		1.711763365	0.000186594	0.000800689
ENSMMUG00000050686		1.017804429	3.82E-05	0.000190118
ENSMMUG00000020249	growth regulating estrogen receptor binding 1 [Source:VGNC Symbol;Acc:VGNC:73227]	0.456752072	0.00100866	0.003627164
ENSMMUG00000023225	E2F transcription factor 6 [Source:VGNC Symbol;Acc:VGNC:72027]	0.809212791	7.92E-08	6.47E-07
ENSMMUG00000057805		1.329604619	0.001218126	0.00429603
ENSMMUG00000059188		2.325552446	4.42E-12	6.66E-11
ENSMMUG00000016678	Rho associated coiled-coil containing protein kinase 2 [Source:VGNC Symbol;Acc:VGNC:76714]	2.836270178	2.70E-21	1.11E-19
ENSMMUG00000060435	Metazoan signal recognition particle RNA [Source:RFAM;Acc:RF00017]	3.239721349	5.24E-13	8.98E-12
ENSMMUG00000033092	U6 spliceosomal RNA [Source:RFAM;Acc:RF00026]	2.914990733	0.005931107	0.017212979
ENSMMUG00000022785	solute carrier family 66 member 3 [Source:VGNC Symbol;Acc:VGNC:76334]	2.673389134	3.72E-07	2.71E-06
ENSMMUG00000010320	ATPase H+ transporting V1 subunit C2 [Source:VGNC Symbol;Acc:VGNC:70091]	-1.395431433	0.00795557	0.02221879
ENSMMUG00000010671	nucleolar protein 10 [Source:VGNC Symbol;Acc:VGNC:75260]	-0.870725171	0.019031511	0.04678225
ENSMMUG00000062012		-3.156243861	0.020399183	0.049674858
ENSMMUG00000063262		-1.4403179	1.21E-09	1.31E-08
ENSMMUG00000037908		-1.516334901	1.73E-06	1.13E-05

ENSMMUG00000022879	EARP complex and GARP complex interacting protein 1 [Source:NCBI gene;Acc:707650]	-3.062929634	8.29E-05	0.000385015
ENSMMUG00000009310	myelin transcription factor 1 like [Source:NCBI gene;Acc:706914]	-0.843033055	2.37E-06	1.50E-05
ENSMMUG00000009662	thyroid peroxidase [Source:VGNC Symbol;Acc:VGNC:78636]	-0.578023899	0.00907524	0.024904849
ENSMMUG00000050687	Metazoan signal recognition particle RNA [Source:RFAM;Acc:RF00017]	-1.908440952	0.004058544	0.012384405
ENSMMUG00000009660	syntrophin gamma 2 [Source:VGNC Symbol;Acc:VGNC:77694]	-0.397831283	0.003443085	0.010706845
ENSMMUG00000061533		3.169791276	4.32E-39	1.02E-36
ENSMMUG00000050543		2.855716114	3.17E-16	7.53E-15
ENSMMUG00000042598	transmembrane protein 18 [Source:NCBI gene;Acc:721515]	3.930132545	5.55E-33	7.48E-31
ENSMMUG00000038858		1.023672915	2.17E-14	4.33E-13
ENSMMUG00000063735		1.373917843	0.000689639	0.002583596
ENSMMUG00000022914	RAB, member of RAS oncogene family like 2B [Source:NCBI gene;Acc:716615]	0.828302215	1.64E-05	8.90E-05
ENSMMUG00000005588	SH3 and multiple ankyrin repeat domains 3 [Source:NCBI gene;Acc:100430318]	-0.687433757	0.003995919	0.01222349
ENSMMUG00000011278	cysteine rich with EGF like domains 2 [Source:NCBI gene;Acc:720586]	0.469914305	7.80E-05	0.000363529
ENSMMUG00000000553	bromodomain containing 1 [Source:VGNC Symbol;Acc:VGNC:70280]	0.978830406	0.008026504	0.022391471
ENSMMUG00000055848		-0.872185259	0.001887361	0.006329618
ENSMMUG00000003764	TBC1 domain family member 22A [Source:VGNC Symbol;Acc:VGNC:82077]	0.754681773	8.58E-05	0.000397748
ENSMMUG00000056186		1.511310603	2.04E-05	0.000108467
ENSMMUG00000020993	cadherin EGF LAG seven-pass G-type receptor 1 [Source:NCBI gene;Acc:718440]	-3.282771047	3.04E-16	7.24E-15
ENSMMUG00000000807	tRNA 5-methylaminomethyl-2-thiouridylate methyltransferase [Source:VGNC Symbol;Acc:VGNC:79500]	-2.649337019	2.94E-07	2.18E-06
ENSMMUG00000008136	G2 and S-phase expressed 1 [Source:VGNC Symbol;Acc:VGNC:73162]	-1.746081445	3.91E-06	2.37E-05
ENSMMUG00000016262	tetratricopeptide repeat domain 38 [Source:VGNC Symbol;Acc:VGNC:79574]	-2.61222067	3.10E-20	1.11E-18
ENSMMUG00000027003	microRNA let-7a-3 [Source:HGNC Symbol;Acc:HGNC:31478]	1.277094131	0.004584744	0.013799828
ENSMMUG00000023004	structural maintenance of chromosomes 1B [Source:VGNC Symbol;Acc:VGNC:77657]	0.613110877	1.47E-06	9.67E-06
ENSMMUG00000023003	family with sequence similarity 118 member A [Source:VGNC Symbol;Acc:VGNC:72265]	0.620023453	4.11E-05	0.000202924
ENSMMUG00000031000	Rho GTPase activating protein 8 [Source:NCBI gene;Acc:716745]	-1.637655148	0.000420743	0.001666565
ENSMMUG00000058874	Metazoan signal recognition particle RNA [Source:RFAM;Acc:RF00017]	-3.129723024	0.002614569	0.008407211
ENSMMUG00000065099		-1.737669555	1.26E-09	1.37E-08
ENSMMUG00000005769	EF-hand calcium binding domain 6 [Source:VGNC Symbol;Acc:VGNC:72092]	0.958520352	0.00015136	0.00066195
ENSMMUG00000022590	metallophosphoesterase domain containing 1 [Source:NCBI gene;Acc:715021]	0.926701621	0.005524475	0.016175315
ENSMMUG00000008106	solute carrier family 25 member 17 [Source:NCBI gene;Acc:705547]	0.914708446	5.48E-05	0.000263559
ENSMMUG00000056662		-1.197983855	6.13E-05	0.000292355
ENSMMUG00000012101	synaptogyrin 1 [Source:VGNC Symbol;Acc:VGNC:78068]	-1.52602506	5.44E-08	4.56E-07
ENSMMUG00000048395	chromobox 6 [Source:NCBI gene;Acc:702133]	5.791634377	1.69E-21	7.10E-20
ENSMMUG00000004869	neuronal pentraxin receptor [Source:NCBI gene;Acc:100428434]	3.572752533	0.00147452	0.005080652
ENSMMUG00000004868	dynein axonemal light chain 4 [Source:VGNC Symbol;Acc:VGNC:71945]	4.20529385	3.29E-06	2.02E-05
ENSMMUG00000007277	GTP binding protein 1 [Source:VGNC Symbol;Acc:VGNC:73320]	-1.683365305	7.65E-06	4.39E-05
ENSMMUG00000021083	Josephin domain containing 1 [Source:VGNC Symbol;Acc:VGNC:73920]	-1.374138927	0.000123155	0.000550119

ENSMMUG00000000364	translocase of outer mitochondrial membrane 22 [Source:NCBI gene;Acc:701286]	-2.010917374	0.000169	0.000730478
ENSMMUG00000061986		-2.067174093	0.000101474	0.000463537
ENSMMUG00000017680	chibby family member 1, beta catenin antagonist [Source:NCBI gene;Acc:100429409]	-2.113416159	0.000153966	0.000671562
ENSMMUG00000012467	family with sequence similarity 227 member A [Source:VGNC Symbol;Acc:VGNC:72475]	-1.747618215	1.14E-07	9.14E-07
ENSMMUG00000061118		-1.941430227	0.000235011	0.000987874
ENSMMUG00000049788		-2.2565867	1.35E-07	1.07E-06
ENSMMUG00000006987	RNA polymerase II subunit F [Source:NCBI gene;Acc:699566]	-0.409701377	0.000614248	0.002330936
ENSMMUG00000019785	eukaryotic translation initiation factor 3 subunit L [Source:NCBI gene;Acc:699189]	-1.490940098	0.020087299	0.049044228
ENSMMUG00000015258	glycine C-acetyltransferase [Source:NCBI gene;Acc:698811]	2.730385429	2.41E-06	1.52E-05
ENSMMUG00000005991	TRIO and F-actin binding protein [Source:NCBI gene;Acc:701241]	1.133002381	2.98E-17	7.93E-16
ENSMMUG00000056629	nucleolar protein 12 [Source:NCBI gene;Acc:698318]	1.641130686	5.39E-05	0.000260082
ENSMMUG00000003007	caspase recruitment domain family member 10 [Source:VGNC Symbol;Acc:VGNC:70570]	3.661532489	1.00E-11	1.43E-10
ENSMMUG00000003005	MFNG O-fucosylpeptide 3-beta-N-acetylglucosaminyltransferase [Source:VGNC Symbol;Acc:VGNC:74712]	5.860963863	1.87E-08	1.69E-07
ENSMMUG00000052785	extracellular leucine rich repeat and fibronectin type III domain containing 2 [Source:NCBI gene;Acc:697071]	4.690660426	1.19E-07	9.46E-07
ENSMMUG00000062922		-0.535885385	0.019863484	0.048569739
ENSMMUG00000052038		-1.402651973	0.017438985	0.043443667
ENSMMUG00000000151	transmembrane serine protease 6 [Source:VGNC Symbol;Acc:VGNC:79038]	0.55127149	5.75E-05	0.000275626
ENSMMUG00000058352		1.47794257	6.12E-06	3.58E-05
ENSMMUG00000012350	mercaptopyruvate sulfurtransferase [Source:VGNC Symbol;Acc:VGNC:74922]	1.355080384	2.84E-06	1.77E-05
ENSMMUG00000014986	testis expressed 33 [Source:VGNC Symbol;Acc:VGNC:79727]	1.439067885	2.15E-13	3.82E-12
ENSMMUG00000008160	neutrophil cytosolic factor 4 [Source:VGNC Symbol;Acc:VGNC:75141]	2.517917086	2.50E-13	4.40E-12
ENSMMUG00000055820		2.757619837	7.82E-13	1.31E-11
ENSMMUG00000008778	parvalbumin [Source:VGNC Symbol;Acc:VGNC:76627]	2.416400731	1.81E-20	6.76E-19
ENSMMUG00000006101	myosin heavy chain 9 [Source:VGNC Symbol;Acc:VGNC:75102]	1.247301928	8.35E-09	7.91E-08
ENSMMUG00000060967		0.899900493	0.000249948	0.001043853
ENSMMUG00000014010	RNA binding fox-1 homolog 2 [Source:VGNC Symbol;Acc:VGNC:76685]	1.175295767	1.04E-15	2.36E-14
ENSMMUG00000048463	apolipoprotein L5 [Source:HGNC Symbol;Acc:HGNC:14869]	1.13407194	6.30E-12	9.28E-11
ENSMMUG00000059824		-2.36679894	0.005856972	0.017021144
ENSMMUG00000062825	TIMP metallopeptidase inhibitor 3 [Source:NCBI gene;Acc:574381]	-0.455694812	0.010005927	0.027068206
ENSMMUG00000051047	uncharacterized protein C22orf42-like [Source:NCBI gene;Acc:106996637]	3.153021251	0.012993634	0.033877564
ENSMMUG00000063383		2.299909935	1.15E-06	7.71E-06
ENSMMUG00000020746	proline rich 14 like [Source:VGNC Symbol;Acc:VGNC:76338]	-0.993551951	0.009455257	0.025809213
ENSMMUG00000002182	phosphatidylserine decarboxylase [Source:VGNC Symbol;Acc:VGNC:76005]	-1.702187097	1.54E-05	8.36E-05
ENSMMUG00000002181	SFI1 centrin binding protein [Source:VGNC Symbol;Acc:VGNC:77328]	1.019634776	1.78E-14	3.61E-13
ENSMMUG00000015001	LIM domain kinase 2 [Source:VGNC Symbol;Acc:VGNC:74270]	0.76162027	0.00055564	0.002134753
ENSMMUG00000056777	Metazoan signal recognition particle RNA [Source:RFAM;Acc:RF00017]	1.360396945	0.003375072	0.010524036
ENSMMUG00000004675	smoothelin [Source:VGNC Symbol;Acc:VGNC:77661]	-1.747203548	2.59E-07	1.95E-06
ENSMMUG00000051960		-2.196015493	0.004958089	0.014758067
ENSMMUG00000056113		-2.259443974	0.000651697	0.002458577

ENSMMUG00000005898	MORC family CW-type zinc finger 2 [Source:VGNC Symbol;Acc:VGNC:74810]	-2.277602494	0.001968141	0.006563419
ENSMMUG00000045603	mml-mir-3200 [Source:miRBase;Acc:MI0020871]	-2.398962226	0.004126143	0.012580323
ENSMMUG00000019805	dual specificity phosphatase 18 [Source:VGNC Symbol;Acc:VGNC:71858]	-0.826520064	1.42E-06	9.36E-06
ENSMMUG00000052343		-0.828799772	0.005778438	0.016826807
ENSMMUG00000021374	EWS RNA binding protein 1 [Source:VGNC Symbol;Acc:VGNC:72446]	-1.63233862	1.33E-11	1.87E-10
ENSMMUG00000021373	rhomboid domain containing 3 [Source:NCBI gene;Acc:714479]	-1.767378794	2.53E-12	3.97E-11
ENSMMUG00000021372	EMI domain containing 1 [Source:VGNC Symbol;Acc:VGNC:72063]	-1.254741197	2.15E-08	1.91E-07
ENSMMUG00000021370	kringle containing transmembrane protein 1 [Source:VGNC Symbol;Acc:VGNC:74066]	-1.70557216	1.68E-06	1.10E-05
ENSMMUG00000058170	U6 spliceosomal RNA [Source:RFAM;Acc:RF00026]	-1.329670389	3.61E-06	2.20E-05
ENSMMUG00000007215	X-box binding protein 1 [Source:VGNC Symbol;Acc:VGNC:78806]	-1.900045787	5.15E-10	5.90E-09
ENSMMUG00000053198	coiled-coil domain containing 117 [Source:NCBI gene;Acc:713804]	0.762232958	0.007936979	0.022179273
ENSMMUG00000020489	HscB mitochondrial iron-sulfur cluster cochaperone [Source:VGNC Symbol;Acc:VGNC:73454]	1.313256988	7.59E-13	1.28E-11
ENSMMUG00000020486	checkpoint kinase 2 [Source:VGNC Symbol;Acc:VGNC:70958]	0.792748512	1.91E-09	2.00E-08
ENSMMUG00000001058	tetratricopeptide repeat domain 28 [Source:VGNC Symbol;Acc:VGNC:79785]	3.001694386	1.13E-21	4.87E-20
ENSMMUG00000042122	Metazoan signal recognition particle RNA [Source:RFAM;Acc:RF00017]	3.50590905	0.001027778	0.003688757
ENSMMUG00000023450	tyrosylprotein sulfotransferase 2 [Source:VGNC Symbol;Acc:VGNC:78639]	-1.022377347	7.81E-05	0.000363924
ENSMMUG00000023446	tuftelin interacting protein 11 [Source:VGNC Symbol;Acc:VGNC:78399]	-1.29368039	2.28E-05	0.000119702
ENSMMUG00000023443	SRR1 domain containing [Source:VGNC Symbol;Acc:VGNC:78036]	-1.813959996	8.65E-12	1.25E-10
ENSMMUG00000023442	HPS4 biogenesis of lysosomal organelles complex 3 subunit 2 [Source:VGNC Symbol;Acc:VGNC:73396]	-1.637181906	0.00751309	0.021130121
ENSMMUG00000016052	seizure related 6 homolog like [Source:VGNC Symbol;Acc:VGNC:77176]	-1.848337114	1.15E-10	1.44E-09
ENSMMUG00000010839	small G protein signaling modulator 1 [Source:VGNC Symbol;Acc:VGNC:77336]	-0.663933976	1.17E-07	9.36E-07
ENSMMUG00000031048	transmembrane protein 211 [Source:VGNC Symbol;Acc:VGNC:79935]	-2.058852818	0.003456666	0.01074007
ENSMMUG00000006853	crystallin beta B2 [Source:NCBI gene;Acc:100144420]	2.355814716	2.39E-08	2.12E-07
ENSMMUG00000044345	ral-GDS-related protein [Source:NCBI gene;Acc:710293]	0.536895252	0.010611963	0.028443054
ENSMMUG00000031051	aspartate-rich protein 1 [Source:NCBI gene;Acc:107000598]	-0.675210094	0.000381731	0.001527129
ENSMMUG00000009106	solute carrier family 2 member 11 [Source:NCBI gene;Acc:699450]	-0.504949876	0.00088372	0.003212149
ENSMMUG00000023053	glutathione S-transferase theta 2B [Source:NCBI gene;Acc:700185]	-0.285525716	0.003486942	0.010825068
ENSMMUG00000053799	glutathione S-transferase theta 1 [Source:NCBI gene;Acc:700314]	-0.456176403	0.002112916	0.006977773
ENSMMUG00000043974	ral-GDS-related protein [Source:NCBI gene;Acc:710293]	1.098759929	0.006387951	0.018359171
ENSMMUG00000044782		-1.034164686	8.30E-11	1.06E-09
ENSMMUG00000020999	gamma-glutamyltransferase 1 [Source:NCBI gene;Acc:707545]	-0.879968191	0.000975367	0.003521103
ENSMMUG00000000263	guanylyl cyclase domain containing 1 [Source:VGNC Symbol;Acc:VGNC:73168]	-1.173957352	0.003970009	0.012149243
ENSMMUG00000000262	beta-ureidopropionase 1 [Source:VGNC Symbol;Acc:VGNC:79029]	-1.140246852	0.001189788	0.004206091

ENSMMUG00000051567		-0.6647166	0.002477599	0.008013696
ENSMMUG00000049542		2.50709031	8.16E-15	1.72E-13
ENSMMUG00000023622	BCR activator of RhoGEF and GTPase [Source:NCBI gene;Acc:709258]	1.688468015	1.13E-09	1.23E-08
ENSMMUG00000001776	G protein subunit alpha z [Source:VGNC Symbol;Acc:VGNC:73094]	0.698961987	1.11E-05	6.16E-05
ENSMMUG00000014641		0.603502534	3.02E-07	2.23E-06
ENSMMUG00000020092		0.668123625	0.001191076	0.004209642
ENSMMUG00000058253		0.816237215	0.017032598	0.042639
ENSMMUG00000044861		0.615592057	0.001456753	0.005026443
ENSMMUG00000054655	immunoglobulin lambda variable 3-10 [Source:HGNC Symbol;Acc:HGNC:5897]	-0.762784924	0.0027744	0.008864457
ENSMMUG00000014309	zinc finger protein 280A [Source:VGNC Symbol;Acc:VGNC:79152]	0.708557705	0.001129714	0.004020326
ENSMMUG00000047452		3.430629487	2.06E-05	0.000109304
ENSMMUG00000056950		1.392397614	2.89E-17	7.71E-16
ENSMMUG00000061925		1.01037862	0.008673477	0.023961936
ENSMMUG00000039205		2.252373394	2.67E-06	1.68E-05
ENSMMUG00000060270		3.409739819	5.12E-05	0.000248559
ENSMMUG00000047978	ral-GDS-related protein-like [Source:NCBI gene;Acc:106995524]	-0.883136168	0.001148147	0.004079305
ENSMMUG00000047015	ral-GDS-related protein-like [Source:NCBI gene;Acc:106995524]	-0.984954598	2.23E-05	0.000117033
ENSMMUG00000018798	stromal cell derived factor 2 like 1 [Source:NCBI gene;Acc:697928]	0.816573672	6.57E-05	0.000311517
ENSMMUG00000052464		1.515193561	0.006494984	0.018617237
ENSMMUG00000018531	peptidylprolyl isomerase like 2 [Source:VGNC Symbol;Acc:VGNC:76239]	0.604381286	0.000254851	0.001061341
ENSMMUG00000056022		0.766367482	0.000306208	0.001249607
ENSMMUG00000062755		1.7382144	2.00E-08	1.80E-07
ENSMMUG00000049130		3.031179355	6.54E-56	1.08E-52
ENSMMUG00000063914		3.164057636	5.68E-81	2.81E-77
ENSMMUG00000023263	mediator complex subunit 15 [Source:VGNC Symbol;Acc:VGNC:74511]	3.132245669	8.85E-39	2.02E-36
ENSMMUG00000011547	reticulon 4 receptor [Source:VGNC Symbol;Acc:VGNC:77047]	0.622303426	0.018641791	0.045976677
ENSMMUG00000021904	cell division cycle 45 [Source:VGNC Symbol;Acc:VGNC:70800]	0.696145313	0.001720003	0.005822333
ENSMMUG00000031106	ubiquitin recognition factor in ER associated degradation 1 [Source:NCBI gene;Acc:711716]	0.88491006	1.18E-07	9.41E-07
ENSMMUG00000009650	HIR histone cell cycle regulation defective homolog A (S. cerevisiae) [Source:NCBI gene;Acc:719142]	-0.314656213	0.002143193	0.007053606
ENSMMUG00000005432	clathrin heavy chain like 1 [Source:VGNC Symbol;Acc:VGNC:71268]	1.986513143	2.80E-18	8.26E-17
ENSMMUG00000019925	DiGeorge syndrome critical region gene 2 [Source:NCBI gene;Acc:100423715]	1.141323624	4.54E-09	4.48E-08
ENSMMUG00000014140	peroxisomal biogenesis factor 26 [Source:NCBI gene;Acc:710470]	-0.84300418	0.004149207	0.012640251
ENSMMUG00000020801	microtubule associated monooxygenase, calponin and LIM domain containing 3 [Source:NCBI gene;Acc:710292]	0.442409249	0.007033971	0.019958761
ENSMMUG00000020799	BH3 interacting domain death agonist [Source:VGNC Symbol;Acc:VGNC:70250]	2.962568077	1.96E-19	6.37E-18
ENSMMUG00000021476	BCL2 like 13 [Source:VGNC Symbol;Acc:VGNC:84544]	3.548815379	2.85E-17	7.61E-16
ENSMMUG00000058916		3.689672873	5.13E-32	5.94E-30
ENSMMUG00000021477	ATPase H+ transporting V1 subunit E1 [Source:NCBI gene;Acc:709739]	3.837193586	2.29E-22	1.07E-20
ENSMMUG00000031121	solute carrier family 25 member 18 [Source:VGNC Symbol;Acc:VGNC:77434]	-1.531760901	4.37E-09	4.33E-08
ENSMMUG00000006731	CECR2 histone acetyl-lysine reader [Source:VGNC Symbol;Acc:VGNC:84530]	-2.622238589	7.24E-22	3.20E-20
ENSMMUG00000059302	Metazoan signal recognition particle RNA [Source:RFAM;Acc:RF00017]	2.44175355	5.41E-11	7.09E-10
ENSMMUG00000022223	transmembrane protein 121B [Source:VGNC Symbol;Acc:VGNC:79139]	0.517385508	0.016417609	0.041331053
ENSMMUG00000022222	interleukin 17 receptor A [Source:VGNC Symbol;Acc:VGNC:73712]	0.558578885	0.000970514	0.00350529
ENSMMUG00000006668	tubulin alpha-3 chain [Source:NCBI gene;Acc:707215]	-1.212351137	2.82E-05	0.000144911
ENSMMUG00000054724		-0.936921701	0.001036309	0.003714882

ENSMMUG00000049841	Metazoan signal recognition particle RNA [Source:RFAM;Acc:RF00017]	-1.566255041	0.007782114	0.021795799
ENSMMUG00000055391		-1.190318088	0.018843658	0.046428218
ENSMMUG00000009953	ninein like [Source:VGNC Symbol;Acc:VGNC:75273]	0.772883661	0.007181503	0.020305374
ENSMMUG00000002397	GINS complex subunit 1 [Source:VGNC Symbol;Acc:VGNC:73025]	0.976373477	0.001407537	0.004880471
ENSMMUG00000059374		1.099942589	0.014159572	0.036430682
ENSMMUG00000009199	glycogen phosphorylase B [Source:VGNC Symbol;Acc:VGNC:76631]	0.688979719	0.019000956	0.046745883
ENSMMUG00000053965		-0.663983362	0.002382436	0.007744746
ENSMMUG00000042668	cilia and flagella associated protein 61 [Source:NCBI gene;Acc:699814]	-1.902073655	7.11E-12	1.04E-10
ENSMMUG00000007709	crooked neck pre-mRNA splicing factor 1 [Source:VGNC Symbol;Acc:VGNC:71412]	-1.729271391	1.84E-11	2.55E-10
ENSMMUG00000007708	N-alpha-acetyltransferase 20, NatB catalytic subunit [Source:VGNC Symbol;Acc:VGNC:84533]	-1.541724221	0.00018307	0.000786709
ENSMMUG00000065262	Metazoan signal recognition particle RNA [Source:RFAM;Acc:RF00017]	-2.25838796	0.002804976	0.008947686
ENSMMUG00000010114	Ras and Rab interactor 2 [Source:VGNC Symbol;Acc:VGNC:76770]	-1.695465579	3.24E-10	3.81E-09
ENSMMUG00000002321	solute carrier family 24 member 3 [Source:VGNC Symbol;Acc:VGNC:77426]	-1.242600781	0.000124749	0.000556064
ENSMMUG00000006519	taspase 1 [Source:VGNC Symbol;Acc:VGNC:78129]	3.222428945	7.48E-05	0.000350243
ENSMMUG00000031166	McKusick-Kaufman syndrome [Source:NCBI gene;Acc:718547]	3.432532009	0.000102878	0.000469009
ENSMMUG00000011064	synaptosome associated protein 25 [Source:VGNC Symbol;Acc:VGNC:77663]	2.393840691	9.12E-06	5.16E-05
ENSMMUG00000015923	ankyrin repeat and EF-hand domain containing 1 [Source:VGNC Symbol;Acc:VGNC:69615]	-0.815568387	0.006510137	0.018652498
ENSMMUG00000021137	thioredoxin related transmembrane protein 4 [Source:VGNC Symbol;Acc:VGNC:79484]	-2.264317208	9.42E-07	6.42E-06
ENSMMUG00000055897		2.351206529	6.31E-26	4.12E-24
ENSMMUG00000057687		2.467714917	2.92E-29	2.73E-27
ENSMMUG00000064554	leucine rich repeat neuronal 4 [Source:NCBI gene;Acc:718283]	1.119865271	0.013522161	0.03506446
ENSMMUG00000013722	chromogranin B [Source:VGNC Symbol;Acc:VGNC:71033]	-0.730051352	0.002741157	0.008777783
ENSMMUG00000056093	shieldin complex subunit 1 [Source:NCBI gene;Acc:718152]	1.678043423	1.29E-11	1.82E-10
ENSMMUG00000022345		1.309528805	5.10E-07	3.64E-06
ENSMMUG00000050080	Metazoan signal recognition particle RNA [Source:RFAM;Acc:RF00017]	1.265948918	0.011337481	0.030174954
ENSMMUG00000064391		1.799012134	4.02E-16	9.42E-15
ENSMMUG00000001809	glycerophosphocholine phosphodiesterase 1 [Source:VGNC Symbol;Acc:VGNC:73017]	1.186837365	0.004336772	0.013135532
ENSMMUG00000065232		-2.262569534	0.00668926	0.019102969
ENSMMUG00000013261	CDP-diacylglycerol synthase 2 [Source:VGNC Symbol;Acc:VGNC:70995]	-1.87184868	1.86E-05	9.93E-05
ENSMMUG00000020005	solute carrier family 23 member 2 [Source:NCBI gene;Acc:717939]	-0.900581046	0.000548312	0.002113166
ENSMMUG00000020004	Ras association domain family member 2 [Source:VGNC Symbol;Acc:VGNC:76674]	-1.141865049	1.86E-10	2.27E-09
ENSMMUG00000051480		0.775811155	0.012635599	0.033095362
ENSMMUG00000020912	spermine oxidase [Source:NCBI gene;Acc:718761]	-0.830535838	0.000237724	0.000997298
ENSMMUG00000018922	cell division cycle 25B [Source:VGNC Symbol;Acc:VGNC:70795]	0.601893154	3.86E-08	3.28E-07
ENSMMUG00000022070	sialic acid binding Ig like lectin 1 [Source:VGNC Symbol;Acc:VGNC:77220]	0.641050699	5.17E-07	3.69E-06
ENSMMUG00000003633	solute carrier family 4 member 11 [Source:VGNC Symbol;Acc:VGNC:77721]	0.544379499	3.01E-08	2.62E-07
ENSMMUG00000061481	inosine triphosphatase [Source:NCBI gene;Acc:717257]	0.603989098	9.15E-09	8.61E-08
ENSMMUG00000063520	DDRGK domain containing 1 [Source:NCBI gene;Acc:717226]	0.543247925	0.003263154	0.010235316
ENSMMUG00000010028	leucine zipper tumor suppressor family member 3 [Source:VGNC Symbol;Acc:VGNC:74403]	1.891199285	1.66E-15	3.71E-14

ENSMMUG00000043281	U-box domain containing 5 [Source:NCBI gene;Acc:717166]	1.696530399	0.004592657	0.01382084
ENSMMUG00000002982	FAST kinase domains 5 [Source:NCBI gene;Acc:100498658]	0.852136878	3.92E-06	2.38E-05
ENSMMUG00000039757	Metazoan signal recognition particle RNA [Source:RFAM;Acc:RF00017]	2.843745646	0.002063708	0.006842118
ENSMMUG00000051923	arginine vasopressin [Source:NCBI gene;Acc:717101]	1.939786456	0.017040069	0.042650501
ENSMMUG00000010678	oxytocin/neurophysin I prepropeptide [Source:VGNC Symbol;Acc:VGNC:75730]	1.885616114	1.82E-06	1.18E-05
ENSMMUG00000063622	mitochondrial ribosomal protein S26 [Source:NCBI gene;Acc:717038]	2.447512722	3.18E-11	4.29E-10
ENSMMUG00000005878	protein tyrosine phosphatase receptor type A [Source:VGNC Symbol;Acc:VGNC:76610]	0.634414882	2.01E-06	1.29E-05
ENSMMUG00000005204	VPS16 core subunit of CORVET and HOPS complexes [Source:VGNC Symbol;Acc:VGNC:78966]	-1.901926502	3.42E-06	2.09E-05
ENSMMUG00000005200	PC-esterase domain containing 1A [Source:VGNC Symbol;Acc:VGNC:75622]	-3.389579789	0.001352977	0.00471444
ENSMMUG00000021332	carboxypeptidase X, M14 family member 1 [Source:VGNC Symbol;Acc:VGNC:71413]	-2.520230022	0.013281769	0.034519563
ENSMMUG00000021331	EBF family member 4 [Source:VGNC Symbol;Acc:VGNC:71932]	-3.080033255	7.11E-07	4.95E-06
ENSMMUG00000061433		-1.573110471	2.54E-09	2.61E-08
ENSMMUG00000013888	NOP56 ribonucleoprotein [Source:VGNC Symbol;Acc:VGNC:75181]	-1.446620598	0.000273963	0.001131087
ENSMMUG00000010050	small nuclear ribonucleoprotein polypeptides B and B1 [Source:VGNC Symbol;Acc:VGNC:77676]	-1.869390981	3.84E-05	0.000190407
ENSMMUG00000063469	transglutaminase 6 [Source:NCBI gene;Acc:100430348]	-2.710635347	0.00694933	0.019758161
ENSMMUG00000018474	signal regulatory protein alpha [Source:NCBI gene;Acc:717811]	-1.976449053	3.25E-08	2.81E-07
ENSMMUG00000015994	NSFL1 cofactor [Source:VGNC Symbol;Acc:VGNC:75407]	-2.477435517	0.00901337	0.024758277
ENSMMUG00000018165	syndecan binding protein 2 [Source:VGNC Symbol;Acc:VGNC:77017]	-2.564781003	9.50E-05	0.000435786
ENSMMUG00000004711	transmembrane protein 74B [Source:VGNC Symbol;Acc:VGNC:78990]	-2.032966533	0.001616503	0.005503383
ENSMMUG00000004709	proteasome inhibitor subunit 1 [Source:VGNC Symbol;Acc:VGNC:76458]	-1.713790917	0.000197376	0.000842086
ENSMMUG00000047879	family with sequence similarity 110 member A [Source:NCBI gene;Acc:717055]	-1.900966628	0.000133614	0.000592375
ENSMMUG00000021670	solute carrier family 52 member 3 [Source:VGNC Symbol;Acc:VGNC:77717]	-1.46211251	7.06E-09	6.79E-08
ENSMMUG00000056387	transcription factor 15 [Source:NCBI gene;Acc:716916]	-3.344211317	3.84E-29	3.49E-27
ENSMMUG00000031224	casein kinase 2 alpha 1 [Source:NCBI gene;Acc:714841]	-1.217811963	1.23E-08	1.14E-07
ENSMMUG00000004461	TBC1 domain family member 20 [Source:VGNC Symbol;Acc:VGNC:78132]	-0.67227849	6.99E-06	4.05E-05
ENSMMUG00000026836	Y RNA [Source:RFAM;Acc:RF00019]	-1.49076019	0.00011019	0.00049775
ENSMMUG00000012601	RANBP2-type and C3HC4-type zinc finger containing 1 [Source:VGNC Symbol;Acc:VGNC:81554]	-0.972191281	1.11E-07	8.87E-07
ENSMMUG00000038935	zinc finger CCHC-type containing 3 [Source:NCBI gene;Acc:714477]	-1.748372238	1.78E-05	9.56E-05
ENSMMUG00000017286	chromosome 10 C20orf96 homolog [Source:VGNC Symbol;Acc:VGNC:70324]	-1.221739754	0.000377824	0.001515179
ENSMMUG00000050398	defensin beta 127 [Source:NCBI gene;Acc:714155]	-1.674730564	0.018195753	0.045063964
ENSMMUG00000061092	defensin beta 125 [Source:HGNC Symbol;Acc:HGNC:18105]	-0.564370367	0.002742634	0.008779034
ENSMMUG00000046141	defensin beta 123 [Source:NCBI gene;Acc:619504]	0.522491068	0.012843662	0.033551437
ENSMMUG00000005116	defensin beta 124 [Source:VGNC Symbol;Acc:VGNC:71655]	0.476580701	0.017269178	0.043085786

ENSMMUG00000058583	RRAD and GEM like GTPase 1 [Source:NCBI gene;Acc:713351]	-1.133208764	7.46E-10	8.37E-09
ENSMMUG00000005119	histocompatibility minor 13 [Source:VGNC Symbol;Acc:VGNC:81418]	-1.942341761	0.000465231	0.001823785
ENSMMUG00000051112	p53 and DNA damage regulated 1 [Source:NCBI gene;Acc:712622]	-2.046508677	7.44E-10	8.34E-09
ENSMMUG00000061404	XK related 7 [Source:NCBI gene;Acc:715770]	-3.117261879	0.007263049	0.020496864
ENSMMUG00000031238	CCM2 like scaffold protein [Source:NCBI gene;Acc:107000439]	-2.71142588	0.001515255	0.005202918
ENSMMUG00000000117	HCK proto-oncogene, Src family tyrosine kinase [Source:VGNC Symbol;Acc:VGNC:73353]	-1.846936732	3.50E-08	3.00E-07
ENSMMUG00000020671	BPI fold containing family B member 3 [Source:VGNC Symbol;Acc:VGNC:70320]	-0.52996083	0.000110308	0.000498134
ENSMMUG00000020676	BPI fold containing family B member 4 [Source:VGNC Symbol;Acc:VGNC:70321]	0.581811541	0.000439925	0.001732365
ENSMMUG00000012912	itchy E3 ubiquitin protein ligase [Source:VGNC Symbol;Acc:VGNC:73589]	-0.371702778	0.000348795	0.001406876
ENSMMUG00000055449	tumor protein p53 inducible nuclear protein 2 [Source:NCBI gene;Acc:707113]	-2.74754391	0.001687908	0.005726761
ENSMMUG00000017931	gamma-glutamyltransferase 7 [Source:VGNC Symbol;Acc:VGNC:72921]	-1.678271036	5.79E-05	0.000277212
ENSMMUG00000017932	acyl-CoA synthetase short chain family member 2 [Source:VGNC Symbol;Acc:VGNC:69410]	-1.835935653	0.00543428	0.015942705
ENSMMUG00000017940	glutathione synthetase [Source:VGNC Symbol;Acc:VGNC:73301]	-1.607405455	0.002071073	0.006863466
ENSMMUG00000000227	myosin heavy chain 7B [Source:VGNC Symbol;Acc:VGNC:74965]	-1.741884152	0.003432534	0.010676276
ENSMMUG00000000229	transient receptor potential cation channel subfamily C member 4 associated protein [Source:VGNC Symbol;Acc:VGNC:79014]	-2.308307408	7.76E-06	4.44E-05
ENSMMUG00000057979	growth differentiation factor 5 [Source:NCBI gene;Acc:705432]	0.830851144	1.24E-10	1.54E-09
ENSMMUG00000031274	GDF5 antisense RNA 1 [Source:HGNC Symbol;Acc:HGNC:33435]	0.604648212	0.009280263	0.025396993
ENSMMUG00000010633	PHD finger protein 20 [Source:VGNC Symbol;Acc:VGNC:75807]	0.423759637	3.42E-05	0.0001724
ENSMMUG00000020690	erythrocyte membrane protein band 4.1 like 1 [Source:VGNC Symbol;Acc:VGNC:72231]	-1.173616508	1.82E-14	3.67E-13
ENSMMUG00000020691	AAR2 splicing factor [Source:VGNC Symbol;Acc:VGNC:69412]	-0.953800341	0.000154802	0.000674616
ENSMMUG00000016448	DLG associated protein 4 [Source:NCBI gene;Acc:702928]	1.563319632	1.72E-15	3.83E-14
ENSMMUG00000059897	myosin light chain 9 [Source:NCBI gene;Acc:709784]	1.590891989	7.90E-07	5.46E-06
ENSMMUG00000008195	TGFB induced factor homeobox 2 [Source:NCBI gene;Acc:100429616]	1.97892918	4.30E-33	5.90E-31
ENSMMUG00000056945		1.96861836	6.51E-26	4.22E-24
ENSMMUG00000008200	Src like adaptor 2 [Source:VGNC Symbol;Acc:VGNC:77499]	2.008712239	3.19E-09	3.24E-08
ENSMMUG00000014380	maestro heat like repeat family member 8 [Source:VGNC Symbol;Acc:VGNC:74774]	-0.558418899	0.007343414	0.020684298
ENSMMUG00000010363	ribophorin II [Source:VGNC Symbol;Acc:VGNC:76925]	1.611832659	1.93E-08	1.74E-07
ENSMMUG00000031295	growth hormone releasing hormone [Source:VGNC Symbol;Acc:VGNC:73049]	0.59153804	0.012126472	0.031948051
ENSMMUG00000017224	catenin beta like 1 [Source:VGNC Symbol;Acc:VGNC:71414]	1.975658386	1.20E-14	2.47E-13
ENSMMUG00000049410	Metazoan signal recognition particle RNA [Source:RFAM;Acc:RF00017]	1.488411671	0.003665392	0.011307649
ENSMMUG00000017230	V-set and transmembrane domain containing 2 like [Source:NCBI gene;Acc:707394]	-1.97905914	6.45E-21	2.52E-19
ENSMMUG00000020172	KIAA1755 [Source:VGNC Symbol;Acc:VGNC:73942]	1.068786519	1.23E-08	1.13E-07
ENSMMUG00000020173	bactericidal permeability increasing protein [Source:VGNC Symbol;Acc:VGNC:70277]	0.897555387	3.17E-09	3.23E-08
ENSMMUG00000006738	L3MBTL histone methyl-lysine binding protein 1 [Source:NCBI gene;Acc:695694]	2.941384595	6.27E-40	1.69E-37
ENSMMUG00000007925	serum/glucocorticoid regulated kinase 2 [Source:VGNC Symbol;Acc:VGNC:77228]	3.492636077	1.56E-33	2.21E-31
ENSMMUG00000000396	intraflagellar transport 52 [Source:NCBI gene;Acc:694933]	2.695638622	6.38E-24	3.47E-22
ENSMMUG00000018137	MYB proto-oncogene like 2 [Source:VGNC Symbol;Acc:VGNC:75092]	3.094396381	2.41E-35	4.11E-33
ENSMMUG00000002810	TOX high mobility group box family member 2 [Source:VGNC Symbol;Acc:VGNC:79007]	1.901952415	7.91E-18	2.24E-16
ENSMMUG00000022865	junctophilin 2 [Source:NCBI gene;Acc:694158]	-2.389118333	0.000114464	0.000514394

ENSMMUG00000045256	cellular communication network factor 5 [Source:NCBI gene;Acc:717872]	0.89315192	0.019446028	0.047690523
ENSMMUG00000013212	serine/threonine kinase 4 [Source:VGNC Symbol;Acc:VGNC:81588]	0.629512328	0.003779753	0.011622172
ENSMMUG00000063049		0.204553776	0.006759138	0.019280249
ENSMMUG00000003402	matrilin 4 [Source:NCBI gene;Acc:717652]	2.443546768	0.009276456	0.025391259
ENSMMUG00000003409	dysbindin domain containing 2 [Source:VGNC Symbol;Acc:VGNC:82133]	-2.140270425	0.016417975	0.041331053
ENSMMUG00000039638	serine peptidase inhibitor, Kunitz type 3 [Source:NCBI gene;Acc:100423908]	0.449850776	0.001317377	0.004604456
ENSMMUG00000051201	zinc finger SWIM-type containing 3 [Source:NCBI gene;Acc:708804]	1.587765475	1.33E-11	1.87E-10
ENSMMUG00000042855	zinc finger SWIM-type containing 1 [Source:NCBI gene;Acc:717180]	1.375652192	3.28E-05	0.000166053
ENSMMUG00000009957	spermatogenesis associated 25 [Source:VGNC Symbol;Acc:VGNC:77928]	2.324846106	0.000148005	0.000648808
ENSMMUG00000058326	neuralized E3 ubiquitin protein ligase 2 [Source:NCBI gene;Acc:708904]	2.121886658	0.000598012	0.002275147
ENSMMUG00000009960	cathepsin A [Source:VGNC Symbol;Acc:VGNC:71617]	1.819454044	2.35E-16	5.68E-15
ENSMMUG00000013702	zinc finger protein 335 [Source:VGNC Symbol;Acc:VGNC:78986]	-1.433320916	0.009458211	0.025812525
ENSMMUG00000016549	matrix metallopeptidase 9 [Source:VGNC Symbol;Acc:VGNC:74903]	2.788575497	1.00E-09	1.10E-08
ENSMMUG00000004479	cadherin 22 [Source:VGNC Symbol;Acc:VGNC:70940]	-0.890579882	0.004301623	0.013040284
ENSMMUG00000016927	solute carrier family 2 member 10 [Source:VGNC Symbol;Acc:VGNC:77465]	-2.089875365	1.12E-19	3.74E-18
ENSMMUG00000065008		-2.217935125	4.80E-06	2.87E-05
ENSMMUG00000015710	zinc finger MYND-type containing 8 [Source:NCBI gene;Acc:716058]	0.476558339	9.62E-05	0.000440701
ENSMMUG00000051667		-2.313058989	0.019388746	0.047565773
ENSMMUG00000012020	nuclear receptor coactivator 3 [Source:VGNC Symbol;Acc:VGNC:75147]	-0.574893033	0.000582247	0.002223151
ENSMMUG00000012029	sulfatase 2 [Source:VGNC Symbol;Acc:VGNC:78179]	-1.543551521	9.08E-10	1.00E-08
ENSMMUG00000060780		1.00222491	1.12E-16	2.79E-15
ENSMMUG00000038447		-0.749143471	0.001495766	0.005146683
ENSMMUG00000057808		-0.866977703	0.001140283	0.004053307
ENSMMUG00000058754		-3.369686473	0.001422847	0.004924345
ENSMMUG00000019635	ADP ribosylation factor guanine nucleotide exchange factor 2 [Source:VGNC Symbol;Acc:VGNC:69836]	0.32236946	0.008277976	0.023023703
ENSMMUG00000023621	DEAD-box helicase 27 [Source:NCBI gene;Acc:703852]	-3.246307687	0.000555025	0.002133036
ENSMMUG00000002124	beta-1,4-galactosyltransferase 5 [Source:VGNC Symbol;Acc:VGNC:70212]	2.214521214	8.82E-17	2.22E-15
ENSMMUG00000002715	solute carrier family 9 member A8 [Source:VGNC Symbol;Acc:VGNC:77749]	2.00241943	5.83E-42	2.01E-39
ENSMMUG00000028471	U7 small nuclear RNA [Source:RFAM;Acc:RF00066]	2.244628617	6.16E-11	8.02E-10
ENSMMUG00000000740	spermatogenesis associated 2 [Source:VGNC Symbol;Acc:VGNC:77923]	2.304777897	3.03E-25	1.83E-23
ENSMMUG00000000757	ring finger protein 114 [Source:VGNC Symbol;Acc:VGNC:76725]	1.882771235	4.15E-07	3.00E-06
ENSMMUG00000065129		2.572741579	6.66E-06	3.87E-05
ENSMMUG00000012228	ubiquitin conjugating enzyme E2 V1 [Source:NCBI gene;Acc:100425552]	2.765412229	2.39E-42	8.87E-40
ENSMMUG00000062321		-0.5815813	0.002109648	0.006972606
ENSMMUG00000002761	activity dependent neuroprotector homeobox [Source:VGNC Symbol;Acc:VGNC:69614]	-0.622601891	0.000192225	0.000822915
ENSMMUG00000002759	dolichyl-phosphate mannosyltransferase subunit 1, catalytic [Source:VGNC Symbol;Acc:VGNC:71975]	-1.464218773	0.007090686	0.020098408
ENSMMUG00000000918	ATPase phospholipid transporting 9A (putative) [Source:VGNC Symbol;Acc:VGNC:84529]	-1.411846888	0.009487335	0.025866337
ENSMMUG00000062664		-1.395910918	0.000165606	0.000717481

ENSMMUG00000018846	ZFP64 zinc finger protein [Source:VGNC Symbol;Acc:VGNC:78994]	-0.324446118	0.001370294	0.004769177
ENSMMUG00000050197		-1.67261963	0.000984038	0.003550678
ENSMMUG00000013035	teashirt zinc finger homeobox 2 [Source:VGNC Symbol;Acc:VGNC:78875]	-1.755838382	5.30E-08	4.45E-07
ENSMMUG00000018358	zinc finger protein 217 [Source:VGNC Symbol;Acc:VGNC:78874]	-2.093374106	5.98E-16	1.39E-14
ENSMMUG00000057302		-1.973812351	5.58E-05	0.000268338
ENSMMUG00000052326	U7 small nuclear RNA [Source:RFAM;Acc:RF00066]	-4.357055263	0.000416269	0.00165149
ENSMMUG00000000252	ribonucleic acid export 1 [Source:VGNC Symbol;Acc:VGNC:82216]	-0.495927916	0.00770659	0.021616934
ENSMMUG00000031391	RNA binding motif protein 38 [Source:VGNC Symbol;Acc:VGNC:76484]	3.496496816	6.65E-23	3.26E-21
ENSMMUG00000022586	phosphoenolpyruvate carboxykinase 1 [Source:VGNC Symbol;Acc:VGNC:75779]	3.745766503	0.002801815	0.008943376
ENSMMUG00000022589	prostate transmembrane protein, androgen induced 1 [Source:VGNC Symbol;Acc:VGNC:76023]	-1.237514356	1.52E-06	9.98E-06
ENSMMUG00000013371	exportin 4 [Source:VGNC Symbol;Acc:VGNC:79091]	-0.312446689	0.012112858	0.031923527
ENSMMUG00000004560	crystallin lambda 1 [Source:VGNC Symbol;Acc:VGNC:71433]	-0.828258171	1.49E-05	8.15E-05
ENSMMUG00000052705	gap junction protein alpha 3 [Source:NCBI gene;Acc:704432]	-2.570758214	0.011551384	0.030656233
ENSMMUG00000020262	paraspeckle component 1 [Source:VGNC Symbol;Acc:VGNC:76293]	-0.628526313	0.013672227	0.035410256
ENSMMUG00000016475	M-phase phosphoprotein 8 [Source:VGNC Symbol;Acc:VGNC:74864]	-1.105291138	1.36E-10	1.69E-09
ENSMMUG00000007373	zinc finger DHHC-type palmitoyltransferase 20 [Source:VGNC Symbol;Acc:VGNC:79277]	-0.470422633	0.020265743	0.049414826
ENSMMUG00000004237	TNF receptor superfamily member 19 [Source:VGNC Symbol;Acc:VGNC:78588]	-1.308781617	2.51E-07	1.89E-06
ENSMMUG00000014610	ring finger protein 17 [Source:NCBI gene;Acc:708198]	-0.738931121	9.14E-06	5.17E-05
ENSMMUG00000008520	ATPase phospholipid transporting 8A2 [Source:VGNC Symbol;Acc:VGNC:70062]	0.766384794	3.39E-06	2.07E-05
ENSMMUG00000057852		-1.11228769	5.21E-07	3.71E-06
ENSMMUG00000005055	WASP family member 3 [Source:VGNC Symbol;Acc:VGNC:78782]	-1.499989431	0.019761641	0.048344629
ENSMMUG00000009030	mitochondrial translational initiation factor 3 [Source:VGNC Symbol;Acc:VGNC:75040]	-0.752231943	2.41E-05	0.0001257
ENSMMUG00000062551		-0.791318628	4.19E-05	0.000206384
ENSMMUG00000058786	ligand of numb-protein X 2 [Source:NCBI gene;Acc:710660]	-0.824659197	8.71E-05	0.000403074
ENSMMUG00000064615	pancreatic and duodenal homeobox 1 [Source:NCBI gene;Acc:710936]	-1.470561514	0.001396431	0.004845361
ENSMMUG00000052070	caudal type homeobox 2 [Source:NCBI gene;Acc:711094]	-1.289245413	2.23E-05	0.000117393
ENSMMUG00000016138	ureidoimidazoline (2-oxo-4-hydroxy-4-carboxy-5-) decarboxylase [Source:NCBI gene;Acc:721710]	-1.366414192	0.002270922	0.007416378
ENSMMUG00000021538	fms related receptor tyrosine kinase 1 [Source:VGNC Symbol;Acc:VGNC:72678]	0.444068167	0.002552597	0.008232931
ENSMMUG00000063649		0.509971062	0.017623521	0.043837103
ENSMMUG00000003771	ubiquitin like 3 [Source:VGNC Symbol;Acc:VGNC:78701]	-0.854115719	0.009123624	0.02500986
ENSMMUG00000005572	arachidonate 5-lipoxygenase activating protein [Source:VGNC Symbol;Acc:VGNC:69799]	-0.73516332	0.006260931	0.018043063
ENSMMUG00000013707	heat shock protein family H (Hsp110) member 1 [Source:NCBI gene;Acc:721956]	-1.283713452	7.30E-13	1.23E-11

ENSMMUG00000013713	beta 3-glucosyltransferase [Source:NCBI gene;Acc:713498]	3.399763905	9.02E-31	9.22E-29
ENSMMUG00000038997	zygote arrest 1 like [Source:NCBI gene;Acc:100427970]	2.452733105	0.000632756	0.002396263
ENSMMUG00000007200	NEDD4 binding protein 2 like 2 [Source:VGNC Symbol;Acc:VGNC:75031]	2.090104946	3.94E-24	2.16E-22
ENSMMUG00000055811		2.471449178	2.93E-20	1.06E-18
ENSMMUG00000007201	PDS5 cohesin associated factor B [Source:VGNC Symbol;Acc:VGNC:75835]	2.111298357	2.09E-28	1.79E-26
ENSMMUG00000025050	Y RNA [Source:RFAM;Acc:RF00019]	2.654591911	1.18E-07	9.45E-07
ENSMMUG00000051911		-1.128227516	2.03E-07	1.56E-06
ENSMMUG00000058110		1.418011667	0.000468221	0.001832936
ENSMMUG00000017052	StAR related lipid transfer domain containing 13 [Source:VGNC Symbol;Acc:VGNC:77962]	-0.4839231	4.44E-07	3.20E-06
ENSMMUG00000055864		-1.761441145	0.001476136	0.005083859
ENSMMUG00000054254		-2.14524348	0.007791721	0.021818585
ENSMMUG00000019831	neurobeachin [Source:NCBI gene;Acc:714386]	-2.362731815	9.39E-11	1.19E-09
ENSMMUG00000016791	doublecortin like kinase 1 [Source:VGNC Symbol;Acc:VGNC:71745]	-2.35894492	7.88E-12	1.14E-10
ENSMMUG00000055861	Metazoan signal recognition particle RNA [Source:RFAM;Acc:RF00017]	-3.09934654	0.018022598	0.044709879
ENSMMUG00000060579		-2.216386604	0.000164134	0.000712142
ENSMMUG00000001172	spermatogenesis and oogenesis specific basic helix-loop-helix 2 [Source:NCBI gene;Acc:693608]	-2.161251516	3.13E-05	0.000158967
ENSMMUG00000003585	ALG5 dolichyl-phosphate beta-glucosyltransferase [Source:VGNC Symbol;Acc:VGNC:69647]	0.966261523	4.32E-06	2.60E-05
ENSMMUG00000003590	SPT20 homolog, SAGA complex component [Source:VGNC Symbol;Acc:VGNC:78182]	0.785674072	0.019523923	0.047841999
ENSMMUG00000012307	periostin [Source:VGNC Symbol;Acc:VGNC:76039]	-1.255174957	3.08E-14	6.03E-13
ENSMMUG00000030786	transient receptor potential cation channel subfamily C member 4 [Source:NCBI gene;Acc:695636]	-0.416222695	0.001537331	0.005267704
ENSMMUG00000002113	NHL repeat containing 3 [Source:VGNC Symbol;Acc:VGNC:75291]	-1.454613052	3.49E-10	4.07E-09
ENSMMUG00000002109	proline and serine rich 1 [Source:VGNC Symbol;Acc:VGNC:76291]	-1.096086349	5.65E-12	8.41E-11
ENSMMUG00000055317		-1.066451872	0.000581241	0.002220456
ENSMMUG00000052676		-1.604622308	1.42E-23	7.61E-22
ENSMMUG00000050501		-1.410447785	0.005106385	0.015109275
ENSMMUG00000001899	mitochondrial ribosomal protein S31 [Source:VGNC Symbol;Acc:VGNC:74777]	1.352737719	0.012046478	0.03178247
ENSMMUG00000007880	E74 like ETS transcription factor 1 [Source:VGNC Symbol;Acc:VGNC:72205]	0.900376247	0.020434261	0.049727615
ENSMMUG00000011590	N-alpha-acetyltransferase 16, NatA auxiliary subunit [Source:NCBI gene;Acc:698843]	-0.441304236	0.000876386	0.003189401
ENSMMUG00000011593	regulator of cell cycle [Source:VGNC Symbol;Acc:VGNC:76783]	-0.738690477	0.002117822	0.006988717
ENSMMUG00000011594	von Willebrand factor A domain containing 8 [Source:VGNC Symbol;Acc:VGNC:79773]	0.462742594	3.01E-07	2.22E-06
ENSMMUG00000055161		-2.5582281	2.04E-05	0.000108355
ENSMMUG00000023076	diacylglycerol kinase eta [Source:VGNC Symbol;Acc:VGNC:71786]	-1.942575113	1.10E-10	1.39E-09
ENSMMUG00000055887		-1.146800995	0.001057858	0.003783895
ENSMMUG00000023083	A-kinase anchoring protein 11 [Source:VGNC Symbol;Acc:VGNC:69765]	-1.935031256	7.05E-11	9.11E-10
ENSMMUG00000054971		-1.878640597	0.003704579	0.011409948
ENSMMUG00000017876	TNF superfamily member 11 [Source:NCBI gene;Acc:699955]	-1.763523446	0.018939314	0.046629699

ENSMMUG00000014926	epithelial stromal interaction 1 [Source:VGNC Symbol;Acc:VGNC:72053]	-0.787148659	0.000493639	0.001922445
ENSMMUG00000050436		3.089389153	0.013732508	0.035543734
ENSMMUG00000012636	potassium channel tetramerization domain containing 4 [Source:VGNC Symbol;Acc:VGNC:73892]	-1.456115379	8.35E-06	4.76E-05
ENSMMUG00000011986	chibby family member 2 [Source:VGNC Symbol;Acc:VGNC:78029]	-0.958734881	0.001332319	0.004651198
ENSMMUG00000017025	zinc finger CCCH-type containing 13 [Source:VGNC Symbol;Acc:VGNC:79248]	-0.718731269	2.62E-06	1.65E-05
ENSMMUG00000017033	lymphocyte cytosolic protein 1 [Source:VGNC Symbol;Acc:VGNC:74242]	-0.520189701	0.00055583	0.002134928
ENSMMUG00000016293	leucine rich repeats and calponin homology domain containing 1 [Source:VGNC Symbol;Acc:VGNC:74317]	0.398582935	9.85E-06	5.54E-05
ENSMMUG00000065094		1.256688204	0.003561591	0.011031409
ENSMMUG00000059778		2.639453736	2.54E-11	3.44E-10
ENSMMUG00000063377	cysteinyl leukotriene receptor 2 [Source:NCBI gene;Acc:705450]	-1.592082293	1.86E-05	9.97E-05
ENSMMUG00000010898	tripartite motif containing 13 [Source:VGNC Symbol;Acc:VGNC:79972]	0.660240322	2.70E-07	2.02E-06
ENSMMUG00000056958	mml-mir-7194 [Source:miRBase;Acc:MI0023684]	0.810877118	0.00028007	0.001153092
ENSMMUG00000010899	potassium channel regulator [Source:VGNC Symbol;Acc:VGNC:73859]	1.346741752	0.020338797	0.049552219
ENSMMUG00000062619	transmembrane protein 272 [Source:NCBI gene;Acc:713644]	-0.665899229	0.011852703	0.031338133
ENSMMUG00000053191		0.793256781	2.91E-06	1.81E-05
ENSMMUG00000018720	cytoskeleton associated protein 2 [Source:VGNC Symbol;Acc:VGNC:71229]	1.58141642	3.23E-28	2.72E-26
ENSMMUG00000007379	phosphatidylinositol 3,4,5-trisphosphate 3-phosphatase TPTE2-like [Source:NCBI gene;Acc:694255]	1.467480212	1.16E-20	4.45E-19
ENSMMUG00000053708	U2 spliceosomal RNA [Source:RFAM;Acc:RF00004]	0.988560091	0.009604942	0.026126428
ENSMMUG00000016401	SGT1 homolog, MIS12 kinetochore complex assembly cochaperone [Source:NCBI gene;Acc:694046]	1.526619321	1.37E-25	8.58E-24
ENSMMUG00000016404	chondromodulin [Source:VGNC Symbol;Acc:VGNC:71191]	3.236726471	2.29E-09	2.37E-08
ENSMMUG00000002657	olfactomedin 4 [Source:VGNC Symbol;Acc:VGNC:75418]	-1.839614278	1.43E-08	1.31E-07
ENSMMUG00000053482	protocadherin 20 [Source:NCBI gene;Acc:696607]	-0.723734918	0.002873347	0.009152006
ENSMMUG00000055156		1.016451142	0.015705199	0.039769793
ENSMMUG00000002533	progesterone immunomodulatory binding factor 1 [Source:NCBI gene;Acc:694190]	-1.222210575	2.85E-06	1.78E-05
ENSMMUG00000064642		-1.165337489	3.96E-06	2.40E-05
ENSMMUG00000059178		-0.991103465	0.00013572	0.000600006
ENSMMUG00000054675		-1.300971098	0.000281342	0.001157042
ENSMMUG00000065060		-1.241256435	0.000641242	0.002425306
ENSMMUG00000046375		0.924730848	0.00401547	0.0122757
ENSMMUG00000061846		-0.728670092	7.59E-05	0.000354838
ENSMMUG00000059231		-0.54949182	9.69E-07	6.58E-06
ENSMMUG00000056466		-0.799802394	3.72E-06	2.26E-05
ENSMMUG00000062938		-3.760315965	0.004824945	0.014414573
ENSMMUG00000054625		-0.855914291	4.14E-07	2.99E-06
ENSMMUG00000062636	Metazoan signal recognition particle RNA [Source:RFAM;Acc:RF00017]	-2.48040762	0.001938024	0.006476092
ENSMMUG00000009018	60S ribosomal protein L13a-like [Source:NCBI gene;Acc:698713]	-1.505018924	0.00299505	0.009482621

ENSMMUG00000054008		-3.381900708	0.007092188	0.020098824
ENSMMUG00000060359		-2.489895143	0.005189571	0.015312642
ENSMMUG00000021817	dopachrome tautomerase [Source:VGNC Symbol;Acc:VGNC:71749]	-0.675955351	0.013088945	0.034072153
ENSMMUG00000054711		-1.405459504	2.39E-07	1.80E-06
ENSMMUG00000009721	claudin 10 [Source:VGNC Symbol;Acc:VGNC:71243]	-1.291183717	0.000381164	0.001525306
ENSMMUG00000022744	muscleblind like splicing regulator 2 [Source:VGNC Symbol;Acc:VGNC:74519]	0.702638406	1.69E-05	9.13E-05
ENSMMUG00000051235		0.898546607	0.002300519	0.007506423
ENSMMUG00000009890	FERM, ARH/RhoGEF and pleckstrin domain protein 1 [Source:VGNC Symbol;Acc:VGNC:72614]	1.037699771	3.43E-19	1.08E-17
ENSMMUG00000009173	serine/threonine kinase 24 [Source:VGNC Symbol;Acc:VGNC:81583]	0.710739856	0.00058194	0.002222553
ENSMMUG00000001402	solute carrier family 15 member 1 [Source:VGNC Symbol;Acc:VGNC:77393]	-1.773742376	0.005902442	0.017136495
ENSMMUG00000018657	dedicator of cytokinesis 9 [Source:VGNC Symbol;Acc:VGNC:71835]	-0.888269153	0.008152189	0.022712186
ENSMMUG00000012332	UBA domain containing 2 [Source:NCBI gene;Acc:703484]	-1.336144133	8.99E-22	3.95E-20
ENSMMUG00000060781	G protein-coupled receptor 18 [Source:NCBI gene;Acc:698714]	-1.304911018	8.53E-07	5.86E-06
ENSMMUG00000012338	G protein-coupled receptor 183 [Source:VGNC Symbol;Acc:VGNC:73042]	-1.228829495	6.00E-12	8.87E-11
ENSMMUG00000019556	transmembrane 9 superfamily member 2 [Source:VGNC Symbol;Acc:VGNC:78568]	0.714819897	2.35E-15	5.15E-14
ENSMMUG00000003558	citrate lyase beta like [Source:VGNC Symbol;Acc:VGNC:71202]	-1.739412069	0.010383037	0.027946798
ENSMMUG00000004493	propionyl-CoA carboxylase subunit alpha [Source:VGNC Symbol;Acc:VGNC:75773]	-1.39643491	1.03E-07	8.33E-07
ENSMMUG00000050578		-1.313379915	0.000597566	0.002274033
ENSMMUG00000061728	Metazoan signal recognition particle RNA [Source:RFAM;Acc:RF00017]	-2.594370976	0.019228259	0.047203292
ENSMMUG00000002875	fibroblast growth factor 14 [Source:VGNC Symbol;Acc:VGNC:72645]	-3.083620482	0.017200631	0.042943662
ENSMMUG00000052642	protein O-glucosyltransferase 2 [Source:NCBI gene;Acc:702156]	-2.114638157	8.86E-06	5.03E-05
ENSMMUG00000000350	basic, immunoglobulin-like variable motif containing [Source:NCBI gene;Acc:100426213]	-1.19971456	1.46E-06	9.64E-06
ENSMMUG00000000353	putative methyltransferase-like protein 21E pseudogene [Source:NCBI gene;Acc:707949]	-1.076609916	1.93E-05	0.000103103
ENSMMUG00000053838		-0.338575428	0.014201451	0.036500436
ENSMMUG00000054803		1.017803249	0.003071427	0.00968515
ENSMMUG00000022282	collagen type IV alpha 2 chain [Source:NCBI gene;Acc:100429807]	4.473476253	8.00E-08	6.53E-07
ENSMMUG00000022818	inhibitor of growth family member 1 [Source:VGNC Symbol;Acc:VGNC:73742]	3.729647666	3.32E-05	0.000167812
ENSMMUG00000064401		2.902010492	0.000366149	0.001470746
ENSMMUG00000018929	ankyrin repeat domain 10 [Source:VGNC Symbol;Acc:VGNC:69646]	-0.743530879	1.99E-07	1.53E-06
ENSMMUG00000013138	Rho guanine nucleotide exchange factor 7 [Source:VGNC Symbol;Acc:VGNC:69833]	-1.147391522	1.40E-08	1.29E-07
ENSMMUG00000056306		-1.547221067	5.71E-14	1.10E-12
ENSMMUG00000013625	ATPase phospholipid transporting 11A [Source:VGNC Symbol;Acc:VGNC:70064]	-0.656104645	4.33E-05	0.000212858
ENSMMUG00000048566	MCF.2 cell line derived transforming sequence like [Source:NCBI gene;Acc:713961]	2.30508896	3.38E-36	6.42E-34
ENSMMUG00000001036	coagulation factor VII [Source:VGNC Symbol;Acc:VGNC:72466]	2.168721343	2.46E-27	1.95E-25

ENSMMUG00000058677		-2.057788684	0.000685661	0.002572313
ENSMMUG00000016740	defective in cullin neddylation 1 domain containing 2 [Source:VGNC Symbol;Acc:VGNC:71684]	-0.620568357	3.73E-12	5.70E-11
ENSMMUG00000064494	Metazoan signal recognition particle RNA [Source:RFAM;Acc:RF00017]	-1.965181203	0.008446531	0.023417845
ENSMMUG00000007423	transcription factor Dp-1 [Source:NCBI gene;Acc:715345]	1.579269178	4.25E-11	5.65E-10
ENSMMUG00000051400		-0.446084709	0.001138307	0.004047252
ENSMMUG00000050639	transmembrane protein 255B [Source:NCBI gene;Acc:715939]	-0.427400783	0.02042962	0.049727615
ENSMMUG00000054800	chromosome 17 C13orf46 homolog [Source:NCBI gene;Acc:106994405]	0.389379692	0.011835918	0.031299335
ENSMMUG00000023599	chromosome alignment maintaining phosphoprotein 1 [Source:VGNC Symbol;Acc:VGNC:71061]	0.716114916	3.00E-06	1.86E-05
ENSMMUG00000038897	rabphilin 3A like (without C2 domains) [Source:NCBI gene;Acc:106993852]	2.197140309	0.002531089	0.008172449
ENSMMUG00000003042	VPS53 subunit of GARP complex [Source:VGNC Symbol;Acc:VGNC:79522]	1.069172256	0.000627831	0.00237883
ENSMMUG00000055344	TLC domain containing 3A [Source:NCBI gene;Acc:699707]	-1.502312006	6.82E-08	5.64E-07
ENSMMUG00000030401	gem nuclear organelle associated protein 4 [Source:VGNC Symbol;Acc:VGNC:72723]	-0.978654586	6.81E-05	0.000321272
ENSMMUG00000050361		-0.672466148	0.014504181	0.037188279
ENSMMUG00000011417	glyoxalase domain containing 4 [Source:VGNC Symbol;Acc:VGNC:72939]	-0.705252849	0.000242431	0.001015035
ENSMMUG00000008130	ABR activator of RhoGEF and GTPase [Source:VGNC Symbol;Acc:VGNC:69589]	0.729793675	3.26E-08	2.82E-07
ENSMMUG00000002371	myosin IC [Source:VGNC Symbol;Acc:VGNC:75112]	0.87384153	8.02E-07	5.54E-06
ENSMMUG00000055241		0.537954486	0.000366057	0.001470746
ENSMMUG00000040268	phosphatidylinositol transfer protein alpha [Source:NCBI gene;Acc:721228]	1.037433246	9.71E-06	5.46E-05
ENSMMUG00000017561	solute carrier family 43 member 2 [Source:VGNC Symbol;Acc:VGNC:77554]	1.889802646	1.44E-16	3.56E-15
ENSMMUG00000039266	Metazoan signal recognition particle RNA [Source:RFAM;Acc:RF00017]	2.022126802	2.38E-07	1.80E-06
ENSMMUG00000055528		1.593540767	0.001083276	0.003868286
ENSMMUG00000002376	scavenger receptor class F member 1 [Source:VGNC Symbol;Acc:VGNC:77027]	2.197144579	1.74E-15	3.87E-14
ENSMMUG00000002377	Rab interacting lysosomal protein [Source:VGNC Symbol;Acc:VGNC:76866]	2.211240079	2.49E-09	2.56E-08
ENSMMUG00000004820	pre-mRNA processing factor 8 [Source:VGNC Symbol;Acc:VGNC:76275]	1.338298736	9.24E-11	1.17E-09
ENSMMUG00000002382	serpin family F member 2 [Source:VGNC Symbol;Acc:VGNC:77372]	1.711546326	5.98E-21	2.36E-19
ENSMMUG00000048710	HIC ZBTB transcriptional repressor 1 [Source:NCBI gene;Acc:721284]	-1.393005049	0.004959124	0.014758067
ENSMMUG00000053298		-0.714806954	4.73E-07	3.39E-06
ENSMMUG00000020524	platelet activating factor acetylhydrolase 1b regulatory subunit 1 [Source:VGNC Symbol;Acc:VGNC:75747]	0.464856446	0.008462705	0.023445154
ENSMMUG00000062095		0.560149414	0.003028378	0.009563638
ENSMMUG00000022989	RAP1 GTPase activating protein 2 [Source:VGNC Symbol;Acc:VGNC:76561]	0.891227065	0.00027814	0.0011461
ENSMMUG00000061370		2.167356968	0.000467903	0.001832807
ENSMMUG00000030389		1.463323723	0.000443917	0.001745767
ENSMMUG00000062583	olfactory receptor 1A1 [Source:NCBI gene;Acc:721403]	1.237390774	2.45E-09	2.53E-08
ENSMMUG00000042407		1.18738086	0.003401722	0.010595995
ENSMMUG00000055048		1.270438342	1.70E-13	3.06E-12
ENSMMUG00000008556	integrin subunit alpha E [Source:NCBI gene;Acc:721483]	0.433700911	0.000426418	0.001686792
ENSMMUG00000063482		-0.785121504	0.018510163	0.045712858
ENSMMUG00000057632		3.498498779	4.82E-21	1.92E-19
ENSMMUG00000019184	ankyrin repeat and FYVE domain containing 1 [Source:VGNC Symbol;Acc:VGNC:69651]	0.354335212	0.004128447	0.012584761
ENSMMUG00000058966		-1.131379438	7.50E-06	4.32E-05
ENSMMUG00000005080	ubiquitin conjugating enzyme E2 G1 [Source:VGNC Symbol;Acc:VGNC:78695]	0.797020789	2.99E-11	4.04E-10

ENSMMUG00000060937	Metazoan signal recognition particle RNA [Source:RFAM;Acc:RF00017]	1.75058748	0.001102531	0.003932307
ENSMMUG00000030381	arachidonate 15-lipoxygenase [Source:VGNC Symbol;Acc:VGNC:69796]	-1.654473001	2.29E-11	3.13E-10
ENSMMUG00000017654	arrestin beta 2 [Source:VGNC Symbol;Acc:VGNC:70022]	1.406848648	0.00029142	0.001192867
ENSMMUG00000049649	proteasome 20S subunit beta 6 [Source:NCBI gene;Acc:710028]	1.638626894	2.67E-17	7.18E-16
ENSMMUG00000061951		1.834403866	2.00E-06	1.28E-05
ENSMMUG00000005666	phospholipase D2 [Source:VGNC Symbol;Acc:VGNC:76172]	1.33806724	2.46E-11	3.34E-10
ENSMMUG00000009176	calmodulin binding transcription activator 2 [Source:VGNC Symbol;Acc:VGNC:70578]	1.027936972	3.32E-06	2.04E-05
ENSMMUG00000011344	solute carrier family 52 member 1 [Source:VGNC Symbol;Acc:VGNC:77795]	2.592901544	0.000398015	0.001586179
ENSMMUG00000018648	zinc finger protein 594 [Source:NCBI gene;Acc:711492]	0.796573654	0.002177229	0.007148175
ENSMMUG00000058586		-2.143043573	6.08E-06	3.56E-05
ENSMMUG00000006594	DEAH-box helicase 33 [Source:VGNC Symbol;Acc:VGNC:71633]	-0.317413671	0.016684352	0.041930001
ENSMMUG00000046195	MIS12 kinetochore complex component [Source:NCBI gene;Acc:712202]	-0.83409918	0.000694767	0.002600556
ENSMMUG00000030453	NLR family pyrin domain containing 1 [Source:NCBI gene;Acc:721648]	-0.504511429	2.31E-05	0.000120695
ENSMMUG00000010536	discs large MAGUK scaffold protein 4 [Source:VGNC Symbol;Acc:VGNC:71920]	1.274556465	1.88E-14	3.79E-13
ENSMMUG00000052091	dishevelled segment polarity protein 2 [Source:NCBI gene;Acc:714381]	-1.224597728	0.000826158	0.003031521
ENSMMUG00000010543	PHD finger protein 23 [Source:VGNC Symbol;Acc:VGNC:75877]	-1.911836208	0.00092608	0.003352151
ENSMMUG00000010547	CTD nuclear envelope phosphatase 1 [Source:VGNC Symbol;Acc:VGNC:71440]	2.248088172	0.017577616	0.043737592
ENSMMUG00000064650	SPEM family member 3 [Source:NCBI gene;Acc:106993878]	1.4588991	1.60E-26	1.15E-24
ENSMMUG00000009709	transmembrane protein 102 [Source:NCBI gene;Acc:715461]	-0.590294508	0.009471829	0.025844936
ENSMMUG00000017690	cholinergic receptor nicotinic beta 1 subunit [Source:VGNC Symbol;Acc:VGNC:71049]	0.464014631	0.001007436	0.003623643
ENSMMUG00000006327	tumor necrosis factor (ligand) superfamily, member 12 [Source:NCBI gene;Acc:100551506]	-0.291434265	0.018365259	0.045430657
ENSMMUG00000006332	SUMO specific peptidase 3 [Source:NCBI gene;Acc:715884]	0.459059803	0.016797391	0.042156931
ENSMMUG00000013501	CD68 molecule [Source:VGNC Symbol;Acc:VGNC:82121]	-2.297543724	2.60E-07	1.95E-06
ENSMMUG00000013503	mannose-P-dolichol utilization defect 1 [Source:VGNC Symbol;Acc:VGNC:74913]	-1.50307363	4.88E-05	0.000237655
ENSMMUG00000013510	FMR1 autosomal homolog 2 [Source:NCBI gene;Acc:721889]	-0.705605284	0.001362621	0.004744698
ENSMMUG00000013513	sex hormone binding globulin [Source:VGNC Symbol;Acc:VGNC:77208]	-1.178887005	5.46E-06	3.23E-05
ENSMMUG00000008639	tumor protein p53 [Source:VGNC Symbol;Acc:VGNC:78627]	-1.506315955	4.36E-09	4.32E-08
ENSMMUG00000054769	Metazoan signal recognition particle RNA [Source:RFAM;Acc:RF00017]	-1.79398181	0.004784749	0.014311784
ENSMMUG00000008637	WD repeat containing antisense to TP53 [Source:VGNC Symbol;Acc:VGNC:79508]	1.012681483	4.76E-05	0.000232883
ENSMMUG00000048204	ephrin B3 [Source:NCBI gene;Acc:716257]	1.425671936	1.20E-15	2.70E-14
ENSMMUG00000008166	dynein axonemal heavy chain 2 [Source:VGNC Symbol;Acc:VGNC:71940]	0.485306415	0.000123923	0.000552716
ENSMMUG00000008173	lysine demethylase 6B [Source:VGNC Symbol;Acc:VGNC:73923]	-1.475047099	0.019029035	0.04678225
ENSMMUG00000063668	cytochrome b5 domain containing 1 [Source:NCBI gene;Acc:716429]	-1.113740034	7.45E-05	0.00034886
ENSMMUG00000009995	arachidonate 15-lipoxygenase type B [Source:VGNC Symbol;Acc:VGNC:69797]	1.894893699	2.38E-21	9.79E-20
ENSMMUG00000065208		1.647904008	4.32E-08	3.65E-07
ENSMMUG00000020329	arachidonate 12-lipoxygenase, 12R type [Source:NCBI gene;Acc:721936]	-1.29609024	0.000596696	0.002271305
ENSMMUG00000019850	arachidonate lipoxygenase 3 [Source:VGNC Symbol;Acc:VGNC:69607]	-1.851044132	2.22E-40	6.34E-38
ENSMMUG00000021798	period circadian regulator 1 [Source:VGNC Symbol;Acc:VGNC:75942]	-0.676051652	0.001992349	0.006635198

ENSMMUG00000021938	nudE neurodevelopment protein 1 like 1 [Source:VGNC Symbol;Acc:VGNC:74982]	1.013787827	3.26E-07	2.40E-06
ENSMMUG00000055290	netrin 1 [Source:NCBI gene;Acc:717645]	1.01817913	7.77E-07	5.38E-06
ENSMMUG00000054855	Metazoan signal recognition particle RNA [Source:RFAM;Acc:RF00017]	0.969927998	0.005498008	0.01610736
ENSMMUG00000009771	myosin heavy chain 4 [Source:VGNC Symbol;Acc:VGNC:75100]	0.639057065	0.012624594	0.033072378
ENSMMUG00000051001		-1.956960934	0.00956337	0.026022895
ENSMMUG00000055729		-0.876965294	2.53E-05	0.000131699
ENSMMUG00000060547	Metazoan signal recognition particle RNA [Source:RFAM;Acc:RF00017]	-1.035505638	1.18E-05	6.55E-05
ENSMMUG00000060042		-0.889630898	0.006138916	0.017739698
ENSMMUG00000016723	zinc finger protein 287 [Source:NCBI gene;Acc:695524]	-1.842946162	4.54E-05	0.000222568
ENSMMUG00000002523	nuclear receptor corepressor 1 [Source:VGNC Symbol;Acc:VGNC:81479]	-0.419580179	0.012941352	0.033768525
ENSMMUG00000059034		0.302201191	0.002714496	0.008698353
ENSMMUG00000050263	TBC1 domain family member 26-like [Source:NCBI gene;Acc:100426631]	0.973804777	0.000842419	0.003082425
ENSMMUG00000013345	folliculin [Source:NCBI gene;Acc:698082]	-1.620378078	0.003560281	0.011029657
ENSMMUG00000021091	COP9 signalosome subunit 3 [Source:VGNC Symbol;Acc:VGNC:71316]	-1.76903321	0.00018372	0.000789043
ENSMMUG00000038569	5',3'-nucleotidase, mitochondrial [Source:NCBI gene;Acc:698591]	-0.831946136	0.000155699	0.000677727
ENSMMUG00000006491	mediator complex subunit 9 [Source:VGNC Symbol;Acc:VGNC:74577]	-0.904801924	0.000131294	0.000583309
ENSMMUG00000006493	ras related dexamethasone induced 1 [Source:VGNC Symbol;Acc:VGNC:76485]	-0.920783978	2.26E-09	2.35E-08
ENSMMUG00000023375	sterol regulatory element binding transcription factor 1 [Source:VGNC Symbol;Acc:VGNC:77880]	-0.533328443	0.018022633	0.044709879
ENSMMUG00000003349	target of myb1 like 2 membrane trafficking protein [Source:VGNC Symbol;Acc:VGNC:78617]	-1.230240318	0.014862799	0.037963366
ENSMMUG00000002942	dynein regulatory complex subunit 3 [Source:VGNC Symbol;Acc:VGNC:71906]	-1.214954943	0.004177582	0.012713634
ENSMMUG00000005900	GID complex subunit 4 homolog [Source:NCBI gene;Acc:699842]	-0.910920604	0.004490166	0.013539885
ENSMMUG00000021409	developmentally regulated GTP binding protein 2 [Source:VGNC Symbol;Acc:VGNC:71993]	-1.140701616	0.000224631	0.000947193
ENSMMUG00000018344	myosin XVA [Source:VGNC Symbol;Acc:VGNC:75109]	0.793165641	3.33E-05	0.000168126
ENSMMUG00000056257		-1.156949726	9.04E-07	6.18E-06
ENSMMUG00000053675		-1.080177408	6.83E-06	3.96E-05
ENSMMUG00000005323	phosphoribosyl pyrophosphate synthetase associated protein 2 [Source:VGNC Symbol;Acc:VGNC:76430]	0.512165929	5.24E-06	3.10E-05
ENSMMUG00000000140	epsin 2 [Source:VGNC Symbol;Acc:VGNC:72070]	-0.633017897	0.014907337	0.038064016
ENSMMUG00000008536	mitogen-activated protein kinase 7 [Source:VGNC Symbol;Acc:VGNC:74648]	1.489039656	0.000292729	0.001197566
ENSMMUG00000008538	microfibril associated protein 4 [Source:VGNC Symbol;Acc:VGNC:74710]	1.802598111	0.000278681	0.001148013
ENSMMUG00000008540	ring finger protein 112 [Source:VGNC Symbol;Acc:VGNC:76724]	-2.681609625	3.39E-17	8.97E-16
ENSMMUG00000051286		-1.836243591	1.62E-14	3.30E-13
ENSMMUG00000030279	A-kinase anchoring protein 10 [Source:VGNC Symbol;Acc:VGNC:69764]	1.16375438	0.001731741	0.005858056
ENSMMUG00000018579	sperm antigen with calponin homology and coiled-coil domains 1 [Source:VGNC Symbol;Acc:VGNC:78021]	0.55579288	5.21E-05	0.000251942
ENSMMUG00000024170	U6 spliceosomal RNA [Source:RFAM;Acc:RF00026]	0.720672954	0.001507787	0.005183235
ENSMMUG00000050178		0.540515764	5.84E-05	0.00027942

ENSMMUG00000052714		-1.509022311	0.01478735	0.037809724
ENSMMUG00000060502		1.396711933	1.11E-07	8.87E-07
ENSMMUG00000061335		1.792863514	1.13E-10	1.42E-09
ENSMMUG00000054233		0.426489054	1.69E-05	9.12E-05
ENSMMUG00000020226	WD repeat and SOCS box containing 1 [Source:VGNC Symbol;Acc:VGNC:79186]	1.737473127	1.67E-17	4.57E-16
ENSMMUG00000054784		1.45903865	1.44E-09	1.55E-08
ENSMMUG00000061836		1.121674024	3.76E-05	0.000187134
ENSMMUG00000065113		1.024635141	6.28E-05	0.000299005
ENSMMUG00000061698		0.860388978	7.38E-09	7.07E-08
ENSMMUG00000051341		-0.721738762	0.003785486	0.011630154
ENSMMUG00000018719	nemo like kinase [Source:VGNC Symbol;Acc:VGNC:75277]	1.744821	1.82E-17	4.97E-16
ENSMMUG00000063312		-1.498743448	7.41E-09	7.10E-08
ENSMMUG00000054461	transmembrane protein 97 [Source:NCBI gene;Acc:707879]	-1.756948429	0.004581231	0.013792053
ENSMMUG00000019145	TNF alpha induced protein 1 [Source:VGNC Symbol;Acc:VGNC:78578]	-1.60688776	1.45E-11	2.03E-10
ENSMMUG00000019146	DNA polymerase delta interacting protein 2 [Source:VGNC Symbol;Acc:VGNC:76099]	-1.555226972	5.58E-08	4.67E-07
ENSMMUG00000021351	transmembrane protein 199 [Source:VGNC Symbol;Acc:VGNC:79113]	-1.965155082	0.000112611	0.000507299
ENSMMUG00000019148	vitronectin [Source:NCBI gene;Acc:708499]	-1.65687825	8.15E-10	9.05E-09
ENSMMUG00000012240	phosphatidylinositol glycan anchor biosynthesis class S [Source:VGNC Symbol;Acc:VGNC:75800]	-0.743698113	0.000293176	0.001199063
ENSMMUG00000006018	sperm associated antigen 5 [Source:NCBI gene;Acc:709400]	-0.945296436	5.12E-05	0.000248351
ENSMMUG00000004708	SPT6 homolog, histone chaperone and transcription elongation factor [Source:VGNC Symbol;Acc:VGNC:78057]	-0.555608948	0.001793945	0.006053488
ENSMMUG00000004710	protein interacting with cyclin A1 [Source:VGNC Symbol;Acc:VGNC:76336]	-1.201680387	0.000455933	0.001789796
ENSMMUG00000007293	RAB34, member RAS oncogene family [Source:VGNC Symbol;Acc:VGNC:81533]	-1.348912768	4.30E-10	4.97E-09
ENSMMUG00000051743	TLC domain containing 1 [Source:NCBI gene;Acc:710391]	-1.183499756	5.68E-07	4.03E-06
ENSMMUG00000021408	family with sequence similarity 222 member B [Source:VGNC Symbol;Acc:VGNC:72352]	1.488303584	8.46E-09	8.01E-08
ENSMMUG00000012523	flotillin 2 [Source:NCBI gene;Acc:114669752]	1.754903502	3.05E-16	7.26E-15
ENSMMUG00000021553	dehydrogenase/reductase 13 [Source:VGNC Symbol;Acc:VGNC:81387]	1.778683113	2.47E-15	5.41E-14
ENSMMUG00000021554	PHD finger protein 12 [Source:VGNC Symbol;Acc:VGNC:75838]	1.791516437	4.15E-17	1.09E-15
ENSMMUG00000009938	myosin XVIIIA [Source:VGNC Symbol;Acc:VGNC:75058]	0.753448358	5.09E-09	4.98E-08
ENSMMUG00000012856	nuclear FMR1 interacting protein 2 [Source:VGNC Symbol;Acc:VGNC:75422]	2.265152367	3.75E-05	0.000186995
ENSMMUG00000019667	TAO kinase 1 [Source:VGNC Symbol;Acc:VGNC:81591]	1.77405323	7.60E-09	7.28E-08
ENSMMUG00000057447		3.90194158	0.000281885	0.001158636
ENSMMUG00000003866	tumor protein p53 inducible protein 13 [Source:VGNC Symbol;Acc:VGNC:79474]	1.511045072	0.011053309	0.029488045
ENSMMUG00000014408	GIT ArfGAP 1 [Source:VGNC Symbol;Acc:VGNC:73053]	1.695703422	2.37E-05	0.000123673
ENSMMUG00000014409	ankyrin repeat domain 13B [Source:VGNC Symbol;Acc:VGNC:69708]	1.153989216	5.33E-06	3.15E-05
ENSMMUG00000014411	coronin 6 [Source:NCBI gene;Acc:711818]	1.316252411	3.32E-10	3.89E-09
ENSMMUG00000014413	slingshot protein phosphatase 2 [Source:VGNC Symbol;Acc:VGNC:78018]	0.980388911	8.76E-14	1.64E-12
ENSMMUG00000056200	transmembrane and immunoglobulin domain containing 1 [Source:NCBI gene;Acc:712350]	-0.740996438	0.007353993	0.020710162
ENSMMUG00000023698	golgi SNAP receptor complex member 1 [Source:VGNC Symbol;Acc:VGNC:73110]	-0.669331233	0.000627014	0.002376946
ENSMMUG00000059292		1.472213204	7.61E-25	4.52E-23
ENSMMUG00000011411	sperm acrosome associated 3 [Source:VGNC Symbol;Acc:VGNC:77930]	2.311634847	2.83E-15	6.15E-14

ENSMMUG00000011413	acid sensing ion channel subunit 2 [Source:VGNC Symbol;Acc:VGNC:70241]	0.865785354	0.015659597	0.039663847
ENSMMUG00000002324		1.339499947	3.89E-13	6.74E-12
ENSMMUG00000000288	C-C motif chemokine ligand 2 [Source:VGNC Symbol;Acc:VGNC:70698]	-1.372326291	1.16E-05	6.47E-05
ENSMMUG00000009782	C-C motif chemokine ligand 7 [Source:NCBI gene;Acc:714751]	-0.843932016	0.012755744	0.033362906
ENSMMUG00000006399	chaperonin containing TCP1 subunit 6B [Source:VGNC Symbol;Acc:VGNC:70748]	0.493711525	2.52E-09	2.60E-08
ENSMMUG00000004794	DNA ligase 3 [Source:VGNC Symbol;Acc:VGNC:74266]	2.929055347	9.35E-13	1.55E-11
ENSMMUG00000005170	unc-45 myosin chaperone B [Source:VGNC Symbol;Acc:VGNC:78954]	-1.288960161	1.52E-06	9.99E-06
ENSMMUG00000037555	schlafen family member 11 [Source:NCBI gene;Acc:715511]	1.762141867	0.001733943	0.005864168
ENSMMUG00000031736		-1.755331406	0.014009853	0.036114395
ENSMMUG00000026432	small nucleolar RNA, C/D box 7 [Source:HGNC Symbol;Acc:HGNC:32704]	-2.415399363	0.007615272	0.02139316
ENSMMUG00000006048	adaptor related protein complex 2 subunit beta 1 [Source:VGNC Symbol;Acc:VGNC:69964]	-0.845571862	4.14E-06	2.50E-05
ENSMMUG00000013142	growth arrest specific 2 like 2 [Source:VGNC Symbol;Acc:VGNC:72776]	-1.002711568	0.006445264	0.018505957
ENSMMUG00000022229	matrix metallopeptidase 28 [Source:VGNC Symbol;Acc:VGNC:74755]	-1.080091658	0.01017939	0.027477313
ENSMMUG00000022233	HEAT repeat containing 9 [Source:VGNC Symbol;Acc:VGNC:73243]	-1.234680785	0.001997177	0.006649784
ENSMMUG00000054829		-0.449126418	0.017568755	0.04372288
ENSMMUG00000050758		-1.529832617	0.002358377	0.007681688
ENSMMUG00000054472		1.023759539	0.000123677	0.000551952
ENSMMUG00000054210	C-C motif chemokine 4 [Source:NCBI gene;Acc:100426632]	-1.691826548	2.44E-17	6.59E-16
ENSMMUG00000058233	C-C motif chemokine 3-like [Source:NCBI gene;Acc:100426537]	-2.604022769	4.89E-06	2.92E-05
ENSMMUG00000061594		-2.442083407	8.34E-07	5.74E-06
ENSMMUG00000052786		-3.265371653	8.53E-08	6.94E-07
ENSMMUG00000000620	dehydrogenase/reductase 11 [Source:VGNC Symbol;Acc:VGNC:71792]	-1.959392284	7.77E-06	4.45E-05
ENSMMUG00000000618	gametogenetin binding protein 2 [Source:VGNC Symbol;Acc:VGNC:72746]	-1.693472498	2.29E-10	2.76E-09
ENSMMUG00000051190	Metazoan signal recognition particle RNA [Source:RFAM;Acc:RF00017]	-2.397992469	9.61E-06	5.41E-05
ENSMMUG00000007920	myosin XIX [Source:VGNC Symbol;Acc:VGNC:75026]	-1.637771945	3.35E-07	2.46E-06
ENSMMUG00000007923	phosphatidylinositol glycan anchor biosynthesis class W [Source:VGNC Symbol;Acc:VGNC:75850]	-1.326531923	0.000229572	0.000967201
ENSMMUG00000007918	zinc finger HIT-type containing 3 [Source:NCBI gene;Acc:574277]	-1.838435063	0.000601901	0.002289354
ENSMMUG00000007917	carbonic anhydrase 4 [Source:NCBI gene;Acc:716919]	-0.854518958	0.000828824	0.003039433
ENSMMUG00000017113	ubiquitin specific peptidase 32 [Source:NCBI gene;Acc:716857]	-1.529420237	2.93E-12	4.56E-11
ENSMMUG00000052977	putative protein FAM106C [Source:NCBI gene;Acc:106994084]	-1.349598294	9.95E-08	8.03E-07
ENSMMUG00000065117	septin 4 [Source:HGNC Symbol;Acc:HGNC:9165]	-1.282078294	3.93E-10	4.56E-09

ENSMMUG00000008423	septin 4 [Source:NCBI gene;Acc:714724]	-0.902121136	8.68E-15	1.83E-13
ENSMMUG00000002264	lactoperoxidase [Source:VGNC Symbol;Acc:VGNC:74440]	0.44277176	0.002859912	0.00911117
ENSMMUG00000056064	serine and arginine rich splicing factor 1 [Source:NCBI gene;Acc:713958]	-0.452444374	0.002664047	0.008549625
ENSMMUG00000004723	vascular endothelial zinc finger 1 [Source:NCBI gene;Acc:713848]	-0.353655216	0.003515917	0.010905889
ENSMMUG00000012644	A-kinase anchoring protein 1 [Source:VGNC Symbol;Acc:VGNC:69763]	-0.635135303	0.003629139	0.011217201
ENSMMUG00000002269	serine carboxypeptidase 1 [Source:VGNC Symbol;Acc:VGNC:82232]	-0.902820323	2.76E-05	0.000142368
ENSMMUG00000005793	ankyrin repeat and fibronectin type III domain containing 1 [Source:VGNC Symbol;Acc:VGNC:69712]	0.940996582	1.38E-05	7.59E-05
ENSMMUG00000059321		2.484038693	0.000342033	0.001383621
ENSMMUG00000006066	phosphatidylcholine transfer protein [Source:VGNC Symbol;Acc:VGNC:75789]	2.010199748	1.90E-06	1.23E-05
ENSMMUG00000006068	transmembrane protein 100 [Source:VGNC Symbol;Acc:VGNC:79521]	0.690335257	3.50E-08	3.00E-07
ENSMMUG00000022627	HLF transcription factor, PAR bZIP family member [Source:VGNC Symbol;Acc:VGNC:73478]	-1.757805754	0.000538469	0.002078472
ENSMMUG00000050544		-2.336723267	5.17E-10	5.91E-09
ENSMMUG00000049997		-2.128176791	4.26E-12	6.44E-11
ENSMMUG00000056602		-2.306122922	4.10E-06	2.48E-05
ENSMMUG00000010550	target of myb1 like 1 membrane trafficking protein [Source:VGNC Symbol;Acc:VGNC:78616]	-0.414306434	0.01408291	0.036271198
ENSMMUG00000013737	carbonic anhydrase 10 [Source:VGNC Symbol;Acc:VGNC:70486]	1.080620364	0.003710066	0.011422106
ENSMMUG00000062178	Metazoan signal recognition particle RNA [Source:RFAM;Acc:RF00017]	1.267963225	0.003642124	0.011247959
ENSMMUG00000051004		0.351989529	0.016418155	0.041331053
ENSMMUG00000001942	mbt domain containing 1 [Source:VGNC Symbol;Acc:VGNC:74538]	-1.802882001	4.59E-12	6.90E-11
ENSMMUG00000001940	NME/NM23 nucleoside diphosphate kinase 2 [Source:NCBI gene;Acc:100533957]	-2.127503991	5.43E-17	1.40E-15
ENSMMUG00000022528	sperm associated antigen 9 [Source:VGNC Symbol;Acc:VGNC:77920]	-0.426451574	0.00230207	0.00750818
ENSMMUG00000022741	WAP, follistatin/kazal, immunoglobulin, kunitz and netrin domain containing 2 [Source:VGNC Symbol;Acc:VGNC:79900]	1.775235103	2.31E-23	1.21E-21
ENSMMUG00000010117	LUC7 like 3 pre-mRNA splicing factor [Source:VGNC Symbol;Acc:VGNC:74346]	0.830965844	0.017301082	0.043156354
ENSMMUG00000003421	ankyrin repeat domain 40 [Source:VGNC Symbol;Acc:VGNC:69725]	0.927228439	0.009774666	0.02652485
ENSMMUG00000003414	ATP binding cassette subfamily C member 3 [Source:VGNC Symbol;Acc:VGNC:69572]	0.508352625	0.011730969	0.031071669
ENSMMUG00000017256	calcium voltage-gated channel subunit alpha1 G [Source:VGNC Symbol;Acc:VGNC:70504]	2.277085116	7.71E-34	1.14E-31
ENSMMUG00000017242	MYCBP associated protein [Source:VGNC Symbol;Acc:VGNC:74994]	1.466581069	3.27E-05	0.000165583
ENSMMUG00000017234	leucine rich repeat containing 59 [Source:VGNC Symbol;Acc:VGNC:81436]	-4.072404397	2.48E-49	1.84E-46
ENSMMUG00000020061	protein phosphatase 1 regulatory subunit 9B [Source:VGNC Symbol;Acc:VGNC:76372]	-1.478003215	2.56E-07	1.93E-06
ENSMMUG00000014687	sterile alpha motif domain containing 14 [Source:VGNC Symbol;Acc:VGNC:77112]	-1.99569945	2.70E-05	0.000139538
ENSMMUG00000014685	pyruvate dehydrogenase kinase 2 [Source:VGNC Symbol;Acc:VGNC:75933]	-1.89812774	0.017437255	0.043443667
ENSMMUG00000000153	integrin subunit alpha 3 [Source:VGNC Symbol;Acc:VGNC:73777]	-1.629105194	0.012066036	0.031828409
ENSMMUG00000015845	beta-1,4-N-acetyl-galactosaminyltransferase 2 [Source:VGNC Symbol;Acc:VGNC:70120]	-1.464624603	0.00100464	0.00361534
ENSMMUG00000012178	calcium binding and coiled-coil domain 2 [Source:VGNC Symbol;Acc:VGNC:70675]	0.423179222	0.008444532	0.023417845
ENSMMUG00000021087	SNF8 subunit of ESCRT-II [Source:VGNC Symbol;Acc:VGNC:77729]	-0.606375356	0.001307684	0.004575967
ENSMMUG00000003436	tubulin tyrosine ligase like 6 [Source:VGNC Symbol;Acc:VGNC:79632]	1.727559197	2.70E-25	1.64E-23
ENSMMUG00000060879	homeobox B13 [Source:NCBI gene;Acc:697572]	1.823870881	7.09E-25	4.24E-23

ENSMMUG00000055122		1.025391085	7.81E-09	7.45E-08
ENSMMUG00000052505	homeobox B9 [Source:NCBI gene;Acc:106994009]	1.506151594	7.59E-18	2.16E-16
ENSMMUG00000020868	homeobox B8 [Source:VGNC Symbol;Acc:VGNC:73505]	1.495736532	3.64E-20	1.30E-18
ENSMMUG00000055324	homeobox B7 [Source:NCBI gene;Acc:697089]	1.538224256	1.09E-13	2.01E-12
ENSMMUG00000062283	homeobox B6 [Source:NCBI gene;Acc:106994013]	1.515450137	1.89E-22	8.94E-21
ENSMMUG00000051420	homeobox B5 [Source:NCBI gene;Acc:696732]	1.585831016	2.42E-12	3.80E-11
ENSMMUG00000045203		1.39165309	3.04E-07	2.24E-06
ENSMMUG00000052130	homeobox B4 [Source:NCBI gene;Acc:106994012]	1.288492612	0.004724155	0.0141557
ENSMMUG00000057001	homeobox B2 [Source:NCBI gene;Acc:106994010]	1.154016937	1.21E-10	1.51E-09
ENSMMUG00000057095		3.320385379	1.20E-06	8.00E-06
ENSMMUG00000050742	src kinase associated phosphoprotein 1 [Source:NCBI gene;Acc:697043]	0.572730123	1.06E-09	1.16E-08
ENSMMUG00000019882	chromobox 1 [Source:NCBI gene;Acc:696673]	0.416064761	0.012346226	0.03246057
ENSMMUG00000003325	pyridoxamine 5'-phosphate oxidase [Source:VGNC Symbol;Acc:VGNC:76141]	-0.937326727	6.47E-08	5.36E-07
ENSMMUG00000004765	Sp2 transcription factor [Source:VGNC Symbol;Acc:VGNC:77845]	1.264440423	6.66E-06	3.87E-05
ENSMMUG00000021486	secernin 2 [Source:NCBI gene;Acc:694571]	1.484102214	1.89E-05	0.00010123
ENSMMUG00000057231	T-box transcription factor 21 [Source:NCBI gene;Acc:694044]	1.400518829	5.38E-09	5.25E-08
ENSMMUG00000019760	karyopherin subunit beta 1 [Source:VGNC Symbol;Acc:VGNC:74203]	-0.816954091	0.000216865	0.000917055
ENSMMUG00000020528	G protein-coupled receptor 179 [Source:VGNC Symbol;Acc:VGNC:73043]	-1.550788702	0.007154175	0.020235823
ENSMMUG00000020020	Rho GTPase activating protein 23 [Source:NCBI gene;Acc:693996]	1.149677772	4.17E-05	0.000205766
ENSMMUG00000007799	LIM and SH3 protein 1 [Source:VGNC Symbol;Acc:VGNC:74237]	1.213672328	1.80E-05	9.69E-05
ENSMMUG00000050458		0.916159817	0.018140536	0.044972275
ENSMMUG00000056248		2.906158854	2.77E-19	8.85E-18
ENSMMUG00000063779		3.077401381	2.62E-22	1.21E-20
ENSMMUG00000000289	calcium voltage-gated channel auxiliary subunit beta 1 [Source:VGNC Symbol;Acc:VGNC:70509]	-0.586433778	0.000718902	0.002675363
ENSMMUG00000030060	F-box and leucine rich repeat protein 20 [Source:VGNC Symbol;Acc:VGNC:81407]	-0.654277056	4.88E-06	2.91E-05
ENSMMUG00000009132	mediator complex subunit 1 [Source:VGNC Symbol;Acc:VGNC:74724]	-0.946221807	0.000648662	0.002448373
ENSMMUG00000001404	neuronal differentiation 2 [Source:VGNC Symbol;Acc:VGNC:75321]	-3.010796929	0.01056766	0.028360188
ENSMMUG00000004587	gasdermin B [Source:VGNC Symbol;Acc:VGNC:73219]	2.270441711	0.000133395	0.000591757
ENSMMUG00000057790		2.046251759	0.00600823	0.017409544
ENSMMUG00000058758		2.608003094	8.07E-06	4.61E-05
ENSMMUG00000064026	Metazoan signal recognition particle RNA [Source:RFAM;Acc:RF00017]	1.078556276	3.27E-06	2.01E-05
ENSMMUG00000064723	gasdermin A [Source:NCBI gene;Acc:698841]	2.10724872	0.002541633	0.008201134
ENSMMUG00000002923	colony stimulating factor 3 [Source:VGNC Symbol;Acc:VGNC:71520]	1.221438773	9.90E-10	1.09E-08
ENSMMUG00000002926	mediator complex subunit 24 [Source:NCBI gene;Acc:699536]	1.43231528	3.14E-25	1.89E-23
ENSMMUG00000002933	nuclear receptor subfamily 1 group D member 1 [Source:VGNC Symbol;Acc:VGNC:75502]	-0.44132296	0.012418229	0.032623974
ENSMMUG00000045187	CASC3 exon junction complex subunit [Source:NCBI gene;Acc:699712]	1.255359086	2.04E-14	4.07E-13
ENSMMUG00000015160	Rap guanine nucleotide exchange factor like 1 [Source:VGNC Symbol;Acc:VGNC:76501]	1.116186814	6.35E-09	6.15E-08
ENSMMUG00000056646		1.140139439	1.07E-06	7.26E-06
ENSMMUG00000004750	WAS/WASL interacting protein family member 2 [Source:NCBI gene;Acc:700279]	0.97345498	0.000844836	0.003090509
ENSMMUG00000008941	DNA topoisomerase II alpha [Source:VGNC Symbol;Acc:VGNC:78620]	-0.853590017	1.24E-09	1.34E-08
ENSMMUG00000009386	tensin 4 [Source:VGNC Symbol;Acc:VGNC:79432]	1.344744597	0.007525031	0.02115969
ENSMMUG00000001497	keratin 27 [Source:VGNC Symbol;Acc:VGNC:74169]	-0.491879752	0.000879921	0.003199909
ENSMMUG00000043282		-0.653641847	0.004250797	0.012909957

ENSMMUG00000018644	keratin 32 [Source:VGNC Symbol;Acc:VGNC:74216]	-0.422793835	0.012910325	0.033707766
ENSMMUG00000053216	U2 spliceosomal RNA [Source:RFAM;Acc:RF00004]	-0.655587045	0.006999745	0.019878632
ENSMMUG00000018653	keratin, type I cytoskeletal 19 [Source:NCBI gene;Acc:707129]	0.652632947	0.00713019	0.020179525
ENSMMUG00000013048	junction plakoglobin [Source:VGNC Symbol;Acc:VGNC:73960]	1.089803767	1.91E-05	0.000101887
ENSMMUG00000021753	tetratricopeptide repeat domain 25 [Source:NCBI gene;Acc:708614]	0.685181113	3.15E-06	1.95E-05
ENSMMUG00000016114	2',3'-cyclic nucleotide 3' phosphodiesterase [Source:VGNC Symbol;Acc:VGNC:71279]	0.694384772	1.90E-06	1.22E-05
ENSMMUG00000016118	DnaJ heat shock protein family (Hsp40) member C7 [Source:NCBI gene;Acc:708729]	0.387539	0.0067271	0.019192554
ENSMMUG00000003894	zinc finger protein 385C [Source:NCBI gene;Acc:708928]	0.471919437	0.000538802	0.002079219
ENSMMUG00000049440	chromosome 16 C17orf113 homolog [Source:NCBI gene;Acc:106994045]	0.705342754	5.61E-16	1.31E-14
ENSMMUG00000003437	signal transducer and activator of transcription 5B [Source:NCBI gene;Acc:709760]	2.268935955	8.97E-32	1.00E-29
ENSMMUG00000052220		-0.639264098	7.03E-05	0.000331308
ENSMMUG00000035823	U7 small nuclear RNA [Source:RFAM;Acc:RF00066]	-0.711691838	0.007005563	0.019891346
ENSMMUG00000050793	N-acetyl-alpha-glucosaminidase [Source:NCBI gene;Acc:707186]	2.252943727	6.42E-30	6.14E-28
ENSMMUG00000009884	hydroxysteroid 17-beta dehydrogenase 1 [Source:VGNC Symbol;Acc:VGNC:73528]	2.378297447	9.97E-14	1.85E-12
ENSMMUG00000009886	Coenzyme A synthase [Source:VGNC Symbol;Acc:VGNC:81373]	2.05342186	1.11E-47	6.84E-45
ENSMMUG00000009887	MAX dimerization protein MLX [Source:VGNC Symbol;Acc:VGNC:74726]	2.083903778	9.86E-20	3.34E-18
ENSMMUG00000009889	PSMC3 interacting protein [Source:VGNC Symbol;Acc:VGNC:76282]	1.987983842	3.77E-24	2.08E-22
ENSMMUG00000016796	reticulophagy regulator family member 3 [Source:VGNC Symbol;Acc:VGNC:76845]	2.087678792	4.31E-38	9.27E-36
ENSMMUG00000016797	tubulin gamma 1 [Source:NCBI gene;Acc:710756]	1.988651123	1.76E-26	1.25E-24
ENSMMUG00000006086	pleckstrin homology, MyTH4 and FERM domain containing H3 [Source:VGNC Symbol;Acc:VGNC:76113]	1.619780875	8.61E-11	1.09E-09
ENSMMUG00000006090	C-C motif chemokine receptor 10 [Source:VGNC Symbol;Acc:VGNC:82119]	1.937490953	0.000878455	0.003195362
ENSMMUG00000006091	contactin associated protein 1 [Source:VGNC Symbol;Acc:VGNC:71390]	2.191689367	9.39E-32	1.04E-29
ENSMMUG00000006093	enhancer of zeste 1 polycomb repressive complex 2 subunit [Source:VGNC Symbol;Acc:VGNC:72457]	2.131869645	6.24E-36	1.14E-33
ENSMMUG00000056565	Metazoan signal recognition particle RNA [Source:RFAM;Acc:RF00017]	2.304595965	0.018133395	0.044962089
ENSMMUG00000058566		1.701169791	1.61E-39	4.12E-37
ENSMMUG00000029971	beclin 1 [Source:VGNC Symbol;Acc:VGNC:70044]	0.573411546	5.47E-05	0.000263559
ENSMMUG00000012685	proteasome activator subunit 3 [Source:VGNC Symbol;Acc:VGNC:76457]	0.422550771	0.000591176	0.002253183
ENSMMUG00000014123	alanyl-tRNA editing protein Aarsd1 [Source:NCBI gene;Acc:712203]	1.134191389	7.31E-06	4.22E-05
ENSMMUG00000008091	RUN domain containing 1 [Source:VGNC Symbol;Acc:VGNC:77101]	1.028363389	4.87E-05	0.000237302
ENSMMUG00000064920		1.347011964	1.94E-22	9.11E-21
ENSMMUG00000008090	ribosomal protein L27 [Source:NCBI gene;Acc:712352]	1.151087555	0.000279132	0.00114955
ENSMMUG00000015999	interferon induced protein 35 [Source:VGNC Symbol;Acc:VGNC:73564]	1.141005547	8.26E-06	4.71E-05
ENSMMUG00000016001	vesicle amine transport 1 [Source:VGNC Symbol;Acc:VGNC:79058]	1.104623059	4.82E-11	6.34E-10
ENSMMUG00000065269	Rho family GTPase 2 [Source:NCBI gene;Acc:712559]	1.070539482	1.15E-06	7.70E-06
ENSMMUG00000052607		1.339063396	5.67E-37	1.12E-34
ENSMMUG00000012071	DEAH-box helicase 8 [Source:VGNC Symbol;Acc:VGNC:71765]	-0.665564806	9.41E-08	7.61E-07
ENSMMUG00000006808	homologous recombination factor with OB-fold [Source:VGNC Symbol;Acc:VGNC:70421]	-2.464796249	0.006340922	0.018248763
ENSMMUG00000019923	ataxin 7 like 3 [Source:VGNC Symbol;Acc:VGNC:70128]	-0.581548264	0.002057058	0.006821594
ENSMMUG00000017100	solute carrier family 4 member 1 (Diego blood group) [Source:NCBI gene;Acc:100427618]	-0.482044525	0.008790797	0.024240893
ENSMMUG00000013983	RUN domain containing 3A [Source:VGNC Symbol;Acc:VGNC:77032]	0.629521156	1.21E-08	1.12E-07
ENSMMUG00000017944	solute carrier family 25 member 39 [Source:VGNC Symbol;Acc:VGNC:77568]	0.495798004	0.000682325	0.002561095
ENSMMUG00000023751	granulin precursor [Source:VGNC Symbol;Acc:VGNC:73293]	0.449188009	0.007802458	0.021844525
ENSMMUG00000041006	family with sequence similarity 171 member A2 [Source:NCBI gene;Acc:712099]	0.552173916	3.07E-06	1.90E-05
ENSMMUG00000012659	integrin subunit alpha 2b [Source:VGNC Symbol;Acc:VGNC:73776]	0.293564039	0.003641862	0.011247959
ENSMMUG00000012658	G-patch domain containing 8 [Source:VGNC Symbol;Acc:VGNC:73026]	0.342702286	0.000113766	0.000511724

ENSMMUG00000029927	ADAM metallopeptidase domain 11 [Source:VGNC Symbol;Acc:VGNC:69466]	-0.863859999	0.001358005	0.004730847
ENSMMUG00000013115	glial fibrillary acidic protein [Source:VGNC Symbol;Acc:VGNC:72913]	0.919447395	0.003894591	0.011940622
ENSMMUG00000062273	dephospho-CoA kinase domain containing [Source:NCBI gene;Acc:715841]	1.911933128	2.17E-08	1.94E-07
ENSMMUG00000060665		-1.103658362	2.34E-05	0.000122495
ENSMMUG00000054246		-1.700133758	0.001331852	0.004650661
ENSMMUG00000021932	Rho GTPase activating protein 27 [Source:VGNC Symbol;Acc:VGNC:69935]	-1.228022414	0.005336661	0.015690461
ENSMMUG00000008459	pleckstrin homology and RUN domain containing M1 [Source:NCBI gene;Acc:716464]	-0.849012907	0.001080008	0.003858472
ENSMMUG00000049983		-1.280720756	0.008698305	0.024021576
ENSMMUG00000057840	leucine-rich repeat-containing protein 37A3 [Source:NCBI gene;Acc:715030]	-0.806971873	0.004543712	0.013687437
ENSMMUG00000005548	N-ethylmaleimide sensitive factor, vesicle fusing ATPase [Source:VGNC Symbol;Acc:VGNC:75536]	1.020492846	1.90E-12	3.03E-11
ENSMMUG00000057209		-0.793693975	1.04E-05	5.82E-05
ENSMMUG00000004122	microtubule associated protein tau [Source:NCBI gene;Acc:574327]	0.76473035	1.45E-07	1.14E-06
ENSMMUG00000053257	Y RNA [Source:RFAM;Acc:RF00019]	1.280660038	1.39E-12	2.26E-11
ENSMMUG00000003289	integrin subunit beta 3 [Source:NCBI gene;Acc:717379]	0.952209081	1.02E-14	2.13E-13
ENSMMUG00000010588	EF-hand calcium binding domain 13 [Source:HGNC Symbol;Acc:HGNC:26864]	0.763273546	0.000380493	0.001523822
ENSMMUG00000002166	tousled like kinase 2 [Source:VGNC Symbol;Acc:VGNC:78368]	-3.324959825	0.000153433	0.000669634
ENSMMUG00000002167	mannose receptor C type 2 [Source:VGNC Symbol;Acc:VGNC:74784]	-2.003968996	0.006833958	0.019459983
ENSMMUG00000002168	membrane associated ring-CH-type finger 10 [Source:VGNC Symbol;Acc:VGNC:74593]	-1.692900704	0.005523661	0.016175315
ENSMMUG00000015185	endoplasmic reticulum to nucleus signaling 1 [Source:VGNC Symbol;Acc:VGNC:72429]	0.412228034	0.000156642	0.000681233
ENSMMUG00000011809	platelet and endothelial cell adhesion molecule 1 [Source:VGNC Symbol;Acc:VGNC:75938]	-0.864212254	5.49E-06	3.24E-05
ENSMMUG00000059400		-0.732032619	0.007160117	0.020248768
ENSMMUG00000006317	mast cell immunoglobulin like receptor 1 [Source:VGNC Symbol;Acc:VGNC:74843]	-0.871806635	0.002923764	0.009294625
ENSMMUG00000009463	DEAD-box helicase 5 [Source:VGNC Symbol;Acc:VGNC:71763]	-0.526683019	2.97E-05	0.000151832
ENSMMUG00000063689		-0.705310872	0.001418976	0.004913242
ENSMMUG00000009461	SMAD specific E3 ubiquitin protein ligase 2 [Source:VGNC Symbol;Acc:VGNC:77727]	-0.838070594	1.24E-09	1.34E-08
ENSMMUG00000060767		3.383221829	1.54E-18	4.65E-17
ENSMMUG00000060015	axin 2 [Source:NCBI gene;Acc:718566]	0.378623478	0.003558531	0.011026539
ENSMMUG00000051240		0.994767757	0.00033127	0.001343745
ENSMMUG00000018378	protein kinase C alpha [Source:VGNC Symbol;Acc:VGNC:76409]	-1.401534525	0.005326554	0.015670065
ENSMMUG00000002959	helicase with zinc finger [Source:VGNC Symbol;Acc:VGNC:73178]	3.119937106	5.71E-31	5.92E-29
ENSMMUG00000057157		2.829050117	1.72E-20	6.42E-19
ENSMMUG00000054673		2.231997477	0.001951269	0.006515945
ENSMMUG00000051322	somatostatin receptor 2 [Source:NCBI gene;Acc:695526]	1.145857181	0.002469608	0.007991334
ENSMMUG00000063103		-0.624429071	0.001428957	0.00494103
ENSMMUG00000001266	component of oligomeric golgi complex 1 [Source:VGNC Symbol;Acc:VGNC:71383]	-1.391201572	0.000335817	0.00136033

ENSMMUG00000001269	chromosome 16 C17orf80 homolog [Source:VGNC Symbol;Acc:VGNC:70440]	1.162301922	7.64E-14	1.45E-12
ENSMMUG00000001271	cleavage and polyadenylation specific factor 4 like [Source:NCBI gene;Acc:698279]	1.472438062	0.00130331	0.004561739
ENSMMUG00000049948		-1.416012441	1.48E-09	1.58E-08
ENSMMUG00000064463		1.482392738	7.43E-06	4.28E-05
ENSMMUG00000049403		0.876046618	4.00E-08	3.40E-07
ENSMMUG00000064052	CD300 molecule like family member d [Source:NCBI gene;Acc:703142]	1.943136602	7.14E-12	1.04E-10
ENSMMUG00000058592		2.016795163	1.84E-17	4.99E-16
ENSMMUG00000020462	CD300e molecule [Source:NCBI gene;Acc:114673189]	2.277920851	7.82E-10	8.74E-09
ENSMMUG00000005737	SLC9A3 regulator 1 [Source:VGNC Symbol;Acc:VGNC:77639]	2.880651237	3.72E-08	3.17E-07
ENSMMUG00000005738	N-acetyltransferase 9 (putative) [Source:NCBI gene;Acc:699085]	4.020316411	0.000112145	0.000505505
ENSMMUG00000005744	glutamate ionotropic receptor NMDA type subunit 2C [Source:VGNC Symbol;Acc:VGNC:73284]	0.50665445	0.017132263	0.042816221
ENSMMUG00000023405	otopetrin 3 [Source:VGNC Symbol;Acc:VGNC:75654]	0.557271032	0.000657276	0.002477104
ENSMMUG00000023406	HID1 domain containing [Source:VGNC Symbol;Acc:VGNC:73394]	0.613521933	0.000139365	0.000614382
ENSMMUG00000018031	solute carrier family 25 member 19 [Source:VGNC Symbol;Acc:VGNC:77474]	-1.806050768	1.15E-06	7.71E-06
ENSMMUG00000004756	growth factor receptor bound protein 2 [Source:NCBI gene;Acc:702041]	-0.940665727	0.012465829	0.032714253
ENSMMUG00000058142		-1.667528409	3.86E-12	5.86E-11
ENSMMUG00000034931	U6 spliceosomal RNA [Source:RFAM;Acc:RF00026]	-2.694021111	0.004374809	0.013230079
ENSMMUG00000022120	transmembrane protein 94 [Source:VGNC Symbol;Acc:VGNC:79760]	-1.331321145	3.11E-17	8.25E-16
ENSMMUG00000022121	CASK interacting protein 2 [Source:VGNC Symbol;Acc:VGNC:70577]	-1.664521172	1.08E-07	8.71E-07
ENSMMUG00000022123	tRNA splicing endonuclease subunit 54 [Source:VGNC Symbol;Acc:VGNC:79666]	-1.380372607	2.09E-10	2.53E-09
ENSMMUG00000001228	LLGL scribble cell polarity complex component 2 [Source:NCBI gene;Acc:706973]	-0.71847342	3.25E-06	2.00E-05
ENSMMUG00000064684		0.548326976	0.006385879	0.018356771
ENSMMUG00000021636	SAP30 binding protein [Source:VGNC Symbol;Acc:VGNC:77142]	-1.296814993	0.002643482	0.008490978
ENSMMUG00000013646	unk zinc finger [Source:VGNC Symbol;Acc:VGNC:79803]	1.429618257	3.45E-10	4.04E-09
ENSMMUG00000002822	unc-13 homolog D [Source:VGNC Symbol;Acc:VGNC:79375]	1.911238396	1.76E-21	7.36E-20
ENSMMUG00000009381	WW domain binding protein 2 [Source:VGNC Symbol;Acc:VGNC:78786]	1.734200004	1.16E-19	3.87E-18
ENSMMUG00000009377	tripartite motif containing 47 [Source:VGNC Symbol;Acc:VGNC:79171]	2.271014031	5.17E-17	1.34E-15
ENSMMUG00000016774	tripartite motif containing 65 [Source:NCBI gene;Acc:708355]	2.055859668	0.002246968	0.007347258
ENSMMUG00000008885	exocyst complex component 7 [Source:VGNC Symbol;Acc:VGNC:72319]	0.48963184	2.96E-06	1.83E-05
ENSMMUG00000054437		0.523568312	3.88E-05	0.000192583
ENSMMUG00000003533	forkhead box J1 [Source:VGNC Symbol;Acc:VGNC:72701]	0.367257031	0.018885608	0.046523848
ENSMMUG00000003534	ring finger protein 157 [Source:VGNC Symbol;Acc:VGNC:76860]	0.972677597	1.21E-08	1.12E-07
ENSMMUG00000055131	UBA like domain containing 2 [Source:NCBI gene;Acc:709614]	-1.851383422	0.010500056	0.028199173
ENSMMUG00000003539	glutamine rich 2 [Source:VGNC Symbol;Acc:VGNC:76534]	1.981625818	5.25E-08	4.41E-07
ENSMMUG00000018967	ubiquitin conjugating enzyme E2 O [Source:VGNC Symbol;Acc:VGNC:79813]	1.367324256	4.15E-22	1.86E-20
ENSMMUG00000046918		2.637138218	7.22E-08	5.94E-07
ENSMMUG00000054111	Metazoan signal recognition particle RNA [Source:RFAM;Acc:RF00017]	2.166288993	0.000103245	0.000470391
ENSMMUG00000055379		1.063596361	6.16E-08	5.12E-07
ENSMMUG00000053937		1.241133566	4.73E-10	5.45E-09
ENSMMUG00000011932	transmembrane channel like 6 [Source:VGNC Symbol;Acc:VGNC:79109]	-0.865071288	0.018376086	0.04544986
ENSMMUG00000011935	transmembrane channel like 8 [Source:VGNC Symbol;Acc:VGNC:79253]	-0.998778346	0.000621578	0.002358149

ENSMMUG00000021864	synaptogyrin 2 [Source:NCBI gene;Acc:709310]	-3.293540128	0.006373661	0.018332311
ENSMMUG00000013767	baculoviral IAP repeat containing 5 [Source:NCBI gene;Acc:709565]	-1.058501281	0.012433541	0.032646501
ENSMMUG00000049183	transmembrane protein 235 [Source:NCBI gene;Acc:715249]	-1.020876206	0.01545682	0.039190399
ENSMMUG00000059852		-1.625454765	0.008161932	0.022722254
ENSMMUG00000008237	phosphatidylglycerophosphate synthase 1 [Source:VGNC Symbol;Acc:VGNC:75890]	-0.819572501	0.001769997	0.005976562
ENSMMUG00000008239	dynein axonemal heavy chain 17 [Source:NCBI gene;Acc:715774]	-0.55368298	0.009482822	0.025860656
ENSMMUG00000049898		-0.749919658	0.010031641	0.027113038
ENSMMUG00000051537		3.169660561	1.17E-09	1.27E-08
ENSMMUG00000013313	cytohesin 1 [Source:VGNC Symbol;Acc:VGNC:71828]	0.568990031	0.00418845	0.012739252
ENSMMUG00000005355	TIMP metallopeptidase inhibitor 2 [Source:NCBI gene;Acc:574357]	1.77743166	0.010670408	0.028573881
ENSMMUG00000023027	endo-beta-N-acetylglucosaminidase [Source:VGNC Symbol;Acc:VGNC:72099]	-0.676094141	0.001281413	0.004493581
ENSMMUG00000023028	RNA binding fox-1 homolog 3 [Source:VGNC Symbol;Acc:VGNC:76550]	-0.423730547	0.011515839	0.030572841
ENSMMUG00000061312		2.568707799	0.015648178	0.039648468
ENSMMUG00000058715		1.899288416	0.000270174	0.001116688
ENSMMUG00000016414	ectonucleotide pyrophosphatase/phosphodiesterase 7 [Source:VGNC Symbol;Acc:VGNC:72090]	1.810690467	0.000260938	0.001083952
ENSMMUG00000046417	chromobox 2 [Source:NCBI gene;Acc:717462]	3.367920954	3.77E-20	1.34E-18
ENSMMUG00000016417	chromobox 8 [Source:NCBI gene;Acc:717514]	2.109759202	7.82E-20	2.68E-18
ENSMMUG00000016418	chromobox 4 [Source:VGNC Symbol;Acc:VGNC:70558]	0.685716182	3.21E-05	0.000162772
ENSMMUG00000020840	TBC1 domain family member 16 [Source:VGNC Symbol;Acc:VGNC:78228]	0.684535727	4.79E-10	5.52E-09
ENSMMUG00000064603	caspase recruitment domain family member 14 [Source:NCBI gene;Acc:712205]	0.420338258	0.001562143	0.005341802
ENSMMUG00000005455	ring finger protein 213 [Source:VGNC Symbol;Acc:VGNC:76734]	0.974831603	2.85E-07	2.12E-06
ENSMMUG00000056437	Metazoan signal recognition particle RNA [Source:RFAM;Acc:RF00017]	2.509707902	2.31E-07	1.75E-06
ENSMMUG00000064563		-1.173506965	3.29E-06	2.02E-05
ENSMMUG00000000589	N-methylpurine DNA glycosylase [Source:VGNC Symbol;Acc:VGNC:74915]	-1.256538003	0.019981685	0.048818517
ENSMMUG00000000590	NPR3 like, GATOR1 complex subunit [Source:VGNC Symbol;Acc:VGNC:75173]	1.179752115	0.002908668	0.009254568
ENSMMUG00000029310	hemoglobin, alpha 2 [Source:NCBI gene;Acc:693930]	-0.644869227	0.000107594	0.000487806
ENSMMUG00000019566	hemoglobin subunit alpha 1 [Source:NCBI gene;Acc:701930]	-0.937117655	3.53E-06	2.15E-05
ENSMMUG00000000610	post-glycosylphosphatidylinositol attachment to proteins 6 [Source:NCBI gene;Acc:722297]	-0.933864526	0.001284735	0.004504166
ENSMMUG00000029296	phosphatidylinositol glycan anchor biosynthesis class Q [Source:VGNC Symbol;Acc:VGNC:75798]	0.971036671	2.97E-06	1.84E-05
ENSMMUG00000038629	RAB40C, member RAS oncogene family [Source:NCBI gene;Acc:722336]	-0.421187227	0.008968761	0.024644567
ENSMMUG00000001986	WD repeat domain 24 [Source:VGNC Symbol;Acc:VGNC:79289]	-0.963862557	0.000196902	0.000840788
ENSMMUG00000050065		-1.097663038	0.000401328	0.001597771
ENSMMUG00000016906	F-box and leucine rich repeat protein 16 [Source:VGNC Symbol;Acc:VGNC:81405]	-1.226805657	8.91E-10	9.83E-09
ENSMMUG00000014213	calcium voltage-gated channel subunit alpha1 H [Source:VGNC Symbol;Acc:VGNC:70505]	-1.90577056	0.006928917	0.0197039
ENSMMUG00000050892	mastin-like [Source:NCBI gene;Acc:722432]	-0.34728739	0.008773828	0.0241986
ENSMMUG00000063740		-0.419753164	0.003161521	0.009945981

ENSMMUG00000051011	Metazoan signal recognition particle RNA [Source:RFAM;Acc:RF00017]	-2.718192505	0.016528505	0.041552421
ENSMMUG00000044277	ubiquitin conjugating enzyme E2 I [Source:NCBI gene;Acc:722434]	-0.714865084	0.000802744	0.002953285
ENSMMUG00000021415	BAI1 associated protein 3 [Source:VGNC Symbol;Acc:VGNC:70223]	0.943617276	0.001015126	0.003648347
ENSMMUG00000012439	N-acetylglucosamine-1-phosphate transferase subunit gamma [Source:NCBI gene;Acc:722441]	0.769182204	0.003678315	0.011340835
ENSMMUG00000008119	telomere maintenance 2 [Source:NCBI gene;Acc:701679]	-1.841831056	3.27E-12	5.06E-11
ENSMMUG00000000044	intraflagellar transport 140 [Source:VGNC Symbol;Acc:VGNC:73456]	-0.712672473	0.000378802	0.00151787
ENSMMUG00000062198	transmembrane protein 204 [Source:NCBI gene;Acc:701926]	-1.466847973	0.000456346	0.001790846
ENSMMUG00000000978	cramped chromatin regulator homolog 1 [Source:NCBI gene;Acc:722473]	-0.833052417	5.81E-10	6.60E-09
ENSMMUG00000017496	mitogen-activated protein kinase 8 interacting protein 3 [Source:VGNC Symbol;Acc:VGNC:74651]	-0.377161997	0.001247049	0.004385521
ENSMMUG00000007936	methionine sulfoxide reductase B1 [Source:NCBI gene;Acc:693358]	-0.514821037	0.010266511	0.027687281
ENSMMUG00000007937	ribosomal protein L3 like [Source:VGNC Symbol;Acc:VGNC:76923]	-2.291318455	0.000594744	0.002265036
ENSMMUG00000029265	zinc finger protein 598 [Source:NCBI gene;Acc:694129]	-0.72923872	1.35E-05	7.41E-05
ENSMMUG00000002808	TSC complex subunit 2 [Source:VGNC Symbol;Acc:VGNC:78659]	-0.489557543	0.00917897	0.025147631
ENSMMUG00000029263	polycystin 1, transient receptor potential channel interacting [Source:NCBI gene;Acc:694905]	-1.193865622	7.49E-12	1.09E-10
ENSMMUG00000019223	TNF receptor associated factor 7 [Source:VGNC Symbol;Acc:VGNC:79252]	0.893073224	5.57E-05	0.000267994
ENSMMUG00000055128	phosphoglycolate phosphatase [Source:NCBI gene;Acc:695591]	-0.710454877	0.001264515	0.004438523
ENSMMUG00000019237	enoyl-CoA delta isomerase 1 [Source:VGNC Symbol;Acc:VGNC:81391]	-0.400695456	0.014972671	0.0382111
ENSMMUG00000019238	RNA binding protein with serine rich domain 1 [Source:NCBI gene;Acc:695816]	-0.444867962	1.60E-06	1.04E-05
ENSMMUG00000000001	cyclin F [Source:VGNC Symbol;Acc:VGNC:70729]	-0.632086424	0.004258509	0.012930732
ENSMMUG00000019095	TBC1 domain family member 24 [Source:NCBI gene;Acc:697106]	1.148609052	2.39E-08	2.12E-07
ENSMMUG00000011459	3-phosphoinositide dependent protein kinase 1 [Source:NCBI gene;Acc:697523]	0.751093154	1.25E-14	2.57E-13
ENSMMUG00000061321	host cell factor C1 regulator 1 [Source:NCBI gene;Acc:700351]	0.709800056	0.012570983	0.032937753
ENSMMUG00000007666	THO complex 6 [Source:VGNC Symbol;Acc:VGNC:79703]	0.714562082	0.010486778	0.028168614
ENSMMUG00000007667	BICD family like cargo adaptor 2 [Source:NCBI gene;Acc:700092]	1.053496006	0.000705356	0.002634869
ENSMMUG00000009487	zinc finger protein 205 [Source:VGNC Symbol;Acc:VGNC:78869]	1.011563521	1.04E-14	2.18E-13
ENSMMUG00000064194		0.90950743	0.000513495	0.001992483
ENSMMUG00000020970	zinc finger protein 213 [Source:VGNC Symbol;Acc:VGNC:78872]	0.872812988	1.00E-12	1.65E-11
ENSMMUG00000051323	caspase-14-like [Source:NCBI gene;Acc:702430]	1.175783459	1.18E-09	1.28E-08
ENSMMUG00000064961		-1.187608393	0.000196828	0.000840713
ENSMMUG00000017972	zinc finger protein 200 [Source:VGNC Symbol;Acc:VGNC:78867]	-1.076400374	3.12E-09	3.17E-08
ENSMMUG00000007569	clusterin associated protein 1 [Source:VGNC Symbol;Acc:VGNC:71211]	-0.602080502	0.001835064	0.006173775
ENSMMUG00000021866	deoxyribonuclease 1 [Source:VGNC Symbol;Acc:VGNC:71946]	0.396054619	0.000814975	0.002994567
ENSMMUG00000021868	TNF receptor associated protein 1 [Source:VGNC Symbol;Acc:VGNC:79019]	0.908984258	1.02E-10	1.29E-09
ENSMMUG00000039943	adenylate cyclase 9 [Source:NCBI gene;Acc:706789]	1.73130628	3.65E-05	0.000182693
ENSMMUG00000061940		3.355963155	0.001271421	0.004461708
ENSMMUG00000051196		-2.209201769	0.000134822	0.000597017

ENSMMUG00000040803	sarcalumenin [Source:NCBI gene;Acc:704655]	-1.99591674	4.96E-07	3.55E-06
ENSMMUG00000044729	transcription factor AP-4 [Source:NCBI gene;Acc:707513]	-1.719528807	8.16E-06	4.65E-05
ENSMMUG00000044344	vasorin [Source:NCBI gene;Acc:100426861]	-0.984185526	2.71E-06	1.70E-05
ENSMMUG00000008134	DnaJ heat shock protein family (Hsp40) member A3 [Source:NCBI gene;Acc:708783]	0.684070192	7.26E-07	5.05E-06
ENSMMUG00000020540	NmrA like redox sensor 1 [Source:VGNC Symbol;Acc:VGNC:75252]	-1.845073296	1.76E-12	2.82E-11
ENSMMUG00000001888	mahogunin ring finger 1 [Source:VGNC Symbol;Acc:VGNC:74542]	-1.224849059	5.17E-05	0.000250373
ENSMMUG00000011001	ankyrin repeat and sterile alpha motif domain containing 3 [Source:VGNC Symbol;Acc:VGNC:69735]	0.54933999	0.001388897	0.004820348
ENSMMUG00000014311	chromosome 20 C16orf71 homolog [Source:NCBI gene;Acc:709711]	0.454782095	0.003288603	0.010291197
ENSMMUG00000049336		0.523894465	0.016273838	0.041030435
ENSMMUG00000014313	zinc finger protein 500 [Source:VGNC Symbol;Acc:VGNC:79406]	0.655715146	1.44E-05	7.87E-05
ENSMMUG00000017945	septin 12 [Source:VGNC Symbol;Acc:VGNC:82235]	0.81211717	1.81E-07	1.40E-06
ENSMMUG00000049327		1.188890969	0.000105193	0.000477944
ENSMMUG00000017946	rogdi atypical leucine zipper [Source:VGNC Symbol;Acc:VGNC:76862]	0.952005353	5.22E-12	7.82E-11
ENSMMUG00000009567	glyoxylate reductase 1 homolog [Source:NCBI gene;Acc:706933]	3.300545234	7.27E-42	2.45E-39
ENSMMUG00000017822	periplakin [Source:VGNC Symbol;Acc:VGNC:76240]	2.605848317	1.48E-08	1.35E-07
ENSMMUG00000018047	SEC14 like lipid binding 5 [Source:VGNC Symbol;Acc:VGNC:77327]	-0.552015652	0.007383879	0.020786432
ENSMMUG00000011499	N-acetylglucosamine-1-phosphodiester alpha-N-acetylglucosaminidase [Source:VGNC Symbol;Acc:VGNC:74978]	-1.473249013	6.43E-05	0.000305308
ENSMMUG00000046237	4-aminobutyrate aminotransferase [Source:NCBI gene;Acc:714017]	-1.154070964	1.27E-10	1.58E-09
ENSMMUG00000022714	phosphomannomutase 2 [Source:VGNC Symbol;Acc:VGNC:76193]	-0.651829671	0.000211038	0.000895735
ENSMMUG00000047388	ubiquitin specific peptidase 7 [Source:NCBI gene;Acc:714625]	0.271706468	0.001312446	0.004590466
ENSMMUG00000001944	chromosome 20 C16orf72 homolog [Source:VGNC Symbol;Acc:VGNC:70443]	1.519241746	1.45E-17	4.01E-16
ENSMMUG00000010142	glutamate ionotropic receptor NMDA type subunit 2A [Source:VGNC Symbol;Acc:VGNC:73282]	3.462801904	0.001522383	0.005223724
ENSMMUG00000005913	activating transcription factor 7 interacting protein 2 [Source:VGNC Symbol;Acc:VGNC:70103]	-1.169676114	0.000881882	0.003206255
ENSMMUG00000060993	Metazoan signal recognition particle RNA [Source:RFAM;Acc:RF00017]	-1.915177356	0.009052091	0.024845921
ENSMMUG00000056165		-1.914247053	7.65E-06	4.39E-05
ENSMMUG00000060713	epithelial membrane protein 2 [Source:NCBI gene;Acc:710315]	-2.961775867	2.47E-12	3.88E-11
ENSMMUG00000006515	C-type lectin domain containing 16A [Source:VGNC Symbol;Acc:VGNC:71369]	-0.815913744	0.000131168	0.0005831
ENSMMUG00000000566		1.684500765	0.00145531	0.005022633
ENSMMUG00000008784	lipopolysaccharide induced TNF factor [Source:NCBI gene;Acc:711743]	4.234737573	6.26E-06	3.65E-05
ENSMMUG00000056339		2.726855872	0.000481138	0.001878691
ENSMMUG00000062037	stannin [Source:NCBI gene;Acc:711909]	2.741703914	1.37E-17	3.78E-16
ENSMMUG00000014240	thioredoxin domain containing 11 [Source:VGNC Symbol;Acc:VGNC:79678]	3.334099831	7.35E-16	1.69E-14
ENSMMUG00000011242	zinc finger CCCH-type containing 7A [Source:VGNC Symbol;Acc:VGNC:79905]	0.42056533	0.011207952	0.0298731
ENSMMUG00000019632	ribosomal L1 domain containing 1 [Source:VGNC Symbol;Acc:VGNC:77096]	0.739420756	3.13E-10	3.68E-09
ENSMMUG00000057345	calcineurin like phosphoesterase domain containing 1 [Source:NCBI gene;Acc:716978]	1.73767332	1.52E-18	4.60E-17
ENSMMUG00000025991		2.392881409	0.012437097	0.032646501
ENSMMUG00000017379	myocardin related transcription factor B [Source:VGNC Symbol;Acc:VGNC:74865]	-1.296503956	1.46E-08	1.34E-07
ENSMMUG00000062213	Metazoan signal recognition particle RNA [Source:RFAM;Acc:RF00017]	-0.760237345	0.015003164	0.038262583

ENSMMUG00000057747		-1.742048564	0.000380634	0.001523976
ENSMMUG00000064131		-2.10840814	7.12E-12	1.04E-10
ENSMMUG00000027108	mml-mir-193b [Source:miRBase;Acc:MI0007656]	-3.366705092	5.76E-07	4.08E-06
ENSMMUG00000052203		-2.04614549	0.01540743	0.039098594
ENSMMUG00000054410	group 10 secretory phospholipase A2 [Source:NCBI gene;Acc:717517]	-0.943048683	0.004172082	0.012702109
ENSMMUG00000064246		0.80748612	0.0037504	0.011541489
ENSMMUG00000062791		-2.150891895	1.51E-07	1.18E-06
ENSMMUG00000062623	Metazoan signal recognition particle RNA [Source:RFAM;Acc:RF00017]	-1.137037978	2.71E-06	1.70E-05
ENSMMUG00000058016	bMERB domain containing 1 [Source:NCBI gene;Acc:713757]	-1.503470287	4.60E-11	6.10E-10
ENSMMUG00000010367	myosin heavy chain 11 [Source:VGNC Symbol;Acc:VGNC:75097]	0.808538993	1.44E-08	1.31E-07
ENSMMUG00000060748	U6 spliceosomal RNA [Source:RFAM;Acc:RF00026]	1.480802049	0.017561303	0.043719007
ENSMMUG00000017131	xylosyltransferase 1 [Source:VGNC Symbol;Acc:VGNC:78977]	-0.650655516	0.018527189	0.045747289
ENSMMUG00000013329	transmembrane channel like 5 [Source:VGNC Symbol;Acc:VGNC:79366]	1.116533726	0.006805075	0.019392632
ENSMMUG00000002709	G protein-coupled receptor class C group 5 member B [Source:VGNC Symbol;Acc:VGNC:72934]	-0.908974695	3.43E-08	2.95E-07
ENSMMUG00000055853	G protein-coupled receptor 139 [Source:NCBI gene;Acc:695541]	-0.992928123	0.015994849	0.040395709
ENSMMUG00000016753	dynein axonemal heavy chain 3 [Source:VGNC Symbol;Acc:VGNC:71941]	0.868510276	0.00503797	0.014945609
ENSMMUG00000016758	transmembrane protein 159 [Source:VGNC Symbol;Acc:VGNC:79836]	0.900785639	8.44E-07	5.80E-06
ENSMMUG00000016759	zona pellucida glycoprotein 2 [Source:VGNC Symbol;Acc:VGNC:78914]	0.57431529	0.009636503	0.026202666
ENSMMUG00000062042	ankyrin repeat and sterile alpha motif domain containing 4B [Source:NCBI gene;Acc:697713]	1.131611511	0.002214474	0.007254395
ENSMMUG00000064659		0.707898608	0.00071777	0.002671821
ENSMMUG00000017725	eukaryotic elongation factor 2 kinase [Source:VGNC Symbol;Acc:VGNC:72098]	-0.69213822	4.07E-07	2.94E-06
ENSMMUG00000002257	RNA polymerase III subunit E [Source:VGNC Symbol;Acc:VGNC:76142]	-0.564119677	3.83E-05	0.000190352
ENSMMUG00000022035	cerebellar degeneration related protein 2 [Source:VGNC Symbol;Acc:VGNC:70993]	-1.044134715	0.000266124	0.001103023
ENSMMUG00000058042	heparan sulfate-glucosamine 3-sulfotransferase 2 [Source:NCBI gene;Acc:699722]	1.762022935	9.83E-21	3.78E-19
ENSMMUG00000014271	protein kinase C beta [Source:VGNC Symbol;Acc:VGNC:76410]	0.571858519	2.75E-05	0.000141764
ENSMMUG00000054064		-1.396649659	1.21E-08	1.12E-07
ENSMMUG00000013560	calcium voltage-gated channel auxiliary subunit gamma 3 [Source:VGNC Symbol;Acc:VGNC:70513]	0.975398866	1.82E-11	2.52E-10
ENSMMUG00000023891		2.746713233	0.004921717	0.014656439
ENSMMUG00000057850		1.192798905	2.07E-05	0.000109541
ENSMMUG00000057160		1.424972499	9.89E-06	5.55E-05
ENSMMUG00000015908	RB binding protein 6, ubiquitin ligase [Source:VGNC Symbol;Acc:VGNC:76677]	1.180420652	7.24E-10	8.14E-09
ENSMMUG00000001407	solute carrier family 5 member 11 [Source:VGNC Symbol;Acc:VGNC:77759]	-0.740898529	3.29E-12	5.08E-11
ENSMMUG00000029176	Rho GTPase activating protein 17 [Source:NCBI gene;Acc:701988]	-0.540663038	3.83E-05	0.000190243
ENSMMUG00000005942	leucine carboxyl methyltransferase 1 [Source:VGNC Symbol;Acc:VGNC:74067]	-0.688177473	1.32E-08	1.22E-07
ENSMMUG00000015570	KIAA0556 [Source:VGNC Symbol;Acc:VGNC:73927]	0.535420594	0.006317318	0.018187891
ENSMMUG00000015572	GSG1 like [Source:VGNC Symbol;Acc:VGNC:82152]	1.402339708	7.63E-12	1.11E-10
ENSMMUG00000018623	exportin 6 [Source:NCBI gene;Acc:704332]	0.906947941	0.005405036	0.015869469
ENSMMUG00000051475		0.695176016	0.015385905	0.039050655
ENSMMUG00000023526	ATPase sarcoplasmic/endoplasmic reticulum Ca2+ transporting 1 [Source:NCBI gene;Acc:708058]	1.951849553	0.006301848	0.018146876

ENSMMUG00000003303	Tu translation elongation factor, mitochondrial [Source:VGNC Symbol;Acc:VGNC:78674]	2.134218182	2.07E-35	3.57E-33
ENSMMUG00000003298	ataxin 2 like [Source:VGNC Symbol;Acc:VGNC:70159]	2.008927612	4.71E-33	6.41E-31
ENSMMUG00000011798	eukaryotic translation initiation factor 3 subunit C [Source:NCBI gene;Acc:705696]	2.128817046	2.95E-32	3.53E-30
ENSMMUG00000000700	CLN3 lysosomal/endosomal transmembrane protein, battenin [Source:NCBI gene;Acc:705815]	1.91125158	3.63E-21	1.47E-19
ENSMMUG00000011370	SAGA complex associated factor 29 [Source:NCBI gene;Acc:709124]	-2.767516921	0.011770426	0.03117061
ENSMMUG00000022330	sulfotransferase family, cytosolic, 1A, phenol-preferring, member 3 [Source:NCBI gene;Acc:100428398]	-2.482624085	0.000270119	0.001116688
ENSMMUG00000017758	mitogen-activated protein kinase 3 [Source:VGNC Symbol;Acc:VGNC:74646]	1.854535231	1.02E-22	4.89E-21
ENSMMUG00000029164	glycerophosphodiester phosphodiesterase domain containing 3 [Source:VGNC Symbol;Acc:VGNC:72800]	-2.826094455	8.55E-11	1.09E-09
ENSMMUG00000000981	T-box transcription factor 6 [Source:VGNC Symbol;Acc:VGNC:78276]	-1.704603205	1.83E-08	1.65E-07
ENSMMUG00000000982	protein phosphatase 4 catalytic subunit [Source:VGNC Symbol;Acc:VGNC:76384]	-1.722741966	4.16E-08	3.52E-07
ENSMMUG00000000983	aldolase, fructose-bisphosphate A [Source:NCBI gene;Acc:708406]	-1.258723464	4.38E-07	3.15E-06
ENSMMUG00000020277	double C2 domain alpha [Source:VGNC Symbol;Acc:VGNC:71963]	-0.642700357	3.93E-07	2.85E-06
ENSMMUG00000020274	TAO kinase 2 [Source:VGNC Symbol;Acc:VGNC:81592]	-0.456377047	0.000327191	0.001328652
ENSMMUG00000018992	aspartate beta-hydroxylase domain containing 1 [Source:VGNC Symbol;Acc:VGNC:70138]	1.471029625	0.008110736	0.022609438
ENSMMUG00000018986	seizure related 6 homolog like 2 [Source:VGNC Symbol;Acc:VGNC:81567]	1.544165894	3.83E-09	3.84E-08
ENSMMUG00000061749	PAXIP1 associated glutamate rich protein 1 [Source:NCBI gene;Acc:706839]	0.895874464	2.91E-10	3.45E-09
ENSMMUG00000019826	MYC associated zinc finger protein [Source:NCBI gene;Acc:708982]	0.82232412	4.53E-06	2.72E-05
ENSMMUG00000009684	kinesin family member 22 [Source:VGNC Symbol;Acc:VGNC:74033]	1.820243022	1.93E-12	3.08E-11
ENSMMUG00000021210	chromosome 20 C16orf54 homolog [Source:VGNC Symbol;Acc:VGNC:70402]	-1.2277325	0.017303283	0.043156354
ENSMMUG00000016143	sialophorin [Source:VGNC Symbol;Acc:VGNC:77861]	2.18374603	1.17E-26	8.49E-25
ENSMMUG00000021844	CD2 cytoplasmic tail binding protein 2 [Source:NCBI gene;Acc:709961]	-0.486575922	0.000715372	0.002664899
ENSMMUG00000016879	zinc finger protein 768 [Source:VGNC Symbol;Acc:VGNC:82280]	1.996885705	0.016961812	0.04248331
ENSMMUG00000001121	zinc finger protein 689 [Source:VGNC Symbol;Acc:VGNC:79480]	-1.762136771	8.11E-10	9.01E-09
ENSMMUG00000051226	FAD-linked sulfhydryl oxidase ERV1 [Source:NCBI gene;Acc:106994988]	1.287447693	7.19E-17	1.83E-15
ENSMMUG00000010556	Snf2 related CREBBP activator protein [Source:NCBI gene;Acc:712944]	0.885959565	4.35E-12	6.56E-11
ENSMMUG00000047302	cardiotrophin 1 [Source:NCBI gene;Acc:713850]	0.944094359	0.007135955	0.020191988
ENSMMUG00000003230	F-box and leucine rich repeat protein 19 [Source:VGNC Symbol;Acc:VGNC:81406]	-0.58326879	0.00176974	0.005976562
ENSMMUG00000003233	SET domain containing 1A, histone lysine methyltransferase [Source:NCBI gene;Acc:712416]	0.799955446	5.99E-06	3.51E-05
ENSMMUG00000048207	syntaxin 4 [Source:NCBI gene;Acc:704880]	1.123754364	4.72E-12	7.09E-11
ENSMMUG00000002033	serine protease 36 [Source:VGNC Symbol;Acc:VGNC:76325]	1.138769568	4.00E-13	6.93E-12
ENSMMUG00000016276	tripartite motif containing 72 [Source:VGNC Symbol;Acc:VGNC:79927]	0.982965957	0.000145992	0.000640553
ENSMMUG00000014349	integrin subunit alpha X [Source:NCBI gene;Acc:100427850]	2.607144045	3.04E-17	8.07E-16
ENSMMUG00000000901	alpha hemoglobin stabilizing protein [Source:VGNC Symbol;Acc:VGNC:69631]	0.693867607	0.000416558	0.001652194
ENSMMUG00000058907		2.448512963	7.67E-10	8.58E-09
ENSMMUG00000055973	phosphoglycerate mutase 1-like [Source:NCBI gene;Acc:714548]	2.952457262	1.21E-07	9.65E-07
ENSMMUG00000008253	zinc finger protein 267 [Source:NCBI gene;Acc:641441]	0.872917052	6.26E-05	0.000298216
ENSMMUG00000048906	zinc finger protein 91-like [Source:NCBI gene;Acc:716072]	-0.814125196	0.018941153	0.046629699
ENSMMUG00000003288	glutamic--pyruvic transaminase 2 [Source:VGNC Symbol;Acc:VGNC:72962]	-1.302848847	8.47E-11	1.08E-09
ENSMMUG00000019868	NKD inhibitor of WNT signaling pathway 1 [Source:VGNC Symbol;Acc:VGNC:75198]	-1.703630691	8.92E-05	0.000411529
ENSMMUG00000019869	sorting nexin 20 [Source:VGNC Symbol;Acc:VGNC:77796]	-1.576795162	8.90E-05	0.000411109

ENSMMUG00000012209	CYLD lysine 63 deubiquitinase [Source:VGNC Symbol;Acc:VGNC:71581]	-1.599121318	7.14E-13	1.21E-11
ENSMMUG00000055889		-1.908352322	4.16E-07	3.00E-06
ENSMMUG00000064095		4.378623289	3.06E-10	3.62E-09
ENSMMUG00000013967	TOX high mobility group box family member 3 [Source:VGNC Symbol;Acc:VGNC:78612]	2.774404187	1.19E-15	2.67E-14
ENSMMUG00000050057		1.39631595	1.70E-12	2.73E-11
ENSMMUG00000058254		1.370771309	2.27E-09	2.36E-08
ENSMMUG00000020583	FTO alpha-ketoglutarate dependent dioxygenase [Source:VGNC Symbol;Acc:VGNC:72772]	1.071779218	8.93E-08	7.24E-07
ENSMMUG00000050851		2.995056028	0.018261958	0.045212827
ENSMMUG00000054811		2.659461165	0.000645585	0.002438
ENSMMUG00000056555		1.662075476	1.66E-14	3.38E-13
ENSMMUG00000063965		-0.547118635	0.012285613	0.032327118
ENSMMUG00000061439		-1.029188125	0.004881156	0.014561968
ENSMMUG00000023525	cadherin 5 [Source:VGNC Symbol;Acc:VGNC:70973]	-0.481542948	0.000743758	0.002756794
ENSMMUG00000029108	CKLF like MARVEL transmembrane domain containing 1 [Source:HGNC Symbol;Acc:HGNC:19172]	0.491727834	0.002892859	0.009210198
ENSMMUG00000049484		-0.904027485	1.39E-06	9.21E-06
ENSMMUG00000020847	CKLF like MARVEL transmembrane domain containing 3 [Source:VGNC Symbol;Acc:VGNC:71215]	-1.528046381	1.85E-06	1.20E-05
ENSMMUG00000049583	RRAD, Ras related glycolysis inhibitor and calcium channel regulator [Source:NCBI gene;Acc:695086]	0.303253744	0.004805764	0.014371593
ENSMMUG00000022825	carboxylesterase 2 [Source:VGNC Symbol;Acc:VGNC:71012]	0.351012881	0.000189302	0.00081152
ENSMMUG00000064237		-1.043203948	6.16E-06	3.60E-05
ENSMMUG00000054948		1.239975449	0.006822677	0.019439059
ENSMMUG00000058815	core-binding factor subunit beta [Source:NCBI gene;Acc:695767]	0.942303273	0.001181131	0.004181468
ENSMMUG00000018102	chromosome 20 C16orf70 homolog [Source:VGNC Symbol;Acc:VGNC:70438]	0.895456207	0.001567293	0.005354286
ENSMMUG00000005906	heat shock transcription factor 4 [Source:VGNC Symbol;Acc:VGNC:73533]	0.84942013	2.31E-05	0.000120908
ENSMMUG00000020266	KIAA0895 like [Source:VGNC Symbol;Acc:VGNC:73952]	1.526356035	0.002346005	0.00764307
ENSMMUG00000020254	exocyst complex component 3 like 1 [Source:VGNC Symbol;Acc:VGNC:72376]	1.401616709	3.43E-12	5.27E-11
ENSMMUG00000020258	E2F transcription factor 4 [Source:VGNC Symbol;Acc:VGNC:72025]	1.1470692	3.59E-08	3.07E-07
ENSMMUG00000020259	engulfment and cell motility 3 [Source:VGNC Symbol;Acc:VGNC:72060]	1.159512462	0.009531198	0.025954401
ENSMMUG00000018111	formin homology 2 domain containing 1 [Source:VGNC Symbol;Acc:VGNC:72523]	-1.412596952	0.005856769	0.017021144
ENSMMUG00000054637	tubulin polymerization promoting protein family member 3 [Source:NCBI gene;Acc:100429881]	1.920057825	0.001003317	0.00361233
ENSMMUG00000009825	zinc finger DHHC-type containing 1 [Source:VGNC Symbol;Acc:VGNC:79102]	1.226787821	3.02E-15	6.56E-14
ENSMMUG00000029105	ATPase H+ transporting V0 subunit d1 [Source:NCBI gene;Acc:698345]	0.655835728	0.012099394	0.03189371
ENSMMUG00000057511	Metazoan signal recognition particle RNA [Source:RFAM;Acc:RF00017]	0.681915573	0.004629858	0.013910785
ENSMMUG00000017105	RHO family interacting cell polarization regulator 1 [Source:NCBI gene;Acc:699910]	-0.451975591	2.23E-05	0.000117033
ENSMMUG00000009799	capping protein regulator and myosin 1 linker 2 [Source:VGNC Symbol;Acc:VGNC:70647]	-2.513649085	0.012816405	0.033489509
ENSMMUG00000007803	translin associated factor X interacting protein 1 [Source:VGNC Symbol;Acc:VGNC:79151]	-0.380916546	0.013334259	0.034637775
ENSMMUG00000037975	solute carrier family 7 member 6 [Source:NCBI gene;Acc:702903]	-0.911237631	0.00066115	0.00249044
ENSMMUG00000059872		-1.480540764	0.002609382	0.008392351
ENSMMUG00000010393	cadherin 3 [Source:VGNC Symbol;Acc:VGNC:70971]	0.663423473	2.80E-10	3.33E-09
ENSMMUG00000022177	transport and golgi organization 6 homolog [Source:NCBI gene;Acc:704499]	-1.389798033	0.004664162	0.01399343

ENSMMUG00000010925	chromosome transmission fidelity protein 8 homolog [Source:NCBI gene;Acc:114669750]	-2.557411009	0.009178116	0.025147631
ENSMMUG00000012039	cytochrome b5 type B (outer mitochondrial membrane) [Source:NCBI gene;Acc:705093]	4.271432149	3.12E-06	1.93E-05
ENSMMUG00000018255	nuclear factor of activated T cells 5 [Source:VGNC Symbol;Acc:VGNC:75325]	0.988695396	4.28E-10	4.96E-09
ENSMMUG00000019684	NAD(P)H quinone dehydrogenase 1 [Source:NCBI gene;Acc:705635]	0.691052541	1.48E-05	8.10E-05
ENSMMUG00000000680	polyamine modulated factor 1 binding protein 1 [Source:VGNC Symbol;Acc:VGNC:76052]	1.196463135	0.015552256	0.039418896
ENSMMUG00000049699	thioredoxin like 4B [Source:NCBI gene;Acc:706840]	-1.210424103	0.000169764	0.000733139
ENSMMUG00000023312	haptoglobin [Source:NCBI gene;Acc:706640]	-1.288065973	0.000316726	0.001290398
ENSMMUG00000016716	zinc finger protein 821 [Source:VGNC Symbol;Acc:VGNC:79679]	-0.633701369	0.002050687	0.006801987
ENSMMUG00000048077	ataxin 1 like [Source:NCBI gene;Acc:709805]	-1.125704559	6.37E-09	6.17E-08
ENSMMUG00000012007	PH domain and leucine rich repeat protein phosphatase 2 [Source:VGNC Symbol;Acc:VGNC:81494]	0.884821602	0.000698383	0.002611452
ENSMMUG00000052324	MARVEL domain containing 3 [Source:NCBI gene;Acc:710448]	1.006970052	0.000152505	0.000666563
ENSMMUG00000002018	tyrosine aminotransferase [Source:VGNC Symbol;Acc:VGNC:78100]	-0.4785187	0.00094098	0.003402764
ENSMMUG00000061967	carbohydrate sulfotransferase 4 [Source:NCBI gene;Acc:708116]	-0.896519013	0.003117263	0.009821331
ENSMMUG00000023147	pyruvate dehydrogenase phosphatase regulatory subunit [Source:VGNC Symbol;Acc:VGNC:75897]	1.580804134	0.000530125	0.002048399
ENSMMUG00000005751	adenosine deaminase tRNA specific 1 [Source:VGNC Symbol;Acc:VGNC:69504]	-2.183645356	0.000100862	0.000460951
ENSMMUG00000005754	lysyl-tRNA synthetase 1 [Source:NCBI gene;Acc:714516]	-1.421754291	0.00023916	0.001002471
ENSMMUG00000055178	double homeobox B [Source:NCBI gene;Acc:114674350]	-1.757333793	0.009889463	0.026787348
ENSMMUG00000003916	contactin associated protein family member 4 [Source:VGNC Symbol;Acc:VGNC:71209]	-1.77629751	0.000136048	0.00060119
ENSMMUG00000053231		-0.946180777	4.21E-05	0.000207529
ENSMMUG00000037756	MAF bZIP transcription factor [Source:NCBI gene;Acc:106995090]	0.638502567	0.000513504	0.001992483
ENSMMUG00000005334	phospholipase C gamma 2 [Source:VGNC Symbol;Acc:VGNC:76170]	-0.858866527	1.88E-10	2.30E-09
ENSMMUG00000062047		-1.000143522	0.001593594	0.005430378
ENSMMUG00000053215		0.808604317	0.00122773	0.004326813
ENSMMUG00000057329		2.541006061	7.53E-11	9.67E-10
ENSMMUG00000011244	kelch like family member 36 [Source:NCBI gene;Acc:715423]	3.161654422	0.004006534	0.012253435
ENSMMUG00000011893	KIAA0513 [Source:VGNC Symbol;Acc:VGNC:73925]	-1.398300669	4.58E-08	3.87E-07
ENSMMUG00000011894	CBY1 interacting BAR domain containing 2 [Source:VGNC Symbol;Acc:VGNC:72335]	-1.220616227	6.64E-07	4.66E-06
ENSMMUG00000061995		1.045624284	1.04E-12	1.71E-11
ENSMMUG00000052120		-2.52487217	0.000184978	0.000794215
ENSMMUG00000020592	Gse1 coiled-coil protein [Source:VGNC Symbol;Acc:VGNC:73254]	-1.271687394	0.000144571	0.000634504
ENSMMUG00000006763	cytochrome c oxidase subunit IV isoform 1 [Source:NCBI gene;Acc:693811]	0.89652376	4.94E-10	5.66E-09
ENSMMUG00000019500	methenyltetrahydrofolate synthetase domain containing [Source:VGNC Symbol;Acc:VGNC:75023]	0.808269415	0.000329013	0.001334954
ENSMMUG00000060536		-1.701858562	0.00027436	0.001132414
ENSMMUG00000017615	zinc finger CCHC-type containing 14 [Source:VGNC Symbol;Acc:VGNC:79420]	-1.371374195	4.94E-29	4.47E-27
ENSMMUG00000022867	kelch domain containing 4 [Source:VGNC Symbol;Acc:VGNC:74122]	0.705551664	5.07E-05	0.000246579
ENSMMUG00000058437		2.177393404	7.84E-29	6.88E-27
ENSMMUG00000052851	solute carrier family 7 member 5 [Source:NCBI gene;Acc:695886]	1.705251029	1.63E-11	2.26E-10

ENSMMUG00000054935		-1.382059216	0.002359695	0.007684296
ENSMMUG00000008945	zinc finger protein 407 [Source:NCBI gene;Acc:695303]	-1.897366045	0.001557303	0.00532752
ENSMMUG00000008942	carnosine dipeptidase 1 [Source:VGNC Symbol;Acc:VGNC:71257]	-2.023233602	3.62E-05	0.000181655
ENSMMUG00000058864		-2.257874157	0.011173003	0.029785301
ENSMMUG00000015702	divergent protein kinase domain 1C [Source:NCBI gene;Acc:699537]	-2.12174843	0.002325435	0.007581052
ENSMMUG00000020905	chromosome 18 C18orf63 homolog [Source:VGNC Symbol;Acc:VGNC:70465]	-2.36766145	0.001956286	0.00652976
ENSMMUG00000023298	F-box protein 15 [Source:VGNC Symbol;Acc:VGNC:72504]	-2.192180585	0.001912376	0.00640482
ENSMMUG00000056477		0.533789515	0.00180635	0.006085456
ENSMMUG00000050516		0.503941346	0.002074132	0.006868482
ENSMMUG00000060966		-2.863087932	3.17E-05	0.000160843
ENSMMUG00000045074		-2.814332451	0.018655178	0.046002044
ENSMMUG00000047095	serpin family B member 13 [Source:NCBI gene;Acc:701078]	-1.788135723	0.011624867	0.030834696
ENSMMUG00000057978		-1.821257469	0.003666789	0.011307649
ENSMMUG00000001245	zinc finger protein 532 [Source:NCBI gene;Acc:701146]	-5.004864317	1.62E-08	1.46E-07
ENSMMUG00000061572	Metazoan signal recognition particle RNA [Source:RFAM;Acc:RF00017]	-2.632477663	1.15E-06	7.73E-06
ENSMMUG00000065131		-2.512753038	0.004896754	0.01459969
ENSMMUG00000058948		-0.731808472	1.74E-05	9.40E-05
ENSMMUG00000055751		1.35282638	0.000134254	0.000594679
ENSMMUG00000012001	transcription factor 4 [Source:VGNC Symbol;Acc:VGNC:78288]	0.885277269	0.000103568	0.000471429
ENSMMUG00000020898	cap binding complex dependent translation initiation factor [Source:HGNC Symbol;Acc:HGNC:23925]	-1.5925568	1.78E-06	1.15E-05
ENSMMUG00000007816	SMAD family member 2 [Source:VGNC Symbol;Acc:VGNC:77810]	-3.543594688	3.93E-20	1.39E-18
ENSMMUG00000053364		1.059090419	2.79E-07	2.08E-06
ENSMMUG00000058218		0.806304935	0.015890914	0.04020168
ENSMMUG00000015952	katanin catalytic subunit A1 like 2 [Source:VGNC Symbol;Acc:VGNC:73904]	1.941543666	1.29E-05	7.13E-05
ENSMMUG00000015951	elongin A2 [Source:NCBI gene;Acc:702788]	2.546620636	0.015330858	0.038957612
ENSMMUG00000044172	elongin-A3-like [Source:NCBI gene;Acc:106994494]	1.649777987	0.016768248	0.042105168
ENSMMUG00000043738		2.188060972	7.44E-05	0.000348442
ENSMMUG00000004498	protein inhibitor of activated STAT 2 [Source:VGNC Symbol;Acc:VGNC:75815]	1.903197273	5.81E-09	5.65E-08
ENSMMUG00000002742	ST8 alpha-N-acetyl-neuraminide alpha-2,8-sialyltransferase 5 [Source:VGNC Symbol;Acc:VGNC:77942]	-2.977663197	1.01E-32	1.32E-30
ENSMMUG00000017246	lipoxygenase homology domains 1 [Source:VGNC Symbol;Acc:VGNC:74368]	-2.343939401	1.65E-20	6.19E-19
ENSMMUG00000053435		-1.867548976	0.000339627	0.00137426
ENSMMUG00000017243	ring finger protein 165 [Source:VGNC Symbol;Acc:VGNC:76877]	-0.972535315	0.000720749	0.002681561
ENSMMUG00000064342		-2.164510404	0.01565566	0.03966065
ENSMMUG00000049194		-1.153969456	0.012697717	0.033228717

ENSMMUG00000006864	KIAA1328 ortholog [Source:NCBI gene;Acc:709850]	-1.824386902	0.013004204	0.033899163
ENSMMUG00000018791	molybdenum cofactor sulfurase [Source:VGNC Symbol;Acc:VGNC:74792]	-1.113444099	7.62E-06	4.38E-05
ENSMMUG00000018784	solute carrier family 39 member 6 [Source:VGNC Symbol;Acc:VGNC:77508]	-0.909901524	3.80E-14	7.37E-13
ENSMMUG00000018785	elongator acetyltransferase complex subunit 2 [Source:VGNC Symbol;Acc:VGNC:72069]	-1.038967816	2.10E-09	2.20E-08
ENSMMUG00000018777	regulation of nuclear pre-mRNA domain containing 1A [Source:VGNC Symbol;Acc:VGNC:77103]	-0.750535355	0.000710213	0.002649009
ENSMMUG00000003302	dystrobrevin alpha [Source:VGNC Symbol;Acc:VGNC:72007]	-1.395350058	8.82E-05	0.000407753
ENSMMUG00000017276	nucleolar protein 4 [Source:VGNC Symbol;Acc:VGNC:75366]	-0.835820822	5.75E-05	0.000275918
ENSMMUG00000014451	ASXL transcriptional regulator 3 [Source:VGNC Symbol;Acc:VGNC:70152]	-1.026832309	1.07E-05	5.98E-05
ENSMMUG00000052277	transthyretin [Source:NCBI gene;Acc:705943]	-1.637001077	0.00068486	0.002569956
ENSMMUG00000021095	desmoglein 2 [Source:VGNC Symbol;Acc:VGNC:72002]	-1.892563958	0.005090955	0.015072643
ENSMMUG00000049366	Metazoan signal recognition particle RNA [Source:RFAM;Acc:RF00017]	-1.786512804	0.011813078	0.031255656
ENSMMUG00000048746		1.033464841	0.001103454	0.003934654
ENSMMUG00000060604		0.762683597	0.016422667	0.041335395
ENSMMUG00000057475		-2.030624625	9.53E-06	5.37E-05
ENSMMUG00000009669	cutaneous T cell lymphoma-associated antigen 1 [Source:NCBI gene;Acc:106994443]	-2.024204887	1.43E-06	9.40E-06
ENSMMUG00000061690		-2.15461127	0.00039872	0.001588241
ENSMMUG00000010944	GATA binding protein 6 [Source:VGNC Symbol;Acc:VGNC:72891]	-1.190285591	8.11E-06	4.63E-05
ENSMMUG00000064806		-1.365907161	1.50E-06	9.88E-06
ENSMMUG00000004382	abhydrolase domain containing 3 [Source:VGNC Symbol;Acc:VGNC:69464]	-1.641422513	0.004921188	0.014656439
ENSMMUG00000051922	small nuclear ribonucleoprotein D1 polypeptide [Source:NCBI gene;Acc:698965]	-3.140219246	0.016090206	0.040615787
ENSMMUG00000003974	GREB1 like retinoic acid receptor coactivator [Source:NCBI gene;Acc:700655]	-2.09440017	0.014756068	0.037742754
ENSMMUG00000019892	myomesin 1 [Source:VGNC Symbol;Acc:VGNC:75123]	-1.646428751	8.70E-17	2.20E-15
ENSMMUG00000052605	Metazoan signal recognition particle RNA [Source:RFAM;Acc:RF00017]	-2.891520803	5.25E-06	3.11E-05
ENSMMUG00000005086	homeobox protein TGIF1 [Source:NCBI gene;Acc:694580]	1.09105523	2.10E-05	0.00011103
ENSMMUG00000001471	DLG associated protein 1 [Source:NCBI gene;Acc:694049]	-0.491164165	0.002017439	0.006706707
ENSMMUG00000019418	erythrocyte membrane protein band 4.1 like 3 [Source:VGNC Symbol;Acc:VGNC:72233]	2.282227873	3.37E-30	3.31E-28
ENSMMUG00000016302	L3MBTL histone methyl-lysine binding protein 4 [Source:VGNC Symbol;Acc:VGNC:74133]	0.645687843	0.000248591	0.00103848
ENSMMUG00000015761	microtubule crosslinking factor 1 [Source:VGNC Symbol;Acc:VGNC:74852]	-0.423308138	0.007188302	0.020320724
ENSMMUG00000053008		-1.687874526	1.12E-08	1.05E-07
ENSMMUG00000049523	ankyrin repeat domain 12 [Source:NCBI gene;Acc:114673693]	-1.442087332	7.18E-07	5.00E-06
ENSMMUG00000023717	MIER family member 2 [Source:VGNC Symbol;Acc:VGNC:74855]	1.747596432	2.63E-16	6.33E-15
ENSMMUG00000059175		1.979589448	2.94E-10	3.48E-09
ENSMMUG00000053083	C2 calcium dependent domain containing 4C [Source:NCBI gene;Acc:100424440]	2.143086005	3.25E-06	2.00E-05

ENSMMUG00000045966	granzyme M [Source:NCBI gene;Acc:715031]	-1.598164409	0.000838192	0.003070748
ENSMMUG00000012768	hyperpolarization activated cyclic nucleotide gated potassium and sodium channel 2 [Source:NCBI gene;Acc:100425114]	-1.112424453	0.000677529	0.002543734
ENSMMUG00000049968	RNA polymerase mitochondrial [Source:NCBI gene;Acc:100426183]	-1.271709028	3.09E-19	9.85E-18
ENSMMUG00000055423	fibroblast growth factor 22 [Source:NCBI gene;Acc:702727]	-0.409634749	0.002377663	0.007736012
ENSMMUG00000008672	ring finger protein 126 [Source:VGNC Symbol;Acc:VGNC:76791]	-0.80012517	9.82E-05	0.000449512
ENSMMUG00000056220	follistatin like 3 [Source:NCBI gene;Acc:721100]	-0.886030266	0.013508947	0.035036644
ENSMMUG00000016450	polypyrimidine tract binding protein 1 [Source:VGNC Symbol;Acc:VGNC:76462]	-0.39567465	0.016777938	0.042115238
ENSMMUG00000028948	azurocidin 1 [Source:VGNC Symbol;Acc:VGNC:70202]	2.394709832	1.31E-07	1.04E-06
ENSMMUG00000061589	proteinase 3 [Source:HGNC Symbol;Acc:HGNC:9495]	2.709654939	0.000694599	0.002600556
ENSMMUG00000009674	elastase, neutrophil expressed [Source:VGNC Symbol;Acc:VGNC:72199]	2.27379991	0.000557634	0.002141276
ENSMMUG00000028947	complement factor D [Source:VGNC Symbol;Acc:VGNC:71115]	2.142057297	9.83E-24	5.28E-22
ENSMMUG00000009682	R3H domain containing 4 [Source:VGNC Symbol;Acc:VGNC:76554]	1.167845499	0.000320344	0.001304049
ENSMMUG00000002278	AT-rich interaction domain 3A [Source:VGNC Symbol;Acc:VGNC:69930]	1.585196714	1.74E-08	1.57E-07
ENSMMUG00000058621		1.685328636	1.72E-16	4.20E-15
ENSMMUG00000002638	WD repeat domain 18 [Source:VGNC Symbol;Acc:VGNC:79103]	-0.78222082	0.005053791	0.014986547
ENSMMUG00000048135	glutamate ionotropic receptor NMDA type subunit 3B [Source:NCBI gene;Acc:721155]	-0.4491836	0.002229314	0.007299784
ENSMMUG00000013577	transmembrane protein 259 [Source:NCBI gene;Acc:721161]	-0.409311266	0.002021645	0.006717677
ENSMMUG00000015306	calponin 2 [Source:NCBI gene;Acc:721164]	1.069838583	5.73E-11	7.48E-10
ENSMMUG00000015303	ATP binding cassette subfamily A member 7 [Source:VGNC Symbol;Acc:VGNC:69567]	0.940893037	1.19E-06	7.99E-06
ENSMMUG00000058856	Metazoan signal recognition particle RNA [Source:RFAM;Acc:RF00017]	1.114664975	0.003908152	0.011969825
ENSMMUG00000010886	strawberry notch homolog 2 [Source:VGNC Symbol;Acc:VGNC:77123]	1.822064931	3.46E-05	0.000174299
ENSMMUG00000058928	Metazoan signal recognition particle RNA [Source:RFAM;Acc:RF00017]	2.832862446	7.10E-26	4.54E-24
ENSMMUG00000022100	midnolin [Source:VGNC Symbol;Acc:VGNC:82186]	1.872699342	5.50E-30	5.29E-28
ENSMMUG00000003043	PWWP domain containing 3A, DNA repair factor [Source:VGNC Symbol;Acc:VGNC:76565]	-1.138224781	2.09E-12	3.31E-11
ENSMMUG00000003053	APC regulator of WNT signaling pathway 2 [Source:VGNC Symbol;Acc:VGNC:69888]	-1.166509973	2.18E-05	0.00011467
ENSMMUG00000009014	methyl-CpG binding domain protein 3 [Source:VGNC Symbol;Acc:VGNC:81470]	-0.786325865	0.00754172	0.021202598
ENSMMUG00000016539	abhydrolase domain containing 17A [Source:VGNC Symbol;Acc:VGNC:69543]	-0.756219294	0.000701362	0.002621931
ENSMMUG00000013747	secretory carrier membrane protein 4 [Source:VGNC Symbol;Acc:VGNC:77135]	-0.531804782	0.000165889	0.000718495
ENSMMUG00000006620	casein kinase 1 gamma 2 [Source:NCBI gene;Acc:709484]	0.783583571	0.000653213	0.002463043
ENSMMUG00000059725		0.864765303	0.009929867	0.026875954
ENSMMUG00000015660	IZUMO family member 4 [Source:VGNC Symbol;Acc:VGNC:73665]	0.923474883	0.017104669	0.042773913
ENSMMUG00000015663	adaptor related protein complex 3 subunit delta 1 [Source:VGNC Symbol;Acc:VGNC:69969]	0.928073218	2.10E-07	1.60E-06
ENSMMUG00000017949	ornithine decarboxylase antizyme 1 [Source:NCBI gene;Acc:721477]	0.850682096	1.55E-14	3.17E-13
ENSMMUG00000058241	leucine rich repeat and Ig domain containing 3 [Source:NCBI gene;Acc:721484]	1.029409134	0.000504224	0.001960063
ENSMMUG00000064612	Metazoan signal recognition particle RNA [Source:RFAM;Acc:RF00017]	1.067054517	0.000123876	0.00055267
ENSMMUG00000000452	LSM7 homolog, U6 small nuclear RNA and mRNA degradation associated [Source:NCBI gene;Acc:721486]	2.346436014	2.68E-06	1.68E-05
ENSMMUG00000055417		-0.683931941	1.63E-05	8.83E-05
ENSMMUG00000003468	growth arrest and DNA damage inducible beta [Source:VGNC Symbol;Acc:VGNC:72864]	-0.696077957	0.000558807	0.00214414
ENSMMUG00000065054	G protein subunit gamma 7 [Source:NCBI gene;Acc:711335]	1.868136246	1.35E-14	2.77E-13
ENSMMUG00000007268	thimet oligopeptidase 1 [Source:VGNC Symbol;Acc:VGNC:78347]	0.78257858	0.000552432	0.002125179

ENSMMUG00000007485	zinc finger protein 555 [Source:VGNC Symbol;Acc:VGNC:79705]	-0.999617202	1.10E-05	6.11E-05
ENSMMUG00000002912	TLE family member 6, subcortical maternal complex member [Source:VGNC Symbol;Acc:VGNC:79879]	-0.907865566	0.000209497	0.000889446
ENSMMUG00000002961	G protein subunit alpha 11 [Source:VGNC Symbol;Acc:VGNC:73085]	-1.470496668	2.59E-11	3.52E-10
ENSMMUG00000017036	nicalin [Source:VGNC Symbol;Acc:VGNC:75032]	2.237269373	1.35E-17	3.75E-16
ENSMMUG00000015513	CUGBP Elav-like family member 5 [Source:NCBI gene;Acc:721694]	1.970445855	1.51E-06	9.91E-06
ENSMMUG00000060497		2.402763757	1.47E-09	1.57E-08
ENSMMUG00000003439	nuclear factor I C [Source:VGNC Symbol;Acc:VGNC:75336]	1.042787804	0.001002429	0.003610011
ENSMMUG00000018096	high mobility group 20B [Source:NCBI gene;Acc:721723]	1.556353833	0.00648836	0.018615278
ENSMMUG00000028931	cactin, spliceosome C complex subunit [Source:NCBI gene;Acc:721737]	-0.739993536	0.004342793	0.013148963
ENSMMUG00000028930	tight junction protein 3 [Source:NCBI gene;Acc:721760]	-0.49221499	0.007035603	0.019958761
ENSMMUG00000001917	megakaryocyte-associated tyrosine kinase [Source:NCBI gene;Acc:100430078]	0.765372015	0.001832732	0.006168728
ENSMMUG00000039195	ATCAY kinesin light chain interacting caytaxin [Source:NCBI gene;Acc:721783]	-0.906237036	0.001525811	0.005234163
ENSMMUG00000056147		-1.428117436	1.61E-06	1.05E-05
ENSMMUG00000022767	eukaryotic translation elongation factor 2 [Source:NCBI gene;Acc:721806]	-2.233711934	0.00772429	0.021662486
ENSMMUG00000019564	mitogen-activated protein kinase kinase 2 [Source:VGNC Symbol;Acc:VGNC:81453]	-0.543948109	2.56E-05	0.000132984
ENSMMUG00000028928	ankyrin repeat domain 24 [Source:NCBI gene;Acc:721829]	0.517791547	6.80E-05	0.000321035
ENSMMUG00000010100	YJU2 splicing factor homolog [Source:NCBI gene;Acc:718892]	0.986456967	0.012228603	0.032190001
ENSMMUG00000028925	HDGF like 2 [Source:NCBI gene;Acc:693476]	-3.592375693	4.18E-09	4.16E-08
ENSMMUG00000022656	perilipin 4 [Source:NCBI gene;Acc:693610]	-1.807005546	0.005074017	0.015039846
ENSMMUG00000021716	dipeptidyl peptidase 9 [Source:NCBI gene;Acc:695587]	-0.479905375	0.016170174	0.040796815
ENSMMUG00000062995		0.94284257	3.25E-08	2.81E-07
ENSMMUG00000058275	arrestin domain containing 5 [Source:NCBI gene;Acc:697277]	0.877432799	0.000119376	0.000534041
ENSMMUG00000012677	lysine demethylase 4B [Source:VGNC Symbol;Acc:VGNC:73930]	-1.103551549	4.79E-08	4.03E-07
ENSMMUG00000011962	protein tyrosine phosphatase receptor type S [Source:VGNC Symbol;Acc:VGNC:76622]	0.784407137	0.003266683	0.010244219
ENSMMUG00000050702	fucosyltransferase 5 [Source:HGNC Symbol;Acc:HGNC:4016]	0.655458672	3.48E-05	0.000174981
ENSMMUG00000016665	NADH:ubiquinone oxidoreductase subunit A11 [Source:NCBI gene;Acc:697843]	-1.535294641	2.59E-05	0.000134395
ENSMMUG00000011963	vimentin type intermediate filament associated coiled-coil protein [Source:NCBI gene;Acc:697958]	-1.165578924	6.49E-16	1.50E-14
ENSMMUG00000011964	RAN binding protein 3 [Source:VGNC Symbol;Acc:VGNC:76655]	-0.7364733	4.83E-06	2.88E-05
ENSMMUG00000005697	MLLT1 super elongation complex subunit [Source:VGNC Symbol;Acc:VGNC:74881]	0.888173043	0.004712489	0.014126989
ENSMMUG00000063389		3.308077794	0.004300966	0.013040284
ENSMMUG00000005698	alkaline ceramidase 1 [Source:VGNC Symbol;Acc:VGNC:69460]	2.446167278	2.06E-06	1.32E-05
ENSMMUG00000016526	solute carrier family 25 member 41 [Source:VGNC Symbol;Acc:VGNC:77581]	-2.386317941	1.54E-11	2.15E-10
ENSMMUG00000019314	vav guanine nucleotide exchange factor 1 [Source:VGNC Symbol;Acc:VGNC:78757]	2.184649176	0.000102536	0.000467735
ENSMMUG00000001220	adhesion G protein-coupled receptor E1 [Source:VGNC Symbol;Acc:VGNC:69561]	2.217932423	6.35E-23	3.13E-21
ENSMMUG00000063988		1.954080385	2.10E-06	1.34E-05
ENSMMUG00000019966	zinc finger protein 557 [Source:NCBI gene;Acc:100424983]	1.390919727	9.03E-22	3.95E-20
ENSMMUG00000057682		1.471248779	6.86E-22	3.04E-20
ENSMMUG00000028907	insulin receptor [Source:NCBI gene;Acc:706009]	3.238525648	4.86E-21	1.93E-19
ENSMMUG00000028906	Rho/Rac guanine nucleotide exchange factor 18 [Source:NCBI gene;Acc:707510]	1.637634975	0.000602426	0.002290763

ENSMMUG00000011389	mucolipin 1 [Source:VGNC Symbol;Acc:VGNC:74503]	2.21366116	0.002380976	0.007743393
ENSMMUG00000009540	calmodulin regulated spectrin associated protein family member 3 [Source:VGNC Symbol;Acc:VGNC:70669]	-0.563940096	5.44E-05	0.00026266
ENSMMUG00000009539	XPA binding protein 2 [Source:VGNC Symbol;Acc:VGNC:78943]	-0.67196275	2.99E-05	0.000152971
ENSMMUG00000022463	C-type lectin CD209L2 [Source:NCBI gene;Acc:574373]	1.532493045	0.000426369	0.001686792
ENSMMUG00000023303	ecotropic viral integration site 5 like [Source:NCBI gene;Acc:706628]	1.909657972	2.63E-37	5.35E-35
ENSMMUG00000014032	proline rich 36 [Source:NCBI gene;Acc:710443]	5.711938376	4.68E-21	1.87E-19
ENSMMUG00000006540	small nuclear RNA activating complex polypeptide 2 [Source:VGNC Symbol;Acc:VGNC:77666]	0.509275018	0.001569852	0.005359323
ENSMMUG00000010614	C-C motif chemokine ligand 25 [Source:VGNC Symbol;Acc:VGNC:70700]	3.321167446	0.000230517	0.000970632
ENSMMUG00000011178	ceramide synthase 4 [Source:VGNC Symbol;Acc:VGNC:71077]	-0.418265108	0.015933151	0.040274603
ENSMMUG00000016695	CD320 molecule [Source:VGNC Symbol;Acc:VGNC:70763]	-1.375305034	0.000134238	0.000594679
ENSMMUG00000064610	long intergenic non-protein coding RNA 1862 [Source:HGNC Symbol;Acc:HGNC:52681]	0.668176671	0.006713923	0.019162333
ENSMMUG00000049311	zinc finger protein 558 [Source:NCBI gene;Acc:712590]	-0.484723215	0.000108127	0.000489475
ENSMMUG00000019965	methyl-CpG binding domain protein 3 like 1 [Source:VGNC Symbol;Acc:VGNC:74513]	2.186326807	2.78E-05	0.000142988
ENSMMUG00000064532	mucin 16, cell surface associated [Source:NCBI gene;Acc:106996460]	2.183782562	1.60E-31	1.72E-29
ENSMMUG00000007875	F-box and leucine rich repeat protein 12 [Source:VGNC Symbol;Acc:VGNC:72502]	-1.146948675	2.66E-10	3.18E-09
ENSMMUG00000028889	60S ribosomal protein L21 [Source:NCBI gene;Acc:711174]	-0.778544065	0.008081016	0.022535062
ENSMMUG00000037639	complement C3 [Source:NCBI gene;Acc:714514]	0.5674685	0.010025283	0.027100794
ENSMMUG00000017211	peter pan homolog (Drosophila) [Source:NCBI gene;Acc:100533472]	-1.734123536	0.01598077	0.040367028
ENSMMUG00000014897	ribonucleoprotein, PTB binding 1 [Source:NCBI gene;Acc:715250]	0.709576574	0.000662826	0.002495484
ENSMMUG00000003110	tyrosine kinase 2 [Source:VGNC Symbol;Acc:VGNC:78680]	-0.759396291	1.04E-05	5.81E-05
ENSMMUG00000003111	cell division cycle 37 [Source:NCBI gene;Acc:715390]	-0.450581414	0.007266469	0.020502612
ENSMMUG00000005218	kelch like ECH associated protein 1 [Source:VGNC Symbol;Acc:VGNC:73891]	-1.390976715	0.000823613	0.003023311
ENSMMUG00000011951	KRI1 homolog [Source:VGNC Symbol;Acc:VGNC:74127]	1.407903159	4.70E-06	2.81E-05
ENSMMUG00000016593	adaptor related protein complex 1 subunit mu 2 [Source:NCBI gene;Acc:720531]	1.671660683	0.005801515	0.016883445
ENSMMUG00000003744	solute carrier family 44 member 2 [Source:VGNC Symbol;Acc:VGNC:77498]	0.891197253	1.37E-07	1.08E-06
ENSMMUG00000014677	dynamin 2 [Source:VGNC Symbol;Acc:VGNC:71957]	-1.081940934	6.68E-05	0.000316224
ENSMMUG00000055775	transmembrane p24 trafficking protein 1 [Source:NCBI gene;Acc:713618]	1.337259772	8.01E-12	1.16E-10
ENSMMUG00000049703	Metazoan signal recognition particle RNA [Source:RFAM;Acc:RF00017]	1.799909186	2.96E-20	1.07E-18
ENSMMUG00000004400	Yip1 domain family member 2 [Source:VGNC Symbol;Acc:VGNC:79717]	-0.76199361	1.45E-05	7.91E-05
ENSMMUG00000003611	low density lipoprotein receptor [Source:VGNC Symbol;Acc:VGNC:74248]	1.047106865	0.001017465	0.003654397
ENSMMUG00000003612	SPC24 component of NDC80 kinetochore complex [Source:VGNC Symbol;Acc:VGNC:78002]	2.197349309	4.97E-06	2.96E-05
ENSMMUG00000023251	KN motif and ankyrin repeat domains 2 [Source:VGNC Symbol;Acc:VGNC:73938]	-0.525512172	0.014031213	0.036156194
ENSMMUG00000009334	dedicator of cytokinesis 6 [Source:VGNC Symbol;Acc:VGNC:71863]	-0.604401803	0.000581108	0.002220456
ENSMMUG00000043134	coiled-coil domain containing 159 [Source:NCBI gene;Acc:100424702]	2.163345985	3.46E-10	4.05E-09
ENSMMUG00000022207	phospholipid phosphatase related 2 [Source:NCBI gene;Acc:714388]	2.259015419	1.93E-11	2.66E-10
ENSMMUG00000010824	SWIM-type zinc finger 7 associated protein 1 [Source:VGNC Symbol;Acc:VGNC:78220]	2.813383459	6.51E-21	2.54E-19
ENSMMUG00000038989	erythropoietin receptor [Source:NCBI gene;Acc:714546]	2.696836577	4.62E-11	6.11E-10
ENSMMUG00000010826	ral guanine nucleotide dissociation stimulator like 3 [Source:VGNC Symbol;Acc:VGNC:76869]	1.6260494	2.71E-07	2.03E-06
ENSMMUG00000016505	coiled-coil domain containing 151 [Source:VGNC Symbol;Acc:VGNC:70892]	1.213586219	0.016854823	0.042286759
ENSMMUG00000002974	acid phosphatase 5, tartrate resistant [Source:VGNC Symbol;Acc:VGNC:69384]	2.611371925	7.24E-05	0.000340102
ENSMMUG00000001715	zinc finger protein 627 [Source:VGNC Symbol;Acc:VGNC:79856]	1.166726549	3.12E-10	3.67E-09

ENSMMUG00000053329		1.75173683	3.00E-08	2.62E-07
ENSMMUG00000012587	zinc finger protein 69 [Source:NCBI gene;Acc:717030]	-0.750950872	0.000433882	0.001712514
ENSMMUG00000054946	zinc finger protein 433 [Source:NCBI gene;Acc:106992362]	-0.875651818	0.015282663	0.038861779
ENSMMUG00000005236	zinc finger protein 709 [Source:NCBI gene;Acc:717515]	1.486465832	2.44E-07	1.84E-06
ENSMMUG00000058614		-2.145512905	0.000109513	0.000495145
ENSMMUG00000016860	zinc finger protein 490 [Source:NCBI gene;Acc:100426568]	-0.971113455	0.001502446	0.005166072
ENSMMUG00000016862	zinc finger protein 791 [Source:VGNC Symbol;Acc:VGNC:79639]	-0.866313721	0.015177264	0.038633509
ENSMMUG00000017971	nucleus accumbens associated 1 [Source:VGNC Symbol;Acc:VGNC:74996]	1.883817421	1.31E-05	7.22E-05
ENSMMUG00000057038		1.386143009	0.000419533	0.001662661
ENSMMUG00000011427	syntaxin 10 [Source:VGNC Symbol;Acc:VGNC:78040]	2.21488186	1.64E-32	2.09E-30
ENSMMUG00000003555	calcium voltage-gated channel subunit alpha1 A [Source:VGNC Symbol;Acc:VGNC:70498]	0.468067445	0.000119055	0.000532926
ENSMMUG00000058941	Metazoan signal recognition particle RNA [Source:RFAM;Acc:RF00017]	-1.467669936	0.014843686	0.037921078
ENSMMUG00000014137	adhesion G protein-coupled receptor L1 [Source:VGNC Symbol;Acc:VGNC:69658]	-0.360944308	0.003318317	0.010368868
ENSMMUG00000060000		1.707099053	0.0001971	0.000841391
ENSMMUG00000005727	protein kinase N1 [Source:VGNC Symbol;Acc:VGNC:76014]	-1.61438549	2.30E-16	5.58E-15
ENSMMUG00000005729	GIPC PDZ domain containing family member 1 [Source:VGNC Symbol;Acc:VGNC:72932]	1.279829181	1.12E-12	1.83E-11
ENSMMUG00000017906	DnaJ heat shock protein family (Hsp40) member B1 [Source:NCBI gene;Acc:718890]	2.060606414	2.18E-06	1.39E-05
ENSMMUG00000018932	adhesion G protein-coupled receptor E2 [Source:NCBI gene;Acc:654420]	2.936077665	4.22E-26	2.82E-24
ENSMMUG00000060289		3.145402304	2.48E-34	3.91E-32
ENSMMUG00000053889	olfactory receptor 7C1 [Source:NCBI gene;Acc:114674087]	-1.527757622	3.36E-06	2.05E-05
ENSMMUG00000064639	olfactory receptor 7A10-like [Source:NCBI gene;Acc:114674167]	1.697506569	0.019140396	0.047010944
ENSMMUG00000053457	caspase 14 [Source:NCBI gene;Acc:718049]	2.85472606	2.12E-16	5.15E-15
ENSMMUG00000053220	olfactory receptor 1I1 [Source:NCBI gene;Acc:719077]	1.787855416	0.00701423	0.019912142
ENSMMUG00000007726	synapse defective Rho GTPase homolog 1 [Source:NCBI gene;Acc:719089]	3.00171754	2.45E-15	5.37E-14
ENSMMUG00000007729	ilvB acetolactate synthase like [Source:VGNC Symbol;Acc:VGNC:73739]	2.413487833	1.43E-12	2.31E-11
ENSMMUG00000013637	notch receptor 3 [Source:NCBI gene;Acc:719119]	0.832259814	0.004023964	0.012299132
ENSMMUG00000028863	WIZ zinc finger [Source:VGNC Symbol;Acc:VGNC:79901]	-0.783481645	0.004768211	0.014265195
ENSMMUG00000016996	cytochrome P450, family 4, subfamily F, polypeptide 22 [Source:NCBI gene;Acc:718303]	0.760529661	4.68E-05	0.000229381
ENSMMUG00000010734	cytochrome P450, family 4, subfamily F, polypeptide 8 [Source:NCBI gene;Acc:718335]	0.686132384	4.45E-07	3.20E-06
ENSMMUG00000056778		3.282818042	0.019743045	0.04832305
ENSMMUG00000057552	hematopoietic SH2 domain containing [Source:NCBI gene;Acc:719440]	3.873544797	3.79E-05	0.000188857
ENSMMUG00000004283	calcium and integrin binding family member 3 [Source:VGNC Symbol;Acc:VGNC:71339]	3.128520838	5.44E-17	1.40E-15
ENSMMUG00000000994	epidermal growth factor receptor pathway substrate 15 like 1 [Source:VGNC Symbol;Acc:VGNC:72100]	2.264069335	6.99E-07	4.88E-06
ENSMMUG00000039774	Metazoan signal recognition particle RNA [Source:RFAM;Acc:RF00017]	0.834137379	4.56E-07	3.28E-06
ENSMMUG00000012959	solute carrier family 35 member E1 [Source:VGNC Symbol;Acc:VGNC:77536]	0.719701033	3.39E-05	0.000170891
ENSMMUG00000018503	NACHT and WD repeat domain containing 1 [Source:NCBI gene;Acc:719640]	0.524445933	0.0007399	0.002743866
ENSMMUG00000057655		-0.873891099	1.80E-05	9.69E-05
ENSMMUG00000011588	myosin IXB [Source:HGNC Symbol;Acc:HGNC:7609]	1.05174147	6.57E-09	6.34E-08
ENSMMUG00000013923	BRISC and BRCA1 A complex member 1 [Source:VGNC Symbol;Acc:VGNC:70126]	-0.618599001	0.003093282	0.009747849
ENSMMUG00000016228	ankyrin repeat and LEM domain containing 1 [Source:VGNC Symbol;Acc:VGNC:69715]	-1.055696984	0.011948022	0.031556397
ENSMMUG00000016231	DET1 and DDB1 associated 1 [Source:VGNC Symbol;Acc:VGNC:71774]	-0.942872625	0.016902334	0.042384445

ENSMMUG00000023403	GTP binding protein 3, mitochondrial [Source:VGNC Symbol;Acc:VGNC:73170]	2.945475906	1.86E-06	1.20E-05
ENSMMUG00000000194	solute carrier family 27 member 1 [Source:VGNC Symbol;Acc:VGNC:77438]	1.010016218	6.31E-09	6.11E-08
ENSMMUG00000000196	niban apoptosis regulator 3 [Source:VGNC Symbol;Acc:VGNC:75241]	0.992653987	0.00049014	0.001910321
ENSMMUG00000059371		1.385967083	0.000451451	0.001773986
ENSMMUG00000000198	collagen beta(1-O)galactosyltransferase 1 [Source:NCBI gene;Acc:719980]	1.338430442	4.23E-19	1.33E-17
ENSMMUG00000015476	unc-13 homolog A [Source:NCBI gene;Acc:720000]	1.235924824	6.89E-14	1.31E-12
ENSMMUG00000057222	Metazoan signal recognition particle RNA [Source:RFAM;Acc:RF00017]	1.579979465	0.011235059	0.029934588
ENSMMUG00000038308	coiled-coil domain containing 124 [Source:NCBI gene;Acc:720072]	-0.59723477	0.009183057	0.02515418
ENSMMUG00000007398	potassium calcium-activated channel subfamily N member 1 [Source:VGNC Symbol;Acc:VGNC:74002]	-1.466151613	1.30E-07	1.03E-06
ENSMMUG00000007401	arrestin domain containing 2 [Source:VGNC Symbol;Acc:VGNC:69901]	1.468709467	1.58E-10	1.94E-09
ENSMMUG00000003161	microtubule associated serine/threonine kinase 3 [Source:VGNC Symbol;Acc:VGNC:74533]	0.712057511	0.000285664	0.001172219
ENSMMUG00000057067	MPV17 mitochondrial inner membrane protein like 2 [Source:NCBI gene;Acc:719413]	-1.546126384	0.006164496	0.017806678
ENSMMUG00000011317	RAB3D, member RAS oncogene family [Source:VGNC Symbol;Acc:VGNC:81542]	-1.142200536	0.001246041	0.004383013
ENSMMUG00000061452		-2.025822586	9.34E-05	0.000428946
ENSMMUG00000005028	phosphodiesterase 4C [Source:NCBI gene;Acc:720171]	-0.998464603	4.84E-05	0.00023564
ENSMMUG00000065139	leucine-rich repeat extensin-like protein 5 [Source:NCBI gene;Acc:721121]	-2.997256318	0.003597594	0.011126658
ENSMMUG00000016354	IQ motif containing N [Source:VGNC Symbol;Acc:VGNC:73683]	-1.413337147	1.13E-14	2.34E-13
ENSMMUG00000038371	JunD proto-oncogene, AP-1 transcription factor subunit [Source:NCBI gene;Acc:720182]	-1.361223156	1.98E-05	0.000105451
ENSMMUG00000013671	elongation factor for RNA polymerase II [Source:VGNC Symbol;Acc:VGNC:72088]	1.188133877	7.74E-11	9.92E-10
ENSMMUG00000016085	KxDL motif containing 1 [Source:NCBI gene;Acc:720235]	1.210138573	2.22E-13	3.94E-12
ENSMMUG00000064485	ubiquitin A-52 residue ribosomal protein fusion product 1 [Source:NCBI gene;Acc:720243]	0.967446019	2.11E-07	1.61E-06
ENSMMUG00000054512		1.52567234	4.26E-07	3.07E-06
ENSMMUG00000009988	transmembrane protein 59 like [Source:VGNC Symbol;Acc:VGNC:78919]	2.204133193	3.89E-31	4.06E-29
ENSMMUG00000064204	kelch like family member 26 [Source:NCBI gene;Acc:719583]	0.914990631	0.00112992	0.004020326
ENSMMUG00000006509	CREB regulated transcription coactivator 1 [Source:VGNC Symbol;Acc:VGNC:71418]	-1.190843516	3.01E-05	0.000153813
ENSMMUG00000023286	UPF1 RNA helicase and ATPase [Source:VGNC Symbol;Acc:VGNC:79971]	2.806384748	2.88E-35	4.86E-33
ENSMMUG00000050739		2.070279334	1.09E-06	7.37E-06
ENSMMUG00000019668	armadillo repeat containing 6 [Source:VGNC Symbol;Acc:VGNC:69899]	0.917035223	0.000357328	0.001438078
ENSMMUG00000008578	hyaluronan and proteoglycan link protein 4 [Source:VGNC Symbol;Acc:VGNC:73261]	-1.123067792	0.00032417	0.001317894
ENSMMUG00000008931	MAU2 sister chromatid cohesion factor [Source:NCBI gene;Acc:720347]	0.640150639	2.44E-08	2.16E-07
ENSMMUG00000003164	GATA zinc finger domain containing 2A [Source:NCBI gene;Acc:720361]	-0.616183274	0.000370185	0.001485748
ENSMMUG00000011351	YjeF N-terminal domain containing 3 [Source:NCBI gene;Acc:721218]	-0.437440522	0.002963442	0.009401585
ENSMMUG00000048108	PBX homeobox 4 [Source:NCBI gene;Acc:106994668]	-2.369327438	0.005246184	0.015448951
ENSMMUG00000053470	Metazoan signal recognition particle RNA [Source:RFAM;Acc:RF00017]	-3.126545855	0.001940895	0.006484226
ENSMMUG00000054055	zinc finger protein 93 [Source:NCBI gene;Acc:720434]	1.252333364	3.32E-11	4.46E-10
ENSMMUG00000056683		-0.897039432	0.000396466	0.001580959
ENSMMUG00000061085		-0.795399323	1.37E-07	1.08E-06

ENSMMUG00000062866		-2.040814197	2.40E-07	1.81E-06
ENSMMUG00000015533	zinc finger protein 493 [Source:NCBI gene;Acc:693611]	-1.176757306	0.000325686	0.001323267
ENSMMUG00000047027	zinc finger protein 100-like [Source:NCBI gene;Acc:693860]	-0.983377017	0.000722273	0.002686558
ENSMMUG00000046906		-0.806162044	5.26E-05	0.000254177
ENSMMUG00000061186		-0.960537838	1.18E-05	6.53E-05
ENSMMUG00000040273	zinc finger protein 257 [Source:NCBI gene;Acc:694388]	-1.195256747	0.000150845	0.00065989
ENSMMUG00000063815	zinc finger protein 208-like [Source:NCBI gene;Acc:721315]	-0.956010442	0.001217345	0.004294296
ENSMMUG00000060408		-1.503095567	1.99E-05	0.000105729
ENSMMUG00000037794	zinc finger protein 724 [Source:NCBI gene;Acc:698907]	-0.811863718	0.01211605	0.031926266
ENSMMUG00000003688	zinc finger protein 91 [Source:HGNC Symbol;Acc:HGNC:13166]	-1.231292667	0.000357338	0.001438078
ENSMMUG00000003645	zinc finger protein 726 [Source:HGNC Symbol;Acc:HGNC:32462]	-1.942766559	8.46E-05	0.000392392
ENSMMUG00000052284	zinc finger protein 254 [Source:NCBI gene;Acc:114674197]	3.839134406	0.002741651	0.008777783
ENSMMUG00000059910		-0.410209282	0.015355579	0.038980354
ENSMMUG00000051220		1.023403918	0.013893455	0.035901715
ENSMMUG00000058669		-0.879636924	0.013383162	0.03474655
ENSMMUG00000020668	cyclin E1 [Source:VGNC Symbol;Acc:VGNC:70727]	2.903072577	1.24E-44	6.12E-42
ENSMMUG00000063586	zinc finger protein 536 [Source:NCBI gene;Acc:713142]	-1.890929012	2.39E-06	1.51E-05
ENSMMUG00000058740		-0.90897203	1.97E-07	1.51E-06
ENSMMUG00000057215		-0.935291656	0.003904736	0.011961899
ENSMMUG00000050177		-1.975053141	1.18E-08	1.09E-07
ENSMMUG00000060455		-1.986684001	0.000643848	0.002433921
ENSMMUG00000010840	zinc finger protein 507 [Source:VGNC Symbol;Acc:VGNC:79410]	0.826486913	0.00250323	0.008086017
ENSMMUG00000008041	ankyrin repeat domain 27 [Source:VGNC Symbol;Acc:VGNC:69693]	0.668811026	9.92E-06	5.57E-05
ENSMMUG00000015608	tudor domain containing 12 [Source:VGNC Symbol;Acc:VGNC:82254]	-1.291306265	1.56E-06	1.03E-05
ENSMMUG00000028800	solute carrier family 7 member 9 [Source:VGNC Symbol;Acc:VGNC:77633]	-1.455136475	6.61E-09	6.38E-08
ENSMMUG00000018351	centrosomal protein 89 [Source:VGNC Symbol;Acc:VGNC:71105]	-0.728134567	0.015201686	0.03867432
ENSMMUG00000017939	FA core complex associated protein 24 [Source:VGNC Symbol;Acc:VGNC:72388]	-0.858684048	0.003361119	0.010489352
ENSMMUG00000028798	rhophilin Rho GTPase binding protein 2 [Source:NCBI gene;Acc:716602]	-0.443110125	0.017162109	0.042869139
ENSMMUG00000011790	G-patch domain containing 1 [Source:VGNC Symbol;Acc:VGNC:72996]	-1.030186067	0.002254029	0.0073677
ENSMMUG00000064983		-0.733233476	0.009755323	0.026486898
ENSMMUG00000061372		-0.779371035	0.010765408	0.028802272

ENSMMUG00000006124	CCAAT enhancer binding protein alpha [Source:VGNC Symbol;Acc:VGNC:71000]	-1.174740429	0.003397806	0.010590472
ENSMMUG00000062413	CCAAT enhancer binding protein gamma [Source:NCBI gene;Acc:704322]	-1.245882506	5.04E-05	0.000245021
ENSMMUG00000006128	peptidase D [Source:VGNC Symbol;Acc:VGNC:82205]	-1.280279333	6.55E-06	3.81E-05
ENSMMUG00000011319	potassium channel tetramerization domain containing 15 [Source:VGNC Symbol;Acc:VGNC:73894]	-0.800721095	3.06E-05	0.000155798
ENSMMUG00000005880	glucose-6-phosphate isomerase [Source:NCBI gene;Acc:717980]	-1.322156312	0.006495709	0.018617237
ENSMMUG00000015744	zinc finger protein 302 [Source:NCBI gene;Acc:706279]	2.621761428	3.67E-24	2.03E-22
ENSMMUG00000006440	zinc finger protein 599 [Source:VGNC Symbol;Acc:VGNC:79605]	1.97810027	0.002676908	0.008587183
ENSMMUG00000059543	zinc finger protein 30 [Source:NCBI gene;Acc:718310]	-0.933436447	1.66E-13	2.99E-12
ENSMMUG00000008313	zinc finger protein 792 [Source:VGNC Symbol;Acc:VGNC:79456]	-0.85438461	2.29E-10	2.76E-09
ENSMMUG00000001538	GRAM domain containing 1A [Source:VGNC Symbol;Acc:VGNC:73029]	1.224289509	3.62E-17	9.55E-16
ENSMMUG00000004843	sodium voltage-gated channel beta subunit 1 [Source:VGNC Symbol;Acc:VGNC:76983]	1.175816878	1.06E-11	1.50E-10
ENSMMUG00000058405	dermokine [Source:NCBI gene;Acc:718709]	-0.722205646	0.010481787	0.028160309
ENSMMUG00000018954	RNA binding motif protein 42 [Source:VGNC Symbol;Acc:VGNC:76553]	2.075510331	0.015424374	0.039128201
ENSMMUG00000055747	cytochrome c oxidase subunit 6B [Source:NCBI gene;Acc:692062]	1.654663167	2.52E-16	6.07E-15
ENSMMUG00000065025		1.754440318	3.45E-21	1.40E-19
ENSMMUG00000002381	lysine methyltransferase 2B [Source:VGNC Symbol;Acc:VGNC:74053]	0.983121135	5.03E-17	1.31E-15
ENSMMUG00000002384	IGF like family receptor 1 [Source:VGNC Symbol;Acc:VGNC:73434]	1.870644926	1.51E-19	4.97E-18
ENSMMUG00000048190	U2 small nuclear RNA auxiliary factor 1 like 4 [Source:NCBI gene;Acc:718865]	1.792660336	4.32E-06	2.60E-05
ENSMMUG00000002386	lin-37 DREAM MuvB core complex component [Source:NCBI gene;Acc:710495]	-1.072846122	0.012541547	0.032872241
ENSMMUG00000019749	NPHS1 adhesion molecule, nephrin [Source:NCBI gene;Acc:718929]	-0.903621267	0.000439788	0.001732287
ENSMMUG00000019756	NFKB inhibitor delta [Source:VGNC Symbol;Acc:VGNC:75179]	-0.946239875	4.56E-06	2.74E-05
ENSMMUG00000008004	transmembrane immune signaling adaptor TYROBP [Source:NCBI gene;Acc:574207]	-1.089180332	8.40E-12	1.22E-10
ENSMMUG00000062667	leucine rich repeat and fibronectin type III domain containing 3 [Source:NCBI gene;Acc:711565]	-0.84194567	0.001695539	0.005748707
ENSMMUG00000062586		-1.240460561	0.008731164	0.024098858
ENSMMUG00000053233		-0.873107975	0.004380954	0.013245575
ENSMMUG00000051879	CAP-Gly domain containing linker protein 3 [Source:NCBI gene;Acc:712058]	2.296685867	1.81E-05	9.72E-05
ENSMMUG00000019704	WD repeat domain 62 [Source:NCBI gene;Acc:719053]	3.776443094	0.00013179	0.000585165
ENSMMUG00000028785	zinc finger protein 146 [Source:VGNC Symbol;Acc:VGNC:78846]	2.933762389	1.55E-06	1.02E-05
ENSMMUG00000057189		3.025118273	0.001263994	0.004437745
ENSMMUG00000060977	ZFP14 zinc finger protein [Source:NCBI gene;Acc:719135]	-1.471524719	0.008963544	0.024634795
ENSMMUG00000061035	ZFP82 zinc finger protein [Source:NCBI gene;Acc:719145]	-0.967837669	1.09E-05	6.08E-05
ENSMMUG00000022793	zinc finger protein 529 [Source:VGNC Symbol;Acc:VGNC:79801]	-0.842086529	0.000207092	0.000880243
ENSMMUG00000063196		-1.058004788	0.007815018	0.02187556
ENSMMUG00000016449	zinc finger protein 382 [Source:VGNC Symbol;Acc:VGNC:79078]	-0.815080836	0.000512269	0.001988728
ENSMMUG00000062165		0.741604947	0.001286663	0.00450986
ENSMMUG00000014214	zinc finger protein 420 [Source:VGNC Symbol;Acc:VGNC:82277]	0.576935281	0.000398096	0.001586179
ENSMMUG00000000107	zinc finger protein 585A [Source:NCBI gene;Acc:100424957]	0.523530874	0.000756028	0.00279738

ENSMMUG00000011092	zinc finger protein 540 [Source:VGNC Symbol;Acc:VGNC:79490]	-1.922797404	0.007129734	0.020179525
ENSMMUG00000006820	WD repeat domain 87 [Source:NCBI gene;Acc:719441]	0.288753092	0.005711343	0.016653404
ENSMMUG00000028771	Yip1 interacting factor homolog B, membrane trafficking protein [Source:VGNC Symbol;Acc:VGNC:79852]	1.835273721	4.91E-10	5.64E-09
ENSMMUG00000062296	potassium two pore domain channel subfamily K member 6 [Source:NCBI gene;Acc:714912]	4.774375229	1.85E-08	1.66E-07
ENSMMUG00000056109	Metazoan signal recognition particle RNA [Source:RFAM;Acc:RF00017]	4.288944607	6.40E-06	3.73E-05
ENSMMUG00000014273	cation channel sperm associated auxiliary subunit gamma [Source:VGNC Symbol;Acc:VGNC:70626]	3.618635888	2.80E-13	4.90E-12
ENSMMUG00000038221	proteasome 26S subunit, non-ATPase 8 [Source:NCBI gene;Acc:721897]	0.638228023	0.000270151	0.001116688
ENSMMUG00000022975	sprouty related EVH1 domain containing 3 [Source:VGNC Symbol;Acc:VGNC:78030]	-2.969747519	9.78E-14	1.82E-12
ENSMMUG00000021630	mitogen-activated protein kinase kinase kinase kinase 1 [Source:NCBI gene;Acc:694259]	-1.901747998	0.011679898	0.030958515
ENSMMUG00000006431	calpain 12 [Source:VGNC Symbol;Acc:VGNC:70473]	0.948302755	0.007866067	0.022005377
ENSMMUG00000028766	galectin 4 [Source:VGNC Symbol;Acc:VGNC:74257]	1.045800117	0.000224753	0.000947439
ENSMMUG00000004877	heterogeneous nuclear ribonucleoprotein L [Source:VGNC Symbol;Acc:VGNC:81187]	0.797509591	0.003169299	0.009966222
ENSMMUG00000012787	acid phosphatase 7, tartrate resistant (putative) [Source:NCBI gene;Acc:695532]	1.3076313	0.000177553	0.000764772
ENSMMUG00000005552	PAF1 homolog, Paf1/RNA polymerase II complex component [Source:VGNC Symbol;Acc:VGNC:81492]	4.333199605	1.09E-08	1.02E-07
ENSMMUG00000005554	mediator complex subunit 29 [Source:VGNC Symbol;Acc:VGNC:74557]	6.151843006	2.20E-83	3.27E-79
ENSMMUG00000038702	ZFP36 ring finger protein [Source:NCBI gene;Acc:722000]	6.263417616	4.19E-12	6.35E-11
ENSMMUG00000019369	pleckstrin homology and RhoGEF domain containing G2 [Source:VGNC Symbol;Acc:VGNC:76133]	5.598614979	2.68E-17	7.19E-16
ENSMMUG00000019093	delta like canonical Notch ligand 3 [Source:VGNC Symbol;Acc:VGNC:71925]	-2.911530529	0.001005348	0.003617009
ENSMMUG00000048215		4.694008631	8.98E-36	1.62E-33
ENSMMUG00000022726	dual specificity tyrosine phosphorylation regulated kinase 1B [Source:VGNC Symbol;Acc:VGNC:72016]	-2.295411435	5.49E-07	3.90E-06
ENSMMUG00000022724		-1.726145093	0.000275242	0.001135736
ENSMMUG00000051261		-1.875268527	2.65E-09	2.72E-08
ENSMMUG00000017188	proteasome 26S subunit, ATPase 4 [Source:VGNC Symbol;Acc:VGNC:76448]	0.79000709	0.002533825	0.008179499
ENSMMUG00000017191	zinc finger protein 546 [Source:VGNC Symbol;Acc:VGNC:79732]	-0.889522366	0.001556166	0.005324859
ENSMMUG00000041976	zinc finger protein 780B [Source:NCBI gene;Acc:701034]	-0.800575318	2.34E-06	1.48E-05
ENSMMUG00000003621	AKT serine/threonine kinase 2 [Source:VGNC Symbol;Acc:VGNC:69777]	-1.222895469	7.08E-10	7.98E-09
ENSMMUG00000053624		-1.225707835	1.33E-05	7.34E-05
ENSMMUG00000008946	chromosome 19 C19orf47 homolog [Source:VGNC Symbol;Acc:VGNC:70404]	-0.94526901	3.46E-08	2.97E-07
ENSMMUG00000022625	periaxin [Source:VGNC Symbol;Acc:VGNC:76248]	-1.773399152	7.50E-05	0.000350751
ENSMMUG00000051535		-1.257945672	0.003356901	0.010480355
ENSMMUG00000063763	cytochrome P450 2A13-like [Source:NCBI gene;Acc:114674219]	1.200893675	0.000511713	0.001987612
ENSMMUG00000057008		0.637977239	0.016312753	0.041107583
ENSMMUG00000009145	AXL receptor tyrosine kinase [Source:VGNC Symbol;Acc:VGNC:70200]	-0.803156706	0.00031278	0.001275374
ENSMMUG00000014346	transmembrane protein 91 [Source:NCBI gene;Acc:100426021]	-1.072057426	0.005793576	0.016866653
ENSMMUG00000059229	CEA cell adhesion molecule 7 [Source:NCBI gene;Acc:705572]	2.002603177	9.09E-26	5.76E-24
ENSMMUG00000022305	Rho guanine nucleotide exchange factor 1 [Source:VGNC Symbol;Acc:VGNC:70012]	-1.155063362	3.55E-07	2.60E-06
ENSMMUG00000022309	ETS repressor factor like [Source:NCBI gene;Acc:722195]	-1.14017057	0.005334508	0.015687241

ENSMMUG00000017427	ATPase Na+/K+ transporting subunit alpha 3 [Source:NCBI gene;Acc:706513]	-1.623621861	0.000744168	0.002757623
ENSMMUG00000017431	glutamate ionotropic receptor kainate type subunit 5 [Source:VGNC Symbol;Acc:VGNC:73280]	-1.138255235	0.000215892	0.000913982
ENSMMUG00000013376	POU class 2 homeobox 2 [Source:NCBI gene;Acc:706835]	-1.180454824	1.47E-11	2.06E-10
ENSMMUG00000012174	capicua transcriptional repressor [Source:NCBI gene;Acc:707463]	1.975033636	0.006673078	0.01906043
ENSMMUG00000050799		3.864292876	5.47E-05	0.000263559
ENSMMUG00000055813	pregnancy-specific beta-1-glycoprotein 5-like [Source:NCBI gene;Acc:114674020]	0.922819652	0.000664378	0.002500059
ENSMMUG00000064477	Metazoan signal recognition particle RNA [Source:RFAM;Acc:RF00017]	-1.431167143	0.015190421	0.038660365
ENSMMUG00000019265	zinc finger protein 575 [Source:VGNC Symbol;Acc:VGNC:79667]	-0.732132882	0.005081067	0.015052881
ENSMMUG00000004467	X-ray repair cross complementing 1 [Source:NCBI gene;Acc:711457]	-0.62126163	0.001150298	0.004084991
ENSMMUG00000004615	cell adhesion molecule 4 [Source:VGNC Symbol;Acc:VGNC:70681]	0.339778162	0.014607458	0.037414267
ENSMMUG00000014543	SMG9 nonsense mediated mRNA decay factor [Source:VGNC Symbol;Acc:VGNC:77775]	-1.512158804	0.003518783	0.010910212
ENSMMUG00000021474	zinc finger protein 45 [Source:NCBI gene;Acc:712591]	-1.013759089	1.42E-12	2.29E-11
ENSMMUG00000064975	zinc finger protein 221 [Source:NCBI gene;Acc:100423743]	-2.612432207	5.63E-10	6.41E-09
ENSMMUG00000047704	zinc finger protein 223 [Source:NCBI gene;Acc:711652]	1.940115199	0.020473972	0.049799739
ENSMMUG00000021469	zinc finger protein 284 [Source:NCBI gene;Acc:713143]	-1.032259993	0.01912759	0.046987272
ENSMMUG00000012797	zinc finger protein 233 [Source:VGNC Symbol;Acc:VGNC:79903]	2.148206083	1.61E-21	6.80E-20
ENSMMUG00000012801	zinc finger protein 235 [Source:VGNC Symbol;Acc:VGNC:78825]	2.205608834	8.44E-32	9.48E-30
ENSMMUG00000012802	zinc finger protein 112 [Source:NCBI gene;Acc:713527]	0.533908513	0.002628094	0.008445207
ENSMMUG00000003273	basal cell adhesion molecule (Lutheran blood group) [Source:NCBI gene;Acc:714568]	-0.785923669	5.62E-06	3.31E-05
ENSMMUG00000014303	translocase of outer mitochondrial membrane 40 [Source:VGNC Symbol;Acc:VGNC:78419]	-2.918486953	0.001166599	0.004135945
ENSMMUG00000002472	CLPTM1 regulator of GABA type A receptor forward trafficking [Source:VGNC Symbol;Acc:VGNC:71265]	-1.485129262	0.001065627	0.003810768
ENSMMUG00000014155	CLK4 associating serine/arginine rich protein [Source:VGNC Symbol;Acc:VGNC:71198]	-1.709261101	0.000874944	0.003186498
ENSMMUG00000022931	NTPase KAP family P-loop domain containing 1 [Source:VGNC Symbol;Acc:VGNC:75250]	1.348442619	1.97E-12	3.13E-11
ENSMMUG00000018879	exocyst complex component 3 like 2 [Source:NCBI gene;Acc:713992]	0.861087739	2.13E-05	0.000112617
ENSMMUG00000018672	microtubule affinity regulating kinase 4 [Source:VGNC Symbol;Acc:VGNC:74508]	-0.430535076	0.001329047	0.004641961
ENSMMUG00000013477	creatine kinase, M-type [Source:VGNC Symbol;Acc:VGNC:71230]	3.796175395	5.98E-32	6.87E-30
ENSMMUG00000014419	ERCC excision repair 2, TFIIH core complex helicase subunit [Source:VGNC Symbol;Acc:VGNC:72423]	0.464190843	0.001711578	0.005797784
ENSMMUG00000000715	protein phosphatase 1 regulatory subunit 13 like [Source:NCBI gene;Acc:714389]	0.530049987	8.55E-05	0.000396342
ENSMMUG00000000717	CD3e molecule associated protein [Source:NCBI gene;Acc:714440]	-1.5349333	5.98E-11	7.79E-10
ENSMMUG00000000718	ERCC excision repair 1, endonuclease non-catalytic subunit [Source:VGNC Symbol;Acc:VGNC:72422]	-1.108444053	1.72E-07	1.33E-06
ENSMMUG00000053242	outer mitochondrial membrane lipid metabolism regulator OPA3 [Source:NCBI gene;Acc:714806]	2.376749985	0.006051781	0.017518624
ENSMMUG00000019031	G protein-coupled receptor 4 [Source:VGNC Symbol;Acc:VGNC:73136]	3.006479804	0.013805057	0.035698168
ENSMMUG00000019032	EMAP like 2 [Source:NCBI gene;Acc:715009]	2.793378218	0.012652281	0.033133202
ENSMMUG00000006936	gastric inhibitory polypeptide receptor [Source:VGNC Symbol;Acc:VGNC:73052]	0.747955959	2.91E-05	0.00014907
ENSMMUG00000032809	mml-mir-642 [Source:miRBase;Acc:MI0007885]	0.695433093	0.001933689	0.006468893
ENSMMUG00000006937	small nuclear ribonucleoprotein D2 polypeptide [Source:NCBI gene;Acc:715183]	-1.985268649	0.001177815	0.004171722
ENSMMUG00000006940	glutaminyl-peptide cyclotransferase like [Source:VGNC Symbol;Acc:VGNC:76540]	-1.266276445	3.65E-05	0.000182693

ENSMMUG00000021360	F-box protein 46 [Source:VGNC Symbol;Acc:VGNC:72570]	-0.819833843	0.002595353	0.00835448
ENSMMUG00000049025	basic helix-loop-helix and HMG-box containing 1 [Source:NCBI gene;Acc:716325]	-0.627450988	0.007211148	0.020376093
ENSMMUG00000051573	SIX homeobox 5 [Source:NCBI gene;Acc:715373]	-0.616968575	0.000116657	0.000522823
ENSMMUG00000016457	DM1 protein kinase [Source:NCBI gene;Acc:715328]	0.967149274	0.000855552	0.003126623
ENSMMUG00000051115		-1.745293763	0.000896134	0.00325408
ENSMMUG00000017927	protein kinase D2 [Source:VGNC Symbol;Acc:VGNC:76273]	1.884239122	0.005081235	0.015052881
ENSMMUG00000017930	striatin 4 [Source:VGNC Symbol;Acc:VGNC:78128]	2.501403977	0.004606377	0.013853695
ENSMMUG00000011332	Rho GTPase activating protein 35 [Source:VGNC Symbol;Acc:VGNC:69942]	3.920047098	1.37E-56	2.63E-53
ENSMMUG00000057361	Metazoan signal recognition particle RNA [Source:RFAM;Acc:RF00017]	3.37688954	6.46E-31	6.65E-29
ENSMMUG00000011330	neuronal PAS domain protein 1 [Source:VGNC Symbol;Acc:VGNC:75377]	1.26665441	4.85E-06	2.89E-05
ENSMMUG00000057454	transmembrane protein 160 [Source:NCBI gene;Acc:716996]	1.554042365	2.64E-06	1.66E-05
ENSMMUG00000014709	zinc finger CCCH-type containing 4 [Source:VGNC Symbol;Acc:VGNC:79094]	0.40587636	0.012005086	0.03168454
ENSMMUG00000009506	SUMO1 activating enzyme subunit 1 [Source:NCBI gene;Acc:717918]	0.34259136	0.003854894	0.011833592
ENSMMUG00000055893		1.373356784	6.86E-07	4.80E-06
ENSMMUG00000012466	BCL2 binding component 3 [Source:NCBI gene;Acc:717955]	1.053629393	1.60E-07	1.25E-06
ENSMMUG00000003075	coiled-coil domain containing 9 [Source:VGNC Symbol;Acc:VGNC:70851]	1.023858333	3.56E-08	3.05E-07
ENSMMUG00000014718	solute carrier family 8 member A2 [Source:VGNC Symbol;Acc:VGNC:77635]	-0.344258313	0.005561696	0.016271444
ENSMMUG00000014727	NSF attachment protein alpha [Source:VGNC Symbol;Acc:VGNC:75131]	-1.186982987	2.53E-10	3.03E-09
ENSMMUG00000054090		1.018173582	0.005178295	0.015288497
ENSMMUG00000006857	epididymal sperm binding protein 1 [Source:VGNC Symbol;Acc:VGNC:72046]	-1.048759807	0.000738614	0.002739782
ENSMMUG00000059158	Metazoan signal recognition particle RNA [Source:RFAM;Acc:RF00017]	-1.682863993	0.003526228	0.010928726
ENSMMUG00000063571	Metazoan signal recognition particle RNA [Source:RFAM;Acc:RF00017]	-3.024604612	0.017855858	0.044357433
ENSMMUG00000055107		0.430519364	0.001578062	0.005383636
ENSMMUG00000042157	caspase recruitment domain-containing protein 8 [Source:NCBI gene;Acc:718594]	-0.470847487	0.001322959	0.004621785
ENSMMUG00000022155	sulfotransferase family 2B member 1 [Source:NCBI gene;Acc:718739]	0.716406588	0.001114047	0.003970515
ENSMMUG00000064152	netrin 5 [Source:NCBI gene;Acc:718157]	-0.439370065	0.012527341	0.032840811
ENSMMUG00000055036	MEF2 activating motif and SAP domain containing transcriptional regulator [Source:NCBI gene;Acc:718823]	-0.548715046	0.001526131	0.005234163
ENSMMUG00000018415	Ras interacting protein 1 [Source:HGNC Symbol;Acc:HGNC:24716]	-0.63396857	0.001882377	0.006317187
ENSMMUG00000004019	izumo sperm-egg fusion 1 [Source:VGNC Symbol;Acc:VGNC:73670]	-1.059672521	0.00013574	0.000600006
ENSMMUG00000028736	hydroxysteroid 17-beta dehydrogenase 14 [Source:NCBI gene;Acc:106994816]	-1.015671409	0.000134122	0.00059445
ENSMMUG00000009222	pleckstrin homology domain containing A4 [Source:VGNC Symbol;Acc:VGNC:76027]	-1.107771212	0.005848629	0.017003563
ENSMMUG00000048854	TUB like protein 2 [Source:NCBI gene;Acc:718915]	-1.480158915	0.000154023	0.000671616
ENSMMUG00000003904	nucleobindin 1 [Source:VGNC Symbol;Acc:VGNC:75548]	-2.139671758	0.000418381	0.001658981
ENSMMUG00000003911	glycogen synthase 1 [Source:VGNC Symbol;Acc:VGNC:73334]	-1.889812274	0.004157165	0.012661538
ENSMMUG00000007538	transient receptor potential cation channel subfamily M member 4 [Source:VGNC Symbol;Acc:VGNC:79105]	2.965592261	3.71E-34	5.73E-32
ENSMMUG00000061126	golgi associated olfactory signaling regulator [Source:NCBI gene;Acc:719194]	1.262862027	0.005174648	0.015283814
ENSMMUG00000022116	PIH1 domain containing 1 [Source:VGNC Symbol;Acc:VGNC:75868]	1.581674137	0.000502373	0.001953379

ENSMMUG00000051892	fms related receptor tyrosine kinase 3 ligand [Source:NCBI gene;Acc:719239]	1.515452423	0.000136092	0.000601206
ENSMMUG00000003819	nitric oxide synthase interacting protein [Source:VGNC Symbol;Acc:VGNC:75207]	2.294081051	2.74E-14	5.40E-13
ENSMMUG00000049747	proline rich and Gla domain 2 [Source:NCBI gene;Acc:719318]	0.476680168	0.001428009	0.004939904
ENSMMUG00000012937	proline rich 12 [Source:NCBI gene;Acc:719344]	3.686003646	9.65E-40	2.56E-37
ENSMMUG00000028733	RAS related [Source:VGNC Symbol;Acc:VGNC:76937]	3.084293363	1.17E-26	8.49E-25
ENSMMUG00000012938	SR-related CTD associated factor 1 [Source:VGNC Symbol;Acc:VGNC:77137]	4.012898316	1.49E-07	1.17E-06
ENSMMUG00000003926	BCL2 like 12 [Source:NCBI gene;Acc:718981]	3.989111168	0.000132372	0.00058757
ENSMMUG00000003029	protein arginine methyltransferase 1 [Source:NCBI gene;Acc:719419]	-1.433547359	1.59E-08	1.44E-07
ENSMMUG00000014735	carnitine palmitoyltransferase 1C [Source:NCBI gene;Acc:719429]	-1.645163377	1.23E-06	8.22E-06
ENSMMUG00000020175	mediator complex subunit 25 [Source:VGNC Symbol;Acc:VGNC:74607]	-1.64594953	0.000114981	0.000516336
ENSMMUG00000011627	interleukin 4 induced 1 [Source:VGNC Symbol;Acc:VGNC:73625]	2.955709441	1.82E-20	6.77E-19
ENSMMUG00000044817	nucleoporin 62 [Source:NCBI gene;Acc:100426308]	3.510078655	2.70E-82	2.00E-78
ENSMMUG00000054473	activating transcription factor 5 [Source:NCBI gene;Acc:719150]	1.309666077	1.55E-10	1.91E-09
ENSMMUG00000050456	Metazoan signal recognition particle RNA [Source:RFAM;Acc:RF00017]	1.733117357	0.007302123	0.020595375
ENSMMUG00000064146	Metazoan signal recognition particle RNA [Source:RFAM;Acc:RF00017]	3.182960904	1.20E-19	4.02E-18
ENSMMUG00000054052	sialic acid binding Ig like lectin 11 [Source:NCBI gene;Acc:719127]	3.571748087	2.20E-09	2.29E-08
ENSMMUG00000010837	VRK serine/threonine kinase 3 [Source:VGNC Symbol;Acc:VGNC:81605]	2.935136464	1.05E-11	1.50E-10
ENSMMUG00000001427	myosin heavy chain 14 [Source:VGNC Symbol;Acc:VGNC:75006]	2.131037005	1.98E-19	6.41E-18
ENSMMUG00000055885	napsin-A [Source:NCBI gene;Acc:719235]	-1.069257262	0.000290757	0.001190484
ENSMMUG00000023418	nuclear receptor subfamily 1 group H member 2 [Source:VGNC Symbol;Acc:VGNC:75504]	0.741197889	1.10E-05	6.14E-05
ENSMMUG00000006718	ER membrane protein complex subunit 10 [Source:NCBI gene;Acc:719285]	2.697215177	0.005567773	0.016286011
ENSMMUG00000021269	Josephin domain containing 2 [Source:VGNC Symbol;Acc:VGNC:73919]	3.298264768	0.006964425	0.019789696
ENSMMUG00000021059	leucine rich repeat containing 4B [Source:VGNC Symbol;Acc:VGNC:74350]	0.610934848	9.03E-05	0.000416172
ENSMMUG00000056558		0.716203259	2.94E-06	1.82E-05
ENSMMUG00000056529	synaptotagmin 3 [Source:NCBI gene;Acc:719650]	2.283648574	0.00025984	0.001080299
ENSMMUG00000046099	chromosome 19 C19orf81 homolog [Source:NCBI gene;Acc:719661]	3.244764979	8.07E-08	6.59E-07
ENSMMUG00000059087		4.310898689	9.51E-09	8.93E-08
ENSMMUG00000012841	kallikrein 3 [Source:NCBI gene;Acc:719444]	1.435458381	3.99E-07	2.89E-06
ENSMMUG00000065035	kallikrein-2 [Source:NCBI gene;Acc:719436]	-0.735710862	0.000243068	0.001017415
ENSMMUG00000060764		0.547286736	1.42E-06	9.37E-06
ENSMMUG00000028729	kallikrein related peptidase 4 [Source:NCBI gene;Acc:719422]	-0.940007552	0.003413021	0.010624498
ENSMMUG00000013597	kallikrein related peptidase 5 [Source:VGNC Symbol;Acc:VGNC:74191]	-0.702623961	0.004898184	0.014601019
ENSMMUG00000061396	kallikrein related peptidase 6 [Source:NCBI gene;Acc:719776]	-1.032995879	0.000112698	0.000507527
ENSMMUG00000015445	kallikrein related peptidase 8 [Source:VGNC Symbol;Acc:VGNC:74194]	-0.61172925	0.014661587	0.037533461
ENSMMUG00000028728	kallikrein related peptidase 9 [Source:NCBI gene;Acc:100424925]	-0.993249958	0.007306714	0.020600487
ENSMMUG00000059154	kallikrein related peptidase 10 [Source:NCBI gene;Acc:106994828]	-1.73909128	0.011970143	0.031609192
ENSMMUG00000064856	kallikrein related peptidase 14 [Source:NCBI gene;Acc:722692]	-1.008237922	0.010206114	0.027544435
ENSMMUG00000064898	cytosolic thiouridylase subunit 1 [Source:NCBI gene;Acc:719536]	-0.973211128	0.000689714	0.002583596
ENSMMUG00000044243	sialic acid-binding Ig-like lectin 9 [Source:NCBI gene;Acc:719831]	-1.142784405	0.005165959	0.015261189
ENSMMUG00000047370	IgLON family member 5 [Source:NCBI gene;Acc:719890]	-1.293315846	0.000207309	0.000880897
ENSMMUG00000044652	sialic acid-binding Ig-like lectin 13 [Source:NCBI gene;Acc:719667]	-0.547349932	0.004052324	0.012367969

ENSMMUG00000062017		-2.379637277	7.43E-05	0.000348036
ENSMMUG00000012764	zinc finger protein 175 [Source:VGNC Symbol;Acc:VGNC:78856]	-0.939428449	3.74E-05	0.000186376
ENSMMUG00000012761	sialic acid binding Ig like lectin 5 [Source:NCBI gene;Acc:719730]	-0.52601537	1.98E-05	0.000105451
ENSMMUG00000013053	sperm acrosome associated 6 [Source:NCBI gene;Acc:114674203]	-1.579196074	1.23E-12	2.00E-11
ENSMMUG00000053150	Metazoan signal recognition particle RNA [Source:RFAM;Acc:RF00017]	-1.134246757	0.015933317	0.040274603
ENSMMUG00000013049	formyl peptide receptor 1 [Source:NCBI gene;Acc:719782]	-0.905890764	0.002385557	0.007753192
ENSMMUG00000051078		-0.872677584	1.78E-05	9.58E-05
ENSMMUG00000002310	zinc finger protein 577 [Source:VGNC Symbol;Acc:VGNC:79733]	-0.696347502	5.82E-06	3.42E-05
ENSMMUG00000023136	zinc finger protein 613 [Source:VGNC Symbol;Acc:VGNC:79548]	-0.532024427	3.25E-05	0.000164792
ENSMMUG00000052090		-0.479958404	0.000183438	0.000788061
ENSMMUG00000023137	zinc finger protein 350 [Source:VGNC Symbol;Acc:VGNC:79041]	-1.565501091	3.62E-11	4.84E-10
ENSMMUG00000003712	zinc finger protein 615 [Source:VGNC Symbol;Acc:VGNC:79454]	-1.11829946	6.71E-05	0.000317334
ENSMMUG00000041450	zinc finger protein 432 [Source:NCBI gene;Acc:100424341]	-1.670876236	4.87E-09	4.79E-08
ENSMMUG00000038056	zinc finger protein 841 [Source:NCBI gene;Acc:720128]	-1.6717474	0.015066773	0.038404991
ENSMMUG00000001768	zinc finger protein 616 [Source:NCBI gene;Acc:719902]	-0.640774861	0.000508227	0.001974589
ENSMMUG00000056763		-3.083149396	0.003425842	0.010659933
ENSMMUG00000033290	mml-mir-643 [Source:miRBase;Acc:MI0007886]	-0.781653647	0.000572054	0.002189306
ENSMMUG00000058137	zinc finger protein 816 [Source:NCBI gene;Acc:720058]	1.773045791	6.33E-08	5.26E-07
ENSMMUG00000046400	endogenous retrovirus group V, member 1 [Source:NCBI gene;Acc:100428948]	-1.215100942	0.01154182	0.030636334
ENSMMUG00000064519		-1.656030753	8.13E-05	0.000378145
ENSMMUG00000058242	zinc finger protein 160 [Source:NCBI gene;Acc:722722]	-2.383592768	0.011900893	0.031448726
ENSMMUG00000057187		0.751722258	4.91E-06	2.93E-05
ENSMMUG00000060222		-0.333058044	0.013151872	0.034211938
ENSMMUG00000007972	protein kinase C gamma [Source:VGNC Symbol;Acc:VGNC:76413]	0.528687267	0.000647054	0.002442925
ENSMMUG00000055976	CD99 antigen [Source:NCBI gene;Acc:106995376]	1.14197823	3.57E-05	0.000179154
ENSMMUG00000038768	U6 spliceosomal RNA [Source:RFAM;Acc:RF00026]	-1.614915042	4.87E-05	0.000237302
ENSMMUG00000042225		-0.521985593	0.001385643	0.004813559

SD 2 vs. Baseline

Ensembl Gene ID	Description	Log2(FC)	p-value	FDR
ENSMMUG00000000632	SH3 binding domain protein 5 like [Source:VGNC Symbol;Acc:VGNC:77334]	5.183920678	7.52E-20	5.52E-18
ENSMMUG00000064799		1.711049402	0.001860073	0.007745598
ENSMMUG00000049101	olfactory receptor 2T10 [Source:NCBI gene;Acc:100431025]	0.594080452	0.006834955	0.023395275
ENSMMUG00000017368	olfactory receptor 2G6 [Source:NCBI gene;Acc:694380]	-0.702042117	6.64E-05	0.000414917
ENSMMUG00000038524	olfactory receptor 2M4 [Source:NCBI gene;Acc:100424541]	2.04559061	2.69E-24	2.99E-22
ENSMMUG00000049432	olfactory receptor 2T33 [Source:NCBI gene;Acc:706331]	0.874760839	5.47E-10	1.01E-08
ENSMMUG00000064763		0.693724414	0.005780996	0.020348267
ENSMMUG00000005949	olfactory receptor 2B11 [Source:NCBI gene;Acc:709256]	-1.29993007	0.005688274	0.020065624
ENSMMUG00000052686		1.16405897	0.005923475	0.020749292
ENSMMUG00000004566	zinc finger protein 124 [Source:VGNC Symbol;Acc:VGNC:78836]	-3.327315437	0.001729649	0.007279647
ENSMMUG00000011380	consortin, connexin sorting protein [Source:VGNC Symbol;Acc:VGNC:71355]	-4.720062773	3.99E-05	0.0002658
ENSMMUG00000059690		-3.435285327	0.008241502	0.027516471
ENSMMUG00000000021	catsper channel auxiliary subunit epsilon [Source:VGNC Symbol;Acc:VGNC:70657]	-2.945424687	0.000882106	0.004068247
ENSMMUG00000063991		-1.616382556	0.009618613	0.031379213
ENSMMUG00000005668	kynurenine 3-monooxygenase [Source:VGNC Symbol;Acc:VGNC:74197]	1.089881152	7.91E-06	6.18E-05
ENSMMUG00000005667	fumarate hydratase [Source:VGNC Symbol;Acc:VGNC:72659]	0.820123226	0.000138154	0.000796327
ENSMMUG00000064824	gremlin 2, DAN family BMP antagonist [Source:NCBI gene;Acc:708280]	1.737101626	6.39E-06	5.09E-05
ENSMMUG00000061845		1.947801872	3.66E-07	3.93E-06
ENSMMUG00000001060	ryanodine receptor 2 [Source:VGNC Symbol;Acc:VGNC:76954]	1.432684069	7.21E-35	2.76E-32
ENSMMUG00000015132	galectin 8 [Source:VGNC Symbol;Acc:VGNC:74258]	-0.932771259	0.004967085	0.017872901
ENSMMUG00000052225		2.604956735	0.00450172	0.016393164
ENSMMUG00000046785		-1.7305501	0.000386705	0.001967994
ENSMMUG00000012615	pecanex 2 [Source:NCBI gene;Acc:712828]	-1.230815593	0.00021918	0.001201441
ENSMMUG00000063981	nucleoside-triphosphatase, cancer-related [Source:NCBI gene;Acc:712959]	2.453232218	1.67E-10	3.36E-09
ENSMMUG00000040055		-0.737719895	0.000836644	0.003886956
ENSMMUG00000061796		-1.207922105	6.53E-05	0.000408993
ENSMMUG00000012068	signal induced proliferation associated 1 like 2 [Source:NCBI gene;Acc:100425123]	1.597630175	0.000492963	0.002440804
ENSMMUG00000064226		1.360885497	0.005789978	0.020370017
ENSMMUG00000016204	translin associated factor X [Source:NCBI gene;Acc:713347]	-2.731539136	0.007443354	0.025304967
ENSMMUG00000005801	glyceronephosphate O-acyltransferase [Source:VGNC Symbol;Acc:VGNC:73100]	-1.564660823	5.22E-12	1.37E-10
ENSMMUG00000005888	tetratricopeptide repeat domain 13 [Source:VGNC Symbol;Acc:VGNC:79588]	-0.466041065	0.006382789	0.02209205
ENSMMUG00000009508	TATA-box binding protein associated factor 5 like [Source:VGNC Symbol;Acc:VGNC:78148]	1.539030577	0.003967597	0.014747367
ENSMMUG00000005185	regulator of G protein signaling 4 [Source:VGNC Symbol;Acc:VGNC:76690]	2.909444243	0.00564514	0.019952199
ENSMMUG00000005186	regulator of G protein signaling 5 [Source:NCBI gene;Acc:693510]	0.770362216	0.012507851	0.038976769
ENSMMUG00000023462	immunoglobulin like domain containing receptor 2 [Source:VGNC Symbol;Acc:VGNC:73619]	-1.880538726	0.001291078	0.005637851
ENSMMUG00000023463	maelstrom spermatogenic transposon silencer [Source:VGNC Symbol;Acc:VGNC:74362]	-1.952309665	0.003611531	0.013640121

ENSMMUG00000063120	cellular repressor of E1A stimulated genes 1 [Source:NCBI gene;Acc:701021]	-0.499265064	0.002039366	0.008407603
ENSMMUG00000023372	RCSD domain containing 1 [Source:VGNC Symbol;Acc:VGNC:76850]	-0.661381597	0.00126773	0.005550901
ENSMMUG00000009184	myelin protein zero like 1 [Source:NCBI gene;Acc:698437]	2.073877945	2.93E-08	3.88E-07
ENSMMUG00000060218	chemokine (C motif) ligand 1 [Source:NCBI gene;Acc:574367]	4.98232562	6.70E-08	8.32E-07
ENSMMUG00000056707		1.657810554	0.002098361	0.008614517
ENSMMUG00000059777		0.676296382	1.48E-05	0.000108536
ENSMMUG00000021078	ATPase Na+/K+ transporting subunit beta 1 [Source:VGNC Symbol;Acc:VGNC:70176]	-1.005717704	0.003905291	0.01454926
ENSMMUG00000021079	NME/NM23 family member 7 [Source:VGNC Symbol;Acc:VGNC:75221]	1.211340786	6.00E-05	0.000379955
ENSMMUG00000021080	basic leucine zipper nuclear factor 1 [Source:VGNC Symbol;Acc:VGNC:70258]	1.275320581	4.74E-33	1.32E-30
ENSMMUG00000000065	coiled-coil domain containing 181 [Source:VGNC Symbol;Acc:VGNC:70884]	-0.689988341	2.11E-08	2.86E-07
ENSMMUG00000013039	solute carrier family 19 member 2 [Source:VGNC Symbol;Acc:VGNC:81571]	-0.566065915	7.32E-06	5.78E-05
ENSMMUG00000042510	coagulation factor V [Source:NCBI gene;Acc:704695]	0.913152943	4.75E-07	4.96E-06
ENSMMUG00000013044	selectin P [Source:VGNC Symbol;Acc:VGNC:77157]	1.206591521	5.55E-07	5.71E-06
ENSMMUG00000016579	selectin L [Source:NCBI gene;Acc:701419]	2.33408769	2.03E-26	3.01E-24
ENSMMUG00000016580	selectin E [Source:VGNC Symbol;Acc:VGNC:77156]	2.441864301	1.13E-31	2.73E-29
ENSMMUG00000016582	chromosome 1 C1orf112 homolog [Source:VGNC Symbol;Acc:VGNC:70385]	1.373552403	2.69E-09	4.35E-08
ENSMMUG00000059773	methyltransferase like 11B [Source:NCBI gene;Acc:705503]	-1.264920679	0.001646783	0.006979384
ENSMMUG00000063298		-1.014461481	0.001067249	0.004781077
ENSMMUG00000014674	golgin, RAB6 interacting [Source:VGNC Symbol;Acc:VGNC:73012]	1.052454039	2.18E-08	2.95E-07
ENSMMUG00000007279	phosphatidylinositol glycan anchor biosynthesis class C [Source:VGNC Symbol;Acc:VGNC:75986]	0.712212069	0.000936191	0.004283731
ENSMMUG00000065134	Fas ligand [Source:NCBI gene;Acc:574159]	4.339549147	5.35E-06	4.35E-05
ENSMMUG00000000629	serpin family C member 1 [Source:VGNC Symbol;Acc:VGNC:77360]	0.449763852	8.28E-05	0.00050528
ENSMMUG00000000630	ring finger and CCCH-type domains 1 [Source:VGNC Symbol;Acc:VGNC:76861]	-0.534929885	4.07E-06	3.40E-05
ENSMMUG00000064770		-0.951425015	0.003158858	0.012154664
ENSMMUG00000000546	KIAA0040 ortholog [Source:NCBI gene;Acc:100423509]	-0.92251083	0.00029036	0.001532571
ENSMMUG00000015723	tenascin R [Source:VGNC Symbol;Acc:VGNC:78609]	-0.893596724	0.011346257	0.035973946
ENSMMUG00000023216	astrotactin 1 [Source:VGNC Symbol;Acc:VGNC:70167]	-0.58678464	0.000874432	0.004039282
ENSMMUG00000022935	BMP/retinoic acid inducible neural specific 2 [Source:VGNC Symbol;Acc:VGNC:70303]	-0.639030675	0.000853343	0.003955689
ENSMMUG00000020320	protein transport protein Sec16B [Source:NCBI gene;Acc:709635]	0.47265303	0.00537906	0.019132713
ENSMMUG00000021073	RAS protein activator like 2 [Source:VGNC Symbol;Acc:VGNC:76670]	2.349096963	9.06E-16	4.32E-14
ENSMMUG00000056058	C-type lectin domain containing 20A [Source:HGNC Symbol;Acc:HGNC:34521]	2.994345656	2.21E-05	0.000156356
ENSMMUG00000054486	testis expressed 35 [Source:NCBI gene;Acc:717255]	2.311587114	0.001115804	0.004964934
ENSMMUG00000055256	family with sequence similarity 163 member A [Source:NCBI gene;Acc:718704]	1.185753197	8.35E-09	1.24E-07
ENSMMUG00000031279	torsin A interacting protein 2 [Source:NCBI gene;Acc:716654]	1.213282777	6.94E-10	1.26E-08
ENSMMUG00000053617	torsin 1A interacting protein 2 [Source:NCBI gene;Acc:716691]	0.463092292	0.009915493	0.032217597
ENSMMUG00000014873	torsin 1A interacting protein 1 [Source:VGNC Symbol;Acc:VGNC:79810]	0.460977536	0.001219837	0.00536896
ENSMMUG00000054568		1.121160441	4.10E-06	3.42E-05
ENSMMUG00000061785		1.120064891	5.44E-19	3.75E-17
ENSMMUG00000022884	glutamate-ammonia ligase [Source:NCBI gene;Acc:715952]	2.8836619	1.70E-07	1.97E-06
ENSMMUG00000031291	regulator of G protein signaling like 1 [Source:NCBI gene;Acc:715693]	1.307077169	7.14E-14	2.49E-12
ENSMMUG00000005634	ribonuclease L [Source:VGNC Symbol;Acc:VGNC:82223]	-1.053424566	6.46E-09	9.80E-08

ENSMMUG00000055481	regulator of G protein signaling 16 [Source:NCBI gene;Acc:715599]	-0.706897883	0.000731881	0.003449805
ENSMMUG00000008990	regulator of G protein signaling 8 [Source:NCBI gene;Acc:720353]	2.553992504	0.001685435	0.007122435
ENSMMUG00000001479	laminin subunit gamma 2 [Source:VGNC Symbol;Acc:VGNC:74233]	-1.886600245	2.70E-09	4.36E-08
ENSMMUG00000001481	nicotinamide nucleotide adenylyltransferase 2 [Source:VGNC Symbol;Acc:VGNC:75193]	-3.398208368	5.72E-08	7.20E-07
ENSMMUG00000013878	ring finger protein 2 [Source:NCBI gene;Acc:717024]	-1.062396609	0.000196023	0.001085145
ENSMMUG00000016857	prostaglandin-endoperoxide synthase 2 [Source:VGNC Symbol;Acc:VGNC:76589]	-1.022212515	1.32E-09	2.26E-08
ENSMMUG00000059986		-1.015338042	1.56E-05	0.000113954
ENSMMUG00000062021		-1.026832918	0.000963062	0.004385973
ENSMMUG00000022647	cell division cycle 73 [Source:VGNC Symbol;Acc:VGNC:70766]	-1.067165007	1.31E-06	1.23E-05
ENSMMUG00000021082	potassium sodium-activated channel subfamily T member 2 [Source:VGNC Symbol;Acc:VGNC:73857]	0.729079785	4.28E-08	5.50E-07
ENSMMUG00000045497	complement factor H [Source:NCBI gene;Acc:717346]	-1.135734228	6.34E-05	0.000398565
ENSMMUG00000000244	coagulation factor XIII B chain [Source:VGNC Symbol;Acc:VGNC:72463]	-0.628472069	0.001459785	0.006269097
ENSMMUG00000000009	protein tyrosine phosphatase receptor type C [Source:VGNC Symbol;Acc:VGNC:76612]	-3.023444452	4.35E-14	1.62E-12
ENSMMUG00000012218	calmodulin regulated spectrin associated protein family member 2 [Source:VGNC Symbol;Acc:VGNC:70666]	-0.737470518	0.004014258	0.014883234
ENSMMUG00000017414	calcium voltage-gated channel subunit alpha1 S [Source:VGNC Symbol;Acc:VGNC:70507]	-1.60348404	5.94E-10	1.09E-08
ENSMMUG00000064915	Metazoan signal recognition particle RNA [Source:RFAM;Acc:RF00017]	1.990308797	3.58E-05	0.000240295
ENSMMUG00000050920	Metazoan signal recognition particle RNA [Source:RFAM;Acc:RF00017]	1.215378646	0.003672433	0.013834194
ENSMMUG00000021642	fibromodulin [Source:VGNC Symbol;Acc:VGNC:72686]	0.897366575	0.015735223	0.047068224
ENSMMUG00000055783		-1.83327105	2.07E-05	0.000146873
ENSMMUG00000064841	golgi transport 1A [Source:NCBI gene;Acc:700305]	-2.80726475	0.008651632	0.028727451
ENSMMUG00000017114	pleckstrin homology domain containing A6 [Source:VGNC Symbol;Acc:VGNC:76040]	-1.48342277	5.27E-05	0.000339208
ENSMMUG00000063658	leucine rich repeat neuronal 2 [Source:NCBI gene;Acc:100429709]	1.28493269	0.000432073	0.002179406
ENSMMUG00000054201	U6 spliceosomal RNA [Source:RFAM;Acc:RF00026]	-1.91671099	0.00520953	0.018602538
ENSMMUG00000001024	ETS transcription factor ELK4 [Source:VGNC Symbol;Acc:VGNC:72211]	-1.54053313	0.001262	0.005534146
ENSMMUG00000004116	solute carrier family 45 member 3 [Source:VGNC Symbol;Acc:VGNC:77817]	-1.528332821	0.003208844	0.012317657
ENSMMUG00000055835	nuclear casein kinase and cyclin dependent kinase substrate 1 [Source:NCBI gene;Acc:697298]	-1.179456665	3.76E-07	4.02E-06
ENSMMUG00000023570	interleukin 19 [Source:NCBI gene;Acc:694806]	3.742371896	0.00089386	0.004114644
ENSMMUG00000011221	Fc fragment of IgA and IgM receptor [Source:VGNC Symbol;Acc:VGNC:72567]	1.084544549	0.009155177	0.030162714
ENSMMUG00000040888	complement C3b/C4b receptor 1 like [Source:HGNC Symbol;Acc:HGNC:2335]	0.983259927	1.83E-08	2.52E-07
ENSMMUG00000051230		0.695056689	0.001220269	0.00536896
ENSMMUG00000057560		0.846364771	1.26E-06	1.18E-05
ENSMMUG00000064202		-1.122399821	0.000177148	0.000992751
ENSMMUG00000045482	G0/G1 switch 2 [Source:NCBI gene;Acc:717581]	-0.450573241	5.14E-07	5.32E-06
ENSMMUG00000016099	hedgehog acyltransferase [Source:NCBI gene;Acc:712333]	1.315244432	0.001264179	0.005542029
ENSMMUG00000011914	prospero homeobox 1 [Source:VGNC Symbol;Acc:VGNC:76427]	3.244098013	3.60E-05	0.000241744
ENSMMUG00000015634	centromere protein F [Source:VGNC Symbol;Acc:VGNC:71009]	-2.17105515	0.000299115	0.001569761
ENSMMUG00000058133		-1.887606245	0.000225634	0.001229861

ENSMMUG00000017396	3'(2'), 5'-bisphosphate nucleotidase 1 [Source:NCBI gene;Acc:721277]	-0.728338606	0.01041944	0.033525322
ENSMMUG00000007783	isoleucyl-tRNA synthetase 2, mitochondrial [Source:VGNC Symbol;Acc:VGNC:73458]	-0.974736505	8.90E-13	2.61E-11
ENSMMUG00000026897	mml-mir-215 [Source:miRBase;Acc:MI0003025]	-1.305962497	0.011327861	0.035930409
ENSMMUG00000007784	RAB3 GTPase activating non-catalytic protein subunit 2 [Source:VGNC Symbol;Acc:VGNC:76499]	0.378715103	0.015350745	0.046136124
ENSMMUG00000022288	microtubule affinity regulating kinase 1 [Source:VGNC Symbol;Acc:VGNC:74658]	2.22378655	0.000336654	0.001742237
ENSMMUG00000018525	presenilin 2 [Source:VGNC Symbol;Acc:VGNC:76436]	-0.907935522	1.52E-10	3.09E-09
ENSMMUG00000008638	CDC42 binding protein kinase alpha [Source:VGNC Symbol;Acc:VGNC:70799]	0.472349166	5.86E-05	0.000372483
ENSMMUG00000023096	obscurin, cytoskeletal calmodulin and titin-interacting RhoGEF [Source:NCBI gene;Acc:694413]	-0.748571746	1.48E-12	4.19E-11
ENSMMUG00000019513	U2AF homology motif kinase 1 [Source:VGNC Symbol;Acc:VGNC:79198]	2.008942035	0.001400723	0.006054727
ENSMMUG00000042262	nitric oxide synthase 1 adaptor protein [Source:NCBI gene;Acc:720378]	0.991744039	0.00060953	0.0029421
ENSMMUG00000004790	spermatogenesis associated 46 [Source:VGNC Symbol;Acc:VGNC:78006]	3.362455509	0.015149634	0.045634112
ENSMMUG00000023042	olfactomedin like 2B [Source:VGNC Symbol;Acc:VGNC:75455]	0.472074233	0.000144886	0.000832166
ENSMMUG00000004049	activating transcription factor 6 [Source:VGNC Symbol;Acc:VGNC:70171]	0.370354702	0.000577952	0.002806175
ENSMMUG00000004042	Fc receptor like A [Source:VGNC Symbol;Acc:VGNC:72526]	2.057239677	4.62E-06	3.80E-05
ENSMMUG00000054686		3.136689124	7.56E-24	7.79E-22
ENSMMUG00000008251	cilia and flagella associated protein 126 [Source:VGNC Symbol;Acc:VGNC:71150]	2.85443535	2.00E-05	0.000142637
ENSMMUG00000012411	succinate dehydrogenase complex subunit C [Source:VGNC Symbol;Acc:VGNC:77147]	2.072832034	0.004628515	0.016791794
ENSMMUG00000018128	nuclear receptor subfamily 1 group I member 3 [Source:NCBI gene;Acc:574249]	1.112355534	0.000441966	0.002224673
ENSMMUG00000060496	translocase of outer mitochondrial membrane 40 like [Source:NCBI gene;Acc:100426891]	1.236033472	9.74E-05	0.000582658
ENSMMUG00000004516	apolipoprotein A2 [Source:NCBI gene;Acc:719946]	1.303845284	0.002638909	0.010395347
ENSMMUG00000004512	Fc fragment of IgE receptor Ig [Source:VGNC Symbol;Acc:VGNC:72629]	1.569468957	3.84E-10	7.35E-09
ENSMMUG00000004520	NADH:ubiquinone oxidoreductase core subunit S2 [Source:NCBI gene;Acc:719935]	1.230007433	2.53E-08	3.40E-07
ENSMMUG00000050419	ADAM metallopeptidase with thrombospondin type 1 motif 4 [Source:NCBI gene;Acc:719927]	1.416009281	1.11E-06	1.06E-05
ENSMMUG00000060555		1.364117462	6.32E-06	5.06E-05
ENSMMUG00000022630	protoporphyrinogen oxidase [Source:VGNC Symbol;Acc:VGNC:76365]	-1.247724069	6.38E-06	5.09E-05
ENSMMUG00000022631	ubiquitin specific peptidase 21 [Source:VGNC Symbol;Acc:VGNC:78741]	-1.210593004	0.000191073	0.001061442
ENSMMUG00000022632	ubiquitin-fold modifier conjugating enzyme 1 [Source:VGNC Symbol;Acc:VGNC:79643]	-0.852538157	0.000678097	0.003234086
ENSMMUG00000002007	kelch domain containing 9 [Source:NCBI gene;Acc:719863]	-0.802272785	0.006408398	0.022149044
ENSMMUG00000064337	nectin cell adhesion molecule 4 [Source:NCBI gene;Acc:719843]	-0.681834512	0.000775745	0.003624493
ENSMMUG00000002005	Rho GTPase activating protein 30 [Source:NCBI gene;Acc:718236]	-0.673790898	0.000755176	0.003544719
ENSMMUG00000002003	upstream transcription factor 1 [Source:NCBI gene;Acc:719835]	-0.743572749	9.68E-05	0.000579822
ENSMMUG00000011335	Fc receptor like 6 [Source:VGNC Symbol;Acc:VGNC:72602]	-0.646241805	1.09E-08	1.59E-07
ENSMMUG00000004492	absent in melanoma 2 [Source:NCBI gene;Acc:719260]	-1.106692523	0.00049229	0.002438302
ENSMMUG00000060051	olfactory receptor 6N1-like [Source:NCBI gene;Acc:719970]	-0.741277515	0.002280707	0.009230134
ENSMMUG00000010626	CD1c molecule [Source:VGNC Symbol;Acc:VGNC:70756]	1.475265489	0.002708237	0.010614623
ENSMMUG00000003191	kirre like nephrin family adhesion molecule 1 [Source:VGNC Symbol;Acc:VGNC:73837]	-1.196718292	4.88E-16	2.40E-14
ENSMMUG00000050541		-0.650995103	0.016869909	0.049835826

ENSMMUG00000019835	CD5 molecule like [Source:VGNC Symbol;Acc:VGNC:70774]	1.158416134	6.30E-07	6.37E-06
ENSMMUG00000019833	Fc receptor like 1 [Source:VGNC Symbol;Acc:VGNC:72528]	1.164750556	1.03E-14	4.06E-13
ENSMMUG00000019830	Fc receptor like 2 [Source:VGNC Symbol;Acc:VGNC:72509]	1.153656	9.29E-08	1.12E-06
ENSMMUG00000046969	Fc receptor like 3 [Source:NCBI gene;Acc:718801]	1.264432255	6.94E-09	1.05E-07
ENSMMUG00000008973	Fc receptor like 4 [Source:VGNC Symbol;Acc:VGNC:72527]	1.151166464	0.006767524	0.023224621
ENSMMUG00000008974	Fc receptor like 5 [Source:NCBI gene;Acc:718771]	-1.711097499	8.74E-12	2.20E-10
ENSMMUG00000042886	ETS variant transcription factor 3 [Source:NCBI gene;Acc:719741]	0.850900624	0.006833961	0.023395275
ENSMMUG00000064007	leucine rich repeat containing 71 [Source:NCBI gene;Acc:718714]	0.224112581	0.014915505	0.04505958
ENSMMUG00000000037	interferon stimulated exonuclease gene 20 like 2 [Source:NCBI gene;Acc:718621]	-1.087062765	0.005133661	0.018369293
ENSMMUG00000060011	hyaluronan and proteoglycan link protein 2 [Source:NCBI gene;Acc:718500]	-2.231799345	0.002044606	0.008422341
ENSMMUG00000004648	KH domain containing 4, pre-mRNA splicing factor [Source:VGNC Symbol;Acc:VGNC:73931]	-0.850627008	5.39E-07	5.55E-06
ENSMMUG00000009987	GON-4-like protein [Source:NCBI gene;Acc:719341]	-0.689168648	0.015735817	0.047068224
ENSMMUG00000062397		1.062770273	3.28E-07	3.56E-06
ENSMMUG00000023146	ASH1 like histone lysine methyltransferase [Source:NCBI gene;Acc:717778]	1.096075564	0.006838276	0.023401133
ENSMMUG00000023145	RUN and SH3 domain containing 1 [Source:VGNC Symbol;Acc:VGNC:77036]	-0.858252247	0.009441868	0.030924165
ENSMMUG00000023143	pyruvate kinase L/R [Source:VGNC Symbol;Acc:VGNC:76011]	-2.182437857	0.002789472	0.010918296
ENSMMUG00000061381	hyperpolarization activated cyclic nucleotide gated potassium channel 3 [Source:NCBI gene;Acc:717719]	-1.715132485	0.003438244	0.013080805
ENSMMUG00000023141	CDC like kinase 2 [Source:VGNC Symbol;Acc:VGNC:71259]	-1.02836651	0.000104516	0.00062065
ENSMMUG00000003902	thrombospondin 3 [Source:VGNC Symbol;Acc:VGNC:78345]	1.650910644	2.67E-06	2.32E-05
ENSMMUG00000010978	S100 calcium binding protein A2 [Source:VGNC Symbol;Acc:VGNC:76956]	-0.803550213	5.12E-09	7.88E-08
ENSMMUG00000010979	S100 calcium binding protein A4 [Source:VGNC Symbol;Acc:VGNC:76958]	-0.527962844	0.005091024	0.0182466
ENSMMUG00000010980	S100 calcium binding protein A5 [Source:VGNC Symbol;Acc:VGNC:76959]	-1.095742065	0.002554672	0.010136873
ENSMMUG00000008917	S100 calcium binding protein A6 [Source:VGNC Symbol;Acc:VGNC:76960]	-0.773910023	1.84E-05	0.000131591
ENSMMUG00000040678	Metazoan signal recognition particle RNA [Source:RFAM;Acc:RF00017]	-2.155181552	0.003439301	0.013081402
ENSMMUG00000051645		3.282140284	1.27E-06	1.20E-05
ENSMMUG00000061474		1.771065535	2.46E-05	0.000171881
ENSMMUG00000036441		2.841683229	0.010448232	0.033600545
ENSMMUG00000042618	involucrin [Source:NCBI gene;Acc:713728]	1.815114049	1.18E-05	8.94E-05
ENSMMUG00000017740	sperm mitochondria associated cysteine rich protein [Source:VGNC Symbol;Acc:VGNC:77816]	2.5524791	5.91E-08	7.41E-07
ENSMMUG00000055038		2.553580048	4.45E-09	6.91E-08
ENSMMUG00000054634	late cornified envelope protein 1C-like [Source:NCBI gene;Acc:713600]	2.602533034	0.003523995	0.013347659
ENSMMUG00000051719	late cornified envelope 1C [Source:HGNC Symbol;Acc:HGNC:29464]	2.665963207	2.60E-05	0.000180273
ENSMMUG00000058472	late cornified envelope protein 1C [Source:NCBI gene;Acc:107000798]	1.990828071	3.90E-06	3.28E-05
ENSMMUG00000056940	late cornified envelope protein 1C [Source:NCBI gene;Acc:713408]	2.700482829	2.08E-05	0.000147386
ENSMMUG00000055266	late cornified envelope protein 1C-like [Source:NCBI gene;Acc:107000794]	2.688971816	8.89E-08	1.07E-06
ENSMMUG00000051816	keratinocyte proline rich protein [Source:NCBI gene;Acc:100424312]	2.128953349	6.59E-08	8.19E-07
ENSMMUG00000001966	chromosome 1 C1orf68 homolog [Source:VGNC Symbol;Acc:VGNC:70436]	1.799987177	0.006658559	0.02291017
ENSMMUG00000049843	late cornified envelope protein 2D [Source:NCBI gene;Acc:100424118]	3.849368225	1.04E-05	7.92E-05
ENSMMUG00000062996		2.63102025	2.61E-07	2.89E-06
ENSMMUG00000001967	late cornified envelope 5A [Source:VGNC Symbol;Acc:VGNC:74061]	2.537882644	0.005990007	0.020936941
ENSMMUG00000009040	cornulin [Source:VGNC Symbol;Acc:VGNC:71397]	2.517407335	2.35E-07	2.63E-06

ENSMMUG00000056511		2.324057821	5.87E-07	6.00E-06
ENSMMUG00000059472	filaggrin family member 2 [Source:HGNC Symbol;Acc:HGNC:33276]	1.853103173	1.53E-08	2.15E-07
ENSMMUG00000010067	MINDY lysine 48 deubiquitinase 1 [Source:VGNC Symbol;Acc:VGNC:74828]	-2.028230077	0.015576972	0.046698791
ENSMMUG00000008218	ADAMTS like 4 [Source:VGNC Symbol;Acc:VGNC:69480]	1.07508721	1.05E-05	8.02E-05
ENSMMUG00000021388	circadian associated repressor of transcription [Source:VGNC Symbol;Acc:VGNC:71345]	-0.531798248	0.014216043	0.043356122
ENSMMUG00000021387	chromosome 1 C1orf54 homolog [Source:VGNC Symbol;Acc:VGNC:70396]	-0.703047939	0.007480613	0.025407866
ENSMMUG00000021385	carbonic anhydrase 14 [Source:VGNC Symbol;Acc:VGNC:70489]	-0.440790478	0.001916782	0.007956672
ENSMMUG00000007244	OTU deubiquitinase 7B [Source:VGNC Symbol;Acc:VGNC:75634]	-1.101902983	2.14E-08	2.90E-07
ENSMMUG00000052282		-1.360717906	7.43E-09	1.11E-07
ENSMMUG00000007246	myotubularin related protein 11 [Source:VGNC Symbol;Acc:VGNC:75016]	-1.099709032	1.47E-08	2.08E-07
ENSMMUG00000007256	splicing factor 3b subunit 4 [Source:VGNC Symbol;Acc:VGNC:77182]	-1.28255906	3.47E-09	5.50E-08
ENSMMUG00000007257	synaptic vesicle glycoprotein 2A [Source:VGNC Symbol;Acc:VGNC:78181]	-1.035907471	2.24E-11	5.20E-10
ENSMMUG00000061036	histone cluster 2, H2ab [Source:NCBI gene;Acc:705170]	-1.457098258	0.001126874	0.005011124
ENSMMUG00000037540	histone cluster 2, H2ac [Source:NCBI gene;Acc:705054]	-1.384507697	0.004233813	0.01556968
ENSMMUG00000052382	histone H4 [Source:NCBI gene;Acc:107000818]	-1.42424592	9.29E-15	3.73E-13
ENSMMUG00000054615		-1.136293764	7.56E-05	0.000467389
ENSMMUG00000051523	Metazoan signal recognition particle RNA [Source:RFAM;Acc:RF00017]	-1.082656899	0.002810838	0.010990089
ENSMMUG00000054078	histone H3 [Source:NCBI gene;Acc:114669830]	-1.097540753	8.41E-05	0.000512441
ENSMMUG00000040506	H2B clustered histone 18 [Source:HGNC Symbol;Acc:HGNC:24700]	-0.983165821	0.000354946	0.001825204
ENSMMUG00000004362	Fc fragment of IgG receptor Ia [Source:NCBI gene;Acc:714664]	-1.072411782	3.97E-06	3.32E-05
ENSMMUG00000062140		-1.303918595	4.42E-07	4.66E-06
ENSMMUG00000024186	U1 spliceosomal RNA [Source:RFAM;Acc:RF00003]	-1.681000328	0.000100883	0.000600794
ENSMMUG00000036857	U1 spliceosomal RNA [Source:RFAM;Acc:RF00003]	-1.619839111	0.001993044	0.008244987
ENSMMUG00000064626	U1 spliceosomal RNA [Source:RFAM;Acc:RF00003]	-1.645098309	0.000762679	0.003576472
ENSMMUG00000061239		-1.415493801	3.43E-12	9.20E-11
ENSMMUG00000054590		-1.297549494	5.96E-09	9.09E-08
ENSMMUG00000054496	U1 spliceosomal RNA [Source:RFAM;Acc:RF00003]	-1.384076257	0.010772532	0.034447931
ENSMMUG00000048493	neuroblastoma breakpoint family member 6-like protein [Source:NCBI gene;Acc:711999]	-2.764071741	0.009479176	0.031021789
ENSMMUG00000041725	60S ribosomal protein L6-like [Source:NCBI gene;Acc:710287]	-1.912406978	0.009952705	0.032295193
ENSMMUG00000049105	NRAS proto-oncogene, GTPase [Source:NCBI gene;Acc:709089]	-1.398108084	0.000715899	0.003384398
ENSMMUG00000001978	adenosine monophosphate deaminase 1 [Source:VGNC Symbol;Acc:VGNC:69806]	-0.68513364	1.67E-05	0.000120707

ENSMMUG00000021535	homeodomain interacting protein kinase 1 [Source:VGNC Symbol;Acc:VGNC:73420]	- 0.199488966	0.015604394	0.046771355
ENSMMUG00000019528	adaptor related protein complex 4 subunit beta 1 [Source:VGNC Symbol;Acc:VGNC:69972]	2.725579808	6.24E-79	9.06E-75
ENSMMUG00000019527	DNA cross-link repair 1B [Source:VGNC Symbol;Acc:VGNC:71647]	1.249162058	2.31E-05	0.000162733
ENSMMUG00000006786	protein tyrosine phosphatase non-receptor type 22 [Source:VGNC Symbol;Acc:VGNC:76603]	1.167961683	4.94E-07	5.14E-06
ENSMMUG00000017819	putative homeodomain transcription factor 1 [Source:VGNC Symbol;Acc:VGNC:75982]	- 1.062095441	7.26E-07	7.22E-06
ENSMMUG00000017817	membrane associated guanylate kinase, WW and PDZ domain containing 3 [Source:VGNC Symbol;Acc:VGNC:74400]	0.507907975	0.003193757	0.012266222
ENSMMUG00000007766	suppression of tumorigenicity 7 like [Source:VGNC Symbol;Acc:VGNC:77954]	- 1.348813123	9.08E-09	1.34E-07
ENSMMUG00000007765	Wnt family member 2B [Source:VGNC Symbol;Acc:VGNC:78797]	- 1.167163759	1.67E-12	4.72E-11
ENSMMUG00000037458	endoplasmic reticulum resident protein 29 [Source:NCBI gene;Acc:720396]	- 1.787614633	0.001332403	0.00579394
ENSMMUG00000050825	potassium voltage-gated channel subfamily D member 3 [Source:NCBI gene;Acc:641437]	- 0.569716516	5.02E-06	4.10E-05
ENSMMUG00000065322	ATP synthase peripheral stalk-membrane subunit b [Source:NCBI gene;Acc:704072]	- 0.611927237	1.02E-06	9.84E-06
ENSMMUG00000016056	potassium voltage-gated channel subfamily A member 10 [Source:VGNC Symbol;Acc:VGNC:73963]	0.498518317	0.000510423	0.002519531
ENSMMUG00000062177		0.533574065	1.04E-05	7.96E-05
ENSMMUG00000064173	prokineticin 1 [Source:NCBI gene;Acc:701766]	1.260828833	0.000400394	0.002030888
ENSMMUG00000022715	adenosylhomocysteinase like 1 [Source:VGNC Symbol;Acc:VGNC:81350]	- 0.463858396	0.004014388	0.014883234
ENSMMUG00000051186	proteasome 20S subunit alpha 5 [Source:NCBI gene;Acc:698435]	0.746945996	9.41E-13	2.74E-11
ENSMMUG00000005812	sortilin 1 [Source:VGNC Symbol;Acc:VGNC:77836]	- 0.363004825	3.31E-05	0.00022412
ENSMMUG00000008713	myosin binding protein H like [Source:NCBI gene;Acc:698183]	-1.12628142	1.61E-18	1.03E-16
ENSMMUG00000015380	proline and serine rich coiled-coil 1 [Source:VGNC Symbol;Acc:VGNC:76307]	- 0.883227875	1.77E-09	2.96E-08
ENSMMUG00000015375	cadherin EGF LAG seven-pass G-type receptor 2 [Source:VGNC Symbol;Acc:VGNC:71149]	-0.87139498	1.55E-10	3.16E-09
ENSMMUG00000021837	seryl-tRNA synthetase 1 [Source:NCBI gene;Acc:695046]	- 0.879207523	1.13E-05	8.58E-05
ENSMMUG00000017266	UPF0577 protein KIAA1324 [Source:NCBI gene;Acc:697670]	1.348248907	5.54E-07	5.69E-06
ENSMMUG00000010156	fibronectin type III domain containing 7 [Source:VGNC Symbol;Acc:VGNC:72582]	0.47542484	0.006812908	0.023336297
ENSMMUG00000042232	NBPF member 6 [Source:NCBI gene;Acc:698524]	0.304806945	0.000246842	0.001330492
ENSMMUG00000031785		0.951846644	0.001492675	0.006393344
ENSMMUG00000002789	solute carrier family 25 (mitochondrial carrier; phosphate carrier), member 24 [Source:NCBI gene;Acc:694803]	1.376492012	0.000479245	0.002381806
ENSMMUG00000019714	vav guanine nucleotide exchange factor 3 [Source:VGNC Symbol;Acc:VGNC:78759]	- 1.500946894	0.003112986	0.012003576
ENSMMUG00000008197	netrin G1 [Source:VGNC Symbol;Acc:VGNC:75450]	2.097310805	1.52E-06	1.40E-05
ENSMMUG00000060795		1.767187763	2.36E-06	2.08E-05
ENSMMUG00000061766		- 1.587072229	0.004438644	0.016216276
ENSMMUG00000004103	collagen type XI alpha 1 chain [Source:VGNC Symbol;Acc:VGNC:71289]	1.7937565	7.72E-45	8.63E-42
ENSMMUG00000060790		- 3.374574504	0.000522502	0.00256956
ENSMMUG00000022906	vascular cell adhesion molecule 1 [Source:VGNC Symbol;Acc:VGNC:78761]	- 0.476521083	0.003341051	0.012761128
ENSMMUG00000062643		- 1.822981731	0.011216246	0.035616113
ENSMMUG00000055443	G protein-coupled receptor 88 [Source:NCBI gene;Acc:716520]	0.61948814	7.66E-07	7.57E-06
ENSMMUG00000019105	RNA 3'-terminal phosphate cyclase [Source:VGNC Symbol;Acc:VGNC:77043]	- 1.979246256	2.22E-06	1.96E-05
ENSMMUG00000007706	dihydrolipoamide branched chain transacylase E2 [Source:VGNC Symbol;Acc:VGNC:71744]	- 1.836457226	0.001860961	0.007747075

ENSMMUG00000000931	solute carrier family 35 member A3 [Source:VGNC Symbol;Acc:VGNC:77461]	1.968350371	1.28E-06	1.20E-05
ENSMMUG00000021441	amylo-alpha-1, 6-glucosidase, 4-alpha-glucanotransferase [Source:VGNC Symbol;Acc:VGNC:69744]	-0.417892166	0.006772856	0.023227606
ENSMMUG00000038667		-1.062436923	0.0021725	0.008871248
ENSMMUG00000008518	phospholipid phosphatase related 5 [Source:NCBI gene;Acc:710549]	1.774568889	2.22E-24	2.48E-22
ENSMMUG00000008515	sorting nexin 7 [Source:NCBI gene;Acc:716015]	0.884740137	4.28E-08	5.50E-07
ENSMMUG00000049494		1.572890796	1.93E-07	2.20E-06
ENSMMUG00000020726	dihydropyrimidine dehydrogenase [Source:VGNC Symbol;Acc:VGNC:71982]	1.576897775	1.71E-15	7.84E-14
ENSMMUG00000049297		1.093513756	1.92E-09	3.18E-08
ENSMMUG00000050661		0.733726032	0.000253037	0.001358845
ENSMMUG00000001690	calponin 3 [Source:VGNC Symbol;Acc:VGNC:71278]	1.248483325	5.16E-10	9.59E-09
ENSMMUG00000056548	coagulation factor III, tissue factor [Source:NCBI gene;Acc:714456]	0.581093937	1.00E-06	9.68E-06
ENSMMUG00000032677	microRNA 760 [Source:HGNC Symbol;Acc:HGNC:33666]	0.622394921	0.002419172	0.009682681
ENSMMUG00000045959		0.879693885	2.92E-05	0.000199874
ENSMMUG00000015924	BCAR3 adaptor protein, NSP family member [Source:VGNC Symbol;Acc:VGNC:70235]	0.807770394	2.98E-06	2.57E-05
ENSMMUG00000015922	formin binding protein 1 like [Source:VGNC Symbol;Acc:VGNC:72543]	0.805251008	1.58E-08	2.20E-07
ENSMMUG00000016812	coiled-coil domain containing 18 [Source:VGNC Symbol;Acc:VGNC:70913]	-0.70987559	0.000728017	0.003434984
ENSMMUG00000005070	ecotropic viral integration site 5 [Source:NCBI gene;Acc:706592]	1.783753952	2.23E-16	1.12E-14
ENSMMUG00000014018	bromodomain testis associated [Source:VGNC Symbol;Acc:VGNC:70282]	-2.309640447	6.79E-06	5.40E-05
ENSMMUG00000001742	transforming growth factor beta receptor 3 [Source:VGNC Symbol;Acc:VGNC:78339]	-2.77788796	1.49E-31	3.55E-29
ENSMMUG00000051155	U2 spliceosomal RNA [Source:RFAM;Acc:RF00004]	-4.300439592	3.83E-05	0.000255695
ENSMMUG00000006819	helicase for meiosis 1 [Source:NCBI gene;Acc:704516]	0.756158276	0.000221966	0.001213055
ENSMMUG00000007635	zinc finger protein 644 [Source:VGNC Symbol;Acc:VGNC:79789]	1.366909039	0.009237313	0.030373855
ENSMMUG00000049678		2.257310028	4.01E-11	8.96E-10
ENSMMUG00000060600		-0.818634893	0.000566276	0.002754085
ENSMMUG00000053737	BarH like homeobox 2 [Source:NCBI gene;Acc:696831]	0.987881951	1.45E-08	2.05E-07
ENSMMUG00000000663	guanylate-binding protein 6-like [Source:NCBI gene;Acc:695158]	-1.116043142	1.29E-05	9.62E-05
ENSMMUG00000000662	guanylate-binding protein 1 [Source:NCBI gene;Acc:694538]	-0.610570824	0.000218311	0.001197129
ENSMMUG00000037887	RBMX like 1 [Source:NCBI gene;Acc:100424767]	0.821784137	5.35E-06	4.35E-05
ENSMMUG00000002794	general transcription factor IIB [Source:VGNC Symbol;Acc:VGNC:73306]	-2.121769339	1.89E-37	9.49E-35
ENSMMUG00000020588	protein kinase N2 [Source:VGNC Symbol;Acc:VGNC:76015]	0.632525703	2.31E-07	2.59E-06
ENSMMUG00000065295		2.236989968	1.41E-12	3.99E-11
ENSMMUG00000006836	chloride channel accessory 1 [Source:NCBI gene;Acc:574295]	1.88692002	4.07E-10	7.75E-09
ENSMMUG00000001380	outer dense fiber of sperm tails 2 like [Source:VGNC Symbol;Acc:VGNC:75482]	2.26454911	2.42E-05	0.000169301
ENSMMUG00000013203	zinc finger HIT-type containing 6 [Source:VGNC Symbol;Acc:VGNC:79576]	-0.779236848	0.004422544	0.016165581
ENSMMUG00000014573	dimethylarginine dimethylaminohydrolase 1 [Source:VGNC Symbol;Acc:VGNC:71753]	0.699514617	1.28E-11	3.11E-10
ENSMMUG00000005720	BCL10 immune signaling adaptor [Source:VGNC Symbol;Acc:VGNC:70240]	0.451606802	0.004307891	0.015822086
ENSMMUG00000010127	chitobiase [Source:VGNC Symbol;Acc:VGNC:71540]	-2.770253735	4.05E-11	9.02E-10
ENSMMUG00000022183	spermatogenesis associated 1 [Source:NCBI gene;Acc:710462]	-1.832091692	6.59E-18	3.93E-16
ENSMMUG00000059938		-1.21077825	0.000530654	0.00260278
ENSMMUG00000000404	uricase [Source:NCBI gene;Acc:714004]	-1.425538297	0.006114777	0.021289917
ENSMMUG00000003457	protein kinase cAMP-activated catalytic subunit beta [Source:NCBI gene;Acc:709728]	1.208698077	8.30E-25	9.97E-23

ENSMMUG00000057763		-1.348248024	0.000109275	0.000644955
ENSMMUG00000050832		2.971104451	0.002334942	0.00940072
ENSMMUG00000023345	adhesion G protein-coupled receptor L4 [Source:VGNC Symbol;Acc:VGNC:69652]	1.022702398	0.009027368	0.029804871
ENSMMUG00000049326	GIPC PDZ domain containing family member 2 [Source:NCBI gene;Acc:707432]	-0.577556135	0.00091748	0.004207382
ENSMMUG00000050425	DnaJ heat shock protein family (Hsp40) member B4 [Source:NCBI gene;Acc:707206]	-0.486401602	0.001489572	0.006381936
ENSMMUG00000017312	zinc finger RANBP2-type containing 2 [Source:NCBI gene;Acc:703513]	0.763648064	1.93E-06	1.73E-05
ENSMMUG00000017309	prostaglandin E receptor 3 [Source:VGNC Symbol;Acc:VGNC:76469]	0.374329119	0.010185257	0.032917535
ENSMMUG00000041837	Metazoan signal recognition particle RNA [Source:RFAM;Acc:RF00017]	-1.202936784	0.006178246	0.021470991
ENSMMUG00000056103	growth arrest and DNA damage inducible alpha [Source:NCBI gene;Acc:701054]	-1.678361949	4.33E-17	2.37E-15
ENSMMUG00000003265	leptin receptor [Source:VGNC Symbol;Acc:VGNC:81429]	0.876377471	2.28E-14	8.83E-13
ENSMMUG00000017259	DnaJ heat shock protein family (Hsp40) member C6 [Source:NCBI gene;Acc:698682]	-0.695746574	0.011539899	0.036421083
ENSMMUG00000053254	adenylate kinase 4 [Source:NCBI gene;Acc:698560]	0.60136139	7.48E-06	5.89E-05
ENSMMUG00000053072		0.520851295	0.01141011	0.036136988
ENSMMUG00000018565	ribonucleoprotein, PTB binding 2 [Source:VGNC Symbol;Acc:VGNC:76536]	-2.928197826	2.34E-07	2.62E-06
ENSMMUG00000012538	ubiquitin conjugating enzyme E2 U [Source:VGNC Symbol;Acc:VGNC:79726]	1.463286525	1.72E-09	2.89E-08
ENSMMUG00000056799		1.033629676	0.00131486	0.005734812
ENSMMUG00000004668	receptor tyrosine kinase like orphan receptor 1 [Source:VGNC Symbol;Acc:VGNC:76715]	1.210342797	5.99E-06	4.82E-05
ENSMMUG00000061816		1.321031262	5.16E-06	4.21E-05
ENSMMUG00000020049	phosphoglucomutase 1 [Source:NCBI gene;Acc:699401]	-0.681505292	1.58E-06	1.45E-05
ENSMMUG00000020047	integrin subunit beta 3 binding protein [Source:VGNC Symbol;Acc:VGNC:73787]	-1.15649817	1.27E-07	1.51E-06
ENSMMUG00000010351	KN motif and ankyrin repeat domains 4 [Source:VGNC Symbol;Acc:VGNC:73916]	-0.609211742	0.000895215	0.004119578
ENSMMUG00000055834		1.642409548	0.000775084	0.003622955
ENSMMUG00000011800	Myb like, SWIRM and MPN domains 1 [Source:VGNC Symbol;Acc:VGNC:75039]	-0.643667554	4.46E-06	3.69E-05
ENSMMUG00000022762	OMA1 zinc metallopeptidase [Source:VGNC Symbol;Acc:VGNC:75485]	-2.04588	1.08E-12	3.12E-11
ENSMMUG00000049840		0.438848196	0.005687746	0.020065624
ENSMMUG00000010406	protein kinase AMP-activated catalytic subunit alpha 2 [Source:VGNC Symbol;Acc:VGNC:76400]	-3.467024737	0.000752635	0.003533934
ENSMMUG00000002200	phospholipid phosphatase 3 [Source:NCBI gene;Acc:715993]	-1.556721606	5.28E-20	3.96E-18
ENSMMUG00000059612		0.453267962	6.82E-05	0.000424389
ENSMMUG00000032084	prolyl-tRNA synthetase 2, mitochondrial [Source:VGNC Symbol;Acc:VGNC:75707]	-0.560112119	0.00031422	0.001638972
ENSMMUG00000006754	tetratricopeptide repeat domain 4 [Source:VGNC Symbol;Acc:VGNC:78668]	-0.788607016	0.000121892	0.000712485
ENSMMUG00000008828	BEN domain containing 5 [Source:NCBI gene;Acc:711150]	2.292821921	3.43E-15	1.49E-13
ENSMMUG00000015371	microtubule associated serine/threonine kinase 2 [Source:VGNC Symbol;Acc:VGNC:74532]	-1.089162171	1.40E-05	0.000103634
ENSMMUG00000005986	Kruppel like factor 17 [Source:NCBI gene;Acc:702810]	1.361825298	0.000772534	0.003615673
ENSMMUG00000015555	ST3 beta-galactoside alpha-2,3-sialyltransferase 3 [Source:VGNC Symbol;Acc:VGNC:77828]	2.474290606	2.60E-12	7.09E-11
ENSMMUG00000012479	lysine demethylase 4A [Source:NCBI gene;Acc:701272]	-2.332541421	0.008790661	0.029135851
ENSMMUG00000053498		1.665386135	4.02E-05	0.000267454
ENSMMUG00000059313	small vasohibin binding protein [Source:NCBI gene;Acc:698308]	3.635977736	0.007491363	0.025426556
ENSMMUG00000016111	chromosome 1 C1orf50 homolog [Source:VGNC Symbol;Acc:VGNC:70432]	2.502655377	0.000680296	0.003243512

ENSMMUG00000022381	prolyl 3-hydroxylase 1 [Source:VGNC Symbol;Acc:VGNC:75648]	2.209647193	0.000238559	0.001289193
ENSMMUG00000057076	claudin 19 [Source:NCBI gene;Acc:697807]	4.459521216	1.17E-05	8.85E-05
ENSMMUG00000013129	peptidylprolyl isomerase H [Source:NCBI gene;Acc:697669]	1.771355103	0.001951345	0.008086287
ENSMMUG00000013128	coiled-coil domain containing 30 [Source:VGNC Symbol;Acc:VGNC:70866]	0.535008661	0.00346255	0.013149188
ENSMMUG00000014138	forkhead box J3 [Source:VGNC Symbol;Acc:VGNC:72596]	1.696034915	4.99E-06	4.08E-05
ENSMMUG00000052046		0.46182866	0.001752469	0.007358647
ENSMMUG00000044175	forkhead box O6 [Source:NCBI gene;Acc:697505]	-1.043496692	7.61E-06	5.96E-05
ENSMMUG00000043675	Cbp/p300 interacting transactivator with Glu/Asp rich carboxy-terminal domain 4 [Source:NCBI gene;Acc:114672840]	1.947085905	3.50E-17	1.94E-15
ENSMMUG00000019536	potassium voltage-gated channel subfamily Q member 4 [Source:VGNC Symbol;Acc:VGNC:74007]	0.899471093	3.92E-07	4.17E-06
ENSMMUG00000018664	collagen type IX alpha 2 chain [Source:VGNC Symbol;Acc:VGNC:71307]	1.168018519	2.31E-06	2.04E-05
ENSMMUG00000005992	zinc metallopeptidase STE24 [Source:VGNC Symbol;Acc:VGNC:78830]	1.012026729	0.014106345	0.043089713
ENSMMUG00000009189	palmitoyl-protein thioesterase 1 [Source:VGNC Symbol;Acc:VGNC:76386]	-0.492182604	1.46E-05	0.000107468
ENSMMUG00000009188	cyclase associated actin cytoskeleton regulatory protein 1 [Source:NCBI gene;Acc:693719]	-0.324908743	0.002745087	0.010752761
ENSMMUG00000058881	bone morphogenetic protein 8b [Source:NCBI gene;Acc:100427030]	1.052316822	0.006313409	0.02189342
ENSMMUG00000008419	G protein nucleolar 2 [Source:VGNC Symbol;Acc:VGNC:73037]	-1.15382911	0.00098943	0.004487784
ENSMMUG00000059855	Smad nuclear interacting protein 1 [Source:NCBI gene;Acc:713733]	-0.990384061	9.22E-05	0.000555077
ENSMMUG00000010323	zinc finger CCCH-type containing 12A [Source:VGNC Symbol;Acc:VGNC:79595]	-0.627577482	0.002885542	0.011251911
ENSMMUG00000017338	LSM10, U7 small nuclear RNA associated [Source:VGNC Symbol;Acc:VGNC:74307]	1.170885795	0.000566192	0.002754085
ENSMMUG00000058709		1.127163841	0.012009501	0.037657919
ENSMMUG00000014622	thyroid hormone receptor associated protein 3 [Source:NCBI gene;Acc:713033]	-0.468335099	0.007529136	0.025513893
ENSMMUG00000006252	argonaute RISC catalytic component 3 [Source:NCBI gene;Acc:712543]	-1.800142327	1.81E-19	1.29E-17
ENSMMUG00000006251	argonaute RISC component 1 [Source:VGNC Symbol;Acc:VGNC:81346]	-1.49838142	3.23E-12	8.70E-11
ENSMMUG00000019815	transcription factor AP-2 epsilon [Source:VGNC Symbol;Acc:VGNC:79878]	-2.637670525	3.68E-05	0.000246583
ENSMMUG00000002741	CUB and Sushi multiple domains 2 [Source:VGNC Symbol;Acc:VGNC:71441]	4.557896224	2.13E-08	2.90E-07
ENSMMUG00000006389	zinc finger and SCAN domain containing 20 [Source:VGNC Symbol;Acc:VGNC:78895]	4.799198893	2.70E-07	2.98E-06
ENSMMUG00000062949		4.526453738	4.58E-05	0.000301179
ENSMMUG00000003269	polyhomeotic homolog 2 [Source:VGNC Symbol;Acc:VGNC:75903]	2.100635229	0.016899025	0.049860183
ENSMMUG00000003268	alpha 1,3-galactosyltransferase 2 [Source:VGNC Symbol;Acc:VGNC:69550]	1.197826976	0.006687456	0.022993269
ENSMMUG00000003267	zinc finger protein 362 [Source:NCBI gene;Acc:710013]	1.101527781	5.54E-07	5.69E-06
ENSMMUG00000015097	tripartite motif containing 62 [Source:VGNC Symbol;Acc:VGNC:79513]	1.13419023	1.66E-08	2.31E-07
ENSMMUG00000006285	antizyme inhibitor 2 [Source:VGNC Symbol;Acc:VGNC:70153]	1.228597584	7.82E-22	7.10E-20
ENSMMUG00000006284	adenylate kinase 2 [Source:VGNC Symbol;Acc:VGNC:69761]	1.238471348	6.87E-10	1.25E-08
ENSMMUG00000044265	Metazoan signal recognition particle RNA [Source:RFAM;Acc:RF00017]	1.042634238	6.98E-07	7.00E-06
ENSMMUG00000054846	Metazoan signal recognition particle RNA [Source:RFAM;Acc:RF00017]	0.99681375	0.00370935	0.013951571
ENSMMUG00000055316		-3.392343745	0.002080353	0.008550212
ENSMMUG00000001264	BSD domain containing 1 [Source:VGNC Symbol;Acc:VGNC:70384]	-3.718208506	3.55E-06	3.01E-05
ENSMMUG00000053323		-2.870732302	0.00311865	0.012022228
ENSMMUG00000009951	MARCKS like 1 [Source:VGNC Symbol;Acc:VGNC:74723]	-3.58669018	0.00175323	0.007359717
ENSMMUG00000009948	histone deacetylase 1 [Source:NCBI gene;Acc:708441]	-2.496469585	5.91E-16	2.85E-14
ENSMMUG00000040694	LCK proto-oncogene, Src family tyrosine kinase [Source:NCBI gene;Acc:717704]	-2.122725576	6.32E-22	5.78E-20

ENSMMUG00000060894	Metazoan signal recognition particle RNA [Source:RFAM;Acc:RF00017]	-0.660649812	0.006866139	0.023485425
ENSMMUG00000046901	protein tyrosine phosphatase 4A2 [Source:NCBI gene;Acc:706489]	-0.848739548	0.000641708	0.003075672
ENSMMUG00000005466	SPOC domain containing 1 [Source:VGNC Symbol;Acc:VGNC:77997]	-0.911876452	4.48E-06	3.71E-05
ENSMMUG00000023401	adhesion G protein-coupled receptor B2 [Source:VGNC Symbol;Acc:VGNC:69598]	-0.583323556	0.002974552	0.011540183
ENSMMUG00000023398	collagen type XVI alpha 1 chain [Source:VGNC Symbol;Acc:VGNC:71293]	-0.652516737	6.79E-05	0.000423202
ENSMMUG00000023397	penta-EF-hand domain containing 1 [Source:NCBI gene;Acc:706038]	-0.793018756	9.92E-05	0.000592297
ENSMMUG00000060319	Metazoan signal recognition particle RNA [Source:RFAM;Acc:RF00017]	-0.647259331	0.015765323	0.04714678
ENSMMUG00000013188	serine incorporator 2 [Source:VGNC Symbol;Acc:VGNC:77294]	-0.524521359	0.004241983	0.015595779
ENSMMUG00000049574	antizyme inhibitor 2-like [Source:NCBI gene;Acc:705256]	-0.5391386	0.000804552	0.003748643
ENSMMUG00000012156	zinc finger CCHC-type containing 17 [Source:VGNC Symbol;Acc:VGNC:79840]	-0.410617195	0.008957167	0.029591648
ENSMMUG00000064353	fatty acid binding protein 3 [Source:NCBI gene;Acc:717237]	-0.590033033	0.000489277	0.002425034
ENSMMUG00000063979		-0.71123276	2.81E-11	6.45E-10
ENSMMUG00000057452		0.716132916	0.00398478	0.014799886
ENSMMUG00000051008		-2.291488115	0.005817157	0.020440902
ENSMMUG00000057173		-2.453075287	0.00104301	0.004692737
ENSMMUG00000015661	protein tyrosine phosphatase receptor type U [Source:VGNC Symbol;Acc:VGNC:76624]	-1.977963053	1.09E-05	8.29E-05
ENSMMUG00000007110	serine and arginine rich splicing factor 4 [Source:NCBI gene;Acc:717401]	-1.697809197	0.005787268	0.020365412
ENSMMUG00000016893	solute carrier family 9 member A1 [Source:VGNC Symbol;Acc:VGNC:82241]	-0.853657733	2.45E-06	2.15E-05
ENSMMUG00000015115	terminal nucleotidyltransferase 5B [Source:VGNC Symbol;Acc:VGNC:79692]	0.850747293	1.81E-05	0.000130153
ENSMMUG00000021031	TMF1 regulated nuclear protein 1 [Source:VGNC Symbol;Acc:VGNC:79946]	0.818150795	5.78E-06	4.67E-05
ENSMMUG00000050286		0.578021691	0.012770539	0.03960856
ENSMMUG00000058432	zinc finger DHHC-type palmitoyltransferase 18 [Source:NCBI gene;Acc:719959]	1.437678009	6.27E-08	7.82E-07
ENSMMUG00000012697	AT-rich interaction domain 1A [Source:VGNC Symbol;Acc:VGNC:69822]	2.333545907	1.41E-18	9.12E-17
ENSMMUG00000056266	Metazoan signal recognition particle RNA [Source:RFAM;Acc:RF00017]	1.69394658	1.64E-06	1.50E-05
ENSMMUG00000000649	UBX domain protein 11 [Source:VGNC Symbol;Acc:VGNC:79859]	2.107176517	9.71E-12	2.42E-10
ENSMMUG00000000644	centrosomal protein 85 [Source:VGNC Symbol;Acc:VGNC:71096]	2.141381501	8.51E-11	1.80E-09
ENSMMUG00000032217	transmembrane protein 50A [Source:VGNC Symbol;Acc:VGNC:79857]	0.426728345	0.001588458	0.006755824
ENSMMUG00000057953		0.80821742	0.002580447	0.010206767
ENSMMUG00000057675	non-compact myelin associated protein [Source:NCBI gene;Acc:712192]	0.882617512	0.005831092	0.020470076
ENSMMUG00000053036		-1.227532406	4.55E-05	0.000299136
ENSMMUG00000022927	RCAN family member 3 [Source:VGNC Symbol;Acc:VGNC:76875]	-1.008114538	0.002559974	0.010148445
ENSMMUG00000004463	grainyhead like transcription factor 3 [Source:VGNC Symbol;Acc:VGNC:73235]	0.587235085	0.006314859	0.02189342
ENSMMUG00000050770		-1.214766852	1.99E-08	2.72E-07
ENSMMUG00000059931		-0.531228002	0.001066696	0.004780072
ENSMMUG00000057438	Metazoan signal recognition particle RNA [Source:RFAM;Acc:RF00017]	-1.203407407	0.002488737	0.009922865

ENSMMUG00000045584	leucine zipper protein 1 [Source:NCBI gene;Acc:718626]	-1.844343068	4.36E-14	1.62E-12
ENSMMUG00000009773	lysine demethylase 1A [Source:VGNC Symbol;Acc:VGNC:73928]	-1.89751663	4.60E-14	1.69E-12
ENSMMUG00000058332	testis expressed 46 [Source:NCBI gene;Acc:709002]	-2.478569537	6.58E-07	6.62E-06
ENSMMUG00000041482	lactamase beta like 1 [Source:NCBI gene;Acc:718590]	-1.089110603	0.000467307	0.002332871
ENSMMUG00000023382	EPH receptor B2 [Source:NCBI gene;Acc:718570]	0.402070329	0.009220396	0.030345685
ENSMMUG00000002217	alkaline phosphatase, biomineralization associated [Source:VGNC Symbol;Acc:VGNC:69801]	1.48737092	2.25E-23	2.23E-21
ENSMMUG00000004149	eukaryotic translation initiation factor 4 gamma 3 [Source:NCBI gene;Acc:717555]	1.494647068	1.66E-20	1.33E-18
ENSMMUG00000002492	transmembrane and coiled-coil domains 4 [Source:VGNC Symbol;Acc:VGNC:79664]	2.21482983	0.016225039	0.048293078
ENSMMUG00000006688	ER membrane protein complex subunit 1 [Source:VGNC Symbol;Acc:VGNC:72134]	-0.834893408	1.33E-07	1.57E-06
ENSMMUG00000006669	ubiquitin protein ligase E3 component n-recognin 4 [Source:VGNC Symbol;Acc:VGNC:79845]	-0.504923801	0.001813654	0.007580532
ENSMMUG00000004754	intermediate filament family orphan 2 [Source:NCBI gene;Acc:106995162]	-0.95403352	2.67E-06	2.32E-05
ENSMMUG00000064850	kelch domain containing 7A [Source:NCBI gene;Acc:713771]	-0.818124442	0.002845731	0.011105607
ENSMMUG00000010849	Rho guanine nucleotide exchange factor 10 like [Source:VGNC Symbol;Acc:VGNC:69908]	-0.371068864	0.004016265	0.014886396
ENSMMUG00000022437	peptidyl arginine deiminase 6 [Source:VGNC Symbol;Acc:VGNC:75657]	2.790303792	0.001619787	0.006881016
ENSMMUG00000051619	peptidyl arginine deiminase 3 [Source:NCBI gene;Acc:700560]	0.482635238	0.004376882	0.016026886
ENSMMUG00000007315	ciliary rootlet coiled-coil, rootletin [Source:NCBI gene;Acc:710793]	0.51357763	0.000221884	0.001213055
ENSMMUG00000018245	EPH receptor A2 [Source:VGNC Symbol;Acc:VGNC:72235]	0.988855028	7.28E-07	7.24E-06
ENSMMUG00000058587	family with sequence similarity 131 member C [Source:NCBI gene;Acc:703604]	0.90943284	1.42E-05	0.000104626
ENSMMUG00000018235	chloride voltage-gated channel Kb [Source:NCBI gene;Acc:703476]	0.798014398	0.006935905	0.023712899
ENSMMUG00000001910	heat shock protein family B (small) member 7 [Source:NCBI gene;Acc:696704]	1.159446521	0.000785052	0.003662485
ENSMMUG00000001911	steroid receptor associated and regulated protein [Source:VGNC Symbol;Acc:VGNC:78008]	0.686387991	0.006886276	0.023548763
ENSMMUG00000008031	zinc finger and BTB domain containing 17 [Source:VGNC Symbol;Acc:VGNC:78849]	1.167145113	2.05E-05	0.000145714
ENSMMUG00000002802	agmatinase [Source:NCBI gene;Acc:713190]	-0.907149258	0.005988267	0.020936941
ENSMMUG00000032244	DnaJ heat shock protein family (Hsp40) member C16 [Source:NCBI gene;Acc:700894]	-1.464714619	0.00020394	0.001123835
ENSMMUG00000018163	caspase 9 [Source:NCBI gene;Acc:694544]	-2.522459533	0.000877397	0.004050403
ENSMMUG00000031821	chymotrypsin-like elastase family member 2B [Source:NCBI gene;Acc:106992406]	-1.985851401	3.07E-05	0.000209346
ENSMMUG00000053550		-2.981859179	0.000171699	0.000964821
ENSMMUG00000060541		-1.599770512	0.00367366	0.013835231
ENSMMUG00000003987	forkhead associated phosphopeptide binding domain 1 [Source:NCBI gene;Acc:699900]	-1.397452167	2.26E-05	0.000159416
ENSMMUG00000053824	leucine rich repeat containing 38 [Source:NCBI gene;Acc:722785]	0.807576804	0.000107366	0.000635495
ENSMMUG00000055233	PRAME family member 17-like [Source:NCBI gene;Acc:100427847]	-0.758253515	2.05E-06	1.83E-05
ENSMMUG00000054309	PRAME family member 5 [Source:NCBI gene;Acc:722781]	-1.429737927	0.003358748	0.012818619
ENSMMUG00000057550		-0.670390402	0.005478933	0.01943564
ENSMMUG00000021449	vacuolar protein sorting 13 homolog D [Source:NCBI gene;Acc:722775]	1.077447632	1.97E-20	1.56E-18
ENSMMUG00000064242		1.096753698	3.09E-06	2.66E-05
ENSMMUG00000063313	Small nucleolar RNA U3 [Source:RFAM;Acc:RF00012]	0.939365336	0.008335995	0.027787286
ENSMMUG00000021447	TNF receptor superfamily member 1B [Source:VGNC Symbol;Acc:VGNC:78590]	4.518816117	0.000102123	0.000607432

ENSMMUG00000023449	mitofusin 2 [Source:VGNC Symbol;Acc:VGNC:74522]	1.492974674	2.11E-05	0.000149318
ENSMMUG00000032249	procollagen-lysine,2-oxoglutarate 5-dioxygenase 1 [Source:NCBI gene;Acc:722763]	1.19474072	3.19E-15	1.40E-13
ENSMMUG00000012469	KIAA2013 [Source:VGNC Symbol;Acc:VGNC:73918]	0.89933786	2.75E-09	4.42E-08
ENSMMUG00000062316	Metazoan signal recognition particle RNA [Source:RFAM;Acc:RF00017]	1.114786139	1.36E-09	2.33E-08
ENSMMUG00000037413	U5 spliceosomal RNA [Source:RFAM;Acc:RF00020]	1.061112791	7.91E-09	1.18E-07
ENSMMUG00000051669		0.83289674	2.87E-07	3.15E-06
ENSMMUG00000002261	F-box protein 2 [Source:VGNC Symbol;Acc:VGNC:72486]	-1.879861522	0.00377904	0.014169693
ENSMMUG00000049196	dispatched RND transporter family member 3 [Source:NCBI gene;Acc:722727]	-1.109810132	5.92E-15	2.47E-13
ENSMMUG00000032252	UbiA prenyltransferase domain containing 1 [Source:VGNC Symbol;Acc:VGNC:79881]	-1.126090759	2.26E-16	1.13E-14
ENSMMUG00000019325	mechanistic target of rapamycin kinase [Source:VGNC Symbol;Acc:VGNC:75062]	-1.426589293	1.29E-15	5.98E-14
ENSMMUG00000053062	U6 spliceosomal RNA [Source:RFAM;Acc:RF00026]	-2.149327511	0.000991719	0.004496763
ENSMMUG00000006187	angiopoietin like 7 [Source:VGNC Symbol;Acc:VGNC:69683]	-1.194313774	2.07E-09	3.41E-08
ENSMMUG00000062294		4.096021279	5.02E-05	0.000325485
ENSMMUG00000058771	PHD finger protein 13 [Source:NCBI gene;Acc:704846]	-1.165341029	0.001780466	0.007467579
ENSMMUG00000032281	zinc finger and BTB domain containing 48 [Source:VGNC Symbol;Acc:VGNC:79959]	-0.846871058	0.002279898	0.009229428
ENSMMUG00000023681	nucleolar protein 9 [Source:VGNC Symbol;Acc:VGNC:75263]	0.853360071	0.009920858	0.03222655
ENSMMUG00000023678	pleckstrin homology and RhoGEF domain containing G5 [Source:VGNC Symbol;Acc:VGNC:76130]	1.107311428	4.42E-05	0.000291708
ENSMMUG00000023676	espin [Source:NCBI gene;Acc:722532]	-1.318336509	4.60E-14	1.69E-12
ENSMMUG00000016838	chromodomain helicase DNA binding protein 5 [Source:VGNC Symbol;Acc:VGNC:70959]	-1.07298184	0.002113203	0.008663178
ENSMMUG00000055126		1.421902636	4.54E-05	0.000298676
ENSMMUG00000056383		0.790967036	0.012131783	0.037975824
ENSMMUG00000054719		-0.917892279	8.95E-05	0.00054158
ENSMMUG00000032284	leucine rich repeat containing 47 [Source:VGNC Symbol;Acc:VGNC:74388]	3.534980101	0.00039589	0.002010147
ENSMMUG00000050239		-1.603780875	7.18E-08	8.86E-07
ENSMMUG00000014075	cilia and flagella associated protein 74 [Source:NCBI gene;Acc:709689]	-1.025141874	0.009183685	0.030238557
ENSMMUG00000010879	NAD kinase [Source:VGNC Symbol;Acc:VGNC:75047]	-0.657126992	0.015728475	0.047065634
ENSMMUG00000005578	solute carrier family 35 member E2 [Source:VGNC Symbol;Acc:VGNC:77590]	-0.931248863	0.002308891	0.009318252
ENSMMUG00000001894	cyclin dependent kinase 11B [Source:NCBI gene;Acc:703046]	-1.071523306	0.000173279	0.000972943
ENSMMUG00000012961	mindbomb E3 ubiquitin protein ligase 2 [Source:VGNC Symbol;Acc:VGNC:74627]	-0.697600506	0.0023025	0.009297621
ENSMMUG00000048805		-1.002515611	1.35E-05	0.0001007
ENSMMUG00000040063	fibronectin type III domain containing 10 [Source:NCBI gene;Acc:106996973]	-1.0961752	0.004670551	0.016918965
ENSMMUG00000060167		-0.999794772	0.000107005	0.000633616
ENSMMUG00000001551	SSU72 homolog, RNA polymerase II CTD phosphatase [Source:VGNC Symbol;Acc:VGNC:77986]	-1.010135373	4.61E-07	4.85E-06
ENSMMUG00000051354		-1.408906884	0.004096039	0.015128078

ENSMMUG00000001549	ATPase family AAA domain-containing protein 3A-like [Source:NCBI gene;Acc:114674453]	-0.890824371	1.10E-09	1.90E-08
ENSMMUG00000064596	ATPase family AAA domain-containing protein 3A-like [Source:NCBI gene;Acc:114674454]	-1.105869022	0.000566837	0.002755887
ENSMMUG00000006904	cyclin L2 [Source:NCBI gene;Acc:114669744]	-0.614920779	0.015263461	0.045910395
ENSMMUG00000032292	sodium channel epithelial 1 delta subunit [Source:VGNC Symbol;Acc:VGNC:76993]	-1.270869162	0.001584573	0.006743247
ENSMMUG00000045769	tubulin tyrosine ligase like 10 [Source:NCBI gene;Acc:702662]	0.466182519	3.07E-06	2.64E-05
ENSMMUG00000056613		0.66122497	6.18E-07	6.26E-06
ENSMMUG00000000972	tyrosine kinase non receptor 2 [Source:VGNC Symbol;Acc:VGNC:79192]	-2.737336207	3.45E-09	5.49E-08
ENSMMUG00000053601		1.794710264	0.007568232	0.025621655
ENSMMUG00000065323		-2.828021491	0.010810971	0.034555648
ENSMMUG00000022562	interleukin 1 receptor accessory protein [Source:VGNC Symbol;Acc:VGNC:73718]	-3.205877375	6.13E-05	0.000387055
ENSMMUG00000006596	CCR4-NOT transcription complex subunit 10 [Source:VGNC Symbol;Acc:VGNC:71327]	1.20127459	4.10E-08	5.30E-07
ENSMMUG00000049551		1.453451417	1.39E-05	0.000103324
ENSMMUG00000055585		-2.418540418	3.10E-13	9.70E-12
ENSMMUG00000000056	5-azacytidine induced 2 [Source:VGNC Symbol;Acc:VGNC:70115]	-0.887826538	0.010770332	0.034447931
ENSMMUG00000051896		-3.108348758	0.002903146	0.011308424
ENSMMUG00000060958	leucine rich repeat containing 3B [Source:NCBI gene;Acc:701323]	-1.340063914	0.008233866	0.027497291
ENSMMUG00000051809		-1.905199657	0.014761141	0.04470473
ENSMMUG00000009667	zinc finger protein 385D [Source:VGNC Symbol;Acc:VGNC:79587]	2.224899808	0.005621875	0.019903848
ENSMMUG00000051975		1.948170236	1.61E-09	2.72E-08
ENSMMUG00000020761	ELL associated factor 1 [Source:VGNC Symbol;Acc:VGNC:71874]	3.29018252	0.012897139	0.039958591
ENSMMUG00000020377	collagen type VI alpha 5 chain [Source:NCBI gene;Acc:719331]	-0.817967605	1.30E-05	9.72E-05
ENSMMUG00000005137	ATPase secretory pathway Ca2+ transporting 1 [Source:VGNC Symbol;Acc:VGNC:70054]	-2.807690425	2.59E-12	7.06E-11
ENSMMUG00000001154	U8 snoRNA-decapping enzyme-like [Source:NCBI gene;Acc:717946]	0.641900156	0.002556693	0.010140968
ENSMMUG00000024125	small nucleolar RNA, H/ACA box 58 [Source:HGNC Symbol;Acc:HGNC:32652]	-4.015143316	0.000497708	0.002460107
ENSMMUG00000003277	copine 4 [Source:VGNC Symbol;Acc:VGNC:71466]	-1.374895371	1.14E-08	1.65E-07
ENSMMUG00000056585		-1.982805388	5.71E-05	0.000364289
ENSMMUG00000016004	acid phosphatase 3 [Source:VGNC Symbol;Acc:VGNC:69385]	-1.295250873	4.67E-05	0.000306327
ENSMMUG00000016005	DnaJ heat shock protein family (Hsp40) member C13 [Source:VGNC Symbol;Acc:VGNC:84191]	-0.382572623	3.89E-09	6.08E-08
ENSMMUG00000054158	acyl-CoA dehydrogenase family member 11 [Source:NCBI gene;Acc:106997118]	1.448324212	5.71E-12	1.49E-10
ENSMMUG00000019854	transmembrane protein 108 [Source:VGNC Symbol;Acc:VGNC:79714]	-0.841449743	0.000936758	0.004284975
ENSMMUG00000019857	beaded filament structural protein 2 [Source:VGNC Symbol;Acc:VGNC:70245]	-0.872109689	0.000220312	0.001206583
ENSMMUG00000000058	CDV3 homolog [Source:VGNC Symbol;Acc:VGNC:71114]	-0.776362505	0.006018612	0.02102176
ENSMMUG00000000059	DNA topoisomerase II binding protein 1 [Source:VGNC Symbol;Acc:VGNC:79061]	-0.747955515	4.63E-05	0.000304098

ENSMMUG00000008916	transferrin [Source:VGNC Symbol;Acc:VGNC:82716]	-0.642129151	0.000770898	0.003609195
ENSMMUG00000008918	SRP receptor subunit beta [Source:VGNC Symbol;Acc:VGNC:77980]	-0.601195027	0.006788112	0.023273297
ENSMMUG00000007760	centrosomal protein 70 [Source:VGNC Symbol;Acc:VGNC:71140]	0.501657688	0.001078081	0.00481977
ENSMMUG00000003051	COPI coat complex subunit beta 2 [Source:VGNC Symbol;Acc:VGNC:71313]	-0.694212648	1.91E-05	0.00013638
ENSMMUG00000062527		-1.606476566	3.59E-09	5.67E-08
ENSMMUG00000022153	solute carrier family 25 member 36 [Source:VGNC Symbol;Acc:VGNC:84079]	-1.904818557	4.27E-11	9.46E-10
ENSMMUG00000053344	splA/ryanodine receptor domain and SOCS box containing 4 [Source:NCBI gene;Acc:715278]	-2.091268435	8.32E-06	6.45E-05
ENSMMUG00000057297		-1.665986517	0.000493818	0.002444204
ENSMMUG00000056024		2.027563254	8.25E-24	8.44E-22
ENSMMUG00000063351	Metazoan signal recognition particle RNA [Source:RFAM;Acc:RF00017]	2.626115551	1.47E-09	2.49E-08
ENSMMUG00000007387	G protein-coupled receptor kinase 7 [Source:VGNC Symbol;Acc:VGNC:73182]	2.537044139	4.56E-27	7.39E-25
ENSMMUG00000052474		2.75953418	2.78E-29	6.12E-27
ENSMMUG00000011134	transcription factor Dp-2 [Source:VGNC Symbol;Acc:VGNC:78324]	0.390590702	0.00509025	0.0182466
ENSMMUG00000012151	glycerol kinase 5 [Source:VGNC Symbol;Acc:VGNC:73022]	-2.663397759	2.83E-06	2.44E-05
ENSMMUG00000012405	transient receptor potential cation channel subfamily C member 1 [Source:VGNC Symbol;Acc:VGNC:78651]	4.109184164	1.62E-19	1.17E-17
ENSMMUG00000009694	solute carrier family 9 member A9 [Source:VGNC Symbol;Acc:VGNC:77747]	-1.969409111	0.000357806	0.001837312
ENSMMUG00000017055	divergent protein kinase domain 2A [Source:VGNC Symbol;Acc:VGNC:71781]	0.861232467	0.00384095	0.014357214
ENSMMUG00000049859		-1.60907937	8.05E-11	1.72E-09
ENSMMUG00000062175	carboxypeptidase B1 [Source:NCBI gene;Acc:712639]	0.655774765	0.003073249	0.011872646
ENSMMUG00000005519	carboxypeptidase A3 [Source:VGNC Symbol;Acc:VGNC:71456]	0.803576286	2.62E-05	0.000181629
ENSMMUG00000059895		0.801930937	0.000514125	0.002535224
ENSMMUG00000005521	glycogenin 1 [Source:VGNC Symbol;Acc:VGNC:73332]	0.740913661	1.74E-06	1.58E-05
ENSMMUG00000005526	helicase like transcription factor [Source:VGNC Symbol;Acc:VGNC:73383]	0.625270132	0.003340252	0.012761128
ENSMMUG00000047621	transmembrane 4 L six family member 18 [Source:VGNC Symbol;Acc:VGNC:82713]	0.538455028	1.55E-08	2.17E-07
ENSMMUG00000031534	ankyrin repeat and ubiquitin domain containing 1 [Source:VGNC Symbol;Acc:VGNC:84239]	2.155427891	7.21E-34	2.19E-31
ENSMMUG00000035943	U6 spliceosomal RNA [Source:RFAM;Acc:RF00026]	2.483514479	3.10E-11	7.07E-10
ENSMMUG00000023335	ring finger protein 13 [Source:VGNC Symbol;Acc:VGNC:81557]	1.370442877	7.21E-07	7.17E-06
ENSMMUG00000059788		0.562639589	5.45E-05	0.000350385
ENSMMUG00000048232	MINDY family member 4B [Source:VGNC Symbol;Acc:VGNC:84153]	-0.737470669	0.000119488	0.000700122
ENSMMUG00000038998	purinergic receptor P2Y13 [Source:VGNC Symbol;Acc:VGNC:83950]	0.637175121	0.003074947	0.011872646
ENSMMUG00000001105	purinergic receptor P2Y12 [Source:VGNC Symbol;Acc:VGNC:75642]	0.406518026	0.012524535	0.039020395
ENSMMUG00000057501		-1.929199575	2.50E-08	3.35E-07
ENSMMUG00000031537		-2.156693835	0.016877598	0.049835826
ENSMMUG00000061194		1.291782148	1.23E-05	9.24E-05
ENSMMUG00000061684		1.221811259	3.20E-05	0.000217656
ENSMMUG00000053540		1.821461223	0.0027457	0.010752761
ENSMMUG00000031540	purinergic receptor P2Y1 [Source:VGNC Symbol;Acc:VGNC:75737]	5.074248819	2.12E-06	1.88E-05
ENSMMUG00000011347	DEAH-box helicase 36 [Source:VGNC Symbol;Acc:VGNC:71623]	0.853768026	4.54E-29	9.70E-27
ENSMMUG00000051847		1.002542335	0.016337709	0.048568769
ENSMMUG00000060572		0.834651987	4.49E-05	0.000295925
ENSMMUG00000056116		-1.564527264	1.94E-10	3.88E-09

ENSMMUG00000009564	NMD3 ribosome export adaptor [Source:VGNC Symbol;Acc:VGNC:75242]	1.801708474	0.001132688	0.005029286
ENSMMUG00000063228		-2.027353124	0.012249772	0.038290689
ENSMMUG00000042979		-1.957337081	0.010108861	0.03269244
ENSMMUG00000055530	Metazoan signal recognition particle RNA [Source:RFAM;Acc:RF00017]	-1.307553423	0.012111963	0.037930118
ENSMMUG00000001302	sucrase-isomaltase [Source:VGNC Symbol;Acc:VGNC:77213]	0.693978772	5.68E-09	8.71E-08
ENSMMUG00000023045	zinc finger B-box domain containing [Source:VGNC Symbol;Acc:VGNC:79592]	1.771006076	0.00037244	0.001901709
ENSMMUG00000005939	serpin family I member 1 [Source:VGNC Symbol;Acc:VGNC:77366]	0.872032162	0.014858854	0.044935128
ENSMMUG00000020734	golgi integral membrane protein 4 [Source:VGNC Symbol;Acc:VGNC:72945]	0.300068808	0.00764691	0.025868774
ENSMMUG00000051641		-1.088676722	1.15E-05	8.70E-05
ENSMMUG00000063303	U6 spliceosomal RNA [Source:RFAM;Acc:RF00026]	-2.685231651	0.015786992	0.047192165
ENSMMUG00000022199	polyhomeotic homolog 3 [Source:VGNC Symbol;Acc:VGNC:75804]	-1.544066528	2.21E-11	5.17E-10
ENSMMUG00000056664	U6 spliceosomal RNA [Source:RFAM;Acc:RF00026]	-1.745920972	0.004024619	0.014913557
ENSMMUG00000023289	phospholipase D1 [Source:VGNC Symbol;Acc:VGNC:76171]	-2.308120518	0.011551155	0.036440787
ENSMMUG00000040869	spermatogenesis associated 16 [Source:VGNC Symbol;Acc:VGNC:84073]	0.61354614	0.003432144	0.013066063
ENSMMUG00000045509	N-acetylated alpha-linked acidic dipeptidase like 2 [Source:VGNC Symbol;Acc:VGNC:83927]	-1.359681292	0.002948726	0.011467531
ENSMMUG00000014426	potassium calcium-activated channel subfamily M regulatory beta subunit 2 [Source:VGNC Symbol;Acc:VGNC:84158]	-1.445882024	0.004738559	0.017139693
ENSMMUG00000003216	zinc finger matrin-type 3 [Source:VGNC Symbol;Acc:VGNC:79831]	-1.206526623	0.010686411	0.0342177
ENSMMUG00000003594	ubiquitin specific peptidase 13 [Source:VGNC Symbol;Acc:VGNC:78735]	-0.782727604	1.70E-06	1.55E-05
ENSMMUG00000064088	U2 spliceosomal RNA [Source:RFAM;Acc:RF00004]	-0.512150157	0.00346424	0.01315217
ENSMMUG00000003595	peroxisomal biogenesis factor 5 like [Source:VGNC Symbol;Acc:VGNC:75889]	-1.427219908	0.014138602	0.043158794
ENSMMUG00000001456	MCF.2 cell line derived transforming sequence-like 2 [Source:VGNC Symbol;Acc:VGNC:74624]	3.961844076	0.00069253	0.003292123
ENSMMUG00000008070	YEATS domain containing 2 [Source:VGNC Symbol;Acc:VGNC:79502]	1.567559291	5.52E-35	2.17E-32
ENSMMUG00000005515	ATP binding cassette subfamily C member 5 [Source:VGNC Symbol;Acc:VGNC:69574]	-0.964776111	3.22E-09	5.13E-08
ENSMMUG00000013846	5-hydroxytryptamine receptor 3C [Source:VGNC Symbol;Acc:VGNC:84171]	0.631136254	0.00725663	0.024710619
ENSMMUG00000006553	dishevelled segment polarity protein 3 [Source:VGNC Symbol;Acc:VGNC:72014]	-1.227322264	1.88E-05	0.000134345
ENSMMUG00000018955	EEF1A lysine methyltransferase 4 [Source:NCBI gene;Acc:106996971]	-0.300727774	0.005913059	0.020722793
ENSMMUG00000000800	proteasome 26S subunit, non-ATPase 2 [Source:VGNC Symbol;Acc:VGNC:84144]	-0.434318004	0.000144771	0.000831988
ENSMMUG00000010934	eukaryotic translation initiation factor 4 gamma 1 [Source:VGNC Symbol;Acc:VGNC:72195]	-0.431983049	0.000296756	0.001560897
ENSMMUG00000007503	family with sequence similarity 131 member A [Source:VGNC Symbol;Acc:VGNC:72385]	-0.398728791	0.016870773	0.049835826
ENSMMUG00000007507	chloride voltage-gated channel 2 [Source:VGNC Symbol;Acc:VGNC:71235]	-0.332796429	0.00101753	0.004592313
ENSMMUG00000007524	RNA polymerase II subunit H [Source:VGNC Symbol;Acc:VGNC:76216]	-0.963509379	1.01E-05	7.71E-05
ENSMMUG00000007528	chordin [Source:VGNC Symbol;Acc:VGNC:71042]	2.47920114	2.70E-09	4.36E-08
ENSMMUG00000060581		1.963955378	3.98E-05	0.000265735

ENSMMUG00000000425	VPS8 subunit of CORVET complex [Source:VGNC Symbol;Acc:VGNC:79779]	-0.421328829	0.005713747	0.020145706
ENSMMUG00000021972	diacylglycerol kinase gamma [Source:VGNC Symbol;Acc:VGNC:71785]	1.434984109	1.80E-18	1.15E-16
ENSMMUG00000052418		-0.622200816	9.88E-06	7.56E-05
ENSMMUG00000018909	fetuin B [Source:VGNC Symbol;Acc:VGNC:72638]	0.508054516	0.000325617	0.00169295
ENSMMUG00000018910	histidine rich glycoprotein [Source:VGNC Symbol;Acc:VGNC:73521]	1.173695247	0.000195663	0.001083975
ENSMMUG00000022532	EPM2A interacting protein 1 [Source:VGNC Symbol;Acc:VGNC:84184]	-0.713580198	0.014534866	0.044120558
ENSMMUG00000022533	mutL homolog 1 [Source:VGNC Symbol;Acc:VGNC:74880]	-1.47907793	0.009845488	0.032011598
ENSMMUG00000022539	LRR binding FLII interacting protein 2 [Source:VGNC Symbol;Acc:VGNC:74449]	0.403504193	0.001710328	0.007219231
ENSMMUG00000054175		0.593619607	1.61E-07	1.88E-06
ENSMMUG00000000987	DLEC1 cilia and flagella associated protein [Source:VGNC Symbol;Acc:VGNC:71808]	-0.471353589	0.008620666	0.028650805
ENSMMUG00000004847	xylulokinase [Source:VGNC Symbol;Acc:VGNC:78815]	0.407234754	0.001129252	0.00501863
ENSMMUG00000018790	activin A receptor type 2B [Source:VGNC Symbol;Acc:VGNC:69450]	1.946447767	7.18E-15	2.93E-13
ENSMMUG00000015827	sodium voltage-gated channel alpha subunit 10 [Source:VGNC Symbol;Acc:VGNC:76980]	-0.206814684	0.014065581	0.042983258
ENSMMUG00000020150	xin actin binding repeat containing 1 [Source:VGNC Symbol;Acc:VGNC:78952]	-0.80117474	9.32E-08	1.12E-06
ENSMMUG00000051711		-0.339750805	0.004861373	0.017527227
ENSMMUG00000051408	myelin associated oligodendrocyte basic protein [Source:NCBI gene;Acc:717265]	0.818989169	5.91E-05	0.00037486
ENSMMUG00000009197	myelin associated oligodendrocyte basic protein [Source:VGNC Symbol;Acc:VGNC:74908]	1.192080644	8.84E-13	2.60E-11
ENSMMUG00000017701	myosin VIIA and Rab interacting protein [Source:VGNC Symbol;Acc:VGNC:74992]	2.576694266	2.02E-10	4.02E-09
ENSMMUG00000013330	ectonucleoside triphosphate diphosphohydrolase 3 [Source:VGNC Symbol;Acc:VGNC:72226]	1.861162282	1.83E-11	4.38E-10
ENSMMUG00000009437	zinc finger protein 619 [Source:VGNC Symbol;Acc:VGNC:82562]	2.374194566	2.29E-05	0.000161038
ENSMMUG00000024178	U5 spliceosomal RNA [Source:RFAM;Acc:RF00020]	2.678797187	0.005588988	0.019797055
ENSMMUG00000050991		-0.551499015	0.013042785	0.04032927
ENSMMUG00000001074	unc-51 like kinase 4 [Source:VGNC Symbol;Acc:VGNC:82565]	-0.526859834	0.006402536	0.022139313
ENSMMUG00000050275		-0.598253906	0.016238262	0.048322541
ENSMMUG00000021242	natural killer cell triggering receptor [Source:VGNC Symbol;Acc:VGNC:81486]	-2.957852501	0.000208707	0.001148364
ENSMMUG00000021244	kelch like family member 40 [Source:VGNC Symbol;Acc:VGNC:74165]	-3.442174316	4.73E-05	0.000308784
ENSMMUG00000021246	hedgehog acyltransferase like [Source:NCBI gene;Acc:717333]	-1.892940225	0.001803051	0.007549237
ENSMMUG00000031684	KRAB box domain containing 1 [Source:NCBI gene;Acc:717189]	2.048605724	0.005843092	0.020505158
ENSMMUG00000056798		1.846377603	7.82E-05	0.000481082
ENSMMUG00000054286		1.265112509	3.97E-05	0.000265287
ENSMMUG00000001028	SNF related kinase [Source:VGNC Symbol;Acc:VGNC:84074]	0.538511092	2.13E-06	1.89E-05
ENSMMUG00000040696	Metazoan signal recognition particle RNA [Source:RFAM;Acc:RF00017]	2.545042313	0.000265752	0.001421877
ENSMMUG00000051589		-0.824959578	0.000598761	0.002895606
ENSMMUG00000019740	zinc finger DHHC-type palmitoyltransferase 3 [Source:VGNC Symbol;Acc:VGNC:79143]	-0.782987757	0.016505089	0.048996219
ENSMMUG00000004576	C-C motif chemokine receptor 3 [Source:VGNC Symbol;Acc:VGNC:70738]	1.076373136	0.003916956	0.014579316
ENSMMUG00000062394	C-C motif chemokine receptor 2 [Source:NCBI gene;Acc:574098]	1.037885527	2.87E-05	0.000197076
ENSMMUG00000006454	kelch like family member 18 [Source:VGNC Symbol;Acc:VGNC:74154]	-0.715330382	0.003764749	0.014130688
ENSMMUG00000014045	protein tyrosine phosphatase non-receptor type 23 [Source:VGNC Symbol;Acc:VGNC:76477]	1.69735252	1.55E-10	3.16E-09
ENSMMUG00000014046	SREBF chaperone [Source:VGNC Symbol;Acc:VGNC:77140]	2.312415354	4.34E-45	5.26E-42

ENSMMUG00000013385	elongator acetyltransferase complex subunit 6 [Source:VGNC Symbol;Acc:VGNC:72113]	1.157104289	4.75E-14	1.73E-12
ENSMMUG00000064458		-1.596992808	1.37E-06	1.28E-05
ENSMMUG00000003074	plexin B1 [Source:VGNC Symbol;Acc:VGNC:76187]	2.400711911	0.001801389	0.007544452
ENSMMUG00000010058	ATR interacting protein [Source:VGNC Symbol;Acc:VGNC:84235]	0.798113367	4.56E-14	1.69E-12
ENSMMUG00000043955	three prime repair exonuclease 1 [Source:VGNC Symbol;Acc:VGNC:82708]	0.721005635	0.000351437	0.00180972
ENSMMUG00000010062	shisa family member 5 [Source:VGNC Symbol;Acc:VGNC:84082]	0.722788221	7.18E-07	7.15E-06
ENSMMUG00000000591	6-phosphofructo-2-kinase/fructose-2,6-biphosphatase 4 [Source:VGNC Symbol;Acc:VGNC:75960]	0.730527411	1.50E-08	2.10E-07
ENSMMUG00000059318	urocortin 2 [Source:NCBI gene;Acc:106996925]	2.160982405	6.18E-34	1.95E-31
ENSMMUG00000014699		1.714133404	4.84E-57	1.76E-53
ENSMMUG00000032659	mml-mir-711 [Source:miRBase;Acc:MI0012553]	1.951187721	6.20E-06	4.97E-05
ENSMMUG00000014707	cadherin EGF LAG seven-pass G-type receptor 3 [Source:VGNC Symbol;Acc:VGNC:71148]	1.552368587	0.002635451	0.010388166
ENSMMUG00000064174		1.597409778	0.003070345	0.011870912
ENSMMUG00000014720	NCK interacting protein with SH3 domain [Source:VGNC Symbol;Acc:VGNC:74960]	1.79093758	1.90E-06	1.71E-05
ENSMMUG00000052238		0.709224549	0.000279412	0.001486753
ENSMMUG00000014537	bassoon presynaptic cytomatrix protein [Source:VGNC Symbol;Acc:VGNC:70284]	0.502966646	0.00103962	0.004678936
ENSMMUG00000014540	acylaminoacyl-peptide hydrolase [Source:VGNC Symbol;Acc:VGNC:69982]	-0.767428257	0.002642465	0.010404528
ENSMMUG00000014564	ring finger protein 123 [Source:VGNC Symbol;Acc:VGNC:76790]	-0.602754301	2.26E-09	3.70E-08
ENSMMUG00000014589	inositol hexakisphosphate kinase 1 [Source:VGNC Symbol;Acc:VGNC:73621]	1.041686986	4.03E-05	0.00026821
ENSMMUG00000031794		1.316198083	1.89E-05	0.000135271
ENSMMUG00000020979	inka box actin regulator 1 [Source:VGNC Symbol;Acc:VGNC:73692]	1.55275752	1.93E-13	6.21E-12
ENSMMUG00000020980	ubiquitin like modifier activating enzyme 7 [Source:VGNC Symbol;Acc:VGNC:78690]	1.529977418	3.77E-09	5.93E-08
ENSMMUG00000016476	TRAF interacting protein [Source:VGNC Symbol;Acc:VGNC:82261]	1.351734802	1.44E-10	2.95E-09
ENSMMUG00000016480	CaM kinase like vesicle associated [Source:VGNC Symbol;Acc:VGNC:70663]	1.512892999	1.81E-10	3.64E-09
ENSMMUG00000044412	Metazoan signal recognition particle RNA [Source:RFAM;Acc:RF00017]	2.791237906	0.009684498	0.031572898
ENSMMUG00000003353	macrophage stimulating 1 receptor [Source:VGNC Symbol;Acc:VGNC:74941]	4.658720531	5.64E-05	0.000360327
ENSMMUG00000056280	G protein subunit alpha i2 [Source:NCBI gene;Acc:703780]	-0.734036929	2.48E-06	2.17E-05
ENSMMUG00000020117	semaphorin 3B [Source:VGNC Symbol;Acc:VGNC:77160]	-1.176468416	3.62E-06	3.06E-05
ENSMMUG00000004273	dedicator of cytokinesis 3 [Source:VGNC Symbol;Acc:VGNC:71967]	1.308525493	6.09E-11	1.32E-09
ENSMMUG00000004280	DDB1 and CUL4 associated factor 1 [Source:VGNC Symbol;Acc:VGNC:71819]	1.552442617	5.79E-05	0.000368683
ENSMMUG00000017351	stabilin 1 [Source:VGNC Symbol;Acc:VGNC:77956]	-0.784458658	6.54E-07	6.59E-06
ENSMMUG00000050505	small integral membrane protein 4 [Source:NCBI gene;Acc:696553]	1.317849888	2.23E-12	6.17E-11
ENSMMUG00000012526	glycosyltransferase 8 domain containing 1 [Source:VGNC Symbol;Acc:VGNC:73001]	1.590424205	1.03E-15	4.87E-14
ENSMMUG00000017370	signal peptidase complex subunit 1 [Source:VGNC Symbol;Acc:VGNC:84072]	1.411583889	0.00016557	0.000933991
ENSMMUG00000014433	transketolase [Source:VGNC Symbol;Acc:VGNC:78362]	3.411472526	0.003868167	0.014440588
ENSMMUG00000011208	decapping mRNA 1A [Source:VGNC Symbol;Acc:VGNC:71668]	-1.238408	0.011107918	0.035333875
ENSMMUG00000019266	choline dehydrogenase [Source:VGNC Symbol;Acc:VGNC:71086]	-0.937336057	2.55E-05	0.000177275
ENSMMUG00000021620	ELKS/RAB6-interacting/CAST family member 2 [Source:VGNC Symbol;Acc:VGNC:72164]	-0.641802241	1.20E-06	1.14E-05
ENSMMUG00000039085	adaptor protein, phosphotyrosine interacting with PH domain and leucine zipper 1 [Source:NCBI gene;Acc:100425753]	-2.133407129	0.000685137	0.003263381
ENSMMUG00000021008	ankyrin repeat and SOCS box containing 14 [Source:VGNC Symbol;Acc:VGNC:84237]	-2.479252166	0.001155599	0.005113832
ENSMMUG00000055244	U6 spliceosomal RNA [Source:RFAM;Acc:RF00026]	-1.396773389	6.28E-10	1.15E-08
ENSMMUG00000024983	U6 spliceosomal RNA [Source:RFAM;Acc:RF00026]	-0.43031413	0.01294066	0.040076347
ENSMMUG00000018619	DENN domain containing 6A [Source:VGNC Symbol;Acc:VGNC:71726]	2.449640852	2.36E-15	1.05E-13

ENSMMUG00000000706	filamin B [Source:VGNC Symbol;Acc:VGNC:72672]	1.78356702	3.79E-21	3.20E-19
ENSMMUG00000017510	membrane associated guanylate kinase, WW and PDZ domain containing 1 [Source:VGNC Symbol;Acc:VGNC:74499]	2.239792093	1.49E-21	1.32E-19
ENSMMUG00000057539		2.638941141	3.44E-12	9.20E-11
ENSMMUG00000001531	leucine rich repeats and immunoglobulin like domains 1 [Source:VGNC Symbol;Acc:VGNC:84156]	-0.617765065	0.000175597	0.00098444
ENSMMUG00000062338		-1.032689962	0.000435132	0.002194071
ENSMMUG00000054412		-0.709101511	0.000217563	0.001194377
ENSMMUG00000057486		0.470353576	0.000296153	0.001559845
ENSMMUG00000055556		3.899380953	0.000685012	0.003263381
ENSMMUG00000054043		-1.668583468	0.01025944	0.033120471
ENSMMUG00000057543	MyoD family inhibitor domain containing 2 [Source:HGNC Symbol;Acc:HGNC:53442]	0.726227162	0.000533633	0.00261545
ENSMMUG00000009425	actin, cytoplasmic 2-like [Source:NCBI gene;Acc:705671]	-0.471991652	0.015608381	0.046773661
ENSMMUG00000056158		1.536046576	7.86E-05	0.000482772
ENSMMUG00000054107		-0.655982436	0.004290039	0.015760501
ENSMMUG00000001849	interleukin 5 receptor subunit alpha [Source:VGNC Symbol;Acc:VGNC:73731]	-1.025467026	6.18E-07	6.26E-06
ENSMMUG00000017983	tRNA nucleotidyl transferase 1 [Source:VGNC Symbol;Acc:VGNC:79073]	0.763646403	0.005510148	0.019536835
ENSMMUG00000060798		-1.017288578	0.000158958	0.000901591
ENSMMUG00000057502		3.197844238	0.016279989	0.048426889
ENSMMUG00000018281	interleukin 17 receptor E [Source:VGNC Symbol;Acc:VGNC:73623]	-0.45822635	0.000959108	0.004372079
ENSMMUG00000018282	interleukin 17 receptor C [Source:VGNC Symbol;Acc:VGNC:84164]	-0.560473193	0.000775911	0.003624493
ENSMMUG00000018285	cysteine rich with EGF like domains 1 [Source:VGNC Symbol;Acc:VGNC:71407]	-0.678834541	4.73E-05	0.000309009
ENSMMUG00000018289	proline rich transmembrane protein 3 [Source:VGNC Symbol;Acc:VGNC:76324]	-0.541231384	0.001467533	0.00629887
ENSMMUG00000018290	ER membrane protein complex subunit 3 [Source:VGNC Symbol;Acc:VGNC:72091]	-0.685945222	0.003365132	0.012836245
ENSMMUG00000019933	TatD DNase domain containing 2 [Source:NCBI gene;Acc:701390]	1.126872359	0.00055387	0.00270279
ENSMMUG00000007195	tRNA splicing endonuclease subunit 2 [Source:VGNC Symbol;Acc:VGNC:82707]	0.732092306	0.01082768	0.034601449
ENSMMUG00000009536	makorin ring finger protein 2 [Source:VGNC Symbol;Acc:VGNC:74878]	-1.297500973	4.21E-08	5.42E-07
ENSMMUG00000023125	XPC complex subunit, DNA damage recognition and repair factor [Source:VGNC Symbol;Acc:VGNC:78809]	-0.684325201	0.002836969	0.011077362
ENSMMUG00000023124	transmembrane protein 43 [Source:VGNC Symbol;Acc:VGNC:82711]	-1.190714789	3.60E-11	8.14E-10
ENSMMUG00000006340	solute carrier family 6 member 6 [Source:VGNC Symbol;Acc:VGNC:77622]	-0.618980966	0.000190675	0.001060384
ENSMMUG00000044822	coiled-coil domain containing 174 [Source:VGNC Symbol;Acc:VGNC:84219]	1.137491356	5.26E-12	1.38E-10
ENSMMUG00000000158	minichromosome maintenance complex component 2 [Source:VGNC Symbol;Acc:VGNC:74680]	-1.251028495	1.81E-16	9.22E-15
ENSMMUG00000017125	oxysterol binding protein like 11 [Source:VGNC Symbol;Acc:VGNC:75631]	-1.493607456	1.36E-24	1.54E-22
ENSMMUG00000055571		2.333067503	0.008280666	0.027634537
ENSMMUG00000032031		-1.284772417	0.000614633	0.002963489
ENSMMUG00000001943	GRAM domain containing 1C [Source:VGNC Symbol;Acc:VGNC:73007]	0.7890243	0.002390347	0.009577743
ENSMMUG00000052581	upstream transcription factor family member 3 [Source:NCBI gene;Acc:709660]	-0.741618715	9.52E-07	9.21E-06

ENSMMUG00000057363		1.039420262	0.005948183	0.02082079
ENSMMUG00000014874	GTP binding protein 8 (putative) [Source:VGNC Symbol;Acc:VGNC:84180]	1.762240016	3.58E-16	1.77E-14
ENSMMUG00000014876	nucleolus and neural progenitor protein [Source:VGNC Symbol;Acc:VGNC:75244]	1.81805811	4.90E-14	1.78E-12
ENSMMUG00000003199	CD200 receptor 1 [Source:VGNC Symbol;Acc:VGNC:70845]	1.424936853	0.00085032	0.003944193
ENSMMUG00000050558		0.592794381	0.008634929	0.028678539
ENSMMUG00000000894	cellular inhibitor of PP2A [Source:VGNC Symbol;Acc:VGNC:71371]	2.088829729	0.001361623	0.005906869
ENSMMUG00000019977	myosin heavy chain 15 [Source:VGNC Symbol;Acc:VGNC:84148]	-1.169663934	3.61E-08	4.71E-07
ENSMMUG00000019978	HERV-H LTR-associating 2 [Source:VGNC Symbol;Acc:VGNC:73377]	-1.25097096	0.002955457	0.011490633
ENSMMUG00000008791	CD47 molecule [Source:VGNC Symbol;Acc:VGNC:70767]	3.405195342	1.14E-28	2.27E-26
ENSMMUG00000008787	BBX high mobility group box domain containing [Source:VGNC Symbol;Acc:VGNC:82110]	0.342929935	0.000589382	0.002854045
ENSMMUG00000055345	Y RNA [Source:RFAM;Acc:RF00019]	0.756539149	0.000221751	0.001213055
ENSMMUG00000007565	Cbl proto-oncogene B [Source:VGNC Symbol;Acc:VGNC:70555]	-0.808743347	8.88E-09	1.32E-07
ENSMMUG00000026103	U1 spliceosomal RNA [Source:RFAM;Acc:RF00003]	-1.637305364	0.00107408	0.004805757
ENSMMUG00000060744		-2.028866953	5.19E-11	1.13E-09
ENSMMUG00000063181		0.438774204	0.016934657	0.04994504
ENSMMUG00000059753		-0.532165981	0.000626653	0.003011454
ENSMMUG00000021567	neurexophilin and PC-esterase domain family member 3 [Source:VGNC Symbol;Acc:VGNC:75476]	-0.36000764	0.006000674	0.020969182
ENSMMUG00000057768		-0.82333113	7.17E-06	5.67E-05
ENSMMUG00000038349	crystallin beta-gamma domain containing 3 [Source:NCBI gene;Acc:698282]	0.674126808	1.88E-07	2.15E-06
ENSMMUG00000043727	zinc finger protein 654 [Source:VGNC Symbol;Acc:VGNC:82560]	-2.379488115	6.97E-07	6.98E-06
ENSMMUG00000047994	CGG triplet repeat binding protein 1 [Source:VGNC Symbol;Acc:VGNC:84203]	-1.705485898	5.83E-08	7.32E-07
ENSMMUG00000033738	RNA, U6atac small nuclear 6, pseudogene [Source:HGNC Symbol;Acc:HGNC:46905]	-0.955248698	3.59E-09	5.67E-08
ENSMMUG00000000519	vestigial like family member 3 [Source:VGNC Symbol;Acc:VGNC:79430]	-0.891353606	0.006035174	0.021069484
ENSMMUG00000050994		-1.433430727	0.001727606	0.007273155
ENSMMUG00000055096		1.144529051	2.27E-08	3.08E-07
ENSMMUG00000049913		1.288240432	0.00115504	0.005113832
ENSMMUG00000056687		-1.721901746	2.05E-13	6.58E-12
ENSMMUG00000052831		0.821136896	2.15E-05	0.000152434
ENSMMUG00000032295	zinc finger protein 595 [Source:VGNC Symbol;Acc:VGNC:79655]	-0.697802448	0.013942442	0.042660763
ENSMMUG00000047256	zinc finger protein 732 [Source:NCBI gene;Acc:716016]	-1.079910452	0.012466086	0.03887481
ENSMMUG00000051381	Metazoan signal recognition particle RNA [Source:RFAM;Acc:RF00017]	-1.976551881	4.02E-14	1.50E-12
ENSMMUG00000039020	zinc finger protein 141 [Source:NCBI gene;Acc:714265]	-1.647065303	1.96E-17	1.10E-15
ENSMMUG00000065084		-0.426035684	0.001087287	0.004851411
ENSMMUG00000008323	C-terminal binding protein 1 [Source:VGNC Symbol;Acc:VGNC:71539]	-0.691035171	7.13E-09	1.07E-07
ENSMMUG00000008357	macrophage erythroblast attacher [Source:VGNC Symbol;Acc:VGNC:74282]	0.529521574	0.009931187	0.032246967
ENSMMUG00000032306	zinc finger FYVE-type containing 28 [Source:NCBI gene;Acc:720600]	-0.75193418	0.000108808	0.000642724
ENSMMUG00000008379	adducin 1 [Source:VGNC Symbol;Acc:VGNC:69528]	1.342809881	1.82E-08	2.51E-07

ENSMMUG00000008383	major facilitator superfamily domain containing 10 [Source:NCBI gene;Acc:720098]	1.442792425	1.75E-09	2.94E-08
ENSMMUG00000008385	NOP14 nucleolar protein [Source:VGNC Symbol;Acc:VGNC:75194]	1.183366308	6.99E-13	2.07E-11
ENSMMUG00000008392	huntingtin [Source:VGNC Symbol;Acc:VGNC:73468]	-0.679382504	1.75E-06	1.58E-05
ENSMMUG00000006234	acyl-CoA oxidase 3, pristanoyl [Source:VGNC Symbol;Acc:VGNC:69382]	0.573384461	0.000329918	0.001711031
ENSMMUG00000030400	SH3 domain and tetratricopeptide repeats 1 [Source:NCBI gene;Acc:722649]	-0.523543354	0.000275246	0.001467273
ENSMMUG00000015099	janus kinase and microtubule interacting protein 1 [Source:VGNC Symbol;Acc:VGNC:73662]	-0.682782535	0.001534436	0.006547139
ENSMMUG00000019563	collapsin response mediator protein 1 [Source:VGNC Symbol;Acc:VGNC:71494]	1.18065825	6.93E-14	2.43E-12
ENSMMUG00000057703		1.148921338	7.16E-06	5.67E-05
ENSMMUG00000020861	EvC ciliary complex subunit 1 [Source:VGNC Symbol;Acc:VGNC:72445]	1.052181737	2.42E-07	2.70E-06
ENSMMUG00000016922	serine/threonine kinase 32B [Source:VGNC Symbol;Acc:VGNC:78114]	1.006022694	0.002335145	0.00940072
ENSMMUG00000060830		-2.856043941	0.003029182	0.01172709
ENSMMUG00000023394	zinc finger and BTB domain containing 49 [Source:VGNC Symbol;Acc:VGNC:79207]	-1.476378671	0.005119856	0.018327344
ENSMMUG00000023242	transmembrane protein 128 [Source:VGNC Symbol;Acc:VGNC:82257]	-0.70644516	0.000244594	0.001319927
ENSMMUG00000056982		0.379073573	0.001080737	0.004828111
ENSMMUG00000061061		3.803612535	0.001074001	0.004805757
ENSMMUG00000009388	F-box and leucine rich repeat protein 5 [Source:VGNC Symbol;Acc:VGNC:81408]	3.812499086	0.000540293	0.002642747
ENSMMUG00000061298		3.293296732	0.001737871	0.007303681
ENSMMUG00000015294	bone marrow stromal cell antigen 1 [Source:VGNC Symbol;Acc:VGNC:70285]	3.359659505	0.00625699	0.021713501
ENSMMUG00000015298	CD38 molecule [Source:VGNC Symbol;Acc:VGNC:70762]	1.373324039	0.000199134	0.001100273
ENSMMUG00000008162	fibroblast growth factor binding protein 1 [Source:VGNC Symbol;Acc:VGNC:72536]	1.228233571	1.91E-08	2.62E-07
ENSMMUG00000023116	LIM domain binding 2 [Source:VGNC Symbol;Acc:VGNC:74246]	-0.77753714	1.85E-06	1.66E-05
ENSMMUG00000018073	ligand dependent nuclear receptor corepressor like [Source:NCBI gene;Acc:715285]	-0.552358877	0.003486575	0.013226603
ENSMMUG00000057338		-1.09568017	3.61E-07	3.88E-06
ENSMMUG00000060440	leucine rich repeat LGI family member 2 [Source:NCBI gene;Acc:693308]	-0.428428657	7.80E-05	0.00047991
ENSMMUG00000003629	phosphatidylinositol 4-kinase type 2 beta [Source:NCBI gene;Acc:693561]	1.670950958	0.001276732	0.005585269
ENSMMUG00000049298	cholecystokinin A receptor [Source:NCBI gene;Acc:694742]	3.819873789	0.001623675	0.006893504
ENSMMUG00000057711		0.664448611	0.014116759	0.043112463
ENSMMUG00000009439	stromal interaction molecule 2 [Source:VGNC Symbol;Acc:VGNC:77965]	1.287979786	0.005964522	0.020867932
ENSMMUG00000051768		-1.122503992	0.000281743	0.001497514
ENSMMUG00000055079		0.443753483	0.004333245	0.015887207
ENSMMUG00000004471	family with sequence similarity 114 member A1 [Source:VGNC Symbol;Acc:VGNC:72342]	1.034902005	0.011522636	0.036385652
ENSMMUG00000002755	kelch like family member 5 [Source:VGNC Symbol;Acc:VGNC:74188]	-3.493457254	1.82E-06	1.64E-05
ENSMMUG00000002756	WD repeat domain 19 [Source:VGNC Symbol;Acc:VGNC:79132]	-0.361612711	0.011572657	0.036500697
ENSMMUG00000008834	klotho beta [Source:VGNC Symbol;Acc:VGNC:73835]	1.617283901	0.009798345	0.031901129
ENSMMUG00000001892	UDP-glucose 6-dehydrogenase [Source:VGNC Symbol;Acc:VGNC:78711]	-0.901169895	8.45E-05	0.000514294
ENSMMUG00000036851	U7 small nuclear RNA [Source:RFAM;Acc:RF00066]	-0.530308285	0.01630325	0.048476241
ENSMMUG00000012036	RNA binding motif protein 47 [Source:VGNC Symbol;Acc:VGNC:76576]	-0.816430005	1.35E-11	3.29E-10
ENSMMUG00000012561	NOP2/Sun RNA methyltransferase family member 7 [Source:VGNC Symbol;Acc:VGNC:75463]	-0.967261032	3.69E-09	5.81E-08
ENSMMUG00000055441		1.628885361	2.45E-05	0.000171495

ENSMMUG00000051296	paired like homeobox 2B [Source:NCBI gene;Acc:701940]	-1.646673279	0.000116484	0.000684174
ENSMMUG00000018625	NIPA like domain containing 1 [Source:NCBI gene;Acc:704998]	-0.667168739	0.015909943	0.047501097
ENSMMUG00000018628	tec protein tyrosine kinase [Source:VGNC Symbol;Acc:VGNC:78305]	0.573483671	0.001365129	0.005920309
ENSMMUG00000006703	solute carrier family 10 member 4 [Source:VGNC Symbol;Acc:VGNC:77553]	-1.029045196	4.27E-05	0.000283001
ENSMMUG00000006704	zygote arrest 1 [Source:VGNC Symbol;Acc:VGNC:79251]	-1.276162498	5.91E-08	7.41E-07
ENSMMUG00000014868	OCIA domain containing 1 [Source:VGNC Symbol;Acc:VGNC:75409]	1.559116584	9.22E-09	1.35E-07
ENSMMUG00000063785		1.274971426	0.003011158	0.011666631
ENSMMUG00000058229	U6 spliceosomal RNA [Source:RFAM;Acc:RF00026]	2.850155999	5.35E-06	4.35E-05
ENSMMUG00000008514	lin-54 DREAM MuvB core complex component [Source:VGNC Symbol;Acc:VGNC:74123]	1.014055355	0.016047232	0.047832401
ENSMMUG00000048621	enolase-phosphatase 1 [Source:NCBI gene;Acc:106998330]	1.565096037	2.18E-07	2.46E-06
ENSMMUG00000060484	heterogeneous nuclear ribonucleoprotein D [Source:NCBI gene;Acc:694867]	0.771522038	0.002385996	0.009563061
ENSMMUG00000052348		1.314923719	9.14E-07	8.87E-06
ENSMMUG00000021988	N-alpha-acetyltransferase 11, NatA catalytic subunit [Source:NCBI gene;Acc:696758]	1.273754529	7.83E-09	1.17E-07
ENSMMUG00000007366		1.159477336	2.35E-06	2.07E-05
ENSMMUG00000002564	Fraser extracellular matrix complex subunit 1 [Source:VGNC Symbol;Acc:VGNC:72743]	1.104460486	6.73E-10	1.22E-08
ENSMMUG00000045609	CCR4-NOT transcription complex subunit 6 like [Source:NCBI gene;Acc:697981]	2.178654214	1.84E-16	9.29E-15
ENSMMUG00000018186	sosondowah ankyrin repeat domain family member B [Source:VGNC Symbol;Acc:VGNC:78033]	-0.543925889	2.05E-07	2.33E-06
ENSMMUG00000020916	coiled-coil domain containing 158 [Source:NCBI gene;Acc:699735]	0.885044382	4.60E-09	7.12E-08
ENSMMUG00000020913	scavenger receptor class B member 2 [Source:VGNC Symbol;Acc:VGNC:77026]	-0.930732588	0.000368272	0.001884398
ENSMMUG00000020910	nucleoporin 54 [Source:VGNC Symbol;Acc:VGNC:75420]	-1.389146452	1.26E-07	1.49E-06
ENSMMUG00000017503	SDA1 domain containing 1 [Source:NCBI gene;Acc:700863]	2.256825348	1.72E-05	0.000123888
ENSMMUG00000017501	N-acylethanolamine acid amidase [Source:VGNC Symbol;Acc:VGNC:75068]	2.080474916	2.07E-07	2.35E-06
ENSMMUG00000015716	protein phosphatase with EF-hand domain 2 [Source:VGNC Symbol;Acc:VGNC:76233]	1.454895555	0.007503764	0.02545676
ENSMMUG00000003833	Ras association domain family member 6 [Source:VGNC Symbol;Acc:VGNC:76515]	2.38149905	4.83E-13	1.46E-11
ENSMMUG00000003830	alpha fetoprotein [Source:VGNC Symbol;Acc:VGNC:69741]	1.332844847	0.001060032	0.004757546
ENSMMUG00000003827	albumin [Source:VGNC Symbol;Acc:VGNC:69782]	1.151461408	3.32E-06	2.83E-05
ENSMMUG00000000790	ankyrin repeat domain 17 [Source:VGNC Symbol;Acc:VGNC:84240]	3.297052449	2.09E-33	6.08E-31
ENSMMUG00000062248	follicular dendritic cell secreted protein [Source:NCBI gene;Acc:707784]	0.728828875	0.002007229	0.008296584
ENSMMUG00000006029	odontogenic, ameloblast associated [Source:NCBI gene;Acc:707906]	1.278361427	1.05E-07	1.25E-06
ENSMMUG00000048823		-1.052871071	6.07E-08	7.58E-07
ENSMMUG00000004849	casein beta [Source:VGNC Symbol;Acc:VGNC:71523]	-1.034584248	3.92E-07	4.17E-06
ENSMMUG00000047324	casein alpha s1 [Source:HGNC Symbol;Acc:HGNC:2445]	-2.388205026	3.36E-05	0.000227328
ENSMMUG00000001919	clock circadian regulator [Source:VGNC Symbol;Acc:VGNC:71262]	-0.996734876	1.23E-08	1.77E-07
ENSMMUG00000001918	transmembrane protein 165 [Source:VGNC Symbol;Acc:VGNC:79875]	-1.735317784	3.53E-07	3.81E-06
ENSMMUG00000010113	steroid 5 alpha-reductase 3 [Source:VGNC Symbol;Acc:VGNC:82246]	-1.46736104	7.63E-08	9.35E-07
ENSMMUG00000052882	RAS like family 11 member B [Source:NCBI gene;Acc:701397]	1.980432854	0.004622288	0.016773389
ENSMMUG00000018943	spermatogenesis associated 18 [Source:VGNC Symbol;Acc:VGNC:78013]	-1.313449602	5.65E-06	4.57E-05
ENSMMUG00000018939	leucine rich repeat containing 66 [Source:VGNC Symbol;Acc:VGNC:74415]	-0.878825993	7.17E-05	0.000444463
ENSMMUG00000008579	defective in cullin neddylation 1 domain containing 4 [Source:VGNC Symbol;Acc:VGNC:71809]	-0.901833004	0.006562792	0.022596708

ENSMMUG00000008341	Rho GTPase activating protein 24 [Source:VGNC Symbol;Acc:VGNC:69904]	-1.123957264	9.69E-05	0.000580184
ENSMMUG00000003726	protein tyrosine phosphatase non-receptor type 13 [Source:VGNC Symbol;Acc:VGNC:76599]	-0.905127124	3.69E-05	0.000247181
ENSMMUG00000014076	AF4/FMR2 family member 1 [Source:VGNC Symbol;Acc:VGNC:69817]	2.119988056	7.48E-09	1.12E-07
ENSMMUG00000051677		1.194453453	2.59E-08	3.47E-07
ENSMMUG00000014080	kelch like family member 8 [Source:VGNC Symbol;Acc:VGNC:74076]	-0.760468546	0.001653405	0.007005407
ENSMMUG00000019040	hydroxysteroid 17-beta dehydrogenase 13 [Source:VGNC Symbol;Acc:VGNC:73417]	0.710395882	2.95E-05	0.000201979
ENSMMUG00000019041	hydroxysteroid 17-beta dehydrogenase 11 [Source:VGNC Symbol;Acc:VGNC:73431]	0.729743895	0.004793881	0.01730965
ENSMMUG00000008795	polycystin 2, transient receptor potential cation channel [Source:NCBI gene;Acc:702179]	-1.232966391	6.71E-09	1.01E-07
ENSMMUG00000008797	ATP binding cassette subfamily G member 2 (Junior blood group) [Source:NCBI gene;Acc:574307]	-1.02677668	2.11E-09	3.47E-08
ENSMMUG00000028039	U6 spliceosomal RNA [Source:RFAM;Acc:RF00026]	-1.904010295	0.007037319	0.024020078
ENSMMUG00000010314	HECT and RLD domain containing E3 ubiquitin protein ligase family member 6 [Source:VGNC Symbol;Acc:VGNC:73238]	1.942034514	1.05E-10	2.18E-09
ENSMMUG00000010316	HECT and RLD domain containing E3 ubiquitin protein ligase 5 [Source:VGNC Symbol;Acc:VGNC:73226]	4.055313155	1.04E-34	3.86E-32
ENSMMUG00000010318	phosphatidylinositol glycan anchor biosynthesis class Y [Source:NCBI gene;Acc:100426107]	4.574840584	2.67E-34	9.24E-32
ENSMMUG00000010138	HECT and RLD domain containing E3 ubiquitin protein ligase 3 [Source:VGNC Symbol;Acc:VGNC:73365]	0.768180663	1.06E-06	1.02E-05
ENSMMUG00000058544	nucleosome assembly protein 1 like 5 [Source:NCBI gene;Acc:703223]	0.925129468	1.35E-08	1.92E-07
ENSMMUG00000010141	family with sequence similarity 13 member A [Source:VGNC Symbol;Acc:VGNC:72299]	1.023716382	1.75E-20	1.40E-18
ENSMMUG00000032793	U6 spliceosomal RNA [Source:RFAM;Acc:RF00026]	-1.719161887	0.016760396	0.049622379
ENSMMUG00000057606	GPRIN family member 3 [Source:NCBI gene;Acc:703567]	-1.787527945	6.08E-17	3.24E-15
ENSMMUG00000049396	Metazoan signal recognition particle RNA [Source:RFAM;Acc:RF00017]	-1.669589195	0.000467646	0.002333737
ENSMMUG00000022003	multimerin 1 [Source:VGNC Symbol;Acc:VGNC:74904]	2.672010342	3.05E-15	1.34E-13
ENSMMUG00000022246	PDZ and LIM domain 5 [Source:VGNC Symbol;Acc:VGNC:75816]	1.290825183	1.83E-08	2.52E-07
ENSMMUG00000060760		1.572125298	2.45E-09	3.99E-08
ENSMMUG00000006702	bone morphogenetic protein receptor type 1B [Source:VGNC Symbol;Acc:VGNC:70267]	-0.978635117	0.015946538	0.047581039
ENSMMUG00000006309	unc-5 netrin receptor C [Source:VGNC Symbol;Acc:VGNC:78723]	-2.58220348	7.96E-06	6.20E-05
ENSMMUG00000011702	tetraspanin 5 [Source:VGNC Symbol;Acc:VGNC:79086]	-1.336280355	2.77E-06	2.40E-05
ENSMMUG00000063972		-0.833163083	0.000159562	0.000904663
ENSMMUG00000040764	alcohol dehydrogenase 1A [Source:NCBI gene;Acc:100424103]	-0.880497641	0.00014912	0.000851769
ENSMMUG00000018738	alcohol dehydrogenase 1B (class I), beta polypeptide [Source:NCBI gene;Acc:707908]	-0.700168449	0.000290447	0.001532571
ENSMMUG00000061366		0.617376197	0.000479594	0.002382727
ENSMMUG00000016880	solute carrier family 39 member 8 [Source:VGNC Symbol;Acc:VGNC:77541]	-2.916957847	3.36E-13	1.04E-11
ENSMMUG00000037551	Metazoan signal recognition particle RNA [Source:RFAM;Acc:RF00017]	-2.27895057	6.26E-08	7.81E-07
ENSMMUG00000003736	nuclear factor kappa B subunit 1 [Source:VGNC Symbol;Acc:VGNC:75339]	-3.127979639	8.08E-12	2.04E-10
ENSMMUG00000003739	mannosidase beta [Source:VGNC Symbol;Acc:VGNC:74486]	1.459121637	0.000855615	0.003961173
ENSMMUG00000011206	solute carrier family 9 member B1 [Source:NCBI gene;Acc:710949]	0.696650138	5.70E-05	0.000363809
ENSMMUG00000019083	solute carrier family 9 member B2 [Source:VGNC Symbol;Acc:VGNC:77773]	0.676671875	0.015219345	0.045823847
ENSMMUG00000019085	3-hydroxybutyrate dehydrogenase 2 [Source:VGNC Symbol;Acc:VGNC:70161]	1.276413571	1.63E-16	8.33E-15
ENSMMUG00000019089	centromere protein E [Source:VGNC Symbol;Acc:VGNC:71008]	1.326133048	2.14E-14	8.30E-13
ENSMMUG00000049998	tachykinin receptor 3 [Source:NCBI gene;Acc:653013]	0.839148856	0.012253106	0.038290689

ENSMMUG00000010941	Rho guanine nucleotide exchange factor 38 [Source:VGNC Symbol;Acc:VGNC:82087]	-0.418729772	0.000243323	0.00131396
ENSMMUG00000060432		-0.841570265	0.015931064	0.047544628
ENSMMUG00000052609	ribosomal protein L34 [Source:NCBI gene;Acc:696636]	-1.851415166	1.35E-06	1.26E-05
ENSMMUG00000011902	oligosaccharyltransferase complex non-catalytic subunit [Source:NCBI gene;Acc:697283]	-1.963755811	1.76E-09	2.94E-08
ENSMMUG00000055759	Metazoan signal recognition particle RNA [Source:RFAM;Acc:RF00017]	-1.631003815	3.10E-07	3.38E-06
ENSMMUG00000062794		3.365378096	4.05E-26	5.77E-24
ENSMMUG00000009816	exosome component 9 [Source:VGNC Symbol;Acc:VGNC:72477]	1.625746618	0.000230431	0.001252251
ENSMMUG00000061098		1.846271043	4.59E-35	1.85E-32
ENSMMUG00000007422	spermatogenesis associated 5 [Source:VGNC Symbol;Acc:VGNC:77952]	1.562603104	2.11E-10	4.16E-09
ENSMMUG00000001848	sprouty RTK signaling antagonist 1 [Source:VGNC Symbol;Acc:VGNC:77867]	1.27405504	9.12E-10	1.60E-08
ENSMMUG00000011810	FAT atypical cadherin 4 [Source:VGNC Symbol;Acc:VGNC:72552]	-1.45024355	0.000748498	0.003519052
ENSMMUG00000057895		-1.792758938	0.011046822	0.035176231
ENSMMUG00000010444	ring finger protein 150 [Source:VGNC Symbol;Acc:VGNC:76805]	3.077753714	0.001402267	0.006054727
ENSMMUG00000060157	zinc finger protein 330 [Source:NCBI gene;Acc:699364]	0.702746992	7.10E-05	0.000440501
ENSMMUG00000003697	inositol polyphosphate-4-phosphatase type II B [Source:VGNC Symbol;Acc:VGNC:73748]	1.241368181	2.43E-07	2.71E-06
ENSMMUG00000000827	ubiquitin specific peptidase 38 [Source:VGNC Symbol;Acc:VGNC:79224]	2.444190498	1.01E-05	7.72E-05
ENSMMUG00000044429	glycophorin A (MNS blood group) [Source:NCBI gene;Acc:574101]	2.211029901	4.27E-17	2.35E-15
ENSMMUG00000014173		-1.040387498	0.001852314	0.007719922
ENSMMUG00000007968	hedgehog interacting protein [Source:VGNC Symbol;Acc:VGNC:73169]	-1.329791198	1.93E-08	2.65E-07
ENSMMUG00000005380	zinc finger protein 827 [Source:NCBI gene;Acc:706018]	1.399200456	3.37E-08	4.43E-07
ENSMMUG00000014955	nuclear receptor subfamily 3 group C member 2 [Source:VGNC Symbol;Acc:VGNC:75516]	-1.163084768	0.002469734	0.009860622
ENSMMUG00000002211	doublecortin like kinase 2 [Source:VGNC Symbol;Acc:VGNC:71670]	1.012959807	0.000110352	0.000650788
ENSMMUG00000002212	LPS responsive beige-like anchor protein [Source:NCBI gene;Acc:693823]	-1.263859166	0.000763777	0.003580465
ENSMMUG00000059101		-1.854871676	6.23E-06	4.99E-05
ENSMMUG00000063665		-1.376345091	0.001955028	0.008099242
ENSMMUG00000018490	ADP ribosylation factor interacting protein 1 [Source:VGNC Symbol;Acc:VGNC:69878]	-1.030813958	2.82E-08	3.75E-07
ENSMMUG00000018493	FH2 domain containing 1 [Source:VGNC Symbol;Acc:VGNC:72598]	-0.685454708	0.011231217	0.035655863
ENSMMUG00000039869		-0.480260402	0.001351078	0.005866372
ENSMMUG00000009001	transmembrane 131 like [Source:VGNC Symbol;Acc:VGNC:79781]	-0.904889421	0.000981434	0.004454296
ENSMMUG00000057643		0.471795867	0.001435956	0.00617954
ENSMMUG00000052999	lecithin retinol acyltransferase [Source:NCBI gene;Acc:698871]	0.753322015	7.41E-12	1.89E-10
ENSMMUG00000064074		0.717187597	2.45E-05	0.000171392
ENSMMUG00000021147	RNA binding motif protein 46 [Source:VGNC Symbol;Acc:VGNC:76555]	0.598793108	0.000686444	0.003267464
ENSMMUG00000005447	guanylate cyclase 1 soluble subunit beta 1 [Source:VGNC Symbol;Acc:VGNC:73328]	-0.538573139	0.001505911	0.006442444
ENSMMUG00000057700	acid sensing ion channel subunit family member 5 [Source:NCBI gene;Acc:699617]	-0.566330269	4.97E-06	4.07E-05
ENSMMUG00000022018	tryptophan 2,3-dioxygenase [Source:VGNC Symbol;Acc:VGNC:78298]	-0.467147124	0.001499091	0.006415155

ENSMMUG00000064707		1.888075508	2.98E-10	5.75E-09
ENSMMUG00000053790		3.278098565	5.69E-33	1.56E-30
ENSMMUG00000045101		0.550778122	0.012755851	0.039571448
ENSMMUG00000006851	SPARC (osteonectin), cwcv and kazal like domains proteoglycan 3 [Source:NCBI gene;Acc:705964]	4.536340072	4.18E-32	1.06E-29
ENSMMUG00000001879	annexin A10 [Source:VGNC Symbol;Acc:VGNC:69945]	0.569359608	0.009383092	0.030769683
ENSMMUG00000001880	DExD/H-box helicase 60 [Source:VGNC Symbol;Acc:VGNC:71711]	0.583477427	5.61E-05	0.000358413
ENSMMUG00000003634	DExD/H-box 60 like [Source:VGNC Symbol;Acc:VGNC:71720]	-1.16152306	2.49E-05	0.000173749
ENSMMUG00000013730	WD repeat domain 17 [Source:NCBI gene;Acc:700546]	0.40122529	0.000513671	0.002533843
ENSMMUG00000064304		-0.36647425	0.012896971	0.039958591
ENSMMUG00000065183		4.346353576	0.000211982	0.001165502
ENSMMUG00000058974		-0.965632697	0.008595204	0.02857925
ENSMMUG00000049670		-3.408237567	0.005115903	0.018317704
ENSMMUG00000011633	kallikrein B1 [Source:VGNC Symbol;Acc:VGNC:74195]	2.470932962	2.54E-05	0.000176608
ENSMMUG00000011634	coagulation factor XI [Source:VGNC Symbol;Acc:VGNC:72460]	2.554529593	0.000366491	0.001877267
ENSMMUG00000018051	FAT atypical cadherin 1 [Source:VGNC Symbol;Acc:VGNC:72617]	-0.690877682	1.65E-07	1.92E-06
ENSMMUG00000050600		2.288480069	0.01476645	0.044711496
ENSMMUG00000058448		0.58340838	0.011969924	0.037558122
ENSMMUG00000050465	ZFP42 zinc finger protein [Source:NCBI gene;Acc:707621]	1.781333042	9.88E-08	1.18E-06
ENSMMUG00000054799		-1.576588698	0.003806631	0.014247422
ENSMMUG00000064721		-1.922160385	0.001418029	0.006113252
ENSMMUG00000052287		-2.16087261	2.32E-05	0.000163368
ENSMMUG00000044628		-1.901026572	0.000119695	0.000701052
ENSMMUG00000012914	pericentrin [Source:VGNC Symbol;Acc:VGNC:75619]	-0.435106361	0.007712822	0.02603234
ENSMMUG00000022324	formimidoyltransferase cyclodeaminase [Source:VGNC Symbol;Acc:VGNC:72825]	-1.168387133	0.007386476	0.025129229
ENSMMUG00000062496		3.09963699	0.011419037	0.036155036
ENSMMUG00000000544	collagen type VI alpha 1 chain [Source:VGNC Symbol;Acc:VGNC:82084]	2.50631676	0.00595668	0.020845512
ENSMMUG00000052777		1.60889575	7.49E-06	5.89E-05
ENSMMUG00000004477	solute carrier family 19 member 1 [Source:VGNC Symbol;Acc:VGNC:77408]	0.917991238	0.014873649	0.044970514
ENSMMUG00000010695	adenosine deaminase RNA specific B1 [Source:VGNC Symbol;Acc:VGNC:69503]	-1.465167035	9.45E-07	9.15E-06
ENSMMUG00000000953	family with sequence similarity 207 member A [Source:VGNC Symbol;Acc:VGNC:72273]	0.910214701	0.000148182	0.000847749
ENSMMUG00000000951	integrin subunit beta 2 [Source:VGNC Symbol;Acc:VGNC:73786]	2.130753094	2.82E-28	5.32E-26
ENSMMUG00000046352	PTTG1 interacting protein [Source:VGNC Symbol;Acc:VGNC:81748]	0.905469987	0.010273741	0.033159276
ENSMMUG00000049755	leucine rich repeat containing 3 [Source:NCBI gene;Acc:712418]	2.62017454	1.08E-06	1.03E-05
ENSMMUG00000004175	transient receptor potential cation channel subfamily M member 2 [Source:VGNC Symbol;Acc:VGNC:78655]	0.611619612	0.015011221	0.045310352
ENSMMUG00000049578	cilia and flagella associated protein 410 [Source:NCBI gene;Acc:722168]	-0.311680433	0.010957678	0.034947733
ENSMMUG00000004173	phosphofructokinase, liver type [Source:VGNC Symbol;Acc:VGNC:81737]	-0.707266283	4.70E-05	0.000307313
ENSMMUG00000010648	DNA methyltransferase 3 like [Source:NCBI gene;Acc:722184]	-1.830604819	5.53E-12	1.44E-10
ENSMMUG00000045179	1-acylglycerol-3-phosphate O-acyltransferase 3 [Source:NCBI gene;Acc:713301]	0.63785221	0.000514653	0.002536965
ENSMMUG00000012304	ribosomal RNA processing 1 [Source:VGNC Symbol;Acc:VGNC:77050]	-0.648521538	0.001368538	0.005931554
ENSMMUG00000012303	cystatin B [Source:VGNC Symbol;Acc:VGNC:71534]	1.3002282	0.006556355	0.022579896

ENSMMUG00000062457	pyridoxal kinase [Source:NCBI gene;Acc:713564]	-0.623068076	0.003358106	0.012818619
ENSMMUG00000000104	ribosomal RNA processing 1B [Source:VGNC Symbol;Acc:VGNC:77089]	-1.293506259	1.14E-13	3.84E-12
ENSMMUG00000022791	heat shock transcription factor 2 binding protein [Source:VGNC Symbol;Acc:VGNC:73532]	-0.939818908	0.01193312	0.037458813
ENSMMUG00000020050	salt inducible kinase 1 [Source:NCBI gene;Acc:722303]	-1.793733845	0.000172199	0.000967253
ENSMMUG00000022107	phosphodiesterase 9A [Source:VGNC Symbol;Acc:VGNC:75925]	-2.368473587	0.008131836	0.0272003
ENSMMUG00000018081	ubiquitin associated and SH3 domain containing A [Source:VGNC Symbol;Acc:VGNC:78687]	-1.043939615	1.55E-05	0.000113096
ENSMMUG00000018079	transmembrane serine protease 3 [Source:VGNC Symbol;Acc:VGNC:78576]	-1.254535986	3.12E-07	3.40E-06
ENSMMUG00000018058	ATP binding cassette subfamily G member 1 [Source:VGNC Symbol;Acc:VGNC:69584]	-1.740903701	1.10E-11	2.72E-10
ENSMMUG00000018057	uromodulin like 1 [Source:VGNC Symbol;Acc:VGNC:78717]	-1.3255404	3.77E-06	3.18E-05
ENSMMUG00000018053	zinc finger and BTB domain containing 21 [Source:VGNC Symbol;Acc:VGNC:78892]	-0.517937995	0.000310102	0.001622151
ENSMMUG00000015329	MX dynamin like GTPase 1 [Source:VGNC Symbol;Acc:VGNC:75086]	3.499816257	5.89E-23	5.63E-21
ENSMMUG00000044257	MX dynamin like GTPase 2 [Source:VGNC Symbol;Acc:VGNC:81723]	4.166778446	1.11E-37	6.00E-35
ENSMMUG00000004257	beta-secretase 2 [Source:VGNC Symbol;Acc:VGNC:70216]	3.312260182	2.65E-32	6.89E-30
ENSMMUG00000008498	proteasome assembly chaperone 1 [Source:VGNC Symbol;Acc:VGNC:76347]	-0.398362331	0.005777288	0.020340139
ENSMMUG00000058004		1.087911484	0.013612963	0.041828713
ENSMMUG00000056593		2.959946836	0.000163587	0.000923878
ENSMMUG00000017505	tetratricopeptide repeat domain 3 [Source:VGNC Symbol;Acc:VGNC:81785]	0.669398536	2.93E-08	3.88E-07
ENSMMUG00000062048		0.808952337	8.98E-10	1.59E-08
ENSMMUG00000010336	DOP1 leucine zipper like protein B [Source:VGNC Symbol;Acc:VGNC:71832]	0.835896479	7.75E-07	7.64E-06
ENSMMUG00000064897		-1.119067811	0.002569994	0.010182611
ENSMMUG00000001649	RUNX family transcription factor 1 [Source:VGNC Symbol;Acc:VGNC:76946]	-0.443783264	0.015675964	0.046947044
ENSMMUG00000054417		-0.693730557	8.82E-06	6.80E-05
ENSMMUG00000005511	SON DNA binding protein [Source:VGNC Symbol;Acc:VGNC:77689]	1.42692995	2.70E-07	2.99E-06
ENSMMUG00000022702	phosphoribosylglycinamide formyltransferase, phosphoribosylglycinamide synthetase, phosphoribosylaminoimidazole synthetase [Source:VGNC Symbol;Acc:VGNC:72882]	0.968933832	0.006543521	0.022541035
ENSMMUG00000003956	interferon alpha and beta receptor subunit 2 [Source:VGNC Symbol;Acc:VGNC:81330]	0.756581015	4.50E-06	3.72E-05
ENSMMUG00000065005	Metazoan signal recognition particle RNA [Source:RFAM;Acc:RF00017]	1.322453794	1.29E-06	1.21E-05
ENSMMUG00000052780		1.579944161	1.86E-17	1.05E-15
ENSMMUG00000027541	Small nucleolar RNA SNORA70 [Source:RFAM;Acc:RF00156]	2.341020462	0.013444304	0.041406746
ENSMMUG00000004624	synaptojanin 1 [Source:VGNC Symbol;Acc:VGNC:78071]	-0.54802112	0.000558561	0.002723852
ENSMMUG00000010304	cilia and flagella associated protein 298 [Source:NCBI gene;Acc:700721]	-0.510340639	6.03E-07	6.16E-06
ENSMMUG00000048824	eva-1 homolog C [Source:NCBI gene;Acc:708681]	1.280064746	2.09E-18	1.32E-16
ENSMMUG00000010295	URB1 ribosome biogenesis homolog [Source:VGNC Symbol;Acc:VGNC:79074]	-1.697381934	0.005203624	0.018586015
ENSMMUG00000059707	MIS18 kinetochore protein A [Source:NCBI gene;Acc:709275]	-2.037682515	3.66E-07	3.93E-06
ENSMMUG00000064916		-1.752822524	0.010794278	0.034509879
ENSMMUG00000056363		-2.176306104	8.34E-07	8.16E-06
ENSMMUG00000004886	glutamate ionotropic receptor kainate type subunit 1 [Source:VGNC Symbol;Acc:VGNC:73277]	3.28301989	0.000622108	0.002993573

ENSMMUG00000011095	BTB domain and CNC homolog 1 [Source:VGNC Symbol;Acc:VGNC:70217]	2.562498721	8.89E-06	6.85E-05
ENSMMUG00000033200	Small nucleolar RNA U3 [Source:RFAM;Acc:RF00012]	3.200848834	0.000650788	0.003117135
ENSMMUG00000061208	MAP3K7 C-terminal like [Source:NCBI gene;Acc:713717]	1.569707065	1.68E-26	2.56E-24
ENSMMUG00000003020	ubiquitin specific peptidase 16 [Source:VGNC Symbol;Acc:VGNC:78738]	3.313515926	0.006095272	0.021248672
ENSMMUG00000017605	mitochondrial ribosomal protein L39 [Source:VGNC Symbol;Acc:VGNC:74737]	1.501887242	4.60E-14	1.69E-12
ENSMMUG00000018743	ubiquitin specific peptidase 25 [Source:VGNC Symbol;Acc:VGNC:78744]	4.13637004	5.24E-07	5.42E-06
ENSMMUG00000052195		2.682782055	0.001810827	0.007573069
ENSMMUG00000060808		2.92346994	4.75E-05	0.000310071
ENSMMUG00000058750	nuclear receptor interacting protein 1 [Source:NCBI gene;Acc:693866]	1.047037535	9.66E-05	0.000578635
ENSMMUG00000020090	ATP-binding cassette transporter 13 [Source:NCBI gene;Acc:574326]	-0.445427564	0.006092545	0.02124426
ENSMMUG00000060576		1.539963907	4.74E-17	2.55E-15
ENSMMUG00000057702		1.393817571	4.26E-18	2.60E-16
ENSMMUG00000060168		-0.581704771	0.006116961	0.021289917
ENSMMUG00000017056	Sad1 and UNC84 domain containing 1 [Source:VGNC Symbol;Acc:VGNC:78161]	2.042517672	9.07E-05	0.000547712
ENSMMUG00000007206	chromosome 3 C7orf50 homolog [Source:VGNC Symbol;Acc:VGNC:70609]	2.375089032	0.000148799	0.000850608
ENSMMUG00000003283	extracellular leucine rich repeat and fibronectin type III domain containing 1 [Source:VGNC Symbol;Acc:VGNC:72204]	0.860151902	1.58E-06	1.45E-05
ENSMMUG00000022624	putative speedy protein E16 [Source:NCBI gene;Acc:718058]	-1.082669231	3.08E-06	2.65E-05
ENSMMUG00000021075	Rac family small GTPase 1 [Source:NCBI gene;Acc:717755]	1.050613813	3.91E-15	1.68E-13
ENSMMUG00000003167	cytohesin 3 [Source:VGNC Symbol;Acc:VGNC:71829]	1.372732528	6.49E-10	1.19E-08
ENSMMUG00000030422	radial spoke head 10 homolog B [Source:NCBI gene;Acc:100424218]	-0.541301957	1.67E-05	0.000120638
ENSMMUG00000007970	CCZ1 homolog, vacuolar protein trafficking and biogenesis associated [Source:NCBI gene;Acc:718128]	1.156596152	3.31E-06	2.81E-05
ENSMMUG00000015477	lemur tyrosine kinase 2 [Source:VGNC Symbol;Acc:VGNC:74309]	0.698271239	4.92E-06	4.04E-05
ENSMMUG00000015479	tectonin beta-propeller repeat containing 1 [Source:VGNC Symbol;Acc:VGNC:79348]	1.251439647	1.91E-25	2.50E-23
ENSMMUG00000049558	brain protein I3 [Source:NCBI gene;Acc:706525]	-0.75894613	0.00058156	0.002821807
ENSMMUG00000013276	BAR/IMD domain containing adaptor protein 2 like 1 [Source:VGNC Symbol;Acc:VGNC:70109]	-0.693793619	5.46E-05	0.000350935
ENSMMUG00000023318	transformation/transcription domain associated protein [Source:VGNC Symbol;Acc:VGNC:78657]	0.639269213	1.03E-11	2.55E-10
ENSMMUG00000040393	zinc finger protein 655 [Source:NCBI gene;Acc:718811]	2.344491663	1.18E-13	3.96E-12
ENSMMUG00000055483	transmembrane protein 225B [Source:NCBI gene;Acc:718838]	1.99432172	0.003879809	0.014472872
ENSMMUG00000022728	zinc finger and SCAN domain containing 25 [Source:VGNC Symbol;Acc:VGNC:79344]	1.409976231	3.21E-07	3.49E-06
ENSMMUG00000022730	cytochrome P450, family 3, subfamily A, polypeptide 5 [Source:NCBI gene;Acc:678692]	0.98304652	2.80E-12	7.57E-11
ENSMMUG00000050824		-1.411925187	0.009160632	0.030169485
ENSMMUG00000022490	cytochrome P450, family 3, subfamily A, polypeptide 43 [Source:NCBI gene;Acc:709770]	-0.549505922	3.37E-05	0.000227537
ENSMMUG00000063929	olfactory receptor 2AE1 [Source:NCBI gene;Acc:106997573]	-0.563922549	0.013800776	0.04229852
ENSMMUG00000022504	alpha-2-glycoprotein 1, zinc-binding [Source:VGNC Symbol;Acc:VGNC:70201]	0.568667456	0.0019165	0.007956672
ENSMMUG00000004021	zinc finger protein 3 [Source:VGNC Symbol;Acc:VGNC:81889]	-1.533619161	0.013466931	0.04145887
ENSMMUG00000013440	COP9 signalosome subunit 6 [Source:VGNC Symbol;Acc:VGNC:71286]	0.407477751	0.011939786	0.037471648
ENSMMUG00000013442	minichromosome maintenance complex component 7 [Source:VGNC Symbol;Acc:VGNC:74686]	0.458033826	0.005315111	0.018933049
ENSMMUG00000013447	adaptor related protein complex 4 subunit mu 1 [Source:VGNC Symbol;Acc:VGNC:69974]	1.042024996	0.000890458	0.004101581
ENSMMUG00000013450	TATA-box binding protein associated factor 6 [Source:VGNC Symbol;Acc:VGNC:78093]	2.279094251	5.69E-08	7.17E-07
ENSMMUG00000013452	canopy FGF signaling regulator 4 [Source:VGNC Symbol;Acc:VGNC:71364]	1.819164475	0.000772779	0.003615673
ENSMMUG00000046663	Metazoan signal recognition particle RNA [Source:RFAM;Acc:RF00017]	-0.810324243	6.33E-05	0.000398239
ENSMMUG00000048302	ArfGAP with FG repeats 2 [Source:VGNC Symbol;Acc:VGNC:80329]	-0.984757097	6.53E-13	1.94E-11

ENSMMUG00000015311	leucine rich repeats and calponin homology domain containing 4 [Source:VGNC Symbol;Acc:VGNC:74441]	-0.985504845	4.68E-16	2.31E-14
ENSMMUG00000015309	F-box protein 24 [Source:VGNC Symbol;Acc:VGNC:72495]	-1.152037079	1.21E-15	5.68E-14
ENSMMUG00000002947	procollagen C-endopeptidase enhancer [Source:VGNC Symbol;Acc:VGNC:75782]	-1.122064868	8.17E-18	4.77E-16
ENSMMUG00000002949	motile sperm domain containing 3 [Source:VGNC Symbol;Acc:VGNC:74823]	1.828666007	2.73E-09	4.40E-08
ENSMMUG00000002950	transferrin receptor 2 [Source:VGNC Symbol;Acc:VGNC:81770]	1.803463348	3.12E-16	1.55E-14
ENSMMUG00000004082	GRB10 interacting GYF protein 1 [Source:VGNC Symbol;Acc:VGNC:73158]	-1.727091192	1.10E-05	8.37E-05
ENSMMUG00000022840	zonadhesin [Source:VGNC Symbol;Acc:VGNC:81797]	-1.019732601	2.96E-06	2.55E-05
ENSMMUG00000052734		2.36817862	0.000820933	0.003817628
ENSMMUG00000060459		0.414519638	0.005425034	0.01927266
ENSMMUG00000063570	Metazoan signal recognition particle RNA [Source:RFAM;Acc:RF00017]	-3.52758075	0.000866957	0.004009848
ENSMMUG00000011367	BUD23 rRNA methyltransferase and ribosome maturation factor [Source:VGNC Symbol;Acc:VGNC:70325]	-3.067968306	0.001807912	0.007563057
ENSMMUG00000050132		0.918596358	0.000349432	0.001801946
ENSMMUG00000056980		2.304754466	0.000232987	0.00126378
ENSMMUG00000001225	calneuron 1 [Source:VGNC Symbol;Acc:VGNC:82078]	-1.734915655	2.04E-06	1.82E-05
ENSMMUG00000052019		-1.236854018	9.50E-05	0.000569678
ENSMMUG00000061002		-1.086674775	5.93E-05	0.000375858
ENSMMUG00000021235	vitamin K epoxide reductase complex subunit 1 like 1 [Source:VGNC Symbol;Acc:VGNC:79322]	-0.979747368	0.005677347	0.020041671
ENSMMUG00000049696		-0.42900857	0.000110844	0.000653424
ENSMMUG00000060757		-0.356930253	0.010503024	0.033719626
ENSMMUG00000051107		-1.416999367	2.24E-05	0.000158161
ENSMMUG00000060184	SEC61 translocon subunit gamma [Source:NCBI gene;Acc:716898]	3.435603128	1.84E-06	1.66E-05
ENSMMUG00000019992	growth factor receptor bound protein 10 [Source:VGNC Symbol;Acc:VGNC:73157]	-1.64564313	0.007203903	0.024542569
ENSMMUG00000019991	dopa decarboxylase [Source:VGNC Symbol;Acc:VGNC:71755]	1.083001976	3.85E-25	4.88E-23
ENSMMUG00000049620		-0.825816955	0.014882484	0.044986689
ENSMMUG00000000505	fidgetin like 1 [Source:VGNC Symbol;Acc:VGNC:72483]	-0.990336895	0.004701203	0.01701728
ENSMMUG00000000504	IKAROS family zinc finger 1 [Source:VGNC Symbol;Acc:VGNC:73585]	-1.162806343	0.003634395	0.013719345
ENSMMUG00000044759		-1.299129306	1.77E-15	8.07E-14
ENSMMUG00000043922	spermatogenesis associated 48 [Source:VGNC Symbol;Acc:VGNC:81762]	-0.861165366	2.44E-06	2.14E-05
ENSMMUG00000016191	ATP binding cassette subfamily A member 13 [Source:VGNC Symbol;Acc:VGNC:69402]	1.534013009	0.014424271	0.043858111
ENSMMUG00000019393	tensin 3 [Source:VGNC Symbol;Acc:VGNC:79324]	-1.977736529	0.015114243	0.045555825
ENSMMUG00000050300		-0.816484283	0.0088175	0.029191529
ENSMMUG00000000962	H2A.Z variant histone 2 [Source:VGNC Symbol;Acc:VGNC:81324]	-0.833510851	0.000746067	0.003508757
ENSMMUG00000002433	calcium/calmodulin dependent protein kinase II beta [Source:VGNC Symbol;Acc:VGNC:70524]	2.112175165	7.55E-08	9.27E-07
ENSMMUG00000002430	YKT6 v-SNARE homolog [Source:VGNC Symbol;Acc:VGNC:81796]	2.35817135	3.94E-09	6.14E-08
ENSMMUG00000002427	glucokinase [Source:VGNC Symbol;Acc:VGNC:72901]	1.547504795	1.39E-16	7.15E-15

ENSMMUG00000002411	drebrin like [Source:NCBI gene;Acc:720621]	1.799234068	2.97E-23	2.90E-21
ENSMMUG00000002426	myosin light chain 7 [Source:VGNC Symbol;Acc:VGNC:75002]	1.586769913	1.25E-15	5.83E-14
ENSMMUG00000002423	DNA polymerase delta 2, accessory subunit [Source:VGNC Symbol;Acc:VGNC:76203]	1.59153334	4.03E-13	1.24E-11
ENSMMUG00000002422	AE binding protein 1 [Source:VGNC Symbol;Acc:VGNC:80328]	1.668949354	2.73E-10	5.29E-09
ENSMMUG00000002421	DNA polymerase mu [Source:VGNC Symbol;Acc:VGNC:81742]	3.104434053	0.010858791	0.034670382
ENSMMUG00000064860	phosphoglycerate mutase 2 [Source:NCBI gene;Acc:720615]	2.785823721	0.001836385	0.007660121
ENSMMUG00000019023	cytochrome c oxidase assembly factor 1 homolog [Source:VGNC Symbol;Acc:VGNC:81290]	0.57610904	0.002559379	0.010148445
ENSMMUG00000017382	biliverdin reductase A [Source:VGNC Symbol;Acc:VGNC:70256]	0.605661513	4.63E-07	4.86E-06
ENSMMUG00000019022	serine/threonine kinase 17a [Source:VGNC Symbol;Acc:VGNC:77913]	0.674399137	4.38E-07	4.62E-06
ENSMMUG00000019015	HECT, C2 and WW domain containing E3 ubiquitin protein ligase 1 [Source:VGNC Symbol;Acc:VGNC:73217]	0.514189788	0.000351714	0.001810503
ENSMMUG00000024428	Y RNA [Source:RFAM;Acc:RF00019]	1.56869783	0.01667086	0.049417678
ENSMMUG00000060273	mitochondrial ribosomal protein L32 [Source:NCBI gene;Acc:701820]	2.114001403	1.10E-13	3.72E-12
ENSMMUG00000022451	proteasome 20S subunit alpha 2 [Source:NCBI gene;Acc:701683]	1.734007916	1.73E-06	1.57E-05
ENSMMUG00000052530		1.124354702	0.012223599	0.038230306
ENSMMUG00000001702	succinyl-CoA:glutarate-CoA transferase [Source:VGNC Symbol;Acc:VGNC:81767]	1.763749965	2.42E-17	1.35E-15
ENSMMUG00000065307		-0.999748946	0.002541144	0.010098582
ENSMMUG00000006980	septin 7 [Source:VGNC Symbol;Acc:VGNC:81759]	2.601972521	0.001155349	0.005113832
ENSMMUG00000001350	HERPUD family member 2 [Source:VGNC Symbol;Acc:VGNC:73216]	2.056316704	5.26E-12	1.38E-10
ENSMMUG00000058423		2.485389745	1.34E-12	3.83E-11
ENSMMUG00000009865	probable C-mannosyltransferase DPY19L2 [Source:NCBI gene;Acc:699789]	-0.506279935	0.0115962	0.036559088
ENSMMUG00000002886	Bardet-Biedl syndrome 9 [Source:NCBI gene;Acc:708412]	1.751676293	1.05E-14	4.15E-13
ENSMMUG00000050858	Metazoan signal recognition particle RNA [Source:RFAM;Acc:RF00017]	-0.948523755	0.002390977	0.009577743
ENSMMUG00000022484	ITPR interacting domain containing 1 [Source:VGNC Symbol;Acc:VGNC:73667]	-4.058894996	1.60E-06	1.47E-05
ENSMMUG00000055809		-3.71139405	5.65E-13	1.70E-11
ENSMMUG00000062454		2.228753222	1.69E-22	1.58E-20
ENSMMUG00000039009		1.481455821	0.000286196	0.001516743
ENSMMUG00000012529	pleckstrin homology domain containing A8 [Source:VGNC Symbol;Acc:VGNC:81741]	0.761320984	0.010449753	0.033600545
ENSMMUG00000032973	mml-mir-550 [Source:miRBase;Acc:MI0007826]	-1.070535673	0.000519411	0.002556504
ENSMMUG00000020184	carboxypeptidase vitellogenic like [Source:VGNC Symbol;Acc:VGNC:71406]	-1.494672118	1.52E-05	0.000111275
ENSMMUG00000039317	cAMP responsive element binding protein 5 [Source:VGNC Symbol;Acc:VGNC:81295]	-2.239487331	3.20E-05	0.000217449
ENSMMUG00000055193		-3.057972642	0.004277979	0.015720168
ENSMMUG00000001562	kelch like family member 7 [Source:VGNC Symbol;Acc:VGNC:74050]	-1.732711108	0.010412838	0.033522908
ENSMMUG00000065311	translocase of outer mitochondrial membrane 7 [Source:NCBI gene;Acc:705701]	-1.611535872	0.010508311	0.033729148
ENSMMUG00000042472	Metazoan signal recognition particle RNA [Source:RFAM;Acc:RF00017]	0.836324942	0.000509441	0.002515537
ENSMMUG00000007821	ATP binding cassette subfamily B member 5 [Source:VGNC Symbol;Acc:VGNC:80321]	-2.536472269	6.10E-25	7.52E-23
ENSMMUG00000006898	integrin subunit beta 8 [Source:VGNC Symbol;Acc:VGNC:73791]	-2.833659112	1.04E-28	2.11E-26
ENSMMUG00000052574		-1.156337821	3.18E-15	1.40E-13
ENSMMUG00000020418	diacylglycerol kinase beta [Source:VGNC Symbol;Acc:VGNC:71782]	-0.923185766	0.000221872	0.001213055
ENSMMUG00000008632	myosin, light chain 6, alkali, smooth muscle and non-muscle-like [Source:NCBI gene;Acc:710137]	-1.446347632	0.000296585	0.001560897

ENSMMUG00000012694	thrombospondin type 1 domain containing 7A [Source:VGNC Symbol;Acc:VGNC:79347]	-1.256174153	0.005404608	0.019214181
ENSMMUG00000041616	G protein subunit gamma 11 [Source:VGNC Symbol;Acc:VGNC:81319]	2.712597128	0.011980104	0.037581953
ENSMMUG00000045513		1.815286368	8.81E-08	1.07E-06
ENSMMUG00000015685	zinc finger protein 804B [Source:VGNC Symbol;Acc:VGNC:81890]	1.397303065	1.31E-13	4.34E-12
ENSMMUG00000022697	STEAP4 metalloreductase [Source:VGNC Symbol;Acc:VGNC:77974]	-1.032587404	7.83E-10	1.40E-08
ENSMMUG00000006766	RUN domain containing 3B [Source:VGNC Symbol;Acc:VGNC:77129]	1.739015241	1.14E-10	2.36E-09
ENSMMUG00000010788	ATP binding cassette subfamily B member 1 [Source:NCBI gene;Acc:574235]	-0.635178083	2.75E-05	0.000189408
ENSMMUG00000022513	glutamate metabotropic receptor 3 [Source:VGNC Symbol;Acc:VGNC:73287]	1.036700006	0.001280986	0.005598823
ENSMMUG00000001979	semaphorin 3E [Source:VGNC Symbol;Acc:VGNC:77163]	1.555123845	0.002355747	0.009470545
ENSMMUG00000018652	hepatocyte growth factor [Source:VGNC Symbol;Acc:VGNC:73373]	-0.395090742	0.003513166	0.013313199
ENSMMUG00000053516		1.709069846	5.12E-09	7.88E-08
ENSMMUG00000001801	membrane associated guanylate kinase, WW and PDZ domain containing 2 [Source:VGNC Symbol;Acc:VGNC:74312]	0.80899395	1.39E-05	0.000103322
ENSMMUG00000049939		-1.006330339	2.05E-17	1.15E-15
ENSMMUG00000009006	round spermatid basic protein 1 like [Source:VGNC Symbol;Acc:VGNC:77097]	-0.730913791	4.70E-07	4.93E-06
ENSMMUG00000012427	F-box and leucine rich repeat protein 13 [Source:VGNC Symbol;Acc:VGNC:81312]	-0.578746087	0.000161325	0.000913235
ENSMMUG00000012066	origin recognition complex subunit 5 [Source:VGNC Symbol;Acc:VGNC:75717]	-0.439112551	0.016730509	0.049554077
ENSMMUG00000022727	lysine methyltransferase 2E [Source:VGNC Symbol;Acc:VGNC:74073]	-2.849586257	0.000267485	0.001429044
ENSMMUG00000022732	SRSF protein kinase 2 [Source:VGNC Symbol;Acc:VGNC:81764]	-0.628723398	0.000415889	0.00210361
ENSMMUG00000008189	cadherin related family member 3 [Source:HGNC Symbol;Acc:HGNC:26308]	1.042982798	2.42E-10	4.72E-09
ENSMMUG00000003572	nicotinamide phosphoribosyltransferase [Source:VGNC Symbol;Acc:VGNC:75056]	2.154948598	1.19E-18	7.82E-17
ENSMMUG00000011385	phosphatidylinositol-4,5-bisphosphate 3-kinase catalytic subunit gamma [Source:VGNC Symbol;Acc:VGNC:75998]	-0.36553151	0.010416478	0.033523211
ENSMMUG00000030218	B cell receptor associated protein 29 [Source:VGNC Symbol;Acc:VGNC:70119]	0.285954662	0.005630303	0.019919133
ENSMMUG00000019125	solute carrier family 26 member 4 [Source:VGNC Symbol;Acc:VGNC:77595]	0.344005789	0.00266816	0.010485819
ENSMMUG00000015535	inner mitochondrial membrane peptidase subunit 2 [Source:VGNC Symbol;Acc:VGNC:73596]	0.586655901	0.008109484	0.027138021
ENSMMUG00000062092		4.069166555	8.72E-06	6.74E-05
ENSMMUG00000058817		3.61811111	7.95E-32	1.99E-29
ENSMMUG00000002894	dedicator of cytokinesis 4 [Source:VGNC Symbol;Acc:VGNC:71866]	-0.857912195	0.001138326	0.005048154
ENSMMUG00000011269	CF transmembrane conductance regulator [Source:VGNC Symbol;Acc:VGNC:71020]	-0.787396892	0.000133457	0.000773548
ENSMMUG00000023310	tetraspanin 12 [Source:VGNC Symbol;Acc:VGNC:79328]	2.735808696	0.006273282	0.021764845
ENSMMUG00000002434	inhibitor of growth family member 3 [Source:VGNC Symbol;Acc:VGNC:73595]	-1.269011964	6.38E-05	0.000401022
ENSMMUG00000015240	glutamate metabotropic receptor 8 [Source:VGNC Symbol;Acc:VGNC:73292]	-0.747382027	0.001328318	0.005779633
ENSMMUG00000002428	zinc finger protein 800 [Source:VGNC Symbol;Acc:VGNC:79658]	0.652248933	0.000198781	0.001098737
ENSMMUG00000015427	fascin actin-bundling protein 3 [Source:VGNC Symbol;Acc:VGNC:72821]	-0.712364491	0.007707769	0.026021326
ENSMMUG00000015432	staphylococcal nuclease and tudor domain containing 1 [Source:VGNC Symbol;Acc:VGNC:77798]	-0.858080665	2.19E-05	0.000155055
ENSMMUG00000007250	RNA binding motif protein 28 [Source:VGNC Symbol;Acc:VGNC:76519]	-0.979427324	1.72E-05	0.000123751
ENSMMUG00000037001	U7 small nuclear RNA [Source:RFAM;Acc:RF00066]	-2.093413598	0.007623794	0.025797742
ENSMMUG00000037473	proline rich transmembrane protein 4 [Source:VGNC Symbol;Acc:VGNC:81746]	0.428690901	0.004980658	0.01791287

ENSMMUG00000012110	smoothened, frizzled class receptor [Source:VGNC Symbol;Acc:VGNC:77658]	-1.694079149	2.53E-11	5.82E-10
ENSMMUG00000017542	ubiquitin conjugating enzyme E2 H [Source:VGNC Symbol;Acc:VGNC:78696]	-0.526489972	0.009138584	0.030131003
ENSMMUG00000006155	zinc finger C3HC-type containing 1 [Source:VGNC Symbol;Acc:VGNC:79827]	-0.991706949	0.006447484	0.022268254
ENSMMUG00000010060	serine rich single-pass membrane protein 1 [Source:VGNC Symbol;Acc:VGNC:78014]	-0.952485199	0.002352139	0.009458657
ENSMMUG00000009494	carboxypeptidase A5 [Source:VGNC Symbol;Acc:VGNC:71410]	0.818317239	0.000652465	0.003124137
ENSMMUG00000009495	carboxypeptidase A1 [Source:VGNC Symbol;Acc:VGNC:71455]	1.126276432	5.05E-05	0.000327086
ENSMMUG00000009496	centrosomal protein 41 [Source:VGNC Symbol;Acc:VGNC:70937]	0.819048496	0.001351614	0.005866949
ENSMMUG00000009499	mesoderm specific transcript [Source:VGNC Symbol;Acc:VGNC:74707]	3.692427911	1.70E-25	2.24E-23
ENSMMUG00000004801	COPI coat complex subunit gamma 2 [Source:VGNC Symbol;Acc:VGNC:71315]	3.627287638	4.24E-27	7.01E-25
ENSMMUG00000001671	coiled-coil-helix-coiled-coil-helix domain containing 3 [Source:VGNC Symbol;Acc:VGNC:80860]	-1.106544362	0.002140448	0.008760047
ENSMMUG00000000916	solute carrier family 35 member B4 [Source:VGNC Symbol;Acc:VGNC:77528]	-1.447646936	0.010447172	0.033600545
ENSMMUG00000000917	aldo-keto reductase family 1 member B [Source:NCBI gene;Acc:705957]	1.342093596	0.011927402	0.037457041
ENSMMUG00000004598	bisphosphoglycerate mutase [Source:VGNC Symbol;Acc:VGNC:70275]	-2.159041031	0.01150098	0.036345598
ENSMMUG00000037485		-3.647042753	7.78E-05	0.000479301
ENSMMUG00000054821		-2.432122408	0.002132362	0.008734334
ENSMMUG00000006530	ATP/GTP binding protein like 3 [Source:VGNC Symbol;Acc:VGNC:69723]	1.227917196	6.51E-17	3.45E-15
ENSMMUG00000050339	Metazoan signal recognition particle RNA [Source:RFAM;Acc:RF00017]	2.492103667	1.37E-13	4.54E-12
ENSMMUG00000062419		2.361358229	0.000137315	0.000792436
ENSMMUG00000002318	stimulated by retinoic acid 8 [Source:VGNC Symbol;Acc:VGNC:78250]	-0.978869697	0.003865153	0.014436726
ENSMMUG00000016384	nucleoporin 205 [Source:VGNC Symbol;Acc:VGNC:75432]	0.802153338	0.000193082	0.001071722
ENSMMUG00000064014		2.723491174	0.013566363	0.041711975
ENSMMUG00000002995	tripartite motif containing 24 [Source:VGNC Symbol;Acc:VGNC:78562]	0.658043831	0.00013369	0.000774591
ENSMMUG00000050262		1.363530667	0.010592451	0.033954237
ENSMMUG00000021676	SVOP like [Source:VGNC Symbol;Acc:VGNC:78224]	0.712403516	0.000236046	0.001277513
ENSMMUG00000006547	ATPase H+ transporting V0 subunit a4 [Source:VGNC Symbol;Acc:VGNC:70189]	-2.508191205	0.001198098	0.005285811
ENSMMUG00000006548	KIAA1549 [Source:VGNC Symbol;Acc:VGNC:73885]	-3.15985624	2.36E-08	3.19E-07
ENSMMUG00000006549	zinc finger CCCH-type containing, antiviral 1 like [Source:VGNC Symbol;Acc:VGNC:79356]	0.612305345	0.000459085	0.002298457
ENSMMUG00000006550	zinc finger CCCH-type containing, antiviral 1 [Source:VGNC Symbol;Acc:VGNC:79408]	0.456377604	0.003830468	0.014325576
ENSMMUG00000006770	putative RNA-binding protein Luc7-like 2 [Source:NCBI gene;Acc:711104]	-0.649654959	0.008174249	0.027317024
ENSMMUG00000004222	thromboxane A synthase 1 [Source:VGNC Symbol;Acc:VGNC:78278]	1.563580974	1.01E-11	2.50E-10
ENSMMUG00000052228	Metazoan signal recognition particle RNA [Source:RFAM;Acc:RF00017]	1.866450835	0.00145106	0.006235312
ENSMMUG00000004224	poly(ADP-ribose) polymerase family member 12 [Source:VGNC Symbol;Acc:VGNC:81734]	0.968320242	2.45E-12	6.70E-11
ENSMMUG00000056175		0.696878373	2.49E-05	0.000173698
ENSMMUG00000008126	lysine demethylase 7A [Source:VGNC Symbol;Acc:VGNC:73886]	0.798304116	5.33E-08	6.76E-07
ENSMMUG00000042299	DENN domain containing 2A [Source:VGNC Symbol;Acc:VGNC:81302]	-2.483014641	0.004476618	0.016314011
ENSMMUG00000015282	aarF domain containing kinase 2 [Source:VGNC Symbol;Acc:VGNC:69473]	-0.491048554	0.000132656	0.000769828
ENSMMUG00000060893		-1.779762169	1.34E-05	0.00010002
ENSMMUG00000064529		-1.418311071	8.79E-11	1.86E-09

ENSMMUG00000021017	acylglycerol kinase [Source:VGNC Symbol;Acc:VGNC:69669]	0.950692159	1.04E-06	1.00E-05
ENSMMUG00000022442	single stranded DNA binding protein 1 [Source:VGNC Symbol;Acc:VGNC:77886]	-0.626409425	5.86E-05	0.000372483
ENSMMUG00000055710	taste 2 receptor member 38 [Source:NCBI gene;Acc:695549]	2.006416512	1.74E-06	1.57E-05
ENSMMUG00000016273	maltase-glucoamylase [Source:VGNC Symbol;Acc:VGNC:74713]	2.049662282	4.67E-18	2.84E-16
ENSMMUG00000019937	maltase-glucoamylase 2 (putative) [Source:VGNC Symbol;Acc:VGNC:81720]	2.126130268	0.000634095	0.00304219
ENSMMUG00000049575	T cell receptor beta variable 7-2 [Source:HGNC Symbol;Acc:HGNC:12236]	-0.978395991	0.001076176	0.004813651
ENSMMUG00000051385		-1.114005984	0.000623744	0.00299946
ENSMMUG00000050984	T cell receptor beta variable 5-4 [Source:HGNC Symbol;Acc:HGNC:12221]	1.310683818	0.016643906	0.049347839
ENSMMUG00000048246		-2.679858928	0.005644927	0.019952199
ENSMMUG00000057494	T cell receptor beta variable 7-9 [Source:HGNC Symbol;Acc:HGNC:12243]	-1.870071109	1.21E-06	1.15E-05
ENSMMUG00000050269	T cell receptor beta variable 14 [Source:HGNC Symbol;Acc:HGNC:12189]	-2.080040063	3.37E-11	7.63E-10
ENSMMUG00000016286	protease, serine, 1 (trypsin 1) [Source:NCBI gene;Acc:698729]	-1.414836121	8.54E-08	1.03E-06
ENSMMUG00000051032	serine protease 2 [Source:NCBI gene;Acc:698352]	-2.382828545	1.27E-27	2.25E-25
ENSMMUG00000052682		-2.37345361	6.52E-09	9.87E-08
ENSMMUG00000059019		-2.613450187	1.14E-13	3.84E-12
ENSMMUG00000058459		-2.290800131	0.001401866	0.006054727
ENSMMUG00000055756		-3.620313768	0.004108774	0.015167406
ENSMMUG00000043809	caspase 2 [Source:VGNC Symbol;Acc:VGNC:80804]	-0.943445855	4.70E-07	4.93E-06
ENSMMUG00000045428	Metazoan signal recognition particle RNA [Source:RFAM;Acc:RF00017]	-1.758703216	0.012501438	0.038965138
ENSMMUG00000012635	zyxin [Source:VGNC Symbol;Acc:VGNC:78917]	1.784071368	8.65E-21	7.10E-19
ENSMMUG00000055322	EPH receptor A1 [Source:NCBI gene;Acc:706295]	1.473447153	8.55E-21	7.06E-19
ENSMMUG00000058370	taste receptor type 2 member 62 [Source:NCBI gene;Acc:704388]	4.019660077	9.87E-07	9.53E-06
ENSMMUG00000042798		-1.136083909	2.38E-08	3.21E-07
ENSMMUG00000063823	U6 spliceosomal RNA [Source:RFAM;Acc:RF00026]	-2.725992714	0.000249235	0.001341403
ENSMMUG00000046165	chromosome 3 C7orf33 homolog [Source:NCBI gene;Acc:710503]	-0.918606806	0.007511851	0.02547825
ENSMMUG00000017541	cullin 1 [Source:VGNC Symbol;Acc:VGNC:71565]	-3.003887888	0.015552366	0.046644264
ENSMMUG00000057973		-1.378850937	0.000795843	0.003710447
ENSMMUG00000049864		-1.765261317	0.013067704	0.040392349
ENSMMUG00000018549	zinc finger protein 786 [Source:VGNC Symbol;Acc:VGNC:79338]	2.600572245	2.04E-07	2.32E-06
ENSMMUG00000002497	zinc finger protein 777 [Source:HGNC Symbol;Acc:HGNC:22213]	-1.09570947	3.15E-06	2.70E-05
ENSMMUG00000051020	zinc finger protein 467 [Source:NCBI gene;Acc:712106]	-1.116699708	5.09E-05	0.000329873
ENSMMUG00000048892	SCO-spondin [Source:VGNC Symbol;Acc:VGNC:81765]	-0.890939567	0.000223073	0.001217727
ENSMMUG00000022633	replication initiator 1 [Source:VGNC Symbol;Acc:VGNC:76758]	1.320293303	0.000145591	0.000835881
ENSMMUG00000062696		-0.95632336	0.001918639	0.007962107

ENSMMUG00000022636	GTPase, IMAP family member 8 [Source:VGNC Symbol;Acc:VGNC:72981]	-1.221996329	7.44E-05	0.000460026
ENSMMUG00000011386	GTPase, IMAP family member 4 [Source:VGNC Symbol;Acc:VGNC:72982]	-1.907709772	3.00E-24	3.31E-22
ENSMMUG00000052830		-1.704892727	1.61E-08	2.24E-07
ENSMMUG00000047276	GTPase, IMAP family member 6 [Source:VGNC Symbol;Acc:VGNC:81315]	-1.083729551	5.02E-10	9.36E-09
ENSMMUG00000049295	GTPase, IMAP family member 5 [Source:NCBI gene;Acc:713835]	-0.667090224	0.002069867	0.008514335
ENSMMUG00000020436	potassium voltage-gated channel subfamily H member 2 [Source:VGNC Symbol;Acc:VGNC:73980]	-0.746005909	1.82E-07	2.08E-06
ENSMMUG00000006682	chondroitin polymerizing factor 2 [Source:VGNC Symbol;Acc:VGNC:71131]	-1.795620914	4.95E-05	0.000321577
ENSMMUG00000038898	Metazoan signal recognition particle RNA [Source:RFAM;Acc:RF00017]	0.525156572	0.008633759	0.028678539
ENSMMUG00000010943	protein kinase AMP-activated non-catalytic subunit gamma 2 [Source:VGNC Symbol;Acc:VGNC:81745]	1.009285899	8.01E-05	0.000491086
ENSMMUG00000012238	polypeptide N-acetylgalactosaminyltransferase 11 [Source:VGNC Symbol;Acc:VGNC:72749]	-0.806796185	0.002360777	0.009482902
ENSMMUG00000007212	lysine methyltransferase 2C [Source:VGNC Symbol;Acc:VGNC:74043]	0.414802871	0.0089183	0.029478245
ENSMMUG00000027964	5S ribosomal RNA [Source:RFAM;Acc:RF00001]	1.500454297	0.010456543	0.033607511
ENSMMUG00000002878	X-ray repair cross complementing 2 [Source:VGNC Symbol;Acc:VGNC:78813]	0.717023032	0.001972494	0.008164621
ENSMMUG00000007770	canopy FGF signaling regulator 1 [Source:VGNC Symbol;Acc:VGNC:71363]	-1.770460123	0.013903395	0.042556848
ENSMMUG00000008733	ubiquitin protein ligase E3C [Source:VGNC Symbol;Acc:VGNC:79049]	-0.78745791	3.02E-05	0.000206233
ENSMMUG00000009611	vasoactive intestinal peptide receptor 2 [Source:VGNC Symbol;Acc:VGNC:78772]	-1.16678054	2.72E-05	0.000187528
ENSMMUG00000052713	leucine rich repeat containing 14B [Source:NCBI gene;Acc:723071]	0.456184221	0.013759625	0.042207989
ENSMMUG00000003791	centrosomal protein 72 [Source:VGNC Symbol;Acc:VGNC:71099]	-1.625994971	0.004456801	0.016258096
ENSMMUG00000052291		-1.660367053	0.000469768	0.002342717
ENSMMUG00000061096	tubulin polymerization promoting protein [Source:NCBI gene;Acc:708834]	-1.699256469	0.008741709	0.029013292
ENSMMUG00000014738	bromodomain containing 9 [Source:VGNC Symbol;Acc:VGNC:70386]	3.899518932	0.001207993	0.005323006
ENSMMUG00000019441	solute carrier family 12 member 7 [Source:VGNC Symbol;Acc:VGNC:77390]	1.030112786	4.86E-05	0.00031684
ENSMMUG00000010940	telomerase reverse transcriptase [Source:VGNC Symbol;Acc:VGNC:78313]	0.924462732	1.04E-12	3.02E-11
ENSMMUG00000058051		-0.636984985	0.009297539	0.03054351
ENSMMUG00000061176		-0.408639086	0.000230734	0.001253267
ENSMMUG00000002561	NOP2/Sun RNA methyltransferase 2 [Source:VGNC Symbol;Acc:VGNC:75466]	-2.036879537	0.004074706	0.01506945
ENSMMUG00000018745	chromosome 6 C5orf49 homolog [Source:VGNC Symbol;Acc:VGNC:70646]	-1.171455213	0.004329397	0.015887207
ENSMMUG00000018746	FAST kinase domains 3 [Source:VGNC Symbol;Acc:VGNC:72588]	-1.2939426	9.96E-12	2.48E-10
ENSMMUG00000018747	5-methyltetrahydrofolate-homocysteine methyltransferase reductase [Source:VGNC Symbol;Acc:VGNC:75079]	-1.363451039	1.04E-09	1.80E-08
ENSMMUG00000061922		0.327952633	0.00092861	0.004253057
ENSMMUG00000065128	Metazoan signal recognition particle RNA [Source:RFAM;Acc:RF00017]	-0.841403693	4.21E-05	0.000279431
ENSMMUG00000055028		-1.704561074	0.007784399	0.026208764
ENSMMUG00000009574	solute carrier family 45 member 2 [Source:VGNC Symbol;Acc:VGNC:82240]	-0.669578037	0.011575547	0.036501894
ENSMMUG00000018739	sperm flagellar 2 [Source:VGNC Symbol;Acc:VGNC:82243]	-4.324503009	1.21E-06	1.15E-05

ENSMMUG00000006202	solute carrier family 1 member 3 [Source:VGNC Symbol;Acc:VGNC:77411]	-0.306567235	0.004448719	0.016240832
ENSMMUG00000052836		0.467133556	0.002813712	0.010998369
ENSMMUG00000018963	glial cell derived neurotrophic factor [Source:VGNC Symbol;Acc:VGNC:72912]	-1.435202015	0.004984981	0.017919355
ENSMMUG00000017163	EGF like, fibronectin type III and laminin G domains [Source:VGNC Symbol;Acc:VGNC:72122]	-0.508946995	0.005804477	0.020401277
ENSMMUG00000061318		3.810805387	0.000612497	0.002954171
ENSMMUG00000004774	maestro heat like repeat family member 2B [Source:VGNC Symbol;Acc:VGNC:74837]	2.514195307	1.97E-08	2.69E-07
ENSMMUG00000021034	complement C6 [Source:VGNC Symbol;Acc:VGNC:70477]	-0.898759311	0.002341889	0.009422653
ENSMMUG00000011878	poly(A) binding protein interacting protein 1 [Source:VGNC Symbol;Acc:VGNC:84476]	3.562905712	1.31E-24	1.51E-22
ENSMMUG00000055022	Metazoan signal recognition particle RNA [Source:RFAM;Acc:RF00017]	4.167093575	2.36E-06	2.08E-05
ENSMMUG00000011734	nicotinamide nucleotide transhydrogenase [Source:VGNC Symbol;Acc:VGNC:75363]	-1.202358112	0.007730402	0.026061422
ENSMMUG00000047162	fibroblast growth factor 10 [Source:VGNC Symbol;Acc:VGNC:84378]	-0.738732544	0.002856504	0.011141669
ENSMMUG00000016149	embigin [Source:VGNC Symbol;Acc:VGNC:72144]	-4.108568188	8.25E-05	0.000504129
ENSMMUG00000001753	integrin subunit alpha 1 [Source:VGNC Symbol;Acc:VGNC:73772]	1.912506005	9.15E-25	1.08E-22
ENSMMUG00000039101	pelota mRNA surveillance and ribosome rescue factor [Source:VGNC Symbol;Acc:VGNC:84478]	2.098762126	1.72E-16	8.79E-15
ENSMMUG00000010379	Mtr4 exosome RNA helicase [Source:VGNC Symbol;Acc:VGNC:74989]	-1.553741375	2.21E-11	5.17E-10
ENSMMUG00000030099	phospholipid phosphatase 1 [Source:VGNC Symbol;Acc:VGNC:76226]	-2.639574851	9.26E-12	2.32E-10
ENSMMUG00000061973		-2.654855604	1.35E-27	2.37E-25
ENSMMUG00000010466	solute carrier family 38 member 9 [Source:VGNC Symbol;Acc:VGNC:77574]	2.446959474	1.18E-16	6.10E-15
ENSMMUG00000049885		-0.547964427	0.008410307	0.027990071
ENSMMUG00000051661		-1.150172816	0.001091654	0.004866413
ENSMMUG00000005181	RAB3C, member RAS oncogene family [Source:VGNC Symbol;Acc:VGNC:81541]	-1.582966434	1.94E-06	1.74E-05
ENSMMUG00000021812	ERCC excision repair 8, CSA ubiquitin ligase complex subunit [Source:VGNC Symbol;Acc:VGNC:72425]	0.848856836	1.85E-14	7.24E-13
ENSMMUG00000060505		-1.169691931	0.000853649	0.003955786
ENSMMUG00000021131	regulator of G protein signaling 7 binding protein [Source:VGNC Symbol;Acc:VGNC:76808]	-1.100975931	0.002575493	0.010196062
ENSMMUG00000015041	CWC27 spliceosome associated cyclophilin [Source:VGNC Symbol;Acc:VGNC:71396]	-2.497624917	4.26E-06	3.54E-05
ENSMMUG00000051169		-2.189053856	8.35E-05	0.000509461
ENSMMUG00000018100	ADAM metallopeptidase with thrombospondin type 1 motif 6 [Source:VGNC Symbol;Acc:VGNC:69500]	0.53351718	0.010017566	0.032491154
ENSMMUG00000016522	neurolysin [Source:VGNC Symbol;Acc:VGNC:75183]	-0.621555683	8.94E-07	8.68E-06
ENSMMUG00000049935		1.173807164	1.38E-05	0.000102208
ENSMMUG00000046550	coiled-coil domain containing 125 [Source:NCBI gene;Acc:704391]	1.036435786	2.70E-10	5.23E-09
ENSMMUG00000009158	MARVEL domain containing 2 [Source:VGNC Symbol;Acc:VGNC:74592]	-0.437708603	0.003933819	0.014629299
ENSMMUG00000040485	Metazoan signal recognition particle RNA [Source:RFAM;Acc:RF00017]	-0.653233561	0.000250702	0.0013478
ENSMMUG00000006217	general transcription factor IIH subunit 2 [Source:NCBI gene;Acc:700737]	-0.864454464	0.00391737	0.014579316
ENSMMUG00000054237	Y RNA [Source:RFAM;Acc:RF00019]	1.691687752	0.001155577	0.005113832

ENSMMUG00000059671		1.299412961	1.57E-08	2.20E-07
ENSMMUG00000063792		0.652462554	9.10E-06	7.00E-05
ENSMMUG00000064344	Metazoan signal recognition particle RNA [Source:RFAM;Acc:RF00017]	-3.154078756	0.003337103	0.012756107
ENSMMUG00000011061	ankyrin repeat domain 31 [Source:VGNC Symbol;Acc:VGNC:84277]	0.771180063	2.56E-07	2.85E-06
ENSMMUG00000006139	3-hydroxy-3-methylglutaryl-CoA reductase [Source:VGNC Symbol;Acc:VGNC:73484]	0.690274758	1.06E-08	1.54E-07
ENSMMUG00000006141	ceramide transporter 1 [Source:VGNC Symbol;Acc:VGNC:71298]	0.653550896	6.94E-06	5.51E-05
ENSMMUG00000019887	synaptic vesicle glycoprotein 2C [Source:VGNC Symbol;Acc:VGNC:78252]	-0.689140318	0.011652721	0.036713278
ENSMMUG00000022982	IQ motif containing GTPase activating protein 2 [Source:VGNC Symbol;Acc:VGNC:73756]	-1.484648186	0.001127417	0.005012006
ENSMMUG00000021354	corticotropin releasing hormone binding protein [Source:VGNC Symbol;Acc:VGNC:71491]	-1.613066629	0.004486247	0.016345006
ENSMMUG00000021357	zinc finger BED-type containing 3 [Source:VGNC Symbol;Acc:VGNC:79271]	2.145755364	0.000107749	0.000637246
ENSMMUG00000006792	arylsulfatase B [Source:VGNC Symbol;Acc:VGNC:70023]	1.228880406	0.004150047	0.015296459
ENSMMUG00000036820	U7 small nuclear RNA [Source:RFAM;Acc:RF00066]	0.977869758	0.000119277	0.000699168
ENSMMUG00000051429		-2.034961558	0.000333281	0.001726624
ENSMMUG00000018283	dihydrofolate reductase [Source:NCBI gene;Acc:711268]	-1.511043913	0.003166484	0.012177559
ENSMMUG00000018284	mutS homolog 3 [Source:VGNC Symbol;Acc:VGNC:74932]	-0.883945442	1.37E-06	1.28E-05
ENSMMUG00000039944	Ras protein specific guanine nucleotide releasing factor 2 [Source:VGNC Symbol;Acc:VGNC:84486]	-1.759849587	1.28E-08	1.84E-07
ENSMMUG00000006932	creatine kinase, mitochondrial 2 [Source:VGNC Symbol;Acc:VGNC:71231]	-1.155827364	0.00258971	0.010235621
ENSMMUG00000017583	single stranded DNA binding protein 2 [Source:NCBI gene;Acc:711752]	-1.057053536	3.55E-08	4.64E-07
ENSMMUG00000046560	transmembrane protein 167A [Source:VGNC Symbol;Acc:VGNC:84513]	0.891691581	0.005770539	0.0203213
ENSMMUG00000064265	X-ray repair cross complementing 4 [Source:NCBI gene;Acc:712291]	0.829814246	0.000374504	0.001910904
ENSMMUG00000011513	versican [Source:VGNC Symbol;Acc:VGNC:79445]	2.080918382	4.75E-20	3.62E-18
ENSMMUG00000061282	hyaluronan and proteoglycan link protein 1 [Source:NCBI gene;Acc:712501]	3.809768072	0.00413241	0.015243047
ENSMMUG00000009319	transmembrane protein 161B [Source:VGNC Symbol;Acc:VGNC:84508]	-1.373262113	4.37E-05	0.000288739
ENSMMUG00000019836	myocyte enhancer factor 2C [Source:VGNC Symbol;Acc:VGNC:84438]	-1.157606862	9.05E-05	0.000546734
ENSMMUG00000055392		-1.211415308	0.000147593	0.000845375
ENSMMUG00000056790		-2.958675732	0.006027871	0.02104904
ENSMMUG00000018974	LysM domain containing 3 [Source:VGNC Symbol;Acc:VGNC:74373]	-1.716648979	3.13E-34	1.06E-31
ENSMMUG00000055271		-1.710969897	1.91E-07	2.18E-06
ENSMMUG00000018976	adhesion G protein-coupled receptor V1 [Source:VGNC Symbol;Acc:VGNC:69625]	-1.259469061	0.000305778	0.001602415
ENSMMUG00000001397	multiple C2 and transmembrane domain containing 1 [Source:VGNC Symbol;Acc:VGNC:74589]	1.557752037	6.97E-13	2.07E-11
ENSMMUG00000010632	family with sequence similarity 81 member B [Source:VGNC Symbol;Acc:VGNC:72365]	2.650154998	0.006773117	0.023227606
ENSMMUG00000001209	arylsulfatase family member K [Source:VGNC Symbol;Acc:VGNC:84280]	-0.872430006	0.002277016	0.009220775
ENSMMUG00000001211	G protein-coupled receptor 150 [Source:VGNC Symbol;Acc:VGNC:72988]	-1.061550785	0.011021115	0.035103846
ENSMMUG00000001212	Rieske Fe-S domain containing [Source:VGNC Symbol;Acc:VGNC:84487]	-1.046702893	8.26E-20	6.03E-18

ENSMMUG00000001213	spermatogenesis associated 9 [Source:VGNC Symbol;Acc:VGNC:77976]	-1.250356389	1.60E-09	2.71E-08
ENSMMUG00000011043	endoplasmic reticulum aminopeptidase 2 [Source:VGNC Symbol;Acc:VGNC:72141]	0.914273205	5.27E-21	4.40E-19
ENSMMUG00000065221		1.069285359	5.86E-12	1.52E-10
ENSMMUG00000000307	RIO kinase 2 [Source:VGNC Symbol;Acc:VGNC:76774]	0.350225887	2.33E-05	0.00016349
ENSMMUG00000054789	family with sequence similarity 174 member A [Source:NCBI gene;Acc:702300]	0.740296991	1.30E-06	1.22E-05
ENSMMUG00000007773	solute carrier organic anion transporter family member 4C1 [Source:VGNC Symbol;Acc:VGNC:77764]	2.670159951	1.68E-06	1.53E-05
ENSMMUG00000029885	solute carrier organic anion transporter family member 6A1 [Source:VGNC Symbol;Acc:VGNC:77765]	2.578072119	0.005549032	0.01966511
ENSMMUG00000007787	diphosphoinositol pentakisphosphate kinase 2 [Source:VGNC Symbol;Acc:VGNC:76128]	-0.546085561	0.00370543	0.013940434
ENSMMUG00000013183	F-box and leucine rich repeat protein 17 [Source:VGNC Symbol;Acc:VGNC:82142]	-0.62006569	6.70E-05	0.000418157
ENSMMUG00000003316	mannosidase alpha class 2A member 1 [Source:VGNC Symbol;Acc:VGNC:81449]	-0.676638214	1.35E-08	1.92E-07
ENSMMUG00000038051	uncharacterized LOC106998885 [Source:NCBI gene;Acc:106998885]	-1.061711049	2.86E-07	3.15E-06
ENSMMUG00000011974	calcium/calmodulin dependent protein kinase IV [Source:VGNC Symbol;Acc:VGNC:70526]	-2.104088315	1.40E-13	4.62E-12
ENSMMUG00000063177	StAR related lipid transfer domain containing 4 [Source:NCBI gene;Acc:706654]	-2.184922297	2.57E-39	1.49E-36
ENSMMUG00000002768	neuronal regeneration related protein [Source:VGNC Symbol;Acc:VGNC:84454]	-2.5712845	0.010041224	0.032536177
ENSMMUG00000012192	erythrocyte membrane protein band 4.1 like 4A [Source:VGNC Symbol;Acc:VGNC:72039]	-1.395145911	4.26E-11	9.45E-10
ENSMMUG00000042951	signal recognition particle 19 [Source:NCBI gene;Acc:693829]	0.942939848	6.20E-06	4.97E-05
ENSMMUG00000020488	receptor accessory protein 5 [Source:VGNC Symbol;Acc:VGNC:76864]	0.777519121	1.72E-07	1.99E-06
ENSMMUG00000005018	protein geranylgeranyltransferase type I subunit beta [Source:VGNC Symbol;Acc:VGNC:75965]	2.43301244	0.005046073	0.018107816
ENSMMUG00000015114	toll like receptor adaptor molecule 2 [Source:NCBI gene;Acc:100426353]	0.563919278	1.49E-09	2.53E-08
ENSMMUG00000060812	autophagy related 12 [Source:NCBI gene;Acc:696765]	1.193941908	5.96E-12	1.54E-10
ENSMMUG00000015288	adaptor related protein complex 3 subunit sigma 1 [Source:VGNC Symbol;Acc:VGNC:69867]	0.940740358	7.11E-06	5.63E-05
ENSMMUG00000022192		-0.908702737	0.001186696	0.005241874
ENSMMUG00000037127	U6 spliceosomal RNA [Source:RFAM;Acc:RF00026]	-1.281259231	0.007573127	0.025632258
ENSMMUG00000018640	Dmx like 1 [Source:VGNC Symbol;Acc:VGNC:71935]	-0.649770079	0.014394689	0.043804813
ENSMMUG00000063474		-0.577061195	1.57E-07	1.84E-06
ENSMMUG00000017685	hydroxysteroid 17-beta dehydrogenase 4 [Source:VGNC Symbol;Acc:VGNC:73530]	-0.371875249	7.43E-06	5.85E-05
ENSMMUG00000059145		-0.966201166	0.005825658	0.020460017
ENSMMUG00000018764		-1.211295582	0.000665551	0.003179464
ENSMMUG00000063354		-1.415990164	0.000922416	0.004228687
ENSMMUG00000053794		-0.598245225	0.010028889	0.032513371
ENSMMUG00000021006	zinc finger protein 474 [Source:VGNC Symbol;Acc:VGNC:81617]	-1.241329627	0.000547802	0.002677674
ENSMMUG00000046428		-0.894561138	0.00217535	0.008880387
ENSMMUG00000023598	sorting nexin 2 [Source:VGNC Symbol;Acc:VGNC:77682]	2.051992698	0.000288676	0.001525992
ENSMMUG00000006646	casein kinase 1 gamma 3 [Source:VGNC Symbol;Acc:VGNC:81384]	-0.49179035	0.014069411	0.042985924
ENSMMUG00000016683	phosphorylated adaptor for RNA export [Source:NCBI gene;Acc:702980]	-1.513677645	0.000689294	0.003278881
ENSMMUG00000037806	chromosome 6 C5orf63 homolog [Source:NCBI gene;Acc:100423212]	-1.88325838	0.007981657	0.026747198

ENSMMUG00000011214	solute carrier family 12 member 2 [Source:VGNC Symbol;Acc:VGNC:77386]	-1.158538841	1.45E-05	0.000106655
ENSMMUG00000010682	fibrillin 2 [Source:VGNC Symbol;Acc:VGNC:72624]	-1.27220853	3.73E-13	1.15E-11
ENSMMUG00000058082		0.43343424	0.000297628	0.001563821
ENSMMUG00000016909	meiotic kinetochore factor [Source:NCBI gene;Acc:709179]	0.790547537	2.30E-06	2.04E-05
ENSMMUG00000003131	interleukin 13 [Source:VGNC Symbol;Acc:VGNC:84393]	1.681339245	1.27E-13	4.23E-12
ENSMMUG00000003133	interleukin 4 [Source:VGNC Symbol;Acc:VGNC:73729]	1.924434556	4.15E-08	5.37E-07
ENSMMUG00000010313	kinesin family member 3A [Source:VGNC Symbol;Acc:VGNC:74017]	1.712351938	2.36E-22	2.20E-20
ENSMMUG00000010308	cyclin I family member 2 [Source:VGNC Symbol;Acc:VGNC:70923]	1.522883489	6.65E-10	1.21E-08
ENSMMUG00000010309	septin 8 [Source:VGNC Symbol;Acc:VGNC:77248]	1.605314216	3.28E-06	2.80E-05
ENSMMUG00000039781	sosondowah ankyrin repeat domain family member A [Source:VGNC Symbol;Acc:VGNC:84498]	-0.933118756	0.002561522	0.010151813
ENSMMUG00000010312	shroom family member 1 [Source:VGNC Symbol;Acc:VGNC:77299]	-0.772662175	6.48E-05	0.00040619
ENSMMUG00000060221	growth differentiation factor 9 [Source:NCBI gene;Acc:712792]	-0.902456954	8.21E-07	8.05E-06
ENSMMUG00000015640	AF4/FMR2 family member 4 [Source:VGNC Symbol;Acc:VGNC:69629]	-0.835746748	4.82E-05	0.000314334
ENSMMUG00000026029	Y RNA [Source:RFAM;Acc:RF00019]	-1.298084951	0.004000486	0.014847696
ENSMMUG00000013047	S-phase kinase associated protein 1 [Source:NCBI gene;Acc:100423634]	-2.289146658	8.84E-32	2.18E-29
ENSMMUG00000053384		-1.938495005	6.20E-20	4.60E-18
ENSMMUG00000004417	secretion associated Ras related GTPase 1B [Source:VGNC Symbol;Acc:VGNC:76970]	0.754012564	4.23E-11	9.40E-10
ENSMMUG00000012463	DEAD-box helicase 46 [Source:VGNC Symbol;Acc:VGNC:71663]	-0.959122046	1.01E-06	9.77E-06
ENSMMUG00000060605		0.66389867	0.003426514	0.013049837
ENSMMUG00000000342	transient receptor potential cation channel subfamily C member 7 [Source:VGNC Symbol;Acc:VGNC:79280]	1.546921856	0.008696343	0.028869317
ENSMMUG00000016357	polycystin 2 like 2, transient receptor potential cation channel [Source:VGNC Symbol;Acc:VGNC:76009]	-1.983482112	0.000412244	0.002088082
ENSMMUG00000008009	family with sequence similarity 13 member B [Source:VGNC Symbol;Acc:VGNC:72268]	-1.728026644	1.24E-10	2.55E-09
ENSMMUG00000005871	eukaryotic translation termination factor 1 [Source:VGNC Symbol;Acc:VGNC:84370]	-0.430642083	0.005308612	0.018914531
ENSMMUG00000017985	catenin alpha 1 [Source:VGNC Symbol;Acc:VGNC:84291]	0.523334183	1.89E-05	0.000135196
ENSMMUG00000058902	leucine rich repeat transmembrane neuronal 2 [Source:NCBI gene;Acc:714713]	0.410923323	0.013091352	0.040450557
ENSMMUG00000001941	CXXC finger protein 5 [Source:VGNC Symbol;Acc:VGNC:71594]	-0.647097703	1.80E-12	5.02E-11
ENSMMUG00000014636	pleckstrin and Sec7 domain containing 2 [Source:VGNC Symbol;Acc:VGNC:76286]	-0.730864214	0.002301871	0.009297621
ENSMMUG00000028372	Y RNA [Source:RFAM;Acc:RF00019]	-2.918138888	0.000435946	0.002197415
ENSMMUG00000019582	ankyrin repeat and KH domain-containing protein 1 [Source:NCBI gene;Acc:698045]	-0.316203123	0.002494436	0.009940135
ENSMMUG00000001707	amyloid beta precursor protein binding family B member 3 [Source:VGNC Symbol;Acc:VGNC:69869]	1.896315944	9.64E-26	1.30E-23
ENSMMUG00000001725	solute carrier family 35 member A4 [Source:VGNC Symbol;Acc:VGNC:77532]	1.791623092	9.89E-10	1.72E-08
ENSMMUG00000062326		1.925508118	1.36E-12	3.87E-11
ENSMMUG00000024537		2.708993706	1.39E-06	1.30E-05
ENSMMUG00000010007	CD14 molecule [Source:VGNC Symbol;Acc:VGNC:70751]	1.685393516	6.99E-07	7.00E-06
ENSMMUG00000060632		1.212193751	0.015123931	0.045575578
ENSMMUG00000018246	transmembrane and coiled-coil domains 6 [Source:VGNC Symbol;Acc:VGNC:84507]	1.312650093	8.69E-15	3.51E-13
ENSMMUG00000056796	NADH:ubiquinone oxidoreductase subunit A2 [Source:NCBI gene;Acc:697736]	1.652699485	0.001088492	0.004855295
ENSMMUG00000010008	IK cytokine [Source:VGNC Symbol;Acc:VGNC:73703]	1.424896038	5.55E-06	4.49E-05

ENSMMUG00000014571	protocadherin alpha-C2 [Source:NCBI gene;Acc:698874]	0.429686921	0.00047763	0.002375405
ENSMMUG00000008710	protocadherin 1 [Source:VGNC Symbol;Acc:VGNC:75775]	-1.400291146	3.20E-06	2.74E-05
ENSMMUG00000006287	DAP3 binding cell death enhancer 1 [Source:VGNC Symbol;Acc:VGNC:71806]	0.591431183	7.67E-06	6.01E-05
ENSMMUG00000006291	protocadherin 12 [Source:VGNC Symbol;Acc:VGNC:75776]	0.943513075	1.66E-06	1.52E-05
ENSMMUG00000009768	ring finger protein 14 [Source:VGNC Symbol;Acc:VGNC:84488]	0.692746345	1.27E-08	1.83E-07
ENSMMUG00000000419	Rho GTPase activating protein 26 [Source:VGNC Symbol;Acc:VGNC:69846]	1.689788282	4.00E-10	7.62E-09
ENSMMUG00000050405		1.726132992	2.22E-10	4.38E-09
ENSMMUG00000000421	nuclear receptor subfamily 3 group C member 1 [Source:VGNC Symbol;Acc:VGNC:75515]	1.485615596	2.25E-13	7.16E-12
ENSMMUG00000035921	U7 small nuclear RNA [Source:RFAM;Acc:RF00066]	1.497418994	0.005247863	0.018721027
ENSMMUG00000063348		-0.701228899	0.000194387	0.001078142
ENSMMUG00000019820	SH3 domain and tetratricopeptide repeats 2 [Source:VGNC Symbol;Acc:VGNC:77337]	0.661728269	7.94E-06	6.20E-05
ENSMMUG00000056140	U6 spliceosomal RNA [Source:RFAM;Acc:RF00026]	1.143733643	0.004878864	0.017581569
ENSMMUG00000017714	Rho guanine nucleotide exchange factor 37 [Source:VGNC Symbol;Acc:VGNC:69941]	-0.79243498	0.011785839	0.037052495
ENSMMUG00000004749	PPARG coactivator 1 beta [Source:VGNC Symbol;Acc:VGNC:76134]	2.831009667	2.30E-05	0.000161532
ENSMMUG00000011557	platelet derived growth factor receptor beta [Source:VGNC Symbol;Acc:VGNC:75929]	0.334505584	2.31E-08	3.13E-07
ENSMMUG00000005414	N-deacetylase and N-sulfotransferase 1 [Source:VGNC Symbol;Acc:VGNC:75151]	-0.89650272	1.04E-12	3.02E-11
ENSMMUG00000051062		-0.697646161	0.004788325	0.01729818
ENSMMUG00000020679	myozenin 3 [Source:VGNC Symbol;Acc:VGNC:74988]	1.485905659	4.37E-08	5.60E-07
ENSMMUG00000020683	RNA binding motif protein 22 [Source:VGNC Symbol;Acc:VGNC:76535]	2.174811329	9.73E-14	3.34E-12
ENSMMUG00000019764	dynactin subunit 4 [Source:VGNC Symbol;Acc:VGNC:71625]	1.725692699	6.10E-07	6.21E-06
ENSMMUG00000021051	TNFAIP3 interacting protein 1 [Source:VGNC Symbol;Acc:VGNC:79057]	-1.087731326	2.05E-06	1.83E-05
ENSMMUG00000021053	annexin A6 [Source:VGNC Symbol;Acc:VGNC:69951]	-1.094268975	5.14E-07	5.32E-06
ENSMMUG00000053656		0.844125997	6.02E-06	4.84E-05
ENSMMUG00000003499	solute carrier family 36 member 1 [Source:VGNC Symbol;Acc:VGNC:77509]	0.834793553	0.000969746	0.004405376
ENSMMUG00000003497	FAT atypical cadherin 2 [Source:VGNC Symbol;Acc:VGNC:72618]	-0.791831866	6.05E-07	6.17E-06
ENSMMUG00000053367	secreted protein acidic and cysteine rich [Source:NCBI gene;Acc:712388]	1.246704861	1.83E-15	8.27E-14
ENSMMUG00000054302	antioxidant 1 copper chaperone [Source:NCBI gene;Acc:712464]	-1.313819136	5.93E-06	4.78E-05
ENSMMUG00000056369	SAP30 like [Source:NCBI gene;Acc:714400]	-3.200117149	0.005175469	0.018503638
ENSMMUG00000021228	gem nuclear organelle associated protein 5 [Source:VGNC Symbol;Acc:VGNC:72751]	-0.609627852	0.000104975	0.000623123
ENSMMUG00000062861		-0.953994463	0.000734199	0.003457417
ENSMMUG00000059241		-5.626830117	3.28E-13	1.02E-11
ENSMMUG00000050043	adrenoceptor alpha 1B [Source:NCBI gene;Acc:695106]	1.014833606	0.01143658	0.036197161
ENSMMUG00000011472	PWWP domain containing 2A [Source:VGNC Symbol;Acc:VGNC:76564]	1.146999002	0.007898781	0.026524503
ENSMMUG00000015840	RAN binding protein 17 [Source:VGNC Symbol;Acc:VGNC:84485]	0.480493865	0.001012582	0.004573301
ENSMMUG00000003067	endoplasmic reticulum-golgi intermediate compartment 1 [Source:VGNC Symbol;Acc:VGNC:82141]	0.576480112	2.47E-06	2.17E-05
ENSMMUG00000019926	cytoplasmic polyadenylation element binding protein 4 [Source:VGNC Symbol;Acc:VGNC:71450]	0.740792795	7.85E-06	6.13E-05
ENSMMUG00000053691	Metazoan signal recognition particle RNA [Source:RFAM;Acc:RF00017]	2.022997732	0.004497687	0.01638258
ENSMMUG00000009059	Fas associated factor family member 2 [Source:VGNC Symbol;Acc:VGNC:72332]	-1.62095802	0.000331224	0.001717192
ENSMMUG00000013283	cadherin related family member 2 [Source:NCBI gene;Acc:697249]	2.481973673	1.33E-08	1.90E-07
ENSMMUG00000013284	G protein regulated inducer of neurite outgrowth 1 [Source:VGNC Symbol;Acc:VGNC:72998]	2.402040368	0.002307151	0.009313819
ENSMMUG00000000137	synuclein beta [Source:VGNC Symbol;Acc:VGNC:77670]	3.713369238	0.000327389	0.001700342

ENSMMUG00000002649	unc-5 netrin receptor A [Source:VGNC Symbol;Acc:VGNC:78721]	-2.278889958	0.001009843	0.004564703
ENSMMUG00000002653	hexokinase 3 [Source:VGNC Symbol;Acc:VGNC:73476]	-0.979812631	2.23E-10	4.39E-09
ENSMMUG00000009041	nuclear receptor binding SET domain protein 1 [Source:VGNC Symbol;Acc:VGNC:75401]	-1.012842464	0.001695947	0.007160612
ENSMMUG00000009045	RAB24, member RAS oncogene family [Source:VGNC Symbol;Acc:VGNC:81524]	-1.571954101	0.000628767	0.003019619
ENSMMUG00000016192	lectin, mannose binding 2 [Source:VGNC Symbol;Acc:VGNC:74302]	-0.418421234	0.012533189	0.039038991
ENSMMUG00000019807	regulator of G protein signaling 14 [Source:VGNC Symbol;Acc:VGNC:76918]	0.593947942	2.56E-07	2.84E-06
ENSMMUG00000016911	DEAD-box helicase 41 [Source:VGNC Symbol;Acc:VGNC:71660]	-0.612641342	0.011275429	0.035772788
ENSMMUG00000004002	CDC like kinase 4 [Source:VGNC Symbol;Acc:VGNC:71173]	1.392730482	7.09E-25	8.59E-23
ENSMMUG00000041519	zinc finger protein 354A [Source:VGNC Symbol;Acc:VGNC:84517]	1.92874639	0.000127108	0.000739997
ENSMMUG00000049557		-1.237048182	0.005263595	0.018767937
ENSMMUG00000001728	mitogen-activated protein kinase 9 [Source:VGNC Symbol;Acc:VGNC:74652]	-1.215215549	8.31E-10	1.48E-08
ENSMMUG00000054010		0.526229274	0.003412347	0.012999288
ENSMMUG00000031833	butyrophilin like 9 [Source:NCBI gene;Acc:106996463]	0.692501277	0.008221906	0.027468782
ENSMMUG00000062522		1.444025665	3.47E-10	6.67E-09
ENSMMUG00000059428	olfactory receptor 2V2 [Source:NCBI gene;Acc:707790]	0.863098076	0.013240111	0.040838423
ENSMMUG00000061151		1.594771943	4.55E-08	5.82E-07
ENSMMUG00000054414		1.859353651	1.26E-13	4.21E-12
ENSMMUG00000003598	tripartite motif containing 7 [Source:VGNC Symbol;Acc:VGNC:79022]	1.401063891	4.50E-13	1.38E-11
ENSMMUG00000021145	family with sequence similarity 120B [Source:VGNC Symbol;Acc:VGNC:72311]	-1.448986181	1.32E-18	8.60E-17
ENSMMUG00000057577		-3.231209729	0.001568931	0.006684511
ENSMMUG00000039829		-0.962720091	0.00039468	0.002005406
ENSMMUG00000014474	SPARC related modular calcium binding 2 [Source:VGNC Symbol;Acc:VGNC:83470]	-0.866590501	2.17E-13	6.92E-12
ENSMMUG00000021044	afadin, adherens junction formation factor [Source:VGNC Symbol;Acc:VGNC:69818]	0.83304979	5.55E-08	7.01E-07
ENSMMUG00000064719		-1.392205451	0.003021731	0.011704478
ENSMMUG00000053990		0.996308087	0.002514806	0.010015817
ENSMMUG00000055611		0.669773166	0.006341274	0.021958813
ENSMMUG00000055168		0.613539014	0.000285375	0.001513496
ENSMMUG00000062443		-1.394073384	4.44E-08	5.69E-07
ENSMMUG00000057859		-2.57714128	3.91E-08	5.08E-07
ENSMMUG00000029352	T-complex protein 10A homolog 2 [Source:NCBI gene;Acc:693619]	-3.455014603	0.000459711	0.002300453
ENSMMUG00000005680	tubulin tyrosine ligase like 2 [Source:VGNC Symbol;Acc:VGNC:78421]	-2.500171365	0.009115158	0.030060584
ENSMMUG00000008228	unc-93 homolog A [Source:VGNC Symbol;Acc:VGNC:78724]	-2.474410402	5.64E-07	5.79E-06
ENSMMUG00000057983		3.25035592	9.95E-05	0.000593623
ENSMMUG00000054834		0.513718634	7.95E-06	6.20E-05
ENSMMUG00000020412	parkin coregulated [Source:VGNC Symbol;Acc:VGNC:75646]	-0.553571186	0.001000418	0.004528086
ENSMMUG00000004181	PARN like, ribonuclease domain containing 1 [Source:VGNC Symbol;Acc:VGNC:76089]	-1.350109358	0.007523677	0.025512407

ENSMMUG00000057544		-0.894117154	0.000813691	0.003788105
ENSMMUG00000000500	fibronectin type III domain containing 1 [Source:VGNC Symbol;Acc:VGNC:72546]	0.363497364	0.014828755	0.044879489
ENSMMUG00000011994	T cell activation RhoGTPase activating protein [Source:VGNC Symbol;Acc:VGNC:78126]	0.760383888	0.000109045	0.000643861
ENSMMUG00000022247	osteoclastogenesis associated transmembrane protein 1 [Source:VGNC Symbol;Acc:VGNC:75664]	2.018828029	2.55E-14	9.81E-13
ENSMMUG00000007010	coiled-coil domain-containing protein 162-like [Source:NCBI gene;Acc:700544]	1.691365366	0.001021311	0.004606516
ENSMMUG00000021534	solute carrier family 16 member 10 [Source:NCBI gene;Acc:696132]	-1.856050594	0.000101123	0.000601978
ENSMMUG00000013782	laminin subunit alpha 4 [Source:VGNC Symbol;Acc:VGNC:74227]	-0.367338136	0.003177436	0.012213216
ENSMMUG00000061031		2.902559441	4.50E-17	2.44E-15
ENSMMUG00000058557		1.566338212	3.57E-07	3.84E-06
ENSMMUG00000017189	regulatory factor X6 [Source:VGNC Symbol;Acc:VGNC:76800]	1.953369901	6.89E-12	1.77E-10
ENSMMUG00000059327	NUS1 dehydrodolichyl diphosphate synthase subunit [Source:NCBI gene;Acc:715015]	-0.810219754	5.82E-09	8.89E-08
ENSMMUG00000008525	minichromosome maintenance 9 homologous recombination repair factor [Source:VGNC Symbol;Acc:VGNC:83432]	-2.628111913	2.54E-05	0.000176694
ENSMMUG00000013302	family with sequence similarity 184 member A [Source:VGNC Symbol;Acc:VGNC:72308]	-3.700903797	4.30E-08	5.52E-07
ENSMMUG00000005225	gap junction protein alpha 1 [Source:VGNC Symbol;Acc:VGNC:73055]	-0.728076201	0.000130669	0.000759209
ENSMMUG00000061165		0.588774258	1.58E-05	0.000115009
ENSMMUG00000051170		0.817997184	0.008752283	0.029041759
ENSMMUG00000060822		-0.783988289	2.37E-08	3.20E-07
ENSMMUG00000018176	clavesin 2 [Source:VGNC Symbol;Acc:VGNC:71322]	0.894344844	1.03E-06	9.92E-06
ENSMMUG00000009725	triadin [Source:HGNC Symbol;Acc:HGNC:12261]	0.8255173	1.33E-09	2.28E-08
ENSMMUG00000065276		0.551308894	0.001020092	0.004602445
ENSMMUG00000003134	ring finger protein 217 [Source:VGNC Symbol;Acc:VGNC:76801]	-0.902407725	0.000170232	0.000957318
ENSMMUG00000062110	TPD52 like 1 [Source:NCBI gene;Acc:716568]	-0.896947486	0.001202236	0.005300851
ENSMMUG00000060901		1.465879711	1.99E-21	1.73E-19
ENSMMUG00000015355	R-spondin 3 [Source:VGNC Symbol;Acc:VGNC:77066]	-2.054307168	0.001188934	0.005250162
ENSMMUG00000048882	KIAA0408 ortholog [Source:NCBI gene;Acc:100429459]	-0.575066584	4.01E-06	3.35E-05
ENSMMUG00000029421	protein tyrosine phosphatase receptor type K [Source:VGNC Symbol;Acc:VGNC:83453]	1.116712864	0.005356405	0.019061458
ENSMMUG00000058574		0.943004315	1.07E-05	8.17E-05
ENSMMUG00000022136	laminin subunit alpha 2 [Source:VGNC Symbol;Acc:VGNC:74225]	0.474863328	8.50E-05	0.000517524
ENSMMUG00000021498	L3MBTL histone methyl-lysine binding protein 3 [Source:VGNC Symbol;Acc:VGNC:74106]	1.170097766	2.85E-11	6.53E-10
ENSMMUG00000021499	sterile alpha motif domain containing 3 [Source:VGNC Symbol;Acc:VGNC:77079]	-0.803606133	0.000189706	0.001056208
ENSMMUG00000059338	Y RNA [Source:RFAM;Acc:RF00019]	1.316529761	0.002072376	0.008521744
ENSMMUG00000065212		1.197230635	1.81E-07	2.08E-06
ENSMMUG00000059491		0.696713301	0.000162421	0.000917733
ENSMMUG00000017549	ectonucleotide pyrophosphatase/phosphodiesterase 3 [Source:VGNC Symbol;Acc:VGNC:72224]	-0.890415681	9.56E-06	7.34E-05
ENSMMUG00000004996	monooxygenase DBH like 1 [Source:VGNC Symbol;Acc:VGNC:74800]	0.53750627	0.003682751	0.013862285
ENSMMUG00000004988	trace amine-associated receptor 3 [Source:NCBI gene;Acc:709130]	-0.859304246	0.000917379	0.004207382
ENSMMUG00000004987	trace amine associated receptor 2 [Source:VGNC Symbol;Acc:VGNC:83475]	-0.819033151	0.004041179	0.014967292

ENSMMUG00000013236	solute carrier family 18 member B1 [Source:VGNC Symbol;Acc:VGNC:77549]	-0.621759864	0.008796001	0.029146903
ENSMMUG00000005745	EYA transcriptional coactivator and phosphatase 4 [Source:VGNC Symbol;Acc:VGNC:72456]	1.026870452	4.60E-09	7.12E-08
ENSMMUG00000022921	solute carrier family 2 member 12 [Source:VGNC Symbol;Acc:VGNC:77503]	3.629336473	4.78E-40	3.66E-37
ENSMMUG00000061787		2.287999067	0.006359115	0.02201535
ENSMMUG00000053927		0.642069599	0.007028345	0.023995079
ENSMMUG00000018166	MYB proto-oncogene, transcription factor [Source:VGNC Symbol;Acc:VGNC:75089]	0.938593906	8.91E-09	1.32E-07
ENSMMUG00000009368	Abelson helper integration site 1 [Source:VGNC Symbol;Acc:VGNC:81010]	0.690146966	8.11E-06	6.30E-05
ENSMMUG00000044284	40S ribosomal protein S11-like [Source:NCBI gene;Acc:706532]	0.755158201	0.002072841	0.008521744
ENSMMUG00000053936		0.527967156	0.011717423	0.03688527
ENSMMUG00000016376	mitogen-activated protein kinase kinase kinase 5 [Source:VGNC Symbol;Acc:VGNC:74495]	0.890271641	0.001552274	0.006617421
ENSMMUG00000048283	mml-mir-3145 [Source:miRBase;Acc:MI0020897]	1.168292104	0.009320983	0.03058671
ENSMMUG00000000224	RALBP1 associated Eps domain containing 1 [Source:VGNC Symbol;Acc:VGNC:76738]	-1.381831793	0.012083882	0.037858489
ENSMMUG00000019136	taxilin beta [Source:VGNC Symbol;Acc:VGNC:79325]	0.541933274	1.40E-06	1.30E-05
ENSMMUG00000046765		-0.738979189	0.012628748	0.039252537
ENSMMUG00000058187		1.151468791	1.16E-14	4.56E-13
ENSMMUG00000023012	vesicle trafficking 1 [Source:VGNC Symbol;Acc:VGNC:79296]	-1.368038641	7.88E-05	0.000483301
ENSMMUG00000044958	adhesion G protein-coupled receptor G6 [Source:VGNC Symbol;Acc:VGNC:81009]	-1.181551298	1.03E-09	1.80E-08
ENSMMUG00000064366		-0.919805463	0.012401759	0.03872087
ENSMMUG00000061117		-1.113643964	3.61E-07	3.88E-06
ENSMMUG00000007998	HIVEP zinc finger 2 [Source:VGNC Symbol;Acc:VGNC:83427]	-0.616839797	0.003355612	0.012813377
ENSMMUG00000006430	androgen induced 1 [Source:VGNC Symbol;Acc:VGNC:69667]	0.834082258	0.011873367	0.037303466
ENSMMUG00000005348	LTV1 ribosome biogenesis factor [Source:VGNC Symbol;Acc:VGNC:83430]	2.478969127	0.002439469	0.009753181
ENSMMUG00000003750	PLAG1 like zinc finger 1 [Source:VGNC Symbol;Acc:VGNC:83444]	1.772681584	2.54E-06	2.21E-05
ENSMMUG00000019443	syntaxin 11 [Source:VGNC Symbol;Acc:VGNC:78041]	3.717963443	0.000101813	0.000605833
ENSMMUG00000019438	utrophin [Source:VGNC Symbol;Acc:VGNC:78749]	0.889723518	0.002839804	0.011085456
ENSMMUG00000023265	A-kinase anchoring protein 12 [Source:VGNC Symbol;Acc:VGNC:69766]	-2.167627463	1.03E-11	2.55E-10
ENSMMUG00000060839		-3.306930311	0.005656335	0.019986907
ENSMMUG00000029475	zinc finger and BTB domain containing 2 [Source:VGNC Symbol;Acc:VGNC:79291]	-2.090996737	3.91E-06	3.28E-05
ENSMMUG00000018342	required for meiotic nuclear division 1 homolog [Source:VGNC Symbol;Acc:VGNC:76797]	-2.480412823	2.42E-13	7.68E-12
ENSMMUG00000060940	acidic residue methyltransferase 1 [Source:NCBI gene;Acc:704570]	-1.914933217	0.00049997	0.002470451
ENSMMUG00000059807	Metazoan signal recognition particle RNA [Source:RFAM;Acc:RF00017]	-3.199359398	0.005190893	0.018549657
ENSMMUG00000018346	coiled-coil domain containing 170 [Source:VGNC Symbol;Acc:VGNC:70832]	-2.165343702	2.69E-07	2.98E-06
ENSMMUG00000025949	U6 spliceosomal RNA [Source:RFAM;Acc:RF00026]	-3.076627053	0.002960998	0.011506023
ENSMMUG00000019045	estrogen receptor 1 [Source:VGNC Symbol;Acc:VGNC:72432]	-1.252534443	7.52E-07	7.44E-06
ENSMMUG00000013483	prolyl endopeptidase [Source:VGNC Symbol;Acc:VGNC:76396]	0.646679691	0.002202715	0.008976976
ENSMMUG00000055978		0.673146822	0.000452566	0.002271737
ENSMMUG00000057697		-1.304101648	6.33E-07	6.39E-06

ENSMMUG00000051522	Metazoan signal recognition particle RNA [Source:RFAM;Acc:RF00017]	-2.454432688	0.001060835	0.004758212
ENSMMUG00000051271		-3.354112294	3.75E-21	3.18E-19
ENSMMUG00000000808	melanin concentrating hormone receptor 2 [Source:VGNC Symbol;Acc:VGNC:74550]	1.108107386	1.94E-23	1.95E-21
ENSMMUG00000050740	coenzyme Q3, methyltransferase [Source:NCBI gene;Acc:694215]	-1.051031414	0.010960997	0.03495065
ENSMMUG00000005923	failed axon connections homolog, metaxin like GST domain containing [Source:VGNC Symbol;Acc:VGNC:72541]	-0.887553122	0.007694296	0.025987913
ENSMMUG00000037890		1.091449987	0.003070415	0.011870912
ENSMMUG00000003464	midasin AAA ATPase 1 [Source:VGNC Symbol;Acc:VGNC:74530]	-0.257537713	0.004768056	0.017229241
ENSMMUG00000003462	ankyrin repeat domain 6 [Source:VGNC Symbol;Acc:VGNC:81023]	-0.519719131	2.63E-05	0.000182054
ENSMMUG00000060841		-0.96862728	0.002852121	0.01112756
ENSMMUG00000002824	akirin 2 [Source:VGNC Symbol;Acc:VGNC:69665]	-0.717241081	0.013726582	0.042115519
ENSMMUG00000002821	origin recognition complex subunit 3 [Source:VGNC Symbol;Acc:VGNC:75715]	-0.633452256	0.009197847	0.03027833
ENSMMUG00000045108	cilia and flagella associated protein 206 [Source:NCBI gene;Acc:700665]	3.289681266	0.009596325	0.03132056
ENSMMUG00000064804		-1.38493253	1.58E-07	1.84E-06
ENSMMUG00000005442	5'-nucleotidase ecto [Source:VGNC Symbol;Acc:VGNC:75537]	-1.532775347	7.23E-10	1.30E-08
ENSMMUG00000061590		-2.247659464	8.55E-05	0.000519465
ENSMMUG00000017186	pleckstrin homology domain interacting protein [Source:VGNC Symbol;Acc:VGNC:75803]	-1.86226433	2.36E-09	3.85E-08
ENSMMUG00000004491	SUMO specific peptidase 6 [Source:VGNC Symbol;Acc:VGNC:77287]	-0.295048622	0.002937125	0.011428529
ENSMMUG00000017806	CD109 molecule [Source:VGNC Symbol;Acc:VGNC:70834]	0.668193435	4.16E-08	5.37E-07
ENSMMUG00000008133	solute carrier family 17 member 5 [Source:VGNC Symbol;Acc:VGNC:77405]	0.706919489	3.02E-08	3.99E-07
ENSMMUG00000053928		0.586550331	0.00390939	0.014560801
ENSMMUG00000041338	eyes shut homolog [Source:HGNC Symbol;Acc:HGNC:21555]	-0.332215318	0.003892512	0.01450537
ENSMMUG00000017160	PHD finger protein 3 [Source:VGNC Symbol;Acc:VGNC:75975]	-0.475952161	0.011123823	0.035368114
ENSMMUG00000062339	endogenous retrovirus group K member 25 Env polyprotein-like [Source:NCBI gene;Acc:114677579]	-0.615404807	0.007939491	0.026642748
ENSMMUG00000002777	protein tyrosine phosphatase 4A1 [Source:VGNC Symbol;Acc:VGNC:83452]	-0.379129995	0.007730301	0.026061422
ENSMMUG00000050075		-3.112874736	0.006773187	0.023227606
ENSMMUG00000001399	3-hydroxymethyl-3-methylglutaryl-CoA lyase like 1 [Source:VGNC Symbol;Acc:VGNC:73429]	-0.506645071	0.002256453	0.009152359
ENSMMUG00000001395	hypocretin receptor 2 [Source:VGNC Symbol;Acc:VGNC:73357]	-3.181019451	0.002275483	0.009219259
ENSMMUG00000036060	U6 spliceosomal RNA [Source:RFAM;Acc:RF00026]	3.08320977	0.000369638	0.001889752
ENSMMUG00000012784	tubulointerstitial nephritis antigen [Source:VGNC Symbol;Acc:VGNC:78382]	-1.854770316	0.00127318	0.005572853
ENSMMUG00000012781	muscular LMNA interacting protein [Source:VGNC Symbol;Acc:VGNC:83434]	-2.193480894	1.72E-11	4.12E-10
ENSMMUG00000011049	leucine rich repeat containing 1 [Source:VGNC Symbol;Acc:VGNC:81087]	-1.672020069	1.14E-06	1.09E-05
ENSMMUG00000006422	ELOVL fatty acid elongase 5 [Source:VGNC Symbol;Acc:VGNC:72083]	-1.196526057	0.016479329	0.048939736
ENSMMUG00000013718	translocation associated membrane protein 2 [Source:VGNC Symbol;Acc:VGNC:83515]	-0.815946998	3.86E-06	3.25E-05

ENSMMUG00000013716	EF-hand domain containing 1 [Source:VGNC Symbol;Acc:VGNC:72052]	-0.568857221	0.000698933	0.003318222
ENSMMUG00000052626	progestin and adipoQ receptor family member 8 [Source:NCBI gene;Acc:708418]	0.616937731	4.27E-06	3.54E-05
ENSMMUG00000026975	mml-mir-206 [Source:miRBase;Acc:MI0007667]	5.348939206	1.99E-05	0.000142025
ENSMMUG00000016993	PKHD1 ciliary IPT domain containing fibrocystin/polyductin [Source:VGNC Symbol;Acc:VGNC:76010]	-2.454963506	4.83E-13	1.46E-11
ENSMMUG00000038096	Metazoan signal recognition particle RNA [Source:RFAM;Acc:RF00017]	-3.326475478	0.01018774	0.032918243
ENSMMUG00000053984		3.421991607	7.64E-11	1.63E-09
ENSMMUG00000056168		4.298842591	1.62E-05	0.000117471
ENSMMUG00000055199	cytochrome P450, family 39, subfamily A, polypeptide 1 [Source:NCBI gene;Acc:704242]	2.631586218	1.71E-10	3.45E-09
ENSMMUG00000019246	regulator of calcineurin 2 [Source:VGNC Symbol;Acc:VGNC:82092]	2.102460491	9.08E-10	1.60E-08
ENSMMUG00000042260	ectonucleotide pyrophosphatase/phosphodiesterase 4 [Source:VGNC Symbol;Acc:VGNC:81066]	-2.008611431	0.014449706	0.043908914
ENSMMUG00000003511	chloride intracellular channel 5 [Source:VGNC Symbol;Acc:VGNC:71171]	-0.526299643	0.005038355	0.01808905
ENSMMUG00000051732		-2.424970167	0.011492085	0.036327922
ENSMMUG00000021377	SPT3 homolog, SAGA and STAGA complex component [Source:VGNC Symbol;Acc:VGNC:78055]	-1.004886269	3.25E-07	3.53E-06
ENSMMUG00000060503	Metazoan signal recognition particle RNA [Source:RFAM;Acc:RF00017]	-0.802882383	0.008756088	0.029047753
ENSMMUG00000063288		-0.61786748	0.000154112	0.000877184
ENSMMUG00000013920	triggering receptor expressed on myeloid cells 2 [Source:VGNC Symbol;Acc:VGNC:79089]	-1.031837886	8.26E-05	0.000504573
ENSMMUG00000062538	adenylate cyclase type 10 [Source:NCBI gene;Acc:719662]	-0.972310907	0.00011134	0.000655546
ENSMMUG00000021173	nuclear transcription factor Y subunit alpha [Source:VGNC Symbol;Acc:VGNC:75344]	-0.878546667	2.22E-12	6.15E-11
ENSMMUG00000021169	translocator protein 2 [Source:VGNC Symbol;Acc:VGNC:83960]	-0.778241054	6.17E-05	0.00038918
ENSMMUG00000021168	unc-5 family C-terminal like [Source:VGNC Symbol;Acc:VGNC:79314]	-0.901196247	3.28E-12	8.83E-11
ENSMMUG00000013651	kinesin family member 6 [Source:VGNC Symbol;Acc:VGNC:73873]	3.449495401	0.000182659	0.001019704
ENSMMUG00000013648	potassium two pore domain channel subfamily K member 16 [Source:VGNC Symbol;Acc:VGNC:73827]	2.986214934	0.002169553	0.008869178
ENSMMUG00000013644	potassium two pore domain channel subfamily K member 17 [Source:VGNC Symbol;Acc:VGNC:73828]	2.29522587	1.07E-11	2.65E-10
ENSMMUG00000023381	dynein axonemal heavy chain 8 [Source:VGNC Symbol;Acc:VGNC:71943]	0.570275453	1.63E-06	1.49E-05
ENSMMUG00000062055	glyoxalase I [Source:NCBI gene;Acc:719518]	0.779267563	2.19E-10	4.32E-09
ENSMMUG00000011575	coiled-coil domain containing 167 [Source:VGNC Symbol;Acc:VGNC:70833]	-1.979320188	0.000137236	0.000792294
ENSMMUG00000011573	cap methyltransferase 1 [Source:VGNC Symbol;Acc:VGNC:71271]	-1.375541276	1.41E-05	0.00010457
ENSMMUG00000063198		4.231768626	6.28E-13	1.87E-11
ENSMMUG00000055914		1.309540592	1.20E-06	1.13E-05
ENSMMUG00000015416	bromodomain and PHD finger containing 3 [Source:VGNC Symbol;Acc:VGNC:70309]	1.441710167	1.00E-08	1.47E-07
ENSMMUG00000023823	Y RNA [Source:RFAM;Acc:RF00019]	4.295468668	0.000781887	0.003650062
ENSMMUG00000000869	small nuclear ribonucleoprotein polypeptide C [Source:NCBI gene;Acc:718654]	2.98982407	4.70E-09	7.26E-08
ENSMMUG00000007811	protein kinase C and casein kinase substrate in neurons 1 [Source:VGNC Symbol;Acc:VGNC:75744]	0.422544879	0.015926168	0.047539776
ENSMMUG00000004287	death domain associated protein [Source:VGNC Symbol;Acc:VGNC:71737]	-0.90845433	0.000226966	0.00123573
ENSMMUG00000003869	WD repeat domain 46 [Source:VGNC Symbol;Acc:VGNC:78940]	-1.077888343	0.000153242	0.000873204
ENSMMUG00000003864	VPS52 subunit of GARP complex [Source:VGNC Symbol;Acc:VGNC:78551]	-0.572306836	0.010310183	0.033247367
ENSMMUG00000003867	ribosomal protein S18 [Source:NCBI gene;Acc:706414]	-1.115117113	0.015351203	0.046136124

ENSMMUG00000003858	retinoid X receptor beta [Source:VGNC Symbol;Acc:VGNC:76952]	-0.738017787	0.000703688	0.003337522
ENSMMUG00000003850	collagen type XI alpha 2 chain [Source:VGNC Symbol;Acc:VGNC:71290]	-0.735665397	2.16E-06	1.92E-05
ENSMMUG00000019371	major histocompatibility complex, class II, DP alpha [Source:NCBI gene;Acc:717992]	-1.168526086	0.004169648	0.015357023
ENSMMUG00000023138	bromodomain containing 2 [Source:VGNC Symbol;Acc:VGNC:83422]	-1.592424992	0.003693155	0.013897851
ENSMMUG00000018461	notch receptor 4 [Source:VGNC Symbol;Acc:VGNC:75372]	-1.316942551	0.000103365	0.000614313
ENSMMUG00000018459	G protein signaling modulator 3 [Source:VGNC Symbol;Acc:VGNC:72938]	-1.054616807	0.007477942	0.02540473
ENSMMUG00000018456	PBX homeobox 2 [Source:VGNC Symbol;Acc:VGNC:75771]	-1.013872766	0.011492888	0.036327922
ENSMMUG00000018434	1-acylglycerol-3-phosphate O-acyltransferase 1 [Source:VGNC Symbol;Acc:VGNC:69745]	-0.917931791	0.013851129	0.042438875
ENSMMUG00000064499		2.258529822	0.016641709	0.049347839
ENSMMUG00000006828	valyl-tRNA synthetase 1 [Source:VGNC Symbol;Acc:VGNC:78755]	-1.875646677	0.00215425	0.008814054
ENSMMUG00000006827	von Willebrand factor A domain containing 7 [Source:VGNC Symbol;Acc:VGNC:78941]	-2.566469235	0.000998062	0.00452129
ENSMMUG00000009702	mutS homolog 5 [Source:NCBI gene;Acc:715494]	-1.503073271	0.003669038	0.013824987
ENSMMUG00000058534		2.992921345	3.15E-09	5.03E-08
ENSMMUG00000015693	lymphocyte antigen 6 family member G6C [Source:VGNC Symbol;Acc:VGNC:74285]	2.557526189	2.18E-10	4.30E-09
ENSMMUG00000053324	lymphocyte antigen 6 complex locus protein G6d [Source:NCBI gene;Acc:715304]	2.352242281	6.66E-20	4.91E-18
ENSMMUG00000039268	lymphocyte antigen 6G6e-like [Source:NCBI gene;Acc:100427419]	2.613589535	0.014885188	0.044986689
ENSMMUG00000015686	abhydrolase domain containing 16A [Source:VGNC Symbol;Acc:VGNC:69393]	2.232600112	5.49E-36	2.57E-33
ENSMMUG00000054159	lymphocyte antigen 6 family member G5C [Source:VGNC Symbol;Acc:VGNC:83431]	2.575556638	0.000328256	0.001704233
ENSMMUG00000005294	casein kinase 2 beta [Source:NCBI gene;Acc:715807]	2.460256122	2.22E-11	5.17E-10
ENSMMUG00000005293	G-patch domain and ankyrin repeats 1 [Source:VGNC Symbol;Acc:VGNC:81073]	2.630445656	2.35E-09	3.85E-08
ENSMMUG00000005302	BAG cochaperone 6 [Source:VGNC Symbol;Acc:VGNC:81044]	2.369046144	2.12E-39	1.34E-36
ENSMMUG00000005303	apolipoprotein M [Source:VGNC Symbol;Acc:VGNC:69825]	2.588624531	2.44E-07	2.72E-06
ENSMMUG00000005297	proline rich coiled-coil 2A [Source:VGNC Symbol;Acc:VGNC:83448]	2.045813591	7.22E-30	1.61E-27
ENSMMUG00000025918	small nucleolar RNA, H/ACA box 38 [Source:HGNC Symbol;Acc:HGNC:32631]	2.44861182	6.22E-06	4.98E-05
ENSMMUG00000049735	allograft inflammatory factor 1 [Source:NCBI gene;Acc:574115]	2.243355339	3.51E-28	6.45E-26
ENSMMUG00000008854	natural cytotoxicity triggering receptor 3 [Source:VGNC Symbol;Acc:VGNC:74990]	2.447342656	1.79E-27	3.05E-25
ENSMMUG00000008853	leukocyte specific transcript 1 [Source:HGNC Symbol;Acc:HGNC:14189]	1.558505292	0.006125298	0.021302263
ENSMMUG00000045654	tumor necrosis factor [Source:VGNC Symbol;Acc:VGNC:83501]	1.745231989	0.000166942	0.000940632
ENSMMUG00000052422	ATPase H+ transporting V1 subunit G2 [Source:NCBI gene;Acc:715331]	4.052199927	0.000328375	0.001704245
ENSMMUG00000008839	DExD-box helicase 39B [Source:VGNC Symbol;Acc:VGNC:71621]	1.689733886	1.30E-05	9.67E-05
ENSMMUG00000026474	Small nucleolar RNA SNORD83 [Source:RFAM;Acc:RF00137]	3.05932	0.007527419	0.025513893
ENSMMUG00000019961	protein phosphatase 1 regulatory subunit 18 [Source:NCBI gene;Acc:712176]	3.472513165	0.000186123	0.001037453
ENSMMUG00000053627		2.833216564	0.000138903	0.000800328
ENSMMUG00000011682	ribonuclease P/MRP subunit p21 [Source:NCBI gene;Acc:713056]	3.368585251	1.66E-06	1.52E-05
ENSMMUG00000060527		-1.507164708	1.86E-08	2.55E-07
ENSMMUG00000023679	tripartite motif containing 26 [Source:VGNC Symbol;Acc:VGNC:78854]	-1.408175415	3.72E-08	4.84E-07
ENSMMUG00000002890	tripartite motif containing 15 [Source:VGNC Symbol;Acc:VGNC:83958]	-1.270420024	1.48E-08	2.08E-07
ENSMMUG00000012129	tripartite motif containing 10 [Source:VGNC Symbol;Acc:VGNC:78550]	-1.258718267	3.82E-09	6.00E-08

ENSMMUG00000058580	tripartite motif containing 40 [Source:NCBI gene;Acc:100427925]	-1.060395381	0.000472811	0.002355466
ENSMMUG00000062264		-1.384283365	2.78E-05	0.000191245
ENSMMUG00000012124	ring finger protein 39 [Source:VGNC Symbol;Acc:VGNC:83458]	-1.467108455	1.16E-06	1.10E-05
ENSMMUG00000012120	zinc ribbon domain containing 1 [Source:VGNC Symbol;Acc:VGNC:78912]	-1.429766935	7.15E-07	7.13E-06
ENSMMUG00000055272	putative uncharacterized protein ZNRD1-AS1 [Source:NCBI gene;Acc:712069]	-1.047839339	0.007657766	0.02588859
ENSMMUG00000056544		-1.395991159	0.004354416	0.015952658
ENSMMUG00000037625		-1.54563748	2.07E-06	1.84E-05
ENSMMUG00000053561		3.831668694	1.51E-05	0.000110658
ENSMMUG00000029883	olfactory receptor, family 2, subfamily J, member 3 [Source:NCBI gene;Acc:708943]	3.598370291	7.43E-05	0.000459706
ENSMMUG00000059442		2.037110464	0.001517628	0.006488752
ENSMMUG00000005415	olfactory receptor, family 2, subfamily B, member 3 [Source:NCBI gene;Acc:708747]	1.860029029	0.014938783	0.045111152
ENSMMUG00000004617	glutathione peroxidase 6 [Source:VGNC Symbol;Acc:VGNC:73154]	-0.707990985	0.012458306	0.038864032
ENSMMUG00000012396	zinc finger and SCAN domain containing 23 [Source:VGNC Symbol;Acc:VGNC:79313]	-0.912348361	1.91E-06	1.72E-05
ENSMMUG00000011675	zinc finger and SCAN domain containing 12 [Source:VGNC Symbol;Acc:VGNC:78910]	-0.963553255	8.62E-10	1.53E-08
ENSMMUG00000005964	CMP-N-acetylneuraminic acid hydroxylase [Source:NCBI gene;Acc:574186]	1.041111898	0.000571347	0.002776886
ENSMMUG00000049021	Metazoan signal recognition particle RNA [Source:RFAM;Acc:RF00017]	2.386192866	5.10E-05	0.000330209
ENSMMUG00000058151	chromosome 4 C6orf62 homolog [Source:NCBI gene;Acc:708218]	-1.354009396	1.30E-20	1.06E-18
ENSMMUG00000051037		4.297112117	1.16E-17	6.70E-16
ENSMMUG00000054532		-1.111912506	5.22E-05	0.000336802
ENSMMUG00000052442		-0.836991837	1.69E-07	1.95E-06
ENSMMUG00000063576	Metazoan signal recognition particle RNA [Source:RFAM;Acc:RF00017]	-1.229675309	0.000789703	0.003683002
ENSMMUG00000052333		0.613847348	0.002229386	0.009062805
ENSMMUG00000052891		1.760737212	7.27E-12	1.85E-10
ENSMMUG00000020289	CD83 molecule [Source:VGNC Symbol;Acc:VGNC:70783]	-1.390333375	1.18E-11	2.90E-10
ENSMMUG00000044473		-1.182662616	7.61E-08	9.33E-07
ENSMMUG00000009922	mitochondrial calcium uniporter regulator 1 [Source:VGNC Symbol;Acc:VGNC:81471]	-0.765600134	0.000308318	0.00161398
ENSMMUG00000020122	RAN binding protein 9 [Source:VGNC Symbol;Acc:VGNC:76475]	-0.567271851	0.004668496	0.016915735
ENSMMUG00000008681	nucleolar protein 7 [Source:VGNC Symbol;Acc:VGNC:75226]	-1.111540108	0.004319099	0.015855239
ENSMMUG00000020040	phosphatase and actin regulator 1 [Source:VGNC Symbol;Acc:VGNC:75841]	1.192187733	0.000297884	0.001564182
ENSMMUG00000058800		1.193099664	1.47E-10	2.99E-09
ENSMMUG00000016135	neural precursor cell expressed, developmentally down-regulated 9 [Source:VGNC Symbol;Acc:VGNC:75304]	-1.16896525	2.71E-07	2.99E-06
ENSMMUG00000046520	small integral membrane protein 13 [Source:NCBI gene;Acc:703387]	-0.613809284	0.010321446	0.033268928
ENSMMUG00000043161	endogenous retrovirus group FRD member 1, envelope [Source:VGNC Symbol;Acc:VGNC:81068]	-1.304945507	6.15E-07	6.25E-06
ENSMMUG00000063667		-3.016201982	0.014393956	0.043804813

ENSMMUG00000005532	male germ cell associated kinase [Source:VGNC Symbol;Acc:VGNC:74480]	0.669647747	0.000287624	0.001522159
ENSMMUG00000018788	transmembrane protein 14B [Source:NCBI gene;Acc:698237]	0.552850124	0.006850311	0.023436803
ENSMMUG00000018787	PAK1 interacting protein 1 [Source:VGNC Symbol;Acc:VGNC:75661]	0.94737254	3.92E-08	5.08E-07
ENSMMUG00000061586	chromosome 4 C6orf52 homolog [Source:NCBI gene;Acc:702114]	0.955552705	5.53E-07	5.69E-06
ENSMMUG00000038560	eukaryotic translation elongation factor 1 epsilon 1 [Source:NCBI gene;Acc:695426]	-1.29511167	2.41E-19	1.71E-17
ENSMMUG00000044859		-1.254817719	0.007557074	0.025595804
ENSMMUG00000021600	bone morphogenetic protein 6 [Source:VGNC Symbol;Acc:VGNC:70265]	-0.719662118	0.007971807	0.026726512
ENSMMUG00000015577	RIO kinase 1 [Source:VGNC Symbol;Acc:VGNC:76767]	-0.936621364	0.001260643	0.005529861
ENSMMUG00000016352	phenylalanyl-tRNA synthetase 2, mitochondrial [Source:VGNC Symbol;Acc:VGNC:72545]	0.608287314	0.01153438	0.036411569
ENSMMUG00000060973		0.517713274	0.00266373	0.010476906
ENSMMUG00000023558	exocyst complex component 2 [Source:VGNC Symbol;Acc:VGNC:72338]	-1.138861836	0.009093879	0.030004023
ENSMMUG00000058002	zinc finger protein 669-like [Source:NCBI gene;Acc:696282]	1.266128656	4.97E-26	6.95E-24
ENSMMUG00000003013	NIPA magnesium transporter 1 [Source:VGNC Symbol;Acc:VGNC:75196]	-3.571898805	0.012482544	0.038914593
ENSMMUG00000052887		-1.968758586	0.004668091	0.016915735
ENSMMUG00000062676		-0.991585758	0.000393021	0.001997671
ENSMMUG00000054713		-0.98677208	0.002578114	0.010200881
ENSMMUG00000065031		-1.359976842	4.89E-10	9.16E-09
ENSMMUG00000023139	small nuclear ribonucleoprotein polypeptide N [Source:VGNC Symbol;Acc:VGNC:78437]	2.712590626	4.85E-09	7.47E-08
ENSMMUG00000051503	nuclear pore associated protein 1 [Source:NCBI gene;Acc:719891]	2.807570107	1.31E-05	9.80E-05
ENSMMUG00000019483	putative golgin subfamily A member 8D [Source:NCBI gene;Acc:100430266]	-1.959422724	0.013957071	0.042696536
ENSMMUG00000021656	golgin subfamily A member 8S [Source:NCBI gene;Acc:722977]	-2.82820888	0.003107343	0.011988175
ENSMMUG00000054315		-3.524374917	1.99E-09	3.29E-08
ENSMMUG00000055117		-2.843431488	2.34E-08	3.17E-07
ENSMMUG00000063268		-1.744729634	4.15E-06	3.46E-05
ENSMMUG00000058136	Metazoan signal recognition particle RNA [Source:RFAM;Acc:RF00017]	-2.395667908	0.015688749	0.046966092
ENSMMUG00000009430	transient receptor potential cation channel subfamily M member 1 [Source:VGNC Symbol;Acc:VGNC:79964]	0.527812003	1.47E-06	1.36E-05
ENSMMUG00000005270	ryanodine receptor 3 [Source:NCBI gene;Acc:694996]	0.500860139	1.19E-06	1.13E-05
ENSMMUG00000014227	cholinergic receptor muscarinic 5 [Source:VGNC Symbol;Acc:VGNC:71044]	3.022022669	0.014332247	0.043660493
ENSMMUG00000002337	aquarius intron-binding spliceosomal factor [Source:VGNC Symbol;Acc:VGNC:69925]	-0.676038465	0.013515226	0.041563538
ENSMMUG00000046486	zinc finger protein 770 [Source:NCBI gene;Acc:697740]	-0.541358688	0.015507099	0.046540199
ENSMMUG00000063659		-1.345448109	0.000169535	0.000954136
ENSMMUG00000051611		-1.472364776	0.014919872	0.045063407
ENSMMUG00000049271		1.544829406	0.009824201	0.031970989
ENSMMUG00000060667		1.396875625	0.000494959	0.00244902
ENSMMUG00000018332	family with sequence similarity 98 member B [Source:VGNC Symbol;Acc:VGNC:72583]	-1.235237733	1.18E-08	1.71E-07
ENSMMUG00000006035	thrombospondin 1 [Source:VGNC Symbol;Acc:VGNC:78344]	-2.13493814	0.004357618	0.015960365
ENSMMUG00000016686	BUB1 mitotic checkpoint serine/threonine kinase B [Source:VGNC Symbol;Acc:VGNC:70293]	0.524526302	0.007567103	0.025621655

ENSMMUG00000020534	p21 (RAC1) activated kinase 6 [Source:NCBI gene;Acc:701589]	2.487448708	1.96E-28	3.84E-26
ENSMMUG00000020539	coiled-coil domain containing 9B [Source:VGNC Symbol;Acc:VGNC:70920]	-0.659642767	0.00129517	0.00565402
ENSMMUG00000012408	dispatched RND transporter family member 2 [Source:VGNC Symbol;Acc:VGNC:71676]	-0.770299045	0.000120303	0.000704045
ENSMMUG00000058981	kinetochore localized astrin (SPAG5) binding protein [Source:NCBI gene;Acc:702753]	-0.651693969	0.000526482	0.002586506
ENSMMUG00000043229	carbohydrate sulfotransferase 14 [Source:NCBI gene;Acc:703115]	-1.160476465	0.001026737	0.004626684
ENSMMUG00000048910	kinetochore scaffold 1 [Source:VGNC Symbol;Acc:VGNC:84366]	-0.626820363	0.001423892	0.00613489
ENSMMUG00000000306	regulator of microtubule dynamics 3 [Source:VGNC Symbol;Acc:VGNC:76821]	-0.792897404	0.003528221	0.013360186
ENSMMUG00000013160	DnaJ heat shock protein family (Hsp40) member C17 [Source:VGNC Symbol;Acc:VGNC:84335]	1.590280261	0.000111687	0.000657324
ENSMMUG00000014541	delta like canonical Notch ligand 4 [Source:VGNC Symbol;Acc:VGNC:71926]	-0.418885973	0.007867736	0.026432466
ENSMMUG00000010168	MAX dimerization protein MGA [Source:VGNC Symbol;Acc:VGNC:84390]	-0.333879153	0.011507766	0.036351238
ENSMMUG00000015449	phospholipase A2 group IVF [Source:VGNC Symbol;Acc:VGNC:76123]	0.572897663	9.96E-05	0.000594189
ENSMMUG00000001532	glucosidase alpha, neutral C [Source:NCBI gene;Acc:708126]	0.746078611	7.37E-09	1.11E-07
ENSMMUG00000001534	calpain 3 [Source:VGNC Symbol;Acc:VGNC:70533]	0.67713687	0.000284845	0.001511236
ENSMMUG00000001547	zinc finger protein 106 [Source:VGNC Symbol;Acc:VGNC:78831]	1.377892536	2.62E-27	4.37E-25
ENSMMUG00000064341	U6 spliceosomal RNA [Source:RFAM;Acc:RF00026]	3.398004058	0.00053069	0.00260278
ENSMMUG00000008093	StAR related lipid transfer domain containing 9 [Source:NCBI gene;Acc:708953]	-0.967989629	6.34E-14	2.24E-12
ENSMMUG00000015123	codanin 1 [Source:VGNC Symbol;Acc:VGNC:70788]	-0.638960924	0.000525202	0.002581089
ENSMMUG00000010036	tau tubulin kinase 2 [Source:VGNC Symbol;Acc:VGNC:81598]	-0.52538397	0.001044276	0.004696978
ENSMMUG00000006498	ubiquitin protein ligase E3 component n-recognin 1 [Source:VGNC Symbol;Acc:VGNC:79051]	0.366648101	0.000152844	0.000871331
ENSMMUG00000022256	transglutaminase 5 [Source:VGNC Symbol;Acc:VGNC:78341]	-0.695293193	0.004553448	0.016552505
ENSMMUG00000022251	transglutaminase 7 [Source:VGNC Symbol;Acc:VGNC:79880]	-0.466931788	1.67E-05	0.000120801
ENSMMUG00000055498	leucine carboxyl methyltransferase 2 [Source:NCBI gene;Acc:710047]	0.767781201	0.001630129	0.006918886
ENSMMUG00000017067	adenosine deaminase like [Source:VGNC Symbol;Acc:VGNC:69555]	-0.642501847	0.000347901	0.00179469
ENSMMUG00000017070	zinc finger and SCAN domain containing 29 [Source:VGNC Symbol;Acc:VGNC:79633]	-1.144498855	2.12E-09	3.49E-08
ENSMMUG00000017076	tubulin gamma complex associated protein 4 [Source:VGNC Symbol;Acc:VGNC:79045]	-1.277822256	9.19E-09	1.35E-07
ENSMMUG00000007255	microtubule associated protein 1A [Source:VGNC Symbol;Acc:VGNC:74489]	-1.34318789	0.000583526	0.00282946
ENSMMUG00000007860	diphosphoinositol pentakisphosphate kinase 1 [Source:VGNC Symbol;Acc:VGNC:76127]	-0.665273975	0.00944231	0.030924165
ENSMMUG00000007870	creatine kinase U-type, mitochondrial [Source:NCBI gene;Acc:114669858]	-0.446207456	0.000291525	0.001536584
ENSMMUG00000021840	stereocilin [Source:NCBI gene;Acc:106996176]	0.647198924	4.97E-06	4.07E-05
ENSMMUG00000016742	CTD small phosphatase like 2 [Source:VGNC Symbol;Acc:VGNC:71593]	0.635621596	3.66E-07	3.93E-06
ENSMMUG00000014288	eukaryotic translation initiation factor 3 subunit J [Source:VGNC Symbol;Acc:VGNC:72188]	0.481669159	0.002671167	0.010494798
ENSMMUG00000014291	SPG11 vesicle trafficking associated, spatacsin [Source:VGNC Symbol;Acc:VGNC:77856]	1.373209504	1.11E-10	2.31E-09
ENSMMUG00000001284	solute carrier family 28 member 2 [Source:VGNC Symbol;Acc:VGNC:77445]	-0.507828894	0.011523667	0.036385652
ENSMMUG00000005856	glycine amidinotransferase [Source:VGNC Symbol;Acc:VGNC:72892]	-1.866373593	5.23E-16	2.56E-14
ENSMMUG00000049265	Metazoan signal recognition particle RNA [Source:RFAM;Acc:RF00017]	1.706834545	1.27E-05	9.55E-05
ENSMMUG00000062254		1.718411721	3.11E-06	2.67E-05

ENSMMUG00000016896	solute carrier family 12 member 1 [Source:VGNC Symbol;Acc:VGNC:77385]	-1.039831711	4.35E-07	4.60E-06
ENSMMUG00000009838	COP9 signalosome subunit 2 [Source:VGNC Symbol;Acc:VGNC:71376]	-0.632613344	0.002395282	0.009592344
ENSMMUG00000009839	galactokinase 2 [Source:VGNC Symbol;Acc:VGNC:72870]	-1.013964277	0.000119754	0.000701115
ENSMMUG00000009840	family with sequence similarity 227 member B [Source:VGNC Symbol;Acc:VGNC:72370]	1.138253164	3.08E-06	2.65E-05
ENSMMUG00000013496	DTW domain containing 1 [Source:VGNC Symbol;Acc:VGNC:72017]	3.115338403	0.00147491	0.006325479
ENSMMUG00000056522		1.54073356	4.01E-05	0.000267296
ENSMMUG00000050608		1.433649437	6.47E-09	9.81E-08
ENSMMUG00000003684	ATPase phospholipid transporting 8B4 (putative) [Source:VGNC Symbol;Acc:VGNC:84311]	1.513395115	1.28E-11	3.13E-10
ENSMMUG00000057283		1.49646851	1.37E-05	0.000101616
ENSMMUG00000002879	GA binding protein transcription factor subunit beta 1 [Source:VGNC Symbol;Acc:VGNC:72845]	-0.605575312	8.16E-05	0.000499651
ENSMMUG00000060278	Metazoan signal recognition particle RNA [Source:RFAM;Acc:RF00017]	-2.076977741	6.38E-06	5.09E-05
ENSMMUG00000008575	transient receptor potential cation channel subfamily M member 7 [Source:VGNC Symbol;Acc:VGNC:79106]	-0.293251381	0.000455416	0.002283676
ENSMMUG00000000754	Dmx like 2 [Source:VGNC Symbol;Acc:VGNC:71936]	2.313545335	1.80E-13	5.84E-12
ENSMMUG00000017432	tropomodulin 2 [Source:VGNC Symbol;Acc:VGNC:78571]	-1.107192375	4.91E-10	9.19E-09
ENSMMUG00000002345	tropomodulin 3 [Source:VGNC Symbol;Acc:VGNC:78572]	1.017082074	7.22E-34	2.19E-31
ENSMMUG00000017374	G protein subunit beta 5 [Source:VGNC Symbol;Acc:VGNC:73097]	-3.209503131	1.22E-06	1.16E-05
ENSMMUG00000007504	myosin VC [Source:VGNC Symbol;Acc:VGNC:75117]	-2.188436366	0.000125235	0.000729969
ENSMMUG00000007509	myosin VA [Source:VGNC Symbol;Acc:VGNC:75116]	-0.654741645	0.000235643	0.001276283
ENSMMUG00000048644		-1.022119484	0.013905553	0.042556848
ENSMMUG00000001423	family with sequence similarity 214 member A [Source:VGNC Symbol;Acc:VGNC:72354]	-0.447557838	0.000517851	0.002550999
ENSMMUG00000010798	cell cycle progression 1 [Source:VGNC Symbol;Acc:VGNC:70844]	0.441962451	0.003714035	0.013961967
ENSMMUG00000010797	phosphatidylinositol glycan anchor biosynthesis class B [Source:VGNC Symbol;Acc:VGNC:75985]	0.577314322	0.000467312	0.002332871
ENSMMUG00000029638	chromosome 7 C15orf65 homolog [Source:VGNC Symbol;Acc:VGNC:70689]	0.914801509	0.001061418	0.004759359
ENSMMUG00000000863	dynein axonemal assembly factor 4 [Source:VGNC Symbol;Acc:VGNC:71881]	1.415564555	2.09E-14	8.15E-13
ENSMMUG00000008112	NEDD4 E3 ubiquitin protein ligase [Source:VGNC Symbol;Acc:VGNC:75302]	-0.652232947	0.004167414	0.015352684
ENSMMUG00000008718	zinc finger protein 280D [Source:NCBI gene;Acc:699509]	-1.635383974	5.48E-16	2.65E-14
ENSMMUG00000004091	transcription factor 12 [Source:VGNC Symbol;Acc:VGNC:78283]	-0.860888765	1.55E-12	4.37E-11
ENSMMUG00000063795		-1.426372783	0.00422751	0.015550435
ENSMMUG00000012690	myocardial zonula adherens protein [Source:NCBI gene;Acc:700247]	-3.631297568	5.46E-06	4.43E-05
ENSMMUG00000018426	glucosaminyl (N-acetyl) transferase 3, mucin type [Source:VGNC Symbol;Acc:VGNC:84342]	-2.40681666	0.010746669	0.034395491
ENSMMUG00000020348	BCL2 interacting protein 2 [Source:VGNC Symbol;Acc:VGNC:70272]	-2.296039185	1.59E-08	2.22E-07
ENSMMUG00000049749		-1.168520417	6.12E-09	9.32E-08
ENSMMUG00000060717	C2 calcium dependent domain containing 4A [Source:NCBI gene;Acc:704354]	0.464563501	0.000539809	0.002641267
ENSMMUG00000013460	RAB8B, member RAS oncogene family [Source:VGNC Symbol;Acc:VGNC:81550]	1.160566678	3.96E-14	1.49E-12
ENSMMUG00000059755	aph-1 homolog B, gamma-secretase subunit [Source:NCBI gene;Acc:705718]	0.928969742	0.005420194	0.019264881
ENSMMUG00000012117	HECT and RLD domain containing E3 ubiquitin protein ligase family member 1 [Source:VGNC Symbol;Acc:VGNC:73364]	0.546922846	4.16E-10	7.90E-09

ENSMMUG00000049375		0.592464551	1.22E-05	9.16E-05
ENSMMUG00000018177	death associated protein kinase 2 [Source:VGNC Symbol;Acc:VGNC:71733]	0.736696735	0.000677014	0.00322998
ENSMMUG00000013270	RNA binding protein, mRNA processing factor 2 [Source:VGNC Symbol;Acc:VGNC:76511]	1.253582402	0.011711005	0.03687305
ENSMMUG00000053704	Metazoan signal recognition particle RNA [Source:RFAM;Acc:RF00017]	-2.611613688	0.016016955	0.047751944
ENSMMUG00000010949	SPG21 abhydrolase domain containing, maspardin [Source:VGNC Symbol;Acc:VGNC:77970]	0.886490805	3.87E-07	4.12E-06
ENSMMUG00000004028	kelch repeat and BTB domain containing 13 [Source:VGNC Symbol;Acc:VGNC:73953]	1.758248596	7.00E-06	5.55E-05
ENSMMUG00000006297	programmed cell death 7 [Source:NCBI gene;Acc:708758]	0.407337189	0.002019627	0.008338348
ENSMMUG00000021788	immunoglobulin superfamily DCC subclass member 4 [Source:VGNC Symbol;Acc:VGNC:73388]	-1.165883528	0.003123571	0.012034815
ENSMMUG00000015450	3-hydroxyacyl-CoA dehydratase 3 [Source:VGNC Symbol;Acc:VGNC:73225]	-0.401118603	0.016833209	0.049756885
ENSMMUG00000022056	DENN domain containing 4A [Source:VGNC Symbol;Acc:VGNC:71697]	-1.006167217	6.22E-10	1.14E-08
ENSMMUG00000054184	Metazoan signal recognition particle RNA [Source:RFAM;Acc:RF00017]	0.769126099	3.69E-05	0.000247181
ENSMMUG00000035748	U6 spliceosomal RNA [Source:RFAM;Acc:RF00026]	0.962068081	0.00659169	0.022685464
ENSMMUG00000008006	RAB11A, member RAS oncogene family [Source:VGNC Symbol;Acc:VGNC:81517]	0.977250631	2.00E-11	4.75E-10
ENSMMUG00000008007	multiple EGF like domains 11 [Source:VGNC Symbol;Acc:VGNC:84376]	1.094626391	1.19E-09	2.06E-08
ENSMMUG00000052828		0.850120816	0.002573457	0.010190779
ENSMMUG00000003693	DIS3 like exosome 3'-5' exoribonuclease [Source:VGNC Symbol;Acc:VGNC:71796]	-4.141151222	6.74E-05	0.00042037
ENSMMUG00000041415	alpha and gamma adaptin binding protein [Source:NCBI gene;Acc:711436]	-1.239822611	0.010250694	0.033099587
ENSMMUG00000015458	IQ motif containing H [Source:VGNC Symbol;Acc:VGNC:73655]	-0.526228596	0.005306009	0.018909892
ENSMMUG00000004026	integrin subunit alpha 11 [Source:VGNC Symbol;Acc:VGNC:73774]	0.480184778	4.74E-05	0.000309009
ENSMMUG00000006230	NADPH oxidase 5 [Source:VGNC Symbol;Acc:VGNC:82198]	0.332082321	0.011365903	0.036028376
ENSMMUG00000007570	uveal autoantigen with coiled-coil domains and ankyrin repeats [Source:VGNC Symbol;Acc:VGNC:78995]	-0.787858538	0.001000496	0.004528086
ENSMMUG00000053647		-0.395757925	0.010993831	0.035032293
ENSMMUG00000059258		0.56699681	0.006791631	0.023274383
ENSMMUG00000013685	Bardet-Biedl syndrome 4 [Source:NCBI gene;Acc:704508]	0.523079793	0.004639656	0.016827657
ENSMMUG00000011309	ADP dependent glucokinase [Source:VGNC Symbol;Acc:VGNC:69692]	0.44702587	5.13E-05	0.000332168
ENSMMUG00000057906		0.874344453	2.39E-15	1.06E-13
ENSMMUG00000059627		0.663694276	0.007728824	0.026061422
ENSMMUG00000063495		1.099194224	4.32E-15	1.83E-13
ENSMMUG00000056086		1.040296072	0.000107715	0.000637246
ENSMMUG00000001835	immunoglobulin superfamily containing leucine rich repeat 2 [Source:VGNC Symbol;Acc:VGNC:73682]	0.566193745	0.005659163	0.019992043
ENSMMUG00000001568	AT-rich interaction domain 3B [Source:VGNC Symbol;Acc:VGNC:69827]	1.138551751	4.12E-08	5.33E-07
ENSMMUG00000022481	cytochrome P450, family 1, subfamily A, polypeptide 1 [Source:NCBI gene;Acc:678694]	0.530448505	0.002186679	0.008919123
ENSMMUG00000015267	C-terminal Src kinase [Source:VGNC Symbol;Acc:VGNC:81380]	0.459666268	2.06E-07	2.34E-06
ENSMMUG00000008529	chromosome 7 C15orf39 homolog [Source:VGNC Symbol;Acc:VGNC:70620]	1.580245155	8.28E-06	6.43E-05
ENSMMUG00000061079	golgin subfamily A member 6C [Source:NCBI gene;Acc:715898]	1.560473933	1.15E-24	1.34E-22
ENSMMUG00000016104	chondroitin sulfate proteoglycan 4 [Source:VGNC Symbol;Acc:VGNC:71525]	0.779691104	3.58E-06	3.03E-05
ENSMMUG00000053989		1.004436424	2.14E-13	6.82E-12
ENSMMUG00000058835		1.1009175	3.04E-05	0.000207675
ENSMMUG00000050124	transmembrane protein 266 [Source:NCBI gene;Acc:707482]	0.393133593	0.010355002	0.03336969
ENSMMUG00000010171	proline-serine-threonine phosphatase interacting protein 1 [Source:VGNC Symbol;Acc:VGNC:76459]	-0.402465433	0.0150571	0.045428141
ENSMMUG00000042378	pseudopodium enriched atypical kinase 1 [Source:VGNC Symbol;Acc:VGNC:84419]	0.467218091	0.000297008	0.001561519
ENSMMUG00000054259		0.709327815	0.015797742	0.047204886

ENSMMUG00000058749	golgin subfamily A member 6C [Source:NCBI gene;Acc:716687]	-3.016576819	0.00670856	0.023054927
ENSMMUG00000045680	iron responsive element binding protein 2 [Source:VGNC Symbol;Acc:VGNC:84360]	-0.928141694	0.005989682	0.020936941
ENSMMUG00000059973		-0.40702873	0.007118415	0.024278602
ENSMMUG00000023736	cholinergic receptor nicotinic alpha 3 subunit [Source:VGNC Symbol;Acc:VGNC:71047]	0.418463147	0.002554963	0.010136873
ENSMMUG00000007541	cholinergic receptor nicotinic beta 4 subunit [Source:NCBI gene;Acc:106996685]	0.379955408	0.006401804	0.022139313
ENSMMUG00000056992	zinc finger AN1-type containing 6 [Source:NCBI gene;Acc:717543]	1.03748854	0.001532145	0.00653928
ENSMMUG00000009361	fumarylacetoacetate hydrolase [Source:NCBI gene;Acc:712716]	1.785672291	1.75E-06	1.58E-05
ENSMMUG00000009409	mesoderm development LRP chaperone [Source:VGNC Symbol;Acc:VGNC:74507]	-1.045438475	0.001278084	0.005587821
ENSMMUG00000011564	transmembrane channel like 3 [Source:VGNC Symbol;Acc:VGNC:79360]	4.142617541	1.03E-05	7.83E-05
ENSMMUG00000057295		0.39744596	0.007964856	0.026709367
ENSMMUG00000003002		-0.596107353	0.003094527	0.011941901
ENSMMUG00000009087	HDGF like 3 [Source:VGNC Symbol;Acc:VGNC:84346]	-1.243732471	3.31E-08	4.36E-07
ENSMMUG00000009084	transmembrane 6 superfamily member 1 [Source:VGNC Symbol;Acc:VGNC:78566]	-1.145207614	2.77E-07	3.05E-06
ENSMMUG00000062992	U7 small nuclear RNA [Source:RFAM;Acc:RF00066]	-1.49694156	0.00343347	0.013066063
ENSMMUG00000062559		-1.089254357	0.004748139	0.017170074
ENSMMUG00000011328	cytoplasmic polyadenylation element binding protein 1 [Source:NCBI gene;Acc:694357]	1.086180122	1.48E-05	0.0001087
ENSMMUG00000056767		0.963185246	0.002596832	0.010260981
ENSMMUG00000019838	phosphodiesterase 8A [Source:VGNC Symbol;Acc:VGNC:75924]	-0.681753143	0.000310841	0.001624262
ENSMMUG00000056737		-1.153745099	0.000307317	0.001609318
ENSMMUG00000049563		-1.318603755	1.53E-07	1.79E-06
ENSMMUG00000016772	solute carrier family 28 member 1 [Source:VGNC Symbol;Acc:VGNC:77444]	0.777007868	2.43E-09	3.97E-08
ENSMMUG00000010432	zinc finger protein 592 [Source:VGNC Symbol;Acc:VGNC:79762]	0.302248121	0.010409657	0.033522908
ENSMMUG00000057531	mitochondrial ribosomal protein S11 [Source:NCBI gene;Acc:697743]	-0.559308871	0.012966245	0.040138481
ENSMMUG00000043001	milk fat globule EGF and factor V/VIII domain containing [Source:NCBI gene;Acc:703501]	-1.436266007	0.000192259	0.001067559
ENSMMUG00000014414	abhydrolase domain containing 2 [Source:VGNC Symbol;Acc:VGNC:69463]	0.807318494	0.011790925	0.03706047
ENSMMUG00000011155	FA complementation group I [Source:VGNC Symbol;Acc:VGNC:72575]	1.127485503	6.20E-10	1.14E-08
ENSMMUG00000011160	DNA polymerase gamma, catalytic subunit [Source:VGNC Symbol;Acc:VGNC:76205]	0.792455974	0.002138591	0.00875738
ENSMMUG00000022678	Rh family C glycoprotein [Source:VGNC Symbol;Acc:VGNC:84427]	-0.965052744	8.18E-05	0.000500886
ENSMMUG00000015394	WD repeat domain 93 [Source:VGNC Symbol;Acc:VGNC:79642]	1.04202138	6.68E-26	9.16E-24
ENSMMUG00000010586	mesoderm posterior bHLH transcription factor 1 [Source:VGNC Symbol;Acc:VGNC:74618]	1.33763215	0.000385902	0.001965946
ENSMMUG00000003584	mesoderm posterior bHLH transcription factor 2 [Source:VGNC Symbol;Acc:VGNC:74619]	0.976907323	2.45E-18	1.52E-16
ENSMMUG00000014983	alanyl aminopeptidase, membrane [Source:VGNC Symbol;Acc:VGNC:69943]	0.986001304	1.10E-18	7.26E-17
ENSMMUG00000029507	adaptor related protein complex 3 subunit sigma 2 [Source:NCBI gene;Acc:701217]	0.942503929	6.88E-25	8.40E-23
ENSMMUG00000023816	semaphorin 4B [Source:VGNC Symbol;Acc:VGNC:77165]	1.947205398	4.72E-24	4.97E-22
ENSMMUG00000004376	calcium and integrin binding 1 [Source:VGNC Symbol;Acc:VGNC:71189]	2.116071785	5.17E-21	4.35E-19
ENSMMUG00000050794	zinc finger protein 774 [Source:NCBI gene;Acc:702985]	-1.383469727	0.000228878	0.001244745
ENSMMUG00000019526	BLM RecQ like helicase [Source:VGNC Symbol;Acc:VGNC:70254]	2.59819923	2.63E-13	8.29E-12
ENSMMUG00000062217	HD domain containing 3 [Source:NCBI gene;Acc:715621]	2.665945734	1.70E-17	9.67E-16
ENSMMUG00000005106	unc-45 myosin chaperone A [Source:VGNC Symbol;Acc:VGNC:79858]	3.356610186	1.18E-05	8.93E-05
ENSMMUG00000005108	RCC1 domain containing 1 [Source:VGNC Symbol;Acc:VGNC:76876]	2.890766512	3.70E-14	1.40E-12

ENSMMUG00000007534	protein regulator of cytokinesis 1 [Source:NCBI gene;Acc:710182]	2.477647752	4.21E-06	3.50E-05
ENSMMUG00000008399	synaptic vesicle glycoprotein 2B [Source:VGNC Symbol;Acc:VGNC:78140]	0.445849208	0.006475215	0.022332195
ENSMMUG00000063322		0.934098641	2.05E-06	1.83E-05
ENSMMUG00000005220	synemin [Source:VGNC Symbol;Acc:VGNC:78201]	0.735121566	6.93E-10	1.26E-08
ENSMMUG00000005224	tetratricopeptide repeat domain 23 [Source:VGNC Symbol;Acc:VGNC:79534]	0.604646032	1.43E-05	0.000105789
ENSMMUG00000065319		3.513369967	4.34E-05	0.000287326
ENSMMUG00000060063		1.521855628	1.29E-12	3.69E-11
ENSMMUG00000006659	aldehyde dehydrogenase 1 family member A3 [Source:VGNC Symbol;Acc:VGNC:81353]	2.011217904	1.96E-07	2.23E-06
ENSMMUG00000060984	olfactory receptor, family 4, subfamily K, member 13 [Source:NCBI gene;Acc:700920]	0.519844534	0.009935517	0.032253821
ENSMMUG00000029455	olfactory receptor 4Q2 [Source:NCBI gene;Acc:114679271]	0.669092643	0.000254313	0.001364689
ENSMMUG00000045269		2.921010532	0.001723194	0.007260891
ENSMMUG00000058162		2.394677091	0.002661374	0.010470472
ENSMMUG00000016255	telomerase associated protein 1 [Source:VGNC Symbol;Acc:VGNC:78310]	0.99795292	5.15E-05	0.000333053
ENSMMUG00000048039	apurinic/apyrimidinic endodeoxyribonuclease 1 [Source:VGNC Symbol;Acc:VGNC:84305]	1.18082956	0.016893832	0.04985498
ENSMMUG00000019126	purine nucleoside phosphorylase [Source:NCBI gene;Acc:704183]	1.165003638	0.001468017	0.00629887
ENSMMUG00000020747	NDRG family member 2 [Source:VGNC Symbol;Acc:VGNC:74953]	-2.541764494	0.00267746	0.01051367
ENSMMUG00000050445	ribonuclease A family member 8 [Source:NCBI gene;Acc:716333]	-2.776054255	0.004807121	0.017353144
ENSMMUG00000006457	Rho guanine nucleotide exchange factor 40 [Source:VGNC Symbol;Acc:VGNC:69907]	-2.948939343	1.14E-06	1.08E-05
ENSMMUG00000014809	heterogeneous nuclear ribonucleoprotein C [Source:VGNC Symbol;Acc:VGNC:84348]	-2.245201623	0.000759576	0.00356416
ENSMMUG00000021552	RPGR interacting protein 1 [Source:VGNC Symbol;Acc:VGNC:77007]	-2.618931013	4.25E-10	8.04E-09
ENSMMUG00000055229		-2.913457406	3.16E-21	2.71E-19
ENSMMUG00000015000	SPT16 homolog, facilitates chromatin remodeling subunit [Source:VGNC Symbol;Acc:VGNC:78054]	-2.149336134	1.50E-13	4.94E-12
ENSMMUG00000015002	chromodomain helicase DNA binding protein 8 [Source:VGNC Symbol;Acc:VGNC:71060]	-2.368462645	3.71E-08	4.83E-07
ENSMMUG00000015004	TOX high mobility group box family member 4 [Source:NCBI gene;Acc:706950]	-1.68597947	0.000184884	0.001031732
ENSMMUG00000057062		-1.202620844	0.004540596	0.016509915
ENSMMUG00000062974		-1.493355768	3.89E-09	6.08E-08
ENSMMUG00000040154	T cell receptor alpha variable 9-2 [Source:HGNC Symbol;Acc:HGNC:12154]	-1.443798799	4.45E-06	3.69E-05
ENSMMUG00000038070	T cell receptor alpha variable 12-3 [Source:HGNC Symbol;Acc:HGNC:12107]	-1.515377199	1.14E-11	2.80E-10
ENSMMUG00000054116	T cell receptor alpha variable 8-6 [Source:HGNC Symbol;Acc:HGNC:12151]	-1.645820995	1.24E-11	3.02E-10
ENSMMUG00000056910		-1.86692489	1.12E-10	2.33E-09
ENSMMUG00000063215	T cell receptor alpha variable 29/delta variable 5 (gene/pseudogene) [Source:HGNC Symbol;Acc:HGNC:12127]	0.97901706	0.00209837	0.008614517
ENSMMUG00000009441	abhydrolase domain containing 4 [Source:VGNC Symbol;Acc:VGNC:84296]	2.22323252	7.02E-06	5.56E-05
ENSMMUG00000000413	solute carrier family 7 member 7 [Source:VGNC Symbol;Acc:VGNC:77631]	-0.718515852	0.001000443	0.004528086
ENSMMUG00000014750	embryonal Fyn-associated substrate [Source:VGNC Symbol;Acc:VGNC:72056]	-0.810344413	0.016883913	0.049835826
ENSMMUG00000014762	myosin heavy chain 6 [Source:NCBI gene;Acc:713471]	-1.152479717	7.39E-18	4.33E-16
ENSMMUG00000029420		1.781388733	2.28E-07	2.56E-06
ENSMMUG00000014766	neuroguidin [Source:VGNC Symbol;Acc:VGNC:75220]	1.318496303	8.57E-12	2.16E-10
ENSMMUG00000045970	thiamine triphosphatase [Source:NCBI gene;Acc:714403]	1.210087368	5.99E-17	3.20E-15

ENSMMUG00000014771	zinc finger homeobox 2 [Source:NCBI gene;Acc:100429448]	1.398174852	1.58E-20	1.28E-18
ENSMMUG00000004144	adaptor related protein complex 1 subunit gamma 2 [Source:VGNC Symbol;Acc:VGNC:69959]	0.364746681	0.013334009	0.041110602
ENSMMUG00000059196		0.507276181	3.42E-07	3.70E-06
ENSMMUG00000012462	proteasome activator subunit 1 [Source:VGNC Symbol;Acc:VGNC:76455]	-1.77266006	9.38E-07	9.09E-06
ENSMMUG00000012473	proteasome activator subunit 2 [Source:VGNC Symbol;Acc:VGNC:76456]	-2.173750848	3.56E-05	0.000239689
ENSMMUG00000012474	ring finger protein 31 [Source:NCBI gene;Acc:715239]	-2.228681302	1.48E-05	0.000108583
ENSMMUG00000040817	interferon regulatory factor 9 [Source:VGNC Symbol;Acc:VGNC:84361]	-2.529526773	2.21E-05	0.000156044
ENSMMUG00000012480	REC8 meiotic recombination protein [Source:VGNC Symbol;Acc:VGNC:76746]	-2.542172539	1.23E-11	3.01E-10
ENSMMUG00000012481	importin 4 [Source:VGNC Symbol;Acc:VGNC:73630]	-2.057808506	6.61E-10	1.20E-08
ENSMMUG00000029407	TERF1 interacting nuclear factor 2 [Source:VGNC Symbol;Acc:VGNC:78359]	-2.477601688	0.001517441	0.006488752
ENSMMUG00000012452	transglutaminase 1 [Source:NCBI gene;Acc:715854]	-2.471282446	0.00515482	0.018434347
ENSMMUG00000010049	NOP9 nucleolar protein [Source:VGNC Symbol;Acc:VGNC:75216]	-2.437274349	0.000604507	0.002920476
ENSMMUG00000020698	adenylate cyclase 4 [Source:NCBI gene;Acc:714684]	-2.746741064	0.012186921	0.038132
ENSMMUG00000019692	sec1 family domain containing 1 [Source:VGNC Symbol;Acc:VGNC:77065]	0.522094588	0.006468121	0.022313024
ENSMMUG00000011515	striatin 3 [Source:VGNC Symbol;Acc:VGNC:78127]	-1.328478066	0.000321102	0.001671872
ENSMMUG00000003120	HECT domain E3 ubiquitin protein ligase 1 [Source:VGNC Symbol;Acc:VGNC:73200]	-0.952319214	2.19E-05	0.000155055
ENSMMUG00000056906		1.295278723	2.26E-11	5.24E-10
ENSMMUG00000012576	sorting nexin 6 [Source:NCBI gene;Acc:693626]	-0.833898349	0.003743223	0.014060785
ENSMMUG00000015073	signal recognition particle 54 [Source:VGNC Symbol;Acc:VGNC:82247]	-0.399216863	0.008302099	0.027693348
ENSMMUG00000003530	protein phosphatase 2 regulatory subunit B''gamma [Source:VGNC Symbol;Acc:VGNC:76279]	-0.368502963	0.013770899	0.042224754
ENSMMUG00000056013	forkhead box A1 [Source:NCBI gene;Acc:697909]	2.38821074	0.000862803	0.003991906
ENSMMUG00000017677	chromosome 7 C14orf28 homolog [Source:VGNC Symbol;Acc:VGNC:70581]	-0.501131966	8.73E-05	0.000529637
ENSMMUG00000003913	DNA polymerase epsilon 2, accessory subunit [Source:VGNC Symbol;Acc:VGNC:76204]	0.627104869	0.001078207	0.00481977
ENSMMUG00000003914	kelch domain containing 1 [Source:VGNC Symbol;Acc:VGNC:74086]	-0.677172115	0.013993975	0.042791422
ENSMMUG00000055903	Metazoan signal recognition particle RNA [Source:RFAM;Acc:RF00017]	-1.423847923	0.014853974	0.044929717
ENSMMUG00000049543		1.027014873	0.00058736	0.002845201
ENSMMUG00000059134		1.393925206	0.000356601	0.001831769
ENSMMUG00000007460	valosin containing protein lysine methyltransferase [Source:VGNC Symbol;Acc:VGNC:81602]	-1.265199601	0.007393376	0.025146818
ENSMMUG00000009577	atlastin GTPase 1 [Source:VGNC Symbol;Acc:VGNC:70049]	0.574589131	1.83E-05	0.000131154
ENSMMUG00000014658	ninein [Source:VGNC Symbol;Acc:VGNC:75177]	2.459027971	3.91E-11	8.78E-10
ENSMMUG00000056844		1.499242126	0.00102644	0.004626684
ENSMMUG00000018274	FERM domain containing 6 [Source:VGNC Symbol;Acc:VGNC:72747]	0.585403576	0.002164614	0.008851473
ENSMMUG00000018200	RNA transcription, translation and transport factor [Source:VGNC Symbol;Acc:VGNC:77087]	2.109144318	0.001180497	0.005217658
ENSMMUG00000000024	prostaglandin D2 receptor [Source:VGNC Symbol;Acc:VGNC:76467]	1.123129079	5.58E-05	0.000357149
ENSMMUG00000000023	prostaglandin E receptor 2 [Source:VGNC Symbol;Acc:VGNC:76468]	1.248575513	1.49E-06	1.38E-05
ENSMMUG00000006997	thioredoxin domain containing 16 [Source:VGNC Symbol;Acc:VGNC:79214]	1.784612227	2.37E-28	4.59E-26

ENSMMUG00000010689	fermitin family member 2 [Source:VGNC Symbol;Acc:VGNC:82144]	-0.860321375	1.44E-07	1.69E-06
ENSMMUG00000052273	glia maturation factor beta [Source:NCBI gene;Acc:696440]	1.144214193	0.005666892	0.020014482
ENSMMUG00000010315	sterile alpha motif domain containing 4A [Source:VGNC Symbol;Acc:VGNC:77085]	1.088454338	0.014466702	0.04394117
ENSMMUG00000004084	GTP cyclohydrolase 1 [Source:VGNC Symbol;Acc:VGNC:72900]	-0.912583306	0.000297945	0.001564182
ENSMMUG00000000302	WD repeat and HMG-box DNA binding protein 1 [Source:VGNC Symbol;Acc:VGNC:79376]	1.609334083	1.94E-18	1.23E-16
ENSMMUG00000054112		-0.418158022	0.003368762	0.012846718
ENSMMUG00000003567	DLG associated protein 5 [Source:VGNC Symbol;Acc:VGNC:71850]	-0.422956185	0.01421742	0.043356122
ENSMMUG00000049334		-0.726667259	0.001109509	0.004939951
ENSMMUG00000023041	TATA-box binding protein like 2 [Source:VGNC Symbol;Acc:VGNC:78175]	-0.635746233	0.00544566	0.019331762
ENSMMUG00000061576		1.62579604	0.000100202	0.000596982
ENSMMUG00000053489		1.021599126	3.23E-05	0.000219382
ENSMMUG00000059984	pellino E3 ubiquitin protein ligase family member 2 [Source:NCBI gene;Acc:699169]	1.260611909	0.012406611	0.0387277
ENSMMUG00000003508	actin related protein 10 [Source:VGNC Symbol;Acc:VGNC:69449]	0.40743035	0.001372742	0.005948003
ENSMMUG00000011965	coiled-coil domain containing 175 [Source:VGNC Symbol;Acc:VGNC:84317]	1.476548796	0.000340687	0.001759972
ENSMMUG00000011966	reticulon 1 [Source:NCBI gene;Acc:619184]	1.317418904	0.000251097	0.001348928
ENSMMUG00000004854	leucine rich repeat containing 9 [Source:VGNC Symbol;Acc:VGNC:81443]	1.035148896	6.12E-07	6.22E-06
ENSMMUG00000002540	dehydrogenase/reductase 7 [Source:VGNC Symbol;Acc:VGNC:71690]	0.525020156	1.24E-07	1.47E-06
ENSMMUG00000001640	SIX homeobox 4 [Source:NCBI gene;Acc:703798]	-0.730351859	0.010516679	0.033748557
ENSMMUG00000001642	MNAT1 component of CDK activating kinase [Source:VGNC Symbol;Acc:VGNC:74905]	-0.632066414	0.013600028	0.041802581
ENSMMUG00000051446		-0.651268758	0.009289581	0.030525008
ENSMMUG00000007264	transmembrane protein 30B [Source:VGNC Symbol;Acc:VGNC:79656]	-0.67563671	0.013346926	0.041141701
ENSMMUG00000002260	protein kinase C eta [Source:VGNC Symbol;Acc:VGNC:84424]	1.097006504	1.26E-13	4.21E-12
ENSMMUG00000008203	ras homolog family member J [Source:VGNC Symbol;Acc:VGNC:76885]	-0.542004157	0.007792517	0.02622218
ENSMMUG00000001008	spectrin repeat containing nuclear envelope protein 2 [Source:NCBI gene;Acc:706759]	-0.75181036	0.000372024	0.001900252
ENSMMUG00000001319	methylenetetrahydrofolate dehydrogenase, cyclohydrolase and formyltetrahydrofolate synthetase 1 [Source:VGNC Symbol;Acc:VGNC:84399]	-1.05863189	1.68E-12	4.73E-11
ENSMMUG00000004748	zinc finger and BTB domain containing 1 [Source:VGNC Symbol;Acc:VGNC:79238]	1.059398938	6.30E-09	9.58E-08
ENSMMUG00000056063		2.405707482	1.04E-05	7.91E-05
ENSMMUG00000019172	spectrin beta, erythrocytic [Source:VGNC Symbol;Acc:VGNC:77870]	0.728811888	0.000236661	0.001279889
ENSMMUG00000004339	RAB15, member RAS oncogene family [Source:VGNC Symbol;Acc:VGNC:81519]	0.790452239	1.63E-06	1.49E-05
ENSMMUG00000004341	MYC associated factor X [Source:VGNC Symbol;Acc:VGNC:74667]	4.372113105	0.002956597	0.011491994
ENSMMUG00000053774		-2.152902055	0.002442596	0.009762997
ENSMMUG00000013848	fucosyltransferase 8 [Source:VGNC Symbol;Acc:VGNC:72829]	-1.522085085	2.25E-10	4.42E-09
ENSMMUG00000013907	ribosomal protein S27a [Source:NCBI gene;Acc:718909]	-2.064968834	5.60E-05	0.000357946
ENSMMUG00000006768	coiled-coil domain containing 196 [Source:VGNC Symbol;Acc:VGNC:70828]	-1.474799034	5.57E-05	0.000356839
ENSMMUG00000009520	gephyrin [Source:VGNC Symbol;Acc:VGNC:72948]	-1.470760406	3.81E-06	3.21E-05
ENSMMUG00000021418	ATPase H+ transporting V1 subunit D [Source:VGNC Symbol;Acc:VGNC:70060]	0.780049781	1.23E-05	9.21E-05
ENSMMUG00000015865	arginase 2 [Source:VGNC Symbol;Acc:VGNC:70005]	1.516221631	2.16E-06	1.92E-05
ENSMMUG00000015866	vesicle transport through interaction with t-SNAREs 1B [Source:NCBI gene;Acc:711113]	1.112519901	7.10E-07	7.10E-06

ENSMMUG00000015869	retinol dehydrogenase 12 [Source:VGNC Symbol;Acc:VGNC:76781]	-0.872876828	0.01680176	0.049694239
ENSMMUG00000015871	zinc finger FYVE-type containing 26 [Source:VGNC Symbol;Acc:VGNC:79283]	1.100257045	0.013788968	0.042271241
ENSMMUG00000059398		3.254288714	0.007342005	0.024983785
ENSMMUG00000010767	actinin alpha 1 [Source:VGNC Symbol;Acc:VGNC:69423]	-1.119913547	0.00786486	0.026428911
ENSMMUG00000021572	solute carrier family 39 member 9 [Source:VGNC Symbol;Acc:VGNC:77523]	-0.514403224	0.013988858	0.042784774
ENSMMUG00000003240	pleckstrin homology and coiled-coil domain containing D1 [Source:VGNC Symbol;Acc:VGNC:76080]	0.302343685	0.009318136	0.03058671
ENSMMUG00000055018	sushi domain containing 6 [Source:NCBI gene;Acc:712719]	1.66042169	8.34E-14	2.88E-12
ENSMMUG00000022407	serine and arginine rich splicing factor 5 [Source:NCBI gene;Acc:712867]	1.073804514	3.51E-05	0.000236288
ENSMMUG00000022409	solute carrier family 10 member 1 [Source:VGNC Symbol;Acc:VGNC:77380]	1.356495472	9.18E-15	3.70E-13
ENSMMUG00000045443		1.565947752	0.002422191	0.009692097
ENSMMUG00000052692	disintegrin and metalloproteinase domain-containing protein 20-like [Source:NCBI gene;Acc:114679594]	-0.883840951	0.002204297	0.008980905
ENSMMUG00000019470	pecanex 1 [Source:VGNC Symbol;Acc:VGNC:75655]	-0.490535932	0.00209109	0.00858948
ENSMMUG00000001820	regulator of G protein signaling 6 [Source:VGNC Symbol;Acc:VGNC:76691]	2.980073761	0.016283854	0.048428474
ENSMMUG00000002332	RNA binding motif protein 25 [Source:VGNC Symbol;Acc:VGNC:76521]	-1.779498863	1.88E-07	2.15E-06
ENSMMUG00000010173	presenilin 1 [Source:VGNC Symbol;Acc:VGNC:76435]	1.032175488	5.37E-06	4.36E-05
ENSMMUG00000056580	Metazoan signal recognition particle RNA [Source:RFAM;Acc:RF00017]	2.307536552	0.004640715	0.016827657
ENSMMUG00000000313	HEAT repeat containing 4 [Source:VGNC Symbol;Acc:VGNC:73176]	1.155673585	1.06E-12	3.07E-11
ENSMMUG00000053481		1.421296721	1.36E-08	1.94E-07
ENSMMUG00000049894		1.669957905	1.51E-05	0.000110489
ENSMMUG00000006855	acyl-CoA thioesterase 2 [Source:NCBI gene;Acc:697011]	1.412866575	3.49E-12	9.33E-11
ENSMMUG00000004910	dynein axonemal light chain 1 [Source:VGNC Symbol;Acc:VGNC:71885]	1.190105837	0.000235312	0.00127544
ENSMMUG00000004913	mitotic deacetylase associated SANT domain protein [Source:VGNC Symbol;Acc:VGNC:72073]	0.744549939	1.01E-08	1.47E-07
ENSMMUG00000038996	synapse differentiation inducing 1 like [Source:NCBI gene;Acc:699046]	1.222490654	0.011764569	0.037001631
ENSMMUG00000056376		2.467790905	6.69E-14	2.35E-12
ENSMMUG00000004255	YLP motif containing 1 [Source:VGNC Symbol;Acc:VGNC:79092]	1.313228009	2.55E-05	0.000177334
ENSMMUG00000015166	NIMA related kinase 9 [Source:VGNC Symbol;Acc:VGNC:75209]	-0.900761268	5.90E-18	3.53E-16
ENSMMUG00000015168	transmembrane p24 trafficking protein 10 [Source:NCBI gene;Acc:701522]	-0.981511194	3.29E-05	0.000222722
ENSMMUG00000040100	Fos proto-oncogene, AP-1 transcription factor subunit [Source:VGNC Symbol;Acc:VGNC:84340]	-0.669881067	3.82E-08	4.97E-07
ENSMMUG00000003834	basic leucine zipper ATF-like transcription factor [Source:VGNC Symbol;Acc:VGNC:70229]	1.021286707	3.33E-05	0.00022579
ENSMMUG00000012065	tubulin tyrosine ligase like 5 [Source:NCBI gene;Acc:703116]	1.326980965	2.25E-09	3.70E-08
ENSMMUG00000008792	G-patch domain containing 2 like [Source:VGNC Symbol;Acc:VGNC:72973]	-1.151507474	8.68E-05	0.000526789
ENSMMUG00000016773	estrogen related receptor beta [Source:VGNC Symbol;Acc:VGNC:72434]	0.591111444	0.001449761	0.006231573
ENSMMUG00000057021	CLOCK interacting pacemaker [Source:NCBI gene;Acc:704679]	-1.35906711	3.44E-05	0.000232057
ENSMMUG00000007561	zinc finger DHHC-type palmitoyltransferase 22 [Source:NCBI gene;Acc:706468]	-1.507306768	3.14E-11	7.16E-10
ENSMMUG00000023626	alkB homolog 1, histone H2A dioxygenase [Source:VGNC Symbol;Acc:VGNC:69630]	-1.397269411	0.002050085	0.008440127
ENSMMUG00000019710	centrosomal protein 128 [Source:VGNC Symbol;Acc:VGNC:71062]	-0.950374934	0.000737032	0.003469635
ENSMMUG00000019713	thyroid stimulating hormone receptor [Source:VGNC Symbol;Acc:VGNC:78662]	-0.935627114	0.000731569	0.003449502
ENSMMUG00000022122	general transcription factor IIA subunit 1 [Source:VGNC Symbol;Acc:VGNC:73305]	-1.257736742	0.013479955	0.041472614

ENSMMUG00000022124	stonin 2 [Source:VGNC Symbol;Acc:VGNC:78249]	-1.251950946	0.000665027	0.003178009
ENSMMUG00000010016	SEL1L adaptor subunit of ERAD E3 ubiquitin ligase [Source:VGNC Symbol;Acc:VGNC:77155]	2.038997913	0.013282487	0.040960441
ENSMMUG00000017261	galactosylceramidase [Source:VGNC Symbol;Acc:VGNC:72867]	1.088205236	4.57E-12	1.21E-10
ENSMMUG00000017260	G protein-coupled receptor 65 [Source:VGNC Symbol;Acc:VGNC:73140]	0.889147239	1.76E-05	0.000126344
ENSMMUG00000021011	spermatogenesis associated 7 [Source:VGNC Symbol;Acc:VGNC:77971]	-1.3020927	0.008896732	0.029420334
ENSMMUG00000004603	zinc finger CCCH-type containing 14 [Source:VGNC Symbol;Acc:VGNC:79255]	-0.637186429	6.83E-08	8.48E-07
ENSMMUG00000004606	EMAP like 5 [Source:VGNC Symbol;Acc:VGNC:72067]	-0.987738662	1.39E-10	2.86E-09
ENSMMUG00000004460	tyrosyl-DNA phosphodiesterase 1 [Source:VGNC Symbol;Acc:VGNC:79164]	-0.818514839	0.001726478	0.007270513
ENSMMUG00000008964	D-glutamate cyclase [Source:VGNC Symbol;Acc:VGNC:71685]	1.13209611	0.001410978	0.006086463
ENSMMUG00000008447	fibulin 5 [Source:VGNC Symbol;Acc:VGNC:72622]	-1.022509928	0.000218296	0.001197129
ENSMMUG00000000699	cleavage and polyadenylation specific factor 2 [Source:VGNC Symbol;Acc:VGNC:71473]	0.499809715	0.007804616	0.026250852
ENSMMUG00000009974	inositol-tetrakisphosphate 1-kinase [Source:VGNC Symbol;Acc:VGNC:73802]	1.423436843	0.002805466	0.010977944
ENSMMUG00000044581	modulator of apoptosis 1 [Source:VGNC Symbol;Acc:VGNC:84392]	1.209735847	2.47E-06	2.17E-05
ENSMMUG00000061833	transmembrane protein 251 [Source:NCBI gene;Acc:708131]	1.451317072	7.18E-06	5.68E-05
ENSMMUG00000053729		-0.366713135	0.012253442	0.038290689
ENSMMUG00000052453	serpin family A member 11 [Source:NCBI gene;Acc:701483]	2.36572358	7.83E-05	0.000481511
ENSMMUG00000012356	serpin family A member 9 [Source:VGNC Symbol;Acc:VGNC:77241]	1.780902583	9.71E-07	9.37E-06
ENSMMUG00000020586	serpin family A member 12 [Source:VGNC Symbol;Acc:VGNC:77266]	4.241214081	0.000191012	0.001061442
ENSMMUG00000000681	calmin [Source:VGNC Symbol;Acc:VGNC:71232]	-0.778907476	8.12E-10	1.45E-08
ENSMMUG00000003658	autophagy related 2B [Source:VGNC Symbol;Acc:VGNC:84310]	1.082647677	0.002572496	0.010189749
ENSMMUG00000003261	BAF chromatin remodeling complex subunit BCL11B [Source:VGNC Symbol;Acc:VGNC:70056]	-1.527150262	2.61E-09	4.23E-08
ENSMMUG00000051552		-1.392055279	1.66E-07	1.93E-06
ENSMMUG00000061892		-1.364829713	1.86E-11	4.42E-10
ENSMMUG00000049437	Metazoan signal recognition particle RNA [Source:RFAM;Acc:RF00017]	-1.367734767	0.000107989	0.000638405
ENSMMUG00000029197	YY1 transcription factor [Source:NCBI gene;Acc:717370]	-1.297535521	2.73E-06	2.36E-05
ENSMMUG00000013871	WD repeat domain 25 [Source:VGNC Symbol;Acc:VGNC:78420]	2.737088769	1.36E-24	1.54E-22
ENSMMUG00000000186	protein phosphatase 2 regulatory subunit B'gamma [Source:NCBI gene;Acc:718894]	0.460107045	1.18E-07	1.40E-06
ENSMMUG00000005780	dynein cytoplasmic 1 heavy chain 1 [Source:VGNC Symbol;Acc:VGNC:71951]	0.410834819	1.22E-05	9.16E-05
ENSMMUG00000007140	MOK protein kinase [Source:VGNC Symbol;Acc:VGNC:84394]	1.674937093	1.61E-05	0.000116821
ENSMMUG00000003483	cyclin dependent kinase 2 interacting protein [Source:VGNC Symbol;Acc:VGNC:71326]	-0.760640387	0.015081929	0.045477288
ENSMMUG00000003484	tectonin beta-propeller repeat containing 2 [Source:NCBI gene;Acc:708959]	1.446215227	9.92E-10	1.73E-08
ENSMMUG00000005845	REST corepressor 1 [Source:VGNC Symbol;Acc:VGNC:76752]	0.74429019	0.000466779	0.002332612
ENSMMUG00000008380	CDC42 binding protein kinase beta [Source:VGNC Symbol;Acc:VGNC:81369]	0.625469486	0.005106539	0.018288682
ENSMMUG00000029186	exocyst complex component 3 like 4 [Source:VGNC Symbol;Acc:VGNC:72305]	3.519775478	0.000713223	0.003372844
ENSMMUG00000050495		2.639112608	1.19E-06	1.13E-05
ENSMMUG00000021634	TNF alpha induced protein 2 [Source:VGNC Symbol;Acc:VGNC:78579]	2.382044005	2.17E-23	2.16E-21
ENSMMUG00000049145	creatine kinase B [Source:NCBI gene;Acc:711167]	0.40004922	0.000615464	0.002966511
ENSMMUG00000018121	zinc finger FYVE-type containing 21 [Source:VGNC Symbol;Acc:VGNC:79282]	0.317054543	0.006406294	0.022147039
ENSMMUG00000052518	SIVA1 apoptosis inducing factor [Source:NCBI gene;Acc:706688]	-2.426299621	9.20E-05	0.000554193
ENSMMUG00000064694	AHNAK nucleoprotein 2 [Source:NCBI gene;Acc:707626]	3.069438125	0.000108252	0.000639701

ENSMMUG00000001273	clathrin binding box of aftiphilin containing 1 [Source:VGNC Symbol;Acc:VGNC:84325]	2.100513517	5.94E-10	1.09E-08
ENSMMUG00000065019	Metazoan signal recognition particle RNA [Source:RFAM;Acc:RF00017]	-2.129295722	1.58E-05	0.000115182
ENSMMUG00000022939	phosphofurin acidic cluster sorting protein 2 [Source:VGNC Symbol;Acc:VGNC:75671]	-1.061792935	0.006493542	0.022390091
ENSMMUG00000007159	tubulin epsilon and delta complex 1 [Source:VGNC Symbol;Acc:VGNC:79235]	-1.823708705	9.92E-05	0.000592297
ENSMMUG00000015202		-2.182913429	0.001481171	0.006349686
ENSMMUG00000040771		-1.632820668	1.52E-05	0.000111622
ENSMMUG00000059525		-1.878854791	5.71E-05	0.000363981
ENSMMUG00000055207		-3.775307821	2.83E-08	3.76E-07
ENSMMUG00000060892		-2.650089086	1.41E-13	4.65E-12
ENSMMUG00000064429	immunoglobulin heavy variable 1-45 [Source:HGNC Symbol;Acc:HGNC:5553]	-3.844042017	0.001328226	0.005779633
ENSMMUG00000052929		-4.14904743	2.90E-08	3.86E-07
ENSMMUG00000052535		-3.188992329	0.000461451	0.002308365
ENSMMUG00000057719		2.480100028	0.005691659	0.020072695
ENSMMUG00000063346		3.549773568	5.20E-06	4.24E-05
ENSMMUG00000038067		1.097650601	0.000291188	0.001535922
ENSMMUG00000053333	acetylserotonin O-methyltransferase like [Source:NCBI gene;Acc:106995142]	3.587355775	0.005243875	0.018711392
ENSMMUG00000000443	arylsulfatase F [Source:VGNC Symbol;Acc:VGNC:81362]	-0.890534695	1.70E-05	0.000122939
ENSMMUG00000017378	steroid sulfatase [Source:VGNC Symbol;Acc:VGNC:81590]	-1.428842969	0.004605324	0.016716004
ENSMMUG00000010252	FERM and PDZ domain containing 4 [Source:VGNC Symbol;Acc:VGNC:72802]	-0.848590041	0.000261808	0.001402323
ENSMMUG00000003482	glycine receptor alpha 2 [Source:VGNC Symbol;Acc:VGNC:73075]	1.054574574	0.000204663	0.001127117
ENSMMUG00000015737	phosphatidylinositol glycan anchor biosynthesis class A [Source:VGNC Symbol;Acc:VGNC:75984]	-0.918117124	2.53E-06	2.21E-05
ENSMMUG00000015740	pirin [Source:VGNC Symbol;Acc:VGNC:75893]	-0.676042478	0.009144365	0.030143227
ENSMMUG00000004797	CTP synthase 2 [Source:VGNC Symbol;Acc:VGNC:71550]	1.638543273	8.96E-08	1.08E-06
ENSMMUG00000058700	Metazoan signal recognition particle RNA [Source:RFAM;Acc:RF00017]	3.221015467	0.000732836	0.003452118
ENSMMUG00000019363	synapse associated protein 1 [Source:VGNC Symbol;Acc:VGNC:78135]	2.279642601	2.93E-12	7.90E-11
ENSMMUG00000016237	taxilin gamma [Source:NCBI gene;Acc:713769]	3.050655004	3.65E-05	0.000244717
ENSMMUG00000002134	ribosomal protein S6 kinase A3 [Source:VGNC Symbol;Acc:VGNC:76929]	-0.748779852	0.009815662	0.031950354
ENSMMUG00000001088	patched domain containing 1 [Source:VGNC Symbol;Acc:VGNC:76322]	1.464075171	3.55E-08	4.64E-07
ENSMMUG00000061261	Metazoan signal recognition particle RNA [Source:RFAM;Acc:RF00017]	-1.057516663	0.002099876	0.008618265
ENSMMUG00000042317	putative transcription factor SPT20 homolog-like 2 [Source:NCBI gene;Acc:100429227]	-0.808292807	0.005432894	0.019295867
ENSMMUG00000012819	pyruvate dehydrogenase kinase 3 [Source:VGNC Symbol;Acc:VGNC:75934]	-1.197232341	0.00028035	0.001490653
ENSMMUG00000012775	nuclear receptor subfamily 0 group B member 1 [Source:NCBI gene;Acc:574154]	-1.324840958	0.010155888	0.032837219
ENSMMUG00000005959	glycerol kinase [Source:NCBI gene;Acc:707531]	0.457475388	0.015222079	0.045823847
ENSMMUG00000013220	cytochrome b-245 beta chain [Source:VGNC Symbol;Acc:VGNC:71576]	-0.779954072	0.00591878	0.020737843

ENSMMUG00000045877	mitochondrial pyruvate carrier 1 like [Source:NCBI gene;Acc:705293]	2.733795616	0.015616776	0.046789175
ENSMMUG00000005264	mediator complex subunit 14 [Source:VGNC Symbol;Acc:VGNC:74561]	0.643993366	0.002288202	0.009257887
ENSMMUG00000063721		-0.7584249	7.39E-09	1.11E-07
ENSMMUG00000055907		0.627510019	0.001805503	0.007557329
ENSMMUG00000057909		1.220688986	0.002266385	0.00919008
ENSMMUG00000057804		-1.826542879	0.002038171	0.008405358
ENSMMUG00000003897	solute carrier family 9 member A7 [Source:NCBI gene;Acc:710906]	-0.744163887	3.32E-07	3.60E-06
ENSMMUG00000049666		0.457292715	0.015955539	0.047598127
ENSMMUG00000023016	jade family PHD finger 3 [Source:VGNC Symbol;Acc:VGNC:73640]	1.327259988	4.52E-15	1.91E-13
ENSMMUG00000019546	regucalcin [Source:VGNC Symbol;Acc:VGNC:76912]	1.673726519	5.15E-05	0.000332637
ENSMMUG00000017423	RNA binding motif protein 10 [Source:VGNC Symbol;Acc:VGNC:76680]	-1.871288254	0.005855837	0.020542062
ENSMMUG00000014002	cyclin dependent kinase 16 [Source:VGNC Symbol;Acc:VGNC:71160]	-1.231535882	0.001583642	0.006741258
ENSMMUG00000016608	ubiquitin specific peptidase 11 [Source:VGNC Symbol;Acc:VGNC:78734]	-1.318898325	0.010164212	0.032856827
ENSMMUG00000008566	zinc finger protein 157 [Source:VGNC Symbol;Acc:VGNC:78850]	0.714744366	2.05E-06	1.83E-05
ENSMMUG00000010404	zinc finger protein 41 [Source:VGNC Symbol;Acc:VGNC:78900]	-0.830783419	0.000369709	0.001889752
ENSMMUG00000063703		-0.856012519	0.00253587	0.010083136
ENSMMUG00000019768	suppressor of variegation 3-9 homolog 1 [Source:VGNC Symbol;Acc:VGNC:78061]	-0.756857238	0.002064251	0.008493635
ENSMMUG00000016465	histone deacetylase 6 [Source:VGNC Symbol;Acc:VGNC:73164]	0.954067025	5.92E-12	1.53E-10
ENSMMUG00000016467	ES cell expressed Ras [Source:VGNC Symbol;Acc:VGNC:72256]	1.07292571	0.016949852	0.049969579
ENSMMUG00000016469	proprotein convertase subtilisin/kexin type 1 inhibitor [Source:VGNC Symbol;Acc:VGNC:75639]	1.93579871	0.001178338	0.0052097
ENSMMUG00000004558	OTU deubiquitinase 5 [Source:VGNC Symbol;Acc:VGNC:75677]	0.954840518	9.59E-10	1.67E-08
ENSMMUG00000004559	potassium voltage-gated channel subfamily D member 1 [Source:VGNC Symbol;Acc:VGNC:73975]	-1.41837096	0.005625945	0.019908559
ENSMMUG00000010617	coiled-coil domain containing 120 [Source:VGNC Symbol;Acc:VGNC:70664]	0.423059594	0.004419785	0.016163628
ENSMMUG00000023025	synaptophysin [Source:VGNC Symbol;Acc:VGNC:78072]	0.623585688	1.77E-06	1.60E-05
ENSMMUG00000008618	calcium voltage-gated channel subunit alpha1 F [Source:VGNC Symbol;Acc:VGNC:70503]	1.018774996	2.87E-20	2.23E-18
ENSMMUG00000008623	coiled-coil domain containing 22 [Source:VGNC Symbol;Acc:VGNC:70899]	1.127902208	4.71E-14	1.71E-12
ENSMMUG00000052252	protein phosphatase 1 regulatory subunit 3F [Source:NCBI gene;Acc:715950]	1.425259964	1.38E-23	1.40E-21
ENSMMUG00000060311	G antigen 10-like [Source:NCBI gene;Acc:114674954]	0.422470811	0.010382198	0.033449915
ENSMMUG00000041376	G antigen 10 [Source:NCBI gene;Acc:693330]	1.265256048	5.35E-10	9.94E-09
ENSMMUG00000026584	Y RNA [Source:RFAM;Acc:RF00019]	1.893039048	0.008377624	0.027900462
ENSMMUG00000056042	Y RNA [Source:RFAM;Acc:RF00019]	2.749588628	0.003323134	0.012712738
ENSMMUG00000023060	A-kinase anchoring protein 4 [Source:VGNC Symbol;Acc:VGNC:69770]	2.469120587	4.65E-09	7.20E-08
ENSMMUG00000045093	cyclin B3 [Source:NCBI gene;Acc:694534]	2.213290869	3.36E-15	1.47E-13
ENSMMUG00000049267		5.050299395	4.51E-25	5.65E-23
ENSMMUG00000060192	bone morphogenetic protein 15 [Source:NCBI gene;Acc:695457]	3.718997044	0.004459929	0.016265427
ENSMMUG00000020202	melanoma-associated antigen D4 [Source:NCBI gene;Acc:106996411]	-1.021990649	0.006399931	0.022139313
ENSMMUG00000012073	X antigen family member 1-like [Source:NCBI gene;Acc:106996044]	-0.46785563	0.015454306	0.046417199
ENSMMUG00000046218	X antigen family member 5 [Source:NCBI gene;Acc:114669729]	-1.130070149	0.000463698	0.00231801
ENSMMUG00000053262		-0.644551655	0.001290591	0.005637417
ENSMMUG00000020555	IQ motif and Sec7 domain ArfGEF 2 [Source:VGNC Symbol;Acc:VGNC:73676]	-0.46199999	0.000580501	0.002817611
ENSMMUG00000047741	Metazoan signal recognition particle RNA [Source:RFAM;Acc:RF00017]	-0.877888114	0.000125697	0.000732365

ENSMMUG00000008422	trophinin [Source:NCBI gene;Acc:704512]	-0.746466184	3.24E-05	0.000220083
ENSMMUG00000059922	family with sequence similarity 104 member B [Source:NCBI gene;Acc:705418]	-3.973978265	5.90E-07	6.03E-06
ENSMMUG00000051670		-1.40124092	5.72E-09	8.75E-08
ENSMMUG00000054377		0.770075014	1.98E-10	3.95E-09
ENSMMUG00000063913	endogenous retrovirus group K member 7 Env polyprotein-like [Source:NCBI gene;Acc:114674933]	3.12287277	0.000144798	0.000831988
ENSMMUG00000059335		5.225182954	6.28E-08	7.83E-07
ENSMMUG00000004309	APC membrane recruitment protein 1 [Source:VGNC Symbol;Acc:VGNC:69716]	5.639327132	1.30E-07	1.53E-06
ENSMMUG00000048462	Metazoan signal recognition particle RNA [Source:RFAM;Acc:RF00017]	4.591456196	3.69E-05	0.000247181
ENSMMUG00000065109	ankyrin repeat and SOCS box containing 12 [Source:NCBI gene;Acc:707931]	3.858513324	0.000341385	0.001762953
ENSMMUG00000004274	myotubularin related protein 8 [Source:VGNC Symbol;Acc:VGNC:74974]	4.077758049	0.000272548	0.001453956
ENSMMUG00000065001		-0.533701587	8.60E-05	0.000522341
ENSMMUG00000047336	praja ring finger ubiquitin ligase 1 [Source:NCBI gene;Acc:695038]	-0.418247255	0.001633072	0.006927331
ENSMMUG00000061393	OTU deubiquitinase 6A [Source:NCBI gene;Acc:694498]	-1.684173339	0.00975667	0.031779679
ENSMMUG00000054817	immunoglobulin binding protein 1 [Source:NCBI gene;Acc:694632]	-1.232613784	4.97E-05	0.000322979
ENSMMUG00000013934	pyrimidinergic receptor P2Y4 [Source:VGNC Symbol;Acc:VGNC:75739]	-1.231058079	0.016491835	0.048966873
ENSMMUG00000013937	RAB41, member RAS oncogene family [Source:VGNC Symbol;Acc:VGNC:81544]	-1.07332758	0.002576729	0.010198178
ENSMMUG00000013940	kinesin family member 4A [Source:NCBI gene;Acc:695574]	-1.343489704	0.000963363	0.004385973
ENSMMUG00000013943	glycerophosphodiester phosphodiesterase domain containing 2 [Source:VGNC Symbol;Acc:VGNC:72787]	-1.937646467	4.61E-05	0.000302628
ENSMMUG00000003049	testis expressed 11 [Source:VGNC Symbol;Acc:VGNC:78316]	-3.105894413	1.70E-09	2.86E-08
ENSMMUG00000032429	forkhead box O4 [Source:VGNC Symbol;Acc:VGNC:72712]	1.591107692	9.43E-15	3.78E-13
ENSMMUG00000032427	neuroligin 3 [Source:NCBI gene;Acc:574216]	-1.167002406	8.98E-09	1.33E-07
ENSMMUG00000000669	gap junction protein beta 1 [Source:VGNC Symbol;Acc:VGNC:73059]	-2.137618285	2.92E-05	0.000199996
ENSMMUG00000000667	zinc finger MYM-type containing 3 [Source:VGNC Symbol;Acc:VGNC:78886]	-1.238702572	5.55E-05	0.000355456
ENSMMUG00000015238	O-linked N-acetylglucosamine (GlcNAc) transferase [Source:NCBI gene;Acc:698268]	-1.00736573	6.06E-08	7.58E-07
ENSMMUG00000011469	germ cell nuclear acidic peptidase [Source:VGNC Symbol;Acc:VGNC:72724]	-1.9038939	0.000317261	0.001654239
ENSMMUG00000054132		-2.836263532	0.003299527	0.012639058
ENSMMUG00000063858		-0.387242849	0.00163149	0.006922641
ENSMMUG00000009249	ring finger protein, LIM domain interacting [Source:NCBI gene;Acc:703471]	0.733347777	3.55E-07	3.82E-06
ENSMMUG00000053505	U6 spliceosomal RNA [Source:RFAM;Acc:RF00026]	1.215238154	0.007661337	0.025894642
ENSMMUG00000017660		1.336819253	2.79E-12	7.55E-11
ENSMMUG00000011440	neurite extension and migration factor [Source:VGNC Symbol;Acc:VGNC:75274]	1.364336886	1.69E-06	1.54E-05
ENSMMUG00000015619	ATP binding cassette subfamily B member 7 [Source:VGNC Symbol;Acc:VGNC:81340]	-0.913474859	0.000383316	0.001954496
ENSMMUG00000049351	MAGE family member E1 [Source:NCBI gene;Acc:705379]	-1.438404222	2.27E-15	1.01E-13
ENSMMUG00000014407	magnesium transporter 1 [Source:VGNC Symbol;Acc:VGNC:74386]	0.810272139	1.32E-06	1.23E-05
ENSMMUG00000015861	cytochrome c oxidase subunit VIIb [Source:NCBI gene;Acc:706099]	0.839683475	1.53E-05	0.000112069
ENSMMUG00000013387	cysteinyl leukotriene receptor 1 [Source:VGNC Symbol;Acc:VGNC:71646]	1.426668695	2.12E-12	5.89E-11
ENSMMUG00000052073		1.313220413	1.51E-15	6.96E-14

ENSMMUG00000049425		0.663756191	0.010950259	0.034931735
ENSMMUG00000053430		0.998367558	0.000687285	0.003270397
ENSMMUG00000008212	tenomodulin [Source:VGNC Symbol;Acc:VGNC:79087]	-0.805913222	0.00396984	0.014751932
ENSMMUG00000013008	cleavage stimulation factor subunit 2 [Source:VGNC Symbol;Acc:VGNC:71536]	-1.103269698	0.000135317	0.000783085
ENSMMUG00000004178	NADPH oxidase 1 [Source:VGNC Symbol;Acc:VGNC:75374]	-1.751852033	6.22E-12	1.60E-10
ENSMMUG00000065018	transmembrane protein 35A [Source:NCBI gene;Acc:702205]	-1.253667285	2.68E-06	2.33E-05
ENSMMUG00000007601	centromere protein I [Source:NCBI gene;Acc:702316]	-0.905911964	0.000324712	0.001689457
ENSMMUG00000059432		2.178644408	0.000254574	0.00136559
ENSMMUG00000022281	family with sequence similarity 199, X-linked [Source:NCBI gene;Acc:699523]	2.165524	0.005141034	0.018389571
ENSMMUG00000013150	MORC family CW-type zinc finger 4 [Source:VGNC Symbol;Acc:VGNC:74808]	1.469419875	4.41E-19	3.06E-17
ENSMMUG00000013146	claudin 2 [Source:HGNC Symbol;Acc:HGNC:2041]	1.556640053	1.82E-21	1.59E-19
ENSMMUG00000022293	FERM and PDZ domain containing 3 [Source:VGNC Symbol;Acc:VGNC:72805]	-0.646180775	1.27E-07	1.51E-06
ENSMMUG00000004674	phosphoribosyl pyrophosphate synthetase 1 [Source:NCBI gene;Acc:702529]	-1.031142093	3.13E-13	9.80E-12
ENSMMUG00000007688	angiomotin [Source:VGNC Symbol;Acc:VGNC:69627]	4.153800951	4.46E-05	0.000293916
ENSMMUG00000006377	plastin 3 [Source:VGNC Symbol;Acc:VGNC:76181]	0.738923324	0.001789916	0.007500725
ENSMMUG00000055906	angiotensin II receptor type 2 [Source:NCBI gene;Acc:709513]	1.968939556	8.69E-06	6.72E-05
ENSMMUG00000026243	U6 spliceosomal RNA [Source:RFAM;Acc:RF00026]	2.2947595	0.004557352	0.016562557
ENSMMUG00000057063	solute carrier family 6 member 14 [Source:NCBI gene;Acc:709598]	1.715745667	0.004463187	0.016273227
ENSMMUG00000008781	BCL6 corepressor like 1 [Source:VGNC Symbol;Acc:VGNC:70131]	-0.731901457	4.93E-07	5.14E-06
ENSMMUG00000063900	Metazoan signal recognition particle RNA [Source:RFAM;Acc:RF00017]	-2.209784968	0.016810691	0.04971054
ENSMMUG00000004954	apoptosis inducing factor mitochondria associated 1 [Source:VGNC Symbol;Acc:VGNC:69820]	-0.695270909	0.001434651	0.006175756
ENSMMUG00000003479	FERM domain containing 7 [Source:VGNC Symbol;Acc:VGNC:72927]	-1.137892375	0.001402371	0.006054727
ENSMMUG00000048745	RAP2C, member of RAS oncogene family [Source:NCBI gene;Acc:705986]	-0.774977689	0.002332196	0.009396656
ENSMMUG00000025616		-3.384244608	0.000282069	0.001498699
ENSMMUG00000003456	glypican 4 [Source:VGNC Symbol;Acc:VGNC:73118]	-0.533621436	7.11E-07	7.10E-06
ENSMMUG00000009622	glypican 3 [Source:VGNC Symbol;Acc:VGNC:73117]	0.888446504	0.003754531	0.014099617
ENSMMUG00000032341	coiled-coil domain containing 160 [Source:VGNC Symbol;Acc:VGNC:70926]	0.860782304	2.16E-09	3.55E-08
ENSMMUG00000016610	CD99 molecule like 2 [Source:VGNC Symbol;Acc:VGNC:70823]	-1.749145592	4.61E-08	5.89E-07
ENSMMUG00000016929	proline rich and Gla domain 3 [Source:VGNC Symbol;Acc:VGNC:76350]	1.975180067	1.31E-05	9.79E-05
ENSMMUG00000040974	fetal and adult testis expressed 1 [Source:NCBI gene;Acc:704368]	2.883446816	0.000847516	0.003933695
ENSMMUG00000063694	cyclic nucleotide gated channel subunit alpha 2 [Source:NCBI gene;Acc:704486]	0.973068324	9.21E-13	2.70E-11
ENSMMUG00000062628		1.132537017	0.000381778	0.001947334
ENSMMUG00000040274	MAGE family member A4 [Source:NCBI gene;Acc:710825]	1.004118861	1.73E-07	1.99E-06
ENSMMUG00000039292		1.527200038	3.60E-10	6.90E-09
ENSMMUG00000062063	melanoma-associated antigen 3 [Source:NCBI gene;Acc:711544]	1.523855033	9.19E-14	3.17E-12
ENSMMUG00000037641	chondrosarcoma-associated gene 2/3 protein [Source:NCBI gene;Acc:106992305]	2.577120275	5.84E-05	0.000371466
ENSMMUG00000049656	melanoma-associated antigen 2 [Source:NCBI gene;Acc:706218]	1.760536416	0.001172706	0.005186377
ENSMMUG00000058013	melanoma-associated antigen 3 [Source:NCBI gene;Acc:706103]	1.818484951	4.15E-10	7.89E-09
ENSMMUG00000040071		2.490887483	1.68E-07	1.94E-06

ENSMMUG00000020743	centrin 2 [Source:NCBI gene;Acc:693376]	1.937852999	0.001241986	0.00545362
ENSMMUG00000013176	NAD(P) dependent steroid dehydrogenase-like [Source:NCBI gene;Acc:693333]	2.221769673	8.44E-10	1.50E-08
ENSMMUG00000010897	PNMA family member 3 [Source:VGNC Symbol;Acc:VGNC:76063]	0.450632656	0.006045534	0.021100582
ENSMMUG00000064249		-1.238837566	0.005452943	0.01935289
ENSMMUG00000041072		-1.07213426	0.007648345	0.025868774
ENSMMUG00000061278		-1.330726579	6.58E-08	8.18E-07
ENSMMUG00000010137	microcephalin 1 [Source:VGNC Symbol;Acc:VGNC:74687]	2.199143811	1.34E-06	1.26E-05
ENSMMUG00000058176	b-defensin2-like [Source:NCBI gene;Acc:100169969]	2.692502203	3.73E-20	2.85E-18
ENSMMUG00000049842		-3.115087611	0.000120917	0.000707065
ENSMMUG00000052334	defensin beta 108B [Source:NCBI gene;Acc:696933]	-2.225761752	0.009313279	0.03058214
ENSMMUG00000029034	defensin beta 136 [Source:VGNC Symbol;Acc:VGNC:71826]	1.88777615	5.01E-06	4.10E-05
ENSMMUG00000019206	BLK proto-oncogene, Src family tyrosine kinase [Source:VGNC Symbol;Acc:VGNC:70253]	-0.894055954	0.009360721	0.030710177
ENSMMUG00000050140	Metazoan signal recognition particle RNA [Source:RFAM;Acc:RF00017]	2.540630606	0.001137253	0.005046472
ENSMMUG00000063525		2.693097494	1.94E-15	8.74E-14
ENSMMUG00000054960		2.18003132	2.02E-09	3.33E-08
ENSMMUG00000027220	microRNA 598 [Source:HGNC Symbol;Acc:HGNC:32854]	2.717294605	0.00332095	0.012707725
ENSMMUG00000010180	PIN2 (TERF1) interacting telomerase inhibitor 1 [Source:NCBI gene;Acc:701523]	2.400141849	8.40E-11	1.79E-09
ENSMMUG00000008867	exoribonuclease 1 [Source:VGNC Symbol;Acc:VGNC:72324]	-2.660982577	0.000189835	0.001056521
ENSMMUG00000007068	tumor suppressor candidate 3 [Source:VGNC Symbol;Acc:VGNC:84132]	3.602538335	1.82E-06	1.64E-05
ENSMMUG00000022303	solute carrier family 7 member 2 [Source:VGNC Symbol;Acc:VGNC:77629]	-0.535509725	2.24E-11	5.20E-10
ENSMMUG00000001151	microtubule associated scaffold protein 1 [Source:VGNC Symbol;Acc:VGNC:84018]	2.356122106	2.40E-32	6.46E-30
ENSMMUG00000015487	pericentriolar material 1 [Source:VGNC Symbol;Acc:VGNC:75781]	4.39177431	0.001841069	0.007677457
ENSMMUG00000005920	N-acetyltransferase 1 [Source:VGNC Symbol;Acc:VGNC:75133]	3.209557227	5.13E-06	4.18E-05
ENSMMUG00000011774	integrator complex subunit 10 [Source:VGNC Symbol;Acc:VGNC:73653]	1.222807846	2.41E-16	1.21E-14
ENSMMUG00000002032	exportin 7 [Source:VGNC Symbol;Acc:VGNC:78946]	-1.02709717	4.68E-05	0.000306484
ENSMMUG00000063764		-2.038131075	0.0008576	0.003969096
ENSMMUG00000013029	surfactant protein C [Source:VGNC Symbol;Acc:VGNC:84113]	-2.209323567	7.90E-10	1.41E-08
ENSMMUG00000013031	bone morphogenetic protein 1 [Source:VGNC Symbol;Acc:VGNC:70259]	-0.857324515	1.82E-05	0.000130928
ENSMMUG00000013032	phytanoyl-CoA 2-hydroxylase interacting protein [Source:VGNC Symbol;Acc:VGNC:75812]	-1.125370528	3.01E-07	3.29E-06
ENSMMUG00000023226	RNA polymerase III subunit D [Source:VGNC Symbol;Acc:VGNC:76017]	-0.720510335	1.80E-05	0.000129634
ENSMMUG00000052651	Metazoan signal recognition particle RNA [Source:RFAM;Acc:RF00017]	-1.030476803	1.43E-05	0.000105742
ENSMMUG00000014438	piwi like RNA-mediated gene silencing 2 [Source:VGNC Symbol;Acc:VGNC:81502]	-0.72529693	1.14E-07	1.35E-06
ENSMMUG00000017713	solute carrier family 39 member 14 [Source:VGNC Symbol;Acc:VGNC:77537]	0.704295768	0.010294402	0.033211213
ENSMMUG00000018345	phosphatidylethanolamine binding protein 4 [Source:VGNC Symbol;Acc:VGNC:82204]	1.886127148	1.76E-12	4.93E-11
ENSMMUG00000050868		1.268417085	0.000964406	0.004387972
ENSMMUG00000007540	Rho related BTB domain containing 2 [Source:VGNC Symbol;Acc:VGNC:76772]	0.582068157	0.004831002	0.017432847
ENSMMUG00000048076	TNF receptor superfamily member 10b [Source:NCBI gene;Acc:716650]	0.651991584	0.000955465	0.004358202
ENSMMUG00000056749	Metazoan signal recognition particle RNA [Source:RFAM;Acc:RF00017]	2.415157142	0.002514722	0.010015817
ENSMMUG00000050549	lysyl oxidase like 2 [Source:NCBI gene;Acc:710151]	1.616376105	7.70E-08	9.43E-07
ENSMMUG00000016658	ectonucleoside triphosphate diphosphohydrolase 4 [Source:NCBI gene;Acc:717197]	1.019811059	0.01510987	0.045552092
ENSMMUG00000046186	stanniocalcin 1 [Source:VGNC Symbol;Acc:VGNC:84120]	2.13530781	0.011330054	0.035930409

ENSMMUG00000003348	neurofilament light [Source:VGNC Symbol;Acc:VGNC:75307]	1.961410989	6.26E-21	5.20E-19
ENSMMUG00000056605	Metazoan signal recognition particle RNA [Source:RFAM;Acc:RF00017]	2.243662972	4.72E-05	0.000308348
ENSMMUG00000059181		0.932581229	0.005798478	0.020385122
ENSMMUG00000016018	establishment of sister chromatid cohesion N-acetyltransferase 2 [Source:VGNC Symbol;Acc:VGNC:72374]	- 1.995707601	0.004086517	0.015104423
ENSMMUG00000016019	PDZ binding kinase [Source:VGNC Symbol;Acc:VGNC:75643]	-3.35392283	5.92E-05	0.000375156
ENSMMUG00000022044	scavenger receptor class A member 5 [Source:VGNC Symbol;Acc:VGNC:77117]	- 0.791584693	6.30E-07	6.37E-06
ENSMMUG00000001040	nuclear GTPase, germinal center associated [Source:VGNC Symbol;Acc:VGNC:75497]	- 2.814688515	0.016748307	0.049596686
ENSMMUG00000005067	elongator acetyltransferase complex subunit 3 [Source:VGNC Symbol;Acc:VGNC:72080]	- 1.827051582	2.96E-10	5.72E-09
ENSMMUG00000014981	homeobox containing 1 [Source:VGNC Symbol;Acc:VGNC:73443]	0.803445367	2.53E-05	0.000176406
ENSMMUG00000061408		- 3.949105399	8.37E-08	1.02E-06
ENSMMUG00000063345		- 4.955037473	6.09E-11	1.32E-09
ENSMMUG00000034978	U6 spliceosomal RNA [Source:RFAM;Acc:RF00026]	2.117775537	0.003825876	0.014312083
ENSMMUG00000001635	zinc finger protein 703 [Source:VGNC Symbol;Acc:VGNC:79552]	0.659142515	0.002967373	0.011521556
ENSMMUG00000051162		- 2.634877481	0.002251593	0.009137748
ENSMMUG00000003591	pleckstrin homology domain containing A2 [Source:VGNC Symbol;Acc:VGNC:76026]	1.483879252	0.000130256	0.000757715
ENSMMUG00000019304	indoleamine 2,3-dioxygenase 1 [Source:VGNC Symbol;Acc:VGNC:73580]	2.603426367	1.19E-10	2.46E-09
ENSMMUG00000019306	indoleamine 2,3-dioxygenase 2 [Source:VGNC Symbol;Acc:VGNC:84005]	2.79078527	0.00031189	0.001628573
ENSMMUG00000000776	zinc finger matrin-type 4 [Source:VGNC Symbol;Acc:VGNC:78424]	1.210846046	1.44E-09	2.45E-08
ENSMMUG00000014721	glycerol-3-phosphate acyltransferase 4 [Source:VGNC Symbol;Acc:VGNC:84002]	- 0.810291809	0.00121711	0.00535993
ENSMMUG00000010891	lysine acetyltransferase 6A [Source:VGNC Symbol;Acc:VGNC:73821]	- 0.940611432	4.84E-05	0.000315678
ENSMMUG00000020185	voltage dependent anion channel 3 [Source:VGNC Symbol;Acc:VGNC:84135]	1.832092593	6.43E-06	5.12E-05
ENSMMUG00000020186	solute carrier family 20 member 2 [Source:VGNC Symbol;Acc:VGNC:77416]	1.221855212	0.0023004	0.009296892
ENSMMUG00000053488	small integral membrane protein 19 [Source:NCBI gene;Acc:705371]	0.784720146	3.89E-10	7.44E-09
ENSMMUG00000062518		1.171335956	0.000571992	0.002779093
ENSMMUG00000002781	peroxidasin like [Source:VGNC Symbol;Acc:VGNC:84107]	- 0.527130803	0.000225507	0.001229632
ENSMMUG00000065247	opioid receptor kappa 1 [Source:NCBI gene;Acc:710783]	1.994980654	2.64E-28	5.04E-26
ENSMMUG00000025039	Small nucleolar RNA SNORA73 family [Source:RFAM;Acc:RF00045]	3.297583771	0.008166639	0.027297867
ENSMMUG00000005478	trimethylguanosine synthase 1 [Source:VGNC Symbol;Acc:VGNC:78404]	0.988790728	4.10E-05	0.000272136
ENSMMUG00000014149	LYN proto-oncogene, Src family tyrosine kinase [Source:VGNC Symbol;Acc:VGNC:74464]	0.893136998	6.04E-15	2.51E-13
ENSMMUG00000055121	Metazoan signal recognition particle RNA [Source:RFAM;Acc:RF00017]	1.230301131	4.65E-06	3.82E-05
ENSMMUG00000039862	Metazoan signal recognition particle RNA [Source:RFAM;Acc:RF00017]	- 1.433640644	0.0035407	0.013393484
ENSMMUG00000061268	PLAG1 zinc finger [Source:NCBI gene;Acc:696149]	- 1.374408373	9.78E-06	7.50E-05
ENSMMUG00000046464	coiled-coil-helix-coiled-coil-helix domain containing 7 [Source:VGNC Symbol;Acc:VGNC:83989]	- 1.306234874	1.88E-07	2.15E-06
ENSMMUG00000061068		- 1.129541548	0.000193317	0.001072613
ENSMMUG00000014810	short chain dehydrogenase/reductase family 16C member 5 [Source:VGNC Symbol;Acc:VGNC:77343]	- 1.049658991	3.70E-07	3.97E-06
ENSMMUG00000052791		-1.23677277	1.55E-08	2.17E-07
ENSMMUG00000006775	proenkephalin [Source:VGNC Symbol;Acc:VGNC:75941]	- 1.316878113	1.39E-09	2.37E-08

ENSMMUG00000006543	cytochrome P450, family 7, subfamily A, polypeptide 1 [Source:NCBI gene;Acc:698127]	-3.288815092	0.007317376	0.024905804
ENSMMUG00000017038	RAB2A, member RAS oncogene family [Source:VGNC Symbol;Acc:VGNC:81529]	0.604718544	1.20E-05	9.05E-05
ENSMMUG00000059575		-2.129673651	1.38E-27	2.39E-25
ENSMMUG00000038661		-2.297887452	9.06E-25	1.08E-22
ENSMMUG00000049866		1.183346545	4.01E-11	8.96E-10
ENSMMUG00000014424	ADP ribosylation factor guanine nucleotide exchange factor 1 [Source:VGNC Symbol;Acc:VGNC:69834]	-1.695711051	0.008985529	0.029673476
ENSMMUG00000045333	Metazoan signal recognition particle RNA [Source:RFAM;Acc:RF00017]	-0.5878625	0.000477897	0.00237592
ENSMMUG00000007576	EYA transcriptional coactivator and phosphatase 1 [Source:VGNC Symbol;Acc:VGNC:72453]	-0.587971314	9.56E-07	9.24E-06
ENSMMUG00000010748	transient receptor potential cation channel subfamily A member 1 [Source:VGNC Symbol;Acc:VGNC:78429]	-0.655317912	0.006742566	0.023160841
ENSMMUG00000060062		-1.602268114	0.010620379	0.034021254
ENSMMUG00000046641		-0.491538791	0.002367742	0.009503402
ENSMMUG00000007115	RALY RNA binding protein like [Source:VGNC Symbol;Acc:VGNC:82217]	-1.191131378	0.002277126	0.009220775
ENSMMUG00000028699		-0.951343855	0.012089584	0.037868194
ENSMMUG00000028701		-1.079705639	9.41E-05	0.000565287
ENSMMUG00000013193	matrix metallopeptidase 16 [Source:VGNC Symbol;Acc:VGNC:74894]	1.00614805	1.02E-10	2.13E-09
ENSMMUG00000061720		-0.713537807	0.013431562	0.041376271
ENSMMUG00000021112	epithelial splicing regulatory protein 1 [Source:VGNC Symbol;Acc:VGNC:72358]	-0.801426655	2.60E-06	2.26E-05
ENSMMUG00000057219	tumor protein p53 inducible nuclear protein 1 [Source:NCBI gene;Acc:700632]	2.618511963	1.09E-05	8.31E-05
ENSMMUG00000003878	carboxypeptidase Q [Source:VGNC Symbol;Acc:VGNC:71426]	0.929903198	0.011629576	0.036656362
ENSMMUG00000055454		0.493985685	0.000116322	0.000683497
ENSMMUG00000060329		0.480020929	0.002704959	0.010607495
ENSMMUG00000061917		0.795439761	2.06E-08	2.81E-07
ENSMMUG00000014210	metadherin [Source:VGNC Symbol;Acc:VGNC:74859]	0.667165194	2.30E-07	2.58E-06
ENSMMUG00000008926	lysosomal protein transmembrane 4 beta [Source:VGNC Symbol;Acc:VGNC:84010]	0.585549401	1.50E-06	1.39E-05
ENSMMUG00000060771		0.801776253	2.94E-08	3.89E-07
ENSMMUG00000020072	matrilin 2 [Source:VGNC Symbol;Acc:VGNC:74665]	0.467062731	0.00133408	0.005799497
ENSMMUG00000058389		0.48520177	0.01418214	0.043266689
ENSMMUG00000022185	odd-skipped related transciption factor 2 [Source:VGNC Symbol;Acc:VGNC:84097]	-1.352906988	1.38E-11	3.35E-10
ENSMMUG00000022186	vacuolar protein sorting 13 homolog B [Source:VGNC Symbol;Acc:VGNC:79339]	-0.446271761	0.006978245	0.023840832
ENSMMUG00000013742	regulator of G protein signaling 22 [Source:VGNC Symbol;Acc:VGNC:82221]	-1.629413963	0.007494842	0.025432427
ENSMMUG00000004386	sperm associated antigen 1 [Source:VGNC Symbol;Acc:VGNC:77849]	-1.337958999	0.004339257	0.015905138
ENSMMUG00000061687		-1.23825833	0.001237647	0.005438664
ENSMMUG00000004387	ring finger protein 19A, RBR E3 ubiquitin protein ligase [Source:VGNC Symbol;Acc:VGNC:82224]	-1.329512783	0.000178516	0.000998879
ENSMMUG00000051088		-1.078151417	0.004136821	0.015255447
ENSMMUG00000009958	ankyrin repeat domain 46 [Source:VGNC Symbol;Acc:VGNC:69719]	-1.75721554	5.27E-07	5.45E-06
ENSMMUG00000034608	U6 spliceosomal RNA [Source:RFAM;Acc:RF00026]	0.804722894	0.004831896	0.017432847

ENSMMUG00000051999		-1.638338681	3.21E-05	0.000218054
ENSMMUG00000001338	antizyme inhibitor 1 [Source:VGNC Symbol;Acc:VGNC:70080]	2.828497985	0.000185641	0.001035162
ENSMMUG00000054357		-2.565470742	0.002382556	0.009551906
ENSMMUG00000010744		-1.399317205	0.00881009	0.029180288
ENSMMUG00000011359	eukaryotic translation initiation factor 3 subunit E [Source:VGNC Symbol;Acc:VGNC:83997]	-0.801878611	0.000326175	0.001694643
ENSMMUG00000003435	transcriptional repressor GATA binding 1 [Source:VGNC Symbol;Acc:VGNC:78656]	-0.472810257	0.000969575	0.004405376
ENSMMUG00000002068	mediator complex subunit 30 [Source:VGNC Symbol;Acc:VGNC:74556]	-3.208801082	0.011401322	0.036117022
ENSMMUG00000004903	ectonucleotide pyrophosphatase/phosphodiesterase 2 [Source:VGNC Symbol;Acc:VGNC:72223]	0.777307307	0.000577824	0.002806175
ENSMMUG00000014702	TATA-box binding protein associated factor 2 [Source:VGNC Symbol;Acc:VGNC:78090]	0.846625987	0.000415357	0.002101649
ENSMMUG00000058797		0.788442459	1.73E-05	0.000124628
ENSMMUG00000052557		0.534161721	1.12E-07	1.33E-06
ENSMMUG00000021157	TBC1 domain family member 31 [Source:VGNC Symbol;Acc:VGNC:84121]	3.508223027	6.03E-06	4.84E-05
ENSMMUG00000020467	ATPase family AAA domain containing 2 [Source:VGNC Symbol;Acc:VGNC:70154]	-0.22434525	0.003225417	0.01237474
ENSMMUG00000047542	U4 spliceosomal RNA [Source:RFAM;Acc:RF00015]	-1.708502237	0.000840882	0.003905401
ENSMMUG00000023778	F-box protein 32 [Source:VGNC Symbol;Acc:VGNC:72517]	-1.126623201	0.000386746	0.001967994
ENSMMUG00000054878		-1.283986229	0.009482603	0.031026019
ENSMMUG00000054755	transmembrane protein 65 [Source:NCBI gene;Acc:705727]	1.042123339	0.0033136	0.012682938
ENSMMUG00000060708		0.877179098	0.000623312	0.002998374
ENSMMUG00000058106	otoconin 90 [Source:NCBI gene;Acc:100429699]	0.844190584	3.46E-05	0.000233429
ENSMMUG00000012560	HERV-H LTR-associating 1 [Source:NCBI gene;Acc:100430297]	0.892296554	0.00016395	0.000925567
ENSMMUG00000048066	trafficking protein particle complex 9 [Source:NCBI gene;Acc:711466]	1.140772379	1.26E-08	1.81E-07
ENSMMUG00000053837		4.575685734	0.000354556	0.001823841
ENSMMUG00000052421		-1.149469355	0.000448743	0.002255664
ENSMMUG00000059617		-0.881508476	3.48E-05	0.000234753
ENSMMUG00000001432	t-SNARE domain containing 1 [Source:NCBI gene;Acc:701269]	-0.660728531	3.49E-05	0.000234871
ENSMMUG00000019877	adhesion G protein-coupled receptor B1 [Source:VGNC Symbol;Acc:VGNC:69597]	0.680724367	0.011082003	0.035274594
ENSMMUG00000062642		-0.81107241	9.42E-05	0.000565319
ENSMMUG00000000959	cytochrome P450, family 11, subfamily B, polypeptide 2 [Source:NCBI gene;Acc:695914]	0.469787933	0.014120078	0.043113543
ENSMMUG00000022933	DNA topoisomerase I mitochondrial [Source:VGNC Symbol;Acc:VGNC:79821]	0.862518637	1.29E-09	2.22E-08
ENSMMUG00000059932	Metazoan signal recognition particle RNA [Source:RFAM;Acc:RF00017]	1.309198913	0.002106761	0.008644083
ENSMMUG00000010939	rhophilin Rho GTPase binding protein 1 [Source:VGNC Symbol;Acc:VGNC:76779]	-0.738301214	1.29E-06	1.21E-05
ENSMMUG00000013552	scribble planar cell polarity protein [Source:HGNC Symbol;Acc:HGNC:30377]	-1.052458568	8.50E-08	1.03E-06
ENSMMUG00000013558	poly(U) binding splicing factor 60 [Source:VGNC Symbol;Acc:VGNC:84106]	-0.860541212	0.002299835	0.009296892
ENSMMUG00000000388	plectin [Source:VGNC Symbol;Acc:VGNC:76173]	-0.908784972	3.11E-07	3.40E-06
ENSMMUG00000060194	mml-mir-661 [Source:miRBase;Acc:MI0007902]	-1.66397521	0.011450193	0.036232358
ENSMMUG00000021279	zinc finger MYND-type containing 11 [Source:VGNC Symbol;Acc:VGNC:79060]	0.354161893	0.000202061	0.001114327
ENSMMUG00000050907		-0.793845622	6.31E-05	0.000397

ENSMMUG00000053106		2.157571462	6.33E-11	1.37E-09
ENSMMUG00000023282	aldo-keto reductase family 1, member C1 (dihydrodiol dehydrogenase 1; 20-alpha (3-alpha)-hydroxysteroid dehydrogenase) [Source:NCBI gene;Acc:711198]	1.248226717	8.48E-07	8.27E-06
ENSMMUG00000000958	aldo-keto reductase family 1, member C2 [Source:NCBI gene;Acc:711360]	1.431825275	1.14E-12	3.28E-11
ENSMMUG00000001290	ankyrin repeat and SOCS box containing 13 [Source:VGNC Symbol;Acc:VGNC:69865]	-2.431725824	0.004175818	0.015375851
ENSMMUG00000015141	ankyrin repeat domain 16 [Source:NCBI gene;Acc:720787]	-2.34637835	1.42E-08	2.02E-07
ENSMMUG00000015143	F-box DNA helicase 1 [Source:NCBI gene;Acc:720797]	-1.652299385	3.06E-06	2.63E-05
ENSMMUG00000056045		-1.971215539	1.05E-13	3.57E-12
ENSMMUG00000015149	interleukin 15 receptor subunit alpha [Source:VGNC Symbol;Acc:VGNC:73711]	-1.62461423	0.002546312	0.010113591
ENSMMUG00000015151	interleukin 2 receptor subunit alpha [Source:NCBI gene;Acc:574300]	-1.677336494	2.73E-06	2.36E-05
ENSMMUG00000054646		-2.645161719	0.00601545	0.021015766
ENSMMUG00000010596	6-phosphofructo-2-kinase/fructose-2,6-biphosphatase 3 [Source:VGNC Symbol;Acc:VGNC:75959]	-2.172175488	0.014665783	0.04446225
ENSMMUG00000061189		0.426420681	0.004792133	0.017307638
ENSMMUG00000052242		0.558163538	0.00767354	0.025929857
ENSMMUG00000064980		-1.348887001	0.000446889	0.00224712
ENSMMUG00000037449	CUGBP Elav-like family member 2 [Source:NCBI gene;Acc:714291]	0.78314403	0.00149644	0.006405697
ENSMMUG00000017145	cell division cycle 123 [Source:VGNC Symbol;Acc:VGNC:70768]	-1.107957035	4.45E-07	4.69E-06
ENSMMUG00000017170	cerebral dopamine neurotrophic factor [Source:VGNC Symbol;Acc:VGNC:71094]	-0.473839108	2.73E-05	0.000188604
ENSMMUG00000017173	heat shock protein family A (Hsp70) member 14 [Source:NCBI gene;Acc:697257]	-1.718861996	1.19E-05	9.01E-05
ENSMMUG00000011661	integrin subunit alpha 8 [Source:VGNC Symbol;Acc:VGNC:73782]	1.577640942	2.38E-11	5.52E-10
ENSMMUG00000024234	U6 spliceosomal RNA [Source:RFAM;Acc:RF00026]	2.254692285	1.80E-11	4.31E-10
ENSMMUG00000016069	cubilin [Source:VGNC Symbol;Acc:VGNC:82132]	2.328402114	5.42E-10	1.00E-08
ENSMMUG00000010589	nebulette [Source:VGNC Symbol;Acc:VGNC:75195]	-0.35600315	0.009056213	0.029893316
ENSMMUG00000002930	G protein-coupled receptor 158 [Source:NCBI gene;Acc:707065]	0.623275522	0.001459662	0.006269097
ENSMMUG00000058873		0.65584583	0.006117438	0.021289917
ENSMMUG00000037243	U6 spliceosomal RNA [Source:RFAM;Acc:RF00026]	1.540568341	7.75E-09	1.16E-07
ENSMMUG00000016410	acyl-CoA binding domain containing 5 [Source:VGNC Symbol;Acc:VGNC:69509]	-0.819509244	4.09E-05	0.000271556
ENSMMUG00000058108	BMP and activin membrane bound inhibitor [Source:NCBI gene;Acc:710152]	1.09412066	0.004047155	0.014985606
ENSMMUG00000001374	supervillin [Source:NCBI gene;Acc:695267]	-0.938813035	3.73E-06	3.15E-05
ENSMMUG00000050047	Metazoan signal recognition particle RNA [Source:RFAM;Acc:RF00017]	0.703615837	0.004182663	0.015397156
ENSMMUG00000041067	Metazoan signal recognition particle RNA [Source:RFAM;Acc:RF00017]	-1.219319127	7.53E-06	5.91E-05
ENSMMUG00000040958	lysozyme-like protein 1 [Source:NCBI gene;Acc:698176]	-1.030340526	1.46E-08	2.06E-07
ENSMMUG00000044214		-1.24806716	1.76E-09	2.94E-08
ENSMMUG00000017767	integrin subunit beta 1 [Source:VGNC Symbol;Acc:VGNC:73784]	2.248863161	0.003818004	0.014286311
ENSMMUG00000043648		1.758366578	7.34E-05	0.000454353
ENSMMUG00000057199		-1.579886894	2.17E-05	0.000153343
ENSMMUG00000006713	cyclin Y [Source:VGNC Symbol;Acc:VGNC:70840]	-0.808573636	2.06E-07	2.33E-06
ENSMMUG00000054392	zinc finger protein 239 [Source:NCBI gene;Acc:717737]	-0.778651368	0.013092101	0.040450557

ENSMMUG00000056797		2.695768636	0.004349521	0.015938741
ENSMMUG00000064634		-0.403299904	0.0030501	0.011798644
ENSMMUG00000021308	chromosome 9 C10orf71 homolog [Source:NCBI gene;Acc:710332]	-0.937128608	0.00013748	0.000793075
ENSMMUG00000033001	U6 spliceosomal RNA [Source:RFAM;Acc:RF00026]	-0.87638537	1.10E-08	1.60E-07
ENSMMUG00000017380	neuregulin 3 [Source:NCBI gene;Acc:699268]	2.451214853	0.000603699	0.002917544
ENSMMUG00000055918		3.153242087	2.46E-05	0.000171881
ENSMMUG00000040878		3.426202845	0.005683538	0.020058652
ENSMMUG00000058251		-1.451637332	0.014504415	0.044046518
ENSMMUG00000057196	pulmonary surfactant-associated protein A [Source:NCBI gene;Acc:701715]	1.564643129	4.59E-19	3.17E-17
ENSMMUG00000051972	zinc finger CCHC-type containing 24 [Source:NCBI gene;Acc:702083]	2.544640444	0.000157545	0.000894624
ENSMMUG00000055601		0.849942881	1.33E-08	1.89E-07
ENSMMUG00000005514	RNA polymerase III subunit A [Source:VGNC Symbol;Acc:VGNC:76138]	0.562599642	0.000958859	0.004372079
ENSMMUG00000009128	discs large MAGUK scaffold protein 5 [Source:VGNC Symbol;Acc:VGNC:71921]	0.654454404	1.71E-08	2.36E-07
ENSMMUG00000050271		-0.911413231	0.00094953	0.004333851
ENSMMUG00000053916		0.846127583	1.58E-06	1.45E-05
ENSMMUG00000050841		0.663957708	0.002313499	0.009334259
ENSMMUG00000021393	calcium/calmodulin dependent protein kinase II gamma [Source:VGNC Symbol;Acc:VGNC:70525]	-1.410078851	3.45E-08	4.52E-07
ENSMMUG00000061526	synaptopodin 2 like [Source:NCBI gene;Acc:714736]	1.096599373	1.63E-07	1.90E-06
ENSMMUG00000013696	myozenin 1 [Source:VGNC Symbol;Acc:VGNC:74951]	1.123342599	9.13E-06	7.02E-05
ENSMMUG00000011282	DnaJ heat shock protein family (Hsp40) member C9 [Source:NCBI gene;Acc:707599]	-3.205813435	0.015345642	0.046136124
ENSMMUG00000011274	prolyl 4-hydroxylase subunit alpha 1 [Source:VGNC Symbol;Acc:VGNC:75741]	-0.404297076	0.003652818	0.013778158
ENSMMUG00000055226	DNA damage inducible transcript 4 [Source:NCBI gene;Acc:715307]	-1.877517446	0.000153495	0.000874011
ENSMMUG00000000826	storkhead box 1 [Source:VGNC Symbol;Acc:VGNC:78197]	2.236229612	0.001527497	0.00652519
ENSMMUG00000016652	cell division cycle and apoptosis regulator 1 [Source:NCBI gene;Acc:693585]	0.66398777	0.000191084	0.001061442
ENSMMUG00000023198	tet methylcytosine dioxygenase 1 [Source:VGNC Symbol;Acc:VGNC:79811]	0.412415485	0.004333272	0.015887207
ENSMMUG00000030532	solute carrier family 25 member 16 [Source:NCBI gene;Acc:694145]	0.822538985	1.63E-05	0.000117991
ENSMMUG00000019703	RUN and FYVE domain containing 2 [Source:VGNC Symbol;Acc:VGNC:77057]	0.655656823	0.000986435	0.004475593
ENSMMUG00000050104	Metazoan signal recognition particle RNA [Source:RFAM;Acc:RF00017]	0.826099493	0.000943216	0.004313161
ENSMMUG00000009792	heterogeneous nuclear ribonucleoprotein H3 [Source:VGNC Symbol;Acc:VGNC:73497]	0.758795638	0.000168844	0.000950615
ENSMMUG00000061385		2.207322307	0.001831295	0.007645475
ENSMMUG00000064928		1.906240284	8.21E-05	0.000502266
ENSMMUG00000017727	jumonji domain containing 1C [Source:VGNC Symbol;Acc:VGNC:73819]	-0.938612503	0.002706287	0.010609841
ENSMMUG00000039940		-1.472936871	0.000407176	0.002064566
ENSMMUG00000009794	protocadherin related 15 [Source:VGNC Symbol;Acc:VGNC:75608]	1.4193666	2.99E-23	2.90E-21
ENSMMUG00000007077	APOBEC1 complementation factor [Source:VGNC Symbol;Acc:VGNC:69523]	-0.342776071	0.007466442	0.025371591
ENSMMUG00000007078	N-acylsphingosine amidohydrolase 2 [Source:NCBI gene;Acc:703913]	-0.571512148	1.61E-06	1.48E-05
ENSMMUG00000007081	sphingomyelin synthase 1 [Source:VGNC Symbol;Acc:VGNC:77341]	0.816687392	0.003490281	0.013233756
ENSMMUG00000014343	lipase family member J [Source:VGNC Symbol;Acc:VGNC:74333]	-0.818157012	0.003888645	0.014494675
ENSMMUG00000061761	interferon induced protein with tetratricopeptide repeats 2 [Source:NCBI gene;Acc:695452]	-1.17044736	8.66E-07	8.44E-06
ENSMMUG00000058942		-1.454264886	9.46E-10	1.66E-08

ENSMMUG00000017533	interferon induced protein with tetratricopeptide repeats 1 [Source:NCBI gene;Acc:695683]	0.937631358	0.001006354	0.00455035
ENSMMUG00000059038	5-hydroxytryptamine receptor 7 [Source:NCBI gene;Acc:696317]	-1.498047456	8.39E-08	1.02E-06
ENSMMUG00000000238	HECT domain E3 ubiquitin protein ligase 2 [Source:VGNC Symbol;Acc:VGNC:73246]	-1.90730969	8.80E-13	2.59E-11
ENSMMUG00000058603		-2.514079041	1.83E-07	2.10E-06
ENSMMUG00000059608	fibroblast growth factor binding protein 3 [Source:NCBI gene;Acc:697881]	1.153176867	7.95E-06	6.20E-05
ENSMMUG00000017627	B-TFIID TATA-box binding protein associated factor 1 [Source:VGNC Symbol;Acc:VGNC:70336]	1.635204992	2.73E-25	3.54E-23
ENSMMUG00000004816	membrane associated ring-CH-type finger 5 [Source:VGNC Symbol;Acc:VGNC:74588]	1.059846421	1.62E-10	3.27E-09
ENSMMUG00000011789	hematopoietically expressed homeobox [Source:VGNC Symbol;Acc:VGNC:73376]	1.605301056	4.98E-14	1.80E-12
ENSMMUG00000052637	Metazoan signal recognition particle RNA [Source:RFAM;Acc:RF00017]	2.049461655	0.000104249	0.000619319
ENSMMUG00000014708	centrosomal protein 55 [Source:VGNC Symbol;Acc:VGNC:70935]	0.352050081	0.010542148	0.033815359
ENSMMUG00000014715	fragile site, folic acid type, rare, fra(10)(q23.3) or fra(10)(q24.2) candidate 1 [Source:NCBI gene;Acc:700389]	2.105618992	0.000289952	0.001531069
ENSMMUG00000050975		-1.324154537	6.31E-07	6.38E-06
ENSMMUG00000022658	ectonucleoside triphosphate diphosphohydrolase 1 [Source:HGNC Symbol;Acc:HGNC:3363]	-0.477392538	0.003511435	0.01331049
ENSMMUG00000001518	zinc finger protein 518A [Source:VGNC Symbol;Acc:VGNC:79767]	-0.568599056	1.46E-06	1.35E-05
ENSMMUG00000008687	B cell linker [Source:VGNC Symbol;Acc:VGNC:70306]	-0.759865598	7.49E-12	1.90E-10
ENSMMUG00000001876	phosphoinositide-3-kinase adaptor protein 1 [Source:VGNC Symbol;Acc:VGNC:75891]	-0.747647436	0.000608626	0.002939402
ENSMMUG00000010427	ligand dependent nuclear receptor corepressor [Source:NCBI gene;Acc:705646]	-0.480896249	0.004753367	0.017180433
ENSMMUG00000019634	ribosomal RNA processing 12 homolog [Source:VGNC Symbol;Acc:VGNC:77122]	-0.446299538	0.000232756	0.001262997
ENSMMUG00000015503	zinc finger FYVE-type containing 27 [Source:VGNC Symbol;Acc:VGNC:79623]	0.626914443	1.67E-07	1.94E-06
ENSMMUG00000060995		-2.938470585	0.001912064	0.00794162
ENSMMUG00000020603	pyridine nucleotide-disulphide oxidoreductase domain 2 [Source:NCBI gene;Acc:707797]	0.975719191	4.49E-11	9.87E-10
ENSMMUG00000054904	Metazoan signal recognition particle RNA [Source:RFAM;Acc:RF00017]	1.263938888	2.21E-18	1.38E-16
ENSMMUG00000020604	HPS1 biogenesis of lysosomal organelles complex 3 subunit 1 [Source:VGNC Symbol;Acc:VGNC:73515]	1.18648869	5.98E-17	3.20E-15
ENSMMUG00000049770	Metazoan signal recognition particle RNA [Source:RFAM;Acc:RF00017]	1.566574775	1.46E-06	1.36E-05
ENSMMUG00000020605	heparanase 2 (inactive) [Source:NCBI gene;Acc:708795]	1.019723209	2.61E-08	3.49E-07
ENSMMUG00000060230	Metazoan signal recognition particle RNA [Source:RFAM;Acc:RF00017]	0.77781916	0.006109004	0.021281235
ENSMMUG00000010357	cyclin and CBS domain divalent metal cation transport mediator 1 [Source:NCBI gene;Acc:708335]	1.004454426	1.25E-07	1.49E-06
ENSMMUG00000010356	glutamic-oxaloacetic transaminase 1 [Source:VGNC Symbol;Acc:VGNC:73111]	-2.523944198	0.002367842	0.009503402
ENSMMUG00000063121		1.166369785	0.00752938	0.025513893
ENSMMUG00000003610	sortilin related VPS10 domain containing receptor 3 [Source:VGNC Symbol;Acc:VGNC:77934]	0.664348169	0.008920966	0.029480356
ENSMMUG00000058962		3.913270976	0.001277787	0.005587821
ENSMMUG00000015117	X-prolyl aminopeptidase 1 [Source:VGNC Symbol;Acc:VGNC:78810]	-1.482302677	4.62E-07	4.85E-06
ENSMMUG00000006233	adducin 3 [Source:VGNC Symbol;Acc:VGNC:69531]	1.084924535	0.005757107	0.020282664
ENSMMUG00000014350	MAX interactor 1, dimerization protein [Source:VGNC Symbol;Acc:VGNC:75087]	-0.855317389	8.23E-05	0.000502859
ENSMMUG00000007775	survival motor neuron domain containing 1 [Source:NCBI gene;Acc:694761]	-1.151106552	5.49E-05	0.000352652
ENSMMUG00000057793	dual specificity phosphatase 5 [Source:NCBI gene;Acc:698178]	-2.410516815	0.007489113	0.025426341
ENSMMUG00000017204	programmed cell death 4 [Source:VGNC Symbol;Acc:VGNC:75794]	-3.507796625	4.86E-06	3.99E-05

ENSMMUG00000056056		-3.818104062	0.000118286	0.000693914
ENSMMUG00000017206	SHOC2 leucine rich repeat scaffold protein [Source:VGNC Symbol;Acc:VGNC:81569]	-3.35620377	1.89E-05	0.000135196
ENSMMUG00000003548	tectorin beta [Source:VGNC Symbol;Acc:VGNC:78306]	1.92423118	0.000552628	0.002697634
ENSMMUG00000053789		-0.783323085	0.000321792	0.001674862
ENSMMUG00000001217	caspase 7 [Source:VGNC Symbol;Acc:VGNC:70546]	-0.786098976	0.000194788	0.00107954
ENSMMUG00000055239		0.718375353	1.40E-05	0.000103659
ENSMMUG00000022514	coiled-coil domain containing 186 [Source:VGNC Symbol;Acc:VGNC:70847]	0.648955923	4.30E-11	9.50E-10
ENSMMUG00000022524	actin binding LIM protein 1 [Source:VGNC Symbol;Acc:VGNC:69587]	-0.315320182	0.016478255	0.048939736
ENSMMUG00000058240		-1.325710264	5.13E-05	0.000332168
ENSMMUG00000015414	family with sequence similarity 160 member B1 [Source:VGNC Symbol;Acc:VGNC:72403]	-1.076673054	1.57E-05	0.000114369
ENSMMUG00000001902	TruB pseudouridine synthase family member 1 [Source:VGNC Symbol;Acc:VGNC:79005]	1.014931377	0.008116245	0.027154397
ENSMMUG00000020080	GDNF family receptor alpha 1 [Source:VGNC Symbol;Acc:VGNC:72917]	2.86710254	0.000155879	0.000886895
ENSMMUG00000021610	regulator of G protein signaling 10 [Source:VGNC Symbol;Acc:VGNC:76915]	1.862970781	3.10E-18	1.92E-16
ENSMMUG00000054942		1.274895763	0.001298027	0.005664789
ENSMMUG00000001472	TIA1 cytotoxic granule associated RNA binding protein like 1 [Source:VGNC Symbol;Acc:VGNC:78354]	1.432905989	4.62E-06	3.80E-05
ENSMMUG00000009559	minichromosome maintenance complex binding protein [Source:VGNC Symbol;Acc:VGNC:74583]	1.712421812	1.92E-08	2.63E-07
ENSMMUG00000009561	SEC23 interacting protein [Source:VGNC Symbol;Acc:VGNC:77255]	-1.028873609	0.001530018	0.006534039
ENSMMUG00000064321		1.656964839	4.70E-08	5.99E-07
ENSMMUG00000064557		1.615552813	2.72E-15	1.20E-13
ENSMMUG00000052711		-0.462589735	0.007930374	0.026618297
ENSMMUG00000000684	acyl-CoA dehydrogenase short/branched chain [Source:VGNC Symbol;Acc:VGNC:81343]	-0.986618855	0.015778091	0.04717526
ENSMMUG00000020346	carboxypeptidase X, M14 family member 2 [Source:VGNC Symbol;Acc:VGNC:71596]	0.853992859	1.46E-07	1.72E-06
ENSMMUG00000058933		-1.38164407	0.001130916	0.005024487
ENSMMUG00000008539	uroporphyrinogen III synthase [Source:VGNC Symbol;Acc:VGNC:78731]	-0.705995556	0.000122115	0.000713498
ENSMMUG00000008541	BRCA2 and CDKN1A interacting protein [Source:NCBI gene;Acc:699437]	-0.701815545	0.000133088	0.000771718
ENSMMUG00000008542	DEAH-box helicase 32 (putative) [Source:NCBI gene;Acc:695919]	-0.489822874	0.003921482	0.014590141
ENSMMUG00000053682		1.412878513	0.003641385	0.013740001
ENSMMUG00000013653	lysine demethylase 5A [Source:VGNC Symbol;Acc:VGNC:82169]	0.456824421	5.46E-05	0.000350935
ENSMMUG00000013894	beta-1,4-N-acetyl-galactosaminyltransferase 3 [Source:VGNC Symbol;Acc:VGNC:70121]	-1.495080316	5.25E-05	0.000338577
ENSMMUG00000007405	FKBP prolyl isomerase 4 [Source:VGNC Symbol;Acc:VGNC:72664]	-1.557132218	0.006799683	0.023296487
ENSMMUG00000007411	integrin alpha FG-GAP repeat containing 2 [Source:VGNC Symbol;Acc:VGNC:73690]	-1.813254501	5.30E-14	1.90E-12
ENSMMUG00000007424	nuclear receptor interacting protein 2 [Source:VGNC Symbol;Acc:VGNC:75448]	-1.705771674	2.57E-13	8.11E-12
ENSMMUG00000037675	testis expressed 52 [Source:NCBI gene;Acc:107000723]	-1.190464568	3.60E-15	1.55E-13
ENSMMUG00000061935		3.495840831	2.84E-26	4.13E-24
ENSMMUG00000015189	RAD51 associated protein 1 [Source:VGNC Symbol;Acc:VGNC:76497]	-0.998195181	0.000111218	0.000655098
ENSMMUG00000020811	dual specificity tyrosine phosphorylation regulated kinase 4 [Source:NCBI gene;Acc:710999]	-0.549312469	0.005637418	0.019939452

ENSMMUG00000020812	A-kinase anchoring protein 3 [Source:VGNC Symbol;Acc:VGNC:69769]	-1.674933549	1.28E-05	9.59E-05
ENSMMUG00000020813	NADH:ubiquinone oxidoreductase subunit A9 [Source:VGNC Symbol;Acc:VGNC:81480]	-0.815993126	0.000134501	0.00077867
ENSMMUG00000022893	von Willebrand factor [Source:VGNC Symbol;Acc:VGNC:78778]	3.381470015	1.69E-08	2.34E-07
ENSMMUG00000047465	Metazoan signal recognition particle RNA [Source:RFAM;Acc:RF00017]	5.600435207	3.39E-10	6.52E-09
ENSMMUG00000061974		3.964198219	0.001670593	0.007065876
ENSMMUG00000004470	CD9 molecule [Source:VGNC Symbol;Acc:VGNC:70787]	3.026282758	6.91E-07	6.93E-06
ENSMMUG00000000375	pleckstrin homology and RhoGEF domain containing G6 [Source:VGNC Symbol;Acc:VGNC:76110]	3.061170537	4.70E-51	1.14E-47
ENSMMUG00000053013		1.567404108	1.89E-08	2.60E-07
ENSMMUG00000014265	TAP binding protein like [Source:NCBI gene;Acc:712626]	2.051844624	5.28E-13	1.59E-11
ENSMMUG00000018679	glyceraldehyde-3-phosphate dehydrogenase [Source:NCBI gene;Acc:574353]	-1.070603673	0.000610804	0.002946984
ENSMMUG00000018685	chromodomain helicase DNA binding protein 4 [Source:VGNC Symbol;Acc:VGNC:71029]	-0.483881738	0.006968363	0.023812669
ENSMMUG00000017619	lysophosphatidic acid receptor 5 [Source:VGNC Symbol;Acc:VGNC:74277]	0.580402404	8.74E-06	6.74E-05
ENSMMUG00000016983	acrosin binding protein [Source:VGNC Symbol;Acc:VGNC:69445]	-1.754699254	9.67E-16	4.58E-14
ENSMMUG00000016985	inhibitor of growth family member 4 [Source:VGNC Symbol;Acc:VGNC:73628]	-0.449899877	0.001012689	0.004573301
ENSMMUG00000016990	zinc finger protein 384 [Source:VGNC Symbol;Acc:VGNC:78610]	-0.37934563	0.004448239	0.016240832
ENSMMUG00000050979	PILR alpha associated neural protein [Source:NCBI gene;Acc:713548]	-1.540175015	0.004523142	0.016452619
ENSMMUG00000009583	COP9 signalosome subunit 7A [Source:VGNC Symbol;Acc:VGNC:71185]	1.49415954	0.000694005	0.003298057
ENSMMUG00000010201	atrophin 1 [Source:VGNC Symbol;Acc:VGNC:70157]	0.895747329	8.38E-05	0.000510719
ENSMMUG00000010203	protein tyrosine phosphatase non-receptor type 6 [Source:VGNC Symbol;Acc:VGNC:76607]	0.906371592	0.000343956	0.001774968
ENSMMUG00000010210	lysophosphatidylcholine acyltransferase 3 [Source:VGNC Symbol;Acc:VGNC:74405]	-2.780581746	0.009612109	0.031365034
ENSMMUG00000012786	complement C1s [Source:VGNC Symbol;Acc:VGNC:70297]	-0.531793459	0.000717503	0.003389776
ENSMMUG00000006470	complement C1r subcomponent [Source:NCBI gene;Acc:722133]	0.78286895	1.89E-09	3.15E-08
ENSMMUG00000022109	CD163 molecule like 1 [Source:VGNC Symbol;Acc:VGNC:70914]	1.152067721	1.08E-06	1.04E-05
ENSMMUG00000057570		2.147872541	6.68E-18	3.97E-16
ENSMMUG00000048744		1.522084138	0.001636571	0.006940151
ENSMMUG00000057785		5.259874854	1.66E-07	1.93E-06
ENSMMUG00000061774	taste 2 receptor member 10 [Source:NCBI gene;Acc:717960]	-3.941122247	4.87E-05	0.000316868
ENSMMUG00000050862		-1.023812609	0.000287884	0.001522916
ENSMMUG00000018298	killer cell lectin like receptor D1 [Source:NCBI gene;Acc:574145]	3.729659926	3.61E-08	4.71E-07
ENSMMUG00000015305	C-type lectin domain family 2 member A [Source:VGNC Symbol;Acc:VGNC:71329]	-1.431546661	0.003179994	0.012217766
ENSMMUG00000045446		-3.183500887	6.44E-05	0.000404524
ENSMMUG00000032892	U6 spliceosomal RNA [Source:RFAM;Acc:RF00026]	-1.02010419	0.011455101	0.03624
ENSMMUG00000044047	killer cell lectin like receptor B1 [Source:NCBI gene;Acc:100430783]	-0.927609639	0.000872367	0.004031023
ENSMMUG00000056741	ovostatin-like [Source:NCBI gene;Acc:722294]	2.027355229	0.000683625	0.003258315
ENSMMUG00000003238	pregnancy zone protein [Source:NCBI gene;Acc:100430319]	-0.853034573	0.003860524	0.014423141
ENSMMUG00000022680	alpha-2-macroglobulin like 1 [Source:VGNC Symbol;Acc:VGNC:69507]	-1.127133587	0.000628276	0.00301826
ENSMMUG00000022676	ribosomal modification protein rimK like family member B [Source:NCBI gene;Acc:722261]	-3.157322093	0.002274633	0.009218384

ENSMMUG00000052134		1.175285002	0.002336887	0.009405129
ENSMMUG00000060675		1.127442436	1.83E-06	1.65E-05
ENSMMUG00000021005	taste receptor type 2 member 50 [Source:NCBI gene;Acc:693513]	1.213520733	0.000458656	0.00229834
ENSMMUG00000056428	taste receptor type 2 member 20 [Source:NCBI gene;Acc:693643]	1.113429002	0.001833873	0.007651841
ENSMMUG00000050696	taste receptor type 2 member 31-like [Source:NCBI gene;Acc:693892]	1.247709373	8.61E-07	8.40E-06
ENSMMUG00000054225	taste receptor type 2 member 43 [Source:NCBI gene;Acc:694161]	1.680500902	6.08E-05	0.000384222
ENSMMUG00000042870	Macaca mulatta taste receptor, type 2, member 46 (TAS2R46), mRNA. [Source:RefSeq mRNA;Acc:NM_001080757]	1.282974981	3.51E-05	0.000236088
ENSMMUG00000055308	basic salivary proline-rich protein 2-like [Source:NCBI gene;Acc:695018]	3.636484443	0.000657343	0.003145423
ENSMMUG00000064931		3.529875333	3.78E-09	5.94E-08
ENSMMUG00000014370	ETS variant transcription factor 6 [Source:VGNC Symbol;Acc:VGNC:72444]	-0.75509435	0.006164229	0.021427404
ENSMMUG00000023317	LDL receptor related protein 6 [Source:VGNC Symbol;Acc:VGNC:74446]	-0.670474318	0.009868049	0.032070606
ENSMMUG00000047247	matrix Gla protein [Source:NCBI gene;Acc:574116]	2.286453547	0.000486968	0.002414414
ENSMMUG00000018030	endoplasmic reticulum protein 27 [Source:VGNC Symbol;Acc:VGNC:72369]	1.88629792	2.01E-08	2.74E-07
ENSMMUG00000053781	Rho GDP dissociation inhibitor beta [Source:NCBI gene;Acc:702320]	2.450147029	5.06E-07	5.26E-06
ENSMMUG00000050529	phosphodiesterase 6H [Source:NCBI gene;Acc:700447]	2.277235679	0.00039505	0.002006586
ENSMMUG00000022176	capping actin protein of muscle Z-line subunit alpha 3 [Source:VGNC Symbol;Acc:VGNC:70618]	-1.857763516	0.010758045	0.034416748
ENSMMUG00000049682	Metazoan signal recognition particle RNA [Source:RFAM;Acc:RF00017]	-4.303161312	5.32E-07	5.49E-06
ENSMMUG00000048030	ferritin, heavy polypeptide 1-like [Source:NCBI gene;Acc:707011]	-1.874303334	0.016204457	0.048241695
ENSMMUG00000020370	SRY-box transcription factor 5 [Source:VGNC Symbol;Acc:VGNC:77843]	-2.59373845	1.91E-05	0.000136105
ENSMMUG00000057276		-2.474676406	2.85E-05	0.000195707
ENSMMUG00000038076	Metazoan signal recognition particle RNA [Source:RFAM;Acc:RF00017]	1.161044013	0.000440362	0.002218136
ENSMMUG00000060493		0.810256739	1.01E-08	1.47E-07
ENSMMUG00000012223	branched chain amino acid transaminase 1 [Source:VGNC Symbol;Acc:VGNC:70236]	0.501304344	1.39E-05	0.000102819
ENSMMUG00000053657		-3.229364845	3.62E-05	0.000242861
ENSMMUG00000018630	sarcospan [Source:VGNC Symbol;Acc:VGNC:77887]	0.485196423	0.000456898	0.002290319
ENSMMUG00000060495		0.473784364	0.00384189	0.014357214
ENSMMUG00000007050		0.785457863	0.00573064	0.02019547
ENSMMUG00000050627		0.415326007	0.000759808	0.00356416
ENSMMUG00000023128	inositol 1,4,5-trisphosphate receptor type 2 [Source:VGNC Symbol;Acc:VGNC:73805]	0.370251218	0.002050719	0.008440346
ENSMMUG00000006468	FGFR1 oncogene partner 2 [Source:VGNC Symbol;Acc:VGNC:72551]	1.693711882	5.60E-15	2.35E-13
ENSMMUG00000003217	transmembrane 7 superfamily member 3 [Source:VGNC Symbol;Acc:VGNC:79371]	1.715108924	4.18E-18	2.57E-16
ENSMMUG00000023248	serine/threonine kinase 38 like [Source:VGNC Symbol;Acc:VGNC:78156]	-0.978593754	0.000350282	0.00180441
ENSMMUG00000023249	aryl hydrocarbon receptor nuclear translocator like 2 [Source:NCBI gene;Acc:709547]	-1.109792268	2.73E-09	4.40E-08
ENSMMUG00000021736	single-pass membrane protein with coiled-coil domains 2 [Source:VGNC Symbol;Acc:VGNC:77808]	0.81548134	1.21E-05	9.12E-05
ENSMMUG00000001361	importin 8 [Source:VGNC Symbol;Acc:VGNC:73815]	-0.719976715	0.008164759	0.027297863
ENSMMUG00000001359	caprin family member 2 [Source:VGNC Symbol;Acc:VGNC:70599]	-0.513636916	0.0102865	0.033193086
ENSMMUG00000001357	tetraspanin 11 [Source:VGNC Symbol;Acc:VGNC:79882]	-1.211352555	6.93E-24	7.20E-22
ENSMMUG00000009155	SIN3-HDAC complex associated factor [Source:NCBI gene;Acc:694162]	-3.210702158	4.23E-10	8.03E-09
ENSMMUG00000039300		-2.838419065	1.73E-06	1.57E-05
ENSMMUG00000003221	DENN domain containing 5B [Source:VGNC Symbol;Acc:VGNC:71772]	-1.005909234	0.000105489	0.00062566

ENSMMUG00000063112		1.458952669	7.84E-05	0.000482128
ENSMMUG00000056303		-1.521478274	0.002301279	0.009297621
ENSMMUG00000000205	transmembrane protein 117 [Source:VGNC Symbol;Acc:VGNC:79488]	-1.04435489	1.54E-18	9.92E-17
ENSMMUG00000060215	Y RNA [Source:RFAM;Acc:RF00019]	-0.617746705	0.000907796	0.004169553
ENSMMUG00000052030	developing brain homeobox 2 [Source:NCBI gene;Acc:701170]	-0.929061657	7.80E-08	9.53E-07
ENSMMUG00000015962	AT-rich interaction domain 2 [Source:VGNC Symbol;Acc:VGNC:69858]	-1.071595298	1.04E-05	7.93E-05
ENSMMUG00000023791	SR-related CTD associated factor 11 [Source:VGNC Symbol;Acc:VGNC:77002]	-2.532465461	1.28E-05	9.61E-05
ENSMMUG00000022359	solute carrier family 38 member 2 [Source:VGNC Symbol;Acc:VGNC:77469]	-0.452633938	0.00825969	0.027570864
ENSMMUG00000064730		2.641213669	2.03E-20	1.60E-18
ENSMMUG00000012331	solute carrier family 38 member 4 [Source:VGNC Symbol;Acc:VGNC:77484]	-0.504243406	0.007781085	0.02620798
ENSMMUG00000049514	solute carrier family 48 member 1 [Source:NCBI gene;Acc:704304]	0.550335508	0.003922286	0.014590141
ENSMMUG00000021655	histone deacetylase 7 [Source:NCBI gene;Acc:718225]	0.679402225	1.53E-08	2.14E-07
ENSMMUG00000006727	vitamin D receptor [Source:VGNC Symbol;Acc:VGNC:78764]	0.529865197	2.15E-05	0.000152434
ENSMMUG00000020125	SUMO specific peptidase 1 [Source:NCBI gene;Acc:707603]	-1.460411681	0.006325014	0.021918171
ENSMMUG00000014484	zinc finger protein 641 [Source:NCBI gene;Acc:708246]	-2.191075853	6.46E-13	1.92E-11
ENSMMUG00000003877	KAT8 regulatory NSL complex subunit 2 [Source:VGNC Symbol;Acc:VGNC:73910]	-1.448572469	3.25E-06	2.78E-05
ENSMMUG00000054025	testis expressed 49 [Source:NCBI gene;Acc:709603]	-0.665531896	0.001098698	0.004893312
ENSMMUG00000012769	limb development membrane protein 1 like [Source:VGNC Symbol;Acc:VGNC:74310]	-0.540394947	0.010206539	0.032964331
ENSMMUG00000021081	tubulin alpha 1b [Source:NCBI gene;Acc:709643]	-0.595488857	0.004092996	0.015120682
ENSMMUG00000028047	Y RNA [Source:RFAM;Acc:RF00019]	-1.429275382	0.0145269	0.044105586
ENSMMUG00000009788	tubulin alpha-1C chain [Source:NCBI gene;Acc:710110]	1.409231286	1.98E-09	3.29E-08
ENSMMUG00000031976	peripherin [Source:NCBI gene;Acc:709827]	1.139541524	0.003737769	0.014043927
ENSMMUG00000007544	trophinin associated protein [Source:VGNC Symbol;Acc:VGNC:78650]	0.928201482	9.41E-06	7.23E-05
ENSMMUG00000007546	complement C1q like 4 [Source:VGNC Symbol;Acc:VGNC:70450]	1.17210075	0.000558839	0.002724293
ENSMMUG00000007548	DnaJ heat shock protein family (Hsp40) member C22 [Source:NCBI gene;Acc:710205]	1.264426013	8.90E-05	0.000539117
ENSMMUG00000064430		0.721288232	0.002545337	0.01011248
ENSMMUG00000003762	spermatogenesis associated serine rich 2 [Source:VGNC Symbol;Acc:VGNC:77958]	-0.397749778	0.001196093	0.005278566
ENSMMUG00000049374		0.678935907	0.002928548	0.011398207
ENSMMUG00000005960	transmembrane BAX inhibitor motif containing 6 [Source:NCBI gene;Acc:677702]	0.211939831	0.005064071	0.018167916
ENSMMUG00000038256	aquaporin 2 [Source:NCBI gene;Acc:711719]	0.998803468	2.26E-18	1.41E-16
ENSMMUG00000058855	aquaporin 5 [Source:NCBI gene;Acc:711804]	-0.909252592	0.002693677	0.010566104
ENSMMUG00000006529	acid sensing ion channel subunit 1 [Source:VGNC Symbol;Acc:VGNC:70035]	-0.755897947	0.008342777	0.027797147
ENSMMUG00000006531	SWI/SNF related, matrix associated, actin dependent regulator of chromatin, subfamily d, member 1 [Source:VGNC Symbol;Acc:VGNC:77653]	-0.757151704	0.003440574	0.01308282
ENSMMUG00000018485	methyltransferase like 7A [Source:VGNC Symbol;Acc:VGNC:74567]	1.185850599	0.001060835	0.004758212
ENSMMUG00000000152	transcription factor CP2 [Source:VGNC Symbol;Acc:VGNC:78323]	2.075126709	1.12E-06	1.06E-05
ENSMMUG00000044229	POU class 6 homeobox 1 [Source:NCBI gene;Acc:694640]	3.111760524	0.014686318	0.044515222
ENSMMUG00000004982	activin A receptor like type 1 [Source:VGNC Symbol;Acc:VGNC:69451]	3.377912282	0.000146082	0.00083804

ENSMMUG00000022922	nuclear receptor subfamily 4 group A member 1 [Source:VGNC Symbol;Acc:VGNC:75517]	-0.532458246	2.00E-05	0.000142247
ENSMMUG00000015392	autophagy related 101 [Source:VGNC Symbol;Acc:VGNC:70132]	1.714834743	0.008327502	0.027765345
ENSMMUG00000010000	keratin 7 [Source:VGNC Symbol;Acc:VGNC:74214]	-0.717574754	4.58E-13	1.40E-11
ENSMMUG00000064514	keratin, type II cuticular Hb1 [Source:NCBI gene;Acc:697511]	-1.122893936	2.03E-11	4.81E-10
ENSMMUG00000018216	keratin, type II cuticular Hb5 [Source:NCBI gene;Acc:698320]	-1.237580231	0.002481446	0.009899227
ENSMMUG00000018217	keratin 84 [Source:VGNC Symbol;Acc:VGNC:74220]	-1.150555775	0.00185705	0.007737443
ENSMMUG00000057134	keratin, type II cytoskeletal 6C [Source:NCBI gene;Acc:100424162]	0.936193218	0.002653731	0.010446057
ENSMMUG00000055016	keratin, type II cytoskeletal 6A [Source:NCBI gene;Acc:719283]	0.522589374	8.25E-08	1.00E-06
ENSMMUG00000014980	keratin 76 [Source:VGNC Symbol;Acc:VGNC:74112]	-1.481523083	7.58E-05	0.000468191
ENSMMUG00000005246	zinc finger protein 740 [Source:VGNC Symbol;Acc:VGNC:79665]	-1.720347253	7.90E-11	1.69E-09
ENSMMUG00000052817	Sp7 transcription factor [Source:NCBI gene;Acc:702575]	-1.571098209	4.91E-07	5.12E-06
ENSMMUG00000020036	Sp1 transcription factor [Source:VGNC Symbol;Acc:VGNC:77844]	-1.171625981	2.84E-05	0.000195132
ENSMMUG00000018492	TARBP2 subunit of RISC loading complex [Source:VGNC Symbol;Acc:VGNC:78099]	1.139487149	1.98E-06	1.77E-05
ENSMMUG00000022313	activating transcription factor 7 [Source:NCBI gene;Acc:700403]	1.241661665	3.66E-06	3.09E-05
ENSMMUG00000055610	homeobox C4 [Source:NCBI gene;Acc:100430434]	-2.493051674	1.15E-18	7.58E-17
ENSMMUG00000052854	mucin like 1 [Source:NCBI gene;Acc:100423942]	1.186201612	5.67E-13	1.70E-11
ENSMMUG00000016203	thymocyte expressed, positive selection associated 1 [Source:VGNC Symbol;Acc:VGNC:79775]	0.448994335	0.005401355	0.019207313
ENSMMUG00000057094	olfactory receptor, family 6, subfamily C, member 4 [Source:NCBI gene;Acc:708807]	-1.192840564	0.012453984	0.038858891
ENSMMUG00000055590		-0.920288582	1.57E-06	1.45E-05
ENSMMUG00000006293	SAP domain containing ribonucleoprotein [Source:NCBI gene;Acc:709932]	0.791861783	2.21E-09	3.62E-08
ENSMMUG00000053458	ORMDL sphingolipid biosynthesis regulator 2 [Source:NCBI gene;Acc:710296]	0.703524128	2.61E-06	2.27E-05
ENSMMUG00000051937	uncharacterized LOC707807 [Source:NCBI gene;Acc:707807]	0.592218716	0.014351375	0.043700447
ENSMMUG00000006295	matrix metallopeptidase 19 [Source:VGNC Symbol;Acc:VGNC:74896]	0.666189619	0.000702964	0.00333518
ENSMMUG00000064364	sulfite oxidase [Source:NCBI gene;Acc:711164]	-1.244307058	6.54E-06	5.20E-05
ENSMMUG00000064075	ring finger protein 41 [Source:NCBI gene;Acc:711969]	-1.007581727	5.73E-05	0.000365025
ENSMMUG00000054020	U6 spliceosomal RNA [Source:RFAM;Acc:RF00026]	-0.957101296	0.004505797	0.016399793
ENSMMUG00000059663		-0.963088737	8.33E-07	8.16E-06
ENSMMUG00000031834	coenzyme Q10A [Source:VGNC Symbol;Acc:VGNC:71356]	-0.798895623	0.015790567	0.047193149
ENSMMUG00000002483	citrate synthase [Source:VGNC Symbol;Acc:VGNC:71509]	1.155233838	0.000120731	0.000706266
ENSMMUG00000013482	poly(A) specific ribonuclease subunit PAN2 [Source:NCBI gene;Acc:712478]	1.653628253	0.016584149	0.049220861
ENSMMUG00000000803	signal transducer and activator of transcription 2 [Source:VGNC Symbol;Acc:VGNC:77905]	2.039261786	2.55E-09	4.15E-08
ENSMMUG00000063898	apolipoprotein F [Source:NCBI gene;Acc:712761]	2.398722658	5.47E-11	1.19E-09
ENSMMUG00000053460		1.874238585	8.22E-08	1.00E-06
ENSMMUG00000021878	timeless circadian regulator [Source:VGNC Symbol;Acc:VGNC:78356]	-1.137993117	1.36E-07	1.60E-06
ENSMMUG00000013766	prostaglandin E synthase 3 [Source:NCBI gene;Acc:713353]	0.584296183	0.001815961	0.007585811
ENSMMUG00000004522	DNA primase subunit 1 [Source:NCBI gene;Acc:710253]	0.45970185	9.18E-05	0.000553669
ENSMMUG00000057617	hydroxysteroid 17-beta dehydrogenase 6 [Source:NCBI gene;Acc:713549]	0.91412929	0.010845771	0.034644029

ENSMMUG00000043411	zinc finger and BTB domain containing 39 [Source:NCBI gene;Acc:713914]	-1.336823425	1.26E-05	9.43E-05
ENSMMUG00000021114	G protein-coupled receptor 182 [Source:VGNC Symbol;Acc:VGNC:72936]	-1.271167268	4.29E-08	5.51E-07
ENSMMUG00000003203	tachykinin precursor 3 [Source:VGNC Symbol;Acc:VGNC:78079]	-2.079846524	0.004154513	0.015309039
ENSMMUG00000013518	myosin IA [Source:VGNC Symbol;Acc:VGNC:75110]	-0.460618022	0.00022803	0.001241007
ENSMMUG00000021782	LDL receptor related protein 1 [Source:VGNC Symbol;Acc:VGNC:74442]	-0.941964217	7.08E-11	1.52E-09
ENSMMUG00000004233	leucine rich repeats and immunoglobulin like domains 3 [Source:NCBI gene;Acc:716344]	-1.223819017	2.21E-11	5.17E-10
ENSMMUG00000055477		-1.466926489	7.60E-12	1.93E-10
ENSMMUG00000001210	elongation factor 1-alpha 1 [Source:NCBI gene;Acc:100425937]	-2.573959491	0.015846424	0.047330896
ENSMMUG00000063744		4.206345192	0.000363568	0.001862949
ENSMMUG00000062139		1.856485282	6.99E-07	7.00E-06
ENSMMUG00000011579	TAFA chemokine like family member 2 [Source:VGNC Symbol;Acc:VGNC:78189]	0.541949648	3.61E-06	3.05E-05
ENSMMUG00000020533	chromosome 11 C12orf56 homolog [Source:VGNC Symbol;Acc:VGNC:70409]	-4.338393208	4.21E-15	1.80E-13
ENSMMUG00000007658	TANK binding kinase 1 [Source:VGNC Symbol;Acc:VGNC:78107]	-0.256372707	0.006076324	0.021194755
ENSMMUG00000003669	methionine sulfoxide reductase B3 [Source:VGNC Symbol;Acc:VGNC:74842]	0.816333102	2.60E-07	2.89E-06
ENSMMUG00000041729		1.18216614	1.64E-05	0.000118766
ENSMMUG00000011650	dual specificity tyrosine phosphorylation regulated kinase 2 [Source:VGNC Symbol;Acc:VGNC:72018]	1.431971973	1.95E-05	0.000139374
ENSMMUG00000055447	MDM2 proto-oncogene [Source:NCBI gene;Acc:718284]	-1.003086826	5.74E-13	1.72E-11
ENSMMUG00000053722		2.7384487	5.56E-06	4.50E-05
ENSMMUG00000010819	fibroblast growth factor receptor substrate 2 [Source:VGNC Symbol;Acc:VGNC:72732]	-1.096388362	2.86E-11	6.54E-10
ENSMMUG00000010820	chaperonin containing TCP1 subunit 2 [Source:VGNC Symbol;Acc:VGNC:70745]	-2.705826013	9.79E-14	3.35E-12
ENSMMUG00000054953	leucine rich repeat containing 10 [Source:NCBI gene;Acc:114669753]	-4.223665419	7.46E-07	7.40E-06
ENSMMUG00000012290	GLIPR1 like 2 [Source:VGNC Symbol;Acc:VGNC:81413]	-0.79730228	0.01180527	0.037097538
ENSMMUG00000059985		3.960544376	1.63E-05	0.000118335
ENSMMUG00000005530	synaptotagmin 1 [Source:VGNC Symbol;Acc:VGNC:78074]	0.816133155	0.004918189	0.017710112
ENSMMUG00000017463		-2.925508353	0.007911311	0.026560446
ENSMMUG00000038410		-1.319641232	0.001609669	0.006844037
ENSMMUG00000055284		-1.333368546	0.013154393	0.040625751
ENSMMUG00000058404		-0.511804482	0.006216919	0.021583555
ENSMMUG00000049117	CLLU1 antisense RNA 1 [Source:NCBI gene;Acc:100424000]	-3.812821548	0.000370904	0.001895197
ENSMMUG00000002243	pleckstrin homology and RhoGEF domain containing G7 [Source:VGNC Symbol;Acc:VGNC:76149]	-0.908706712	2.35E-08	3.18E-07
ENSMMUG00000002244	early endosome antigen 1 [Source:VGNC Symbol;Acc:VGNC:72161]	-0.829721689	3.26E-06	2.78E-05
ENSMMUG00000052496		-0.863602407	1.82E-13	5.89E-12
ENSMMUG00000055071	Y RNA [Source:RFAM;Acc:RF00019]	-1.371417761	0.011655209	0.036713278

ENSMMUG00000018237	plexin C1 [Source:VGNC Symbol;Acc:VGNC:76190]	1.396371353	1.25E-15	5.84E-14
ENSMMUG00000018239	centrosomal protein 83 [Source:VGNC Symbol;Acc:VGNC:70992]	1.504416056	7.30E-14	2.54E-12
ENSMMUG00000018901	FYVE, RhoGEF and PH domain containing 6 [Source:VGNC Symbol;Acc:VGNC:72548]	-0.657120481	0.000349825	0.001802924
ENSMMUG00000062006	methionyl aminopeptidase 2 [Source:NCBI gene;Acc:712400]	-0.907695549	0.000213125	0.001170899
ENSMMUG00000009303	coiled-coil domain containing 38 [Source:VGNC Symbol;Acc:VGNC:70880]	-1.171552122	3.60E-06	3.04E-05
ENSMMUG00000009308	histidine ammonia-lyase [Source:VGNC Symbol;Acc:VGNC:73344]	2.879176229	1.63E-06	1.49E-05
ENSMMUG00000009312	leukotriene A4 hydrolase [Source:VGNC Symbol;Acc:VGNC:74453]	2.330679691	9.43E-27	1.47E-24
ENSMMUG00000053376		2.777464438	9.18E-20	6.67E-18
ENSMMUG00000050585		2.577421906	1.63E-14	6.39E-13
ENSMMUG00000022404	apoptotic peptidase activating factor 1 [Source:VGNC Symbol;Acc:VGNC:69975]	0.424862429	4.89E-05	0.00031818
ENSMMUG00000022406	ankyrin repeat and sterile alpha motif domain containing 1B [Source:VGNC Symbol;Acc:VGNC:69689]	1.07834174	1.40E-10	2.86E-09
ENSMMUG00000061260	Spi-C transcription factor [Source:NCBI gene;Acc:695809]	-1.095681357	1.21E-09	2.08E-08
ENSMMUG00000042077	DNA damage regulated autophagy modulator 1 [Source:NCBI gene;Acc:697427]	0.834731509	6.04E-13	1.81E-11
ENSMMUG00000046478	WASH complex subunit 3 [Source:NCBI gene;Acc:697824]	2.366892916	2.52E-22	2.34E-20
ENSMMUG00000061923		2.177162703	6.84E-06	5.44E-05
ENSMMUG00000005504	nucleoporin 37 [Source:VGNC Symbol;Acc:VGNC:75491]	2.29230438	7.03E-08	8.69E-07
ENSMMUG00000005506	PARP1 binding protein [Source:VGNC Symbol;Acc:VGNC:75682]	0.627936606	0.000166737	0.000939842
ENSMMUG00000038958	Metazoan signal recognition particle RNA [Source:RFAM;Acc:RF00017]	-2.140546324	0.006076889	0.021194755
ENSMMUG00000023469	heat shock protein 90 beta family member 1 [Source:NCBI gene;Acc:699568]	-0.936693424	0.01596875	0.047627765
ENSMMUG00000021291	thymine DNA glycosylase [Source:VGNC Symbol;Acc:VGNC:78297]	-0.998287711	0.000134078	0.000776531
ENSMMUG00000021292	glycosyltransferase 8 domain containing 2 [Source:VGNC Symbol;Acc:VGNC:73004]	-1.031607371	0.005987137	0.020936941
ENSMMUG00000022570	nuclear transcription factor Y subunit beta [Source:NCBI gene;Acc:700451]	-0.623453853	0.000174227	0.000977515
ENSMMUG00000012091	thioredoxin reductase 1 [Source:VGNC Symbol;Acc:VGNC:78679]	-0.679088211	8.39E-06	6.50E-05
ENSMMUG00000017127	carbohydrate sulfotransferase 11 [Source:VGNC Symbol;Acc:VGNC:82123]	0.974633704	0.000713162	0.003372844
ENSMMUG00000017129	solute carrier family 41 member 2 [Source:VGNC Symbol;Acc:VGNC:77585]	1.026390801	9.13E-09	1.35E-07
ENSMMUG00000052372		-1.256526964	0.00084979	0.003942995
ENSMMUG00000009519	WASH complex subunit 4 [Source:VGNC Symbol;Acc:VGNC:79783]	-1.159007938	1.69E-06	1.54E-05
ENSMMUG00000009524	adaptor protein, phosphotyrosine interacting with PH domain and leucine zipper 2 [Source:VGNC Symbol;Acc:VGNC:69861]	-1.032258517	3.51E-09	5.55E-08
ENSMMUG00000005208	RNA polymerase III subunit B [Source:VGNC Symbol;Acc:VGNC:76143]	2.093808705	1.33E-08	1.90E-07
ENSMMUG00000022118	regulatory factor X4 [Source:VGNC Symbol;Acc:VGNC:76909]	-0.944249428	0.012655643	0.039310914
ENSMMUG00000022278	mitochondrial transcription termination factor 2 [Source:VGNC Symbol;Acc:VGNC:74868]	-1.378680416	0.00013304	0.000771718
ENSMMUG00000021808	cryptochrome circadian regulator 1 [Source:VGNC Symbol;Acc:VGNC:71499]	-0.467158176	0.001386465	0.006003884
ENSMMUG00000015540	BTB domain containing 11 [Source:VGNC Symbol;Acc:VGNC:70367]	-1.564891869	6.30E-05	0.00039657
ENSMMUG00000015544	PWP1 homolog, endonuclein [Source:NCBI gene;Acc:705262]	-2.131808414	0.001832083	0.007646567
ENSMMUG00000015550	PR/SET domain 4 [Source:VGNC Symbol;Acc:VGNC:76393]	-1.889866539	7.29E-07	7.24E-06

ENSMMUG00000021049	ret finger protein-like 3 [Source:NCBI gene;Acc:705507]	- 1.997451779	0.002667045	0.010484271
ENSMMUG00000017124	spliceosome associated factor 3, U4/U6 recycling protein [Source:VGNC Symbol;Acc:VGNC:77029]	1.14517227	0.00171936	0.007248937
ENSMMUG00000054621		1.994533324	0.005826819	0.020460017
ENSMMUG00000006893	D-amino acid oxidase [Source:VGNC Symbol;Acc:VGNC:71729]	1.009510857	3.02E-07	3.30E-06
ENSMMUG00000015108	SV2 related protein [Source:VGNC Symbol;Acc:VGNC:78208]	0.539769546	0.002039872	0.008407603
ENSMMUG00000056729	U6 spliceosomal RNA [Source:RFAM;Acc:RF00026]	0.833128211	2.82E-05	0.000194489
ENSMMUG00000023032	intraflagellar transport 81 [Source:VGNC Symbol;Acc:VGNC:73389]	0.916122777	8.35E-07	8.16E-06
ENSMMUG00000001739	ATPase sarcoplasmic/endoplasmic reticulum Ca2+ transporting 2 [Source:VGNC Symbol;Acc:VGNC:70178]	0.866735367	1.17E-08	1.69E-07
ENSMMUG00000003297	coiled-coil domain containing 63 [Source:VGNC Symbol;Acc:VGNC:70879]	0.897165704	2.10E-05	0.000149035
ENSMMUG00000002347	myosin light chain 2 [Source:VGNC Symbol;Acc:VGNC:75103]	0.874514971	0.000925981	0.004242355
ENSMMUG00000008336	acyl-CoA dehydrogenase family member 10 [Source:NCBI gene;Acc:711080]	0.760535395	1.91E-12	5.33E-11
ENSMMUG00000053496		0.717240651	0.002178048	0.008887387
ENSMMUG00000060754	Metazoan signal recognition particle RNA [Source:RFAM;Acc:RF00017]	0.876810756	1.33E-08	1.89E-07
ENSMMUG00000060917		0.549141276	0.009948266	0.032287997
ENSMMUG00000005255	TRAF-type zinc finger domain containing 1 [Source:VGNC Symbol;Acc:VGNC:79459]	1.041721222	2.70E-06	2.35E-05
ENSMMUG00000012778	rabphilin 3A [Source:VGNC Symbol;Acc:VGNC:77033]	-1.18815322	1.11E-06	1.06E-05
ENSMMUG00000004435	two pore segment channel 1 [Source:VGNC Symbol;Acc:VGNC:79117]	3.071842264	2.48E-19	1.75E-17
ENSMMUG00000059861	Metazoan signal recognition particle RNA [Source:RFAM;Acc:RF00017]	3.699980521	0.00773922	0.026079054
ENSMMUG00000006723	solute carrier family 8 member B1 [Source:NCBI gene;Acc:714561]	2.858680446	1.28E-22	1.22E-20
ENSMMUG00000004205	phospholipase B domain containing 2 [Source:VGNC Symbol;Acc:VGNC:76122]	- 2.419147839	0.000413492	0.002093673
ENSMMUG00000004208	serine dehydratase [Source:VGNC Symbol;Acc:VGNC:77148]	- 2.038570144	2.57E-05	0.00017858
ENSMMUG00000051363		1.126044152	3.28E-05	0.000222273
ENSMMUG00000054768		0.860064497	4.83E-06	3.97E-05
ENSMMUG00000057506		0.664658646	4.34E-05	0.000287013
ENSMMUG00000052680		0.653538261	5.24E-05	0.000338069
ENSMMUG00000017478	tescalcin [Source:VGNC Symbol;Acc:VGNC:79571]	- 0.673130858	0.001089883	0.004860008
ENSMMUG00000014027	nitric oxide synthase 1 [Source:VGNC Symbol;Acc:VGNC:75368]	- 1.372834507	1.75E-05	0.000126042
ENSMMUG00000062615		- 1.286852953	0.00065664	0.003143092
ENSMMUG00000005421	kinase suppressor of ras 2 [Source:VGNC Symbol;Acc:VGNC:74075]	- 1.162873093	0.000352738	0.001815133
ENSMMUG00000020629	SDS3 homolog, SIN3A corepressor complex component [Source:NCBI gene;Acc:696166]	0.873484331	2.98E-09	4.77E-08
ENSMMUG00000053710		0.831956909	7.74E-09	1.16E-07
ENSMMUG00000011209	BICD family like cargo adaptor 1 [Source:HGNC Symbol;Acc:HGNC:28095]	0.945429251	8.24E-05	0.00050335
ENSMMUG00000055209		0.876503199	2.84E-07	3.12E-06
ENSMMUG00000046665	glutamyl-tRNA amidotransferase subunit C [Source:NCBI gene;Acc:702258]	- 0.888004082	0.014662304	0.044460975
ENSMMUG00000004223	ring finger protein 10 [Source:VGNC Symbol;Acc:VGNC:76702]	- 0.766698159	2.52E-06	2.20E-05
ENSMMUG00000055513		- 1.628152245	0.008287997	0.027652655
ENSMMUG00000058536		2.062103411	2.33E-27	3.94E-25
ENSMMUG00000002536	calcium binding protein 1 [Source:VGNC Symbol;Acc:VGNC:70495]	1.729531932	8.44E-10	1.50E-08
ENSMMUG00000039520	chromosome 11 C12orf43 homolog [Source:NCBI gene;Acc:698821]	0.544761433	0.011125981	0.035368114
ENSMMUG00000006441	2'-5'-oligoadenylate synthetase like [Source:VGNC Symbol;Acc:VGNC:75572]	- 0.691756343	0.009774442	0.031830437
ENSMMUG00000021564	lysine demethylase 2B [Source:VGNC Symbol;Acc:VGNC:81425]	4.308539054	1.48E-46	2.39E-43
ENSMMUG00000059782		4.982135786	9.12E-48	1.89E-44

ENSMMUG00000038707	U6 spliceosomal RNA [Source:RFAM;Acc:RF00026]	3.581144635	0.003340899	0.012761128
ENSMMUG00000041124	MORN repeat containing 3 [Source:NCBI gene;Acc:700317]	0.830148744	0.001667549	0.007057105
ENSMMUG00000063813	Metazoan signal recognition particle RNA [Source:RFAM;Acc:RF00017]	1.167251177	0.012568442	0.039132029
ENSMMUG00000031504	transmembrane protein 120B [Source:VGNC Symbol;Acc:VGNC:79917]	0.355104048	0.003712626	0.013960282
ENSMMUG00000016454	4-hydroxyphenylpyruvate dioxygenase [Source:VGNC Symbol;Acc:VGNC:73511]	0.715982215	7.30E-07	7.24E-06
ENSMMUG00000013643	zinc finger CCHC-type containing 8 [Source:VGNC Symbol;Acc:VGNC:79487]	-1.771559427	0.001812191	0.007576593
ENSMMUG00000004412	arginine and serine rich coiled-coil 2 [Source:VGNC Symbol;Acc:VGNC:77139]	-2.856529689	0.000271475	0.001449294
ENSMMUG00000005301	ATP binding cassette subfamily B member 9 [Source:VGNC Symbol;Acc:VGNC:81341]	-1.692743163	1.49E-11	3.60E-10
ENSMMUG00000015926	2-oxoglutarate and iron dependent oxygenase domain containing 2 [Source:VGNC Symbol;Acc:VGNC:75462]	1.163314176	1.37E-07	1.61E-06
ENSMMUG00000015930	ADP ribosylation factor like GTPase 6 interacting protein 4 [Source:VGNC Symbol;Acc:VGNC:69860]	1.121627702	0.006210658	0.021573319
ENSMMUG00000015931	phosphatidylinositol transfer protein membrane associated 2 [Source:VGNC Symbol;Acc:VGNC:75843]	1.15130932	2.24E-07	2.52E-06
ENSMMUG00000031495	chromosome 11 C12orf65 homolog [Source:NCBI gene;Acc:708974]	-2.02724584	0.000222612	0.001216128
ENSMMUG00000000434	cyclin dependent kinase 2 associated protein 1 [Source:VGNC Symbol;Acc:VGNC:70944]	-1.292053723	0.000198337	0.001097119
ENSMMUG00000013514	lysine methyltransferase 5A [Source:VGNC Symbol;Acc:VGNC:74159]	-0.414563915	0.009506389	0.03108985
ENSMMUG00000023048	cytochrome b-c1 complex subunit Rieske, mitochondrial [Source:NCBI gene;Acc:705178]	1.692464184	1.95E-06	1.75E-05
ENSMMUG00000058836	transmembrane p24 trafficking protein 2 [Source:NCBI gene;Acc:106992483]	-0.788927625	8.52E-05	0.000518474
ENSMMUG00000064225	tectonic family member 2 [Source:NCBI gene;Acc:721415]	0.416528647	0.001742176	0.007319657
ENSMMUG00000006546	ATPase H+ transporting V0 subunit a2 [Source:VGNC Symbol;Acc:VGNC:70096]	-0.383666472	0.000212642	0.001168688
ENSMMUG00000006861	dynein axonemal heavy chain 10 [Source:NCBI gene;Acc:710343]	-0.61144685	7.86E-06	6.14E-05
ENSMMUG00000020804	transmembrane protein 132B [Source:NCBI gene;Acc:707118]	-1.206732386	8.54E-05	0.00051901
ENSMMUG00000050145	Metazoan signal recognition particle RNA [Source:RFAM;Acc:RF00017]	-2.962174872	0.006953254	0.023766625
ENSMMUG00000020926	syntaxin 2 [Source:VGNC Symbol;Acc:VGNC:78259]	-3.157393883	2.55E-06	2.22E-05
ENSMMUG00000058674		-2.958120997	0.012432767	0.038801016
ENSMMUG00000017026	fibrosin like 1 [Source:NCBI gene;Acc:696295]	0.441292083	0.01349562	0.041512024
ENSMMUG00000015463	DNA polymerase epsilon, catalytic subunit [Source:NCBI gene;Acc:698023]	-0.511364707	0.000618864	0.002979937
ENSMMUG00000031451	golgin A3 [Source:VGNC Symbol;Acc:VGNC:73106]	1.269514297	0.003074503	0.011872646
ENSMMUG00000015468	checkpoint with forkhead and ring finger domains [Source:VGNC Symbol;Acc:VGNC:71064]	3.194487228	1.75E-10	3.53E-09
ENSMMUG00000021159	tubulin alpha 3e [Source:NCBI gene;Acc:718034]	-0.770301493	0.003715607	0.013964265
ENSMMUG00000049232	AMMECR1 like [Source:VGNC Symbol;Acc:VGNC:84208]	7.622445102	1.67E-10	3.36E-09
ENSMMUG00000001989	RNA polymerase II subunit D [Source:VGNC Symbol;Acc:VGNC:76213]	8.421997491	5.42E-14	1.94E-12
ENSMMUG00000027543	Y RNA [Source:RFAM;Acc:RF00019]	8.236239924	2.35E-13	7.45E-12
ENSMMUG00000004177	myosin VIIB [Source:NCBI gene;Acc:714563]	0.315916587	0.001248995	0.005482076
ENSMMUG00000056151		1.939099613	0.000454863	0.002281692
ENSMMUG00000000376	bridging integrator 1 [Source:VGNC Symbol;Acc:VGNC:70251]	2.248673543	0.000363565	0.001862949
ENSMMUG00000020928	nucleolar protein interacting with the FHA domain of MKI67 [Source:VGNC Symbol;Acc:VGNC:75201]	-0.861238721	1.59E-05	0.000115972
ENSMMUG00000046774	RAS like proto-oncogene B [Source:NCBI gene;Acc:695852]	1.629970128	2.59E-10	5.03E-09
ENSMMUG00000059182		1.988051552	1.05E-13	3.57E-12
ENSMMUG00000063759	transmembrane protein 185B [Source:NCBI gene;Acc:695744]	1.790442475	4.43E-14	1.64E-12
ENSMMUG00000059504		2.448970386	8.99E-10	1.59E-08

ENSMMUG00000005610	protein tyrosine phosphatase non-receptor type 4 [Source:VGNC Symbol;Acc:VGNC:76605]	-0.833453334	0.000486908	0.002414414
ENSMMUG00000011129	cilia and flagella associated protein 221 [Source:VGNC Symbol;Acc:VGNC:71153]	-2.428864388	0.007771943	0.026183253
ENSMMUG00000008875	solute carrier family 35 member F5 [Source:VGNC Symbol;Acc:VGNC:77567]	1.056913779	3.48E-09	5.52E-08
ENSMMUG00000043114	U6 spliceosomal RNA [Source:RFAM;Acc:RF00026]	0.652269201	0.002464368	0.009841901
ENSMMUG00000017486	minichromosome maintenance complex component 6 [Source:VGNC Symbol;Acc:VGNC:74685]	-0.886783717	0.000141628	0.000815061
ENSMMUG00000040854	aspartyl-tRNA synthetase 1 [Source:VGNC Symbol;Acc:VGNC:84226]	-0.725578744	0.00014723	0.000843629
ENSMMUG00000061455		2.535258781	0.001393976	0.006029225
ENSMMUG00000006528	Rho GTPase activating protein 15 [Source:VGNC Symbol;Acc:VGNC:69874]	0.748648657	1.46E-11	3.53E-10
ENSMMUG00000016845	zinc finger E-box binding homeobox 2 [Source:VGNC Symbol;Acc:VGNC:78969]	-1.845074604	0.004645505	0.016840823
ENSMMUG00000051691		1.069132703	0.000504476	0.002491869
ENSMMUG00000008615	nebulin [Source:NCBI gene;Acc:100430324]	0.930970025	5.75E-12	1.50E-10
ENSMMUG00000010521	ADP ribosylation factor like GTPase 5A [Source:VGNC Symbol;Acc:VGNC:70017]	2.380017382	6.37E-06	5.08E-05
ENSMMUG00000010526	calcium voltage-gated channel auxiliary subunit beta 4 [Source:VGNC Symbol;Acc:VGNC:82116]	1.651980382	4.86E-10	9.13E-09
ENSMMUG00000010527	signal transducing adaptor molecule 2 [Source:VGNC Symbol;Acc:VGNC:77903]	0.204375047	0.012591585	0.039178911
ENSMMUG00000014176	cytohesin 1 interacting protein [Source:VGNC Symbol;Acc:VGNC:71831]	-0.65867069	0.016078344	0.047905489
ENSMMUG00000011637	coiled-coil domain containing 148 [Source:VGNC Symbol;Acc:VGNC:70856]	2.09836023	0.000709039	0.003356331
ENSMMUG00000011638	plakophilin 4 [Source:VGNC Symbol;Acc:VGNC:76153]	-1.577042946	0.01053203	0.033790361
ENSMMUG00000006443		-2.406519826	0.016792861	0.049688137
ENSMMUG00000052818	death associated protein like 1 [Source:NCBI gene;Acc:699071]	-2.080258866	0.004332745	0.015887207
ENSMMUG00000057379		-1.25130618	0.000475993	0.002368884
ENSMMUG00000006180	tetratricopeptide repeat, ankyrin repeat and coiled-coil containing 1 [Source:VGNC Symbol;Acc:VGNC:78238]	1.495530327	5.12E-12	1.35E-10
ENSMMUG00000061606	Metazoan signal recognition particle RNA [Source:RFAM;Acc:RF00017]	1.575041749	0.000247215	0.001332012
ENSMMUG00000006181	WD repeat, sterile alpha motif and U-box domain containing 1 [Source:VGNC Symbol;Acc:VGNC:79634]	1.151988082	4.95E-07	5.15E-06
ENSMMUG00000063868	Metazoan signal recognition particle RNA [Source:RFAM;Acc:RF00017]	1.424118741	0.000160628	0.000909995
ENSMMUG00000006182	bromodomain adjacent to zinc finger domain 2B [Source:NCBI gene;Acc:702780]	0.624272268	0.000255683	0.001371032
ENSMMUG00000051450	phospholipase A2 receptor 1 [Source:NCBI gene;Acc:700191]	0.545000376	5.90E-05	0.000374474
ENSMMUG00000002678	integrin subunit beta 6 [Source:VGNC Symbol;Acc:VGNC:73789]	-1.273441378	1.81E-06	1.64E-05
ENSMMUG00000038083	Metazoan signal recognition particle RNA [Source:RFAM;Acc:RF00017]	0.686535532	0.016883599	0.049835826
ENSMMUG00000055970		-0.764430216	6.36E-06	5.08E-05
ENSMMUG00000008384	solute carrier family 4 member 10 [Source:VGNC Symbol;Acc:VGNC:77695]	2.926999851	7.35E-27	1.16E-24
ENSMMUG00000008387	dipeptidyl peptidase 4 [Source:VGNC Symbol;Acc:VGNC:71979]	1.771116267	2.74E-09	4.41E-08
ENSMMUG00000013251	xin actin binding repeat containing 2 [Source:VGNC Symbol;Acc:VGNC:78953]	3.211812981	4.75E-07	4.96E-06
ENSMMUG00000063463		-0.957232915	0.004723367	0.01709325
ENSMMUG00000021775	beta-1,3-galactosyltransferase 1 [Source:VGNC Symbol;Acc:VGNC:70204]	-1.20135616	0.000175333	0.000983339
ENSMMUG00000006215	serine/threonine kinase 39 [Source:VGNC Symbol;Acc:VGNC:81587]	-0.933777712	1.41E-05	0.000104563
ENSMMUG00000013830	glucose-6-phosphatase catalytic subunit 2 [Source:NCBI gene;Acc:709062]	2.31963463	1.08E-12	3.12E-11
ENSMMUG00000013832	ATP binding cassette subfamily B member 11 [Source:VGNC Symbol;Acc:VGNC:81339]	2.225866865	1.36E-07	1.60E-06
ENSMMUG00000020510	kelch like family member 41 [Source:VGNC Symbol;Acc:VGNC:74062]	-0.684674535	9.64E-05	0.000577477
ENSMMUG00000023051	kelch like family member 23 [Source:NCBI gene;Acc:706609]	0.416031764	0.000106883	0.000633154
ENSMMUG00000015198	glutamate decarboxylase 1 [Source:VGNC Symbol;Acc:VGNC:72862]	-0.789119718	0.000742886	0.00349493

ENSMMUG00000015203	golgi reassembly stacking protein 2 [Source:VGNC Symbol;Acc:VGNC:72957]	-0.777677253	9.98E-05	0.000594796
ENSMMUG00000011189	tousled like kinase 1 [Source:VGNC Symbol;Acc:VGNC:78367]	-0.822420719	7.60E-05	0.000468558
ENSMMUG00000054176	methyltransferase like 8 [Source:NCBI gene;Acc:695361]	-0.927788075	0.00028894	0.001526834
ENSMMUG00000023370	DDB1 and CUL4 associated factor 17 [Source:VGNC Symbol;Acc:VGNC:71709]	1.153633664	1.79E-05	0.000128579
ENSMMUG00000007301	dynein cytoplasmic 1 intermediate chain 2 [Source:VGNC Symbol;Acc:VGNC:71953]	-0.411437736	0.016428268	0.048808038
ENSMMUG00000007310	solute carrier family 25 member 12 [Source:VGNC Symbol;Acc:VGNC:77430]	-0.678993853	0.00013582	0.000785368
ENSMMUG00000007313	histone acetyltransferase 1 [Source:VGNC Symbol;Acc:VGNC:73349]	-2.142693008	1.04E-09	1.80E-08
ENSMMUG00000002370	integrin subunit alpha 6 [Source:VGNC Symbol;Acc:VGNC:73780]	-0.956501655	4.74E-07	4.96E-06
ENSMMUG00000054462		-0.969031705	3.92E-06	3.29E-05
ENSMMUG00000061038		-1.318326063	0.010753859	0.03441093
ENSMMUG00000020792	Rap guanine nucleotide exchange factor 4 [Source:VGNC Symbol;Acc:VGNC:76490]	-1.141596556	0.000117248	0.000688106
ENSMMUG00000014672	mitogen-activated protein kinase kinase kinase 20 [Source:NCBI gene;Acc:698274]	-1.348056665	0.001337463	0.005810728
ENSMMUG00000049482		-0.571908709	0.014607168	0.044321519
ENSMMUG00000007092	Obg like ATPase 1 [Source:NCBI gene;Acc:697309]	-0.781239986	0.006828697	0.023384868
ENSMMUG00000021012	G protein-coupled receptor 155 [Source:VGNC Symbol;Acc:VGNC:72985]	0.605631474	2.75E-07	3.03E-06
ENSMMUG00000053004		0.739990442	7.06E-05	0.000438315
ENSMMUG00000009593	WAS/WASL interacting protein family member 1 [Source:VGNC Symbol;Acc:VGNC:78784]	1.3914573	1.53E-11	3.68E-10
ENSMMUG00000065344	homeobox D1 [Source:NCBI gene;Acc:701064]	-0.548305969	0.005134102	0.018369293
ENSMMUG00000008502	alkylglycerone phosphate synthase [Source:VGNC Symbol;Acc:VGNC:69747]	-0.311254356	0.012948326	0.040091546
ENSMMUG00000002183	oxysterol binding protein like 6 [Source:VGNC Symbol;Acc:VGNC:75627]	-1.811583313	8.05E-15	3.28E-13
ENSMMUG00000002185	protein activator of interferon induced protein kinase EIF2AK2 [Source:VGNC Symbol;Acc:VGNC:76421]	-0.489660963	0.000231105	0.001254511
ENSMMUG00000054264	pejvakin [Source:NCBI gene;Acc:708668]	-0.474170049	0.004993305	0.0179406
ENSMMUG00000009038	titin [Source:NCBI gene;Acc:703527]	-1.576783889	0.000106813	0.000632995
ENSMMUG00000019690	SEC14 and spectrin domain containing 1 [Source:VGNC Symbol;Acc:VGNC:77267]	2.543724372	3.35E-10	6.47E-09
ENSMMUG00000053406		3.418321069	0.000266242	0.001423971
ENSMMUG00000000300	integrin subunit alpha 4 [Source:VGNC Symbol;Acc:VGNC:73778]	0.370264498	0.016062416	0.047867844
ENSMMUG00000014277	ITPR interacting domain containing 2 [Source:VGNC Symbol;Acc:VGNC:73584]	1.452308214	1.47E-22	1.39E-20
ENSMMUG00000014394	DnaJ heat shock protein family (Hsp40) member C10 [Source:VGNC Symbol;Acc:VGNC:84229]	-1.044544892	1.39E-12	3.94E-11
ENSMMUG00000014397	NCK associated protein 1 [Source:VGNC Symbol;Acc:VGNC:75143]	2.831399289	4.41E-17	2.41E-15
ENSMMUG00000019462	integrin subunit alpha V [Source:VGNC Symbol;Acc:VGNC:73783]	-0.941571963	9.59E-09	1.41E-07
ENSMMUG00000010405	family with sequence similarity 171 member B [Source:VGNC Symbol;Acc:VGNC:72404]	-0.748864681	0.003680223	0.013856357
ENSMMUG00000004022	zinc finger SWIM-type containing 2 [Source:VGNC Symbol;Acc:VGNC:79908]	2.043864365	1.44E-15	6.64E-14
ENSMMUG00000014314	ankyrin and armadillo repeat containing [Source:VGNC Symbol;Acc:VGNC:69706]	-0.865453946	0.000525011	0.002581024

ENSMMUG00000014318	PMS1 homolog 1, mismatch repair system component [Source:VGNC Symbol;Acc:VGNC:81506]	2.037224664	0.002520049	0.010033951
ENSMMUG00000061305		-1.103909087	0.000385967	0.001965946
ENSMMUG00000003260		-1.555270895	2.39E-12	6.59E-11
ENSMMUG00000064089		-0.702239189	8.06E-05	0.000493581
ENSMMUG00000007283	3-hydroxyisobutyryl-CoA hydrolase [Source:NCBI gene;Acc:713951]	-0.892948435	0.000443191	0.002229426
ENSMMUG00000051366		-0.844570499	0.00262251	0.010351622
ENSMMUG00000005612	glutaminase [Source:NCBI gene;Acc:693520]	-1.910337143	0.001214122	0.005348393
ENSMMUG00000005613	signal transducer and activator of transcription 1 [Source:VGNC Symbol;Acc:VGNC:77904]	-0.386884174	0.000244609	0.001319927
ENSMMUG00000001761	myosin IB [Source:VGNC Symbol;Acc:VGNC:75111]	1.572147254	2.08E-13	6.68E-12
ENSMMUG00000037175	U6 spliceosomal RNA [Source:RFAM;Acc:RF00026]	2.202566074	4.10E-09	6.37E-08
ENSMMUG00000001475	transmembrane protein with EGF like and two follistatin like domains 2 [Source:VGNC Symbol;Acc:VGNC:78569]	2.085260726	0.000125067	0.000729279
ENSMMUG00000061797	U6 spliceosomal RNA [Source:RFAM;Acc:RF00026]	1.268646839	0.000582295	0.002824433
ENSMMUG00000061123	Metazoan signal recognition particle RNA [Source:RFAM;Acc:RF00017]	1.329603833	0.001645536	0.006976134
ENSMMUG00000057432	serine/threonine kinase 17b [Source:NCBI gene;Acc:696908]	-1.107564583	0.00036149	0.001854433
ENSMMUG00000015970	coiled-coil domain containing 150 [Source:VGNC Symbol;Acc:VGNC:84222]	-1.189407426	8.12E-14	2.81E-12
ENSMMUG00000008643	general transcription factor IIIC subunit 3 [Source:VGNC Symbol;Acc:VGNC:73317]	-1.853593412	1.24E-18	8.11E-17
ENSMMUG00000022430	splicing factor 3b subunit 1 [Source:VGNC Symbol;Acc:VGNC:77179]	-1.234448341	0.006278799	0.021778787
ENSMMUG00000023809	raftlin family member 2 [Source:VGNC Symbol;Acc:VGNC:76837]	-1.130001827	0.010602043	0.033977492
ENSMMUG00000023810	methionyl-tRNA synthetase 2, mitochondrial [Source:VGNC Symbol;Acc:VGNC:74572]	-0.690567446	0.000361523	0.001854433
ENSMMUG00000023811	boule homolog, RNA binding protein [Source:VGNC Symbol;Acc:VGNC:70310]	-0.807670125	0.001611037	0.006846052
ENSMMUG00000051936		-2.124925984	1.38E-06	1.29E-05
ENSMMUG00000050082		-0.649671525	0.006579564	0.022649094
ENSMMUG00000047974	aldehyde oxidase 4 [Source:NCBI gene;Acc:701502]	-0.786836055	1.08E-08	1.57E-07
ENSMMUG00000022202	basic leucine zipper and W2 domains 1 [Source:VGNC Symbol;Acc:VGNC:84213]	-1.224347806	1.45E-09	2.47E-08
ENSMMUG00000053805	Metazoan signal recognition particle RNA [Source:RFAM;Acc:RF00017]	-2.346344218	0.00267814	0.01051367
ENSMMUG00000012310	CASP8 and FADD like apoptosis regulator [Source:VGNC Symbol;Acc:VGNC:71019]	-1.5563289	0.009263203	0.030445213
ENSMMUG00000031296	caspase 8 [Source:VGNC Symbol;Acc:VGNC:70547]	-0.854296267	5.50E-05	0.000352857
ENSMMUG00000018356	flagellum associated containing coiled-coil domains 1 [Source:VGNC Symbol;Acc:VGNC:72508]	-1.238799554	0.003275141	0.012555571
ENSMMUG00000018144	C2 calcium-dependent domain-containing protein 6 [Source:NCBI gene;Acc:702140]	1.665172264	5.49E-18	3.31E-16
ENSMMUG00000018151	membrane palmitoylated protein 4 [Source:VGNC Symbol;Acc:VGNC:74734]	2.781806033	3.26E-06	2.78E-05
ENSMMUG00000000582	NOP58 ribonucleoprotein [Source:VGNC Symbol;Acc:VGNC:75284]	1.0368739	2.86E-06	2.47E-05
ENSMMUG00000061021		0.891897905	0.000286653	0.001518616
ENSMMUG00000059518		-1.55533517	0.011930488	0.037458641

ENSMMUG00000056824		-2.661258889	6.18E-06	4.96E-05
ENSMMUG00000022601	cAMP responsive element binding protein 1 [Source:VGNC Symbol;Acc:VGNC:71485]	-0.984475936	5.56E-05	0.000356019
ENSMMUG00000022602	methyltransferase like 21A [Source:VGNC Symbol;Acc:VGNC:74625]	-1.278245028	4.62E-06	3.80E-05
ENSMMUG00000020211	cyclin Y like 1 [Source:VGNC Symbol;Acc:VGNC:84224]	-1.418138023	3.96E-10	7.55E-09
ENSMMUG00000057651	frizzled class receptor 5 [Source:NCBI gene;Acc:710796]	-2.018529348	0.000312709	0.001631674
ENSMMUG00000008113	pleckstrin homology domain containing M3 [Source:NCBI gene;Acc:711125]	-1.382491971	0.000143663	0.00082612
ENSMMUG00000010871	crystallin gamma B [Source:VGNC Symbol;Acc:VGNC:71507]	-3.55626619	0.001543525	0.006583984
ENSMMUG00000014299	chromosome 12 C2orf80 homolog [Source:VGNC Symbol;Acc:VGNC:70459]	-4.183221927	2.92E-05	0.000199874
ENSMMUG00000016077	isocitrate dehydrogenase (NADP(+)) 1 [Source:NCBI gene;Acc:710019]	-2.324197006	3.20E-06	2.73E-05
ENSMMUG00000017524	phosphoinositide kinase, FYVE-type zinc finger containing [Source:VGNC Symbol;Acc:VGNC:75854]	-2.170618411	4.06E-07	4.29E-06
ENSMMUG00000005295	parathyroid hormone 2 receptor [Source:VGNC Symbol;Acc:VGNC:76592]	-2.29399058	0.001014672	0.004580834
ENSMMUG00000002809	unc-80 homolog, NALCN channel complex subunit [Source:NCBI gene;Acc:713007]	-1.509019161	1.63E-05	0.000118335
ENSMMUG00000010508	carbamoyl-phosphate synthase 1 [Source:VGNC Symbol;Acc:VGNC:71471]	-3.634018809	6.19E-08	7.73E-07
ENSMMUG00000059604		0.43795643	0.009575395	0.031267092
ENSMMUG00000061342		1.303034998	1.62E-08	2.25E-07
ENSMMUG00000000381	IKAROS family zinc finger 2 [Source:VGNC Symbol;Acc:VGNC:73586]	0.762646589	2.50E-15	1.11E-13
ENSMMUG00000000382	sperm associated antigen 16 [Source:VGNC Symbol;Acc:VGNC:77977]	0.958665485	1.25E-05	9.36E-05
ENSMMUG00000064880		-0.917102724	2.85E-06	2.46E-05
ENSMMUG00000053721		-1.716967091	5.55E-08	7.01E-07
ENSMMUG00000006755	tubulin tyrosine ligase like 4 [Source:VGNC Symbol;Acc:VGNC:79758]	-0.668784122	0.001862615	0.007751743
ENSMMUG00000006758	cytochrome P450, family 27, subfamily A, polypeptide 1 [Source:NCBI gene;Acc:700192]	1.280918543	0.011751717	0.036969207
ENSMMUG00000021877	protein kinase AMP-activated non-catalytic subunit gamma 3 [Source:VGNC Symbol;Acc:VGNC:76406]	1.363970785	0.01376412	0.042212873
ENSMMUG00000052954	Wnt family member 6 [Source:NCBI gene;Acc:700573]	1.745654554	0.003631657	0.013712573
ENSMMUG00000002519	serine/threonine kinase 11 interacting protein [Source:VGNC Symbol;Acc:VGNC:77963]	-0.914729368	2.39E-11	5.54E-10
ENSMMUG00000002520	solute carrier family 4 member 3 [Source:VGNC Symbol;Acc:VGNC:77604]	-0.996804091	1.94E-07	2.21E-06
ENSMMUG00000061834		-1.073133958	7.07E-10	1.28E-08
ENSMMUG00000046282		-1.084327504	9.49E-16	4.51E-14
ENSMMUG00000051349		3.536865572	0.002037366	0.008404425
ENSMMUG00000058085	coiled-coil domain containing 195 [Source:HGNC Symbol;Acc:HGNC:53441]	2.829401499	0.00404976	0.014991434
ENSMMUG00000018139	dedicator of cytokinesis 10 [Source:VGNC Symbol;Acc:VGNC:71888]	0.510811654	2.93E-08	3.88E-07
ENSMMUG00000051482		-2.066833361	5.81E-07	5.95E-06
ENSMMUG00000054072		1.472269049	1.46E-16	7.48E-15
ENSMMUG00000045544	neuronal tyrosine-phosphorylated phosphoinositide-3-kinase adaptor 2 [Source:NCBI gene;Acc:711209]	1.259022307	9.49E-12	2.37E-10
ENSMMUG00000000722	rhomboid domain containing 1 [Source:VGNC Symbol;Acc:VGNC:76803]	-0.550611605	0.013011278	0.04026075

ENSMMUG00000013233	collagen type IV alpha 4 chain [Source:VGNC Symbol;Acc:VGNC:71299]	-0.652418545	0.014797757	0.044796957
ENSMMUG00000013235	collagen type IV alpha 3 chain [Source:VGNC Symbol;Acc:VGNC:81375]	-0.829205747	5.20E-05	0.000335469
ENSMMUG00000002103	delta/notch like EGF repeat containing [Source:VGNC Symbol;Acc:VGNC:71895]	-1.455733035	1.80E-08	2.49E-07
ENSMMUG00000011777	UDP-GlcNAc:betaGal beta-1,3-N-acetylglucosaminyltransferase 7 [Source:VGNC Symbol;Acc:VGNC:82108]	1.909159441	0.009442137	0.030924165
ENSMMUG00000015167	DIS3 like 3'-5' exoribonuclease 2 [Source:VGNC Symbol;Acc:VGNC:71793]	-1.737934787	0.000244859	0.001320786
ENSMMUG00000016871	alkaline phosphatase, intestinal [Source:VGNC Symbol;Acc:VGNC:69800]	-2.369142563	4.51E-06	3.73E-05
ENSMMUG00000051779		0.575897156	0.001997055	0.008259227
ENSMMUG00000005650	ubiquitin specific peptidase 40 [Source:VGNC Symbol;Acc:VGNC:79226]	-0.339408792	0.003836977	0.01434623
ENSMMUG00000021216	UDP glucuronosyltransferase 1 family, polypeptide A1 [Source:NCBI gene;Acc:574210]	-0.696989032	8.38E-05	0.000510719
ENSMMUG00000051807		1.208821358	4.68E-05	0.00030642
ENSMMUG00000061244		1.057042006	0.007823731	0.026308946
ENSMMUG00000054449	Metazoan signal recognition particle RNA [Source:RFAM;Acc:RF00017]	1.368016819	0.000749093	0.003520713
ENSMMUG00000009346	ArfGAP with GTPase domain, ankyrin repeat and PH domain 1 [Source:VGNC Symbol;Acc:VGNC:84206]	0.921022384	0.001657387	0.00702023
ENSMMUG00000017404	IQ motif containing with AAA domain 1 [Source:VGNC Symbol;Acc:VGNC:73660]	-2.555818718	0.002522389	0.010037782
ENSMMUG00000060487	atypical chemokine receptor 3 [Source:NCBI gene;Acc:694428]	-1.186390513	0.000516652	0.002545957
ENSMMUG00000014049	collagen type VI alpha 3 chain [Source:VGNC Symbol;Acc:VGNC:71303]	-1.066227313	0.001769947	0.007425605
ENSMMUG00000059353	phosphatidylserine synthase 2 [Source:NCBI gene;Acc:698582]	-0.676270416	6.48E-05	0.000406251
ENSMMUG00000056711	ribonuclease inhibitor-like [Source:NCBI gene;Acc:114672186]	-0.644238937	6.32E-07	6.38E-06
ENSMMUG00000062863		1.904072415	2.42E-11	5.59E-10
ENSMMUG00000004622	NLR family pyrin domain containing 6 [Source:VGNC Symbol;Acc:VGNC:75234]	-1.80320663	0.00219102	0.008934323
ENSMMUG00000057640	plakophilin-3-like [Source:NCBI gene;Acc:114669833]	3.487411138	5.12E-43	5.32E-40
ENSMMUG00000002917	single Ig IL-1-related receptor [Source:NCBI gene;Acc:100425060]	3.734098882	1.64E-21	1.44E-19
ENSMMUG00000010782	phosphatidylserine synthase 2 [Source:NCBI gene;Acc:114669774]	-0.580594487	0.002807719	0.010980847
ENSMMUG00000010783	ribonuclease inhibitor [Source:NCBI gene;Acc:720791]	0.597138573	0.000264579	0.001416641
ENSMMUG00000021661	leucine rich repeat containing 56 [Source:VGNC Symbol;Acc:VGNC:74356]	1.450852425	9.26E-10	1.63E-08
ENSMMUG00000021664	lamin tail domain containing 2 [Source:VGNC Symbol;Acc:VGNC:74380]	1.510142288	6.89E-18	4.07E-16
ENSMMUG00000054421	Ras association domain family member 7 [Source:NCBI gene;Acc:699206]	-0.31565698	0.005576375	0.019757192
ENSMMUG00000004024	cadherin related family member 5 [Source:VGNC Symbol;Acc:VGNC:71158]	1.23527763	5.36E-06	4.36E-05
ENSMMUG00000016715	p53-induced death domain protein 1 [Source:VGNC Symbol;Acc:VGNC:81498]	0.605488898	8.50E-06	6.58E-05
ENSMMUG00000043846		0.502514888	0.001475089	0.006325479
ENSMMUG00000010544	mucin 5B, oligomeric mucus/gel-forming [Source:VGNC Symbol;Acc:VGNC:75081]	-0.604708395	4.54E-07	4.79E-06
ENSMMUG00000017450	MOB kinase activator 2 [Source:NCBI gene;Acc:720951]	1.414408004	0.000594161	0.002874314
ENSMMUG00000003874	interferon induced transmembrane protein 10 [Source:NCBI gene;Acc:721010]	3.402379356	5.86E-18	3.52E-16
ENSMMUG00000003912	tyrosine hydroxylase [Source:VGNC Symbol;Acc:VGNC:78342]	0.296011582	0.014595219	0.044294509
ENSMMUG00000007742	potassium voltage-gated channel subfamily Q member 1 [Source:VGNC Symbol;Acc:VGNC:82168]	-0.898565698	0.000190012	0.001057102
ENSMMUG00000051131	MAS related GPR family member G [Source:NCBI gene;Acc:721253]	0.782975291	0.000496629	0.002456445
ENSMMUG00000052168		3.703915065	3.46E-42	3.36E-39
ENSMMUG00000060517		1.664651616	6.94E-09	1.05E-07
ENSMMUG00000001777	testis expressed metallothionein like protein [Source:VGNC Symbol;Acc:VGNC:79965]	0.483188213	0.005099348	0.018271931

ENSMMUG00000014237	lysine methyltransferase 5B [Source:VGNC Symbol;Acc:VGNC:74110]	2.126806767	1.86E-39	1.25E-36
ENSMMUG00000056342	Metazoan signal recognition particle RNA [Source:RFAM;Acc:RF00017]	2.78786517	0.000519496	0.002556504
ENSMMUG00000023192	choline kinase alpha [Source:VGNC Symbol;Acc:VGNC:71039]	2.118118382	8.74E-13	2.58E-11
ENSMMUG00000023188	aldehyde dehydrogenase 3 family member B1 [Source:VGNC Symbol;Acc:VGNC:69789]	0.498053551	0.008068679	0.027020131
ENSMMUG00000056105	T-box transcription factor 10 [Source:NCBI gene;Acc:721689]	-0.655741917	1.17E-05	8.83E-05
ENSMMUG00000042611	nudix hydrolase 8 [Source:NCBI gene;Acc:721695]	-2.659419723	0.011086828	0.03527551
ENSMMUG00000011821	phosphatidylinositol transfer protein membrane associated 1 [Source:VGNC Symbol;Acc:VGNC:76007]	-1.336816371	0.014406753	0.043817065
ENSMMUG00000011817	aryl hydrocarbon receptor interacting protein [Source:VGNC Symbol;Acc:VGNC:69759]	1.091780158	7.27E-10	1.31E-08
ENSMMUG00000053378		0.970233956	1.61E-05	0.000117074
ENSMMUG00000005488	coronin 1B [Source:VGNC Symbol;Acc:VGNC:71320]	1.637569323	9.46E-14	3.25E-12
ENSMMUG00000005486	protein tyrosine phosphatase receptor type C associated protein [Source:VGNC Symbol;Acc:VGNC:76613]	0.901410671	8.05E-06	6.27E-05
ENSMMUG00000005483	ribosomal protein S6 kinase B2 [Source:VGNC Symbol;Acc:VGNC:76934]	0.970127836	2.66E-09	4.31E-08
ENSMMUG00000005480	carnosine synthase 1 [Source:VGNC Symbol;Acc:VGNC:81367]	0.760288234	5.40E-18	3.27E-16
ENSMMUG00000017280	TBC1 domain family member 10C [Source:VGNC Symbol;Acc:VGNC:78203]	1.145185916	2.33E-05	0.00016349
ENSMMUG00000004523	DNA polymerase delta 4, accessory subunit [Source:NCBI gene;Acc:712632]	0.729631418	0.003456692	0.013133802
ENSMMUG00000015928	ankyrin repeat domain 13D [Source:VGNC Symbol;Acc:VGNC:69722]	1.045345304	0.004037659	0.014958067
ENSMMUG00000015925	G protein-coupled receptor kinase 2 [Source:VGNC Symbol;Acc:VGNC:73253]	0.874234467	3.91E-06	3.29E-05
ENSMMUG00000008771	lysine demethylase 2A [Source:VGNC Symbol;Acc:VGNC:73824]	0.672319855	8.77E-07	8.53E-06
ENSMMUG00000004529	chromosome 14 C11orf80 homolog [Source:NCBI gene;Acc:721782]	-0.409728755	0.000706853	0.003348166
ENSMMUG00000062635		-0.603090573	0.000139603	0.000804046
ENSMMUG00000010577	RNA-binding protein 4B [Source:NCBI gene;Acc:713553]	0.415209102	0.014463973	0.04394117
ENSMMUG00000031207	Bardet-Biedl syndrome 1 [Source:NCBI gene;Acc:720059]	-1.0325822	0.012053296	0.037778949
ENSMMUG00000015987	Ras and Rab interactor 1 [Source:VGNC Symbol;Acc:VGNC:76769]	-0.722847429	0.000111003	0.000654094
ENSMMUG00000006950	cornichon family AMPA receptor auxiliary protein 2 [Source:VGNC Symbol;Acc:VGNC:71366]	-1.147722703	0.001798301	0.007533687
ENSMMUG00000031202	RAB1B, member RAS oncogene family [Source:NCBI gene;Acc:721852]	-0.974495527	3.44E-05	0.000232412
ENSMMUG00000022520	kinesin light chain 2 [Source:VGNC Symbol;Acc:VGNC:73869]	-0.917079528	0.000148043	0.000847287
ENSMMUG00000057179	cation channel sperm associated 1 [Source:NCBI gene;Acc:721869]	3.204814012	2.69E-14	1.03E-12
ENSMMUG00000056794	cystatin E/M [Source:NCBI gene;Acc:714999]	3.724856401	0.000310381	0.001622442
ENSMMUG00000019115	eukaryotic translation initiation factor 1A domain containing [Source:VGNC Symbol;Acc:VGNC:72127]	0.667401259	0.000222973	0.001217638
ENSMMUG00000008065	spliceosome associated factor 1, recruiter of U4/U6.U5 tri-snRNP [Source:VGNC Symbol;Acc:VGNC:76972]	-0.378733294	0.006398221	0.022139313
ENSMMUG00000008652	coiled-coil domain containing 85B [Source:VGNC Symbol;Acc:VGNC:70853]	1.05934385	6.94E-08	8.59E-07
ENSMMUG00000008651	FGF1 intracellular binding protein [Source:VGNC Symbol;Acc:VGNC:72663]	1.118280736	7.54E-07	7.46E-06
ENSMMUG00000038363	adaptor related protein complex 5 subunit beta 1 [Source:NCBI gene;Acc:721904]	1.368613694	5.82E-19	3.99E-17
ENSMMUG00000050028		1.182101232	1.82E-10	3.66E-09
ENSMMUG00000064005		0.955426931	0.000993732	0.004504485
ENSMMUG00000060437	Metazoan signal recognition particle RNA [Source:RFAM;Acc:RF00017]	1.475145255	0.00019583	0.001084486
ENSMMUG00000019837	ribonuclease H2 subunit C [Source:NCBI gene;Acc:700330]	1.014929868	1.58E-07	1.85E-06
ENSMMUG00000019839	lysine acetyltransferase 5 [Source:VGNC Symbol;Acc:VGNC:73955]	1.205723458	8.32E-15	3.37E-13
ENSMMUG00000061174		0.586682377	0.00450824	0.016404576
ENSMMUG00000023479	latent transforming growth factor beta binding protein 3 [Source:VGNC Symbol;Acc:VGNC:74458]	2.193855876	9.83E-19	6.52E-17
ENSMMUG00000023477		1.957510032	1.49E-38	8.34E-36
ENSMMUG00000052424		0.574365629	0.001712464	0.007226151
ENSMMUG00000023300	FERM domain containing 8 [Source:NCBI gene;Acc:716379]	1.081383335	0.000451142	0.002266355

ENSMMUG00000056343		0.912069276	0.000876414	0.004047149
ENSMMUG00000012316	CDC42 effector protein 2 [Source:VGNC Symbol;Acc:VGNC:70750]	2.075217915	0.003432978	0.013066063
ENSMMUG00000022031	DNA polymerase alpha 2, accessory subunit [Source:NCBI gene;Acc:716541]	1.005747679	8.29E-07	8.12E-06
ENSMMUG00000018767	cell division cycle associated 5 [Source:NCBI gene;Acc:721998]	4.063113816	5.76E-07	5.90E-06
ENSMMUG00000006568	basic leucine zipper ATF-like transcription factor 2 [Source:VGNC Symbol;Acc:VGNC:70127]	1.128910538	2.76E-06	2.39E-05
ENSMMUG00000031163	membrane anchored junction protein [Source:VGNC Symbol;Acc:VGNC:74375]	1.469845226	4.26E-26	6.01E-24
ENSMMUG00000003976	glycoprotein hormone subunit alpha 2 [Source:VGNC Symbol;Acc:VGNC:72961]	1.229425617	4.36E-10	8.22E-09
ENSMMUG00000041101	protein phosphatase 2 regulatory subunit B'beta [Source:NCBI gene;Acc:722026]	1.491616087	5.82E-31	1.34E-28
ENSMMUG00000003973	autophagy related 2A [Source:VGNC Symbol;Acc:VGNC:70145]	-1.463695868	1.12E-06	1.07E-05
ENSMMUG00000003972	EH domain containing 1 [Source:VGNC Symbol;Acc:VGNC:72175]	0.689243993	5.41E-05	0.000348157
ENSMMUG00000031160	menin 1 [Source:NCBI gene;Acc:106995582]	0.285573235	0.009922683	0.03222655
ENSMMUG00000030597	mitogen-activated protein kinase kinase kinase kinase 2 [Source:VGNC Symbol;Acc:VGNC:81465]	0.372408365	0.000306769	0.001607025
ENSMMUG00000007610	ribosomal protein S6 kinase A4 [Source:VGNC Symbol;Acc:VGNC:76930]	0.998123799	1.72E-19	1.23E-17
ENSMMUG00000007603	coiled-coil domain containing 88B [Source:NCBI gene;Acc:722080]	1.208963856	3.38E-07	3.65E-06
ENSMMUG00000013397	peroxiredoxin 5 [Source:NCBI gene;Acc:719764]	0.962861704	1.09E-10	2.27E-09
ENSMMUG00000019647	tRNA methyltransferase subunit 11-2 [Source:NCBI gene;Acc:717676]	-2.099950532	0.01692259	0.04991958
ENSMMUG00000060596	BCL2 associated agonist of cell death [Source:NCBI gene;Acc:717740]	1.031555532	3.61E-08	4.71E-07
ENSMMUG00000012944	vascular endothelial growth factor B [Source:VGNC Symbol;Acc:VGNC:78765]	1.957994937	4.43E-24	4.72E-22
ENSMMUG00000012942	DnaJ heat shock protein family (Hsp40) member C4 [Source:NCBI gene;Acc:717847]	0.931735429	0.006120284	0.021289917
ENSMMUG00000012941	nudix hydrolase 22 [Source:VGNC Symbol;Acc:VGNC:75475]	2.026982535	4.57E-27	7.39E-25
ENSMMUG00000012939	fermitin family member 3 [Source:VGNC Symbol;Acc:VGNC:72553]	-0.806839901	1.33E-06	1.25E-05
ENSMMUG00000012953	stress induced phosphoprotein 1 [Source:NCBI gene;Acc:717926]	-1.109911248	3.36E-11	7.62E-10
ENSMMUG00000056349		-2.791568692	0.000900086	0.004138061
ENSMMUG00000000478	OTU deubiquitinase, ubiquitin aldehyde binding 1 [Source:NCBI gene;Acc:717989]	0.727556181	4.60E-06	3.80E-05
ENSMMUG00000052569		3.818200613	4.26E-15	1.82E-13
ENSMMUG00000014131	microtubule affinity regulating kinase 2 [Source:VGNC Symbol;Acc:VGNC:74634]	0.925260812	0.000284797	0.001511236
ENSMMUG00000005776	atlastin GTPase 3 [Source:VGNC Symbol;Acc:VGNC:70125]	0.496474464	0.000784154	0.003659469
ENSMMUG00000005775	phospholipase A and acyltransferase 3 [Source:VGNC Symbol;Acc:VGNC:76055]	0.533204674	2.65E-10	5.14E-09
ENSMMUG00000045105	phospholipase A and acyltransferase 4 [Source:NCBI gene;Acc:722189]	-0.845189727	0.000552143	0.002696174
ENSMMUG00000056054	zinc finger and BTB domain containing 3 [Source:NCBI gene;Acc:722240]	-0.816122602	1.67E-07	1.94E-06
ENSMMUG00000006598	eukaryotic translation elongation factor 1 gamma [Source:NCBI gene;Acc:722274]	-0.221925119	0.01512729	0.045576252
ENSMMUG00000031123	secretoglobin family 1A member 1 [Source:VGNC Symbol;Acc:VGNC:77004]	-0.667118103	1.90E-05	0.00013573
ENSMMUG00000049661	Metazoan signal recognition particle RNA [Source:RFAM;Acc:RF00017]	-0.804040582	0.000853908	0.003955786
ENSMMUG00000000327	fatty acid desaturase 2 [Source:VGNC Symbol;Acc:VGNC:72472]	1.526906578	2.40E-10	4.70E-09
ENSMMUG00000013798	fatty acid desaturase 1 [Source:VGNC Symbol;Acc:VGNC:72471]	3.305671541	9.85E-22	8.90E-20
ENSMMUG00000006655	myelin regulatory factor [Source:VGNC Symbol;Acc:VGNC:74949]	3.38000366	2.24E-10	4.40E-09
ENSMMUG00000015280	cleavage and polyadenylation specific factor 7 [Source:VGNC Symbol;Acc:VGNC:71606]	2.100237297	0.002633468	0.010383164
ENSMMUG00000042208	pepsin A-4 [Source:NCBI gene;Acc:694646]	-1.18596722	3.00E-09	4.79E-08
ENSMMUG00000015019	VPS37C subunit of ESCRT-I [Source:VGNC Symbol;Acc:VGNC:79578]	2.630322278	0.011736469	0.036929231
ENSMMUG00000044417	CD6 molecule [Source:NCBI gene;Acc:695183]	-1.672353639	0.004982223	0.017914067
ENSMMUG00000014531	transmembrane protein 109 [Source:VGNC Symbol;Acc:VGNC:79740]	-0.908145181	0.004212034	0.015501313

ENSMMUG00000014530	pre-mRNA processing factor 19 [Source:VGNC Symbol;Acc:VGNC:76281]	1.776745182	1.15E-21	1.03E-19
ENSMMUG00000000019	coiled-coil domain containing 86 [Source:VGNC Symbol;Acc:VGNC:70893]	-0.309714008	0.015239183	0.045862815
ENSMMUG00000031100	membrane-spanning 4-domains subfamily A member 4A-like [Source:NCBI gene;Acc:698152]	2.886148485	1.01E-09	1.75E-08
ENSMMUG00000004821	membrane spanning 4-domains A14 [Source:NCBI gene;Acc:697083]	-3.641487829	5.16E-11	1.13E-09
ENSMMUG00000020177	membrane spanning 4-domains A4A [Source:NCBI gene;Acc:698027]	1.692095617	0.000702287	0.003333052
ENSMMUG00000061603		3.444348903	0.002043156	0.008418751
ENSMMUG00000059299	membrane spanning 4-domains A2 [Source:NCBI gene;Acc:697830]	1.771704454	0.001229921	0.005408153
ENSMMUG00000053140	putative oocyte-secreted protein 1 homolog [Source:NCBI gene;Acc:698403]	1.932286762	0.008318316	0.027741076
ENSMMUG00000017287	PAT1 homolog 1, processing body mRNA decay factor [Source:VGNC Symbol;Acc:VGNC:75684]	1.933047854	5.43E-05	0.000349031
ENSMMUG00000001745	oxysterol binding protein [Source:VGNC Symbol;Acc:VGNC:75718]	1.508288198	4.51E-13	1.38E-11
ENSMMUG00000060712		1.401881458	5.23E-14	1.88E-12
ENSMMUG00000045775	macrophage expressed 1 [Source:NCBI gene;Acc:700274]	-1.643609504	0.014753272	0.04469021
ENSMMUG00000001083	glycine N-acyltransferase-like protein 1 [Source:NCBI gene;Acc:701437]	1.004539946	9.61E-11	2.02E-09
ENSMMUG00000011713	leupaxin [Source:VGNC Symbol;Acc:VGNC:74286]	-0.319890253	6.80E-05	0.000423775
ENSMMUG00000011712	olfactory receptor 5B21 [Source:NCBI gene;Acc:702145]	-0.848838057	0.003407545	0.012987804
ENSMMUG00000054737		-0.521485178	7.39E-06	5.82E-05
ENSMMUG00000050844	BTB domain containing 18 [Source:NCBI gene;Acc:703264]	2.006506974	7.79E-07	7.66E-06
ENSMMUG00000056184		2.897632343	1.63E-06	1.49E-05
ENSMMUG00000002566	selenoprotein H [Source:VGNC Symbol;Acc:VGNC:82234]	2.044399145	0.015334294	0.046113908
ENSMMUG00000040741	thioredoxin related transmembrane protein 2 [Source:NCBI gene;Acc:100169970]	1.978202118	4.45E-12	1.18E-10
ENSMMUG00000002562	mediator complex subunit 19 [Source:VGNC Symbol;Acc:VGNC:74615]	2.189182868	0.000173377	0.000973121
ENSMMUG00000002563	zinc finger DHHC-type palmitoyltransferase 5 [Source:VGNC Symbol;Acc:VGNC:79145]	2.034638297	2.56E-06	2.23E-05
ENSMMUG00000058453	cleavage factor polyribonucleotide kinase subunit 1 [Source:NCBI gene;Acc:705071]	-0.631585372	0.009382913	0.030769683
ENSMMUG00000010982	reticulon 4 receptor like 2 [Source:VGNC Symbol;Acc:VGNC:82229]	0.759614977	1.28E-05	9.61E-05
ENSMMUG00000017810	structure specific recognition protein 1 [Source:VGNC Symbol;Acc:VGNC:77891]	1.545131908	1.17E-11	2.88E-10
ENSMMUG00000010627	apelin receptor [Source:VGNC Symbol;Acc:VGNC:69939]	-1.25717363	9.26E-10	1.63E-08
ENSMMUG00000010869	nucleoporin 160 [Source:VGNC Symbol;Acc:VGNC:75429]	1.035543829	0.008958715	0.029591648
ENSMMUG00000040099	Y RNA [Source:RFAM;Acc:RF00019]	1.548866214	0.002474845	0.009878315
ENSMMUG00000058885	formin binding protein 4 [Source:NCBI gene;Acc:712633]	1.311173062	2.06E-09	3.40E-08
ENSMMUG00000012881	ATP/GTP binding protein like 2 [Source:NCBI gene;Acc:711126]	-2.743079923	0.003458392	0.013136829
ENSMMUG00000043682	mitochondrial carrier 2 [Source:NCBI gene;Acc:712767]	-3.090667728	5.74E-06	4.64E-05
ENSMMUG00000004286	protein tyrosine phosphatase mitochondrial 1 [Source:NCBI gene;Acc:713104]	-1.540814726	0.000272343	0.001453398
ENSMMUG00000015606	Spi-1 proto-oncogene [Source:NCBI gene;Acc:712010]	1.088336689	0.001283826	0.005609552
ENSMMUG00000015604	myosin binding protein C3 [Source:VGNC Symbol;Acc:VGNC:75094]	-0.644229454	0.000766708	0.003591891
ENSMMUG00000022964		-0.387731953	7.27E-06	5.74E-05
ENSMMUG00000060750	Metazoan signal recognition particle RNA [Source:RFAM;Acc:RF00017]	2.040041168	0.002382335	0.009551906
ENSMMUG00000003631	cytoskeleton associated protein 5 [Source:VGNC Symbol;Acc:VGNC:71368]	-1.153328536	0.012170245	0.038088021
ENSMMUG00000019674	coagulation factor II, thrombin [Source:VGNC Symbol;Acc:VGNC:72464]	-1.448360378	0.008480385	0.028216837
ENSMMUG00000021446	diacylglycerol kinase zeta [Source:VGNC Symbol;Acc:VGNC:71789]	0.884605693	9.36E-10	1.64E-08
ENSMMUG00000002452	solute carrier family 35 member C1 [Source:VGNC Symbol;Acc:VGNC:77524]	1.023034999	5.62E-08	7.08E-07

ENSMMUG00000049305	Metazoan signal recognition particle RNA [Source:RFAM;Acc:RF00017]	1.220129352	7.57E-05	0.000467389
ENSMMUG00000047219		0.794809961	0.00107391	0.004805757
ENSMMUG00000000874	carbohydrate sulfotransferase 1 [Source:VGNC Symbol;Acc:VGNC:71055]	0.827557072	6.54E-05	0.000409309
ENSMMUG00000050952		- 1.690102879	5.88E-06	4.74E-05
ENSMMUG00000059728	tumor protein p53 inducible protein 11 [Source:NCBI gene;Acc:715295]	- 2.043572333	5.97E-05	0.00037812
ENSMMUG00000000368	tetraspanin 18 [Source:VGNC Symbol;Acc:VGNC:79265]	- 2.340760791	0.00533567	0.018996971
ENSMMUG00000008827	apoptosis inhibitor 5 [Source:NCBI gene;Acc:716346]	- 0.801409318	0.003132928	0.012067668
ENSMMUG00000007093	low density lipoprotein receptor class A domain containing 3 [Source:VGNC Symbol;Acc:VGNC:74137]	0.779659124	0.012576626	0.039149124
ENSMMUG00000051583		0.927190047	4.17E-05	0.000276525
ENSMMUG00000054427	Metazoan signal recognition particle RNA [Source:RFAM;Acc:RF00017]	1.352448971	0.000342175	0.001766405
ENSMMUG00000004332	tripartite motif containing 44 [Source:VGNC Symbol;Acc:VGNC:79168]	2.632968495	4.71E-09	7.26E-08
ENSMMUG00000004330	peptidase domain containing associated with muscle regeneration 1 [Source:VGNC Symbol;Acc:VGNC:75673]	3.498080598	3.41E-08	4.47E-07
ENSMMUG00000004329	solute carrier family 1 member 2 [Source:VGNC Symbol;Acc:VGNC:77410]	- 0.618633524	0.000150362	0.00085853
ENSMMUG00000005927	ETS homologous factor [Source:VGNC Symbol;Acc:VGNC:72178]	- 3.481984862	0.000467212	0.002332871
ENSMMUG00000002429	catalase [Source:VGNC Symbol;Acc:VGNC:70552]	- 0.641176986	8.30E-06	6.44E-05
ENSMMUG00000055602	CD59 molecule (CD59 blood group) [Source:NCBI gene;Acc:693402]	-0.7527669	0.000695047	0.003301927
ENSMMUG00000017218	KIAA1549 like [Source:VGNC Symbol;Acc:VGNC:73902]	- 0.441433661	0.010076229	0.032623194
ENSMMUG00000019988	homeodomain interacting protein kinase 3 [Source:VGNC Symbol;Acc:VGNC:73472]	0.878065032	0.000171622	0.000964758
ENSMMUG00000001536	glutamine and serine rich 1 [Source:VGNC Symbol;Acc:VGNC:76544]	0.999095227	1.18E-16	6.10E-15
ENSMMUG00000055352		1.189464473	4.90E-23	4.72E-21
ENSMMUG00000049850	proline rich and Gla domain 4 [Source:NCBI gene;Acc:694707]	0.989884036	6.22E-10	1.14E-08
ENSMMUG00000065337		- 0.548240602	6.05E-05	0.000382449
ENSMMUG00000006842	doublecortin domain containing 1 [Source:NCBI gene;Acc:696721]	- 0.965153751	1.14E-12	3.28E-11
ENSMMUG00000023119	lin-7 homolog C, crumbs cell polarity complex component [Source:VGNC Symbol;Acc:VGNC:74070]	-1.94680431	0.000325321	0.00169202
ENSMMUG00000014615	lactate dehydrogenase C [Source:VGNC Symbol;Acc:VGNC:74247]	0.892404663	0.0020208	0.008340819
ENSMMUG00000031024	lactate dehydrogenase A [Source:NCBI gene;Acc:694309]	0.978955563	4.17E-06	3.47E-05
ENSMMUG00000006405	secretion regulating guanine nucleotide exchange factor [Source:VGNC Symbol;Acc:VGNC:77258]	0.719312233	0.013220717	0.040787259
ENSMMUG00000006403	potassium voltage-gated channel subfamily C member 1 [Source:VGNC Symbol;Acc:VGNC:73971]	0.554777143	0.000284821	0.001511236
ENSMMUG00000005895	nucleobindin 2 [Source:VGNC Symbol;Acc:VGNC:75549]	- 0.939190447	0.000136566	0.000788742
ENSMMUG00000064558		- 1.097507204	0.00013758	0.000793335
ENSMMUG00000022548	phosphatidylinositol-4-phosphate 3-kinase catalytic subunit type 2 alpha [Source:VGNC Symbol;Acc:VGNC:75992]	- 0.777371699	0.001743449	0.00732289
ENSMMUG00000017628	pleckstrin homology domain containing A7 [Source:VGNC Symbol;Acc:VGNC:76121]	- 1.803953874	0.00054939	0.002683631
ENSMMUG00000060216		- 3.638589498	0.002888316	0.011259709
ENSMMUG00000055212		- 2.111199891	0.013011282	0.04026075
ENSMMUG00000021914	spondin 1 [Source:VGNC Symbol;Acc:VGNC:77862]	- 0.906374687	3.06E-07	3.34E-06
ENSMMUG00000054213	parathyroid hormone [Source:NCBI gene;Acc:700195]	- 1.332881515	0.000544595	0.002662893

ENSMMUG00000009695	BTB domain containing 10 [Source:VGNC Symbol;Acc:VGNC:70361]	-1.179916226	6.46E-06	5.14E-05
ENSMMUG00000010875	TEA domain transcription factor 1 [Source:VGNC Symbol;Acc:VGNC:78301]	0.338051459	0.000661763	0.003163449
ENSMMUG00000020956	parvin alpha [Source:VGNC Symbol;Acc:VGNC:75605]	0.511712861	0.015061766	0.045428141
ENSMMUG00000043504		-0.878114777	2.42E-10	4.72E-09
ENSMMUG00000008758	zinc finger protein 143 [Source:VGNC Symbol;Acc:VGNC:81612]	1.518796564	6.95E-06	5.51E-05
ENSMMUG00000023636	importin 7 [Source:NCBI gene;Acc:704213]	1.30842639	1.32E-08	1.89E-07
ENSMMUG00000052166		1.072204015	0.007046333	0.024045198
ENSMMUG00000005639	transmembrane protein 41B [Source:VGNC Symbol;Acc:VGNC:79754]	1.115172036	7.97E-05	0.000489103
ENSMMUG00000058029	Metazoan signal recognition particle RNA [Source:RFAM;Acc:RF00017]	4.165375348	3.40E-05	0.000229924
ENSMMUG00000021142	DENN domain containing 5A [Source:VGNC Symbol;Acc:VGNC:71674]	0.756138215	0.001979052	0.008189433
ENSMMUG00000006692	signal peptide, CUB domain and EGF like domain containing 2 [Source:VGNC Symbol;Acc:VGNC:77138]	1.126455086	3.26E-08	4.30E-07
ENSMMUG00000006689	nuclear receptor interacting protein 3 [Source:VGNC Symbol;Acc:VGNC:75397]	1.326998249	5.13E-07	5.32E-06
ENSMMUG00000038288	TMEM9 domain family member B [Source:NCBI gene;Acc:708447]	-0.867057309	1.18E-07	1.40E-06
ENSMMUG00000051468	Metazoan signal recognition particle RNA [Source:RFAM;Acc:RF00017]	-0.962049805	2.17E-07	2.45E-06
ENSMMUG00000001995	chromosome 14 C11orf16 homolog [Source:NCBI gene;Acc:705319]	-0.934442183	8.08E-06	6.28E-05
ENSMMUG00000001994	A-kinase interacting protein 1 [Source:VGNC Symbol;Acc:VGNC:69600]	-0.897019326	0.007987488	0.026760571
ENSMMUG00000018471	serine/threonine kinase 33 [Source:VGNC Symbol;Acc:VGNC:78118]	1.269702064	0.000527183	0.002589073
ENSMMUG00000035632	U6 spliceosomal RNA [Source:RFAM;Acc:RF00026]	-0.838945819	0.007726158	0.026061422
ENSMMUG00000050864		-1.898958366	9.34E-13	2.73E-11
ENSMMUG00000041831	hemoglobin subunit epsilon 1 [Source:NCBI gene;Acc:715414]	0.739479468	0.012646626	0.039291302
ENSMMUG00000058081	olfactory receptor 52D1-like [Source:NCBI gene;Acc:106992372]	-1.429541479	0.002626174	0.010360024
ENSMMUG00000049802	olfactory receptor 51G1 [Source:NCBI gene;Acc:716561]	0.611265473	0.012230609	0.038244
ENSMMUG00000022682	tripartite motif containing 68 [Source:NCBI gene;Acc:717644]	0.373109455	0.004917937	0.017710112
ENSMMUG00000063820	olfactory receptor, family 52, subfamily M, member 1 [Source:NCBI gene;Acc:717723]	3.105705694	0.000384243	0.001958533
ENSMMUG00000004974	tripartite motif containing 21 [Source:VGNC Symbol;Acc:VGNC:78556]	-1.058335123	6.62E-07	6.66E-06
ENSMMUG00000003962	ribonucleotide reductase catalytic subunit M1 [Source:VGNC Symbol;Acc:VGNC:76941]	-0.739711398	0.009390311	0.030786413
ENSMMUG00000003955	post-GPI attachment to proteins 2 [Source:VGNC Symbol;Acc:VGNC:75823]	1.416426962	2.91E-21	2.50E-19
ENSMMUG00000003950	nucleoporin 98 and 96 precursor [Source:VGNC Symbol;Acc:VGNC:75563]	1.207434744	5.58E-20	4.16E-18
ENSMMUG00000003948	ADP-ribosyltransferase 1 [Source:VGNC Symbol;Acc:VGNC:70024]	-1.073732388	0.009230909	0.030366535
ENSMMUG00000003946	ADP-ribosyltransferase 5 [Source:VGNC Symbol;Acc:VGNC:69890]	-1.695484956	0.001401292	0.006054727
ENSMMUG00000007325	Xrcc1 N-terminal domain containing 1 [Source:NCBI gene;Acc:115945140]	-1.8466389	6.93E-08	8.58E-07
ENSMMUG00000059115		-2.001716813	1.39E-06	1.29E-05
ENSMMUG00000007326	ring finger protein 121 [Source:VGNC Symbol;Acc:VGNC:76788]	-2.224929212	1.20E-15	5.65E-14
ENSMMUG00000007330	nuclear mitotic apparatus protein 1 [Source:VGNC Symbol;Acc:VGNC:75556]	-1.651631094	9.86E-18	5.74E-16
ENSMMUG00000021280	leucine rich transmembrane and O-methyltransferase domain containing [Source:NCBI gene;Acc:718286]	-1.921078712	3.70E-11	8.32E-10
ENSMMUG00000021282	late endosomal/lysosomal adaptor, MAPK and MTOR activator 1 [Source:VGNC Symbol;Acc:VGNC:74129]	-1.75261594	6.80E-11	1.47E-09
ENSMMUG00000010453	folate receptor beta [Source:VGNC Symbol;Acc:VGNC:72690]	-1.81628791	6.72E-15	2.77E-13

ENSMMUG00000010451	inositol polyphosphate phosphatase like 1 [Source:VGNC Symbol;Acc:VGNC:73752]	1.993487666	2.50E-06	2.19E-05
ENSMMUG00000005423	caseinolytic mitochondrial matrix peptidase chaperone subunit B [Source:VGNC Symbol;Acc:VGNC:71375]	0.650731661	0.010464424	0.033625405
ENSMMUG00000015054	ArfGAP with RhoGAP domain, ankyrin repeat and PH domain 1 [Source:VGNC Symbol;Acc:VGNC:69844]	0.435248581	0.003534804	0.013378143
ENSMMUG00000001294	FCH and double SH3 domains 2 [Source:VGNC Symbol;Acc:VGNC:72593]	-1.376253213	1.01E-07	1.21E-06
ENSMMUG00000003139	Rho guanine nucleotide exchange factor 17 [Source:VGNC Symbol;Acc:VGNC:69880]	0.550371996	0.004832797	0.017432847
ENSMMUG00000004791	pleckstrin homology domain containing B1 [Source:VGNC Symbol;Acc:VGNC:76069]	-1.647554856	2.46E-07	2.74E-06
ENSMMUG00000030964	RAB6A, member RAS oncogene family [Source:NCBI gene;Acc:718717]	1.214303207	3.51E-15	1.52E-13
ENSMMUG00000010321	C2 domain containing 3 centriole elongation regulator [Source:VGNC Symbol;Acc:VGNC:70375]	-0.608117579	2.39E-05	0.000167858
ENSMMUG00000017402	phosphoglucomutase 2 like 1 [Source:VGNC Symbol;Acc:VGNC:75839]	0.906246643	1.51E-06	1.40E-05
ENSMMUG00000056316		0.525315294	0.010304042	0.033234935
ENSMMUG00000013094	lipoyl(octanoyl) transferase 2 [Source:NCBI gene;Acc:718425]	-0.941185801	5.67E-11	1.23E-09
ENSMMUG00000013093	uncharacterized LOC106993385 [Source:NCBI gene;Acc:106993385]	-0.960598368	7.59E-11	1.62E-09
ENSMMUG00000021039	X-ray radiation resistance associated 1 [Source:NCBI gene;Acc:693788]	1.844769147	1.36E-12	3.87E-11
ENSMMUG00000009126	solute carrier organic anion transporter family member 2B1 [Source:VGNC Symbol;Acc:VGNC:77603]	-0.602035351	0.000248116	0.001336372
ENSMMUG00000047594		-0.446892836	0.009081681	0.029970578
ENSMMUG00000017529	arrestin beta 1 [Source:VGNC Symbol;Acc:VGNC:70021]	-0.634040289	0.002013859	0.008319258
ENSMMUG00000000187	UV radiation resistance associated [Source:VGNC Symbol;Acc:VGNC:78751]	0.987919759	8.41E-05	0.000512441
ENSMMUG00000022401	EMSY transcriptional repressor, BRCA2 interacting [Source:VGNC Symbol;Acc:VGNC:72065]	2.474986008	3.27E-20	2.51E-18
ENSMMUG00000018168	alkaline ceramidase 3 [Source:VGNC Symbol;Acc:VGNC:69415]	-0.603150927	2.42E-05	0.000169301
ENSMMUG00000050318	UDP-GlcNAc:betaGal beta-1,3-N-acetylglucosaminyltransferase 6 [Source:NCBI gene;Acc:697946]	-0.595542122	0.000883378	0.004070396
ENSMMUG00000055575	olfactory marker protein [Source:HGNC Symbol;Acc:HGNC:8136]	-0.862977153	0.006316464	0.021893762
ENSMMUG00000001387	p21 (RAC1) activated kinase 1 [Source:NCBI gene;Acc:698585]	1.403665794	1.59E-10	3.23E-09
ENSMMUG00000018945	chloride nucleotide-sensitive channel 1A [Source:VGNC Symbol;Acc:VGNC:71261]	-1.077258144	0.001388325	0.006010149
ENSMMUG00000017355	remodeling and spacing factor 1 [Source:VGNC Symbol;Acc:VGNC:77044]	-1.13054072	4.40E-05	0.000290448
ENSMMUG00000060971		-0.880955725	0.001115648	0.004964934
ENSMMUG00000051769		-0.715816174	6.76E-05	0.00042165
ENSMMUG00000050245		-1.457903603	0.000146402	0.000839276
ENSMMUG00000050119		0.724026017	6.14E-07	6.24E-06
ENSMMUG00000016543	CREB/ATF bZIP transcription factor [Source:VGNC Symbol;Acc:VGNC:71538]	-0.847612891	0.001005699	0.0045488
ENSMMUG00000012449	synaptotagmin like 2 [Source:VGNC Symbol;Acc:VGNC:78123]	-0.967846186	0.002538095	0.010089224
ENSMMUG00000013045	coiled-coil domain containing 81 [Source:VGNC Symbol;Acc:VGNC:70868]	-0.918849118	0.003167666	0.012178883
ENSMMUG00000013055	malic enzyme 3 [Source:VGNC Symbol;Acc:VGNC:74695]	-1.019554673	0.000157524	0.000894624
ENSMMUG00000011116	NADPH oxidase 4 [Source:VGNC Symbol;Acc:VGNC:75376]	-1.067343475	2.37E-06	2.08E-05
ENSMMUG00000060452	FAT atypical cadherin 3 [Source:NCBI gene;Acc:695935]	-0.562328248	0.014140836	0.043158794

ENSMMUG00000017741	solute carrier family 36 member 4 [Source:NCBI gene;Acc:696671]	0.691044152	2.85E-05	0.000195798
ENSMMUG00000013601	chromosome 14 C11orf54 homolog [Source:NCBI gene;Acc:696483]	-0.524549852	0.002082718	0.008557512
ENSMMUG00000004112	MRE11 homolog, double strand break repair nuclease [Source:VGNC Symbol;Acc:VGNC:74926]	-0.351045005	0.014316458	0.043621536
ENSMMUG00000061391	ankyrin repeat domain 49 [Source:NCBI gene;Acc:697693]	1.256509257	2.86E-05	0.000196418
ENSMMUG00000062781	chromosome 14 C11orf97 homolog [Source:NCBI gene;Acc:106993442]	2.559155095	3.20E-46	4.22E-43
ENSMMUG00000008965	piwi like RNA-mediated gene silencing 4 [Source:VGNC Symbol;Acc:VGNC:81504]	-0.557545779	3.62E-05	0.000242861
ENSMMUG00000009394	sestrin 3 [Source:VGNC Symbol;Acc:VGNC:77291]	-0.550138204	0.000291451	0.001536584
ENSMMUG00000061815		-1.063346708	6.60E-10	1.20E-08
ENSMMUG00000016737	contactin 5 [Source:VGNC Symbol;Acc:VGNC:71287]	3.419247517	2.46E-05	0.000171881
ENSMMUG00000020784	centrosomal protein 126 [Source:NCBI gene;Acc:701911]	-0.739909084	4.52E-10	8.51E-09
ENSMMUG00000003394	cilia and flagella associated protein 300 [Source:VGNC Symbol;Acc:VGNC:71120]	-0.708154996	0.007552417	0.025585991
ENSMMUG00000057934		3.02852627	2.75E-12	7.45E-11
ENSMMUG00000005440	ELMO domain containing 1 [Source:VGNC Symbol;Acc:VGNC:72109]	1.254583961	0.000903058	0.004150411
ENSMMUG00000010358	solute carrier family 35 member F2 [Source:VGNC Symbol;Acc:VGNC:77565]	-1.094224055	0.010090704	0.032655513
ENSMMUG00000052526	RAB39A, member RAS oncogene family [Source:NCBI gene;Acc:707451]	-1.803302288	2.33E-46	3.39E-43
ENSMMUG00000001800	cullin 5 [Source:VGNC Symbol;Acc:VGNC:71570]	-1.17524543	0.001317183	0.005743217
ENSMMUG00000004157	nuclear protein, coactivator of histone transcription [Source:VGNC Symbol;Acc:VGNC:75379]	-1.351232608	4.90E-10	9.18E-09
ENSMMUG00000004153	ATM serine/threonine kinase [Source:VGNC Symbol;Acc:VGNC:70173]	-1.147708294	0.003210655	0.012321355
ENSMMUG00000000797	exophilin 5 [Source:VGNC Symbol;Acc:VGNC:72412]	-1.372211612	1.65E-09	2.77E-08
ENSMMUG00000052460		-1.189926846	0.00414061	0.015265546
ENSMMUG00000062793		-3.106458034	2.45E-09	3.99E-08
ENSMMUG00000014001	protein phosphatase 2 scaffold subunit Abeta [Source:VGNC Symbol;Acc:VGNC:76375]	-1.220242385	0.001267463	0.005550901
ENSMMUG00000014334	dopamine receptor D2 [Source:VGNC Symbol;Acc:VGNC:71989]	-0.765011224	0.008629954	0.028675119
ENSMMUG00000007085	zinc finger and BTB domain containing 16 [Source:VGNC Symbol;Acc:VGNC:78845]	-1.68576196	0.002427516	0.009710732
ENSMMUG00000050683		3.072274686	0.010080174	0.0326287
ENSMMUG00000051510		-0.464829335	1.11E-05	8.44E-05
ENSMMUG00000020002	BUD13 homolog [Source:VGNC Symbol;Acc:VGNC:82112]	1.003694083	0.000424953	0.002147962
ENSMMUG00000060987		0.931736546	4.30E-11	9.50E-10
ENSMMUG00000044545	apolipoprotein A1 [Source:NCBI gene;Acc:696969]	1.333177181	9.77E-05	0.000583945
ENSMMUG00000016128	SIK family kinase 3 [Source:VGNC Symbol;Acc:VGNC:77330]	0.690537601	7.14E-07	7.12E-06
ENSMMUG00000002050	platelet activating factor acetylhydrolase 1b catalytic subunit 2 [Source:VGNC Symbol;Acc:VGNC:75748]	-0.627749418	0.009528655	0.03114165
ENSMMUG00000002053	SID1 transmembrane family member 2 [Source:VGNC Symbol;Acc:VGNC:77300]	-0.569396292	0.005516336	0.019554004
ENSMMUG00000007785	beta-secretase 1 [Source:VGNC Symbol;Acc:VGNC:70215]	-3.449885101	0.001929169	0.008001235
ENSMMUG00000007794	DS cell adhesion molecule like 1 [Source:NCBI gene;Acc:698212]	0.550758265	0.000698372	0.003316637
ENSMMUG00000059436	interleukin 10 receptor subunit alpha [Source:NCBI gene;Acc:704383]	1.324034075	7.42E-08	9.12E-07

ENSMMUG00000007620	transmembrane serine protease 4 [Source:VGNC Symbol;Acc:VGNC:78577]	-1.565877302	6.09E-09	9.28E-08
ENSMMUG00000063655	sodium voltage-gated channel beta subunit 4 [Source:NCBI gene;Acc:100430940]	-0.973769576	0.000363125	0.001861993
ENSMMUG00000061384	sodium voltage-gated channel beta subunit 2 [Source:NCBI gene;Acc:678698]	0.395749961	0.000483002	0.002398576
ENSMMUG00000006268	junction adhesion molecule like [Source:VGNC Symbol;Acc:VGNC:73860]	-0.756313485	5.78E-08	7.27E-07
ENSMMUG00000017593	CD3e molecule [Source:VGNC Symbol;Acc:VGNC:70764]	-0.997777073	6.07E-14	2.16E-12
ENSMMUG00000065091	myelin protein zero like 2 [Source:NCBI gene;Acc:699330]	-0.761567724	7.06E-09	1.06E-07
ENSMMUG00000005482	lysine methyltransferase 2A [Source:NCBI gene;Acc:705511]	2.118788298	5.48E-06	4.45E-05
ENSMMUG00000051519	tetratricopeptide repeat domain 36 [Source:NCBI gene;Acc:700199]	-0.316257868	0.007995794	0.026782226
ENSMMUG00000005492	transmembrane protein 25 [Source:VGNC Symbol;Acc:VGNC:79553]	-0.319462946	1.64E-05	0.000118934
ENSMMUG00000046655	pleckstrin homology like domain family B member 1 [Source:NCBI gene;Acc:706336]	-3.440568873	1.07E-23	1.09E-21
ENSMMUG00000002549	trehalase [Source:NCBI gene;Acc:706449]	-3.630565339	2.53E-39	1.49E-36
ENSMMUG00000009318	BCL9 like [Source:VGNC Symbol;Acc:VGNC:70113]	0.770943028	3.12E-06	2.67E-05
ENSMMUG00000056381	forkhead box R1 [Source:NCBI gene;Acc:707285]	1.806185531	0.000296264	0.001559868
ENSMMUG00000016894	ribosomal protein S25 [Source:NCBI gene;Acc:702593]	1.582347686	0.000181282	0.001012409
ENSMMUG00000016946	trafficking protein particle complex 4 [Source:VGNC Symbol;Acc:VGNC:79211]	0.890331107	0.003598799	0.013599101
ENSMMUG00000016890	solute carrier family 37 member 4 [Source:VGNC Symbol;Acc:VGNC:77591]	1.137047149	7.14E-09	1.07E-07
ENSMMUG00000030891	hypoxia up-regulated 1 [Source:VGNC Symbol;Acc:VGNC:73399]	0.889870384	1.50E-06	1.39E-05
ENSMMUG00000014864	VPS11 core subunit of CORVET and HOPS complexes [Source:VGNC Symbol;Acc:VGNC:78965]	0.92417187	3.61E-05	0.000242362
ENSMMUG00000059341	Metazoan signal recognition particle RNA [Source:RFAM;Acc:RF00017]	1.185089931	3.55E-11	8.03E-10
ENSMMUG00000014866	hydroxymethylbilane synthase [Source:VGNC Symbol;Acc:VGNC:73479]	0.816096834	0.01583274	0.04729974
ENSMMUG00000044496	H2A histone family, member X [Source:NCBI gene;Acc:703073]	1.026933047	4.61E-05	0.000302628
ENSMMUG00000014872	dolichyl-phosphate N-acetylglucosaminephosphotransferase 1 [Source:VGNC Symbol;Acc:VGNC:71971]	1.101361324	3.48E-13	1.08E-11
ENSMMUG00000013799	ATP binding cassette subfamily G member 4 [Source:VGNC Symbol;Acc:VGNC:69390]	-3.131506137	4.19E-08	5.40E-07
ENSMMUG00000012191	coiled-coil domain containing 153 [Source:NCBI gene;Acc:704216]	-1.820567065	0.000289937	0.001531069
ENSMMUG00000008923	Thy-1 cell surface antigen [Source:VGNC Symbol;Acc:VGNC:78352]	2.190225202	5.87E-06	4.74E-05
ENSMMUG00000039397	tubulin folding cofactor E like [Source:NCBI gene;Acc:706506]	0.99708979	1.82E-06	1.64E-05
ENSMMUG00000053625	sterol-C5-desaturase [Source:NCBI gene;Acc:706827]	0.87924931	3.49E-06	2.96E-05
ENSMMUG00000014880	sortilin related receptor 1 [Source:NCBI gene;Acc:713011]	0.891682855	6.58E-05	0.000411846
ENSMMUG00000054956		-3.222771487	0.004397767	0.016095251
ENSMMUG00000015522	GRAM domain containing 1B [Source:VGNC Symbol;Acc:VGNC:73028]	-1.65514111	3.41E-13	1.06E-11
ENSMMUG00000008474	transforming growth factor beta regulator 1 [Source:VGNC Symbol;Acc:VGNC:78239]	1.365504354	0.000750707	0.003527157
ENSMMUG00000057600	V-set and immunoglobulin domain containing 2 [Source:NCBI gene;Acc:712047]	-0.819997532	0.000366763	0.001877995
ENSMMUG00000008486	endothelial cell adhesion molecule [Source:VGNC Symbol;Acc:VGNC:72290]	-0.831314978	3.93E-07	4.17E-06
ENSMMUG00000065277	hepatic and glial cell adhesion molecule [Source:NCBI gene;Acc:716321]	1.030689717	0.010024492	0.032506367
ENSMMUG00000006375	coiled-coil domain containing 15 [Source:NCBI gene;Acc:716362]	1.253722038	4.05E-05	0.00026978
ENSMMUG00000010009	EI24 autophagy associated transmembrane protein [Source:VGNC Symbol;Acc:VGNC:72038]	-0.576532335	0.002624296	0.010355426
ENSMMUG00000038587	prostate and testis expressed 2 [Source:NCBI gene;Acc:717085]	-3.522772809	1.69E-05	0.000121688

ENSMMUG00000060291	prostate and testis expressed 3 [Source:NCBI gene;Acc:100379154]	-2.450208402	0.003602283	0.01360873
ENSMMUG00000014120	sc328 [Source:NCBI gene;Acc:574167]	-2.226647035	0.0050784	0.018214827
ENSMMUG00000065066	V-set and immunoglobulin domain containing 10 like 2 [Source:NCBI gene;Acc:717387]	-0.617611436	1.38E-06	1.28E-05
ENSMMUG00000007628	cell adhesion associated, oncogene regulated [Source:VGNC Symbol;Acc:VGNC:70961]	-2.547984694	1.48E-07	1.74E-06
ENSMMUG00000007935	family with sequence similarity 118 member B [Source:VGNC Symbol;Acc:VGNC:72360]	-0.724019271	0.005885359	0.020635664
ENSMMUG00000006929	ST3 beta-galactoside alpha-2,3-sialyltransferase 4 [Source:VGNC Symbol;Acc:VGNC:77827]	1.823925316	5.67E-15	2.37E-13
ENSMMUG00000006931	kirre like nephrin family adhesion molecule 3 [Source:NCBI gene;Acc:714534]	2.231860546	2.34E-23	2.30E-21
ENSMMUG00000020706	ETS proto-oncogene 1, transcription factor [Source:VGNC Symbol;Acc:VGNC:72439]	-1.773514525	1.88E-26	2.85E-24
ENSMMUG00000051915		-3.55351878	0.002525566	0.010047662
ENSMMUG00000060836		-4.123781508	7.93E-07	7.79E-06
ENSMMUG00000002963	Rho GTPase activating protein 32 [Source:VGNC Symbol;Acc:VGNC:69855]	2.460107158	4.05E-07	4.29E-06
ENSMMUG00000032694	U6 spliceosomal RNA [Source:RFAM;Acc:RF00026]	2.889962698	0.00225395	0.009144759
ENSMMUG00000057813		2.96084601	1.92E-07	2.19E-06
ENSMMUG00000017555	PR/SET domain 10 [Source:VGNC Symbol;Acc:VGNC:76250]	-0.58474023	4.57E-06	3.78E-05
ENSMMUG00000018029	amyloid beta precursor like protein 2 [Source:VGNC Symbol;Acc:VGNC:69984]	0.835272449	1.18E-05	8.92E-05
ENSMMUG00000018034	suppression of tumorigenicity 14 [Source:VGNC Symbol;Acc:VGNC:77897]	1.59692912	5.64E-22	5.19E-20
ENSMMUG00000022944	zinc finger and BTB domain containing 44 [Source:VGNC Symbol;Acc:VGNC:79466]	-1.349692226	0.004589267	0.016666044
ENSMMUG00000007107	acyl-CoA dehydrogenase family member 8 [Source:VGNC Symbol;Acc:VGNC:69593]	-2.695481961	0.001335479	0.005803843
ENSMMUG00000002250	beta-1,3-glucuronyltransferase 1 [Source:VGNC Symbol;Acc:VGNC:70207]	-1.941188866	0.000751065	0.003527702
ENSMMUG00000009187	calcium voltage-gated channel subunit alpha1 B [Source:VGNC Symbol;Acc:VGNC:70499]	2.196513813	0.000425859	0.002151794
ENSMMUG00000019607	patatin like phospholipase domain containing 7 [Source:VGNC Symbol;Acc:VGNC:76104]	0.801040715	0.000143136	0.000823415
ENSMMUG00000046202	transmembrane protein 203 [Source:NCBI gene;Acc:706164]	-0.495475448	0.001126843	0.005011124
ENSMMUG00000001677	taperin [Source:NCBI gene;Acc:721581]	-0.448884261	0.006337582	0.021951257
ENSMMUG00000001669	anaphase promoting complex subunit 2 [Source:VGNC Symbol;Acc:VGNC:69641]	-0.428348469	0.015881953	0.047427271
ENSMMUG00000001667	transmembrane protein 210 [Source:VGNC Symbol;Acc:VGNC:79945]	0.712552271	2.83E-05	0.00019513
ENSMMUG00000063598	leucine rich repeat containing 26 [Source:NCBI gene;Acc:706616]	-1.094429929	0.0167838	0.049671436
ENSMMUG00000001661	glutamate ionotropic receptor NMDA type subunit 1 [Source:VGNC Symbol;Acc:VGNC:73281]	-0.908378883	0.015186279	0.045735019
ENSMMUG00000021428	lipocalin 12 [Source:VGNC Symbol;Acc:VGNC:74143]	1.128128063	9.23E-05	0.000555077
ENSMMUG00000011527	TNF receptor associated factor 2 [Source:VGNC Symbol;Acc:VGNC:78641]	0.675927277	0.005325632	0.018965879
ENSMMUG00000005048	MAM domain containing 4 [Source:VGNC Symbol;Acc:VGNC:74345]	-0.817508644	1.84E-06	1.66E-05
ENSMMUG00000005042	RAB, member RAS oncogene family like 6 [Source:VGNC Symbol;Acc:VGNC:76529]	-0.854482024	3.34E-05	0.000225883
ENSMMUG00000005040	coiled-coil domain containing 183 [Source:NCBI gene;Acc:709019]	-1.013671937	0.002581005	0.010206767
ENSMMUG00000026981	EGF like domain multiple 7 [Source:VGNC Symbol;Acc:VGNC:72079]	-0.737071903	0.000219438	0.001202403
ENSMMUG00000056009		1.157504339	1.47E-08	2.07E-07
ENSMMUG00000003106	SEC16 homolog A, endoplasmic reticulum export factor [Source:VGNC Symbol;Acc:VGNC:77326]	1.131780026	6.96E-08	8.61E-07
ENSMMUG00000003105	inositol polyphosphate-5-phosphatase E [Source:VGNC Symbol;Acc:VGNC:73637]	0.97385503	6.05E-10	1.11E-08

ENSMMUG00000003098	peptidase, mitochondrial processing alpha subunit [Source:VGNC Symbol;Acc:VGNC:76062]	1.520253073	7.73E-14	2.68E-12
ENSMMUG00000003081	G protein signaling modulator 1 [Source:VGNC Symbol;Acc:VGNC:72960]	-0.693723199	6.13E-05	0.000387055
ENSMMUG00000001409	coiled-coil domain containing 187 [Source:NCBI gene;Acc:710749]	1.617623151	1.13E-10	2.34E-09
ENSMMUG00000004885	quiescin sulfhydryl oxidase 2 [Source:VGNC Symbol;Acc:VGNC:76568]	-1.647031896	0.014700984	0.044550387
ENSMMUG00000045749		-1.672268349	0.00132265	0.005760143
ENSMMUG00000000276	NACC family member 2 [Source:VGNC Symbol;Acc:VGNC:75013]	3.29989335	0.000130559	0.000758872
ENSMMUG00000016827	UBA domain containing 1 [Source:VGNC Symbol;Acc:VGNC:79837]	2.286962803	2.40E-06	2.11E-05
ENSMMUG00000021496	potassium sodium-activated channel subfamily T member 1 [Source:VGNC Symbol;Acc:VGNC:73856]	-0.809768131	0.000178811	0.000999762
ENSMMUG00000055942	spermatogenesis and oogenesis specific basic helix-loop-helix 1 [Source:NCBI gene;Acc:721833]	-1.335859662	0.0150141	0.045310352
ENSMMUG00000012147	collagen type V alpha 1 chain [Source:NCBI gene;Acc:722014]	2.8143977	2.37E-10	4.63E-09
ENSMMUG00000051925		1.942440223	9.33E-10	1.64E-08
ENSMMUG00000064229		1.742730076	2.59E-06	2.26E-05
ENSMMUG00000005463	bromodomain containing 3 [Source:VGNC Symbol;Acc:VGNC:70281]	-0.917393684	8.36E-07	8.16E-06
ENSMMUG00000017214	vav guanine nucleotide exchange factor 2 [Source:VGNC Symbol;Acc:VGNC:78758]	-1.439148343	0.002965585	0.011520766
ENSMMUG00000017210	sarcosine dehydrogenase [Source:VGNC Symbol;Acc:VGNC:76971]	-2.888958604	7.27E-13	2.15E-11
ENSMMUG00000017207	dopamine beta-hydroxylase [Source:VGNC Symbol;Acc:VGNC:71740]	-2.911363732	2.40E-08	3.23E-07
ENSMMUG00000022348	surfeit 4 [Source:VGNC Symbol;Acc:VGNC:78060]	0.965926793	0.00031945	0.001663862
ENSMMUG00000057105	Metazoan signal recognition particle RNA [Source:RFAM;Acc:RF00017]	0.638685249	0.016372586	0.048652553
ENSMMUG00000050752	Metazoan signal recognition particle RNA [Source:RFAM;Acc:RF00017]	-1.470683958	0.004331931	0.015887207
ENSMMUG00000051984		-1.35619646	0.006197873	0.021534054
ENSMMUG00000009976	uridine-cytidine kinase 1 [Source:VGNC Symbol;Acc:VGNC:78967]	0.962319581	3.82E-11	8.57E-10
ENSMMUG00000002267	laminin subunit gamma 3 [Source:NCBI gene;Acc:722430]	3.154390772	9.45E-07	9.15E-06
ENSMMUG00000036223		2.642791418	0.000534978	0.002620276
ENSMMUG00000059085	PR/SET domain 12 [Source:NCBI gene;Acc:716040]	-1.021645159	3.35E-05	0.000226441
ENSMMUG00000008249	far upstream element binding protein 3 [Source:NCBI gene;Acc:722470]	-0.79823901	0.000156899	0.000891998
ENSMMUG00000001522	argininosuccinate synthase 1 [Source:NCBI gene;Acc:716168]	-0.571726029	0.010998627	0.035039896
ENSMMUG00000061477		-0.435803143	0.001266715	0.005549799
ENSMMUG00000049177	neuronal calcium sensor 1 [Source:NCBI gene;Acc:722507]	-0.380924884	0.014844815	0.044911359
ENSMMUG00000003797	ubiquitin specific peptidase 20 [Source:VGNC Symbol;Acc:VGNC:78740]	2.318161384	1.55E-09	2.62E-08
ENSMMUG00000003793	torsin family 1 member B [Source:VGNC Symbol;Acc:VGNC:78624]	-0.976295723	0.016129991	0.04803968
ENSMMUG00000009965	chromosome 15 C9orf50 homolog [Source:VGNC Symbol;Acc:VGNC:70365]	-2.151107159	2.97E-20	2.30E-18
ENSMMUG00000049483		-3.18961927	0.002924539	0.011385652
ENSMMUG00000055560		-1.707670321	0.000226179	0.001232372
ENSMMUG00000051852	Metazoan signal recognition particle RNA [Source:RFAM;Acc:RF00017]	1.30467687	0.000536686	0.002626868
ENSMMUG00000011767	carnitine O-acetyltransferase [Source:VGNC Symbol;Acc:VGNC:71483]	0.656508563	0.002333418	0.009398973
ENSMMUG00000005989	GLE1 RNA export mediator [Source:VGNC Symbol;Acc:VGNC:73067]	1.137372134	2.62E-12	7.13E-11
ENSMMUG00000005987	outer dense fiber of sperm tails 2 [Source:VGNC Symbol;Acc:VGNC:75580]	-0.601589468	0.004876262	0.017576548

ENSMMUG00000023495	Ral GEF with PH domain and SH3 binding motif 1 [Source:VGNC Symbol;Acc:VGNC:76492]	0.439386999	0.002996085	0.011614423
ENSMMUG00000052476	zinc finger and BTB domain containing 43 [Source:NCBI gene;Acc:701797]	0.718294907	0.000160698	0.000910036
ENSMMUG00000016826	MAPK associated protein 1 [Source:VGNC Symbol;Acc:VGNC:74531]	1.194396438	6.83E-07	6.86E-06
ENSMMUG00000003668	GTPase activating protein and VPS9 domains 1 [Source:VGNC Symbol;Acc:VGNC:72767]	1.152417519	1.81E-10	3.63E-09
ENSMMUG00000005217	heat shock protein family A (Hsp70) member 5 [Source:NCBI gene;Acc:703193]	0.70748452	0.000453754	0.002276915
ENSMMUG00000005215	Rab9 effector protein with kelch motifs [Source:VGNC Symbol;Acc:VGNC:76495]	1.230201933	4.00E-09	6.22E-08
ENSMMUG00000020549	protein phosphatase 6 catalytic subunit [Source:NCBI gene;Acc:703658]	0.985938261	0.000711418	0.003366496
ENSMMUG00000051739	Metazoan signal recognition particle RNA [Source:RFAM;Acc:RF00017]	2.007962029	0.008096451	0.027100648
ENSMMUG00000020633	suppressor of cancer cell invasion [Source:NCBI gene;Acc:712803]	1.11748197	5.97E-06	4.81E-05
ENSMMUG00000061871		-2.475363088	0.000773493	0.003617848
ENSMMUG00000011183	olfactomedin like 2A [Source:VGNC Symbol;Acc:VGNC:75472]	-1.645609279	7.71E-06	6.04E-05
ENSMMUG00000033102	mml-mir-181b-2 [Source:miRBase;Acc:MI0007646]	-2.020755604	0.001193474	0.00526861
ENSMMUG00000026876	mml-mir-181a-2 [Source:miRBase;Acc:MI0002808]	-2.774723184	2.61E-05	0.00018093
ENSMMUG00000014110	adhesion G protein-coupled receptor D2 [Source:VGNC Symbol;Acc:VGNC:69462]	0.702356014	3.95E-09	6.17E-08
ENSMMUG00000054736		3.47727771	6.84E-11	1.47E-09
ENSMMUG00000000496	zinc finger and BTB domain containing 6 [Source:VGNC Symbol;Acc:VGNC:79047]	-2.766577916	0.007786727	0.026208764
ENSMMUG00000063639		1.671893482	2.18E-06	1.93E-05
ENSMMUG00000038944		2.659417947	3.42E-34	1.12E-31
ENSMMUG00000058731	olfactory receptor 1B1 [Source:NCBI gene;Acc:699905]	-0.310128198	0.011199002	0.035576899
ENSMMUG00000043396	Metazoan signal recognition particle RNA [Source:RFAM;Acc:RF00017]	5.033292396	4.42E-11	9.74E-10
ENSMMUG00000005595	RAB14, member RAS oncogene family [Source:NCBI gene;Acc:699837]	3.75577655	2.82E-25	3.62E-23
ENSMMUG00000012668	centriolin [Source:VGNC Symbol;Acc:VGNC:71204]	2.256636612	2.40E-21	2.08E-19
ENSMMUG00000058237		0.726620199	1.07E-05	8.14E-05
ENSMMUG00000013105	astrotactin 2 [Source:VGNC Symbol;Acc:VGNC:70087]	0.749053792	0.000557828	0.002721188
ENSMMUG00000065273		-1.157360054	0.014028005	0.042886458
ENSMMUG00000013107	tripartite motif containing 32 [Source:VGNC Symbol;Acc:VGNC:79032]	0.57789558	1.42E-05	0.000104834
ENSMMUG00000013099	pappalysin 1 [Source:VGNC Symbol;Acc:VGNC:75756]	1.10990928	4.69E-05	0.0003068
ENSMMUG00000003356	tenascin C [Source:VGNC Symbol;Acc:VGNC:79960]	1.223086375	8.75E-09	1.30E-07
ENSMMUG00000041301	TNF superfamily member 8 [Source:NCBI gene;Acc:702944]	-1.083100776	0.000585337	0.002837293
ENSMMUG00000012892	TNF superfamily member 15 [Source:VGNC Symbol;Acc:VGNC:78596]	0.420864967	7.60E-05	0.000468558
ENSMMUG00000064892		1.013922956	1.16E-05	8.82E-05
ENSMMUG00000062461	chromosome 15 C9orf43 homolog [Source:NCBI gene;Acc:704968]	-3.657830897	0.001787957	0.007494674
ENSMMUG00000044537	DNA polymerase epsilon 3, accessory subunit [Source:NCBI gene;Acc:705074]	-3.266618045	3.95E-06	3.31E-05
ENSMMUG00000031965	aminolevulinate dehydratase [Source:VGNC Symbol;Acc:VGNC:69779]	-1.618414279	0.002478507	0.009890214
ENSMMUG00000057031		-3.669097323	2.68E-07	2.96E-06
ENSMMUG00000007187	haloacid dehalogenase like hydrolase domain containing 3 [Source:VGNC Symbol;Acc:VGNC:73250]	-3.882719186	3.48E-06	2.95E-05
ENSMMUG00000005851	B-box and SPRY domain containing [Source:VGNC Symbol;Acc:VGNC:70342]	-3.343289227	2.33E-10	4.56E-09
ENSMMUG00000021097	sushi domain containing 1 [Source:VGNC Symbol;Acc:VGNC:78207]	-1.076055345	0.001611085	0.006846052
ENSMMUG00000000695	transmembrane protein 245 [Source:VGNC Symbol;Acc:VGNC:78933]	0.635835499	0.006419187	0.022175788

ENSMMUG00000000690	catenin alpha like 1 [Source:VGNC Symbol;Acc:VGNC:71544]	-1.481835227	1.76E-07	2.03E-06
ENSMMUG00000053648	actin binding transcription modulator [Source:NCBI gene;Acc:710486]	-1.044231399	9.80E-06	7.50E-05
ENSMMUG00000064066		-1.374053951	0.000779558	0.003640358
ENSMMUG00000019354	actin like 7B [Source:VGNC Symbol;Acc:VGNC:69421]	1.491717822	0.001138166	0.005048154
ENSMMUG00000023797	post-GPI attachment to proteins GalNAc transferase 4 [Source:VGNC Symbol;Acc:VGNC:79688]	1.302669103	0.000562141	0.002738552
ENSMMUG00000053976	bile acid-CoA:amino acid N-acyltransferase [Source:NCBI gene;Acc:100429419]	1.604720612	2.30E-07	2.58E-06
ENSMMUG00000057599		1.017668849	0.016279511	0.048426889
ENSMMUG00000001827	phospholipid phosphatase related 1 [Source:NCBI gene;Acc:714164]	0.922018876	0.000765717	0.003588404
ENSMMUG00000009775	caveolae associated protein 4 [Source:NCBI gene;Acc:714379]	-0.94643819	5.17E-13	1.56E-11
ENSMMUG00000057137		-0.510976096	0.008826576	0.02921492
ENSMMUG00000011410	nuclear receptor subfamily 4 group A member 3 [Source:VGNC Symbol;Acc:VGNC:75519]	-1.129256333	0.000930192	0.004258962
ENSMMUG00000010661	SEC61 translocon subunit beta [Source:NCBI gene;Acc:717815]	-0.576314086	0.001062259	0.004761658
ENSMMUG00000009831	TBC1 domain family member 2 [Source:VGNC Symbol;Acc:VGNC:78157]	-1.326545069	0.002008893	0.008301102
ENSMMUG00000008670	SH2 domain containing adaptor protein B [Source:VGNC Symbol;Acc:VGNC:82236]	0.435199297	0.005624888	0.019908559
ENSMMUG00000047381	DDB1 and CUL4 associated factor 10 [Source:NCBI gene;Acc:716256]	-0.407109949	0.001219957	0.00536896
ENSMMUG00000017886	F-box protein 10 [Source:NCBI gene;Acc:718443]	-0.937831926	9.81E-05	0.0005866
ENSMMUG00000017892	RNA polymerase I subunit E [Source:VGNC Symbol;Acc:VGNC:76047]	-0.910165735	3.09E-05	0.000210675
ENSMMUG00000007421	zinc finger CCHC-type containing 7 [Source:VGNC Symbol;Acc:VGNC:82088]	-0.620120932	4.69E-06	3.85E-05
ENSMMUG00000019639	paired box 5 [Source:VGNC Symbol;Acc:VGNC:75764]	-4.038174094	2.44E-12	6.68E-11
ENSMMUG00000006132	clathrin light chain A [Source:VGNC Symbol;Acc:VGNC:71266]	-0.576017922	0.012285765	0.038375197
ENSMMUG00000054523	Metazoan signal recognition particle RNA [Source:RFAM;Acc:RF00017]	0.509310982	0.003119667	0.012022957
ENSMMUG00000010213	GLI pathogenesis related 2 [Source:VGNC Symbol;Acc:VGNC:81414]	3.343760873	0.000798304	0.003720727
ENSMMUG00000022613	reversion inducing cysteine rich protein with kazal motifs [Source:VGNC Symbol;Acc:VGNC:76722]	1.520773705	1.25E-30	2.84E-28
ENSMMUG00000059406	RGP1 homolog, RAB6A GEF complex partner 1 [Source:NCBI gene;Acc:695936]	-2.495936888	5.69E-07	5.83E-06
ENSMMUG00000016331	glucosylceramidase beta 2 [Source:VGNC Symbol;Acc:VGNC:72741]	-3.903616971	5.65E-06	4.57E-05
ENSMMUG00000031856	talin 1 [Source:VGNC Symbol;Acc:VGNC:78371]	-0.634817365	4.17E-05	0.000276607
ENSMMUG00000050822	carbonic anhydrase 9 [Source:NCBI gene;Acc:696729]	2.500966819	1.89E-39	1.25E-36
ENSMMUG00000048375	Metazoan signal recognition particle RNA [Source:RFAM;Acc:RF00017]	2.184318917	3.19E-59	1.55E-55
ENSMMUG00000016323	Rho guanine nucleotide exchange factor 39 [Source:VGNC Symbol;Acc:VGNC:69912]	2.055070879	1.03E-41	8.84E-39
ENSMMUG00000016327	coiled-coil domain containing 107 [Source:VGNC Symbol;Acc:VGNC:70656]	2.087715291	5.25E-37	2.54E-34
ENSMMUG00000038988	RNase MRP [Source:RFAM;Acc:RF00030]	3.057455061	0.00035521	0.001825916
ENSMMUG00000016316	testis associated actin remodelling kinase 1 [Source:VGNC Symbol;Acc:VGNC:78314]	-1.284119802	2.66E-05	0.00018379
ENSMMUG00000016314	family with sequence similarity 166 member B [Source:VGNC Symbol;Acc:VGNC:72421]	-2.728610286	4.38E-28	7.96E-26
ENSMMUG00000016313	RUN and SH3 domain containing 2 [Source:VGNC Symbol;Acc:VGNC:82230]	-1.244127307	4.54E-11	9.97E-10
ENSMMUG00000059191	stomatin like 2 [Source:NCBI gene;Acc:698458]	1.236176527	0.016013603	0.047751743
ENSMMUG00000011866	phosphatidylinositol glycan anchor biosynthesis class O [Source:VGNC Symbol;Acc:VGNC:75851]	1.502245334	0.003025877	0.011717414

ENSMMUG00000020190	valosin containing protein [Source:VGNC Symbol;Acc:VGNC:78762]	1.600287725	3.86E-07	4.11E-06
ENSMMUG00000061896		1.691093202	5.36E-10	9.94E-09
ENSMMUG00000044048	Metazoan signal recognition particle RNA [Source:RFAM;Acc:RF00017]	1.715194277	7.44E-07	7.38E-06
ENSMMUG00000020188	chromosome 15 C9orf131 homolog [Source:VGNC Symbol;Acc:VGNC:70451]	0.333510376	0.011917062	0.037432657
ENSMMUG00000022895	DnaJ heat shock protein family (Hsp40) member B5 [Source:NCBI gene;Acc:699081]	0.400985544	5.03E-05	0.000326376
ENSMMUG00000058149		0.847806479	0.00669349	0.023008575
ENSMMUG00000017458	DDB1 and CUL4 associated factor 12 [Source:VGNC Symbol;Acc:VGNC:71678]	0.995436018	0.001896805	0.007885002
ENSMMUG00000012597	ubiquitin associated protein 2 [Source:VGNC Symbol;Acc:VGNC:78949]	0.931391115	0.007720378	0.026051797
ENSMMUG00000035888	small nucleolar RNA, C/D box 121A [Source:HGNC Symbol;Acc:HGNC:33560]	1.279965163	0.008385744	0.027914716
ENSMMUG00000053200	ubiquitin conjugating enzyme E2 R2 [Source:NCBI gene;Acc:701507]	0.644886725	0.003248645	0.012457281
ENSMMUG00000000389	nucleolar protein 6 [Source:VGNC Symbol;Acc:VGNC:75218]	0.843428438	1.31E-06	1.23E-05
ENSMMUG00000003314	aquaporin 3 (Gill blood group) [Source:NCBI gene;Acc:702721]	0.824715038	0.000186665	0.001039677
ENSMMUG00000001545	aquaporin 7 [Source:NCBI gene;Acc:702836]	2.460359405	4.60E-13	1.40E-11
ENSMMUG00000002088	SMU1 DNA replication regulator and spliceosomal factor [Source:VGNC Symbol;Acc:VGNC:77733]	-0.433381526	0.007028344	0.023995079
ENSMMUG00000012442	DExD/H-box helicase 58 [Source:VGNC Symbol;Acc:VGNC:71672]	0.813282747	2.41E-12	6.62E-11
ENSMMUG00000008196	aconitase 1 [Source:VGNC Symbol;Acc:VGNC:69378]	3.063886855	3.02E-35	1.25E-32
ENSMMUG00000061225		1.932387237	0.00542419	0.01927266
ENSMMUG00000062843	MOB kinase activator 3B [Source:NCBI gene;Acc:706919]	0.795375294	7.65E-05	0.000471416
ENSMMUG00000061449	equatorin [Source:NCBI gene;Acc:707334]	-1.439877226	0.00291786	0.011362692
ENSMMUG00000006076	intraflagellar transport 74 [Source:VGNC Symbol;Acc:VGNC:73430]	1.249973852	0.003769408	0.01414263
ENSMMUG00000006079	leucine rich repeat containing 19 [Source:VGNC Symbol;Acc:VGNC:74337]	2.016369763	0.000823221	0.003827044
ENSMMUG00000064752	phospholipase A2 activating protein [Source:NCBI gene;Acc:707876]	-1.420994718	0.011769733	0.037009866
ENSMMUG00000050918		0.532700499	0.008095092	0.027100648
ENSMMUG00000054514	interferon alpha 6 [Source:NCBI gene;Acc:106995851]	-2.825505534	0.013852409	0.042438875
ENSMMUG00000019070	focadhesin [Source:VGNC Symbol;Acc:VGNC:72556]	1.885081806	4.16E-24	4.48E-22
ENSMMUG00000013997	perilipin-2 [Source:NCBI gene;Acc:712661]	3.415954514	0.003032694	0.01173756
ENSMMUG00000018396	HAUS augmin like complex subunit 6 [Source:VGNC Symbol;Acc:VGNC:73237]	3.551532585	5.24E-26	7.26E-24
ENSMMUG00000063140	Metazoan signal recognition particle RNA [Source:RFAM;Acc:RF00017]	4.144835082	0.000197088	0.001090623
ENSMMUG00000044658	ADAMTS like 1 [Source:NCBI gene;Acc:712844]	-0.412028375	0.01367937	0.041988388
ENSMMUG00000013466	tetratricopeptide repeat domain 39B [Source:VGNC Symbol;Acc:VGNC:79402]	-0.784806802	6.91E-12	1.77E-10
ENSMMUG00000052889	Metazoan signal recognition particle RNA [Source:RFAM;Acc:RF00017]	-0.681821751	0.006102803	0.021269826
ENSMMUG00000055400	U6 spliceosomal RNA [Source:RFAM;Acc:RF00026]	-1.137027105	2.01E-09	3.33E-08
ENSMMUG00000008128	FRAS1 related extracellular matrix 1 [Source:VGNC Symbol;Acc:VGNC:72768]	0.610482208	0.000201622	0.001112747
ENSMMUG00000002176	ubiquitin like with PHD and ring finger domains 2 [Source:VGNC Symbol;Acc:VGNC:78714]	-0.932640599	4.98E-12	1.31E-10
ENSMMUG00000050923		-0.874412619	4.95E-05	0.000321577
ENSMMUG00000002175	TPD52 like 3 [Source:HGNC Symbol;Acc:HGNC:23382]	-0.729001477	0.001259056	0.005524568
ENSMMUG00000006197	plasminogen receptor with a C-terminal lysine [Source:VGNC Symbol;Acc:VGNC:76098]	-0.923800508	8.06E-06	6.28E-05
ENSMMUG00000052972		0.414877439	0.009156155	0.030162714
ENSMMUG00000065127	insulin like 6 [Source:NCBI gene;Acc:693735]	-0.460210427	1.81E-05	0.000130209
ENSMMUG00000057331	RNA terminal phosphate cyclase like 1 [Source:NCBI gene;Acc:694568]	3.55270555	9.90E-42	8.84E-39
ENSMMUG00000026950	microRNA 101-2 [Source:HGNC Symbol;Acc:HGNC:31489]	3.757300782	1.08E-13	3.68E-12

ENSMMUG00000026024	U6 spliceosomal RNA [Source:RFAM;Acc:RF00026]	1.343769529	0.009829657	0.031974428
ENSMMUG00000056693		-0.550739131	2.04E-06	1.82E-05
ENSMMUG00000015093	very low density lipoprotein receptor [Source:VGNC Symbol;Acc:VGNC:78774]	-0.399439627	0.011110625	0.035334753
ENSMMUG00000057073		-2.483013922	1.31E-05	9.75E-05
ENSMMUG00000015279	SWI/SNF related, matrix associated, actin dependent regulator of chromatin, subfamily a, member 2 [Source:VGNC Symbol;Acc:VGNC:77647]	-1.247209357	4.55E-10	8.56E-09
ENSMMUG00000055563		-0.50160223	0.005758173	0.020282664
ENSMMUG00000059950		0.909411871	0.009318991	0.03058671
ENSMMUG00000012472	phosphatidylinositol-4-phosphate 5-kinase type 1 beta [Source:VGNC Symbol;Acc:VGNC:76001]	0.872825016	8.88E-08	1.07E-06
ENSMMUG00000027986	U6 spliceosomal RNA [Source:RFAM;Acc:RF00026]	2.391688396	6.45E-11	1.40E-09
ENSMMUG00000011401	amyloid beta precursor protein binding family A member 1 [Source:VGNC Symbol;Acc:VGNC:69976]	-2.616401032	1.47E-07	1.72E-06
ENSMMUG00000012667	annexin A1 [Source:VGNC Symbol;Acc:VGNC:69944]	0.5335042	5.23E-07	5.41E-06
ENSMMUG00000056840		1.360641266	8.45E-09	1.26E-07
ENSMMUG00000008685	solute carrier family 28 member 3 [Source:VGNC Symbol;Acc:VGNC:77493]	-3.177157013	0.011704031	0.036859076
ENSMMUG00000053862		-3.700445959	0.004999375	0.017957973
ENSMMUG00000023117	neurotrophic receptor tyrosine kinase 2 [Source:VGNC Symbol;Acc:VGNC:75541]	-0.74009656	1.36E-09	2.33E-08
ENSMMUG00000060761		1.764203359	2.59E-17	1.44E-15
ENSMMUG00000017053	death associated protein kinase 1 [Source:VGNC Symbol;Acc:VGNC:71732]	3.244851023	0.003596926	0.013595559
ENSMMUG00000054211		2.277272126	1.29E-05	9.66E-05
ENSMMUG00000057499		2.457828696	5.10E-10	9.49E-09
ENSMMUG00000033550		2.877443966	0.012263649	0.03831435
ENSMMUG00000049479		3.319921305	0.000177712	0.000995528
ENSMMUG00000053778		-0.801683405	0.006499417	0.022405033
ENSMMUG00000007223	DIRAS family GTPase 2 [Source:VGNC Symbol;Acc:VGNC:71673]	0.519648079	0.004077609	0.015075332
ENSMMUG00000001748	spleen associated tyrosine kinase [Source:VGNC Symbol;Acc:VGNC:78063]	1.643562908	0.00042807	0.002159963
ENSMMUG00000020009	receptor tyrosine kinase like orphan receptor 2 [Source:VGNC Symbol;Acc:VGNC:76716]	1.118471097	2.50E-05	0.00017393
ENSMMUG00000060533		0.911229515	4.35E-10	8.22E-09
ENSMMUG00000003006	serine palmitoyltransferase long chain base subunit 1 [Source:VGNC Symbol;Acc:VGNC:77873]	1.18164572	3.26E-07	3.54E-06
ENSMMUG00000004629	nucleolar protein 8 [Source:VGNC Symbol;Acc:VGNC:75237]	-0.410648832	0.003790733	0.014202549
ENSMMUG00000004630	centromere protein P [Source:NCBI gene;Acc:100427110]	-0.721981858	0.009466019	0.030985706
ENSMMUG00000009493	family with sequence similarity 120A [Source:NCBI gene;Acc:717459]	-2.013546631	4.01E-11	8.96E-10
ENSMMUG00000002517	protein tyrosine phosphatase domain containing 1 [Source:VGNC Symbol;Acc:VGNC:76567]	-1.173916157	4.07E-05	0.000270733
ENSMMUG00000060049		-0.995551908	0.000630618	0.003027506
ENSMMUG00000050773		-0.805376058	0.00120078	0.005296037
ENSMMUG00000005997	cell division cycle 14B [Source:NCBI gene;Acc:710300]	3.260882	7.33E-08	9.02E-07
ENSMMUG00000019423	zinc finger protein 2 [Source:NCBI gene;Acc:703864]	-3.268646116	0.00132131	0.005756032
ENSMMUG00000060932	zinc finger protein 383-like [Source:NCBI gene;Acc:106993074]	-1.420973015	3.18E-06	2.72E-05
ENSMMUG00000049314	dual specificity phosphatase 2 [Source:NCBI gene;Acc:718442]	-1.895895987	0.014407757	0.043817065

ENSMMUG00000053389		-0.85793961	6.63E-07	6.67E-06
ENSMMUG00000057143	transmembrane protein 127 [Source:NCBI gene;Acc:705069]	-1.564596163	1.67E-08	2.32E-07
ENSMMUG00000015515	REV1 DNA directed polymerase [Source:NCBI gene;Acc:709475]	0.525995073	0.00097395	0.00442309
ENSMMUG00000057336		0.529159708	4.79E-07	5.00E-06
ENSMMUG00000019682	mitochondrial ribosomal protein S9 [Source:VGNC Symbol;Acc:VGNC:74762]	1.472396415	0.009741612	0.031737743
ENSMMUG00000001390	NCK adaptor protein 2 [Source:VGNC Symbol;Acc:VGNC:75142]	-1.478182285	0.00042606	0.002152059
ENSMMUG00000058257		0.844072164	0.000912332	0.004189062
ENSMMUG00000002589	sulfotransferase 1C3-like [Source:NCBI gene;Acc:114671897]	-0.57634244	0.005436443	0.019303754
ENSMMUG00000055529	sulfotransferase family 1C member 4 [Source:NCBI gene;Acc:693903]	-3.952829739	2.25E-05	0.000158826
ENSMMUG00000009156	LIM zinc finger domain containing 1 [Source:VGNC Symbol;Acc:VGNC:74271]	-2.3651761	0.00063853	0.003062456
ENSMMUG00000021019	E3 SUMO-protein ligase RanBP2 [Source:NCBI gene;Acc:694823]	2.394534873	0.001950832	0.008086287
ENSMMUG00000060346		-2.818838474	1.76E-09	2.94E-08
ENSMMUG00000002231	transmembrane protein 87B [Source:VGNC Symbol;Acc:VGNC:79557]	-0.838018256	0.00013592	0.000785635
ENSMMUG00000044163	zinc finger CCCH-type containing 8 [Source:NCBI gene;Acc:698828]	-0.467698849	0.000981119	0.004454255
ENSMMUG00000016430	zinc finger CCCH-type containing 6 [Source:VGNC Symbol;Acc:VGNC:79458]	-1.082092015	0.016140884	0.048062275
ENSMMUG00000059850	immunoglobulin kappa variable 1-9 [Source:NCBI gene;Acc:106993071]	-1.319490091	0.004114052	0.015183036
ENSMMUG00000051042		-0.936907779	1.27E-08	1.82E-07
ENSMMUG00000061619		-0.790962129	0.008812833	0.029182724
ENSMMUG00000046169		3.32942507	3.37E-26	4.84E-24
ENSMMUG00000063603		1.828085089	0.00510497	0.018287569
ENSMMUG00000000293	catenin alpha 2 [Source:VGNC Symbol;Acc:VGNC:71543]	1.036494761	5.03E-06	4.11E-05
ENSMMUG00000062818	regenerating islet-derived protein 3-alpha [Source:NCBI gene;Acc:716890]	-1.363258577	9.21E-05	0.000554468
ENSMMUG00000054863		-1.669167269	0.001689552	0.007137757
ENSMMUG00000056785		-0.83020039	0.00061592	0.002966741
ENSMMUG00000056765		-0.580892313	0.000228106	0.001241007
ENSMMUG00000064788		-0.738318722	0.005720818	0.020165743
ENSMMUG00000062747		0.467489872	4.05E-06	3.38E-05
ENSMMUG00000023758	mitochondrial ribosomal protein L19 [Source:VGNC Symbol;Acc:VGNC:74743]	-0.370397399	0.008616777	0.02864443
ENSMMUG00000048322	eva-1 homolog A, regulator of programmed cell death [Source:NCBI gene;Acc:710843]	-1.052412444	0.000152513	0.000869784
ENSMMUG00000051843		-0.860784817	8.70E-07	8.47E-06
ENSMMUG00000001845	DNA polymerase epsilon 4, accessory subunit [Source:VGNC Symbol;Acc:VGNC:76065]	3.24098264	3.46E-24	3.78E-22
ENSMMUG00000058044		-0.846734555	2.08E-08	2.83E-07
ENSMMUG00000006330	poly(A) binding protein interacting protein 2B [Source:NCBI gene;Acc:711595]	0.508096215	6.47E-05	0.000406069
ENSMMUG00000059332	Metazoan signal recognition particle RNA [Source:RFAM;Acc:RF00017]	2.848636564	8.07E-07	7.92E-06
ENSMMUG00000008396	methylmalonyl-CoA epimerase [Source:NCBI gene;Acc:704094]	-0.725866144	5.91E-08	7.41E-07
ENSMMUG00000052504	Metazoan signal recognition particle RNA [Source:RFAM;Acc:RF00017]	1.339666394	0.000960592	0.004377467

ENSMMUG00000002093	testis expressed 261 [Source:NCBI gene;Acc:703863]	-1.427998096	7.04E-15	2.88E-13
ENSMMUG00000055845	ankyrin repeat domain 53 [Source:NCBI gene;Acc:703757]	-1.6527324	8.37E-11	1.78E-09
ENSMMUG00000041775	Metazoan signal recognition particle RNA [Source:RFAM;Acc:RF00017]	-1.454510425	0.001724028	0.007262298
ENSMMUG00000002090	ATPase H+ transporting V1 subunit B1 [Source:VGNC Symbol;Acc:VGNC:70184]	-1.700005775	2.78E-20	2.17E-18
ENSMMUG00000061424	ventral anterior homeobox 2 [Source:NCBI gene;Acc:703531]	1.919127024	5.33E-07	5.49E-06
ENSMMUG00000059700		2.343375442	0.002665524	0.010481125
ENSMMUG00000020854	C-type lectin domain family 4 member F [Source:VGNC Symbol;Acc:VGNC:71349]	3.333835678	0.013133823	0.040570843
ENSMMUG00000020852	folliculogenesis specific bHLH transcription factor [Source:VGNC Symbol;Acc:VGNC:72563]	3.607181226	0.010982566	0.035011746
ENSMMUG00000010592	adducin 2 [Source:VGNC Symbol;Acc:VGNC:69529]	2.295349195	0.001079032	0.004821978
ENSMMUG00000063699		3.347961271	0.000806024	0.003754301
ENSMMUG00000064749		-1.977747256	6.96E-05	0.000432617
ENSMMUG00000007868	AP2 associated kinase 1 [Source:VGNC Symbol;Acc:VGNC:82094]	-0.839214229	9.20E-06	7.08E-05
ENSMMUG00000049539		-1.078622707	1.75E-07	2.02E-06
ENSMMUG00000058336		-0.790102071	0.015679156	0.046947044
ENSMMUG00000013797	glutamine--fructose-6-phosphate transaminase 1 [Source:VGNC Symbol;Acc:VGNC:72915]	0.293009453	0.001135573	0.005040558
ENSMMUG00000007552	aprataxin and PNKP like factor [Source:NCBI gene;Acc:705875]	-0.720530874	0.010561644	0.033870421
ENSMMUG00000017209	C1D nuclear receptor corepressor [Source:NCBI gene;Acc:698450]	1.918161589	2.61E-05	0.000180998
ENSMMUG00000057729		3.254561317	0.009446108	0.030927495
ENSMMUG00000059267	pellino E3 ubiquitin protein ligase 1 [Source:NCBI gene;Acc:696416]	-1.118821494	8.88E-12	2.23E-10
ENSMMUG00000008772	VPS54 subunit of GARP complex [Source:VGNC Symbol;Acc:VGNC:79159]	0.52795325	3.55E-06	3.00E-05
ENSMMUG00000008770	UDP-glucose pyrophosphorylase 2 [Source:VGNC Symbol;Acc:VGNC:78712]	0.647856527	0.000240537	0.001299401
ENSMMUG00000060943		0.371869651	0.003112539	0.012003576
ENSMMUG00000006163	malate dehydrogenase 1 [Source:VGNC Symbol;Acc:VGNC:74690]	-0.67691874	4.73E-05	0.000308822
ENSMMUG00000063238		1.9113681	3.10E-05	0.000211315
ENSMMUG00000006941	exportin 1 [Source:VGNC Symbol;Acc:VGNC:78812]	1.105186367	6.79E-07	6.82E-06
ENSMMUG00000008098	ubiquitin specific peptidase 34 [Source:VGNC Symbol;Acc:VGNC:79223]	-0.382186849	0.016690999	0.049467288
ENSMMUG00000005685	pseudouridine synthase 10 [Source:VGNC Symbol;Acc:VGNC:76547]	0.911335371	0.00199806	0.008261033
ENSMMUG00000055963		1.421645985	1.03E-08	1.50E-07
ENSMMUG00000010834	REL proto-oncogene, NF-kB subunit [Source:VGNC Symbol;Acc:VGNC:76892]	0.653797828	2.63E-06	2.29E-05
ENSMMUG00000049993		-0.377943933	1.20E-05	9.01E-05
ENSMMUG00000007003	polyribonucleotide nucleotidyltransferase 1 [Source:VGNC Symbol;Acc:VGNC:76095]	-3.757950943	9.54E-06	7.33E-05
ENSMMUG00000007001	protein phosphatase 4 regulatory subunit 3B [Source:VGNC Symbol;Acc:VGNC:76342]	-3.673901439	0.000124501	0.000726272
ENSMMUG00000007000	cilia and flagella associated protein 36 [Source:VGNC Symbol;Acc:VGNC:71142]	-3.889592194	0.002209324	0.008996344
ENSMMUG00000008104	proteasome activator subunit 4 [Source:VGNC Symbol;Acc:VGNC:76295]	0.551281824	1.10E-05	8.37E-05
ENSMMUG00000008105	G protein-coupled receptor 75 [Source:VGNC Symbol;Acc:VGNC:73143]	0.50178547	6.84E-05	0.000425964
ENSMMUG00000062091		3.100106637	6.38E-12	1.64E-10
ENSMMUG00000061344		-0.972173721	0.006330917	0.021933397
ENSMMUG00000052169	potassium two pore domain channel subfamily K member 12 [Source:NCBI gene;Acc:715556]	-3.741073252	4.34E-05	0.000287013

ENSMMUG00000053577		-2.944481488	5.76E-14	2.05E-12
ENSMMUG00000000974	mutS homolog 2 [Source:VGNC Symbol;Acc:VGNC:74931]	-2.707198879	2.11E-14	8.20E-13
ENSMMUG00000059300		0.625560237	0.002111859	0.00866011
ENSMMUG00000038319	Metazoan signal recognition particle RNA [Source:RFAM;Acc:RF00017]	-2.102821593	0.005935186	0.020785303
ENSMMUG00000014425	protein kinase C epsilon [Source:VGNC Symbol;Acc:VGNC:76412]	0.757373955	0.002218124	0.009024594
ENSMMUG00000054734		-0.933897063	5.27E-08	6.70E-07
ENSMMUG00000023752	SIX homeobox 2 [Source:VGNC Symbol;Acc:VGNC:81570]	-0.879149301	0.000945116	0.004320489
ENSMMUG00000053683		-0.798222378	0.000625917	0.003008912
ENSMMUG00000014824	SIX homeobox 3 [Source:HGNC Symbol;Acc:HGNC:10889]	-0.975742049	5.91E-15	2.47E-13
ENSMMUG00000051072		1.167108357	6.14E-12	1.58E-10
ENSMMUG00000019841	leucine rich pentatricopeptide repeat containing [Source:VGNC Symbol;Acc:VGNC:82177]	-0.46634138	0.014174047	0.043251075
ENSMMUG00000008081	ATP binding cassette subfamily G member 8 [Source:VGNC Symbol;Acc:VGNC:69392]	1.325894028	2.94E-07	3.23E-06
ENSMMUG00000008080	ATP binding cassette subfamily G member 5 [Source:VGNC Symbol;Acc:VGNC:69391]	1.13890663	7.58E-10	1.36E-08
ENSMMUG00000062474	Metazoan signal recognition particle RNA [Source:RFAM;Acc:RF00017]	1.376558041	2.99E-05	0.000204422
ENSMMUG00000008077	dynein cytoplasmic 2 light intermediate chain 1 [Source:VGNC Symbol;Acc:VGNC:71899]	1.012142043	3.81E-12	1.02E-10
ENSMMUG00000064735	3-hydroxyanthranilate 3,4-dioxygenase [Source:NCBI gene;Acc:713101]	0.464079091	0.000145823	0.000836887
ENSMMUG00000063947	oxoeicosanoid receptor 1 [Source:NCBI gene;Acc:713039]	0.747402326	0.00308323	0.011901463
ENSMMUG00000020744	metastasis associated 1 family member 3 [Source:VGNC Symbol;Acc:VGNC:74814]	0.790296995	0.00046949	0.002342133
ENSMMUG00000056008		0.832073011	1.02E-09	1.78E-08
ENSMMUG00000055395	Y RNA [Source:RFAM;Acc:RF00019]	0.913703393	0.001843509	0.007685427
ENSMMUG00000043458	transmembrane protein 178A [Source:NCBI gene;Acc:714010]	-0.774145251	0.000439255	0.002213328
ENSMMUG00000056330		1.582444862	5.26E-05	0.000338957
ENSMMUG00000013901	mitogen-activated protein kinase kinase kinase kinase 3 [Source:VGNC Symbol;Acc:VGNC:81466]	0.838019595	0.00052743	0.002589411
ENSMMUG00000020575	SOS Ras/Rac guanine nucleotide exchange factor 1 [Source:VGNC Symbol;Acc:VGNC:77837]	0.496274068	0.015393478	0.046253614
ENSMMUG00000060562		0.711996841	0.004443703	0.016230681
ENSMMUG00000002338	heterogeneous nuclear ribonucleoprotein L like [Source:VGNC Symbol;Acc:VGNC:73441]	-0.743170319	8.80E-11	1.86E-09
ENSMMUG00000013944	regulator of microtubule dynamics 2 [Source:VGNC Symbol;Acc:VGNC:76839]	1.253226508	1.42E-11	3.44E-10
ENSMMUG00000054398		1.024360195	0.000224319	0.001224073
ENSMMUG00000016442	glutaminyl-peptide cyclotransferase [Source:VGNC Symbol;Acc:VGNC:76636]	1.516775079	0.002630952	0.010376059
ENSMMUG00000016438	protein kinase D3 [Source:VGNC Symbol;Acc:VGNC:76415]	1.441443071	5.18E-07	5.36E-06
ENSMMUG00000016434	NADH:ubiquinone oxidoreductase complex assembly factor 7 [Source:VGNC Symbol;Acc:VGNC:75035]	1.774247956	8.90E-07	8.65E-06
ENSMMUG00000001278	vitrin [Source:VGNC Symbol;Acc:VGNC:78773]	1.178412016	7.11E-07	7.10E-06
ENSMMUG00000049720		-1.542025413	0.014391959	0.043804813
ENSMMUG00000048261	cysteine rich transmembrane BMP regulator 1 [Source:NCBI gene;Acc:711451]	-0.292747802	0.002233537	0.009074604
ENSMMUG00000053593		-0.776659145	0.000113252	0.000665998
ENSMMUG00000021436	latent transforming growth factor beta binding protein 1 [Source:VGNC Symbol;Acc:VGNC:74456]	1.636113567	6.48E-14	2.29E-12
ENSMMUG00000021691	spastin [Source:VGNC Symbol;Acc:VGNC:77857]	-1.234166364	1.45E-08	2.05E-07
ENSMMUG00000058351		0.626235608	0.000146413	0.000839276
ENSMMUG00000048513	tudor domain containing 15 [Source:NCBI gene;Acc:703066]	-0.968148426	0.000474685	0.002363186

ENSMMUG00000007692	apolipoprotein B [Source:VGNC Symbol;Acc:VGNC:69985]	-0.872088546	4.25E-09	6.60E-08
ENSMMUG00000003355	lipid droplet associated hydrolase [Source:VGNC Symbol;Acc:VGNC:74130]	0.621746407	0.000310997	0.001624492
ENSMMUG00000022996	HCLS1 binding protein 3 [Source:VGNC Symbol;Acc:VGNC:82158]	1.140915428	1.52E-09	2.58E-08
ENSMMUG00000064216		-2.645856597	3.57E-05	0.000239689
ENSMMUG00000012058	GEN1 Holliday junction 5' flap endonuclease [Source:VGNC Symbol;Acc:VGNC:72790]	-3.469666748	5.72E-09	8.75E-08
ENSMMUG00000012056	structural maintenance of chromosomes 6 [Source:VGNC Symbol;Acc:VGNC:77746]	-4.937282488	4.80E-12	1.27E-10
ENSMMUG00000040529	visinin like 1 [Source:NCBI gene;Acc:699459]	-2.654191692	0.002019537	0.008338348
ENSMMUG00000004215	LRAT domain containing 1 [Source:VGNC Symbol;Acc:VGNC:74321]	-2.755793416	4.27E-05	0.000282746
ENSMMUG00000052377		-0.659872744	0.003974723	0.014766304
ENSMMUG00000056755		1.496044972	0.002244997	0.009113523
ENSMMUG00000020249	growth regulating estrogen receptor binding 1 [Source:VGNC Symbol;Acc:VGNC:73227]	0.379037477	0.010941325	0.034910897
ENSMMUG00000023225	E2F transcription factor 6 [Source:VGNC Symbol;Acc:VGNC:72027]	0.441725421	0.010470524	0.033637571
ENSMMUG00000059188		1.177639515	0.000355626	0.001827405
ENSMMUG00000016678	Rho associated coiled-coil containing protein kinase 2 [Source:VGNC Symbol;Acc:VGNC:76714]	1.686708595	5.92E-07	6.05E-06
ENSMMUG00000022785	solute carrier family 66 member 3 [Source:VGNC Symbol;Acc:VGNC:76334]	1.873251302	0.000178103	0.000997334
ENSMMUG00000063262		-1.078261435	2.09E-10	4.15E-09
ENSMMUG00000052047		1.230850236	1.68E-06	1.53E-05
ENSMMUG00000037908		-0.818720028	0.006454994	0.022283602
ENSMMUG00000022879	EARP complex and GARP complex interacting protein 1 [Source:NCBI gene;Acc:707650]	-2.251019671	0.001681811	0.007111251
ENSMMUG00000051814		0.468148952	0.013044523	0.04032927
ENSMMUG00000009310	myelin transcription factor 1 like [Source:NCBI gene;Acc:706914]	-0.538856297	0.006110798	0.021282384
ENSMMUG00000050687	Metazoan signal recognition particle RNA [Source:RFAM;Acc:RF00017]	-1.529938928	0.012610752	0.039210956
ENSMMUG00000009660	syntrophin gamma 2 [Source:VGNC Symbol;Acc:VGNC:77694]	-0.630473247	0.001734668	0.007292358
ENSMMUG00000061533		3.044550344	1.53E-35	6.97E-33
ENSMMUG00000050543		2.787508549	7.60E-17	4.02E-15
ENSMMUG00000042598	transmembrane protein 18 [Source:NCBI gene;Acc:721515]	4.128440864	2.25E-15	1.01E-13
ENSMMUG00000038858		0.533253945	0.000359418	0.001844938
ENSMMUG00000063735		1.340958477	0.000166475	0.000938732
ENSMMUG00000022914	RAB, member of RAS oncogene family like 2B [Source:NCBI gene;Acc:716615]	1.375301094	8.10E-15	3.29E-13
ENSMMUG00000009115	tubulin tyrosine ligase like 8 [Source:VGNC Symbol;Acc:VGNC:79943]	-0.32031519	0.001970947	0.008161648
ENSMMUG00000058324		3.026959252	0.014341042	0.043678131
ENSMMUG00000011278	cysteine rich with EGF like domains 2 [Source:NCBI gene;Acc:720586]	1.00474607	3.53E-08	4.62E-07
ENSMMUG00000003764	TBC1 domain family member 22A [Source:VGNC Symbol;Acc:VGNC:82077]	0.420931437	0.015672796	0.046947044
ENSMMUG00000056186		1.499412933	3.12E-06	2.67E-05
ENSMMUG00000020993	cadherin EGF LAG seven-pass G-type receptor 1 [Source:NCBI gene;Acc:718440]	-1.767057377	8.64E-08	1.05E-06
ENSMMUG00000000807	tRNA 5-methylaminomethyl-2-thiouridylate methyltransferase [Source:VGNC Symbol;Acc:VGNC:79500]	-1.631273948	0.000831386	0.003863764
ENSMMUG00000016262	tetratricopeptide repeat domain 38 [Source:VGNC Symbol;Acc:VGNC:79574]	-1.927000163	1.10E-10	2.29E-09
ENSMMUG00000008955	ataxin 10 [Source:VGNC Symbol;Acc:VGNC:70046]	0.543952451	0.006119324	0.021289917

ENSMMUG00000023004	structural maintenance of chromosomes 1B [Source:VGNC Symbol;Acc:VGNC:77657]	0.531104728	2.61E-05	0.00018093
ENSMMUG00000023003	family with sequence similarity 118 member A [Source:VGNC Symbol;Acc:VGNC:72265]	0.607668249	0.000220488	0.00120679
ENSMMUG00000031000	Rho GTPase activating protein 8 [Source:NCBI gene;Acc:716745]	-3.001643027	4.99E-06	4.09E-05
ENSMMUG00000058874	Metazoan signal recognition particle RNA [Source:RFAM;Acc:RF00017]	-3.5712024	0.001730503	0.007281133
ENSMMUG00000065099		-2.833861852	2.71E-20	2.13E-18
ENSMMUG00000005769	EF-hand calcium binding domain 6 [Source:VGNC Symbol;Acc:VGNC:72092]	0.708747655	0.002639418	0.010395347
ENSMMUG00000008106	solute carrier family 25 member 17 [Source:NCBI gene;Acc:705547]	0.595625072	0.003886222	0.014493077
ENSMMUG00000056662		-1.302330658	9.63E-08	1.15E-06
ENSMMUG00000012101	synaptogyrin 1 [Source:VGNC Symbol;Acc:VGNC:78068]	-1.473894598	4.20E-08	5.41E-07
ENSMMUG00000004868	dynein axonemal light chain 4 [Source:VGNC Symbol;Acc:VGNC:71945]	3.599647991	0.000288545	0.001525856
ENSMMUG00000021083	Josephin domain containing 1 [Source:VGNC Symbol;Acc:VGNC:73920]	-1.124788039	0.000913216	0.004191799
ENSMMUG00000000364	translocase of outer mitochondrial membrane 22 [Source:NCBI gene;Acc:701286]	-1.382488706	0.001549599	0.006607956
ENSMMUG00000061986		-1.774301267	0.000158604	0.000899932
ENSMMUG00000012467	family with sequence similarity 227 member A [Source:VGNC Symbol;Acc:VGNC:72475]	-1.246061732	2.39E-05	0.00016774
ENSMMUG00000061118		-1.373748937	0.01264042	0.039280415
ENSMMUG00000049788		-1.694159269	9.78E-06	7.50E-05
ENSMMUG00000002697	protein interacting with PRKCA 1 [Source:VGNC Symbol;Acc:VGNC:76013]	-0.255637845	0.012072285	0.037830311
ENSMMUG00000006987	RNA polymerase II subunit F [Source:NCBI gene;Acc:699566]	-0.35197618	0.002321027	0.009359435
ENSMMUG00000019785	eukaryotic translation initiation factor 3 subunit L [Source:NCBI gene;Acc:699189]	-1.344473684	0.006669155	0.022940635
ENSMMUG00000015258	glycine C-acetyltransferase [Source:NCBI gene;Acc:698811]	2.458703335	6.27E-05	0.000395025
ENSMMUG00000005991	TRIO and F-actin binding protein [Source:NCBI gene;Acc:701241]	0.631402295	6.08E-07	6.20E-06
ENSMMUG00000056629	nucleolar protein 12 [Source:NCBI gene;Acc:698318]	1.255980758	0.002967083	0.011521556
ENSMMUG00000003007	caspase recruitment domain family member 10 [Source:VGNC Symbol;Acc:VGNC:70570]	2.873220307	8.64E-06	6.68E-05
ENSMMUG00000003005	MFNG O-fucosylpeptide 3-beta-N-acetylglucosaminyltransferase [Source:VGNC Symbol;Acc:VGNC:74712]	5.101331066	9.55E-08	1.14E-06
ENSMMUG00000052785	extracellular leucine rich repeat and fibronectin type III domain containing 2 [Source:NCBI gene;Acc:697071]	3.678010032	8.54E-05	0.00051901
ENSMMUG00000062922		-0.818313322	0.00096154	0.004380414
ENSMMUG00000000151	transmembrane serine protease 6 [Source:VGNC Symbol;Acc:VGNC:79038]	0.644685515	0.000483689	0.002400612
ENSMMUG00000012350	mercaptopyruvate sulfurtransferase [Source:VGNC Symbol;Acc:VGNC:74922]	0.779402502	0.003869168	0.014440588
ENSMMUG00000012349	thiosulfate sulfurtransferase [Source:VGNC Symbol;Acc:VGNC:78664]	1.488320035	0.012021049	0.037686001
ENSMMUG00000014986	testis expressed 33 [Source:VGNC Symbol;Acc:VGNC:79727]	1.111618142	9.15E-09	1.35E-07
ENSMMUG00000008160	neutrophil cytosolic factor 4 [Source:VGNC Symbol;Acc:VGNC:75141]	1.110544603	0.007843972	0.026370913
ENSMMUG00000055820		1.76830359	5.84E-06	4.71E-05
ENSMMUG00000008778	parvalbumin [Source:VGNC Symbol;Acc:VGNC:76627]	1.104007873	5.54E-08	7.01E-07
ENSMMUG00000006101	myosin heavy chain 9 [Source:VGNC Symbol;Acc:VGNC:75102]	0.84951979	1.51E-05	0.000110489
ENSMMUG00000014010	RNA binding fox-1 homolog 2 [Source:VGNC Symbol;Acc:VGNC:76685]	0.627439249	7.58E-06	5.94E-05
ENSMMUG00000048463	apolipoprotein L5 [Source:HGNC Symbol;Acc:HGNC:14869]	0.495598994	0.003073693	0.011872646
ENSMMUG00000059824		-3.488297373	0.000738912	0.003477362
ENSMMUG00000062825	TIMP metallopeptidase inhibitor 3 [Source:NCBI gene;Acc:574381]	-0.4581443	0.016705194	0.049499269
ENSMMUG00000006720	RNA 2',3'-cyclic phosphate and 5'-OH ligase [Source:VGNC Symbol;Acc:VGNC:77111]	-0.538989088	0.003335217	0.01275225

ENSMMUG00000056552		0.912434897	8.76E-05	0.000531156
ENSMMUG00000002182	phosphatidylserine decarboxylase [Source:VGNC Symbol;Acc:VGNC:76005]	-1.384647901	0.00149338	0.006394482
ENSMMUG00000002181	SFI1 centrin binding protein [Source:VGNC Symbol;Acc:VGNC:77328]	0.804618997	5.90E-10	1.09E-08
ENSMMUG00000015001	LIM domain kinase 2 [Source:VGNC Symbol;Acc:VGNC:74270]	0.611197863	0.003539112	0.013390961
ENSMMUG00000004675	smoothelin [Source:VGNC Symbol;Acc:VGNC:77661]	-1.648085822	7.73E-07	7.63E-06
ENSMMUG00000056113		-1.709525643	0.013211341	0.040776534
ENSMMUG00000005898	MORC family CW-type zinc finger 2 [Source:VGNC Symbol;Acc:VGNC:74810]	-1.971427162	0.016344953	0.048580367
ENSMMUG00000019805	dual specificity phosphatase 18 [Source:VGNC Symbol;Acc:VGNC:71858]	-0.532133283	0.000850609	0.003944274
ENSMMUG00000052343		-1.014739297	0.000254272	0.001364689
ENSMMUG00000021374	EWS RNA binding protein 1 [Source:VGNC Symbol;Acc:VGNC:72446]	-0.814321203	0.000129173	0.000751714
ENSMMUG00000021373	rhomboid domain containing 3 [Source:NCBI gene;Acc:714479]	-0.710439995	0.001026051	0.00462646
ENSMMUG00000021372	EMI domain containing 1 [Source:VGNC Symbol;Acc:VGNC:72063]	-0.754125939	0.000235513	0.001276053
ENSMMUG00000058170	U6 spliceosomal RNA [Source:RFAM;Acc:RF00026]	-0.715815612	0.009700227	0.031617086
ENSMMUG00000007215	X-box binding protein 1 [Source:VGNC Symbol;Acc:VGNC:78806]	-1.087184149	1.56E-05	0.000113954
ENSMMUG00000053198	coiled-coil domain containing 117 [Source:NCBI gene;Acc:713804]	0.764029661	0.002638697	0.010395347
ENSMMUG00000020489	HscB mitochondrial iron-sulfur cluster cochaperone [Source:VGNC Symbol;Acc:VGNC:73454]	0.658054589	4.01E-07	4.25E-06
ENSMMUG00000020486	checkpoint kinase 2 [Source:VGNC Symbol;Acc:VGNC:70958]	0.627739903	1.65E-05	0.000119226
ENSMMUG00000001058	tetratricopeptide repeat domain 28 [Source:VGNC Symbol;Acc:VGNC:79785]	3.083275487	2.74E-19	1.92E-17
ENSMMUG00000042122	Metazoan signal recognition particle RNA [Source:RFAM;Acc:RF00017]	3.693860026	0.000732108	0.003449805
ENSMMUG00000023450	tyrosylprotein sulfotransferase 2 [Source:VGNC Symbol;Acc:VGNC:78639]	-1.045085461	1.25E-05	9.36E-05
ENSMMUG00000023446	tuftelin interacting protein 11 [Source:VGNC Symbol;Acc:VGNC:78399]	-1.295127863	4.46E-06	3.69E-05
ENSMMUG00000023443	SRR1 domain containing [Source:VGNC Symbol;Acc:VGNC:78036]	-1.194507062	7.15E-08	8.83E-07
ENSMMUG00000023442	HPS4 biogenesis of lysosomal organelles complex 3 subunit 2 [Source:VGNC Symbol;Acc:VGNC:73396]	-2.339342869	0.000560913	0.00273349
ENSMMUG00000016052	seizure related 6 homolog like [Source:VGNC Symbol;Acc:VGNC:77176]	-1.47250721	5.56E-09	8.53E-08
ENSMMUG00000006853	crystallin beta B2 [Source:NCBI gene;Acc:100144420]	3.046349431	7.72E-10	1.38E-08
ENSMMUG00000039465		-1.559958555	0.00748955	0.025426341
ENSMMUG00000044345	ral-GDS-related protein [Source:NCBI gene;Acc:710293]	0.761431457	0.00042411	0.002144449
ENSMMUG00000031051	aspartate-rich protein 1 [Source:NCBI gene;Acc:107000598]	-0.576107834	0.003661738	0.013807298
ENSMMUG00000020992	calcineurin binding protein 1 [Source:NCBI gene;Acc:700566]	-0.514054912	0.005792363	0.020373477
ENSMMUG00000005980	glutathione hydrolase 5 proenzyme [Source:NCBI gene;Acc:720345]	-0.335039069	0.001857942	0.007738942
ENSMMUG00000002288	glutathione hydrolase 1 proenzyme [Source:NCBI gene;Acc:700936]	0.651794276	0.009720697	0.031676701
ENSMMUG00000044782		-0.991363009	1.60E-08	2.23E-07
ENSMMUG00000020999	gamma-glutamyltransferase 1 [Source:NCBI gene;Acc:707545]	-0.711408315	0.001051328	0.004721394

ENSMMUG00000000262	beta-ureidopropionase 1 [Source:VGNC Symbol;Acc:VGNC:79029]	-1.343217533	0.000112623	0.000662567
ENSMMUG00000051040		0.915768943	0.016084989	0.047915468
ENSMMUG00000060794		0.384219649	0.013601583	0.041802581
ENSMMUG00000049542		1.667477399	2.71E-07	2.99E-06
ENSMMUG00000023622	BCR activator of RhoGEF and GTPase [Source:NCBI gene;Acc:709258]	1.501200778	7.78E-07	7.66E-06
ENSMMUG00000014641		0.394707626	0.000349869	0.001802924
ENSMMUG00000020092		0.602479993	0.003154746	0.012142055
ENSMMUG00000054655	immunoglobulin lambda variable 3-10 [Source:HGNC Symbol;Acc:HGNC:5897]	-1.347642249	3.69E-06	3.11E-05
ENSMMUG00000056950		1.063228281	2.86E-14	1.09E-12
ENSMMUG00000047015	ral-GDS-related protein-like [Source:NCBI gene;Acc:106995524]	-1.026008613	0.000140439	0.00080854
ENSMMUG00000018798	stromal cell derived factor 2 like 1 [Source:NCBI gene;Acc:697928]	0.615583435	0.000246324	0.001328196
ENSMMUG00000018531	peptidylprolyl isomerase like 2 [Source:VGNC Symbol;Acc:VGNC:76239]	0.387794298	0.00567556	0.020040226
ENSMMUG00000018823	transmembrane protein 191C [Source:NCBI gene;Acc:697070]	1.840898837	0.0037959	0.014216542
ENSMMUG00000056022		0.627366404	0.000881901	0.004068247
ENSMMUG00000062755		1.27621812	0.000165087	0.000931626
ENSMMUG00000049130		2.347658076	1.79E-37	9.32E-35
ENSMMUG00000063914		2.487396061	5.04E-52	1.47E-48
ENSMMUG00000023263	mediator complex subunit 15 [Source:VGNC Symbol;Acc:VGNC:74511]	2.422158818	9.68E-19	6.45E-17
ENSMMUG00000017171	scavenger receptor class F member 2 [Source:VGNC Symbol;Acc:VGNC:77058]	-0.993601806	0.004400361	0.016100691
ENSMMUG00000011547	reticulon 4 receptor [Source:VGNC Symbol;Acc:VGNC:77047]	0.548121353	0.014450041	0.043908914
ENSMMUG00000021904	cell division cycle 45 [Source:VGNC Symbol;Acc:VGNC:70800]	0.758515743	0.000102646	0.000610292
ENSMMUG00000031106	ubiquitin recognition factor in ER associated degradation 1 [Source:NCBI gene;Acc:711716]	0.793478967	9.53E-10	1.67E-08
ENSMMUG00000009650	HIR histone cell cycle regulation defective homolog A (S. cerevisiae) [Source:NCBI gene;Acc:719142]	-0.260864987	0.011550077	0.036440787
ENSMMUG00000005432	clathrin heavy chain like 1 [Source:VGNC Symbol;Acc:VGNC:71268]	1.425895182	9.10E-10	1.60E-08
ENSMMUG00000019925	DiGeorge syndrome critical region gene 2 [Source:NCBI gene;Acc:100423715]	1.216336192	1.59E-09	2.68E-08
ENSMMUG00000062702		1.409990486	0.001429941	0.006157302
ENSMMUG00000020799	BH3 interacting domain death agonist [Source:VGNC Symbol;Acc:VGNC:70250]	1.87852032	2.51E-07	2.79E-06
ENSMMUG00000021476	BCL2 like 13 [Source:VGNC Symbol;Acc:VGNC:84544]	2.539124619	2.39E-08	3.22E-07
ENSMMUG00000058916		2.635137871	9.95E-15	3.95E-13
ENSMMUG00000021477	ATPase H+ transporting V1 subunit E1 [Source:NCBI gene;Acc:709739]	2.624318792	3.78E-08	4.92E-07
ENSMMUG00000031121	solute carrier family 25 member 18 [Source:VGNC Symbol;Acc:VGNC:77434]	-1.293420743	7.55E-06	5.92E-05
ENSMMUG00000006731	CECR2 histone acetyl-lysine reader [Source:VGNC Symbol;Acc:VGNC:84530]	-1.990272837	1.29E-13	4.28E-12
ENSMMUG00000059302	Metazoan signal recognition particle RNA [Source:RFAM;Acc:RF00017]	1.820344824	0.000162233	0.000917488
ENSMMUG00000049841	Metazoan signal recognition particle RNA [Source:RFAM;Acc:RF00017]	-2.743442012	0.000334763	0.001733068
ENSMMUG00000009953	ninein like [Source:VGNC Symbol;Acc:VGNC:75273]	1.694876714	1.59E-07	1.85E-06
ENSMMUG00000002397	GINS complex subunit 1 [Source:VGNC Symbol;Acc:VGNC:73025]	1.866730761	5.73E-10	1.06E-08
ENSMMUG00000059374		2.195973423	9.39E-11	1.98E-09
ENSMMUG00000009201	abhydrolase domain containing 12 [Source:VGNC Symbol;Acc:VGNC:69411]	1.555315463	3.80E-07	4.06E-06
ENSMMUG00000060399	Metazoan signal recognition particle RNA [Source:RFAM;Acc:RF00017]	1.308114519	0.001929167	0.008001235
ENSMMUG00000009199	glycogen phosphorylase B [Source:VGNC Symbol;Acc:VGNC:76631]	1.671666169	8.48E-08	1.03E-06
ENSMMUG00000052572		1.484335571	0.003446388	0.013098078
ENSMMUG00000058095	visual system homeobox 1 [Source:NCBI gene;Acc:715973]	1.663067587	0.004000717	0.014847696
ENSMMUG00000052227		1.454080839	0.002682996	0.010529889
ENSMMUG00000020597	acyl-CoA synthetase short chain family member 1 [Source:VGNC Symbol;Acc:VGNC:69416]	1.070332006	0.002490534	0.00992731
ENSMMUG00000061222	adipocyte plasma membrane associated protein [Source:NCBI gene;Acc:704948]	0.99714412	0.000914359	0.004195721

ENSMMUG00000049391		2.286780225	0.000459154	0.002298457
ENSMMUG00000042668	cilia and flagella associated protein 61 [Source:NCBI gene;Acc:699814]	-0.885796666	0.0015314	0.006538022
ENSMMUG00000007709	crooked neck pre-mRNA splicing factor 1 [Source:VGNC Symbol;Acc:VGNC:71412]	-1.136208813	2.61E-08	3.49E-07
ENSMMUG00000010114	Ras and Rab interactor 2 [Source:VGNC Symbol;Acc:VGNC:76770]	-1.069542201	7.25E-05	0.000448968
ENSMMUG00000002321	solute carrier family 24 member 3 [Source:VGNC Symbol;Acc:VGNC:77426]	-1.070760776	4.64E-05	0.000304623
ENSMMUG00000012354	NADH:ubiquinone oxidoreductase complex assembly factor 5 [Source:VGNC Symbol;Acc:VGNC:74964]	1.036014386	0.012467109	0.03887481
ENSMMUG00000062485	ESF1 nucleolar pre-rRNA processing protein homolog [Source:NCBI gene;Acc:694159]	0.941845129	0.000233166	0.001264282
ENSMMUG00000006519	taspase 1 [Source:VGNC Symbol;Acc:VGNC:78129]	2.380880497	0.005260697	0.018762207
ENSMMUG00000011064	synaptosome associated protein 25 [Source:VGNC Symbol;Acc:VGNC:77663]	3.242505257	2.01E-13	6.45E-12
ENSMMUG00000015923	ankyrin repeat and EF-hand domain containing 1 [Source:VGNC Symbol;Acc:VGNC:69615]	-0.908284951	0.00096909	0.004405376
ENSMMUG00000021137	thioredoxin related transmembrane protein 4 [Source:VGNC Symbol;Acc:VGNC:79484]	-1.687862465	6.95E-05	0.000432152
ENSMMUG00000055897		1.462306795	1.93E-15	8.72E-14
ENSMMUG00000057687		1.699860793	4.66E-14	1.71E-12
ENSMMUG00000064554	leucine rich repeat neuronal 4 [Source:NCBI gene;Acc:718283]	2.283099332	2.63E-07	2.91E-06
ENSMMUG00000013740	minichromosome maintenance 8 homologous recombination repair factor [Source:VGNC Symbol;Acc:VGNC:74516]	2.485315761	6.90E-11	1.49E-09
ENSMMUG00000056093	shieldin complex subunit 1 [Source:NCBI gene;Acc:718152]	0.864323389	1.34E-05	9.95E-05
ENSMMUG00000064391		1.147298476	2.38E-07	2.66E-06
ENSMMUG00000065232		-2.377182181	0.003445558	0.013098078
ENSMMUG00000013261	CDP-diacylglycerol synthase 2 [Source:VGNC Symbol;Acc:VGNC:70995]	-1.01761497	0.015508064	0.046540199
ENSMMUG00000020004	Ras association domain family member 2 [Source:VGNC Symbol;Acc:VGNC:76674]	-0.758118532	0.000414996	0.002100556
ENSMMUG00000020912	spermine oxidase [Source:NCBI gene;Acc:718761]	-0.685277978	0.004386871	0.016059416
ENSMMUG00000022070	sialic acid binding Ig like lectin 1 [Source:VGNC Symbol;Acc:VGNC:77220]	0.390070931	0.000452041	0.002269886
ENSMMUG00000022076	ADAM metallopeptidase domain 33 [Source:VGNC Symbol;Acc:VGNC:69409]	0.628911247	0.007054091	0.024066021
ENSMMUG00000063747		-1.065106561	2.21E-05	0.000155834
ENSMMUG00000010028	leucine zipper tumor suppressor family member 3 [Source:VGNC Symbol;Acc:VGNC:74403]	1.534277977	6.59E-11	1.43E-09
ENSMMUG00000002982	FAST kinase domains 5 [Source:NCBI gene;Acc:100498658]	0.688546108	3.78E-05	0.000252419
ENSMMUG00000010678	oxytocin/neurophysin I prepropeptide [Source:VGNC Symbol;Acc:VGNC:75730]	1.171542647	0.013176041	0.040683965
ENSMMUG00000063622	mitochondrial ribosomal protein S26 [Source:NCBI gene;Acc:717038]	2.153466058	1.06E-11	2.61E-10
ENSMMUG00000005878	protein tyrosine phosphatase receptor type A [Source:VGNC Symbol;Acc:VGNC:76610]	0.381104427	0.001561908	0.00665654
ENSMMUG00000005204	VPS16 core subunit of CORVET and HOPS complexes [Source:VGNC Symbol;Acc:VGNC:78966]	-2.151397484	4.58E-08	5.85E-07
ENSMMUG00000005200	PC-esterase domain containing 1A [Source:VGNC Symbol;Acc:VGNC:75622]	-3.431744646	0.000329461	0.001709268
ENSMMUG00000021332	carboxypeptidase X, M14 family member 1 [Source:VGNC Symbol;Acc:VGNC:71413]	-2.878017732	0.016871407	0.049835826
ENSMMUG00000021331	EBF family member 4 [Source:VGNC Symbol;Acc:VGNC:71932]	-3.396271355	1.22E-06	1.15E-05
ENSMMUG00000061433		-0.83464771	6.61E-05	0.000413385
ENSMMUG00000013889	transmembrane channel like 2 [Source:VGNC Symbol;Acc:VGNC:79039]	-2.533693883	0.002686895	0.010542347
ENSMMUG00000010050	small nuclear ribonucleoprotein polypeptides B and B1 [Source:VGNC Symbol;Acc:VGNC:77676]	-2.319566036	4.32E-06	3.58E-05
ENSMMUG00000018474	signal regulatory protein alpha [Source:NCBI gene;Acc:717811]	-1.974519444	7.79E-12	1.97E-10

ENSMMUG00000015994	NSFL1 cofactor [Source:VGNC Symbol;Acc:VGNC:75407]	-<br>2.831752651	0.005084713	0.018232972
ENSMMUG00000018165	syndecan binding protein 2 [Source:VGNC Symbol;Acc:VGNC:77017]	-1.65539273	0.002290571	0.009262319
ENSMMUG00000004711	transmembrane protein 74B [Source:VGNC Symbol;Acc:VGNC:78990]	-<br>1.668360469	0.012582036	0.039157579
ENSMMUG00000004709	proteasome inhibitor subunit 1 [Source:VGNC Symbol;Acc:VGNC:76458]	-<br>1.461648677	0.003279219	0.012567889
ENSMMUG00000031223	R-spondin 4 [Source:VGNC Symbol;Acc:VGNC:77023]	-<br>3.301358661	0.015241333	0.045862815
ENSMMUG00000047879	family with sequence similarity 110 member A [Source:NCBI gene;Acc:717055]	-<br>1.384153336	0.003160471	0.012157652
ENSMMUG00000021670	solute carrier family 52 member 3 [Source:VGNC Symbol;Acc:VGNC:77717]	-<br>1.910578626	6.35E-15	2.62E-13
ENSMMUG00000056387	transcription factor 15 [Source:NCBI gene;Acc:716916]	-<br>2.963088724	1.77E-13	5.74E-12
ENSMMUG00000031224	casein kinase 2 alpha 1 [Source:NCBI gene;Acc:714841]	-<br>1.019871514	1.12E-06	1.06E-05
ENSMMUG00000004461	TBC1 domain family member 20 [Source:VGNC Symbol;Acc:VGNC:78132]	-<br>0.384050502	0.000633167	0.003038741
ENSMMUG00000026836	Y RNA [Source:RFAM;Acc:RF00019]	-<br>1.500637568	5.23E-05	0.00033721
ENSMMUG00000012601	RANBP2-type and C3HC4-type zinc finger containing 1 [Source:VGNC Symbol;Acc:VGNC:81554]	-<br>0.643728614	3.57E-05	0.000239689
ENSMMUG00000038935	zinc finger CCHC-type containing 3 [Source:NCBI gene;Acc:714477]	-<br>1.070542504	0.000620685	0.002987716
ENSMMUG00000017286	chromosome 10 C20orf96 homolog [Source:VGNC Symbol;Acc:VGNC:70324]	-<br>0.939220084	0.008147188	0.027245381
ENSMMUG00000061092	defensin beta 125 [Source:HGNC Symbol;Acc:HGNC:18105]	-<br>0.496562228	0.0031803	0.012217766
ENSMMUG00000005116	defensin beta 124 [Source:VGNC Symbol;Acc:VGNC:71655]	0.456182378	0.016631239	0.049330405
ENSMMUG00000058583	RRAD and GEM like GTPase 1 [Source:NCBI gene;Acc:713351]	-<br>1.226755178	1.69E-12	4.74E-11
ENSMMUG00000005119	histocompatibility minor 13 [Source:VGNC Symbol;Acc:VGNC:81418]	-<br>2.682874969	0.000130509	0.000758872
ENSMMUG00000051112	p53 and DNA damage regulated 1 [Source:NCBI gene;Acc:712622]	-<br>1.313736898	0.000296782	0.001560897
ENSMMUG00000000117	HCK proto-oncogene, Src family tyrosine kinase [Source:VGNC Symbol;Acc:VGNC:73353]	-<br>1.511561917	2.86E-05	0.000196418
ENSMMUG00000008979	protein O-fucosyltransferase 1 [Source:VGNC Symbol;Acc:VGNC:76030]	0.892848545	0.001206004	0.005315852
ENSMMUG00000009414	Sad1 and UNC84 domain containing 5 [Source:VGNC Symbol;Acc:VGNC:78133]	1.121159064	0.000268379	0.001433293
ENSMMUG00000053133	BPI fold containing family B member 2 [Source:NCBI gene;Acc:710915]	0.833421899	5.89E-05	0.000374254
ENSMMUG00000020671	BPI fold containing family B member 3 [Source:VGNC Symbol;Acc:VGNC:70320]	-0.37464472	0.005597473	0.019822283
ENSMMUG00000012912	itchy E3 ubiquitin protein ligase [Source:VGNC Symbol;Acc:VGNC:73589]	-<br>0.297798928	0.003771844	0.014146363
ENSMMUG00000055449	tumor protein p53 inducible nuclear protein 2 [Source:NCBI gene;Acc:707113]	-2.74952294	0.001899032	0.007892003
ENSMMUG00000017931	gamma-glutamyltransferase 7 [Source:VGNC Symbol;Acc:VGNC:72921]	-<br>1.307557823	0.000217823	0.001195355
ENSMMUG00000017932	acyl-CoA synthetase short chain family member 2 [Source:VGNC Symbol;Acc:VGNC:69410]	-2.29466185	0.001684497	0.007120537
ENSMMUG00000000229	transient receptor potential cation channel subfamily C member 4 associated protein [Source:VGNC Symbol;Acc:VGNC:79014]	-<br>2.089119732	0.000477591	0.002375405
ENSMMUG00000057979	growth differentiation factor 5 [Source:NCBI gene;Acc:705432]	0.716550497	1.99E-07	2.26E-06
ENSMMUG00000031274	GDF5 antisense RNA 1 [Source:HGNC Symbol;Acc:HGNC:33435]	0.644726511	0.00848849	0.028237342
ENSMMUG00000031284	RNA binding motif protein 39 [Source:NCBI gene;Acc:704198]	2.83677148	0.003001519	0.011632386
ENSMMUG00000010633	PHD finger protein 20 [Source:VGNC Symbol;Acc:VGNC:75807]	0.477781526	1.10E-06	1.05E-05

ENSMMUG00000020690	erythrocyte membrane protein band 4.1 like 1 [Source:VGNC Symbol;Acc:VGNC:72231]	-0.550032615	0.003409085	0.012990266
ENSMMUG00000020691	AAR2 splicing factor [Source:VGNC Symbol;Acc:VGNC:69412]	-0.726033716	0.000325785	0.001693222
ENSMMUG00000016448	DLG associated protein 4 [Source:NCBI gene;Acc:702928]	1.299609675	2.41E-12	6.62E-11
ENSMMUG00000059897	myosin light chain 9 [Source:NCBI gene;Acc:709784]	1.195413211	3.91E-05	0.000260777
ENSMMUG00000008195	TGFB induced factor homeobox 2 [Source:NCBI gene;Acc:100429616]	1.232195251	6.26E-14	2.22E-12
ENSMMUG00000056945		1.21513177	2.60E-13	8.19E-12
ENSMMUG00000008200	Src like adaptor 2 [Source:VGNC Symbol;Acc:VGNC:77499]	1.378627658	8.37E-06	6.49E-05
ENSMMUG00000014380	maestro heat like repeat family member 8 [Source:VGNC Symbol;Acc:VGNC:74774]	-0.680283808	0.0006388	0.00306274
ENSMMUG00000017224	catenin beta like 1 [Source:VGNC Symbol;Acc:VGNC:71414]	1.230081334	2.31E-07	2.59E-06
ENSMMUG00000049410	Metazoan signal recognition particle RNA [Source:RFAM;Acc:RF00017]	1.265147503	0.012712147	0.039467558
ENSMMUG00000017230	V-set and transmembrane domain containing 2 like [Source:NCBI gene;Acc:707394]	-1.441928238	1.91E-24	2.15E-22
ENSMMUG00000020172	KIAA1755 [Source:VGNC Symbol;Acc:VGNC:73942]	0.427520825	0.013678489	0.041988388
ENSMMUG00000020173	bactericidal permeability increasing protein [Source:VGNC Symbol;Acc:VGNC:70277]	0.371055467	0.007898319	0.026524503
ENSMMUG00000017350	Ral GTPase activating protein non-catalytic beta subunit [Source:VGNC Symbol;Acc:VGNC:76562]	-0.753008618	0.001932198	0.008011513
ENSMMUG00000031309	adipogenin [Source:VGNC Symbol;Acc:VGNC:84540]	-0.537203612	0.008805157	0.029170594
ENSMMUG00000006738	L3MBTL histone methyl-lysine binding protein 1 [Source:NCBI gene;Acc:695694]	1.877026677	4.80E-13	1.46E-11
ENSMMUG00000007925	serum/glucocorticoid regulated kinase 2 [Source:VGNC Symbol;Acc:VGNC:77228]	2.755763785	1.65E-13	5.40E-12
ENSMMUG00000000396	intraflagellar transport 52 [Source:NCBI gene;Acc:694933]	2.503792158	8.95E-19	6.02E-17
ENSMMUG00000018137	MYB proto-oncogene like 2 [Source:VGNC Symbol;Acc:VGNC:75092]	2.304619968	3.87E-19	2.70E-17
ENSMMUG00000002810	TOX high mobility group box family member 2 [Source:VGNC Symbol;Acc:VGNC:79007]	1.23014668	9.59E-08	1.15E-06
ENSMMUG00000022865	junctophilin 2 [Source:NCBI gene;Acc:694158]	-4.722690129	1.62E-07	1.88E-06
ENSMMUG00000006464	hepatocyte nuclear factor 4 alpha [Source:VGNC Symbol;Acc:VGNC:73492]	-0.706486868	0.002170321	0.008869821
ENSMMUG00000063590		1.248490399	3.21E-07	3.49E-06
ENSMMUG00000059766		-0.343093467	0.014266403	0.043488299
ENSMMUG00000045256	cellular communication network factor 5 [Source:NCBI gene;Acc:717872]	1.269006181	0.002178287	0.008887387
ENSMMUG00000003402	matrilin 4 [Source:NCBI gene;Acc:717652]	1.715899277	0.002378258	0.009542573
ENSMMUG00000003806	epididymal peptidase inhibitor [Source:NCBI gene;Acc:574162]	0.674615994	0.015722959	0.047058812
ENSMMUG00000021022	deoxynucleotidyltransferase terminal interacting protein 1 [Source:VGNC Symbol;Acc:VGNC:71845]	1.178262163	0.000293182	0.001544755
ENSMMUG00000052867		1.141640252	0.001230998	0.005411253
ENSMMUG00000023515	sorting nexin family member 21 [Source:VGNC Symbol;Acc:VGNC:77716]	0.484209197	0.006790016	0.023274336
ENSMMUG00000051201	zinc finger SWIM-type containing 3 [Source:NCBI gene;Acc:708804]	1.70003497	2.17E-11	5.10E-10
ENSMMUG00000042855	zinc finger SWIM-type containing 1 [Source:NCBI gene;Acc:717180]	1.230791872	0.000272677	0.001454111
ENSMMUG00000009957	spermatogenesis associated 25 [Source:VGNC Symbol;Acc:VGNC:77928]	1.714306228	0.007950637	0.026673992
ENSMMUG00000058326	neuralized E3 ubiquitin protein ligase 2 [Source:NCBI gene;Acc:708904]	3.02978671	7.62E-07	7.53E-06
ENSMMUG00000009960	cathepsin A [Source:VGNC Symbol;Acc:VGNC:71617]	1.956660145	4.51E-17	2.44E-15
ENSMMUG00000013697	PDX1 C-terminal inhibiting factor 1 [Source:VGNC Symbol;Acc:VGNC:75621]	0.365783183	0.005492162	0.019477813
ENSMMUG00000016549	matrix metallopeptidase 9 [Source:VGNC Symbol;Acc:VGNC:74903]	3.312425575	1.08E-16	5.66E-15
ENSMMUG00000016553	solute carrier family 12 member 5 [Source:VGNC Symbol;Acc:VGNC:77471]	4.298376077	0.000591443	0.002863073
ENSMMUG00000016927	solute carrier family 2 member 10 [Source:VGNC Symbol;Acc:VGNC:77465]	-1.550713203	5.42E-15	2.28E-13
ENSMMUG00000065008		-0.940957589	0.005177603	0.018506716
ENSMMUG00000012020	nuclear receptor coactivator 3 [Source:VGNC Symbol;Acc:VGNC:75147]	-0.588016999	0.000235817	0.001276749

ENSMMUG00000012029	sulfatase 2 [Source:VGNC Symbol;Acc:VGNC:78179]	-1.062104207	1.65E-06	1.51E-05
ENSMMUG00000060780		0.461156457	0.000181118	0.001011882
ENSMMUG00000038447		-0.942076133	0.000118677	0.000695931
ENSMMUG00000058754		-3.075569288	0.002208044	0.008993648
ENSMMUG00000019635	ADP ribosylation factor guanine nucleotide exchange factor 2 [Source:VGNC Symbol;Acc:VGNC:69836]	0.388443451	0.005199103	0.018574431
ENSMMUG00000023621	DEAD-box helicase 27 [Source:NCBI gene;Acc:703852]	-3.186468553	0.002522392	0.010037782
ENSMMUG00000002124	beta-1,4-galactosyltransferase 5 [Source:VGNC Symbol;Acc:VGNC:70212]	1.389786487	7.86E-07	7.73E-06
ENSMMUG00000002715	solute carrier family 9 member A8 [Source:VGNC Symbol;Acc:VGNC:77749]	1.394501872	6.19E-15	2.56E-13
ENSMMUG00000028471	U7 small nuclear RNA [Source:RFAM;Acc:RF00066]	1.051217442	0.005003063	0.017966779
ENSMMUG00000000740	spermatogenesis associated 2 [Source:VGNC Symbol;Acc:VGNC:77923]	1.453363262	9.84E-09	1.44E-07
ENSMMUG00000065129		1.947894035	0.00769415	0.025987913
ENSMMUG00000012228	ubiquitin conjugating enzyme E2 V1 [Source:NCBI gene;Acc:100425552]	1.429337794	3.01E-14	1.14E-12
ENSMMUG00000002761	activity dependent neuroprotector homeobox [Source:VGNC Symbol;Acc:VGNC:69614]	-0.585706554	6.24E-06	5.00E-05
ENSMMUG00000002758	molybdenum cofactor synthesis 3 [Source:VGNC Symbol;Acc:VGNC:74772]	-2.16243121	0.004099146	0.01513571
ENSMMUG00000012657	nuclear factor of activated T cells 2 [Source:VGNC Symbol;Acc:VGNC:75327]	-1.309924201	0.00139392	0.006029225
ENSMMUG00000018846	ZFP64 zinc finger protein [Source:VGNC Symbol;Acc:VGNC:78994]	-0.443194269	0.000319182	0.001663062
ENSMMUG00000013035	teashirt zinc finger homeobox 2 [Source:VGNC Symbol;Acc:VGNC:78875]	-1.020218766	0.000122375	0.000714443
ENSMMUG00000018358	zinc finger protein 217 [Source:VGNC Symbol;Acc:VGNC:78874]	-2.097192944	6.65E-14	2.34E-12
ENSMMUG00000057302		-2.328029166	2.28E-05	0.000160346
ENSMMUG00000052326	U7 small nuclear RNA [Source:RFAM;Acc:RF00066]	-3.057692042	0.004726214	0.017099297
ENSMMUG00000004074	bone morphogenetic protein 7 [Source:VGNC Symbol;Acc:VGNC:70266]	0.859362154	0.000883408	0.004070396
ENSMMUG00000031391	RNA binding motif protein 38 [Source:VGNC Symbol;Acc:VGNC:76484]	3.136922693	9.49E-15	3.79E-13
ENSMMUG00000022586	phosphoenolpyruvate carboxykinase 1 [Source:VGNC Symbol;Acc:VGNC:75779]	3.894878029	0.001574752	0.006705381
ENSMMUG00000022589	prostate transmembrane protein, androgen induced 1 [Source:VGNC Symbol;Acc:VGNC:76023]	-0.90967488	0.000297661	0.001563821
ENSMMUG00000004560	crystallin lambda 1 [Source:VGNC Symbol;Acc:VGNC:71433]	-0.427406547	0.011161643	0.035465967
ENSMMUG00000016475	M-phase phosphoprotein 8 [Source:VGNC Symbol;Acc:VGNC:74864]	-0.691245435	7.01E-06	5.56E-05
ENSMMUG00000004237	TNF receptor superfamily member 19 [Source:VGNC Symbol;Acc:VGNC:78588]	-1.023092191	2.13E-07	2.41E-06
ENSMMUG00000014610	ring finger protein 17 [Source:NCBI gene;Acc:708198]	-0.851578883	2.02E-06	1.81E-05
ENSMMUG00000018419	myotubularin related protein 6 [Source:VGNC Symbol;Acc:VGNC:75075]	0.728172321	3.08E-06	2.65E-05
ENSMMUG00000008520	ATPase phospholipid transporting 8A2 [Source:VGNC Symbol;Acc:VGNC:70062]	0.366984144	0.002889629	0.011261807
ENSMMUG00000057852		-0.838574884	0.000199242	0.001100446
ENSMMUG00000057481		2.498442607	0.003871235	0.014444595
ENSMMUG00000058786	ligand of numb-protein X 2 [Source:NCBI gene;Acc:710660]	-0.552150429	0.004009512	0.014873564
ENSMMUG00000052070	caudal type homeobox 2 [Source:NCBI gene;Acc:711094]	-0.643366774	0.004678745	0.016944424
ENSMMUG00000016138	ureidoimidazoline (2-oxo-4-hydroxy-4-carboxy-5-) decarboxylase [Source:NCBI gene;Acc:721710]	-1.562057224	0.001147833	0.00508721

ENSMMUG00000003771	ubiquitin like 3 [Source:VGNC Symbol;Acc:VGNC:78701]	-0.912076739	0.010064806	0.032593469
ENSMMUG00000055365		-1.489309989	7.58E-05	0.000468191
ENSMMUG00000005572	arachidonate 5-lipoxygenase activating protein [Source:VGNC Symbol;Acc:VGNC:69799]	-0.862255866	0.00045903	0.002298457
ENSMMUG00000013707	heat shock protein family H (Hsp110) member 1 [Source:NCBI gene;Acc:721956]	-0.758953933	1.59E-06	1.46E-05
ENSMMUG00000013713	beta 3-glucosyltransferase [Source:NCBI gene;Acc:713498]	1.75391169	3.16E-07	3.44E-06
ENSMMUG00000007192	FRY microtubule binding protein [Source:NCBI gene;Acc:713688]	1.213007121	0.000278807	0.001484079
ENSMMUG00000038997	zygote arrest 1 like [Source:NCBI gene;Acc:100427970]	3.083811167	7.99E-07	7.85E-06
ENSMMUG00000007197	BRCA2 DNA repair associated [Source:VGNC Symbol;Acc:VGNC:70279]	0.925545761	9.00E-06	6.93E-05
ENSMMUG00000007198	NEDD4 binding protein 2 like 1 [Source:VGNC Symbol;Acc:VGNC:75017]	1.617908664	0.000226881	0.00123573
ENSMMUG00000007200	NEDD4 binding protein 2 like 2 [Source:VGNC Symbol;Acc:VGNC:75031]	1.680688468	2.73E-14	1.04E-12
ENSMMUG00000049600		1.22330491	0.003911744	0.014565835
ENSMMUG00000055811		1.766872397	2.96E-09	4.73E-08
ENSMMUG00000007201	PDS5 cohesin associated factor B [Source:VGNC Symbol;Acc:VGNC:75835]	1.524038845	2.18E-11	5.13E-10
ENSMMUG00000025050	Y RNA [Source:RFAM;Acc:RF00019]	2.189502766	5.82E-05	0.000370362
ENSMMUG00000058110		1.480467975	3.41E-07	3.69E-06
ENSMMUG00000017052	StAR related lipid transfer domain containing 13 [Source:VGNC Symbol;Acc:VGNC:77962]	-0.437084265	0.000367144	0.001879285
ENSMMUG00000054254		-3.057943898	0.001863252	0.007752173
ENSMMUG00000019831	neurobeachin [Source:NCBI gene;Acc:714386]	-2.366985468	9.91E-09	1.45E-07
ENSMMUG00000016791	doublecortin like kinase 1 [Source:VGNC Symbol;Acc:VGNC:71745]	-2.460870298	1.42E-25	1.89E-23
ENSMMUG00000060579		-2.07787144	0.00262262	0.010351622
ENSMMUG00000001172	spermatogenesis and oogenesis specific basic helix-loop-helix 2 [Source:NCBI gene;Acc:693608]	-2.102567923	3.96E-07	4.21E-06
ENSMMUG00000003585	ALG5 dolichyl-phosphate beta-glucosyltransferase [Source:VGNC Symbol;Acc:VGNC:69647]	0.908987801	3.02E-06	2.60E-05
ENSMMUG00000012307	periostin [Source:VGNC Symbol;Acc:VGNC:76039]	-0.81550373	1.68E-09	2.82E-08
ENSMMUG00000030786	transient receptor potential cation channel subfamily C member 4 [Source:NCBI gene;Acc:695636]	-0.41080701	0.001587378	0.006753209
ENSMMUG00000002113	NHL repeat containing 3 [Source:VGNC Symbol;Acc:VGNC:75291]	-1.280508506	2.33E-09	3.82E-08
ENSMMUG00000002109	proline and serine rich 1 [Source:VGNC Symbol;Acc:VGNC:76291]	-0.846162643	1.45E-08	2.05E-07
ENSMMUG00000052676		-1.201806582	4.31E-11	9.50E-10
ENSMMUG00000011593	regulator of cell cycle [Source:VGNC Symbol;Acc:VGNC:76783]	-0.769181296	6.47E-05	0.000406069
ENSMMUG00000055161		-2.11130789	0.000224449	0.001224321
ENSMMUG00000023076	diacylglycerol kinase eta [Source:VGNC Symbol;Acc:VGNC:71786]	-1.189578288	6.77E-05	0.000422024
ENSMMUG00000055887		-1.536807994	4.43E-05	0.000292514
ENSMMUG00000023083	A-kinase anchoring protein 11 [Source:VGNC Symbol;Acc:VGNC:69765]	-1.146502608	1.49E-05	0.000109106
ENSMMUG00000054971		-1.492795693	0.015251203	0.045883017
ENSMMUG00000002330	ecto-NOX disulfide-thiol exchanger 1 [Source:VGNC Symbol;Acc:VGNC:72110]	2.835957844	1.22E-17	7.05E-16
ENSMMUG00000012636	potassium channel tetramerization domain containing 4 [Source:VGNC Symbol;Acc:VGNC:73892]	-0.692324343	0.013043261	0.04032927
ENSMMUG00000011986	chibby family member 2 [Source:VGNC Symbol;Acc:VGNC:78029]	-0.888675692	0.000870177	0.004023464

ENSMMUG00000017025	zinc finger CCCH-type containing 13 [Source:VGNC Symbol;Acc:VGNC:79248]	-0.516450109	0.000731287	0.003449291
ENSMMUG00000065094		1.418423798	0.000815455	0.003793368
ENSMMUG00000059778		1.944106284	1.37E-05	0.000101519
ENSMMUG00000063377	cysteinyl leukotriene receptor 2 [Source:NCBI gene;Acc:705450]	-1.080702309	0.003589481	0.013570946
ENSMMUG00000023756	fibronectin type III domain containing 3A [Source:VGNC Symbol;Acc:VGNC:72539]	-0.290342215	0.005343646	0.019020709
ENSMMUG00000010898	tripartite motif containing 13 [Source:VGNC Symbol;Acc:VGNC:79972]	0.290049949	0.012603824	0.039204125
ENSMMUG00000016530	NIMA related kinase 3 [Source:VGNC Symbol;Acc:VGNC:75309]	1.942960801	0.007397797	0.02515597
ENSMMUG00000018720	cytoskeleton associated protein 2 [Source:VGNC Symbol;Acc:VGNC:71229]	0.88464671	2.38E-06	2.09E-05
ENSMMUG00000007379	phosphatidylinositol 3,4,5-trisphosphate 3-phosphatase TPTE2-like [Source:NCBI gene;Acc:694255]	0.812931022	5.90E-05	0.000374474
ENSMMUG00000016401	SGT1 homolog, MIS12 kinetochore complex assembly cochaperone [Source:NCBI gene;Acc:694046]	0.809215082	5.43E-06	4.41E-05
ENSMMUG00000016404	chondromodulin [Source:VGNC Symbol;Acc:VGNC:71191]	2.192274581	0.001806473	0.00755921
ENSMMUG00000002657	olfactomedin 4 [Source:VGNC Symbol;Acc:VGNC:75418]	-1.543976588	7.20E-08	8.87E-07
ENSMMUG00000055156		0.770876144	0.011145853	0.035423538
ENSMMUG00000054675		-0.586572	0.014646115	0.044430421
ENSMMUG00000021197	TBC1 domain family member 4 [Source:VGNC Symbol;Acc:VGNC:78166]	-1.454305907	0.000198422	0.001097171
ENSMMUG00000046375		1.122066617	0.000287417	0.001522104
ENSMMUG00000061846		-0.397942989	0.007223618	0.024603969
ENSMMUG00000056466		-0.382441769	0.012714217	0.039467558
ENSMMUG00000021459	MYC binding protein 2 [Source:VGNC Symbol;Acc:VGNC:75011]	-1.020418077	4.70E-05	0.000307313
ENSMMUG00000054625		-0.5088057	0.001358836	0.005896537
ENSMMUG00000054008		-3.877674812	0.007119741	0.024278602
ENSMMUG00000054711		-1.537555617	4.80E-11	1.05E-09
ENSMMUG00000022744	muscleblind like splicing regulator 2 [Source:VGNC Symbol;Acc:VGNC:74519]	0.481287073	0.000547992	0.002677701
ENSMMUG00000009890	FERM, ARH/RhoGEF and pleckstrin domain protein 1 [Source:VGNC Symbol;Acc:VGNC:72614]	1.106808202	9.64E-25	1.13E-22
ENSMMUG00000009173	serine/threonine kinase 24 [Source:VGNC Symbol;Acc:VGNC:81583]	0.638637516	0.000204691	0.001127117
ENSMMUG00000001402	solute carrier family 15 member 1 [Source:VGNC Symbol;Acc:VGNC:77393]	-2.840112992	0.000392377	0.001995096
ENSMMUG00000012332	UBA domain containing 2 [Source:NCBI gene;Acc:703484]	-0.887380007	5.00E-10	9.35E-09
ENSMMUG00000060781	G protein-coupled receptor 18 [Source:NCBI gene;Acc:698714]	-0.716215343	0.011087143	0.03527551
ENSMMUG00000012338	G protein-coupled receptor 183 [Source:VGNC Symbol;Acc:VGNC:73042]	-0.864973913	1.54E-06	1.43E-05
ENSMMUG00000019556	transmembrane 9 superfamily member 2 [Source:VGNC Symbol;Acc:VGNC:78568]	0.529652887	4.06E-05	0.000270086
ENSMMUG00000004493	propionyl-CoA carboxylase subunit alpha [Source:VGNC Symbol;Acc:VGNC:75773]	-1.081111489	9.37E-05	0.000563371
ENSMMUG00000050578		-1.027374539	0.00765059	0.025870348
ENSMMUG00000015856	sodium leak channel, non-selective [Source:VGNC Symbol;Acc:VGNC:74991]	1.201032802	0.009866195	0.032070606
ENSMMUG00000002875	fibroblast growth factor 14 [Source:VGNC Symbol;Acc:VGNC:72645]	-3.438800676	0.011998449	0.037631381
ENSMMUG00000052642	protein O-glucosyltransferase 2 [Source:NCBI gene;Acc:702156]	-1.779111684	5.63E-06	4.56E-05
ENSMMUG00000000350	basic, immunoglobulin-like variable motif containing [Source:NCBI gene;Acc:100426213]	-0.485534764	0.012887679	0.03994631

ENSMMUG00000000353	putative methyltransferase-like protein 21E pseudogene [Source:NCBI gene;Acc:707949]	-0.532623268	0.010411672	0.033522908
ENSMMUG00000054803		0.876510294	0.001318724	0.005748213
ENSMMUG00000022282	collagen type IV alpha 2 chain [Source:NCBI gene;Acc:100429807]	4.554277843	1.07E-06	1.03E-05
ENSMMUG00000022818	inhibitor of growth family member 1 [Source:VGNC Symbol;Acc:VGNC:73742]	3.851974685	0.000259522	0.001390593
ENSMMUG00000064401		3.339996312	6.28E-05	0.00039603
ENSMMUG00000018929	ankyrin repeat domain 10 [Source:VGNC Symbol;Acc:VGNC:69646]	-0.401720817	0.001046435	0.004704151
ENSMMUG00000013138	Rho guanine nucleotide exchange factor 7 [Source:VGNC Symbol;Acc:VGNC:69833]	-0.992469149	1.43E-08	2.03E-07
ENSMMUG00000056306		-1.259122631	3.03E-08	3.99E-07
ENSMMUG00000048566	MCF.2 cell line derived transforming sequence like [Source:NCBI gene;Acc:713961]	1.292577829	1.87E-11	4.46E-10
ENSMMUG00000001036	coagulation factor VII [Source:VGNC Symbol;Acc:VGNC:72466]	1.262564017	7.37E-11	1.58E-09
ENSMMUG00000016740	defective in cullin neddylation 1 domain containing 2 [Source:VGNC Symbol;Acc:VGNC:71684]	-0.38634095	2.22E-05	0.000156944
ENSMMUG00000007423	transcription factor Dp-1 [Source:NCBI gene;Acc:715345]	1.803552153	3.49E-15	1.51E-13
ENSMMUG00000050639	transmembrane protein 255B [Source:NCBI gene;Acc:715939]	-0.515544327	0.004947973	0.017808538
ENSMMUG00000054800	chromosome 17 C13orf46 homolog [Source:NCBI gene;Acc:106994405]	-0.434683982	0.007734543	0.026069338
ENSMMUG00000023599	chromosome alignment maintaining phosphoprotein 1 [Source:VGNC Symbol;Acc:VGNC:71061]	0.950469936	6.40E-09	9.73E-08
ENSMMUG00000005222	double C2 domain beta [Source:VGNC Symbol;Acc:VGNC:71964]	2.072551305	0.010319094	0.033268724
ENSMMUG00000003042	VPS53 subunit of GARP complex [Source:VGNC Symbol;Acc:VGNC:79522]	0.88614668	0.004050799	0.014991463
ENSMMUG00000055344	TLC domain containing 3A [Source:NCBI gene;Acc:699707]	-1.561634136	3.29E-13	1.02E-11
ENSMMUG00000030401	gem nuclear organelle associated protein 4 [Source:VGNC Symbol;Acc:VGNC:72723]	-0.720060315	0.001422473	0.006130594
ENSMMUG00000011417	glyoxalase domain containing 4 [Source:VGNC Symbol;Acc:VGNC:72939]	-0.573308114	2.99E-05	0.00020424
ENSMMUG00000008130	ABR activator of RhoGEF and GTPase [Source:VGNC Symbol;Acc:VGNC:69589]	0.610518642	1.38E-09	2.36E-08
ENSMMUG00000002371	myosin IC [Source:VGNC Symbol;Acc:VGNC:75112]	0.556249227	0.000769975	0.003606032
ENSMMUG00000055241		0.741580245	4.15E-06	3.46E-05
ENSMMUG00000040268	phosphatidylinositol transfer protein alpha [Source:NCBI gene;Acc:721228]	0.687896051	0.002194918	0.008947705
ENSMMUG00000017561	solute carrier family 43 member 2 [Source:VGNC Symbol;Acc:VGNC:77554]	0.972655142	7.53E-06	5.91E-05
ENSMMUG00000039266	Metazoan signal recognition particle RNA [Source:RFAM;Acc:RF00017]	1.461403247	0.000267212	0.001428139
ENSMMUG00000002376	scavenger receptor class F member 1 [Source:VGNC Symbol;Acc:VGNC:77027]	1.738621902	2.89E-09	4.63E-08
ENSMMUG00000002377	Rab interacting lysosomal protein [Source:VGNC Symbol;Acc:VGNC:76866]	1.371322591	5.19E-05	0.000335055
ENSMMUG00000004820	pre-mRNA processing factor 8 [Source:VGNC Symbol;Acc:VGNC:76275]	1.070405671	7.12E-09	1.07E-07
ENSMMUG00000002380	WD repeat domain 81 [Source:NCBI gene;Acc:721254]	-0.361787581	0.005278054	0.018814878
ENSMMUG00000002382	serpin family F member 2 [Source:VGNC Symbol;Acc:VGNC:77372]	1.014491357	2.74E-05	0.000189075
ENSMMUG00000017518	small G protein signaling modulator 2 [Source:NCBI gene;Acc:703543]	-0.437417519	0.002229051	0.009062805
ENSMMUG00000053298		-0.865534033	8.54E-06	6.61E-05
ENSMMUG00000020527	clustered mitochondria homolog [Source:VGNC Symbol;Acc:VGNC:71370]	0.672506416	0.000201965	0.001114217
ENSMMUG00000022989	RAP1 GTPase activating protein 2 [Source:VGNC Symbol;Acc:VGNC:76561]	0.908616933	1.47E-05	0.000108135
ENSMMUG00000061370		1.633090029	0.01682958	0.049756274
ENSMMUG00000030389		1.54218667	1.27E-06	1.20E-05
ENSMMUG00000062583	olfactory receptor 1A1 [Source:NCBI gene;Acc:721403]	1.330814575	2.07E-11	4.91E-10
ENSMMUG00000012825	olfactory receptor 1A2 [Source:NCBI gene;Acc:100429763]	1.344966261	0.002463448	0.009840929
ENSMMUG00000042407		1.183007051	0.000671271	0.003204686
ENSMMUG00000055048		1.120256308	6.96E-12	1.78E-10

ENSMMUG00000008556	integrin subunit alpha E [Source:NCBI gene;Acc:721483]	0.545370501	4.99E-05	0.000324117
ENSMMUG00000063482		-0.836794353	0.00329879	0.012639058
ENSMMUG00000057632		3.946149061	2.52E-26	3.70E-24
ENSMMUG00000058966		-0.871676119	0.000473019	0.002355697
ENSMMUG00000005080	ubiquitin conjugating enzyme E2 G1 [Source:VGNC Symbol;Acc:VGNC:78695]	0.812069969	6.32E-08	7.86E-07
ENSMMUG00000060937	Metazoan signal recognition particle RNA [Source:RFAM;Acc:RF00017]	1.561343433	0.007704687	0.026016966
ENSMMUG00000030381	arachidonate 15-lipoxygenase [Source:VGNC Symbol;Acc:VGNC:69796]	-1.42888285	7.34E-08	9.03E-07
ENSMMUG00000017654	arrestin beta 2 [Source:VGNC Symbol;Acc:VGNC:70022]	1.462974448	0.000708913	0.003356331
ENSMMUG00000049649	proteasome 20S subunit beta 6 [Source:NCBI gene;Acc:710028]	1.076723194	1.30E-09	2.24E-08
ENSMMUG00000061951		1.326339481	4.24E-05	0.000281041
ENSMMUG00000005666	phospholipase D2 [Source:VGNC Symbol;Acc:VGNC:76172]	1.878412135	5.54E-14	1.98E-12
ENSMMUG00000012040	enolase 3 [Source:VGNC Symbol;Acc:VGNC:72220]	2.355630088	0.007804649	0.026250852
ENSMMUG00000009176	calmodulin binding transcription activator 2 [Source:VGNC Symbol;Acc:VGNC:70578]	0.719830323	0.000162266	0.000917488
ENSMMUG00000011344	solute carrier family 52 member 1 [Source:VGNC Symbol;Acc:VGNC:77795]	3.235161186	1.09E-06	1.04E-05
ENSMMUG00000018648	zinc finger protein 594 [Source:NCBI gene;Acc:711492]	0.720101196	0.001873477	0.007792487
ENSMMUG00000058586		-0.998265617	0.012671051	0.039350368
ENSMMUG00000006592	complement C1q binding protein [Source:NCBI gene;Acc:711817]	-0.46516478	0.014040984	0.042917111
ENSMMUG00000006594	DEAH-box helicase 33 [Source:VGNC Symbol;Acc:VGNC:71633]	-0.47779755	0.000250478	0.001347096
ENSMMUG00000006599	derlin 2 [Source:VGNC Symbol;Acc:VGNC:71654]	-0.444646734	0.00088142	0.004067684
ENSMMUG00000046195	MIS12 kinetochore complex component [Source:NCBI gene;Acc:712202]	-0.515718119	0.003769876	0.01414263
ENSMMUG00000043771	uncharacterized LOC712349 [Source:NCBI gene;Acc:712349]	-0.969929261	0.004435824	0.016210049
ENSMMUG00000030453	NLR family pyrin domain containing 1 [Source:NCBI gene;Acc:721648]	-0.741746963	1.90E-08	2.61E-07
ENSMMUG00000064520		4.078936156	3.23E-07	3.51E-06
ENSMMUG00000010536	discs large MAGUK scaffold protein 4 [Source:VGNC Symbol;Acc:VGNC:71920]	0.948690568	1.72E-13	5.61E-12
ENSMMUG00000052091	dishevelled segment polarity protein 2 [Source:NCBI gene;Acc:714381]	-0.985230804	0.00876816	0.029081164
ENSMMUG00000010543	PHD finger protein 23 [Source:VGNC Symbol;Acc:VGNC:75877]	-2.856718052	1.69E-06	1.54E-05
ENSMMUG00000064650	SPEM family member 3 [Source:NCBI gene;Acc:106993878]	0.846624699	3.58E-14	1.35E-12
ENSMMUG00000006327	tumor necrosis factor (ligand) superfamily, member 12 [Source:NCBI gene;Acc:100551506]	-0.580816847	1.23E-08	1.77E-07
ENSMMUG00000006332	SUMO specific peptidase 3 [Source:NCBI gene;Acc:715884]	0.669184687	0.000964194	0.004387972
ENSMMUG00000013501	CD68 molecule [Source:VGNC Symbol;Acc:VGNC:82121]	-2.29039372	2.01E-07	2.28E-06
ENSMMUG00000013503	mannose-P-dolichol utilization defect 1 [Source:VGNC Symbol;Acc:VGNC:74913]	-1.618185838	1.96E-06	1.75E-05
ENSMMUG00000013510	FMR1 autosomal homolog 2 [Source:NCBI gene;Acc:721889]	-0.582777336	0.009442964	0.030924165
ENSMMUG00000013513	sex hormone binding globulin [Source:VGNC Symbol;Acc:VGNC:77208]	-1.150539428	4.01E-05	0.000266995
ENSMMUG00000008639	tumor protein p53 [Source:VGNC Symbol;Acc:VGNC:78627]	-1.07721681	1.00E-06	9.68E-06
ENSMMUG00000008637	WD repeat containing antisense to TP53 [Source:VGNC Symbol;Acc:VGNC:79508]	0.807428703	0.001155478	0.005113832
ENSMMUG00000048204	ephrin B3 [Source:NCBI gene;Acc:716257]	1.216863522	5.69E-07	5.83E-06
ENSMMUG00000008173	lysine demethylase 6B [Source:VGNC Symbol;Acc:VGNC:73923]	-2.048610383	0.006750056	0.023175618
ENSMMUG00000063668	cytochrome b5 domain containing 1 [Source:NCBI gene;Acc:716429]	-1.335757057	1.70E-05	0.000122992
ENSMMUG00000009995	arachidonate 15-lipoxygenase type B [Source:VGNC Symbol;Acc:VGNC:69797]	1.182468396	1.57E-07	1.83E-06

ENSMMUG00000065208		0.920230099	0.002243128	0.009108477
ENSMMUG00000020329	arachidonate 12-lipoxygenase, 12R type [Source:NCBI gene;Acc:721936]	-0.891241363	0.01679891	0.049694239
ENSMMUG00000019850	arachidonate lipoxygenase 3 [Source:VGNC Symbol;Acc:VGNC:69607]	-1.800255751	2.00E-35	8.79E-33
ENSMMUG00000021798	period circadian regulator 1 [Source:VGNC Symbol;Acc:VGNC:75942]	-0.484719437	0.008580228	0.028535982
ENSMMUG00000021938	nudE neurodevelopment protein 1 like 1 [Source:VGNC Symbol;Acc:VGNC:74982]	0.757570638	6.72E-05	0.000419255
ENSMMUG00000055290	netrin 1 [Source:NCBI gene;Acc:717645]	1.001507667	6.09E-07	6.20E-06
ENSMMUG00000051001		-2.514274597	0.002049295	0.008439264
ENSMMUG00000060042		-0.94551643	0.002380035	0.009547068
ENSMMUG00000016723	zinc finger protein 287 [Source:NCBI gene;Acc:695524]	-1.285972883	0.000122915	0.00071731
ENSMMUG00000064698		-1.184476086	0.012612673	0.039210956
ENSMMUG00000013345	folliculin [Source:NCBI gene;Acc:698082]	-2.245506456	0.001302285	0.005681667
ENSMMUG00000021091	COP9 signalosome subunit 3 [Source:VGNC Symbol;Acc:VGNC:71316]	-2.359995305	7.50E-06	5.90E-05
ENSMMUG00000038569	5',3'-nucleotidase, mitochondrial [Source:NCBI gene;Acc:698591]	-0.860625569	0.000390171	0.001984573
ENSMMUG00000006491	mediator complex subunit 9 [Source:VGNC Symbol;Acc:VGNC:74577]	-0.885017536	0.000337982	0.001747865
ENSMMUG00000006493	ras related dexamethasone induced 1 [Source:VGNC Symbol;Acc:VGNC:76485]	-1.03998757	1.89E-09	3.14E-08
ENSMMUG00000023375	sterol regulatory element binding transcription factor 1 [Source:VGNC Symbol;Acc:VGNC:77880]	-0.727339636	0.000998033	0.00452129
ENSMMUG00000003349	target of myb1 like 2 membrane trafficking protein [Source:VGNC Symbol;Acc:VGNC:78617]	-1.59299704	0.010031589	0.032514873
ENSMMUG00000002942	dynein regulatory complex subunit 3 [Source:VGNC Symbol;Acc:VGNC:71906]	-1.168376274	0.010844537	0.034644029
ENSMMUG00000005900	GID complex subunit 4 homolog [Source:NCBI gene;Acc:699842]	-1.119623958	0.002231585	0.009069207
ENSMMUG00000021409	developmentally regulated GTP binding protein 2 [Source:VGNC Symbol;Acc:VGNC:71993]	-1.109732359	2.56E-05	0.000178178
ENSMMUG00000018344	myosin XVA [Source:VGNC Symbol;Acc:VGNC:75109]	0.726006423	5.10E-07	5.29E-06
ENSMMUG00000056257		-0.919669208	7.85E-05	0.000482254
ENSMMUG00000053675		-0.971178888	4.15E-06	3.46E-05
ENSMMUG00000005323	phosphoribosyl pyrophosphate synthetase associated protein 2 [Source:VGNC Symbol;Acc:VGNC:76430]	0.555274235	1.36E-06	1.27E-05
ENSMMUG00000051798	Small nucleolar RNA U3 [Source:RFAM;Acc:RF00012]	-3.502890223	0.001035153	0.004663161
ENSMMUG00000000140	epsin 2 [Source:VGNC Symbol;Acc:VGNC:72070]	-0.660372344	0.010093624	0.032657692
ENSMMUG00000008536	mitogen-activated protein kinase 7 [Source:VGNC Symbol;Acc:VGNC:74648]	1.809632952	8.62E-05	0.000523237
ENSMMUG00000008538	microfibril associated protein 4 [Source:VGNC Symbol;Acc:VGNC:74710]	1.399809024	0.006456888	0.022284847
ENSMMUG00000008540	ring finger protein 112 [Source:VGNC Symbol;Acc:VGNC:76724]	-1.833604869	9.36E-06	7.19E-05
ENSMMUG00000051286		-0.945786727	0.00283466	0.011071321
ENSMMUG00000030279	A-kinase anchoring protein 10 [Source:VGNC Symbol;Acc:VGNC:69764]	1.637681434	1.45E-06	1.35E-05
ENSMMUG00000018579	sperm antigen with calponin homology and coiled-coil domains 1 [Source:VGNC Symbol;Acc:VGNC:78021]	0.436508781	0.001035775	0.004664516
ENSMMUG00000024170	U6 spliceosomal RNA [Source:RFAM;Acc:RF00026]	1.034882357	7.29E-06	5.76E-05
ENSMMUG00000060502		1.319539582	5.32E-08	6.75E-07
ENSMMUG00000061335		1.290104154	1.73E-07	1.99E-06

ENSMMUG00000020226	WD repeat and SOCS box containing 1 [Source:VGNC Symbol;Acc:VGNC:79186]	0.799070039	1.61E-05	0.000117074
ENSMMUG00000054784		0.738590376	0.000178791	0.000999762
ENSMMUG00000065113		0.626468177	0.009518967	0.031116982
ENSMMUG00000061698		0.424277593	0.000374359	0.001910837
ENSMMUG00000051341		-0.745000411	0.00166845	0.007058864
ENSMMUG00000018719	nemo like kinase [Source:VGNC Symbol;Acc:VGNC:75277]	1.417126803	1.57E-20	1.28E-18
ENSMMUG00000063312		-0.914501463	2.12E-05	0.000150081
ENSMMUG00000054461	transmembrane protein 97 [Source:NCBI gene;Acc:707879]	-1.18418285	0.007975013	0.026731098
ENSMMUG00000019145	TNF alpha induced protein 1 [Source:VGNC Symbol;Acc:VGNC:78578]	-1.028088192	1.98E-05	0.000140948
ENSMMUG00000019146	DNA polymerase delta interacting protein 2 [Source:VGNC Symbol;Acc:VGNC:76099]	-0.770112148	0.000304591	0.001596768
ENSMMUG00000019148	vitronectin [Source:NCBI gene;Acc:708499]	-1.087130339	9.02E-05	0.000545336
ENSMMUG00000012240	phosphatidylinositol glycan anchor biosynthesis class S [Source:VGNC Symbol;Acc:VGNC:75800]	-0.550396502	0.006757408	0.023195381
ENSMMUG00000053611	stromal cell derived factor 2 [Source:NCBI gene;Acc:716189]	2.24623494	0.001522279	0.006504811
ENSMMUG00000004708	SPT6 homolog, histone chaperone and transcription elongation factor [Source:VGNC Symbol;Acc:VGNC:78057]	-0.529365526	0.001084256	0.004842345
ENSMMUG00000007293	RAB34, member RAS oncogene family [Source:VGNC Symbol;Acc:VGNC:81533]	-0.586985142	0.007176311	0.024465768
ENSMMUG00000026204	Small nucleolar RNA SNORD42 [Source:RFAM;Acc:RF00150]	-0.477291266	0.014435105	0.043881874
ENSMMUG00000051743	TLC domain containing 1 [Source:NCBI gene;Acc:710391]	-0.472338098	0.0133767	0.041216001
ENSMMUG00000021408	family with sequence similarity 222 member B [Source:VGNC Symbol;Acc:VGNC:72352]	0.735988199	1.85E-05	0.00013274
ENSMMUG00000012523	flotillin 2 [Source:NCBI gene;Acc:114669752]	0.91540698	5.05E-08	6.42E-07
ENSMMUG00000021553	dehydrogenase/reductase 13 [Source:VGNC Symbol;Acc:VGNC:81387]	0.913297936	3.16E-09	5.04E-08
ENSMMUG00000021554	PHD finger protein 12 [Source:VGNC Symbol;Acc:VGNC:75838]	0.913011793	2.70E-08	3.60E-07
ENSMMUG00000009938	myosin XVIIIA [Source:VGNC Symbol;Acc:VGNC:75058]	0.505030992	4.88E-06	4.01E-05
ENSMMUG00000019667	TAO kinase 1 [Source:VGNC Symbol;Acc:VGNC:81591]	1.277876132	0.000150883	0.000861167
ENSMMUG00000057447		4.528687715	7.05E-05	0.000437659
ENSMMUG00000014409	ankyrin repeat domain 13B [Source:VGNC Symbol;Acc:VGNC:69708]	0.756490967	0.001404814	0.006063472
ENSMMUG00000014413	slingshot protein phosphatase 2 [Source:VGNC Symbol;Acc:VGNC:78018]	0.379184931	0.001092993	0.004870889
ENSMMUG00000060080	U6 spliceosomal RNA [Source:RFAM;Acc:RF00026]	-1.013757496	0.001784988	0.007484389
ENSMMUG00000037644	carboxypeptidase D [Source:NCBI gene;Acc:712407]	0.559540244	0.015005451	0.045303061
ENSMMUG00000023698	golgi SNAP receptor complex member 1 [Source:VGNC Symbol;Acc:VGNC:73110]	-0.635322843	3.62E-06	3.06E-05
ENSMMUG00000001116	SUZ12 polycomb repressive complex 2 subunit [Source:VGNC Symbol;Acc:VGNC:78144]	4.023639793	0.000114184	0.000671207
ENSMMUG00000014689	myosin ID [Source:VGNC Symbol;Acc:VGNC:75113]	-0.764160688	0.001662739	0.0070388
ENSMMUG00000059292		0.669242581	6.18E-07	6.26E-06
ENSMMUG00000011411	sperm acrosome associated 3 [Source:VGNC Symbol;Acc:VGNC:77930]	2.464293199	3.19E-18	1.96E-16
ENSMMUG00000002324		0.925223876	1.17E-07	1.39E-06
ENSMMUG00000009782	C-C motif chemokine ligand 7 [Source:NCBI gene;Acc:714751]	-1.372593037	0.000285561	0.00151393
ENSMMUG00000006399	chaperonin containing TCP1 subunit 6B [Source:VGNC Symbol;Acc:VGNC:70748]	0.419706818	7.73E-07	7.63E-06
ENSMMUG00000004794	DNA ligase 3 [Source:VGNC Symbol;Acc:VGNC:74266]	2.51012255	1.67E-09	2.81E-08
ENSMMUG00000005170	unc-45 myosin chaperone B [Source:VGNC Symbol;Acc:VGNC:78954]	-1.140185815	2.46E-05	0.000171972
ENSMMUG00000037555	schlafen family member 11 [Source:NCBI gene;Acc:715511]	1.539859708	0.013460572	0.041448069

ENSMMUG00000060439	schlafen family member 12 like [Source:NCBI gene;Acc:106992394]	-0.812339046	0.00071992	0.003400089
ENSMMUG00000031736		-2.630630164	0.001426159	0.006142836
ENSMMUG00000026432	small nucleolar RNA, C/D box 7 [Source:HGNC Symbol;Acc:HGNC:32704]	-3.463528319	0.000717159	0.00338925
ENSMMUG00000006048	adaptor related protein complex 2 subunit beta 1 [Source:VGNC Symbol;Acc:VGNC:69964]	-0.790107168	1.07E-05	8.17E-05
ENSMMUG00000050758		-1.601332924	0.002024012	0.008351705
ENSMMUG00000062497		-1.274539332	0.002290534	0.009262319
ENSMMUG00000054472		1.072484118	2.96E-05	0.000202427
ENSMMUG00000054210	C-C motif chemokine 4 [Source:NCBI gene;Acc:100426632]	-1.242272435	1.28E-21	1.15E-19
ENSMMUG00000058233	C-C motif chemokine 3-like [Source:NCBI gene;Acc:100426537]	-1.838092854	0.001623244	0.006893504
ENSMMUG00000061594		-1.994181251	4.49E-05	0.000295925
ENSMMUG00000052786		-1.7106908	0.000249875	0.001344349
ENSMMUG00000000620	dehydrogenase/reductase 11 [Source:VGNC Symbol;Acc:VGNC:71792]	-1.204935816	0.000158251	0.000898281
ENSMMUG00000000618	gametogenetin binding protein 2 [Source:VGNC Symbol;Acc:VGNC:72746]	-1.084800322	0.00023643	0.001279114
ENSMMUG00000051190	Metazoan signal recognition particle RNA [Source:RFAM;Acc:RF00017]	-2.03310578	8.81E-05	0.000533901
ENSMMUG00000007920	myosin XIX [Source:VGNC Symbol;Acc:VGNC:75026]	-1.113470587	1.33E-07	1.57E-06
ENSMMUG00000007923	phosphatidylinositol glycan anchor biosynthesis class W [Source:VGNC Symbol;Acc:VGNC:75850]	-0.842478423	0.000399949	0.002029338
ENSMMUG00000053845		-0.696034061	0.003151166	0.012131488
ENSMMUG00000007917	carbonic anhydrase 4 [Source:NCBI gene;Acc:716919]	-0.860013091	0.000284518	0.00151116
ENSMMUG00000017113	ubiquitin specific peptidase 32 [Source:NCBI gene;Acc:716857]	-1.206103052	8.44E-19	5.73E-17
ENSMMUG00000052977	putative protein FAM106C [Source:NCBI gene;Acc:106994084]	-1.055528733	7.94E-08	9.69E-07
ENSMMUG00000065117	septin 4 [Source:HGNC Symbol;Acc:HGNC:9165]	-1.164364384	2.74E-07	3.02E-06
ENSMMUG00000008423	septin 4 [Source:NCBI gene;Acc:714724]	-0.749719377	1.22E-10	2.51E-09
ENSMMUG00000007004	ring finger protein 43 [Source:NCBI gene;Acc:710124]	-0.761747042	0.016617801	0.049300606
ENSMMUG00000056064	serine and arginine rich splicing factor 1 [Source:NCBI gene;Acc:713958]	-0.429820153	0.001409478	0.006081799
ENSMMUG00000004723	vascular endothelial zinc finger 1 [Source:NCBI gene;Acc:713848]	-0.475676532	0.001392837	0.006027887
ENSMMUG00000002269	serine carboxypeptidase 1 [Source:VGNC Symbol;Acc:VGNC:82232]	-0.619956079	0.000427824	0.002159473
ENSMMUG00000049376		1.929881028	0.016874848	0.049835826
ENSMMUG00000005793	ankyrin repeat and fibronectin type III domain containing 1 [Source:VGNC Symbol;Acc:VGNC:69712]	1.061730235	9.88E-07	9.53E-06
ENSMMUG00000059321		2.215573454	0.002456348	0.009815263
ENSMMUG00000006066	phosphatidylcholine transfer protein [Source:VGNC Symbol;Acc:VGNC:75789]	2.431223852	6.03E-06	4.85E-05
ENSMMUG00000006068	transmembrane protein 100 [Source:VGNC Symbol;Acc:VGNC:79521]	0.379073655	0.008900907	0.029427448
ENSMMUG00000022627	HLF transcription factor, PAR bZIP family member [Source:VGNC Symbol;Acc:VGNC:73478]	-1.0389056	0.000949248	0.004333851

ENSMMUG00000050544		-1.723469167	5.60E-08	7.07E-07
ENSMMUG00000049997		-1.434069223	1.71E-07	1.98E-06
ENSMMUG00000056602		-1.519641942	0.002769066	0.010841343
ENSMMUG00000010550	target of myb1 like 1 membrane trafficking protein [Source:VGNC Symbol;Acc:VGNC:78616]	-0.575884228	0.001349218	0.005860046
ENSMMUG00000060858		-1.697426483	0.000136162	0.000786721
ENSMMUG00000063189		-0.424450879	0.015425841	0.046341279
ENSMMUG00000001942	mbt domain containing 1 [Source:VGNC Symbol;Acc:VGNC:74538]	-1.37875988	3.57E-07	3.84E-06
ENSMMUG00000001940	NME/NM23 nucleoside diphosphate kinase 2 [Source:NCBI gene;Acc:100533957]	-1.806216916	1.67E-11	4.00E-10
ENSMMUG00000022741	WAP, follistatin/kazal, immunoglobulin, kunitz and netrin domain containing 2 [Source:VGNC Symbol;Acc:VGNC:79900]	0.530905234	0.001331819	0.005793134
ENSMMUG00000010117	LUC7 like 3 pre-mRNA splicing factor [Source:VGNC Symbol;Acc:VGNC:74346]	0.823665397	0.001221804	0.005374087
ENSMMUG00000003421	ankyrin repeat domain 40 [Source:VGNC Symbol;Acc:VGNC:69725]	0.993461707	0.00523515	0.018684847
ENSMMUG00000017256	calcium voltage-gated channel subunit alpha1 G [Source:VGNC Symbol;Acc:VGNC:70504]	1.658199722	6.76E-24	7.07E-22
ENSMMUG00000017252	spermatogenesis associated 20 [Source:VGNC Symbol;Acc:VGNC:77993]	-0.944378832	0.000148665	0.000850175
ENSMMUG00000017242	MYCBP associated protein [Source:VGNC Symbol;Acc:VGNC:74994]	1.997739719	2.45E-10	4.78E-09
ENSMMUG00000017234	leucine rich repeat containing 59 [Source:VGNC Symbol;Acc:VGNC:81436]	-2.828920156	8.97E-29	1.86E-26
ENSMMUG00000020061	protein phosphatase 1 regulatory subunit 9B [Source:VGNC Symbol;Acc:VGNC:76372]	-1.431612148	2.05E-06	1.83E-05
ENSMMUG00000014687	sterile alpha motif domain containing 14 [Source:VGNC Symbol;Acc:VGNC:77112]	-1.826234385	0.00070487	0.003341865
ENSMMUG00000014685	pyruvate dehydrogenase kinase 2 [Source:VGNC Symbol;Acc:VGNC:75933]	-1.428370942	0.00718764	0.024498644
ENSMMUG00000000153	integrin subunit alpha 3 [Source:VGNC Symbol;Acc:VGNC:73777]	-1.844731489	0.004054912	0.015002864
ENSMMUG00000015845	beta-1,4-N-acetyl-galactosaminyltransferase 2 [Source:VGNC Symbol;Acc:VGNC:70120]	-1.417936984	0.000814618	0.003790687
ENSMMUG00000012178	calcium binding and coiled-coil domain 2 [Source:VGNC Symbol;Acc:VGNC:70675]	1.854965363	1.83E-16	9.29E-15
ENSMMUG00000049405	gastric inhibitory polypeptide [Source:NCBI gene;Acc:698335]	-0.535588425	0.003046703	0.011788641
ENSMMUG00000021087	SNF8 subunit of ESCRT-II [Source:VGNC Symbol;Acc:VGNC:77729]	-0.475754064	0.00923343	0.030367958
ENSMMUG00000003436	tubulin tyrosine ligase like 6 [Source:VGNC Symbol;Acc:VGNC:79632]	0.997266016	8.93E-10	1.58E-08
ENSMMUG00000060879	homeobox B13 [Source:NCBI gene;Acc:697572]	0.966851638	2.03E-10	4.04E-09
ENSMMUG00000055122		0.807559122	1.57E-05	0.00011439
ENSMMUG00000052505	homeobox B9 [Source:NCBI gene;Acc:106994009]	1.279450261	5.02E-20	3.80E-18
ENSMMUG00000020868	homeobox B8 [Source:VGNC Symbol;Acc:VGNC:73505]	1.201580358	7.29E-14	2.54E-12
ENSMMUG00000055324	homeobox B7 [Source:NCBI gene;Acc:697089]	1.232282414	5.52E-08	6.99E-07
ENSMMUG00000062283	homeobox B6 [Source:NCBI gene;Acc:106994013]	1.113807448	3.68E-11	8.29E-10
ENSMMUG00000051420	homeobox B5 [Source:NCBI gene;Acc:696732]	0.966743468	2.93E-07	3.22E-06
ENSMMUG00000045203		1.2210986	0.000131262	0.000762346
ENSMMUG00000057001	homeobox B2 [Source:NCBI gene;Acc:106994010]	0.934287275	5.11E-06	4.17E-05
ENSMMUG00000057095		1.925294889	0.003663539	0.013807848
ENSMMUG00000050742	src kinase associated phosphoprotein 1 [Source:NCBI gene;Acc:697043]	0.479024478	7.72E-07	7.63E-06
ENSMMUG00000004760	CDK5 regulatory subunit associated protein 3 [Source:VGNC Symbol;Acc:VGNC:71015]	-0.884257243	0.000726334	0.003428155
ENSMMUG00000003325	pyridoxamine 5'-phosphate oxidase [Source:VGNC Symbol;Acc:VGNC:76141]	-0.794720514	3.24E-06	2.76E-05

ENSMMUG00000021486	secernin 2 [Source:NCBI gene;Acc:694571]	1.060146896	0.003361284	0.012824932
ENSMMUG00000057231	T-box transcription factor 21 [Source:NCBI gene;Acc:694044]	1.586070205	9.65E-15	3.84E-13
ENSMMUG00000019760	karyopherin subunit beta 1 [Source:VGNC Symbol;Acc:VGNC:74203]	-0.617025834	1.43E-06	1.33E-05
ENSMMUG00000020528	G protein-coupled receptor 179 [Source:VGNC Symbol;Acc:VGNC:73043]	-1.740853029	0.005475739	0.019429051
ENSMMUG00000020020	Rho GTPase activating protein 23 [Source:NCBI gene;Acc:693996]	2.012226262	7.27E-11	1.56E-09
ENSMMUG00000007799	LIM and SH3 protein 1 [Source:VGNC Symbol;Acc:VGNC:74237]	1.213099034	9.13E-05	0.00055164
ENSMMUG00000056248		4.256031536	1.25E-40	1.01E-37
ENSMMUG00000063779		4.385789005	1.65E-47	3.00E-44
ENSMMUG00000000289	calcium voltage-gated channel auxiliary subunit beta 1 [Source:VGNC Symbol;Acc:VGNC:70509]	-0.504316487	0.000906212	0.004163592
ENSMMUG00000030060	F-box and leucine rich repeat protein 20 [Source:VGNC Symbol;Acc:VGNC:81407]	-0.51925571	2.00E-05	0.000142664
ENSMMUG00000009132	mediator complex subunit 1 [Source:VGNC Symbol;Acc:VGNC:74724]	-0.605976114	0.004449834	0.016240832
ENSMMUG00000001405	protein phosphatase 1 regulatory inhibitor subunit 1B [Source:VGNC Symbol;Acc:VGNC:76369]	-0.339219162	0.013667708	0.041979183
ENSMMUG00000004587	gasdermin B [Source:VGNC Symbol;Acc:VGNC:73219]	1.427583063	0.005641311	0.019948369
ENSMMUG00000002923	colony stimulating factor 3 [Source:VGNC Symbol;Acc:VGNC:71520]	0.914702901	4.46E-06	3.69E-05
ENSMMUG00000002926	mediator complex subunit 24 [Source:NCBI gene;Acc:699536]	0.979767781	4.99E-14	1.80E-12
ENSMMUG00000002933	nuclear receptor subfamily 1 group D member 1 [Source:VGNC Symbol;Acc:VGNC:75502]	-0.721319496	0.000180331	0.001007872
ENSMMUG00000045187	CASC3 exon junction complex subunit [Source:NCBI gene;Acc:699712]	0.75969054	6.90E-06	5.48E-05
ENSMMUG00000015160	Rap guanine nucleotide exchange factor like 1 [Source:VGNC Symbol;Acc:VGNC:76501]	0.541198078	0.010985069	0.035012048
ENSMMUG00000008941	DNA topoisomerase II alpha [Source:VGNC Symbol;Acc:VGNC:78620]	-0.930418468	2.16E-11	5.08E-10
ENSMMUG00000062362	keratin 33A [Source:NCBI gene;Acc:705800]	-0.709567672	1.61E-05	0.000117137
ENSMMUG00000018644	keratin 32 [Source:VGNC Symbol;Acc:VGNC:74216]	-0.29725674	0.012552318	0.039090198
ENSMMUG00000018645	keratin 35 [Source:VGNC Symbol;Acc:VGNC:74217]	-0.330401285	0.009667092	0.031523221
ENSMMUG00000040413	keratin 13 [Source:NCBI gene;Acc:706830]	-0.58604425	0.00883707	0.02923634
ENSMMUG00000013048	junction plakoglobin [Source:VGNC Symbol;Acc:VGNC:73960]	0.7619969	6.14E-05	0.000387877
ENSMMUG00000016114	2',3'-cyclic nucleotide 3' phosphodiesterase [Source:VGNC Symbol;Acc:VGNC:71279]	0.506597956	0.000675547	0.003224041
ENSMMUG00000003894	zinc finger protein 385C [Source:NCBI gene;Acc:708928]	0.521163434	0.000185017	0.001032076
ENSMMUG00000049440	chromosome 16 C17orf113 homolog [Source:NCBI gene;Acc:106994045]	0.411273664	0.000105445	0.000625656
ENSMMUG00000003437	signal transducer and activator of transcription 5B [Source:NCBI gene;Acc:709760]	2.399282729	8.49E-19	5.74E-17
ENSMMUG00000052220		-0.574985024	0.004597362	0.016691271
ENSMMUG00000050793	N-acetyl-alpha-glucosaminidase [Source:NCBI gene;Acc:707186]	1.407844367	1.37E-17	7.89E-16
ENSMMUG00000009884	hydroxysteroid 17-beta dehydrogenase 1 [Source:VGNC Symbol;Acc:VGNC:73528]	1.792093993	3.97E-06	3.32E-05
ENSMMUG00000009886	Coenzyme A synthase [Source:VGNC Symbol;Acc:VGNC:81373]	1.318789707	2.20E-17	1.23E-15
ENSMMUG00000009887	MAX dimerization protein MLX [Source:VGNC Symbol;Acc:VGNC:74726]	1.2504294	3.74E-07	4.00E-06
ENSMMUG00000009889	PSMC3 interacting protein [Source:VGNC Symbol;Acc:VGNC:76282]	1.153599436	8.86E-12	2.23E-10
ENSMMUG00000016796	reticulophagy regulator family member 3 [Source:VGNC Symbol;Acc:VGNC:76845]	1.320857525	6.99E-18	4.11E-16
ENSMMUG00000016797	tubulin gamma 1 [Source:NCBI gene;Acc:710756]	1.51942237	1.77E-13	5.74E-12
ENSMMUG00000006086	pleckstrin homology, MyTH4 and FERM domain containing H3 [Source:VGNC Symbol;Acc:VGNC:76113]	1.150546304	0.000251077	0.001348928
ENSMMUG00000006091	contactin associated protein 1 [Source:VGNC Symbol;Acc:VGNC:71390]	1.542291515	2.85E-13	8.94E-12
ENSMMUG00000006093	enhancer of zeste 1 polycomb repressive complex 2 subunit [Source:VGNC Symbol;Acc:VGNC:72457]	1.47639167	3.83E-12	1.02E-10
ENSMMUG00000056565	Metazoan signal recognition particle RNA [Source:RFAM;Acc:RF00017]	2.489913424	0.010003133	0.032451584
ENSMMUG00000058566		1.404085559	1.33E-15	6.15E-14
ENSMMUG00000029971	beclin 1 [Source:VGNC Symbol;Acc:VGNC:70044]	0.440126808	0.006049041	0.021107755
ENSMMUG00000012685	proteasome activator subunit 3 [Source:VGNC Symbol;Acc:VGNC:76457]	0.358811935	0.009230472	0.030366535

ENSMMUG00000001870	amine oxidase copper containing 2 [Source:VGNC Symbol;Acc:VGNC:69955]	0.496589603	0.004265804	0.015679393
ENSMMUG00000014123	alanyl-tRNA editing protein Aarsd1 [Source:NCBI gene;Acc:712203]	1.154663708	2.15E-06	1.90E-05
ENSMMUG00000008091	RUN domain containing 1 [Source:VGNC Symbol;Acc:VGNC:77101]	0.943612763	0.000236903	0.001280719
ENSMMUG00000064920		1.212336698	1.50E-11	3.62E-10
ENSMMUG00000008090	ribosomal protein L27 [Source:NCBI gene;Acc:712352]	1.17690467	0.00033467	0.001733068
ENSMMUG00000015999	interferon induced protein 35 [Source:VGNC Symbol;Acc:VGNC:73564]	0.881929956	0.002715765	0.010641261
ENSMMUG00000016001	vesicle amine transport 1 [Source:VGNC Symbol;Acc:VGNC:79058]	1.008868362	2.33E-06	2.06E-05
ENSMMUG00000065269	Rho family GTPase 2 [Source:NCBI gene;Acc:712559]	1.02887784	9.83E-06	7.52E-05
ENSMMUG00000052607		1.119524633	4.73E-25	5.88E-23
ENSMMUG00000019923	ataxin 7 like 3 [Source:VGNC Symbol;Acc:VGNC:70128]	-0.620157126	5.57E-05	0.000356839
ENSMMUG00000017100	solute carrier family 4 member 1 (Diego blood group) [Source:NCBI gene;Acc:100427618]	-0.651752854	8.71E-07	8.47E-06
ENSMMUG00000013983	RUN domain containing 3A [Source:VGNC Symbol;Acc:VGNC:77032]	0.267126293	0.000510657	0.002519831
ENSMMUG00000041006	family with sequence similarity 171 member A2 [Source:NCBI gene;Acc:712099]	0.331889512	0.003531395	0.013368721
ENSMMUG00000029927	ADAM metallopeptidase domain 11 [Source:VGNC Symbol;Acc:VGNC:69466]	-0.553148797	0.009566937	0.03125271
ENSMMUG00000062273	dephospho-CoA kinase domain containing [Source:NCBI gene;Acc:715841]	1.063255126	0.008223439	0.027468782
ENSMMUG00000060665		-0.948867104	7.54E-05	0.000466402
ENSMMUG00000054246		-2.398182005	0.000135629	0.000784578
ENSMMUG00000021932	Rho GTPase activating protein 27 [Source:VGNC Symbol;Acc:VGNC:69935]	-1.571171184	0.001003355	0.004539613
ENSMMUG00000008459	pleckstrin homology and RUN domain containing M1 [Source:NCBI gene;Acc:716464]	-1.095475216	0.000536679	0.002626868
ENSMMUG00000049983		-1.183222774	0.004835184	0.017437128
ENSMMUG00000005548	N-ethylmaleimide sensitive factor, vesicle fusing ATPase [Source:VGNC Symbol;Acc:VGNC:75536]	0.665054481	3.47E-09	5.50E-08
ENSMMUG00000057209		-0.581488288	0.000562945	0.002741552
ENSMMUG00000053257	Y RNA [Source:RFAM;Acc:RF00019]	1.316913022	3.37E-10	6.49E-09
ENSMMUG00000063877	leucine-rich repeat-containing protein 37A2 [Source:NCBI gene;Acc:716147]	-0.405652546	0.000443217	0.002229426
ENSMMUG00000003289	integrin subunit beta 3 [Source:NCBI gene;Acc:717379]	0.729507743	2.06E-10	4.08E-09
ENSMMUG00000010588	EF-hand calcium binding domain 13 [Source:HGNC Symbol;Acc:HGNC:26864]	0.650993144	0.000685978	0.003266315
ENSMMUG00000002166	tousled like kinase 2 [Source:VGNC Symbol;Acc:VGNC:78368]	-2.4101307	0.002347105	0.009441027
ENSMMUG00000002168	membrane associated ring-CH-type finger 10 [Source:VGNC Symbol;Acc:VGNC:74593]	-1.642892334	0.007311726	0.024892404
ENSMMUG00000011809	platelet and endothelial cell adhesion molecule 1 [Source:VGNC Symbol;Acc:VGNC:75938]	-0.614048157	0.001759826	0.007385276
ENSMMUG00000009463	DEAD-box helicase 5 [Source:VGNC Symbol;Acc:VGNC:71763]	-0.34076527	0.009239721	0.030374905
ENSMMUG00000009459	centrosomal protein 95 [Source:VGNC Symbol;Acc:VGNC:71095]	-0.398985508	0.008779116	0.029104221
ENSMMUG00000063689		-0.754344226	0.002111813	0.00866011
ENSMMUG00000009461	SMAD specific E3 ubiquitin protein ligase 2 [Source:VGNC Symbol;Acc:VGNC:77727]	-0.573301873	0.000705063	0.003341865
ENSMMUG00000060767		2.638848693	4.69E-14	1.71E-12
ENSMMUG00000002959	helicase with zinc finger [Source:VGNC Symbol;Acc:VGNC:73178]	2.801933047	9.21E-19	6.17E-17
ENSMMUG00000057157		2.530377018	7.49E-16	3.58E-14
ENSMMUG00000063817	SRY-box transcription factor 9 [Source:NCBI gene;Acc:574208]	0.732262564	0.000202573	0.001116723
ENSMMUG00000054673		1.828063572	0.010414078	0.033522908
ENSMMUG00000001269	chromosome 16 C17orf80 homolog [Source:VGNC Symbol;Acc:VGNC:70440]	0.873618405	4.25E-10	8.04E-09

ENSMMUG00000049948		-1.57505189	1.18E-13	3.97E-12
ENSMMUG00000064463		1.67817729	2.04E-06	1.82E-05
ENSMMUG00000062704	Metazoan signal recognition particle RNA [Source:RFAM;Acc:RF00017]	-1.830637974	0.003206824	0.012313155
ENSMMUG00000049403		0.56537207	0.001096925	0.004886916
ENSMMUG00000064052	CD300 molecule like family member d [Source:NCBI gene;Acc:703142]	1.645637457	3.62E-09	5.71E-08
ENSMMUG00000058592		1.738238212	3.83E-12	1.02E-10
ENSMMUG00000020462	CD300e molecule [Source:NCBI gene;Acc:114673189]	2.029321404	1.68E-08	2.33E-07
ENSMMUG00000005737	SLC9A3 regulator 1 [Source:VGNC Symbol;Acc:VGNC:77639]	2.476284549	5.14E-05	0.000332521
ENSMMUG00000005738	N-acetyltransferase 9 (putative) [Source:NCBI gene;Acc:699085]	3.320351011	0.013616278	0.041830057
ENSMMUG00000023405	otopetrin 3 [Source:VGNC Symbol;Acc:VGNC:75654]	0.486962246	0.001086735	0.004850434
ENSMMUG00000023406	HID1 domain containing [Source:VGNC Symbol;Acc:VGNC:73394]	0.668298128	9.14E-05	0.00055164
ENSMMUG00000018031	solute carrier family 25 member 19 [Source:VGNC Symbol;Acc:VGNC:77474]	-1.829808758	1.45E-08	2.05E-07
ENSMMUG00000058142		-1.044785042	2.10E-06	1.87E-05
ENSMMUG00000034931	U6 spliceosomal RNA [Source:RFAM;Acc:RF00026]	-2.370120014	0.007964688	0.026709367
ENSMMUG00000022120	transmembrane protein 94 [Source:VGNC Symbol;Acc:VGNC:79760]	-0.968666837	1.01E-08	1.47E-07
ENSMMUG00000022121	CASK interacting protein 2 [Source:VGNC Symbol;Acc:VGNC:70577]	-1.18388603	3.37E-06	2.86E-05
ENSMMUG00000022123	tRNA splicing endonuclease subunit 54 [Source:VGNC Symbol;Acc:VGNC:79666]	-0.964461262	0.000100065	0.000596413
ENSMMUG00000001228	LLGL scribble cell polarity complex component 2 [Source:NCBI gene;Acc:706973]	-0.618510638	9.40E-05	0.00056441
ENSMMUG00000063604	small integral membrane protein 5 [Source:NCBI gene;Acc:703547]	-1.511927274	0.012392371	0.03869987
ENSMMUG00000021636	SAP30 binding protein [Source:VGNC Symbol;Acc:VGNC:77142]	-1.186820592	0.001072483	0.004803043
ENSMMUG00000013646	unk zinc finger [Source:VGNC Symbol;Acc:VGNC:79803]	0.663068719	0.009435699	0.030924165
ENSMMUG00000002822	unc-13 homolog D [Source:VGNC Symbol;Acc:VGNC:79375]	0.870414795	2.62E-05	0.000181165
ENSMMUG00000009381	WW domain binding protein 2 [Source:VGNC Symbol;Acc:VGNC:78786]	0.902557662	1.46E-06	1.36E-05
ENSMMUG00000009377	tripartite motif containing 47 [Source:VGNC Symbol;Acc:VGNC:79171]	1.289171146	3.49E-08	4.57E-07
ENSMMUG00000016774	tripartite motif containing 65 [Source:NCBI gene;Acc:708355]	1.617128232	0.010936557	0.034903344
ENSMMUG00000016775	Fas binding factor 1 [Source:NCBI gene;Acc:708559]	2.959400629	0.011420783	0.036155036
ENSMMUG00000003534	ring finger protein 157 [Source:VGNC Symbol;Acc:VGNC:76860]	0.538519891	0.002211143	0.009001228
ENSMMUG00000003539	glutamine rich 2 [Source:VGNC Symbol;Acc:VGNC:76534]	1.86880467	1.91E-08	2.62E-07
ENSMMUG00000049537	phosphoribosyl pyrophosphate synthetase associated protein 1 [Source:NCBI gene;Acc:706625]	2.116416255	0.001413921	0.006097352
ENSMMUG00000018011	sphingosine kinase 1 [Source:VGNC Symbol;Acc:VGNC:77858]	1.845458206	1.57E-05	0.000114767
ENSMMUG00000018967	ubiquitin conjugating enzyme E2 O [Source:VGNC Symbol;Acc:VGNC:79813]	2.762559296	1.57E-31	3.68E-29
ENSMMUG00000046918		2.371711842	1.19E-06	1.13E-05
ENSMMUG00000054111	Metazoan signal recognition particle RNA [Source:RFAM;Acc:RF00017]	1.75201284	0.002823838	0.011034984
ENSMMUG00000055379		2.549012278	9.43E-29	1.93E-26
ENSMMUG00000053937		2.562900955	3.28E-28	6.12E-26
ENSMMUG00000008237	phosphatidylglycerophosphate synthase 1 [Source:VGNC Symbol;Acc:VGNC:75890]	-0.816671878	0.003662443	0.013807298
ENSMMUG00000008239	dynein axonemal heavy chain 17 [Source:NCBI gene;Acc:715774]	-0.565448353	0.011396787	0.036110527
ENSMMUG00000049898		-0.627132496	0.001821548	0.007606964
ENSMMUG00000051537		2.375259007	0.000300924	0.001578113
ENSMMUG00000013313	cytohesin 1 [Source:VGNC Symbol;Acc:VGNC:71828]	0.356412476	0.006418433	0.022175788
ENSMMUG00000005355	TIMP metallopeptidase inhibitor 2 [Source:NCBI gene;Acc:574357]	1.972282026	0.010675482	0.034190237

ENSMMUG00000023027	endo-beta-N-acetylglucosaminidase [Source:VGNC Symbol;Acc:VGNC:72099]	-0.615641918	7.19E-05	0.000445441
ENSMMUG00000058715		1.81841477	0.000660744	0.003159618
ENSMMUG00000016414	ectonucleotide pyrophosphatase/phosphodiesterase 7 [Source:VGNC Symbol;Acc:VGNC:72090]	1.313598487	0.002969059	0.011525022
ENSMMUG00000046417	chromobox 2 [Source:NCBI gene;Acc:717462]	1.997620235	7.74E-10	1.39E-08
ENSMMUG00000016417	chromobox 8 [Source:NCBI gene;Acc:717514]	1.458379626	2.00E-10	3.98E-09
ENSMMUG00000020840	TBC1 domain family member 16 [Source:VGNC Symbol;Acc:VGNC:78228]	0.598897049	6.33E-05	0.000398275
ENSMMUG00000064603	caspase recruitment domain family member 14 [Source:NCBI gene;Acc:712205]	0.446473575	0.00144879	0.006229242
ENSMMUG00000064563		-0.751140422	0.003783875	0.014184166
ENSMMUG00000000590	NPR3 like, GATOR1 complex subunit [Source:VGNC Symbol;Acc:VGNC:75173]	1.221881335	0.002213888	0.009009878
ENSMMUG00000029310	hemoglobin, alpha 2 [Source:NCBI gene;Acc:693930]	-0.620617305	2.94E-05	0.00020102
ENSMMUG00000019566	hemoglobin subunit alpha 1 [Source:NCBI gene;Acc:701930]	-0.52395998	0.011048681	0.035176231
ENSMMUG00000029296	phosphatidylinositol glycan anchor biosynthesis class Q [Source:VGNC Symbol;Acc:VGNC:75798]	0.993314336	0.000300893	0.001578113
ENSMMUG00000049219	STIP1 homology and U-box containing protein 1 [Source:NCBI gene;Acc:697105]	0.759296401	0.004313337	0.015838086
ENSMMUG00000001986	WD repeat domain 24 [Source:VGNC Symbol;Acc:VGNC:79289]	-0.93462424	0.000774913	0.003622955
ENSMMUG00000050065		-1.021881626	0.001734675	0.007292358
ENSMMUG00000016906	F-box and leucine rich repeat protein 16 [Source:VGNC Symbol;Acc:VGNC:81405]	-0.962854272	2.17E-07	2.45E-06
ENSMMUG00000050892	mastin-like [Source:NCBI gene;Acc:722432]	-0.393386089	0.001971215	0.008161648
ENSMMUG00000063740		-0.493967507	7.39E-05	0.000457679
ENSMMUG00000044277	ubiquitin conjugating enzyme E2 I [Source:NCBI gene;Acc:722434]	-1.147907823	6.53E-07	6.58E-06
ENSMMUG00000021415	BAI1 associated protein 3 [Source:VGNC Symbol;Acc:VGNC:70223]	0.797124178	0.000969581	0.004405376
ENSMMUG00000061546		-1.623742506	0.001163481	0.005147145
ENSMMUG00000008119	telomere maintenance 2 [Source:NCBI gene;Acc:701679]	-0.803476635	0.004453702	0.01625087
ENSMMUG00000062198	transmembrane protein 204 [Source:NCBI gene;Acc:701926]	-1.552781401	0.000411299	0.002084019
ENSMMUG00000000978	cramped chromatin regulator homolog 1 [Source:NCBI gene;Acc:722473]	-0.623244803	6.46E-05	0.000405707
ENSMMUG00000029265	zinc finger protein 598 [Source:NCBI gene;Acc:694129]	-0.913235074	3.08E-05	0.000210199
ENSMMUG00000029263	polycystin 1, transient receptor potential channel interacting [Source:NCBI gene;Acc:694905]	-0.787200019	1.38E-05	0.000102229
ENSMMUG00000019223	TNF receptor associated factor 7 [Source:VGNC Symbol;Acc:VGNC:79252]	0.825538735	0.000486264	0.002412567
ENSMMUG00000019237	enoyl-CoA delta isomerase 1 [Source:VGNC Symbol;Acc:VGNC:81391]	-0.458939598	0.013022356	0.04028644
ENSMMUG00000019238	RNA binding protein with serine rich domain 1 [Source:NCBI gene;Acc:695816]	-0.564775908	9.45E-08	1.14E-06
ENSMMUG00000000001	cyclin F [Source:VGNC Symbol;Acc:VGNC:70729]	-0.723536202	0.00151907	0.006493008
ENSMMUG00000019095	TBC1 domain family member 24 [Source:NCBI gene;Acc:697106]	0.644554242	0.00211755	0.008678553
ENSMMUG00000011459	3-phosphoinositide dependent protein kinase 1 [Source:NCBI gene;Acc:697523]	0.426745067	1.33E-07	1.57E-06
ENSMMUG00000009487	zinc finger protein 205 [Source:VGNC Symbol;Acc:VGNC:78869]	0.370686507	0.01439858	0.043807484
ENSMMUG00000020970	zinc finger protein 213 [Source:VGNC Symbol;Acc:VGNC:78872]	0.424986043	0.000156266	0.000888748
ENSMMUG00000051323	caspase-14-like [Source:NCBI gene;Acc:702430]	0.456458424	0.010856609	0.034670382
ENSMMUG00000064961		-1.022251249	0.001242136	0.00545362

ENSMMUG00000017972	zinc finger protein 200 [Source:VGNC Symbol;Acc:VGNC:78867]	-0.760189164	4.45E-05	0.000293487
ENSMMUG00000017969	MEFV innate immuity regulator, pyrin [Source:VGNC Symbol;Acc:VGNC:74700]	-0.65182614	0.006152096	0.021390341
ENSMMUG00000040952	tigger transposable element derived 7 [Source:NCBI gene;Acc:100428553]	-1.259255068	0.000615838	0.002966741
ENSMMUG00000007568	zinc finger protein 174 [Source:VGNC Symbol;Acc:VGNC:78855]	-1.122193484	0.001131912	0.005027378
ENSMMUG00000018711	N-alpha-acetyltransferase 60, NatF catalytic subunit [Source:NCBI gene;Acc:703207]	-0.317286768	0.014266747	0.043488299
ENSMMUG00000007569	clusterin associated protein 1 [Source:VGNC Symbol;Acc:VGNC:71211]	-0.425875941	0.013211631	0.040776534
ENSMMUG00000021866	deoxyribonuclease 1 [Source:VGNC Symbol;Acc:VGNC:71946]	0.47516818	0.000337732	0.001747193
ENSMMUG00000021868	TNF receptor associated protein 1 [Source:VGNC Symbol;Acc:VGNC:79019]	0.811188184	8.35E-07	8.16E-06
ENSMMUG00000039943	adenylate cyclase 9 [Source:NCBI gene;Acc:706789]	1.492589263	0.002125777	0.008709813
ENSMMUG00000061940		3.81724337	4.87E-05	0.000317034
ENSMMUG00000051196		-1.678931127	0.00260072	0.010270761
ENSMMUG00000040803	sarcalumenin [Source:NCBI gene;Acc:704655]	-1.676000956	3.29E-06	2.80E-05
ENSMMUG00000044729	transcription factor AP-4 [Source:NCBI gene;Acc:707513]	-1.483560212	8.19E-05	0.000501036
ENSMMUG00000044344	vasorin [Source:NCBI gene;Acc:100426861]	-0.700652945	0.000565016	0.002749793
ENSMMUG00000020540	NmrA like redox sensor 1 [Source:VGNC Symbol;Acc:VGNC:75252]	-1.586301163	7.73E-08	9.45E-07
ENSMMUG00000001888	mahogunin ring finger 1 [Source:VGNC Symbol;Acc:VGNC:74542]	-0.836129934	0.003513981	0.013313199
ENSMMUG00000011001	ankyrin repeat and sterile alpha motif domain containing 3 [Source:VGNC Symbol;Acc:VGNC:69735]	0.537100259	0.000602899	0.002914648
ENSMMUG00000014311	chromosome 20 C16orf71 homolog [Source:NCBI gene;Acc:709711]	0.39302741	0.007191334	0.02450549
ENSMMUG00000014313	zinc finger protein 500 [Source:VGNC Symbol;Acc:VGNC:79406]	0.540212797	0.000898029	0.00412991
ENSMMUG00000017945	septin 12 [Source:VGNC Symbol;Acc:VGNC:82235]	0.659555005	1.40E-05	0.000103634
ENSMMUG00000017946	rogdi atypical leucine zipper [Source:VGNC Symbol;Acc:VGNC:76862]	0.552435808	0.000159922	0.000906349
ENSMMUG00000009567	glyoxylate reductase 1 homolog [Source:NCBI gene;Acc:706933]	2.493022883	3.56E-33	1.01E-30
ENSMMUG00000017822	periplakin [Source:VGNC Symbol;Acc:VGNC:76240]	3.69130989	1.54E-26	2.38E-24
ENSMMUG00000011499	N-acetylglucosamine-1-phosphodiester alpha-N-acetylglucosaminidase [Source:VGNC Symbol;Acc:VGNC:74978]	-0.967073126	0.01274396	0.039551442
ENSMMUG00000046237	4-aminobutyrate aminotransferase [Source:NCBI gene;Acc:714017]	-0.861314035	0.000318938	0.001662386
ENSMMUG00000001944	chromosome 20 C16orf72 homolog [Source:VGNC Symbol;Acc:VGNC:70443]	0.873605585	7.24E-10	1.30E-08
ENSMMUG00000005913	activating transcription factor 7 interacting protein 2 [Source:VGNC Symbol;Acc:VGNC:70103]	-1.113984484	0.001046518	0.004704151
ENSMMUG00000056165		-2.295793638	2.45E-08	3.30E-07
ENSMMUG00000052031		-1.298369223	1.25E-06	1.18E-05
ENSMMUG00000064158		-0.997523661	0.01347789	0.041472614
ENSMMUG00000060713	epithelial membrane protein 2 [Source:NCBI gene;Acc:710315]	-2.253565974	9.15E-09	1.35E-07
ENSMMUG00000006515	C-type lectin domain containing 16A [Source:VGNC Symbol;Acc:VGNC:71369]	-0.608811611	0.002435534	0.009740125
ENSMMUG00000000566		2.315028692	2.14E-06	1.90E-05
ENSMMUG00000008784	lipopolysaccharide induced TNF factor [Source:NCBI gene;Acc:711743]	4.118325795	1.37E-05	0.000101616
ENSMMUG00000056339		2.218225214	0.010913643	0.034837862
ENSMMUG00000062037	stannin [Source:NCBI gene;Acc:711909]	1.924696087	3.43E-07	3.71E-06

ENSMMUG00000014240	thioredoxin domain containing 11 [Source:VGNC Symbol;Acc:VGNC:79678]	2.458728661	1.26E-07	1.49E-06
ENSMMUG00000019632	ribosomal L1 domain containing 1 [Source:VGNC Symbol;Acc:VGNC:77096]	0.503182187	2.33E-05	0.000163368
ENSMMUG00000057345	calcineurin like phosphoesterase domain containing 1 [Source:NCBI gene;Acc:716978]	1.265482561	2.52E-11	5.81E-10
ENSMMUG00000017379	myocardin related transcription factor B [Source:VGNC Symbol;Acc:VGNC:74865]	-0.858256539	2.68E-05	0.000185234
ENSMMUG00000062213	Metazoan signal recognition particle RNA [Source:RFAM;Acc:RF00017]	-0.823902218	0.011651978	0.036713278
ENSMMUG00000057747		-1.421231607	0.00330346	0.012650791
ENSMMUG00000064131		-1.280082624	2.23E-07	2.50E-06
ENSMMUG00000027108	mml-mir-193b [Source:miRBase;Acc:MI0007656]	-1.227927596	0.013684935	0.041996602
ENSMMUG00000064246		0.669835201	0.003964036	0.014737902
ENSMMUG00000002989	N-terminal asparagine amidase [Source:NCBI gene;Acc:100427569]	-1.111085514	0.013671804	0.041982894
ENSMMUG00000062791		-2.056382457	7.17E-07	7.15E-06
ENSMMUG00000062623	Metazoan signal recognition particle RNA [Source:RFAM;Acc:RF00017]	-0.927499249	5.73E-05	0.000364995
ENSMMUG00000058016	bMERB domain containing 1 [Source:NCBI gene;Acc:713757]	-1.149297446	3.26E-06	2.78E-05
ENSMMUG00000010367	myosin heavy chain 11 [Source:VGNC Symbol;Acc:VGNC:75097]	0.521928649	2.36E-06	2.08E-05
ENSMMUG00000002709	G protein-coupled receptor class C group 5 member B [Source:VGNC Symbol;Acc:VGNC:72934]	-0.605167192	7.78E-05	0.000479232
ENSMMUG00000055853	G protein-coupled receptor 139 [Source:NCBI gene;Acc:695541]	-1.028441316	0.011380613	0.03606714
ENSMMUG00000016758	transmembrane protein 159 [Source:VGNC Symbol;Acc:VGNC:79836]	0.637942395	6.97E-05	0.000433085
ENSMMUG00000064659		0.6131281	0.000897579	0.004129146
ENSMMUG00000017725	eukaryotic elongation factor 2 kinase [Source:VGNC Symbol;Acc:VGNC:72098]	-0.690553149	8.03E-08	9.79E-07
ENSMMUG00000002257	RNA polymerase III subunit E [Source:VGNC Symbol;Acc:VGNC:76142]	-0.551929428	2.92E-05	0.000199908
ENSMMUG00000013877	otoancorin [Source:VGNC Symbol;Acc:VGNC:75624]	1.186635177	9.15E-05	0.000551944
ENSMMUG00000058042	heparan sulfate-glucosamine 3-sulfotransferase 2 [Source:NCBI gene;Acc:699722]	1.015174045	4.61E-07	4.85E-06
ENSMMUG00000000525	component of oligomeric golgi complex 7 [Source:VGNC Symbol;Acc:VGNC:71208]	0.890119057	0.00231472	0.009336596
ENSMMUG00000054064		-1.597313985	1.77E-13	5.74E-12
ENSMMUG00000013560	calcium voltage-gated channel auxiliary subunit gamma 3 [Source:VGNC Symbol;Acc:VGNC:70513]	0.721619963	0.000265337	0.001420177
ENSMMUG00000057850		0.474314437	0.016948778	0.049969579
ENSMMUG00000015908	RB binding protein 6, ubiquitin ligase [Source:VGNC Symbol;Acc:VGNC:76677]	0.455881978	0.000386776	0.001967994
ENSMMUG00000005942	leucine carboxyl methyltransferase 1 [Source:VGNC Symbol;Acc:VGNC:74067]	-0.439119157	0.000287637	0.001522159
ENSMMUG00000008254	general transcription factor IIIC subunit 1 [Source:VGNC Symbol;Acc:VGNC:73316]	-3.836563063	0.003074693	0.011872646
ENSMMUG00000015572	GSG1 like [Source:VGNC Symbol;Acc:VGNC:82152]	0.797742128	9.90E-05	0.000591282
ENSMMUG00000003303	Tu translation elongation factor, mitochondrial [Source:VGNC Symbol;Acc:VGNC:78674]	1.402896496	2.10E-18	1.32E-16
ENSMMUG00000003298	ataxin 2 like [Source:VGNC Symbol;Acc:VGNC:70159]	1.373956454	2.82E-14	1.08E-12
ENSMMUG00000011798	eukaryotic translation initiation factor 3 subunit C [Source:NCBI gene;Acc:705696]	1.388117996	6.77E-16	3.25E-14
ENSMMUG00000000700	CLN3 lysosomal/endosomal transmembrane protein, battenin [Source:NCBI gene;Acc:705815]	1.40157735	6.53E-12	1.68E-10
ENSMMUG00000022330	sulfotransferase family, cytosolic, 1A, phenol-preferring, member 3 [Source:NCBI gene;Acc:100428398]	-2.321726207	0.00132658	0.005775528
ENSMMUG00000017758	mitogen-activated protein kinase 3 [Source:VGNC Symbol;Acc:VGNC:74646]	2.245002475	5.51E-29	1.16E-26
ENSMMUG00000029164	glycerophosphodiester phosphodiesterase domain containing 3 [Source:VGNC Symbol;Acc:VGNC:72800]	-3.327639655	2.12E-11	4.99E-10

ENSMMUG00000000981	T-box transcription factor 6 [Source:VGNC Symbol;Acc:VGNC:78276]	-1.819722995	4.06E-09	6.31E-08
ENSMMUG00000000982	protein phosphatase 4 catalytic subunit [Source:VGNC Symbol;Acc:VGNC:76384]	-1.762335642	7.13E-08	8.80E-07
ENSMMUG00000000983	aldolase, fructose-bisphosphate A [Source:NCBI gene;Acc:708406]	-1.467984508	4.36E-07	4.60E-06
ENSMMUG00000059051		-1.198024507	0.009299414	0.03054351
ENSMMUG00000037707	TLC domain containing 3B [Source:NCBI gene;Acc:708210]	-0.79488597	0.015460797	0.046427102
ENSMMUG00000020277	double C2 domain alpha [Source:VGNC Symbol;Acc:VGNC:71963]	-0.478919838	0.000533945	0.002616095
ENSMMUG00000020274	TAO kinase 2 [Source:VGNC Symbol;Acc:VGNC:81592]	-0.421793192	0.001050693	0.004720001
ENSMMUG00000018992	aspartate beta-hydroxylase domain containing 1 [Source:VGNC Symbol;Acc:VGNC:70138]	1.812067192	0.000267217	0.001428139
ENSMMUG00000018986	seizure related 6 homolog like 2 [Source:VGNC Symbol;Acc:VGNC:81567]	1.176192111	1.87E-06	1.68E-05
ENSMMUG00000061749	PAXIP1 associated glutamate rich protein 1 [Source:NCBI gene;Acc:706839]	0.451172124	0.000338648	0.001750685
ENSMMUG00000019826	MYC associated zinc finger protein [Source:NCBI gene;Acc:708982]	0.537269757	0.005798428	0.020385122
ENSMMUG00000009684	kinesin family member 22 [Source:VGNC Symbol;Acc:VGNC:74033]	1.644821131	1.94E-12	5.41E-11
ENSMMUG00000016143	sialophorin [Source:VGNC Symbol;Acc:VGNC:77861]	1.590784073	6.57E-28	1.18E-25
ENSMMUG00000021845	TBC1 domain family member 10B [Source:VGNC Symbol;Acc:VGNC:78202]	-0.363627721	0.004559421	0.016565935
ENSMMUG00000016876	integrin subunit alpha L [Source:NCBI gene;Acc:711945]	0.909451422	3.25E-05	0.000220663
ENSMMUG00000001121	zinc finger protein 689 [Source:VGNC Symbol;Acc:VGNC:79480]	-1.205538521	4.35E-05	0.000287759
ENSMMUG00000005625	fibrosin [Source:NCBI gene;Acc:712741]	1.842144187	0.002268411	0.009195729
ENSMMUG00000051226	FAD-linked sulfhydryl oxidase ERV1 [Source:NCBI gene;Acc:106994988]	1.396264795	6.97E-15	2.86E-13
ENSMMUG00000010556	Snf2 related CREBBP activator protein [Source:NCBI gene;Acc:712944]	0.986016473	1.35E-10	2.77E-09
ENSMMUG00000003230	F-box and leucine rich repeat protein 19 [Source:VGNC Symbol;Acc:VGNC:81406]	-0.653087427	0.000931919	0.004265525
ENSMMUG00000003233	SET domain containing 1A, histone lysine methyltransferase [Source:NCBI gene;Acc:712416]	0.909988539	2.58E-09	4.18E-08
ENSMMUG00000048207	syntaxin 4 [Source:NCBI gene;Acc:704880]	0.568507467	1.51E-05	0.000110531
ENSMMUG00000002033	serine protease 36 [Source:VGNC Symbol;Acc:VGNC:76325]	0.627154709	4.94E-07	5.14E-06
ENSMMUG00000014349	integrin subunit alpha X [Source:NCBI gene;Acc:100427850]	2.072634456	3.34E-08	4.39E-07
ENSMMUG00000000901	alpha hemoglobin stabilizing protein [Source:VGNC Symbol;Acc:VGNC:69631]	0.343760562	0.006511722	0.022442129
ENSMMUG00000058907		1.890053574	8.77E-05	0.000531156
ENSMMUG00000055973	phosphoglycerate mutase 1-like [Source:NCBI gene;Acc:714548]	2.248958856	0.000132504	0.000769255
ENSMMUG00000006133		2.601948734	0.006464633	0.022306282
ENSMMUG00000008253	zinc finger protein 267 [Source:NCBI gene;Acc:641441]	1.666095305	8.82E-17	4.64E-15
ENSMMUG00000003288	glutamic--pyruvic transaminase 2 [Source:VGNC Symbol;Acc:VGNC:72962]	-0.874171415	0.000167162	0.000941511
ENSMMUG00000051092		0.846601983	0.010607615	0.033987855
ENSMMUG00000019868	NKD inhibitor of WNT signaling pathway 1 [Source:VGNC Symbol;Acc:VGNC:75198]	-1.258625387	0.001402085	0.006054727
ENSMMUG00000012209	CYLD lysine 63 deubiquitinase [Source:VGNC Symbol;Acc:VGNC:71581]	-0.943690303	2.24E-06	1.98E-05
ENSMMUG00000055889		-1.307759806	0.000966021	0.004393944
ENSMMUG00000064095		3.609529037	1.22E-06	1.16E-05
ENSMMUG00000013967	TOX high mobility group box family member 3 [Source:VGNC Symbol;Acc:VGNC:78612]	2.758757539	3.99E-14	1.50E-12
ENSMMUG00000020580	RPGRIP1 like [Source:VGNC Symbol;Acc:VGNC:77124]	1.320868307	1.46E-05	0.000107456
ENSMMUG00000020583	FTO alpha-ketoglutarate dependent dioxygenase [Source:VGNC Symbol;Acc:VGNC:72772]	0.902697837	8.93E-08	1.08E-06
ENSMMUG00000001389	iroquois homeobox 3 [Source:NCBI gene;Acc:704386]	0.40922102	0.011472231	0.036278404
ENSMMUG00000054811		3.037786445	9.42E-05	0.000565319
ENSMMUG00000056555		1.364826914	3.01E-11	6.88E-10

ENSMMUG00000023525	cadherin 5 [Source:VGNC Symbol;Acc:VGNC:70973]	-0.50959161	0.000332072	0.001720976
ENSMMUG00000049484		-0.865486424	7.39E-10	1.33E-08
ENSMMUG00000020847	CKLF like MARVEL transmembrane domain containing 3 [Source:VGNC Symbol;Acc:VGNC:71215]	-0.982656282	0.002330978	0.009394351
ENSMMUG00000022825	carboxylesterase 2 [Source:VGNC Symbol;Acc:VGNC:71012]	0.326548718	0.000116779	0.000685631
ENSMMUG00000064237		-1.346782341	2.21E-07	2.49E-06
ENSMMUG00000054948		1.259367266	0.000472475	0.002354599
ENSMMUG00000058815	core-binding factor subunit beta [Source:NCBI gene;Acc:695767]	0.922233773	0.000126053	0.000734146
ENSMMUG00000018102	chromosome 20 C16orf70 homolog [Source:VGNC Symbol;Acc:VGNC:70438]	1.148110564	3.75E-05	0.000250841
ENSMMUG00000005906	heat shock transcription factor 4 [Source:VGNC Symbol;Acc:VGNC:73533]	0.868594233	2.57E-05	0.00017862
ENSMMUG00000020268	nucleolar protein 3 [Source:NCBI gene;Acc:696747]	0.780789795	0.014654693	0.044447168
ENSMMUG00000020266	KIAA0895 like [Source:VGNC Symbol;Acc:VGNC:73952]	1.278378343	0.004881907	0.017588172
ENSMMUG00000020254	exocyst complex component 3 like 1 [Source:VGNC Symbol;Acc:VGNC:72376]	1.092183519	9.87E-11	2.07E-09
ENSMMUG00000020258	E2F transcription factor 4 [Source:VGNC Symbol;Acc:VGNC:72025]	0.788005532	7.36E-06	5.80E-05
ENSMMUG00000020259	engulfment and cell motility 3 [Source:VGNC Symbol;Acc:VGNC:72060]	1.123599026	0.006105638	0.021274606
ENSMMUG00000054637	tubulin polymerization promoting protein family member 3 [Source:NCBI gene;Acc:100429881]	1.53881209	0.016713013	0.049512343
ENSMMUG00000009825	zinc finger DHHC-type containing 1 [Source:VGNC Symbol;Acc:VGNC:79102]	0.715058631	2.02E-05	0.000143654
ENSMMUG00000017105	RHO family interacting cell polarization regulator 1 [Source:NCBI gene;Acc:699910]	-0.386462477	0.000410528	0.002080838
ENSMMUG00000007804	RAN binding protein 10 [Source:VGNC Symbol;Acc:VGNC:76563]	0.87475	0.003246158	0.012451027
ENSMMUG00000010393	cadherin 3 [Source:VGNC Symbol;Acc:VGNC:70971]	0.363265982	0.002970412	0.011527198
ENSMMUG00000012039	cytochrome b5 type B (outer mitochondrial membrane) [Source:NCBI gene;Acc:705093]	2.248692584	8.34E-06	6.46E-05
ENSMMUG00000018255	nuclear factor of activated T cells 5 [Source:VGNC Symbol;Acc:VGNC:75325]	0.707091069	1.22E-05	9.19E-05
ENSMMUG00000049699	thioredoxin like 4B [Source:NCBI gene;Acc:706840]	-1.061181014	0.005908404	0.02071147
ENSMMUG00000023312	haptoglobin [Source:NCBI gene;Acc:706640]	-1.215988346	0.000585766	0.002838429
ENSMMUG00000009688	IST1 factor associated with ESCRT-III [Source:VGNC Symbol;Acc:VGNC:73675]	-0.695001975	0.001441664	0.006200436
ENSMMUG00000010878	adaptor related protein complex 1 subunit gamma 1 [Source:VGNC Symbol;Acc:VGNC:69958]	-0.912082301	2.67E-11	6.15E-10
ENSMMUG00000012007	PH domain and leucine rich repeat protein phosphatase 2 [Source:VGNC Symbol;Acc:VGNC:81494]	0.666003342	0.01347933	0.041472614
ENSMMUG00000052324	MARVEL domain containing 3 [Source:NCBI gene;Acc:710448]	0.680959409	0.00582425	0.020460017
ENSMMUG00000061967	carbohydrate sulfotransferase 4 [Source:NCBI gene;Acc:708116]	-0.823068567	0.002533814	0.010077716
ENSMMUG00000023147	pyruvate dehydrogenase phosphatase regulatory subunit [Source:VGNC Symbol;Acc:VGNC:75897]	1.238319171	0.011240723	0.035678251
ENSMMUG00000005751	adenosine deaminase tRNA specific 1 [Source:VGNC Symbol;Acc:VGNC:69504]	-1.392240689	0.001715104	0.007233653
ENSMMUG00000005754	lysyl-tRNA synthetase 1 [Source:NCBI gene;Acc:714516]	-1.039226587	0.002995526	0.011614423
ENSMMUG00000045592		-1.699148589	4.66E-05	0.000305377
ENSMMUG00000003916	contactin associated protein family member 4 [Source:VGNC Symbol;Acc:VGNC:71209]	-1.473746239	0.001572347	0.006697103
ENSMMUG00000010610	WW domain containing oxidoreductase [Source:VGNC Symbol;Acc:VGNC:78805]	0.903241178	0.015062505	0.045428141
ENSMMUG00000053231		-0.681088491	0.00060959	0.0029421
ENSMMUG00000005334	phospholipase C gamma 2 [Source:VGNC Symbol;Acc:VGNC:76170]	-0.681269152	1.58E-05	0.000115009
ENSMMUG00000062047		-0.88932336	0.003544687	0.013405079
ENSMMUG00000057329		1.279740988	0.009826863	0.031972495

ENSMMUG00000011893	KIAA0513 [Source:VGNC Symbol;Acc:VGNC:73925]	-1.111560774	3.19E-07	3.47E-06
ENSMMUG00000011894	CBY1 interacting BAR domain containing 2 [Source:VGNC Symbol;Acc:VGNC:72335]	-1.044782924	9.47E-05	0.000567669
ENSMMUG00000061995		0.938403015	3.43E-11	7.76E-10
ENSMMUG00000006763	cytochrome c oxidase subunit IV isoform 1 [Source:NCBI gene;Acc:693811]	0.636239057	0.000854998	0.003959575
ENSMMUG00000060536		-1.677869366	0.00056454	0.002748397
ENSMMUG00000054493		-0.669032409	2.06E-05	0.000146094
ENSMMUG00000017615	zinc finger CCHC-type containing 14 [Source:VGNC Symbol;Acc:VGNC:79420]	-1.186192668	1.32E-10	2.72E-09
ENSMMUG00000022867	kelch domain containing 4 [Source:VGNC Symbol;Acc:VGNC:74122]	0.544295352	0.000312401	0.001630654
ENSMMUG00000058437		0.938893456	3.43E-06	2.91E-05
ENSMMUG00000008945	zinc finger protein 407 [Source:NCBI gene;Acc:695303]	-2.308434595	0.001438732	0.006189656
ENSMMUG00000008942	carnosine dipeptidase 1 [Source:VGNC Symbol;Acc:VGNC:71257]	-2.686067317	3.48E-06	2.95E-05
ENSMMUG00000051690		-2.602182607	0.010741815	0.03438753
ENSMMUG00000015702	divergent protein kinase domain 1C [Source:NCBI gene;Acc:699537]	-3.404180302	0.000162088	0.000917193
ENSMMUG00000020905	chromosome 18 C18orf63 homolog [Source:VGNC Symbol;Acc:VGNC:70465]	-3.091722348	5.66E-05	0.000361356
ENSMMUG00000023298	F-box protein 15 [Source:VGNC Symbol;Acc:VGNC:72504]	-1.509621861	0.010487001	0.033675619
ENSMMUG00000056477		0.945965394	4.02E-14	1.50E-12
ENSMMUG00000050516		0.961041465	2.41E-12	6.62E-11
ENSMMUG00000060966		-1.54034567	0.00745388	0.025334827
ENSMMUG00000018275	docking protein 6 [Source:VGNC Symbol;Acc:VGNC:71915]	0.726551726	0.009156501	0.030162714
ENSMMUG00000001245	zinc finger protein 532 [Source:NCBI gene;Acc:701146]	-4.689024266	2.22E-07	2.50E-06
ENSMMUG00000061572	Metazoan signal recognition particle RNA [Source:RFAM;Acc:RF00017]	-4.419907428	2.77E-08	3.69E-07
ENSMMUG00000065131		-3.77952974	0.000109866	0.000648181
ENSMMUG00000012001	transcription factor 4 [Source:VGNC Symbol;Acc:VGNC:78288]	1.693508596	9.98E-15	3.95E-13
ENSMMUG00000020898	cap binding complex dependent translation initiation factor [Source:HGNC Symbol;Acc:HGNC:23925]	-0.94874816	0.009561768	0.031242845
ENSMMUG00000007816	SMAD family member 2 [Source:VGNC Symbol;Acc:VGNC:77810]	-2.523229574	9.37E-13	2.73E-11
ENSMMUG00000053364		0.593233008	0.009501294	0.031080177
ENSMMUG00000015952	katanin catalytic subunit A1 like 2 [Source:VGNC Symbol;Acc:VGNC:73904]	1.891667613	2.49E-05	0.000173749
ENSMMUG00000043738		2.037938867	0.000162436	0.000917733
ENSMMUG00000004498	protein inhibitor of activated STAT 2 [Source:VGNC Symbol;Acc:VGNC:75815]	1.728500494	8.90E-06	6.86E-05
ENSMMUG00000002742	ST8 alpha-N-acetyl-neuraminide alpha-2,8-sialyltransferase 5 [Source:VGNC Symbol;Acc:VGNC:77942]	-2.300057044	2.55E-14	9.81E-13
ENSMMUG00000017246	lipoxygenase homology domains 1 [Source:VGNC Symbol;Acc:VGNC:74368]	-1.677556828	1.58E-15	7.23E-14
ENSMMUG00000004997	SET binding protein 1 [Source:NCBI gene;Acc:699652]	1.10840192	0.015531522	0.046600979
ENSMMUG00000049194		-1.584847381	0.003887421	0.01449383
ENSMMUG00000006866	CUGBP Elav-like family member 4 [Source:VGNC Symbol;Acc:VGNC:70945]	-2.492430974	0.003327847	0.012727416
ENSMMUG00000006864	KIAA1328 ortholog [Source:NCBI gene;Acc:709850]	-3.187525326	0.000721483	0.003406365

ENSMMUG00000018784	solute carrier family 39 member 6 [Source:VGNC Symbol;Acc:VGNC:77508]	-0.365037707	0.002673784	0.010502244
ENSMMUG00000018785	elongator acetyltransferase complex subunit 2 [Source:VGNC Symbol;Acc:VGNC:72069]	-0.35230932	0.002172501	0.008871248
ENSMMUG00000003302	dystrobrevin alpha [Source:VGNC Symbol;Acc:VGNC:72007]	-1.601763282	1.21E-05	9.13E-05
ENSMMUG00000017276	nucleolar protein 4 [Source:VGNC Symbol;Acc:VGNC:75366]	-1.04106704	2.78E-06	2.40E-05
ENSMMUG00000014451	ASXL transcriptional regulator 3 [Source:VGNC Symbol;Acc:VGNC:70152]	-0.660908571	0.000105909	0.000627893
ENSMMUG00000052277	transthyretin [Source:NCBI gene;Acc:705943]	-2.591213127	8.42E-08	1.02E-06
ENSMMUG00000021095	desmoglein 2 [Source:VGNC Symbol;Acc:VGNC:72002]	-2.521223705	0.002171525	0.008871248
ENSMMUG00000063743		0.747312312	0.011465479	0.036264941
ENSMMUG00000010944	GATA binding protein 6 [Source:VGNC Symbol;Acc:VGNC:72891]	-0.674528825	0.01150553	0.036351238
ENSMMUG00000064806		-0.87754019	0.001266341	0.005549799
ENSMMUG00000003974	GREB1 like retinoic acid receptor coactivator [Source:NCBI gene;Acc:700655]	-2.68122451	0.005377976	0.019132713
ENSMMUG00000019892	myomesin 1 [Source:VGNC Symbol;Acc:VGNC:75123]	-1.259593467	5.33E-16	2.60E-14
ENSMMUG00000052605	Metazoan signal recognition particle RNA [Source:RFAM;Acc:RF00017]	-3.293959597	7.72E-06	6.04E-05
ENSMMUG00000005086	homeobox protein TGIF1 [Source:NCBI gene;Acc:694580]	1.534884172	2.60E-08	3.48E-07
ENSMMUG00000019418	erythrocyte membrane protein band 4.1 like 3 [Source:VGNC Symbol;Acc:VGNC:72233]	1.870429611	1.58E-34	5.73E-32
ENSMMUG00000016302	L3MBTL histone methyl-lysine binding protein 4 [Source:VGNC Symbol;Acc:VGNC:74133]	0.340129013	0.003995275	0.014835074
ENSMMUG00000059721	Metazoan signal recognition particle RNA [Source:RFAM;Acc:RF00017]	0.969807949	0.009113822	0.030060584
ENSMMUG00000000768	protein tyrosine phosphatase receptor type M [Source:VGNC Symbol;Acc:VGNC:76619]	0.432402064	0.002806467	0.010978905
ENSMMUG00000053008		-1.305859723	1.88E-06	1.69E-05
ENSMMUG00000049556	NADH:ubiquinone oxidoreductase core subunit V2 [Source:NCBI gene;Acc:706067]	0.679250398	0.009588444	0.031301867
ENSMMUG00000049523	ankyrin repeat domain 12 [Source:NCBI gene;Acc:114673693]	-0.938812869	0.000813803	0.003788105
ENSMMUG00000023717	MIER family member 2 [Source:VGNC Symbol;Acc:VGNC:74855]	1.138230434	9.12E-09	1.35E-07
ENSMMUG00000059175		3.232664425	6.68E-19	4.56E-17
ENSMMUG00000053083	C2 calcium dependent domain containing 4C [Source:NCBI gene;Acc:100424440]	3.06752983	5.04E-10	9.39E-09
ENSMMUG00000012768	hyperpolarization activated cyclic nucleotide gated potassium and sodium channel 2 [Source:NCBI gene;Acc:100425114]	-0.762228802	0.010061175	0.032588969
ENSMMUG00000049968	RNA polymerase mitochondrial [Source:NCBI gene;Acc:100426183]	-0.876282383	5.62E-16	2.71E-14
ENSMMUG00000055423	fibroblast growth factor 22 [Source:NCBI gene;Acc:702727]	-0.379748361	0.014735401	0.044645377
ENSMMUG00000008672	ring finger protein 126 [Source:VGNC Symbol;Acc:VGNC:76791]	-0.645557984	0.003390318	0.012925533
ENSMMUG00000016450	polypyrimidine tract binding protein 1 [Source:VGNC Symbol;Acc:VGNC:76462]	-0.405982735	0.008338242	0.027788405
ENSMMUG00000028948	azurocidin 1 [Source:VGNC Symbol;Acc:VGNC:70202]	2.368837253	4.04E-06	3.38E-05
ENSMMUG00000009674	elastase, neutrophil expressed [Source:VGNC Symbol;Acc:VGNC:72199]	1.96661735	0.001048272	0.004710583
ENSMMUG00000028947	complement factor D [Source:VGNC Symbol;Acc:VGNC:71115]	2.640040881	2.47E-32	6.52E-30
ENSMMUG00000058621		0.892574639	6.99E-09	1.05E-07
ENSMMUG00000002638	WD repeat domain 18 [Source:VGNC Symbol;Acc:VGNC:79103]	-0.982584203	1.19E-05	9.00E-05
ENSMMUG00000013577	transmembrane protein 259 [Source:NCBI gene;Acc:721161]	-0.362068956	0.008382194	0.027909289
ENSMMUG00000015306	calponin 2 [Source:NCBI gene;Acc:721164]	0.60668624	8.91E-05	0.000539508
ENSMMUG00000015303	ATP binding cassette subfamily A member 7 [Source:VGNC Symbol;Acc:VGNC:69567]	1.032830944	2.92E-09	4.68E-08

ENSMMUG00000058856	Metazoan signal recognition particle RNA [Source:RFAM;Acc:RF00017]	1.088016215	0.000147897	0.00084678
ENSMMUG00000010886	strawberry notch homolog 2 [Source:VGNC Symbol;Acc:VGNC:77123]	1.406124488	5.52E-05	0.000353947
ENSMMUG00000058928	Metazoan signal recognition particle RNA [Source:RFAM;Acc:RF00017]	1.68665333	2.48E-09	4.04E-08
ENSMMUG00000022100	midnolin [Source:VGNC Symbol;Acc:VGNC:82186]	1.258158939	1.03E-16	5.41E-15
ENSMMUG00000003047	DAZ associated protein 1 [Source:VGNC Symbol;Acc:VGNC:71738]	0.278234832	0.013215466	0.040779712
ENSMMUG00000022104	family with sequence similarity 174 member C [Source:NCBI gene;Acc:706393]	0.735022364	2.09E-07	2.36E-06
ENSMMUG00000003043	PWWP domain containing 3A, DNA repair factor [Source:VGNC Symbol;Acc:VGNC:76565]	-0.834173071	3.11E-09	4.96E-08
ENSMMUG00000009014	methyl-CpG binding domain protein 3 [Source:VGNC Symbol;Acc:VGNC:81470]	-0.722258038	0.011206128	0.03559176
ENSMMUG00000009017	transcription factor 3 [Source:VGNC Symbol;Acc:VGNC:78287]	0.543219532	1.66E-06	1.52E-05
ENSMMUG00000013747	secretory carrier membrane protein 4 [Source:VGNC Symbol;Acc:VGNC:77135]	-0.428777128	0.014896911	0.045012761
ENSMMUG00000006620	casein kinase 1 gamma 2 [Source:NCBI gene;Acc:709484]	0.541221543	0.003308105	0.01266524
ENSMMUG00000059725		1.181824861	0.000955261	0.004358202
ENSMMUG00000017949	ornithine decarboxylase antizyme 1 [Source:NCBI gene;Acc:721477]	0.619424633	1.42E-08	2.02E-07
ENSMMUG00000064612	Metazoan signal recognition particle RNA [Source:RFAM;Acc:RF00017]	0.757921737	0.004972554	0.017888149
ENSMMUG00000000452	LSM7 homolog, U6 small nuclear RNA and mRNA degradation associated [Source:NCBI gene;Acc:721486]	1.985796669	0.00016977	0.00095509
ENSMMUG00000055417		-0.770346715	5.37E-09	8.25E-08
ENSMMUG00000065054	G protein subunit gamma 7 [Source:NCBI gene;Acc:711335]	1.594519115	2.10E-11	4.96E-10
ENSMMUG00000007268	thimet oligopeptidase 1 [Source:VGNC Symbol;Acc:VGNC:78347]	0.479913722	0.004119879	0.015200682
ENSMMUG00000007485	zinc finger protein 555 [Source:VGNC Symbol;Acc:VGNC:79705]	-1.02463731	7.86E-05	0.000482772
ENSMMUG00000002912	TLE family member 6, subcortical maternal complex member [Source:VGNC Symbol;Acc:VGNC:79879]	-1.094159414	0.000156967	0.000892039
ENSMMUG00000002961	G protein subunit alpha 11 [Source:VGNC Symbol;Acc:VGNC:73085]	-0.920199049	2.45E-06	2.15E-05
ENSMMUG00000017036	nicalin [Source:VGNC Symbol;Acc:VGNC:75032]	2.255079901	4.09E-13	1.26E-11
ENSMMUG00000015513	CUGBP Elav-like family member 5 [Source:NCBI gene;Acc:721694]	1.501974556	0.002365072	0.009497529
ENSMMUG00000060497		1.735971104	0.000398675	0.00202358
ENSMMUG00000028930	tight junction protein 3 [Source:NCBI gene;Acc:721760]	-0.436305174	0.00785823	0.026412737
ENSMMUG00000044080	mitochondrial ribosomal protein L54 [Source:NCBI gene;Acc:713878]	0.916780406	0.000248371	0.001337249
ENSMMUG00000001917	megakaryocyte-associated tyrosine kinase [Source:NCBI gene;Acc:100430078]	1.179300119	1.45E-09	2.47E-08
ENSMMUG00000056147		-1.027789415	0.000153293	0.000873204
ENSMMUG00000022765	death associated protein kinase 3 [Source:VGNC Symbol;Acc:VGNC:71734]	-1.738298404	0.016782542	0.049671436
ENSMMUG00000022767	eukaryotic translation elongation factor 2 [Source:NCBI gene;Acc:721806]	-2.257268237	0.006982324	0.023849165
ENSMMUG00000019564	mitogen-activated protein kinase kinase 2 [Source:VGNC Symbol;Acc:VGNC:81453]	-0.435927136	0.004587762	0.016664741
ENSMMUG00000028928	ankyrin repeat domain 24 [Source:NCBI gene;Acc:721829]	0.576333319	2.64E-05	0.000182244
ENSMMUG00000010100	YJU2 splicing factor homolog [Source:NCBI gene;Acc:718892]	1.166368197	0.001715237	0.007233653
ENSMMUG00000028925	HDGF like 2 [Source:NCBI gene;Acc:693476]	-2.347433091	5.61E-05	0.000358413
ENSMMUG00000022656	perilipin 4 [Source:NCBI gene;Acc:693610]	-1.589248082	6.57E-05	0.000411191
ENSMMUG00000021716	dipeptidyl peptidase 9 [Source:NCBI gene;Acc:695587]	-0.468074284	0.009575641	0.031267092
ENSMMUG00000062995		0.486531806	0.010453571	0.033605389
ENSMMUG00000058275	arrestin domain containing 5 [Source:NCBI gene;Acc:697277]	0.652542801	0.002582307	0.010209138
ENSMMUG00000012677	lysine demethylase 4B [Source:VGNC Symbol;Acc:VGNC:73930]	-0.792609482	9.76E-06	7.48E-05

ENSMMUG00000016665	NADH:ubiquinone oxidoreductase subunit A11 [Source:NCBI gene;Acc:697843]	-1.18249537	0.000519382	0.002556504
ENSMMUG00000011963	vimentin type intermediate filament associated coiled-coil protein [Source:NCBI gene;Acc:697958]	-0.843037094	5.50E-06	4.46E-05
ENSMMUG00000011964	RAN binding protein 3 [Source:VGNC Symbol;Acc:VGNC:76655]	-0.488226069	0.000882382	0.004068247
ENSMMUG00000016526	solute carrier family 25 member 41 [Source:VGNC Symbol;Acc:VGNC:77581]	-2.07748264	3.95E-08	5.12E-07
ENSMMUG00000019314	vav guanine nucleotide exchange factor 1 [Source:VGNC Symbol;Acc:VGNC:78757]	2.547634397	1.22E-08	1.77E-07
ENSMMUG00000001220	adhesion G protein-coupled receptor E1 [Source:VGNC Symbol;Acc:VGNC:69561]	0.920289361	3.01E-07	3.30E-06
ENSMMUG00000063988		1.688211633	1.17E-05	8.85E-05
ENSMMUG00000019966	zinc finger protein 557 [Source:NCBI gene;Acc:100424983]	0.70834772	7.70E-09	1.15E-07
ENSMMUG00000057682		0.690863899	5.29E-08	6.72E-07
ENSMMUG00000028907	insulin receptor [Source:NCBI gene;Acc:706009]	2.493877586	8.51E-11	1.80E-09
ENSMMUG00000028906	Rho/Rac guanine nucleotide exchange factor 18 [Source:NCBI gene;Acc:707510]	1.786597798	9.34E-05	0.000562025
ENSMMUG00000009540	calmodulin regulated spectrin associated protein family member 3 [Source:VGNC Symbol;Acc:VGNC:70669]	-0.720795048	3.72E-07	3.99E-06
ENSMMUG00000009539	XPA binding protein 2 [Source:VGNC Symbol;Acc:VGNC:78943]	-0.733468893	5.85E-08	7.34E-07
ENSMMUG00000022463	C-type lectin CD209L2 [Source:NCBI gene;Acc:574373]	1.286744541	0.000668572	0.00319285
ENSMMUG00000023303	ecotropic viral integration site 5 like [Source:NCBI gene;Acc:706628]	1.578427119	2.82E-35	1.21E-32
ENSMMUG00000014032	proline rich 36 [Source:NCBI gene;Acc:710443]	2.492118572	2.44E-05	0.000171194
ENSMMUG00000006544	ELAV like RNA binding protein 1 [Source:VGNC Symbol;Acc:VGNC:72200]	2.918411998	0.001907772	0.007926058
ENSMMUG00000010614	C-C motif chemokine ligand 25 [Source:VGNC Symbol;Acc:VGNC:70700]	3.730896486	0.000529074	0.002596608
ENSMMUG00000061632	fibrillin 3 [Source:NCBI gene;Acc:709446]	0.635995645	0.009663651	0.031519069
ENSMMUG00000011178	ceramide synthase 4 [Source:VGNC Symbol;Acc:VGNC:71077]	-0.45852424	0.002159144	0.008831592
ENSMMUG00000014247	myosin IF [Source:NCBI gene;Acc:712101]	-2.089332642	0.012866139	0.039888048
ENSMMUG00000064610	long intergenic non-protein coding RNA 1862 [Source:HGNC Symbol;Acc:HGNC:52681]	0.969456153	6.28E-06	5.02E-05
ENSMMUG00000049311	zinc finger protein 558 [Source:NCBI gene;Acc:712590]	-0.676274804	1.29E-05	9.66E-05
ENSMMUG00000019965	methyl-CpG binding domain protein 3 like 1 [Source:VGNC Symbol;Acc:VGNC:74513]	1.822549314	0.001721292	0.007254978
ENSMMUG00000064532	mucin 16, cell surface associated [Source:NCBI gene;Acc:106996460]	1.408566014	5.20E-20	3.92E-18
ENSMMUG00000018883	zinc finger protein 177 [Source:NCBI gene;Acc:709851]	0.640166807	0.001661103	0.00703392
ENSMMUG00000007875	F-box and leucine rich repeat protein 12 [Source:VGNC Symbol;Acc:VGNC:72502]	-0.893090143	4.51E-06	3.73E-05
ENSMMUG00000001219	mitochondrial ribosomal protein L4 [Source:VGNC Symbol;Acc:VGNC:74750]	-1.441259484	0.001012628	0.004573301
ENSMMUG00000014897	ribonucleoprotein, PTB binding 1 [Source:NCBI gene;Acc:715250]	1.344329717	5.52E-09	8.47E-08
ENSMMUG00000003110	tyrosine kinase 2 [Source:VGNC Symbol;Acc:VGNC:78680]	-0.486245772	0.002554395	0.010136873
ENSMMUG00000046883	autophagy related 4D cysteine peptidase [Source:NCBI gene;Acc:713111]	1.291494405	0.000483114	0.002398576
ENSMMUG00000011951	KRI1 homolog [Source:VGNC Symbol;Acc:VGNC:74127]	1.028943891	0.00030889	0.001616392
ENSMMUG00000016593	adaptor related protein complex 1 subunit mu 2 [Source:NCBI gene;Acc:720531]	2.188302314	0.000148995	0.000851391
ENSMMUG00000003744	solute carrier family 44 member 2 [Source:VGNC Symbol;Acc:VGNC:77498]	0.626927774	4.23E-06	3.51E-05
ENSMMUG00000014677	dynamin 2 [Source:VGNC Symbol;Acc:VGNC:71957]	-0.754037937	0.009838646	0.031996508
ENSMMUG00000055775	transmembrane p24 trafficking protein 1 [Source:NCBI gene;Acc:713618]	1.878763186	4.51E-17	2.44E-15
ENSMMUG00000049703	Metazoan signal recognition particle RNA [Source:RFAM;Acc:RF00017]	2.280443918	3.86E-25	4.88E-23
ENSMMUG00000018981	chromosome 19 C19orf38 homolog [Source:VGNC Symbol;Acc:VGNC:70457]	-0.555018626	0.000278606	0.001483553
ENSMMUG00000004400	Yip1 domain family member 2 [Source:VGNC Symbol;Acc:VGNC:79717]	-0.704545983	0.000194565	0.001078714
ENSMMUG00000003611	low density lipoprotein receptor [Source:VGNC Symbol;Acc:VGNC:74248]	0.868187216	0.001037287	0.004669879
ENSMMUG00000003612	SPC24 component of NDC80 kinetochore complex [Source:VGNC Symbol;Acc:VGNC:78002]	1.913174553	0.000497144	0.002458157

ENSMMUG00000023251	KN motif and ankyrin repeat domains 2 [Source:VGNC Symbol;Acc:VGNC:73938]	-0.923714742	0.000276035	0.00147094
ENSMMUG00000009334	dedicator of cytokinesis 6 [Source:VGNC Symbol;Acc:VGNC:71863]	-0.381675127	0.002829042	0.011052348
ENSMMUG00000043134	coiled-coil domain containing 159 [Source:NCBI gene;Acc:100424702]	1.704923867	1.21E-08	1.74E-07
ENSMMUG00000022207	phospholipid phosphatase related 2 [Source:NCBI gene;Acc:714388]	1.543401287	3.74E-07	4.00E-06
ENSMMUG00000010824	SWIM-type zinc finger 7 associated protein 1 [Source:VGNC Symbol;Acc:VGNC:78220]	1.615615311	6.12E-07	6.23E-06
ENSMMUG00000038989	erythropoietin receptor [Source:NCBI gene;Acc:714546]	1.683055472	9.20E-05	0.000554212
ENSMMUG00000010826	ral guanine nucleotide dissociation stimulator like 3 [Source:VGNC Symbol;Acc:VGNC:76869]	0.867390643	0.010101006	0.032674304
ENSMMUG00000053329		0.867204195	0.013873628	0.042477028
ENSMMUG00000012587	zinc finger protein 69 [Source:NCBI gene;Acc:717030]	-1.648475544	1.25E-13	4.18E-12
ENSMMUG00000005236	zinc finger protein 709 [Source:NCBI gene;Acc:717515]	0.925406814	0.004475815	0.016314011
ENSMMUG00000058614		-2.068297051	0.000451182	0.002266355
ENSMMUG00000063236	bestrophin 2 [Source:NCBI gene;Acc:716545]	-0.559451799	0.016592124	0.049234476
ENSMMUG00000017971	nucleus accumbens associated 1 [Source:VGNC Symbol;Acc:VGNC:74996]	2.001735301	5.08E-06	4.14E-05
ENSMMUG00000057038		1.194439363	0.004523706	0.016452619
ENSMMUG00000011427	syntaxin 10 [Source:VGNC Symbol;Acc:VGNC:78040]	1.779624304	3.67E-24	3.98E-22
ENSMMUG00000003555	calcium voltage-gated channel subunit alpha1 A [Source:VGNC Symbol;Acc:VGNC:70498]	1.077964501	1.00E-07	1.20E-06
ENSMMUG00000014137	adhesion G protein-coupled receptor L1 [Source:VGNC Symbol;Acc:VGNC:69658]	-0.362383741	0.008772778	0.029089842
ENSMMUG00000060000		1.238315344	0.000948444	0.004331615
ENSMMUG00000005727	protein kinase N1 [Source:VGNC Symbol;Acc:VGNC:76014]	-1.560084974	1.09E-16	5.69E-15
ENSMMUG00000005729	GIPC PDZ domain containing family member 1 [Source:VGNC Symbol;Acc:VGNC:72932]	0.884617837	8.23E-07	8.07E-06
ENSMMUG00000017906	DnaJ heat shock protein family (Hsp40) member B1 [Source:NCBI gene;Acc:718890]	1.798308455	1.56E-06	1.44E-05
ENSMMUG00000018932	adhesion G protein-coupled receptor E2 [Source:NCBI gene;Acc:654420]	1.809786854	1.99E-26	2.97E-24
ENSMMUG00000060289		1.847034901	1.69E-33	5.02E-31
ENSMMUG00000053889	olfactory receptor 7C1 [Source:NCBI gene;Acc:114674087]	-0.992087447	0.001733606	0.00729208
ENSMMUG00000053457	caspase 14 [Source:NCBI gene;Acc:718049]	1.993820706	8.89E-08	1.07E-06
ENSMMUG00000007726	synapse defective Rho GTPase homolog 1 [Source:NCBI gene;Acc:719089]	2.098196183	1.13E-06	1.07E-05
ENSMMUG00000007729	ilvB acetolactate synthase like [Source:VGNC Symbol;Acc:VGNC:73739]	1.370209923	9.14E-05	0.000551696
ENSMMUG00000013637	notch receptor 3 [Source:NCBI gene;Acc:719119]	0.514296055	0.010190467	0.032919736
ENSMMUG00000010734	cytochrome P450, family 4, subfamily F, polypeptide 8 [Source:NCBI gene;Acc:718335]	0.369355249	0.002221156	0.0090344
ENSMMUG00000004283	calcium and integrin binding family member 3 [Source:VGNC Symbol;Acc:VGNC:71339]	1.866588391	7.65E-05	0.000471416
ENSMMUG00000039774	Metazoan signal recognition particle RNA [Source:RFAM;Acc:RF00017]	1.075244975	8.36E-10	1.49E-08
ENSMMUG00000012959	solute carrier family 35 member E1 [Source:VGNC Symbol;Acc:VGNC:77536]	1.072740178	3.00E-08	3.97E-07
ENSMMUG00000042837	mediator complex subunit 26 [Source:NCBI gene;Acc:100424604]	0.970746835	1.46E-05	0.00010771
ENSMMUG00000057655		-0.838078361	3.12E-06	2.67E-05
ENSMMUG00000013923	BRISC and BRCA1 A complex member 1 [Source:VGNC Symbol;Acc:VGNC:70126]	-0.650795648	0.001058303	0.004751252
ENSMMUG00000016228	ankyrin repeat and LEM domain containing 1 [Source:VGNC Symbol;Acc:VGNC:69715]	-1.109420678	0.012754433	0.039571448
ENSMMUG00000023403	GTP binding protein 3, mitochondrial [Source:VGNC Symbol;Acc:VGNC:73170]	3.124234425	1.70E-08	2.35E-07
ENSMMUG00000000194	solute carrier family 27 member 1 [Source:VGNC Symbol;Acc:VGNC:77438]	0.602477227	0.000122372	0.000714443
ENSMMUG00000000196	niban apoptosis regulator 3 [Source:VGNC Symbol;Acc:VGNC:75241]	0.741091906	0.005217962	0.018628073
ENSMMUG00000059371		1.191589279	0.00017851	0.000998879
ENSMMUG00000000198	collagen beta(1-O)galactosyltransferase 1 [Source:NCBI gene;Acc:719980]	0.816076333	4.62E-08	5.90E-07
ENSMMUG00000015476	unc-13 homolog A [Source:NCBI gene;Acc:720000]	0.748862737	3.84E-09	6.02E-08

ENSMMUG00000010996	Janus kinase 3 [Source:VGNC Symbol;Acc:VGNC:73812]	0.728940183	5.07E-07	5.27E-06
ENSMMUG00000038308	coiled-coil domain containing 124 [Source:NCBI gene;Acc:720072]	-0.885671697	0.000593772	0.002873392
ENSMMUG00000007398	potassium calcium-activated channel subfamily N member 1 [Source:VGNC Symbol;Acc:VGNC:74002]	-2.203677919	1.55E-10	3.16E-09
ENSMMUG00000007401	arrestin domain containing 2 [Source:VGNC Symbol;Acc:VGNC:69901]	1.29372865	1.04E-10	2.18E-09
ENSMMUG00000003161	microtubule associated serine/threonine kinase 3 [Source:VGNC Symbol;Acc:VGNC:74533]	0.538985949	0.001140527	0.00505637
ENSMMUG00000011317	RAB3D, member RAS oncogene family [Source:VGNC Symbol;Acc:VGNC:81542]	-0.963717967	0.004939248	0.017781538
ENSMMUG00000005028	phosphodiesterase 4C [Source:NCBI gene;Acc:720171]	-0.967321397	0.000151699	0.000865483
ENSMMUG00000016354	IQ motif containing N [Source:VGNC Symbol;Acc:VGNC:73683]	-0.88517047	6.64E-05	0.000414846
ENSMMUG00000038371	JunD proto-oncogene, AP-1 transcription factor subunit [Source:NCBI gene;Acc:720182]	-0.794494001	0.004074982	0.01506945
ENSMMUG00000013671	elongation factor for RNA polymerase II [Source:VGNC Symbol;Acc:VGNC:72088]	1.086135923	4.28E-12	1.13E-10
ENSMMUG00000016085	KxDL motif containing 1 [Source:NCBI gene;Acc:720235]	0.546634572	0.002140414	0.008760047
ENSMMUG00000054512		1.144341699	0.002356846	0.009472346
ENSMMUG00000009988	transmembrane protein 59 like [Source:VGNC Symbol;Acc:VGNC:78919]	2.040763744	8.80E-26	1.20E-23
ENSMMUG00000064204	kelch like family member 26 [Source:NCBI gene;Acc:719583]	0.701693445	0.008870477	0.029340186
ENSMMUG00000006509	CREB regulated transcription coactivator 1 [Source:VGNC Symbol;Acc:VGNC:71418]	-0.748517361	0.003868876	0.014440588
ENSMMUG00000056644		0.561470841	0.009514777	0.031110283
ENSMMUG00000023286	UPF1 RNA helicase and ATPase [Source:VGNC Symbol;Acc:VGNC:79971]	2.955716173	1.08E-39	7.87E-37
ENSMMUG00000050739		1.186338382	0.004009734	0.014873564
ENSMMUG00000019668	armadillo repeat containing 6 [Source:VGNC Symbol;Acc:VGNC:69899]	0.790990748	0.00468392	0.016958941
ENSMMUG00000008578	hyaluronan and proteoglycan link protein 4 [Source:VGNC Symbol;Acc:VGNC:73261]	-1.228087573	2.89E-05	0.000198403
ENSMMUG00000008931	MAU2 sister chromatid cohesion factor [Source:NCBI gene;Acc:720347]	0.434074867	1.80E-05	0.000129549
ENSMMUG00000003164	GATA zinc finger domain containing 2A [Source:NCBI gene;Acc:720361]	-0.719526666	4.73E-07	4.95E-06
ENSMMUG00000011351	YjeF N-terminal domain containing 3 [Source:NCBI gene;Acc:721218]	-0.614576373	6.15E-05	0.000387977
ENSMMUG00000048108	PBX homeobox 4 [Source:NCBI gene;Acc:106994668]	-2.183536672	0.014273275	0.043499076
ENSMMUG00000054055	zinc finger protein 93 [Source:NCBI gene;Acc:720434]	0.898719627	8.75E-11	1.85E-09
ENSMMUG00000056683		-0.623779714	0.012802685	0.039699792
ENSMMUG00000061085		-0.559195447	1.91E-05	0.00013651
ENSMMUG00000062866		-1.205014896	6.96E-05	0.000432617
ENSMMUG00000061186		-0.799210133	1.65E-06	1.51E-05
ENSMMUG00000060408		-1.037438923	0.003488205	0.013229333
ENSMMUG00000003688	zinc finger protein 91 [Source:HGNC Symbol;Acc:HGNC:13166]	-0.927020519	0.008836714	0.02923634
ENSMMUG00000059910		-0.407874786	0.006218059	0.021583555
ENSMMUG00000020668	cyclin E1 [Source:VGNC Symbol;Acc:VGNC:70727]	2.489899799	3.68E-29	7.99E-27
ENSMMUG00000063586	zinc finger protein 536 [Source:NCBI gene;Acc:713142]	-1.882450975	9.31E-07	9.03E-06
ENSMMUG00000058740		-0.961130036	7.10E-10	1.28E-08

ENSMMUG00000050177		-1.431736501	1.24E-08	1.79E-07
ENSMMUG00000060455		-2.630547642	6.46E-05	0.00040579
ENSMMUG00000008041	ankyrin repeat domain 27 [Source:VGNC Symbol;Acc:VGNC:69693]	0.373406894	0.003641757	0.013740001
ENSMMUG00000015608	tudor domain containing 12 [Source:VGNC Symbol;Acc:VGNC:82254]	-0.771056635	0.001381119	0.005982519
ENSMMUG00000028800	solute carrier family 7 member 9 [Source:VGNC Symbol;Acc:VGNC:77633]	-1.03453077	7.71E-08	9.44E-07
ENSMMUG00000017939	FA core complex associated protein 24 [Source:VGNC Symbol;Acc:VGNC:72388]	-0.794229285	0.00069175	0.003289492
ENSMMUG00000053887	Small nucleolar RNA U3 [Source:RFAM;Acc:RF00012]	-2.217913045	0.014831194	0.044879489
ENSMMUG00000064983		-0.454501383	0.010569728	0.033888869
ENSMMUG00000061372		-1.197932088	0.001319566	0.005750157
ENSMMUG00000062413	CCAAT enhancer binding protein gamma [Source:NCBI gene;Acc:704322]	-0.985157085	7.78E-06	6.08E-05
ENSMMUG00000006128	peptidase D [Source:VGNC Symbol;Acc:VGNC:82205]	-0.85292634	0.000870597	0.004024125
ENSMMUG00000015744	zinc finger protein 302 [Source:NCBI gene;Acc:706279]	1.921883691	9.22E-20	6.67E-18
ENSMMUG00000059543	zinc finger protein 30 [Source:NCBI gene;Acc:718310]	-0.799541942	1.39E-06	1.30E-05
ENSMMUG00000008313	zinc finger protein 792 [Source:VGNC Symbol;Acc:VGNC:79456]	-0.697832041	3.48E-05	0.000234559
ENSMMUG00000001538	GRAM domain containing 1A [Source:VGNC Symbol;Acc:VGNC:73029]	0.557575033	0.000186592	0.001039666
ENSMMUG00000055747	cytochrome c oxidase subunit 6B [Source:NCBI gene;Acc:692062]	1.079985787	1.88E-07	2.15E-06
ENSMMUG00000065025		1.194608693	2.70E-08	3.60E-07
ENSMMUG00000002381	lysine methyltransferase 2B [Source:VGNC Symbol;Acc:VGNC:74053]	0.592775068	3.17E-05	0.000215852
ENSMMUG00000002384	IGF like family receptor 1 [Source:VGNC Symbol;Acc:VGNC:73434]	1.445486112	4.53E-15	1.92E-13
ENSMMUG00000048190	U2 small nuclear RNA auxiliary factor 1 like 4 [Source:NCBI gene;Acc:718865]	1.189423216	0.000258199	0.00138401
ENSMMUG00000002392	proline and serine rich 3 [Source:NCBI gene;Acc:710857]	-1.050059509	0.00651373	0.02244373
ENSMMUG00000019749	NPHS1 adhesion molecule, nephrin [Source:NCBI gene;Acc:718929]	-1.006638832	0.000214951	0.001180485
ENSMMUG00000019756	NFKB inhibitor delta [Source:VGNC Symbol;Acc:VGNC:75179]	-0.781855663	0.000210699	0.001158882
ENSMMUG00000008004	transmembrane immune signaling adaptor TYROBP [Source:NCBI gene;Acc:574207]	-0.50633412	0.002656487	0.010454075
ENSMMUG00000062667	leucine rich repeat and fibronectin type III domain containing 3 [Source:NCBI gene;Acc:711565]	-0.736293474	0.002398186	0.00960133
ENSMMUG00000062586		-1.412524161	0.00421309	0.015501313
ENSMMUG00000053233		-0.797164632	0.011273207	0.035772788
ENSMMUG00000019699	spectrin repeat containing nuclear envelope family member 4 [Source:VGNC Symbol;Acc:VGNC:78221]	-2.270616471	0.004092628	0.015120682
ENSMMUG00000051879	CAP-Gly domain containing linker protein 3 [Source:NCBI gene;Acc:712058]	2.086765688	0.001237982	0.005438664
ENSMMUG00000019704	WD repeat domain 62 [Source:NCBI gene;Acc:719053]	3.301922583	0.006670569	0.022940635
ENSMMUG00000061035	ZFP82 zinc finger protein [Source:NCBI gene;Acc:719145]	-0.9038586	1.83E-10	3.66E-09
ENSMMUG00000000547	zinc finger protein 566 [Source:VGNC Symbol;Acc:VGNC:81619]	-0.658738801	7.53E-06	5.91E-05
ENSMMUG00000016449	zinc finger protein 382 [Source:VGNC Symbol;Acc:VGNC:79078]	-0.991083318	3.05E-05	0.000208029
ENSMMUG00000000107	zinc finger protein 585A [Source:NCBI gene;Acc:100424957]	0.409205167	0.004412457	0.016140887
ENSMMUG00000051305		1.194224638	6.06E-05	0.000382986
ENSMMUG00000028771	Yip1 interacting factor homolog B, membrane trafficking protein [Source:VGNC Symbol;Acc:VGNC:79852]	1.088915531	0.00089148	0.004104986

ENSMMUG00000062296	potassium two pore domain channel subfamily K member 6 [Source:NCBI gene;Acc:714912]	3.423153597	0.000658555	0.003150184
ENSMMUG00000014273	cation channel sperm associated auxiliary subunit gamma [Source:VGNC Symbol;Acc:VGNC:70626]	2.220230864	1.16E-08	1.67E-07
ENSMMUG00000038221	proteasome 26S subunit, non-ATPase 8 [Source:NCBI gene;Acc:721897]	0.920822083	2.77E-06	2.40E-05
ENSMMUG00000022973	gametogenetin [Source:VGNC Symbol;Acc:VGNC:72740]	0.535695576	0.015556259	0.046646316
ENSMMUG00000022975	sprouty related EVH1 domain containing 3 [Source:VGNC Symbol;Acc:VGNC:78030]	-3.990535033	1.91E-34	6.78E-32
ENSMMUG00000020468	family with sequence similarity 98 member C [Source:VGNC Symbol;Acc:VGNC:72584]	-0.325326461	0.015545688	0.046633855
ENSMMUG00000028767	eukaryotic translation initiation factor 3 subunit K [Source:VGNC Symbol;Acc:VGNC:72102]	-0.856794701	0.001467642	0.00629887
ENSMMUG00000028766	galectin 4 [Source:VGNC Symbol;Acc:VGNC:74257]	0.832700691	0.003787799	0.014195212
ENSMMUG00000004877	heterogeneous nuclear ribonucleoprotein L [Source:VGNC Symbol;Acc:VGNC:81187]	0.958454301	5.97E-05	0.00037812
ENSMMUG00000012787	acid phosphatase 7, tartrate resistant (putative) [Source:NCBI gene;Acc:695532]	1.864902989	3.09E-05	0.000210199
ENSMMUG00000013140	p21 (RAC1) activated kinase 4 [Source:NCBI gene;Acc:696676]	2.256643386	0.000491433	0.002434886
ENSMMUG00000005554	mediator complex subunit 29 [Source:VGNC Symbol;Acc:VGNC:74557]	3.462085259	5.05E-27	8.07E-25
ENSMMUG00000038702	ZFP36 ring finger protein [Source:NCBI gene;Acc:722000]	3.872803321	0.000230251	0.001251742
ENSMMUG00000019369	pleckstrin homology and RhoGEF domain containing G2 [Source:VGNC Symbol;Acc:VGNC:76133]	2.397111328	0.005843907	0.020505158
ENSMMUG00000048215		2.368184578	5.31E-07	5.48E-06
ENSMMUG00000022726	dual specificity tyrosine phosphorylation regulated kinase 1B [Source:VGNC Symbol;Acc:VGNC:72016]	-2.307667124	1.47E-07	1.72E-06
ENSMMUG00000022724		-1.39610129	9.37E-05	0.000563371
ENSMMUG00000051261		-1.587089199	4.01E-07	4.25E-06
ENSMMUG00000017191	zinc finger protein 546 [Source:VGNC Symbol;Acc:VGNC:79732]	-0.921512319	0.004422497	0.016165581
ENSMMUG00000041976	zinc finger protein 780B [Source:NCBI gene;Acc:701034]	-0.640612468	0.000199417	0.001100999
ENSMMUG00000003621	AKT serine/threonine kinase 2 [Source:VGNC Symbol;Acc:VGNC:69777]	-0.977852902	1.97E-08	2.69E-07
ENSMMUG00000053624		-1.124542179	8.02E-05	0.000491588
ENSMMUG00000008946	chromosome 19 C19orf47 homolog [Source:VGNC Symbol;Acc:VGNC:70404]	-0.931833534	1.40E-11	3.39E-10
ENSMMUG00000022625	periaxin [Source:VGNC Symbol;Acc:VGNC:76248]	-1.587886208	6.45E-07	6.51E-06
ENSMMUG00000015603	biliverdin reductase B [Source:VGNC Symbol;Acc:VGNC:70257]	-1.733252282	0.005043548	0.018103223
ENSMMUG00000049359		2.794885037	0.001484943	0.006363979
ENSMMUG00000063763	cytochrome P450 2A13-like [Source:NCBI gene;Acc:114674219]	0.948678224	0.001366317	0.005923693
ENSMMUG00000009145	AXL receptor tyrosine kinase [Source:VGNC Symbol;Acc:VGNC:70200]	-0.500317655	0.004504378	0.016398734
ENSMMUG00000064979	B9 domain containing 2 [Source:NCBI gene;Acc:704873]	-1.040803614	0.013349769	0.041141741
ENSMMUG00000013422	CEA cell adhesion molecule 4 [Source:NCBI gene;Acc:705453]	1.320247149	0.006452998	0.022282003
ENSMMUG00000059229	CEA cell adhesion molecule 7 [Source:NCBI gene;Acc:705572]	1.283215653	1.82E-15	8.25E-14
ENSMMUG00000022305	Rho guanine nucleotide exchange factor 1 [Source:VGNC Symbol;Acc:VGNC:70012]	-1.106437257	1.76E-07	2.02E-06
ENSMMUG00000022309	ETS repressor factor like [Source:NCBI gene;Acc:722195]	-1.460049218	0.000470176	0.002343945
ENSMMUG00000017427	ATPase Na+/K+ transporting subunit alpha 3 [Source:NCBI gene;Acc:706513]	-2.272822781	4.64E-05	0.000304623
ENSMMUG00000013376	POU class 2 homeobox 2 [Source:NCBI gene;Acc:706835]	-0.821162286	5.78E-06	4.67E-05
ENSMMUG00000050799		3.414608895	0.00248492	0.009910366
ENSMMUG00000064250		2.578398391	0.000441553	0.002223367

ENSMMUG00000055813	pregnancy-specific beta-1-glycoprotein 5-like [Source:NCBI gene;Acc:114674020]	0.669196547	0.004751659	0.01717853
ENSMMUG00000059128		-2.44910551	4.66E-07	4.89E-06
ENSMMUG00000061010	putative pregnancy-specific beta-1-glycoprotein 7 [Source:NCBI gene;Acc:114673986]	3.717319434	0.00070669	0.003348166
ENSMMUG00000021062	CD177 molecule [Source:VGNC Symbol;Acc:VGNC:70912]	4.002885531	2.44E-16	1.22E-14
ENSMMUG00000003568	testis expressed 101 [Source:VGNC Symbol;Acc:VGNC:79869]	1.392879497	5.34E-08	6.76E-07
ENSMMUG00000044157	pleckstrin homology like domain family B member 3 [Source:NCBI gene;Acc:722281]	0.697945504	0.001814342	0.007581228
ENSMMUG00000019265	zinc finger protein 575 [Source:VGNC Symbol;Acc:VGNC:79667]	-0.716638302	0.004986159	0.017919355
ENSMMUG00000004616	plasminogen activator, urokinase receptor [Source:VGNC Symbol;Acc:VGNC:76163]	1.790086075	0.003762586	0.014126218
ENSMMUG00000021267	potassium calcium-activated channel subfamily N member 4 [Source:NCBI gene;Acc:712383]	0.647924574	0.000427458	0.002158375
ENSMMUG00000021474	zinc finger protein 45 [Source:NCBI gene;Acc:712591]	-0.805515703	3.86E-07	4.11E-06
ENSMMUG00000064975	zinc finger protein 221 [Source:NCBI gene;Acc:100423743]	-1.1759783	0.000462067	0.002310654
ENSMMUG00000012797	zinc finger protein 233 [Source:VGNC Symbol;Acc:VGNC:79903]	1.853989241	1.55E-17	8.84E-16
ENSMMUG00000012801	zinc finger protein 235 [Source:VGNC Symbol;Acc:VGNC:78825]	1.79981689	3.48E-34	1.12E-31
ENSMMUG00000014301	nectin cell adhesion molecule 2 [Source:VGNC Symbol;Acc:VGNC:75396]	-0.510984881	0.000946133	0.004323778
ENSMMUG00000014303	translocase of outer mitochondrial membrane 40 [Source:VGNC Symbol;Acc:VGNC:78419]	-2.289182744	0.005874499	0.020602556
ENSMMUG00000028749	apolipoprotein C1 [Source:NCBI gene;Acc:106994799]	-1.206199258	0.013860021	0.042444306
ENSMMUG00000002472	CLPTM1 regulator of GABA type A receptor forward trafficking [Source:VGNC Symbol;Acc:VGNC:71265]	-1.420942009	0.000276214	0.001471354
ENSMMUG00000014155	CLK4 associating serine/arginine rich protein [Source:VGNC Symbol;Acc:VGNC:71198]	-1.316257986	0.002549595	0.010123862
ENSMMUG00000022931	NTPase KAP family P-loop domain containing 1 [Source:VGNC Symbol;Acc:VGNC:75250]	0.803302089	1.69E-06	1.54E-05
ENSMMUG00000018879	exocyst complex component 3 like 2 [Source:NCBI gene;Acc:713992]	0.541467111	0.000220367	0.001206583
ENSMMUG00000018672	microtubule affinity regulating kinase 4 [Source:VGNC Symbol;Acc:VGNC:74508]	-0.54505367	0.00094786	0.004330308
ENSMMUG00000013477	creatine kinase, M-type [Source:VGNC Symbol;Acc:VGNC:71230]	3.072123399	4.12E-15	1.77E-13
ENSMMUG00000000715	protein phosphatase 1 regulatory subunit 13 like [Source:NCBI gene;Acc:714389]	0.491782423	0.001893037	0.00787159
ENSMMUG00000000717	CD3e molecule associated protein [Source:NCBI gene;Acc:714440]	-1.449946645	2.02E-10	4.01E-09
ENSMMUG00000000718	ERCC excision repair 1, endonuclease non-catalytic subunit [Source:VGNC Symbol;Acc:VGNC:72422]	-0.956363075	7.36E-06	5.80E-05
ENSMMUG00000006936	gastric inhibitory polypeptide receptor [Source:VGNC Symbol;Acc:VGNC:73052]	0.798969647	2.74E-08	3.65E-07
ENSMMUG00000032809	mml-mir-642 [Source:miRBase;Acc:MI0007885]	0.776703218	0.00031035	0.001622442
ENSMMUG00000006937	small nuclear ribonucleoprotein D2 polypeptide [Source:NCBI gene;Acc:715183]	-1.642951377	0.003468711	0.013165705
ENSMMUG00000051573	SIX homeobox 5 [Source:NCBI gene;Acc:715373]	-0.408507632	0.003145684	0.012113593
ENSMMUG00000016457	DM1 protein kinase [Source:NCBI gene;Acc:715328]	0.753541979	0.012936151	0.04007092
ENSMMUG00000051115		-3.001256059	3.87E-09	6.06E-08
ENSMMUG00000017927	protein kinase D2 [Source:VGNC Symbol;Acc:VGNC:76273]	2.655847443	2.71E-05	0.00018744
ENSMMUG00000017930	striatin 4 [Source:VGNC Symbol;Acc:VGNC:78128]	3.553021728	0.000520417	0.002560169
ENSMMUG00000011332	Rho GTPase activating protein 35 [Source:VGNC Symbol;Acc:VGNC:69942]	2.149160349	3.74E-10	7.17E-09
ENSMMUG00000057361	Metazoan signal recognition particle RNA [Source:RFAM;Acc:RF00017]	2.105916803	4.82E-08	6.15E-07
ENSMMUG00000057454	transmembrane protein 160 [Source:NCBI gene;Acc:716996]	0.934292775	0.012605082	0.039204125
ENSMMUG00000055893		0.896897463	0.001693281	0.007151429
ENSMMUG00000014727	NSF attachment protein alpha [Source:VGNC Symbol;Acc:VGNC:75131]	-0.615121823	0.000369141	0.001888178
ENSMMUG00000054090		1.132409138	0.000279699	0.001487737
ENSMMUG00000006857	epididymal sperm binding protein 1 [Source:VGNC Symbol;Acc:VGNC:72046]	-0.765570622	0.006745647	0.02316595

ENSMMUG00000042157	caspase recruitment domain-containing protein 8 [Source:NCBI gene;Acc:718594]	-0.420900905	0.00127351	0.005572853
ENSMMUG00000022155	sulfotransferase family 2B member 1 [Source:NCBI gene;Acc:718739]	0.837217919	0.001086157	0.004849346
ENSMMUG00000055036	MEF2 activating motif and SAP domain containing transcriptional regulator [Source:NCBI gene;Acc:718823]	-0.463940799	0.010475377	0.033645726
ENSMMUG00000018415	Ras interacting protein 1 [Source:HGNC Symbol;Acc:HGNC:24716]	-0.613553367	0.000925743	0.004242355
ENSMMUG00000004019	izumo sperm-egg fusion 1 [Source:VGNC Symbol;Acc:VGNC:73670]	-0.755162862	0.000208211	0.001146065
ENSMMUG00000028736	hydroxysteroid 17-beta dehydrogenase 14 [Source:NCBI gene;Acc:106994816]	-0.982227071	4.51E-05	0.000297223
ENSMMUG00000048854	TUB like protein 2 [Source:NCBI gene;Acc:718915]	-0.934890126	0.01172285	0.036894366
ENSMMUG00000003904	nucleobindin 1 [Source:VGNC Symbol;Acc:VGNC:75548]	-1.732954064	0.002987458	0.011587159
ENSMMUG00000007538	transient receptor potential cation channel subfamily M member 4 [Source:VGNC Symbol;Acc:VGNC:79105]	1.739557603	3.24E-11	7.36E-10
ENSMMUG00000022116	PIH1 domain containing 1 [Source:VGNC Symbol;Acc:VGNC:75868]	1.329800177	0.00380572	0.014247422
ENSMMUG00000007551	ribosomal protein L13a [Source:NCBI gene;Acc:574314]	0.57954075	0.00835481	0.02783086
ENSMMUG00000003819	nitric oxide synthase interacting protein [Source:VGNC Symbol;Acc:VGNC:75207]	1.883649641	9.46E-11	1.99E-09
ENSMMUG00000012937	proline rich 12 [Source:NCBI gene;Acc:719344]	2.661672886	4.45E-24	4.72E-22
ENSMMUG00000028733	RAS related [Source:VGNC Symbol;Acc:VGNC:76937]	2.46176924	1.39E-17	7.94E-16
ENSMMUG00000012938	SR-related CTD associated factor 1 [Source:VGNC Symbol;Acc:VGNC:77137]	3.618072182	6.36E-06	5.08E-05
ENSMMUG00000003926	BCL2 like 12 [Source:NCBI gene;Acc:718981]	3.653396103	0.000643162	0.003081622
ENSMMUG00000003029	protein arginine methyltransferase 1 [Source:NCBI gene;Acc:719419]	-1.468312555	1.57E-05	0.000114566
ENSMMUG00000014735	carnitine palmitoyltransferase 1C [Source:NCBI gene;Acc:719429]	-1.674325134	3.80E-07	4.05E-06
ENSMMUG00000020175	mediator complex subunit 25 [Source:VGNC Symbol;Acc:VGNC:74607]	-1.98735336	1.21E-06	1.15E-05
ENSMMUG00000011627	interleukin 4 induced 1 [Source:VGNC Symbol;Acc:VGNC:73625]	2.422022389	1.21E-10	2.50E-09
ENSMMUG00000044817	nucleoporin 62 [Source:NCBI gene;Acc:100426308]	3.05966563	7.48E-61	5.43E-57
ENSMMUG00000054473	activating transcription factor 5 [Source:NCBI gene;Acc:719150]	2.198571115	8.65E-12	2.18E-10
ENSMMUG00000050456	Metazoan signal recognition particle RNA [Source:RFAM;Acc:RF00017]	1.663158771	0.003475563	0.013188269
ENSMMUG00000064146	Metazoan signal recognition particle RNA [Source:RFAM;Acc:RF00017]	3.148051395	5.38E-16	2.62E-14
ENSMMUG00000054052	sialic acid binding Ig like lectin 11 [Source:NCBI gene;Acc:719127]	3.69028618	1.45E-09	2.47E-08
ENSMMUG00000010837	VRK serine/threonine kinase 3 [Source:VGNC Symbol;Acc:VGNC:81605]	2.098902671	5.03E-05	0.00032599
ENSMMUG00000055885	napsin-A [Source:NCBI gene;Acc:719235]	-0.773531549	0.007786682	0.026208764
ENSMMUG00000023418	nuclear receptor subfamily 1 group H member 2 [Source:VGNC Symbol;Acc:VGNC:75504]	0.622991954	1.74E-06	1.58E-05
ENSMMUG00000021269	Josephin domain containing 2 [Source:VGNC Symbol;Acc:VGNC:73919]	3.566930949	0.002236373	0.009083585
ENSMMUG00000046099	chromosome 19 C19orf81 homolog [Source:NCBI gene;Acc:719661]	2.230887379	0.002329778	0.009392118
ENSMMUG00000059087		2.940582104	0.000339559	0.00175477
ENSMMUG00000009357	SH3 and multiple ankyrin repeat domains 1 [Source:VGNC Symbol;Acc:VGNC:77236]	-0.834698329	0.006214553	0.02158169
ENSMMUG00000012841	kallikrein 3 [Source:NCBI gene;Acc:719444]	0.814032424	0.006730094	0.023123463
ENSMMUG00000065035	kallikrein-2 [Source:NCBI gene;Acc:719436]	-0.702902197	0.001185067	0.005236267
ENSMMUG00000060764		0.455326966	8.27E-06	6.42E-05
ENSMMUG00000013597	kallikrein related peptidase 5 [Source:VGNC Symbol;Acc:VGNC:74191]	-0.736475564	0.003796424	0.014216542
ENSMMUG00000061396	kallikrein related peptidase 6 [Source:NCBI gene;Acc:719776]	-0.552673199	0.012131216	0.037975824
ENSMMUG00000064856	kallikrein related peptidase 14 [Source:NCBI gene;Acc:722692]	-1.089748693	0.005939413	0.020795099

ENSMMUG00000044243	sialic acid-binding Ig-like lectin 9 [Source:NCBI gene;Acc:719831]	-1.373348014	0.002945033	0.011456232
ENSMMUG00000047370	IgLON family member 5 [Source:NCBI gene;Acc:719890]	-0.895074672	0.002893894	0.011275404
ENSMMUG00000044652	sialic acid-binding Ig-like lectin 13 [Source:NCBI gene;Acc:719667]	-0.738085453	0.00023079	0.001253267
ENSMMUG00000012764	zinc finger protein 175 [Source:VGNC Symbol;Acc:VGNC:78856]	-0.655803526	0.002598808	0.010265996
ENSMMUG00000012761	sialic acid binding Ig like lectin 5 [Source:NCBI gene;Acc:719730]	-0.788975278	1.05E-06	1.01E-05
ENSMMUG00000013053	sperm acrosome associated 6 [Source:NCBI gene;Acc:114674203]	-1.316277229	3.94E-06	3.30E-05
ENSMMUG00000013049	formyl peptide receptor 1 [Source:NCBI gene;Acc:719782]	-0.565529699	0.013856064	0.042441131
ENSMMUG00000051078		-0.518611695	0.0122171	0.038218202
ENSMMUG00000002310	zinc finger protein 577 [Source:VGNC Symbol;Acc:VGNC:79733]	-0.849606996	1.99E-12	5.54E-11
ENSMMUG00000023136	zinc finger protein 613 [Source:VGNC Symbol;Acc:VGNC:79548]	-0.607089729	4.62E-06	3.80E-05
ENSMMUG00000052090		-0.659236771	3.51E-07	3.79E-06
ENSMMUG00000023137	zinc finger protein 350 [Source:VGNC Symbol;Acc:VGNC:79041]	-1.346521349	9.50E-08	1.14E-06
ENSMMUG00000003712	zinc finger protein 615 [Source:VGNC Symbol;Acc:VGNC:79454]	-0.699146538	0.010042638	0.032536177
ENSMMUG00000041450	zinc finger protein 432 [Source:NCBI gene;Acc:100424341]	-1.542226997	4.09E-11	9.10E-10
ENSMMUG00000001768	zinc finger protein 616 [Source:NCBI gene;Acc:719902]	-0.560713834	0.002357966	0.009474226
ENSMMUG00000047412	zinc finger protein 28 [Source:NCBI gene;Acc:100430953]	-1.281148235	0.015475021	0.046460219
ENSMMUG00000058137	zinc finger protein 816 [Source:NCBI gene;Acc:720058]	1.451629863	2.56E-07	2.84E-06
ENSMMUG00000064519		-1.274007684	0.000531204	0.002604423
ENSMMUG00000057187		0.656417906	4.24E-06	3.52E-05
ENSMMUG00000060222		-0.524263056	5.49E-05	0.000352428
ENSMMUG00000055976	CD99 antigen [Source:NCBI gene;Acc:106995376]	1.555686146	3.97E-06	3.32E-05
ENSMMUG00000038768	U6 spliceosomal RNA [Source:RFAM;Acc:RF00026]	-1.233464899	0.000842414	0.003911264
ENSMMUG00000042225		-0.739980993	5.74E-05	0.00036555

SD 3 vs. Baseline

Ensembl Gene ID	Description	Log2(FC)	p-value	FDR
ENSMMUG00000000632	SH3 binding domain protein 5 like [Source:VGNC Symbol;Acc:VGNC:77334]	3.343599475	3.69E-09	5.37E-08
ENSMMUG00000049101	olfactory receptor 2T10 [Source:NCBI gene;Acc:100431025]	0.573831174	0.002586152	0.010064164
ENSMMUG00000048289	olfactory receptor 2T5-like [Source:NCBI gene;Acc:707109]	0.686132722	0.00605578	0.020823983
ENSMMUG00000017368	olfactory receptor 2G6 [Source:NCBI gene;Acc:694380]	-0.593613626	0.000705621	0.003255982
ENSMMUG00000038524	olfactory receptor 2M4 [Source:NCBI gene;Acc:100424541]	2.239824045	4.74E-36	1.51E-33
ENSMMUG00000049432	olfactory receptor 2T33 [Source:NCBI gene;Acc:706331]	0.960147986	1.87E-11	3.98E-10
ENSMMUG00000064763		0.915809282	0.000294069	0.001507763
ENSMMUG00000050041	olfactory receptor 2L5 [Source:NCBI gene;Acc:697549]	0.780300535	0.014563109	0.0439274
ENSMMUG00000005949	olfactory receptor 2B11 [Source:NCBI gene;Acc:709256]	-1.885754004	0.000530889	0.002542574
ENSMMUG00000004566	zinc finger protein 124 [Source:VGNC Symbol;Acc:VGNC:78836]	-4.311556736	8.96E-05	0.000517722
ENSMMUG00000000021	catsper channel auxiliary subunit epsilon [Source:VGNC Symbol;Acc:VGNC:70657]	-3.099989041	0.000692543	0.00320407
ENSMMUG00000064143	Metazoan signal recognition particle RNA [Source:RFAM;Acc:RF00017]	2.852588937	0.009452933	0.030522329
ENSMMUG00000005668	kynurenine 3-monooxygenase [Source:VGNC Symbol;Acc:VGNC:74197]	0.898714638	4.48E-07	4.38E-06
ENSMMUG00000005667	fumarate hydratase [Source:VGNC Symbol;Acc:VGNC:72659]	0.630463415	4.27E-05	0.000267101
ENSMMUG00000064824	gremlin 2, DAN family BMP antagonist [Source:NCBI gene;Acc:708280]	1.347808692	0.000105109	0.000597079
ENSMMUG00000061845		1.676062063	0.001535774	0.006381083
ENSMMUG00000001060	ryanodine receptor 2 [Source:VGNC Symbol;Acc:VGNC:76954]	1.534198336	3.65E-65	8.53E-62
ENSMMUG00000015132	galectin 8 [Source:VGNC Symbol;Acc:VGNC:74258]	-0.874152266	0.012292518	0.038038325
ENSMMUG00000021238	EDAR associated death domain [Source:VGNC Symbol;Acc:VGNC:72043]	-0.569741852	0.013322214	0.040789827
ENSMMUG00000064750		0.462251979	0.011538192	0.036124151
ENSMMUG00000046785		-1.102143023	0.014646597	0.044103334
ENSMMUG00000012615	pecanex 2 [Source:NCBI gene;Acc:712828]	-1.558539559	5.44E-05	0.000332136
ENSMMUG00000063981	nucleoside-triphosphatase, cancer-related [Source:NCBI gene;Acc:712959]	2.850278924	2.56E-10	4.52E-09
ENSMMUG00000040055		-0.929020086	7.07E-06	5.34E-05
ENSMMUG00000061796		-1.049076035	0.000456244	0.002223814
ENSMMUG00000052961		2.018212337	3.44E-05	0.000219711
ENSMMUG00000064226		1.469629467	0.000928679	0.004126422
ENSMMUG00000016204	translin associated factor X [Source:NCBI gene;Acc:713347]	-2.981227919	0.00484172	0.017263014
ENSMMUG00000005801	glyceronephosphate O-acyltransferase [Source:VGNC Symbol;Acc:VGNC:73100]	-0.984991032	4.55E-05	0.000282191
ENSMMUG00000009508	TATA-box binding protein associated factor 5 like [Source:VGNC Symbol;Acc:VGNC:78148]	2.308684157	5.61E-05	0.000341011
ENSMMUG00000022094	ATP binding cassette subfamily B member 10 [Source:NCBI gene;Acc:574117]	0.350985506	0.002525289	0.009853122
ENSMMUG00000017720	RAB4A, member RAS oncogene family [Source:VGNC Symbol;Acc:VGNC:81545]	-0.484822183	0.010891359	0.034375317
ENSMMUG00000062933		-0.578721867	0.000448955	0.00219362
ENSMMUG00000005185	regulator of G protein signaling 4 [Source:VGNC Symbol;Acc:VGNC:76690]	2.748560523	0.007637159	0.02549295
ENSMMUG00000058513		0.290079373	0.0082678	0.027311047
ENSMMUG00000039738	U6 spliceosomal RNA [Source:RFAM;Acc:RF00026]	-1.865808556	0.00464427	0.01665216

ENSMMUG00000005585	transmembrane and coiled-coil domains 1 [Source:VGNC Symbol;Acc:VGNC:79118]	-0.57132283	0.016040233	0.047584555
ENSMMUG00000023462	immunoglobulin like domain containing receptor 2 [Source:VGNC Symbol;Acc:VGNC:73619]	-1.463128609	0.005432523	0.018992838
ENSMMUG00000023463	maelstrom spermatogenic transposon silencer [Source:VGNC Symbol;Acc:VGNC:74362]	-1.642334672	0.012518639	0.03861612
ENSMMUG00000063120	cellular repressor of E1A stimulated genes 1 [Source:NCBI gene;Acc:701021]	-0.839485204	0.000204721	0.001093222
ENSMMUG00000023372	RCSD domain containing 1 [Source:VGNC Symbol;Acc:VGNC:76850]	-1.012665829	1.38E-05	9.70E-05
ENSMMUG00000009184	myelin protein zero like 1 [Source:NCBI gene;Acc:698437]	1.300689927	0.000134976	0.000749588
ENSMMUG00000060729	T-box transcription factor 19 [Source:NCBI gene;Acc:699312]	0.428054771	0.007033679	0.023715624
ENSMMUG00000056707		1.781516748	0.004986738	0.017671714
ENSMMUG00000059777		0.905852174	2.48E-05	0.000163497
ENSMMUG00000021078	ATPase Na+/K+ transporting subunit beta 1 [Source:VGNC Symbol;Acc:VGNC:70176]	-1.787603949	6.20E-06	4.75E-05
ENSMMUG00000021080	basic leucine zipper nuclear factor 1 [Source:VGNC Symbol;Acc:VGNC:70258]	0.808667511	1.18E-08	1.56E-07
ENSMMUG00000000065	coiled-coil domain containing 181 [Source:VGNC Symbol;Acc:VGNC:70884]	-0.815705909	1.39E-06	1.22E-05
ENSMMUG00000013039	solute carrier family 19 member 2 [Source:VGNC Symbol;Acc:VGNC:81571]	-0.408979341	0.003009782	0.011437986
ENSMMUG00000042510	coagulation factor V [Source:NCBI gene;Acc:704695]	1.150408811	4.19E-18	2.17E-16
ENSMMUG00000013044	selectin P [Source:VGNC Symbol;Acc:VGNC:77157]	1.317655232	1.12E-10	2.12E-09
ENSMMUG00000016579	selectin L [Source:NCBI gene;Acc:701419]	2.640242526	1.19E-47	8.77E-45
ENSMMUG00000016580	selectin E [Source:VGNC Symbol;Acc:VGNC:77156]	2.662484121	2.09E-60	4.18E-57
ENSMMUG00000016581	methyltransferase like 18 [Source:VGNC Symbol;Acc:VGNC:74606]	-1.148320454	0.009035975	0.029399414
ENSMMUG00000016582	chromosome 1 C1orf112 homolog [Source:VGNC Symbol;Acc:VGNC:70385]	1.698466584	3.81E-15	1.40E-13
ENSMMUG00000010081	kinesin associated protein 3 [Source:VGNC Symbol;Acc:VGNC:73842]	0.442956501	0.005840316	0.020187024
ENSMMUG00000059773	methyltransferase like 11B [Source:NCBI gene;Acc:705503]	-1.099016073	0.000130305	0.000726545
ENSMMUG00000063298		-1.05217604	0.002486289	0.009717178
ENSMMUG00000014674	golgin, RAB6 interacting [Source:VGNC Symbol;Acc:VGNC:73012]	1.061174045	1.52E-08	1.98E-07
ENSMMUG00000007279	phosphatidylinositol glycan anchor biosynthesis class C [Source:VGNC Symbol;Acc:VGNC:75986]	0.664266413	0.000341735	0.001718232
ENSMMUG00000007280	SUN domain containing ossification factor [Source:VGNC Symbol;Acc:VGNC:78112]	3.503121217	0.002917735	0.011151649
ENSMMUG00000022798	aspartyl-tRNA synthetase 2, mitochondrial [Source:VGNC Symbol;Acc:VGNC:71708]	0.809794228	6.67E-05	0.000397142
ENSMMUG00000056971	Metazoan signal recognition particle RNA [Source:RFAM;Acc:RF00017]	1.748000501	2.09E-08	2.66E-07
ENSMMUG00000060229		1.074124174	0.009116322	0.029612735
ENSMMUG00000000629	serpin family C member 1 [Source:VGNC Symbol;Acc:VGNC:77360]	0.862397461	8.38E-10	1.36E-08
ENSMMUG00000000630	ring finger and CCCH-type domains 1 [Source:VGNC Symbol;Acc:VGNC:76861]	-0.45649521	5.70E-05	0.000345571
ENSMMUG00000000546	KIAA0040 ortholog [Source:NCBI gene;Acc:100423509]	-1.03001686	0.000109335	0.000618729
ENSMMUG00000023216	astrotactin 1 [Source:VGNC Symbol;Acc:VGNC:70167]	-0.392284204	0.0143378	0.04338778
ENSMMUG00000022935	BMP/retinoic acid inducible neural specific 2 [Source:VGNC Symbol;Acc:VGNC:70303]	-0.916256832	9.69E-06	7.06E-05
ENSMMUG00000020320	protein transport protein Sec16B [Source:NCBI gene;Acc:709635]	0.735199419	0.000181987	0.000983433
ENSMMUG00000021073	RAS protein activator like 2 [Source:VGNC Symbol;Acc:VGNC:76670]	2.140066643	6.78E-24	6.30E-22
ENSMMUG00000059117	Metazoan signal recognition particle RNA [Source:RFAM;Acc:RF00017]	1.244615659	0.000287845	0.001479747
ENSMMUG00000056058	C-type lectin domain containing 20A [Source:HGNC Symbol;Acc:HGNC:34521]	1.964536245	0.011059433	0.034843054
ENSMMUG00000054486	testis expressed 35 [Source:NCBI gene;Acc:717255]	2.208146948	0.001124222	0.004880793
ENSMMUG00000003770	NPHS2 stomatin family member, podocin [Source:VGNC Symbol;Acc:VGNC:75167]	-0.567994492	0.012685993	0.039037893
ENSMMUG00000055256	family with sequence similarity 163 member A [Source:NCBI gene;Acc:718704]	1.086773138	2.12E-05	0.000142453
ENSMMUG00000031279	torsin A interacting protein 2 [Source:NCBI gene;Acc:716654]	1.100251236	3.15E-11	6.47E-10

ENSMMUG00000053617	torsin 1A interacting protein 2 [Source:NCBI gene;Acc:716691]	0.504872414	0.005067992	0.017901372
ENSMMUG00000014873	torsin 1A interacting protein 1 [Source:VGNC Symbol;Acc:VGNC:79810]	0.41291784	0.007470812	0.024985259
ENSMMUG00000002135	centrosomal protein 350 [Source:VGNC Symbol;Acc:VGNC:71084]	-0.400977209	0.008277263	0.027330363
ENSMMUG00000054568		1.095286724	0.000347374	0.001738326
ENSMMUG00000061785		0.957825041	4.17E-13	1.14E-11
ENSMMUG00000022884	glutamate-ammonia ligase [Source:NCBI gene;Acc:715952]	3.707446721	1.08E-15	4.19E-14
ENSMMUG00000049444		1.888284252	0.003551564	0.013172064
ENSMMUG00000031291	regulator of G protein signaling like 1 [Source:NCBI gene;Acc:715693]	1.45995333	3.69E-29	6.08E-27
ENSMMUG00000005634	ribonuclease L [Source:VGNC Symbol;Acc:VGNC:82223]	-1.089445881	1.75E-06	1.50E-05
ENSMMUG00000055481	regulator of G protein signaling 16 [Source:NCBI gene;Acc:715599]	-1.244338456	7.61E-07	7.09E-06
ENSMMUG00000001479	laminin subunit gamma 2 [Source:VGNC Symbol;Acc:VGNC:74233]	-1.917935006	1.36E-07	1.47E-06
ENSMMUG00000001481	nicotinamide nucleotide adenylyltransferase 2 [Source:VGNC Symbol;Acc:VGNC:75193]	-1.77937384	9.68E-05	0.000553746
ENSMMUG00000013878	ring finger protein 2 [Source:NCBI gene;Acc:717024]	-1.011123434	0.000637702	0.002975871
ENSMMUG00000013879	tRNA methyltransferase 1 like [Source:VGNC Symbol;Acc:VGNC:79048]	-1.510219718	0.003100109	0.011701974
ENSMMUG00000016857	prostaglandin-endoperoxide synthase 2 [Source:VGNC Symbol;Acc:VGNC:76589]	-1.041624195	3.96E-08	4.76E-07
ENSMMUG00000059986		-0.840017501	0.001070109	0.004683565
ENSMMUG00000022647	cell division cycle 73 [Source:VGNC Symbol;Acc:VGNC:70766]	-0.701951639	0.005101586	0.017997367
ENSMMUG00000021082	potassium sodium-activated channel subfamily T member 2 [Source:VGNC Symbol;Acc:VGNC:73857]	0.376922871	0.014745525	0.044325079
ENSMMUG00000045497	complement factor H [Source:NCBI gene;Acc:717346]	-1.086387304	0.000256292	0.001339538
ENSMMUG00000000009	protein tyrosine phosphatase receptor type C [Source:VGNC Symbol;Acc:VGNC:76612]	-2.796035032	4.24E-11	8.50E-10
ENSMMUG00000012218	calmodulin regulated spectrin associated protein family member 2 [Source:VGNC Symbol;Acc:VGNC:70666]	-1.10683842	5.67E-05	0.00034431
ENSMMUG00000063678	innate immunity activator [Source:NCBI gene;Acc:710962]	-0.316794803	9.91E-05	0.000565974
ENSMMUG00000017414	calcium voltage-gated channel subunit alpha1 S [Source:VGNC Symbol;Acc:VGNC:70507]	-1.001437212	7.89E-05	0.000461471
ENSMMUG00000064915	Metazoan signal recognition particle RNA [Source:RFAM;Acc:RF00017]	1.732533035	0.000860593	0.003871701
ENSMMUG00000050920	Metazoan signal recognition particle RNA [Source:RFAM;Acc:RF00017]	1.317507434	0.005530653	0.019273448
ENSMMUG00000055783		-2.316198371	6.52E-05	0.000388955
ENSMMUG00000017114	pleckstrin homology domain containing A6 [Source:VGNC Symbol;Acc:VGNC:76040]	-0.920277785	0.008755227	0.028652171
ENSMMUG00000063658	leucine rich repeat neuronal 2 [Source:NCBI gene;Acc:100429709]	1.340873464	0.000268541	0.001394491
ENSMMUG00000017508	neurofascin [Source:VGNC Symbol;Acc:VGNC:75278]	-0.611614591	5.89E-08	6.87E-07
ENSMMUG00000018797	contactin 2 [Source:VGNC Symbol;Acc:VGNC:71284]	-1.668256563	0.010578563	0.033516309
ENSMMUG00000004113	major facilitator superfamily domain containing 4A [Source:VGNC Symbol;Acc:VGNC:74575]	-0.722081489	3.70E-05	0.000234996
ENSMMUG00000055835	nuclear casein kinase and cyclin dependent kinase substrate 1 [Source:NCBI gene;Acc:697298]	-1.333125502	1.22E-07	1.33E-06
ENSMMUG00000023570	interleukin 19 [Source:NCBI gene;Acc:694806]	3.077285655	0.015161599	0.045342526
ENSMMUG00000011221	Fc fragment of IgA and IgM receptor [Source:VGNC Symbol;Acc:VGNC:72567]	1.447355658	0.002400222	0.009430741
ENSMMUG00000022171	YOD1 deubiquitinase [Source:VGNC Symbol;Acc:VGNC:79471]	0.311794662	0.016524768	0.04872305
ENSMMUG00000040888	complement C3b/C4b receptor 1 like [Source:HGNC Symbol;Acc:HGNC:2335]	0.986954239	3.83E-09	5.57E-08

ENSMMUG00000051230		0.932179979	3.86E-07	3.83E-06
ENSMMUG00000057560		0.908596923	6.73E-08	7.78E-07
ENSMMUG00000064202		-1.231688246	0.000610681	0.002883359
ENSMMUG00000045482	G0/G1 switch 2 [Source:NCBI gene;Acc:717581]	-0.700964031	1.76E-07	1.86E-06
ENSMMUG00000016099	hedgehog acyltransferase [Source:NCBI gene;Acc:712333]	1.788320935	2.82E-05	0.000183677
ENSMMUG00000016102	potassium voltage-gated channel subfamily H member 1 [Source:NCBI gene;Acc:712190]	-2.281704869	0.011654798	0.036432286
ENSMMUG00000011914	prospero homeobox 1 [Source:VGNC Symbol;Acc:VGNC:76427]	3.348899636	6.93E-09	9.56E-08
ENSMMUG00000015634	centromere protein F [Source:VGNC Symbol;Acc:VGNC:71009]	-2.628460027	2.35E-05	0.00015589
ENSMMUG00000058133		-2.169690108	0.000343129	0.001723389
ENSMMUG00000017396	3'(2'), 5'-bisphosphate nucleotidase 1 [Source:NCBI gene;Acc:721277]	-1.261736161	3.84E-05	0.000242418
ENSMMUG00000007783	isoleucyl-tRNA synthetase 2, mitochondrial [Source:VGNC Symbol;Acc:VGNC:73458]	-1.096793912	1.00E-17	4.89E-16
ENSMMUG00000026897	mml-mir-215 [Source:miRBase;Acc:MI0003025]	-1.356944365	0.010163116	0.032427163
ENSMMUG00000027053	mml-mir-194-1 [Source:miRBase;Acc:MI0003024]	-1.380862153	0.014578853	0.043946529
ENSMMUG00000018525	presenilin 2 [Source:VGNC Symbol;Acc:VGNC:76436]	-0.974420993	1.52E-13	4.40E-12
ENSMMUG00000023096	obscurin, cytoskeletal calmodulin and titin-interacting RhoGEF [Source:NCBI gene;Acc:694413]	-0.818935752	5.85E-09	8.19E-08
ENSMMUG00000023042	olfactomedin like 2B [Source:VGNC Symbol;Acc:VGNC:75455]	0.452570624	0.003167952	0.011909969
ENSMMUG00000004042	Fc receptor like A [Source:VGNC Symbol;Acc:VGNC:72526]	1.061405913	0.00971258	0.031181206
ENSMMUG00000054686		2.41587196	2.88E-33	7.22E-31
ENSMMUG00000008251	cilia and flagella associated protein 126 [Source:VGNC Symbol;Acc:VGNC:71150]	1.708270472	0.010551714	0.033446358
ENSMMUG00000004516	apolipoprotein A2 [Source:NCBI gene;Acc:719946]	1.204669293	0.010051672	0.032115423
ENSMMUG00000004512	Fc fragment of IgE receptor Ig [Source:VGNC Symbol;Acc:VGNC:72629]	1.675752121	1.63E-06	1.41E-05
ENSMMUG00000004520	NADH:ubiquinone oxidoreductase core subunit S2 [Source:NCBI gene;Acc:719935]	0.944227862	0.000319618	0.001619807
ENSMMUG00000050419	ADAM metallopeptidase with thrombospondin type 1 motif 4 [Source:NCBI gene;Acc:719927]	1.29438112	2.57E-09	3.82E-08
ENSMMUG00000060555		1.198438502	1.89E-07	1.98E-06
ENSMMUG00000022630	protoporphyrinogen oxidase [Source:VGNC Symbol;Acc:VGNC:76365]	-1.416511079	5.09E-06	3.97E-05
ENSMMUG00000022631	ubiquitin specific peptidase 21 [Source:VGNC Symbol;Acc:VGNC:78741]	-1.113156216	0.001440128	0.006044571
ENSMMUG00000022632	ubiquitin-fold modifier conjugating enzyme 1 [Source:VGNC Symbol;Acc:VGNC:79643]	-0.840140192	0.00035867	0.001787357
ENSMMUG00000002005	Rho GTPase activating protein 30 [Source:NCBI gene;Acc:718236]	-0.435837135	0.009836586	0.031507183
ENSMMUG00000015812	CD48 molecule [Source:VGNC Symbol;Acc:VGNC:70769]	-0.502745768	0.00350212	0.013005887
ENSMMUG00000011335	Fc receptor like 6 [Source:VGNC Symbol;Acc:VGNC:72602]	-0.468624156	1.31E-05	9.21E-05
ENSMMUG00000004492	absent in melanoma 2 [Source:NCBI gene;Acc:719260]	-2.743968271	7.90E-10	1.28E-08
ENSMMUG00000000730	spectrin alpha, erythrocytic 1 [Source:VGNC Symbol;Acc:VGNC:77868]	0.821756223	0.001239241	0.005320844
ENSMMUG00000010626	CD1c molecule [Source:VGNC Symbol;Acc:VGNC:70756]	1.86743893	0.000615467	0.002898208
ENSMMUG00000003191	kirre like nephrin family adhesion molecule 1 [Source:VGNC Symbol;Acc:VGNC:73837]	-1.154454323	2.35E-14	7.77E-13
ENSMMUG00000019835	CD5 molecule like [Source:VGNC Symbol;Acc:VGNC:70774]	1.227197384	3.03E-07	3.06E-06
ENSMMUG00000019833	Fc receptor like 1 [Source:VGNC Symbol;Acc:VGNC:72528]	1.445883374	6.57E-24	6.14E-22

ENSMMUG00000019830	Fc receptor like 2 [Source:VGNC Symbol;Acc:VGNC:72509]	0.732436089	0.000457433	0.002228833
ENSMMUG00000046969	Fc receptor like 3 [Source:NCBI gene;Acc:718801]	1.503868409	5.99E-17	2.74E-15
ENSMMUG00000008974	Fc receptor like 5 [Source:NCBI gene;Acc:718771]	-2.680380841	4.06E-21	2.80E-19
ENSMMUG00000060011	hyaluronan and proteoglycan link protein 2 [Source:NCBI gene;Acc:718500]	-3.355041228	0.000145163	0.000801744
ENSMMUG00000016096	lamin A/C [Source:VGNC Symbol;Acc:VGNC:74427]	2.013606506	1.32E-05	9.28E-05
ENSMMUG00000004648	KH domain containing 4, pre-mRNA splicing factor [Source:VGNC Symbol;Acc:VGNC:73931]	-0.636684703	2.58E-05	0.000169333
ENSMMUG00000021312	synaptotagmin 11 [Source:VGNC Symbol;Acc:VGNC:78167]	-0.527401568	0.002276886	0.008994021
ENSMMUG00000062397		0.826896313	6.46E-05	0.00038581
ENSMMUG00000023146	ASH1 like histone lysine methyltransferase [Source:NCBI gene;Acc:717778]	1.623017097	0.009431688	0.030467764
ENSMMUG00000023145	RUN and SH3 domain containing 1 [Source:VGNC Symbol;Acc:VGNC:77036]	-0.865350619	0.015208418	0.045443776
ENSMMUG00000023141	CDC like kinase 2 [Source:VGNC Symbol;Acc:VGNC:71259]	-1.414628489	4.55E-06	3.60E-05
ENSMMUG00000016093	adenosine deaminase RNA specific [Source:VGNC Symbol;Acc:VGNC:69502]	-2.34709425	1.90E-12	4.72E-11
ENSMMUG00000010978	S100 calcium binding protein A2 [Source:VGNC Symbol;Acc:VGNC:76956]	-0.958801447	8.69E-11	1.67E-09
ENSMMUG00000031655	S100 calcium binding protein A3 [Source:VGNC Symbol;Acc:VGNC:76957]	-0.635096931	0.00060726	0.002871073
ENSMMUG00000008917	S100 calcium binding protein A6 [Source:VGNC Symbol;Acc:VGNC:76960]	-0.8181739	6.70E-07	6.29E-06
ENSMMUG00000051645		2.400978608	0.000276477	0.001430112
ENSMMUG00000042618	involucrin [Source:NCBI gene;Acc:713728]	1.413065968	9.43E-05	0.000541528
ENSMMUG00000017740	sperm mitochondria associated cysteine rich protein [Source:VGNC Symbol;Acc:VGNC:77816]	2.37709267	4.70E-08	5.58E-07
ENSMMUG00000055038		1.789443341	0.000617485	0.002905701
ENSMMUG00000054634	late cornified envelope protein 1C-like [Source:NCBI gene;Acc:713600]	2.089999248	0.01163412	0.036375757
ENSMMUG00000058472	late cornified envelope protein 1C [Source:NCBI gene;Acc:107000798]	1.548560176	0.000298718	0.001529919
ENSMMUG00000055266	late cornified envelope protein 1C-like [Source:NCBI gene;Acc:107000794]	2.193123043	4.11E-05	0.000257891
ENSMMUG00000051816	keratinocyte proline rich protein [Source:NCBI gene;Acc:100424312]	1.458600437	0.000227928	0.001206127
ENSMMUG00000001966	chromosome 1 C1orf68 homolog [Source:VGNC Symbol;Acc:VGNC:70436]	1.839836732	0.005758724	0.019929561
ENSMMUG00000049843	late cornified envelope protein 2D [Source:NCBI gene;Acc:100424118]	2.927867255	0.004151819	0.015126257
ENSMMUG00000062996		1.889633872	0.000309006	0.001572844
ENSMMUG00000001967	late cornified envelope 5A [Source:VGNC Symbol;Acc:VGNC:74061]	2.66245583	0.007429039	0.024863345
ENSMMUG00000009040	cornulin [Source:VGNC Symbol;Acc:VGNC:71397]	1.933033535	5.60E-05	0.000340439
ENSMMUG00000056511		1.80556472	1.06E-06	9.54E-06
ENSMMUG00000059472	filaggrin family member 2 [Source:HGNC Symbol;Acc:HGNC:33276]	1.232002928	4.42E-07	4.32E-06
ENSMMUG00000021386	aph-1 homolog A, gamma-secretase subunit [Source:VGNC Symbol;Acc:VGNC:69924]	-0.545999408	0.00110247	0.004801224
ENSMMUG00000021385	carbonic anhydrase 14 [Source:VGNC Symbol;Acc:VGNC:70489]	-0.528646448	0.000847587	0.003823
ENSMMUG00000013681	pleckstrin homology domain containing O1 [Source:VGNC Symbol;Acc:VGNC:76101]	-0.762093037	3.82E-05	0.000241765
ENSMMUG00000013673	vacuolar protein sorting 45 homolog [Source:VGNC Symbol;Acc:VGNC:78964]	-0.54983127	0.000497325	0.002399031
ENSMMUG00000007244	OTU deubiquitinase 7B [Source:VGNC Symbol;Acc:VGNC:75634]	-0.881032418	2.79E-05	0.000181517
ENSMMUG00000052282		-0.818492209	0.000135807	0.000753332
ENSMMUG00000007246	myotubularin related protein 11 [Source:VGNC Symbol;Acc:VGNC:75016]	-0.866539653	9.51E-06	6.94E-05
ENSMMUG00000007256	splicing factor 3b subunit 4 [Source:VGNC Symbol;Acc:VGNC:77182]	-1.173337979	2.62E-08	3.26E-07

ENSMMUG00000007257	synaptic vesicle glycoprotein 2A [Source:VGNC Symbol;Acc:VGNC:78181]	-0.904103023	1.71E-10	3.13E-09
ENSMMUG00000052382	histone H4 [Source:NCBI gene;Acc:107000818]	-0.98976725	2.48E-10	4.40E-09
ENSMMUG00000054615		-1.598030154	1.39E-07	1.50E-06
ENSMMUG00000051523	Metazoan signal recognition particle RNA [Source:RFAM;Acc:RF00017]	-1.661977416	0.000489869	0.002367954
ENSMMUG00000040506	H2B clustered histone 18 [Source:HGNC Symbol;Acc:HGNC:24700]	-1.036110365	0.000161294	0.000881114
ENSMMUG00000004362	Fc fragment of IgG receptor Ia [Source:NCBI gene;Acc:714664]	-0.844756502	0.000438607	0.002146052
ENSMMUG00000062140		-1.430513747	1.76E-06	1.50E-05
ENSMMUG00000024186	U1 spliceosomal RNA [Source:RFAM;Acc:RF00003]	-1.579961173	0.000807453	0.003675079
ENSMMUG00000036857	U1 spliceosomal RNA [Source:RFAM;Acc:RF00003]	-1.423796658	0.005165139	0.018194108
ENSMMUG00000064626	U1 spliceosomal RNA [Source:RFAM;Acc:RF00003]	-1.210467304	0.015532921	0.046314725
ENSMMUG00000061239		-1.288013322	1.15E-06	1.02E-05
ENSMMUG00000054590		-1.142587843	2.21E-05	0.000147963
ENSMMUG00000054496	U1 spliceosomal RNA [Source:RFAM;Acc:RF00003]	-1.764235674	0.002262511	0.008951257
ENSMMUG00000048493	neuroblastoma breakpoint family member 6-like protein [Source:NCBI gene;Acc:711999]	-2.640179907	0.013787574	0.041967258
ENSMMUG00000018375	notch receptor 2 [Source:NCBI gene;Acc:713798]	-0.574468703	3.44E-05	0.000220178
ENSMMUG00000001978	adenosine monophosphate deaminase 1 [Source:VGNC Symbol;Acc:VGNC:69806]	-0.615037916	0.003777426	0.013910174
ENSMMUG00000019528	adaptor related protein complex 4 subunit beta 1 [Source:VGNC Symbol;Acc:VGNC:69972]	2.411960236	4.32E-42	2.15E-39
ENSMMUG00000019527	DNA cross-link repair 1B [Source:VGNC Symbol;Acc:VGNC:71647]	1.179049911	3.17E-05	0.000204488
ENSMMUG00000006786	protein tyrosine phosphatase non-receptor type 22 [Source:VGNC Symbol;Acc:VGNC:76603]	1.161337085	2.65E-09	3.92E-08
ENSMMUG00000017819	putative homeodomain transcription factor 1 [Source:VGNC Symbol;Acc:VGNC:75982]	-0.872113398	0.000224775	0.001191239
ENSMMUG00000007766	suppression of tumorigenicity 7 like [Source:VGNC Symbol;Acc:VGNC:77954]	-0.913654557	7.43E-05	0.000437114
ENSMMUG00000007765	Wnt family member 2B [Source:VGNC Symbol;Acc:VGNC:78797]	-0.706170553	9.13E-06	6.70E-05
ENSMMUG00000037458	endoplasmic reticulum resident protein 29 [Source:NCBI gene;Acc:720396]	-1.29224899	0.011495102	0.03600532
ENSMMUG00000050825	potassium voltage-gated channel subfamily D member 3 [Source:NCBI gene;Acc:641437]	-0.581897406	1.32E-05	9.32E-05
ENSMMUG00000065322	ATP synthase peripheral stalk-membrane subunit b [Source:NCBI gene;Acc:704072]	-0.711511708	5.87E-06	4.53E-05
ENSMMUG00000016056	potassium voltage-gated channel subfamily A member 10 [Source:VGNC Symbol;Acc:VGNC:73963]	0.542658748	0.000151626	0.000834149
ENSMMUG00000062177		0.370544977	0.004147719	0.015115245
ENSMMUG00000004614	potassium voltage-gated channel subfamily C member 4 [Source:VGNC Symbol;Acc:VGNC:73974]	-1.122778232	0.001041709	0.00456925
ENSMMUG00000021544	solute carrier family 6 member 17 [Source:VGNC Symbol;Acc:VGNC:77805]	-1.404952873	0.000513533	0.002468725
ENSMMUG00000005426	striatin interacting protein 1 [Source:VGNC Symbol;Acc:VGNC:78217]	-0.45232785	0.003097003	0.011696545
ENSMMUG00000022715	adenosylhomocysteinase like 1 [Source:VGNC Symbol;Acc:VGNC:81350]	-0.704581362	1.15E-05	8.26E-05
ENSMMUG00000020851	glutathione S-transferase mu 5 [Source:NCBI gene;Acc:100426406]	-0.690438621	0.004025557	0.014712117

ENSMMUG00000051186	proteasome 20S subunit alpha 5 [Source:NCBI gene;Acc:698435]	0.716615667	3.04E-10	5.31E-09
ENSMMUG00000008713	myosin binding protein H like [Source:NCBI gene;Acc:698183]	- 1.005852524	7.08E-09	9.74E-08
ENSMMUG00000015380	proline and serine rich coiled-coil 1 [Source:VGNC Symbol;Acc:VGNC:76307]	-1.07765419	1.62E-10	2.97E-09
ENSMMUG00000015375	cadherin EGF LAG seven-pass G-type receptor 2 [Source:VGNC Symbol;Acc:VGNC:71149]	- 1.002079108	5.39E-13	1.46E-11
ENSMMUG00000021837	seryl-tRNA synthetase 1 [Source:NCBI gene;Acc:695046]	- 0.903583689	7.34E-09	1.00E-07
ENSMMUG00000010156	fibronectin type III domain containing 7 [Source:VGNC Symbol;Acc:VGNC:72582]	0.464185149	0.009643336	0.030973089
ENSMMUG00000031785		1.637983349	1.21E-10	2.27E-09
ENSMMUG00000002789	solute carrier family 25 (mitochondrial carrier; phosphate carrier), member 24 [Source:NCBI gene;Acc:694803]	1.669604217	1.17E-06	1.04E-05
ENSMMUG00000008197	netrin G1 [Source:VGNC Symbol;Acc:VGNC:75450]	1.81709034	0.00028165	0.001452583
ENSMMUG00000060795		2.204263791	2.22E-10	3.97E-09
ENSMMUG00000061766		- 2.173521615	6.97E-05	0.000412958
ENSMMUG00000004103	collagen type XI alpha 1 chain [Source:VGNC Symbol;Acc:VGNC:71289]	1.752803576	5.55E-57	8.65E-54
ENSMMUG00000060790		- 2.591062636	0.002945683	0.011233971
ENSMMUG00000022906	vascular cell adhesion molecule 1 [Source:VGNC Symbol;Acc:VGNC:78761]	- 0.668991436	7.31E-06	5.51E-05
ENSMMUG00000019106	cell division cycle 14A [Source:VGNC Symbol;Acc:VGNC:70791]	- 0.458779579	0.003008731	0.011437093
ENSMMUG00000019105	RNA 3'-terminal phosphate cyclase [Source:VGNC Symbol;Acc:VGNC:77043]	- 2.005092117	9.69E-06	7.06E-05
ENSMMUG00000007706	dihydrolipoamide branched chain transacylase E2 [Source:VGNC Symbol;Acc:VGNC:71744]	- 2.060063666	0.006143112	0.021076139
ENSMMUG00000000931	solute carrier family 35 member A3 [Source:VGNC Symbol;Acc:VGNC:77461]	1.279531129	0.004415194	0.015946011
ENSMMUG00000038667		- 1.500687894	5.84E-05	0.00035379
ENSMMUG00000008518	phospholipid phosphatase related 5 [Source:NCBI gene;Acc:710549]	1.227966972	5.28E-12	1.24E-10
ENSMMUG00000008515	sorting nexin 7 [Source:NCBI gene;Acc:716015]	0.831017863	3.50E-19	2.00E-17
ENSMMUG00000049494		1.68605598	5.39E-07	5.17E-06
ENSMMUG00000020726	dihydropyrimidine dehydrogenase [Source:VGNC Symbol;Acc:VGNC:71982]	1.932904145	4.55E-16	1.89E-14
ENSMMUG00000049297		0.971821538	2.78E-10	4.89E-09
ENSMMUG00000050661		0.531334061	0.005081423	0.017935261
ENSMMUG00000001690	calponin 3 [Source:VGNC Symbol;Acc:VGNC:71278]	0.61236284	0.000966226	0.004274255
ENSMMUG00000056548	coagulation factor III, tissue factor [Source:NCBI gene;Acc:714456]	1.030892764	1.09E-19	6.48E-18
ENSMMUG00000015924	BCAR3 adaptor protein, NSP family member [Source:VGNC Symbol;Acc:VGNC:70235]	0.943360889	4.11E-07	4.05E-06
ENSMMUG00000015922	formin binding protein 1 like [Source:VGNC Symbol;Acc:VGNC:72543]	0.651693909	3.82E-06	3.07E-05
ENSMMUG00000016812	coiled-coil domain containing 18 [Source:VGNC Symbol;Acc:VGNC:70913]	- 0.944804581	4.04E-05	0.000254283
ENSMMUG00000005070	ecotropic viral integration site 5 [Source:NCBI gene;Acc:706592]	2.056870021	2.80E-21	1.96E-19
ENSMMUG00000024513	Y RNA [Source:RFAM;Acc:RF00019]	3.35570914	0.014116001	0.042818231
ENSMMUG00000029649	ATP synthase, H+ transporting, mitochondrial Fo complex, subunit C3 (subunit 9)-like [Source:NCBI gene;Acc:706691]	4.140627814	0.00015802	0.000865591
ENSMMUG00000014018	bromodomain testis associated [Source:VGNC Symbol;Acc:VGNC:70282]	- 3.382555621	6.53E-07	6.17E-06
ENSMMUG00000001742	transforming growth factor beta receptor 3 [Source:VGNC Symbol;Acc:VGNC:78339]	-3.58063551	1.66E-39	7.05E-37
ENSMMUG00000051155	U2 spliceosomal RNA [Source:RFAM;Acc:RF00004]	- 4.586191118	2.59E-05	0.000169828
ENSMMUG00000006819	helicase for meiosis 1 [Source:NCBI gene;Acc:704516]	0.640580746	0.004320129	0.015678358
ENSMMUG00000007635	zinc finger protein 644 [Source:VGNC Symbol;Acc:VGNC:79789]	1.895695867	0.000293596	0.00150589
ENSMMUG00000049678		2.385322428	1.70E-16	7.32E-15
ENSMMUG00000060600		-0.76344972	0.001077233	0.004705932

ENSMMUG00000000663	guanylate-binding protein 6-like [Source:NCBI gene;Acc:695158]	-0.867315361	0.000842711	0.003807133
ENSMMUG00000000662	guanylate-binding protein 1 [Source:NCBI gene;Acc:694538]	-0.736267462	1.57E-07	1.68E-06
ENSMMUG00000037887	RBMX like 1 [Source:NCBI gene;Acc:100424767]	0.741289351	4.29E-09	6.19E-08
ENSMMUG00000002794	general transcription factor IIB [Source:VGNC Symbol;Acc:VGNC:73306]	-1.824019353	1.32E-27	1.89E-25
ENSMMUG00000020588	protein kinase N2 [Source:VGNC Symbol;Acc:VGNC:76015]	0.688518477	4.16E-08	4.98E-07
ENSMMUG00000065295		2.221687825	1.35E-30	2.55E-28
ENSMMUG00000060366		2.776651841	0.016702611	0.049123473
ENSMMUG00000016554	selenoprotein F [Source:NCBI gene;Acc:712469]	-0.649178448	0.016566422	0.048784319
ENSMMUG00000010306	calcium-activated chloride channel regulator 4 [Source:NCBI gene;Acc:712123]	-1.00169387	0.009441636	0.030492873
ENSMMUG00000006836	chloride channel accessory 1 [Source:NCBI gene;Acc:574295]	1.439187414	5.18E-06	4.03E-05
ENSMMUG00000001380	outer dense fiber of sperm tails 2 like [Source:VGNC Symbol;Acc:VGNC:75482]	2.034462587	0.00032636	0.001648016
ENSMMUG00000014573	dimethylarginine dimethylaminohydrolase 1 [Source:VGNC Symbol;Acc:VGNC:71753]	0.926941347	5.20E-14	1.64E-12
ENSMMUG00000005361	mucolipin 3 [Source:VGNC Symbol;Acc:VGNC:74505]	0.763906224	0.000169684	0.000922281
ENSMMUG00000010127	chitobiase [Source:VGNC Symbol;Acc:VGNC:71540]	-1.813872112	5.47E-08	6.40E-07
ENSMMUG00000022183	spermatogenesis associated 1 [Source:NCBI gene;Acc:710462]	-1.797212105	2.51E-21	1.78E-19
ENSMMUG00000059938		-1.747762355	5.26E-05	0.000321813
ENSMMUG00000000404	uricase [Source:NCBI gene;Acc:714004]	-1.314758103	0.001741636	0.007124551
ENSMMUG00000003457	protein kinase cAMP-activated catalytic subunit beta [Source:NCBI gene;Acc:709728]	1.014493129	1.61E-12	4.04E-11
ENSMMUG00000057763		-1.689729849	5.93E-05	0.000358257
ENSMMUG00000023345	adhesion G protein-coupled receptor L4 [Source:VGNC Symbol;Acc:VGNC:69652]	1.361298683	5.88E-05	0.00035559
ENSMMUG00000049326	GIPC PDZ domain containing family member 2 [Source:NCBI gene;Acc:707432]	-0.556258841	0.009233924	0.029925426
ENSMMUG00000050425	DnaJ heat shock protein family (Hsp40) member B4 [Source:NCBI gene;Acc:707206]	-0.535843154	0.00041191	0.002027452
ENSMMUG00000041837	Metazoan signal recognition particle RNA [Source:RFAM;Acc:RF00017]	-1.192891886	0.007715138	0.025722629
ENSMMUG00000056103	growth arrest and DNA damage inducible alpha [Source:NCBI gene;Acc:701054]	-1.729584467	1.53E-31	3.29E-29
ENSMMUG00000003265	leptin receptor [Source:VGNC Symbol;Acc:VGNC:81429]	0.690059679	2.10E-12	5.19E-11
ENSMMUG00000049908	Metazoan signal recognition particle RNA [Source:RFAM;Acc:RF00017]	-2.127152851	0.000267337	0.001389498
ENSMMUG00000003264	leptin receptor overlapping transcript [Source:VGNC Symbol;Acc:VGNC:74158]	-0.864430504	0.000159522	0.000873477
ENSMMUG00000017259	DnaJ heat shock protein family (Hsp40) member C6 [Source:NCBI gene;Acc:698682]	-1.341805964	8.31E-08	9.46E-07
ENSMMUG00000053254	adenylate kinase 4 [Source:NCBI gene;Acc:698560]	0.735703359	1.94E-05	0.000131878
ENSMMUG00000053072		0.800377331	8.49E-05	0.000492448
ENSMMUG00000018566	Janus kinase 1 [Source:VGNC Symbol;Acc:VGNC:73810]	-0.941512921	0.000475434	0.002305327
ENSMMUG00000018565	ribonucleoprotein, PTB binding 2 [Source:VGNC Symbol;Acc:VGNC:76536]	-2.494865186	5.11E-06	3.98E-05
ENSMMUG00000012538	ubiquitin conjugating enzyme E2 U [Source:VGNC Symbol;Acc:VGNC:79726]	0.86928417	3.51E-05	0.000224118
ENSMMUG00000004668	receptor tyrosine kinase like orphan receptor 1 [Source:VGNC Symbol;Acc:VGNC:76715]	0.653861501	0.009292767	0.03008831
ENSMMUG00000020049	phosphoglucomutase 1 [Source:NCBI gene;Acc:699401]	-1.01184368	1.96E-16	8.38E-15
ENSMMUG00000020047	integrin subunit beta 3 binding protein [Source:VGNC Symbol;Acc:VGNC:73787]	-1.299619762	1.26E-15	4.86E-14

ENSMMUG00000010351	KN motif and ankyrin repeat domains 4 [Source:VGNC Symbol;Acc:VGNC:73916]	-0.744859774	3.61E-05	0.000229716
ENSMMUG00000049952		0.481630661	0.003070904	0.011619882
ENSMMUG00000013840	nuclear factor I A [Source:VGNC Symbol;Acc:VGNC:75334]	0.451479243	0.012386499	0.038259481
ENSMMUG00000055834		1.287350842	0.010749675	0.034004667
ENSMMUG00000022762	OMA1 zinc metallopeptidase [Source:VGNC Symbol;Acc:VGNC:75485]	-2.420285541	4.91E-14	1.56E-12
ENSMMUG00000010406	protein kinase AMP-activated catalytic subunit alpha 2 [Source:VGNC Symbol;Acc:VGNC:76400]	-3.397415153	0.000550609	0.002629834
ENSMMUG00000002200	phospholipid phosphatase 3 [Source:NCBI gene;Acc:715993]	-1.706209604	8.87E-26	1.05E-23
ENSMMUG00000059612		0.485265154	2.55E-09	3.80E-08
ENSMMUG00000005740	ubiquitin specific peptidase 24 [Source:VGNC Symbol;Acc:VGNC:78743]	-0.488556796	0.000336991	0.001696814
ENSMMUG00000032084	prolyl-tRNA synthetase 2, mitochondrial [Source:VGNC Symbol;Acc:VGNC:75707]	-0.793346324	3.35E-06	2.71E-05
ENSMMUG00000006754	tetratricopeptide repeat domain 4 [Source:VGNC Symbol;Acc:VGNC:78668]	-0.897976974	9.93E-06	7.21E-05
ENSMMUG00000009114	maestro heat like repeat family member 7 [Source:NCBI gene;Acc:715310]	-1.381791216	1.16E-05	8.32E-05
ENSMMUG00000008828	BEN domain containing 5 [Source:NCBI gene;Acc:711150]	1.913247688	1.94E-17	9.30E-16
ENSMMUG00000015371	microtubule associated serine/threonine kinase 2 [Source:VGNC Symbol;Acc:VGNC:74532]	-0.896240096	4.21E-05	0.000263677
ENSMMUG00000005986	Kruppel like factor 17 [Source:NCBI gene;Acc:702810]	1.171943395	0.007984551	0.026482345
ENSMMUG00000015555	ST3 beta-galactoside alpha-2,3-sialyltransferase 3 [Source:VGNC Symbol;Acc:VGNC:77828]	1.551312987	4.77E-06	3.75E-05
ENSMMUG00000012479	lysine demethylase 4A [Source:NCBI gene;Acc:701272]	-1.893298133	0.01420443	0.043039915
ENSMMUG00000012477	protein tyrosine phosphatase receptor type F [Source:VGNC Symbol;Acc:VGNC:76615]	-0.447586836	0.000312688	0.001589858
ENSMMUG00000016111	chromosome 1 C1orf50 homolog [Source:VGNC Symbol;Acc:VGNC:70432]	1.589649704	0.009204028	0.029849234
ENSMMUG00000022381	prolyl 3-hydroxylase 1 [Source:VGNC Symbol;Acc:VGNC:75648]	1.984886786	0.000671711	0.003116944
ENSMMUG00000057076	claudin 19 [Source:NCBI gene;Acc:697807]	4.16333663	0.000353988	0.00176591
ENSMMUG00000013129	peptidylprolyl isomerase H [Source:NCBI gene;Acc:697669]	2.039622395	4.74E-05	0.000292566
ENSMMUG00000013128	coiled-coil domain containing 30 [Source:VGNC Symbol;Acc:VGNC:70866]	0.57705398	3.22E-07	3.24E-06
ENSMMUG00000001034	zinc finger MYND-type containing 12 [Source:VGNC Symbol;Acc:VGNC:79312]	-0.561510625	0.000493981	0.002385364
ENSMMUG00000014138	forkhead box J3 [Source:VGNC Symbol;Acc:VGNC:72596]	1.269189916	0.0001121	0.000632842
ENSMMUG00000052046		0.3877727	0.010821652	0.034186084
ENSMMUG00000044175	forkhead box O6 [Source:NCBI gene;Acc:697505]	-1.231910214	8.65E-13	2.25E-11
ENSMMUG00000043675	Cbp/p300 interacting transactivator with Glu/Asp rich carboxy-terminal domain 4 [Source:NCBI gene;Acc:114672840]	2.114494734	7.75E-26	9.29E-24
ENSMMUG00000019536	potassium voltage-gated channel subfamily Q member 4 [Source:VGNC Symbol;Acc:VGNC:74007]	0.881119288	6.45E-10	1.06E-08
ENSMMUG00000018664	collagen type IX alpha 2 chain [Source:VGNC Symbol;Acc:VGNC:71307]	1.931816103	7.45E-15	2.63E-13
ENSMMUG00000005992	zinc metallopeptidase STE24 [Source:VGNC Symbol;Acc:VGNC:78830]	1.948504122	5.90E-07	5.62E-06
ENSMMUG00000052510	transmembrane and coiled-coil domains 2 [Source:NCBI gene;Acc:693977]	2.458864707	0.009542239	0.030752274
ENSMMUG00000032180	RLF zinc finger [Source:VGNC Symbol;Acc:VGNC:76699]	1.597100385	2.90E-07	2.94E-06
ENSMMUG00000009189	palmitoyl-protein thioesterase 1 [Source:VGNC Symbol;Acc:VGNC:76386]	-0.582290837	2.10E-09	3.18E-08
ENSMMUG00000009188	cyclase associated actin cytoskeleton regulatory protein 1 [Source:NCBI gene;Acc:693719]	-0.532508062	2.71E-07	2.77E-06
ENSMMUG00000016220	tRNA isopentenyltransferase 1 [Source:VGNC Symbol;Acc:VGNC:79239]	-1.517422407	0.014897832	0.044706247
ENSMMUG00000058881	bone morphogenetic protein 8b [Source:NCBI gene;Acc:100427030]	1.070149237	0.002874575	0.011001683
ENSMMUG00000052910		0.580722629	0.015691455	0.046727815

ENSMMUG00000061825	U6 spliceosomal RNA [Source:RFAM;Acc:RF00026]	0.538933301	0.015051685	0.045061865
ENSMMUG00000059855	Smad nuclear interacting protein 1 [Source:NCBI gene;Acc:713733]	-0.890464708	0.001920565	0.007786088
ENSMMUG00000010328	MYST/Esa1 associated factor 6 [Source:VGNC Symbol;Acc:VGNC:74580]	-1.135761678	0.012566146	0.038754137
ENSMMUG00000010323	zinc finger CCCH-type containing 12A [Source:VGNC Symbol;Acc:VGNC:79595]	-0.927940411	0.001258257	0.005387647
ENSMMUG00000006252	argonaute RISC catalytic component 3 [Source:NCBI gene;Acc:712543]	-1.967411636	9.06E-23	7.47E-21
ENSMMUG00000006251	argonaute RISC component 1 [Source:VGNC Symbol;Acc:VGNC:81346]	-1.400801898	1.97E-10	3.56E-09
ENSMMUG00000019815	transcription factor AP-2 epsilon [Source:VGNC Symbol;Acc:VGNC:79878]	-1.610512166	0.000160126	0.000876101
ENSMMUG00000002741	CUB and Sushi multiple domains 2 [Source:VGNC Symbol;Acc:VGNC:71441]	2.887059131	0.004866336	0.017333155
ENSMMUG00000003267	zinc finger protein 362 [Source:NCBI gene;Acc:710013]	0.686789466	1.25E-05	8.86E-05
ENSMMUG00000015097	tripartite motif containing 62 [Source:VGNC Symbol;Acc:VGNC:79513]	0.943970382	5.70E-07	5.44E-06
ENSMMUG00000006285	antizyme inhibitor 2 [Source:VGNC Symbol;Acc:VGNC:70153]	1.264812142	4.85E-22	3.65E-20
ENSMMUG00000006284	adenylate kinase 2 [Source:VGNC Symbol;Acc:VGNC:69761]	0.637507289	2.22E-06	1.86E-05
ENSMMUG00000055316		-3.694635527	0.001432219	0.006016779
ENSMMUG00000001264	BSD domain containing 1 [Source:VGNC Symbol;Acc:VGNC:70384]	-4.364398473	2.32E-07	2.40E-06
ENSMMUG00000053323		-3.069754191	0.00293638	0.011207637
ENSMMUG00000009951	MARCKS like 1 [Source:VGNC Symbol;Acc:VGNC:74723]	-3.4634967	0.003100042	0.011701974
ENSMMUG00000009948	histone deacetylase 1 [Source:NCBI gene;Acc:708441]	-2.483076349	2.99E-21	2.08E-19
ENSMMUG00000040694	LCK proto-oncogene, Src family tyrosine kinase [Source:NCBI gene;Acc:717704]	-2.086642019	2.01E-50	2.01E-47
ENSMMUG00000060894	Metazoan signal recognition particle RNA [Source:RFAM;Acc:RF00017]	-1.160710343	4.05E-06	3.24E-05
ENSMMUG00000041790	Metazoan signal recognition particle RNA [Source:RFAM;Acc:RF00017]	-0.731916984	0.001987339	0.00801048
ENSMMUG00000046901	protein tyrosine phosphatase 4A2 [Source:NCBI gene;Acc:706489]	-1.172762193	1.09E-07	1.20E-06
ENSMMUG00000005466	SPOC domain containing 1 [Source:VGNC Symbol;Acc:VGNC:77997]	-0.680500855	0.001353842	0.005729189
ENSMMUG00000023401	adhesion G protein-coupled receptor B2 [Source:VGNC Symbol;Acc:VGNC:69598]	-0.958698505	3.21E-05	0.000206323
ENSMMUG00000023398	collagen type XVI alpha 1 chain [Source:VGNC Symbol;Acc:VGNC:71293]	-0.927635816	6.63E-07	6.23E-06
ENSMMUG00000023397	penta-EF-hand domain containing 1 [Source:NCBI gene;Acc:706038]	-0.642593549	0.000108578	0.000614939
ENSMMUG00000023396	hypocretin receptor 1 [Source:VGNC Symbol;Acc:VGNC:73356]	-0.565541811	0.009043237	0.029415006
ENSMMUG00000060319	Metazoan signal recognition particle RNA [Source:RFAM;Acc:RF00017]	-1.141149466	0.00010322	0.000587739
ENSMMUG00000013188	serine incorporator 2 [Source:VGNC Symbol;Acc:VGNC:77294]	-0.684241332	0.000167729	0.000912716
ENSMMUG00000049574	antizyme inhibitor 2-like [Source:NCBI gene;Acc:705256]	-0.556085239	0.00225633	0.008935474
ENSMMUG00000012156	zinc finger CCHC-type containing 17 [Source:VGNC Symbol;Acc:VGNC:79840]	-1.217510335	2.52E-20	1.62E-18
ENSMMUG00000064353	fatty acid binding protein 3 [Source:NCBI gene;Acc:717237]	-0.549237388	0.001998779	0.008054274
ENSMMUG00000013520	pumilio RNA binding family member 1 [Source:VGNC Symbol;Acc:VGNC:76479]	0.360939042	0.000268695	0.001394491

ENSMMUG00000063979		-0.732640362	1.19E-12	3.05E-11
ENSMMUG00000013519	syndecan 3 [Source:VGNC Symbol;Acc:VGNC:76999]	-0.352239877	0.003265661	0.012232623
ENSMMUG00000057452		1.072571948	0.000862988	0.003877501
ENSMMUG00000057173		-3.001074757	0.000184733	0.00099712
ENSMMUG00000015661	protein tyrosine phosphatase receptor type U [Source:VGNC Symbol;Acc:VGNC:76624]	-1.828227497	4.45E-06	3.52E-05
ENSMMUG00000007110	serine and arginine rich splicing factor 4 [Source:NCBI gene;Acc:717401]	-2.233328793	0.000800965	0.003652662
ENSMMUG00000016893	solute carrier family 9 member A1 [Source:VGNC Symbol;Acc:VGNC:82241]	-0.809114429	7.46E-06	5.61E-05
ENSMMUG00000015115	terminal nucleotidyltransferase 5B [Source:VGNC Symbol;Acc:VGNC:79692]	0.711452848	1.36E-05	9.55E-05
ENSMMUG00000021031	TMF1 regulated nuclear protein 1 [Source:VGNC Symbol;Acc:VGNC:79946]	0.890833678	2.31E-09	3.47E-08
ENSMMUG00000058432	zinc finger DHHC-type palmitoyltransferase 18 [Source:NCBI gene;Acc:719959]	1.235794207	4.18E-08	5.00E-07
ENSMMUG00000012697	AT-rich interaction domain 1A [Source:VGNC Symbol;Acc:VGNC:69822]	1.859995941	3.16E-12	7.61E-11
ENSMMUG00000056266	Metazoan signal recognition particle RNA [Source:RFAM;Acc:RF00017]	1.62841358	2.61E-07	2.68E-06
ENSMMUG00000000649	UBX domain protein 11 [Source:VGNC Symbol;Acc:VGNC:79859]	1.960326547	4.73E-08	5.60E-07
ENSMMUG00000000644	centrosomal protein 85 [Source:VGNC Symbol;Acc:VGNC:71096]	2.095582082	5.72E-11	1.13E-09
ENSMMUG00000032217	transmembrane protein 50A [Source:VGNC Symbol;Acc:VGNC:79857]	0.377547007	0.006144139	0.021076139
ENSMMUG00000002420	RUNX family transcription factor 3 [Source:VGNC Symbol;Acc:VGNC:76948]	-1.118147159	0.010207547	0.032537587
ENSMMUG00000063165		3.223516837	0.011690236	0.036494251
ENSMMUG00000050770		-1.009934659	4.68E-06	3.69E-05
ENSMMUG00000004216	alpha-L-fucosidase 1 [Source:VGNC Symbol;Acc:VGNC:72826]	-0.988242522	0.001711049	0.007021961
ENSMMUG00000045584	leucine zipper protein 1 [Source:NCBI gene;Acc:718626]	-1.540376329	3.24E-16	1.37E-14
ENSMMUG00000009773	lysine demethylase 1A [Source:VGNC Symbol;Acc:VGNC:73928]	-1.942864657	1.53E-15	5.84E-14
ENSMMUG00000058332	testis expressed 46 [Source:NCBI gene;Acc:709002]	-1.454290439	0.000275049	0.001423776
ENSMMUG00000041482	lactamase beta like 1 [Source:NCBI gene;Acc:718590]	-0.871377603	0.001714818	0.007032868
ENSMMUG00000002217	alkaline phosphatase, biomineralization associated [Source:VGNC Symbol;Acc:VGNC:69801]	1.433437502	4.40E-31	9.35E-29
ENSMMUG00000004149	eukaryotic translation initiation factor 4 gamma 3 [Source:NCBI gene;Acc:717555]	1.251253056	1.14E-23	1.04E-21
ENSMMUG00000006688	ER membrane protein complex subunit 1 [Source:VGNC Symbol;Acc:VGNC:72134]	-0.874413026	6.62E-09	9.18E-08
ENSMMUG00000006669	ubiquitin protein ligase E3 component n-recognin 4 [Source:VGNC Symbol;Acc:VGNC:79845]	-0.927202428	4.42E-10	7.48E-09
ENSMMUG00000052628	Metazoan signal recognition particle RNA [Source:RFAM;Acc:RF00017]	-2.602004134	0.010957191	0.034551987
ENSMMUG00000050018		-0.634049541	0.001899583	0.007709943
ENSMMUG00000004754	intermediate filament family orphan 2 [Source:NCBI gene;Acc:106995162]	-1.049304704	1.02E-06	9.17E-06
ENSMMUG00000064850	kelch domain containing 7A [Source:NCBI gene;Acc:713771]	-0.816437356	0.012444471	0.038404229
ENSMMUG00000010849	Rho guanine nucleotide exchange factor 10 like [Source:VGNC Symbol;Acc:VGNC:69908]	-0.524049215	8.27E-06	6.13E-05
ENSMMUG00000022437	peptidyl arginine deiminase 6 [Source:VGNC Symbol;Acc:VGNC:75657]	4.063253607	2.50E-05	0.000165047
ENSMMUG00000051619	peptidyl arginine deiminase 3 [Source:NCBI gene;Acc:700560]	0.437142477	0.002472481	0.009679396
ENSMMUG00000001616	peptidyl arginine deiminase 2 [Source:VGNC Symbol;Acc:VGNC:75644]	1.129484159	0.000137466	0.00076193

ENSMMUG00000018245	EPH receptor A2 [Source:VGNC Symbol;Acc:VGNC:72235]	1.562360635	1.91E-15	7.23E-14
ENSMMUG00000058587	family with sequence similarity 131 member C [Source:NCBI gene;Acc:703604]	1.611396183	4.34E-20	2.66E-18
ENSMMUG00000018235	chloride voltage-gated channel Kb [Source:NCBI gene;Acc:703476]	1.382002107	1.73E-06	1.48E-05
ENSMMUG00000001910	heat shock protein family B (small) member 7 [Source:NCBI gene;Acc:696704]	1.60076285	1.23E-06	1.09E-05
ENSMMUG00000001911	steroid receptor associated and regulated protein [Source:VGNC Symbol;Acc:VGNC:78008]	1.429213871	5.52E-09	7.79E-08
ENSMMUG00000008031	zinc finger and BTB domain containing 17 [Source:VGNC Symbol;Acc:VGNC:78849]	1.483701842	5.35E-17	2.47E-15
ENSMMUG00000002802	agmatinase [Source:NCBI gene;Acc:713190]	-0.850191686	0.011701297	0.036512524
ENSMMUG00000032244	DnaJ heat shock protein family (Hsp40) member C16 [Source:NCBI gene;Acc:700894]	-0.91298711	0.016124435	0.047783802
ENSMMUG00000018163	caspase 9 [Source:NCBI gene;Acc:694544]	-2.901695265	0.000561849	0.002676223
ENSMMUG00000031821	chymotrypsin-like elastase family member 2B [Source:NCBI gene;Acc:106992406]	-1.256823169	0.009933447	0.031788391
ENSMMUG00000053550		-2.10096705	0.00263024	0.010200255
ENSMMUG00000060541		-1.822012441	0.001557519	0.00645995
ENSMMUG00000053235		-1.516513922	0.002489271	0.009720705
ENSMMUG00000003987	forkhead associated phosphopeptide binding domain 1 [Source:NCBI gene;Acc:699900]	-1.522963023	9.37E-06	6.85E-05
ENSMMUG00000055233	PRAME family member 17-like [Source:NCBI gene;Acc:100427847]	-0.702394263	1.13E-05	8.14E-05
ENSMMUG00000054309	PRAME family member 5 [Source:NCBI gene;Acc:722781]	-1.372804899	0.006501217	0.02213318
ENSMMUG00000057550		-0.89562168	0.000690846	0.003197731
ENSMMUG00000057874	arylacetamide deacetylase like 3 [Source:NCBI gene;Acc:722778]	-0.545130891	0.003725377	0.013762108
ENSMMUG00000021449	vacuolar protein sorting 13 homolog D [Source:NCBI gene;Acc:722775]	0.992740088	5.22E-14	1.64E-12
ENSMMUG00000064242		0.876705962	0.001312113	0.005582453
ENSMMUG00000021447	TNF receptor superfamily member 1B [Source:VGNC Symbol;Acc:VGNC:78590]	4.866520003	2.18E-05	0.000146558
ENSMMUG00000023449	mitofusin 2 [Source:VGNC Symbol;Acc:VGNC:74522]	1.880959527	6.53E-07	6.17E-06
ENSMMUG00000032249	procollagen-lysine,2-oxoglutarate 5-dioxygenase 1 [Source:NCBI gene;Acc:722763]	1.10503226	3.57E-11	7.25E-10
ENSMMUG00000012469	KIAA2013 [Source:VGNC Symbol;Acc:VGNC:73918]	0.805486751	1.20E-11	2.65E-10
ENSMMUG00000062316	Metazoan signal recognition particle RNA [Source:RFAM;Acc:RF00017]	1.028018708	1.90E-08	2.44E-07
ENSMMUG00000037413	U5 spliceosomal RNA [Source:RFAM;Acc:RF00020]	0.843560069	0.000324913	0.001643078
ENSMMUG00000051669		0.539230114	0.000139687	0.00077363
ENSMMUG00000055655	natriuretic peptide B [Source:NCBI gene;Acc:715053]	0.748415071	0.009885748	0.031650192
ENSMMUG00000049196	dispatched RND transporter family member 3 [Source:NCBI gene;Acc:722727]	-1.261477899	2.52E-14	8.30E-13
ENSMMUG00000032252	UbiA prenyltransferase domain containing 1 [Source:VGNC Symbol;Acc:VGNC:79881]	-1.291775684	6.19E-12	1.43E-10
ENSMMUG00000019325	mechanistic target of rapamycin kinase [Source:VGNC Symbol;Acc:VGNC:75062]	-1.410441552	1.10E-10	2.09E-09
ENSMMUG00000053062	U6 spliceosomal RNA [Source:RFAM;Acc:RF00026]	-2.056280235	0.001223902	0.005263042
ENSMMUG00000006187	angiopoietin like 7 [Source:VGNC Symbol;Acc:VGNC:69683]	-1.161948262	4.54E-09	6.54E-08
ENSMMUG00000062294		3.337053783	0.001484086	0.006203083
ENSMMUG00000058771	PHD finger protein 13 [Source:NCBI gene;Acc:704846]	-0.905683065	0.013828243	0.042063656
ENSMMUG00000032281	zinc finger and BTB domain containing 48 [Source:VGNC Symbol;Acc:VGNC:79959]	-0.888417575	0.000187982	0.001012704
ENSMMUG00000023677	TNF receptor superfamily member 25 [Source:VGNC Symbol;Acc:VGNC:78584]	-2.331148448	0.008016878	0.026564433

ENSMMUG00000023676	espin [Source:NCBI gene;Acc:722532]	-1.267627069	5.37E-11	1.06E-09
ENSMMUG00000054719		-0.811571585	0.000265819	0.001384171
ENSMMUG00000032284	leucine rich repeat containing 47 [Source:VGNC Symbol;Acc:VGNC:74388]	2.739885252	0.016204305	0.047959682
ENSMMUG00000012334	actin related protein T2 [Source:VGNC Symbol;Acc:VGNC:69522]	-1.640320779	0.016033871	0.047575746
ENSMMUG00000051487		-3.183640376	0.000110482	0.000624461
ENSMMUG00000050239		-1.745467542	7.57E-10	1.23E-08
ENSMMUG00000001896	G protein subunit beta 1 [Source:VGNC Symbol;Acc:VGNC:73095]	-1.169527528	0.012261876	0.037957679
ENSMMUG00000010879	NAD kinase [Source:VGNC Symbol;Acc:VGNC:75047]	-1.289882354	4.64E-05	0.000287054
ENSMMUG00000005578	solute carrier family 35 member E2 [Source:VGNC Symbol;Acc:VGNC:77590]	-1.026368808	0.001367382	0.00578251
ENSMMUG00000001894	cyclin dependent kinase 11B [Source:NCBI gene;Acc:703046]	-1.105765273	2.77E-05	0.000180525
ENSMMUG00000012961	mindbomb E3 ubiquitin protein ligase 2 [Source:VGNC Symbol;Acc:VGNC:74627]	-0.800979635	0.000510966	0.002460604
ENSMMUG00000048805		-0.890649175	0.000401583	0.001982188
ENSMMUG00000060167		-1.16085683	1.43E-05	9.97E-05
ENSMMUG00000001551	SSU72 homolog, RNA polymerase II CTD phosphatase [Source:VGNC Symbol;Acc:VGNC:77986]	-0.886420048	8.05E-06	5.99E-05
ENSMMUG00000001549	ATPase family AAA domain-containing protein 3A-like [Source:NCBI gene;Acc:114674453]	-0.812268001	9.80E-07	8.87E-06
ENSMMUG00000050086		-1.471287544	0.013552706	0.041360085
ENSMMUG00000006904	cyclin L2 [Source:NCBI gene;Acc:114669744]	-0.827251425	0.005556569	0.019344531
ENSMMUG00000013862	ArfGAP with coiled-coil, ankyrin repeat and PH domains 3 [Source:VGNC Symbol;Acc:VGNC:69434]	-1.188546378	1.11E-06	9.90E-06
ENSMMUG00000045769	tubulin tyrosine ligase like 10 [Source:NCBI gene;Acc:702662]	0.818364445	1.66E-11	3.59E-10
ENSMMUG00000056613		1.051395783	1.16E-14	4.02E-13
ENSMMUG00000053601		2.21362644	0.000558161	0.002661372
ENSMMUG00000065323		-3.476786828	0.004742894	0.016955653
ENSMMUG00000006596	CCR4-NOT transcription complex subunit 10 [Source:VGNC Symbol;Acc:VGNC:71327]	0.839010154	0.001151559	0.004985584
ENSMMUG00000049551		1.32373921	0.000571133	0.002715835
ENSMMUG00000031449	zinc finger CW-type and PWWP domain containing 2 [Source:NCBI gene;Acc:702617]	-1.354553502	0.005847302	0.020196237
ENSMMUG00000055585		-2.273824682	2.64E-11	5.49E-10
ENSMMUG00000000056	5-azacytidine induced 2 [Source:VGNC Symbol;Acc:VGNC:70115]	-1.080769765	0.002528557	0.009863129
ENSMMUG00000051896		-2.998932054	0.004174001	0.015195227
ENSMMUG00000060958	leucine rich repeat containing 3B [Source:NCBI gene;Acc:701323]	-1.643904355	0.002253696	0.008930088
ENSMMUG00000051975		2.11799056	6.80E-16	2.73E-14
ENSMMUG00000054204		-0.477106198	0.008098056	0.026814412
ENSMMUG00000020377	collagen type VI alpha 5 chain [Source:NCBI gene;Acc:719331]	-0.723838694	0.001680367	0.00691223

ENSMMUG00000020294	phosphoinositide-3-kinase regulatory subunit 4 [Source:VGNC Symbol;Acc:VGNC:84060]	-0.774133777	0.00467203	0.016743131
ENSMMUG00000005137	ATPase secretory pathway Ca2+ transporting 1 [Source:VGNC Symbol;Acc:VGNC:70054]	-2.712951242	1.55E-14	5.24E-13
ENSMMUG00000001154	U8 snoRNA-decapping enzyme-like [Source:NCBI gene;Acc:717946]	0.508787456	0.009216793	0.029883719
ENSMMUG00000024125	small nucleolar RNA, H/ACA box 58 [Source:HGNC Symbol;Acc:HGNC:32652]	-3.457005277	0.001616559	0.006677174
ENSMMUG00000003277	copine 4 [Source:VGNC Symbol;Acc:VGNC:71466]	-1.459877384	1.08E-07	1.19E-06
ENSMMUG00000016004	acid phosphatase 3 [Source:VGNC Symbol;Acc:VGNC:69385]	-1.345850395	0.00011609	0.000653788
ENSMMUG00000016005	DnaJ heat shock protein family (Hsp40) member C13 [Source:VGNC Symbol;Acc:VGNC:84191]	-0.606155397	1.10E-10	2.09E-09
ENSMMUG00000054158	acyl-CoA dehydrogenase family member 11 [Source:NCBI gene;Acc:106997118]	1.160923876	1.91E-08	2.44E-07
ENSMMUG00000021772	nephrocystin 3 [Source:NCBI gene;Acc:717622]	-1.320789976	8.35E-06	6.19E-05
ENSMMUG00000019857	beaded filament structural protein 2 [Source:VGNC Symbol;Acc:VGNC:70245]	-0.885841734	2.10E-06	1.76E-05
ENSMMUG00000000058	CDV3 homolog [Source:VGNC Symbol;Acc:VGNC:71114]	-1.05631594	0.00064275	0.002995442
ENSMMUG00000000059	DNA topoisomerase II binding protein 1 [Source:VGNC Symbol;Acc:VGNC:79061]	-1.178958759	5.82E-09	8.16E-08
ENSMMUG00000008916	transferrin [Source:VGNC Symbol;Acc:VGNC:82716]	-1.101492129	6.25E-09	8.69E-08
ENSMMUG00000008918	SRP receptor subunit beta [Source:VGNC Symbol;Acc:VGNC:77980]	-1.088934707	1.33E-06	1.16E-05
ENSMMUG00000008919	RAB6B, member RAS oncogene family [Source:VGNC Symbol;Acc:VGNC:81547]	-0.99894154	7.54E-06	5.66E-05
ENSMMUG00000012605	solute carrier organic anion transporter family member 2A1 [Source:VGNC Symbol;Acc:VGNC:77600]	-0.486383199	0.000685945	0.003176687
ENSMMUG00000007757	extended synaptotagmin 3 [Source:VGNC Symbol;Acc:VGNC:84182]	-0.427293418	0.015268296	0.045603263
ENSMMUG00000007760	centrosomal protein 70 [Source:VGNC Symbol;Acc:VGNC:71140]	0.432348241	0.000557837	0.002660733
ENSMMUG00000003051	COPI coat complex subunit beta 2 [Source:VGNC Symbol;Acc:VGNC:71313]	-1.020634044	3.10E-11	6.37E-10
ENSMMUG00000062527		-1.874555716	6.08E-19	3.41E-17
ENSMMUG00000022153	solute carrier family 25 member 36 [Source:VGNC Symbol;Acc:VGNC:84079]	-2.217920552	2.89E-21	2.02E-19
ENSMMUG00000053344	splA/ryanodine receptor domain and SOCS box containing 4 [Source:NCBI gene;Acc:715278]	-2.764993726	3.03E-09	4.46E-08
ENSMMUG00000057297		-1.503435765	0.001234081	0.005301935
ENSMMUG00000056024		1.03103651	1.02E-08	1.36E-07
ENSMMUG00000063351	Metazoan signal recognition particle RNA [Source:RFAM;Acc:RF00017]	1.635095865	0.000695683	0.00321654
ENSMMUG00000007387	G protein-coupled receptor kinase 7 [Source:VGNC Symbol;Acc:VGNC:73182]	1.313525254	1.52E-08	1.98E-07
ENSMMUG00000052474		1.630657541	8.53E-11	1.65E-09
ENSMMUG00000011134	transcription factor Dp-2 [Source:VGNC Symbol;Acc:VGNC:78324]	0.50994674	0.001577274	0.006538018
ENSMMUG00000012151	glycerol kinase 5 [Source:VGNC Symbol;Acc:VGNC:73022]	-2.063884036	1.76E-05	0.000120287
ENSMMUG00000012405	transient receptor potential cation channel subfamily C member 1 [Source:VGNC Symbol;Acc:VGNC:78651]	3.875689495	7.00E-25	7.38E-23
ENSMMUG00000002775	U2 snRNP associated SURP domain containing [Source:VGNC Symbol;Acc:VGNC:79889]	-0.904462154	0.001414615	0.005955314
ENSMMUG00000009694	solute carrier family 9 member A9 [Source:VGNC Symbol;Acc:VGNC:77747]	-2.071228968	0.000534024	0.00255584
ENSMMUG00000062595		0.936576561	0.001944938	0.00786217

ENSMMUG00000049859		-0.765774391	0.002172595	0.008665046
ENSMMUG00000062175	carboxypeptidase B1 [Source:NCBI gene;Acc:712639]	0.636218838	0.0040248	0.014712117
ENSMMUG00000005519	carboxypeptidase A3 [Source:VGNC Symbol;Acc:VGNC:71456]	0.634721904	0.001929372	0.007808251
ENSMMUG00000005521	glycogenin 1 [Source:VGNC Symbol;Acc:VGNC:73332]	0.847887875	2.83E-07	2.87E-06
ENSMMUG00000005526	helicase like transcription factor [Source:VGNC Symbol;Acc:VGNC:73383]	0.569497017	0.011562037	0.036174576
ENSMMUG00000047621	transmembrane 4 L six family member 18 [Source:VGNC Symbol;Acc:VGNC:82713]	0.544981322	2.24E-05	0.000149717
ENSMMUG00000031534	ankyrin repeat and ubiquitin domain containing 1 [Source:VGNC Symbol;Acc:VGNC:84239]	2.193119955	1.17E-34	3.16E-32
ENSMMUG00000035943	U6 spliceosomal RNA [Source:RFAM;Acc:RF00026]	2.79832459	7.15E-14	2.19E-12
ENSMMUG00000023335	ring finger protein 13 [Source:VGNC Symbol;Acc:VGNC:81557]	1.469427712	2.00E-10	3.62E-09
ENSMMUG00000059788		1.0547322	1.45E-14	4.94E-13
ENSMMUG00000015590	siah E3 ubiquitin protein ligase 2 [Source:VGNC Symbol;Acc:VGNC:77215]	-2.079008398	1.20E-05	8.56E-05
ENSMMUG00000048232	MINDY family member 4B [Source:VGNC Symbol;Acc:VGNC:84153]	-0.863906123	0.000114998	0.000648224
ENSMMUG00000038998	purinergic receptor P2Y13 [Source:VGNC Symbol;Acc:VGNC:83950]	0.692527234	0.004357284	0.01578868
ENSMMUG00000057554	Metazoan signal recognition particle RNA [Source:RFAM;Acc:RF00017]	1.415133924	0.003453267	0.012847119
ENSMMUG00000057501		-1.297333777	3.14E-06	2.56E-05
ENSMMUG00000061684		0.875553882	0.014689443	0.044203877
ENSMMUG00000031540	purinergic receptor P2Y1 [Source:VGNC Symbol;Acc:VGNC:75737]	5.472028101	1.92E-08	2.45E-07
ENSMMUG00000011347	DEAH-box helicase 36 [Source:VGNC Symbol;Acc:VGNC:71623]	0.922420268	3.55E-23	2.99E-21
ENSMMUG00000056924	G protein-coupled receptor 149 [Source:NCBI gene;Acc:106997054]	1.083214422	0.000161697	0.000882286
ENSMMUG00000060572		0.942065226	4.57E-05	0.00028333
ENSMMUG00000056116		-1.32435648	8.24E-10	1.33E-08
ENSMMUG00000009564	NMD3 ribosome export adaptor [Source:VGNC Symbol;Acc:VGNC:75242]	1.632796279	0.000299436	0.001531921
ENSMMUG00000042979		-1.842562188	0.012956348	0.039791254
ENSMMUG00000001302	sucrase-isomaltase [Source:VGNC Symbol;Acc:VGNC:77213]	0.661485379	2.68E-07	2.74E-06
ENSMMUG00000003742	butyrylcholinesterase [Source:VGNC Symbol;Acc:VGNC:70238]	0.720536976	0.000604439	0.002859663
ENSMMUG00000023045	zinc finger B-box domain containing [Source:VGNC Symbol;Acc:VGNC:79592]	1.843543989	2.66E-05	0.000174502
ENSMMUG00000047393	protein phosphatase 1 regulatory subunit 7-like [Source:NCBI gene;Acc:699349]	0.680866435	0.005252148	0.018458866
ENSMMUG00000020734	golgi integral membrane protein 4 [Source:VGNC Symbol;Acc:VGNC:72945]	0.337106941	0.00336203	0.012568848
ENSMMUG00000051641		-1.259899207	1.33E-07	1.44E-06
ENSMMUG00000022199	polyhomeotic homolog 3 [Source:VGNC Symbol;Acc:VGNC:75804]	-2.349910366	6.60E-16	2.67E-14
ENSMMUG00000056664	U6 spliceosomal RNA [Source:RFAM;Acc:RF00026]	-1.885250188	0.000455377	0.00222113
ENSMMUG00000022880	eukaryotic translation initiation factor 5A2 [Source:VGNC Symbol;Acc:VGNC:72198]	-0.668935335	0.003884133	0.014258427
ENSMMUG00000023289	phospholipase D1 [Source:VGNC Symbol;Acc:VGNC:76171]	-2.510010528	0.009466257	0.030551253
ENSMMUG00000003594	ubiquitin specific peptidase 13 [Source:VGNC Symbol;Acc:VGNC:78735]	-0.706866291	2.64E-05	0.000172944
ENSMMUG00000064088	U2 spliceosomal RNA [Source:RFAM;Acc:RF00004]	-0.53187997	0.003850985	0.014151561
ENSMMUG00000010553	methylcrotonoyl-CoA carboxylase 1 [Source:VGNC Symbol;Acc:VGNC:74676]	-0.630110551	0.003183248	0.011957858
ENSMMUG00000008070	YEATS domain containing 2 [Source:VGNC Symbol;Acc:VGNC:79502]	1.912629825	2.34E-32	5.46E-30
ENSMMUG00000005515	ATP binding cassette subfamily C member 5 [Source:VGNC Symbol;Acc:VGNC:69574]	-1.001868747	2.12E-09	3.21E-08
ENSMMUG00000006562	eukaryotic translation initiation factor 2B subunit epsilon [Source:VGNC Symbol;Acc:VGNC:72186]	-0.43244098	0.014386244	0.043506733
ENSMMUG00000006553	dishevelled segment polarity protein 3 [Source:VGNC Symbol;Acc:VGNC:72014]	-0.773308593	0.007652974	0.025533583

ENSMMUG00000006557	adaptor related protein complex 2 subunit mu 1 [Source:VGNC Symbol;Acc:VGNC:69965]	-1.188119098	0.016168364	0.047883625
ENSMMUG00000006558	ATP binding cassette subfamily F member 3 [Source:VGNC Symbol;Acc:VGNC:69583]	0.415852652	0.001168647	0.005051769
ENSMMUG00000018955	EEF1A lysine methyltransferase 4 [Source:NCBI gene;Acc:106996971]	-0.429361729	0.000154567	0.000848667
ENSMMUG00000000800	proteasome 26S subunit, non-ATPase 2 [Source:VGNC Symbol;Acc:VGNC:84144]	-0.569571531	1.49E-06	1.29E-05
ENSMMUG00000010934	eukaryotic translation initiation factor 4 gamma 1 [Source:VGNC Symbol;Acc:VGNC:72195]	-0.571818386	2.95E-06	2.42E-05
ENSMMUG00000007503	family with sequence similarity 131 member A [Source:VGNC Symbol;Acc:VGNC:72385]	-0.489421272	0.000556888	0.002657107
ENSMMUG00000007507	chloride voltage-gated channel 2 [Source:VGNC Symbol;Acc:VGNC:71235]	-0.608715137	7.73E-07	7.18E-06
ENSMMUG00000007524	RNA polymerase II subunit H [Source:VGNC Symbol;Acc:VGNC:76216]	-0.989226923	1.76E-07	1.86E-06
ENSMMUG00000007528	chordin [Source:VGNC Symbol;Acc:VGNC:71042]	2.331755771	2.08E-09	3.17E-08
ENSMMUG00000060581		1.905666575	8.09E-07	7.47E-06
ENSMMUG00000002953	MAGE family member F1 [Source:VGNC Symbol;Acc:VGNC:74399]	0.997595663	0.000634904	0.002967751
ENSMMUG00000064748	Metazoan signal recognition particle RNA [Source:RFAM;Acc:RF00017]	-1.514761269	0.000631958	0.002954966
ENSMMUG00000021972	diacylglycerol kinase gamma [Source:VGNC Symbol;Acc:VGNC:71785]	1.601861707	2.62E-27	3.56E-25
ENSMMUG00000052418		-0.654530168	2.26E-05	0.000150837
ENSMMUG00000018909	fetuin B [Source:VGNC Symbol;Acc:VGNC:72638]	0.583099435	2.58E-05	0.000169374
ENSMMUG00000006816	tetratricopeptide repeat and ankyrin repeat containing 1 [Source:VGNC Symbol;Acc:VGNC:82709]	-0.56044511	0.002225846	0.008842222
ENSMMUG00000022539	LRR binding FLII interacting protein 2 [Source:VGNC Symbol;Acc:VGNC:74449]	0.320500359	0.004210312	0.015315489
ENSMMUG00000049937	Y RNA [Source:RFAM;Acc:RF00019]	-0.810552872	0.008600501	0.028251308
ENSMMUG00000054175		1.181847388	6.80E-18	3.39E-16
ENSMMUG00000004847	xylulokinase [Source:VGNC Symbol;Acc:VGNC:78815]	0.652100268	2.60E-06	2.15E-05
ENSMMUG00000018790	activin A receptor type 2B [Source:VGNC Symbol;Acc:VGNC:69450]	2.07765003	2.08E-15	7.84E-14
ENSMMUG00000015827	sodium voltage-gated channel alpha subunit 10 [Source:VGNC Symbol;Acc:VGNC:76980]	-0.340961702	0.001201392	0.005177358
ENSMMUG00000020150	xin actin binding repeat containing 1 [Source:VGNC Symbol;Acc:VGNC:78952]	-0.66256815	6.25E-12	1.45E-10
ENSMMUG00000022963	solute carrier family 25 member 38 [Source:VGNC Symbol;Acc:VGNC:77571]	-1.005250826	0.003854109	0.014159332
ENSMMUG00000051711		-0.384620245	0.001302108	0.005546616
ENSMMUG00000051408	myelin associated oligodendrocyte basic protein [Source:NCBI gene;Acc:717265]	1.193260563	1.30E-06	1.14E-05
ENSMMUG00000009197	myelin associated oligodendrocyte basic protein [Source:VGNC Symbol;Acc:VGNC:74908]	1.486980071	2.13E-11	4.51E-10
ENSMMUG00000017701	myosin VIIA and Rab interacting protein [Source:VGNC Symbol;Acc:VGNC:74992]	1.870495374	2.24E-11	4.71E-10
ENSMMUG00000013330	ectonucleoside triphosphate diphosphohydrolase 3 [Source:VGNC Symbol;Acc:VGNC:72226]	1.334720055	0.000177766	0.000962474
ENSMMUG00000009437	zinc finger protein 619 [Source:VGNC Symbol;Acc:VGNC:82562]	1.988704818	0.000227672	0.001205226
ENSMMUG00000001074	unc-51 like kinase 4 [Source:VGNC Symbol;Acc:VGNC:82565]	-0.779090856	0.001097289	0.004783117
ENSMMUG00000035160	U4 spliceosomal RNA [Source:RFAM;Acc:RF00015]	2.10721692	0.012585275	0.038787541
ENSMMUG00000021242	natural killer cell triggering receptor [Source:VGNC Symbol;Acc:VGNC:81486]	-2.938076918	0.000301263	0.00154014
ENSMMUG00000021243	zinc finger and BTB domain containing 47 [Source:VGNC Symbol;Acc:VGNC:79645]	-2.768942232	0.003485305	0.012954259
ENSMMUG00000021244	kelch like family member 40 [Source:VGNC Symbol;Acc:VGNC:74165]	-2.958830081	0.000231658	0.001223096
ENSMMUG00000021246	hedgehog acyltransferase like [Source:NCBI gene;Acc:717333]	-2.507562351	1.12E-05	8.04E-05
ENSMMUG00000031684	KRAB box domain containing 1 [Source:NCBI gene;Acc:717189]	2.030413599	0.006799015	0.022979655

ENSMMUG00000009745	protein O-linked mannose N-acetylglucosaminyltransferase 2 (beta 1,4-) [Source:VGNC Symbol;Acc:VGNC:84062]	1.206253839	0.008966891	0.029201745
ENSMMUG00000056798		2.449953233	2.03E-09	3.10E-08
ENSMMUG00000054286		1.47684602	6.26E-06	4.80E-05
ENSMMUG00000001028	SNF related kinase [Source:VGNC Symbol;Acc:VGNC:84074]	0.826487256	4.33E-14	1.38E-12
ENSMMUG00000040696	Metazoan signal recognition particle RNA [Source:RFAM;Acc:RF00017]	2.076879432	0.006197737	0.021223656
ENSMMUG00000051139	C-type lectin domain family 3 member B [Source:NCBI gene;Acc:106996937]	-1.120473745	0.007478	0.025003337
ENSMMUG00000005742	CUB domain containing protein 1 [Source:VGNC Symbol;Acc:VGNC:70849]	-1.289396789	0.003699084	0.013672182
ENSMMUG00000056769	C-C motif chemokine receptor 1 [Source:VGNC Symbol;Acc:VGNC:70737]	-0.784135473	0.001701882	0.00699048
ENSMMUG00000004576	C-C motif chemokine receptor 3 [Source:VGNC Symbol;Acc:VGNC:70738]	1.343306325	0.00203567	0.008191161
ENSMMUG00000062394	C-C motif chemokine receptor 2 [Source:NCBI gene;Acc:574098]	0.628452976	2.47E-06	2.05E-05
ENSMMUG00000006454	kelch like family member 18 [Source:VGNC Symbol;Acc:VGNC:74154]	-1.04067223	6.49E-06	4.96E-05
ENSMMUG00000014045	protein tyrosine phosphatase non-receptor type 23 [Source:VGNC Symbol;Acc:VGNC:76477]	1.431139384	1.37E-11	2.99E-10
ENSMMUG00000014046	SREBF chaperone [Source:VGNC Symbol;Acc:VGNC:77140]	2.434485329	2.37E-29	4.05E-27
ENSMMUG00000013385	elongator acetyltransferase complex subunit 6 [Source:VGNC Symbol;Acc:VGNC:72113]	0.91979497	8.08E-07	7.47E-06
ENSMMUG00000020954	cell division cycle 25A [Source:VGNC Symbol;Acc:VGNC:70794]	-0.734649843	0.004455915	0.016063057
ENSMMUG00000054936	Metazoan signal recognition particle RNA [Source:RFAM;Acc:RF00017]	-1.010987817	0.004568595	0.016427027
ENSMMUG00000064458		-1.114169881	6.73E-05	0.00039963
ENSMMUG00000003074	plexin B1 [Source:VGNC Symbol;Acc:VGNC:76187]	1.926263033	0.011459548	0.035918023
ENSMMUG00000010058	ATR interacting protein [Source:VGNC Symbol;Acc:VGNC:84235]	0.575672611	4.46E-09	6.43E-08
ENSMMUG00000043955	three prime repair exonuclease 1 [Source:VGNC Symbol;Acc:VGNC:82708]	0.754684532	7.59E-06	5.69E-05
ENSMMUG00000010062	shisa family member 5 [Source:VGNC Symbol;Acc:VGNC:84082]	0.559751606	1.69E-05	0.00011637
ENSMMUG00000000591	6-phosphofructo-2-kinase/fructose-2,6-biphosphatase 4 [Source:VGNC Symbol;Acc:VGNC:75960]	0.634409156	4.34E-07	4.25E-06
ENSMMUG00000059318	urocortin 2 [Source:NCBI gene;Acc:106996925]	2.116321069	1.62E-42	9.09E-40
ENSMMUG00000014699		1.775864615	4.26E-66	1.19E-62
ENSMMUG00000032659	mml-mir-711 [Source:miRBase;Acc:MI0012553]	2.157499094	2.11E-07	2.19E-06
ENSMMUG00000031754	solute carrier family 26 member 6 [Source:VGNC Symbol;Acc:VGNC:77483]	2.274921443	0.011893848	0.037022737
ENSMMUG00000014707	cadherin EGF LAG seven-pass G-type receptor 3 [Source:VGNC Symbol;Acc:VGNC:71148]	2.15569733	9.61E-07	8.71E-06
ENSMMUG00000064174		1.755033191	0.000887483	0.00397531
ENSMMUG00000014720	NCK interacting protein with SH3 domain [Source:VGNC Symbol;Acc:VGNC:74960]	1.969580018	7.44E-09	1.02E-07
ENSMMUG00000047476	ras homolog family member A [Source:VGNC Symbol;Acc:VGNC:84066]	0.352042529	0.016540455	0.048748801
ENSMMUG00000052238		0.750589529	3.57E-05	0.000227374
ENSMMUG00000014537	bassoon presynaptic cytomatrix protein [Source:VGNC Symbol;Acc:VGNC:70284]	0.392051356	0.012386654	0.038259481
ENSMMUG00000014564	ring finger protein 123 [Source:VGNC Symbol;Acc:VGNC:76790]	-0.80684567	5.26E-12	1.23E-10
ENSMMUG00000031794		1.312752902	0.00099994	0.00441087
ENSMMUG00000020979	inka box actin regulator 1 [Source:VGNC Symbol;Acc:VGNC:73692]	1.076351066	8.24E-05	0.000479492
ENSMMUG00000020980	ubiquitin like modifier activating enzyme 7 [Source:VGNC Symbol;Acc:VGNC:78690]	1.12286027	0.000129752	0.000724328
ENSMMUG00000016476	TRAF interacting protein [Source:VGNC Symbol;Acc:VGNC:82261]	1.014527328	1.37E-05	9.63E-05
ENSMMUG00000016480	CaM kinase like vesicle associated [Source:VGNC Symbol;Acc:VGNC:70663]	0.841875653	0.006325842	0.021614835
ENSMMUG00000010342	solute carrier family 38 member 3 [Source:VGNC Symbol;Acc:VGNC:77502]	-1.224896371	0.007847907	0.026090847
ENSMMUG00000056280	G protein subunit alpha i2 [Source:NCBI gene;Acc:703780]	-0.690658671	3.82E-05	0.000241508
ENSMMUG00000020117	semaphorin 3B [Source:VGNC Symbol;Acc:VGNC:77160]	-0.834060041	0.005576051	0.019393096
ENSMMUG00000010495	MAPK activated protein kinase 3 [Source:VGNC Symbol;Acc:VGNC:74654]	-1.12264881	0.006026887	0.020739886
ENSMMUG00000004273	dedicator of cytokinesis 3 [Source:VGNC Symbol;Acc:VGNC:71967]	1.220026221	8.97E-13	2.33E-11
ENSMMUG00000004280	DDB1 and CUL4 associated factor 1 [Source:VGNC Symbol;Acc:VGNC:71819]	1.557322456	0.00151522	0.006316269

ENSMMUG00000017351	stabilin 1 [Source:VGNC Symbol;Acc:VGNC:77956]	-1.235978243	3.70E-08	4.49E-07
ENSMMUG00000050505	small integral membrane protein 4 [Source:NCBI gene;Acc:696553]	1.741160313	1.97E-20	1.29E-18
ENSMMUG00000012511	G protein nucleolar 3 [Source:VGNC Symbol;Acc:VGNC:73038]	0.310833174	0.004595452	0.016498977
ENSMMUG00000012526	glycosyltransferase 8 domain containing 1 [Source:VGNC Symbol;Acc:VGNC:73001]	1.914157192	6.11E-35	1.75E-32
ENSMMUG00000017370	signal peptidase complex subunit 1 [Source:VGNC Symbol;Acc:VGNC:84072]	1.837624574	3.37E-10	5.80E-09
ENSMMUG00000019262	calcium voltage-gated channel subunit alpha1 D [Source:VGNC Symbol;Acc:VGNC:70501]	-1.185734743	0.000869214	0.003904224
ENSMMUG00000019266	choline dehydrogenase [Source:VGNC Symbol;Acc:VGNC:71086]	-0.810160187	0.000297979	0.001526691
ENSMMUG00000019267	interleukin 17 receptor B [Source:VGNC Symbol;Acc:VGNC:73618]	-0.594909261	0.009414126	0.030418039
ENSMMUG00000021620	ELKS/RAB6-interacting/CAST family member 2 [Source:VGNC Symbol;Acc:VGNC:72164]	-0.975103735	6.13E-11	1.20E-09
ENSMMUG00000039085	adaptor protein, phosphotyrosine interacting with PH domain and leucine zipper 1 [Source:NCBI gene;Acc:100425753]	-2.31260487	0.000488396	0.002362464
ENSMMUG00000021008	ankyrin repeat and SOCS box containing 14 [Source:VGNC Symbol;Acc:VGNC:84237]	-1.976459086	0.002811077	0.010802888
ENSMMUG00000054683	dynein axonemal heavy chain 12 [Source:NCBI gene;Acc:703670]	-1.151339686	3.84E-07	3.83E-06
ENSMMUG00000055244	U6 spliceosomal RNA [Source:RFAM;Acc:RF00026]	-1.663223216	1.25E-12	3.19E-11
ENSMMUG00000004052	phosphodiesterase 12 [Source:VGNC Symbol;Acc:VGNC:75863]	-1.37747481	0.002005622	0.00807953
ENSMMUG00000018619	DENN domain containing 6A [Source:VGNC Symbol;Acc:VGNC:71726]	2.104107212	9.51E-06	6.94E-05
ENSMMUG00000000706	filamin B [Source:VGNC Symbol;Acc:VGNC:72672]	1.558970797	1.19E-21	8.62E-20
ENSMMUG00000017510	membrane associated guanylate kinase, WW and PDZ domain containing 1 [Source:VGNC Symbol;Acc:VGNC:74499]	2.177362112	9.31E-33	2.25E-30
ENSMMUG00000057539		2.676330451	2.51E-14	8.27E-13
ENSMMUG00000054412		-0.605471906	0.001815299	0.007397831
ENSMMUG00000054043		-1.597719573	0.014452715	0.043631953
ENSMMUG00000009425	actin, cytoplasmic 2-like [Source:NCBI gene;Acc:705671]	-1.025491767	1.01E-08	1.35E-07
ENSMMUG00000056158		1.664803814	3.07E-06	2.50E-05
ENSMMUG00000054107		-0.957077279	9.14E-06	6.71E-05
ENSMMUG00000001849	interleukin 5 receptor subunit alpha [Source:VGNC Symbol;Acc:VGNC:73731]	-0.789625348	0.000140537	0.000778031
ENSMMUG00000017983	tRNA nucleotidyl transferase 1 [Source:VGNC Symbol;Acc:VGNC:79073]	2.204277432	2.02E-06	1.70E-05
ENSMMUG00000060798		-1.432879613	2.83E-08	3.51E-07
ENSMMUG00000012574	inositol 1,4,5-trisphosphate receptor type 1 [Source:VGNC Symbol;Acc:VGNC:73804]	-0.825422461	9.24E-06	6.77E-05
ENSMMUG00000043287		-0.494145834	0.002963322	0.01128283
ENSMMUG00000018281	interleukin 17 receptor E [Source:VGNC Symbol;Acc:VGNC:73623]	-0.776797079	5.29E-07	5.09E-06
ENSMMUG00000018282	interleukin 17 receptor C [Source:VGNC Symbol;Acc:VGNC:84164]	-1.01844276	2.04E-07	2.13E-06
ENSMMUG00000018285	cysteine rich with EGF like domains 1 [Source:VGNC Symbol;Acc:VGNC:71407]	-0.85277896	1.77E-07	1.87E-06
ENSMMUG00000018289	proline rich transmembrane protein 3 [Source:VGNC Symbol;Acc:VGNC:76324]	-0.864997454	2.47E-09	3.70E-08
ENSMMUG00000019933	TatD DNase domain containing 2 [Source:NCBI gene;Acc:701390]	0.914457623	0.010017113	0.032019597
ENSMMUG00000021806	vestigial like family member 4 [Source:VGNC Symbol;Acc:VGNC:79755]	-0.867626818	6.15E-05	0.000370067
ENSMMUG00000009536	makorin ring finger protein 2 [Source:VGNC Symbol;Acc:VGNC:74878]	-1.329521192	6.56E-07	6.18E-06

ENSMMUG00000023125	XPC complex subunit, DNA damage recognition and repair factor [Source:VGNC Symbol;Acc:VGNC:78809]	-0.860009979	0.000780961	0.003568398
ENSMMUG00000023124	transmembrane protein 43 [Source:VGNC Symbol;Acc:VGNC:82711]	-0.939524746	2.19E-05	0.000146791
ENSMMUG00000006340	solute carrier family 6 member 6 [Source:VGNC Symbol;Acc:VGNC:77622]	-0.883318401	5.45E-10	9.07E-09
ENSMMUG00000044822	coiled-coil domain containing 174 [Source:VGNC Symbol;Acc:VGNC:84219]	0.754393683	8.47E-08	9.61E-07
ENSMMUG00000000158	minichromosome maintenance complex component 2 [Source:VGNC Symbol;Acc:VGNC:74680]	-1.466514262	4.36E-21	2.99E-19
ENSMMUG00000017125	oxysterol binding protein like 11 [Source:VGNC Symbol;Acc:VGNC:75631]	-1.693539856	2.33E-22	1.82E-20
ENSMMUG00000032031		-1.007929903	0.006568936	0.022347452
ENSMMUG00000001949	coiled-coil domain containing 191 [Source:VGNC Symbol;Acc:VGNC:70901]	1.278690764	0.002322888	0.009155102
ENSMMUG00000001946	zinc finger DHHC-type palmitoyltransferase 23 [Source:VGNC Symbol;Acc:VGNC:79731]	1.082537032	0.003039368	0.011528553
ENSMMUG00000001943	GRAM domain containing 1C [Source:VGNC Symbol;Acc:VGNC:73007]	1.218269943	8.35E-07	7.70E-06
ENSMMUG00000052581	upstream transcription factor family member 3 [Source:NCBI gene;Acc:709660]	-1.031793969	3.47E-11	7.06E-10
ENSMMUG00000057363		1.224430985	0.000569983	0.002713125
ENSMMUG00000014874	GTP binding protein 8 (putative) [Source:VGNC Symbol;Acc:VGNC:84180]	1.575397007	6.17E-13	1.64E-11
ENSMMUG00000014876	nucleolus and neural progenitor protein [Source:VGNC Symbol;Acc:VGNC:75244]	1.695576215	5.18E-14	1.63E-12
ENSMMUG00000003199	CD200 receptor 1 [Source:VGNC Symbol;Acc:VGNC:70845]	1.009573358	0.015643427	0.046604585
ENSMMUG00000000894	cellular inhibitor of PP2A [Source:VGNC Symbol;Acc:VGNC:71371]	2.450965794	0.000433668	0.00212634
ENSMMUG00000019977	myosin heavy chain 15 [Source:VGNC Symbol;Acc:VGNC:84148]	-1.038854074	4.58E-05	0.000283733
ENSMMUG00000019978	HERV-H LTR-associating 2 [Source:VGNC Symbol;Acc:VGNC:73377]	-1.360066109	0.000832415	0.003765469
ENSMMUG00000008791	CD47 molecule [Source:VGNC Symbol;Acc:VGNC:70767]	3.480736865	3.74E-60	6.56E-57
ENSMMUG00000058765		-0.792654526	0.016470657	0.048592541
ENSMMUG00000063790		2.090458647	0.003412291	0.012712687
ENSMMUG00000007565	Cbl proto-oncogene B [Source:VGNC Symbol;Acc:VGNC:70555]	-1.027317156	7.59E-09	1.04E-07
ENSMMUG00000026103	U1 spliceosomal RNA [Source:RFAM;Acc:RF00003]	-2.830021381	3.15E-06	2.56E-05
ENSMMUG00000060744		-1.630121943	5.56E-09	7.83E-08
ENSMMUG00000063181		0.452480713	0.003761949	0.013867983
ENSMMUG00000059753		-0.573091351	0.000449707	0.002196184
ENSMMUG00000021567	neurexophilin and PC-esterase domain family member 3 [Source:VGNC Symbol;Acc:VGNC:75476]	-0.576198968	0.000170474	0.000925853
ENSMMUG00000057768		-0.666108153	0.00027502	0.001423776
ENSMMUG00000024598	U6 spliceosomal RNA [Source:RFAM;Acc:RF00026]	-3.017412561	0.000612913	0.002890977
ENSMMUG00000038349	crystallin beta-gamma domain containing 3 [Source:NCBI gene;Acc:698282]	0.502158412	0.000372221	0.001846359
ENSMMUG00000049786		-1.651997362	0.002053762	0.008247395
ENSMMUG00000043727	zinc finger protein 654 [Source:VGNC Symbol;Acc:VGNC:82560]	-1.636142474	0.000112589	0.00063509
ENSMMUG00000047994	CGG triplet repeat binding protein 1 [Source:VGNC Symbol;Acc:VGNC:84203]	-1.263136766	2.23E-05	0.000149183
ENSMMUG00000033738	RNA, U6atac small nuclear 6, pseudogene [Source:HGNC Symbol;Acc:HGNC:46905]	-0.565014297	0.002622382	0.010178152

ENSMMUG00000015877	1,4-alpha-glucan branching enzyme 1 [Source:VGNC Symbol;Acc:VGNC:72894]	0.554540658	0.001252604	0.005366718
ENSMMUG00000055096		1.069551397	1.08E-11	2.41E-10
ENSMMUG00000056687		-1.537760007	8.61E-08	9.76E-07
ENSMMUG00000052831		0.93637592	3.89E-08	4.69E-07
ENSMMUG00000032295	zinc finger protein 595 [Source:VGNC Symbol;Acc:VGNC:79655]	-0.734917625	0.001872303	0.007610369
ENSMMUG00000051381	Metazoan signal recognition particle RNA [Source:RFAM;Acc:RF00017]	-2.058955351	1.44E-08	1.88E-07
ENSMMUG00000039020	zinc finger protein 141 [Source:NCBI gene;Acc:714265]	-1.695713225	2.49E-11	5.24E-10
ENSMMUG00000003282	phosphodiesterase 6B [Source:VGNC Symbol;Acc:VGNC:75920]	-1.626996198	7.37E-06	5.54E-05
ENSMMUG00000008323	C-terminal binding protein 1 [Source:VGNC Symbol;Acc:VGNC:71539]	-0.39084898	0.000666304	0.00309557
ENSMMUG00000046942	nuclear receptor binding SET domain protein 2 [Source:NCBI gene;Acc:100429321]	-0.563928264	0.001894494	0.007693742
ENSMMUG00000032306	zinc finger FYVE-type containing 28 [Source:NCBI gene;Acc:720600]	-0.802851936	0.005058367	0.01787638
ENSMMUG00000007966	TNFAIP3 interacting protein 2 [Source:VGNC Symbol;Acc:VGNC:79182]	1.76495469	0.002799934	0.010763012
ENSMMUG00000008379	adducin 1 [Source:VGNC Symbol;Acc:VGNC:69528]	1.351235226	3.57E-08	4.34E-07
ENSMMUG00000008383	major facilitator superfamily domain containing 10 [Source:NCBI gene;Acc:720098]	1.561104789	2.80E-13	7.79E-12
ENSMMUG00000008385	NOP14 nucleolar protein [Source:VGNC Symbol;Acc:VGNC:75194]	1.267014674	1.38E-16	5.99E-15
ENSMMUG00000008392	huntingtin [Source:VGNC Symbol;Acc:VGNC:73468]	-0.756897799	5.80E-07	5.53E-06
ENSMMUG00000008398	Myb/SANT DNA binding domain containing 1 [Source:VGNC Symbol;Acc:VGNC:74869]	-0.690314825	0.00192449	0.007795241
ENSMMUG00000006237	carboxypeptidase Z [Source:VGNC Symbol;Acc:VGNC:71478]	3.705265804	0.012712859	0.039107046
ENSMMUG00000006234	acyl-CoA oxidase 3, pristanoyl [Source:VGNC Symbol;Acc:VGNC:69382]	0.62842753	5.46E-06	4.23E-05
ENSMMUG00000030400	SH3 domain and tetratricopeptide repeats 1 [Source:NCBI gene;Acc:722649]	-0.559555449	9.46E-05	0.000542983
ENSMMUG00000016999	KIAA0232 [Source:VGNC Symbol;Acc:VGNC:73922]	4.593051039	6.23E-06	4.77E-05
ENSMMUG00000053541		-0.866177573	0.004072044	0.014858774
ENSMMUG00000015099	janus kinase and microtubule interacting protein 1 [Source:VGNC Symbol;Acc:VGNC:73662]	-0.660224542	0.00067577	0.003133707
ENSMMUG00000019563	collapsin response mediator protein 1 [Source:VGNC Symbol;Acc:VGNC:71494]	1.412968627	1.73E-30	3.24E-28
ENSMMUG00000057703		1.339678706	7.90E-14	2.39E-12
ENSMMUG00000020861	EvC ciliary complex subunit 1 [Source:VGNC Symbol;Acc:VGNC:72445]	1.308363522	2.29E-18	1.23E-16
ENSMMUG00000020860	EvC ciliary complex subunit 2 [Source:VGNC Symbol;Acc:VGNC:72301]	0.658388175	1.36E-05	9.57E-05
ENSMMUG00000016922	serine/threonine kinase 32B [Source:VGNC Symbol;Acc:VGNC:78114]	0.906102425	0.001053592	0.00461848
ENSMMUG00000060830		-2.845269929	0.004415486	0.015946011
ENSMMUG00000049640		-0.620354273	0.008322991	0.027455494
ENSMMUG00000008162	fibroblast growth factor binding protein 1 [Source:VGNC Symbol;Acc:VGNC:72536]	1.369125512	1.86E-06	1.57E-05
ENSMMUG00000008164	prominin 1 [Source:VGNC Symbol;Acc:VGNC:76425]	-0.510078566	2.38E-05	0.00015772
ENSMMUG00000023116	LIM domain binding 2 [Source:VGNC Symbol;Acc:VGNC:74246]	-0.907401897	6.49E-07	6.13E-06
ENSMMUG00000018073	ligand dependent nuclear receptor corepressor like [Source:NCBI gene;Acc:715285]	-0.557101005	0.001813871	0.00739416
ENSMMUG00000057338		-1.306380968	7.52E-12	1.72E-10
ENSMMUG00000060440	leucine rich repeat LGI family member 2 [Source:NCBI gene;Acc:693308]	-0.452909535	0.001038447	0.004556363

ENSMMUG00000020349	SEL1L family member 3 [Source:VGNC Symbol;Acc:VGNC:77329]	-0.326630048	0.012068171	0.037448984
ENSMMUG00000049298	cholecystokinin A receptor [Source:NCBI gene;Acc:694742]	4.363343975	7.31E-05	0.000430525
ENSMMUG00000057711		0.822150305	0.000821509	0.003726958
ENSMMUG00000051768		-1.198095335	0.001928687	0.007807729
ENSMMUG00000000237	TBC1 domain family member 1 [Source:VGNC Symbol;Acc:VGNC:78103]	0.500242599	0.008775116	0.028697167
ENSMMUG00000055079		0.484398932	0.003265987	0.012232623
ENSMMUG00000002755	kelch like family member 5 [Source:VGNC Symbol;Acc:VGNC:74188]	-2.979121082	2.50E-05	0.000164907
ENSMMUG00000002756	WD repeat domain 19 [Source:VGNC Symbol;Acc:VGNC:79132]	-0.490813364	0.000236919	0.001247582
ENSMMUG00000001892	UDP-glucose 6-dehydrogenase [Source:VGNC Symbol;Acc:VGNC:78711]	-0.991338798	2.64E-06	2.18E-05
ENSMMUG00000062569	Metazoan signal recognition particle RNA [Source:RFAM;Acc:RF00017]	-0.905359195	0.012038628	0.037367082
ENSMMUG00000036851	U7 small nuclear RNA [Source:RFAM;Acc:RF00066]	-0.651667311	5.56E-05	0.000338263
ENSMMUG00000012036	RNA binding motif protein 47 [Source:VGNC Symbol;Acc:VGNC:76576]	-0.827292108	3.30E-10	5.72E-09
ENSMMUG00000012561	NOP2/Sun RNA methyltransferase family member 7 [Source:VGNC Symbol;Acc:VGNC:75463]	-1.119457261	5.91E-09	8.27E-08
ENSMMUG00000055441		1.843757245	0.000288799	0.001483997
ENSMMUG00000051296	paired like homeobox 2B [Source:NCBI gene;Acc:701940]	-1.196142987	0.002586634	0.010064164
ENSMMUG00000017733	ATPase phospholipid transporting 8A1 [Source:VGNC Symbol;Acc:VGNC:70061]	-0.386393205	0.006433644	0.02192975
ENSMMUG00000018625	NIPA like domain containing 1 [Source:NCBI gene;Acc:704998]	-1.146159422	4.22E-07	4.14E-06
ENSMMUG00000006703	solute carrier family 10 member 4 [Source:VGNC Symbol;Acc:VGNC:77553]	-0.918333625	0.001016534	0.00447562
ENSMMUG00000006704	zygote arrest 1 [Source:VGNC Symbol;Acc:VGNC:79251]	-1.319715333	1.77E-06	1.51E-05
ENSMMUG00000014868	OCIA domain containing 1 [Source:VGNC Symbol;Acc:VGNC:75409]	0.94358744	7.31E-07	6.82E-06
ENSMMUG00000045266	Metazoan signal recognition particle RNA [Source:RFAM;Acc:RF00017]	2.564735775	0.000874704	0.003926367
ENSMMUG00000058229	U6 spliceosomal RNA [Source:RFAM;Acc:RF00026]	2.494382348	0.00113049	0.004903453
ENSMMUG00000008514	lin-54 DREAM MuvB core complex component [Source:VGNC Symbol;Acc:VGNC:74123]	1.179506878	0.012943427	0.039760281
ENSMMUG00000015777	heterogeneous nuclear ribonucleoprotein D like [Source:VGNC Symbol;Acc:VGNC:81186]	-0.55847203	0.000813303	0.003696903
ENSMMUG00000021988	N-alpha-acetyltransferase 11, NatA catalytic subunit [Source:NCBI gene;Acc:696758]	1.286789164	9.87E-12	2.22E-10
ENSMMUG00000007366		1.391577668	7.88E-14	2.39E-12
ENSMMUG00000002564	Fraser extracellular matrix complex subunit 1 [Source:VGNC Symbol;Acc:VGNC:72743]	0.860898059	2.64E-06	2.18E-05
ENSMMUG00000062865		-0.559090106	0.001604538	0.006635339
ENSMMUG00000045609	CCR4-NOT transcription complex subunit 6 like [Source:NCBI gene;Acc:697981]	1.938494175	2.44E-13	6.80E-12
ENSMMUG00000019904	cyclin I [Source:VGNC Symbol;Acc:VGNC:70733]	-1.53053726	0.004161289	0.015156819
ENSMMUG00000018186	sosondowah ankyrin repeat domain family member B [Source:VGNC Symbol;Acc:VGNC:78033]	-0.49805876	4.38E-05	0.000272736
ENSMMUG00000020919	shroom family member 3 [Source:VGNC Symbol;Acc:VGNC:77352]	1.178289369	0.001972665	0.007962776
ENSMMUG00000020916	coiled-coil domain containing 158 [Source:NCBI gene;Acc:699735]	1.051301147	1.01E-13	2.99E-12
ENSMMUG00000020913	scavenger receptor class B member 2 [Source:VGNC Symbol;Acc:VGNC:77026]	-0.713868016	0.000100839	0.000574896
ENSMMUG00000020910	nucleoporin 54 [Source:VGNC Symbol;Acc:VGNC:75420]	-0.84271635	0.001027877	0.00451847
ENSMMUG00000017503	SDA1 domain containing 1 [Source:NCBI gene;Acc:700863]	2.450259692	1.24E-06	1.10E-05
ENSMMUG00000017501	N-acylethanolamine acid amidase [Source:VGNC Symbol;Acc:VGNC:75068]	2.051660589	1.68E-09	2.57E-08
ENSMMUG00000015716	protein phosphatase with EF-hand domain 2 [Source:VGNC Symbol;Acc:VGNC:76233]	1.515753045	0.00386823	0.014203766

ENSMMUG00000002153	G3BP stress granule assembly factor 2 [Source:NCBI gene;Acc:701343]	- 3.064708616	0.015023982	0.045004208
ENSMMUG00000003833	Ras association domain family member 6 [Source:VGNC Symbol;Acc:VGNC:76515]	1.786266665	3.27E-06	2.66E-05
ENSMMUG00000003827	albumin [Source:VGNC Symbol;Acc:VGNC:69782]	0.873371634	0.000135025	0.000749588
ENSMMUG00000000790	ankyrin repeat domain 17 [Source:VGNC Symbol;Acc:VGNC:84240]	2.598491089	1.32E-39	5.77E-37
ENSMMUG00000006031	casein kappa [Source:VGNC Symbol;Acc:VGNC:71522]	- 1.850072808	0.010004025	0.031992346
ENSMMUG00000006029	odontogenic, ameloblast associated [Source:NCBI gene;Acc:707906]	0.827801375	6.31E-06	4.83E-05
ENSMMUG00000048823		- 1.160316735	8.03E-10	1.30E-08
ENSMMUG00000004849	casein beta [Source:VGNC Symbol;Acc:VGNC:71523]	- 1.034314978	2.62E-08	3.26E-07
ENSMMUG00000047324	casein alpha s1 [Source:HGNC Symbol;Acc:HGNC:2445]	- 2.005630651	0.000513171	0.002467832
ENSMMUG00000009925	centrosomal protein 135 [Source:VGNC Symbol;Acc:VGNC:71128]	- 0.370191182	0.015206033	0.045443776
ENSMMUG00000001919	clock circadian regulator [Source:VGNC Symbol;Acc:VGNC:71262]	- 1.237018126	2.76E-09	4.09E-08
ENSMMUG00000001918	transmembrane protein 165 [Source:VGNC Symbol;Acc:VGNC:79875]	- 1.852272522	1.83E-07	1.92E-06
ENSMMUG00000010113	steroid 5 alpha-reductase 3 [Source:VGNC Symbol;Acc:VGNC:82246]	- 1.523964937	4.18E-07	4.11E-06
ENSMMUG00000046843	Metazoan signal recognition particle RNA [Source:RFAM;Acc:RF00017]	0.795041561	0.00621705	0.021279398
ENSMMUG00000059659		-1.33194235	5.75E-06	4.44E-05
ENSMMUG00000018943	spermatogenesis associated 18 [Source:VGNC Symbol;Acc:VGNC:78013]	- 0.644106736	0.016097659	0.047724624
ENSMMUG00000018939	leucine rich repeat containing 66 [Source:VGNC Symbol;Acc:VGNC:74415]	-0.64389534	0.001249609	0.005360436
ENSMMUG00000008341	Rho GTPase activating protein 24 [Source:VGNC Symbol;Acc:VGNC:69904]	-0.9399431	0.005599081	0.019458714
ENSMMUG00000003726	protein tyrosine phosphatase non-receptor type 13 [Source:VGNC Symbol;Acc:VGNC:76599]	- 0.630764893	0.004897976	0.017423154
ENSMMUG00000014076	AF4/FMR2 family member 1 [Source:VGNC Symbol;Acc:VGNC:69817]	1.071776731	0.007261961	0.024409032
ENSMMUG00000051677		1.093271109	4.12E-15	1.50E-13
ENSMMUG00000014080	kelch like family member 8 [Source:VGNC Symbol;Acc:VGNC:74076]	- 0.839732383	0.003958153	0.014499786
ENSMMUG00000019040	hydroxysteroid 17-beta dehydrogenase 13 [Source:VGNC Symbol;Acc:VGNC:73417]	1.254474547	8.90E-14	2.68E-12
ENSMMUG00000019041	hydroxysteroid 17-beta dehydrogenase 11 [Source:VGNC Symbol;Acc:VGNC:73431]	1.090118986	7.22E-05	0.000426687
ENSMMUG00000008795	polycystin 2, transient receptor potential cation channel [Source:NCBI gene;Acc:702179]	- 1.253596111	1.03E-09	1.63E-08
ENSMMUG00000008797	ATP binding cassette subfamily G member 2 (Junior blood group) [Source:NCBI gene;Acc:574307]	- 1.325367304	6.23E-17	2.83E-15
ENSMMUG00000028039	U6 spliceosomal RNA [Source:RFAM;Acc:RF00026]	- 1.834290264	0.009583716	0.03085226
ENSMMUG00000010314	HECT and RLD domain containing E3 ubiquitin protein ligase family member 6 [Source:VGNC Symbol;Acc:VGNC:73238]	0.802930523	0.0080132	0.026558521
ENSMMUG00000010316	HECT and RLD domain containing E3 ubiquitin protein ligase 5 [Source:VGNC Symbol;Acc:VGNC:73226]	2.236623943	5.22E-08	6.12E-07
ENSMMUG00000010318	phosphatidylinositol glycan anchor biosynthesis class Y [Source:NCBI gene;Acc:100426107]	2.600961999	3.34E-12	8.02E-11
ENSMMUG00000058698		- 1.879874648	5.62E-05	0.000341428
ENSMMUG00000010138	HECT and RLD domain containing E3 ubiquitin protein ligase 3 [Source:VGNC Symbol;Acc:VGNC:73365]	0.380618983	0.002897206	0.011082247
ENSMMUG00000058544	nucleosome assembly protein 1 like 5 [Source:NCBI gene;Acc:703223]	0.65869106	1.46E-06	1.27E-05
ENSMMUG00000010141	family with sequence similarity 13 member A [Source:VGNC Symbol;Acc:VGNC:72299]	0.602423415	1.54E-06	1.34E-05
ENSMMUG00000057606	GPRIN family member 3 [Source:NCBI gene;Acc:703567]	- 1.746407507	1.15E-18	6.36E-17
ENSMMUG00000049396	Metazoan signal recognition particle RNA [Source:RFAM;Acc:RF00017]	- 1.315079851	0.002630259	0.010200255
ENSMMUG00000022003	multimerin 1 [Source:VGNC Symbol;Acc:VGNC:74904]	1.910881321	2.57E-15	9.57E-14

ENSMMUG00000022246	PDZ and LIM domain 5 [Source:VGNC Symbol;Acc:VGNC:75816]	0.918039634	5.52E-05	0.00033654
ENSMMUG00000060760		1.450845419	1.89E-08	2.43E-07
ENSMMUG00000006309	unc-5 netrin receptor C [Source:VGNC Symbol;Acc:VGNC:78723]	-1.99610831	0.000972037	0.004297251
ENSMMUG00000011702	tetraspanin 5 [Source:VGNC Symbol;Acc:VGNC:79086]	-1.116487085	4.94E-05	0.000303631
ENSMMUG00000063972		-0.789184049	0.000998071	0.00440401
ENSMMUG00000040764	alcohol dehydrogenase 1A [Source:NCBI gene;Acc:100424103]	-0.933559498	0.000110009	0.00062229
ENSMMUG00000018738	alcohol dehydrogenase 1B (class I), beta polypeptide [Source:NCBI gene;Acc:707908]	-0.997255619	5.18E-08	6.09E-07
ENSMMUG00000061366		0.771988096	1.19E-07	1.31E-06
ENSMMUG00000016880	solute carrier family 39 member 8 [Source:VGNC Symbol;Acc:VGNC:77541]	-3.312930333	9.39E-22	6.93E-20
ENSMMUG00000037551	Metazoan signal recognition particle RNA [Source:RFAM;Acc:RF00017]	-3.788935479	2.00E-23	1.75E-21
ENSMMUG00000003736	nuclear factor kappa B subunit 1 [Source:VGNC Symbol;Acc:VGNC:75339]	-3.676192902	1.07E-21	7.80E-20
ENSMMUG00000003739	mannosidase beta [Source:VGNC Symbol;Acc:VGNC:74486]	1.454243808	2.29E-05	0.000152795
ENSMMUG00000011206	solute carrier family 9 member B1 [Source:NCBI gene;Acc:710949]	0.419657747	0.016184918	0.047922529
ENSMMUG00000019083	solute carrier family 9 member B2 [Source:VGNC Symbol;Acc:VGNC:77773]	0.577263645	0.001264263	0.00541006
ENSMMUG00000019085	3-hydroxybutyrate dehydrogenase 2 [Source:VGNC Symbol;Acc:VGNC:70161]	1.228029753	5.61E-26	6.85E-24
ENSMMUG00000019089	centromere protein E [Source:VGNC Symbol;Acc:VGNC:71008]	1.206481689	1.63E-24	1.62E-22
ENSMMUG00000010941	Rho guanine nucleotide exchange factor 38 [Source:VGNC Symbol;Acc:VGNC:82087]	-0.460080344	0.000636399	0.00297375
ENSMMUG00000009066	60S ribosomal protein L26 [Source:NCBI gene;Acc:696134]	1.896408483	0.009596028	0.0308595
ENSMMUG00000060432		-0.99137438	0.014289988	0.043252428
ENSMMUG00000052609	ribosomal protein L34 [Source:NCBI gene;Acc:696636]	-2.075175983	2.20E-08	2.78E-07
ENSMMUG00000011902	oligosaccharyltransferase complex non-catalytic subunit [Source:NCBI gene;Acc:697283]	-1.629309351	7.03E-08	8.09E-07
ENSMMUG00000055759	Metazoan signal recognition particle RNA [Source:RFAM;Acc:RF00017]	-1.551380341	3.50E-06	2.83E-05
ENSMMUG00000017703	collagen type XXV alpha 1 chain [Source:VGNC Symbol;Acc:VGNC:81374]	-2.815829354	5.11E-07	4.92E-06
ENSMMUG00000010043	epidermal growth factor [Source:VGNC Symbol;Acc:VGNC:72171]	0.647811464	0.014380091	0.043506368
ENSMMUG00000062794		2.480275169	8.93E-14	2.68E-12
ENSMMUG00000008633	ankyrin 2 [Source:NCBI gene;Acc:702180]	-0.835932852	7.50E-05	0.000440758
ENSMMUG00000001752	transmembrane protein 155 [Source:NCBI gene;Acc:707055]	1.96881723	0.005503552	0.019198852
ENSMMUG00000009816	exosome component 9 [Source:VGNC Symbol;Acc:VGNC:72477]	2.027878491	8.43E-06	6.25E-05
ENSMMUG00000061098		1.836225931	3.14E-28	4.74E-26
ENSMMUG00000007422	spermatogenesis associated 5 [Source:VGNC Symbol;Acc:VGNC:77952]	1.402887512	6.71E-19	3.75E-17
ENSMMUG00000001848	sprouty RTK signaling antagonist 1 [Source:VGNC Symbol;Acc:VGNC:77867]	1.176210989	6.36E-17	2.88E-15
ENSMMUG00000011810	FAT atypical cadherin 4 [Source:VGNC Symbol;Acc:VGNC:72552]	-1.298222595	0.000746014	0.0034221
ENSMMUG00000051329		1.57137543	2.22E-05	0.000148427
ENSMMUG00000056698		1.587960391	0.005551548	0.019331849
ENSMMUG00000015244	uncoupling protein 1 [Source:VGNC Symbol;Acc:VGNC:78708]	2.304210977	0.002377157	0.009347974
ENSMMUG00000010444	ring finger protein 150 [Source:VGNC Symbol;Acc:VGNC:76805]	2.792936	0.010209326	0.032537587
ENSMMUG00000060157	zinc finger protein 330 [Source:NCBI gene;Acc:699364]	0.71465411	5.46E-05	0.00033288
ENSMMUG00000003697	inositol polyphosphate-4-phosphatase type II B [Source:VGNC Symbol;Acc:VGNC:73748]	0.820424156	2.19E-05	0.00014684
ENSMMUG00000055522		-0.778869383	0.006387064	0.021797469

ENSMMUG00000044429	glycophorin A (MNS blood group) [Source:NCBI gene;Acc:574101]	2.192104679	1.93E-15	7.28E-14
ENSMMUG00000014173		-1.12513381	0.001051057	0.004608811
		-		
ENSMMUG00000007968	hedgehog interacting protein [Source:VGNC Symbol;Acc:VGNC:73169]	0.938781934	1.11E-05	7.95E-05
ENSMMUG00000005380	zinc finger protein 827 [Source:NCBI gene;Acc:706018]	1.803538101	1.92E-12	4.75E-11
		-		
ENSMMUG00000014955	nuclear receptor subfamily 3 group C member 2 [Source:VGNC Symbol;Acc:VGNC:75516]	1.025702875	0.002959957	0.011273361
ENSMMUG00000002211	doublecortin like kinase 2 [Source:VGNC Symbol;Acc:VGNC:71670]	1.110809626	2.73E-06	2.25E-05
		-		
ENSMMUG00000059101		1.654760072	2.06E-05	0.000139254
		-		
ENSMMUG00000018490	ADP ribosylation factor interacting protein 1 [Source:VGNC Symbol;Acc:VGNC:69878]	0.982157138	7.87E-08	8.99E-07
		-		
ENSMMUG00000039869		0.770648737	1.15E-06	1.02E-05
		-		
ENSMMUG00000009001	transmembrane 131 like [Source:VGNC Symbol;Acc:VGNC:79781]	0.910333135	0.004959227	0.017601429
ENSMMUG00000057643		0.464757687	0.00495786	0.017601029
ENSMMUG00000052999	lecithin retinol acyltransferase [Source:NCBI gene;Acc:698871]	0.785932065	5.44E-09	7.69E-08
ENSMMUG00000064074		0.782876938	4.28E-06	3.40E-05
ENSMMUG00000021147	RNA binding motif protein 46 [Source:VGNC Symbol;Acc:VGNC:76555]	0.588248969	0.000723312	0.003324485
		-		
ENSMMUG00000005447	guanylate cyclase 1 soluble subunit beta 1 [Source:VGNC Symbol;Acc:VGNC:73328]	0.525696362	0.006137605	0.021064032
		-		
ENSMMUG00000057700	acid sensing ion channel subunit family member 5 [Source:NCBI gene;Acc:699617]	0.576619724	3.94E-05	0.000248591
ENSMMUG00000022018	tryptophan 2,3-dioxygenase [Source:VGNC Symbol;Acc:VGNC:78298]	-0.53069103	0.001469162	0.006151705
ENSMMUG00000064707		1.629419804	1.48E-08	1.93E-07
ENSMMUG00000053790		2.964170234	4.13E-42	2.14E-39
ENSMMUG00000010581	Rap guanine nucleotide exchange factor 2 [Source:VGNC Symbol;Acc:VGNC:76493]	1.504918044	0.009602335	0.030862606
		-		
ENSMMUG00000015799	tolloid like 1 [Source:VGNC Symbol;Acc:VGNC:78369]	0.776194929	0.010401804	0.033045877
ENSMMUG00000006851	SPARC (osteonectin), cwcv and kazal like domains proteoglycan 3 [Source:NCBI gene;Acc:705964]	4.50933754	6.16E-32	1.39E-29
ENSMMUG00000001879	annexin A10 [Source:VGNC Symbol;Acc:VGNC:69945]	0.63624317	8.20E-05	0.00047831
ENSMMUG00000001880	DExD/H-box helicase 60 [Source:VGNC Symbol;Acc:VGNC:71711]	0.45612019	0.001426452	0.005999741
		-		
ENSMMUG00000003634	DExD/H-box 60 like [Source:VGNC Symbol;Acc:VGNC:71720]	1.612687674	3.87E-10	6.62E-09
		-		
ENSMMUG00000016767	polypeptide N-acetylgalactosaminyltransferase like 6 [Source:VGNC Symbol;Acc:VGNC:72813]	1.142273099	0.000342312	0.001720515
ENSMMUG00000013730	WD repeat domain 17 [Source:NCBI gene;Acc:700546]	0.307416036	0.009228786	0.029915687
		-		
ENSMMUG00000064304		0.391162115	0.007240746	0.024349395
		-		
ENSMMUG00000058974		1.176834953	0.000910902	0.004062844
		-		
ENSMMUG00000049670		3.708488341	0.003865836	0.014198696
		-		
ENSMMUG00000034845	U4 spliceosomal RNA [Source:RFAM;Acc:RF00015]	2.352198207	0.014214696	0.043061716
ENSMMUG00000011633	kallikrein B1 [Source:VGNC Symbol;Acc:VGNC:74195]	2.43703542	5.15E-06	4.00E-05
ENSMMUG00000011634	coagulation factor XI [Source:VGNC Symbol;Acc:VGNC:72460]	2.247450463	0.000657036	0.003055032
		-		
ENSMMUG00000018051	FAT atypical cadherin 1 [Source:VGNC Symbol;Acc:VGNC:72617]	0.678384693	1.01E-07	1.13E-06
ENSMMUG00000058448		0.577116122	0.003544388	0.013152409
ENSMMUG00000050465	ZFP42 zinc finger protein [Source:NCBI gene;Acc:707621]	2.112439075	1.11E-12	2.84E-11
		-		
ENSMMUG00000054799		1.446199596	0.007625611	0.025460464

ENSMMUG00000064721		-2.332412703	0.000962987	0.00426531
ENSMMUG00000052287		-2.120518987	0.000138999	0.000770126
ENSMMUG00000044628		-1.89055488	0.000299388	0.001531921
ENSMMUG00000015797	disco interacting protein 2 homolog A [Source:VGNC Symbol;Acc:VGNC:71641]	0.679376623	2.94E-05	0.000190711
ENSMMUG00000052777		1.57192545	0.001277137	0.005456822
ENSMMUG00000004472	collagen type XVIII alpha 1 chain [Source:VGNC Symbol;Acc:VGNC:81291]	1.765341184	0.006494194	0.022114638
ENSMMUG00000010695	adenosine deaminase RNA specific B1 [Source:VGNC Symbol;Acc:VGNC:69503]	-1.419231962	1.16E-05	8.32E-05
ENSMMUG00000000953	family with sequence similarity 207 member A [Source:VGNC Symbol;Acc:VGNC:72273]	0.979887192	2.70E-05	0.000176368
ENSMMUG00000000951	integrin subunit beta 2 [Source:VGNC Symbol;Acc:VGNC:73786]	1.901950427	1.42E-24	1.44E-22
ENSMMUG00000049755	leucine rich repeat containing 3 [Source:NCBI gene;Acc:712418]	2.502629739	2.99E-06	2.44E-05
ENSMMUG00000004175	transient receptor potential cation channel subfamily M member 2 [Source:VGNC Symbol;Acc:VGNC:78655]	0.80088537	0.011766225	0.036682476
ENSMMUG00000004173	phosphofructokinase, liver type [Source:VGNC Symbol;Acc:VGNC:81737]	-0.546247466	0.005567036	0.019371351
ENSMMUG00000010648	DNA methyltransferase 3 like [Source:NCBI gene;Acc:722184]	-2.239440301	1.81E-14	6.06E-13
ENSMMUG00000010647	inducible T cell costimulator ligand [Source:NCBI gene;Acc:722191]	-1.267040072	0.000736291	0.003379709
ENSMMUG00000005272	glutamine amidotransferase like class 1 domain containing 3A [Source:NCBI gene;Acc:713055]	-0.355563598	0.009256598	0.029985047
ENSMMUG00000012304	ribosomal RNA processing 1 [Source:VGNC Symbol;Acc:VGNC:77050]	-0.603164256	0.004405987	0.015919907
ENSMMUG00000062457	pyridoxal kinase [Source:NCBI gene;Acc:713564]	-0.694995872	0.002151388	0.008592898
ENSMMUG00000000104	ribosomal RNA processing 1B [Source:VGNC Symbol;Acc:VGNC:77089]	-0.897772198	1.74E-09	2.66E-08
ENSMMUG00000009288	PBX/knotted 1 homeobox 1 [Source:VGNC Symbol;Acc:VGNC:76150]	-0.86711136	0.000935737	0.00415511
ENSMMUG00000062729	WD repeat domain 4 [Source:NCBI gene;Acc:714230]	-0.340216128	0.016979191	0.049884599
ENSMMUG00000018081	ubiquitin associated and SH3 domain containing A [Source:VGNC Symbol;Acc:VGNC:78687]	-0.6164594	0.005895142	0.020326425
ENSMMUG00000018079	transmembrane serine protease 3 [Source:VGNC Symbol;Acc:VGNC:78576]	-0.712711785	0.001615898	0.00667641
ENSMMUG00000018058	ATP binding cassette subfamily G member 1 [Source:VGNC Symbol;Acc:VGNC:69584]	-1.080854676	0.000311127	0.001583065
ENSMMUG00000018057	uromodulin like 1 [Source:VGNC Symbol;Acc:VGNC:78717]	-1.117753383	7.94E-06	5.93E-05
ENSMMUG00000018053	zinc finger and BTB domain containing 21 [Source:VGNC Symbol;Acc:VGNC:78892]	-0.565303771	0.000100532	0.000573773
ENSMMUG00000018043	receptor interacting serine/threonine kinase 4 [Source:VGNC Symbol;Acc:VGNC:76882]	-2.865778886	0.016554315	0.048758908
ENSMMUG00000015329	MX dynamin like GTPase 1 [Source:VGNC Symbol;Acc:VGNC:75086]	1.616470354	3.76E-05	0.000238283
ENSMMUG00000044257	MX dynamin like GTPase 2 [Source:VGNC Symbol;Acc:VGNC:81723]	2.374155435	3.27E-14	1.06E-12
ENSMMUG00000004257	beta-secretase 2 [Source:VGNC Symbol;Acc:VGNC:70216]	1.930784458	2.82E-12	6.86E-11
ENSMMUG00000008498	proteasome assembly chaperone 1 [Source:VGNC Symbol;Acc:VGNC:76347]	-0.383477233	0.013950626	0.042374755
ENSMMUG00000010296	ETS transcription factor ERG [Source:VGNC Symbol;Acc:VGNC:72427]	2.948953512	0.005320572	0.018657258
ENSMMUG00000056593		2.78174763	0.000304634	0.001554881
ENSMMUG00000017505	tetratricopeptide repeat domain 3 [Source:VGNC Symbol;Acc:VGNC:81785]	0.526013569	4.16E-06	3.31E-05
ENSMMUG00000062048		0.707726367	1.61E-07	1.72E-06
ENSMMUG00000015566	holocarboxylase synthetase [Source:VGNC Symbol;Acc:VGNC:73477]	-0.528548021	0.004506818	0.016229869
ENSMMUG00000010336	DOP1 leucine zipper like protein B [Source:VGNC Symbol;Acc:VGNC:71832]	0.642565641	1.83E-11	3.91E-10
ENSMMUG00000054417		-1.46798637	8.26E-09	1.11E-07

ENSMMUG00000007626	intersectin 1 [Source:VGNC Symbol;Acc:VGNC:73806]	-1.061376622	4.07E-05	0.00025562
ENSMMUG00000005511	SON DNA binding protein [Source:VGNC Symbol;Acc:VGNC:77689]	1.436419561	4.20E-07	4.13E-06
ENSMMUG00000022702	phosphoribosylglycinamide formyltransferase, phosphoribosylglycinamide synthetase, phosphoribosylaminoimidazole synthetase [Source:VGNC Symbol;Acc:VGNC:72882]	0.839131171	0.005668341	0.019658895
ENSMMUG00000005509	transmembrane protein 50B [Source:NCBI gene;Acc:699352]	-0.431970451	0.000151162	0.000831928
ENSMMUG00000005508	interferon gamma receptor 2 [Source:VGNC Symbol;Acc:VGNC:73569]	-0.336029079	0.002557197	0.009963762
ENSMMUG00000003961	interferon alpha and beta receptor subunit 1 [Source:VGNC Symbol;Acc:VGNC:73566]	-0.614380719	0.001755288	0.00717312
ENSMMUG00000003956	interferon alpha and beta receptor subunit 2 [Source:VGNC Symbol;Acc:VGNC:81330]	0.441256806	0.007360317	0.024680468
ENSMMUG00000065005	Metazoan signal recognition particle RNA [Source:RFAM;Acc:RF00017]	0.974188237	0.00122433	0.005263266
ENSMMUG00000052780		0.91400556	1.90E-07	1.99E-06
ENSMMUG00000027541	Small nucleolar RNA SNORA70 [Source:RFAM;Acc:RF00156]	3.060626222	0.000263078	0.001371941
ENSMMUG00000004624	synaptojanin 1 [Source:VGNC Symbol;Acc:VGNC:78071]	-0.69233611	3.06E-05	0.000198259
ENSMMUG00000010304	cilia and flagella associated protein 298 [Source:NCBI gene;Acc:700721]	-0.752660907	1.45E-07	1.56E-06
ENSMMUG00000048824	eva-1 homolog C [Source:NCBI gene;Acc:708681]	1.259611216	1.95E-18	1.06E-16
ENSMMUG00000059707	MIS18 kinetochore protein A [Source:NCBI gene;Acc:709275]	-1.705062839	7.22E-05	0.000426687
ENSMMUG00000056363		-1.903925906	1.77E-05	0.000120951
ENSMMUG00000011095	BTB domain and CNC homolog 1 [Source:VGNC Symbol;Acc:VGNC:70217]	1.539977394	0.011937717	0.037126325
ENSMMUG00000033200	Small nucleolar RNA U3 [Source:RFAM;Acc:RF00012]	2.435571022	0.012039112	0.037367082
ENSMMUG00000061208	MAP3K7 C-terminal like [Source:NCBI gene;Acc:713717]	1.621708883	4.41E-24	4.17E-22
ENSMMUG00000064638	Y RNA [Source:RFAM;Acc:RF00019]	2.470708566	0.004555881	0.016385514
ENSMMUG00000017605	mitochondrial ribosomal protein L39 [Source:VGNC Symbol;Acc:VGNC:74737]	1.161545234	6.01E-09	8.39E-08
ENSMMUG00000018743	ubiquitin specific peptidase 25 [Source:VGNC Symbol;Acc:VGNC:78744]	3.237660051	0.000352714	0.001760809
ENSMMUG00000060576		1.29606748	4.96E-07	4.79E-06
ENSMMUG00000057702		1.208602813	9.47E-07	8.60E-06
ENSMMUG00000060168		-1.119267921	1.78E-05	0.000121921
ENSMMUG00000052328	FAM20C golgi associated secretory pathway kinase [Source:NCBI gene;Acc:695088]	-2.07221767	2.47E-06	2.05E-05
ENSMMUG00000018261	dynein axonemal assembly factor 5 [Source:VGNC Symbol;Acc:VGNC:71907]	-0.706583746	0.000611716	0.002886304
ENSMMUG00000017056	Sad1 and UNC84 domain containing 1 [Source:VGNC Symbol;Acc:VGNC:78161]	3.344052302	2.92E-09	4.31E-08
ENSMMUG00000007206	chromosome 3 C7orf50 homolog [Source:VGNC Symbol;Acc:VGNC:70609]	2.174763782	0.000449793	0.002196184
ENSMMUG00000003283	extracellular leucine rich repeat and fibronectin type III domain containing 1 [Source:VGNC Symbol;Acc:VGNC:72204]	1.132100639	1.03E-10	1.96E-09
ENSMMUG00000055562	Metazoan signal recognition particle RNA [Source:RFAM;Acc:RF00017]	-1.455777999	0.009990846	0.031957489
ENSMMUG00000022624	putative speedy protein E16 [Source:NCBI gene;Acc:718058]	-1.330446331	9.51E-09	1.28E-07
ENSMMUG00000015546	chromosome 3 C7orf26 homolog [Source:VGNC Symbol;Acc:VGNC:70604]	0.731539943	0.009550066	0.030765122
ENSMMUG00000021839	Grid2 interacting protein [Source:VGNC Symbol;Acc:VGNC:73191]	0.853315787	3.95E-05	0.000248847
ENSMMUG00000021075	Rac family small GTPase 1 [Source:NCBI gene;Acc:717755]	1.247536142	6.69E-16	2.69E-14
ENSMMUG00000003167	cytohesin 3 [Source:VGNC Symbol;Acc:VGNC:71829]	1.544203099	3.46E-14	1.12E-12
ENSMMUG00000030422	radial spoke head 10 homolog B [Source:NCBI gene;Acc:100424218]	-0.744750596	5.56E-07	5.33E-06
ENSMMUG00000007970	CCZ1 homolog, vacuolar protein trafficking and biogenesis associated [Source:NCBI gene;Acc:718128]	1.305790309	1.16E-07	1.28E-06
ENSMMUG00000015477	lemur tyrosine kinase 2 [Source:VGNC Symbol;Acc:VGNC:74309]	0.854948938	1.48E-07	1.58E-06
ENSMMUG00000015479	tectonin beta-propeller repeat containing 1 [Source:VGNC Symbol;Acc:VGNC:79348]	1.073459667	1.16E-25	1.34E-23
ENSMMUG00000013276	BAR/IMD domain containing adaptor protein 2 like 1 [Source:VGNC Symbol;Acc:VGNC:70109]	-0.587784806	0.005764251	0.01994377

ENSMMUG00000023318	transformation/transcription domain associated protein [Source:VGNC Symbol;Acc:VGNC:78657]	0.391225145	0.000502211	0.002421767
ENSMMUG00000040393	zinc finger protein 655 [Source:NCBI gene;Acc:718811]	3.03922841	7.45E-12	1.71E-10
ENSMMUG00000055483	transmembrane protein 225B [Source:NCBI gene;Acc:718838]	2.296411849	0.002231183	0.008855895
ENSMMUG00000022728	zinc finger and SCAN domain containing 25 [Source:VGNC Symbol;Acc:VGNC:79344]	1.744818351	7.31E-09	1.00E-07
ENSMMUG00000022730	cytochrome P450, family 3, subfamily A, polypeptide 5 [Source:NCBI gene;Acc:678692]	0.967655846	4.27E-07	4.19E-06
ENSMMUG00000022490	cytochrome P450, family 3, subfamily A, polypeptide 43 [Source:NCBI gene;Acc:709770]	-0.687744837	1.33E-07	1.44E-06
ENSMMUG00000022502	tripartite motif containing 4 [Source:VGNC Symbol;Acc:VGNC:79021]	-0.697405045	0.003458391	0.01286052
ENSMMUG00000022504	alpha-2-glycoprotein 1, zinc-binding [Source:VGNC Symbol;Acc:VGNC:70201]	0.578695846	0.000727804	0.003344033
ENSMMUG00000013447	adaptor related protein complex 4 subunit mu 1 [Source:VGNC Symbol;Acc:VGNC:69974]	1.017781924	0.000796458	0.003635655
ENSMMUG00000013450	TATA-box binding protein associated factor 6 [Source:VGNC Symbol;Acc:VGNC:78093]	2.395956154	1.65E-10	3.03E-09
ENSMMUG00000046663	Metazoan signal recognition particle RNA [Source:RFAM;Acc:RF00017]	-1.037766093	3.72E-06	2.99E-05
ENSMMUG00000048302	ArfGAP with FG repeats 2 [Source:VGNC Symbol;Acc:VGNC:80329]	-1.13136939	3.56E-15	1.31E-13
ENSMMUG00000015311	leucine rich repeats and calponin homology domain containing 4 [Source:VGNC Symbol;Acc:VGNC:74441]	-1.21745993	8.29E-21	5.56E-19
ENSMMUG00000015309	F-box protein 24 [Source:VGNC Symbol;Acc:VGNC:72495]	-1.107328075	3.34E-15	1.23E-13
ENSMMUG00000002947	procollagen C-endopeptidase enhancer [Source:VGNC Symbol;Acc:VGNC:75782]	-1.132373263	7.28E-19	4.05E-17
ENSMMUG00000002949	motile sperm domain containing 3 [Source:VGNC Symbol;Acc:VGNC:74823]	2.156630219	6.71E-11	1.31E-09
ENSMMUG00000002950	transferrin receptor 2 [Source:VGNC Symbol;Acc:VGNC:81770]	2.212937813	3.87E-13	1.06E-11
ENSMMUG00000004080	G protein subunit beta 2 [Source:VGNC Symbol;Acc:VGNC:81318]	-1.983472351	0.0024664	0.009659545
ENSMMUG00000004082	GRB10 interacting GYF protein 1 [Source:VGNC Symbol;Acc:VGNC:73158]	-0.982655211	0.00874842	0.028643262
ENSMMUG00000022840	zonadhesin [Source:VGNC Symbol;Acc:VGNC:81797]	-0.66515603	0.00214169	0.008561267
ENSMMUG00000052734		2.182586035	0.011689925	0.036494251
ENSMMUG00000060459		0.527687921	6.27E-05	0.000376653
ENSMMUG00000010329	SH2B adaptor protein 2 [Source:VGNC Symbol;Acc:VGNC:77257]	3.62859814	0.009818195	0.03145546
ENSMMUG00000063570	Metazoan signal recognition particle RNA [Source:RFAM;Acc:RF00017]	-3.154478916	0.001517994	0.006325952
ENSMMUG00000011367	BUD23 rRNA methyltransferase and ribosome maturation factor [Source:VGNC Symbol;Acc:VGNC:70325]	-3.321507898	0.000997403	0.004402451
ENSMMUG00000056841		1.305007327	0.001219346	0.005246668
ENSMMUG00000050132		1.28080661	1.99E-08	2.54E-07
ENSMMUG00000056980		1.831600367	0.002790301	0.010728927
ENSMMUG00000001225	calneuron 1 [Source:VGNC Symbol;Acc:VGNC:82078]	-1.390969346	0.000170154	0.000924472
ENSMMUG00000061002		-0.78536878	0.005713684	0.019798121
ENSMMUG00000021235	vitamin K epoxide reductase complex subunit 1 like 1 [Source:VGNC Symbol;Acc:VGNC:79322]	-1.026868897	0.004516183	0.016259419
ENSMMUG00000049696		-0.286157385	0.003938427	0.014442616
ENSMMUG00000051107		-1.251386666	0.002953339	0.011257046
ENSMMUG00000060184	SEC61 translocon subunit gamma [Source:NCBI gene;Acc:716898]	3.98994446	8.98E-07	8.19E-06
ENSMMUG00000056368		0.368431946	0.001340801	0.005682095
ENSMMUG00000019991	dopa decarboxylase [Source:VGNC Symbol;Acc:VGNC:71755]	1.069663968	3.67E-25	3.99E-23
ENSMMUG00000049620		-0.834260881	0.014171615	0.042968335
ENSMMUG00000044759		-1.086355331	2.75E-10	4.86E-09

ENSMMUG00000043922	spermatogenesis associated 48 [Source:VGNC Symbol;Acc:VGNC:81762]	-0.786574097	6.23E-11	1.22E-09
ENSMMUG00000019393	tensin 3 [Source:VGNC Symbol;Acc:VGNC:79324]	-3.308229035	0.000231402	0.001222205
ENSMMUG00000050300		-1.405204263	0.000166529	0.000906961
ENSMMUG00000000962	H2A.Z variant histone 2 [Source:VGNC Symbol;Acc:VGNC:81324]	-0.704059471	0.002515853	0.00981904
ENSMMUG00000064180		-0.553030333	0.01663505	0.048945301
ENSMMUG00000057027		0.73057842	0.016933951	0.049782975
ENSMMUG00000002433	calcium/calmodulin dependent protein kinase II beta [Source:VGNC Symbol;Acc:VGNC:70524]	1.422312175	0.005212537	0.018338036
ENSMMUG00000002430	YKT6 v-SNARE homolog [Source:VGNC Symbol;Acc:VGNC:81796]	1.466056067	0.002122888	0.008500643
ENSMMUG00000002427	glucokinase [Source:VGNC Symbol;Acc:VGNC:72901]	1.484207007	2.47E-14	8.18E-13
ENSMMUG00000002411	drebrin like [Source:NCBI gene;Acc:720621]	1.637173193	4.90E-14	1.56E-12
ENSMMUG00000002426	myosin light chain 7 [Source:VGNC Symbol;Acc:VGNC:75002]	1.533442178	6.42E-21	4.37E-19
ENSMMUG00000002423	DNA polymerase delta 2, accessory subunit [Source:VGNC Symbol;Acc:VGNC:76203]	1.586345646	6.73E-15	2.39E-13
ENSMMUG00000002422	AE binding protein 1 [Source:VGNC Symbol;Acc:VGNC:80328]	1.606612567	2.84E-10	4.98E-09
ENSMMUG00000019023	cytochrome c oxidase assembly factor 1 homolog [Source:VGNC Symbol;Acc:VGNC:81290]	0.778828479	0.000279744	0.00144488
ENSMMUG00000017382	biliverdin reductase A [Source:VGNC Symbol;Acc:VGNC:70256]	0.699936055	2.07E-09	3.16E-08
ENSMMUG00000019022	serine/threonine kinase 17a [Source:VGNC Symbol;Acc:VGNC:77913]	0.811931327	3.99E-12	9.44E-11
ENSMMUG00000019015	HECT, C2 and WW domain containing E3 ubiquitin protein ligase 1 [Source:VGNC Symbol;Acc:VGNC:73217]	0.702822718	5.84E-09	8.18E-08
ENSMMUG00000057956		0.543353796	0.004955087	0.017600099
ENSMMUG00000024428	Y RNA [Source:RFAM;Acc:RF00019]	1.778894752	0.001673712	0.006890917
ENSMMUG00000053696	Metazoan signal recognition particle RNA [Source:RFAM;Acc:RF00017]	1.542391563	0.000964112	0.004268067
ENSMMUG00000060273	mitochondrial ribosomal protein L32 [Source:NCBI gene;Acc:701820]	1.66995427	7.07E-08	8.12E-07
ENSMMUG00000022451	proteasome 20S subunit alpha 2 [Source:NCBI gene;Acc:701683]	1.201248899	0.004987852	0.017671714
ENSMMUG00000052530		1.98082166	0.000302188	0.001544307
ENSMMUG00000001702	succinyl-CoA:glutarate-CoA transferase [Source:VGNC Symbol;Acc:VGNC:81767]	0.815439045	8.69E-05	0.000502762
ENSMMUG00000006980	septin 7 [Source:VGNC Symbol;Acc:VGNC:81759]	2.226165635	0.001031159	0.004530059
ENSMMUG00000001350	HERPUD family member 2 [Source:VGNC Symbol;Acc:VGNC:73216]	2.252843067	4.54E-26	5.64E-24
ENSMMUG00000058423		2.936621968	7.54E-17	3.38E-15
ENSMMUG00000002886	Bardet-Biedl syndrome 9 [Source:NCBI gene;Acc:708412]	1.361039092	1.86E-08	2.39E-07
ENSMMUG00000050858	Metazoan signal recognition particle RNA [Source:RFAM;Acc:RF00017]	-0.891159943	0.004044881	0.014778888
ENSMMUG00000022484	ITPR interacting domain containing 1 [Source:VGNC Symbol;Acc:VGNC:73667]	-3.022520453	1.71E-05	0.000117396
ENSMMUG00000055809		-3.063479228	3.23E-12	7.76E-11
ENSMMUG00000062454		1.930102653	1.07E-28	1.68E-26
ENSMMUG00000039009		1.866734931	1.25E-13	3.66E-12
ENSMMUG00000032973	mml-mir-550 [Source:miRBase;Acc:MI0007826]	-1.501371146	3.50E-07	3.51E-06
ENSMMUG00000020184	carboxypeptidase vitellogenic like [Source:VGNC Symbol;Acc:VGNC:71406]	-1.547998458	7.81E-06	5.84E-05
ENSMMUG00000039317	cAMP responsive element binding protein 5 [Source:VGNC Symbol;Acc:VGNC:81295]	-2.613427517	3.60E-07	3.60E-06
ENSMMUG00000052176		-3.485591893	0.008305292	0.027403556
ENSMMUG00000065311	translocase of outer mitochondrial membrane 7 [Source:NCBI gene;Acc:705701]	-1.956460059	0.004773793	0.017046829
ENSMMUG00000042472	Metazoan signal recognition particle RNA [Source:RFAM;Acc:RF00017]	0.778646199	0.000917273	0.004079583
ENSMMUG00000007821	ATP binding cassette subfamily B member 5 [Source:VGNC Symbol;Acc:VGNC:80321]	-2.602410361	6.15E-18	3.09E-16

ENSMMUG00000006898	integrin subunit beta 8 [Source:VGNC Symbol;Acc:VGNC:73791]	-2.724232113	1.80E-22	1.42E-20
ENSMMUG00000052574		-1.242479027	4.93E-13	1.34E-11
ENSMMUG00000012203	aryl hydrocarbon receptor [Source:NCBI gene;Acc:714254]	-1.224399093	3.72E-07	3.71E-06
ENSMMUG00000020418	diacylglycerol kinase beta [Source:VGNC Symbol;Acc:VGNC:71782]	-0.771929673	0.013771885	0.041928602
ENSMMUG00000008632	myosin, light chain 6, alkali, smooth muscle and non-muscle-like [Source:NCBI gene;Acc:710137]	-1.109727665	0.00587847	0.020288896
ENSMMUG00000012694	thrombospondin type 1 domain containing 7A [Source:VGNC Symbol;Acc:VGNC:79347]	-1.129431413	0.013173003	0.040341782
ENSMMUG00000041616	G protein subunit gamma 11 [Source:VGNC Symbol;Acc:VGNC:81319]	2.852528284	0.015188239	0.045402829
ENSMMUG00000045513		1.815757197	2.77E-06	2.27E-05
ENSMMUG00000015685	zinc finger protein 804B [Source:VGNC Symbol;Acc:VGNC:81890]	1.389336082	3.08E-19	1.77E-17
ENSMMUG00000022697	STEAP4 metalloreductase [Source:VGNC Symbol;Acc:VGNC:77974]	-1.175129145	2.46E-12	6.02E-11
ENSMMUG00000022696	sorcin [Source:VGNC Symbol;Acc:VGNC:81763]	0.38771509	0.000900819	0.004026835
ENSMMUG00000006766	RUN domain containing 3B [Source:VGNC Symbol;Acc:VGNC:77129]	0.968907752	0.000229412	0.001213063
ENSMMUG00000010788	ATP binding cassette subfamily B member 1 [Source:NCBI gene;Acc:574235]	-0.860372152	7.85E-07	7.28E-06
ENSMMUG00000000480	KIAA1324 like [Source:VGNC Symbol;Acc:VGNC:73882]	2.55794187	0.002612877	0.010152501
ENSMMUG00000022513	glutamate metabotropic receptor 3 [Source:VGNC Symbol;Acc:VGNC:73287]	1.22357661	2.09E-05	0.000140918
ENSMMUG00000001979	semaphorin 3E [Source:VGNC Symbol;Acc:VGNC:77163]	1.631892824	0.010416484	0.033077527
ENSMMUG00000018652	hepatocyte growth factor [Source:VGNC Symbol;Acc:VGNC:73373]	-0.505036685	0.000631269	0.002954283
ENSMMUG00000053516		1.26514001	8.21E-05	0.00047831
ENSMMUG00000064838	G protein subunit alpha transducin 3 [Source:NCBI gene;Acc:708828]	0.635540085	0.002203764	0.008769405
ENSMMUG00000001801	membrane associated guanylate kinase, WW and PDZ domain containing 2 [Source:VGNC Symbol;Acc:VGNC:74312]	0.558685449	0.000769156	0.003517896
ENSMMUG00000049939		-0.940498023	1.15E-09	1.81E-08
ENSMMUG00000009006	round spermatid basic protein 1 like [Source:VGNC Symbol;Acc:VGNC:77097]	-1.14320623	1.53E-10	2.84E-09
ENSMMUG00000012427	F-box and leucine rich repeat protein 13 [Source:VGNC Symbol;Acc:VGNC:81312]	-0.606728841	5.47E-05	0.000333299
ENSMMUG00000022727	lysine methyltransferase 2E [Source:VGNC Symbol;Acc:VGNC:74073]	-2.854253663	0.000514816	0.002473196
ENSMMUG00000022732	SRSF protein kinase 2 [Source:VGNC Symbol;Acc:VGNC:81764]	-0.862457366	8.94E-07	8.17E-06
ENSMMUG00000008189	cadherin related family member 3 [Source:HGNC Symbol;Acc:HGNC:26308]	1.443664033	2.15E-15	8.10E-14
ENSMMUG00000019593	synaptophysin like 1 [Source:VGNC Symbol;Acc:VGNC:78073]	2.807032589	0.007132428	0.024025473
ENSMMUG00000003572	nicotinamide phosphoribosyltransferase [Source:VGNC Symbol;Acc:VGNC:75056]	1.924928982	4.62E-20	2.80E-18
ENSMMUG00000011385	phosphatidylinositol-4,5-bisphosphate 3-kinase catalytic subunit gamma [Source:VGNC Symbol;Acc:VGNC:75998]	-0.683183412	1.50E-05	0.000104437
ENSMMUG00000062092		3.735264965	2.68E-05	0.000175694
ENSMMUG00000058817		3.441632935	2.50E-30	4.56E-28
ENSMMUG00000002894	dedicator of cytokinesis 4 [Source:VGNC Symbol;Acc:VGNC:71866]	-0.937195401	6.71E-05	0.000399043
ENSMMUG00000011269	CF transmembrane conductance regulator [Source:VGNC Symbol;Acc:VGNC:71020]	-1.133565611	1.02E-09	1.62E-08
ENSMMUG00000023310	tetraspanin 12 [Source:VGNC Symbol;Acc:VGNC:79328]	2.705594299	0.010503347	0.033323174
ENSMMUG00000002434	inhibitor of growth family member 3 [Source:VGNC Symbol;Acc:VGNC:73595]	-1.450453274	2.35E-05	0.000156256
ENSMMUG00000002428	zinc finger protein 800 [Source:VGNC Symbol;Acc:VGNC:79658]	1.119010082	2.62E-07	2.68E-06
ENSMMUG00000015432	staphylococcal nuclease and tudor domain containing 1 [Source:VGNC Symbol;Acc:VGNC:77798]	-0.667188641	0.000624794	0.002931241

ENSMMUG00000007250	RNA binding motif protein 28 [Source:VGNC Symbol;Acc:VGNC:76519]	-0.795829816	0.00028751	0.001478999
ENSMMUG00000037473	proline rich transmembrane protein 4 [Source:VGNC Symbol;Acc:VGNC:81746]	0.539680388	0.002200496	0.008758887
ENSMMUG00000018478	interferon regulatory factor 5 [Source:VGNC Symbol;Acc:VGNC:73763]	1.599867716	1.56E-10	2.88E-09
ENSMMUG00000012110	smoothened, frizzled class receptor [Source:VGNC Symbol;Acc:VGNC:77658]	-1.168267001	4.33E-08	5.16E-07
ENSMMUG00000017542	ubiquitin conjugating enzyme E2 H [Source:VGNC Symbol;Acc:VGNC:78696]	-0.650146203	0.000214362	0.001139502
ENSMMUG00000010060	serine rich single-pass membrane protein 1 [Source:VGNC Symbol;Acc:VGNC:78014]	-1.663203579	1.55E-05	0.000107553
ENSMMUG00000009495	carboxypeptidase A1 [Source:VGNC Symbol;Acc:VGNC:71455]	0.960800072	0.001488332	0.006217121
ENSMMUG00000009499	mesoderm specific transcript [Source:VGNC Symbol;Acc:VGNC:74707]	3.82961002	3.46E-38	1.28E-35
ENSMMUG00000004801	COPI coat complex subunit gamma 2 [Source:VGNC Symbol;Acc:VGNC:71315]	3.999281949	5.49E-52	6.41E-49
ENSMMUG00000000917	aldo-keto reductase family 1 member B [Source:NCBI gene;Acc:705957]	2.064187027	3.26E-06	2.65E-05
ENSMMUG00000037485		-3.106135686	0.001000801	0.004411861
ENSMMUG00000054821		-2.811169052	0.000637417	0.002975871
ENSMMUG00000006530	ATP/GTP binding protein like 3 [Source:VGNC Symbol;Acc:VGNC:69723]	1.162284681	8.29E-18	4.09E-16
ENSMMUG00000050339	Metazoan signal recognition particle RNA [Source:RFAM;Acc:RF00017]	2.232682376	8.82E-11	1.69E-09
ENSMMUG00000062419		1.5645648	0.013684046	0.041706451
ENSMMUG00000016384	nucleoporin 205 [Source:VGNC Symbol;Acc:VGNC:75432]	0.734329505	0.001069157	0.00468086
ENSMMUG00000064014		3.020745903	0.001747696	0.007145174
ENSMMUG00000002995	tripartite motif containing 24 [Source:VGNC Symbol;Acc:VGNC:78562]	0.903181066	1.34E-06	1.17E-05
ENSMMUG00000006547	ATPase H+ transporting V0 subunit a4 [Source:VGNC Symbol;Acc:VGNC:70189]	-2.963007578	0.000152015	0.000835961
ENSMMUG00000006548	KIAA1549 [Source:VGNC Symbol;Acc:VGNC:73885]	-2.944429302	1.67E-05	0.000114793
ENSMMUG00000006550	zinc finger CCCH-type containing, antiviral 1 [Source:VGNC Symbol;Acc:VGNC:79408]	0.35836322	0.002324384	0.009156018
ENSMMUG00000002481	homeodomain interacting protein kinase 2 [Source:VGNC Symbol;Acc:VGNC:73391]	0.687172779	0.009513937	0.030676924
ENSMMUG00000004222	thromboxane A synthase 1 [Source:VGNC Symbol;Acc:VGNC:78278]	0.760535281	8.89E-07	8.12E-06
ENSMMUG00000052228	Metazoan signal recognition particle RNA [Source:RFAM;Acc:RF00017]	2.571210639	6.08E-06	4.68E-05
ENSMMUG00000004224	poly(ADP-ribose) polymerase family member 12 [Source:VGNC Symbol;Acc:VGNC:81734]	0.754963357	4.23E-06	3.37E-05
ENSMMUG00000056175		0.781769332	1.26E-06	1.12E-05
ENSMMUG00000008126	lysine demethylase 7A [Source:VGNC Symbol;Acc:VGNC:73886]	0.748846409	3.78E-08	4.58E-07
ENSMMUG00000010420	makorin ring finger protein 1 [Source:VGNC Symbol;Acc:VGNC:81476]	0.642440601	0.00498481	0.017669867
ENSMMUG00000042299	DENN domain containing 2A [Source:VGNC Symbol;Acc:VGNC:81302]	-2.655369201	0.001534147	0.006376212
ENSMMUG00000015282	aarF domain containing kinase 2 [Source:VGNC Symbol;Acc:VGNC:69473]	-0.524770673	0.00034966	0.001747365
ENSMMUG00000042793	B-Raf proto-oncogene, serine/threonine kinase [Source:NCBI gene;Acc:693554]	0.694198494	0.002149047	0.008588228
ENSMMUG00000060893		-1.369532879	0.000916327	0.004076666
ENSMMUG00000064529		-1.820334557	7.36E-14	2.25E-12
ENSMMUG00000021017	acylglycerol kinase [Source:VGNC Symbol;Acc:VGNC:69669]	1.177865345	9.25E-20	5.49E-18
ENSMMUG00000022442	single stranded DNA binding protein 1 [Source:VGNC Symbol;Acc:VGNC:77886]	-1.112959541	1.32E-09	2.06E-08
ENSMMUG00000055710	taste 2 receptor member 38 [Source:NCBI gene;Acc:695549]	1.439234741	0.003744918	0.013819734
ENSMMUG00000016273	maltase-glucoamylase [Source:VGNC Symbol;Acc:VGNC:74713]	2.060626408	1.59E-14	5.37E-13
ENSMMUG00000049575	T cell receptor beta variable 7-2 [Source:HGNC Symbol;Acc:HGNC:12236]	-0.904098839	0.00040229	0.001984284
ENSMMUG00000050984	T cell receptor beta variable 5-4 [Source:HGNC Symbol;Acc:HGNC:12221]	1.555327137	0.005416728	0.018947062

ENSMMUG00000049767		-3.032917213	0.001781525	0.007271501
ENSMMUG00000048246		-2.157217052	0.006676322	0.022635897
ENSMMUG00000057494	T cell receptor beta variable 7-9 [Source:HGNC Symbol;Acc:HGNC:12243]	-1.714009951	1.02E-07	1.14E-06
ENSMMUG00000050269	T cell receptor beta variable 14 [Source:HGNC Symbol;Acc:HGNC:12189]	-1.790540236	1.85E-11	3.95E-10
ENSMMUG00000016286	protease, serine, 1 (trypsin 1) [Source:NCBI gene;Acc:698729]	-1.088608457	2.03E-10	3.65E-09
ENSMMUG00000051032	serine protease 2 [Source:NCBI gene;Acc:698352]	-2.04505778	1.61E-13	4.64E-12
ENSMMUG00000052682		-3.262881496	6.18E-11	1.21E-09
ENSMMUG00000059019		-3.123331932	6.09E-20	3.65E-18
ENSMMUG00000058459		-2.133660613	0.008532171	0.028046562
ENSMMUG00000055756		-3.175598942	0.007148908	0.024075202
ENSMMUG00000043809	caspase 2 [Source:VGNC Symbol;Acc:VGNC:80804]	-0.737758432	4.18E-06	3.33E-05
ENSMMUG00000045428	Metazoan signal recognition particle RNA [Source:RFAM;Acc:RF00017]	-1.912386843	0.01268108	0.039031341
ENSMMUG00000012633	family with sequence similarity 131 member B [Source:VGNC Symbol;Acc:VGNC:72315]	3.443177021	0.008347181	0.027522342
ENSMMUG00000012635	zyxin [Source:VGNC Symbol;Acc:VGNC:78917]	2.251828987	3.54E-19	2.02E-17
ENSMMUG00000055322	EPH receptor A1 [Source:NCBI gene;Acc:706295]	1.884739218	4.24E-18	2.18E-16
ENSMMUG00000058370	taste receptor type 2 member 62 [Source:NCBI gene;Acc:704388]	4.661310927	4.53E-08	5.39E-07
ENSMMUG00000042798		-0.98064198	2.34E-05	0.00015549
ENSMMUG00000045869		-0.909510423	0.011745159	0.036633089
ENSMMUG00000013955	TRPM8 channel associated factor 2 [Source:NCBI gene;Acc:707579]	-0.5671522	0.003954585	0.0144905
ENSMMUG00000005136	TRPM8 channel associated factor 1 [Source:VGNC Symbol;Acc:VGNC:79346]	-0.626722993	3.83E-05	0.000242136
ENSMMUG00000063362	Metazoan signal recognition particle RNA [Source:RFAM;Acc:RF00017]	1.708451424	0.013689912	0.041715263
ENSMMUG00000057973		-1.186937815	0.004488473	0.016167958
ENSMMUG00000049864		-1.899432726	0.010785786	0.034111204
ENSMMUG00000018549	zinc finger protein 786 [Source:VGNC Symbol;Acc:VGNC:79338]	2.588221789	2.19E-08	2.76E-07
ENSMMUG00000011788	zinc finger protein 212 [Source:VGNC Symbol;Acc:VGNC:78418]	0.961189115	0.005551337	0.019331849
ENSMMUG00000002497	zinc finger protein 777 [Source:HGNC Symbol;Acc:HGNC:22213]	-0.894659621	0.000154391	0.00084803
ENSMMUG00000060626	U6 spliceosomal RNA [Source:RFAM;Acc:RF00026]	1.555668818	0.000404849	0.001995499
ENSMMUG00000051020	zinc finger protein 467 [Source:NCBI gene;Acc:712106]	-1.109343229	2.44E-05	0.000161605
ENSMMUG00000048892	SCO-spondin [Source:VGNC Symbol;Acc:VGNC:81765]	-0.676146803	0.005508474	0.019210477
ENSMMUG00000022633	replication initiator 1 [Source:VGNC Symbol;Acc:VGNC:76758]	1.106441846	0.00063139	0.002954283
ENSMMUG00000062696		-1.160330649	0.001007502	0.004437248
ENSMMUG00000022636	GTPase, IMAP family member 8 [Source:VGNC Symbol;Acc:VGNC:72981]	-1.380243542	1.32E-06	1.15E-05
ENSMMUG00000011386	GTPase, IMAP family member 4 [Source:VGNC Symbol;Acc:VGNC:72982]	-1.901522749	1.69E-22	1.34E-20

ENSMMUG00000052830		-2.153640968	4.59E-11	9.14E-10
ENSMMUG00000047276	GTPase, IMAP family member 6 [Source:VGNC Symbol;Acc:VGNC:81315]	-1.104316144	3.93E-08	4.73E-07
ENSMMUG00000017008	GTPase, IMAP family member 2 [Source:VGNC Symbol;Acc:VGNC:72980]	-2.561220009	0.000230236	0.001216964
ENSMMUG00000020435	amine oxidase copper containing 1 [Source:VGNC Symbol;Acc:VGNC:70033]	-1.066296008	4.94E-07	4.78E-06
ENSMMUG00000020436	potassium voltage-gated channel subfamily H member 2 [Source:VGNC Symbol;Acc:VGNC:73980]	-0.91674047	3.82E-07	3.80E-06
ENSMMUG00000006682	chondroitin polymerizing factor 2 [Source:VGNC Symbol;Acc:VGNC:71131]	-1.307444735	0.001578049	0.006539297
ENSMMUG00000038898	Metazoan signal recognition particle RNA [Source:RFAM;Acc:RF00017]	0.750906063	0.000211875	0.00112799
ENSMMUG00000010943	protein kinase AMP-activated non-catalytic subunit gamma 2 [Source:VGNC Symbol;Acc:VGNC:81745]	0.756286369	0.012803976	0.039375035
ENSMMUG00000012238	polypeptide N-acetylgalactosaminyltransferase 11 [Source:VGNC Symbol;Acc:VGNC:72749]	-0.579897011	0.01138021	0.035693288
ENSMMUG00000002878	X-ray repair cross complementing 2 [Source:VGNC Symbol;Acc:VGNC:78813]	0.724674439	0.003069146	0.011617881
ENSMMUG00000039033	nucleolar protein with MIF4G domain 1 [Source:VGNC Symbol;Acc:VGNC:81729]	-2.539249758	0.001412079	0.005948207
ENSMMUG00000008733	ubiquitin protein ligase E3C [Source:VGNC Symbol;Acc:VGNC:79049]	-0.704826194	0.000679731	0.003151032
ENSMMUG00000052713	leucine rich repeat containing 14B [Source:NCBI gene;Acc:723071]	0.572255232	0.001428407	0.006004364
ENSMMUG00000063025	coiled-coil domain containing 127 [Source:NCBI gene;Acc:710325]	-0.465687948	0.001188408	0.005124553
ENSMMUG00000000752	programmed cell death 6 [Source:NCBI gene;Acc:723043]	0.441798841	0.00148552	0.006207225
ENSMMUG00000052291		-1.523807827	0.001388062	0.005861123
ENSMMUG00000061096	tubulin polymerization promoting protein [Source:NCBI gene;Acc:708834]	-1.216569613	0.014987636	0.044927665
ENSMMUG00000003194		1.106077623	0.00332501	0.012443721
ENSMMUG00000014738	bromodomain containing 9 [Source:VGNC Symbol;Acc:VGNC:70386]	4.250150398	0.000134598	0.000747807
ENSMMUG00000019441	solute carrier family 12 member 7 [Source:VGNC Symbol;Acc:VGNC:77390]	1.096759466	8.17E-06	6.07E-05
ENSMMUG00000010940	telomerase reverse transcriptase [Source:VGNC Symbol;Acc:VGNC:78313]	1.100571039	1.35E-18	7.39E-17
ENSMMUG00000061176		-0.739709683	7.87E-09	1.07E-07
ENSMMUG00000002561	NOP2/Sun RNA methyltransferase 2 [Source:VGNC Symbol;Acc:VGNC:75466]	-1.949820057	0.004691806	0.016801122
ENSMMUG00000018746	FAST kinase domains 3 [Source:VGNC Symbol;Acc:VGNC:72588]	-1.213955854	3.66E-07	3.66E-06
ENSMMUG00000018747	5-methyltetrahydrofolate-homocysteine methyltransferase reductase [Source:VGNC Symbol;Acc:VGNC:75079]	-1.372517499	4.65E-11	9.23E-10
ENSMMUG00000011026	dynein axonemal heavy chain 5 [Source:VGNC Symbol;Acc:VGNC:71942]	3.143942874	0.004374776	0.01583161
ENSMMUG00000065128	Metazoan signal recognition particle RNA [Source:RFAM;Acc:RF00017]	-0.846998459	0.001140411	0.004941898
ENSMMUG00000059412		-0.480218643	0.014771686	0.044384692
ENSMMUG00000062083		1.690089904	0.000827148	0.003744058
ENSMMUG00000059036		2.127363861	0.000620824	0.002918474
ENSMMUG00000059710		1.36431862	0.003765282	0.013876147
ENSMMUG00000013954	PDZ domain containing 2 [Source:VGNC Symbol;Acc:VGNC:75829]	-0.407059061	0.001635877	0.006750107
ENSMMUG00000009574	solute carrier family 45 member 2 [Source:VGNC Symbol;Acc:VGNC:82240]	-1.059292106	0.000264923	0.001381048
ENSMMUG00000013467	alpha-methylacyl-CoA racemase [Source:NCBI gene;Acc:698365]	-0.528207786	0.002916009	0.011148089
ENSMMUG00000018739	sperm flagellar 2 [Source:VGNC Symbol;Acc:VGNC:82243]	-4.24683806	1.13E-06	1.01E-05

ENSMMUG00000006202	solute carrier family 1 member 3 [Source:VGNC Symbol;Acc:VGNC:77411]	-0.410479551	0.000131575	0.00073188
ENSMMUG00000052836		0.538499214	0.00138235	0.005840524
ENSMMUG00000018963	glial cell derived neurotrophic factor [Source:VGNC Symbol;Acc:VGNC:72912]	-1.30046606	0.005335745	0.018696439
ENSMMUG00000017163	EGF like, fibronectin type III and laminin G domains [Source:VGNC Symbol;Acc:VGNC:72122]	-0.500285555	0.003688187	0.013639093
ENSMMUG00000004774	maestro heat like repeat family member 2B [Source:VGNC Symbol;Acc:VGNC:74837]	1.896217504	1.80E-05	0.000122876
ENSMMUG00000021034	complement C6 [Source:VGNC Symbol;Acc:VGNC:70477]	-2.23597167	8.80E-12	2.00E-10
ENSMMUG00000011878	poly(A) binding protein interacting protein 1 [Source:VGNC Symbol;Acc:VGNC:84476]	3.043602631	1.04E-13	3.08E-12
ENSMMUG00000055022	Metazoan signal recognition particle RNA [Source:RFAM;Acc:RF00017]	3.658762362	0.000268659	0.001394491
ENSMMUG00000047162	fibroblast growth factor 10 [Source:VGNC Symbol;Acc:VGNC:84378]	-0.666242347	0.004527849	0.016293051
ENSMMUG00000059786	hyperpolarization activated cyclic nucleotide gated potassium channel 1 [Source:NCBI gene;Acc:704286]	-2.737572992	0.009059258	0.029454118
ENSMMUG00000016149	embigin [Source:VGNC Symbol;Acc:VGNC:72144]	-2.269442395	0.008139878	0.026927462
ENSMMUG00000013841	poly(ADP-ribose) polymerase family member 8 [Source:VGNC Symbol;Acc:VGNC:84477]	1.647238141	0.002187994	0.008714069
ENSMMUG00000001753	integrin subunit alpha 1 [Source:VGNC Symbol;Acc:VGNC:73772]	1.561776396	4.72E-12	1.11E-10
ENSMMUG00000039101	pelota mRNA surveillance and ribosome rescue factor [Source:VGNC Symbol;Acc:VGNC:84478]	2.006649247	4.44E-20	2.70E-18
ENSMMUG00000010379	Mtr4 exosome RNA helicase [Source:VGNC Symbol;Acc:VGNC:74989]	-0.91521922	6.62E-05	0.00039466
ENSMMUG00000030099	phospholipid phosphatase 1 [Source:VGNC Symbol;Acc:VGNC:76226]	-2.197894117	2.03E-10	3.65E-09
ENSMMUG00000061973		-2.420416376	5.93E-38	2.13E-35
ENSMMUG00000010466	solute carrier family 38 member 9 [Source:VGNC Symbol;Acc:VGNC:77574]	2.434203365	2.20E-13	6.18E-12
ENSMMUG00000051661		-1.559108777	9.17E-06	6.72E-05
ENSMMUG00000005181	RAB3C, member RAS oncogene family [Source:VGNC Symbol;Acc:VGNC:81541]	-1.504349099	0.000169035	0.000919106
ENSMMUG00000021812	ERCC excision repair 8, CSA ubiquitin ligase complex subunit [Source:VGNC Symbol;Acc:VGNC:72425]	0.706100995	1.21E-10	2.27E-09
ENSMMUG00000060505		-1.230704824	0.001280018	0.005466058
ENSMMUG00000021131	regulator of G protein signaling 7 binding protein [Source:VGNC Symbol;Acc:VGNC:76808]	-1.032159602	0.002843353	0.01089706
ENSMMUG00000015041	CWC27 spliceosome associated cyclophilin [Source:VGNC Symbol;Acc:VGNC:71396]	-1.436510186	0.001323072	0.005620551
ENSMMUG00000051169		-1.971268282	0.000238468	0.001254796
ENSMMUG00000016522	neurolysin [Source:VGNC Symbol;Acc:VGNC:75183]	-0.581640641	1.40E-05	9.80E-05
ENSMMUG00000038355		-2.899406678	0.01209974	0.037522039
ENSMMUG00000049935		1.421820229	8.46E-10	1.37E-08
ENSMMUG00000046550	coiled-coil domain containing 125 [Source:NCBI gene;Acc:704391]	1.025362212	7.76E-09	1.06E-07
ENSMMUG00000009158	MARVEL domain containing 2 [Source:VGNC Symbol;Acc:VGNC:74592]	-0.477685247	0.002941864	0.011225511
ENSMMUG00000040485	Metazoan signal recognition particle RNA [Source:RFAM;Acc:RF00017]	-0.590421636	0.001425646	0.005998151
ENSMMUG00000006217	general transcription factor IIH subunit 2 [Source:NCBI gene;Acc:700737]	-1.021924301	0.000834837	0.003775209
ENSMMUG00000054237	Y RNA [Source:RFAM;Acc:RF00019]	2.207673043	4.22E-06	3.36E-05
ENSMMUG00000059671		1.507798146	2.39E-16	1.02E-14
ENSMMUG00000063792		0.519377168	0.010282489	0.032726135
ENSMMUG00000064344	Metazoan signal recognition particle RNA [Source:RFAM;Acc:RF00017]	-4.1431731	7.37E-05	0.000433792
ENSMMUG00000011061	ankyrin repeat domain 31 [Source:VGNC Symbol;Acc:VGNC:84277]	0.698529579	1.63E-07	1.74E-06

ENSMMUG00000006139	3-hydroxy-3-methylglutaryl-CoA reductase [Source:VGNC Symbol;Acc:VGNC:73484]	0.688257412	1.94E-10	3.51E-09
ENSMMUG00000006141	ceramide transporter 1 [Source:VGNC Symbol;Acc:VGNC:71298]	0.318740073	0.009513824	0.030676924
ENSMMUG00000022982	IQ motif containing GTPase activating protein 2 [Source:VGNC Symbol;Acc:VGNC:73756]	-1.763531064	3.16E-05	0.000203752
ENSMMUG00000021358	angiogenic factor with G-patch and FHA domains 1 [Source:NCBI gene;Acc:707686]	0.839802651	0.013488811	0.041209934
ENSMMUG00000021357	zinc finger BED-type containing 3 [Source:VGNC Symbol;Acc:VGNC:79271]	2.28941429	6.76E-06	5.13E-05
ENSMMUG00000020731	terminal nucleotidyltransferase 2 [Source:VGNC Symbol;Acc:VGNC:79637]	-1.890678804	0.012079372	0.037475451
ENSMMUG00000003143	thrombospondin 4 [Source:VGNC Symbol;Acc:VGNC:78346]	0.99197642	0.000787125	0.00359539
ENSMMUG00000018283	dihydrofolate reductase [Source:NCBI gene;Acc:711268]	-1.666127793	0.008949503	0.029165438
ENSMMUG00000018284	mutS homolog 3 [Source:VGNC Symbol;Acc:VGNC:74932]	-0.834922551	0.000160015	0.000875838
ENSMMUG00000039944	Ras protein specific guanine nucleotide releasing factor 2 [Source:VGNC Symbol;Acc:VGNC:84486]	-2.183737046	1.59E-12	4.01E-11
ENSMMUG00000006932	creatine kinase, mitochondrial 2 [Source:VGNC Symbol;Acc:VGNC:71231]	-1.172545976	0.002488367	0.009720546
ENSMMUG00000017583	single stranded DNA binding protein 2 [Source:NCBI gene;Acc:711752]	-0.860717932	1.30E-05	9.21E-05
ENSMMUG00000046560	transmembrane protein 167A [Source:VGNC Symbol;Acc:VGNC:84513]	0.743840616	0.007230549	0.024321852
ENSMMUG00000064265	X-ray repair cross complementing 4 [Source:NCBI gene;Acc:712291]	0.827415646	0.000353576	0.001764483
ENSMMUG00000011513	versican [Source:VGNC Symbol;Acc:VGNC:79445]	2.150448011	5.14E-15	1.86E-13
ENSMMUG00000061282	hyaluronan and proteoglycan link protein 1 [Source:NCBI gene;Acc:712501]	3.479853702	0.004811105	0.017175694
ENSMMUG00000003245	RAS p21 protein activator 1 [Source:VGNC Symbol;Acc:VGNC:76667]	-0.297288753	0.006191062	0.021205975
ENSMMUG00000003248	cyclin H [Source:VGNC Symbol;Acc:VGNC:70732]	-0.304243927	0.002436241	0.009558871
ENSMMUG00000009319	transmembrane protein 161B [Source:VGNC Symbol;Acc:VGNC:84508]	-1.371560651	4.78E-05	0.000295064
ENSMMUG00000019836	myocyte enhancer factor 2C [Source:VGNC Symbol;Acc:VGNC:84438]	-1.308416895	6.56E-06	5.00E-05
ENSMMUG00000055392		-1.438901107	9.80E-06	7.12E-05
ENSMMUG00000056790		-4.140763081	9.55E-06	6.96E-05
ENSMMUG00000018972	RNA polymerase III subunit G [Source:VGNC Symbol;Acc:VGNC:84480]	-1.280230086	0.000156424	0.000857854
ENSMMUG00000018974	LysM domain containing 3 [Source:VGNC Symbol;Acc:VGNC:74373]	-1.800686894	9.88E-32	2.20E-29
ENSMMUG00000055271		-1.795542034	5.98E-10	9.87E-09
ENSMMUG00000018976	adhesion G protein-coupled receptor V1 [Source:VGNC Symbol;Acc:VGNC:69625]	-1.295518028	0.000194386	0.001045198
ENSMMUG00000053899		-2.371602089	0.003093893	0.011691096
ENSMMUG00000001397	multiple C2 and transmembrane domain containing 1 [Source:VGNC Symbol;Acc:VGNC:74589]	1.223342568	6.38E-05	0.000382304
ENSMMUG00000010632	family with sequence similarity 81 member B [Source:VGNC Symbol;Acc:VGNC:72365]	3.778173449	2.46E-05	0.000162765
ENSMMUG00000001209	arylsulfatase family member K [Source:VGNC Symbol;Acc:VGNC:84280]	-1.218670622	0.000246089	0.001291988
ENSMMUG00000001212	Rieske Fe-S domain containing [Source:VGNC Symbol;Acc:VGNC:84487]	-1.219870623	7.62E-21	5.16E-19
ENSMMUG00000001213	spermatogenesis associated 9 [Source:VGNC Symbol;Acc:VGNC:77976]	-1.580843332	2.15E-12	5.30E-11
ENSMMUG00000002063	endoplasmic reticulum aminopeptidase 1 [Source:VGNC Symbol;Acc:VGNC:72066]	0.504924242	0.001316017	0.005595666
ENSMMUG00000011043	endoplasmic reticulum aminopeptidase 2 [Source:VGNC Symbol;Acc:VGNC:72141]	0.549701959	1.75E-07	1.85E-06

ENSMMUG00000065221		0.70793963	7.82E-07	7.25E-06
ENSMMUG00000018542	leucyl and cystinyl aminopeptidase [Source:VGNC Symbol;Acc:VGNC:74438]	-0.608198907	3.78E-05	0.000239403
ENSMMUG00000000307	RIO kinase 2 [Source:VGNC Symbol;Acc:VGNC:76774]	0.267340341	0.009781908	0.03135353
ENSMMUG00000054789	family with sequence similarity 174 member A [Source:NCBI gene;Acc:702300]	0.688898381	2.28E-06	1.90E-05
ENSMMUG00000007773	solute carrier organic anion transporter family member 4C1 [Source:VGNC Symbol;Acc:VGNC:77764]	2.477272768	5.77E-05	0.000349324
ENSMMUG00000007844	peptidylglycine alpha-amidating monooxygenase [Source:VGNC Symbol;Acc:VGNC:75752]	-1.047107623	0.00033047	0.001667573
ENSMMUG00000057827		-0.585880943	0.000483812	0.002344332
ENSMMUG00000007786	gypsy retrotransposon integrase 1 [Source:VGNC Symbol;Acc:VGNC:73015]	-0.321670284	0.006957869	0.023488252
ENSMMUG00000007787	diphosphoinositol pentakisphosphate kinase 2 [Source:VGNC Symbol;Acc:VGNC:76128]	-0.696603176	0.000615262	0.002898208
ENSMMUG00000059227		3.698121992	0.015114227	0.045220141
ENSMMUG00000013183	F-box and leucine rich repeat protein 17 [Source:VGNC Symbol;Acc:VGNC:82142]	-0.571099903	0.000850804	0.00383258
ENSMMUG00000003316	mannosidase alpha class 2A member 1 [Source:VGNC Symbol;Acc:VGNC:81449]	-1.02501929	2.48E-09	3.70E-08
ENSMMUG00000038051	uncharacterized LOC106998885 [Source:NCBI gene;Acc:106998885]	-1.254688032	1.05E-06	9.42E-06
ENSMMUG00000042638		-2.940026292	0.010569974	0.033496665
ENSMMUG00000011974	calcium/calmodulin dependent protein kinase IV [Source:VGNC Symbol;Acc:VGNC:70526]	-2.081089299	5.91E-14	1.83E-12
ENSMMUG00000063177	StAR related lipid transfer domain containing 4 [Source:NCBI gene;Acc:706654]	-2.311416311	1.24E-30	2.39E-28
ENSMMUG00000012192	erythrocyte membrane protein band 4.1 like 4A [Source:VGNC Symbol;Acc:VGNC:72039]	-1.353254337	1.28E-09	2.01E-08
ENSMMUG00000042951	signal recognition particle 19 [Source:NCBI gene;Acc:693829]	0.950503387	5.82E-06	4.49E-05
ENSMMUG00000020488	receptor accessory protein 5 [Source:VGNC Symbol;Acc:VGNC:76864]	0.761866163	1.01E-08	1.34E-07
ENSMMUG00000015114	toll like receptor adaptor molecule 2 [Source:NCBI gene;Acc:100426353]	0.38372264	0.000515823	0.002476339
ENSMMUG00000060812	autophagy related 12 [Source:NCBI gene;Acc:696765]	1.008257063	1.03E-07	1.15E-06
ENSMMUG00000015288	adaptor related protein complex 3 subunit sigma 1 [Source:VGNC Symbol;Acc:VGNC:69867]	0.649477605	0.00578566	0.020012904
ENSMMUG00000022191	laeverin [Source:VGNC Symbol;Acc:VGNC:74372]	-2.571872245	0.002992936	0.011383224
ENSMMUG00000022192		-1.290987271	0.000258058	0.001347265
ENSMMUG00000056563	ADP ribosylation factor like GTPase 14 effector protein like [Source:NCBI gene;Acc:698168]	-0.660293194	0.006324779	0.021614835
ENSMMUG00000008738	COMM domain containing 10 [Source:VGNC Symbol;Acc:VGNC:71374]	-0.717955263	0.016239003	0.048031963
ENSMMUG00000037127	U6 spliceosomal RNA [Source:RFAM;Acc:RF00026]	-1.364219914	0.009586829	0.0308552
ENSMMUG00000063474		-0.722852087	4.69E-08	5.57E-07
ENSMMUG00000017685	hydroxysteroid 17-beta dehydrogenase 4 [Source:VGNC Symbol;Acc:VGNC:73530]	-0.432403228	1.20E-06	1.07E-05
ENSMMUG00000059145		-0.929928022	0.014623157	0.04405167
ENSMMUG00000018764		-1.404473113	0.000470006	0.002282957
ENSMMUG00000063354		-1.306033546	0.001390375	0.005867356
ENSMMUG00000053794		-0.83542487	0.000826458	0.003742977

ENSMMUG00000021006	zinc finger protein 474 [Source:VGNC Symbol;Acc:VGNC:81617]	-1.219014017	0.000729352	0.003350051
ENSMMUG00000046428		-1.459376282	6.04E-07	5.74E-06
ENSMMUG00000023598	sorting nexin 2 [Source:VGNC Symbol;Acc:VGNC:77682]	2.047614274	0.000524493	0.002514518
ENSMMUG00000006646	casein kinase 1 gamma 3 [Source:VGNC Symbol;Acc:VGNC:81384]	-0.428891558	0.015180872	0.045390484
ENSMMUG00000016683	phosphorylated adaptor for RNA export [Source:NCBI gene;Acc:702980]	-1.564158882	0.000280962	0.001450101
ENSMMUG00000037806	chromosome 6 C5orf63 homolog [Source:NCBI gene;Acc:100423212]	-1.785210832	0.007776215	0.025883188
ENSMMUG00000011214	solute carrier family 12 member 2 [Source:VGNC Symbol;Acc:VGNC:77386]	-1.348073982	3.06E-06	2.50E-05
ENSMMUG00000010682	fibrillin 2 [Source:VGNC Symbol;Acc:VGNC:72624]	-1.156529849	8.62E-14	2.60E-12
ENSMMUG00000058082		0.518583016	8.55E-06	6.32E-05
ENSMMUG00000016909	meiotic kinetochore factor [Source:NCBI gene;Acc:709179]	0.89083202	2.01E-17	9.59E-16
ENSMMUG00000010858	prolyl 4-hydroxylase subunit alpha 2 [Source:VGNC Symbol;Acc:VGNC:75742]	-2.582279911	3.48E-14	1.12E-12
ENSMMUG00000003131	interleukin 13 [Source:VGNC Symbol;Acc:VGNC:84393]	1.305374425	1.65E-08	2.15E-07
ENSMMUG00000003133	interleukin 4 [Source:VGNC Symbol;Acc:VGNC:73729]	1.704924666	1.64E-08	2.14E-07
ENSMMUG00000010313	kinesin family member 3A [Source:VGNC Symbol;Acc:VGNC:74017]	1.465859109	4.23E-20	2.60E-18
ENSMMUG00000010308	cyclin I family member 2 [Source:VGNC Symbol;Acc:VGNC:70923]	1.601620199	4.31E-12	1.02E-10
ENSMMUG00000010309	septin 8 [Source:VGNC Symbol;Acc:VGNC:77248]	1.826726118	2.25E-07	2.32E-06
ENSMMUG00000039781	sosondowah ankyrin repeat domain family member A [Source:VGNC Symbol;Acc:VGNC:84498]	-1.44305984	2.09E-05	0.000140721
ENSMMUG00000010312	shroom family member 1 [Source:VGNC Symbol;Acc:VGNC:77299]	-1.535256518	7.97E-18	3.95E-16
ENSMMUG00000060221	growth differentiation factor 9 [Source:NCBI gene;Acc:712792]	-1.232377764	6.55E-11	1.28E-09
ENSMMUG00000059080		-1.011600692	0.002926947	0.011183807
ENSMMUG00000015640	AF4/FMR2 family member 4 [Source:VGNC Symbol;Acc:VGNC:69629]	-1.105933508	2.23E-06	1.86E-05
ENSMMUG00000013047	S-phase kinase associated protein 1 [Source:NCBI gene;Acc:100423634]	-1.863278537	5.02E-17	2.33E-15
ENSMMUG00000052915		-0.993779784	0.008400558	0.027665811
ENSMMUG00000016641	ubiquitin conjugating enzyme E2 B [Source:VGNC Symbol;Acc:VGNC:78692]	-0.820192445	0.000248368	0.00130249
ENSMMUG00000053384		-1.630615192	3.01E-20	1.91E-18
ENSMMUG00000000358	jade family PHD finger 2 [Source:VGNC Symbol;Acc:VGNC:73641]	-0.691172055	0.007791761	0.02591033
ENSMMUG00000004417	secretion associated Ras related GTPase 1B [Source:VGNC Symbol;Acc:VGNC:76970]	0.500338782	0.000115705	0.000651883
ENSMMUG00000012463	DEAD-box helicase 46 [Source:VGNC Symbol;Acc:VGNC:71663]	-0.599382039	0.000615479	0.002898208
ENSMMUG00000005286	cation channel sperm associated 3 [Source:VGNC Symbol;Acc:VGNC:70592]	-0.372113254	0.009812865	0.031445567
ENSMMUG00000052860	paired like homeodomain 1 [Source:NCBI gene;Acc:711666]	-0.418194158	0.015460173	0.046127235
ENSMMUG00000016357	polycystin 2 like 2, transient receptor potential cation channel [Source:VGNC Symbol;Acc:VGNC:76009]	-1.736893283	0.001133723	0.004915954
ENSMMUG00000008009	family with sequence similarity 13 member B [Source:VGNC Symbol;Acc:VGNC:72268]	-1.042033319	6.32E-05	0.000379172
ENSMMUG00000013272	GDNF family receptor alpha 3 [Source:VGNC Symbol;Acc:VGNC:72919]	-0.450380648	0.001747097	0.007144804

ENSMMUG00000055069	early growth response 1 [Source:NCBI gene;Acc:716786]	- 0.365115558	0.006509072	0.022149167
ENSMMUG00000005871	eukaryotic translation termination factor 1 [Source:VGNC Symbol;Acc:VGNC:84370]	- 0.456948613	0.003369989	0.012595245
ENSMMUG00000017985	catenin alpha 1 [Source:VGNC Symbol;Acc:VGNC:84291]	0.431091678	0.002184896	0.008705733
ENSMMUG00000001941	CXXC finger protein 5 [Source:VGNC Symbol;Acc:VGNC:71594]	- 0.718567593	5.17E-14	1.63E-12
ENSMMUG00000019582	ankyrin repeat and KH domain-containing protein 1 [Source:NCBI gene;Acc:698045]	- 0.308740207	0.004229412	0.015376987
ENSMMUG00000001707	amyloid beta precursor protein binding family B member 3 [Source:VGNC Symbol;Acc:VGNC:69869]	2.177667135	4.87E-31	1.02E-28
ENSMMUG00000001725	solute carrier family 35 member A4 [Source:VGNC Symbol;Acc:VGNC:77532]	2.280864687	1.37E-21	9.87E-20
ENSMMUG00000062326		2.165692107	1.23E-18	6.76E-17
ENSMMUG00000024537		2.806347605	1.06E-06	9.53E-06
ENSMMUG00000010007	CD14 molecule [Source:VGNC Symbol;Acc:VGNC:70751]	2.162712417	1.16E-11	2.57E-10
ENSMMUG00000060632		1.759881557	2.70E-05	0.000176595
ENSMMUG00000018246	transmembrane and coiled-coil domains 6 [Source:VGNC Symbol;Acc:VGNC:84507]	1.420250664	1.31E-12	3.33E-11
ENSMMUG00000056796	NADH:ubiquinone oxidoreductase subunit A2 [Source:NCBI gene;Acc:697736]	2.181931308	4.00E-05	0.000251856
ENSMMUG00000010008	IK cytokine [Source:VGNC Symbol;Acc:VGNC:73703]	0.80579523	0.014192605	0.043013378
ENSMMUG00000004483	histidyl-tRNA synthetase 2, mitochondrial [Source:VGNC Symbol;Acc:VGNC:73346]	- 0.436939478	0.003752539	0.013836932
ENSMMUG00000014571	protocadherin alpha-C2 [Source:NCBI gene;Acc:698874]	0.254586647	0.015128442	0.045253015
ENSMMUG00000062270	protocadherin beta 3 [Source:NCBI gene;Acc:700243]	- 1.577800833	0.004639437	0.016639084
ENSMMUG00000008710	protocadherin 1 [Source:VGNC Symbol;Acc:VGNC:75775]	- 0.958538368	0.00127713	0.005456822
ENSMMUG00000006287	DAP3 binding cell death enhancer 1 [Source:VGNC Symbol;Acc:VGNC:71806]	0.560803264	1.29E-05	9.11E-05
ENSMMUG00000006291	protocadherin 12 [Source:VGNC Symbol;Acc:VGNC:75776]	0.794610349	6.15E-05	0.000370111
ENSMMUG00000009768	ring finger protein 14 [Source:VGNC Symbol;Acc:VGNC:84488]	0.628117268	9.30E-08	1.04E-06
ENSMMUG00000000419	Rho GTPase activating protein 26 [Source:VGNC Symbol;Acc:VGNC:69846]	1.390154438	1.58E-09	2.44E-08
ENSMMUG00000050405		1.374580078	2.94E-10	5.15E-09
ENSMMUG00000000421	nuclear receptor subfamily 3 group C member 1 [Source:VGNC Symbol;Acc:VGNC:75515]	1.456828357	1.87E-19	1.08E-17
ENSMMUG00000063348		- 0.896668181	1.42E-05	9.94E-05
ENSMMUG00000019820	SH3 domain and tetratricopeptide repeats 2 [Source:VGNC Symbol;Acc:VGNC:77337]	0.692335049	1.88E-06	1.59E-05
ENSMMUG00000064432		- 2.436594279	0.009965459	0.031883557
ENSMMUG00000017714	Rho guanine nucleotide exchange factor 37 [Source:VGNC Symbol;Acc:VGNC:69941]	- 1.209340592	0.000320876	0.001625596
ENSMMUG00000004749	PPARG coactivator 1 beta [Source:VGNC Symbol;Acc:VGNC:76134]	3.482181029	2.83E-10	4.98E-09
ENSMMUG00000011557	platelet derived growth factor receptor beta [Source:VGNC Symbol;Acc:VGNC:75929]	0.31246417	9.73E-07	8.82E-06
ENSMMUG00000005414	N-deacetylase and N-sulfotransferase 1 [Source:VGNC Symbol;Acc:VGNC:75151]	-0.89453907	1.14E-09	1.79E-08
ENSMMUG00000051062		-0.63248967	0.009399372	0.030377368
ENSMMUG00000020679	myozenin 3 [Source:VGNC Symbol;Acc:VGNC:74988]	1.273923497	1.68E-06	1.44E-05
ENSMMUG00000020683	RNA binding motif protein 22 [Source:VGNC Symbol;Acc:VGNC:76535]	2.023521716	9.89E-12	2.22E-10
ENSMMUG00000019764	dynactin subunit 4 [Source:VGNC Symbol;Acc:VGNC:71625]	1.686230376	8.27E-09	1.12E-07
ENSMMUG00000021051	TNFAIP3 interacting protein 1 [Source:VGNC Symbol;Acc:VGNC:79057]	- 0.998733659	1.20E-05	8.56E-05
ENSMMUG00000021053	annexin A6 [Source:VGNC Symbol;Acc:VGNC:69951]	- 1.618660477	4.07E-09	5.90E-08
ENSMMUG00000053656		1.038027904	6.22E-09	8.65E-08
ENSMMUG00000003499	solute carrier family 36 member 1 [Source:VGNC Symbol;Acc:VGNC:77509]	0.637063045	0.013137896	0.040269445
ENSMMUG00000003497	FAT atypical cadherin 2 [Source:VGNC Symbol;Acc:VGNC:72618]	-1.21417571	1.65E-13	4.73E-12
ENSMMUG00000053367	secreted protein acidic and cysteine rich [Source:NCBI gene;Acc:712388]	1.36052904	6.44E-20	3.84E-18

ENSMMUG00000054302	antioxidant 1 copper chaperone [Source:NCBI gene;Acc:712464]	-0.988863752	0.00010323	0.000587739
ENSMMUG00000056369	SAP30 like [Source:NCBI gene;Acc:714400]	-3.041087085	0.00775614	0.025834763
ENSMMUG00000021228	gem nuclear organelle associated protein 5 [Source:VGNC Symbol;Acc:VGNC:72751]	-0.428900867	0.001806459	0.007366086
ENSMMUG00000035086	U2 spliceosomal RNA [Source:RFAM;Acc:RF00004]	-1.544901391	0.005484467	0.019153298
ENSMMUG00000062861		-0.78589709	0.002546423	0.009924539
ENSMMUG00000059241		-4.855807108	6.07E-14	1.87E-12
ENSMMUG00000015840	RAN binding protein 17 [Source:VGNC Symbol;Acc:VGNC:84485]	0.58002208	1.10E-06	9.82E-06
ENSMMUG00000003067	endoplasmic reticulum-golgi intermediate compartment 1 [Source:VGNC Symbol;Acc:VGNC:82141]	0.813109646	1.09E-06	9.77E-06
ENSMMUG00000054793		-2.015366095	7.05E-05	0.000417302
ENSMMUG00000019926	cytoplasmic polyadenylation element binding protein 4 [Source:VGNC Symbol;Acc:VGNC:71450]	0.702748152	1.76E-06	1.50E-05
ENSMMUG00000009059	Fas associated factor family member 2 [Source:VGNC Symbol;Acc:VGNC:72332]	-1.487686335	0.000642193	0.002993838
ENSMMUG00000013283	cadherin related family member 2 [Source:NCBI gene;Acc:697249]	2.046838371	2.09E-07	2.17E-06
ENSMMUG00000013284	G protein regulated inducer of neurite outgrowth 1 [Source:VGNC Symbol;Acc:VGNC:72998]	2.357973672	0.000437186	0.002140013
ENSMMUG00000002649	unc-5 netrin receptor A [Source:VGNC Symbol;Acc:VGNC:78721]	-2.621000143	3.40E-05	0.000217357
ENSMMUG00000002653	hexokinase 3 [Source:VGNC Symbol;Acc:VGNC:73476]	-1.116019878	7.26E-10	1.19E-08
ENSMMUG00000009041	nuclear receptor binding SET domain protein 1 [Source:VGNC Symbol;Acc:VGNC:75401]	-0.817698662	0.016060401	0.047634307
ENSMMUG00000009045	RAB24, member RAS oncogene family [Source:VGNC Symbol;Acc:VGNC:81524]	-1.35744976	0.002749253	0.010588514
ENSMMUG00000016192	lectin, mannose binding 2 [Source:VGNC Symbol;Acc:VGNC:74302]	-0.398670007	0.003681656	0.013618534
ENSMMUG00000019807	regulator of G protein signaling 14 [Source:VGNC Symbol;Acc:VGNC:76918]	0.615044579	4.06E-06	3.24E-05
ENSMMUG00000003146	PDZ and LIM domain 7 [Source:VGNC Symbol;Acc:VGNC:75848]	0.738071903	0.010236153	0.032608262
ENSMMUG00000016911	DEAD-box helicase 41 [Source:VGNC Symbol;Acc:VGNC:71660]	-0.704780485	0.006778183	0.022931354
ENSMMUG00000004002	CDC like kinase 4 [Source:VGNC Symbol;Acc:VGNC:71173]	1.171440692	1.87E-22	1.46E-20
ENSMMUG00000041519	zinc finger protein 354A [Source:VGNC Symbol;Acc:VGNC:84517]	2.010531783	0.000324323	0.001641277
ENSMMUG00000049557		-1.981256186	0.000416808	0.002048683
ENSMMUG00000001728	mitogen-activated protein kinase 9 [Source:VGNC Symbol;Acc:VGNC:74652]	-1.362299042	5.29E-10	8.84E-09
ENSMMUG00000061451	butyrophilin like 8 [Source:NCBI gene;Acc:707591]	-0.414804196	0.016254066	0.048066378
ENSMMUG00000031833	butyrophilin like 9 [Source:NCBI gene;Acc:106996463]	0.646903041	0.009468427	0.030551253
ENSMMUG00000062522		1.647611647	1.18E-10	2.22E-09
ENSMMUG00000059428	olfactory receptor 2V2 [Source:NCBI gene;Acc:707790]	1.539779486	9.02E-08	1.02E-06
ENSMMUG00000061151		1.508222558	1.03E-06	9.29E-06
ENSMMUG00000054414		1.737659908	1.68E-11	3.62E-10
ENSMMUG00000003598	tripartite motif containing 7 [Source:VGNC Symbol;Acc:VGNC:79022]	1.669686332	3.75E-17	1.75E-15
ENSMMUG00000003604	receptor for activated C kinase 1 [Source:VGNC Symbol;Acc:VGNC:76581]	2.505248897	0.014730462	0.044298792
ENSMMUG00000021145	family with sequence similarity 120B [Source:VGNC Symbol;Acc:VGNC:72311]	-1.69438149	1.19E-22	9.72E-21
ENSMMUG00000014474	SPARC related modular calcium binding 2 [Source:VGNC Symbol;Acc:VGNC:83470]	-0.794243585	6.12E-09	8.53E-08
ENSMMUG00000021044	afadin, adherens junction formation factor [Source:VGNC Symbol;Acc:VGNC:69818]	0.454595678	0.000369686	0.001835732
ENSMMUG00000062443		-1.116724366	1.44E-07	1.55E-06

ENSMMUG00000057859		-2.163759955	1.86E-05	0.000126506
ENSMMUG00000008228	unc-93 homolog A [Source:VGNC Symbol;Acc:VGNC:78724]	-2.257837963	1.04E-05	7.53E-05
ENSMMUG00000057983		2.776813323	0.000850273	0.00383142
ENSMMUG00000054834		0.337742603	0.003169642	0.011913131
ENSMMUG00000020412	parkin coregulated [Source:VGNC Symbol;Acc:VGNC:75646]	-0.870667983	1.64E-05	0.000112865
ENSMMUG00000057544		-1.412660785	7.65E-09	1.04E-07
ENSMMUG00000000500	fibronectin type III domain containing 1 [Source:VGNC Symbol;Acc:VGNC:72546]	0.557994364	0.000194784	0.001046935
ENSMMUG00000011994	T cell activation RhoGTPase activating protein [Source:VGNC Symbol;Acc:VGNC:78126]	0.65711443	0.003815899	0.014037342
ENSMMUG00000022247	osteoclastogenesis associated transmembrane protein 1 [Source:VGNC Symbol;Acc:VGNC:75664]	2.150468121	6.09E-14	1.87E-12
ENSMMUG00000021534	solute carrier family 16 member 10 [Source:NCBI gene;Acc:696132]	-1.707077928	0.001731349	0.007088188
ENSMMUG00000061031		2.734316446	2.70E-26	3.47E-24
ENSMMUG00000058557		1.532746334	1.05E-06	9.42E-06
ENSMMUG00000017189	regulatory factor X6 [Source:VGNC Symbol;Acc:VGNC:76800]	1.820749335	3.49E-10	5.99E-09
ENSMMUG00000059327	NUS1 dehydrodolichyl diphosphate synthase subunit [Source:NCBI gene;Acc:715015]	-0.793066681	9.90E-07	8.96E-06
ENSMMUG00000008525	minichromosome maintenance 9 homologous recombination repair factor [Source:VGNC Symbol;Acc:VGNC:83432]	-3.533221789	7.86E-06	5.87E-05
ENSMMUG00000013302	family with sequence similarity 184 member A [Source:VGNC Symbol;Acc:VGNC:72308]	-3.075364778	6.19E-08	7.20E-07
ENSMMUG00000005225	gap junction protein alpha 1 [Source:VGNC Symbol;Acc:VGNC:73055]	-0.812785678	3.36E-05	0.000215033
ENSMMUG00000024966	U4 spliceosomal RNA [Source:RFAM;Acc:RF00015]	-1.824565569	0.003272156	0.012249185
ENSMMUG00000061165		0.452134429	0.000209495	0.001116591
ENSMMUG00000060822		-0.784806491	8.82E-07	8.07E-06
ENSMMUG00000065276		0.532434354	0.000112167	0.000632967
ENSMMUG00000003134	ring finger protein 217 [Source:VGNC Symbol;Acc:VGNC:76801]	-0.955487154	0.000107001	0.000606251
ENSMMUG00000062110	TPD52 like 1 [Source:NCBI gene;Acc:716568]	-0.778325318	0.015050195	0.045061865
ENSMMUG00000015317	HD domain containing 2 [Source:VGNC Symbol;Acc:VGNC:73209]	-1.894210594	6.15E-06	4.72E-05
ENSMMUG00000060901		1.451729987	8.90E-22	6.64E-20
ENSMMUG00000048882	KIAA0408 ortholog [Source:NCBI gene;Acc:100429459]	-0.474749387	0.000467291	0.002273704
ENSMMUG00000058574		0.53537387	0.000148713	0.00081909
ENSMMUG00000022136	laminin subunit alpha 2 [Source:VGNC Symbol;Acc:VGNC:74225]	0.436687242	0.000116889	0.000657496
ENSMMUG00000021498	L3MBTL histone methyl-lysine binding protein 3 [Source:VGNC Symbol;Acc:VGNC:74106]	0.710644602	4.28E-07	4.19E-06
ENSMMUG00000021499	sterile alpha motif domain containing 3 [Source:VGNC Symbol;Acc:VGNC:77079]	-1.517099926	3.45E-08	4.20E-07
ENSMMUG00000065212		0.91216883	0.000347467	0.001738326
ENSMMUG00000059491		0.936186987	6.20E-07	5.88E-06
ENSMMUG00000017549	ectonucleotide pyrophosphatase/phosphodiesterase 3 [Source:VGNC Symbol;Acc:VGNC:72224]	-1.549437767	5.13E-10	8.58E-09
ENSMMUG00000004996	monooxygenase DBH like 1 [Source:VGNC Symbol;Acc:VGNC:74800]	0.594834894	0.002354829	0.009267965
ENSMMUG00000013236	solute carrier family 18 member B1 [Source:VGNC Symbol;Acc:VGNC:77549]	-0.561231336	0.010382732	0.033000237
ENSMMUG00000005745	EYA transcriptional coactivator and phosphatase 4 [Source:VGNC Symbol;Acc:VGNC:72456]	1.066967996	5.90E-13	1.58E-11
ENSMMUG00000022921	solute carrier family 2 member 12 [Source:VGNC Symbol;Acc:VGNC:77503]	4.324428613	6.04E-68	2.12E-64

ENSMMUG00000038064	Metazoan signal recognition particle RNA [Source:RFAM;Acc:RF00017]	3.059942527	0.009483511	0.030592887
ENSMMUG00000061787		2.865965904	4.08E-05	0.000256185
ENSMMUG00000018166	MYB proto-oncogene, transcription factor [Source:VGNC Symbol;Acc:VGNC:75089]	0.849681935	5.44E-07	5.22E-06
ENSMMUG00000009368	Abelson helper integration site 1 [Source:VGNC Symbol;Acc:VGNC:81010]	0.720255362	2.55E-08	3.19E-07
ENSMMUG00000044284	40S ribosomal protein S11-like [Source:NCBI gene;Acc:706532]	0.837109709	0.000795078	0.003630536
ENSMMUG00000053936		0.743304841	0.000680809	0.003154987
ENSMMUG00000016376	mitogen-activated protein kinase kinase kinase 5 [Source:VGNC Symbol;Acc:VGNC:74495]	1.169706565	1.20E-05	8.56E-05
ENSMMUG00000000123	NHS like 1 [Source:VGNC Symbol;Acc:VGNC:75225]	0.286023779	0.007569922	0.025286568
ENSMMUG00000019136	taxilin beta [Source:VGNC Symbol;Acc:VGNC:79325]	0.554881764	1.05E-05	7.60E-05
ENSMMUG00000058187		1.127239359	1.82E-19	1.06E-17
ENSMMUG00000023012	vesicle trafficking 1 [Source:VGNC Symbol;Acc:VGNC:79296]	-1.082054699	6.97E-05	0.000412958
ENSMMUG00000044958	adhesion G protein-coupled receptor G6 [Source:VGNC Symbol;Acc:VGNC:81009]	-1.264662232	5.63E-09	7.91E-08
ENSMMUG00000061117		-0.778959327	0.000365128	0.001815671
ENSMMUG00000006430	androgen induced 1 [Source:VGNC Symbol;Acc:VGNC:69667]	1.088395291	0.000280124	0.001446312
ENSMMUG00000005348	LTV1 ribosome biogenesis factor [Source:VGNC Symbol;Acc:VGNC:83430]	2.096165248	0.014051938	0.042633128
ENSMMUG00000003750	PLAG1 like zinc finger 1 [Source:VGNC Symbol;Acc:VGNC:83444]	1.575529912	9.78E-05	0.000559514
ENSMMUG00000019443	syntaxin 11 [Source:VGNC Symbol;Acc:VGNC:78041]	3.407798097	0.000890225	0.003983286
ENSMMUG00000023265	A-kinase anchoring protein 12 [Source:VGNC Symbol;Acc:VGNC:69766]	-2.346331124	2.71E-14	8.87E-13
ENSMMUG00000060839		-2.737391172	0.015736134	0.046850915
ENSMMUG00000029475	zinc finger and BTB domain containing 2 [Source:VGNC Symbol;Acc:VGNC:79291]	-2.317668809	1.70E-07	1.80E-06
ENSMMUG00000050174		-2.647127129	0.013154177	0.04030173
ENSMMUG00000018342	required for meiotic nuclear division 1 homolog [Source:VGNC Symbol;Acc:VGNC:76797]	-2.564829026	1.04E-11	2.32E-10
ENSMMUG00000060940	acidic residue methyltransferase 1 [Source:NCBI gene;Acc:704570]	-2.854011525	7.34E-06	5.52E-05
ENSMMUG00000018346	coiled-coil domain containing 170 [Source:VGNC Symbol;Acc:VGNC:70832]	-1.988852645	4.25E-05	0.000265921
ENSMMUG00000019045	estrogen receptor 1 [Source:VGNC Symbol;Acc:VGNC:72432]	-0.883957243	0.000695799	0.00321654
ENSMMUG00000019048	spectrin repeat containing nuclear envelope protein 1 [Source:VGNC Symbol;Acc:VGNC:78143]	-0.458782381	0.01083625	0.034224286
ENSMMUG00000013483	prolyl endopeptidase [Source:VGNC Symbol;Acc:VGNC:76396]	0.732375396	0.000308797	0.001572351
ENSMMUG00000055978		0.554509787	0.000938741	0.004165636
ENSMMUG00000057697		-1.392195363	8.74E-06	6.44E-05
ENSMMUG00000051522	Metazoan signal recognition particle RNA [Source:RFAM;Acc:RF00017]	-5.015932832	2.42E-07	2.49E-06
ENSMMUG00000051271		-3.395562569	1.91E-25	2.14E-23
ENSMMUG00000000808	melanin concentrating hormone receptor 2 [Source:VGNC Symbol;Acc:VGNC:74550]	1.079546758	6.62E-16	2.67E-14
ENSMMUG00000005929	ubiquitin specific peptidase 45 [Source:VGNC Symbol;Acc:VGNC:79233]	-0.44739467	0.002587936	0.010064164
ENSMMUG00000005923	failed axon connections homolog, metaxin like GST domain containing [Source:VGNC Symbol;Acc:VGNC:72541]	-0.989271224	2.97E-05	0.000192261
ENSMMUG00000009182	caspase 8 associated protein 2 [Source:VGNC Symbol;Acc:VGNC:70548]	2.732400046	0.015953051	0.047366002
ENSMMUG00000003464	midasin AAA ATPase 1 [Source:VGNC Symbol;Acc:VGNC:74530]	-0.436524226	1.00E-05	7.29E-05
ENSMMUG00000003462	ankyrin repeat domain 6 [Source:VGNC Symbol;Acc:VGNC:81023]	-0.894744026	4.19E-10	7.13E-09

ENSMMUG00000060841		-0.862824795	0.008998169	0.029296803
ENSMMUG00000002824	akirin 2 [Source:VGNC Symbol;Acc:VGNC:69665]	-1.090112997	0.001411328	0.00594683
ENSMMUG00000002821	origin recognition complex subunit 3 [Source:VGNC Symbol;Acc:VGNC:75715]	-0.891962086	0.001547764	0.006423138
ENSMMUG00000017039	arginyl-tRNA synthetase 2, mitochondrial [Source:VGNC Symbol;Acc:VGNC:76516]	0.530428611	0.000709831	0.003271101
ENSMMUG00000064804		-1.545569611	9.69E-08	1.09E-06
ENSMMUG00000005442	5'-nucleotidase ecto [Source:VGNC Symbol;Acc:VGNC:75537]	-1.414819646	4.62E-07	4.50E-06
ENSMMUG00000061590		-2.248342195	1.58E-05	0.000109006
ENSMMUG00000017186	pleckstrin homology domain interacting protein [Source:VGNC Symbol;Acc:VGNC:75803]	-1.919594089	1.65E-08	2.14E-07
ENSMMUG00000019261	collagen type XII alpha 1 chain [Source:VGNC Symbol;Acc:VGNC:71291]	-0.349840748	0.000279087	0.001442018
ENSMMUG00000017806	CD109 molecule [Source:VGNC Symbol;Acc:VGNC:70834]	0.523322519	0.000567869	0.00270398
ENSMMUG00000008133	solute carrier family 17 member 5 [Source:VGNC Symbol;Acc:VGNC:77405]	0.421838859	0.003348904	0.012526456
ENSMMUG00000060308		3.865503278	0.001135357	0.00492152
ENSMMUG00000002777	protein tyrosine phosphatase 4A1 [Source:VGNC Symbol;Acc:VGNC:83452]	-0.476745612	0.003358485	0.012558943
ENSMMUG00000001399	3-hydroxymethyl-3-methylglutaryl-CoA lyase like 1 [Source:VGNC Symbol;Acc:VGNC:73429]	-0.370247835	0.013053183	0.040053564
ENSMMUG00000051828		-1.868018659	0.013815152	0.042032953
ENSMMUG00000012784	tubulointerstitial nephritis antigen [Source:VGNC Symbol;Acc:VGNC:78382]	-1.278738564	0.011837011	0.036878563
ENSMMUG00000012781	muscular LMNA interacting protein [Source:VGNC Symbol;Acc:VGNC:83434]	-1.832207691	5.39E-09	7.62E-08
ENSMMUG00000011049	leucine rich repeat containing 1 [Source:VGNC Symbol;Acc:VGNC:81087]	-1.391729422	8.30E-05	0.00048243
ENSMMUG00000017137	glutathione S-transferase alpha 3 [Source:NCBI gene;Acc:709498]	-0.314123451	0.002215	0.008811616
ENSMMUG00000050626		-0.542106922	0.00574018	0.01987029
ENSMMUG00000052626	progestin and adipoQ receptor family member 8 [Source:NCBI gene;Acc:708418]	0.734115005	1.03E-09	1.64E-08
ENSMMUG00000026975	mml-mir-206 [Source:miRBase;Acc:MI0007667]	4.689959121	6.95E-06	5.25E-05
ENSMMUG00000016993	PKHD1 ciliary IPT domain containing fibrocystin/polyductin [Source:VGNC Symbol;Acc:VGNC:76010]	-1.793088921	3.80E-08	4.59E-07
ENSMMUG00000053984		2.540947267	9.17E-08	1.03E-06
ENSMMUG00000056168		3.676106225	0.001247576	0.005353351
ENSMMUG00000023002	adhesion G protein-coupled receptor F5 [Source:VGNC Symbol;Acc:VGNC:69471]	-0.378011634	0.000331149	0.001670397
ENSMMUG00000042154	ankyrin repeat domain 66 [Source:VGNC Symbol;Acc:VGNC:81024]	-0.583236861	0.000266451	0.001386168
ENSMMUG00000019254	phospholipase A2 group VII [Source:VGNC Symbol;Acc:VGNC:76158]	-0.708578881	0.000463011	0.002253723
ENSMMUG00000019252	tudor domain containing 6 [Source:VGNC Symbol;Acc:VGNC:79319]	-0.497022556	0.005300466	0.018598316
ENSMMUG00000019250	solute carrier family 25 member 27 [Source:VGNC Symbol;Acc:VGNC:77543]	-0.747795741	1.14E-05	8.16E-05
ENSMMUG00000055199	cytochrome P450, family 39, subfamily A, polypeptide 1 [Source:NCBI gene;Acc:704242]	1.950967721	4.15E-09	6.00E-08
ENSMMUG00000019246	regulator of calcineurin 2 [Source:VGNC Symbol;Acc:VGNC:82092]	1.932462103	2.97E-07	3.00E-06
ENSMMUG00000003511	chloride intracellular channel 5 [Source:VGNC Symbol;Acc:VGNC:71171]	-0.663761123	0.00054954	0.002625621

ENSMMUG00000021377	SPT3 homolog, SAGA and STAGA complex component [Source:VGNC Symbol;Acc:VGNC:78055]	-0.995851977	1.04E-06	9.35E-06
ENSMMUG00000014389	cell division cycle 5 like [Source:VGNC Symbol;Acc:VGNC:70801]	-0.877481138	0.001532116	0.006369659
ENSMMUG00000014388	spermatogenesis associated serine rich 1 [Source:VGNC Symbol;Acc:VGNC:77975]	-0.510868076	0.001490692	0.006221421
ENSMMUG00000060503	Metazoan signal recognition particle RNA [Source:RFAM;Acc:RF00017]	-1.02183119	0.000818644	0.003716362
ENSMMUG00000063288		-0.57401683	0.00043722	0.002140013
ENSMMUG00000013920	triggering receptor expressed on myeloid cells 2 [Source:VGNC Symbol;Acc:VGNC:79089]	-0.921869098	0.000768399	0.00351558
ENSMMUG00000062538	adenylate cyclase type 10 [Source:NCBI gene;Acc:719662]	-0.965182323	0.000266224	0.001385767
ENSMMUG00000021173	nuclear transcription factor Y subunit alpha [Source:VGNC Symbol;Acc:VGNC:75344]	-0.835796732	1.67E-09	2.56E-08
ENSMMUG00000021172	O-acyl-ADP-ribose deacylase 1 [Source:VGNC Symbol;Acc:VGNC:75444]	-0.373572414	0.016634063	0.048945301
ENSMMUG00000021171	apolipoprotein B mRNA editing enzyme catalytic subunit 2 [Source:VGNC Symbol;Acc:VGNC:69986]	-0.556707174	0.003021111	0.011471713
ENSMMUG00000021169	translocator protein 2 [Source:VGNC Symbol;Acc:VGNC:83960]	-0.928226572	1.81E-06	1.54E-05
ENSMMUG00000021168	unc-5 family C-terminal like [Source:VGNC Symbol;Acc:VGNC:79314]	-1.01118102	1.52E-11	3.29E-10
ENSMMUG00000013651	kinesin family member 6 [Source:VGNC Symbol;Acc:VGNC:73873]	2.357472009	0.015876952	0.047160028
ENSMMUG00000013644	potassium two pore domain channel subfamily K member 17 [Source:VGNC Symbol;Acc:VGNC:73828]	1.877294157	1.48E-11	3.22E-10
ENSMMUG00000023381	dynein axonemal heavy chain 8 [Source:VGNC Symbol;Acc:VGNC:71943]	0.481967177	1.07E-05	7.74E-05
ENSMMUG00000048502	Metazoan signal recognition particle RNA [Source:RFAM;Acc:RF00017]	1.129256243	0.000588587	0.002791601
ENSMMUG00000062055	glyoxalase I [Source:NCBI gene;Acc:719518]	0.683550293	7.14E-06	5.39E-05
ENSMMUG00000011575	coiled-coil domain containing 167 [Source:VGNC Symbol;Acc:VGNC:70833]	-1.40608804	0.000887756	0.00397531
ENSMMUG00000011573	cap methyltransferase 1 [Source:VGNC Symbol;Acc:VGNC:71271]	-1.022686576	0.000435169	0.002132952
ENSMMUG00000063198		3.845231717	5.21E-08	6.12E-07
ENSMMUG00000022193	FYVE, RhoGEF and PH domain containing 2 [Source:VGNC Symbol;Acc:VGNC:72643]	-1.658882086	0.003581599	0.013279947
ENSMMUG00000020558	serine/threonine kinase 38 [Source:VGNC Symbol;Acc:VGNC:78155]	1.346728711	0.000812109	0.003692673
ENSMMUG00000014508	potassium channel tetramerization domain containing 20 [Source:VGNC Symbol;Acc:VGNC:73872]	1.359065313	9.37E-10	1.50E-08
ENSMMUG00000055914		1.673016013	3.55E-09	5.20E-08
ENSMMUG00000015416	bromodomain and PHD finger containing 3 [Source:VGNC Symbol;Acc:VGNC:70309]	1.810173236	8.35E-13	2.19E-11
ENSMMUG00000023823	Y RNA [Source:RFAM;Acc:RF00019]	3.879556476	0.00107491	0.004701414
ENSMMUG00000000869	small nuclear ribonucleoprotein polypeptide C [Source:NCBI gene;Acc:718654]	3.819855776	1.61E-05	0.000110886
ENSMMUG00000063472		3.563936031	0.016942583	0.049797912
ENSMMUG00000047729	inositol 1,4,5-trisphosphate receptor type 3 [Source:NCBI gene;Acc:718040]	1.473853294	0.010532056	0.033391593
ENSMMUG00000004287	death domain associated protein [Source:VGNC Symbol;Acc:VGNC:71737]	-1.030526506	7.80E-05	0.00045664
ENSMMUG00000003876	zinc finger and BTB domain containing 22 [Source:VGNC Symbol;Acc:VGNC:78893]	-0.579097978	0.013437111	0.041069879
ENSMMUG00000003864	VPS52 subunit of GARP complex [Source:VGNC Symbol;Acc:VGNC:78551]	-0.668638139	0.000474279	0.002300526
ENSMMUG00000003850	collagen type XI alpha 2 chain [Source:VGNC Symbol;Acc:VGNC:71290]	-0.619172748	0.000624607	0.002931241
ENSMMUG00000019371	major histocompatibility complex, class II, DP alpha [Source:NCBI gene;Acc:717992]	-1.143272314	0.00530111	0.018598316
ENSMMUG00000023138	bromodomain containing 2 [Source:VGNC Symbol;Acc:VGNC:83422]	-1.981712492	0.001583965	0.006559935
ENSMMUG00000006828	valyl-tRNA synthetase 1 [Source:VGNC Symbol;Acc:VGNC:78755]	-3.265980134	2.52E-05	0.000166147

ENSMMUG00000006827	von Willebrand factor A domain containing 7 [Source:VGNC Symbol;Acc:VGNC:78941]	-3.033539341	3.04E-05	0.00019656
ENSMMUG00000009702	mutS homolog 5 [Source:NCBI gene;Acc:715494]	-1.993246939	0.000274167	0.00142026
ENSMMUG00000058534		2.537432041	2.41E-07	2.48E-06
ENSMMUG00000015693	lymphocyte antigen 6 family member G6C [Source:VGNC Symbol;Acc:VGNC:74285]	1.992986644	4.63E-06	3.65E-05
ENSMMUG00000053324	lymphocyte antigen 6 complex locus protein G6d [Source:NCBI gene;Acc:715304]	1.574237537	1.35E-09	2.10E-08
ENSMMUG00000015686	abhydrolase domain containing 16A [Source:VGNC Symbol;Acc:VGNC:69393]	1.767908351	2.97E-20	1.90E-18
ENSMMUG00000054159	lymphocyte antigen 6 family member G5C [Source:VGNC Symbol;Acc:VGNC:83431]	2.275825805	0.001936151	0.007828906
ENSMMUG00000005294	casein kinase 2 beta [Source:NCBI gene;Acc:715807]	1.896266164	7.23E-07	6.76E-06
ENSMMUG00000005293	G-patch domain and ankyrin repeats 1 [Source:VGNC Symbol;Acc:VGNC:81073]	1.903122903	7.47E-06	5.61E-05
ENSMMUG00000005302	BAG cochaperone 6 [Source:VGNC Symbol;Acc:VGNC:81044]	2.09090154	1.11E-28	1.71E-26
ENSMMUG00000005303	apolipoprotein M [Source:VGNC Symbol;Acc:VGNC:69825]	1.829595236	0.000886052	0.00397095
ENSMMUG00000005297	proline rich coiled-coil 2A [Source:VGNC Symbol;Acc:VGNC:83448]	1.782979644	4.21E-18	2.17E-16
ENSMMUG00000025918	small nucleolar RNA, H/ACA box 38 [Source:HGNC Symbol;Acc:HGNC:32631]	2.14871073	0.000611642	0.002886304
ENSMMUG00000049735	allograft inflammatory factor 1 [Source:NCBI gene;Acc:574115]	1.896109003	4.35E-18	2.23E-16
ENSMMUG00000008854	natural cytotoxicity triggering receptor 3 [Source:VGNC Symbol;Acc:VGNC:74990]	1.871828405	3.25E-14	1.06E-12
ENSMMUG00000008839	DExD-box helicase 39B [Source:VGNC Symbol;Acc:VGNC:71621]	1.244951543	0.002746525	0.010580912
ENSMMUG00000019961	protein phosphatase 1 regulatory subunit 18 [Source:NCBI gene;Acc:712176]	4.188593435	1.38E-06	1.20E-05
ENSMMUG00000053627		3.51163748	2.60E-08	3.25E-07
ENSMMUG00000011682	ribonuclease P/MRP subunit p21 [Source:NCBI gene;Acc:713056]	4.01209171	6.85E-09	9.46E-08
ENSMMUG00000060527		-0.829007281	0.001326676	0.005634156
ENSMMUG00000023679	tripartite motif containing 26 [Source:VGNC Symbol;Acc:VGNC:78854]	-1.138799652	7.25E-05	0.000428113
ENSMMUG00000002890	tripartite motif containing 15 [Source:VGNC Symbol;Acc:VGNC:83958]	-1.25752265	1.01E-07	1.13E-06
ENSMMUG00000012129	tripartite motif containing 10 [Source:VGNC Symbol;Acc:VGNC:78550]	-1.271331607	2.05E-08	2.61E-07
ENSMMUG00000058580	tripartite motif containing 40 [Source:NCBI gene;Acc:100427925]	-1.242729091	2.03E-07	2.12E-06
ENSMMUG00000062264		-1.345620519	0.000806003	0.003669667
ENSMMUG00000012124	ring finger protein 39 [Source:VGNC Symbol;Acc:VGNC:83458]	-1.122800062	5.00E-05	0.000307084
ENSMMUG00000012120	zinc ribbon domain containing 1 [Source:VGNC Symbol;Acc:VGNC:78912]	-1.520461911	1.08E-09	1.71E-08
ENSMMUG00000055272	putative uncharacterized protein ZNRD1-AS1 [Source:NCBI gene;Acc:712069]	-1.05339398	9.08E-05	0.000523307
ENSMMUG00000056544		-1.586330602	0.000245843	0.001291289
ENSMMUG00000037625		-1.443131394	0.000717496	0.003303169
ENSMMUG00000053561		3.304291058	0.005739319	0.01987029
ENSMMUG00000029883	olfactory receptor, family 2, subfamily J, member 3 [Source:NCBI gene;Acc:708943]	3.586003798	0.000473225	0.002296206
ENSMMUG00000059442		2.255927858	0.002240984	0.008887251
ENSMMUG00000004617	glutathione peroxidase 6 [Source:VGNC Symbol;Acc:VGNC:73154]	-0.745881144	0.012085128	0.037485015
ENSMMUG00000012396	zinc finger and SCAN domain containing 23 [Source:VGNC Symbol;Acc:VGNC:79313]	-0.763353752	0.000190409	0.001024993
ENSMMUG00000011675	zinc finger and SCAN domain containing 12 [Source:VGNC Symbol;Acc:VGNC:78910]	-0.689165469	9.31E-06	6.82E-05
ENSMMUG00000005964	CMP-N-acetylneuraminic acid hydroxylase [Source:NCBI gene;Acc:574186]	0.910767106	0.00248022	0.009696161
ENSMMUG00000049021	Metazoan signal recognition particle RNA [Source:RFAM;Acc:RF00017]	1.667192851	0.01024118	0.032612329
ENSMMUG00000058151	chromosome 4 C6orf62 homolog [Source:NCBI gene;Acc:708218]	-1.029651447	1.28E-10	2.40E-09

ENSMMUG00000051037		4.611350655	8.06E-16	3.21E-14
ENSMMUG00000054532		-0.747285372	0.001954141	0.007894818
ENSMMUG00000052442		-0.77664769	0.00014627	0.000806753
ENSMMUG00000014691	inhibitor of DNA binding 4, HLH protein [Source:VGNC Symbol;Acc:VGNC:73557]	-0.74007047	7.57E-05	0.000444707
ENSMMUG00000063576	Metazoan signal recognition particle RNA [Source:RFAM;Acc:RF00017]	-1.532910112	0.000204654	0.001093222
ENSMMUG00000052333		0.709710656	0.001454117	0.006095989
ENSMMUG00000049917	U6 spliceosomal RNA [Source:RFAM;Acc:RF00026]	0.739435235	0.000627191	0.00294052
ENSMMUG00000019763	DEK proto-oncogene [Source:NCBI gene;Acc:712460]	0.540556566	0.000938987	0.004165636
ENSMMUG00000052891		1.205726693	0.002131595	0.008525773
ENSMMUG00000020289	CD83 molecule [Source:VGNC Symbol;Acc:VGNC:70783]	-1.504230813	5.29E-12	1.24E-10
ENSMMUG00000044473		-0.636657617	0.001521987	0.006338286
ENSMMUG00000020040	phosphatase and actin regulator 1 [Source:VGNC Symbol;Acc:VGNC:75841]	0.773249003	0.01476871	0.044384692
ENSMMUG00000058800		1.287555734	2.56E-11	5.38E-10
ENSMMUG00000016135	neural precursor cell expressed, developmentally down-regulated 9 [Source:VGNC Symbol;Acc:VGNC:75304]	-0.875719419	9.16E-05	0.000527582
ENSMMUG00000046520	small integral membrane protein 13 [Source:NCBI gene;Acc:703387]	-0.680557578	0.007348154	0.024651476
ENSMMUG00000043161	endogenous retrovirus group FRD member 1, envelope [Source:VGNC Symbol;Acc:VGNC:81068]	-1.266202171	3.04E-06	2.48E-05
ENSMMUG00000005532	male germ cell associated kinase [Source:VGNC Symbol;Acc:VGNC:74480]	0.611773061	0.005060959	0.017881034
ENSMMUG00000018788	transmembrane protein 14B [Source:NCBI gene;Acc:698237]	0.564885093	0.006184957	0.021191247
ENSMMUG00000018787	PAK1 interacting protein 1 [Source:VGNC Symbol;Acc:VGNC:75661]	1.052110025	1.99E-07	2.08E-06
ENSMMUG00000061586	chromosome 4 C6orf52 homolog [Source:NCBI gene;Acc:702114]	0.884062078	1.01E-06	9.12E-06
ENSMMUG00000026665	Y RNA [Source:RFAM;Acc:RF00019]	1.120082651	0.012293353	0.038038325
ENSMMUG00000006186	transcription factor AP-2 alpha [Source:VGNC Symbol;Acc:VGNC:78320]	-1.332623016	0.002053585	0.008247395
ENSMMUG00000038560	eukaryotic translation elongation factor 1 epsilon 1 [Source:NCBI gene;Acc:695426]	-1.143576056	5.45E-17	2.51E-15
ENSMMUG00000044859		-1.309427395	0.009173986	0.029772462
ENSMMUG00000015577	RIO kinase 1 [Source:VGNC Symbol;Acc:VGNC:76767]	-2.015899123	2.22E-09	3.36E-08
ENSMMUG00000060973		0.986478505	4.00E-09	5.80E-08
ENSMMUG00000052268		-2.938147328	0.003069546	0.011617881
ENSMMUG00000023558	exocyst complex component 2 [Source:VGNC Symbol;Acc:VGNC:72338]	-1.050238042	0.014856269	0.044600613
ENSMMUG00000056560		-0.878510336	0.000894424	0.004000801
ENSMMUG00000058002	zinc finger protein 669-like [Source:NCBI gene;Acc:696282]	0.831639706	1.31E-09	2.05E-08
ENSMMUG00000062676		-1.027587001	1.98E-05	0.000134638
ENSMMUG00000065031		-1.047080295	2.37E-09	3.56E-08
ENSMMUG00000023139	small nuclear ribonucleoprotein polypeptide N [Source:VGNC Symbol;Acc:VGNC:78437]	2.507866531	1.07E-10	2.04E-09
ENSMMUG00000051503	nuclear pore associated protein 1 [Source:NCBI gene;Acc:719891]	2.717768488	1.33E-06	1.16E-05
ENSMMUG00000046479		-0.587523055	0.005415772	0.018947062
ENSMMUG00000058972		-2.466875527	0.014569154	0.043936183

ENSMMUG00000021656	golgin subfamily A member 8S [Source:NCBI gene;Acc:722977]	-3.057333128	0.001477125	0.006179513
ENSMMUG00000054315		-2.460244622	5.11E-07	4.92E-06
ENSMMUG00000055117		-2.005477574	8.56E-07	7.85E-06
ENSMMUG00000063268		-1.845587906	2.11E-07	2.19E-06
ENSMMUG00000009430	transient receptor potential cation channel subfamily M member 1 [Source:VGNC Symbol;Acc:VGNC:79964]	0.575206611	2.97E-08	3.66E-07
ENSMMUG00000005270	ryanodine receptor 3 [Source:NCBI gene;Acc:694996]	0.62680034	1.38E-06	1.21E-05
ENSMMUG00000014227	cholinergic receptor muscarinic 5 [Source:VGNC Symbol;Acc:VGNC:71044]	3.552823086	0.000641359	0.002990947
ENSMMUG00000002337	aquarius intron-binding spliceosomal factor [Source:VGNC Symbol;Acc:VGNC:69925]	-1.261723757	8.10E-05	0.00047306
ENSMMUG00000046486	zinc finger protein 770 [Source:NCBI gene;Acc:697740]	-0.805702757	0.002195281	0.008740611
ENSMMUG00000009668	transmembrane and coiled-coil domains 5A [Source:VGNC Symbol;Acc:VGNC:79725]	2.55437665	0.000378387	0.001874957
ENSMMUG00000018332	family with sequence similarity 98 member B [Source:VGNC Symbol;Acc:VGNC:72583]	-0.893513794	1.26E-05	8.92E-05
ENSMMUG00000018333	RAS guanyl releasing protein 1 [Source:VGNC Symbol;Acc:VGNC:76671]	-1.580695294	0.012306805	0.038054758
ENSMMUG00000057671		2.882787043	0.007398776	0.024779803
ENSMMUG00000057460		-1.011416535	0.014996253	0.044943889
ENSMMUG00000016686	BUB1 mitotic checkpoint serine/threonine kinase B [Source:VGNC Symbol;Acc:VGNC:70293]	0.706604398	2.46E-05	0.000162474
ENSMMUG00000020534	p21 (RAC1) activated kinase 6 [Source:NCBI gene;Acc:701589]	2.743051619	8.35E-50	7.32E-47
ENSMMUG00000043229	carbohydrate sulfotransferase 14 [Source:NCBI gene;Acc:703115]	-1.724796842	1.80E-05	0.000122801
ENSMMUG00000001482	coiled-coil domain containing 32 [Source:VGNC Symbol;Acc:VGNC:70891]	-1.112941327	2.37E-06	1.98E-05
ENSMMUG00000001483	RNA pseudouridine synthase domain containing 2 [Source:VGNC Symbol;Acc:VGNC:77091]	-0.929268003	0.003142604	0.011830534
ENSMMUG00000048910	kinetochore scaffold 1 [Source:VGNC Symbol;Acc:VGNC:84366]	-0.846395348	0.000124183	0.00069518
ENSMMUG00000000306	regulator of microtubule dynamics 3 [Source:VGNC Symbol;Acc:VGNC:76821]	-0.724704114	0.007000653	0.023609948
ENSMMUG00000013160	DnaJ heat shock protein family (Hsp40) member C17 [Source:VGNC Symbol;Acc:VGNC:84335]	1.619175014	1.18E-05	8.46E-05
ENSMMUG00000013991	exonuclease 3'-5' domain containing 1 [Source:VGNC Symbol;Acc:VGNC:72390]	-0.821207802	2.23E-05	0.000148955
ENSMMUG00000001532	glucosidase alpha, neutral C [Source:NCBI gene;Acc:708126]	0.751054636	9.10E-08	1.03E-06
ENSMMUG00000001534	calpain 3 [Source:VGNC Symbol;Acc:VGNC:70533]	0.677222923	0.000470925	0.002286628
ENSMMUG00000063886	Metazoan signal recognition particle RNA [Source:RFAM;Acc:RF00017]	2.654750484	0.006090185	0.020921719
ENSMMUG00000001547	zinc finger protein 106 [Source:VGNC Symbol;Acc:VGNC:78831]	1.272323216	2.29E-23	1.97E-21
ENSMMUG00000064341	U6 spliceosomal RNA [Source:RFAM;Acc:RF00026]	3.846895993	1.28E-05	9.05E-05
ENSMMUG00000008093	StAR related lipid transfer domain containing 9 [Source:NCBI gene;Acc:708953]	-0.90147626	3.84E-09	5.57E-08
ENSMMUG00000015123	codanin 1 [Source:VGNC Symbol;Acc:VGNC:70788]	-0.524458607	0.013070335	0.040097419
ENSMMUG00000010036	tau tubulin kinase 2 [Source:VGNC Symbol;Acc:VGNC:81598]	-0.420864105	0.000400066	0.001976091
ENSMMUG00000022256	transglutaminase 5 [Source:VGNC Symbol;Acc:VGNC:78341]	-1.137242792	5.85E-05	0.000354314
ENSMMUG00000022251	transglutaminase 7 [Source:VGNC Symbol;Acc:VGNC:79880]	-0.39580922	0.004939135	0.017547881
ENSMMUG00000055498	leucine carboxyl methyltransferase 2 [Source:NCBI gene;Acc:710047]	0.708002859	1.69E-05	0.000116216
ENSMMUG00000017067	adenosine deaminase like [Source:VGNC Symbol;Acc:VGNC:69555]	-0.370039028	0.006736317	0.022806222

ENSMMUG00000017070	zinc finger and SCAN domain containing 29 [Source:VGNC Symbol;Acc:VGNC:79633]	-0.702648407	1.21E-05	8.64E-05
ENSMMUG00000017076	tubulin gamma complex associated protein 4 [Source:VGNC Symbol;Acc:VGNC:79045]	-0.701270034	0.000735649	0.003377866
ENSMMUG00000007255	microtubule associated protein 1A [Source:VGNC Symbol;Acc:VGNC:74489]	-1.600019639	0.000195064	0.001047914
ENSMMUG00000007860	diphosphoinositol pentakisphosphate kinase 1 [Source:VGNC Symbol;Acc:VGNC:76127]	-0.736487444	0.011074253	0.034874073
ENSMMUG00000057376	inositol hexakisphosphate and diphosphoinositol-pentakisphosphate kinase 1-like [Source:NCBI gene;Acc:717098]	0.454220629	0.002274112	0.008985595
ENSMMUG00000016742	CTD small phosphatase like 2 [Source:VGNC Symbol;Acc:VGNC:71593]	0.359195964	0.004257203	0.015470005
ENSMMUG00000014291	SPG11 vesicle trafficking associated, spatacsin [Source:VGNC Symbol;Acc:VGNC:77856]	0.588404641	0.003068971	0.011617881
ENSMMUG00000005856	glycine amidinotransferase [Source:VGNC Symbol;Acc:VGNC:72892]	-1.478143857	1.26E-12	3.22E-11
ENSMMUG00000049265	Metazoan signal recognition particle RNA [Source:RFAM;Acc:RF00017]	1.569569787	0.000130089	0.000725919
ENSMMUG00000062254		1.691317977	7.91E-06	5.90E-05
ENSMMUG00000012144	semaphorin 6D [Source:VGNC Symbol;Acc:VGNC:77250]	2.935675533	0.004585773	0.016471901
ENSMMUG00000016896	solute carrier family 12 member 1 [Source:VGNC Symbol;Acc:VGNC:77385]	-1.061589518	1.50E-08	1.95E-07
ENSMMUG00000009837	SECIS binding protein 2 like [Source:VGNC Symbol;Acc:VGNC:77325]	-0.691325768	1.17E-06	1.04E-05
ENSMMUG00000009839	galactokinase 2 [Source:VGNC Symbol;Acc:VGNC:72870]	-0.71429227	0.002447647	0.009600938
ENSMMUG00000049831		-0.693079719	0.001449245	0.006078843
ENSMMUG00000050608		1.340774246	3.73E-08	4.52E-07
ENSMMUG00000003684	ATPase phospholipid transporting 8B4 (putative) [Source:VGNC Symbol;Acc:VGNC:84311]	1.400610775	1.03E-11	2.30E-10
ENSMMUG00000057283		1.414999469	1.06E-05	7.64E-05
ENSMMUG00000002879	GA binding protein transcription factor subunit beta 1 [Source:VGNC Symbol;Acc:VGNC:72845]	-0.744802842	7.62E-05	0.000447801
ENSMMUG00000060278	Metazoan signal recognition particle RNA [Source:RFAM;Acc:RF00017]	-2.824288829	4.50E-07	4.39E-06
ENSMMUG00000014884	ubiquitin specific peptidase 8 [Source:VGNC Symbol;Acc:VGNC:78748]	-0.752450645	4.49E-09	6.47E-08
ENSMMUG00000008575	transient receptor potential cation channel subfamily M member 7 [Source:VGNC Symbol;Acc:VGNC:79106]	-0.607431489	2.76E-09	4.09E-08
ENSMMUG00000000754	Dmx like 2 [Source:VGNC Symbol;Acc:VGNC:71936]	2.164970145	7.92E-13	2.09E-11
ENSMMUG00000017432	tropomodulin 2 [Source:VGNC Symbol;Acc:VGNC:78571]	-0.89053191	9.02E-07	8.22E-06
ENSMMUG00000002345	tropomodulin 3 [Source:VGNC Symbol;Acc:VGNC:78572]	0.870971121	3.13E-26	3.91E-24
ENSMMUG00000029652	mitogen-activated protein kinase 6 [Source:NCBI gene;Acc:694998]	3.471262147	0.007904135	0.026246668
ENSMMUG00000017374	G protein subunit beta 5 [Source:VGNC Symbol;Acc:VGNC:73097]	-2.672747738	4.88E-06	3.82E-05
ENSMMUG00000007504	myosin VC [Source:VGNC Symbol;Acc:VGNC:75117]	-2.495692155	4.94E-05	0.000303695
ENSMMUG00000007509	myosin VA [Source:VGNC Symbol;Acc:VGNC:75116]	-0.733884345	5.47E-05	0.000333299
ENSMMUG00000048644		-1.187026205	0.008844134	0.028855581
ENSMMUG00000001423	family with sequence similarity 214 member A [Source:VGNC Symbol;Acc:VGNC:72354]	-0.382460304	0.000855046	0.003849217
ENSMMUG00000010797	phosphatidylinositol glycan anchor biosynthesis class B [Source:VGNC Symbol;Acc:VGNC:75985]	0.569137772	0.004370182	0.015819226
ENSMMUG00000029638	chromosome 7 C15orf65 homolog [Source:VGNC Symbol;Acc:VGNC:70689]	0.633151998	0.013354015	0.040860431
ENSMMUG00000000863	dynein axonemal assembly factor 4 [Source:VGNC Symbol;Acc:VGNC:71881]	1.335026856	1.29E-10	2.41E-09
ENSMMUG00000008718	zinc finger protein 280D [Source:NCBI gene;Acc:699509]	-1.668185672	9.03E-16	3.58E-14
ENSMMUG00000004091	transcription factor 12 [Source:VGNC Symbol;Acc:VGNC:78283]	-0.916162084	1.37E-08	1.79E-07

ENSMMUG00000012690	myocardial zonula adherens protein [Source:NCBI gene;Acc:700247]	-2.754359446	4.82E-05	0.000297013
ENSMMUG00000063524		-0.355374692	0.00778574	0.025902595
ENSMMUG00000012933	myosin IE [Source:VGNC Symbol;Acc:VGNC:75114]	0.325900106	0.005500266	0.019196173
ENSMMUG00000018426	glucosaminyl (N-acetyl) transferase 3, mucin type [Source:VGNC Symbol;Acc:VGNC:84342]	-2.187236595	0.016310421	0.048161937
ENSMMUG00000020348	BCL2 interacting protein 2 [Source:VGNC Symbol;Acc:VGNC:70272]	-2.026551306	3.92E-06	3.14E-05
ENSMMUG00000049749		-0.628504177	0.001981162	0.007992473
ENSMMUG00000011503	talin 2 [Source:VGNC Symbol;Acc:VGNC:78385]	0.941995473	0.011890137	0.037019404
ENSMMUG00000013460	RAB8B, member RAS oncogene family [Source:VGNC Symbol;Acc:VGNC:81550]	0.623999874	0.000129662	0.000724113
ENSMMUG00000012114	ubiquitin specific peptidase 3 [Source:VGNC Symbol;Acc:VGNC:78746]	3.405265682	0.012228379	0.037862346
ENSMMUG00000012117	HECT and RLD domain containing E3 ubiquitin protein ligase family member 1 [Source:VGNC Symbol;Acc:VGNC:73364]	0.472196869	1.03E-07	1.15E-06
ENSMMUG00000049375		0.623140245	3.99E-05	0.000251303
ENSMMUG00000018177	death associated protein kinase 2 [Source:VGNC Symbol;Acc:VGNC:71733]	0.983140458	0.000201254	0.00107635
ENSMMUG00000013270	RNA binding protein, mRNA processing factor 2 [Source:VGNC Symbol;Acc:VGNC:76511]	1.698121893	0.001148969	0.004975908
ENSMMUG00000010949	SPG21 abhydrolase domain containing, maspardin [Source:VGNC Symbol;Acc:VGNC:77970]	0.869694653	9.00E-08	1.02E-06
ENSMMUG00000005749	poly(ADP-ribose) polymerase family member 16 [Source:VGNC Symbol;Acc:VGNC:84416]	-0.32698077	0.011552231	0.036160029
ENSMMUG00000015450	3-hydroxyacyl-CoA dehydratase 3 [Source:VGNC Symbol;Acc:VGNC:73225]	-0.510291725	0.008813281	0.028781704
ENSMMUG00000008095	integrator complex subunit 14 [Source:VGNC Symbol;Acc:VGNC:73650]	-0.521561114	9.36E-05	0.000537989
ENSMMUG00000022056	DENN domain containing 4A [Source:VGNC Symbol;Acc:VGNC:71697]	-0.605178628	8.11E-05	0.000473147
ENSMMUG00000054184	Metazoan signal recognition particle RNA [Source:RFAM;Acc:RF00017]	0.971861569	2.39E-15	8.92E-14
ENSMMUG00000035748	U6 spliceosomal RNA [Source:RFAM;Acc:RF00026]	1.297668232	8.70E-05	0.000503138
ENSMMUG00000008006	RAB11A, member RAS oncogene family [Source:VGNC Symbol;Acc:VGNC:81517]	0.753847761	2.56E-08	3.20E-07
ENSMMUG00000008007	multiple EGF like domains 11 [Source:VGNC Symbol;Acc:VGNC:84376]	1.112347679	1.31E-09	2.05E-08
ENSMMUG00000052828		1.071520619	0.000122634	0.00068788
ENSMMUG00000003693	DIS3 like exosome 3'-5' exoribonuclease [Source:VGNC Symbol;Acc:VGNC:71796]	-3.588001302	0.000343323	0.001723744
ENSMMUG00000016840	mitogen-activated protein kinase kinase 1 [Source:VGNC Symbol;Acc:VGNC:81452]	-1.032333542	0.000269236	0.001396778
ENSMMUG00000015458	IQ motif containing H [Source:VGNC Symbol;Acc:VGNC:73655]	-0.489961893	0.01100927	0.034708405
ENSMMUG00000004026	integrin subunit alpha 11 [Source:VGNC Symbol;Acc:VGNC:73774]	0.544086392	7.68E-06	5.75E-05
ENSMMUG00000006230	NADPH oxidase 5 [Source:VGNC Symbol;Acc:VGNC:82198]	0.309336689	0.011093313	0.034918412
ENSMMUG00000007570	uveal autoantigen with coiled-coil domains and ankyrin repeats [Source:VGNC Symbol;Acc:VGNC:78995]	-1.180502481	1.21E-11	2.68E-10
ENSMMUG00000006490	ariadne RBR E3 ubiquitin protein ligase 1 [Source:VGNC Symbol;Acc:VGNC:84308]	-0.463593351	0.015344494	0.04582109
ENSMMUG00000059258		0.703720431	6.98E-05	0.000413168
ENSMMUG00000057906		0.919647131	1.66E-15	6.33E-14
ENSMMUG00000063495		1.078052796	1.29E-16	5.62E-15
ENSMMUG00000001568	AT-rich interaction domain 3B [Source:VGNC Symbol;Acc:VGNC:69827]	1.099284719	2.19E-07	2.27E-06
ENSMMUG00000022481	cytochrome P450, family 1, subfamily A, polypeptide 1 [Source:NCBI gene;Acc:678694]	0.902496257	2.62E-11	5.46E-10
ENSMMUG00000015267	C-terminal Src kinase [Source:VGNC Symbol;Acc:VGNC:81380]	0.549587827	1.19E-07	1.31E-06
ENSMMUG00000008529	chromosome 7 C15orf39 homolog [Source:VGNC Symbol;Acc:VGNC:70620]	1.643787963	9.41E-07	8.56E-06
ENSMMUG00000061079	golgin subfamily A member 6C [Source:NCBI gene;Acc:715898]	1.84759412	2.69E-29	4.55E-27
ENSMMUG00000016104	chondroitin sulfate proteoglycan 4 [Source:VGNC Symbol;Acc:VGNC:71525]	0.765347593	2.87E-06	2.35E-05
ENSMMUG00000053989		0.793607781	7.29E-07	6.81E-06
ENSMMUG00000058835		0.8763964	0.004361447	0.015799682

ENSMMUG00000050124	transmembrane protein 266 [Source:NCBI gene;Acc:707482]	0.425621955	0.009565249	0.030806955
ENSMMUG00000010171	proline-serine-threonine phosphatase interacting protein 1 [Source:VGNC Symbol;Acc:VGNC:76459]	-0.560904831	7.47E-05	0.000439112
ENSMMUG00000042378	pseudopodium enriched atypical kinase 1 [Source:VGNC Symbol;Acc:VGNC:84419]	0.521727381	0.000116814	0.000657335
ENSMMUG00000054259		0.962801659	0.003828549	0.014076492
ENSMMUG00000058749	golgin subfamily A member 6C [Source:NCBI gene;Acc:716687]	-3.292926282	0.004610126	0.016542425
ENSMMUG00000014207	WD repeat domain 61 [Source:VGNC Symbol;Acc:VGNC:79843]	-1.095500649	0.00153006	0.006362997
ENSMMUG00000045680	iron responsive element binding protein 2 [Source:VGNC Symbol;Acc:VGNC:84360]	-0.989987389	0.003848852	0.01414743
ENSMMUG00000059973		-0.908658405	3.96E-07	3.92E-06
ENSMMUG00000064497		-2.928431852	0.002222757	0.00883245
ENSMMUG00000056992	zinc finger AN1-type containing 6 [Source:NCBI gene;Acc:717543]	0.825754045	0.007464032	0.024968541
ENSMMUG00000009361	fumarylacetoacetate hydrolase [Source:NCBI gene;Acc:712716]	1.846145557	2.92E-13	8.09E-12
ENSMMUG00000054653	Metazoan signal recognition particle RNA [Source:RFAM;Acc:RF00017]	3.165482907	0.000996495	0.004399826
ENSMMUG00000054825		-0.657032921	0.013329666	0.040803734
ENSMMUG00000009409	mesoderm development LRP chaperone [Source:VGNC Symbol;Acc:VGNC:74507]	-0.66131561	0.011118764	0.034990671
ENSMMUG00000011564	transmembrane channel like 3 [Source:VGNC Symbol;Acc:VGNC:79360]	3.857483435	3.38E-05	0.000216291
ENSMMUG00000057295		0.376303555	0.008616568	0.028289755
ENSMMUG00000003002		-0.791943111	0.001429081	0.006005396
ENSMMUG00000009087	HDGF like 3 [Source:VGNC Symbol;Acc:VGNC:84346]	-1.146099393	8.65E-08	9.80E-07
ENSMMUG00000009084	transmembrane 6 superfamily member 1 [Source:VGNC Symbol;Acc:VGNC:78566]	-0.843279542	0.000110435	0.000624447
ENSMMUG00000011328	cytoplasmic polyadenylation element binding protein 1 [Source:NCBI gene;Acc:694357]	1.059774349	0.000200684	0.00107412
ENSMMUG00000019838	phosphodiesterase 8A [Source:VGNC Symbol;Acc:VGNC:75924]	-0.857650399	2.08E-05	0.000140448
ENSMMUG00000056737		-1.38446522	3.12E-05	0.000201617
ENSMMUG00000049563		-1.470477743	4.71E-09	6.73E-08
ENSMMUG00000016772	solute carrier family 28 member 1 [Source:VGNC Symbol;Acc:VGNC:77444]	0.742291958	5.52E-09	7.79E-08
ENSMMUG00000010432	zinc finger protein 592 [Source:VGNC Symbol;Acc:VGNC:79762]	0.369050899	0.000750397	0.003438828
ENSMMUG00000063195		0.367233175	0.005653105	0.019617297
ENSMMUG00000057531	mitochondrial ribosomal protein S11 [Source:NCBI gene;Acc:697743]	-0.970633624	0.00022256	0.001180394
ENSMMUG00000063799		-0.480412056	0.002126973	0.008512141
ENSMMUG00000011155	FA complementation group I [Source:VGNC Symbol;Acc:VGNC:72575]	0.911269754	9.60E-08	1.08E-06
ENSMMUG00000022678	Rh family C glycoprotein [Source:VGNC Symbol;Acc:VGNC:84427]	-0.970683174	0.000666443	0.00309557
ENSMMUG00000015394	WD repeat domain 93 [Source:VGNC Symbol;Acc:VGNC:79642]	0.96843294	1.98E-17	9.46E-16
ENSMMUG00000010586	mesoderm posterior bHLH transcription factor 1 [Source:VGNC Symbol;Acc:VGNC:74618]	1.336902262	0.001847743	0.007521304
ENSMMUG00000003584	mesoderm posterior bHLH transcription factor 2 [Source:VGNC Symbol;Acc:VGNC:74619]	0.936017623	9.22E-15	3.22E-13
ENSMMUG00000014983	alanyl aminopeptidase, membrane [Source:VGNC Symbol;Acc:VGNC:69943]	1.049696574	5.84E-14	1.82E-12
ENSMMUG00000029507	adaptor related protein complex 3 subunit sigma 2 [Source:NCBI gene;Acc:701217]	0.766639898	1.69E-08	2.19E-07
ENSMMUG00000023816	semaphorin 4B [Source:VGNC Symbol;Acc:VGNC:77165]	2.234473045	3.27E-28	4.88E-26
ENSMMUG00000004376	calcium and integrin binding 1 [Source:VGNC Symbol;Acc:VGNC:71189]	2.320130113	3.01E-26	3.84E-24
ENSMMUG00000050794	zinc finger protein 774 [Source:NCBI gene;Acc:702985]	-0.908021858	0.004367016	0.015815772

ENSMMUG00000019526	BLM RecQ like helicase [Source:VGNC Symbol;Acc:VGNC:70254]	2.161261369	5.12E-12	1.20E-10
ENSMMUG00000062217	HD domain containing 3 [Source:NCBI gene;Acc:715621]	2.500176826	1.09E-13	3.20E-12
ENSMMUG00000005106	unc-45 myosin chaperone A [Source:VGNC Symbol;Acc:VGNC:79858]	2.782395154	0.000331722	0.001672685
ENSMMUG00000005108	RCC1 domain containing 1 [Source:VGNC Symbol;Acc:VGNC:76876]	2.661087734	2.79E-11	5.78E-10
ENSMMUG00000007534	protein regulator of cytokinesis 1 [Source:NCBI gene;Acc:710182]	2.408934717	4.63E-07	4.50E-06
ENSMMUG00000008399	synaptic vesicle glycoprotein 2B [Source:VGNC Symbol;Acc:VGNC:78140]	0.806196714	5.52E-08	6.45E-07
ENSMMUG00000063322		1.073740807	2.16E-08	2.73E-07
ENSMMUG00000065319		4.731127229	2.62E-09	3.89E-08
ENSMMUG00000060063		1.216341652	2.39E-08	3.01E-07
ENSMMUG00000006659	aldehyde dehydrogenase 1 family member A3 [Source:VGNC Symbol;Acc:VGNC:81353]	2.151728321	3.99E-13	1.09E-11
ENSMMUG00000003034	olfactory receptor 4K15 [Source:NCBI gene;Acc:698499]	-0.976929909	0.015003317	0.044955453
ENSMMUG00000058162		2.440055661	0.007315021	0.024563825
ENSMMUG00000016255	telomerase associated protein 1 [Source:VGNC Symbol;Acc:VGNC:78310]	0.545274786	0.001293155	0.005516857
ENSMMUG00000048039	apurinic/apyrimidinic endodeoxyribonuclease 1 [Source:VGNC Symbol;Acc:VGNC:84305]	1.637884048	1.67E-05	0.000114983
ENSMMUG00000019126	purine nucleoside phosphorylase [Source:NCBI gene;Acc:704183]	2.30217164	1.25E-11	2.76E-10
ENSMMUG00000015599	probable ribonuclease 11 [Source:NCBI gene;Acc:703578]	-0.290915995	0.008825734	0.028814549
ENSMMUG00000020747	NDRG family member 2 [Source:VGNC Symbol;Acc:VGNC:74953]	-2.116591892	0.006747357	0.022838085
ENSMMUG00000050445	ribonuclease A family member 8 [Source:NCBI gene;Acc:716333]	-3.016151794	0.003063242	0.011603417
ENSMMUG00000006457	Rho guanine nucleotide exchange factor 40 [Source:VGNC Symbol;Acc:VGNC:69907]	-2.822107849	1.05E-06	9.47E-06
ENSMMUG00000014809	heterogeneous nuclear ribonucleoprotein C [Source:VGNC Symbol;Acc:VGNC:84348]	-2.494694231	0.000943271	0.004183265
ENSMMUG00000021552	RPGR interacting protein 1 [Source:VGNC Symbol;Acc:VGNC:77007]	-2.400800485	1.81E-07	1.91E-06
ENSMMUG00000055229		-2.494120032	4.20E-18	2.17E-16
ENSMMUG00000015000	SPT16 homolog, facilitates chromatin remodeling subunit [Source:VGNC Symbol;Acc:VGNC:78054]	-2.344370064	1.04E-12	2.68E-11
ENSMMUG00000015002	chromodomain helicase DNA binding protein 8 [Source:VGNC Symbol;Acc:VGNC:71060]	-2.114255311	8.70E-09	1.17E-07
ENSMMUG00000015004	TOX high mobility group box family member 4 [Source:NCBI gene;Acc:706950]	-1.006073801	0.009723047	0.031200525
ENSMMUG00000062974		-0.918453646	0.00040672	0.002003314
ENSMMUG00000040154	T cell receptor alpha variable 9-2 [Source:HGNC Symbol;Acc:HGNC:12154]	-1.52133575	1.63E-06	1.41E-05
ENSMMUG00000038070	T cell receptor alpha variable 12-3 [Source:HGNC Symbol;Acc:HGNC:12107]	-1.251416493	5.26E-09	7.46E-08
ENSMMUG00000054116	T cell receptor alpha variable 8-6 [Source:HGNC Symbol;Acc:HGNC:12151]	-1.237346776	9.37E-06	6.85E-05
ENSMMUG00000056910		-1.500973075	4.02E-08	4.82E-07
ENSMMUG00000063215	T cell receptor alpha variable 29/delta variable 5 (gene/pseudogene) [Source:HGNC Symbol;Acc:HGNC:12127]	1.081770124	0.000706639	0.003258531
ENSMMUG00000009441	abhydrolase domain containing 4 [Source:VGNC Symbol;Acc:VGNC:84296]	1.929994223	2.76E-05	0.00018035
ENSMMUG00000000413	solute carrier family 7 member 7 [Source:VGNC Symbol;Acc:VGNC:77631]	-1.273672328	3.96E-10	6.76E-09
ENSMMUG00000014748	solute carrier family 22 member 17 [Source:VGNC Symbol;Acc:VGNC:77560]	-1.661836153	0.006015275	0.020705006
ENSMMUG00000014750	embryonal Fyn-associated substrate [Source:VGNC Symbol;Acc:VGNC:72056]	-0.984366232	0.004370226	0.015819226
ENSMMUG00000014762	myosin heavy chain 6 [Source:NCBI gene;Acc:713471]	-0.859234922	2.76E-07	2.81E-06

ENSMMUG00000029420		1.507095626	2.34E-05	0.000155803
ENSMMUG00000014766	neuroguidin [Source:VGNC Symbol;Acc:VGNC:75220]	0.901369838	7.00E-07	6.56E-06
ENSMMUG00000014771	zinc finger homeobox 2 [Source:NCBI gene;Acc:100429448]	1.228649292	8.94E-15	3.14E-13
ENSMMUG00000059196		0.352678649	0.000132107	0.000734551
ENSMMUG00000012462	proteasome activator subunit 1 [Source:VGNC Symbol;Acc:VGNC:76455]	-2.195295228	4.84E-08	5.72E-07
ENSMMUG00000012473	proteasome activator subunit 2 [Source:VGNC Symbol;Acc:VGNC:76456]	-2.238761797	1.58E-05	0.000109006
ENSMMUG00000012474	ring finger protein 31 [Source:NCBI gene;Acc:715239]	-1.869794516	4.56E-05	0.000282796
ENSMMUG00000040817	interferon regulatory factor 9 [Source:VGNC Symbol;Acc:VGNC:84361]	-1.563682055	0.001061256	0.004647717
ENSMMUG00000012480	REC8 meiotic recombination protein [Source:VGNC Symbol;Acc:VGNC:76746]	-1.59911514	8.60E-06	6.35E-05
ENSMMUG00000012481	importin 4 [Source:VGNC Symbol;Acc:VGNC:73630]	-1.63501729	4.68E-07	4.55E-06
ENSMMUG00000012452	transglutaminase 1 [Source:NCBI gene;Acc:715854]	-2.600597073	0.000918133	0.004082111
ENSMMUG00000010049	NOP9 nucleolar protein [Source:VGNC Symbol;Acc:VGNC:75216]	-4.157192775	5.12E-06	3.99E-05
ENSMMUG00000003370	nuclear factor of activated T cells 4 [Source:VGNC Symbol;Acc:VGNC:75329]	-2.719481103	0.015830791	0.04707541
ENSMMUG00000019691	G2/M-phase specific E3 ubiquitin protein ligase [Source:VGNC Symbol;Acc:VGNC:72753]	-0.608264616	0.013557994	0.041367221
ENSMMUG00000003120	HECT domain E3 ubiquitin protein ligase 1 [Source:VGNC Symbol;Acc:VGNC:73200]	-1.211124754	1.23E-06	1.09E-05
ENSMMUG00000011779	HEAT repeat containing 5A [Source:VGNC Symbol;Acc:VGNC:84347]	-0.663318689	0.000206725	0.001103502
ENSMMUG00000014839	neuronal PAS domain protein 3 [Source:VGNC Symbol;Acc:VGNC:75213]	-0.413667877	0.014284582	0.043245399
ENSMMUG00000056906		0.762097772	6.16E-08	7.17E-07
ENSMMUG00000049364	serine palmitoyltransferase small subunit A [Source:NCBI gene;Acc:717580]	-0.582627798	0.001447039	0.00607176
ENSMMUG00000012576	sorting nexin 6 [Source:NCBI gene;Acc:693626]	-1.231291023	3.67E-05	0.000233487
ENSMMUG00000003530	protein phosphatase 2 regulatory subunit B''gamma [Source:VGNC Symbol;Acc:VGNC:76279]	-0.325269331	0.014241111	0.043123105
ENSMMUG00000056013	forkhead box A1 [Source:NCBI gene;Acc:697909]	2.164862365	0.001475151	0.006173094
ENSMMUG00000052781		-0.523306387	0.000603691	0.002857089
ENSMMUG00000017677	chromosome 7 C14orf28 homolog [Source:VGNC Symbol;Acc:VGNC:70581]	-0.651716093	4.24E-06	3.37E-05
ENSMMUG00000017678	kelch like family member 28 [Source:VGNC Symbol;Acc:VGNC:74085]	-1.094336594	0.000199214	0.001067066
ENSMMUG00000008284	dynein axonemal assembly factor 2 [Source:VGNC Symbol;Acc:VGNC:71868]	0.478635879	0.013350022	0.040857126
ENSMMUG00000003914	kelch domain containing 1 [Source:VGNC Symbol;Acc:VGNC:74086]	-0.839765518	0.004573252	0.016439558
ENSMMUG00000008214	nuclear export mediator factor [Source:VGNC Symbol;Acc:VGNC:84407]	-1.260794851	8.57E-05	0.000496691
ENSMMUG00000055903	Metazoan signal recognition particle RNA [Source:RFAM;Acc:RF00017]	-1.876292293	0.002117248	0.008482904
ENSMMUG00000049543		1.327222186	5.41E-05	0.000330344
ENSMMUG00000055012		1.791320279	0.001290661	0.005509443
ENSMMUG00000059134		1.709831082	3.83E-07	3.82E-06
ENSMMUG00000060227		-1.335869528	0.015636491	0.046593819

ENSMMUG00000007464	SOS Ras/Rho guanine nucleotide exchange factor 2 [Source:VGNC Symbol;Acc:VGNC:77838]	-0.946590994	0.001692774	0.006957141
ENSMMUG00000007468	L-2-hydroxyglutarate dehydrogenase [Source:VGNC Symbol;Acc:VGNC:74090]	-0.39593095	0.002624123	0.01018209
ENSMMUG00000009577	atlastin GTPase 1 [Source:VGNC Symbol;Acc:VGNC:70049]	0.464541503	0.00180443	0.007359952
ENSMMUG00000014658	ninein [Source:VGNC Symbol;Acc:VGNC:75177]	2.575921008	1.24E-11	2.73E-10
ENSMMUG00000018274	FERM domain containing 6 [Source:VGNC Symbol;Acc:VGNC:72747]	0.933965606	1.51E-12	3.81E-11
ENSMMUG00000000024	prostaglandin D2 receptor [Source:VGNC Symbol;Acc:VGNC:76467]	1.060676286	0.000238324	0.00125451
ENSMMUG00000000023	prostaglandin E receptor 2 [Source:VGNC Symbol;Acc:VGNC:76468]	0.748511368	0.000762953	0.003493519
ENSMMUG00000065036		-0.91229989	0.015750178	0.046882774
ENSMMUG00000006997	thioredoxin domain containing 16 [Source:VGNC Symbol;Acc:VGNC:79214]	0.8280213	8.18E-08	9.33E-07
ENSMMUG00000010689	fermitin family member 2 [Source:VGNC Symbol;Acc:VGNC:82144]	-0.999033452	1.21E-07	1.32E-06
ENSMMUG00000000302	WD repeat and HMG-box DNA binding protein 1 [Source:VGNC Symbol;Acc:VGNC:79376]	1.636282875	6.70E-26	8.10E-24
ENSMMUG00000054112		-0.445331726	0.001697257	0.006973522
ENSMMUG00000003565	galectin 3 [Source:VGNC Symbol;Acc:VGNC:74255]	-0.797376929	0.000903548	0.00403775
ENSMMUG00000003567	DLG associated protein 5 [Source:VGNC Symbol;Acc:VGNC:71850]	-0.616558049	0.002833316	0.010867501
ENSMMUG00000049334		-0.51743583	0.011555394	0.036161858
ENSMMUG00000023041	TATA-box binding protein like 2 [Source:VGNC Symbol;Acc:VGNC:78175]	-0.752422446	0.002033571	0.008185064
ENSMMUG00000061576		1.96730946	1.07E-07	1.18E-06
ENSMMUG00000053489		0.690595645	0.003778354	0.013910174
ENSMMUG00000018278	exocyst complex component 5 [Source:VGNC Symbol;Acc:VGNC:72260]	-1.308342713	0.008383242	0.027621757
ENSMMUG00000018279	adaptor related protein complex 5 subunit mu 1 [Source:VGNC Symbol;Acc:VGNC:69868]	-0.929565018	4.01E-10	6.85E-09
ENSMMUG00000002121	armadillo like helical domain containing 4 [Source:VGNC Symbol;Acc:VGNC:82106]	-1.157016398	2.60E-06	2.15E-05
ENSMMUG00000003508	actin related protein 10 [Source:VGNC Symbol;Acc:VGNC:69449]	0.313384674	0.006473073	0.022048071
ENSMMUG00000011966	reticulon 1 [Source:NCBI gene;Acc:619184]	1.054930054	0.014777982	0.044394096
ENSMMUG00000004854	leucine rich repeat containing 9 [Source:VGNC Symbol;Acc:VGNC:81443]	1.06805519	3.86E-07	3.83E-06
ENSMMUG00000002540	dehydrogenase/reductase 7 [Source:VGNC Symbol;Acc:VGNC:71690]	0.639063396	1.11E-08	1.47E-07
ENSMMUG00000001640	SIX homeobox 4 [Source:NCBI gene;Acc:703798]	-0.909162523	0.000543915	0.00259963
ENSMMUG00000001642	MNAT1 component of CDK activating kinase [Source:VGNC Symbol;Acc:VGNC:74905]	-0.683283039	0.006597176	0.022432638
ENSMMUG00000002260	protein kinase C eta [Source:VGNC Symbol;Acc:VGNC:84424]	1.373170327	3.39E-11	6.92E-10
ENSMMUG00000008203	ras homolog family member J [Source:VGNC Symbol;Acc:VGNC:76885]	-0.55976431	0.005135754	0.018095144
ENSMMUG00000054689		-0.786193362	0.005045856	0.017844595
ENSMMUG00000001008	spectrin repeat containing nuclear envelope protein 2 [Source:NCBI gene;Acc:706759]	-0.928587631	1.54E-06	1.34E-05
ENSMMUG00000001319	methylenetetrahydrofolate dehydrogenase, cyclohydrolase and formyltetrahydrofolate synthetase 1 [Source:VGNC Symbol;Acc:VGNC:84399]	-1.264760184	3.11E-16	1.31E-14
ENSMMUG00000004748	zinc finger and BTB domain containing 1 [Source:VGNC Symbol;Acc:VGNC:79238]	1.383957556	3.48E-14	1.12E-12
ENSMMUG00000056063		2.546949416	1.50E-05	0.000103921
ENSMMUG00000053575	Metazoan signal recognition particle RNA [Source:RFAM;Acc:RF00017]	1.366691565	0.004438661	0.016021453
ENSMMUG00000004339	RAB15, member RAS oncogene family [Source:VGNC Symbol;Acc:VGNC:81519]	0.680069299	2.06E-05	0.000139259
ENSMMUG00000013848	fucosyltransferase 8 [Source:VGNC Symbol;Acc:VGNC:72829]	-1.158367662	1.33E-05	9.35E-05
ENSMMUG00000013907	ribosomal protein S27a [Source:NCBI gene;Acc:718909]	-1.33684349	0.003014646	0.011453366

ENSMMUG00000006768	coiled-coil domain containing 196 [Source:VGNC Symbol;Acc:VGNC:70828]	-1.647732064	5.87E-05	0.000354914
ENSMMUG00000009520	gephyrin [Source:VGNC Symbol;Acc:VGNC:72948]	-1.543696724	7.69E-06	5.76E-05
ENSMMUG00000021419	eukaryotic translation initiation factor 2 subunit alpha [Source:VGNC Symbol;Acc:VGNC:72187]	-0.647647811	0.014635092	0.044078156
ENSMMUG00000021420	pleckstrin 2 [Source:VGNC Symbol;Acc:VGNC:76071]	-0.839177924	3.08E-06	2.51E-05
ENSMMUG00000015865	arginase 2 [Source:VGNC Symbol;Acc:VGNC:70005]	1.736210811	9.09E-10	1.46E-08
ENSMMUG00000015866	vesicle transport through interaction with t-SNAREs 1B [Source:NCBI gene;Acc:711113]	1.203780127	2.48E-09	3.70E-08
ENSMMUG00000015871	zinc finger FYVE-type containing 26 [Source:VGNC Symbol;Acc:VGNC:79283]	1.36643708	0.001952751	0.007891479
ENSMMUG00000050511		1.154693228	0.000363003	0.001806383
ENSMMUG00000015873	RAD51 paralog B [Source:VGNC Symbol;Acc:VGNC:76647]	1.282027707	0.000106678	0.000604668
ENSMMUG00000021572	solute carrier family 39 member 9 [Source:VGNC Symbol;Acc:VGNC:77523]	-1.194402219	6.29E-09	8.74E-08
ENSMMUG00000055018	sushi domain containing 6 [Source:NCBI gene;Acc:712719]	1.535425332	7.69E-17	3.42E-15
ENSMMUG00000022407	serine and arginine rich splicing factor 5 [Source:NCBI gene;Acc:712867]	1.197236422	4.28E-10	7.27E-09
ENSMMUG00000022409	solute carrier family 10 member 1 [Source:VGNC Symbol;Acc:VGNC:77380]	1.457730224	1.51E-14	5.14E-13
ENSMMUG00000053103	disintegrin and metalloproteinase domain-containing protein 20-like [Source:NCBI gene;Acc:713573]	-0.782268681	0.000607981	0.002873516
ENSMMUG00000019470	pecanex 1 [Source:VGNC Symbol;Acc:VGNC:75655]	-0.433672697	0.009388611	0.030349583
ENSMMUG00000001820	regulator of G protein signaling 6 [Source:VGNC Symbol;Acc:VGNC:76691]	3.322802265	0.00129508	0.005523391
ENSMMUG00000002332	RNA binding motif protein 25 [Source:VGNC Symbol;Acc:VGNC:76521]	-1.62284063	5.31E-07	5.10E-06
ENSMMUG00000010173	presenilin 1 [Source:VGNC Symbol;Acc:VGNC:76435]	1.372629852	2.49E-09	3.71E-08
ENSMMUG00000056580	Metazoan signal recognition particle RNA [Source:RFAM;Acc:RF00017]	2.394891216	0.011213456	0.035241214
ENSMMUG00000000313	HEAT repeat containing 4 [Source:VGNC Symbol;Acc:VGNC:73176]	1.303652777	6.40E-13	1.70E-11
ENSMMUG00000053481		1.712927584	1.20E-13	3.52E-12
ENSMMUG00000049894		2.0810702	3.34E-06	2.71E-05
ENSMMUG00000006855	acyl-CoA thioesterase 2 [Source:NCBI gene;Acc:697011]	1.574786906	5.23E-16	2.15E-14
ENSMMUG00000004910	dynein axonemal light chain 1 [Source:VGNC Symbol;Acc:VGNC:71885]	1.253346309	1.36E-05	9.56E-05
ENSMMUG00000004913	mitotic deacetylase associated SANT domain protein [Source:VGNC Symbol;Acc:VGNC:72073]	0.81878945	3.65E-06	2.94E-05
ENSMMUG00000056376		2.122393632	6.91E-13	1.83E-11
ENSMMUG00000015166	NIMA related kinase 9 [Source:VGNC Symbol;Acc:VGNC:75209]	-0.820739395	2.33E-12	5.72E-11
ENSMMUG00000015168	transmembrane p24 trafficking protein 10 [Source:NCBI gene;Acc:701522]	-1.062130066	4.80E-05	0.000296044
ENSMMUG00000040100	Fos proto-oncogene, AP-1 transcription factor subunit [Source:VGNC Symbol;Acc:VGNC:84340]	-0.627770545	6.18E-06	4.75E-05
ENSMMUG00000003834	basic leucine zipper ATF-like transcription factor [Source:VGNC Symbol;Acc:VGNC:70229]	2.035099119	1.38E-11	3.01E-10
ENSMMUG00000012065	tubulin tyrosine ligase like 5 [Source:NCBI gene;Acc:703116]	1.630503763	3.29E-15	1.22E-13
ENSMMUG00000008792	G-patch domain containing 2 like [Source:VGNC Symbol;Acc:VGNC:72973]	-0.781354243	0.005485249	0.019153298
ENSMMUG00000016773	estrogen related receptor beta [Source:VGNC Symbol;Acc:VGNC:72434]	0.66939667	0.000429859	0.002109139
ENSMMUG00000057021	CLOCK interacting pacemaker [Source:NCBI gene;Acc:704679]	-0.691849719	0.013358256	0.04086449
ENSMMUG00000007561	zinc finger DHHC-type palmitoyltransferase 22 [Source:NCBI gene;Acc:706468]	-0.883520481	0.000560787	0.002672074
ENSMMUG00000023626	alkB homolog 1, histone H2A dioxygenase [Source:VGNC Symbol;Acc:VGNC:69630]	-1.554099419	0.001736883	0.007107182
ENSMMUG00000019710	centrosomal protein 128 [Source:VGNC Symbol;Acc:VGNC:71062]	-0.701680522	0.016091192	0.047715541
ENSMMUG00000019713	thyroid stimulating hormone receptor [Source:VGNC Symbol;Acc:VGNC:78662]	-1.018774178	0.001037518	0.004553712

ENSMMUG00000017261	galactosylceramidase [Source:VGNC Symbol;Acc:VGNC:72867]	0.852885203	1.66E-07	1.76E-06
ENSMMUG00000004603	zinc finger CCCH-type containing 14 [Source:VGNC Symbol;Acc:VGNC:79255]	-0.915595439	5.31E-09	7.52E-08
ENSMMUG00000004606	EMAP like 5 [Source:VGNC Symbol;Acc:VGNC:72067]	-1.086895057	4.38E-11	8.75E-10
ENSMMUG00000010442	NRDE-2, necessary for RNA interference, domain containing [Source:VGNC Symbol;Acc:VGNC:75440]	-0.365917768	0.007563015	0.025269518
ENSMMUG00000008964	D-glutamate cyclase [Source:VGNC Symbol;Acc:VGNC:71685]	1.138643205	0.003376551	0.01260584
ENSMMUG00000054889	G protein-coupled receptor 68 [Source:NCBI gene;Acc:696529]	0.84006262	0.014904412	0.044716424
ENSMMUG00000018319	thyroid hormone receptor interactor 11 [Source:VGNC Symbol;Acc:VGNC:78647]	-0.666955108	3.47E-05	0.000221633
ENSMMUG00000022428	NADH:ubiquinone oxidoreductase subunit B1 [Source:NCBI gene;Acc:697745]	-0.498703345	0.013126533	0.040243412
ENSMMUG00000013966	legumain [Source:VGNC Symbol;Acc:VGNC:74274]	0.699059228	0.003086508	0.011666336
ENSMMUG00000009974	inositol-tetrakisphosphate 1-kinase [Source:VGNC Symbol;Acc:VGNC:73802]	1.36336468	0.002813397	0.010808841
ENSMMUG00000044581	modulator of apoptosis 1 [Source:VGNC Symbol;Acc:VGNC:84392]	1.347033351	8.96E-06	6.59E-05
ENSMMUG00000061833	transmembrane protein 251 [Source:NCBI gene;Acc:708131]	1.606148734	8.00E-09	1.08E-07
ENSMMUG00000053729		-0.573824467	0.003813319	0.014031533
ENSMMUG00000012400	ankyrin repeat and SOCS box containing 2 [Source:VGNC Symbol;Acc:VGNC:69840]	-1.001867897	0.00584728	0.020196237
ENSMMUG00000012356	serpin family A member 9 [Source:VGNC Symbol;Acc:VGNC:77241]	1.954236235	5.35E-08	6.26E-07
ENSMMUG00000020586	serpin family A member 12 [Source:VGNC Symbol;Acc:VGNC:77266]	3.218723953	0.002985367	0.011358225
ENSMMUG00000000681	calmin [Source:VGNC Symbol;Acc:VGNC:71232]	-0.892466355	2.12E-09	3.21E-08
ENSMMUG00000003658	autophagy related 2B [Source:VGNC Symbol;Acc:VGNC:84310]	0.747249579	0.014029013	0.042572788
ENSMMUG00000051277		-1.852066668	0.009883722	0.031650192
ENSMMUG00000003261	BAF chromatin remodeling complex subunit BCL11B [Source:VGNC Symbol;Acc:VGNC:70056]	-1.344124646	3.06E-10	5.34E-09
ENSMMUG00000051552		-1.015977038	0.000608363	0.002873838
ENSMMUG00000061892		-0.866403201	4.10E-06	3.28E-05
ENSMMUG00000049437	Metazoan signal recognition particle RNA [Source:RFAM;Acc:RF00017]	-1.085281157	4.48E-05	0.000278201
ENSMMUG00000029197	YY1 transcription factor [Source:NCBI gene;Acc:717370]	-0.979113811	1.09E-06	9.76E-06
ENSMMUG00000051100	Metazoan signal recognition particle RNA [Source:RFAM;Acc:RF00017]	-1.359264513	0.013878019	0.042203986
ENSMMUG00000013865	tryptophanyl-tRNA synthetase 1 [Source:VGNC Symbol;Acc:VGNC:78779]	-1.161032532	0.015576317	0.046424375
ENSMMUG00000013871	WD repeat domain 25 [Source:VGNC Symbol;Acc:VGNC:78420]	1.819739724	6.91E-12	1.59E-10
ENSMMUG00000000186	protein phosphatase 2 regulatory subunit B'gamma [Source:NCBI gene;Acc:718894]	0.29260927	0.000814389	0.003698998
ENSMMUG00000005780	dynein cytoplasmic 1 heavy chain 1 [Source:VGNC Symbol;Acc:VGNC:71951]	0.342528767	8.64E-05	0.000500249
ENSMMUG00000007140	MOK protein kinase [Source:VGNC Symbol;Acc:VGNC:84394]	2.098772223	1.64E-07	1.75E-06
ENSMMUG00000003483	cyclin dependent kinase 2 interacting protein [Source:VGNC Symbol;Acc:VGNC:71326]	-0.677927683	0.000174501	0.000945892
ENSMMUG00000003484	tectonin beta-propeller repeat containing 2 [Source:NCBI gene;Acc:708959]	1.566154102	5.59E-10	9.29E-09
ENSMMUG00000057818		1.800463997	0.0145751	0.043944663
ENSMMUG00000005845	REST corepressor 1 [Source:VGNC Symbol;Acc:VGNC:76752]	0.58359076	0.00468878	0.016794577
ENSMMUG00000011751		-1.198521841	0.004325108	0.015692363
ENSMMUG00000029186	exocyst complex component 3 like 4 [Source:VGNC Symbol;Acc:VGNC:72305]	4.04266091	1.07E-05	7.70E-05
ENSMMUG00000050495		2.874216724	4.00E-08	4.80E-07

ENSMMUG00000021634	TNF alpha induced protein 2 [Source:VGNC Symbol;Acc:VGNC:78579]	2.090216266	3.53E-29	5.89E-27
ENSMMUG00000003641	microtubule affinity regulating kinase 3 [Source:VGNC Symbol;Acc:VGNC:74659]	0.5171844	0.005118637	0.01804844
ENSMMUG00000029181	X-ray repair cross complementing 3 [Source:NCBI gene;Acc:695788]	0.329800129	0.004388357	0.015868472
ENSMMUG00000018121	zinc finger FYVE-type containing 21 [Source:VGNC Symbol;Acc:VGNC:79282]	0.323041817	0.001719516	0.007046397
ENSMMUG00000052518	SIVA1 apoptosis inducing factor [Source:NCBI gene;Acc:706688]	-1.561961114	0.003961144	0.014503164
ENSMMUG00000001047	zinc finger and BTB domain containing 42 [Source:VGNC Symbol;Acc:VGNC:79925]	1.725990908	0.002270782	0.008974964
ENSMMUG00000064694	AHNAK nucleoprotein 2 [Source:NCBI gene;Acc:707626]	3.968491585	7.63E-06	5.72E-05
ENSMMUG00000001273	clathrin binding box of aftiphilin containing 1 [Source:VGNC Symbol;Acc:VGNC:84325]	2.577567035	2.24E-23	1.94E-21
ENSMMUG00000054161	Metazoan signal recognition particle RNA [Source:RFAM;Acc:RF00017]	2.116394319	0.002083463	0.008352318
ENSMMUG00000065019	Metazoan signal recognition particle RNA [Source:RFAM;Acc:RF00017]	-1.635916371	0.000370682	0.001840025
ENSMMUG00000015202		-1.537980523	0.016297547	0.048134057
ENSMMUG00000040771		-2.108657162	5.09E-07	4.91E-06
ENSMMUG00000059525		-1.906748668	8.99E-08	1.02E-06
ENSMMUG00000055207		-5.861240959	1.74E-11	3.74E-10
ENSMMUG00000060892		-3.133005114	1.04E-24	1.08E-22
ENSMMUG00000064429	immunoglobulin heavy variable 1-45 [Source:HGNC Symbol;Acc:HGNC:5553]	-3.721425151	0.002179844	0.008689014
ENSMMUG00000052929		-3.801810627	5.71E-05	0.000345901
ENSMMUG00000052535		-3.509745449	2.21E-05	0.000148395
ENSMMUG00000004955	short stature homeobox [Source:NCBI gene;Acc:114674875]	-1.090897431	0.005451295	0.01904897
ENSMMUG00000063346		3.130441535	0.000173926	0.000943507
ENSMMUG00000038067		1.3058952	0.000313308	0.001592433
ENSMMUG00000053333	acetylserotonin O-methyltransferase like [Source:NCBI gene;Acc:106995142]	3.291204291	0.004121605	0.015029746
ENSMMUG00000004985	arylsulfatase L [Source:VGNC Symbol;Acc:VGNC:81361]	-1.54814015	0.000811604	0.003691573
ENSMMUG00000000443	arylsulfatase F [Source:VGNC Symbol;Acc:VGNC:81362]	-0.668239973	0.003113327	0.011739225
ENSMMUG00000017378	steroid sulfatase [Source:VGNC Symbol;Acc:VGNC:81590]	-1.664668772	0.011244272	0.035313277
ENSMMUG00000010252	FERM and PDZ domain containing 4 [Source:VGNC Symbol;Acc:VGNC:72802]	-1.145025728	2.22E-05	0.000148425
ENSMMUG00000003482	glycine receptor alpha 2 [Source:VGNC Symbol;Acc:VGNC:73075]	0.970103534	0.000236394	0.001245757
ENSMMUG00000015737	phosphatidylinositol glycan anchor biosynthesis class A [Source:VGNC Symbol;Acc:VGNC:75984]	-0.974216401	7.76E-07	7.20E-06
ENSMMUG00000004797	CTP synthase 2 [Source:VGNC Symbol;Acc:VGNC:71550]	2.220762928	5.77E-13	1.55E-11
ENSMMUG00000058700	Metazoan signal recognition particle RNA [Source:RFAM;Acc:RF00017]	4.055121696	1.26E-05	8.93E-05
ENSMMUG00000019363	synapse associated protein 1 [Source:VGNC Symbol;Acc:VGNC:78135]	2.413445735	8.46E-16	3.36E-14
ENSMMUG00000016237	taxilin gamma [Source:NCBI gene;Acc:713769]	2.34918888	0.005920317	0.020398183
ENSMMUG00000012894	NHS actin remodeling regulator [Source:VGNC Symbol;Acc:VGNC:75348]	-1.00099735	0.004697468	0.016817102
ENSMMUG00000004744	SH3 domain containing kinase binding protein 1 [Source:VGNC Symbol;Acc:VGNC:77227]	-0.310492087	0.016551001	0.048758908
ENSMMUG00000001088	patched domain containing 1 [Source:VGNC Symbol;Acc:VGNC:76322]	1.189180022	7.56E-10	1.23E-08
ENSMMUG00000061261	Metazoan signal recognition particle RNA [Source:RFAM;Acc:RF00017]	-1.607869843	8.14E-05	0.000475068
ENSMMUG00000042317	putative transcription factor SPT20 homolog-like 2 [Source:NCBI gene;Acc:100429227]	-0.764629556	0.015077195	0.045128604

ENSMMUG00000012819	pyruvate dehydrogenase kinase 3 [Source:VGNC Symbol;Acc:VGNC:75934]	-0.932977112	0.006381593	0.021789404
ENSMMUG00000012775	nuclear receptor subfamily 0 group B member 1 [Source:NCBI gene;Acc:574154]	-1.497991889	0.007962547	0.026415612
ENSMMUG00000013220	cytochrome b-245 beta chain [Source:VGNC Symbol;Acc:VGNC:71576]	-0.747941634	0.017023171	0.049992865
ENSMMUG00000013493	ornithine carbamoyltransferase [Source:VGNC Symbol;Acc:VGNC:75723]	3.389416543	0.010178495	0.032468843
ENSMMUG00000005264	mediator complex subunit 14 [Source:VGNC Symbol;Acc:VGNC:74561]	0.559942718	0.000160974	0.000879711
ENSMMUG00000063721		-0.639456842	4.41E-06	3.50E-05
ENSMMUG00000057909		1.370528041	0.000534258	0.00255609
ENSMMUG00000057804		-1.893538888	0.001404179	0.005921994
ENSMMUG00000003505	FUN14 domain containing 1 [Source:NCBI gene;Acc:709370]	2.435052738	0.001175956	0.005078667
ENSMMUG00000003897	solute carrier family 9 member A7 [Source:NCBI gene;Acc:710906]	-0.675684802	2.71E-06	2.23E-05
ENSMMUG00000049666		0.621546391	0.000108997	0.000617064
ENSMMUG00000023018	RP2 activator of ARL3 GTPase [Source:VGNC Symbol;Acc:VGNC:76720]	0.553498026	0.016540366	0.048748801
ENSMMUG00000023016	jade family PHD finger 3 [Source:VGNC Symbol;Acc:VGNC:73640]	1.391915539	1.33E-19	7.84E-18
ENSMMUG00000019546	regucalcin [Source:VGNC Symbol;Acc:VGNC:76912]	1.130295304	0.012392552	0.038269271
ENSMMUG00000014002	cyclin dependent kinase 16 [Source:VGNC Symbol;Acc:VGNC:71160]	-1.411495232	0.000553162	0.002641127
ENSMMUG00000016608	ubiquitin specific peptidase 11 [Source:VGNC Symbol;Acc:VGNC:78734]	-1.880539525	0.001851057	0.007532609
ENSMMUG00000008566	zinc finger protein 157 [Source:VGNC Symbol;Acc:VGNC:78850]	1.005412569	6.99E-12	1.60E-10
ENSMMUG00000010404	zinc finger protein 41 [Source:VGNC Symbol;Acc:VGNC:78900]	-0.514232352	0.012816047	0.039403515
ENSMMUG00000063703		-0.860313413	0.003496895	0.012989923
ENSMMUG00000019768	suppressor of variegation 3-9 homolog 1 [Source:VGNC Symbol;Acc:VGNC:78061]	-0.978724197	0.000846965	0.003821424
ENSMMUG00000016465	histone deacetylase 6 [Source:VGNC Symbol;Acc:VGNC:73164]	0.770938835	1.13E-11	2.51E-10
ENSMMUG00000004558	OTU deubiquitinase 5 [Source:VGNC Symbol;Acc:VGNC:75677]	1.120949018	1.10E-09	1.74E-08
ENSMMUG00000004559	potassium voltage-gated channel subfamily D member 1 [Source:VGNC Symbol;Acc:VGNC:73975]	-1.39936358	0.006899875	0.02330931
ENSMMUG00000023025	synaptophysin [Source:VGNC Symbol;Acc:VGNC:78072]	0.672183309	2.45E-06	2.04E-05
ENSMMUG00000008618	calcium voltage-gated channel subunit alpha1 F [Source:VGNC Symbol;Acc:VGNC:70503]	1.047628175	4.97E-14	1.57E-12
ENSMMUG00000008623	coiled-coil domain containing 22 [Source:VGNC Symbol;Acc:VGNC:70899]	1.108317206	3.33E-14	1.08E-12
ENSMMUG00000052252	protein phosphatase 1 regulatory subunit 3F [Source:NCBI gene;Acc:715950]	1.66220133	2.39E-20	1.56E-18
ENSMMUG00000060311	G antigen 10-like [Source:NCBI gene;Acc:114674954]	0.574024029	0.000185069	0.000997993
ENSMMUG00000041376	G antigen 10 [Source:NCBI gene;Acc:693330]	1.471193904	2.03E-14	6.77E-13
ENSMMUG00000026584	Y RNA [Source:RFAM;Acc:RF00019]	1.740221976	0.013624565	0.041541659
ENSMMUG00000023060	A-kinase anchoring protein 4 [Source:VGNC Symbol;Acc:VGNC:69770]	2.233026506	3.36E-10	5.78E-09
ENSMMUG00000045093	cyclin B3 [Source:NCBI gene;Acc:694534]	2.127824913	7.69E-24	7.09E-22
ENSMMUG00000049267		4.269726381	1.93E-20	1.27E-18
ENSMMUG00000020202	melanoma-associated antigen D4 [Source:NCBI gene;Acc:106996411]	-0.707688765	0.012027048	0.037348646
ENSMMUG00000046218	X antigen family member 5 [Source:NCBI gene;Acc:114669729]	-1.042925986	0.002081334	0.008346167
ENSMMUG00000002593	TSPY like 2 [Source:VGNC Symbol;Acc:VGNC:79433]	-0.330915008	0.003766142	0.013876147
ENSMMUG00000053262		-0.76917227	7.72E-05	0.000452849
ENSMMUG00000020553	lysine demethylase 5C [Source:VGNC Symbol;Acc:VGNC:73816]	-0.546691935	0.001963513	0.007930397

ENSMMUG00000020555	IQ motif and Sec7 domain ArfGEF 2 [Source:VGNC Symbol;Acc:VGNC:73676]	-0.610432651	9.83E-06	7.14E-05
ENSMMUG00000047741	Metazoan signal recognition particle RNA [Source:RFAM;Acc:RF00017]	-1.067754225	8.57E-06	6.33E-05
ENSMMUG00000008422	trophinin [Source:NCBI gene;Acc:704512]	-0.779469959	4.01E-06	3.21E-05
ENSMMUG00000059922	family with sequence similarity 104 member B [Source:NCBI gene;Acc:705418]	-2.812155374	1.26E-05	8.92E-05
ENSMMUG00000042087	ubiquilin 2 [Source:NCBI gene;Acc:707967]	0.479024767	0.010213971	0.032544994
ENSMMUG00000051670		-1.147760987	2.46E-05	0.000162657
ENSMMUG00000054377		1.004860611	7.95E-14	2.40E-12
ENSMMUG00000063913	endogenous retrovirus group K member 7 Env polyprotein-like [Source:NCBI gene;Acc:114674933]	3.213383545	8.09E-05	0.000472387
ENSMMUG00000059335		5.10274641	2.16E-07	2.24E-06
ENSMMUG00000004309	APC membrane recruitment protein 1 [Source:VGNC Symbol;Acc:VGNC:69716]	4.981150741	4.88E-07	4.74E-06
ENSMMUG00000004274	myotubularin related protein 8 [Source:VGNC Symbol;Acc:VGNC:74974]	4.053482651	0.000366015	0.001818789
ENSMMUG00000065001		-0.45746248	0.002465403	0.009659545
ENSMMUG00000047336	praja ring finger ubiquitin ligase 1 [Source:NCBI gene;Acc:695038]	-0.492256653	0.00225502	0.008932808
ENSMMUG00000054817	immunoglobulin binding protein 1 [Source:NCBI gene;Acc:694632]	-0.863447547	0.006615844	0.022490661
ENSMMUG00000061295	PDZ domain containing 11 [Source:NCBI gene;Acc:695458]	-0.80060935	0.001036621	0.004551201
ENSMMUG00000013940	kinesin family member 4A [Source:NCBI gene;Acc:695574]	-1.992480607	3.02E-06	2.47E-05
ENSMMUG00000013943	glycerophosphodiester phosphodiesterase domain containing 2 [Source:VGNC Symbol;Acc:VGNC:72787]	-2.027098339	1.36E-06	1.19E-05
ENSMMUG00000003049	testis expressed 11 [Source:VGNC Symbol;Acc:VGNC:78316]	-2.742547773	7.91E-09	1.07E-07
ENSMMUG00000032429	forkhead box O4 [Source:VGNC Symbol;Acc:VGNC:72712]	2.079161262	1.88E-25	2.13E-23
ENSMMUG00000032427	neuroligin 3 [Source:NCBI gene;Acc:574216]	-1.241340669	3.09E-17	1.45E-15
ENSMMUG00000000669	gap junction protein beta 1 [Source:VGNC Symbol;Acc:VGNC:73059]	-1.651705463	0.000822003	0.003727992
ENSMMUG00000000667	zinc finger MYM-type containing 3 [Source:VGNC Symbol;Acc:VGNC:78886]	-1.359823204	3.16E-05	0.000204032
ENSMMUG00000056450		-1.940860182	0.003740581	0.013807361
ENSMMUG00000015238	O-linked N-acetylglucosamine (GlcNAc) transferase [Source:NCBI gene;Acc:698268]	-1.134172705	4.99E-07	4.81E-06
ENSMMUG00000003296	Cbp/p300 interacting transactivator with Glu/Asp rich carboxy-terminal domain 1 [Source:VGNC Symbol;Acc:VGNC:71227]	-0.429949667	0.006653293	0.022585118
ENSMMUG00000013449	caudal type homeobox 4 [Source:VGNC Symbol;Acc:VGNC:70997]	-1.394081485	0.013515178	0.041281494
ENSMMUG00000063858		-0.766509685	1.82E-06	1.55E-05
ENSMMUG00000037903	Metazoan signal recognition particle RNA [Source:RFAM;Acc:RF00017]	-0.589926318	0.001490159	0.00622105
ENSMMUG00000053505	U6 spliceosomal RNA [Source:RFAM;Acc:RF00026]	1.268521725	0.004295745	0.015601977
ENSMMUG00000017660		1.320594891	4.41E-11	8.80E-10
ENSMMUG00000011440	neurite extension and migration factor [Source:VGNC Symbol;Acc:VGNC:75274]	0.671235949	0.004133939	0.015068943
ENSMMUG00000015619	ATP binding cassette subfamily B member 7 [Source:VGNC Symbol;Acc:VGNC:81340]	-1.238976217	1.09E-06	9.76E-06
ENSMMUG00000049351	MAGE family member E1 [Source:NCBI gene;Acc:705379]	-1.220924916	2.67E-14	8.76E-13
ENSMMUG00000014407	magnesium transporter 1 [Source:VGNC Symbol;Acc:VGNC:74386]	0.672840671	2.63E-05	0.000172295

ENSMMUG00000015861	cytochrome c oxidase subunit VIIb [Source:NCBI gene;Acc:706099]	0.690009971	0.000855978	0.003852176
ENSMMUG00000013387	cysteinyl leukotriene receptor 1 [Source:VGNC Symbol;Acc:VGNC:71646]	1.38178785	3.35E-10	5.78E-09
ENSMMUG00000052073		1.402815673	7.01E-18	3.49E-16
ENSMMUG00000000448		-1.412756497	0.007230821	0.024321852
ENSMMUG00000011078	protocadherin 19 [Source:VGNC Symbol;Acc:VGNC:75601]	1.489242012	0.006506883	0.022147092
ENSMMUG00000008212	tenomodulin [Source:VGNC Symbol;Acc:VGNC:79087]	-1.138596942	2.45E-07	2.52E-06
ENSMMUG00000013008	cleavage stimulation factor subunit 2 [Source:VGNC Symbol;Acc:VGNC:71536]	-1.289189261	6.19E-05	0.000372469
ENSMMUG00000004178	NADPH oxidase 1 [Source:VGNC Symbol;Acc:VGNC:75374]	-1.220016464	3.03E-08	3.72E-07
ENSMMUG00000065018	transmembrane protein 35A [Source:NCBI gene;Acc:702205]	-0.806252025	4.86E-05	0.000298871
ENSMMUG00000059432		2.175059023	0.000352393	0.00175983
ENSMMUG00000013150	MORC family CW-type zinc finger 4 [Source:VGNC Symbol;Acc:VGNC:74808]	1.297701527	1.54E-10	2.85E-09
ENSMMUG00000013146	claudin 2 [Source:HGNC Symbol;Acc:HGNC:2041]	1.3331495	3.80E-11	7.65E-10
ENSMMUG00000022293	FERM and PDZ domain containing 3 [Source:VGNC Symbol;Acc:VGNC:72805]	-0.926333429	1.97E-13	5.56E-12
ENSMMUG00000004674	phosphoribosyl pyrophosphate synthetase 1 [Source:NCBI gene;Acc:702529]	-0.792158993	5.23E-08	6.12E-07
ENSMMUG00000007688	angiomotin [Source:VGNC Symbol;Acc:VGNC:69627]	3.83074445	0.000386234	0.001911137
ENSMMUG00000055906	angiotensin II receptor type 2 [Source:NCBI gene;Acc:709513]	1.786946679	5.94E-05	0.000358585
ENSMMUG00000026243	U6 spliceosomal RNA [Source:RFAM;Acc:RF00026]	2.123701382	0.009744868	0.031256241
ENSMMUG00000008818	SWI/SNF related, matrix associated, actin dependent regulator of chromatin, subfamily a, member 1 [Source:VGNC Symbol;Acc:VGNC:77646]	-1.187610728	0.001969468	0.00795216
ENSMMUG00000008781	BCL6 corepressor like 1 [Source:VGNC Symbol;Acc:VGNC:70131]	-0.916444396	6.91E-08	7.97E-07
ENSMMUG00000008785	E74 like ETS transcription factor 4 [Source:VGNC Symbol;Acc:VGNC:72208]	-0.569217148	0.002233052	0.008860807
ENSMMUG00000004954	apoptosis inducing factor mitochondria associated 1 [Source:VGNC Symbol;Acc:VGNC:69820]	-0.569935626	0.003137794	0.011815597
ENSMMUG00000003479	FERM domain containing 7 [Source:VGNC Symbol;Acc:VGNC:72927]	-1.374348578	0.0003047	0.001554881
ENSMMUG00000048745	RAP2C, member of RAS oncogene family [Source:NCBI gene;Acc:705986]	-0.881841853	0.000324621	0.001642195
ENSMMUG00000025616		-3.687691891	0.000118767	0.000667256
ENSMMUG00000003456	glypican 4 [Source:VGNC Symbol;Acc:VGNC:73118]	-0.60879174	1.27E-07	1.38E-06
ENSMMUG00000009622	glypican 3 [Source:VGNC Symbol;Acc:VGNC:73117]	1.517150838	2.01E-06	1.69E-05
ENSMMUG00000032341	coiled-coil domain containing 160 [Source:VGNC Symbol;Acc:VGNC:70926]	0.666564248	7.07E-09	9.74E-08
ENSMMUG00000016610	CD99 molecule like 2 [Source:VGNC Symbol;Acc:VGNC:70823]	-1.09477524	3.04E-05	0.00019656
ENSMMUG00000016929	proline rich and Gla domain 3 [Source:VGNC Symbol;Acc:VGNC:76350]	2.866971063	3.60E-11	7.29E-10
ENSMMUG00000040974	fetal and adult testis expressed 1 [Source:NCBI gene;Acc:704368]	2.998941049	0.001202171	0.005179121
ENSMMUG00000063694	cyclic nucleotide gated channel subunit alpha 2 [Source:NCBI gene;Acc:704486]	1.063866647	2.16E-11	4.57E-10
ENSMMUG00000062628		1.114209053	2.51E-06	2.08E-05
ENSMMUG00000040274	MAGE family member A4 [Source:NCBI gene;Acc:710825]	1.049658633	1.70E-10	3.12E-09
ENSMMUG00000039292		1.570819151	2.08E-10	3.73E-09
ENSMMUG00000062063	melanoma-associated antigen 3 [Source:NCBI gene;Acc:711544]	1.199823185	7.79E-09	1.06E-07
ENSMMUG00000037641	chondrosarcoma-associated gene 2/3 protein [Source:NCBI gene;Acc:106992305]	2.182714001	0.002546324	0.009924539
ENSMMUG00000049656	melanoma-associated antigen 2 [Source:NCBI gene;Acc:706218]	1.584305022	0.01177025	0.036686866
ENSMMUG00000058013	melanoma-associated antigen 3 [Source:NCBI gene;Acc:706103]	1.829920686	7.73E-14	2.35E-12
ENSMMUG00000040071		2.120284537	1.49E-05	0.000103549
ENSMMUG00000020743	centrin 2 [Source:NCBI gene;Acc:693376]	1.599700714	0.004481226	0.016145998

ENSMMUG00000013176	NAD(P) dependent steroid dehydrogenase-like [Source:NCBI gene;Acc:693333]	1.916086156	5.52E-06	4.28E-05
ENSMMUG00000064249		-1.127475667	0.004467703	0.016101413
ENSMMUG00000055393		1.447659108	0.01491368	0.044734659
ENSMMUG00000061278		-0.745838483	0.006102013	0.020952136
ENSMMUG00000010137	microcephalin 1 [Source:VGNC Symbol;Acc:VGNC:74687]	1.68303935	3.97E-05	0.000250207
ENSMMUG00000058176	b-defensin2-like [Source:NCBI gene;Acc:100169969]	2.041992965	6.28E-15	2.24E-13
ENSMMUG00000049842		-4.111663206	5.44E-06	4.22E-05
ENSMMUG00000029034	defensin beta 136 [Source:VGNC Symbol;Acc:VGNC:71826]	1.427116423	3.97E-07	3.92E-06
ENSMMUG00000050140	Metazoan signal recognition particle RNA [Source:RFAM;Acc:RF00017]	2.126991658	0.004069701	0.01485409
ENSMMUG00000063525		2.535115444	4.20E-15	1.53E-13
ENSMMUG00000054960		1.597267896	2.98E-07	3.01E-06
ENSMMUG00000027220	microRNA 598 [Source:HGNC Symbol;Acc:HGNC:32854]	2.866025006	0.005121756	0.018054897
ENSMMUG00000010180	PIN2 (TERF1) interacting telomerase inhibitor 1 [Source:NCBI gene;Acc:701523]	1.673469807	6.89E-08	7.95E-07
ENSMMUG00000008867	exoribonuclease 1 [Source:VGNC Symbol;Acc:VGNC:72324]	-2.747450647	8.33E-07	7.68E-06
ENSMMUG00000022303	solute carrier family 7 member 2 [Source:VGNC Symbol;Acc:VGNC:77629]	-0.7968745	2.58E-09	3.83E-08
ENSMMUG00000001151	microtubule associated scaffold protein 1 [Source:VGNC Symbol;Acc:VGNC:84018]	1.953790404	5.08E-16	2.10E-14
ENSMMUG00000005920	N-acetyltransferase 1 [Source:VGNC Symbol;Acc:VGNC:75133]	3.485585252	1.92E-07	2.01E-06
ENSMMUG00000011774	integrator complex subunit 10 [Source:VGNC Symbol;Acc:VGNC:73653]	1.036464767	9.29E-14	2.77E-12
ENSMMUG00000064162		0.725759776	0.000622949	0.002926504
ENSMMUG00000002032	exportin 7 [Source:VGNC Symbol;Acc:VGNC:78946]	-1.335650739	6.56E-07	6.18E-06
ENSMMUG00000063764		-1.726651052	0.001974806	0.007969124
ENSMMUG00000013029	surfactant protein C [Source:VGNC Symbol;Acc:VGNC:84113]	-1.306842631	3.61E-05	0.000229716
ENSMMUG00000013031	bone morphogenetic protein 1 [Source:VGNC Symbol;Acc:VGNC:70259]	-0.749725621	0.000249204	0.001305413
ENSMMUG00000013032	phytanoyl-CoA 2-hydroxylase interacting protein [Source:VGNC Symbol;Acc:VGNC:75812]	-0.899976052	0.000240729	0.001265746
ENSMMUG00000023226	RNA polymerase III subunit D [Source:VGNC Symbol;Acc:VGNC:76017]	-0.950486339	1.81E-09	2.77E-08
ENSMMUG00000052651	Metazoan signal recognition particle RNA [Source:RFAM;Acc:RF00017]	-1.131602312	2.01E-06	1.69E-05
ENSMMUG00000014438	piwi like RNA-mediated gene silencing 2 [Source:VGNC Symbol;Acc:VGNC:81502]	-0.713816615	3.31E-07	3.33E-06
ENSMMUG00000018345	phosphatidylethanolamine binding protein 4 [Source:VGNC Symbol;Acc:VGNC:82204]	1.780140716	2.09E-17	9.93E-16
ENSMMUG00000050868		1.433849985	0.002974586	0.011322645
ENSMMUG00000007540	Rho related BTB domain containing 2 [Source:VGNC Symbol;Acc:VGNC:76772]	0.777379198	0.001124739	0.004881526
ENSMMUG00000048076	TNF receptor superfamily member 10b [Source:NCBI gene;Acc:716650]	0.68465832	0.001117623	0.004861176
ENSMMUG00000050549	lysyl oxidase like 2 [Source:NCBI gene;Acc:710151]	1.565827229	6.84E-10	1.12E-08
ENSMMUG00000046186	stanniocalcin 1 [Source:VGNC Symbol;Acc:VGNC:84120]	2.026201073	0.009191106	0.029821119
ENSMMUG00000003348	neurofilament light [Source:VGNC Symbol;Acc:VGNC:75307]	2.073799822	1.79E-35	5.22E-33
ENSMMUG00000056605	Metazoan signal recognition particle RNA [Source:RFAM;Acc:RF00017]	2.230333617	1.16E-05	8.28E-05
ENSMMUG00000041905	protein phosphatase 2 regulatory subunit Balpha [Source:VGNC Symbol;Acc:VGNC:84103]	-1.336650998	0.008763697	0.02866651
ENSMMUG00000059181		1.034190847	0.004957677	0.017601029
ENSMMUG00000016018	establishment of sister chromatid cohesion N-acetyltransferase 2 [Source:VGNC Symbol;Acc:VGNC:72374]	-3.652419854	7.96E-06	5.94E-05
ENSMMUG00000016019	PDZ binding kinase [Source:VGNC Symbol;Acc:VGNC:75643]	-2.389663997	0.001291024	0.005509443

ENSMMUG00000022044	scavenger receptor class A member 5 [Source:VGNC Symbol;Acc:VGNC:77117]	-0.710421697	8.30E-05	0.00048243
ENSMMUG00000005067	elongator acetyltransferase complex subunit 3 [Source:VGNC Symbol;Acc:VGNC:72080]	-1.738962009	9.13E-08	1.03E-06
ENSMMUG00000014981	homeobox containing 1 [Source:VGNC Symbol;Acc:VGNC:73443]	0.712904474	3.65E-05	0.000231822
ENSMMUG00000020821	testis expressed 15, meiosis and synapsis associated [Source:VGNC Symbol;Acc:VGNC:78318]	-1.600773273	3.66E-20	2.27E-18
ENSMMUG00000061408		-3.931403197	2.39E-07	2.46E-06
ENSMMUG00000063345		-4.220459247	6.01E-09	8.39E-08
ENSMMUG00000057375		3.056040829	0.002736567	0.010556968
ENSMMUG00000034978	U6 spliceosomal RNA [Source:RFAM;Acc:RF00026]	1.955013542	0.006911767	0.02333824
ENSMMUG00000001635	zinc finger protein 703 [Source:VGNC Symbol;Acc:VGNC:79552]	0.94119476	5.74E-06	4.44E-05
ENSMMUG00000061014		2.925881722	0.009044962	0.029415006
ENSMMUG00000013675	ASH2 like, histone lysine methyltransferase complex subunit [Source:VGNC Symbol;Acc:VGNC:70032]	0.303709411	0.00930042	0.030106137
ENSMMUG00000051162		-3.938904977	7.76E-05	0.00045501
ENSMMUG00000003591	pleckstrin homology domain containing A2 [Source:VGNC Symbol;Acc:VGNC:76026]	1.174791638	0.000271418	0.001407061
ENSMMUG00000019304	indoleamine 2,3-dioxygenase 1 [Source:VGNC Symbol;Acc:VGNC:73580]	2.15637861	1.88E-10	3.42E-09
ENSMMUG00000019306	indoleamine 2,3-dioxygenase 2 [Source:VGNC Symbol;Acc:VGNC:84005]	2.271782868	0.001511	0.006300549
ENSMMUG00000000776	zinc finger matrin-type 4 [Source:VGNC Symbol;Acc:VGNC:78424]	1.167984171	2.73E-08	3.39E-07
ENSMMUG00000058320	U6 spliceosomal RNA [Source:RFAM;Acc:RF00026]	-0.471809923	0.008650478	0.028388873
ENSMMUG00000019082	secreted frizzled related protein 1 [Source:VGNC Symbol;Acc:VGNC:77185]	-0.441083605	0.004015269	0.014682169
ENSMMUG00000014721	glycerol-3-phosphate acyltransferase 4 [Source:VGNC Symbol;Acc:VGNC:84002]	-0.87378501	0.00145585	0.006099608
ENSMMUG00000057151	Y RNA [Source:RFAM;Acc:RF00019]	-0.757051143	0.007403929	0.02479114
ENSMMUG00000014431	adaptor related protein complex 3 subunit mu 2 [Source:VGNC Symbol;Acc:VGNC:69971]	0.302236761	0.013704031	0.041740146
ENSMMUG00000014432	plasminogen activator, tissue type [Source:VGNC Symbol;Acc:VGNC:76161]	0.462398712	4.29E-05	0.00026815
ENSMMUG00000020185	voltage dependent anion channel 3 [Source:VGNC Symbol;Acc:VGNC:84135]	1.879057813	2.64E-06	2.18E-05
ENSMMUG00000020186	solute carrier family 20 member 2 [Source:VGNC Symbol;Acc:VGNC:77416]	1.162495656	0.002218734	0.008818964
ENSMMUG00000053488	small integral membrane protein 19 [Source:NCBI gene;Acc:705371]	0.619581326	4.59E-09	6.59E-08
ENSMMUG00000015347	protein kinase, DNA-activated, catalytic subunit [Source:VGNC Symbol;Acc:VGNC:76418]	-0.936776636	6.90E-06	5.22E-05
ENSMMUG00000002781	peroxidasin like [Source:VGNC Symbol;Acc:VGNC:84107]	-0.709960298	8.96E-07	8.18E-06
ENSMMUG00000065247	opioid receptor kappa 1 [Source:NCBI gene;Acc:710783]	1.694202396	2.43E-19	1.41E-17
ENSMMUG00000025039	Small nucleolar RNA SNORA73 family [Source:RFAM;Acc:RF00045]	2.969527152	0.009598883	0.0308595
ENSMMUG00000005478	trimethylguanosine synthase 1 [Source:VGNC Symbol;Acc:VGNC:78404]	0.981463735	3.51E-05	0.00022364
ENSMMUG00000014149	LYN proto-oncogene, Src family tyrosine kinase [Source:VGNC Symbol;Acc:VGNC:74464]	0.757878718	4.20E-11	8.43E-10
ENSMMUG00000055121	Metazoan signal recognition particle RNA [Source:RFAM;Acc:RF00017]	0.787399982	0.00580069	0.020059946
ENSMMUG00000061268	PLAG1 zinc finger [Source:NCBI gene;Acc:696149]	-1.110471627	0.001793489	0.007317458
ENSMMUG00000046464	coiled-coil-helix-coiled-coil-helix domain containing 7 [Source:VGNC Symbol;Acc:VGNC:83989]	-1.208479231	9.15E-07	8.33E-06
ENSMMUG00000061068		-0.678237981	0.010805327	0.034154776
ENSMMUG00000014810	short chain dehydrogenase/reductase family 16C member 5 [Source:VGNC Symbol;Acc:VGNC:77343]	-0.774847006	0.000329626	0.001663913
ENSMMUG00000052791		-0.893975974	0.000246567	0.001294014

ENSMMUG00000006775	proenkephalin [Source:VGNC Symbol;Acc:VGNC:75941]	-0.962631318	8.52E-06	6.30E-05
ENSMMUG00000006543	cytochrome P450, family 7, subfamily A, polypeptide 1 [Source:NCBI gene;Acc:698127]	-3.577685478	0.006322203	0.021612932
ENSMMUG00000017038	RAB2A, member RAS oncogene family [Source:VGNC Symbol;Acc:VGNC:81529]	0.534288828	0.000404015	0.001992087
ENSMMUG00000059575		-2.278959347	4.78E-40	2.23E-37
ENSMMUG00000038661		-2.354319158	1.82E-17	8.82E-16
ENSMMUG00000049866		0.451679122	0.00991741	0.031744315
ENSMMUG00000004411	25-hydroxycholesterol 7-alpha-hydroxylase [Source:NCBI gene;Acc:710188]	-0.830786265	5.12E-06	3.99E-05
ENSMMUG00000014424	ADP ribosylation factor guanine nucleotide exchange factor 1 [Source:VGNC Symbol;Acc:VGNC:69834]	-2.387888757	0.00070124	0.003238964
ENSMMUG00000012513	lactamase beta 2 [Source:VGNC Symbol;Acc:VGNC:74072]	-1.095622676	0.005306351	0.018612045
ENSMMUG00000045333	Metazoan signal recognition particle RNA [Source:RFAM;Acc:RF00017]	-0.657993425	0.000178497	0.000965687
ENSMMUG00000007576	EYA transcriptional coactivator and phosphatase 1 [Source:VGNC Symbol;Acc:VGNC:72453]	-0.601366252	3.21E-07	3.23E-06
ENSMMUG00000019414	zinc finger homeobox 4 [Source:VGNC Symbol;Acc:VGNC:79902]	-0.723700251	0.013927257	0.042328116
ENSMMUG00000046641		-0.390066284	0.005633065	0.019562275
ENSMMUG00000000507	stathmin 2 [Source:VGNC Symbol;Acc:VGNC:78038]	-0.84651362	0.015246305	0.045547281
ENSMMUG00000007115	RALY RNA binding protein like [Source:VGNC Symbol;Acc:VGNC:82217]	-1.387441284	0.000380818	0.001886334
ENSMMUG00000063555		-1.95480032	6.95E-05	0.000412022
ENSMMUG00000028701		-0.923110392	0.001642263	0.006770566
ENSMMUG00000061720		-0.739661249	0.014667845	0.044157835
ENSMMUG00000020010	cadherin 17 [Source:VGNC Symbol;Acc:VGNC:70810]	3.540340706	0.008269465	0.027311047
ENSMMUG00000021112	epithelial splicing regulatory protein 1 [Source:VGNC Symbol;Acc:VGNC:72358]	-0.472187686	0.00800323	0.026531748
ENSMMUG00000057219	tumor protein p53 inducible nuclear protein 1 [Source:NCBI gene;Acc:700632]	1.936058312	0.004344099	0.015749043
ENSMMUG00000062721	TSPY like 5 [Source:NCBI gene;Acc:702081]	2.360390702	0.008055326	0.026685528
ENSMMUG00000055454		0.324529846	0.014128974	0.042848313
ENSMMUG00000060329		0.555533318	0.000253677	0.001327357
ENSMMUG00000061917		0.693814954	7.20E-06	5.43E-05
ENSMMUG00000014210	metadherin [Source:VGNC Symbol;Acc:VGNC:74859]	0.70637674	4.29E-08	5.12E-07
ENSMMUG00000008926	lysosomal protein transmembrane 4 beta [Source:VGNC Symbol;Acc:VGNC:84010]	0.445647312	0.001339801	0.005679572
ENSMMUG00000060771		0.578563801	0.000695927	0.00321654
ENSMMUG00000020072	matrilin 2 [Source:VGNC Symbol;Acc:VGNC:74665]	0.492794228	0.002672533	0.010332204
ENSMMUG00000054671	Metazoan signal recognition particle RNA [Source:RFAM;Acc:RF00017]	1.023501236	0.011286409	0.035422854
ENSMMUG00000058389		0.634930056	0.00126884	0.005424677
ENSMMUG00000022185	odd-skipped related transciption factor 2 [Source:VGNC Symbol;Acc:VGNC:84097]	-1.371310775	4.15E-15	1.51E-13
ENSMMUG00000022186	vacuolar protein sorting 13 homolog B [Source:VGNC Symbol;Acc:VGNC:79339]	-0.463679844	0.002152969	0.00859655
ENSMMUG00000004386	sperm associated antigen 1 [Source:VGNC Symbol;Acc:VGNC:77849]	-1.395886326	0.002985554	0.011358225
ENSMMUG00000061687		-1.083814258	0.003371795	0.012597256

ENSMMUG00000004387	ring finger protein 19A, RBR E3 ubiquitin protein ligase [Source:VGNC Symbol;Acc:VGNC:82224]	-1.362938199	1.49E-06	1.29E-05
ENSMMUG00000051088		-1.132676517	0.001425331	0.005998151
ENSMMUG00000009958	ankyrin repeat domain 46 [Source:VGNC Symbol;Acc:VGNC:69719]	-1.26075431	0.000615289	0.002898208
ENSMMUG00000034608	U6 spliceosomal RNA [Source:RFAM;Acc:RF00026]	0.897541251	0.000486296	0.002354739
ENSMMUG00000051999		-2.113714784	1.48E-07	1.58E-06
ENSMMUG00000001338	antizyme inhibitor 1 [Source:VGNC Symbol;Acc:VGNC:70080]	2.784397902	0.000207972	0.001109315
ENSMMUG00000054357		-3.141840379	0.00082367	0.003733136
ENSMMUG00000056520		-2.356760351	0.014713911	0.044268006
ENSMMUG00000010744		-1.460796978	0.011228663	0.035281098
ENSMMUG00000054660	R-spondin 2 [Source:NCBI gene;Acc:697376]	-1.015688073	4.12E-06	3.29E-05
ENSMMUG00000011359	eukaryotic translation initiation factor 3 subunit E [Source:VGNC Symbol;Acc:VGNC:83997]	-1.053295582	4.32E-05	0.000270006
ENSMMUG00000062333		-0.351041391	0.001101839	0.004799967
ENSMMUG00000003435	transcriptional repressor GATA binding 1 [Source:VGNC Symbol;Acc:VGNC:78656]	-0.607533036	1.25E-06	1.10E-05
ENSMMUG00000001087		-0.628521758	0.012024846	0.037348646
ENSMMUG00000024448		-1.61636002	0.013627015	0.041541659
ENSMMUG00000004903	ectonucleotide pyrophosphatase/phosphodiesterase 2 [Source:VGNC Symbol;Acc:VGNC:72223]	1.182952254	9.70E-10	1.55E-08
ENSMMUG00000014702	TATA-box binding protein associated factor 2 [Source:VGNC Symbol;Acc:VGNC:78090]	0.933637079	4.35E-07	4.26E-06
ENSMMUG00000058797		0.666614872	0.000215892	0.001147196
ENSMMUG00000021157	TBC1 domain family member 31 [Source:VGNC Symbol;Acc:VGNC:84121]	4.295139078	1.98E-13	5.58E-12
ENSMMUG00000020467	ATPase family AAA domain containing 2 [Source:VGNC Symbol;Acc:VGNC:70154]	-0.54869817	4.20E-05	0.000263077
ENSMMUG00000028923	WDYHV motif containing 1 [Source:VGNC Symbol;Acc:VGNC:79503]	0.267289668	0.008350782	0.027527742
ENSMMUG00000023778	F-box protein 32 [Source:VGNC Symbol;Acc:VGNC:72517]	-1.148218086	0.000326204	0.001648016
ENSMMUG00000016375	fer-1 like family member 6 [Source:VGNC Symbol;Acc:VGNC:72585]	-0.357930752	0.007076058	0.023852778
ENSMMUG00000054755	transmembrane protein 65 [Source:NCBI gene;Acc:705727]	1.772566213	1.42E-06	1.24E-05
ENSMMUG00000058106	otoconin 90 [Source:NCBI gene;Acc:100429699]	0.726158622	4.79E-06	3.76E-05
ENSMMUG00000012560	HERV-H LTR-associating 1 [Source:NCBI gene;Acc:100430297]	1.193771393	4.80E-11	9.51E-10
ENSMMUG00000055934		0.583852409	0.003485447	0.012954259
ENSMMUG00000059464		-2.95280864	0.004314554	0.015662176
ENSMMUG00000048066	trafficking protein particle complex 9 [Source:NCBI gene;Acc:711466]	1.281566241	1.55E-10	2.86E-09
ENSMMUG00000053837		3.96284617	0.004580158	0.016455947
ENSMMUG00000001432	t-SNARE domain containing 1 [Source:NCBI gene;Acc:701269]	-0.504408183	0.000435613	0.00213438
ENSMMUG00000019877	adhesion G protein-coupled receptor B1 [Source:VGNC Symbol;Acc:VGNC:69597]	0.712229879	0.009344372	0.030220508
ENSMMUG00000062642		-0.66182404	0.002816476	0.010817705
ENSMMUG00000022452	Jrk helix-turn-helix protein [Source:VGNC Symbol;Acc:VGNC:73957]	0.48471078	6.01E-05	0.00036246
ENSMMUG00000000959	cytochrome P450, family 11, subfamily B, polypeptide 2 [Source:NCBI gene;Acc:695914]	0.724121032	4.37E-07	4.27E-06
ENSMMUG00000064147	lymphocyte antigen 6 family member L [Source:NCBI gene;Acc:106999990]	0.352527412	0.00399425	0.014612931
ENSMMUG00000022933	DNA topoisomerase I mitochondrial [Source:VGNC Symbol;Acc:VGNC:79821]	0.970614155	3.90E-12	9.26E-11
ENSMMUG00000059932	Metazoan signal recognition particle RNA [Source:RFAM;Acc:RF00017]	1.573061118	2.65E-05	0.000173492
ENSMMUG00000010939	rhophilin Rho GTPase binding protein 1 [Source:VGNC Symbol;Acc:VGNC:76779]	-0.381730353	0.000245163	0.001288578

ENSMMUG00000058733	zinc finger CCCH-type containing 3 [Source:NCBI gene;Acc:708584]	0.469679424	5.03E-06	3.93E-05
ENSMMUG00000001187	mitogen-activated protein kinase 15 [Source:VGNC Symbol;Acc:VGNC:74570]	1.491033961	0.002070398	0.008309441
ENSMMUG00000013552	scribble planar cell polarity protein [Source:HGNC Symbol;Acc:HGNC:30377]	-1.252625482	4.99E-18	2.54E-16
ENSMMUG00000000388	plectin [Source:VGNC Symbol;Acc:VGNC:76173]	-1.081269707	3.02E-08	3.71E-07
ENSMMUG00000050907		-0.868505839	1.01E-06	9.10E-06
ENSMMUG00000053106		1.341628967	0.000128212	0.000716587
ENSMMUG00000064914		0.990345855	0.016549643	0.048758908
ENSMMUG00000023282	aldo-keto reductase family 1, member C1 (dihydrodiol dehydrogenase 1; 20-alpha (3-alpha)-hydroxysteroid dehydrogenase) [Source:NCBI gene;Acc:711198]	1.588621391	2.18E-11	4.60E-10
ENSMMUG00000000958	aldo-keto reductase family 1, member C2 [Source:NCBI gene;Acc:711360]	1.772292807	1.79E-13	5.08E-12
ENSMMUG00000015141	ankyrin repeat domain 16 [Source:NCBI gene;Acc:720787]	-1.79208206	1.09E-05	7.83E-05
ENSMMUG00000015143	F-box DNA helicase 1 [Source:NCBI gene;Acc:720797]	-1.698535677	1.87E-06	1.58E-05
ENSMMUG00000056045		-1.67688357	6.10E-10	1.01E-08
ENSMMUG00000015151	interleukin 2 receptor subunit alpha [Source:NCBI gene;Acc:574300]	-1.296066625	7.65E-05	0.000449085
ENSMMUG00000054646		-2.28536603	0.01569093	0.046727815
ENSMMUG00000061189		0.452007547	1.12E-06	1.00E-05
ENSMMUG00000052242		0.610003674	0.000213379	0.001134703
ENSMMUG00000064980		-1.418403699	0.001362204	0.005762348
ENSMMUG00000037449	CUGBP Elav-like family member 2 [Source:NCBI gene;Acc:714291]	0.483565701	0.009647024	0.030977837
ENSMMUG00000017145	cell division cycle 123 [Source:VGNC Symbol;Acc:VGNC:70768]	-1.123932809	8.74E-10	1.41E-08
ENSMMUG00000017164	FERM domain containing 4A [Source:VGNC Symbol;Acc:VGNC:72786]	-0.607603877	0.005725136	0.019828002
ENSMMUG00000017170	cerebral dopamine neurotrophic factor [Source:VGNC Symbol;Acc:VGNC:71094]	-0.464872428	0.00015089	0.000830754
ENSMMUG00000017173	heat shock protein family A (Hsp70) member 14 [Source:NCBI gene;Acc:697257]	-1.417278473	0.000385121	0.0019063
ENSMMUG00000017174	suppressor of variegation 3-9 homolog 2 [Source:NCBI gene;Acc:697378]	-2.672635309	0.010162608	0.032427163
ENSMMUG00000011661	integrin subunit alpha 8 [Source:VGNC Symbol;Acc:VGNC:73782]	1.376662388	1.02E-15	4.01E-14
ENSMMUG00000024234	U6 spliceosomal RNA [Source:RFAM;Acc:RF00026]	1.520428002	2.90E-07	2.94E-06
ENSMMUG00000016069	cubilin [Source:VGNC Symbol;Acc:VGNC:82132]	1.603071544	1.96E-05	0.000133356
ENSMMUG00000010589	nebulette [Source:VGNC Symbol;Acc:VGNC:75195]	-0.621390795	2.30E-05	0.000152968
ENSMMUG00000053765		0.593880583	0.001478701	0.006182416
ENSMMUG00000056500		0.506699427	0.001437965	0.0060373
ENSMMUG00000002930	G protein-coupled receptor 158 [Source:NCBI gene;Acc:707065]	0.655572673	0.000845661	0.003817378
ENSMMUG00000037243	U6 spliceosomal RNA [Source:RFAM;Acc:RF00026]	1.396314648	4.71E-07	4.58E-06
ENSMMUG00000016410	acyl-CoA binding domain containing 5 [Source:VGNC Symbol;Acc:VGNC:69509]	-0.441124776	0.016343888	0.048240441
ENSMMUG00000046971	RAB18, member RAS oncogene family [Source:NCBI gene;Acc:708843]	-0.87283961	0.013048373	0.040047567
ENSMMUG00000059681		1.352128441	0.001017508	0.004478503
ENSMMUG00000001374	supervillin [Source:NCBI gene;Acc:695267]	-1.074248857	3.88E-05	0.000244553
ENSMMUG00000041067	Metazoan signal recognition particle RNA [Source:RFAM;Acc:RF00017]	-1.205761758	0.00018487	0.000997472
ENSMMUG00000040958	lysozyme-like protein 1 [Source:NCBI gene;Acc:698176]	-0.963021109	1.75E-06	1.50E-05
ENSMMUG00000044214		-1.08726101	2.20E-07	2.27E-06

ENSMMUG00000015058	kinesin family member 5B [Source:VGNC Symbol;Acc:VGNC:74020]	-0.70375123	0.014484698	0.043719097
ENSMMUG00000043648		1.813328352	0.001307701	0.005565369
ENSMMUG00000061710		0.456447689	8.62E-06	6.36E-05
ENSMMUG00000057199		- 1.489197855	4.50E-05	0.000279159
ENSMMUG00000006713	cyclin Y [Source:VGNC Symbol;Acc:VGNC:70840]	- 1.203160245	1.41E-09	2.18E-08
ENSMMUG00000054392	zinc finger protein 239 [Source:NCBI gene;Acc:717737]	- 0.899425156	0.004340937	0.015741648
ENSMMUG00000064634		- 0.656017762	4.44E-06	3.52E-05
ENSMMUG00000064724		-1.55032748	0.006625593	0.022518268
ENSMMUG00000013836	WDFY family member 4 [Source:NCBI gene;Acc:719472]	2.339491848	0.006135393	0.021061595
ENSMMUG00000033001	U6 spliceosomal RNA [Source:RFAM;Acc:RF00026]	- 1.257773017	2.44E-08	3.06E-07
ENSMMUG00000017380	neuregulin 3 [Source:NCBI gene;Acc:699268]	2.268157789	0.000964218	0.004268067
ENSMMUG00000055918		2.962997136	5.18E-05	0.000317737
ENSMMUG00000057196	pulmonary surfactant-associated protein A [Source:NCBI gene;Acc:701715]	1.574957942	5.58E-19	3.14E-17
ENSMMUG00000051972	zinc finger CCHC-type containing 24 [Source:NCBI gene;Acc:702083]	2.323661069	9.60E-05	0.00054987
ENSMMUG00000055601		0.794895375	3.63E-11	7.33E-10
ENSMMUG00000053900		1.198018434	0.008753829	0.028652171
ENSMMUG00000005514	RNA polymerase III subunit A [Source:VGNC Symbol;Acc:VGNC:76138]	0.843743055	4.19E-05	0.00026281
ENSMMUG00000009128	discs large MAGUK scaffold protein 5 [Source:VGNC Symbol;Acc:VGNC:71921]	0.722478477	2.20E-09	3.32E-08
ENSMMUG00000050271		- 1.165868977	2.33E-05	0.00015475
ENSMMUG00000053916		0.801041173	8.19E-06	6.08E-05
ENSMMUG00000060115		- 0.621529782	0.00172352	0.007060744
ENSMMUG00000020823	vinculin [Source:NCBI gene;Acc:721921]	- 0.834330858	1.29E-05	9.10E-05
ENSMMUG00000021393	calcium/calmodulin dependent protein kinase II gamma [Source:VGNC Symbol;Acc:VGNC:70525]	- 1.581208763	1.89E-10	3.43E-09
ENSMMUG00000061526	synaptopodin 2 like [Source:NCBI gene;Acc:714736]	1.110323356	3.86E-07	3.83E-06
ENSMMUG00000013696	myozenin 1 [Source:VGNC Symbol;Acc:VGNC:74951]	1.026182047	2.25E-05	0.000150043
ENSMMUG00000001821	ubiquitin specific peptidase 54 [Source:NCBI gene;Acc:706859]	- 0.549242959	0.014686676	0.044203877
ENSMMUG00000011282	DnaJ heat shock protein family (Hsp40) member C9 [Source:NCBI gene;Acc:707599]	- 3.488505867	0.013550086	0.041360085
ENSMMUG00000011276	nudix hydrolase 13 [Source:VGNC Symbol;Acc:VGNC:75435]	- 0.414410523	0.000432418	0.002120952
ENSMMUG00000011274	prolyl 4-hydroxylase subunit alpha 1 [Source:VGNC Symbol;Acc:VGNC:75741]	-0.77795695	1.29E-06	1.13E-05
ENSMMUG00000016652	cell division cycle and apoptosis regulator 1 [Source:NCBI gene;Acc:693585]	0.776288803	2.22E-05	0.000148799
ENSMMUG00000023198	tet methylcytosine dioxygenase 1 [Source:VGNC Symbol;Acc:VGNC:79811]	0.32940432	0.006977832	0.023544307
ENSMMUG00000030532	solute carrier family 25 member 16 [Source:NCBI gene;Acc:694145]	0.739090381	1.82E-06	1.54E-05
ENSMMUG00000019703	RUN and FYVE domain containing 2 [Source:VGNC Symbol;Acc:VGNC:77057]	0.600633362	2.74E-08	3.41E-07
ENSMMUG00000009792	heterogeneous nuclear ribonucleoprotein H3 [Source:VGNC Symbol;Acc:VGNC:73497]	0.627613211	0.001540917	0.006400558
ENSMMUG00000061385		2.092769924	0.00810139	0.026819121
ENSMMUG00000016902	catenin alpha 3 [Source:VGNC Symbol;Acc:VGNC:82131]	0.569291398	0.004819625	0.01720173
ENSMMUG00000053199		1.056984595	9.31E-11	1.78E-09
ENSMMUG00000059754		1.091451317	1.09E-05	7.83E-05
ENSMMUG00000009794	protocadherin related 15 [Source:VGNC Symbol;Acc:VGNC:75608]	1.031096575	1.74E-11	3.73E-10
ENSMMUG00000007077	APOBEC1 complementation factor [Source:VGNC Symbol;Acc:VGNC:69523]	- 0.820571884	1.33E-07	1.44E-06
ENSMMUG00000007078	N-acylsphingosine amidohydrolase 2 [Source:NCBI gene;Acc:703913]	-0.71758555	2.42E-09	3.63E-08

ENSMMUG00000006411	3'-phosphoadenosine 5'-phosphosulfate synthase 2 [Source:VGNC Symbol;Acc:VGNC:75758]	-0.657864534	0.000705063	0.003254478
ENSMMUG00000000470	ankyrin repeat domain 22 [Source:VGNC Symbol;Acc:VGNC:69728]	-1.206028492	0.009313194	0.03014053
ENSMMUG00000061761	interferon induced protein with tetratricopeptide repeats 2 [Source:NCBI gene;Acc:695452]	-1.7012908	1.79E-18	9.75E-17
ENSMMUG00000058942		-1.917513602	5.71E-17	2.61E-15
ENSMMUG00000059038	5-hydroxytryptamine receptor 7 [Source:NCBI gene;Acc:696317]	-1.278739585	6.85E-06	5.18E-05
ENSMMUG00000000238	HECT domain E3 ubiquitin protein ligase 2 [Source:VGNC Symbol;Acc:VGNC:73246]	-2.643431309	5.33E-19	3.01E-17
ENSMMUG00000058603		-3.345069261	7.48E-09	1.02E-07
ENSMMUG00000007478	tankyrase 2 [Source:VGNC Symbol;Acc:VGNC:78983]	-0.591602103	1.46E-05	0.000101335
ENSMMUG00000059608	fibroblast growth factor binding protein 3 [Source:NCBI gene;Acc:697881]	0.880811826	6.03E-05	0.000363244
ENSMMUG00000017627	B-TFIID TATA-box binding protein associated factor 1 [Source:VGNC Symbol;Acc:VGNC:70336]	1.501067038	8.39E-30	1.47E-27
ENSMMUG00000004816	membrane associated ring-CH-type finger 5 [Source:VGNC Symbol;Acc:VGNC:74588]	1.159886868	9.52E-10	1.52E-08
ENSMMUG00000058935	Metazoan signal recognition particle RNA [Source:RFAM;Acc:RF00017]	1.727172388	0.000775303	0.003544854
ENSMMUG00000011789	hematopoietically expressed homeobox [Source:VGNC Symbol;Acc:VGNC:73376]	1.90092291	2.63E-07	2.69E-06
ENSMMUG00000052637	Metazoan signal recognition particle RNA [Source:RFAM;Acc:RF00017]	2.361758837	1.50E-05	0.000104404
ENSMMUG00000014715	fragile site, folic acid type, rare, fra(10)(q23.3) or fra(10)(q24.2) candidate 1 [Source:NCBI gene;Acc:700389]	1.891937994	1.30E-06	1.14E-05
ENSMMUG00000050975		-1.508140696	6.24E-07	5.90E-06
ENSMMUG00000004818	sorbin and SH3 domain containing 1 [Source:VGNC Symbol;Acc:VGNC:77702]	-0.323935253	0.004208297	0.015312131
ENSMMUG00000003103	tectonic family member 3 [Source:VGNC Symbol;Acc:VGNC:79438]	-0.556411269	0.00222772	0.008845527
ENSMMUG00000001518	zinc finger protein 518A [Source:VGNC Symbol;Acc:VGNC:79767]	-0.881938378	9.14E-15	3.20E-13
ENSMMUG00000008687	B cell linker [Source:VGNC Symbol;Acc:VGNC:70306]	-0.506355263	0.000104189	0.000592714
ENSMMUG00000001876	phosphoinositide-3-kinase adaptor protein 1 [Source:VGNC Symbol;Acc:VGNC:75891]	-0.694555842	0.000416252	0.002046669
ENSMMUG00000010427	ligand dependent nuclear receptor corepressor [Source:NCBI gene;Acc:705646]	-0.427784799	0.002849474	0.010914552
ENSMMUG00000059655		-1.123034139	0.016473575	0.048592541
ENSMMUG00000015503	zinc finger FYVE-type containing 27 [Source:VGNC Symbol;Acc:VGNC:79623]	0.594048049	1.74E-06	1.49E-05
ENSMMUG00000018154	cartilage acidic protein 1 [Source:VGNC Symbol;Acc:VGNC:71408]	0.516916317	0.001781707	0.007271501
ENSMMUG00000052648		0.73823792	0.003122857	0.011771994
ENSMMUG00000020603	pyridine nucleotide-disulphide oxidoreductase domain 2 [Source:NCBI gene;Acc:707797]	0.669702366	4.35E-05	0.000271331
ENSMMUG00000054904	Metazoan signal recognition particle RNA [Source:RFAM;Acc:RF00017]	0.858062735	2.53E-05	0.000166815
ENSMMUG00000020604	HPS1 biogenesis of lysosomal organelles complex 3 subunit 1 [Source:VGNC Symbol;Acc:VGNC:73515]	1.186004774	3.74E-11	7.54E-10
ENSMMUG00000049770	Metazoan signal recognition particle RNA [Source:RFAM;Acc:RF00017]	1.340024285	0.000106321	0.000602885
ENSMMUG00000020605	heparanase 2 (inactive) [Source:NCBI gene;Acc:708795]	0.718307485	0.000334469	0.001685931
ENSMMUG00000060230	Metazoan signal recognition particle RNA [Source:RFAM;Acc:RF00017]	1.147700983	3.12E-06	2.54E-05
ENSMMUG00000010357	cyclin and CBS domain divalent metal cation transport mediator 1 [Source:NCBI gene;Acc:708335]	0.877162571	4.95E-07	4.78E-06
ENSMMUG00000023761	ectonucleoside triphosphate diphosphohydrolase 7 [Source:VGNC Symbol;Acc:VGNC:72071]	0.632613968	0.002067503	0.0083002
ENSMMUG00000063121		1.321618486	0.009613358	0.030890955
ENSMMUG00000052430		-1.13830928	0.00311158	0.011735794
ENSMMUG00000015117	X-prolyl aminopeptidase 1 [Source:VGNC Symbol;Acc:VGNC:78810]	-1.435857534	8.69E-11	1.67E-09
ENSMMUG00000006233	adducin 3 [Source:VGNC Symbol;Acc:VGNC:69531]	1.076103825	0.003776142	0.013909334

ENSMMUG00000051414	Metazoan signal recognition particle RNA [Source:RFAM;Acc:RF00017]	1.209980656	0.005971484	0.020562919
ENSMMUG00000014350	MAX interactor 1, dimerization protein [Source:VGNC Symbol;Acc:VGNC:75087]	-1.133018996	4.95E-09	7.04E-08
ENSMMUG00000007775	survival motor neuron domain containing 1 [Source:NCBI gene;Acc:694761]	-0.915820112	0.000814555	0.003698998
ENSMMUG00000057793	dual specificity phosphatase 5 [Source:NCBI gene;Acc:698178]	-3.867924939	3.12E-05	0.000201717
ENSMMUG00000007982	structural maintenance of chromosomes 3 [Source:VGNC Symbol;Acc:VGNC:77769]	-1.745604371	0.00012247	0.000687233
ENSMMUG00000019883	RNA binding motif protein 20 [Source:NCBI gene;Acc:698677]	-1.675100246	0.00028923	0.001485666
ENSMMUG00000017204	programmed cell death 4 [Source:VGNC Symbol;Acc:VGNC:75794]	-4.034603702	7.08E-08	8.14E-07
ENSMMUG00000056056		-4.813750694	1.17E-05	8.40E-05
ENSMMUG00000017206	SHOC2 leucine rich repeat scaffold protein [Source:VGNC Symbol;Acc:VGNC:81569]	-3.547713829	2.72E-06	2.24E-05
ENSMMUG00000053789		-0.679156348	0.003453871	0.012847119
ENSMMUG00000055239		0.827722222	4.89E-09	6.97E-08
ENSMMUG00000022514	coiled-coil domain containing 186 [Source:VGNC Symbol;Acc:VGNC:70847]	0.806522862	1.71E-10	3.13E-09
ENSMMUG00000022524	actin binding LIM protein 1 [Source:VGNC Symbol;Acc:VGNC:69587]	-0.399213386	0.007313758	0.024563825
ENSMMUG00000058240		-1.099046005	0.000105913	0.000601062
ENSMMUG00000015414	family with sequence similarity 160 member B1 [Source:VGNC Symbol;Acc:VGNC:72403]	-0.675068561	4.82E-05	0.000296993
ENSMMUG00000001902	TruB pseudouridine synthase family member 1 [Source:VGNC Symbol;Acc:VGNC:79005]	0.745962152	0.00925503	0.029985047
ENSMMUG00000020080	GDNF family receptor alpha 1 [Source:VGNC Symbol;Acc:VGNC:72917]	2.120800736	0.005683384	0.019697998
ENSMMUG00000053348	PDZ domain containing 8 [Source:NCBI gene;Acc:709084]	-0.368987666	0.016518895	0.048715976
ENSMMUG00000021610	regulator of G protein signaling 10 [Source:VGNC Symbol;Acc:VGNC:76915]	2.13702036	1.82E-27	2.53E-25
ENSMMUG00000054942		1.512797962	1.83E-07	1.93E-06
ENSMMUG00000001472	TIA1 cytotoxic granule associated RNA binding protein like 1 [Source:VGNC Symbol;Acc:VGNC:78354]	1.667570859	3.46E-18	1.84E-16
ENSMMUG00000009559	minichromosome maintenance complex binding protein [Source:VGNC Symbol;Acc:VGNC:74583]	1.828219256	1.08E-08	1.43E-07
ENSMMUG00000009561	SEC23 interacting protein [Source:VGNC Symbol;Acc:VGNC:77255]	-0.971224548	0.003075133	0.011632747
ENSMMUG00000008724	phospholipid phosphatase 4 [Source:VGNC Symbol;Acc:VGNC:76097]	0.488442376	0.003749788	0.013830426
ENSMMUG00000064321		1.443023542	2.32E-07	2.40E-06
ENSMMUG00000064557		1.802624055	4.57E-32	1.05E-29
ENSMMUG00000052711		-0.824664433	1.05E-05	7.60E-05
ENSMMUG00000061101	Metazoan signal recognition particle RNA [Source:RFAM;Acc:RF00017]	-0.932126978	0.003895093	0.014294919
ENSMMUG00000020346	carboxypeptidase X, M14 family member 2 [Source:VGNC Symbol;Acc:VGNC:71596]	0.808880993	8.61E-06	6.36E-05
ENSMMUG00000058933		-1.70523343	0.000400686	0.00197846
ENSMMUG00000008539	uroporphyrinogen III synthase [Source:VGNC Symbol;Acc:VGNC:78731]	-0.978638075	8.88E-08	1.00E-06
ENSMMUG00000008541	BRCA2 and CDKN1A interacting protein [Source:NCBI gene;Acc:699437]	-0.528125284	0.008801755	0.028764161
ENSMMUG00000008542	DEAH-box helicase 32 (putative) [Source:NCBI gene;Acc:695919]	-0.51279368	0.011063905	0.034849311
ENSMMUG00000035166	U2 spliceosomal RNA [Source:RFAM;Acc:RF00004]	-1.951536004	0.000160321	0.000876826
ENSMMUG00000044121	marker of proliferation Ki-67 [Source:NCBI gene;Acc:697883]	-0.603478685	0.012014522	0.037340347

ENSMMUG00000013653	lysine demethylase 5A [Source:VGNC Symbol;Acc:VGNC:82169]	0.4686305	0.000103942	0.00059155
ENSMMUG00000007411	integrin alpha FG-GAP repeat containing 2 [Source:VGNC Symbol;Acc:VGNC:73690]	-1.351592715	1.15E-11	2.56E-10
ENSMMUG00000007424	nuclear receptor interacting protein 2 [Source:VGNC Symbol;Acc:VGNC:75448]	-1.3476957	1.31E-11	2.87E-10
ENSMMUG00000037675	testis expressed 52 [Source:NCBI gene;Acc:107000723]	-1.304211829	5.07E-17	2.35E-15
ENSMMUG00000061935		3.564096401	1.24E-36	4.22E-34
ENSMMUG00000015189	RAD51 associated protein 1 [Source:VGNC Symbol;Acc:VGNC:76497]	-0.726812866	0.008661993	0.028413363
ENSMMUG00000020811	dual specificity tyrosine phosphorylation regulated kinase 4 [Source:NCBI gene;Acc:710999]	-0.650864546	0.002498854	0.009755409
ENSMMUG00000020812	A-kinase anchoring protein 3 [Source:VGNC Symbol;Acc:VGNC:69769]	-1.71026284	6.64E-06	5.04E-05
ENSMMUG00000020813	NADH:ubiquinone oxidoreductase subunit A9 [Source:VGNC Symbol;Acc:VGNC:81480]	-0.936056247	0.000156878	0.000860009
ENSMMUG00000022893	von Willebrand factor [Source:VGNC Symbol;Acc:VGNC:78778]	3.651060262	2.08E-07	2.17E-06
ENSMMUG00000047465	Metazoan signal recognition particle RNA [Source:RFAM;Acc:RF00017]	5.704724179	5.17E-09	7.34E-08
ENSMMUG00000061974		4.404511776	0.00061608	0.002900065
ENSMMUG00000004470	CD9 molecule [Source:VGNC Symbol;Acc:VGNC:70787]	2.284554791	0.000100852	0.000574896
ENSMMUG00000000375	pleckstrin homology and RhoGEF domain containing G6 [Source:VGNC Symbol;Acc:VGNC:76110]	3.154916496	8.19E-94	1.15E-89
ENSMMUG00000053013		1.58873179	3.45E-10	5.92E-09
ENSMMUG00000014265	TAP binding protein like [Source:NCBI gene;Acc:712626]	1.925373142	1.06E-13	3.14E-12
ENSMMUG00000018685	chromodomain helicase DNA binding protein 4 [Source:VGNC Symbol;Acc:VGNC:71029]	-1.094541341	3.58E-09	5.22E-08
ENSMMUG00000017619	lysophosphatidic acid receptor 5 [Source:VGNC Symbol;Acc:VGNC:74277]	0.749010111	1.53E-05	0.000106251
ENSMMUG00000016983	acrosin binding protein [Source:VGNC Symbol;Acc:VGNC:69445]	-1.526339451	2.24E-10	3.99E-09
ENSMMUG00000050979	PILR alpha associated neural protein [Source:NCBI gene;Acc:713548]	-1.99964264	0.00084903	0.00382828
ENSMMUG00000010203	protein tyrosine phosphatase non-receptor type 6 [Source:VGNC Symbol;Acc:VGNC:76607]	0.530372856	0.008686645	0.028474246
ENSMMUG00000012786	complement C1s [Source:VGNC Symbol;Acc:VGNC:70297]	-0.596609878	4.57E-06	3.61E-05
ENSMMUG00000006470	complement C1r subcomponent [Source:NCBI gene;Acc:722133]	0.934470673	9.21E-14	2.75E-12
ENSMMUG00000022109	CD163 molecule like 1 [Source:VGNC Symbol;Acc:VGNC:70914]	0.909344869	2.10E-05	0.000141564
ENSMMUG00000057570		2.500147458	8.41E-22	6.30E-20
ENSMMUG00000057785		5.708652634	1.47E-06	1.28E-05
ENSMMUG00000061774	taste 2 receptor member 10 [Source:NCBI gene;Acc:717960]	-3.833969552	6.46E-05	0.000385998
ENSMMUG00000050862		-0.717711964	0.00364674	0.013500064
ENSMMUG00000018298	killer cell lectin like receptor D1 [Source:NCBI gene;Acc:574145]	2.61367791	0.000651664	0.003032952
ENSMMUG00000005832	C-type lectin domain family 1 member A [Source:VGNC Symbol;Acc:VGNC:71335]	-1.367373151	0.013161072	0.040314048
ENSMMUG00000015305	C-type lectin domain family 2 member A [Source:VGNC Symbol;Acc:VGNC:71329]	-1.57148119	0.004977747	0.017658219
ENSMMUG00000045446		-2.769804937	0.000203961	0.001089997
ENSMMUG00000032892	U6 spliceosomal RNA [Source:RFAM;Acc:RF00026]	-1.450904243	0.000555163	0.002649777
ENSMMUG00000044047	killer cell lectin like receptor B1 [Source:NCBI gene;Acc:100430783]	-0.869791357	0.003727717	0.013767126
ENSMMUG00000003238	pregnancy zone protein [Source:NCBI gene;Acc:100430319]	-0.593765894	0.002830346	0.01085908
ENSMMUG00000022680	alpha-2-macroglobulin like 1 [Source:VGNC Symbol;Acc:VGNC:69507]	-1.371792299	0.000345671	0.001732434
ENSMMUG00000022676	ribosomal modification protein rimK like family member B [Source:NCBI gene;Acc:722261]	-3.376052783	0.001111599	0.004836474

ENSMMUG00000060675		0.881345672	2.12E-08	2.69E-07
ENSMMUG00000021005	taste receptor type 2 member 50 [Source:NCBI gene;Acc:693513]	0.780433294	0.015477268	0.046168418
ENSMMUG00000056428	taste receptor type 2 member 20 [Source:NCBI gene;Acc:693643]	1.159605574	0.007332586	0.024616914
ENSMMUG00000050696	taste receptor type 2 member 31-like [Source:NCBI gene;Acc:693892]	1.291877195	3.81E-10	6.52E-09
ENSMMUG00000054225	taste receptor type 2 member 43 [Source:NCBI gene;Acc:694161]	1.805192582	2.54E-05	0.000167261
ENSMMUG00000042870	Macaca mulatta taste receptor, type 2, member 46 (TAS2R46), mRNA. [Source:RefSeq mRNA;Acc:NM_001080757]	0.901592372	0.003038699	0.011528553
ENSMMUG00000023317	LDL receptor related protein 6 [Source:VGNC Symbol;Acc:VGNC:74446]	-0.770162299	0.010207228	0.032537587
ENSMMUG00000018030	endoplasmic reticulum protein 27 [Source:VGNC Symbol;Acc:VGNC:72369]	1.055681844	0.005257721	0.018473823
ENSMMUG00000053781	Rho GDP dissociation inhibitor beta [Source:NCBI gene;Acc:702320]	2.046813044	3.35E-05	0.000215018
ENSMMUG00000020443	solute carrier organic anion transporter family member 1B3 [Source:NCBI gene;Acc:574354]	-0.376419762	0.016274471	0.048116574
ENSMMUG00000002679	lactate dehydrogenase B [Source:NCBI gene;Acc:706110]	2.222356079	0.008172934	0.027030437
ENSMMUG00000049682	Metazoan signal recognition particle RNA [Source:RFAM;Acc:RF00017]	-3.723128941	4.25E-06	3.38E-05
ENSMMUG00000048030	ferritin, heavy polypeptide 1-like [Source:NCBI gene;Acc:707011]	-2.910901733	0.001982489	0.007995525
ENSMMUG00000020370	SRY-box transcription factor 5 [Source:VGNC Symbol;Acc:VGNC:77843]	-3.735700537	2.42E-08	3.04E-07
ENSMMUG00000057276		-3.086464997	7.88E-07	7.30E-06
ENSMMUG00000060493		0.491978499	4.79E-05	0.000295697
ENSMMUG00000012223	branched chain amino acid transaminase 1 [Source:VGNC Symbol;Acc:VGNC:70236]	0.379761126	0.004198449	0.015280263
ENSMMUG00000053657		-2.881801224	0.000349772	0.001747365
ENSMMUG00000018630	sarcospan [Source:VGNC Symbol;Acc:VGNC:77887]	0.455390496	0.000336479	0.001695454
ENSMMUG00000060495		0.682202413	4.29E-05	0.000268371
ENSMMUG00000007050		0.89855232	0.0037487	0.013830049
ENSMMUG00000050627		0.289039193	0.012127472	0.037591947
ENSMMUG00000006468	FGFR1 oncogene partner 2 [Source:VGNC Symbol;Acc:VGNC:72551]	1.573375872	5.52E-13	1.49E-11
ENSMMUG00000003217	transmembrane 7 superfamily member 3 [Source:VGNC Symbol;Acc:VGNC:79371]	1.398860896	3.11E-14	1.01E-12
ENSMMUG00000023248	serine/threonine kinase 38 like [Source:VGNC Symbol;Acc:VGNC:78156]	-1.445926081	1.29E-06	1.14E-05
ENSMMUG00000023249	aryl hydrocarbon receptor nuclear translocator like 2 [Source:NCBI gene;Acc:709547]	-1.294099219	1.96E-07	2.05E-06
ENSMMUG00000021736	single-pass membrane protein with coiled-coil domains 2 [Source:VGNC Symbol;Acc:VGNC:77808]	1.105598536	7.70E-07	7.15E-06
ENSMMUG00000053437		0.63073586	4.63E-06	3.65E-05
ENSMMUG00000011802	coiled-coil domain containing 91 [Source:VGNC Symbol;Acc:VGNC:70852]	0.704513567	0.000259272	0.001353098
ENSMMUG00000015810	fatty acyl-CoA reductase 2 [Source:VGNC Symbol;Acc:VGNC:81403]	-0.766039798	0.000326245	0.001648016
ENSMMUG00000001357	tetraspanin 11 [Source:VGNC Symbol;Acc:VGNC:79882]	-1.413465933	3.96E-30	7.12E-28
ENSMMUG00000009155	SIN3-HDAC complex associated factor [Source:NCBI gene;Acc:694162]	-4.898290254	1.82E-14	6.07E-13
ENSMMUG00000039300		-3.171330144	6.98E-08	8.05E-07
ENSMMUG00000003221	DENN domain containing 5B [Source:VGNC Symbol;Acc:VGNC:71772]	-1.537125005	1.81E-06	1.54E-05
ENSMMUG00000063112		1.254600186	0.002667868	0.010320416
ENSMMUG00000013655	dynamin 1 like [Source:VGNC Symbol;Acc:VGNC:71956]	-1.305236169	0.010821567	0.034186084
ENSMMUG00000013666	copine 8 [Source:NCBI gene;Acc:114669741]	-2.645146384	0.005902789	0.020347789

ENSMMUG00000021163	contactin 1 [Source:VGNC Symbol;Acc:VGNC:71283]	-0.277025609	0.012127645	0.037591947
ENSMMUG00000056303		-2.36852091	0.000102648	0.000584898
ENSMMUG00000000205	transmembrane protein 117 [Source:VGNC Symbol;Acc:VGNC:79488]	-0.897359453	8.19E-12	1.86E-10
ENSMMUG00000060215	Y RNA [Source:RFAM;Acc:RF00019]	-0.987507961	1.81E-10	3.31E-09
ENSMMUG00000052030	developing brain homeobox 2 [Source:NCBI gene;Acc:701170]	-0.763193572	1.46E-07	1.57E-06
ENSMMUG00000015962	AT-rich interaction domain 2 [Source:VGNC Symbol;Acc:VGNC:69858]	-1.217921453	1.32E-09	2.05E-08
ENSMMUG00000037874	Metazoan signal recognition particle RNA [Source:RFAM;Acc:RF00017]	-1.073765388	0.006760948	0.022878566
ENSMMUG00000023791	SR-related CTD associated factor 11 [Source:VGNC Symbol;Acc:VGNC:77002]	-2.174103403	0.000287866	0.001479747
ENSMMUG00000022359	solute carrier family 38 member 2 [Source:VGNC Symbol;Acc:VGNC:77469]	-1.109783119	4.54E-07	4.43E-06
ENSMMUG00000064730		2.740620536	4.51E-23	3.75E-21
ENSMMUG00000012331	solute carrier family 38 member 4 [Source:VGNC Symbol;Acc:VGNC:77484]	-0.535791458	0.003612877	0.01338884
ENSMMUG00000007482	RNA polymerase II associated protein 3 [Source:VGNC Symbol;Acc:VGNC:76834]	-0.608675501	0.001507384	0.006289215
ENSMMUG00000049514	solute carrier family 48 member 1 [Source:NCBI gene;Acc:704304]	0.865555158	3.97E-06	3.18E-05
ENSMMUG00000021655	histone deacetylase 7 [Source:NCBI gene;Acc:718225]	0.931070811	8.01E-09	1.08E-07
ENSMMUG00000006727	vitamin D receptor [Source:VGNC Symbol;Acc:VGNC:78764]	0.532925531	3.18E-05	0.000204814
ENSMMUG00000020125	SUMO specific peptidase 1 [Source:NCBI gene;Acc:707603]	-1.641051246	0.001615384	0.006676255
ENSMMUG00000055133		-1.272286573	0.013123769	0.040243412
ENSMMUG00000014484	zinc finger protein 641 [Source:NCBI gene;Acc:708246]	-1.726819211	2.36E-09	3.54E-08
ENSMMUG00000003877	KAT8 regulatory NSL complex subunit 2 [Source:VGNC Symbol;Acc:VGNC:73910]	-0.897489919	0.002931377	0.011194634
ENSMMUG00000012769	limb development membrane protein 1 like [Source:VGNC Symbol;Acc:VGNC:74310]	-0.504439719	0.013808983	0.042023301
ENSMMUG00000021081	tubulin alpha 1b [Source:NCBI gene;Acc:709643]	-0.705698161	0.000666949	0.003096896
ENSMMUG00000009788	tubulin alpha-1C chain [Source:NCBI gene;Acc:710110]	1.015324816	0.000185109	0.000997993
ENSMMUG00000007544	trophinin associated protein [Source:VGNC Symbol;Acc:VGNC:78650]	0.696140602	0.000505302	0.002435835
ENSMMUG00000007548	DnaJ heat shock protein family (Hsp40) member C22 [Source:NCBI gene;Acc:710205]	1.290872451	0.000496679	0.002396742
ENSMMUG00000064430		0.68289196	4.66E-05	0.000288566
ENSMMUG00000003762	spermatogenesis associated serine rich 2 [Source:VGNC Symbol;Acc:VGNC:77958]	-0.463186127	0.005339737	0.018701083
ENSMMUG00000049374		0.59855341	0.000570967	0.002715835
ENSMMUG00000005960	transmembrane BAX inhibitor motif containing 6 [Source:NCBI gene;Acc:677702]	0.372340141	1.29E-06	1.13E-05
ENSMMUG00000038256	aquaporin 2 [Source:NCBI gene;Acc:711719]	1.233839292	5.13E-27	6.86E-25
ENSMMUG00000006529	acid sensing ion channel subunit 1 [Source:VGNC Symbol;Acc:VGNC:70035]	-0.87093922	0.007788042	0.025904107
ENSMMUG00000000152	transcription factor CP2 [Source:VGNC Symbol;Acc:VGNC:78323]	2.389522569	9.41E-11	1.80E-09
ENSMMUG00000004982	activin A receptor like type 1 [Source:VGNC Symbol;Acc:VGNC:69451]	3.285665726	0.000248553	0.001302973
ENSMMUG00000046877	activin A receptor type 1B [Source:NCBI gene;Acc:696587]	1.445211956	0.013880395	0.042203986
ENSMMUG00000022922	nuclear receptor subfamily 4 group A member 1 [Source:VGNC Symbol;Acc:VGNC:75517]	-0.40592457	0.000631354	0.002954283
ENSMMUG00000010000	keratin 7 [Source:VGNC Symbol;Acc:VGNC:74214]	-0.821372224	1.32E-15	5.12E-14
ENSMMUG00000064514	keratin, type II cuticular Hb1 [Source:NCBI gene;Acc:697511]	-0.854373088	1.48E-09	2.29E-08

ENSMMUG00000018217	keratin 84 [Source:VGNC Symbol;Acc:VGNC:74220]	-0.908965846	0.00919798	0.029836521
ENSMMUG00000057134	keratin, type II cytoskeletal 6C [Source:NCBI gene;Acc:100424162]	0.818827101	0.002737298	0.010556968
ENSMMUG00000055016	keratin, type II cytoskeletal 6A [Source:NCBI gene;Acc:719283]	0.446995418	1.32E-05	9.30E-05
ENSMMUG00000005246	zinc finger protein 740 [Source:VGNC Symbol;Acc:VGNC:79665]	-1.950787275	9.90E-09	1.32E-07
ENSMMUG00000052817	Sp7 transcription factor [Source:NCBI gene;Acc:702575]	-1.486481211	6.62E-06	5.03E-05
ENSMMUG00000020036	Sp1 transcription factor [Source:VGNC Symbol;Acc:VGNC:77844]	-1.105124592	5.86E-06	4.52E-05
ENSMMUG00000006608	poly(rC) binding protein 2 [Source:NCBI gene;Acc:703175]	-0.456955646	0.006713572	0.022745691
ENSMMUG00000018492	TARBP2 subunit of RISC loading complex [Source:VGNC Symbol;Acc:VGNC:78099]	1.632889761	2.38E-13	6.67E-12
ENSMMUG00000022313	activating transcription factor 7 [Source:NCBI gene;Acc:700403]	1.651376529	1.47E-11	3.19E-10
ENSMMUG00000055610	homeobox C4 [Source:NCBI gene;Acc:100430434]	-2.874408667	9.74E-35	2.73E-32
ENSMMUG00000052854	mucin like 1 [Source:NCBI gene;Acc:100423942]	1.107375964	9.16E-16	3.62E-14
ENSMMUG00000016203	thymocyte expressed, positive selection associated 1 [Source:VGNC Symbol;Acc:VGNC:79775]	0.444689502	0.001544289	0.006412565
ENSMMUG00000055590		-0.852085696	1.12E-08	1.48E-07
ENSMMUG00000013228	retinol dehydrogenase 5 [Source:VGNC Symbol;Acc:VGNC:82219]	-1.758036679	0.011092341	0.034918412
ENSMMUG00000006293	SAP domain containing ribonucleoprotein [Source:NCBI gene;Acc:709932]	0.801383663	7.33E-08	8.40E-07
ENSMMUG00000053458	ORMDL sphingolipid biosynthesis regulator 2 [Source:NCBI gene;Acc:710296]	0.560400142	0.000136926	0.000759238
ENSMMUG00000006295	matrix metallopeptidase 19 [Source:VGNC Symbol;Acc:VGNC:74896]	0.568621642	0.006789051	0.022957043
ENSMMUG00000011002	PYM homolog 1, exon junction complex associated factor [Source:VGNC Symbol;Acc:VGNC:76570]	-0.714688396	7.33E-06	5.52E-05
ENSMMUG00000018814	diacylglycerol kinase alpha [Source:VGNC Symbol;Acc:VGNC:71780]	-1.120311678	1.35E-05	9.53E-05
ENSMMUG00000031853	RAB5B, member RAS oncogene family [Source:VGNC Symbol;Acc:VGNC:81546]	-0.999519168	2.69E-06	2.22E-05
ENSMMUG00000064364	sulfite oxidase [Source:NCBI gene;Acc:711164]	-0.917819079	0.002040559	0.008203772
ENSMMUG00000064075	ring finger protein 41 [Source:NCBI gene;Acc:711969]	-0.683052514	0.003548559	0.013164404
ENSMMUG00000054020	U6 spliceosomal RNA [Source:RFAM;Acc:RF00026]	-1.261908273	0.000300437	0.001536481
ENSMMUG00000059663		-0.967032086	9.37E-06	6.85E-05
ENSMMUG00000000803	signal transducer and activator of transcription 2 [Source:VGNC Symbol;Acc:VGNC:77905]	1.664672037	1.64E-07	1.75E-06
ENSMMUG00000063898	apolipoprotein F [Source:NCBI gene;Acc:712761]	2.059927312	2.59E-11	5.40E-10
ENSMMUG00000053460		1.569936514	6.00E-07	5.72E-06
ENSMMUG00000021878	timeless circadian regulator [Source:VGNC Symbol;Acc:VGNC:78356]	-1.148438503	1.72E-06	1.47E-05
ENSMMUG00000013766	prostaglandin E synthase 3 [Source:NCBI gene;Acc:713353]	0.801770974	1.64E-05	0.000112736
ENSMMUG00000004522	DNA primase subunit 1 [Source:NCBI gene;Acc:710253]	0.392292674	0.001544723	0.006412565
ENSMMUG00000043411	zinc finger and BTB domain containing 39 [Source:NCBI gene;Acc:713914]	-1.440835051	3.71E-08	4.50E-07
ENSMMUG00000021114	G protein-coupled receptor 182 [Source:VGNC Symbol;Acc:VGNC:72936]	-0.692827167	0.00031783	0.001614245
ENSMMUG00000013518	myosin IA [Source:VGNC Symbol;Acc:VGNC:75110]	-0.403862603	0.00438045	0.015848051
ENSMMUG00000021782	LDL receptor related protein 1 [Source:VGNC Symbol;Acc:VGNC:74442]	-0.905368192	1.26E-12	3.21E-11

ENSMMUG00000004233	leucine rich repeats and immunoglobulin like domains 3 [Source:NCBI gene;Acc:716344]	-1.034758275	3.35E-09	4.91E-08
ENSMMUG00000055477		-1.413332132	3.94E-10	6.74E-09
ENSMMUG00000001210	elongation factor 1-alpha 1 [Source:NCBI gene;Acc:100425937]	-2.861175428	0.010460733	0.033210517
ENSMMUG00000062139		1.937145071	1.53E-07	1.64E-06
ENSMMUG00000011579	TAFA chemokine like family member 2 [Source:VGNC Symbol;Acc:VGNC:78189]	0.535364087	0.00080597	0.003669667
ENSMMUG00000020533	chromosome 11 C12orf56 homolog [Source:VGNC Symbol;Acc:VGNC:70409]	-5.208378393	1.52E-15	5.82E-14
ENSMMUG00000003669	methionine sulfoxide reductase B3 [Source:VGNC Symbol;Acc:VGNC:74842]	0.494064865	0.000605517	0.0028638
ENSMMUG00000041729		1.101536916	0.000221281	0.001174055
ENSMMUG00000054816	interleukin 1 receptor associated kinase 3 [Source:NCBI gene;Acc:717845]	2.369432405	0.014022222	0.042561389
ENSMMUG00000016631	glutamate receptor interacting protein 1 [Source:VGNC Symbol;Acc:VGNC:73195]	-0.906061431	0.010207674	0.032537587
ENSMMUG00000011650	dual specificity tyrosine phosphorylation regulated kinase 2 [Source:VGNC Symbol;Acc:VGNC:72018]	1.168210246	1.44E-05	0.000100435
ENSMMUG00000059548		1.709567495	0.006385913	0.021797469
ENSMMUG00000055447	MDM2 proto-oncogene [Source:NCBI gene;Acc:718284]	-1.502788845	3.49E-20	2.19E-18
ENSMMUG00000053722		2.193227733	0.000439015	0.002147298
ENSMMUG00000046235	YEATS domain containing 4 [Source:NCBI gene;Acc:718376]	-2.954476855	0.011362214	0.035644816
ENSMMUG00000044169	Metazoan signal recognition particle RNA [Source:RFAM;Acc:RF00017]	-2.251994503	0.016283686	0.048133671
ENSMMUG00000010819	fibroblast growth factor receptor substrate 2 [Source:VGNC Symbol;Acc:VGNC:72732]	-1.047784439	6.05E-13	1.61E-11
ENSMMUG00000010820	chaperonin containing TCP1 subunit 2 [Source:VGNC Symbol;Acc:VGNC:70745]	-2.450974441	1.77E-13	5.03E-12
ENSMMUG00000054953	leucine rich repeat containing 10 [Source:NCBI gene;Acc:114669753]	-2.714386717	7.85E-05	0.0004598
ENSMMUG00000007727	TBC1 domain family member 15 [Source:VGNC Symbol;Acc:VGNC:78215]	3.303595888	0.006686076	0.022663486
ENSMMUG00000012289	GLIPR1 like 1 [Source:VGNC Symbol;Acc:VGNC:81412]	-0.783700812	0.000631795	0.002954966
ENSMMUG00000012290	GLIPR1 like 2 [Source:VGNC Symbol;Acc:VGNC:81413]	-0.973671179	0.008478579	0.027903099
ENSMMUG00000017864	oxysterol binding protein like 8 [Source:VGNC Symbol;Acc:VGNC:75630]	1.151636758	0.00134238	0.005685991
ENSMMUG00000059985		5.042139462	1.41E-06	1.23E-05
ENSMMUG00000005530	synaptotagmin 1 [Source:VGNC Symbol;Acc:VGNC:78074]	0.600123608	0.014877111	0.044653623
ENSMMUG00000062826		-3.50375082	0.005195678	0.018287898
ENSMMUG00000017463		-3.562389396	0.003239471	0.012153787
ENSMMUG00000015862	centrosomal protein 290 [Source:VGNC Symbol;Acc:VGNC:71126]	-0.579260675	0.000364338	0.001812385
ENSMMUG00000059975		0.369809545	0.008199663	0.027112445
ENSMMUG00000049117	CLLU1 antisense RNA 1 [Source:NCBI gene;Acc:100424000]	-4.101628315	0.000245863	0.001291289
ENSMMUG00000002243	pleckstrin homology and RhoGEF domain containing G7 [Source:VGNC Symbol;Acc:VGNC:76149]	-0.777162363	8.40E-07	7.73E-06
ENSMMUG00000002244	early endosome antigen 1 [Source:VGNC Symbol;Acc:VGNC:72161]	-0.949168921	3.18E-07	3.20E-06
ENSMMUG00000052496		-0.88098085	1.41E-12	3.57E-11
ENSMMUG00000055071	Y RNA [Source:RFAM;Acc:RF00019]	-1.29220773	0.013472703	0.041169691
ENSMMUG00000018237	plexin C1 [Source:VGNC Symbol;Acc:VGNC:76190]	1.424131724	5.44E-16	2.23E-14
ENSMMUG00000018239	centrosomal protein 83 [Source:VGNC Symbol;Acc:VGNC:70992]	1.324752024	1.30E-09	2.04E-08

ENSMMUG00000018901	FYVE, RhoGEF and PH domain containing 6 [Source:VGNC Symbol;Acc:VGNC:72548]	-1.720011135	6.47E-13	1.72E-11
ENSMMUG00000018903	vezatin, adherens junctions transmembrane protein [Source:VGNC Symbol;Acc:VGNC:79120]	-0.68978838	1.06E-05	7.68E-05
ENSMMUG00000062006	methionyl aminopeptidase 2 [Source:NCBI gene;Acc:712400]	-1.15609535	8.21E-08	9.35E-07
ENSMMUG00000013812	ubiquitin specific peptidase 44 [Source:VGNC Symbol;Acc:VGNC:79221]	-1.196570142	4.71E-06	3.71E-05
ENSMMUG00000009303	coiled-coil domain containing 38 [Source:VGNC Symbol;Acc:VGNC:70880]	-1.11438903	6.49E-06	4.96E-05
ENSMMUG00000009308	histidine ammonia-lyase [Source:VGNC Symbol;Acc:VGNC:73344]	2.814059338	1.26E-07	1.37E-06
ENSMMUG00000009312	leukotriene A4 hydrolase [Source:VGNC Symbol;Acc:VGNC:74453]	2.779323689	1.89E-23	1.66E-21
ENSMMUG00000053376		2.236107523	4.90E-16	2.02E-14
ENSMMUG00000050585		2.101953654	6.04E-13	1.61E-11
ENSMMUG00000022404	apoptotic peptidase activating factor 1 [Source:VGNC Symbol;Acc:VGNC:69975]	0.53321351	2.83E-07	2.88E-06
ENSMMUG00000022406	ankyrin repeat and sterile alpha motif domain containing 1B [Source:VGNC Symbol;Acc:VGNC:69689]	1.132155825	4.98E-08	5.87E-07
ENSMMUG00000061260	Spi-C transcription factor [Source:NCBI gene;Acc:695809]	-0.980694928	3.56E-07	3.57E-06
ENSMMUG00000042077	DNA damage regulated autophagy modulator 1 [Source:NCBI gene;Acc:697427]	0.720625293	1.33E-08	1.75E-07
ENSMMUG00000046478	WASH complex subunit 3 [Source:NCBI gene;Acc:697824]	2.063786607	1.01E-16	4.44E-15
ENSMMUG00000061923		1.801714289	0.000588659	0.002791601
ENSMMUG00000005504	nucleoporin 37 [Source:VGNC Symbol;Acc:VGNC:75491]	2.035368107	1.02E-07	1.14E-06
ENSMMUG00000061953		-2.883522111	0.005562289	0.019359636
ENSMMUG00000023469	heat shock protein 90 beta family member 1 [Source:NCBI gene;Acc:699568]	-0.844897826	0.005078161	0.017928262
ENSMMUG00000021291	thymine DNA glycosylase [Source:VGNC Symbol;Acc:VGNC:78297]	-0.784321445	0.003243527	0.012161492
ENSMMUG00000057796	host cell factor C2 [Source:NCBI gene;Acc:700316]	-0.685092754	4.43E-05	0.000275302
ENSMMUG00000022570	nuclear transcription factor Y subunit beta [Source:NCBI gene;Acc:700451]	-0.722713785	5.48E-05	0.000333875
ENSMMUG00000012091	thioredoxin reductase 1 [Source:VGNC Symbol;Acc:VGNC:78679]	-0.568114122	1.71E-05	0.000117544
ENSMMUG00000017127	carbohydrate sulfotransferase 11 [Source:VGNC Symbol;Acc:VGNC:82123]	0.95607829	0.003640543	0.01348068
ENSMMUG00000017129	solute carrier family 41 member 2 [Source:VGNC Symbol;Acc:VGNC:77585]	1.283731446	4.02E-16	1.69E-14
ENSMMUG00000009519	WASH complex subunit 4 [Source:VGNC Symbol;Acc:VGNC:79783]	-1.078645473	9.42E-05	0.000541005
ENSMMUG00000009524	adaptor protein, phosphotyrosine interacting with PH domain and leucine zipper 2 [Source:VGNC Symbol;Acc:VGNC:69861]	-1.235321916	1.22E-07	1.33E-06
ENSMMUG00000005208	RNA polymerase III subunit B [Source:VGNC Symbol;Acc:VGNC:76143]	2.081918051	7.25E-09	9.95E-08
ENSMMUG00000021808	cryptochrome circadian regulator 1 [Source:VGNC Symbol;Acc:VGNC:71499]	-0.562348736	0.000486791	0.002356322
ENSMMUG00000015544	PWP1 homolog, endonuclein [Source:NCBI gene;Acc:705262]	-1.495726843	0.014408524	0.043555252
ENSMMUG00000015550	PR/SET domain 4 [Source:VGNC Symbol;Acc:VGNC:76393]	-1.299599311	9.40E-05	0.000539752
ENSMMUG00000057713	Metazoan signal recognition particle RNA [Source:RFAM;Acc:RF00017]	-1.6483875	0.002385031	0.009373681
ENSMMUG00000006893	D-amino acid oxidase [Source:VGNC Symbol;Acc:VGNC:71729]	1.234953641	1.44E-17	6.96E-16
ENSMMUG00000015108	SV2 related protein [Source:VGNC Symbol;Acc:VGNC:78208]	0.832496833	1.26E-07	1.37E-06
ENSMMUG00000056729	U6 spliceosomal RNA [Source:RFAM;Acc:RF00026]	0.89358914	1.92E-05	0.000130589
ENSMMUG00000039553	ubiquitin specific peptidase 30 [Source:NCBI gene;Acc:706607]	0.978003712	6.72E-05	0.00039963
ENSMMUG00000006957	uracil DNA glycosylase [Source:VGNC Symbol;Acc:VGNC:78725]	0.772354099	0.001086727	0.004742971
ENSMMUG00000023032	intraflagellar transport 81 [Source:VGNC Symbol;Acc:VGNC:73389]	0.871076368	1.10E-09	1.74E-08
ENSMMUG00000001739	ATPase sarcoplasmic/endoplasmic reticulum Ca2+ transporting 2 [Source:VGNC Symbol;Acc:VGNC:70178]	0.928692337	1.86E-11	3.96E-10
ENSMMUG00000003297	coiled-coil domain containing 63 [Source:VGNC Symbol;Acc:VGNC:70879]	0.725306645	1.36E-05	9.53E-05
ENSMMUG00000002347	myosin light chain 2 [Source:VGNC Symbol;Acc:VGNC:75103]	0.69108031	0.000803526	0.003661958

ENSMMUG00000008336	acyl-CoA dehydrogenase family member 10 [Source:NCBI gene;Acc:711080]	0.784905231	5.32E-14	1.66E-12
ENSMMUG00000053496		0.864000513	0.00047317	0.002296206
ENSMMUG00000060754	Metazoan signal recognition particle RNA [Source:RFAM;Acc:RF00017]	0.875340858	1.74E-07	1.84E-06
ENSMMUG00000005255	TRAF-type zinc finger domain containing 1 [Source:VGNC Symbol;Acc:VGNC:79459]	1.015603607	4.97E-07	4.80E-06
ENSMMUG00000007126	HECT domain E3 ubiquitin protein ligase 4 [Source:VGNC Symbol;Acc:VGNC:73245]	-0.281404311	0.013888965	0.042220889
ENSMMUG00000012778	rabphilin 3A [Source:VGNC Symbol;Acc:VGNC:77033]	-1.939772383	1.22E-13	3.57E-12
ENSMMUG00000004435	two pore segment channel 1 [Source:VGNC Symbol;Acc:VGNC:79117]	1.404945802	6.83E-06	5.18E-05
ENSMMUG00000006723	solute carrier family 8 member B1 [Source:NCBI gene;Acc:714561]	0.961917339	0.000765479	0.003503363
ENSMMUG00000004205	phospholipase B domain containing 2 [Source:VGNC Symbol;Acc:VGNC:76122]	-2.263369631	0.000325498	0.001645444
ENSMMUG00000004208	serine dehydratase [Source:VGNC Symbol;Acc:VGNC:77148]	-1.653991601	0.000580976	0.002757962
ENSMMUG00000051363		1.036949982	0.000105127	0.000597079
ENSMMUG00000054768		0.82583343	1.58E-05	0.000109215
ENSMMUG00000057506		0.556661495	0.000321546	0.001628399
ENSMMUG00000052680		0.786644632	9.20E-07	8.37E-06
ENSMMUG00000017478	tescalcin [Source:VGNC Symbol;Acc:VGNC:79571]	-0.717738413	0.000690945	0.003197731
ENSMMUG00000014027	nitric oxide synthase 1 [Source:VGNC Symbol;Acc:VGNC:75368]	-1.221662736	0.000278099	0.001437441
ENSMMUG00000062615		-1.226276372	0.000247371	0.001297747
ENSMMUG00000020629	SDS3 homolog, SIN3A corepressor complex component [Source:NCBI gene;Acc:696166]	0.721821541	1.60E-06	1.38E-05
ENSMMUG00000053710		0.690774677	5.70E-07	5.44E-06
ENSMMUG00000011209	BICD family like cargo adaptor 1 [Source:HGNC Symbol;Acc:HGNC:28095]	0.913507349	1.27E-05	8.99E-05
ENSMMUG00000055209		0.869854494	2.25E-09	3.39E-08
ENSMMUG00000046665	glutamyl-tRNA amidotransferase subunit C [Source:NCBI gene;Acc:702258]	-0.917479081	0.010895059	0.034379257
ENSMMUG00000020601	coenzyme Q5, methyltransferase [Source:VGNC Symbol;Acc:VGNC:81376]	0.506679399	9.07E-05	0.000523125
ENSMMUG00000046646	Y RNA [Source:RFAM;Acc:RF00019]	0.763411881	7.63E-05	0.000448126
ENSMMUG00000004223	ring finger protein 10 [Source:VGNC Symbol;Acc:VGNC:76702]	-0.929915952	2.96E-07	3.00E-06
ENSMMUG00000055513		-1.477584124	0.015866066	0.04713768
ENSMMUG00000058536		2.292743838	2.50E-20	1.62E-18
ENSMMUG00000002536	calcium binding protein 1 [Source:VGNC Symbol;Acc:VGNC:70495]	1.725370213	1.14E-10	2.14E-09
ENSMMUG00000021564	lysine demethylase 2B [Source:VGNC Symbol;Acc:VGNC:81425]	3.334133218	4.12E-20	2.55E-18
ENSMMUG00000059782		4.005267406	5.45E-15	1.96E-13
ENSMMUG00000057798		-1.645983878	0.000849567	0.003829471
ENSMMUG00000016454	4-hydroxyphenylpyruvate dioxygenase [Source:VGNC Symbol;Acc:VGNC:73511]	0.702083936	1.15E-07	1.27E-06
ENSMMUG00000013643	zinc finger CCHC-type containing 8 [Source:VGNC Symbol;Acc:VGNC:79487]	-1.899162694	0.002637121	0.010221211
ENSMMUG00000004412	arginine and serine rich coiled-coil 2 [Source:VGNC Symbol;Acc:VGNC:77139]	-3.235769717	4.78E-07	4.64E-06
ENSMMUG00000007556	kinetochore associated 1 [Source:VGNC Symbol;Acc:VGNC:74064]	0.464222264	0.000757218	0.003468956
ENSMMUG00000063387	Metazoan signal recognition particle RNA [Source:RFAM;Acc:RF00017]	1.848245428	0.002960031	0.011273361
ENSMMUG00000015221	huntingtin interacting protein 1 related [Source:VGNC Symbol;Acc:VGNC:73412]	0.662575477	0.000484422	0.002346475
ENSMMUG00000005301	ATP binding cassette subfamily B member 9 [Source:VGNC Symbol;Acc:VGNC:81341]	-1.39561398	3.75E-08	4.54E-07
ENSMMUG00000015926	2-oxoglutarate and iron dependent oxygenase domain containing 2 [Source:VGNC Symbol;Acc:VGNC:75462]	1.247523431	2.99E-07	3.02E-06
ENSMMUG00000015930	ADP ribosylation factor like GTPase 6 interacting protein 4 [Source:VGNC Symbol;Acc:VGNC:69860]	1.507115077	0.000828996	0.003751212
ENSMMUG00000015931	phosphatidylinositol transfer protein membrane associated 2 [Source:VGNC Symbol;Acc:VGNC:75843]	1.421835054	4.96E-10	8.31E-09

ENSMMUG00000031495	chromosome 11 C12orf65 homolog [Source:NCBI gene;Acc:708974]	-1.609255737	0.002646202	0.010253576
ENSMMUG00000000434	cyclin dependent kinase 2 associated protein 1 [Source:VGNC Symbol;Acc:VGNC:70944]	-0.863723991	0.007945237	0.026364425
ENSMMUG00000013514	lysine methyltransferase 5A [Source:VGNC Symbol;Acc:VGNC:74159]	-0.564146296	0.000255881	0.001337889
ENSMMUG00000023048	cytochrome b-c1 complex subunit Rieske, mitochondrial [Source:NCBI gene;Acc:705178]	1.200381171	0.001122554	0.004876571
ENSMMUG00000058836	transmembrane p24 trafficking protein 2 [Source:NCBI gene;Acc:106992483]	-1.077278279	3.56E-08	4.33E-07
ENSMMUG00000059674		-1.717105733	0.003676635	0.013603549
ENSMMUG00000006546	ATPase H+ transporting V0 subunit a2 [Source:VGNC Symbol;Acc:VGNC:70096]	-0.449010308	0.000211099	0.001124711
ENSMMUG00000006861	dynein axonemal heavy chain 10 [Source:NCBI gene;Acc:710343]	-0.704987864	1.05E-05	7.59E-05
ENSMMUG00000011117	acetoacetyl-CoA synthetase [Source:NCBI gene;Acc:707015]	-0.593420681	0.003055906	0.011578754
ENSMMUG00000060158		-3.251952335	0.005033566	0.017815673
ENSMMUG00000050145	Metazoan signal recognition particle RNA [Source:RFAM;Acc:RF00017]	-3.701302589	0.002306161	0.009094291
ENSMMUG00000020926	syntaxin 2 [Source:VGNC Symbol;Acc:VGNC:78259]	-3.344469819	6.60E-06	5.03E-05
ENSMMUG00000058674		-2.838717474	0.016963812	0.049849862
ENSMMUG00000017026	fibrosin like 1 [Source:NCBI gene;Acc:696295]	0.570905533	0.004926413	0.017511556
ENSMMUG00000015463	DNA polymerase epsilon, catalytic subunit [Source:NCBI gene;Acc:698023]	-0.558917915	0.000674529	0.003128983
ENSMMUG00000031451	golgin A3 [Source:VGNC Symbol;Acc:VGNC:73106]	1.135084819	2.92E-06	2.39E-05
ENSMMUG00000015468	checkpoint with forkhead and ring finger domains [Source:VGNC Symbol;Acc:VGNC:71064]	3.116534279	3.76E-25	4.06E-23
ENSMMUG00000023321	UDP-glucose glycoprotein glucosyltransferase 1 [Source:VGNC Symbol;Acc:VGNC:78981]	0.532551841	0.000415555	0.00204396
ENSMMUG00000049232	AMMECR1 like [Source:VGNC Symbol;Acc:VGNC:84208]	6.631948114	8.86E-12	2.01E-10
ENSMMUG00000001989	RNA polymerase II subunit D [Source:VGNC Symbol;Acc:VGNC:76213]	7.208056848	7.32E-15	2.59E-13
ENSMMUG00000027543	Y RNA [Source:RFAM;Acc:RF00019]	7.11995716	1.26E-08	1.66E-07
ENSMMUG00000004177	myosin VIIB [Source:NCBI gene;Acc:714563]	0.330372511	0.001628534	0.006722678
ENSMMUG00000056151		1.6256449	0.011754484	0.036654021
ENSMMUG00000000376	bridging integrator 1 [Source:VGNC Symbol;Acc:VGNC:70251]	2.207512217	0.000258012	0.001347265
ENSMMUG00000020928	nucleolar protein interacting with the FHA domain of MKI67 [Source:VGNC Symbol;Acc:VGNC:75201]	-1.377341755	2.62E-08	3.26E-07
ENSMMUG00000046774	RAS like proto-oncogene B [Source:NCBI gene;Acc:695852]	1.962732536	3.32E-13	9.17E-12
ENSMMUG00000059182		1.95385831	2.95E-16	1.25E-14
ENSMMUG00000063759	transmembrane protein 185B [Source:NCBI gene;Acc:695744]	1.934548097	1.70E-26	2.21E-24
ENSMMUG00000059504		2.492368645	3.25E-08	3.99E-07
ENSMMUG00000051751		0.62937339	0.000427615	0.002099594
ENSMMUG00000005610	protein tyrosine phosphatase non-receptor type 4 [Source:VGNC Symbol;Acc:VGNC:76605]	-0.42575409	0.007762507	0.025843692
ENSMMUG00000006446	transmembrane protein 177 [Source:VGNC Symbol;Acc:VGNC:79686]	-2.467174849	0.000489702	0.002367954
ENSMMUG00000008875	solute carrier family 35 member F5 [Source:VGNC Symbol;Acc:VGNC:77567]	1.058612276	6.56E-07	6.18E-06
ENSMMUG00000043114	U6 spliceosomal RNA [Source:RFAM;Acc:RF00026]	0.646457063	0.004637088	0.016634916
ENSMMUG00000009844	R3H domain containing 1 [Source:VGNC Symbol;Acc:VGNC:76637]	-0.835102376	0.014223522	0.043079148
ENSMMUG00000017486	minichromosome maintenance complex component 6 [Source:VGNC Symbol;Acc:VGNC:74685]	-0.514496116	0.009599168	0.0308595
ENSMMUG00000040854	aspartyl-tRNA synthetase 1 [Source:VGNC Symbol;Acc:VGNC:84226]	-1.244565095	9.99E-07	9.04E-06

ENSMMUG00000006528	Rho GTPase activating protein 15 [Source:VGNC Symbol;Acc:VGNC:69874]	0.605978235	4.36E-10	7.39E-09
ENSMMUG00000016845	zinc finger E-box binding homeobox 2 [Source:VGNC Symbol;Acc:VGNC:78969]	-2.821310056	9.17E-05	0.000527868
ENSMMUG00000000042	activin A receptor type 2A [Source:VGNC Symbol;Acc:VGNC:69447]	-1.306132207	0.010089142	0.032220459
ENSMMUG00000008615	nebulin [Source:NCBI gene;Acc:100430324]	0.685220605	4.61E-06	3.64E-05
ENSMMUG00000010521	ADP ribosylation factor like GTPase 5A [Source:VGNC Symbol;Acc:VGNC:70017]	1.967575462	0.000365284	0.001815802
ENSMMUG00000010526	calcium voltage-gated channel auxiliary subunit beta 4 [Source:VGNC Symbol;Acc:VGNC:82116]	1.252116338	2.05E-05	0.000138661
ENSMMUG00000011637	coiled-coil domain containing 148 [Source:VGNC Symbol;Acc:VGNC:70856]	1.822728005	0.005678973	0.019687576
ENSMMUG00000011638	plakophilin 4 [Source:VGNC Symbol;Acc:VGNC:76153]	-1.665878134	0.005520779	0.019248602
ENSMMUG00000052818	death associated protein like 1 [Source:NCBI gene;Acc:699071]	-1.672562543	0.010143712	0.032379986
ENSMMUG00000057379		-1.451673905	0.000337413	0.001698328
ENSMMUG00000006180	tetratricopeptide repeat, ankyrin repeat and coiled-coil containing 1 [Source:VGNC Symbol;Acc:VGNC:78238]	1.347972692	2.81E-11	5.82E-10
ENSMMUG00000055201	U6 spliceosomal RNA [Source:RFAM;Acc:RF00026]	2.571725362	0.006163195	0.021126006
ENSMMUG00000006181	WD repeat, sterile alpha motif and U-box domain containing 1 [Source:VGNC Symbol;Acc:VGNC:79634]	1.16261593	2.38E-08	3.00E-07
ENSMMUG00000063868	Metazoan signal recognition particle RNA [Source:RFAM;Acc:RF00017]	1.00041683	0.003452829	0.012847119
ENSMMUG00000006182	bromodomain adjacent to zinc finger domain 2B [Source:NCBI gene;Acc:702780]	0.553113707	1.34E-05	9.46E-05
ENSMMUG00000051450	phospholipase A2 receptor 1 [Source:NCBI gene;Acc:700191]	0.602765699	2.88E-07	2.92E-06
ENSMMUG00000002678	integrin subunit beta 6 [Source:VGNC Symbol;Acc:VGNC:73789]	-1.549047243	7.62E-09	1.04E-07
ENSMMUG00000055970		-1.041750498	1.81E-07	1.91E-06
ENSMMUG00000008384	solute carrier family 4 member 10 [Source:VGNC Symbol;Acc:VGNC:77695]	1.674479026	1.37E-12	3.47E-11
ENSMMUG00000008387	dipeptidyl peptidase 4 [Source:VGNC Symbol;Acc:VGNC:71979]	2.123563063	1.19E-05	8.50E-05
ENSMMUG00000013251	xin actin binding repeat containing 2 [Source:VGNC Symbol;Acc:VGNC:78953]	1.842540537	0.000125874	0.000704079
ENSMMUG00000063463		-1.249015934	2.05E-05	0.000138922
ENSMMUG00000021775	beta-1,3-galactosyltransferase 1 [Source:VGNC Symbol;Acc:VGNC:70204]	-0.89873091	0.000297044	0.001522459
ENSMMUG00000006215	serine/threonine kinase 39 [Source:VGNC Symbol;Acc:VGNC:81587]	-0.815797229	0.000336933	0.001696814
ENSMMUG00000013830	glucose-6-phosphatase catalytic subunit 2 [Source:NCBI gene;Acc:709062]	2.200419143	8.39E-13	2.19E-11
ENSMMUG00000013832	ATP binding cassette subfamily B member 11 [Source:VGNC Symbol;Acc:VGNC:81339]	2.297045267	8.52E-11	1.65E-09
ENSMMUG00000017143	LDL receptor related protein 2 [Source:VGNC Symbol;Acc:VGNC:81430]	-1.907707129	0.003954409	0.0144905
ENSMMUG00000058098		-0.570479582	0.000344446	0.00172818
ENSMMUG00000020510	kelch like family member 41 [Source:VGNC Symbol;Acc:VGNC:74062]	-0.987359734	4.90E-08	5.78E-07
ENSMMUG00000015198	glutamate decarboxylase 1 [Source:VGNC Symbol;Acc:VGNC:72862]	-0.804096097	0.00016334	0.000890907
ENSMMUG00000015203	golgi reassembly stacking protein 2 [Source:VGNC Symbol;Acc:VGNC:72957]	-0.726012667	0.001099234	0.004790104
ENSMMUG00000011189	tousled like kinase 1 [Source:VGNC Symbol;Acc:VGNC:78367]	-0.581567765	0.009019455	0.029359288
ENSMMUG00000054176	methyltransferase like 8 [Source:NCBI gene;Acc:695361]	-0.897396426	0.002618011	0.010169631
ENSMMUG00000023370	DDB1 and CUL4 associated factor 17 [Source:VGNC Symbol;Acc:VGNC:71709]	1.702591275	2.61E-12	6.38E-11
ENSMMUG00000007301	dynein cytoplasmic 1 intermediate chain 2 [Source:VGNC Symbol;Acc:VGNC:71953]	-0.619620952	0.000266913	0.001387808
ENSMMUG00000007310	solute carrier family 25 member 12 [Source:VGNC Symbol;Acc:VGNC:77430]	-1.092262619	1.85E-08	2.38E-07

ENSMMUG00000058130	Metazoan signal recognition particle RNA [Source:RFAM;Acc:RF00017]	-1.936313849	0.01584138	0.04707541
ENSMMUG00000007313	histone acetyltransferase 1 [Source:VGNC Symbol;Acc:VGNC:73349]	-1.625639714	6.18E-06	4.74E-05
ENSMMUG00000002370	integrin subunit alpha 6 [Source:VGNC Symbol;Acc:VGNC:73780]	-1.035666157	3.39E-11	6.91E-10
ENSMMUG00000054462		-1.111568229	1.81E-10	3.30E-09
ENSMMUG00000061038		-2.251080879	0.000463023	0.002253723
ENSMMUG00000022553	pyruvate dehydrogenase kinase 1 [Source:VGNC Symbol;Acc:VGNC:75932]	-3.455151994	0.001089042	0.004750694
ENSMMUG00000020792	Rap guanine nucleotide exchange factor 4 [Source:VGNC Symbol;Acc:VGNC:76490]	-1.550944381	2.67E-06	2.20E-05
ENSMMUG00000014672	mitogen-activated protein kinase kinase kinase 20 [Source:NCBI gene;Acc:698274]	-2.188037589	6.23E-05	0.000374418
ENSMMUG00000023006	corepressor interacting with RBPJ, 1 [Source:VGNC Symbol;Acc:VGNC:71331]	-0.989155399	0.001157793	0.005011029
ENSMMUG00000021012	G protein-coupled receptor 155 [Source:VGNC Symbol;Acc:VGNC:72985]	0.4744023	3.31E-06	2.68E-05
ENSMMUG00000053004		0.724665864	6.71E-05	0.000399206
ENSMMUG00000009593	WAS/WASL interacting protein family member 1 [Source:VGNC Symbol;Acc:VGNC:78784]	1.573178825	6.12E-16	2.49E-14
ENSMMUG00000065344	homeobox D1 [Source:NCBI gene;Acc:701064]	-0.727373664	0.000517678	0.002483546
ENSMMUG00000008502	alkylglycerone phosphate synthase [Source:VGNC Symbol;Acc:VGNC:69747]	-0.285794044	0.013534171	0.041321505
ENSMMUG00000002183	oxysterol binding protein like 6 [Source:VGNC Symbol;Acc:VGNC:75627]	-1.262315845	2.43E-07	2.50E-06
ENSMMUG00000002185	protein activator of interferon induced protein kinase EIF2AK2 [Source:VGNC Symbol;Acc:VGNC:76421]	-0.664468841	2.82E-05	0.000183677
ENSMMUG00000054264	pejvakin [Source:NCBI gene;Acc:708668]	-0.640045245	0.000938998	0.004165636
ENSMMUG00000009038	titin [Source:NCBI gene;Acc:703527]	-2.040986796	2.41E-05	0.00016008
ENSMMUG00000019690	SEC14 and spectrin domain containing 1 [Source:VGNC Symbol;Acc:VGNC:77267]	2.321676634	3.73E-12	8.88E-11
ENSMMUG00000053406		3.640454794	1.88E-05	0.000128112
ENSMMUG00000049746		-0.37311349	0.009162272	0.02974133
ENSMMUG00000014277	ITPR interacting domain containing 2 [Source:VGNC Symbol;Acc:VGNC:73584]	1.24771164	2.08E-13	5.84E-12
ENSMMUG00000014394	DnaJ heat shock protein family (Hsp40) member C10 [Source:VGNC Symbol;Acc:VGNC:84229]	-1.359520014	8.29E-21	5.56E-19
ENSMMUG00000014397	NCK associated protein 1 [Source:VGNC Symbol;Acc:VGNC:75143]	3.011324217	4.70E-22	3.56E-20
ENSMMUG00000019462	integrin subunit alpha V [Source:VGNC Symbol;Acc:VGNC:73783]	-0.944041824	7.14E-06	5.39E-05
ENSMMUG00000010405	family with sequence similarity 171 member B [Source:VGNC Symbol;Acc:VGNC:72404]	-1.410903882	2.02E-06	1.70E-05
ENSMMUG00000004022	zinc finger SWIM-type containing 2 [Source:VGNC Symbol;Acc:VGNC:79908]	2.07348911	1.49E-19	8.73E-18
ENSMMUG00000003506	solute carrier family 40 member 1 [Source:VGNC Symbol;Acc:VGNC:77384]	-1.1351855	0.01358418	0.041438101
ENSMMUG00000014314	ankyrin and armadillo repeat containing [Source:VGNC Symbol;Acc:VGNC:69706]	-1.47136973	9.86E-15	3.44E-13
ENSMMUG00000061305		-0.78179117	0.002126617	0.008512141
ENSMMUG00000003260		-1.507598785	1.16E-14	4.02E-13
ENSMMUG00000064089		-1.155799992	7.55E-15	2.66E-13
ENSMMUG00000014307	myostatin [Source:VGNC Symbol;Acc:VGNC:74870]	-1.193508849	1.04E-11	2.32E-10

ENSMMUG00000007283	3-hydroxyisobutyryl-CoA hydrolase [Source:NCBI gene;Acc:713951]	-0.729475201	0.004830764	0.017228334
ENSMMUG00000005612	glutaminase [Source:NCBI gene;Acc:693520]	-1.67826988	0.007252553	0.024383255
ENSMMUG00000005613	signal transducer and activator of transcription 1 [Source:VGNC Symbol;Acc:VGNC:77904]	-0.72052741	8.33E-08	9.48E-07
ENSMMUG00000001761	myosin IB [Source:VGNC Symbol;Acc:VGNC:75111]	0.717708336	0.001935133	0.007827045
ENSMMUG00000037175	U6 spliceosomal RNA [Source:RFAM;Acc:RF00026]	1.441696273	0.000684924	0.003173004
ENSMMUG00000001475	transmembrane protein with EGF like and two follistatin like domains 2 [Source:VGNC Symbol;Acc:VGNC:78569]	2.638188145	3.58E-09	5.22E-08
ENSMMUG00000055355		-0.441184494	0.001508905	0.006293687
ENSMMUG00000061797	U6 spliceosomal RNA [Source:RFAM;Acc:RF00026]	1.087265372	0.006150741	0.021093628
ENSMMUG00000061123	Metazoan signal recognition particle RNA [Source:RFAM;Acc:RF00017]	1.386579193	0.005357571	0.018758856
ENSMMUG00000005622	dynein axonemal heavy chain 7 [Source:VGNC Symbol;Acc:VGNC:71859]	-1.291508944	7.68E-08	8.79E-07
ENSMMUG00000057432	serine/threonine kinase 17b [Source:NCBI gene;Acc:696908]	-0.931503309	0.00168696	0.006935281
ENSMMUG00000015970	coiled-coil domain containing 150 [Source:VGNC Symbol;Acc:VGNC:84222]	-1.410458178	9.92E-22	7.29E-20
ENSMMUG00000008643	general transcription factor IIIC subunit 3 [Source:VGNC Symbol;Acc:VGNC:73317]	-1.927227271	1.90E-17	9.16E-16
ENSMMUG00000022430	splicing factor 3b subunit 1 [Source:VGNC Symbol;Acc:VGNC:77179]	-1.322705284	0.000319291	0.001618735
ENSMMUG00000023810	methionyl-tRNA synthetase 2, mitochondrial [Source:VGNC Symbol;Acc:VGNC:74572]	-1.011684671	1.32E-07	1.44E-06
ENSMMUG00000023811	boule homolog, RNA binding protein [Source:VGNC Symbol;Acc:VGNC:70310]	-1.160441788	6.94E-06	5.25E-05
ENSMMUG00000012822	phospholipase C like 1 (inactive) [Source:NCBI gene;Acc:698200]	-0.704393288	0.002287142	0.009029447
ENSMMUG00000051936		-2.29236086	7.68E-07	7.14E-06
ENSMMUG00000050082		-0.700683562	0.011331427	0.035556188
ENSMMUG00000047974	aldehyde oxidase 4 [Source:NCBI gene;Acc:701502]	-1.034194373	1.03E-14	3.57E-13
ENSMMUG00000022202	basic leucine zipper and W2 domains 1 [Source:VGNC Symbol;Acc:VGNC:84213]	-1.105292805	2.10E-08	2.66E-07
ENSMMUG00000031296	caspase 8 [Source:VGNC Symbol;Acc:VGNC:70547]	-1.462168237	8.03E-12	1.83E-10
ENSMMUG00000018356	flagellum associated containing coiled-coil domains 1 [Source:VGNC Symbol;Acc:VGNC:72508]	-1.361612573	0.000888833	0.003978328
ENSMMUG00000018144	C2 calcium-dependent domain-containing protein 6 [Source:NCBI gene;Acc:702140]	1.936451111	2.02E-25	2.25E-23
ENSMMUG00000018151	membrane palmitoylated protein 4 [Source:VGNC Symbol;Acc:VGNC:74734]	2.668746143	1.72E-06	1.47E-05
ENSMMUG00000045285	frizzled class receptor 7 [Source:NCBI gene;Acc:703064]	-0.390211111	0.011124844	0.035000375
ENSMMUG00000000582	NOP58 ribonucleoprotein [Source:VGNC Symbol;Acc:VGNC:75284]	1.060914723	3.92E-07	3.89E-06
ENSMMUG00000061021		1.147053565	3.11E-05	0.00020109
ENSMMUG00000038427	Metazoan signal recognition particle RNA [Source:RFAM;Acc:RF00017]	2.65724997	0.009780373	0.03135353
ENSMMUG00000021152	family with sequence similarity 117 member B [Source:NCBI gene;Acc:705134]	-0.405391154	0.009715536	0.031183558
ENSMMUG00000056824		-1.85887295	0.000286918	0.001476494
ENSMMUG00000022601	cAMP responsive element binding protein 1 [Source:VGNC Symbol;Acc:VGNC:71485]	-0.764186435	0.000161421	0.000881468
ENSMMUG00000022602	methyltransferase like 21A [Source:VGNC Symbol;Acc:VGNC:74625]	-1.4990445	4.42E-09	6.37E-08
ENSMMUG00000020211	cyclin Y like 1 [Source:VGNC Symbol;Acc:VGNC:84224]	-1.770245639	2.96E-17	1.40E-15
ENSMMUG00000057651	frizzled class receptor 5 [Source:NCBI gene;Acc:710796]	-1.69162709	0.00192353	0.007794515
ENSMMUG00000008113	pleckstrin homology domain containing M3 [Source:NCBI gene;Acc:711125]	-2.18827996	4.30E-07	4.22E-06

ENSMMUG00000010871	crystallin gamma B [Source:VGNC Symbol;Acc:VGNC:71507]	-3.400850237	0.002299527	0.009070679
ENSMMUG00000016077	isocitrate dehydrogenase (NADP(+)) 1 [Source:NCBI gene;Acc:710019]	-2.843077656	5.08E-06	3.96E-05
ENSMMUG00000017524	phosphoinositide kinase, FYVE-type zinc finger containing [Source:VGNC Symbol;Acc:VGNC:75854]	-2.925073141	4.38E-05	0.000272648
ENSMMUG00000005295	parathyroid hormone 2 receptor [Source:VGNC Symbol;Acc:VGNC:76592]	-2.921942826	2.78E-05	0.000181099
ENSMMUG00000002809	unc-80 homolog, NALCN channel complex subunit [Source:NCBI gene;Acc:713007]	-1.551563487	6.06E-09	8.45E-08
ENSMMUG00000010508	carbamoyl-phosphate synthase 1 [Source:VGNC Symbol;Acc:VGNC:71471]	-3.185653518	4.76E-07	4.62E-06
ENSMMUG00000059604		0.501704424	0.009744372	0.031256241
ENSMMUG00000061342		1.691111577	2.29E-18	1.23E-16
ENSMMUG00000000381	IKAROS family zinc finger 2 [Source:VGNC Symbol;Acc:VGNC:73586]	0.865254018	2.87E-11	5.92E-10
ENSMMUG00000000382	sperm associated antigen 16 [Source:VGNC Symbol;Acc:VGNC:77977]	0.799266265	7.29E-05	0.000429672
ENSMMUG00000064880		-0.887592843	8.44E-08	9.59E-07
ENSMMUG00000053721		-2.005678216	2.10E-09	3.18E-08
ENSMMUG00000006758	cytochrome P450, family 27, subfamily A, polypeptide 1 [Source:NCBI gene;Acc:700192]	1.842539997	6.64E-05	0.000395412
ENSMMUG00000021877	protein kinase AMP-activated non-catalytic subunit gamma 3 [Source:VGNC Symbol;Acc:VGNC:76406]	2.02798389	6.46E-05	0.00038581
ENSMMUG00000052954	Wnt family member 6 [Source:NCBI gene;Acc:700573]	2.636763788	2.72E-07	2.77E-06
ENSMMUG00000017933	desmin [Source:VGNC Symbol;Acc:VGNC:71770]	0.415126788	0.001076618	0.004704711
ENSMMUG00000002519	serine/threonine kinase 11 interacting protein [Source:VGNC Symbol;Acc:VGNC:77963]	-0.989200504	4.32E-22	3.29E-20
ENSMMUG00000002520	solute carrier family 4 member 3 [Source:VGNC Symbol;Acc:VGNC:77604]	-0.916323424	2.58E-06	2.14E-05
ENSMMUG00000061834		-0.982783967	2.05E-05	0.000138707
ENSMMUG00000046282		-0.903862405	1.79E-11	3.84E-10
ENSMMUG00000051349		2.808822585	0.01395984	0.042390394
ENSMMUG00000018139	dedicator of cytokinesis 10 [Source:VGNC Symbol;Acc:VGNC:71888]	0.378525679	0.000276735	0.001430919
ENSMMUG00000051482		-2.631622567	9.87E-13	2.55E-11
ENSMMUG00000054072		0.640803393	5.37E-07	5.16E-06
ENSMMUG00000045544	neuronal tyrosine-phosphorylated phosphoinositide-3-kinase adaptor 2 [Source:NCBI gene;Acc:711209]	0.516123324	0.000315806	0.001604548
ENSMMUG00000013233	collagen type IV alpha 4 chain [Source:VGNC Symbol;Acc:VGNC:71299]	-0.672467462	0.0079333	0.026331044
ENSMMUG00000013235	collagen type IV alpha 3 chain [Source:VGNC Symbol;Acc:VGNC:81375]	-0.789502151	9.57E-05	0.000548595
ENSMMUG00000054830		-1.052842423	0.008284681	0.02734842
ENSMMUG00000002103	delta/notch like EGF repeat containing [Source:VGNC Symbol;Acc:VGNC:71895]	-1.434108609	2.35E-08	2.95E-07
ENSMMUG00000011777	UDP-GlcNAc:betaGal beta-1,3-N-acetylglucosaminyltransferase 7 [Source:VGNC Symbol;Acc:VGNC:82108]	2.922905004	0.000342513	0.001720912
ENSMMUG00000016871	alkaline phosphatase, intestinal [Source:VGNC Symbol;Acc:VGNC:69800]	-2.155081957	9.71E-06	7.07E-05
ENSMMUG00000051779		0.517959268	0.009598877	0.0308595
ENSMMUG00000005646	diacylglycerol kinase delta [Source:VGNC Symbol;Acc:VGNC:71783]	-0.456857454	0.000711796	0.003279079
ENSMMUG00000005650	ubiquitin specific peptidase 40 [Source:VGNC Symbol;Acc:VGNC:79226]	-0.320424894	0.005361059	0.018764628

ENSMMUG00000021216	UDP glucuronosyltransferase 1 family, polypeptide A1 [Source:NCBI gene;Acc:574210]	-0.915003887	4.90E-07	4.75E-06
ENSMMUG00000051807		1.422144675	2.44E-10	4.33E-09
ENSMMUG00000061244		1.095965284	0.006711031	0.022742577
ENSMMUG00000011516	ADP ribosylation factor like GTPase 4C [Source:VGNC Symbol;Acc:VGNC:70018]	2.291373758	0.000408241	0.0020101
ENSMMUG00000054449	Metazoan signal recognition particle RNA [Source:RFAM;Acc:RF00017]	1.219102378	0.002252251	0.008926884
ENSMMUG00000060487	atypical chemokine receptor 3 [Source:NCBI gene;Acc:694428]	-0.908012836	0.008405251	0.027674767
ENSMMUG00000014049	collagen type VI alpha 3 chain [Source:VGNC Symbol;Acc:VGNC:71303]	-1.142617027	0.002042948	0.008211022
ENSMMUG00000021723	beta-1,4-N-acetyl-galactosaminyltransferase 4 [Source:NCBI gene;Acc:114669831]	0.472834189	0.00138919	0.00586412
ENSMMUG00000059353	phosphatidylserine synthase 2 [Source:NCBI gene;Acc:698582]	-1.06879251	8.78E-11	1.69E-09
ENSMMUG00000056711	ribonuclease inhibitor-like [Source:NCBI gene;Acc:114672186]	-0.874536546	9.75E-11	1.86E-09
ENSMMUG00000062863		1.460768418	2.80E-09	4.13E-08
ENSMMUG00000004622	NLR family pyrin domain containing 6 [Source:VGNC Symbol;Acc:VGNC:75234]	-1.746331244	0.001387303	0.005859685
ENSMMUG00000057640	plakophilin-3-like [Source:NCBI gene;Acc:114669833]	2.520039895	5.32E-29	8.68E-27
ENSMMUG00000002917	single Ig IL-1-related receptor [Source:NCBI gene;Acc:100425060]	3.042149062	1.03E-15	4.04E-14
ENSMMUG00000010782	phosphatidylserine synthase 2 [Source:NCBI gene;Acc:114669774]	-0.542306782	0.010325816	0.032841668
ENSMMUG00000021661	leucine rich repeat containing 56 [Source:VGNC Symbol;Acc:VGNC:74356]	1.657774445	5.80E-09	8.14E-08
ENSMMUG00000021664	lamin tail domain containing 2 [Source:VGNC Symbol;Acc:VGNC:74380]	1.637600921	2.68E-25	2.93E-23
ENSMMUG00000004014	interferon regulatory factor 7 [Source:VGNC Symbol;Acc:VGNC:73765]	0.756520245	0.001923755	0.007794515
ENSMMUG00000004024	cadherin related family member 5 [Source:VGNC Symbol;Acc:VGNC:71158]	1.298810331	5.44E-06	4.22E-05
ENSMMUG00000016715	p53-induced death domain protein 1 [Source:VGNC Symbol;Acc:VGNC:81498]	0.335441423	0.01298958	0.039884581
ENSMMUG00000043846		0.605924127	0.000225341	0.001193789
ENSMMUG00000010544	mucin 5B, oligomeric mucus/gel-forming [Source:VGNC Symbol;Acc:VGNC:75081]	-0.525210227	8.53E-06	6.31E-05
ENSMMUG00000017450	MOB kinase activator 2 [Source:NCBI gene;Acc:720951]	2.046611607	1.61E-07	1.71E-06
ENSMMUG00000003874	interferon induced transmembrane protein 10 [Source:NCBI gene;Acc:721010]	4.031147968	1.02E-30	2.02E-28
ENSMMUG00000004773	transient receptor potential cation channel subfamily M member 5 [Source:VGNC Symbol;Acc:VGNC:78955]	-0.728779353	0.007527985	0.025158469
ENSMMUG00000000511	cysteinyl-tRNA synthetase 1 [Source:VGNC Symbol;Acc:VGNC:70540]	-0.702666453	0.014591023	0.043973761
ENSMMUG00000051131	MAS related GPR family member G [Source:NCBI gene;Acc:721253]	0.723854354	0.000655756	0.003050984
ENSMMUG00000019736	SH3 and multiple ankyrin repeat domains 2 [Source:NCBI gene;Acc:708192]	0.767661814	0.004930851	0.017522891
ENSMMUG00000052168		3.710581102	3.34E-54	4.69E-51
ENSMMUG00000060517		1.538984106	2.67E-07	2.72E-06
ENSMMUG00000014237	lysine methyltransferase 5B [Source:VGNC Symbol;Acc:VGNC:74110]	1.975432538	2.87E-42	1.55E-39
ENSMMUG00000056342	Metazoan signal recognition particle RNA [Source:RFAM;Acc:RF00017]	2.655579119	0.000860971	0.003872162
ENSMMUG00000023192	choline kinase alpha [Source:VGNC Symbol;Acc:VGNC:71039]	1.931991516	4.83E-09	6.89E-08
ENSMMUG00000023188	aldehyde dehydrogenase 3 family member B1 [Source:VGNC Symbol;Acc:VGNC:69789]	0.4622855	0.006667588	0.02261175
ENSMMUG00000056105	T-box transcription factor 10 [Source:NCBI gene;Acc:721689]	-0.402567867	0.003160306	0.011884413
ENSMMUG00000042611	nudix hydrolase 8 [Source:NCBI gene;Acc:721695]	-2.519808641	0.011662788	0.036441016
ENSMMUG00000062171		-2.015763183	0.002664509	0.010310269
ENSMMUG00000011821	phosphatidylinositol transfer protein membrane associated 1 [Source:VGNC Symbol;Acc:VGNC:76007]	-1.423041431	0.007916755	0.026282353
ENSMMUG00000011817	aryl hydrocarbon receptor interacting protein [Source:VGNC Symbol;Acc:VGNC:69759]	1.416425352	1.46E-16	6.33E-15
ENSMMUG00000053378		1.232080202	6.06E-08	7.05E-07
ENSMMUG00000005488	coronin 1B [Source:VGNC Symbol;Acc:VGNC:71320]	1.830418589	1.15E-18	6.36E-17

ENSMMUG00000005486	protein tyrosine phosphatase receptor type C associated protein [Source:VGNC Symbol;Acc:VGNC:76613]	1.113309583	2.40E-08	3.01E-07
ENSMMUG00000005483	ribosomal protein S6 kinase B2 [Source:VGNC Symbol;Acc:VGNC:76934]	1.001562535	2.22E-08	2.79E-07
ENSMMUG00000005480	carnosine synthase 1 [Source:VGNC Symbol;Acc:VGNC:81367]	0.982860804	2.45E-20	1.59E-18
ENSMMUG00000017280	TBC1 domain family member 10C [Source:VGNC Symbol;Acc:VGNC:78203]	1.41539076	1.64E-06	1.41E-05
ENSMMUG00000064122		0.624209109	0.001705495	0.007003268
ENSMMUG00000015925	G protein-coupled receptor kinase 2 [Source:VGNC Symbol;Acc:VGNC:73253]	0.61046215	0.002240865	0.008887251
ENSMMUG00000008771	lysine demethylase 2A [Source:VGNC Symbol;Acc:VGNC:73824]	0.524188174	0.000283297	0.001460003
ENSMMUG00000062635		-0.39936787	0.003817061	0.014037936
ENSMMUG00000020359	spectrin beta, non-erythrocytic 2 [Source:VGNC Symbol;Acc:VGNC:77872]	-0.556287049	0.016357779	0.048271283
ENSMMUG00000010577	RNA-binding protein 4B [Source:NCBI gene;Acc:713553]	0.641045089	7.71E-06	5.77E-05
ENSMMUG00000002494	actinin alpha 3 [Source:NCBI gene;Acc:713811]	-0.597598462	0.005648043	0.019604581
ENSMMUG00000015991	BRMS1 transcriptional repressor and anoikis regulator [Source:VGNC Symbol;Acc:VGNC:70334]	-0.941043942	0.012417874	0.038330585
ENSMMUG00000015987	Ras and Rab interactor 1 [Source:VGNC Symbol;Acc:VGNC:76769]	-0.6397033	0.000608459	0.002873838
ENSMMUG00000006950	cornichon family AMPA receptor auxiliary protein 2 [Source:VGNC Symbol;Acc:VGNC:71366]	-1.203160453	0.000763078	0.003493519
ENSMMUG00000031202	RAB1B, member RAS oncogene family [Source:NCBI gene;Acc:721852]	-1.015754657	2.77E-06	2.27E-05
ENSMMUG00000022520	kinesin light chain 2 [Source:VGNC Symbol;Acc:VGNC:73869]	-0.721919413	0.002479464	0.009695909
ENSMMUG00000057179	cation channel sperm associated 1 [Source:NCBI gene;Acc:721869]	3.849344471	1.28E-22	1.04E-20
ENSMMUG00000056794	cystatin E/M [Source:NCBI gene;Acc:714999]	3.33578042	0.000303015	0.001547406
ENSMMUG00000019115	eukaryotic translation initiation factor 1A domain containing [Source:VGNC Symbol;Acc:VGNC:72127]	0.590647195	0.000146768	0.000809012
ENSMMUG00000008652	coiled-coil domain containing 85B [Source:VGNC Symbol;Acc:VGNC:70853]	0.935454548	6.12E-06	4.70E-05
ENSMMUG00000008651	FGF1 intracellular binding protein [Source:VGNC Symbol;Acc:VGNC:72663]	0.561047604	0.00135034	0.005717336
ENSMMUG00000038363	adaptor related protein complex 5 subunit beta 1 [Source:NCBI gene;Acc:721904]	1.523589358	9.12E-22	6.77E-20
ENSMMUG00000050028		1.334004301	5.83E-10	9.64E-09
ENSMMUG00000064005		1.402044333	8.24E-06	6.11E-05
ENSMMUG00000060437	Metazoan signal recognition particle RNA [Source:RFAM;Acc:RF00017]	1.702412838	4.64E-06	3.65E-05
ENSMMUG00000019837	ribonuclease H2 subunit C [Source:NCBI gene;Acc:700330]	0.666621003	0.00107447	0.004701183
ENSMMUG00000019839	lysine acetyltransferase 5 [Source:VGNC Symbol;Acc:VGNC:73955]	0.944033369	2.53E-08	3.17E-07
ENSMMUG00000007418	RELA proto-oncogene, NF-kB subunit [Source:VGNC Symbol;Acc:VGNC:76893]	0.353455599	0.001872336	0.007610369
ENSMMUG00000007416	signal-induced proliferation-associated 1 [Source:VGNC Symbol;Acc:VGNC:77218]	0.585863706	0.002671051	0.01032988
ENSMMUG00000007406	pecanex 3 [Source:NCBI gene;Acc:716000]	-0.299512276	0.007374094	0.024720755
ENSMMUG00000050788	mitogen-activated protein kinase kinase kinase 11 [Source:NCBI gene;Acc:716038]	-0.352885801	0.002168861	0.008655075
ENSMMUG00000023479	latent transforming growth factor beta binding protein 3 [Source:VGNC Symbol;Acc:VGNC:74458]	1.824404233	1.08E-25	1.27E-23
ENSMMUG00000023477		1.670764705	8.14E-34	2.08E-31
ENSMMUG00000052424		0.985499882	1.08E-06	9.71E-06
ENSMMUG00000023300	FERM domain containing 8 [Source:NCBI gene;Acc:716379]	1.592768052	4.83E-06	3.79E-05
ENSMMUG00000023297	solute carrier family 25 member 45 [Source:VGNC Symbol;Acc:VGNC:77576]	1.444035389	0.000637665	0.002975871
ENSMMUG00000056343		1.059269212	6.91E-05	0.000409852
ENSMMUG00000012316	CDC42 effector protein 2 [Source:VGNC Symbol;Acc:VGNC:70750]	2.390313098	0.000511707	0.002462552
ENSMMUG00000022031	DNA polymerase alpha 2, accessory subunit [Source:NCBI gene;Acc:716541]	0.863200966	1.64E-06	1.41E-05
ENSMMUG00000013475	calpain 1 [Source:VGNC Symbol;Acc:VGNC:70529]	-0.719675735	8.11E-06	6.03E-05
ENSMMUG00000018767	cell division cycle associated 5 [Source:NCBI gene;Acc:721998]	2.531950971	0.00482959	0.017228334
ENSMMUG00000006568	basic leucine zipper ATF-like transcription factor 2 [Source:VGNC Symbol;Acc:VGNC:70127]	2.064180749	2.75E-18	1.47E-16
ENSMMUG00000064306	Metazoan signal recognition particle RNA [Source:RFAM;Acc:RF00017]	1.043826698	0.011034539	0.034780252
ENSMMUG00000031163	membrane anchored junction protein [Source:VGNC Symbol;Acc:VGNC:74375]	1.730071292	2.40E-24	2.32E-22

ENSMMUG00000003976	glycoprotein hormone subunit alpha 2 [Source:VGNC Symbol;Acc:VGNC:72961]	1.515646706	1.81E-12	4.52E-11
ENSMMUG00000041101	protein phosphatase 2 regulatory subunit B'beta [Source:NCBI gene;Acc:722026]	1.660037622	1.44E-23	1.30E-21
ENSMMUG00000003973	autophagy related 2A [Source:VGNC Symbol;Acc:VGNC:70145]	-1.220607833	5.05E-07	4.87E-06
ENSMMUG00000003972	EH domain containing 1 [Source:VGNC Symbol;Acc:VGNC:72175]	0.882224022	4.69E-09	6.72E-08
ENSMMUG00000031160	menin 1 [Source:NCBI gene;Acc:106995582]	0.538996415	6.73E-09	9.32E-08
ENSMMUG00000030597	mitogen-activated protein kinase kinase kinase kinase 2 [Source:VGNC Symbol;Acc:VGNC:81465]	0.644807379	2.59E-10	4.57E-09
ENSMMUG00000007610	ribosomal protein S6 kinase A4 [Source:VGNC Symbol;Acc:VGNC:76930]	0.975062909	1.32E-08	1.73E-07
ENSMMUG00000007603	coiled-coil domain containing 88B [Source:NCBI gene;Acc:722080]	1.00136901	3.38E-07	3.39E-06
ENSMMUG00000013397	peroxiredoxin 5 [Source:NCBI gene;Acc:719764]	0.980646359	3.43E-12	8.20E-11
ENSMMUG00000060596	BCL2 associated agonist of cell death [Source:NCBI gene;Acc:717740]	1.041803353	2.17E-08	2.75E-07
ENSMMUG00000012948	phospholipase C beta 3 [Source:VGNC Symbol;Acc:VGNC:76165]	-0.541650004	0.001668561	0.006873744
ENSMMUG00000012944	vascular endothelial growth factor B [Source:VGNC Symbol;Acc:VGNC:78765]	1.937519112	1.08E-28	1.68E-26
ENSMMUG00000012941	nudix hydrolase 22 [Source:VGNC Symbol;Acc:VGNC:75475]	2.007051541	1.30E-35	3.95E-33
ENSMMUG00000012940	tRNA phosphotransferase 1 [Source:VGNC Symbol;Acc:VGNC:79242]	0.596083367	0.002280219	0.009004651
ENSMMUG00000012939	fermitin family member 3 [Source:VGNC Symbol;Acc:VGNC:72553]	-0.396659008	0.016290936	0.048134057
ENSMMUG00000012953	stress induced phosphoprotein 1 [Source:NCBI gene;Acc:717926]	-0.778661187	2.25E-06	1.88E-05
ENSMMUG00000052569		3.922159785	5.77E-18	2.91E-16
ENSMMUG00000014131	microtubule affinity regulating kinase 2 [Source:VGNC Symbol;Acc:VGNC:74634]	1.596778181	5.00E-06	3.91E-05
ENSMMUG00000059969		1.217270802	0.000117247	0.000659245
ENSMMUG00000018152	spindlin interactor and repressor of chromatin binding [Source:VGNC Symbol;Acc:VGNC:77987]	0.522778819	0.002384249	0.009373233
ENSMMUG00000005776	atlastin GTPase 3 [Source:VGNC Symbol;Acc:VGNC:70125]	0.759396935	6.27E-05	0.000376266
ENSMMUG00000005775	phospholipase A and acyltransferase 3 [Source:VGNC Symbol;Acc:VGNC:76055]	0.372830013	0.000156357	0.000857821
ENSMMUG00000056054	zinc finger and BTB domain containing 3 [Source:NCBI gene;Acc:722240]	-1.228828156	4.19E-18	2.17E-16
ENSMMUG00000005761	UBX domain protein 1 [Source:VGNC Symbol;Acc:VGNC:79135]	-0.534732595	0.00896345	0.029197319
ENSMMUG00000013949	chromosome 14 C11orf98 homolog [Source:NCBI gene;Acc:100429527]	-0.784575393	0.00664203	0.022557807
ENSMMUG00000006598	eukaryotic translation elongation factor 1 gamma [Source:NCBI gene;Acc:722274]	-0.210669867	0.008394387	0.027651983
ENSMMUG00000031123	secretoglobin family 1A member 1 [Source:VGNC Symbol;Acc:VGNC:77004]	-0.593948929	5.69E-05	0.000345514
ENSMMUG00000049661	Metazoan signal recognition particle RNA [Source:RFAM;Acc:RF00017]	-1.526400833	4.64E-10	7.80E-09
ENSMMUG00000000327	fatty acid desaturase 2 [Source:VGNC Symbol;Acc:VGNC:72472]	1.46388448	1.24E-14	4.27E-13
ENSMMUG00000013798	fatty acid desaturase 1 [Source:VGNC Symbol;Acc:VGNC:72471]	3.893024512	2.69E-36	8.98E-34
ENSMMUG00000006655	myelin regulatory factor [Source:VGNC Symbol;Acc:VGNC:74949]	3.754325992	1.01E-12	2.61E-11
ENSMMUG00000015280	cleavage and polyadenylation specific factor 7 [Source:VGNC Symbol;Acc:VGNC:71606]	2.099368299	0.001266221	0.005416425
ENSMMUG00000022372	cytochrome b561 family member A3 [Source:VGNC Symbol;Acc:VGNC:71458]	-1.664289448	4.05E-05	0.000254429
ENSMMUG00000042208	pepsin A-4 [Source:NCBI gene;Acc:694646]	-1.357324089	4.77E-10	8.02E-09
ENSMMUG00000015018	CD5 molecule [Source:VGNC Symbol;Acc:VGNC:70770]	-1.599555738	0.000650974	0.003031751
ENSMMUG00000044417	CD6 molecule [Source:NCBI gene;Acc:695183]	-1.855071764	0.002754744	0.010606746
ENSMMUG00000014530	pre-mRNA processing factor 19 [Source:VGNC Symbol;Acc:VGNC:76281]	1.61824995	4.59E-11	9.14E-10
ENSMMUG00000052454	prostaglandin D2 receptor 2 [Source:NCBI gene;Acc:695853]	-0.84269335	0.005892836	0.020323474
ENSMMUG00000031100	membrane-spanning 4-domains subfamily A member 4A-like [Source:NCBI gene;Acc:698152]	2.065255556	0.006091662	0.020921719

ENSMMUG00000004821	membrane spanning 4-domains A14 [Source:NCBI gene;Acc:697083]	-5.042639851	2.38E-15	8.92E-14
ENSMMUG00000020177	membrane spanning 4-domains A4A [Source:NCBI gene;Acc:698027]	2.223862285	2.43E-05	0.000161224
ENSMMUG00000017287	PAT1 homolog 1, processing body mRNA decay factor [Source:VGNC Symbol;Acc:VGNC:75684]	1.769320238	2.18E-09	3.30E-08
ENSMMUG00000001745	oxysterol binding protein [Source:VGNC Symbol;Acc:VGNC:75718]	1.015128033	2.64E-08	3.28E-07
ENSMMUG00000060712		0.851178997	1.99E-08	2.54E-07
ENSMMUG00000045775	macrophage expressed 1 [Source:NCBI gene;Acc:700274]	-2.771332645	0.000929808	0.004130093
ENSMMUG00000064798		-1.747466509	0.006206568	0.021248705
ENSMMUG00000001083	glycine N-acyltransferase-like protein 1 [Source:NCBI gene;Acc:701437]	0.537938339	0.000142564	0.000788629
ENSMMUG00000011713	leupaxin [Source:VGNC Symbol;Acc:VGNC:74286]	-0.41179325	0.000286334	0.001474032
ENSMMUG00000054737		-0.659134442	3.81E-08	4.60E-07
ENSMMUG00000059848	olfactory receptor 5B2 [Source:NCBI gene;Acc:100429465]	-0.646217403	0.00787604	0.02617197
ENSMMUG00000050844	BTB domain containing 18 [Source:NCBI gene;Acc:703264]	1.311804208	0.004972653	0.017644612
ENSMMUG00000056184		2.97727657	1.15E-08	1.53E-07
ENSMMUG00000002566	selenoprotein H [Source:VGNC Symbol;Acc:VGNC:82234]	2.064804631	0.004440747	0.016024858
ENSMMUG00000040741	thioredoxin related transmembrane protein 2 [Source:NCBI gene;Acc:100169970]	1.300154106	1.41E-06	1.23E-05
ENSMMUG00000002563	zinc finger DHHC-type palmitoyltransferase 5 [Source:VGNC Symbol;Acc:VGNC:79145]	1.683543578	0.000822641	0.00372968
ENSMMUG00000058453	cleavage factor polyribonucleotide kinase subunit 1 [Source:NCBI gene;Acc:705071]	-0.624677016	0.008704247	0.028518611
ENSMMUG00000010601	solute carrier family 43 member 3 [Source:NCBI gene;Acc:706267]	-1.462791506	1.57E-06	1.35E-05
ENSMMUG00000017810	structure specific recognition protein 1 [Source:VGNC Symbol;Acc:VGNC:77891]	1.097196973	2.58E-06	2.14E-05
ENSMMUG00000010627	apelin receptor [Source:VGNC Symbol;Acc:VGNC:69939]	-0.946411703	5.00E-06	3.91E-05
ENSMMUG00000010869	nucleoporin 160 [Source:VGNC Symbol;Acc:VGNC:75429]	0.896591077	0.01170805	0.036525468
ENSMMUG00000040099	Y RNA [Source:RFAM;Acc:RF00019]	1.660372313	0.000707754	0.003262603
ENSMMUG00000058885	formin binding protein 4 [Source:NCBI gene;Acc:712633]	1.141716221	3.44E-08	4.20E-07
ENSMMUG00000012881	ATP/GTP binding protein like 2 [Source:NCBI gene;Acc:711126]	-2.360891631	0.007857504	0.026116562
ENSMMUG00000043682	mitochondrial carrier 2 [Source:NCBI gene;Acc:712767]	-4.770978691	2.89E-08	3.57E-07
ENSMMUG00000011657	CUGBP Elav-like family member 1 [Source:VGNC Symbol;Acc:VGNC:71098]	-0.50622778	7.71E-05	0.000452354
ENSMMUG00000015607	solute carrier family 39 member 13 [Source:VGNC Symbol;Acc:VGNC:77538]	-0.842989358	0.008678259	0.028453408
ENSMMUG00000015606	Spi-1 proto-oncogene [Source:NCBI gene;Acc:712010]	0.786370271	0.014600261	0.043992148
ENSMMUG00000062208	Metazoan signal recognition particle RNA [Source:RFAM;Acc:RF00017]	1.065395965	0.004122114	0.015029746
ENSMMUG00000015604	myosin binding protein C3 [Source:VGNC Symbol;Acc:VGNC:75094]	-0.943893271	6.40E-07	6.05E-06
ENSMMUG00000022964		-0.256262276	0.002260681	0.00894765
ENSMMUG00000060750	Metazoan signal recognition particle RNA [Source:RFAM;Acc:RF00017]	1.984784258	0.00304239	0.011536895
ENSMMUG00000003631	cytoskeleton associated protein 5 [Source:VGNC Symbol;Acc:VGNC:71368]	-1.263428323	0.001410657	0.00594579
ENSMMUG00000021446	diacylglycerol kinase zeta [Source:VGNC Symbol;Acc:VGNC:71789]	0.889471982	2.89E-11	5.95E-10
ENSMMUG00000002452	solute carrier family 35 member C1 [Source:VGNC Symbol;Acc:VGNC:77524]	0.975774787	2.70E-07	2.76E-06
ENSMMUG00000049305	Metazoan signal recognition particle RNA [Source:RFAM;Acc:RF00017]	1.105946547	0.000398989	0.001971466
ENSMMUG00000047219		1.011068884	1.03E-07	1.15E-06
ENSMMUG00000000874	carbohydrate sulfotransferase 1 [Source:VGNC Symbol;Acc:VGNC:71055]	0.752512913	0.000203915	0.001089997
ENSMMUG00000050952		-1.214359451	0.000371277	0.001842329

ENSMMUG00000059728	tumor protein p53 inducible protein 11 [Source:NCBI gene;Acc:715295]	-1.239741646	0.00532208	0.018657883
ENSMMUG00000049355	ALX homeobox 4 [Source:NCBI gene;Acc:715652]	1.521159473	0.001477839	0.006180655
ENSMMUG00000008827	apoptosis inhibitor 5 [Source:NCBI gene;Acc:716346]	-0.827190921	0.00524319	0.018432001
ENSMMUG00000049945		-0.558428364	0.002705664	0.010446456
ENSMMUG00000053160		-0.329091979	0.002077484	0.008333111
ENSMMUG00000051583		0.863949622	0.004050126	0.014790345
ENSMMUG00000054427	Metazoan signal recognition particle RNA [Source:RFAM;Acc:RF00017]	0.972812564	0.015544025	0.04633798
ENSMMUG00000004332	tripartite motif containing 44 [Source:VGNC Symbol;Acc:VGNC:79168]	2.754312363	9.50E-16	3.74E-14
ENSMMUG00000004330	peptidase domain containing associated with muscle regeneration 1 [Source:VGNC Symbol;Acc:VGNC:75673]	3.218207661	8.09E-07	7.47E-06
ENSMMUG00000004329	solute carrier family 1 member 2 [Source:VGNC Symbol;Acc:VGNC:77410]	-0.808434957	4.38E-05	0.000272648
ENSMMUG00000004327	CD44 molecule (Indian blood group) [Source:NCBI gene;Acc:717204]	-0.78099319	0.008797192	0.028755951
ENSMMUG00000002432	E74 like ETS transcription factor 5 [Source:VGNC Symbol;Acc:VGNC:72209]	-0.585403834	0.013702361	0.041740146
ENSMMUG00000002429	catalase [Source:VGNC Symbol;Acc:VGNC:70552]	-0.512704951	0.00016405	0.000894427
ENSMMUG00000017218	KIAA1549 like [Source:VGNC Symbol;Acc:VGNC:73902]	-0.710890047	6.33E-05	0.000379522
ENSMMUG00000001536	glutamine and serine rich 1 [Source:VGNC Symbol;Acc:VGNC:76544]	0.987169443	9.11E-12	2.06E-10
ENSMMUG00000055352		1.175750923	4.85E-14	1.55E-12
ENSMMUG00000049850	proline rich and Gla domain 4 [Source:NCBI gene;Acc:694707]	1.043117923	1.39E-11	3.03E-10
ENSMMUG00000065337		-0.6477491	5.01E-06	3.92E-05
ENSMMUG00000050093		-0.554932239	0.001895802	0.007696823
ENSMMUG00000006842	doublecortin domain containing 1 [Source:NCBI gene;Acc:696721]	-0.93838143	8.81E-08	9.98E-07
ENSMMUG00000023119	lin-7 homolog C, crumbs cell polarity complex component [Source:VGNC Symbol;Acc:VGNC:74070]	-1.233975051	0.011044393	0.034803487
ENSMMUG00000031024	lactate dehydrogenase A [Source:NCBI gene;Acc:694309]	0.76860493	0.000360133	0.001793377
ENSMMUG00000006403	potassium voltage-gated channel subfamily C member 1 [Source:VGNC Symbol;Acc:VGNC:73971]	0.449016967	0.002647219	0.010254685
ENSMMUG00000005895	nucleobindin 2 [Source:VGNC Symbol;Acc:VGNC:75549]	-1.410712711	1.33E-06	1.16E-05
ENSMMUG00000056603		-1.154460637	0.013526533	0.041307181
ENSMMUG00000064558		-1.133450504	9.63E-05	0.000551214
ENSMMUG00000017628	pleckstrin homology domain containing A7 [Source:VGNC Symbol;Acc:VGNC:76121]	-2.602649698	5.33E-05	0.000325617
ENSMMUG00000055212		-3.134015997	0.001104292	0.004807664
ENSMMUG00000054213	parathyroid hormone [Source:NCBI gene;Acc:700195]	-1.103057908	0.011683004	0.036494251
ENSMMUG00000009695	BTB domain containing 10 [Source:VGNC Symbol;Acc:VGNC:70361]	-0.959636172	7.66E-05	0.000449676
ENSMMUG00000010875	TEA domain transcription factor 1 [Source:VGNC Symbol;Acc:VGNC:78301]	0.328035159	0.008812904	0.028781704
ENSMMUG00000020956	parvin alpha [Source:VGNC Symbol;Acc:VGNC:75605]	0.711776791	0.000638619	0.00297916
ENSMMUG00000043504		-1.006694778	9.39E-10	1.50E-08
ENSMMUG00000009205	SET binding factor 2 [Source:VGNC Symbol;Acc:VGNC:77075]	-0.392063936	0.01070227	0.03386235
ENSMMUG00000008758	zinc finger protein 143 [Source:VGNC Symbol;Acc:VGNC:81612]	1.606556334	6.84E-06	5.18E-05
ENSMMUG00000023636	importin 7 [Source:NCBI gene;Acc:704213]	1.47773316	3.22E-09	4.72E-08

ENSMMUG00000005639	transmembrane protein 41B [Source:VGNC Symbol;Acc:VGNC:79754]	0.955112037	0.002695413	0.010409743
ENSMMUG00000021142	DENN domain containing 5A [Source:VGNC Symbol;Acc:VGNC:71674]	0.537194437	0.016330378	0.048210713
ENSMMUG00000006692	signal peptide, CUB domain and EGF like domain containing 2 [Source:VGNC Symbol;Acc:VGNC:77138]	1.278950339	4.26E-11	8.54E-10
ENSMMUG00000006689	nuclear receptor interacting protein 3 [Source:VGNC Symbol;Acc:VGNC:75397]	1.291426954	1.28E-05	9.09E-05
ENSMMUG00000038288	TMEM9 domain family member B [Source:NCBI gene;Acc:708447]	-1.271153397	1.30E-13	3.78E-12
ENSMMUG00000051468	Metazoan signal recognition particle RNA [Source:RFAM;Acc:RF00017]	-1.192844125	2.15E-11	4.56E-10
ENSMMUG00000001997	achaete-scute family bHLH transcription factor 3 [Source:VGNC Symbol;Acc:VGNC:70031]	-0.995372048	0.00681989	0.023044655
ENSMMUG00000001995	chromosome 14 C11orf16 homolog [Source:NCBI gene;Acc:705319]	-1.112094414	4.56E-06	3.60E-05
ENSMMUG00000021827	tripartite motif containing 66 [Source:VGNC Symbol;Acc:VGNC:79766]	-0.370634162	0.006398993	0.021827554
ENSMMUG00000018471	serine/threonine kinase 33 [Source:VGNC Symbol;Acc:VGNC:78118]	1.543465413	0.000281081	0.001450181
ENSMMUG00000015016	RIC3 acetylcholine receptor chaperone [Source:VGNC Symbol;Acc:VGNC:76872]	-0.642606627	0.006047617	0.020801013
ENSMMUG00000035632	U6 spliceosomal RNA [Source:RFAM;Acc:RF00026]	-0.837306735	0.000861913	0.003873913
ENSMMUG00000049802	olfactory receptor 51G1 [Source:NCBI gene;Acc:716561]	1.139134857	5.21E-05	0.000319169
ENSMMUG00000022682	tripartite motif containing 68 [Source:NCBI gene;Acc:717644]	0.423091802	0.000200904	0.001074888
ENSMMUG00000063820	olfactory receptor, family 52, subfamily M, member 1 [Source:NCBI gene;Acc:717723]	2.771120871	0.010859227	0.034281618
ENSMMUG00000004974	tripartite motif containing 21 [Source:VGNC Symbol;Acc:VGNC:78556]	-0.708885669	0.01258204	0.038786095
ENSMMUG00000003955	post-GPI attachment to proteins 2 [Source:VGNC Symbol;Acc:VGNC:75823]	1.55540674	1.31E-24	1.33E-22
ENSMMUG00000003950	nucleoporin 98 and 96 precursor [Source:VGNC Symbol;Acc:VGNC:75563]	1.432039785	1.42E-27	2.01E-25
ENSMMUG00000003949	cholinergic receptor nicotinic alpha 10 subunit [Source:NCBI gene;Acc:718133]	-0.670905575	0.002823415	0.010841388
ENSMMUG00000003948	ADP-ribosyltransferase 1 [Source:VGNC Symbol;Acc:VGNC:70024]	-1.379147828	0.0020771	0.008333111
ENSMMUG00000003946	ADP-ribosyltransferase 5 [Source:VGNC Symbol;Acc:VGNC:69890]	-1.59359656	0.000660401	0.003069542
ENSMMUG00000007325	Xrcc1 N-terminal domain containing 1 [Source:NCBI gene;Acc:115945140]	-1.925654194	1.13E-09	1.78E-08
ENSMMUG00000059115		-1.739381446	3.83E-06	3.08E-05
ENSMMUG00000007326	ring finger protein 121 [Source:VGNC Symbol;Acc:VGNC:76788]	-1.670578214	1.17E-09	1.84E-08
ENSMMUG00000007330	nuclear mitotic apparatus protein 1 [Source:VGNC Symbol;Acc:VGNC:75556]	-1.876979342	2.22E-25	2.45E-23
ENSMMUG00000021280	leucine rich transmembrane and O-methyltransferase domain containing [Source:NCBI gene;Acc:718286]	-1.589982197	2.11E-10	3.78E-09
ENSMMUG00000021282	late endosomal/lysosomal adaptor, MAPK and MTOR activator 1 [Source:VGNC Symbol;Acc:VGNC:74129]	-1.628300133	7.14E-10	1.17E-08
ENSMMUG00000010453	folate receptor beta [Source:VGNC Symbol;Acc:VGNC:72690]	-1.772026588	7.45E-17	3.35E-15
ENSMMUG00000010451	inositol polyphosphate phosphatase like 1 [Source:VGNC Symbol;Acc:VGNC:73752]	2.136139332	4.34E-05	0.000270761
ENSMMUG00000005423	caseinolytic mitochondrial matrix peptidase chaperone subunit B [Source:VGNC Symbol;Acc:VGNC:71375]	0.602034881	0.006407579	0.021850512
ENSMMUG00000015054	ArfGAP with RhoGAP domain, ankyrin repeat and PH domain 1 [Source:VGNC Symbol;Acc:VGNC:69844]	0.453985676	0.006266367	0.021437733
ENSMMUG00000001294	FCH and double SH3 domains 2 [Source:VGNC Symbol;Acc:VGNC:72593]	-1.151050858	7.92E-05	0.000463192
ENSMMUG00000004791	pleckstrin homology domain containing B1 [Source:VGNC Symbol;Acc:VGNC:76069]	-1.052764881	0.001375688	0.005814125
ENSMMUG00000030964	RAB6A, member RAS oncogene family [Source:NCBI gene;Acc:718717]	1.264702738	1.13E-24	1.17E-22
ENSMMUG00000010321	C2 domain containing 3 centriole elongation regulator [Source:VGNC Symbol;Acc:VGNC:70375]	-1.007459434	5.58E-09	7.85E-08

ENSMMUG00000017402	phosphoglucomutase 2 like 1 [Source:VGNC Symbol;Acc:VGNC:75839]	0.909770986	2.24E-06	1.87E-05
ENSMMUG00000056316		0.645491213	0.001125585	0.004883686
ENSMMUG00000013094	lipoyl(octanoyl) transferase 2 [Source:NCBI gene;Acc:718425]	-1.09677607	1.61E-13	4.63E-12
ENSMMUG00000013093	uncharacterized LOC106993385 [Source:NCBI gene;Acc:106993385]	-1.166156696	9.52E-17	4.22E-15
ENSMMUG00000021039	X-ray radiation resistance associated 1 [Source:NCBI gene;Acc:693788]	2.178520329	7.69E-17	3.42E-15
ENSMMUG00000064190		-0.820288362	0.009317999	0.030149122
ENSMMUG00000009126	solute carrier organic anion transporter family member 2B1 [Source:VGNC Symbol;Acc:VGNC:77603]	-0.74991242	4.47E-05	0.000277545
ENSMMUG00000058539		-0.851715525	0.005218331	0.018353813
ENSMMUG00000047594		-0.965153978	3.40E-07	3.41E-06
ENSMMUG00000017529	arrestin beta 1 [Source:VGNC Symbol;Acc:VGNC:70021]	-0.663407053	0.000881978	0.003953956
ENSMMUG00000000187	UV radiation resistance associated [Source:VGNC Symbol;Acc:VGNC:78751]	0.642666103	0.007990866	0.026497023
ENSMMUG00000022401	EMSY transcriptional repressor, BRCA2 interacting [Source:VGNC Symbol;Acc:VGNC:72065]	2.323911619	6.13E-25	6.52E-23
ENSMMUG00000062043		1.906311476	0.002257316	0.008936856
ENSMMUG00000050318	UDP-GlcNAc:betaGal beta-1,3-N-acetylglucosaminyltransferase 6 [Source:NCBI gene;Acc:697946]	-0.474614839	0.003518321	0.013059138
ENSMMUG00000018188	glycerophosphodiester phosphodiesterase domain containing 4 [Source:VGNC Symbol;Acc:VGNC:72777]	-0.833195108	0.011967335	0.037210187
ENSMMUG00000001387	p21 (RAC1) activated kinase 1 [Source:NCBI gene;Acc:698585]	1.803334695	1.91E-23	1.68E-21
ENSMMUG00000018945	chloride nucleotide-sensitive channel 1A [Source:VGNC Symbol;Acc:VGNC:71261]	-0.941483233	0.004285873	0.015570154
ENSMMUG00000017355	remodeling and spacing factor 1 [Source:VGNC Symbol;Acc:VGNC:77044]	-0.984054689	2.10E-05	0.000141564
ENSMMUG00000051202		-1.792640731	0.013427394	0.041049128
ENSMMUG00000060971		-0.842716115	0.002269133	0.008970977
ENSMMUG00000051769		-0.802885042	0.000224257	0.001188946
ENSMMUG00000050245		-1.920458087	1.07E-05	7.72E-05
ENSMMUG00000050119		0.684109587	1.24E-06	1.10E-05
ENSMMUG00000016540	transmembrane protein 126B [Source:VGNC Symbol;Acc:VGNC:79893]	-0.516147881	0.001329925	0.005645537
ENSMMUG00000016543	CREB/ATF bZIP transcription factor [Source:VGNC Symbol;Acc:VGNC:71538]	-0.884996667	0.001236677	0.005311462
ENSMMUG00000012449	synaptotagmin like 2 [Source:VGNC Symbol;Acc:VGNC:78123]	-1.520363554	2.70E-05	0.000176422
ENSMMUG00000013045	coiled-coil domain containing 81 [Source:VGNC Symbol;Acc:VGNC:70868]	-0.892083284	0.007512114	0.025111413
ENSMMUG00000013055	malic enzyme 3 [Source:VGNC Symbol;Acc:VGNC:74695]	-0.932152009	0.000628722	0.002946714
ENSMMUG00000011116	NADPH oxidase 4 [Source:VGNC Symbol;Acc:VGNC:75376]	-1.360487318	9.96E-09	1.33E-07
ENSMMUG00000060452	FAT atypical cadherin 3 [Source:NCBI gene;Acc:695935]	-0.601685405	0.011922745	0.037087989
ENSMMUG00000057627		-1.029580328	0.000220612	0.001170948
ENSMMUG00000013601	chromosome 14 C11orf54 homolog [Source:NCBI gene;Acc:696483]	-0.910360142	1.18E-07	1.30E-06
ENSMMUG00000061600	V-set and transmembrane domain containing 5 [Source:NCBI gene;Acc:697779]	-0.883270019	0.001548186	0.006423138

ENSMMUG00000004112	MRE11 homolog, double strand break repair nuclease [Source:VGNC Symbol;Acc:VGNC:74926]	-0.384699645	0.006996361	0.023601148
ENSMMUG00000061391	ankyrin repeat domain 49 [Source:NCBI gene;Acc:697693]	1.260770939	7.30E-05	0.000430116
ENSMMUG00000062781	chromosome 14 C11orf97 homolog [Source:NCBI gene;Acc:106993442]	2.291297521	7.55E-40	3.42E-37
ENSMMUG00000008965	piwi like RNA-mediated gene silencing 4 [Source:VGNC Symbol;Acc:VGNC:81504]	-1.199235001	1.19E-16	5.21E-15
ENSMMUG00000009394	sestrin 3 [Source:VGNC Symbol;Acc:VGNC:77291]	-0.67188894	6.26E-05	0.000376045
ENSMMUG00000061815		-0.657864748	8.65E-05	0.000500896
ENSMMUG00000016737	contactin 5 [Source:VGNC Symbol;Acc:VGNC:71287]	3.733798006	6.84E-06	5.18E-05
ENSMMUG00000020784	centrosomal protein 126 [Source:NCBI gene;Acc:701911]	-1.331457274	2.51E-17	1.19E-15
ENSMMUG00000003394	cilia and flagella associated protein 300 [Source:VGNC Symbol;Acc:VGNC:71120]	-1.495160395	3.71E-19	2.11E-17
ENSMMUG00000050759	Metazoan signal recognition particle RNA [Source:RFAM;Acc:RF00017]	-1.585347613	0.004450112	0.016049173
ENSMMUG00000057934		2.031744583	3.40E-13	9.34E-12
ENSMMUG00000052526	RAB39A, member RAS oncogene family [Source:NCBI gene;Acc:707451]	-1.556435743	1.02E-32	2.43E-30
ENSMMUG00000001800	cullin 5 [Source:VGNC Symbol;Acc:VGNC:71570]	-1.029406365	0.000623878	0.002929887
ENSMMUG00000004157	nuclear protein, coactivator of histone transcription [Source:VGNC Symbol;Acc:VGNC:75379]	-1.872085949	1.44E-14	4.93E-13
ENSMMUG00000004153	ATM serine/threonine kinase [Source:VGNC Symbol;Acc:VGNC:70173]	-1.343123643	0.00118568	0.005115937
ENSMMUG00000000797	exophilin 5 [Source:VGNC Symbol;Acc:VGNC:72412]	-1.140357237	9.11E-07	8.30E-06
ENSMMUG00000052460		-1.110501337	0.002952358	0.011256366
ENSMMUG00000062793		-4.006269676	2.84E-10	4.98E-09
ENSMMUG00000021438	BTG anti-proliferation factor 4 [Source:VGNC Symbol;Acc:VGNC:70305]	-0.618140052	9.80E-05	0.000560434
ENSMMUG00000030914	salt inducible kinase 2 [Source:VGNC Symbol;Acc:VGNC:82237]	-0.925476766	0.007342044	0.024636872
ENSMMUG00000014001	protein phosphatase 2 scaffold subunit Abeta [Source:VGNC Symbol;Acc:VGNC:76375]	-0.953846683	0.000799792	0.003648496
ENSMMUG00000014334	dopamine receptor D2 [Source:VGNC Symbol;Acc:VGNC:71989]	-1.387485801	0.000265074	0.001381211
ENSMMUG00000050683		2.864097625	0.00906119	0.029454118
ENSMMUG00000051510		-0.629434256	4.05E-08	4.86E-07
ENSMMUG00000020002	BUD13 homolog [Source:VGNC Symbol;Acc:VGNC:82112]	0.743885481	0.010343395	0.032890118
ENSMMUG00000057425	apolipoprotein A5 [Source:NCBI gene;Acc:696484]	1.232574911	0.01580759	0.047023723
ENSMMUG00000060987		0.694129509	2.02E-06	1.70E-05
ENSMMUG00000016128	SIK family kinase 3 [Source:VGNC Symbol;Acc:VGNC:77330]	0.67230773	4.12E-08	4.94E-07
ENSMMUG00000002053	SID1 transmembrane family member 2 [Source:VGNC Symbol;Acc:VGNC:77300]	-0.609343031	0.004171375	0.01518961
ENSMMUG00000007785	beta-secretase 1 [Source:VGNC Symbol;Acc:VGNC:70215]	-3.325954439	0.003384139	0.012621218
ENSMMUG00000007794	DS cell adhesion molecule like 1 [Source:NCBI gene;Acc:698212]	0.429630486	0.00257905	0.010040536
ENSMMUG00000059436	interleukin 10 receptor subunit alpha [Source:NCBI gene;Acc:704383]	1.493330486	1.30E-14	4.46E-13
ENSMMUG00000007620	transmembrane serine protease 4 [Source:VGNC Symbol;Acc:VGNC:78577]	-1.38588516	2.62E-06	2.17E-05
ENSMMUG00000063655	sodium voltage-gated channel beta subunit 4 [Source:NCBI gene;Acc:100430940]	-0.921518937	1.12E-05	8.02E-05

ENSMMUG00000061384	sodium voltage-gated channel beta subunit 2 [Source:NCBI gene;Acc:678698]	0.446769918	1.67E-05	0.000114839
ENSMMUG00000006268	junction adhesion molecule like [Source:VGNC Symbol;Acc:VGNC:73860]	- 0.540191041	4.31E-05	0.000269535
ENSMMUG00000017593	CD3e molecule [Source:VGNC Symbol;Acc:VGNC:70764]	- 0.977657013	7.75E-11	1.51E-09
ENSMMUG00000065091	myelin protein zero like 2 [Source:NCBI gene;Acc:699330]	- 0.627283321	1.17E-08	1.55E-07
ENSMMUG00000005482	lysine methyltransferase 2A [Source:NCBI gene;Acc:705511]	1.942035299	5.71E-08	6.67E-07
ENSMMUG00000046655	pleckstrin homology like domain family B member 1 [Source:NCBI gene;Acc:706336]	- 3.847464871	1.13E-21	8.22E-20
ENSMMUG00000002549	trehalase [Source:NCBI gene;Acc:706449]	- 3.488452139	4.45E-42	2.15E-39
ENSMMUG00000009318	BCL9 like [Source:VGNC Symbol;Acc:VGNC:70113]	0.922104788	4.20E-09	6.08E-08
ENSMMUG00000005982	coiled-coil domain containing 84 [Source:NCBI gene;Acc:707400]	0.815356041	0.000236702	0.001246909
ENSMMUG00000056381	forkhead box R1 [Source:NCBI gene;Acc:707285]	2.6197664	3.04E-12	7.33E-11
ENSMMUG00000016894	ribosomal protein S25 [Source:NCBI gene;Acc:702593]	1.583982951	0.001330161	0.005645537
ENSMMUG00000016946	trafficking protein particle complex 4 [Source:VGNC Symbol;Acc:VGNC:79211]	1.036230975	0.000720411	0.003313324
ENSMMUG00000016890	solute carrier family 37 member 4 [Source:VGNC Symbol;Acc:VGNC:77591]	1.022653408	1.24E-11	2.73E-10
ENSMMUG00000030891	hypoxia up-regulated 1 [Source:VGNC Symbol;Acc:VGNC:73399]	0.913278716	7.66E-10	1.25E-08
ENSMMUG00000014864	VPS11 core subunit of CORVET and HOPS complexes [Source:VGNC Symbol;Acc:VGNC:78965]	0.658917809	8.21E-05	0.000478505
ENSMMUG00000059341	Metazoan signal recognition particle RNA [Source:RFAM;Acc:RF00017]	1.075345578	1.31E-10	2.45E-09
ENSMMUG00000014866	hydroxymethylbilane synthase [Source:VGNC Symbol;Acc:VGNC:73479]	0.754783715	0.011687052	0.036494251
ENSMMUG00000044496	H2A histone family, member X [Source:NCBI gene;Acc:703073]	0.7924687	0.002827031	0.010849332
ENSMMUG00000014872	dolichyl-phosphate N-acetylglucosaminephosphotransferase 1 [Source:VGNC Symbol;Acc:VGNC:71971]	1.263387266	3.69E-18	1.95E-16
ENSMMUG00000013799	ATP binding cassette subfamily G member 4 [Source:VGNC Symbol;Acc:VGNC:69390]	- 3.924464658	1.02E-09	1.63E-08
ENSMMUG00000012191	coiled-coil domain containing 153 [Source:NCBI gene;Acc:704216]	- 1.424744289	0.008592189	0.028230617
ENSMMUG00000008923	Thy-1 cell surface antigen [Source:VGNC Symbol;Acc:VGNC:78352]	2.618971572	5.68E-10	9.42E-09
ENSMMUG00000064895		2.180609325	0.000701688	0.003239962
ENSMMUG00000022347	glutamate ionotropic receptor kainate type subunit 4 [Source:VGNC Symbol;Acc:VGNC:73279]	- 1.320919548	0.002171454	0.008662959
ENSMMUG00000039397	tubulin folding cofactor E like [Source:NCBI gene;Acc:706506]	1.014037265	3.66E-06	2.95E-05
ENSMMUG00000053625	sterol-C5-desaturase [Source:NCBI gene;Acc:706827]	1.03685161	3.68E-09	5.36E-08
ENSMMUG00000014880	sortilin related receptor 1 [Source:NCBI gene;Acc:713011]	0.787021639	1.52E-06	1.32E-05
ENSMMUG00000015522	GRAM domain containing 1B [Source:VGNC Symbol;Acc:VGNC:73028]	- 1.256914285	3.14E-09	4.61E-08
ENSMMUG00000061604		- 2.184927483	0.012612129	0.038844692
ENSMMUG00000008474	transforming growth factor beta regulator 1 [Source:VGNC Symbol;Acc:VGNC:78239]	1.03309261	0.00117827	0.005087095
ENSMMUG00000057600	V-set and immunoglobulin domain containing 2 [Source:NCBI gene;Acc:712047]	- 0.651675935	0.016235982	0.048031963
ENSMMUG00000008486	endothelial cell adhesion molecule [Source:VGNC Symbol;Acc:VGNC:72290]	- 0.799023368	2.04E-05	0.000138168
ENSMMUG00000057529		- 0.645477635	0.001299948	0.005539099
ENSMMUG00000010009	EI24 autophagy associated transmembrane protein [Source:VGNC Symbol;Acc:VGNC:72038]	- 0.776786614	0.000526841	0.002524911
ENSMMUG00000060925		- 2.799584764	0.013619242	0.041536023
ENSMMUG00000038587	prostate and testis expressed 2 [Source:NCBI gene;Acc:717085]	- 3.004979688	0.00025336	0.00132619
ENSMMUG00000060291	prostate and testis expressed 3 [Source:NCBI gene;Acc:100379154]	- 2.745133908	0.003381939	0.012617728

ENSMMUG00000014120	sc328 [Source:NCBI gene;Acc:574167]	-2.259077198	0.007151083	0.024076742
ENSMMUG00000065066	V-set and immunoglobulin domain containing 10 like 2 [Source:NCBI gene;Acc:717387]	-0.692587039	3.50E-06	2.83E-05
ENSMMUG00000007628	cell adhesion associated, oncogene regulated [Source:VGNC Symbol;Acc:VGNC:70961]	-2.596613406	1.27E-07	1.38E-06
ENSMMUG00000007938	SRP receptor subunit alpha [Source:VGNC Symbol;Acc:VGNC:77885]	-0.473476565	0.002934315	0.011202805
ENSMMUG00000006929	ST3 beta-galactoside alpha-2,3-sialyltransferase 4 [Source:VGNC Symbol;Acc:VGNC:77827]	2.010387208	4.83E-26	5.94E-24
ENSMMUG00000006931	kirre like nephrin family adhesion molecule 3 [Source:NCBI gene;Acc:714534]	2.261364021	2.39E-37	8.38E-35
ENSMMUG00000063056		-2.122734912	0.014536425	0.043858155
ENSMMUG00000020706	ETS proto-oncogene 1, transcription factor [Source:VGNC Symbol;Acc:VGNC:72439]	-1.417580889	1.72E-35	5.13E-33
ENSMMUG00000060836		-3.218479132	1.62E-05	0.000111751
ENSMMUG00000003144	potassium inwardly rectifying channel subfamily J member 1 [Source:VGNC Symbol;Acc:VGNC:73984]	0.309392312	0.000824955	0.003737754
ENSMMUG00000002963	Rho GTPase activating protein 32 [Source:VGNC Symbol;Acc:VGNC:69855]	2.107726372	7.78E-06	5.82E-05
ENSMMUG00000032694	U6 spliceosomal RNA [Source:RFAM;Acc:RF00026]	3.073820717	0.001730632	0.007087806
ENSMMUG00000057813		2.993625981	3.09E-09	4.55E-08
ENSMMUG00000017555	PR/SET domain 10 [Source:VGNC Symbol;Acc:VGNC:76250]	-0.659165292	1.93E-05	0.000131152
ENSMMUG00000018029	amyloid beta precursor like protein 2 [Source:VGNC Symbol;Acc:VGNC:69984]	0.837330131	1.07E-07	1.18E-06
ENSMMUG00000018034	suppression of tumorigenicity 14 [Source:VGNC Symbol;Acc:VGNC:77897]	1.534530396	7.03E-17	3.17E-15
ENSMMUG00000002250	beta-1,3-glucuronyltransferase 1 [Source:VGNC Symbol;Acc:VGNC:70207]	-1.962633231	0.003241419	0.012156839
ENSMMUG00000009187	calcium voltage-gated channel subunit alpha1 B [Source:VGNC Symbol;Acc:VGNC:70499]	2.059872331	0.003372324	0.012597256
ENSMMUG00000019607	patatin like phospholipase domain containing 7 [Source:VGNC Symbol;Acc:VGNC:76104]	0.812229226	2.03E-06	1.70E-05
ENSMMUG00000001667	transmembrane protein 210 [Source:VGNC Symbol;Acc:VGNC:79945]	0.673405317	1.37E-09	2.14E-08
ENSMMUG00000021432	PAXX non-homologous end joining factor [Source:VGNC Symbol;Acc:VGNC:75686]	2.984757789	0.011914322	0.03707001
ENSMMUG00000021428	lipocalin 12 [Source:VGNC Symbol;Acc:VGNC:74143]	1.379884323	7.41E-09	1.01E-07
ENSMMUG00000011528	F-box and WD repeat domain containing 5 [Source:VGNC Symbol;Acc:VGNC:72503]	1.620015362	1.95E-06	1.64E-05
ENSMMUG00000011527	TNF receptor associated factor 2 [Source:VGNC Symbol;Acc:VGNC:78641]	1.037795384	0.000514269	0.002471416
ENSMMUG00000005048	MAM domain containing 4 [Source:VGNC Symbol;Acc:VGNC:74345]	-0.678768579	4.70E-05	0.000290531
ENSMMUG00000005042	RAB, member RAS oncogene family like 6 [Source:VGNC Symbol;Acc:VGNC:76529]	-0.706489041	0.003124739	0.01177276
ENSMMUG00000026981	EGF like domain multiple 7 [Source:VGNC Symbol;Acc:VGNC:72079]	-0.642388267	0.000227379	0.001204132
ENSMMUG00000056009		1.35464618	5.30E-10	8.85E-09
ENSMMUG00000017022	notch receptor 1 [Source:VGNC Symbol;Acc:VGNC:75371]	2.066936118	0.003028465	0.011496524
ENSMMUG00000003106	SEC16 homolog A, endoplasmic reticulum export factor [Source:VGNC Symbol;Acc:VGNC:77326]	1.545835066	3.37E-12	8.07E-11
ENSMMUG00000003105	inositol polyphosphate-5-phosphatase E [Source:VGNC Symbol;Acc:VGNC:73637]	1.134190363	8.12E-13	2.13E-11
ENSMMUG00000003098	peptidase, mitochondrial processing alpha subunit [Source:VGNC Symbol;Acc:VGNC:76062]	1.535083891	1.96E-27	2.69E-25
ENSMMUG00000003081	G protein signaling modulator 1 [Source:VGNC Symbol;Acc:VGNC:72960]	-0.573814412	0.000307489	0.001566828
ENSMMUG00000001409	coiled-coil domain containing 187 [Source:NCBI gene;Acc:710749]	1.258061261	3.67E-06	2.96E-05
ENSMMUG00000004885	quiescin sulfhydryl oxidase 2 [Source:VGNC Symbol;Acc:VGNC:76568]	-2.011005095	0.002775132	0.010679385
ENSMMUG00000000276	NACC family member 2 [Source:VGNC Symbol;Acc:VGNC:75013]	3.579471499	1.53E-05	0.000106092
ENSMMUG00000016827	UBA domain containing 1 [Source:VGNC Symbol;Acc:VGNC:79837]	2.538028619	1.49E-09	2.31E-08
ENSMMUG00000021496	potassium sodium-activated channel subfamily T member 1 [Source:VGNC Symbol;Acc:VGNC:73856]	-1.296948041	4.72E-08	5.60E-07

ENSMMUG00000055942	spermatogenesis and oogenesis specific basic helix-loop-helix 1 [Source:NCBI gene;Acc:721833]	-1.045883129	0.001642549	0.006770566
ENSMMUG00000012147	collagen type V alpha 1 chain [Source:NCBI gene;Acc:722014]	2.494058481	6.88E-07	6.45E-06
ENSMMUG00000051925		2.335639296	4.06E-18	2.13E-16
ENSMMUG00000064229		1.872724952	1.67E-07	1.77E-06
ENSMMUG00000005463	bromodomain containing 3 [Source:VGNC Symbol;Acc:VGNC:70281]	-0.588780645	0.001374729	0.005811825
ENSMMUG00000017210	sarcosine dehydrogenase [Source:VGNC Symbol;Acc:VGNC:76971]	-3.885437984	7.50E-14	2.29E-12
ENSMMUG00000017207	dopamine beta-hydroxylase [Source:VGNC Symbol;Acc:VGNC:71740]	-3.353119399	3.92E-14	1.26E-12
ENSMMUG00000022348	surfeit 4 [Source:VGNC Symbol;Acc:VGNC:78060]	0.941215951	3.49E-06	2.82E-05
ENSMMUG00000001267	general transcription factor IIIC subunit 5 [Source:VGNC Symbol;Acc:VGNC:73319]	-0.573336068	0.010242081	0.032612329
ENSMMUG00000009918	TSC complex subunit 1 [Source:VGNC Symbol;Acc:VGNC:78658]	-0.511982164	0.004422956	0.015968875
ENSMMUG00000018468	senataxin [Source:VGNC Symbol;Acc:VGNC:77356]	-0.521980594	0.003443794	0.012819834
ENSMMUG00000009976	uridine-cytidine kinase 1 [Source:VGNC Symbol;Acc:VGNC:78967]	0.4189318	0.010662563	0.033744328
ENSMMUG00000009973	proline rich coiled-coil 2B [Source:VGNC Symbol;Acc:VGNC:76327]	1.639333741	0.000718572	0.003307033
ENSMMUG00000002267	laminin subunit gamma 3 [Source:NCBI gene;Acc:722430]	3.334871542	9.21E-10	1.48E-08
ENSMMUG00000036223		2.830200007	0.000146301	0.000806753
ENSMMUG00000008249	far upstream element binding protein 3 [Source:NCBI gene;Acc:722470]	-0.676033861	0.000820425	0.003723244
ENSMMUG00000047733	hemicentin 2 [Source:NCBI gene;Acc:720622]	-0.328745964	0.002038652	0.008198457
ENSMMUG00000061477		-0.439058424	0.003987534	0.014592169
ENSMMUG00000003797	ubiquitin specific peptidase 20 [Source:VGNC Symbol;Acc:VGNC:78740]	2.527840558	3.44E-12	8.21E-11
ENSMMUG00000009965	chromosome 15 C9orf50 homolog [Source:VGNC Symbol;Acc:VGNC:70365]	-1.949954802	2.55E-13	7.10E-12
ENSMMUG00000055560		-1.190990303	0.003147946	0.011844284
ENSMMUG00000051852	Metazoan signal recognition particle RNA [Source:RFAM;Acc:RF00017]	1.215188645	0.000913765	0.004068416
ENSMMUG00000011767	carnitine O-acetyltransferase [Source:VGNC Symbol;Acc:VGNC:71483]	0.979465562	6.62E-06	5.03E-05
ENSMMUG00000005989	GLE1 RNA export mediator [Source:VGNC Symbol;Acc:VGNC:73067]	1.134068159	2.74E-12	6.67E-11
ENSMMUG00000012163	GTPase activating Rap/RanGAP domain like 3 [Source:VGNC Symbol;Acc:VGNC:72785]	-0.4503304	0.006185251	0.021191247
ENSMMUG00000050175	zinc finger and BTB domain containing 34 [Source:NCBI gene;Acc:701665]	0.746495875	0.01112684	0.035000375
ENSMMUG00000052476	zinc finger and BTB domain containing 43 [Source:NCBI gene;Acc:701797]	0.569580697	0.000468337	0.002276981
ENSMMUG00000013763	PBX homeobox 3 [Source:VGNC Symbol;Acc:VGNC:82202]	-0.393718508	0.003407796	0.012699315
ENSMMUG00000016826	MAPK associated protein 1 [Source:VGNC Symbol;Acc:VGNC:74531]	0.662302243	0.007781997	0.025896285
ENSMMUG00000003668	GTPase activating protein and VPS9 domains 1 [Source:VGNC Symbol;Acc:VGNC:72767]	0.893757918	1.27E-10	2.39E-09
ENSMMUG00000005217	heat shock protein family A (Hsp70) member 5 [Source:NCBI gene;Acc:703193]	0.6286686	0.003008606	0.011437093
ENSMMUG00000005215	Rab9 effector protein with kelch motifs [Source:VGNC Symbol;Acc:VGNC:76495]	0.887123161	9.60E-07	8.70E-06
ENSMMUG00000020549	protein phosphatase 6 catalytic subunit [Source:NCBI gene;Acc:703658]	1.015266153	3.41E-05	0.0002183
ENSMMUG00000051739	Metazoan signal recognition particle RNA [Source:RFAM;Acc:RF00017]	1.864931062	0.00846359	0.02786031
ENSMMUG00000011183	olfactomedin like 2A [Source:VGNC Symbol;Acc:VGNC:75472]	-1.80615474	1.82E-05	0.000124213
ENSMMUG00000033102	mml-mir-181b-2 [Source:miRBase;Acc:MI0007646]	-2.384203772	0.00034566	0.001732434
ENSMMUG00000026876	mml-mir-181a-2 [Source:miRBase;Acc:MI0002808]	-3.266656698	8.89E-06	6.54E-05
ENSMMUG00000014111	nuclear receptor subfamily 5 group A member 1 [Source:VGNC Symbol;Acc:VGNC:75520]	-0.454243157	0.000637275	0.002975871

ENSMMUG00000014110	adhesion G protein-coupled receptor D2 [Source:VGNC Symbol;Acc:VGNC:69462]	0.730590831	5.77E-13	1.55E-11
ENSMMUG00000054736		3.867903107	1.73E-11	3.73E-10
ENSMMUG00000000496	zinc finger and BTB domain containing 6 [Source:VGNC Symbol;Acc:VGNC:79047]	-3.46180961	0.00235774	0.009276822
ENSMMUG00000063639		1.561599098	2.30E-07	2.37E-06
ENSMMUG00000038944		2.765724961	2.84E-47	1.99E-44
ENSMMUG00000058731	olfactory receptor 1B1 [Source:NCBI gene;Acc:699905]	-0.334845197	0.00615663	0.021108662
ENSMMUG00000043396	Metazoan signal recognition particle RNA [Source:RFAM;Acc:RF00017]	3.96962818	2.04E-07	2.13E-06
ENSMMUG00000005595	RAB14, member RAS oncogene family [Source:NCBI gene;Acc:699837]	2.89468348	5.70E-12	1.32E-10
ENSMMUG00000012668	centriolin [Source:VGNC Symbol;Acc:VGNC:71204]	2.200447377	2.02E-24	2.00E-22
ENSMMUG00000058237		0.87322714	1.40E-07	1.51E-06
ENSMMUG00000013105	astrotactin 2 [Source:VGNC Symbol;Acc:VGNC:70087]	0.752347488	0.000382065	0.001891845
ENSMMUG00000013107	tripartite motif containing 32 [Source:VGNC Symbol;Acc:VGNC:79032]	0.427568418	0.005846688	0.020196237
ENSMMUG00000013099	pappalysin 1 [Source:VGNC Symbol;Acc:VGNC:75756]	1.817298358	9.52E-10	1.52E-08
ENSMMUG00000003356	tenascin C [Source:VGNC Symbol;Acc:VGNC:79960]	0.73847642	0.000468451	0.002276981
ENSMMUG00000041301	TNF superfamily member 8 [Source:NCBI gene;Acc:702944]	-0.64962743	0.00958903	0.030855201
ENSMMUG00000062461	chromosome 15 C9orf43 homolog [Source:NCBI gene;Acc:704968]	-3.537914343	0.002413304	0.009476829
ENSMMUG00000044537	DNA polymerase epsilon 3, accessory subunit [Source:NCBI gene;Acc:705074]	-3.118524099	1.97E-05	0.00013348
ENSMMUG00000031965	aminolevulinate dehydratase [Source:VGNC Symbol;Acc:VGNC:69779]	-2.104183056	6.41E-05	0.000383618
ENSMMUG00000057031		-2.709263405	1.09E-05	7.83E-05
ENSMMUG00000007187	haloacid dehalogenase like hydrolase domain containing 3 [Source:VGNC Symbol;Acc:VGNC:73250]	-3.669661904	6.60E-06	5.03E-05
ENSMMUG00000005851	B-box and SPRY domain containing [Source:VGNC Symbol;Acc:VGNC:70342]	-3.77081983	2.36E-11	4.95E-10
ENSMMUG00000000464	polypyrimidine tract binding protein 3 [Source:VGNC Symbol;Acc:VGNC:76244]	-0.51893302	0.007289232	0.024494823
ENSMMUG00000021097	sushi domain containing 1 [Source:VGNC Symbol;Acc:VGNC:78207]	-1.105711325	0.001985249	0.008004356
ENSMMUG00000000695	transmembrane protein 245 [Source:VGNC Symbol;Acc:VGNC:78933]	0.464617705	0.000195498	0.001049569
ENSMMUG00000000690	catenin alpha like 1 [Source:VGNC Symbol;Acc:VGNC:71544]	-0.899761657	0.000198227	0.001062588
ENSMMUG00000053648	actin binding transcription modulator [Source:NCBI gene;Acc:710486]	-1.321750722	1.16E-06	1.03E-05
ENSMMUG00000019354	actin like 7B [Source:VGNC Symbol;Acc:VGNC:69421]	1.740259189	0.000359561	0.001791162
ENSMMUG00000023797	post-GPI attachment to proteins GalNAc transferase 4 [Source:VGNC Symbol;Acc:VGNC:79688]	1.778716321	2.42E-05	0.000160435
ENSMMUG00000052330	mitochondrial ribosomal protein L50 [Source:NCBI gene;Acc:713923]	2.496290309	0.007300159	0.024525667
ENSMMUG00000009775	caveolae associated protein 4 [Source:NCBI gene;Acc:714379]	-0.970867145	1.30E-11	2.84E-10
ENSMMUG00000057137		-0.903194863	4.80E-05	0.000295861
ENSMMUG00000011410	nuclear receptor subfamily 4 group A member 3 [Source:VGNC Symbol;Acc:VGNC:75519]	-0.910123178	0.009100948	0.029576499
ENSMMUG00000058899		3.391480499	0.00874315	0.028632693
ENSMMUG00000010661	SEC61 translocon subunit beta [Source:NCBI gene;Acc:717815]	-0.78050202	3.47E-05	0.000221432
ENSMMUG00000010662	ALG2 alpha-1,3/1,6-mannosyltransferase [Source:VGNC Symbol;Acc:VGNC:69672]	0.949492504	0.00099234	0.004382897
ENSMMUG00000001304	transforming growth factor beta receptor 1 [Source:VGNC Symbol;Acc:VGNC:78337]	0.487926403	0.006635581	0.022541366
ENSMMUG00000009831	TBC1 domain family member 2 [Source:VGNC Symbol;Acc:VGNC:78157]	-1.59813596	8.98E-05	0.000518305
ENSMMUG00000002853	coronin 2A [Source:VGNC Symbol;Acc:VGNC:71451]	-0.700963925	0.000228033	0.001206229
ENSMMUG00000047381	DDB1 and CUL4 associated factor 10 [Source:NCBI gene;Acc:716256]	-0.723644293	2.77E-06	2.27E-05
ENSMMUG00000017886	F-box protein 10 [Source:NCBI gene;Acc:718443]	-0.88261549	0.000626349	0.002937554

ENSMMUG00000017892	RNA polymerase I subunit E [Source:VGNC Symbol;Acc:VGNC:76047]	-0.905109982	0.000441591	0.002159145
ENSMMUG00000007421	zinc finger CCHC-type containing 7 [Source:VGNC Symbol;Acc:VGNC:82088]	-0.389824133	0.004387102	0.015868026
ENSMMUG00000019639	paired box 5 [Source:VGNC Symbol;Acc:VGNC:75764]	-4.796790402	7.52E-13	1.98E-11
ENSMMUG00000010213	GLI pathogenesis related 2 [Source:VGNC Symbol;Acc:VGNC:81414]	3.368045875	0.001884772	0.007656476
ENSMMUG00000022613	reversion inducing cysteine rich protein with kazal motifs [Source:VGNC Symbol;Acc:VGNC:76722]	1.525236661	3.06E-26	3.87E-24
ENSMMUG00000059406	RGP1 homolog, RAB6A GEF complex partner 1 [Source:NCBI gene;Acc:695936]	-2.442777434	3.91E-06	3.13E-05
ENSMMUG00000016331	glucosylceramidase beta 2 [Source:VGNC Symbol;Acc:VGNC:72741]	-3.933930908	5.15E-06	4.00E-05
ENSMMUG00000031856	talin 1 [Source:VGNC Symbol;Acc:VGNC:78371]	-0.772889196	1.31E-06	1.15E-05
ENSMMUG00000050822	carbonic anhydrase 9 [Source:NCBI gene;Acc:696729]	2.085389178	1.74E-27	2.44E-25
ENSMMUG00000048375	Metazoan signal recognition particle RNA [Source:RFAM;Acc:RF00017]	1.894257334	5.14E-30	9.12E-28
ENSMMUG00000016323	Rho guanine nucleotide exchange factor 39 [Source:VGNC Symbol;Acc:VGNC:69912]	1.919219724	2.09E-24	2.05E-22
ENSMMUG00000016327	coiled-coil domain containing 107 [Source:VGNC Symbol;Acc:VGNC:70656]	1.951604333	3.46E-23	2.94E-21
ENSMMUG00000038988	RNase MRP [Source:RFAM;Acc:RF00030]	2.788676988	0.001595332	0.006605061
ENSMMUG00000016316	testis associated actin remodelling kinase 1 [Source:VGNC Symbol;Acc:VGNC:78314]	-1.358245901	1.22E-05	8.68E-05
ENSMMUG00000016314	family with sequence similarity 166 member B [Source:VGNC Symbol;Acc:VGNC:72421]	-3.371339952	1.16E-34	3.16E-32
ENSMMUG00000016313	RUN and SH3 domain containing 2 [Source:VGNC Symbol;Acc:VGNC:82230]	-0.899345281	1.83E-06	1.55E-05
ENSMMUG00000020190	valosin containing protein [Source:VGNC Symbol;Acc:VGNC:78762]	1.467166083	1.46E-07	1.57E-06
ENSMMUG00000061896		1.637408853	4.63E-09	6.64E-08
ENSMMUG00000044048	Metazoan signal recognition particle RNA [Source:RFAM;Acc:RF00017]	1.52721175	9.34E-06	6.83E-05
ENSMMUG00000022895	DnaJ heat shock protein family (Hsp40) member B5 [Source:NCBI gene;Acc:699081]	0.413763504	5.11E-05	0.000313685
ENSMMUG00000031826	ubiquitin associated protein 1 [Source:VGNC Symbol;Acc:VGNC:78686]	1.239449622	0.007187467	0.024193434
ENSMMUG00000058149		1.006497241	0.00310814	0.011725974
ENSMMUG00000017458	DDB1 and CUL4 associated factor 12 [Source:VGNC Symbol;Acc:VGNC:71678]	1.125382233	0.001187973	0.005124254
ENSMMUG00000000389	nucleolar protein 6 [Source:VGNC Symbol;Acc:VGNC:75218]	0.834680076	9.00E-05	0.000519514
ENSMMUG00000003314	aquaporin 3 (Gill blood group) [Source:NCBI gene;Acc:702721]	0.999752853	2.92E-05	0.000189571
ENSMMUG00000001545	aquaporin 7 [Source:NCBI gene;Acc:702836]	2.405557144	1.20E-14	4.15E-13
ENSMMUG00000038377		-0.321342308	0.010070363	0.032167815
ENSMMUG00000007724	aprataxin [Source:VGNC Symbol;Acc:VGNC:69838]	-0.945826792	4.70E-09	6.72E-08
ENSMMUG00000012442	DExD/H-box helicase 58 [Source:VGNC Symbol;Acc:VGNC:71672]	0.274950985	0.008492399	0.02793288
ENSMMUG00000008196	aconitase 1 [Source:VGNC Symbol;Acc:VGNC:69378]	1.381277804	1.08E-10	2.05E-09
ENSMMUG00000061449	equatorin [Source:NCBI gene;Acc:707334]	-1.732832691	0.001081481	0.004721546
ENSMMUG00000054514	interferon alpha 6 [Source:NCBI gene;Acc:106995851]	-3.129632689	0.010838626	0.034224286
ENSMMUG00000010959	kelch like family member 9 [Source:VGNC Symbol;Acc:VGNC:74080]	-2.636730212	0.00349179	0.012974396
ENSMMUG00000019070	focadhesin [Source:VGNC Symbol;Acc:VGNC:72556]	2.125847957	4.63E-79	3.24E-75
ENSMMUG00000018396	HAUS augmin like complex subunit 6 [Source:VGNC Symbol;Acc:VGNC:73237]	3.795592005	1.90E-45	1.27E-42
ENSMMUG00000044658	ADAMTS like 1 [Source:NCBI gene;Acc:712844]	-0.587208952	0.002543451	0.009918468
ENSMMUG00000013466	tetratricopeptide repeat domain 39B [Source:VGNC Symbol;Acc:VGNC:79402]	-0.787996444	1.70E-12	4.24E-11

ENSMMUG00000052889	Metazoan signal recognition particle RNA [Source:RFAM;Acc:RF00017]	-0.933745306	5.97E-06	4.59E-05
ENSMMUG00000055400	U6 spliceosomal RNA [Source:RFAM;Acc:RF00026]	-1.167003782	2.45E-07	2.52E-06
ENSMMUG00000014697	nuclear factor I B [Source:VGNC Symbol;Acc:VGNC:75335]	-2.047307965	3.24E-05	0.000208197
ENSMMUG00000002176	ubiquitin like with PHD and ring finger domains 2 [Source:VGNC Symbol;Acc:VGNC:78714]	-1.044540371	5.30E-12	1.24E-10
ENSMMUG00000050923		-1.161825307	6.33E-08	7.34E-07
ENSMMUG00000002175	TPD52 like 3 [Source:HGNC Symbol;Acc:HGNC:23382]	-0.658363466	0.002954427	0.011258133
ENSMMUG00000021344	interleukin 33 [Source:VGNC Symbol;Acc:VGNC:73606]	-0.70531491	0.005281677	0.018539413
ENSMMUG00000005469	endoplasmic reticulum metallopeptidase 1 [Source:VGNC Symbol;Acc:VGNC:72322]	-0.293506312	0.009336798	0.030202981
ENSMMUG00000006197	plasminogen receptor with a C-terminal lysine [Source:VGNC Symbol;Acc:VGNC:76098]	-1.111868124	2.03E-08	2.59E-07
ENSMMUG00000047971	relaxin 2 [Source:NCBI gene;Acc:693473]	0.486588883	0.016294639	0.048134057
ENSMMUG00000052972		0.496885285	0.001312578	0.005582735
ENSMMUG00000065127	insulin like 6 [Source:NCBI gene;Acc:693735]	-0.384817704	0.000573071	0.002724125
ENSMMUG00000057331	RNA terminal phosphate cyclase like 1 [Source:NCBI gene;Acc:694568]	2.78284422	7.22E-44	4.22E-41
ENSMMUG00000026950	microRNA 101-2 [Source:HGNC Symbol;Acc:HGNC:31489]	3.381653292	4.58E-09	6.59E-08
ENSMMUG00000057520		3.306492743	0.000235085	0.001239786
ENSMMUG00000058311		2.378403371	1.11E-10	2.09E-09
ENSMMUG00000000802	regulatory factor X3 [Source:VGNC Symbol;Acc:VGNC:76908]	1.719128604	2.61E-05	0.000171434
ENSMMUG00000056693		-0.539095308	1.20E-07	1.31E-06
ENSMMUG00000015093	very low density lipoprotein receptor [Source:VGNC Symbol;Acc:VGNC:78774]	-0.291865315	0.009279284	0.030051594
ENSMMUG00000057073		-2.470363106	8.98E-06	6.60E-05
ENSMMUG00000015279	SWI/SNF related, matrix associated, actin dependent regulator of chromatin, subfamily a, member 2 [Source:VGNC Symbol;Acc:VGNC:77647]	-1.458664857	8.53E-10	1.38E-08
ENSMMUG00000012472	phosphatidylinositol-4-phosphate 5-kinase type 1 beta [Source:VGNC Symbol;Acc:VGNC:76001]	0.704810501	1.05E-07	1.17E-06
ENSMMUG00000027986	U6 spliceosomal RNA [Source:RFAM;Acc:RF00026]	2.047814207	1.45E-09	2.24E-08
ENSMMUG00000003780	tight junction protein 2 [Source:NCBI gene;Acc:699586]	3.393730037	0.00222794	0.008845527
ENSMMUG00000011401	amyloid beta precursor protein binding family A member 1 [Source:VGNC Symbol;Acc:VGNC:69976]	-2.032285375	3.80E-05	0.000240881
ENSMMUG00000008346	structural maintenance of chromosomes 5 [Source:NCBI gene;Acc:702219]	-2.724634517	0.000905259	0.004040246
ENSMMUG00000063718	Kruppel like factor 9 [Source:NCBI gene;Acc:700585]	-2.464087695	0.001926631	0.007801661
ENSMMUG00000012667	annexin A1 [Source:VGNC Symbol;Acc:VGNC:69944]	0.403998008	8.53E-05	0.000494352
ENSMMUG00000056840		1.75667797	1.71E-23	1.53E-21
ENSMMUG00000023117	neurotrophic receptor tyrosine kinase 2 [Source:VGNC Symbol;Acc:VGNC:75541]	-0.758953693	1.66E-09	2.55E-08
ENSMMUG00000059207		-2.23717358	0.003689828	0.013641564
ENSMMUG00000060761		1.487410242	1.19E-25	1.36E-23
ENSMMUG00000054211		2.236839797	1.74E-05	0.000119589
ENSMMUG00000057499		2.492245033	9.18E-13	2.38E-11
ENSMMUG00000033550		3.099774086	0.000603606	0.002857089
ENSMMUG00000049479		2.824666871	0.002692966	0.010403156
ENSMMUG00000053778		-0.889971854	0.001460812	0.006118569

ENSMMUG00000007223	DIRAS family GTPase 2 [Source:VGNC Symbol;Acc:VGNC:71673]	0.493769944	0.000100695	0.000574471
ENSMMUG00000001748	spleen associated tyrosine kinase [Source:VGNC Symbol;Acc:VGNC:78063]	2.50361541	4.25E-10	7.22E-09
ENSMMUG00000020009	receptor tyrosine kinase like orphan receptor 2 [Source:VGNC Symbol;Acc:VGNC:76716]	0.849317447	0.00014084	0.000779399
ENSMMUG00000060533		1.133497542	1.09E-13	3.20E-12
ENSMMUG00000003006	serine palmitoyltransferase long chain base subunit 1 [Source:VGNC Symbol;Acc:VGNC:77873]	1.280805259	5.61E-10	9.31E-09
ENSMMUG00000009493	family with sequence similarity 120A [Source:NCBI gene;Acc:717459]	-1.544265135	1.19E-08	1.57E-07
ENSMMUG00000002517	protein tyrosine phosphatase domain containing 1 [Source:VGNC Symbol;Acc:VGNC:76567]	-1.185683215	6.82E-05	0.000404817
ENSMMUG00000060049		-0.878444507	0.004109334	0.014990946
ENSMMUG00000050773		-1.116791588	5.93E-06	4.57E-05
ENSMMUG00000005997	cell division cycle 14B [Source:NCBI gene;Acc:710300]	3.125288286	5.59E-07	5.34E-06
ENSMMUG00000019423	zinc finger protein 2 [Source:NCBI gene;Acc:703864]	-2.436639698	0.012410333	0.038315741
ENSMMUG00000060932	zinc finger protein 383-like [Source:NCBI gene;Acc:106993074]	-1.253340479	3.73E-06	3.00E-05
ENSMMUG00000049314	dual specificity phosphatase 2 [Source:NCBI gene;Acc:718442]	-2.830996238	0.001676295	0.006899525
ENSMMUG00000053389		-0.870791861	3.52E-05	0.000224175
ENSMMUG00000057143	transmembrane protein 127 [Source:NCBI gene;Acc:705069]	-1.509376312	4.55E-10	7.68E-09
ENSMMUG00000015515	REV1 DNA directed polymerase [Source:NCBI gene;Acc:709475]	0.493251103	0.002636476	0.010221211
ENSMMUG00000057336		0.381034874	0.001001108	0.004411861
ENSMMUG00000001390	NCK adaptor protein 2 [Source:VGNC Symbol;Acc:VGNC:75142]	-1.333132626	0.000275688	0.001426561
ENSMMUG00000058257		0.82443821	4.51E-06	3.57E-05
ENSMMUG00000055529	sulfotransferase family 1C member 4 [Source:NCBI gene;Acc:693903]	-4.251595672	9.01E-06	6.62E-05
ENSMMUG00000021019	E3 SUMO-protein ligase RanBP2 [Source:NCBI gene;Acc:694823]	3.081311634	0.002136514	0.008543012
ENSMMUG00000060346		-2.023924508	7.02E-07	6.57E-06
ENSMMUG00000002231	transmembrane protein 87B [Source:VGNC Symbol;Acc:VGNC:79557]	-0.644799432	0.002218487	0.008818964
ENSMMUG00000044163	zinc finger CCCH-type containing 8 [Source:NCBI gene;Acc:698828]	-0.509095713	0.001731736	0.007088188
ENSMMUG00000059850	immunoglobulin kappa variable 1-9 [Source:NCBI gene;Acc:106993071]	-1.514741913	0.000706412	0.003258531
ENSMMUG00000050630		-1.971738744	0.002575164	0.010028192
ENSMMUG00000051042		-1.624578835	1.30E-24	1.33E-22
ENSMMUG00000046169		3.669103954	7.18E-50	6.71E-47
ENSMMUG00000025625	U6 spliceosomal RNA [Source:RFAM;Acc:RF00026]	1.168102363	0.00508729	0.017951452
ENSMMUG00000000293	catenin alpha 2 [Source:VGNC Symbol;Acc:VGNC:71543]	1.637776857	1.31E-13	3.81E-12
ENSMMUG00000055639		0.853653445	0.002478393	0.009694421
ENSMMUG00000000291	leucine rich repeat transmembrane neuronal 1 [Source:VGNC Symbol;Acc:VGNC:74315]	0.870990238	8.42E-05	0.000488655
ENSMMUG00000062818	regenerating islet-derived protein 3-alpha [Source:NCBI gene;Acc:716890]	-0.975575864	0.001902544	0.007719727
ENSMMUG00000040046	lithostathine-1-alpha [Source:NCBI gene;Acc:711810]	-1.343124021	0.005918235	0.020396022
ENSMMUG00000049292	regenerating islet-derived 1 alpha [Source:NCBI gene;Acc:711892]	-3.122396981	0.008956423	0.029181209

ENSMMUG00000054863		-1.596959439	0.001333357	0.005657386
ENSMMUG00000056785		-0.802300764	0.000406245	0.002001679
ENSMMUG00000056765		-0.809828137	4.30E-07	4.22E-06
ENSMMUG00000064788		-0.727965408	0.006783761	0.02294469
ENSMMUG00000064821		0.979203523	1.43E-05	9.98E-05
ENSMMUG00000062747		0.544353935	2.04E-06	1.71E-05
ENSMMUG00000023758	mitochondrial ribosomal protein L19 [Source:VGNC Symbol;Acc:VGNC:74743]	-0.42366028	0.001299596	0.005539099
ENSMMUG00000048322	eva-1 homolog A, regulator of programmed cell death [Source:NCBI gene;Acc:710843]	-1.033825309	0.005055812	0.017871855
ENSMMUG00000051843		-0.983693631	5.50E-07	5.27E-06
ENSMMUG00000053587		2.284321901	0.014795074	0.044435923
ENSMMUG00000001845	DNA polymerase epsilon 4, accessory subunit [Source:VGNC Symbol;Acc:VGNC:76065]	2.959796509	1.52E-24	1.53E-22
ENSMMUG00000011845	hexokinase 2 [Source:VGNC Symbol;Acc:VGNC:73475]	-1.172231216	7.37E-06	5.54E-05
ENSMMUG00000058044		-0.867004366	1.96E-06	1.65E-05
ENSMMUG00000008396	methylmalonyl-CoA epimerase [Source:NCBI gene;Acc:704094]	-1.035353086	3.41E-11	6.93E-10
ENSMMUG00000002096	N-acetylglucosamine kinase [Source:VGNC Symbol;Acc:VGNC:74977]	-0.621283583	0.000182409	0.000985333
ENSMMUG00000002093	testis expressed 261 [Source:NCBI gene;Acc:703863]	-1.436752885	6.63E-16	2.67E-14
ENSMMUG00000055845	ankyrin repeat domain 53 [Source:NCBI gene;Acc:703757]	-1.383831898	1.90E-08	2.43E-07
ENSMMUG00000041775	Metazoan signal recognition particle RNA [Source:RFAM;Acc:RF00017]	-1.399652227	0.006578863	0.022375794
ENSMMUG00000002090	ATPase H+ transporting V1 subunit B1 [Source:VGNC Symbol;Acc:VGNC:70184]	-1.444257588	4.03E-16	1.69E-14
ENSMMUG00000061424	ventral anterior homeobox 2 [Source:NCBI gene;Acc:703531]	2.414457537	8.08E-06	6.01E-05
ENSMMUG00000059700		2.663170228	0.000305832	0.00156009
ENSMMUG00000020852	folliculogenesis specific bHLH transcription factor [Source:VGNC Symbol;Acc:VGNC:72563]	3.679621612	0.015421944	0.046022968
ENSMMUG00000010592	adducin 2 [Source:VGNC Symbol;Acc:VGNC:69529]	1.986902294	0.000117603	0.000660983
ENSMMUG00000063699		3.206829906	0.000208183	0.00111002
ENSMMUG00000064749		-1.315244904	0.006287066	0.021503299
ENSMMUG00000007868	AP2 associated kinase 1 [Source:VGNC Symbol;Acc:VGNC:82094]	-0.98494309	4.09E-07	4.03E-06
ENSMMUG00000049539		-1.307800363	4.47E-08	5.32E-07
ENSMMUG00000013797	glutamine--fructose-6-phosphate transaminase 1 [Source:VGNC Symbol;Acc:VGNC:72915]	0.279176836	0.001006115	0.004432533
ENSMMUG00000017209	C1D nuclear receptor corepressor [Source:NCBI gene;Acc:698450]	1.588123483	0.003270975	0.012248032
ENSMMUG00000047067	SERTA domain containing 2 [Source:NCBI gene;Acc:696216]	-0.645060583	0.002763674	0.010638211
ENSMMUG00000059267	pellino E3 ubiquitin protein ligase 1 [Source:NCBI gene;Acc:696416]	-1.209578538	7.34E-12	1.68E-10
ENSMMUG00000008772	VPS54 subunit of GARP complex [Source:VGNC Symbol;Acc:VGNC:79159]	0.390309879	0.000592714	0.00280988
ENSMMUG00000008770	UDP-glucose pyrophosphorylase 2 [Source:VGNC Symbol;Acc:VGNC:78712]	0.669910871	0.000111663	0.000630626
ENSMMUG00000006163	malate dehydrogenase 1 [Source:VGNC Symbol;Acc:VGNC:74690]	-0.574388327	6.35E-05	0.000380601
ENSMMUG00000006941	exportin 1 [Source:VGNC Symbol;Acc:VGNC:78812]	1.109674452	1.11E-10	2.09E-09
ENSMMUG00000010834	REL proto-oncogene, NF-kB subunit [Source:VGNC Symbol;Acc:VGNC:76892]	0.340887732	0.004051845	0.01479277

ENSMMUG00000049993		-0.401884752	7.26E-07	6.79E-06
ENSMMUG00000007003	polyribonucleotide nucleotidyltransferase 1 [Source:VGNC Symbol;Acc:VGNC:76095]	-3.521645141	8.87E-06	6.53E-05
ENSMMUG00000007001	protein phosphatase 4 regulatory subunit 3B [Source:VGNC Symbol;Acc:VGNC:76342]	-4.587824947	1.63E-05	0.000112467
ENSMMUG00000007000	cilia and flagella associated protein 36 [Source:VGNC Symbol;Acc:VGNC:71142]	-2.854316264	0.012301896	0.038047968
ENSMMUG00000054164		-0.631701098	0.00081426	0.003698998
ENSMMUG00000008104	proteasome activator subunit 4 [Source:VGNC Symbol;Acc:VGNC:76295]	0.334006826	0.010013022	0.032013819
ENSMMUG00000008105	G protein-coupled receptor 75 [Source:VGNC Symbol;Acc:VGNC:73143]	0.375708032	0.005882822	0.020298922
ENSMMUG00000062091		3.60168096	2.95E-12	7.14E-11
ENSMMUG00000065301		-0.903076017	8.53E-07	7.83E-06
ENSMMUG00000061344		-1.07613936	0.000796813	0.00363609
ENSMMUG00000052169	potassium two pore domain channel subfamily K member 12 [Source:NCBI gene;Acc:715556]	-3.6112188	5.25E-05	0.000321181
ENSMMUG00000053577		-3.434923537	3.23E-13	8.92E-12
ENSMMUG00000000974	mutS homolog 2 [Source:VGNC Symbol;Acc:VGNC:74931]	-2.914403491	6.15E-15	2.21E-13
ENSMMUG00000059300		0.570363275	0.005639888	0.01958112
ENSMMUG00000014425	protein kinase C epsilon [Source:VGNC Symbol;Acc:VGNC:76412]	0.621588903	0.004450919	0.016049173
ENSMMUG00000054734		-0.77233174	0.000506119	0.002438702
ENSMMUG00000023752	SIX homeobox 2 [Source:VGNC Symbol;Acc:VGNC:81570]	-0.977292108	0.001096605	0.00478162
ENSMMUG00000014824	SIX homeobox 3 [Source:HGNC Symbol;Acc:HGNC:10889]	-0.873020831	7.24E-09	9.95E-08
ENSMMUG00000006311	prolyl endopeptidase like [Source:VGNC Symbol;Acc:VGNC:76346]	-0.898085847	4.47E-08	5.32E-07
ENSMMUG00000006310	solute carrier family 3 member 1 [Source:VGNC Symbol;Acc:VGNC:77462]	-0.951022792	0.00042784	0.002099964
ENSMMUG00000051072		0.936195865	1.91E-13	5.40E-12
ENSMMUG00000019841	leucine rich pentatricopeptide repeat containing [Source:VGNC Symbol;Acc:VGNC:82177]	-0.537747436	0.015999811	0.04748473
ENSMMUG00000008080	ATP binding cassette subfamily G member 5 [Source:VGNC Symbol;Acc:VGNC:69391]	1.16930341	8.46E-13	2.21E-11
ENSMMUG00000062474	Metazoan signal recognition particle RNA [Source:RFAM;Acc:RF00017]	0.987227872	0.00152231	0.006338286
ENSMMUG00000008077	dynein cytoplasmic 2 light intermediate chain 1 [Source:VGNC Symbol;Acc:VGNC:71899]	1.084353455	4.15E-10	7.07E-09
ENSMMUG00000025150	Y RNA [Source:RFAM;Acc:RF00019]	2.924172451	0.010800089	0.034148738
ENSMMUG00000000642	THADA armadillo repeat containing [Source:VGNC Symbol;Acc:VGNC:79185]	0.34838122	0.005503771	0.019198852
ENSMMUG00000063947	oxoeicosanoid receptor 1 [Source:NCBI gene;Acc:713039]	0.785889758	0.000291461	0.00149573
ENSMMUG00000020744	metastasis associated 1 family member 3 [Source:VGNC Symbol;Acc:VGNC:74814]	0.729458045	0.000183922	0.000993124
ENSMMUG00000056008		0.708771266	8.14E-10	1.32E-08
ENSMMUG00000055395	Y RNA [Source:RFAM;Acc:RF00019]	0.988803251	0.001916169	0.007770514
ENSMMUG00000043458	transmembrane protein 178A [Source:NCBI gene;Acc:714010]	-1.097543432	5.20E-06	4.04E-05
ENSMMUG00000056330		1.576565574	2.71E-05	0.000176794
ENSMMUG00000013901	mitogen-activated protein kinase kinase kinase kinase 3 [Source:VGNC Symbol;Acc:VGNC:81466]	0.698212403	0.004575303	0.016442716
ENSMMUG00000020575	SOS Ras/Rac guanine nucleotide exchange factor 1 [Source:VGNC Symbol;Acc:VGNC:77837]	0.471109322	0.002267085	0.008965409
ENSMMUG00000060562		0.759134487	0.004352088	0.015773929
ENSMMUG00000002338	heterogeneous nuclear ribonucleoprotein L like [Source:VGNC Symbol;Acc:VGNC:73441]	-0.850548768	1.49E-06	1.30E-05
ENSMMUG00000013944	regulator of microtubule dynamics 2 [Source:VGNC Symbol;Acc:VGNC:76839]	1.518764319	3.31E-18	1.76E-16
ENSMMUG00000016442	glutaminyl-peptide cyclotransferase [Source:VGNC Symbol;Acc:VGNC:76636]	2.218434329	8.92E-05	0.000515494

ENSMMUG00000016438	protein kinase D3 [Source:VGNC Symbol;Acc:VGNC:76415]	2.035193196	1.35E-10	2.52E-09
ENSMMUG00000016434	NADH:ubiquinone oxidoreductase complex assembly factor 7 [Source:VGNC Symbol;Acc:VGNC:75035]	2.373884836	1.90E-08	2.44E-07
ENSMMUG00000053593		-0.744312328	0.002741557	0.010564676
ENSMMUG00000021436	latent transforming growth factor beta binding protein 1 [Source:VGNC Symbol;Acc:VGNC:74456]	1.511028888	2.30E-09	3.46E-08
ENSMMUG00000055130		-0.471119662	0.007096601	0.023910531
ENSMMUG00000021691	spastin [Source:VGNC Symbol;Acc:VGNC:77857]	-0.987513249	6.49E-06	4.96E-05
ENSMMUG00000058351		0.48539423	0.000629367	0.002948749
ENSMMUG00000048513	tudor domain containing 15 [Source:NCBI gene;Acc:703066]	-0.749897819	0.000841308	0.00380202
ENSMMUG00000007692	apolipoprotein B [Source:VGNC Symbol;Acc:VGNC:69985]	-1.121584865	2.69E-16	1.14E-14
ENSMMUG00000003355	lipid droplet associated hydrolase [Source:VGNC Symbol;Acc:VGNC:74130]	0.631275957	0.000879287	0.003944413
ENSMMUG00000050446	growth differentiation factor 7 [Source:NCBI gene;Acc:702462]	0.600992792	0.001855619	0.007548983
ENSMMUG00000022996	HCLS1 binding protein 3 [Source:VGNC Symbol;Acc:VGNC:82158]	1.416645337	1.66E-10	3.05E-09
ENSMMUG00000064216		-2.87953654	1.41E-05	9.87E-05
ENSMMUG00000012058	GEN1 Holliday junction 5' flap endonuclease [Source:VGNC Symbol;Acc:VGNC:72790]	-3.291792004	8.57E-09	1.15E-07
ENSMMUG00000012056	structural maintenance of chromosomes 6 [Source:VGNC Symbol;Acc:VGNC:77746]	-5.855492656	1.75E-13	4.99E-12
ENSMMUG00000040529	visinin like 1 [Source:NCBI gene;Acc:699459]	-3.282479784	0.000312272	0.001588317
ENSMMUG00000052363	MYCN proto-oncogene, bHLH transcription factor [Source:NCBI gene;Acc:698699]	0.360074903	0.003636747	0.013470179
ENSMMUG00000004215	LRAT domain containing 1 [Source:VGNC Symbol;Acc:VGNC:74321]	-2.192974082	0.000302704	0.001546382
ENSMMUG00000052377		-1.807919939	6.37E-12	1.47E-10
ENSMMUG00000056755		1.512211649	0.002118149	0.008484092
ENSMMUG00000020249	growth regulating estrogen receptor binding 1 [Source:VGNC Symbol;Acc:VGNC:73227]	0.357341569	0.006909468	0.023336097
ENSMMUG00000023225	E2F transcription factor 6 [Source:VGNC Symbol;Acc:VGNC:72027]	0.474161323	0.00165725	0.006829156
ENSMMUG00000059188		1.962291007	8.12E-07	7.50E-06
ENSMMUG00000016678	Rho associated coiled-coil containing protein kinase 2 [Source:VGNC Symbol;Acc:VGNC:76714]	2.474104096	4.67E-18	2.38E-16
ENSMMUG00000060435	Metazoan signal recognition particle RNA [Source:RFAM;Acc:RF00017]	2.28884471	9.31E-05	0.000535614
ENSMMUG00000022785	solute carrier family 66 member 3 [Source:VGNC Symbol;Acc:VGNC:76334]	1.441018448	0.01165743	0.036432391
ENSMMUG00000064867	chromosome 13 C2orf50 homolog [Source:NCBI gene;Acc:708351]	1.767658214	0.002457719	0.009635055
ENSMMUG00000063262		-0.936986836	7.29E-09	1.00E-07
ENSMMUG00000052047		1.836555351	2.45E-24	2.36E-22
ENSMMUG00000022879	EARP complex and GARP complex interacting protein 1 [Source:NCBI gene;Acc:707650]	-2.705986638	0.000319196	0.001618735
ENSMMUG00000051814		0.638637379	0.001280078	0.005466058
ENSMMUG00000009310	myelin transcription factor 1 like [Source:NCBI gene;Acc:706914]	-0.75620651	2.10E-05	0.00014133
ENSMMUG00000009660	syntrophin gamma 2 [Source:VGNC Symbol;Acc:VGNC:77694]	-0.627038484	0.000368925	0.001832604
ENSMMUG00000061533		2.889590768	1.10E-25	1.28E-23
ENSMMUG00000050543		2.533237677	8.02E-13	2.11E-11
ENSMMUG00000042598	transmembrane protein 18 [Source:NCBI gene;Acc:721515]	3.128334162	1.71E-19	1.00E-17
ENSMMUG00000038858		0.544811864	3.26E-05	0.000209117
ENSMMUG00000022914	RAB, member of RAS oncogene family like 2B [Source:NCBI gene;Acc:716615]	0.687338146	0.000805124	0.003668049
ENSMMUG00000009119	modulator of VRAC current 1 [Source:VGNC Symbol;Acc:VGNC:74787]	0.263396474	0.001023421	0.004503115
ENSMMUG00000011278	cysteine rich with EGF like domains 2 [Source:NCBI gene;Acc:720586]	0.537160988	8.72E-06	6.42E-05

ENSMMUG00000011273	ALG12 alpha-1,6-mannosyltransferase [Source:VGNC Symbol;Acc:VGNC:69637]	-0.848210118	0.010392419	0.033023542
ENSMMUG00000056186		1.202571577	0.001242723	0.005334161
ENSMMUG00000020993	cadherin EGF LAG seven-pass G-type receptor 1 [Source:NCBI gene;Acc:718440]	-1.653496887	9.32E-06	6.82E-05
ENSMMUG00000016262	tetratricopeptide repeat domain 38 [Source:VGNC Symbol;Acc:VGNC:79574]	-1.926811533	1.68E-11	3.62E-10
ENSMMUG00000057898		-0.605480534	0.005589196	0.019429176
ENSMMUG00000023003	family with sequence similarity 118 member A [Source:VGNC Symbol;Acc:VGNC:72265]	0.43656187	0.005238238	0.018419209
ENSMMUG00000031000	Rho GTPase activating protein 8 [Source:NCBI gene;Acc:716745]	-3.732118182	5.45E-07	5.23E-06
ENSMMUG00000065099		-2.998270473	1.63E-16	7.05E-15
ENSMMUG00000064395		-0.686447764	6.63E-05	0.000395079
ENSMMUG00000005769	EF-hand calcium binding domain 6 [Source:VGNC Symbol;Acc:VGNC:72092]	0.830718441	6.65E-05	0.00039571
ENSMMUG00000008106	solute carrier family 25 member 17 [Source:NCBI gene;Acc:705547]	0.718840768	0.000436093	0.002135986
ENSMMUG00000056662		-1.083065374	2.50E-05	0.00016468
ENSMMUG00000012101	synaptogyrin 1 [Source:VGNC Symbol;Acc:VGNC:78068]	-0.955110587	9.62E-05	0.000550882
ENSMMUG00000048395	chromobox 6 [Source:NCBI gene;Acc:702133]	3.814661366	0.000445153	0.002175801
ENSMMUG00000004868	dynein axonemal light chain 4 [Source:VGNC Symbol;Acc:VGNC:71945]	3.356965897	0.012622066	0.038866761
ENSMMUG00000007277	GTP binding protein 1 [Source:VGNC Symbol;Acc:VGNC:73320]	-1.06456534	0.010585177	0.033529691
ENSMMUG00000012467	family with sequence similarity 227 member A [Source:VGNC Symbol;Acc:VGNC:72475]	-0.876372995	0.001434027	0.006022571
ENSMMUG00000049788		-1.69189456	0.000128374	0.000717208
ENSMMUG00000006987	RNA polymerase II subunit F [Source:NCBI gene;Acc:699566]	-0.371302112	0.00089752	0.004013368
ENSMMUG00000019785	eukaryotic translation initiation factor 3 subunit L [Source:NCBI gene;Acc:699189]	-1.352258848	0.003124285	0.01177276
ENSMMUG00000015258	glycine C-acetyltransferase [Source:NCBI gene;Acc:698811]	2.376563271	0.00245096	0.009611244
ENSMMUG00000005991	TRIO and F-actin binding protein [Source:NCBI gene;Acc:701241]	0.713679404	1.83E-06	1.56E-05
ENSMMUG00000003005	MFNG O-fucosylpeptide 3-beta-N-acetylglucosaminyltransferase [Source:VGNC Symbol;Acc:VGNC:74712]	4.756211246	1.61E-06	1.39E-05
ENSMMUG00000052785	extracellular leucine rich repeat and fibronectin type III domain containing 2 [Source:NCBI gene;Acc:697071]	3.804705994	0.000374578	0.001857393
ENSMMUG00000052038		-1.743899123	0.009455707	0.030524257
ENSMMUG00000000151	transmembrane serine protease 6 [Source:VGNC Symbol;Acc:VGNC:79038]	0.520529265	3.53E-05	0.000224611
ENSMMUG00000058352		1.325906864	8.33E-05	0.000483643
ENSMMUG00000012350	mercaptopyruvate sulfurtransferase [Source:VGNC Symbol;Acc:VGNC:74922]	0.82377668	0.00772032	0.025733787
ENSMMUG00000012349	thiosulfate sulfurtransferase [Source:VGNC Symbol;Acc:VGNC:78664]	2.144691339	0.00285756	0.010939549
ENSMMUG00000014986	testis expressed 33 [Source:VGNC Symbol;Acc:VGNC:79727]	1.060167943	3.36E-08	4.11E-07
ENSMMUG00000008160	neutrophil cytosolic factor 4 [Source:VGNC Symbol;Acc:VGNC:75141]	1.563409089	0.000392851	0.001942508
ENSMMUG00000055820		1.862526929	9.89E-05	0.000564873
ENSMMUG00000008778	parvalbumin [Source:VGNC Symbol;Acc:VGNC:76627]	1.164577234	1.57E-06	1.36E-05
ENSMMUG00000060967		0.822133193	0.000879688	0.003944953
ENSMMUG00000001537	apolipoprotein L2 [Source:VGNC Symbol;Acc:VGNC:69989]	-0.77797783	7.30E-07	6.82E-06
ENSMMUG00000014010	RNA binding fox-1 homolog 2 [Source:VGNC Symbol;Acc:VGNC:76685]	0.88865185	1.53E-14	5.19E-13
ENSMMUG00000048463	apolipoprotein L5 [Source:HGNC Symbol;Acc:HGNC:14869]	0.838542777	5.01E-08	5.90E-07
ENSMMUG00000056010		2.069340346	0.001166217	0.005042818
ENSMMUG00000059824		-3.316136345	0.001870355	0.007606724
ENSMMUG00000012113	LARGE xylosyl- and glucuronyltransferase 1 [Source:VGNC Symbol;Acc:VGNC:74236]	0.446655635	0.014429406	0.043587483

ENSMMUG00000013626	solute carrier family 5 member 1 [Source:VGNC Symbol;Acc:VGNC:77609]	0.38084847	0.008699834	0.028510815
ENSMMUG00000002182	phosphatidylserine decarboxylase [Source:VGNC Symbol;Acc:VGNC:76005]	-2.347555465	1.72E-06	1.47E-05
ENSMMUG00000002181	SFI1 centrin binding protein [Source:VGNC Symbol;Acc:VGNC:77328]	0.657277114	2.39E-06	1.99E-05
ENSMMUG00000037662	selenoprotein M [Source:NCBI gene;Acc:718290]	-1.845024381	0.004311448	0.015654955
ENSMMUG00000004675	smoothelin [Source:VGNC Symbol;Acc:VGNC:77661]	-1.882380397	4.01E-07	3.96E-06
ENSMMUG00000056113		-2.013128976	0.000992348	0.004382897
ENSMMUG00000005898	MORC family CW-type zinc finger 2 [Source:VGNC Symbol;Acc:VGNC:74810]	-2.127209384	0.006627176	0.022518268
ENSMMUG00000019805	dual specificity phosphatase 18 [Source:VGNC Symbol;Acc:VGNC:71858]	-0.470022938	0.004595665	0.016498977
ENSMMUG00000019804	solute carrier family 35 member E4 [Source:NCBI gene;Acc:716119]	-0.52637486	0.015837499	0.04707541
ENSMMUG00000019803	transcobalamin 2 [Source:VGNC Symbol;Acc:VGNC:78293]	-0.542626349	0.011440375	0.035865947
ENSMMUG00000017562	pescadillo ribosomal biogenesis factor 1 [Source:VGNC Symbol;Acc:VGNC:75944]	0.751829798	0.005264448	0.018492824
ENSMMUG00000052343		-0.92730691	0.002474883	0.009686097
ENSMMUG00000046054	SEC14 like lipid binding 4 [Source:NCBI gene;Acc:715914]	-1.227391607	0.006454892	0.021996828
ENSMMUG00000021374	EWS RNA binding protein 1 [Source:VGNC Symbol;Acc:VGNC:72446]	-0.604599801	0.015765766	0.046919215
ENSMMUG00000058170	U6 spliceosomal RNA [Source:RFAM;Acc:RF00026]	-0.679507361	0.014730418	0.044298792
ENSMMUG00000007215	X-box binding protein 1 [Source:VGNC Symbol;Acc:VGNC:78806]	-0.797062934	0.00822541	0.027184757
ENSMMUG00000020486	checkpoint kinase 2 [Source:VGNC Symbol;Acc:VGNC:70958]	0.642366653	4.83E-06	3.79E-05
ENSMMUG00000001058	tetratricopeptide repeat domain 28 [Source:VGNC Symbol;Acc:VGNC:79785]	2.937961321	3.37E-22	2.60E-20
ENSMMUG00000023450	tyrosylprotein sulfotransferase 2 [Source:VGNC Symbol;Acc:VGNC:78639]	-0.895417863	0.002945377	0.011233971
ENSMMUG00000023446	tuftelin interacting protein 11 [Source:VGNC Symbol;Acc:VGNC:78399]	-1.011850133	7.48E-07	6.97E-06
ENSMMUG00000023443	SRR1 domain containing [Source:VGNC Symbol;Acc:VGNC:78036]	-0.817929073	3.35E-06	2.71E-05
ENSMMUG00000023442	HPS4 biogenesis of lysosomal organelles complex 3 subunit 2 [Source:VGNC Symbol;Acc:VGNC:73396]	-1.5964882	0.009543885	0.030752274
ENSMMUG00000016052	seizure related 6 homolog like [Source:VGNC Symbol;Acc:VGNC:77176]	-0.988906244	3.09E-05	0.000199648
ENSMMUG00000010839	small G protein signaling modulator 1 [Source:VGNC Symbol;Acc:VGNC:77336]	-0.312837111	0.016779628	0.049339636
ENSMMUG00000006853	crystallin beta B2 [Source:NCBI gene;Acc:100144420]	3.492826182	3.25E-17	1.53E-15
ENSMMUG00000044345	ral-GDS-related protein [Source:NCBI gene;Acc:710293]	0.695939817	0.001227768	0.005276429
ENSMMUG00000009106	solute carrier family 2 member 11 [Source:NCBI gene;Acc:699450]	-0.455140069	0.000910145	0.004060757
ENSMMUG00000004552	D-dopachrome tautomerase [Source:NCBI gene;Acc:699933]	0.411774896	0.003737097	0.013798132
ENSMMUG00000020992	calcineurin binding protein 1 [Source:NCBI gene;Acc:700566]	-1.149415905	4.71E-09	6.73E-08
ENSMMUG00000005980	glutathione hydrolase 5 proenzyme [Source:NCBI gene;Acc:720345]	-0.624695667	2.05E-08	2.61E-07
ENSMMUG00000044782		-0.97980983	6.44E-10	1.06E-08
ENSMMUG00000020999	gamma-glutamyltransferase 1 [Source:NCBI gene;Acc:707545]	-0.617643203	0.004887031	0.01739803
ENSMMUG00000000262	beta-ureidopropionase 1 [Source:VGNC Symbol;Acc:VGNC:79029]	-1.032106654	0.005182125	0.018249358

ENSMMUG00000051567		- 0.579375173	0.010321241	0.032834566
ENSMMUG00000051040		1.105116674	0.008618258	0.028289755
ENSMMUG00000049542		1.716253276	4.15E-06	3.31E-05
ENSMMUG00000023622	BCR activator of RhoGEF and GTPase [Source:NCBI gene;Acc:709258]	1.126739114	0.000912609	0.004066576
ENSMMUG00000014641		0.430904564	0.000285708	0.001471347
ENSMMUG00000020092		0.637312696	0.001076223	0.004704451
ENSMMUG00000044861		0.502100115	0.005569665	0.019375692
ENSMMUG00000054655	immunoglobulin lambda variable 3-10 [Source:HGNC Symbol;Acc:HGNC:5897]	- 1.049634549	0.00023055	0.001218164
ENSMMUG00000056950		1.179544497	1.40E-10	2.60E-09
ENSMMUG00000060270		2.311866954	0.016610391	0.04890353
ENSMMUG00000047015	ral-GDS-related protein-like [Source:NCBI gene;Acc:106995524]	- 0.996364242	0.000122926	0.000689239
ENSMMUG00000018798	stromal cell derived factor 2 like 1 [Source:NCBI gene;Acc:697928]	0.442400424	0.016632917	0.048945301
ENSMMUG00000064215	yippee like 1 [Source:NCBI gene;Acc:107000563]	-1.18634688	0.000123195	0.000690471
ENSMMUG00000018531	peptidylprolyl isomerase like 2 [Source:VGNC Symbol;Acc:VGNC:76239]	0.397535634	0.007889253	0.026203457
ENSMMUG00000018823	transmembrane protein 191C [Source:NCBI gene;Acc:697070]	2.465040075	1.34E-05	9.46E-05
ENSMMUG00000056022		0.69351138	6.86E-05	0.000407102
ENSMMUG00000062755		1.037622243	0.002345477	0.00923375
ENSMMUG00000061040		1.597708124	0.006300303	0.02154332
ENSMMUG00000049130		2.247936454	1.49E-31	3.27E-29
ENSMMUG00000063914		2.307250052	1.41E-38	5.65E-36
ENSMMUG00000023263	mediator complex subunit 15 [Source:VGNC Symbol;Acc:VGNC:74511]	2.620648719	1.50E-16	6.48E-15
ENSMMUG00000017171	scavenger receptor class F member 2 [Source:VGNC Symbol;Acc:VGNC:77058]	- 1.071222322	0.009526463	0.03071025
ENSMMUG00000062236		- 0.876040191	0.016984149	0.049888715
ENSMMUG00000011547	reticulon 4 receptor [Source:VGNC Symbol;Acc:VGNC:77047]	0.76954622	0.001026984	0.004515959
ENSMMUG00000021904	cell division cycle 45 [Source:VGNC Symbol;Acc:VGNC:70800]	0.676156904	0.002597788	0.010099467
ENSMMUG00000031106	ubiquitin recognition factor in ER associated degradation 1 [Source:NCBI gene;Acc:711716]	0.93985695	1.89E-12	4.70E-11
ENSMMUG00000009650	HIR histone cell cycle regulation defective homolog A (S. cerevisiae) [Source:NCBI gene;Acc:719142]	- 0.257463644	0.008659328	0.028411267
ENSMMUG00000005432	clathrin heavy chain like 1 [Source:VGNC Symbol;Acc:VGNC:71268]	1.471297425	1.73E-09	2.66E-08
ENSMMUG00000019925	DiGeorge syndrome critical region gene 2 [Source:NCBI gene;Acc:100423715]	1.325640864	1.70E-13	4.87E-12
ENSMMUG00000062702		1.332490071	0.003980294	0.014569477
ENSMMUG00000020799	BH3 interacting domain death agonist [Source:VGNC Symbol;Acc:VGNC:70250]	1.947557794	1.75E-08	2.26E-07
ENSMMUG00000021476	BCL2 like 13 [Source:VGNC Symbol;Acc:VGNC:84544]	2.001463519	7.33E-05	0.000431427
ENSMMUG00000058916		2.715797605	1.93E-13	5.45E-12
ENSMMUG00000021477	ATPase H+ transporting V1 subunit E1 [Source:NCBI gene;Acc:709739]	2.820104656	3.60E-11	7.29E-10
ENSMMUG00000006731	CECR2 histone acetyl-lysine reader [Source:VGNC Symbol;Acc:VGNC:84530]	- 2.051053543	6.23E-15	2.23E-13
ENSMMUG00000059302	Metazoan signal recognition particle RNA [Source:RFAM;Acc:RF00017]	2.029852599	1.70E-07	1.81E-06
ENSMMUG00000022222	interleukin 17 receptor A [Source:VGNC Symbol;Acc:VGNC:73712]	0.346564262	0.004980049	0.017661919
ENSMMUG00000009953	ninein like [Source:VGNC Symbol;Acc:VGNC:75273]	1.608572128	1.28E-08	1.68E-07
ENSMMUG00000002397	GINS complex subunit 1 [Source:VGNC Symbol;Acc:VGNC:73025]	1.876801177	1.41E-12	3.57E-11
ENSMMUG00000059374		1.813211946	0.000105269	0.000597648
ENSMMUG00000059370	Metazoan signal recognition particle RNA [Source:RFAM;Acc:RF00017]	2.712101038	0.015957058	0.047367871
ENSMMUG00000009201	abhydrolase domain containing 12 [Source:VGNC Symbol;Acc:VGNC:69411]	1.680784728	2.01E-11	4.27E-10
ENSMMUG00000060399	Metazoan signal recognition particle RNA [Source:RFAM;Acc:RF00017]	1.470329807	0.000265151	0.001381211
ENSMMUG00000009199	glycogen phosphorylase B [Source:VGNC Symbol;Acc:VGNC:76631]	1.765374751	2.71E-11	5.63E-10
ENSMMUG00000024668	Y RNA [Source:RFAM;Acc:RF00019]	3.205809246	0.005328968	0.018677361

ENSMMUG00000052572		1.477179059	0.001412637	0.00594877
ENSMMUG00000058095	visual system homeobox 1 [Source:NCBI gene;Acc:715973]	1.414361394	0.005443991	0.019028188
ENSMMUG00000052227		1.811499081	0.000268486	0.001394491
ENSMMUG00000020597	acyl-CoA synthetase short chain family member 1 [Source:VGNC Symbol;Acc:VGNC:69416]	1.111987251	0.000175576	0.000950983
ENSMMUG00000061222	adipocyte plasma membrane associated protein [Source:NCBI gene;Acc:704948]	1.131669271	2.08E-05	0.000140378
ENSMMUG00000049391		2.497424505	6.29E-07	5.95E-06
ENSMMUG00000053965		-0.711729449	0.004517644	0.016260504
ENSMMUG00000042668	cilia and flagella associated protein 61 [Source:NCBI gene;Acc:699814]	-1.055350207	6.44E-05	0.000385147
ENSMMUG00000007709	crooked neck pre-mRNA splicing factor 1 [Source:VGNC Symbol;Acc:VGNC:71412]	-1.155208468	1.01E-11	2.28E-10
ENSMMUG00000010114	Ras and Rab interactor 2 [Source:VGNC Symbol;Acc:VGNC:76770]	-0.698737493	0.001252442	0.005366718
ENSMMUG00000002321	solute carrier family 24 member 3 [Source:VGNC Symbol;Acc:VGNC:77426]	-1.318646139	1.85E-06	1.57E-05
ENSMMUG00000002202	SEL1L2 adaptor subunit of ERAD E3 ligase [Source:VGNC Symbol;Acc:VGNC:77239]	0.690646165	0.002262869	0.008951257
ENSMMUG00000062485	ESF1 nucleolar pre-rRNA processing protein homolog [Source:NCBI gene;Acc:694159]	0.898445184	0.000714097	0.003288598
ENSMMUG00000011064	synaptosome associated protein 25 [Source:VGNC Symbol;Acc:VGNC:77663]	3.739306564	9.60E-17	4.25E-15
ENSMMUG00000015923	ankyrin repeat and EF-hand domain containing 1 [Source:VGNC Symbol;Acc:VGNC:69615]	-1.081046969	9.24E-05	0.000531805
ENSMMUG00000065218		0.444884789	0.004606639	0.016534145
ENSMMUG00000021137	thioredoxin related transmembrane protein 4 [Source:VGNC Symbol;Acc:VGNC:79484]	-0.949176165	0.014974234	0.04490669
ENSMMUG00000055897		1.548585188	6.62E-10	1.09E-08
ENSMMUG00000057687		2.054055248	5.35E-20	3.23E-18
ENSMMUG00000064554	leucine rich repeat neuronal 4 [Source:NCBI gene;Acc:718283]	2.293347463	2.09E-07	2.18E-06
ENSMMUG00000056093	shieldin complex subunit 1 [Source:NCBI gene;Acc:718152]	1.295806796	8.53E-07	7.83E-06
ENSMMUG00000022345		0.998531523	0.002849119	0.010914552
ENSMMUG00000050080	Metazoan signal recognition particle RNA [Source:RFAM;Acc:RF00017]	1.515082301	0.001335564	0.00566504
ENSMMUG00000064391		1.388311197	3.67E-13	1.00E-11
ENSMMUG00000013261	CDP-diacylglycerol synthase 2 [Source:VGNC Symbol;Acc:VGNC:70995]	-1.011416374	0.007734138	0.025767598
ENSMMUG00000020004	Ras association domain family member 2 [Source:VGNC Symbol;Acc:VGNC:76674]	-0.670729562	0.001822416	0.007424677
ENSMMUG00000051480		0.82030407	0.001640254	0.006765082
ENSMMUG00000017150	mitochondrial antiviral signaling protein [Source:VGNC Symbol;Acc:VGNC:74609]	0.753545062	0.008878807	0.028955235
ENSMMUG00000018922	cell division cycle 25B [Source:VGNC Symbol;Acc:VGNC:70795]	0.311181637	0.003374149	0.012600717
ENSMMUG00000022070	sialic acid binding Ig like lectin 1 [Source:VGNC Symbol;Acc:VGNC:77220]	0.504388081	5.04E-05	0.00030939
ENSMMUG00000003633	solute carrier family 4 member 11 [Source:VGNC Symbol;Acc:VGNC:77721]	0.379569899	1.50E-09	2.31E-08
ENSMMUG00000061481	inosine triphosphatase [Source:NCBI gene;Acc:717257]	0.364060038	2.31E-06	1.92E-05
ENSMMUG00000010028	leucine zipper tumor suppressor family member 3 [Source:VGNC Symbol;Acc:VGNC:74403]	1.663808906	1.33E-20	8.86E-19
ENSMMUG00000043281	U-box domain containing 5 [Source:NCBI gene;Acc:717166]	1.651615593	0.01376462	0.041915583
ENSMMUG00000002982	FAST kinase domains 5 [Source:NCBI gene;Acc:100498658]	0.54586704	0.000904244	0.004037858
ENSMMUG00000039757	Metazoan signal recognition particle RNA [Source:RFAM;Acc:RF00017]	2.640384657	0.006342387	0.02166081
ENSMMUG00000010678	oxytocin/neurophysin I prepropeptide [Source:VGNC Symbol;Acc:VGNC:75730]	1.549102446	2.63E-05	0.000172295
ENSMMUG00000063622	mitochondrial ribosomal protein S26 [Source:NCBI gene;Acc:717038]	2.564591956	1.64E-12	4.11E-11
ENSMMUG00000005878	protein tyrosine phosphatase receptor type A [Source:VGNC Symbol;Acc:VGNC:76610]	0.553983693	1.25E-05	8.86E-05
ENSMMUG00000005204	VPS16 core subunit of CORVET and HOPS complexes [Source:VGNC Symbol;Acc:VGNC:78966]	-1.783699505	1.67E-05	0.000115104
ENSMMUG00000005200	PC-esterase domain containing 1A [Source:VGNC Symbol;Acc:VGNC:75622]	-2.441931809	0.002217806	0.008818964

ENSMMUG00000021332	carboxypeptidase X, M14 family member 1 [Source:VGNC Symbol;Acc:VGNC:71413]	-3.702793965	0.002840296	0.010889529
ENSMMUG00000064862		-3.014797144	0.008673382	0.028444068
ENSMMUG00000021331	EBF family member 4 [Source:VGNC Symbol;Acc:VGNC:71932]	-3.123082919	3.83E-06	3.07E-05
ENSMMUG00000061433		-0.967608076	5.89E-05	0.00035589
ENSMMUG00000013888	NOP56 ribonucleoprotein [Source:VGNC Symbol;Acc:VGNC:75181]	-1.055715851	0.005204574	0.018314617
ENSMMUG00000010050	small nuclear ribonucleoprotein polypeptides B and B1 [Source:VGNC Symbol;Acc:VGNC:77676]	-3.098146487	4.77E-08	5.63E-07
ENSMMUG00000018474	signal regulatory protein alpha [Source:NCBI gene;Acc:717811]	-1.712701915	8.49E-10	1.37E-08
ENSMMUG00000018473	signal-regulatory protein gamma [Source:NCBI gene;Acc:716034]	-2.856373747	0.015393625	0.045948234
ENSMMUG00000018165	syndecan binding protein 2 [Source:VGNC Symbol;Acc:VGNC:77017]	-1.703540198	0.004650455	0.016670073
ENSMMUG00000018159	syntaphilin [Source:NCBI gene;Acc:100429237]	0.747839589	4.22E-05	0.000264487
ENSMMUG00000004711	transmembrane protein 74B [Source:VGNC Symbol;Acc:VGNC:78990]	-1.936781516	0.001932726	0.007819567
ENSMMUG00000004709	proteasome inhibitor subunit 1 [Source:VGNC Symbol;Acc:VGNC:76458]	-1.464612105	0.005857842	0.020222679
ENSMMUG00000021670	solute carrier family 52 member 3 [Source:VGNC Symbol;Acc:VGNC:77717]	-1.05118954	3.45E-05	0.000220485
ENSMMUG00000056387	transcription factor 15 [Source:NCBI gene;Acc:716916]	-2.619212428	1.53E-15	5.84E-14
ENSMMUG00000031224	casein kinase 2 alpha 1 [Source:NCBI gene;Acc:714841]	-0.830245428	0.000174884	0.000947604
ENSMMUG00000012601	RANBP2-type and C3HC4-type zinc finger containing 1 [Source:VGNC Symbol;Acc:VGNC:81554]	-0.594977235	0.0001857	0.001000794
ENSMMUG00000017286	chromosome 10 C20orf96 homolog [Source:VGNC Symbol;Acc:VGNC:70324]	-0.949908234	0.008760079	0.028661359
ENSMMUG00000061092	defensin beta 125 [Source:HGNC Symbol;Acc:HGNC:18105]	-0.504695514	0.003790027	0.013949487
ENSMMUG00000046141	defensin beta 123 [Source:NCBI gene;Acc:619504]	0.607537518	0.002651393	0.010265179
ENSMMUG00000005116	defensin beta 124 [Source:VGNC Symbol;Acc:VGNC:71655]	0.708566556	0.000131415	0.000731575
ENSMMUG00000058583	RRAD and GEM like GTPase 1 [Source:NCBI gene;Acc:713351]	-1.021239267	2.00E-10	3.62E-09
ENSMMUG00000005119	histocompatibility minor 13 [Source:VGNC Symbol;Acc:VGNC:81418]	-1.3872195	0.00833525	0.027489466
ENSMMUG00000051112	p53 and DNA damage regulated 1 [Source:NCBI gene;Acc:712622]	-1.424195793	3.93E-07	3.89E-06
ENSMMUG00000000117	HCK proto-oncogene, Src family tyrosine kinase [Source:VGNC Symbol;Acc:VGNC:73353]	-0.913906019	0.006069053	0.020859393
ENSMMUG00000053133	BPI fold containing family B member 2 [Source:NCBI gene;Acc:710915]	0.685894636	0.002361707	0.009289821
ENSMMUG00000020676	BPI fold containing family B member 4 [Source:VGNC Symbol;Acc:VGNC:70321]	0.509658873	0.003082235	0.011653323
ENSMMUG00000012912	itchy E3 ubiquitin protein ligase [Source:VGNC Symbol;Acc:VGNC:73589]	-0.620344232	5.40E-10	9.00E-09
ENSMMUG00000055449	tumor protein p53 inducible nuclear protein 2 [Source:NCBI gene;Acc:707113]	-1.990524149	0.008258212	0.027286735
ENSMMUG00000017931	gamma-glutamyltransferase 7 [Source:VGNC Symbol;Acc:VGNC:72921]	-1.554140217	8.08E-05	0.000472236
ENSMMUG00000017932	acyl-CoA synthetase short chain family member 2 [Source:VGNC Symbol;Acc:VGNC:69410]	-2.209820595	0.00152968	0.006362997
ENSMMUG00000000229	transient receptor potential cation channel subfamily C member 4 associated protein [Source:VGNC Symbol;Acc:VGNC:79014]	-2.753414854	2.82E-05	0.000183443
ENSMMUG00000057979	growth differentiation factor 5 [Source:NCBI gene;Acc:705432]	0.80333605	3.85E-07	3.83E-06

ENSMMUG00000031274	GDF5 antisense RNA 1 [Source:HGNC Symbol;Acc:HGNC:33435]	0.683366136	0.002659392	0.010293307
ENSMMUG00000031284	RNA binding motif protein 39 [Source:NCBI gene;Acc:704198]	2.902916197	0.000657062	0.003055032
ENSMMUG00000010633	PHD finger protein 20 [Source:VGNC Symbol;Acc:VGNC:75807]	0.45787917	1.07E-05	7.70E-05
ENSMMUG00000020690	erythrocyte membrane protein band 4.1 like 1 [Source:VGNC Symbol;Acc:VGNC:72231]	-0.550630946	0.000344453	0.00172818
ENSMMUG00000020691	AAR2 splicing factor [Source:VGNC Symbol;Acc:VGNC:69412]	-0.5337775	0.003146332	0.01184139
ENSMMUG00000016448	DLG associated protein 4 [Source:NCBI gene;Acc:702928]	1.28781545	4.91E-09	6.99E-08
ENSMMUG00000059897	myosin light chain 9 [Source:NCBI gene;Acc:709784]	1.191609431	0.000594239	0.002816159
ENSMMUG00000008195	TGFB induced factor homeobox 2 [Source:NCBI gene;Acc:100429616]	1.634019411	1.33E-22	1.08E-20
ENSMMUG00000056945		1.60095375	8.32E-18	4.09E-16
ENSMMUG00000008200	Src like adaptor 2 [Source:VGNC Symbol;Acc:VGNC:77499]	1.661596374	1.77E-08	2.29E-07
ENSMMUG00000010363	ribophorin II [Source:VGNC Symbol;Acc:VGNC:76925]	1.227105099	9.48E-05	0.000543813
ENSMMUG00000017224	catenin beta like 1 [Source:VGNC Symbol;Acc:VGNC:71414]	1.306888806	1.84E-05	0.000125272
ENSMMUG00000017230	V-set and transmembrane domain containing 2 like [Source:NCBI gene;Acc:707394]	-1.225388838	5.89E-11	1.16E-09
ENSMMUG00000020172	KIAA1755 [Source:VGNC Symbol;Acc:VGNC:73942]	0.672899636	3.07E-05	0.00019887
ENSMMUG00000020173	bactericidal permeability increasing protein [Source:VGNC Symbol;Acc:VGNC:70277]	0.565622518	0.000318741	0.001617116
ENSMMUG00000031309	adipogenin [Source:VGNC Symbol;Acc:VGNC:84540]	-0.689664255	0.005076107	0.017925521
ENSMMUG00000006738	L3MBTL histone methyl-lysine binding protein 1 [Source:NCBI gene;Acc:695694]	1.902834336	5.33E-15	1.92E-13
ENSMMUG00000007925	serum/glucocorticoid regulated kinase 2 [Source:VGNC Symbol;Acc:VGNC:77228]	2.231681746	1.54E-10	2.84E-09
ENSMMUG00000000396	intraflagellar transport 52 [Source:NCBI gene;Acc:694933]	2.115520258	3.32E-12	7.96E-11
ENSMMUG00000018137	MYB proto-oncogene like 2 [Source:VGNC Symbol;Acc:VGNC:75092]	2.254797357	1.23E-20	8.24E-19
ENSMMUG00000002810	TOX high mobility group box family member 2 [Source:VGNC Symbol;Acc:VGNC:79007]	1.335936575	5.29E-14	1.65E-12
ENSMMUG00000022865	junctophilin 2 [Source:NCBI gene;Acc:694158]	-3.923969682	2.31E-06	1.92E-05
ENSMMUG00000006464	hepatocyte nuclear factor 4 alpha [Source:VGNC Symbol;Acc:VGNC:73492]	-0.854412506	0.001581517	0.006551732
ENSMMUG00000063590		0.758651159	0.001489544	0.006220333
ENSMMUG00000045256	cellular communication network factor 5 [Source:NCBI gene;Acc:717872]	1.697969482	3.46E-06	2.80E-05
ENSMMUG00000013206	tyrosine 3-monooxygenase/tryptophan 5-monooxygenase activation protein beta [Source:VGNC Symbol;Acc:VGNC:78819]	1.105430133	0.004238901	0.015407494
ENSMMUG00000063049		-0.217209666	0.00500257	0.017719383
ENSMMUG00000039638	serine peptidase inhibitor, Kunitz type 3 [Source:NCBI gene;Acc:100423908]	0.316445375	0.014408672	0.043555252
ENSMMUG00000021022	deoxynucleotidyltransferase terminal interacting protein 1 [Source:VGNC Symbol;Acc:VGNC:71845]	0.921440108	0.001075193	0.004701414
ENSMMUG00000052867		1.149581783	6.71E-06	5.09E-05
ENSMMUG00000051201	zinc finger SWIM-type containing 3 [Source:NCBI gene;Acc:708804]	1.215295817	1.07E-07	1.19E-06
ENSMMUG00000058326	neuralized E3 ubiquitin protein ligase 2 [Source:NCBI gene;Acc:708904]	2.230022761	0.000600246	0.002843666
ENSMMUG00000009960	cathepsin A [Source:VGNC Symbol;Acc:VGNC:71617]	1.522448447	2.57E-14	8.44E-13
ENSMMUG00000013697	PDX1 C-terminal inhibiting factor 1 [Source:VGNC Symbol;Acc:VGNC:75621]	0.857395139	1.48E-09	2.30E-08
ENSMMUG00000013702	zinc finger protein 335 [Source:VGNC Symbol;Acc:VGNC:78986]	-1.780768722	0.00365146	0.013513967
ENSMMUG00000016549	matrix metallopeptidase 9 [Source:VGNC Symbol;Acc:VGNC:74903]	3.692483859	1.10E-22	9.05E-21
ENSMMUG00000016553	solute carrier family 12 member 5 [Source:VGNC Symbol;Acc:VGNC:77471]	4.483613695	7.24E-05	0.000427335
ENSMMUG00000016927	solute carrier family 2 member 10 [Source:VGNC Symbol;Acc:VGNC:77465]	-1.134075725	6.39E-12	1.47E-10
ENSMMUG00000065008		-0.984465377	0.000160529	0.000877621
ENSMMUG00000015710	zinc finger MYND-type containing 8 [Source:NCBI gene;Acc:716058]	0.475532984	0.001164548	0.005037153
ENSMMUG00000012020	nuclear receptor coactivator 3 [Source:VGNC Symbol;Acc:VGNC:75147]	-0.639676217	1.31E-06	1.15E-05
ENSMMUG00000012029	sulfatase 2 [Source:VGNC Symbol;Acc:VGNC:78179]	-1.099525898	2.79E-07	2.84E-06

ENSMMUG00000060780		0.592933668	3.87E-06	3.10E-05
ENSMMUG00000055085		0.809797287	0.01235461	0.038177324
ENSMMUG00000058754		-2.484507965	0.011618434	0.036334814
ENSMMUG00000021692	prostaglandin I2 synthase [Source:VGNC Symbol;Acc:VGNC:76587]	0.391341622	0.005856053	0.02022148
ENSMMUG00000002124	beta-1,4-galactosyltransferase 5 [Source:VGNC Symbol;Acc:VGNC:70212]	1.366539313	6.84E-06	5.18E-05
ENSMMUG00000002715	solute carrier family 9 member A8 [Source:VGNC Symbol;Acc:VGNC:77749]	1.915847302	8.74E-31	1.78E-28
ENSMMUG00000028471	U7 small nuclear RNA [Source:RFAM;Acc:RF00066]	1.33581746	3.25E-05	0.000208476
ENSMMUG00000000740	spermatogenesis associated 2 [Source:VGNC Symbol;Acc:VGNC:77923]	1.536947741	2.94E-08	3.63E-07
ENSMMUG00000000757	ring finger protein 114 [Source:VGNC Symbol;Acc:VGNC:76725]	1.104224182	0.005009113	0.01773808
ENSMMUG00000065129		2.280500092	0.001061052	0.004647717
ENSMMUG00000012228	ubiquitin conjugating enzyme E2 V1 [Source:NCBI gene;Acc:100425552]	1.826275083	3.56E-22	2.73E-20
ENSMMUG00000062321		-0.466321877	0.012666536	0.038995133
ENSMMUG00000002761	activity dependent neuroprotector homeobox [Source:VGNC Symbol;Acc:VGNC:69614]	-0.439775017	0.005924888	0.020408917
ENSMMUG00000018846	ZFP64 zinc finger protein [Source:VGNC Symbol;Acc:VGNC:78994]	-0.520097553	1.00E-07	1.12E-06
ENSMMUG00000013035	teashirt zinc finger homeobox 2 [Source:VGNC Symbol;Acc:VGNC:78875]	-0.902682587	0.001216616	0.005238133
ENSMMUG00000018358	zinc finger protein 217 [Source:VGNC Symbol;Acc:VGNC:78874]	-2.427659979	1.18E-16	5.18E-15
ENSMMUG00000057302		-2.34820288	1.43E-05	1.00E-04
ENSMMUG00000052112		1.261212867	5.88E-05	0.00035559
ENSMMUG00000004074	bone morphogenetic protein 7 [Source:VGNC Symbol;Acc:VGNC:70266]	0.924414791	0.000157584	0.00086354
ENSMMUG00000031391	RNA binding motif protein 38 [Source:VGNC Symbol;Acc:VGNC:76484]	1.838701864	4.34E-05	0.000270707
ENSMMUG00000022586	phosphoenolpyruvate carboxykinase 1 [Source:VGNC Symbol;Acc:VGNC:75779]	3.378164949	0.006037967	0.020772917
ENSMMUG00000022589	prostate transmembrane protein, androgen induced 1 [Source:VGNC Symbol;Acc:VGNC:76023]	-1.029232407	2.84E-05	0.000184314
ENSMMUG00000004560	crystallin lambda 1 [Source:VGNC Symbol;Acc:VGNC:71433]	-0.42883677	0.010639432	0.033678725
ENSMMUG00000016475	M-phase phosphoprotein 8 [Source:VGNC Symbol;Acc:VGNC:74864]	-0.696556984	2.01E-05	0.00013652
ENSMMUG00000004237	TNF receptor superfamily member 19 [Source:VGNC Symbol;Acc:VGNC:78588]	-1.113853057	3.51E-13	9.64E-12
ENSMMUG00000014610	ring finger protein 17 [Source:NCBI gene;Acc:708198]	-0.559394806	0.001342531	0.005685991
ENSMMUG00000057852		-0.741013493	0.001686873	0.006935281
ENSMMUG00000057481		3.975578033	2.80E-07	2.85E-06
ENSMMUG00000058786	ligand of numb-protein X 2 [Source:NCBI gene;Acc:710660]	-0.758582759	0.000213365	0.001134703
ENSMMUG00000052070	caudal type homeobox 2 [Source:NCBI gene;Acc:711094]	-0.623413943	0.014984011	0.044926402
ENSMMUG00000016138	ureidoimidazoline (2-oxo-4-hydroxy-4-carboxy-5-) decarboxylase [Source:NCBI gene;Acc:721710]	-1.378215846	0.003193051	0.011991472
ENSMMUG00000063649		0.61103884	0.003382303	0.012617728
ENSMMUG00000008658	microtubule associated scaffold protein 2 [Source:NCBI gene;Acc:721776]	-0.656906319	3.61E-05	0.000229716
ENSMMUG00000003771	ubiquitin like 3 [Source:VGNC Symbol;Acc:VGNC:78701]	-1.179596062	0.000779224	0.003561623
ENSMMUG00000061779		-0.920856679	2.46E-05	0.000162765
ENSMMUG00000050015	Metazoan signal recognition particle RNA [Source:RFAM;Acc:RF00017]	-1.329905013	0.005338067	0.018699902

ENSMMUG00000013707	heat shock protein family H (Hsp110) member 1 [Source:NCBI gene;Acc:721956]	-1.012410233	6.14E-10	1.01E-08
ENSMMUG00000013713	beta 3-glucosyltransferase [Source:NCBI gene;Acc:713498]	2.005682287	1.08E-07	1.19E-06
ENSMMUG00000007192	FRY microtubule binding protein [Source:NCBI gene;Acc:713688]	1.128354353	0.001216251	0.005238133
ENSMMUG00000038997	zygote arrest 1 like [Source:NCBI gene;Acc:100427970]	2.790991128	9.60E-05	0.000550098
ENSMMUG00000007197	BRCA2 DNA repair associated [Source:VGNC Symbol;Acc:VGNC:70279]	0.960715405	3.17E-05	0.00020426
ENSMMUG00000007198	NEDD4 binding protein 2 like 1 [Source:VGNC Symbol;Acc:VGNC:75017]	1.533031339	0.001185615	0.005115937
ENSMMUG00000007200	NEDD4 binding protein 2 like 2 [Source:VGNC Symbol;Acc:VGNC:75031]	2.031810121	7.86E-29	1.25E-26
ENSMMUG00000049600		1.116885549	0.007379944	0.024734453
ENSMMUG00000055811		2.137397895	1.40E-15	5.40E-14
ENSMMUG00000007201	PDS5 cohesin associated factor B [Source:VGNC Symbol;Acc:VGNC:75835]	1.809353988	2.07E-30	3.82E-28
ENSMMUG00000025050	Y RNA [Source:RFAM;Acc:RF00019]	2.440710254	1.36E-05	9.57E-05
ENSMMUG00000017048	klotho [Source:VGNC Symbol;Acc:VGNC:74026]	-0.790398528	2.26E-06	1.89E-05
ENSMMUG00000063615		-0.642459171	1.00E-06	9.07E-06
ENSMMUG00000017052	StAR related lipid transfer domain containing 13 [Source:VGNC Symbol;Acc:VGNC:77962]	-0.399124285	0.00016865	0.000917368
ENSMMUG00000019831	neurobeachin [Source:NCBI gene;Acc:714386]	-2.740881727	2.15E-12	5.30E-11
ENSMMUG00000016791	doublecortin like kinase 1 [Source:VGNC Symbol;Acc:VGNC:71745]	-2.647892143	1.31E-27	1.89E-25
ENSMMUG00000060579		-2.358230548	3.73E-06	3.00E-05
ENSMMUG00000001172	spermatogenesis and oogenesis specific basic helix-loop-helix 2 [Source:NCBI gene;Acc:693608]	-2.545104914	2.84E-08	3.52E-07
ENSMMUG00000003585	ALG5 dolichyl-phosphate beta-glucosyltransferase [Source:VGNC Symbol;Acc:VGNC:69647]	0.671292957	0.000619036	0.002911046
ENSMMUG00000012307	periostin [Source:VGNC Symbol;Acc:VGNC:76039]	-0.926329328	1.41E-10	2.62E-09
ENSMMUG00000030786	transient receptor potential cation channel subfamily C member 4 [Source:NCBI gene;Acc:695636]	-0.527102139	3.27E-05	0.000209889
ENSMMUG00000002113	NHL repeat containing 3 [Source:VGNC Symbol;Acc:VGNC:75291]	-1.57267562	2.58E-11	5.40E-10
ENSMMUG00000002109	proline and serine rich 1 [Source:VGNC Symbol;Acc:VGNC:76291]	-1.16021162	5.49E-10	9.13E-09
ENSMMUG00000052676		-1.106003276	1.27E-09	1.99E-08
ENSMMUG00000011593	regulator of cell cycle [Source:VGNC Symbol;Acc:VGNC:76783]	-1.09466076	1.60E-09	2.46E-08
ENSMMUG00000023076	diacylglycerol kinase eta [Source:VGNC Symbol;Acc:VGNC:71786]	-1.016128802	3.73E-05	0.00023675
ENSMMUG00000055887		-0.989301888	0.003622363	0.01342045
ENSMMUG00000023083	A-kinase anchoring protein 11 [Source:VGNC Symbol;Acc:VGNC:69765]	-1.496873791	1.53E-07	1.64E-06
ENSMMUG00000014926	epithelial stromal interaction 1 [Source:VGNC Symbol;Acc:VGNC:72053]	-0.591387914	0.004684072	0.016781997
ENSMMUG00000002330	ecto-NOX disulfide-thiol exchanger 1 [Source:VGNC Symbol;Acc:VGNC:72110]	1.199337486	0.000135663	0.000752832
ENSMMUG00000050436		3.318712201	0.003402819	0.012687513
ENSMMUG00000011986	chibby family member 2 [Source:VGNC Symbol;Acc:VGNC:78029]	-1.18091294	4.12E-05	0.00025845
ENSMMUG00000017025	zinc finger CCCH-type containing 13 [Source:VGNC Symbol;Acc:VGNC:79248]	-0.881225962	6.47E-08	7.49E-07
ENSMMUG00000065094		1.310755838	0.001598424	0.00661591
ENSMMUG00000059778		1.812017512	3.74E-05	0.000237257
ENSMMUG00000063377	cysteinyl leukotriene receptor 2 [Source:NCBI gene;Acc:705450]	-0.882094053	0.011598699	0.036281186
ENSMMUG00000023756	fibronectin type III domain containing 3A [Source:VGNC Symbol;Acc:VGNC:72539]	-0.333967924	0.002244523	0.008898771

ENSMMUG00000013476	PHD finger protein 11 [Source:VGNC Symbol;Acc:VGNC:75814]	-2.08490054	0.006964861	0.023506195
ENSMMUG00000018720	cytoskeleton associated protein 2 [Source:VGNC Symbol;Acc:VGNC:71229]	1.1151594	4.42E-10	7.48E-09
ENSMMUG00000007379	phosphatidylinositol 3,4,5-trisphosphate 3-phosphatase TPTE2-like [Source:NCBI gene;Acc:694255]	1.118098176	4.95E-10	8.30E-09
ENSMMUG00000053708	U2 spliceosomal RNA [Source:RFAM;Acc:RF00004]	0.960030523	0.011829025	0.03686187
ENSMMUG00000016401	SGT1 homolog, MIS12 kinetochore complex assembly cochaperone [Source:NCBI gene;Acc:694046]	0.952538733	3.34E-08	4.09E-07
ENSMMUG00000016404	chondromodulin [Source:VGNC Symbol;Acc:VGNC:71191]	2.475506131	5.19E-05	0.000318311
ENSMMUG00000002657	olfactomedin 4 [Source:VGNC Symbol;Acc:VGNC:75418]	-1.055363608	0.000153332	0.000842877
ENSMMUG00000054675		-0.728492612	0.002604205	0.01012161
ENSMMUG00000021197	TBC1 domain family member 4 [Source:VGNC Symbol;Acc:VGNC:78166]	-1.484689713	0.000269678	0.001398556
ENSMMUG00000046375		1.053210056	0.003095991	0.011695873
ENSMMUG00000061846		-0.670474883	1.83E-05	0.000125072
ENSMMUG00000059231		-0.615904694	3.07E-08	3.77E-07
ENSMMUG00000056466		-0.79139472	1.24E-06	1.09E-05
ENSMMUG00000021459	MYC binding protein 2 [Source:VGNC Symbol;Acc:VGNC:75011]	-1.514619345	3.78E-15	1.39E-13
ENSMMUG00000054625		-0.645475127	8.85E-05	0.000511838
ENSMMUG00000049796		0.829268887	0.012601553	0.038829175
ENSMMUG00000060359		-3.207020282	9.11E-05	0.000525305
ENSMMUG00000021817	dopachrome tautomerase [Source:VGNC Symbol;Acc:VGNC:71749]	-0.568869987	0.002127948	0.008513613
ENSMMUG00000054711		-1.210263788	1.07E-07	1.19E-06
ENSMMUG00000021807	ATP binding cassette subfamily C member 4 [Source:VGNC Symbol;Acc:VGNC:69573]	-0.773960289	0.008065047	0.026711422
ENSMMUG00000009721	claudin 10 [Source:VGNC Symbol;Acc:VGNC:71243]	-0.919991768	0.01052522	0.033385017
ENSMMUG00000022744	muscleblind like splicing regulator 2 [Source:VGNC Symbol;Acc:VGNC:74519]	0.655365572	3.77E-05	0.000238881
ENSMMUG00000009890	FERM, ARH/RhoGEF and pleckstrin domain protein 1 [Source:VGNC Symbol;Acc:VGNC:72614]	1.192085408	3.99E-24	3.81E-22
ENSMMUG00000009173	serine/threonine kinase 24 [Source:VGNC Symbol;Acc:VGNC:81583]	0.591859323	2.85E-05	0.000185209
ENSMMUG00000001402	solute carrier family 15 member 1 [Source:VGNC Symbol;Acc:VGNC:77393]	-2.35849888	0.00114181	0.004946433
ENSMMUG00000012332	UBA domain containing 2 [Source:NCBI gene;Acc:703484]	-0.6243226	1.44E-05	0.000100636
ENSMMUG00000060781	G protein-coupled receptor 18 [Source:NCBI gene;Acc:698714]	-0.683968048	0.004849967	0.01728363
ENSMMUG00000012338	G protein-coupled receptor 183 [Source:VGNC Symbol;Acc:VGNC:73042]	-0.613091327	0.002434961	0.009556524
ENSMMUG00000004493	propionyl-CoA carboxylase subunit alpha [Source:VGNC Symbol;Acc:VGNC:75773]	-0.625398193	0.010120759	0.032314071
ENSMMUG00000002875	fibroblast growth factor 14 [Source:VGNC Symbol;Acc:VGNC:72645]	-3.727224589	0.012296811	0.038040633
ENSMMUG00000052642	protein O-glucosyltransferase 2 [Source:NCBI gene;Acc:702156]	-1.890414435	1.83E-05	0.000124722
ENSMMUG00000000350	basic, immunoglobulin-like variable motif containing [Source:NCBI gene;Acc:100426213]	-0.655733919	0.014421549	0.043575388
ENSMMUG00000000353	putative methyltransferase-like protein 21E pseudogene [Source:NCBI gene;Acc:707949]	-0.823218592	0.000559685	0.002667728
ENSMMUG00000053838		-0.389926041	0.006398363	0.021827554
ENSMMUG00000022282	collagen type IV alpha 2 chain [Source:NCBI gene;Acc:100429807]	4.03773363	4.25E-07	4.17E-06

ENSMMUG00000022818	inhibitor of growth family member 1 [Source:VGNC Symbol;Acc:VGNC:73742]	3.985354432	0.000212778	0.001132368
ENSMMUG00000064401		3.074749266	0.000211855	0.00112799
ENSMMUG00000018929	ankyrin repeat domain 10 [Source:VGNC Symbol;Acc:VGNC:69646]	-0.440627828	4.68E-05	0.00028967
ENSMMUG00000013138	Rho guanine nucleotide exchange factor 7 [Source:VGNC Symbol;Acc:VGNC:69833]	-0.768440642	1.65E-06	1.42E-05
ENSMMUG00000056306		-1.121295792	4.53E-08	5.39E-07
ENSMMUG00000048566	MCF.2 cell line derived transforming sequence like [Source:NCBI gene;Acc:713961]	1.542606453	1.54E-23	1.39E-21
ENSMMUG00000001036	coagulation factor VII [Source:VGNC Symbol;Acc:VGNC:72466]	1.606454637	1.40E-12	3.56E-11
ENSMMUG00000016740	defective in cullin neddylation 1 domain containing 2 [Source:VGNC Symbol;Acc:VGNC:71684]	-0.516526504	3.30E-08	4.04E-07
ENSMMUG00000007423	transcription factor Dp-1 [Source:NCBI gene;Acc:715345]	1.70249809	6.03E-16	2.46E-14
ENSMMUG00000051400		-0.3818803	0.002466721	0.009659545
ENSMMUG00000023599	chromosome alignment maintaining phosphoprotein 1 [Source:VGNC Symbol;Acc:VGNC:71061]	0.978278477	2.04E-11	4.33E-10
ENSMMUG00000003042	VPS53 subunit of GARP complex [Source:VGNC Symbol;Acc:VGNC:79522]	0.831559613	0.000216394	0.001149431
ENSMMUG00000055344	TLC domain containing 3A [Source:NCBI gene;Acc:699707]	-1.448677108	2.62E-09	3.89E-08
ENSMMUG00000030401	gem nuclear organelle associated protein 4 [Source:VGNC Symbol;Acc:VGNC:72723]	-0.785690785	0.002740432	0.010563243
ENSMMUG00000050361		-0.624340751	0.011906964	0.03705534
ENSMMUG00000011417	glyoxalase domain containing 4 [Source:VGNC Symbol;Acc:VGNC:72939]	-0.576795159	0.000913292	0.004068326
ENSMMUG00000008130	ABR activator of RhoGEF and GTPase [Source:VGNC Symbol;Acc:VGNC:69589]	0.620823565	2.30E-09	3.47E-08
ENSMMUG00000002371	myosin IC [Source:VGNC Symbol;Acc:VGNC:75112]	0.616280641	0.000257414	0.0013449
ENSMMUG00000055241		0.814090724	1.61E-08	2.09E-07
ENSMMUG00000040268	phosphatidylinositol transfer protein alpha [Source:NCBI gene;Acc:721228]	0.736910899	0.00054237	0.002593131
ENSMMUG00000017561	solute carrier family 43 member 2 [Source:VGNC Symbol;Acc:VGNC:77554]	1.467833535	2.19E-07	2.27E-06
ENSMMUG00000039266	Metazoan signal recognition particle RNA [Source:RFAM;Acc:RF00017]	1.155192843	0.003948996	0.014477587
ENSMMUG00000002376	scavenger receptor class F member 1 [Source:VGNC Symbol;Acc:VGNC:77027]	2.10628113	6.29E-18	3.15E-16
ENSMMUG00000002377	Rab interacting lysosomal protein [Source:VGNC Symbol;Acc:VGNC:76866]	1.828328102	6.43E-08	7.45E-07
ENSMMUG00000004820	pre-mRNA processing factor 8 [Source:VGNC Symbol;Acc:VGNC:76275]	1.275179063	2.59E-21	1.83E-19
ENSMMUG00000002380	WD repeat domain 81 [Source:NCBI gene;Acc:721254]	-0.404078426	0.000289914	0.001488633
ENSMMUG00000002382	serpin family F member 2 [Source:VGNC Symbol;Acc:VGNC:77372]	0.582447756	0.004340647	0.015741648
ENSMMUG00000017518	small G protein signaling modulator 2 [Source:NCBI gene;Acc:703543]	-0.304607181	0.014386417	0.043506733
ENSMMUG00000053298		-0.972500459	2.17E-08	2.74E-07
ENSMMUG00000020524	platelet activating factor acetylhydrolase 1b regulatory subunit 1 [Source:VGNC Symbol;Acc:VGNC:75747]	0.497669798	0.003157621	0.0118775
ENSMMUG00000020527	clustered mitochondria homolog [Source:VGNC Symbol;Acc:VGNC:71370]	0.597501885	0.00209613	0.008400695
ENSMMUG00000022989	RAP1 GTPase activating protein 2 [Source:VGNC Symbol;Acc:VGNC:76561]	0.826175516	0.00034633	0.00173469
ENSMMUG00000061370		1.850958546	0.006730791	0.022793016
ENSMMUG00000030389		1.098342233	0.001089174	0.004750694
ENSMMUG00000062583	olfactory receptor 1A1 [Source:NCBI gene;Acc:721403]	1.018316948	5.43E-06	4.22E-05
ENSMMUG00000012825	olfactory receptor 1A2 [Source:NCBI gene;Acc:100429763]	1.40904646	0.001599824	0.00661975
ENSMMUG00000042407		1.011002717	0.005189589	0.018271053
ENSMMUG00000055048		1.061858807	8.08E-11	1.57E-09
ENSMMUG00000008556	integrin subunit alpha E [Source:NCBI gene;Acc:721483]	0.610987014	0.000195921	0.001051034
ENSMMUG00000063482		-0.914226437	0.000749534	0.003435999
ENSMMUG00000057632		3.552927573	6.96E-31	1.43E-28

ENSMMUG00000058966		-0.766014322	0.00031847	0.001616912
ENSMMUG00000005080	ubiquitin conjugating enzyme E2 G1 [Source:VGNC Symbol;Acc:VGNC:78695]	0.595924926	0.001636142	0.006750107
ENSMMUG00000030381	arachidonate 15-lipoxygenase [Source:VGNC Symbol;Acc:VGNC:69796]	-1.317911708	3.82E-08	4.61E-07
ENSMMUG00000017654	arrestin beta 2 [Source:VGNC Symbol;Acc:VGNC:70022]	1.128058366	0.001568312	0.006502793
ENSMMUG00000049649	proteasome 20S subunit beta 6 [Source:NCBI gene;Acc:710028]	1.319225984	1.35E-14	4.63E-13
ENSMMUG00000061951		1.372395014	3.96E-05	0.000249533
ENSMMUG00000005666	phospholipase D2 [Source:VGNC Symbol;Acc:VGNC:76172]	1.86814868	4.44E-15	1.61E-13
ENSMMUG00000012040	enolase 3 [Source:VGNC Symbol;Acc:VGNC:72220]	2.941229071	0.000192424	0.001035045
ENSMMUG00000009176	calmodulin binding transcription activator 2 [Source:VGNC Symbol;Acc:VGNC:70578]	1.594133581	1.86E-12	4.63E-11
ENSMMUG00000011344	solute carrier family 52 member 1 [Source:VGNC Symbol;Acc:VGNC:77795]	4.183037972	3.36E-10	5.78E-09
ENSMMUG00000018648	zinc finger protein 594 [Source:NCBI gene;Acc:711492]	0.96921116	0.000180846	0.000977643
ENSMMUG00000010468	RPA interacting protein [Source:VGNC Symbol;Acc:VGNC:76854]	-0.500595438	0.01584175	0.04707541
ENSMMUG00000006594	DEAH-box helicase 33 [Source:VGNC Symbol;Acc:VGNC:71633]	-0.571128652	0.000154182	0.000847217
ENSMMUG00000006599	derlin 2 [Source:VGNC Symbol;Acc:VGNC:71654]	-0.59558309	1.09E-05	7.82E-05
ENSMMUG00000046195	MIS12 kinetochore complex component [Source:NCBI gene;Acc:712202]	-0.934461116	6.98E-07	6.54E-06
ENSMMUG00000043771	uncharacterized LOC712349 [Source:NCBI gene;Acc:712349]	-0.877862917	0.00963073	0.030939686
ENSMMUG00000030453	NLR family pyrin domain containing 1 [Source:NCBI gene;Acc:721648]	-1.335299504	1.53E-20	1.01E-18
ENSMMUG00000064520		2.552825122	0.006660365	0.022598186
ENSMMUG00000010536	discs large MAGUK scaffold protein 4 [Source:VGNC Symbol;Acc:VGNC:71920]	0.722475475	1.49E-07	1.60E-06
ENSMMUG00000052091	dishevelled segment polarity protein 2 [Source:NCBI gene;Acc:714381]	-1.026551198	0.004401569	0.015908043
ENSMMUG00000010543	PHD finger protein 23 [Source:VGNC Symbol;Acc:VGNC:75877]	-2.567196899	9.90E-06	7.19E-05
ENSMMUG00000064650	SPEM family member 3 [Source:NCBI gene;Acc:106993878]	1.052911892	3.19E-20	2.01E-18
ENSMMUG00000023620	RNA polymerase II subunit A [Source:NCBI gene;Acc:721865]	-0.47247231	0.009153437	0.029719529
ENSMMUG00000006327	tumor necrosis factor (ligand) superfamily, member 12 [Source:NCBI gene;Acc:100551506]	-0.31768315	0.010356321	0.032923757
ENSMMUG00000006332	SUMO specific peptidase 3 [Source:NCBI gene;Acc:715884]	0.725291923	2.38E-05	0.000158176
ENSMMUG00000033155	small nucleolar RNA, H/ACA box 48 [Source:HGNC Symbol;Acc:HGNC:32641]	-1.21174673	0.007340093	0.024636218
ENSMMUG00000013501	CD68 molecule [Source:VGNC Symbol;Acc:VGNC:82121]	-2.14102367	6.19E-08	7.20E-07
ENSMMUG00000013503	mannose-P-dolichol utilization defect 1 [Source:VGNC Symbol;Acc:VGNC:74913]	-1.292558582	0.000511722	0.002462552
ENSMMUG00000013510	FMR1 autosomal homolog 2 [Source:NCBI gene;Acc:721889]	-1.111957318	1.90E-06	1.61E-05
ENSMMUG00000013513	sex hormone binding globulin [Source:VGNC Symbol;Acc:VGNC:77208]	-0.896174527	0.001427769	0.006003482
ENSMMUG00000008639	tumor protein p53 [Source:VGNC Symbol;Acc:VGNC:78627]	-0.740876419	0.00203764	0.008196736
ENSMMUG00000048204	ephrin B3 [Source:NCBI gene;Acc:716257]	1.313469203	5.72E-10	9.47E-09
ENSMMUG00000063668	cytochrome b5 domain containing 1 [Source:NCBI gene;Acc:716429]	-1.076457521	2.73E-05	0.00017837
ENSMMUG00000009995	arachidonate 15-lipoxygenase type B [Source:VGNC Symbol;Acc:VGNC:69797]	1.453150105	1.50E-13	4.35E-12
ENSMMUG00000065208		1.571798119	1.23E-06	1.09E-05
ENSMMUG00000019850	arachidonate lipoxygenase 3 [Source:VGNC Symbol;Acc:VGNC:69607]	-1.589938818	1.39E-28	2.11E-26
ENSMMUG00000023803	myosin heavy chain 10 [Source:NCBI gene;Acc:721979]	-2.443226781	6.21E-07	5.89E-06
ENSMMUG00000055290	netrin 1 [Source:NCBI gene;Acc:717645]	0.919204707	1.35E-08	1.77E-07

ENSMMUG00000058374		-0.887741762	0.000911876	0.004065892
ENSMMUG00000051001		-2.611947424	0.002320082	0.009146614
ENSMMUG00000016723	zinc finger protein 287 [Source:NCBI gene;Acc:695524]	-1.164781091	0.000488378	0.002362464
ENSMMUG00000013345	folliculin [Source:NCBI gene;Acc:698082]	-3.46458752	1.58E-06	1.36E-05
ENSMMUG00000021091	COP9 signalosome subunit 3 [Source:VGNC Symbol;Acc:VGNC:71316]	-3.197249371	5.00E-08	5.89E-07
ENSMMUG00000006493	ras related dexamethasone induced 1 [Source:VGNC Symbol;Acc:VGNC:76485]	-0.759910508	0.000161552	0.000881838
ENSMMUG00000023377	retinoic acid induced 1 [Source:VGNC Symbol;Acc:VGNC:76651]	0.648464836	0.002574394	0.01002798
ENSMMUG00000023375	sterol regulatory element binding transcription factor 1 [Source:VGNC Symbol;Acc:VGNC:77880]	-0.56332524	0.012515276	0.038614239
ENSMMUG00000005900	GID complex subunit 4 homolog [Source:NCBI gene;Acc:699842]	-1.001993445	0.003044415	0.011541453
ENSMMUG00000021409	developmentally regulated GTP binding protein 2 [Source:VGNC Symbol;Acc:VGNC:71993]	-0.927267372	0.000614253	0.002896323
ENSMMUG00000018344	myosin XVA [Source:VGNC Symbol;Acc:VGNC:75109]	0.984962411	6.39E-09	8.86E-08
ENSMMUG00000056257		-0.713607612	0.000913892	0.004068416
ENSMMUG00000053675		-0.564653736	0.010477873	0.033257405
ENSMMUG00000005323	phosphoribosyl pyrophosphate synthetase associated protein 2 [Source:VGNC Symbol;Acc:VGNC:76430]	0.619735524	2.10E-08	2.66E-07
ENSMMUG00000051798	Small nucleolar RNA U3 [Source:RFAM;Acc:RF00012]	-3.384039924	0.001252405	0.005366718
ENSMMUG00000051453		1.443426642	0.000579478	0.002751785
ENSMMUG00000008536	mitogen-activated protein kinase 7 [Source:VGNC Symbol;Acc:VGNC:74648]	2.351901777	5.55E-06	4.30E-05
ENSMMUG00000008538	microfibril associated protein 4 [Source:VGNC Symbol;Acc:VGNC:74710]	2.499620702	2.65E-07	2.71E-06
ENSMMUG00000008540	ring finger protein 112 [Source:VGNC Symbol;Acc:VGNC:76724]	-1.674536339	2.91E-07	2.94E-06
ENSMMUG00000051286		-0.775019929	0.005129303	0.018076957
ENSMMUG00000030279	A-kinase anchoring protein 10 [Source:VGNC Symbol;Acc:VGNC:69764]	1.374368747	8.48E-05	0.000492038
ENSMMUG00000018579	sperm antigen with calponin homology and coiled-coil domains 1 [Source:VGNC Symbol;Acc:VGNC:78021]	0.361520623	0.011696395	0.036505352
ENSMMUG00000024170	U6 spliceosomal RNA [Source:RFAM;Acc:RF00026]	0.825212183	0.00048303	0.002341353
ENSMMUG00000040830		0.546583203	0.014537025	0.043858155
ENSMMUG00000060502		1.766470648	1.35E-11	2.96E-10
ENSMMUG00000061335		1.368918065	1.38E-07	1.49E-06
ENSMMUG00000020226	WD repeat and SOCS box containing 1 [Source:VGNC Symbol;Acc:VGNC:79186]	1.296522372	5.73E-11	1.13E-09
ENSMMUG00000054784		1.241746536	9.67E-09	1.30E-07
ENSMMUG00000061836		1.178738376	5.70E-05	0.000345571
ENSMMUG00000065113		0.985185309	0.000104738	0.000595598
ENSMMUG00000018719	nemo like kinase [Source:VGNC Symbol;Acc:VGNC:75277]	1.325540525	2.45E-16	1.05E-14
ENSMMUG00000063312		-0.814378681	0.00049266	0.002380484
ENSMMUG00000019145	TNF alpha induced protein 1 [Source:VGNC Symbol;Acc:VGNC:78578]	-0.908706198	0.000249833	0.00130822
ENSMMUG00000019146	DNA polymerase delta interacting protein 2 [Source:VGNC Symbol;Acc:VGNC:76099]	-0.965884482	6.62E-06	5.03E-05
ENSMMUG00000019148	vitronectin [Source:NCBI gene;Acc:708499]	-0.770814242	0.000719986	0.003312455
ENSMMUG00000012240	phosphatidylinositol glycan anchor biosynthesis class S [Source:VGNC Symbol;Acc:VGNC:75800]	-0.744740937	0.000467492	0.002273897
ENSMMUG00000053611	stromal cell derived factor 2 [Source:NCBI gene;Acc:716189]	2.839914773	1.07E-05	7.70E-05

ENSMMUG00000004708	SPT6 homolog, histone chaperone and transcription elongation factor [Source:VGNC Symbol;Acc:VGNC:78057]	-0.657309691	5.25E-05	0.000321337
ENSMMUG00000004710	protein interacting with cyclin A1 [Source:VGNC Symbol;Acc:VGNC:76336]	-0.753895396	0.004003305	0.014642241
ENSMMUG00000007293	RAB34, member RAS oncogene family [Source:VGNC Symbol;Acc:VGNC:81533]	-0.912244779	8.07E-06	6.00E-05
ENSMMUG00000026204	Small nucleolar RNA SNORD42 [Source:RFAM;Acc:RF00150]	-0.448792993	0.015918257	0.047272705
ENSMMUG00000051743	TLC domain containing 1 [Source:NCBI gene;Acc:710391]	-0.650244323	0.008612197	0.028283102
ENSMMUG00000021408	family with sequence similarity 222 member B [Source:VGNC Symbol;Acc:VGNC:72352]	1.156348045	9.43E-07	8.57E-06
ENSMMUG00000012523	flotillin 2 [Source:NCBI gene;Acc:114669752]	1.23490389	2.25E-09	3.39E-08
ENSMMUG00000021553	dehydrogenase/reductase 13 [Source:VGNC Symbol;Acc:VGNC:81387]	1.362864054	7.55E-14	2.30E-12
ENSMMUG00000021554	PHD finger protein 12 [Source:VGNC Symbol;Acc:VGNC:75838]	1.346254421	3.34E-10	5.78E-09
ENSMMUG00000009938	myosin XVIIIA [Source:VGNC Symbol;Acc:VGNC:75058]	0.475614047	0.001055	0.004623207
ENSMMUG00000019667	TAO kinase 1 [Source:VGNC Symbol;Acc:VGNC:81591]	1.261594184	0.000629903	0.002950279
ENSMMUG00000057447		5.092785962	7.99E-06	5.96E-05
ENSMMUG00000014411	coronin 6 [Source:NCBI gene;Acc:711818]	0.581059058	0.015835551	0.04707541
ENSMMUG00000014413	slingshot protein phosphatase 2 [Source:VGNC Symbol;Acc:VGNC:78018]	0.476025754	7.13E-05	0.000421845
ENSMMUG00000060080	U6 spliceosomal RNA [Source:RFAM;Acc:RF00026]	-1.488808408	8.22E-05	0.000478505
ENSMMUG00000023698	golgi SNAP receptor complex member 1 [Source:VGNC Symbol;Acc:VGNC:73110]	-0.855070816	1.01E-10	1.92E-09
ENSMMUG00000014689	myosin ID [Source:VGNC Symbol;Acc:VGNC:75113]	-0.644346107	0.005669298	0.019658895
ENSMMUG00000059292		0.938233155	8.15E-11	1.58E-09
ENSMMUG00000011411	sperm acrosome associated 3 [Source:VGNC Symbol;Acc:VGNC:77930]	1.928771878	3.37E-11	6.89E-10
ENSMMUG00000062784		-0.767161989	0.00557814	0.01939555
ENSMMUG00000011413	acid sensing ion channel subunit 2 [Source:VGNC Symbol;Acc:VGNC:70241]	-0.907338168	0.001905014	0.007727512
ENSMMUG00000060024		-1.141131031	5.35E-05	0.000327058
ENSMMUG00000000288	C-C motif chemokine ligand 2 [Source:VGNC Symbol;Acc:VGNC:70698]	-1.481568498	1.85E-06	1.57E-05
ENSMMUG00000009782	C-C motif chemokine ligand 7 [Source:NCBI gene;Acc:714751]	-2.415530851	4.40E-06	3.49E-05
ENSMMUG00000064793	C-C motif chemokine ligand 11 [Source:NCBI gene;Acc:574218]	-1.772953415	0.016156666	0.047859089
ENSMMUG00000009785	C-C motif chemokine ligand 13 [Source:VGNC Symbol;Acc:VGNC:70696]	-2.92532828	0.000166542	0.000906961
ENSMMUG00000004794	DNA ligase 3 [Source:VGNC Symbol;Acc:VGNC:74266]	2.256340386	1.25E-06	1.11E-05
ENSMMUG00000005170	unc-45 myosin chaperone B [Source:VGNC Symbol;Acc:VGNC:78954]	-0.803669796	0.001717375	0.007039681
ENSMMUG00000037555	schlafen family member 11 [Source:NCBI gene;Acc:715511]	1.85052746	0.001299587	0.005539099
ENSMMUG00000060439	schlafen family member 12 like [Source:NCBI gene;Acc:106992394]	-1.0404864	4.55E-06	3.60E-05
ENSMMUG00000031736		-2.205052674	0.003017025	0.011459302
ENSMMUG00000026432	small nucleolar RNA, C/D box 7 [Source:HGNC Symbol;Acc:HGNC:32704]	-3.349619307	0.001033032	0.004536866
ENSMMUG00000054472		0.828834197	0.003218543	0.012080737
ENSMMUG00000054210	C-C motif chemokine 4 [Source:NCBI gene;Acc:100426632]	-1.258994193	6.07E-14	1.87E-12
ENSMMUG00000058233	C-C motif chemokine 3-like [Source:NCBI gene;Acc:100426537]	-1.468661842	0.00476881	0.017033372

ENSMMUG00000061594		-1.391516059	0.001322358	0.005619281
ENSMMUG00000000618	gametogenetin binding protein 2 [Source:VGNC Symbol;Acc:VGNC:72746]	-0.919480467	6.41E-05	0.000383421
ENSMMUG00000051190	Metazoan signal recognition particle RNA [Source:RFAM;Acc:RF00017]	-1.703520804	0.000420857	0.002067864
ENSMMUG00000007920	myosin XIX [Source:VGNC Symbol;Acc:VGNC:75026]	-0.856464091	2.23E-05	0.000149091
ENSMMUG00000007923	phosphatidylinositol glycan anchor biosynthesis class W [Source:VGNC Symbol;Acc:VGNC:75850]	-0.973647634	5.25E-05	0.000321181
ENSMMUG00000053845		-1.107545332	3.55E-06	2.86E-05
ENSMMUG00000007917	carbonic anhydrase 4 [Source:NCBI gene;Acc:716919]	-1.257489764	9.00E-09	1.21E-07
ENSMMUG00000017113	ubiquitin specific peptidase 32 [Source:NCBI gene;Acc:716857]	-1.177468955	1.07E-13	3.16E-12
ENSMMUG00000052977	putative protein FAM106C [Source:NCBI gene;Acc:106994084]	-0.982004289	6.63E-08	7.67E-07
ENSMMUG00000065117	septin 4 [Source:HGNC Symbol;Acc:HGNC:9165]	-0.891359023	2.89E-05	0.000187314
ENSMMUG00000008423	septin 4 [Source:NCBI gene;Acc:714724]	-0.75664915	1.83E-13	5.20E-12
ENSMMUG00000046171	myotubularin related protein 4 [Source:NCBI gene;Acc:714621]	-0.675695951	3.41E-08	4.17E-07
ENSMMUG00000007004	ring finger protein 43 [Source:NCBI gene;Acc:710124]	-0.880690437	0.012027846	0.037348646
ENSMMUG00000056064	serine and arginine rich splicing factor 1 [Source:NCBI gene;Acc:713958]	-0.790261367	2.95E-08	3.64E-07
ENSMMUG00000004723	vascular endothelial zinc finger 1 [Source:NCBI gene;Acc:713848]	-0.626253926	7.57E-06	5.68E-05
ENSMMUG00000002269	serine carboxypeptidase 1 [Source:VGNC Symbol;Acc:VGNC:82232]	-0.588034657	0.005497723	0.019192077
ENSMMUG00000049376		2.382258105	0.001123023	0.004877099
ENSMMUG00000005793	ankyrin repeat and fibronectin type III domain containing 1 [Source:VGNC Symbol;Acc:VGNC:69712]	1.245805281	6.60E-07	6.21E-06
ENSMMUG00000059321		2.195888056	0.002028512	0.008167048
ENSMMUG00000006066	phosphatidylcholine transfer protein [Source:VGNC Symbol;Acc:VGNC:75789]	2.776124859	1.28E-06	1.12E-05
ENSMMUG00000050544		-1.228100125	1.18E-05	8.43E-05
ENSMMUG00000049997		-1.09767422	1.25E-07	1.37E-06
ENSMMUG00000063189		-0.488269844	0.006238255	0.021346765
ENSMMUG00000062178	Metazoan signal recognition particle RNA [Source:RFAM;Acc:RF00017]	-1.121926539	0.003247253	0.012168956
ENSMMUG00000001942	mbt domain containing 1 [Source:VGNC Symbol;Acc:VGNC:74538]	-1.143635396	3.16E-10	5.50E-09
ENSMMUG00000001940	NME/NM23 nucleoside diphosphate kinase 2 [Source:NCBI gene;Acc:100533957]	-1.438011724	4.45E-10	7.52E-09
ENSMMUG00000054539		-0.825208196	0.007729462	0.025758139
ENSMMUG00000022741	WAP, follistatin/kazal, immunoglobulin, kunitz and netrin domain containing 2 [Source:VGNC Symbol;Acc:VGNC:79900]	0.7761333	1.80E-05	0.000122985
ENSMMUG00000010117	LUC7 like 3 pre-mRNA splicing factor [Source:VGNC Symbol;Acc:VGNC:74346]	0.923869923	0.002713104	0.010472297
ENSMMUG00000017256	calcium voltage-gated channel subunit alpha1 G [Source:VGNC Symbol;Acc:VGNC:70504]	1.928899085	1.30E-27	1.89E-25
ENSMMUG00000017252	spermatogenesis associated 20 [Source:VGNC Symbol;Acc:VGNC:77993]	-1.328777153	1.04E-06	9.37E-06
ENSMMUG00000017242	MYCBP associated protein [Source:VGNC Symbol;Acc:VGNC:74994]	1.433726178	4.38E-05	0.000272648
ENSMMUG00000017234	leucine rich repeat containing 59 [Source:VGNC Symbol;Acc:VGNC:81436]	-2.961061711	9.87E-31	1.98E-28

ENSMMUG00000020061	protein phosphatase 1 regulatory subunit 9B [Source:VGNC Symbol;Acc:VGNC:76372]	-1.268794412	8.88E-07	8.12E-06
ENSMMUG00000014687	sterile alpha motif domain containing 14 [Source:VGNC Symbol;Acc:VGNC:77112]	-1.844557263	0.000361434	0.001799216
ENSMMUG00000014685	pyruvate dehydrogenase kinase 2 [Source:VGNC Symbol;Acc:VGNC:75933]	-1.640438972	0.000389006	0.001924171
ENSMMUG00000012178	calcium binding and coiled-coil domain 2 [Source:VGNC Symbol;Acc:VGNC:70675]	0.799543344	4.73E-06	3.72E-05
ENSMMUG00000049405	gastric inhibitory polypeptide [Source:NCBI gene;Acc:698335]	-0.858018444	1.66E-05	0.000114666
ENSMMUG00000021087	SNF8 subunit of ESCRT-II [Source:VGNC Symbol;Acc:VGNC:77729]	-0.554558047	0.004541536	0.016338111
ENSMMUG00000003436	tubulin tyrosine ligase like 6 [Source:VGNC Symbol;Acc:VGNC:79632]	1.366177536	3.51E-27	4.73E-25
ENSMMUG00000060879	homeobox B13 [Source:NCBI gene;Acc:697572]	1.271336971	2.15E-14	7.15E-13
ENSMMUG00000055122		0.566319294	0.001400303	0.005907475
ENSMMUG00000052505	homeobox B9 [Source:NCBI gene;Acc:106994009]	1.399356093	2.15E-24	2.10E-22
ENSMMUG00000020868	homeobox B8 [Source:VGNC Symbol;Acc:VGNC:73505]	1.196176147	1.02E-15	4.01E-14
ENSMMUG00000055324	homeobox B7 [Source:NCBI gene;Acc:697089]	1.280617561	2.67E-12	6.50E-11
ENSMMUG00000062283	homeobox B6 [Source:NCBI gene;Acc:106994013]	1.214009181	1.47E-13	4.26E-12
ENSMMUG00000051420	homeobox B5 [Source:NCBI gene;Acc:696732]	1.17793892	4.73E-08	5.60E-07
ENSMMUG00000045203		1.165394275	0.000172333	0.000935227
ENSMMUG00000057001	homeobox B2 [Source:NCBI gene;Acc:106994010]	0.880669059	4.22E-08	5.04E-07
ENSMMUG00000057095		3.407704164	9.22E-08	1.04E-06
ENSMMUG00000050742	src kinase associated phosphoprotein 1 [Source:NCBI gene;Acc:697043]	0.577181132	1.24E-08	1.64E-07
ENSMMUG00000004760	CDK5 regulatory subunit associated protein 3 [Source:VGNC Symbol;Acc:VGNC:71015]	-0.622034478	0.016211863	0.047971925
ENSMMUG00000003325	pyridoxamine 5'-phosphate oxidase [Source:VGNC Symbol;Acc:VGNC:76141]	-0.959400586	3.88E-07	3.84E-06
ENSMMUG00000057231	T-box transcription factor 21 [Source:NCBI gene;Acc:694044]	1.030456054	1.44E-06	1.26E-05
ENSMMUG00000019760	karyopherin subunit beta 1 [Source:VGNC Symbol;Acc:VGNC:74203]	-0.739331538	1.68E-08	2.18E-07
ENSMMUG00000020020	Rho GTPase activating protein 23 [Source:NCBI gene;Acc:693996]	1.970351937	1.86E-10	3.40E-09
ENSMMUG00000007799	LIM and SH3 protein 1 [Source:VGNC Symbol;Acc:VGNC:74237]	2.101441816	5.80E-14	1.81E-12
ENSMMUG00000050458		-1.52117005	5.39E-12	1.25E-10
ENSMMUG00000056248		4.285973311	1.40E-53	1.78E-50
ENSMMUG00000063779		4.385983159	8.95E-69	4.18E-65
ENSMMUG00000030060	F-box and leucine rich repeat protein 20 [Source:VGNC Symbol;Acc:VGNC:81407]	-0.550165709	0.000130603	0.00072763
ENSMMUG00000004587	gasdermin B [Source:VGNC Symbol;Acc:VGNC:73219]	2.524247138	2.09E-05	0.000141062
ENSMMUG00000057790		2.930703138	0.000131565	0.00073188
ENSMMUG00000058758		2.454665133	2.49E-05	0.000164087
ENSMMUG00000064723	gasdermin A [Source:NCBI gene;Acc:698841]	1.564487842	0.008211655	0.027145694
ENSMMUG00000002923	colony stimulating factor 3 [Source:VGNC Symbol;Acc:VGNC:71520]	0.8751083	7.91E-08	9.03E-07
ENSMMUG00000002926	mediator complex subunit 24 [Source:NCBI gene;Acc:699536]	1.09154254	2.59E-11	5.40E-10
ENSMMUG00000045187	CASC3 exon junction complex subunit [Source:NCBI gene;Acc:699712]	0.936148679	2.18E-07	2.25E-06
ENSMMUG00000015160	Rap guanine nucleotide exchange factor like 1 [Source:VGNC Symbol;Acc:VGNC:76501]	0.498222631	0.011246459	0.035313277
ENSMMUG00000056646		0.717999617	0.000807734	0.003675162
ENSMMUG00000012486	retinoic acid receptor alpha [Source:VGNC Symbol;Acc:VGNC:76662]	-1.397426896	6.57E-06	5.01E-05
ENSMMUG00000008941	DNA topoisomerase II alpha [Source:VGNC Symbol;Acc:VGNC:78620]	-1.158622021	3.69E-15	1.36E-13
ENSMMUG00000009386	tensin 4 [Source:VGNC Symbol;Acc:VGNC:79432]	1.198475404	0.013968182	0.042406541
ENSMMUG00000001497	keratin 27 [Source:VGNC Symbol;Acc:VGNC:74169]	-0.430643567	0.014836931	0.044552094

ENSMMUG00000062362	keratin 33A [Source:NCBI gene;Acc:705800]	-0.562386715	0.000959022	0.00425043
ENSMMUG00000018644	keratin 32 [Source:VGNC Symbol;Acc:VGNC:74216]	-0.552116094	0.000125634	0.00070302
ENSMMUG00000016114	2',3'-cyclic nucleotide 3' phosphodiesterase [Source:VGNC Symbol;Acc:VGNC:71279]	0.377979303	0.004822405	0.017207274
ENSMMUG00000003437	signal transducer and activator of transcription 5B [Source:NCBI gene;Acc:709760]	1.979224387	1.12E-25	1.30E-23
ENSMMUG00000052220		-0.481333134	0.002619758	0.010173597
ENSMMUG00000010707	signal transducer and activator of transcription 3 [Source:VGNC Symbol;Acc:VGNC:77906]	-0.411721818	3.02E-05	0.000195999
ENSMMUG00000050793	N-acetyl-alpha-glucosaminidase [Source:NCBI gene;Acc:707186]	1.380843963	7.05E-11	1.37E-09
ENSMMUG00000009884	hydroxysteroid 17-beta dehydrogenase 1 [Source:VGNC Symbol;Acc:VGNC:73528]	1.8980602	1.53E-07	1.64E-06
ENSMMUG00000009886	Coenzyme A synthase [Source:VGNC Symbol;Acc:VGNC:81373]	1.208601237	1.75E-14	5.90E-13
ENSMMUG00000009887	MAX dimerization protein MLX [Source:VGNC Symbol;Acc:VGNC:74726]	1.197739867	2.97E-06	2.42E-05
ENSMMUG00000009889	PSMC3 interacting protein [Source:VGNC Symbol;Acc:VGNC:76282]	1.236144897	2.06E-10	3.71E-09
ENSMMUG00000016796	reticulophagy regulator family member 3 [Source:VGNC Symbol;Acc:VGNC:76845]	1.205084699	9.57E-13	2.48E-11
ENSMMUG00000016797	tubulin gamma 1 [Source:NCBI gene;Acc:710756]	1.484441442	2.00E-18	1.08E-16
ENSMMUG00000006086	pleckstrin homology, MyTH4 and FERM domain containing H3 [Source:VGNC Symbol;Acc:VGNC:76113]	1.308176824	7.26E-05	0.000428113
ENSMMUG00000006091	contactin associated protein 1 [Source:VGNC Symbol;Acc:VGNC:71390]	1.681598104	3.06E-15	1.14E-13
ENSMMUG00000006093	enhancer of zeste 1 polycomb repressive complex 2 subunit [Source:VGNC Symbol;Acc:VGNC:72457]	1.591782609	3.38E-13	9.30E-12
ENSMMUG00000056565	Metazoan signal recognition particle RNA [Source:RFAM;Acc:RF00017]	2.466133849	0.009753905	0.031278072
ENSMMUG00000058566		1.44805562	6.88E-16	2.75E-14
ENSMMUG00000012685	proteasome activator subunit 3 [Source:VGNC Symbol;Acc:VGNC:76457]	0.306950529	0.006460728	0.022011366
ENSMMUG00000001870	amine oxidase copper containing 2 [Source:VGNC Symbol;Acc:VGNC:69955]	0.479173916	0.003175883	0.011933388
ENSMMUG00000008087	glucose-6-phosphatase catalytic subunit [Source:VGNC Symbol;Acc:VGNC:72839]	-1.650274505	0.005886775	0.020307564
ENSMMUG00000014123	alanyl-tRNA editing protein Aarsd1 [Source:NCBI gene;Acc:712203]	1.171402794	4.94E-07	4.78E-06
ENSMMUG00000008091	RUN domain containing 1 [Source:VGNC Symbol;Acc:VGNC:77101]	1.136358258	9.47E-06	6.92E-05
ENSMMUG00000064920		1.250678478	4.60E-16	1.91E-14
ENSMMUG00000008090	ribosomal protein L27 [Source:NCBI gene;Acc:712352]	1.076142699	0.004399142	0.015903368
ENSMMUG00000015999	interferon induced protein 35 [Source:VGNC Symbol;Acc:VGNC:73564]	1.196025523	1.39E-05	9.73E-05
ENSMMUG00000016001	vesicle amine transport 1 [Source:VGNC Symbol;Acc:VGNC:79058]	1.07198266	3.35E-10	5.78E-09
ENSMMUG00000065269	Rho family GTPase 2 [Source:NCBI gene;Acc:712559]	1.066012999	8.71E-07	7.98E-06
ENSMMUG00000052607		1.183483444	6.86E-27	9.08E-25
ENSMMUG00000019923	ataxin 7 like 3 [Source:VGNC Symbol;Acc:VGNC:70128]	-0.788676264	2.23E-06	1.87E-05
ENSMMUG00000019924	upstream binding transcription factor [Source:NCBI gene;Acc:711597]	-0.71441102	1.40E-05	9.81E-05
ENSMMUG00000017100	solute carrier family 4 member 1 (Diego blood group) [Source:NCBI gene;Acc:100427618]	-0.739832008	5.98E-06	4.60E-05
ENSMMUG00000029927	ADAM metallopeptidase domain 11 [Source:VGNC Symbol;Acc:VGNC:69466]	-0.563009502	0.013374393	0.040904932
ENSMMUG00000062273	dephospho-CoA kinase domain containing [Source:NCBI gene;Acc:715841]	1.064336211	0.005042941	0.017844352
ENSMMUG00000060665		-1.130891569	1.30E-05	9.17E-05
ENSMMUG00000054246		-1.526963992	0.004735235	0.016935017
ENSMMUG00000021932	Rho GTPase activating protein 27 [Source:VGNC Symbol;Acc:VGNC:69935]	-1.210733974	0.005266448	0.018495218
ENSMMUG00000008459	pleckstrin homology and RUN domain containing M1 [Source:NCBI gene;Acc:716464]	-0.881703773	0.001266524	0.005416425
ENSMMUG00000008199	leucine-rich repeat-containing protein 37A [Source:NCBI gene;Acc:714675]	-0.858359263	0.009131449	0.029655005
ENSMMUG00000005548	N-ethylmaleimide sensitive factor, vesicle fusing ATPase [Source:VGNC Symbol;Acc:VGNC:75536]	0.746909251	1.93E-10	3.50E-09

ENSMMUG00000057209		-0.963941899	2.77E-08	3.43E-07
ENSMMUG00000004108	corticotropin releasing hormone receptor 1 [Source:VGNC Symbol;Acc:VGNC:81184]	0.752010417	0.000192025	0.001033294
ENSMMUG00000004122	microtubule associated protein tau [Source:NCBI gene;Acc:574327]	0.453850625	0.004046552	0.014781141
ENSMMUG00000004129	KAT8 regulatory NSL complex subunit 1 [Source:VGNC Symbol;Acc:VGNC:73900]	0.635554067	0.008515198	0.027997334
ENSMMUG00000053257	Y RNA [Source:RFAM;Acc:RF00019]	1.183711627	3.21E-10	5.56E-09
ENSMMUG00000063877	leucine-rich repeat-containing protein 37A2 [Source:NCBI gene;Acc:716147]	-0.300557975	0.000904343	0.004037858
ENSMMUG00000003289	integrin subunit beta 3 [Source:NCBI gene;Acc:717379]	0.74106232	3.08E-10	5.37E-09
ENSMMUG00000010588	EF-hand calcium binding domain 13 [Source:HGNC Symbol;Acc:HGNC:26864]	0.597551253	0.001766804	0.00721698
ENSMMUG00000002166	tousled like kinase 2 [Source:VGNC Symbol;Acc:VGNC:78368]	-2.375799819	0.010404696	0.033047576
ENSMMUG00000015185	endoplasmic reticulum to nucleus signaling 1 [Source:VGNC Symbol;Acc:VGNC:72429]	0.395121422	0.000539621	0.002580868
ENSMMUG00000017521	testis expressed 2 [Source:VGNC Symbol;Acc:VGNC:79894]	0.712892536	0.002716782	0.01048361
ENSMMUG00000016371	G protein subunit alpha 13 [Source:VGNC Symbol;Acc:VGNC:73087]	-0.276115956	0.002900536	0.011091958
ENSMMUG00000060767		2.750891471	7.70E-11	1.50E-09
ENSMMUG00000060015	axin 2 [Source:NCBI gene;Acc:718566]	0.407165108	0.003422407	0.01274699
ENSMMUG00000002959	helicase with zinc finger [Source:VGNC Symbol;Acc:VGNC:73178]	2.628019908	1.67E-21	1.19E-19
ENSMMUG00000057157		1.985029892	4.56E-10	7.69E-09
ENSMMUG00000063817	SRY-box transcription factor 9 [Source:NCBI gene;Acc:574208]	1.058455512	2.85E-12	6.93E-11
ENSMMUG00000054673		2.113168907	0.003081411	0.011653323
ENSMMUG00000001269	chromosome 16 C17orf80 homolog [Source:VGNC Symbol;Acc:VGNC:70440]	1.177457004	2.66E-13	7.40E-12
ENSMMUG00000001271	cleavage and polyadenylation specific factor 4 like [Source:NCBI gene;Acc:698279]	1.297930022	0.007358153	0.024679116
ENSMMUG00000061561		-3.436231898	0.012879926	0.039582556
ENSMMUG00000049948		-1.040986135	3.10E-07	3.12E-06
ENSMMUG00000064463		1.364041305	8.29E-05	0.000481899
ENSMMUG00000049403		0.493602215	0.000345497	0.001732434
ENSMMUG00000064052	CD300 molecule like family member d [Source:NCBI gene;Acc:703142]	1.532474698	3.61E-12	8.59E-11
ENSMMUG00000058592		1.923723404	1.42E-13	4.13E-12
ENSMMUG00000020462	CD300e molecule [Source:NCBI gene;Acc:114673189]	1.905802081	7.87E-11	1.53E-09
ENSMMUG00000005737	SLC9A3 regulator 1 [Source:VGNC Symbol;Acc:VGNC:77639]	2.913979118	1.21E-07	1.33E-06
ENSMMUG00000005738	N-acetyltransferase 9 (putative) [Source:NCBI gene;Acc:699085]	4.328811619	0.000178178	0.000964335
ENSMMUG00000005744	glutamate ionotropic receptor NMDA type subunit 2C [Source:VGNC Symbol;Acc:VGNC:73284]	0.43231975	0.012714043	0.039107046
ENSMMUG00000023405	otopetrin 3 [Source:VGNC Symbol;Acc:VGNC:75654]	0.421445573	0.000986353	0.004359163
ENSMMUG00000023406	HID1 domain containing [Source:VGNC Symbol;Acc:VGNC:73394]	0.670249979	1.92E-06	1.62E-05
ENSMMUG00000018031	solute carrier family 25 member 19 [Source:VGNC Symbol;Acc:VGNC:77474]	-0.964642833	0.005529063	0.019272693
ENSMMUG00000058142		-0.924950843	5.58E-07	5.34E-06
ENSMMUG00000022120	transmembrane protein 94 [Source:VGNC Symbol;Acc:VGNC:79760]	-0.77910501	2.24E-07	2.32E-06
ENSMMUG00000022121	CASK interacting protein 2 [Source:VGNC Symbol;Acc:VGNC:70577]	-1.055835591	1.13E-05	8.14E-05
ENSMMUG00000022123	tRNA splicing endonuclease subunit 54 [Source:VGNC Symbol;Acc:VGNC:79666]	-0.667319681	0.000570527	0.002714794
ENSMMUG00000001228	LLGL scribble cell polarity complex component 2 [Source:NCBI gene;Acc:706973]	-0.458461323	0.000651292	0.003032225
ENSMMUG00000021636	SAP30 binding protein [Source:VGNC Symbol;Acc:VGNC:77142]	-1.00169587	0.003245394	0.012165238
ENSMMUG00000013646	unk zinc finger [Source:VGNC Symbol;Acc:VGNC:79803]	0.688949028	0.002053169	0.008247395
ENSMMUG00000002822	unc-13 homolog D [Source:VGNC Symbol;Acc:VGNC:79375]	0.87709879	1.34E-07	1.45E-06
ENSMMUG00000009381	WW domain binding protein 2 [Source:VGNC Symbol;Acc:VGNC:78786]	0.949452258	3.08E-11	6.34E-10

ENSMMUG00000009377	tripartite motif containing 47 [Source:VGNC Symbol;Acc:VGNC:79171]	1.365259532	1.10E-09	1.74E-08
ENSMMUG00000008885	exocyst complex component 7 [Source:VGNC Symbol;Acc:VGNC:72319]	0.339171065	0.000282578	0.001456837
ENSMMUG00000054437		0.373751544	3.70E-05	0.00023477
ENSMMUG00000003534	ring finger protein 157 [Source:VGNC Symbol;Acc:VGNC:76860]	0.686553732	2.44E-05	0.000161528
ENSMMUG00000003539	glutamine rich 2 [Source:VGNC Symbol;Acc:VGNC:76534]	2.155591964	3.14E-10	5.46E-09
ENSMMUG00000049537	phosphoribosyl pyrophosphate synthetase associated protein 1 [Source:NCBI gene;Acc:706625]	2.585106049	0.000254154	0.001329358
ENSMMUG00000018011	sphingosine kinase 1 [Source:VGNC Symbol;Acc:VGNC:77858]	1.465593277	0.004897809	0.017423154
ENSMMUG00000018967	ubiquitin conjugating enzyme E2 O [Source:VGNC Symbol;Acc:VGNC:79813]	2.084064569	1.36E-29	2.36E-27
ENSMMUG00000046918		1.973768583	7.07E-05	0.000418421
ENSMMUG00000055379		1.435280405	4.65E-13	1.26E-11
ENSMMUG00000053937		1.381353391	2.96E-11	6.10E-10
ENSMMUG00000049183	transmembrane protein 235 [Source:NCBI gene;Acc:715249]	-0.966946444	0.010530081	0.033391593
ENSMMUG00000008237	phosphatidylglycerophosphate synthase 1 [Source:VGNC Symbol;Acc:VGNC:75890]	-0.747923855	0.002464151	0.00965757
ENSMMUG00000008239	dynein axonemal heavy chain 17 [Source:NCBI gene;Acc:715774]	-0.668630471	0.001253039	0.005366941
ENSMMUG00000051537		2.935880804	1.24E-07	1.35E-06
ENSMMUG00000013313	cytohesin 1 [Source:VGNC Symbol;Acc:VGNC:71828]	0.47040339	0.00162466	0.006708658
ENSMMUG00000005352	ubiquitin specific peptidase 36 [Source:VGNC Symbol;Acc:VGNC:79219]	0.379734775	0.002928644	0.011187246
ENSMMUG00000005355	TIMP metallopeptidase inhibitor 2 [Source:NCBI gene;Acc:574357]	2.731974698	7.18E-05	0.000424675
ENSMMUG00000046417	chromobox 2 [Source:NCBI gene;Acc:717462]	2.195696401	1.71E-08	2.21E-07
ENSMMUG00000016417	chromobox 8 [Source:NCBI gene;Acc:717514]	2.089718779	6.02E-25	6.45E-23
ENSMMUG00000020840	TBC1 domain family member 16 [Source:VGNC Symbol;Acc:VGNC:78228]	0.40033429	0.002857118	0.010939549
ENSMMUG00000064603	caspase recruitment domain family member 14 [Source:NCBI gene;Acc:712205]	0.459705508	0.002587989	0.010064164
ENSMMUG00000064563		-0.565183805	0.012024192	0.037348646
ENSMMUG00000029310	hemoglobin, alpha 2 [Source:NCBI gene;Acc:693930]	-0.566334048	0.000624156	0.002930212
ENSMMUG00000019566	hemoglobin subunit alpha 1 [Source:NCBI gene;Acc:701930]	-0.550781912	0.005108356	0.01801672
ENSMMUG00000000596	LUC7 like [Source:VGNC Symbol;Acc:VGNC:74461]	0.689964641	0.014412228	0.04355661
ENSMMUG00000029296	phosphatidylinositol glycan anchor biosynthesis class Q [Source:VGNC Symbol;Acc:VGNC:75798]	0.96428357	0.00034736	0.001738326
ENSMMUG00000049219	STIP1 homology and U-box containing protein 1 [Source:NCBI gene;Acc:697105]	0.742250556	0.003239739	0.012153787
ENSMMUG00000050065		-0.777229198	0.007411706	0.024811256
ENSMMUG00000016906	F-box and leucine rich repeat protein 16 [Source:VGNC Symbol;Acc:VGNC:81405]	-0.927991882	1.81E-06	1.54E-05
ENSMMUG00000000842	RNA pseudouridine synthase domain containing 1 [Source:NCBI gene;Acc:722393]	2.151797047	0.013415881	0.041022873
ENSMMUG00000063740		-0.404959638	0.004763272	0.017022263
ENSMMUG00000044277	ubiquitin conjugating enzyme E2 I [Source:NCBI gene;Acc:722434]	-0.706013932	0.001614443	0.00667433
ENSMMUG00000021415	BAI1 associated protein 3 [Source:VGNC Symbol;Acc:VGNC:70223]	0.924521977	0.000621135	0.00291896
ENSMMUG00000012439	N-acetylglucosamine-1-phosphate transferase subunit gamma [Source:NCBI gene;Acc:722441]	0.819954843	0.005813458	0.020099141
ENSMMUG00000061546		-2.097464108	0.00052104	0.002498819
ENSMMUG00000008119	telomere maintenance 2 [Source:NCBI gene;Acc:701679]	-0.696359269	0.003511865	0.013038625
ENSMMUG00000029265	zinc finger protein 598 [Source:NCBI gene;Acc:694129]	-0.762552131	1.59E-05	0.00010986
ENSMMUG00000029263	polycystin 1, transient receptor potential channel interacting [Source:NCBI gene;Acc:694905]	-0.418501588	0.015356565	0.045847372
ENSMMUG00000019223	TNF receptor associated factor 7 [Source:VGNC Symbol;Acc:VGNC:79252]	0.889856148	6.31E-05	0.000378537

ENSMMUG00000019238	RNA binding protein with serine rich domain 1 [Source:NCBI gene;Acc:695816]	-0.43085328	6.40E-06	4.90E-05
ENSMMUG00000011459	3-phosphoinositide dependent protein kinase 1 [Source:NCBI gene;Acc:697523]	0.427320227	1.09E-06	9.74E-06
ENSMMUG00000061321	host cell factor C1 regulator 1 [Source:NCBI gene;Acc:700351]	0.711431406	0.013014089	0.039951089
ENSMMUG00000009483	matrix metallopeptidase 25 [Source:VGNC Symbol;Acc:VGNC:74747]	0.489189566	0.013100391	0.040180836
ENSMMUG00000009484	zinc finger and SCAN domain-containing protein 10 [Source:NCBI gene;Acc:700600]	0.599951324	0.012320101	0.038079079
ENSMMUG00000009487	zinc finger protein 205 [Source:VGNC Symbol;Acc:VGNC:78869]	0.555444408	0.000144852	0.00080097
ENSMMUG00000064194		0.904258584	0.000145083	0.000801744
ENSMMUG00000020970	zinc finger protein 213 [Source:VGNC Symbol;Acc:VGNC:78872]	0.535442815	0.000236133	0.001244845
ENSMMUG00000051323	caspase-14-like [Source:NCBI gene;Acc:702430]	0.824078484	3.25E-05	0.000208476
ENSMMUG00000017972	zinc finger protein 200 [Source:VGNC Symbol;Acc:VGNC:78867]	-0.933494795	6.86E-07	6.44E-06
ENSMMUG00000007568	zinc finger protein 174 [Source:VGNC Symbol;Acc:VGNC:78855]	-1.023479525	0.010293655	0.032754237
ENSMMUG00000007569	clusterin associated protein 1 [Source:VGNC Symbol;Acc:VGNC:71211]	-0.607121301	0.001755555	0.00717312
ENSMMUG00000021866	deoxyribonuclease 1 [Source:VGNC Symbol;Acc:VGNC:71946]	0.401492824	0.000845796	0.003817378
ENSMMUG00000021868	TNF receptor associated protein 1 [Source:VGNC Symbol;Acc:VGNC:79019]	0.677735771	9.78E-06	7.12E-05
ENSMMUG00000039943	adenylate cyclase 9 [Source:NCBI gene;Acc:706789]	1.631469035	0.002157935	0.008613926
ENSMMUG00000051196		-1.808006424	0.000803006	0.003660776
ENSMMUG00000040803	sarcalumenin [Source:NCBI gene;Acc:704655]	-1.306978003	0.000174036	0.000943741
ENSMMUG00000044729	transcription factor AP-4 [Source:NCBI gene;Acc:707513]	-1.088526341	0.002875809	0.011003401
ENSMMUG00000044344	vasorin [Source:NCBI gene;Acc:100426861]	-1.057649323	1.57E-05	0.000108604
ENSMMUG00000020540	NmrA like redox sensor 1 [Source:VGNC Symbol;Acc:VGNC:75252]	-1.324659518	7.28E-08	8.36E-07
ENSMMUG00000001888	mahogunin ring finger 1 [Source:VGNC Symbol;Acc:VGNC:74542]	-0.911908271	0.001171258	0.005059935
ENSMMUG00000011001	ankyrin repeat and sterile alpha motif domain containing 3 [Source:VGNC Symbol;Acc:VGNC:69735]	0.657257674	1.30E-05	9.21E-05
ENSMMUG00000014311	chromosome 20 C16orf71 homolog [Source:NCBI gene;Acc:709711]	0.597934731	0.000130525	0.000727485
ENSMMUG00000014313	zinc finger protein 500 [Source:VGNC Symbol;Acc:VGNC:79406]	0.828845885	3.65E-06	2.95E-05
ENSMMUG00000017945	septin 12 [Source:VGNC Symbol;Acc:VGNC:82235]	0.907431746	7.87E-05	0.000460592
ENSMMUG00000049327		1.162005635	0.001258672	0.005387775
ENSMMUG00000017946	rogdi atypical leucine zipper [Source:VGNC Symbol;Acc:VGNC:76862]	0.965303275	2.26E-05	0.000150779
ENSMMUG00000009567	glyoxylate reductase 1 homolog [Source:NCBI gene;Acc:706933]	2.535287563	1.06E-24	1.10E-22
ENSMMUG00000017822	periplakin [Source:VGNC Symbol;Acc:VGNC:76240]	3.541874	1.34E-23	1.22E-21
ENSMMUG00000046237	4-aminobutyrate aminotransferase [Source:NCBI gene;Acc:714017]	-1.111104228	1.15E-09	1.81E-08
ENSMMUG00000001944	chromosome 20 C16orf72 homolog [Source:VGNC Symbol;Acc:VGNC:70443]	0.98646023	3.80E-18	1.99E-16
ENSMMUG00000010142	glutamate ionotropic receptor NMDA type subunit 2A [Source:VGNC Symbol;Acc:VGNC:73282]	3.615419523	0.000340961	0.001714954
ENSMMUG00000005913	activating transcription factor 7 interacting protein 2 [Source:VGNC Symbol;Acc:VGNC:70103]	-1.406846975	4.93E-05	0.000303226
ENSMMUG00000060993	Metazoan signal recognition particle RNA [Source:RFAM;Acc:RF00017]	-1.70665882	0.016700534	0.049123473
ENSMMUG00000056165		-1.80959346	2.77E-06	2.27E-05
ENSMMUG00000052031		-1.997421174	1.59E-14	5.37E-13
ENSMMUG00000064158		-2.194445113	3.50E-08	4.27E-07
ENSMMUG00000060713	epithelial membrane protein 2 [Source:NCBI gene;Acc:710315]	-2.138270424	1.02E-07	1.14E-06
ENSMMUG00000006515	C-type lectin domain containing 16A [Source:VGNC Symbol;Acc:VGNC:71369]	-0.810492362	0.000164441	0.000896211

ENSMMUG00000000566		1.159690122	0.007386837	0.024751639
ENSMMUG00000008784	lipopolysaccharide induced TNF factor [Source:NCBI gene;Acc:711743]	3.792522089	0.001000722	0.004411861
ENSMMUG00000062037	stannin [Source:NCBI gene;Acc:711909]	2.102462577	5.52E-12	1.28E-10
ENSMMUG00000014240	thioredoxin domain containing 11 [Source:VGNC Symbol;Acc:VGNC:79678]	2.087198252	6.01E-10	9.92E-09
ENSMMUG00000057345	calcineurin like phosphoesterase domain containing 1 [Source:NCBI gene;Acc:716978]	0.927654315	7.57E-07	7.06E-06
ENSMMUG00000017379	myocardin related transcription factor B [Source:VGNC Symbol;Acc:VGNC:74865]	-0.817137169	5.76E-05	0.00034878
ENSMMUG00000062213	Metazoan signal recognition particle RNA [Source:RFAM;Acc:RF00017]	-1.134201879	0.00152103	0.006336722
ENSMMUG00000057747		-1.904030867	0.000395527	0.00195505
ENSMMUG00000064131		-1.228692775	1.50E-06	1.30E-05
ENSMMUG00000027108	mml-mir-193b [Source:miRBase;Acc:MI0007656]	-1.174389139	0.012224371	0.037858293
ENSMMUG00000002989	N-terminal asparagine amidase [Source:NCBI gene;Acc:100427569]	-1.752503194	0.001080284	0.00471779
ENSMMUG00000062791		-1.798484612	1.62E-06	1.40E-05
ENSMMUG00000062623	Metazoan signal recognition particle RNA [Source:RFAM;Acc:RF00017]	-1.105660433	1.91E-05	0.000130276
ENSMMUG00000058016	bMERB domain containing 1 [Source:NCBI gene;Acc:713757]	-1.170849036	7.31E-08	8.38E-07
ENSMMUG00000010367	myosin heavy chain 11 [Source:VGNC Symbol;Acc:VGNC:75097]	0.607039257	6.23E-07	5.90E-06
ENSMMUG00000060959		1.010871546	0.000195793	0.001050748
ENSMMUG00000058281	ITPRIP like 2 [Source:NCBI gene;Acc:694060]	-1.789314279	3.13E-05	0.000201867
ENSMMUG00000053309		-0.342107248	0.008827448	0.028814549
ENSMMUG00000002709	G protein-coupled receptor class C group 5 member B [Source:VGNC Symbol;Acc:VGNC:72934]	-0.597298626	0.003440203	0.01280987
ENSMMUG00000016753	dynein axonemal heavy chain 3 [Source:VGNC Symbol;Acc:VGNC:71941]	-1.217623501	1.66E-11	3.59E-10
ENSMMUG00000016758	transmembrane protein 159 [Source:VGNC Symbol;Acc:VGNC:79836]	0.490594865	0.01118783	0.035176444
ENSMMUG00000017725	eukaryotic elongation factor 2 kinase [Source:VGNC Symbol;Acc:VGNC:72098]	-0.770055098	3.32E-08	4.07E-07
ENSMMUG00000002257	RNA polymerase III subunit E [Source:VGNC Symbol;Acc:VGNC:76142]	-0.571611589	9.46E-07	8.59E-06
ENSMMUG00000013877	otoancorin [Source:VGNC Symbol;Acc:VGNC:75624]	0.940162324	0.012005675	0.037321121
ENSMMUG00000058042	heparan sulfate-glucosamine 3-sulfotransferase 2 [Source:NCBI gene;Acc:699722]	0.813316955	5.90E-05	0.000356563
ENSMMUG00000054064		-1.776514279	5.87E-14	1.82E-12
ENSMMUG00000013560	calcium voltage-gated channel auxiliary subunit gamma 3 [Source:VGNC Symbol;Acc:VGNC:70513]	0.800464695	0.000197199	0.001057487
ENSMMUG00000001407	solute carrier family 5 member 11 [Source:VGNC Symbol;Acc:VGNC:77759]	-0.365402316	0.002673125	0.010332204
ENSMMUG00000029176	Rho GTPase activating protein 17 [Source:NCBI gene;Acc:701988]	-0.893566857	9.60E-12	2.17E-10
ENSMMUG00000005942	leucine carboxyl methyltransferase 1 [Source:VGNC Symbol;Acc:VGNC:74067]	-0.529553651	3.83E-05	0.000242136
ENSMMUG00000008254	general transcription factor IIIC subunit 1 [Source:VGNC Symbol;Acc:VGNC:73316]	-3.026509938	0.012318303	0.038079079
ENSMMUG00000015572	GSG1 like [Source:VGNC Symbol;Acc:VGNC:82152]	0.74456644	6.93E-09	9.56E-08
ENSMMUG00000018623	exportin 6 [Source:NCBI gene;Acc:704332]	0.922228852	0.005277944	0.018530948
ENSMMUG00000023526	ATPase sarcoplasmic/endoplasmic reticulum Ca2+ transporting 1 [Source:NCBI gene;Acc:708058]	2.395035369	0.002488537	0.009720546
ENSMMUG00000003303	Tu translation elongation factor, mitochondrial [Source:VGNC Symbol;Acc:VGNC:78674]	1.858802178	3.44E-38	1.28E-35

ENSMMUG00000003298	ataxin 2 like [Source:VGNC Symbol;Acc:VGNC:70159]	1.499571563	2.50E-34	6.60E-32
ENSMMUG00000011798	eukaryotic translation initiation factor 3 subunit C [Source:NCBI gene;Acc:705696]	1.629286175	4.28E-36	1.40E-33
ENSMMUG00000000700	CLN3 lysosomal/endosomal transmembrane protein, battenin [Source:NCBI gene;Acc:705815]	1.522985832	1.74E-21	1.24E-19
ENSMMUG00000022334	sulfotransferase 1A1 [Source:NCBI gene;Acc:114669765]	-2.076533339	0.010031212	0.032057358
ENSMMUG00000017758	mitogen-activated protein kinase 3 [Source:VGNC Symbol;Acc:VGNC:74646]	2.318648181	7.75E-45	4.73E-42
ENSMMUG00000029164	glycerophosphodiester phosphodiesterase domain containing 3 [Source:VGNC Symbol;Acc:VGNC:72800]	-3.589251986	3.33E-17	1.56E-15
ENSMMUG00000000981	T-box transcription factor 6 [Source:VGNC Symbol;Acc:VGNC:78276]	-1.70089309	3.52E-09	5.15E-08
ENSMMUG00000000982	protein phosphatase 4 catalytic subunit [Source:VGNC Symbol;Acc:VGNC:76384]	-1.701112	1.55E-12	3.91E-11
ENSMMUG00000000983	aldolase, fructose-bisphosphate A [Source:NCBI gene;Acc:708406]	-1.31020559	1.00E-07	1.12E-06
ENSMMUG00000037707	TLC domain containing 3B [Source:NCBI gene;Acc:708210]	-1.278011957	0.000106095	0.000601848
ENSMMUG00000020277	double C2 domain alpha [Source:VGNC Symbol;Acc:VGNC:71963]	-0.383809673	0.000167332	0.000910908
ENSMMUG00000018986	seizure related 6 homolog like 2 [Source:VGNC Symbol;Acc:VGNC:81567]	1.273138541	2.14E-08	2.71E-07
ENSMMUG00000061749	PAXIP1 associated glutamate rich protein 1 [Source:NCBI gene;Acc:706839]	0.846352136	2.20E-12	5.41E-11
ENSMMUG00000019826	MYC associated zinc finger protein [Source:NCBI gene;Acc:708982]	0.642073061	0.000965035	0.004270335
ENSMMUG00000009684	kinesin family member 22 [Source:VGNC Symbol;Acc:VGNC:74033]	1.407089938	1.55E-09	2.39E-08
ENSMMUG00000016143	sialophorin [Source:VGNC Symbol;Acc:VGNC:77861]	1.633818963	5.41E-18	2.74E-16
ENSMMUG00000016876	integrin subunit alpha L [Source:NCBI gene;Acc:711945]	0.714031577	9.54E-06	6.95E-05
ENSMMUG00000016879	zinc finger protein 768 [Source:VGNC Symbol;Acc:VGNC:82280]	2.719785301	0.000601106	0.002846779
ENSMMUG00000001121	zinc finger protein 689 [Source:VGNC Symbol;Acc:VGNC:79480]	-0.987849945	0.000145121	0.000801744
ENSMMUG00000005625	fibrosin [Source:NCBI gene;Acc:712741]	2.047168796	0.002423325	0.00951352
ENSMMUG00000051226	FAD-linked sulfhydryl oxidase ERV1 [Source:NCBI gene;Acc:106994988]	1.195111541	2.98E-10	5.21E-09
ENSMMUG00000010556	Snf2 related CREBBP activator protein [Source:NCBI gene;Acc:712944]	0.948882078	6.70E-10	1.10E-08
ENSMMUG00000050996	zinc finger protein 629 [Source:NCBI gene;Acc:713331]	-0.45377056	0.008493603	0.02793288
ENSMMUG00000003233	SET domain containing 1A, histone lysine methyltransferase [Source:NCBI gene;Acc:712416]	0.994164813	9.50E-08	1.07E-06
ENSMMUG00000037727	hydroxy-delta-5-steroid dehydrogenase, 3 beta- and steroid delta-isomerase 7 [Source:NCBI gene;Acc:714136]	0.80982481	1.20E-05	8.56E-05
ENSMMUG00000048207	syntaxin 4 [Source:NCBI gene;Acc:704880]	0.649586583	3.64E-07	3.64E-06
ENSMMUG00000002033	serine protease 36 [Source:VGNC Symbol;Acc:VGNC:76325]	0.698629704	1.31E-07	1.42E-06
ENSMMUG00000014349	integrin subunit alpha X [Source:NCBI gene;Acc:100427850]	1.656345365	4.41E-06	3.50E-05
ENSMMUG00000000901	alpha hemoglobin stabilizing protein [Source:VGNC Symbol;Acc:VGNC:69631]	0.735537431	7.94E-08	9.06E-07
ENSMMUG00000058907		1.630805052	0.000577954	0.002745476
ENSMMUG00000055973	phosphoglycerate mutase 1-like [Source:NCBI gene;Acc:714548]	2.498108729	5.97E-05	0.000359977
ENSMMUG00000008253	zinc finger protein 267 [Source:NCBI gene;Acc:641441]	1.442709605	8.71E-18	4.27E-16
ENSMMUG00000003288	glutamic--pyruvic transaminase 2 [Source:VGNC Symbol;Acc:VGNC:72962]	-1.091474842	1.28E-06	1.13E-05
ENSMMUG00000019869	sorting nexin 20 [Source:VGNC Symbol;Acc:VGNC:77796]	-0.805204937	0.015780343	0.04695263
ENSMMUG00000012209	CYLD lysine 63 deubiquitinase [Source:VGNC Symbol;Acc:VGNC:71581]	-0.741718523	0.000123949	0.000694146
ENSMMUG00000055889		-0.885546508	0.00542594	0.018974552
ENSMMUG00000064095		3.038809043	2.02E-05	0.000137183
ENSMMUG00000013967	TOX high mobility group box family member 3 [Source:VGNC Symbol;Acc:VGNC:78612]	2.041500323	3.56E-07	3.57E-06
ENSMMUG00000043440	RB transcriptional corepressor like 2 [Source:NCBI gene;Acc:696992]	-0.536618939	0.00167771	0.006903322
ENSMMUG00000020583	FTO alpha-ketoglutarate dependent dioxygenase [Source:VGNC Symbol;Acc:VGNC:72772]	0.487779874	0.005658211	0.019630155
ENSMMUG00000054811		2.594003098	0.000748628	0.003432965
ENSMMUG00000056555		1.555318196	1.52E-15	5.84E-14

ENSMMUG00000023525	cadherin 5 [Source:VGNC Symbol;Acc:VGNC:70973]	-0.389808419	0.009031745	0.029392473
ENSMMUG00000049484		-0.85950763	2.59E-11	5.40E-10
ENSMMUG00000020847	CKLF like MARVEL transmembrane domain containing 3 [Source:VGNC Symbol;Acc:VGNC:71215]	-1.129576962	0.000887875	0.00397531
ENSMMUG00000011268	dynein cytoplasmic 1 light intermediate chain 2 [Source:VGNC Symbol;Acc:VGNC:71954]	-0.356204836	0.001353956	0.005729189
ENSMMUG00000054235		0.600113672	0.011200236	0.035207555
ENSMMUG00000016727	cadherin 16 [Source:VGNC Symbol;Acc:VGNC:70809]	0.339707077	0.000306941	0.001565176
ENSMMUG00000022825	carboxylesterase 2 [Source:VGNC Symbol;Acc:VGNC:71012]	0.444550358	2.88E-06	2.36E-05
ENSMMUG00000064237		-1.2160487	1.97E-07	2.06E-06
ENSMMUG00000054948		1.046691752	0.008487583	0.027926179
ENSMMUG00000058815	core-binding factor subunit beta [Source:NCBI gene;Acc:695767]	0.70949688	0.011132472	0.035010239
ENSMMUG00000018102	chromosome 20 C16orf70 homolog [Source:VGNC Symbol;Acc:VGNC:70438]	1.234170527	0.000207695	0.001108258
ENSMMUG00000060314	TNFRSF1A associated via death domain [Source:NCBI gene;Acc:698160]	1.224381189	0.012159308	0.037673438
ENSMMUG00000005906	heat shock transcription factor 4 [Source:VGNC Symbol;Acc:VGNC:73533]	0.749037006	3.39E-05	0.000217007
ENSMMUG00000020268	nucleolar protein 3 [Source:NCBI gene;Acc:696747]	0.889319009	0.00321389	0.012066502
ENSMMUG00000020266	KIAA0895 like [Source:VGNC Symbol;Acc:VGNC:73952]	1.539937808	0.000495065	0.002389775
ENSMMUG00000020254	exocyst complex component 3 like 1 [Source:VGNC Symbol;Acc:VGNC:72376]	1.165352464	3.20E-11	6.55E-10
ENSMMUG00000020258	E2F transcription factor 4 [Source:VGNC Symbol;Acc:VGNC:72025]	0.932939803	5.19E-08	6.10E-07
ENSMMUG00000020259	engulfment and cell motility 3 [Source:VGNC Symbol;Acc:VGNC:72060]	1.190709756	0.005044766	0.017844595
ENSMMUG00000054637	tubulin polymerization promoting protein family member 3 [Source:NCBI gene;Acc:100429881]	1.680861936	0.009372272	0.030303752
ENSMMUG00000009825	zinc finger DHHC-type containing 1 [Source:VGNC Symbol;Acc:VGNC:79102]	0.91952882	3.93E-08	4.73E-07
ENSMMUG00000017105	RHO family interacting cell polarization regulator 1 [Source:NCBI gene;Acc:699910]	-0.52244778	6.61E-05	0.000394438
ENSMMUG00000007804	RAN binding protein 10 [Source:VGNC Symbol;Acc:VGNC:76563]	0.980179503	3.61E-08	4.38E-07
ENSMMUG00000037975	solute carrier family 7 member 6 [Source:NCBI gene;Acc:702903]	-0.70185693	0.000871158	0.003911703
ENSMMUG00000059872		-1.309268714	0.007458441	0.02495579
ENSMMUG00000010393	cadherin 3 [Source:VGNC Symbol;Acc:VGNC:70971]	0.387160344	0.00052914	0.002535061
ENSMMUG00000011850	cadherin 1 [Source:VGNC Symbol;Acc:VGNC:70803]	-1.031616928	1.81E-07	1.91E-06
ENSMMUG00000012039	cytochrome b5 type B (outer mitochondrial membrane) [Source:NCBI gene;Acc:705093]	1.137474491	0.010264058	0.032674888
ENSMMUG00000018255	nuclear factor of activated T cells 5 [Source:VGNC Symbol;Acc:VGNC:75325]	0.681201438	0.00011501	0.000648224
ENSMMUG00000049699	thioredoxin like 4B [Source:NCBI gene;Acc:706840]	-1.074900184	0.006666686	0.02261175
ENSMMUG00000023312	haptoglobin [Source:NCBI gene;Acc:706640]	-1.189661304	0.002019044	0.008131263
ENSMMUG00000016717	polycystin 1 like 3, transient receptor potential channel interacting [Source:VGNC Symbol;Acc:VGNC:75846]	-1.190169935	9.33E-05	0.000536142
ENSMMUG00000009688	IST1 factor associated with ESCRT-III [Source:VGNC Symbol;Acc:VGNC:73675]	-1.00393539	1.89E-05	0.000128838
ENSMMUG00000010878	adaptor related protein complex 1 subunit gamma 1 [Source:VGNC Symbol;Acc:VGNC:69958]	-0.959163709	8.94E-09	1.20E-07
ENSMMUG00000052324	MARVEL domain containing 3 [Source:NCBI gene;Acc:710448]	-0.586462129	0.000916161	0.004076666
ENSMMUG00000002018	tyrosine aminotransferase [Source:VGNC Symbol;Acc:VGNC:78100]	-0.593252376	8.03E-06	5.98E-05
ENSMMUG00000005751	adenosine deaminase tRNA specific 1 [Source:VGNC Symbol;Acc:VGNC:69504]	-1.646274042	0.001322372	0.005619281
ENSMMUG00000050837		-2.332119629	0.002675047	0.010336784
ENSMMUG00000003916	contactin associated protein family member 4 [Source:VGNC Symbol;Acc:VGNC:71209]	-1.232586222	0.012143871	0.037633925
ENSMMUG00000010610	WW domain containing oxidoreductase [Source:VGNC Symbol;Acc:VGNC:78805]	1.48511714	1.67E-06	1.43E-05

ENSMMUG00000053231		-0.583185619	0.002337821	0.009206198
ENSMMUG00000037756	MAF bZIP transcription factor [Source:NCBI gene;Acc:106995090]	0.549853328	0.002478216	0.009694421
ENSMMUG00000005334	phospholipase C gamma 2 [Source:VGNC Symbol;Acc:VGNC:76170]	-0.60408952	2.32E-05	0.00015475
ENSMMUG00000062047		-0.909222659	0.006649849	0.022578894
ENSMMUG00000057329		1.754835116	0.000126424	0.000706875
ENSMMUG00000020114	cadherin 13 [Source:VGNC Symbol;Acc:VGNC:70807]	-1.566795825	0.0008619	0.003873913
ENSMMUG00000011893	KIAA0513 [Source:VGNC Symbol;Acc:VGNC:73925]	-0.816526987	1.27E-05	8.97E-05
ENSMMUG00000011894	CBY1 interacting BAR domain containing 2 [Source:VGNC Symbol;Acc:VGNC:72335]	-0.814848679	0.00184135	0.007497461
ENSMMUG00000061995		0.991835816	3.01E-09	4.44E-08
ENSMMUG00000020592	Gse1 coiled-coil protein [Source:VGNC Symbol;Acc:VGNC:73254]	-0.760097992	0.005625736	0.019541662
ENSMMUG00000006763	cytochrome c oxidase subunit IV isoform 1 [Source:NCBI gene;Acc:693811]	0.691694056	2.88E-05	0.000186897
ENSMMUG00000060536		-2.10005697	0.00013326	0.00074067
ENSMMUG00000054493		-0.775810587	7.40E-08	8.48E-07
ENSMMUG00000017615	zinc finger CCHC-type containing 14 [Source:VGNC Symbol;Acc:VGNC:79420]	-1.156012388	3.00E-20	1.91E-18
ENSMMUG00000022867	kelch domain containing 4 [Source:VGNC Symbol;Acc:VGNC:74122]	0.654146564	0.000469856	0.002282957
ENSMMUG00000058437		1.564308906	1.61E-10	2.97E-09
ENSMMUG00000052851	solute carrier family 7 member 5 [Source:NCBI gene;Acc:695886]	0.921452849	0.000346369	0.00173469
ENSMMUG00000064830		-0.817268838	0.000412123	0.002027788
ENSMMUG00000054935		-1.164313489	0.015026007	0.045004208
ENSMMUG00000008945	zinc finger protein 407 [Source:NCBI gene;Acc:695303]	-4.147461906	1.15E-09	1.81E-08
ENSMMUG00000008942	carnosine dipeptidase 1 [Source:VGNC Symbol;Acc:VGNC:71257]	-4.630068599	6.28E-11	1.23E-09
ENSMMUG00000058864		-2.516854955	0.011245742	0.035313277
ENSMMUG00000051690		-2.78190505	0.005534395	0.019281694
ENSMMUG00000015702	divergent protein kinase domain 1C [Source:NCBI gene;Acc:699537]	-4.798825596	5.07E-06	3.96E-05
ENSMMUG00000020905	chromosome 18 C18orf63 homolog [Source:VGNC Symbol;Acc:VGNC:70465]	-3.312259372	2.75E-06	2.26E-05
ENSMMUG00000056477		0.368650698	0.015501561	0.046231049
ENSMMUG00000050516		0.433171309	0.004899058	0.017423154
ENSMMUG00000060966		-1.868414228	0.001834908	0.0074734
ENSMMUG00000018275	docking protein 6 [Source:VGNC Symbol;Acc:VGNC:71915]	0.943725254	0.00034951	0.001747365
ENSMMUG00000057825		3.015547595	5.58E-05	0.000339743
ENSMMUG00000001245	zinc finger protein 532 [Source:NCBI gene;Acc:701146]	-4.972975639	5.09E-08	5.98E-07
ENSMMUG00000061572	Metazoan signal recognition particle RNA [Source:RFAM;Acc:RF00017]	-5.500736193	2.11E-10	3.77E-09
ENSMMUG00000065131		-3.655513919	8.65E-05	0.000500673
ENSMMUG00000012001	transcription factor 4 [Source:VGNC Symbol;Acc:VGNC:78288]	0.980486047	3.87E-06	3.10E-05
ENSMMUG00000007816	SMAD family member 2 [Source:VGNC Symbol;Acc:VGNC:77810]	-2.309987188	6.35E-12	1.47E-10

ENSMMUG00000015952	katanin catalytic subunit A1 like 2 [Source:VGNC Symbol;Acc:VGNC:73904]	1.63838486	0.000131353	0.000731519
ENSMMUG00000043738		1.637939679	0.001711924	0.007023496
ENSMMUG00000004498	protein inhibitor of activated STAT 2 [Source:VGNC Symbol;Acc:VGNC:75815]	1.434864046	0.000356146	0.001775413
ENSMMUG00000002742	ST8 alpha-N-acetyl-neuraminide alpha-2,8-sialyltransferase 5 [Source:VGNC Symbol;Acc:VGNC:77942]	-2.597900118	7.20E-29	1.16E-26
ENSMMUG00000017246	lipoxygenase homology domains 1 [Source:VGNC Symbol;Acc:VGNC:74368]	-1.854909022	2.43E-23	2.08E-21
ENSMMUG00000017243	ring finger protein 165 [Source:VGNC Symbol;Acc:VGNC:76877]	-1.170515875	0.000147242	0.000811304
ENSMMUG00000004997	SET binding protein 1 [Source:NCBI gene;Acc:699652]	1.470832643	0.001306959	0.005564065
ENSMMUG00000049194		-1.915999043	0.0027796	0.010690712
ENSMMUG00000006866	CUGBP Elav-like family member 4 [Source:VGNC Symbol;Acc:VGNC:70945]	-2.260314679	0.005046828	0.017844595
ENSMMUG00000006864	KIAA1328 ortholog [Source:NCBI gene;Acc:709850]	-2.899731368	0.00489062	0.017406388
ENSMMUG00000018785	elongator acetyltransferase complex subunit 2 [Source:VGNC Symbol;Acc:VGNC:72069]	-0.485450173	0.000156138	0.000856956
ENSMMUG00000018777	regulation of nuclear pre-mRNA domain containing 1A [Source:VGNC Symbol;Acc:VGNC:77103]	-0.498132122	0.016154678	0.047859089
ENSMMUG00000003302	dystrobrevin alpha [Source:VGNC Symbol;Acc:VGNC:72007]	-1.469097443	6.25E-05	0.000375669
ENSMMUG00000017276	nucleolar protein 4 [Source:VGNC Symbol;Acc:VGNC:75366]	-1.057168409	2.65E-06	2.19E-05
ENSMMUG00000014451	ASXL transcriptional regulator 3 [Source:VGNC Symbol;Acc:VGNC:70152]	-0.674623428	2.14E-05	0.000143747
ENSMMUG00000016674	beta-1,4-galactosyltransferase 6 [Source:VGNC Symbol;Acc:VGNC:70213]	-2.279286513	0.007879534	0.026177376
ENSMMUG00000052277	transthyretin [Source:NCBI gene;Acc:705943]	-3.586318256	9.76E-10	1.56E-08
ENSMMUG00000021095	desmoglein 2 [Source:VGNC Symbol;Acc:VGNC:72002]	-3.655427386	1.51E-05	0.000104837
ENSMMUG00000021094	desmoglein 3 [Source:VGNC Symbol;Acc:VGNC:72003]	-3.088921182	0.000120778	0.00067801
ENSMMUG00000011690	desmocollin 1 [Source:VGNC Symbol;Acc:VGNC:71996]	-1.235105182	0.001472074	0.006162058
ENSMMUG00000060604		-1.386188032	0.006727266	0.022786581
ENSMMUG00000010944	GATA binding protein 6 [Source:VGNC Symbol;Acc:VGNC:72891]	-0.851086113	5.19E-05	0.000318318
ENSMMUG00000064806		-0.732899869	0.01060238	0.033576599
ENSMMUG00000019892	myomesin 1 [Source:VGNC Symbol;Acc:VGNC:75123]	-1.246312614	9.74E-12	2.20E-10
ENSMMUG00000052605	Metazoan signal recognition particle RNA [Source:RFAM;Acc:RF00017]	-2.874336717	3.21E-05	0.000206323
ENSMMUG00000005086	homeobox protein TGIF1 [Source:NCBI gene;Acc:694580]	1.512863967	2.30E-10	4.09E-09
ENSMMUG00000041530	Metazoan signal recognition particle RNA [Source:RFAM;Acc:RF00017]	-0.431186214	0.008370306	0.027585617
ENSMMUG00000019418	erythrocyte membrane protein band 4.1 like 3 [Source:VGNC Symbol;Acc:VGNC:72233]	2.016780134	1.12E-30	2.18E-28
ENSMMUG00000016302	L3MBTL histone methyl-lysine binding protein 4 [Source:VGNC Symbol;Acc:VGNC:74133]	0.504689683	0.000515129	0.002473856
ENSMMUG00000000768	protein tyrosine phosphatase receptor type M [Source:VGNC Symbol;Acc:VGNC:76619]	0.594954773	0.000116492	0.000655787
ENSMMUG00000015761	microtubule crosslinking factor 1 [Source:VGNC Symbol;Acc:VGNC:74852]	-0.359700097	0.011487782	0.035998473
ENSMMUG00000053008		-1.505505192	1.71E-07	1.82E-06

ENSMMUG00000049556	NADH:ubiquinone oxidoreductase core subunit V2 [Source:NCBI gene;Acc:706067]	0.819166834	0.008135713	0.026920035
ENSMMUG00000049523	ankyrin repeat domain 12 [Source:NCBI gene;Acc:114673693]	-1.399089566	5.69E-06	4.40E-05
ENSMMUG00000023717	MIER family member 2 [Source:VGNC Symbol;Acc:VGNC:74855]	1.181142727	3.13E-09	4.61E-08
ENSMMUG00000059175		3.127645986	3.45E-38	1.28E-35
ENSMMUG00000053083	C2 calcium dependent domain containing 4C [Source:NCBI gene;Acc:100424440]	3.186463183	1.08E-17	5.25E-16
ENSMMUG00000049968	RNA polymerase mitochondrial [Source:NCBI gene;Acc:100426183]	-0.61623539	6.01E-07	5.72E-06
ENSMMUG00000055423	fibroblast growth factor 22 [Source:NCBI gene;Acc:702727]	-0.434011959	0.000355223	0.00177144
ENSMMUG00000008672	ring finger protein 126 [Source:VGNC Symbol;Acc:VGNC:76791]	-0.627996513	0.004720007	0.01688917
ENSMMUG00000028948	azurocidin 1 [Source:VGNC Symbol;Acc:VGNC:70202]	2.960393513	1.32E-08	1.73E-07
ENSMMUG00000009674	elastase, neutrophil expressed [Source:VGNC Symbol;Acc:VGNC:72199]	1.50115394	0.016101417	0.047725675
ENSMMUG00000028947	complement factor D [Source:VGNC Symbol;Acc:VGNC:71115]	3.097049108	2.17E-49	1.69E-46
ENSMMUG00000009677	mediator complex subunit 16 [Source:VGNC Symbol;Acc:VGNC:74525]	0.579052448	0.004848838	0.01728363
ENSMMUG00000002278	AT-rich interaction domain 3A [Source:VGNC Symbol;Acc:VGNC:69930]	1.112329204	0.001190547	0.005132198
ENSMMUG00000058621		1.168878224	1.37E-05	9.61E-05
ENSMMUG00000002638	WD repeat domain 18 [Source:VGNC Symbol;Acc:VGNC:79103]	-0.745406139	0.001306998	0.005564065
ENSMMUG00000015306	calponin 2 [Source:NCBI gene;Acc:721164]	0.993951452	2.87E-12	6.95E-11
ENSMMUG00000015303	ATP binding cassette subfamily A member 7 [Source:VGNC Symbol;Acc:VGNC:69567]	1.036100792	7.80E-12	1.78E-10
ENSMMUG00000058856	Metazoan signal recognition particle RNA [Source:RFAM;Acc:RF00017]	1.235486391	0.000307204	0.001565947
ENSMMUG00000015301	Rho GTPase activating protein 45 [Source:VGNC Symbol;Acc:VGNC:69847]	0.346866136	0.002299025	0.009070679
ENSMMUG00000010886	strawberry notch homolog 2 [Source:VGNC Symbol;Acc:VGNC:77123]	0.896088954	8.64E-06	6.37E-05
ENSMMUG00000058928	Metazoan signal recognition particle RNA [Source:RFAM;Acc:RF00017]	2.083803959	5.60E-13	1.51E-11
ENSMMUG00000022100	midnolin [Source:VGNC Symbol;Acc:VGNC:82186]	1.58296772	6.45E-15	2.30E-13
ENSMMUG00000003047	DAZ associated protein 1 [Source:VGNC Symbol;Acc:VGNC:71738]	0.289437303	0.001251276	0.005365944
ENSMMUG00000022104	family with sequence similarity 174 member C [Source:NCBI gene;Acc:706393]	0.548546797	3.16E-06	2.57E-05
ENSMMUG00000003043	PWWP domain containing 3A, DNA repair factor [Source:VGNC Symbol;Acc:VGNC:76565]	-0.734625717	1.47E-05	0.000102222
ENSMMUG00000003056	proprotein convertase subtilisin/kexin type 4 [Source:VGNC Symbol;Acc:VGNC:75786]	0.436994027	0.000171421	0.000930636
ENSMMUG00000009017	transcription factor 3 [Source:VGNC Symbol;Acc:VGNC:78287]	0.452504031	1.88E-06	1.59E-05
ENSMMUG00000006620	casein kinase 1 gamma 2 [Source:NCBI gene;Acc:709484]	0.663855581	0.003926801	0.014403747
ENSMMUG00000059725		1.103076018	0.000375247	0.001860053
ENSMMUG00000015663	adaptor related protein complex 3 subunit delta 1 [Source:VGNC Symbol;Acc:VGNC:69969]	0.92918665	8.51E-07	7.82E-06
ENSMMUG00000017949	ornithine decarboxylase antizyme 1 [Source:NCBI gene;Acc:721477]	0.731188689	8.28E-13	2.17E-11
ENSMMUG00000000452	LSM7 homolog, U6 small nuclear RNA and mRNA degradation associated [Source:NCBI gene;Acc:721486]	2.153659838	5.95E-05	0.000358842
ENSMMUG00000055417		-0.819649011	3.51E-07	3.52E-06
ENSMMUG00000065054	G protein subunit gamma 7 [Source:NCBI gene;Acc:711335]	1.612018488	1.19E-15	4.64E-14
ENSMMUG00000007268	thimet oligopeptidase 1 [Source:VGNC Symbol;Acc:VGNC:78347]	0.611502122	0.000644799	0.00300399
ENSMMUG00000007485	zinc finger protein 555 [Source:VGNC Symbol;Acc:VGNC:79705]	-0.875491622	0.000111062	0.000627486
ENSMMUG00000002912	TLE family member 6, subcortical maternal complex member [Source:VGNC Symbol;Acc:VGNC:79879]	-0.669524712	0.008735247	0.028613494
ENSMMUG00000002961	G protein subunit alpha 11 [Source:VGNC Symbol;Acc:VGNC:73085]	-0.868664322	9.18E-14	2.75E-12
ENSMMUG00000017036	nicalin [Source:VGNC Symbol;Acc:VGNC:75032]	2.289597702	3.63E-23	3.05E-21
ENSMMUG00000015513	CUGBP Elav-like family member 5 [Source:NCBI gene;Acc:721694]	1.251531406	0.004917163	0.017483107
ENSMMUG00000060497		1.431572269	0.002621162	0.010176234
ENSMMUG00000003439	nuclear factor I C [Source:VGNC Symbol;Acc:VGNC:75336]	0.899010152	0.003377319	0.01260584
ENSMMUG00000044080	mitochondrial ribosomal protein L54 [Source:NCBI gene;Acc:713878]	1.143334512	4.74E-06	3.73E-05
ENSMMUG00000001917	megakaryocyte-associated tyrosine kinase [Source:NCBI gene;Acc:100430078]	1.094317279	1.70E-08	2.20E-07

ENSMMUG00000054579	zinc finger RNA binding protein 2 [Source:NCBI gene;Acc:100430166]	1.259568368	1.04E-05	7.53E-05
ENSMMUG00000022765	death associated protein kinase 3 [Source:VGNC Symbol;Acc:VGNC:71734]	-1.753005806	0.013951668	0.042374755
ENSMMUG00000022767	eukaryotic translation elongation factor 2 [Source:NCBI gene;Acc:721806]	-2.721541379	0.001602739	0.006629853
ENSMMUG00000019564	mitogen-activated protein kinase kinase 2 [Source:VGNC Symbol;Acc:VGNC:81453]	-0.431248813	0.00051728	0.002482486
ENSMMUG00000028928	ankyrin repeat domain 24 [Source:NCBI gene;Acc:721829]	0.732225257	1.48E-07	1.58E-06
ENSMMUG00000028927	Src homology 2 domain containing transforming protein D [Source:VGNC Symbol;Acc:VGNC:77353]	1.052452574	0.011414076	0.0357915
ENSMMUG00000045860	transmembrane and immunoglobulin domain containing 2 [Source:NCBI gene;Acc:721840]	1.774711992	0.003703323	0.013684241
ENSMMUG00000028925	HDGF like 2 [Source:NCBI gene;Acc:693476]	-2.567895589	1.03E-05	7.47E-05
ENSMMUG00000022656	perilipin 4 [Source:NCBI gene;Acc:693610]	-1.437866991	0.000962156	0.004262975
ENSMMUG00000062995		0.819714736	8.05E-06	5.99E-05
ENSMMUG00000058275	arrestin domain containing 5 [Source:NCBI gene;Acc:697277]	0.663163698	0.000944399	0.004186943
ENSMMUG00000012677	lysine demethylase 4B [Source:VGNC Symbol;Acc:VGNC:73930]	-0.658027219	0.00121745	0.005240115
ENSMMUG00000011962	protein tyrosine phosphatase receptor type S [Source:VGNC Symbol;Acc:VGNC:76622]	1.026469116	6.95E-05	0.000412022
ENSMMUG00000016665	NADH:ubiquinone oxidoreductase subunit A11 [Source:NCBI gene;Acc:697843]	-1.007177513	0.000844699	0.003814883
ENSMMUG00000011963	vimentin type intermediate filament associated coiled-coil protein [Source:NCBI gene;Acc:697958]	-0.69978742	1.37E-08	1.80E-07
ENSMMUG00000011964	RAN binding protein 3 [Source:VGNC Symbol;Acc:VGNC:76655]	-0.550714737	0.001777472	0.007258441
ENSMMUG00000016526	solute carrier family 25 member 41 [Source:VGNC Symbol;Acc:VGNC:77581]	-1.67278806	4.32E-05	0.000269938
ENSMMUG00000019314	vav guanine nucleotide exchange factor 1 [Source:VGNC Symbol;Acc:VGNC:78757]	2.571635753	9.81E-09	1.31E-07
ENSMMUG00000001220	adhesion G protein-coupled receptor E1 [Source:VGNC Symbol;Acc:VGNC:69561]	1.142554005	6.01E-07	5.72E-06
ENSMMUG00000063988		1.747209658	2.73E-05	0.000178446
ENSMMUG00000019966	zinc finger protein 557 [Source:NCBI gene;Acc:100424983]	0.844499225	1.37E-10	2.55E-09
ENSMMUG00000057682		0.93581088	1.89E-09	2.88E-08
ENSMMUG00000028907	insulin receptor [Source:NCBI gene;Acc:706009]	2.258477486	9.84E-09	1.32E-07
ENSMMUG00000028906	Rho/Rac guanine nucleotide exchange factor 18 [Source:NCBI gene;Acc:707510]	1.637075	0.000284277	0.001464519
ENSMMUG00000022463	C-type lectin CD209L2 [Source:NCBI gene;Acc:574373]	1.257832202	0.008135356	0.026920035
ENSMMUG00000023303	ecotropic viral integration site 5 like [Source:NCBI gene;Acc:706628]	1.655801087	5.16E-24	4.86E-22
ENSMMUG00000014032	proline rich 36 [Source:NCBI gene;Acc:710443]	2.502060735	0.002650219	0.010263469
ENSMMUG00000010614	C-C motif chemokine ligand 25 [Source:VGNC Symbol;Acc:VGNC:70700]	4.120029965	4.58E-06	3.62E-05
ENSMMUG00000061632	fibrillin 3 [Source:NCBI gene;Acc:709446]	0.987010163	5.63E-06	4.36E-05
ENSMMUG00000016695	CD320 molecule [Source:VGNC Symbol;Acc:VGNC:70763]	-0.892141518	0.008917012	0.029073066
ENSMMUG00000064610	long intergenic non-protein coding RNA 1862 [Source:HGNC Symbol;Acc:HGNC:52681]	1.162452662	2.00E-08	2.55E-07
ENSMMUG00000049311	zinc finger protein 558 [Source:NCBI gene;Acc:712590]	-0.65194245	8.18E-07	7.54E-06
ENSMMUG00000019965	methyl-CpG binding domain protein 3 like 1 [Source:VGNC Symbol;Acc:VGNC:74513]	1.810652845	0.004702891	0.01683222
ENSMMUG00000064532	mucin 16, cell surface associated [Source:NCBI gene;Acc:106996460]	1.581843583	2.95E-22	2.29E-20
ENSMMUG00000018883	zinc finger protein 177 [Source:NCBI gene;Acc:709851]	0.64546467	0.002778164	0.010688119
ENSMMUG00000007875	F-box and leucine rich repeat protein 12 [Source:VGNC Symbol;Acc:VGNC:72502]	-0.929991783	1.46E-05	0.000101622
ENSMMUG00000042707	angiopoietin like 6 [Source:NCBI gene;Acc:106994633]	-1.657919455	5.08E-09	7.22E-08
ENSMMUG00000014897	ribonucleoprotein, PTB binding 1 [Source:NCBI gene;Acc:715250]	0.700301639	0.000928688	0.004126422
ENSMMUG00000003110	tyrosine kinase 2 [Source:VGNC Symbol;Acc:VGNC:78680]	-0.57025484	0.00310651	0.011722978
ENSMMUG00000046883	autophagy related 4D cysteine peptidase [Source:NCBI gene;Acc:713111]	0.952215552	0.011490917	0.036000252
ENSMMUG00000003744	solute carrier family 44 member 2 [Source:VGNC Symbol;Acc:VGNC:77498]	0.794588642	1.66E-08	2.15E-07
ENSMMUG00000055775	transmembrane p24 trafficking protein 1 [Source:NCBI gene;Acc:713618]	2.065106138	5.87E-36	1.83E-33

ENSMMUG00000049703	Metazoan signal recognition particle RNA [Source:RFAM;Acc:RF00017]	2.304167011	5.73E-45	3.65E-42
ENSMMUG00000004400	Yip1 domain family member 2 [Source:VGNC Symbol;Acc:VGNC:79717]	-0.596608912	0.000299011	0.001530863
ENSMMUG00000003611	low density lipoprotein receptor [Source:VGNC Symbol;Acc:VGNC:74248]	0.965707741	0.001669791	0.006876792
ENSMMUG00000003612	SPC24 component of NDC80 kinetochore complex [Source:VGNC Symbol;Acc:VGNC:78002]	1.469783783	0.006062639	0.020842459
ENSMMUG00000023251	KN motif and ankyrin repeat domains 2 [Source:VGNC Symbol;Acc:VGNC:73938]	-0.672802425	0.00632984	0.021623227
ENSMMUG00000043134	coiled-coil domain containing 159 [Source:NCBI gene;Acc:100424702]	1.372411229	0.006074839	0.020874163
ENSMMUG00000022207	phospholipid phosphatase related 2 [Source:NCBI gene;Acc:714388]	1.218383782	0.004221035	0.015350513
ENSMMUG00000010824	SWIM-type zinc finger 7 associated protein 1 [Source:VGNC Symbol;Acc:VGNC:78220]	1.606543607	0.000123306	0.000690821
ENSMMUG00000038989	erythropoietin receptor [Source:NCBI gene;Acc:714546]	1.770917598	0.004446624	0.016041938
ENSMMUG00000002974	acid phosphatase 5, tartrate resistant [Source:VGNC Symbol;Acc:VGNC:69384]	2.16030554	0.014436211	0.043591514
ENSMMUG00000001715	zinc finger protein 627 [Source:VGNC Symbol;Acc:VGNC:79856]	0.606626149	0.00096903	0.004285307
ENSMMUG00000053329		1.208083626	0.000700267	0.003235534
ENSMMUG00000012587	zinc finger protein 69 [Source:NCBI gene;Acc:717030]	-0.788112894	0.00768448	0.025626508
ENSMMUG00000064114	zinc finger protein 709 [Source:NCBI gene;Acc:100427466]	-0.950301954	6.64E-05	0.000395412
ENSMMUG00000005236	zinc finger protein 709 [Source:NCBI gene;Acc:717515]	1.047720224	0.000669498	0.003107701
ENSMMUG00000058614		-2.411651078	0.000195115	0.001047914
ENSMMUG00000016860	zinc finger protein 490 [Source:NCBI gene;Acc:100426568]	-1.28132603	1.44E-05	0.000100435
ENSMMUG00000016862	zinc finger protein 791 [Source:VGNC Symbol;Acc:VGNC:79639]	-1.151919522	0.001169507	0.00505393
ENSMMUG00000005689	peroxiredoxin 2 [Source:NCBI gene;Acc:716665]	-2.052853586	0.016292756	0.048134057
ENSMMUG00000008740	microtubule associated serine/threonine kinase 1 [Source:VGNC Symbol;Acc:VGNC:82182]	-1.043821591	0.011881895	0.037001958
ENSMMUG00000007043	glutaryl-CoA dehydrogenase [Source:VGNC Symbol;Acc:VGNC:72898]	-0.677930703	4.33E-05	0.000270402
ENSMMUG00000019292	nuclear factor I X [Source:VGNC Symbol;Acc:VGNC:75338]	0.278131204	0.004726145	0.016906821
ENSMMUG00000001167	tRNA methyltransferase 1 [Source:VGNC Symbol;Acc:VGNC:79562]	0.444976841	0.014741352	0.044322038
ENSMMUG00000017971	nucleus accumbens associated 1 [Source:VGNC Symbol;Acc:VGNC:74996]	1.494686303	0.00053239	0.00254889
ENSMMUG00000057038		1.457799044	9.97E-05	0.000569095
ENSMMUG00000011427	syntaxin 10 [Source:VGNC Symbol;Acc:VGNC:78040]	1.845964634	3.54E-34	9.20E-32
ENSMMUG00000003555	calcium voltage-gated channel subunit alpha1 A [Source:VGNC Symbol;Acc:VGNC:70498]	0.642170232	0.000318727	0.001617116
ENSMMUG00000058941	Metazoan signal recognition particle RNA [Source:RFAM;Acc:RF00017]	-2.083349772	0.002840612	0.010889529
ENSMMUG00000060000		1.734518333	2.43E-05	0.000161224
ENSMMUG00000005727	protein kinase N1 [Source:VGNC Symbol;Acc:VGNC:76014]	-1.334138172	7.34E-11	1.43E-09
ENSMMUG00000005729	GIPC PDZ domain containing family member 1 [Source:VGNC Symbol;Acc:VGNC:72932]	0.64918412	0.001529848	0.006362997
ENSMMUG00000017906	DnaJ heat shock protein family (Hsp40) member B1 [Source:NCBI gene;Acc:718890]	1.946700905	2.04E-06	1.71E-05
ENSMMUG00000018932	adhesion G protein-coupled receptor E2 [Source:NCBI gene;Acc:654420]	1.731036225	4.49E-23	3.74E-21
ENSMMUG00000060289		1.626191072	1.55E-22	1.24E-20
ENSMMUG00000053889	olfactory receptor 7C1 [Source:NCBI gene;Acc:114674087]	-0.881536302	0.008804887	0.02876769
ENSMMUG00000064639	olfactory receptor 7A10-like [Source:NCBI gene;Acc:114674167]	1.98828752	0.012576072	0.038776222
ENSMMUG00000053457	caspase 14 [Source:NCBI gene;Acc:718049]	2.79905263	5.53E-13	1.49E-11
ENSMMUG00000053220	olfactory receptor 1I1 [Source:NCBI gene;Acc:719077]	2.536539219	4.17E-05	0.000261813
ENSMMUG00000007726	synapse defective Rho GTPase homolog 1 [Source:NCBI gene;Acc:719089]	2.069254479	2.59E-06	2.15E-05
ENSMMUG00000007729	ilvB acetolactate synthase like [Source:VGNC Symbol;Acc:VGNC:73739]	1.62182144	8.41E-07	7.74E-06
ENSMMUG00000028863	WIZ zinc finger [Source:VGNC Symbol;Acc:VGNC:79901]	-0.624264666	0.008835904	0.02883544

ENSMMUG00000016996	cytochrome P450, family 4, subfamily F, polypeptide 22 [Source:NCBI gene;Acc:718303]	0.653255986	0.000585304	0.002777568
ENSMMUG00000010734	cytochrome P450, family 4, subfamily F, polypeptide 8 [Source:NCBI gene;Acc:718335]	0.284902502	0.013147626	0.040290462
ENSMMUG00000057552	hematopoietic SH2 domain containing [Source:NCBI gene;Acc:719440]	3.076996344	0.007394814	0.02477245
ENSMMUG00000004283	calcium and integrin binding family member 3 [Source:VGNC Symbol;Acc:VGNC:71339]	2.113415388	1.77E-06	1.51E-05
ENSMMUG00000000994	epidermal growth factor receptor pathway substrate 15 like 1 [Source:VGNC Symbol;Acc:VGNC:72100]	1.852176579	0.000912515	0.004066576
ENSMMUG00000039774	Metazoan signal recognition particle RNA [Source:RFAM;Acc:RF00017]	1.248853135	2.55E-10	4.51E-09
ENSMMUG00000012959	solute carrier family 35 member E1 [Source:VGNC Symbol;Acc:VGNC:77536]	1.165098306	6.60E-08	7.64E-07
ENSMMUG00000042837	mediator complex subunit 26 [Source:NCBI gene;Acc:100424604]	0.540084998	0.015105472	0.045203591
ENSMMUG00000018503	NACHT and WD repeat domain containing 1 [Source:NCBI gene;Acc:719640]	0.52804842	0.000450158	0.002197201
ENSMMUG00000057655		-0.860569581	3.09E-06	2.52E-05
ENSMMUG00000011588	myosin IXB [Source:HGNC Symbol;Acc:HGNC:7609]	0.555863001	0.00764706	0.025519924
ENSMMUG00000013923	BRISC and BRCA1 A complex member 1 [Source:VGNC Symbol;Acc:VGNC:70126]	-0.485476351	0.010928518	0.034477077
ENSMMUG00000023403	GTP binding protein 3, mitochondrial [Source:VGNC Symbol;Acc:VGNC:73170]	3.360317534	1.26E-05	8.92E-05
ENSMMUG00000000194	solute carrier family 27 member 1 [Source:VGNC Symbol;Acc:VGNC:77438]	0.746297902	8.05E-05	0.000470461
ENSMMUG00000000196	niban apoptosis regulator 3 [Source:VGNC Symbol;Acc:VGNC:75241]	1.162675818	4.98E-05	0.000306165
ENSMMUG00000059371		1.134439551	0.002825456	0.010846255
ENSMMUG00000000198	collagen beta(1-O)galactosyltransferase 1 [Source:NCBI gene;Acc:719980]	0.846330141	3.64E-09	5.31E-08
ENSMMUG00000015476	unc-13 homolog A [Source:NCBI gene;Acc:720000]	0.81902298	2.63E-08	3.27E-07
ENSMMUG00000010996	Janus kinase 3 [Source:VGNC Symbol;Acc:VGNC:73812]	0.569893633	6.09E-07	5.79E-06
ENSMMUG00000038308	coiled-coil domain containing 124 [Source:NCBI gene;Acc:720072]	-0.85566552	0.001121836	0.004876473
ENSMMUG00000007398	potassium calcium-activated channel subfamily N member 1 [Source:VGNC Symbol;Acc:VGNC:74002]	-2.190590305	1.79E-05	0.00012273
ENSMMUG00000007401	arrestin domain containing 2 [Source:VGNC Symbol;Acc:VGNC:69901]	1.088847967	8.61E-07	7.89E-06
ENSMMUG00000003161	microtubule associated serine/threonine kinase 3 [Source:VGNC Symbol;Acc:VGNC:74533]	0.509564754	0.000401899	0.001983052
ENSMMUG00000005028	phosphodiesterase 4C [Source:NCBI gene;Acc:720171]	-1.091367927	4.11E-07	4.05E-06
ENSMMUG00000016354	IQ motif containing N [Source:VGNC Symbol;Acc:VGNC:73683]	-0.654454509	0.001159349	0.005016216
ENSMMUG00000038371	JunD proto-oncogene, AP-1 transcription factor subunit [Source:NCBI gene;Acc:720182]	-0.698742787	0.005724663	0.019828002
ENSMMUG00000013671	elongation factor for RNA polymerase II [Source:VGNC Symbol;Acc:VGNC:72088]	1.28074619	3.69E-18	1.95E-16
ENSMMUG00000016085	KxDL motif containing 1 [Source:NCBI gene;Acc:720235]	0.573348805	0.007598244	0.025375129
ENSMMUG00000054512		1.396458454	0.001121441	0.004876271
ENSMMUG00000009988	transmembrane protein 59 like [Source:VGNC Symbol;Acc:VGNC:78919]	2.067535509	1.06E-26	1.39E-24
ENSMMUG00000064204	kelch like family member 26 [Source:NCBI gene;Acc:719583]	0.981092293	0.000826642	0.003742977
ENSMMUG00000056644		0.578489988	0.011500531	0.03601428
ENSMMUG00000023286	UPF1 RNA helicase and ATPase [Source:VGNC Symbol;Acc:VGNC:79971]	3.033806028	1.84E-50	1.99E-47
ENSMMUG00000050739		1.407005317	0.000180641	0.000976908
ENSMMUG00000019665	SURP and G-patch domain containing 2 [Source:VGNC Symbol;Acc:VGNC:78163]	-0.875951292	0.001873821	0.007614196
ENSMMUG00000008578	hyaluronan and proteoglycan link protein 4 [Source:VGNC Symbol;Acc:VGNC:73261]	-1.150057346	0.000234577	0.001237575
ENSMMUG00000008931	MAU2 sister chromatid cohesion factor [Source:NCBI gene;Acc:720347]	0.579941469	1.01E-06	9.14E-06
ENSMMUG00000003164	GATA zinc finger domain containing 2A [Source:NCBI gene;Acc:720361]	-0.631784351	4.59E-05	0.000284351
ENSMMUG00000011351	YjeF N-terminal domain containing 3 [Source:NCBI gene;Acc:721218]	-0.653100805	0.000145956	0.000805802
ENSMMUG00000054055	zinc finger protein 93 [Source:NCBI gene;Acc:720434]	1.136076714	4.62E-14	1.47E-12
ENSMMUG00000063297		1.325761545	0.004743423	0.016955653
ENSMMUG00000056683		-0.550247013	0.00893694	0.029131266

ENSMMUG00000061085		-0.801808941	1.06E-07	1.18E-06
ENSMMUG00000062866		-1.109447959	0.000130142	0.000725927
ENSMMUG00000047027	zinc finger protein 100-like [Source:NCBI gene;Acc:693860]	-1.011384469	0.000321873	0.001629466
ENSMMUG00000046906		-0.697401031	0.000512938	0.002467559
ENSMMUG00000061186		-0.891320289	0.000456189	0.002223814
ENSMMUG00000060408		-0.921945902	0.008296993	0.027382615
ENSMMUG00000061458	zinc finger protein 681 [Source:NCBI gene;Acc:698090]	-0.986337804	6.34E-05	0.000379815
ENSMMUG00000059910		-0.602930305	4.72E-05	0.000291729
ENSMMUG00000020668	cyclin E1 [Source:VGNC Symbol;Acc:VGNC:70727]	2.970193799	2.30E-39	9.50E-37
ENSMMUG00000063586	zinc finger protein 536 [Source:NCBI gene;Acc:713142]	-1.274362708	0.001404589	0.005921994
ENSMMUG00000058740		-1.062029493	2.17E-10	3.88E-09
ENSMMUG00000057215		-0.963463092	0.003898984	0.014305455
ENSMMUG00000050177		-1.616558545	3.98E-11	8.01E-10
ENSMMUG00000060455		-3.008574347	8.50E-06	6.30E-05
ENSMMUG00000008041	ankyrin repeat domain 27 [Source:VGNC Symbol;Acc:VGNC:69693]	0.723024975	1.89E-07	1.98E-06
ENSMMUG00000015608	tudor domain containing 12 [Source:VGNC Symbol;Acc:VGNC:82254]	-0.657782435	0.002740051	0.010563243
ENSMMUG00000028800	solute carrier family 7 member 9 [Source:VGNC Symbol;Acc:VGNC:77633]	-1.212123201	6.49E-06	4.96E-05
ENSMMUG00000017939	FA core complex associated protein 24 [Source:VGNC Symbol;Acc:VGNC:72388]	-1.213995047	5.03E-06	3.93E-05
ENSMMUG00000028798	rhophilin Rho GTPase binding protein 2 [Source:NCBI gene;Acc:716602]	-0.628046376	0.003134061	0.011804712
ENSMMUG00000011790	G-patch domain containing 1 [Source:VGNC Symbol;Acc:VGNC:72996]	-1.003097443	0.001030008	0.004526419
ENSMMUG00000064983		-0.810061066	3.20E-05	0.000205829
ENSMMUG00000061372		-1.183599216	0.001706589	0.007005707
ENSMMUG00000062413	CCAAT enhancer binding protein gamma [Source:NCBI gene;Acc:704322]	-0.6669669	0.001449594	0.006078843
ENSMMUG00000006128	peptidase D [Source:VGNC Symbol;Acc:VGNC:82205]	-1.323898561	1.18E-07	1.30E-06
ENSMMUG00000011319	potassium channel tetramerization domain containing 15 [Source:VGNC Symbol;Acc:VGNC:73894]	-0.551542356	0.003329043	0.012455489
ENSMMUG00000015744	zinc finger protein 302 [Source:NCBI gene;Acc:706279]	2.208793789	4.47E-21	3.06E-19
ENSMMUG00000059543	zinc finger protein 30 [Source:NCBI gene;Acc:718310]	-0.733975016	1.68E-07	1.79E-06
ENSMMUG00000008313	zinc finger protein 792 [Source:VGNC Symbol;Acc:VGNC:79456]	-0.77794648	4.39E-08	5.24E-07
ENSMMUG00000001538	GRAM domain containing 1A [Source:VGNC Symbol;Acc:VGNC:73029]	0.682473175	7.95E-07	7.35E-06
ENSMMUG00000004843	sodium voltage-gated channel beta subunit 1 [Source:VGNC Symbol;Acc:VGNC:76983]	0.688186884	0.000248751	0.001303524
ENSMMUG00000055747	cytochrome c oxidase subunit 6B [Source:NCBI gene;Acc:692062]	0.764548231	0.00019994	0.001070546
ENSMMUG00000065025		1.296635488	4.64E-12	1.09E-10
ENSMMUG00000002381	lysine methyltransferase 2B [Source:VGNC Symbol;Acc:VGNC:74053]	0.662402476	1.21E-07	1.33E-06

ENSMMUG00000002384	IGF like family receptor 1 [Source:VGNC Symbol;Acc:VGNC:73434]	1.807682285	6.38E-16	2.59E-14
ENSMMUG00000048190	U2 small nuclear RNA auxiliary factor 1 like 4 [Source:NCBI gene;Acc:718865]	0.950096737	0.00145573	0.006099608
ENSMMUG00000062667	leucine rich repeat and fibronectin type III domain containing 3 [Source:NCBI gene;Acc:711565]	-0.822375478	0.002292822	0.009049321
ENSMMUG00000053233		-1.31463661	0.000198702	0.00106473
ENSMMUG00000061035	ZFP82 zinc finger protein [Source:NCBI gene;Acc:719145]	-1.060810797	1.15E-08	1.53E-07
ENSMMUG00000000547	zinc finger protein 566 [Source:VGNC Symbol;Acc:VGNC:81619]	-0.699851204	7.63E-07	7.10E-06
ENSMMUG00000000107	zinc finger protein 585A [Source:NCBI gene;Acc:100424957]	0.401607321	0.006408839	0.021850512
ENSMMUG00000006820	WD repeat domain 87 [Source:NCBI gene;Acc:719441]	0.323299697	0.001108644	0.004825113
ENSMMUG00000028771	Yip1 interacting factor homolog B, membrane trafficking protein [Source:VGNC Symbol;Acc:VGNC:79852]	1.176032631	0.001528909	0.006362997
ENSMMUG00000062296	potassium two pore domain channel subfamily K member 6 [Source:NCBI gene;Acc:714912]	3.294002599	0.00074128	0.003401497
ENSMMUG00000056109	Metazoan signal recognition particle RNA [Source:RFAM;Acc:RF00017]	2.724459458	0.012179803	0.037728602
ENSMMUG00000014273	cation channel sperm associated auxiliary subunit gamma [Source:VGNC Symbol;Acc:VGNC:70626]	2.10641864	6.19E-06	4.75E-05
ENSMMUG00000038221	proteasome 26S subunit, non-ATPase 8 [Source:NCBI gene;Acc:721897]	0.940136693	6.60E-06	5.03E-05
ENSMMUG00000022973	gametogenetin [Source:VGNC Symbol;Acc:VGNC:72740]	0.630156605	2.82E-05	0.000183677
ENSMMUG00000022975	sprouty related EVH1 domain containing 3 [Source:VGNC Symbol;Acc:VGNC:78030]	-4.082050539	6.44E-17	2.92E-15
ENSMMUG00000006429	actinin alpha 4 [Source:VGNC Symbol;Acc:VGNC:69432]	1.024030183	0.006795933	0.022974774
ENSMMUG00000006431	calpain 12 [Source:VGNC Symbol;Acc:VGNC:70473]	0.887370158	0.005608076	0.019485145
ENSMMUG00000004877	heterogeneous nuclear ribonucleoprotein L [Source:VGNC Symbol;Acc:VGNC:81187]	1.208464922	2.20E-05	0.000147831
ENSMMUG00000012787	acid phosphatase 7, tartrate resistant (putative) [Source:NCBI gene;Acc:695532]	1.434167024	0.00133821	0.005674544
ENSMMUG00000005554	mediator complex subunit 29 [Source:VGNC Symbol;Acc:VGNC:74557]	4.147812847	6.83E-09	9.45E-08
ENSMMUG00000038702	ZFP36 ring finger protein [Source:NCBI gene;Acc:722000]	3.590080861	0.001122534	0.004876571
ENSMMUG00000019369	pleckstrin homology and RhoGEF domain containing G2 [Source:VGNC Symbol;Acc:VGNC:76133]	2.956845024	0.007194559	0.024211496
ENSMMUG00000048215		2.412762571	7.78E-09	1.06E-07
ENSMMUG00000022726	dual specificity tyrosine phosphorylation regulated kinase 1B [Source:VGNC Symbol;Acc:VGNC:72016]	-2.126082363	6.58E-07	6.20E-06
ENSMMUG00000022725	fibrillarin [Source:VGNC Symbol;Acc:VGNC:72619]	-2.940346839	8.34E-05	0.00048456
ENSMMUG00000022724		-1.395564621	0.000577898	0.002745476
ENSMMUG00000051261		-1.300373194	2.14E-05	0.00014368
ENSMMUG00000017191	zinc finger protein 546 [Source:VGNC Symbol;Acc:VGNC:79732]	-0.877742827	0.00218528	0.008705733
ENSMMUG00000041976	zinc finger protein 780B [Source:NCBI gene;Acc:701034]	-0.913355823	1.90E-06	1.61E-05
ENSMMUG00000003621	AKT serine/threonine kinase 2 [Source:VGNC Symbol;Acc:VGNC:69777]	-1.248205464	1.71E-12	4.26E-11
ENSMMUG00000053624		-1.04698883	0.000450509	0.00219815
ENSMMUG00000065016		-0.876497597	0.011272463	0.035387005
ENSMMUG00000008946	chromosome 19 C19orf47 homolog [Source:VGNC Symbol;Acc:VGNC:70404]	-1.069346898	2.27E-10	4.04E-09
ENSMMUG00000022625	periaxin [Source:VGNC Symbol;Acc:VGNC:76248]	-1.671707792	2.93E-06	2.40E-05
ENSMMUG00000015603	biliverdin reductase B [Source:VGNC Symbol;Acc:VGNC:70257]	-1.953571791	0.002178922	0.008687807
ENSMMUG00000063763	cytochrome P450 2A13-like [Source:NCBI gene;Acc:114674219]	0.842717989	0.008582086	0.028204028
ENSMMUG00000057008		0.716412268	0.005027332	0.017798101
ENSMMUG00000014345	exosome component 5 [Source:VGNC Symbol;Acc:VGNC:72331]	2.403595235	0.009114411	0.029612735
ENSMMUG00000013422	CEA cell adhesion molecule 4 [Source:NCBI gene;Acc:705453]	1.888822879	0.000120538	0.000676931

ENSMMUG00000059229	CEA cell adhesion molecule 7 [Source:NCBI gene;Acc:705572]	1.603369715	3.53E-20	2.20E-18
ENSMMUG00000022305	Rho guanine nucleotide exchange factor 1 [Source:VGNC Symbol;Acc:VGNC:70012]	-1.805222453	1.97E-14	6.58E-13
ENSMMUG00000017427	ATPase Na+/K+ transporting subunit alpha 3 [Source:NCBI gene;Acc:706513]	-1.417507858	0.010949401	0.034535189
ENSMMUG00000017431	glutamate ionotropic receptor kainate type subunit 5 [Source:VGNC Symbol;Acc:VGNC:73280]	-0.74967352	0.006657398	0.022593584
ENSMMUG00000056518		-0.915115949	0.01080687	0.034154776
ENSMMUG00000050799		3.130626085	0.003959966	0.014502638
ENSMMUG00000064250		2.811852585	0.000272972	0.001414594
ENSMMUG00000049459	pregnancy specific beta-1-glycoprotein 7 (gene/pseudogene) [Source:HGNC Symbol;Acc:HGNC:9524]	-0.479221052	0.004874627	0.017358275
ENSMMUG00000021062	CD177 molecule [Source:VGNC Symbol;Acc:VGNC:70912]	2.441794696	4.98E-06	3.90E-05
ENSMMUG00000003568	testis expressed 101 [Source:VGNC Symbol;Acc:VGNC:79869]	1.066829094	4.51E-05	0.000279988
ENSMMUG00000019265	zinc finger protein 575 [Source:VGNC Symbol;Acc:VGNC:79667]	-1.055506433	1.58E-05	0.000109328
ENSMMUG00000004467	X-ray repair cross complementing 1 [Source:NCBI gene;Acc:711457]	-0.897840483	5.57E-05	0.00033879
ENSMMUG00000004616	plasminogen activator, urokinase receptor [Source:VGNC Symbol;Acc:VGNC:76163]	2.312720813	0.000266498	0.001386168
ENSMMUG00000021267	potassium calcium-activated channel subfamily N member 4 [Source:NCBI gene;Acc:712383]	0.808773528	1.30E-05	9.19E-05
ENSMMUG00000021474	zinc finger protein 45 [Source:NCBI gene;Acc:712591]	-1.121663967	4.59E-12	1.08E-10
ENSMMUG00000064975	zinc finger protein 221 [Source:NCBI gene;Acc:100423743]	-1.091395631	0.000840123	0.003797889
ENSMMUG00000012797	zinc finger protein 233 [Source:VGNC Symbol;Acc:VGNC:79903]	1.42293492	4.29E-11	8.58E-10
ENSMMUG00000012801	zinc finger protein 235 [Source:VGNC Symbol;Acc:VGNC:78825]	1.540582445	4.33E-28	6.39E-26
ENSMMUG00000012802	zinc finger protein 112 [Source:NCBI gene;Acc:713527]	0.613540776	0.001221282	0.005253385
ENSMMUG00000014301	nectin cell adhesion molecule 2 [Source:VGNC Symbol;Acc:VGNC:75396]	-0.940828963	9.91E-09	1.32E-07
ENSMMUG00000014305	apolipoprotein E [Source:HGNC Symbol;Acc:HGNC:613]	-1.31832419	0.014179222	0.042982109
ENSMMUG00000002472	CLPTM1 regulator of GABA type A receptor forward trafficking [Source:VGNC Symbol;Acc:VGNC:71265]	-1.07229252	0.003596448	0.013331479
ENSMMUG00000014155	CLK4 associating serine/arginine rich protein [Source:VGNC Symbol;Acc:VGNC:71198]	-1.109564726	0.014431768	0.043587483
ENSMMUG00000022931	NTPase KAP family P-loop domain containing 1 [Source:VGNC Symbol;Acc:VGNC:75250]	0.955962884	5.54E-09	7.81E-08
ENSMMUG00000018879	exocyst complex component 3 like 2 [Source:NCBI gene;Acc:713992]	1.063627773	2.81E-08	3.49E-07
ENSMMUG00000013477	creatine kinase, M-type [Source:VGNC Symbol;Acc:VGNC:71230]	3.06146054	1.83E-22	1.44E-20
ENSMMUG00000021891	kinesin light chain 3 [Source:VGNC Symbol;Acc:VGNC:73870]	0.676940197	0.004767206	0.017031983
ENSMMUG00000014419	ERCC excision repair 2, TFIIH core complex helicase subunit [Source:VGNC Symbol;Acc:VGNC:72423]	0.435474523	0.000904436	0.004037858
ENSMMUG00000000715	protein phosphatase 1 regulatory subunit 13 like [Source:NCBI gene;Acc:714389]	0.495295738	0.000421861	0.002072071
ENSMMUG00000000717	CD3e molecule associated protein [Source:NCBI gene;Acc:714440]	-1.315918819	3.07E-08	3.77E-07
ENSMMUG00000000718	ERCC excision repair 1, endonuclease non-catalytic subunit [Source:VGNC Symbol;Acc:VGNC:72422]	-0.854558529	4.75E-05	0.000293367
ENSMMUG00000006936	gastric inhibitory polypeptide receptor [Source:VGNC Symbol;Acc:VGNC:73052]	1.141332665	2.03E-10	3.65E-09
ENSMMUG00000032809	mml-mir-642 [Source:miRBase;Acc:MI0007885]	1.074341777	1.94E-07	2.03E-06
ENSMMUG00000006940	glutaminyl-peptide cyclotransferase like [Source:VGNC Symbol;Acc:VGNC:76540]	-0.71755484	0.016202057	0.047959682
ENSMMUG00000051115		-3.283515062	3.20E-10	5.56E-09
ENSMMUG00000017927	protein kinase D2 [Source:VGNC Symbol;Acc:VGNC:76273]	2.216341928	0.00061878	0.002910819
ENSMMUG00000017930	striatin 4 [Source:VGNC Symbol;Acc:VGNC:78128]	3.305131446	0.000291509	0.00149573
ENSMMUG00000011332	Rho GTPase activating protein 35 [Source:VGNC Symbol;Acc:VGNC:69942]	2.015186313	1.93E-05	0.000131513
ENSMMUG00000057361	Metazoan signal recognition particle RNA [Source:RFAM;Acc:RF00017]	2.527429465	1.04E-11	2.32E-10
ENSMMUG00000014723	kaptin, actin binding protein [Source:VGNC Symbol;Acc:VGNC:74204]	-0.825731958	0.000219145	0.001163599

ENSMMUG00000014727	NSF attachment protein alpha [Source:VGNC Symbol;Acc:VGNC:75131]	-1.025817965	5.77E-06	4.45E-05
ENSMMUG00000054090		0.774443411	0.001715211	0.007032868
ENSMMUG00000049999	cone-rod homeobox [Source:NCBI gene;Acc:717550]	-1.069317277	8.25E-05	0.000479942
ENSMMUG00000006857	epididymal sperm binding protein 1 [Source:VGNC Symbol;Acc:VGNC:72046]	-1.539209625	5.59E-07	5.34E-06
ENSMMUG00000014592	zinc finger SWIM-type containing 9 [Source:VGNC Symbol;Acc:VGNC:79949]	0.764526203	0.011873474	0.036983947
ENSMMUG00000017111	caspase recruitment domain-containing protein 8 [Source:NCBI gene;Acc:718576]	-0.654186024	0.013934006	0.04233945
ENSMMUG00000042157	caspase recruitment domain-containing protein 8 [Source:NCBI gene;Acc:718594]	-0.803506747	6.30E-08	7.32E-07
ENSMMUG00000001791	glutamate rich WD repeat containing 1 [Source:VGNC Symbol;Acc:VGNC:73213]	-0.692099481	0.002151442	0.008592898
ENSMMUG00000001793	cytohesin 2 [Source:NCBI gene;Acc:718682]	-0.876573373	0.000146234	0.000806753
ENSMMUG00000022155	sulfotransferase family 2B member 1 [Source:NCBI gene;Acc:718739]	0.599554272	0.00775881	0.025837518
ENSMMUG00000022159	sphingosine kinase 2 [Source:VGNC Symbol;Acc:VGNC:77960]	-0.799941415	0.005475287	0.019128041
ENSMMUG00000064152	netrin 5 [Source:NCBI gene;Acc:718157]	-0.806359261	2.20E-05	0.00014736
ENSMMUG00000019738	galactoside 2-alpha-L-fucosyltransferase 2 [Source:NCBI gene;Acc:722582]	-1.503883506	0.005972529	0.020562919
ENSMMUG00000055036	MEF2 activating motif and SAP domain containing transcriptional regulator [Source:NCBI gene;Acc:718823]	-0.919647143	0.000492801	0.002380484
ENSMMUG00000018415	Ras interacting protein 1 [Source:HGNC Symbol;Acc:HGNC:24716]	-1.536474577	7.92E-09	1.07E-07
ENSMMUG00000004019	izumo sperm-egg fusion 1 [Source:VGNC Symbol;Acc:VGNC:73670]	-0.891067496	0.000262718	0.001370569
ENSMMUG00000028736	hydroxysteroid 17-beta dehydrogenase 14 [Source:NCBI gene;Acc:106994816]	-1.039855581	3.87E-05	0.000244553
ENSMMUG00000009222	pleckstrin homology domain containing A4 [Source:VGNC Symbol;Acc:VGNC:76027]	-1.593420731	0.000188248	0.001013751
ENSMMUG00000048854	TUB like protein 2 [Source:NCBI gene;Acc:718915]	-1.017309695	0.004858677	0.017310271
ENSMMUG00000007538	transient receptor potential cation channel subfamily M member 4 [Source:VGNC Symbol;Acc:VGNC:79105]	2.428073506	5.41E-20	3.26E-18
ENSMMUG00000022116	PIH1 domain containing 1 [Source:VGNC Symbol;Acc:VGNC:75868]	1.450511847	0.001026831	0.004515959
ENSMMUG00000003819	nitric oxide synthase interacting protein [Source:VGNC Symbol;Acc:VGNC:75207]	2.039793319	4.49E-16	1.87E-14
ENSMMUG00000012937	proline rich 12 [Source:NCBI gene;Acc:719344]	2.686001518	3.75E-33	9.23E-31
ENSMMUG00000028733	RAS related [Source:VGNC Symbol;Acc:VGNC:76937]	2.42965509	9.96E-14	2.96E-12
ENSMMUG00000012938	SR-related CTD associated factor 1 [Source:VGNC Symbol;Acc:VGNC:77137]	3.092263403	0.0002322	0.001225495
ENSMMUG00000003926	BCL2 like 12 [Source:NCBI gene;Acc:718981]	3.904182613	0.000349586	0.001747365
ENSMMUG00000003029	protein arginine methyltransferase 1 [Source:NCBI gene;Acc:719419]	-1.185597997	3.68E-07	3.67E-06
ENSMMUG00000014735	carnitine palmitoyltransferase 1C [Source:NCBI gene;Acc:719429]	-1.423349784	1.50E-07	1.60E-06
ENSMMUG00000020175	mediator complex subunit 25 [Source:VGNC Symbol;Acc:VGNC:74607]	-1.333457784	0.000506244	0.002438702
ENSMMUG00000011627	interleukin 4 induced 1 [Source:VGNC Symbol;Acc:VGNC:73625]	2.202698376	5.02E-11	9.94E-10
ENSMMUG00000044817	nucleoporin 62 [Source:NCBI gene;Acc:100426308]	2.826354855	1.70E-49	1.40E-46
ENSMMUG00000054473	activating transcription factor 5 [Source:NCBI gene;Acc:719150]	2.047839348	3.08E-20	1.95E-18
ENSMMUG00000050456	Metazoan signal recognition particle RNA [Source:RFAM;Acc:RF00017]	1.73764087	0.008865783	0.028919486
ENSMMUG00000064146	Metazoan signal recognition particle RNA [Source:RFAM;Acc:RF00017]	3.189099155	1.78E-23	1.58E-21
ENSMMUG00000054052	sialic acid binding Ig like lectin 11 [Source:NCBI gene;Acc:719127]	3.073979031	1.38E-07	1.49E-06
ENSMMUG00000010837	VRK serine/threonine kinase 3 [Source:VGNC Symbol;Acc:VGNC:81605]	2.314443331	2.55E-05	0.000167601

ENSMMUG00000001427	myosin heavy chain 14 [Source:VGNC Symbol;Acc:VGNC:75006]	0.863565501	0.008793983	0.028752162
ENSMMUG00000023418	nuclear receptor subfamily 1 group H member 2 [Source:VGNC Symbol;Acc:VGNC:75504]	0.697176483	1.24E-07	1.36E-06
ENSMMUG00000021059	leucine rich repeat containing 4B [Source:VGNC Symbol;Acc:VGNC:74350]	0.427079424	0.012889089	0.039602037
ENSMMUG00000056558		0.389208789	0.007670661	0.025586509
ENSMMUG00000046099	chromosome 19 C19orf81 homolog [Source:NCBI gene;Acc:719661]	2.442776175	0.002324426	0.009156018
ENSMMUG00000059087		3.150800288	0.000239791	0.001261285
ENSMMUG00000009357	SH3 and multiple ankyrin repeat domains 1 [Source:VGNC Symbol;Acc:VGNC:77236]	-0.794691134	0.009570171	0.030815731
ENSMMUG00000012841	kallikrein 3 [Source:NCBI gene;Acc:719444]	1.037223617	0.000462388	0.002252193
ENSMMUG00000065035	kallikrein-2 [Source:NCBI gene;Acc:719436]	-0.72723088	0.003407226	0.012699315
ENSMMUG00000060764		0.561110294	2.46E-06	2.04E-05
ENSMMUG00000013597	kallikrein related peptidase 5 [Source:VGNC Symbol;Acc:VGNC:74191]	-0.540673026	0.010485163	0.033273013
ENSMMUG00000061396	kallikrein related peptidase 6 [Source:NCBI gene;Acc:719776]	-0.603259674	0.002401219	0.009432016
ENSMMUG00000047370	IgLON family member 5 [Source:NCBI gene;Acc:719890]	-0.675353567	0.012835785	0.039455547
ENSMMUG00000044652	sialic acid-binding Ig-like lectin 13 [Source:NCBI gene;Acc:719667]	-0.677651585	0.000338074	0.001701042
ENSMMUG00000012764	zinc finger protein 175 [Source:VGNC Symbol;Acc:VGNC:78856]	-0.68615705	0.007090008	0.02389406
ENSMMUG00000012761	sialic acid binding Ig like lectin 5 [Source:NCBI gene;Acc:719730]	-0.861686512	1.75E-06	1.50E-05
ENSMMUG00000013053	sperm acrosome associated 6 [Source:NCBI gene;Acc:114674203]	-1.204212762	2.02E-07	2.11E-06
ENSMMUG00000053150	Metazoan signal recognition particle RNA [Source:RFAM;Acc:RF00017]	-1.210071727	0.005361896	0.018764628
ENSMMUG00000051078		-0.551711429	0.010630625	0.033658445
ENSMMUG00000002310	zinc finger protein 577 [Source:VGNC Symbol;Acc:VGNC:79733]	-0.833892352	9.05E-08	1.02E-06
ENSMMUG00000023136	zinc finger protein 613 [Source:VGNC Symbol;Acc:VGNC:79548]	-0.65586134	1.17E-06	1.04E-05
ENSMMUG00000052090		-0.632864822	5.90E-08	6.88E-07
ENSMMUG00000023137	zinc finger protein 350 [Source:VGNC Symbol;Acc:VGNC:79041]	-1.169191502	4.07E-07	4.02E-06
ENSMMUG00000003712	zinc finger protein 615 [Source:VGNC Symbol;Acc:VGNC:79454]	-0.959579535	0.000307837	0.001568035
ENSMMUG00000041450	zinc finger protein 432 [Source:NCBI gene;Acc:100424341]	-1.463514473	4.88E-10	8.20E-09
ENSMMUG00000001768	zinc finger protein 616 [Source:NCBI gene;Acc:719902]	-0.771813567	9.19E-05	0.000528917
ENSMMUG00000020234	zinc finger protein 480 [Source:NCBI gene;Acc:106994837]	-0.415139037	0.000854466	0.003847841
ENSMMUG00000033290	mml-mir-643 [Source:miRBase;Acc:MI0007886]	-1.406940595	8.47E-07	7.79E-06
ENSMMUG00000047412	zinc finger protein 28 [Source:NCBI gene;Acc:100430953]	-1.571329198	0.004984361	0.017669867
ENSMMUG00000058137	zinc finger protein 816 [Source:NCBI gene;Acc:720058]	0.893039687	0.003055864	0.011578754
ENSMMUG00000064519		-1.325379175	0.000722521	0.00332194
ENSMMUG00000057187		0.761726834	7.05E-08	8.11E-07
ENSMMUG00000060222		-0.517780189	0.000104959	0.000596609
ENSMMUG00000007972	protein kinase C gamma [Source:VGNC Symbol;Acc:VGNC:76413]	0.511970998	0.012604896	0.038830945
ENSMMUG00000055976	CD99 antigen [Source:NCBI gene;Acc:106995376]	1.586469536	1.10E-08	1.47E-07

ENSMMUG00000051966	-1.267496434	3.95E-07	3.91E-06
ENSMMUG00000042225	-0.709798225	0.000877649	0.003938326
ENSMMUG00000042267	2.108111873	0.00303021	0.011500035
ENSMMUG00000061536	-1.787967985	1.31E-06	1.15E-05

SD 35 vs. Baseline

Ensembl Gene ID	Description	Log2(FC)	p-value	FDR
ENSMMUG00000000632	SH3 binding domain protein 5 like [Source:VGNC Symbol;Acc:VGNC:77334]	4.43333743	1.43E-13	3.64E-11
ENSMMUG00000057389	LY6/PLAUR domain containing 8 [Source:NCBI gene;Acc:710465]	-1.271092081	1.57E-05	0.000212857
ENSMMUG00000017368	olfactory receptor 2G6 [Source:NCBI gene;Acc:694380]	-1.425326456	9.62E-07	2.06E-05
ENSMMUG00000038524	olfactory receptor 2M4 [Source:NCBI gene;Acc:100424541]	1.861727464	7.64E-09	3.67E-07
ENSMMUG00000049432	olfactory receptor 2T33 [Source:NCBI gene;Acc:706331]	0.920205495	9.39E-05	0.000947017
ENSMMUG00000004566	zinc finger protein 124 [Source:VGNC Symbol;Acc:VGNC:78836]	-4.742714616	0.000553951	0.004075047
ENSMMUG00000000021	catsper channel auxiliary subunit epsilon [Source:VGNC Symbol;Acc:VGNC:70657]	-3.301550141	0.006076311	0.027826936
ENSMMUG00000004265	phospholipase D family member 5 [Source:VGNC Symbol;Acc:VGNC:76120]	-1.794470578	0.011027113	0.044347133
ENSMMUG00000005668	kynurenine 3-monooxygenase [Source:VGNC Symbol;Acc:VGNC:74197]	0.837628433	0.000505199	0.003761083
ENSMMUG00000064824	gremlin 2, DAN family BMP antagonist [Source:NCBI gene;Acc:708280]	1.889273353	0.000227927	0.001960111
ENSMMUG00000061845		1.430148638	0.003367318	0.017462258
ENSMMUG00000001060	ryanodine receptor 2 [Source:VGNC Symbol;Acc:VGNC:76954]	0.934050468	0.000164632	0.001490054
ENSMMUG00000015132	galectin 8 [Source:VGNC Symbol;Acc:VGNC:74258]	-2.075691248	6.38E-05	0.000683483
ENSMMUG00000046785		-1.462248774	0.01032193	0.042309179
ENSMMUG00000063981	nucleoside-triphosphatase, cancer-related [Source:NCBI gene;Acc:712959]	3.554698745	1.43E-08	6.21E-07
ENSMMUG00000012068	signal induced proliferation associated 1 like 2 [Source:NCBI gene;Acc:100425123]	2.169035295	2.03E-05	0.0002648
ENSMMUG00000005243	exocyst complex component 8 [Source:VGNC Symbol;Acc:VGNC:72330]	-1.273569789	0.008239255	0.035389615
ENSMMUG00000005801	glyceronephosphate O-acyltransferase [Source:VGNC Symbol;Acc:VGNC:73100]	-1.499353419	0.000473661	0.003542517
ENSMMUG00000009508	TATA-box binding protein associated factor 5 like [Source:VGNC Symbol;Acc:VGNC:78148]	2.282969736	0.000424382	0.003256352
ENSMMUG00000022094	ATP binding cassette subfamily B member 10 [Source:NCBI gene;Acc:574117]	1.778834143	4.43E-07	1.08E-05
ENSMMUG00000061675	5S ribosomal RNA [Source:RFAM;Acc:RF00001]	4.950822723	0.006241112	0.028401645
ENSMMUG00000050099	5S ribosomal RNA [Source:RFAM;Acc:RF00001]	3.797714807	0.000899721	0.006053686
ENSMMUG00000017789	pogo transposable element derived with KRAB domain [Source:VGNC Symbol;Acc:VGNC:76066]	1.954852142	1.33E-06	2.70E-05
ENSMMUG00000017790	transcriptional adaptor 1 [Source:VGNC Symbol;Acc:VGNC:78246]	2.081511822	2.14E-05	0.000276921
ENSMMUG00000023462	immunoglobulin like domain containing receptor 2 [Source:VGNC Symbol;Acc:VGNC:73619]	-2.551596868	0.001333637	0.00837532
ENSMMUG00000051788	dual specificity phosphatase 27, atypical [Source:NCBI gene;Acc:697299]	-2.019677977	0.01289322	0.049907507
ENSMMUG00000009184	myelin protein zero like 1 [Source:NCBI gene;Acc:698437]	2.366280834	3.36E-16	1.68E-13
ENSMMUG00000060218	chemokine (C motif) ligand 1 [Source:NCBI gene;Acc:574367]	4.949973435	3.43E-06	5.98E-05
ENSMMUG00000056707		1.869906349	0.011174482	0.044842737
ENSMMUG00000059777		1.013759285	0.000310333	0.002505395
ENSMMUG00000021078	ATPase Na+/K+ transporting subunit beta 1 [Source:VGNC Symbol;Acc:VGNC:70176]	-1.477609543	0.001568069	0.009597319
ENSMMUG00000021080	basic leucine zipper nuclear factor 1 [Source:VGNC Symbol;Acc:VGNC:70258]	0.740336065	0.001431194	0.00887654

ENSMMUG00000013039	solute carrier family 19 member 2 [Source:VGNC Symbol;Acc:VGNC:81571]	-0.683138609	5.17E-05	0.000574521
ENSMMUG00000042510	coagulation factor V [Source:NCBI gene;Acc:704695]	0.574455845	0.000847929	0.005740844
ENSMMUG00000016579	selectin L [Source:NCBI gene;Acc:701419]	1.539565168	1.22E-05	0.000172744
ENSMMUG00000016580	selectin E [Source:VGNC Symbol;Acc:VGNC:77156]	1.545370818	1.17E-07	3.44E-06
ENSMMUG00000016582	chromosome 1 C1orf112 homolog [Source:VGNC Symbol;Acc:VGNC:70385]	1.245527055	5.24E-05	0.000579964
ENSMMUG00000059773	methyltransferase like 11B [Source:NCBI gene;Acc:705503]	-1.061225056	0.0024902	0.013700315
ENSMMUG00000063298		-1.15081	0.00432618	0.021224558
ENSMMUG00000054261		2.009116974	0.000451598	0.003422366
ENSMMUG00000022798	aspartyl-tRNA synthetase 2, mitochondrial [Source:VGNC Symbol;Acc:VGNC:71708]	0.79784654	0.002066633	0.011914079
ENSMMUG00000056971	Metazoan signal recognition particle RNA [Source:RFAM;Acc:RF00017]	1.696441203	1.14E-06	2.37E-05
ENSMMUG00000022801	zinc finger and BTB domain containing 37 [Source:VGNC Symbol;Acc:VGNC:79702]	1.449049087	0.006637926	0.029811074
ENSMMUG00000000629	serpin family C member 1 [Source:VGNC Symbol;Acc:VGNC:77360]	1.074692828	2.70E-05	0.000336586
ENSMMUG00000000546	KIAA0040 ortholog [Source:NCBI gene;Acc:100423509]	-1.24496728	0.000632961	0.004549137
ENSMMUG00000020320	protein transport protein Sec16B [Source:NCBI gene;Acc:709635]	0.787020243	0.000913817	0.006116886
ENSMMUG00000021073	RAS protein activator like 2 [Source:VGNC Symbol;Acc:VGNC:76670]	1.585196858	1.57E-06	3.07E-05
ENSMMUG00000055256	family with sequence similarity 163 member A [Source:NCBI gene;Acc:718704]	0.899127628	0.003359613	0.017443167
ENSMMUG00000031279	torsin A interacting protein 2 [Source:NCBI gene;Acc:716654]	0.927337477	0.000133689	0.001269163
ENSMMUG00000054568		1.073422001	0.001718972	0.010288517
ENSMMUG00000061785		1.296271485	2.65E-08	1.03E-06
ENSMMUG00000022884	glutamate-ammonia ligase [Source:NCBI gene;Acc:715952]	2.748949096	4.43E-06	7.33E-05
ENSMMUG00000049444		2.643226912	0.002581376	0.014100534
ENSMMUG00000031291	regulator of G protein signaling like 1 [Source:NCBI gene;Acc:715693]	1.178533	3.22E-08	1.20E-06
ENSMMUG00000005634	ribonuclease L [Source:VGNC Symbol;Acc:VGNC:82223]	-0.915129946	0.004010174	0.020052414
ENSMMUG00000001479	laminin subunit gamma 2 [Source:VGNC Symbol;Acc:VGNC:74233]	-2.849082081	1.12E-09	7.74E-08
ENSMMUG00000001481	nicotinamide nucleotide adenylyltransferase 2 [Source:VGNC Symbol;Acc:VGNC:75193]	-2.137839582	0.000394189	0.003064452
ENSMMUG00000013879	tRNA methyltransferase 1 like [Source:VGNC Symbol;Acc:VGNC:79048]	1.635278074	0.001122074	0.007260547
ENSMMUG00000009076	proteoglycan 4 [Source:NCBI gene;Acc:716849]	3.731265204	4.14E-07	1.02E-05
ENSMMUG00000016857	prostaglandin-endoperoxide synthase 2 [Source:VGNC Symbol;Acc:VGNC:76589]	-1.319741373	1.70E-07	4.80E-06
ENSMMUG00000062021		-1.641775562	0.000298455	0.002436724
ENSMMUG00000022647	cell division cycle 73 [Source:VGNC Symbol;Acc:VGNC:70766]	-0.650301483	0.01236353	0.048448349
ENSMMUG00000056654	translationally-controlled tumor protein [Source:NCBI gene;Acc:711798]	2.221513342	0.00196761	0.011446075
ENSMMUG00000021082	potassium sodium-activated channel subfamily T member 2 [Source:VGNC Symbol;Acc:VGNC:73857]	1.233491091	2.04E-05	0.000265958
ENSMMUG00000045497	complement factor H [Source:NCBI gene;Acc:717346]	-1.201724219	0.002426365	0.013411517
ENSMMUG00000000243	complement factor H related 2 [Source:NCBI gene;Acc:106994228]	-1.658405408	0.001410957	0.008780338

ENSMMUG00000000009	protein tyrosine phosphatase receptor type C [Source:VGNC Symbol;Acc:VGNC:76612]	-2.56386762	1.19E-07	3.48E-06
ENSMMUG00000052462	maestro heat-like repeat-containing protein family member 7 [Source:NCBI gene;Acc:100427155]	-0.684340062	0.001260825	0.007979705
ENSMMUG00000017414	calcium voltage-gated channel subunit alpha1 S [Source:VGNC Symbol;Acc:VGNC:70507]	-1.35271968	8.92E-06	0.000131311
ENSMMUG00000064915	Metazoan signal recognition particle RNA [Source:RFAM;Acc:RF00017]	2.776079633	0.001804437	0.010691801
ENSMMUG00000050920	Metazoan signal recognition particle RNA [Source:RFAM;Acc:RF00017]	2.568508296	0.001646295	0.009958934
ENSMMUG00000021116	ATPase plasma membrane Ca2+ transporting 4 [Source:VGNC Symbol;Acc:VGNC:70183]	1.734752908	5.51E-07	1.30E-05
ENSMMUG00000055783		-4.480366643	9.98E-07	2.11E-05
ENSMMUG00000008460	SRY-box transcription factor 13 [Source:VGNC Symbol;Acc:VGNC:77839]	-3.206833175	0.010575861	0.042951939
ENSMMUG00000063658	leucine rich repeat neuronal 2 [Source:NCBI gene;Acc:100429709]	1.478127334	0.000304829	0.002473242
ENSMMUG00000040888	complement C3b/C4b receptor 1 like [Source:HGNC Symbol;Acc:HGNC:2335]	0.992001629	0.002959872	0.015783557
ENSMMUG00000064202		-1.086002971	0.007551708	0.033121069
ENSMMUG00000016099	hedgehog acyltransferase [Source:NCBI gene;Acc:712333]	1.737376026	0.002346627	0.013053991
ENSMMUG00000015634	centromere protein F [Source:VGNC Symbol;Acc:VGNC:71009]	-1.768445402	0.005698311	0.02644976
ENSMMUG00000058133		-2.101310373	0.000438373	0.00334006
ENSMMUG00000017396	3'(2'), 5'-bisphosphate nucleotidase 1 [Source:NCBI gene;Acc:721277]	-1.397232846	0.000371628	0.002918714
ENSMMUG00000007783	isoleucyl-tRNA synthetase 2, mitochondrial [Source:VGNC Symbol;Acc:VGNC:73458]	-1.078398524	3.91E-07	9.75E-06
ENSMMUG00000007784	RAB3 GTPase activating non-catalytic protein subunit 2 [Source:VGNC Symbol;Acc:VGNC:76499]	0.595822801	0.006782683	0.030335482
ENSMMUG00000018525	presenilin 2 [Source:VGNC Symbol;Acc:VGNC:76436]	-0.833168926	0.002519921	0.013822899
ENSMMUG00000001869	synaptosome associated protein 47 [Source:VGNC Symbol;Acc:VGNC:77800]	1.27719192	0.010576268	0.042951939
ENSMMUG00000023096	obscurin, cytoskeletal calmodulin and titin-interacting RhoGEF [Source:NCBI gene;Acc:694413]	-0.715126508	0.006020393	0.027619483
ENSMMUG00000019513	U2AF homology motif kinase 1 [Source:VGNC Symbol;Acc:VGNC:79198]	3.248617473	1.48E-05	0.0002025
ENSMMUG00000042262	nitric oxide synthase 1 adaptor protein [Source:NCBI gene;Acc:720378]	1.274873659	0.000798167	0.005472305
ENSMMUG00000004042	Fc receptor like A [Source:VGNC Symbol;Acc:VGNC:72526]	1.578450628	0.011781261	0.046758341
ENSMMUG00000054686		2.567510144	1.70E-17	1.10E-14
ENSMMUG00000008251	cilia and flagella associated protein 126 [Source:VGNC Symbol;Acc:VGNC:71150]	2.535586944	0.004654362	0.022545488
ENSMMUG00000004512	Fc fragment of IgE receptor Ig [Source:VGNC Symbol;Acc:VGNC:72629]	1.31434115	0.001364216	0.008530287
ENSMMUG00000004520	NADH:ubiquinone oxidoreductase core subunit S2 [Source:NCBI gene;Acc:719935]	1.230060276	6.47E-06	0.000100248
ENSMMUG00000050419	ADAM metallopeptidase with thrombospondin type 1 motif 4 [Source:NCBI gene;Acc:719927]	1.366710899	6.66E-09	3.27E-07
ENSMMUG00000060555		1.290268485	3.72E-06	6.38E-05
ENSMMUG00000022630	protoporphyrinogen oxidase [Source:VGNC Symbol;Acc:VGNC:76365]	-1.260784032	0.002165111	0.012312825

ENSMMUG00000022631	ubiquitin specific peptidase 21 [Source:VGNC Symbol;Acc:VGNC:78741]	-1.47629625	0.002033737	0.011758157
ENSMMUG00000011335	Fc receptor like 6 [Source:VGNC Symbol;Acc:VGNC:72602]	-0.839069336	0.003102865	0.016411495
ENSMMUG00000000730	spectrin alpha, erythrocytic 1 [Source:VGNC Symbol;Acc:VGNC:77868]	1.121942309	0.000723879	0.005079495
ENSMMUG00000003191	kirre like nephrin family adhesion molecule 1 [Source:VGNC Symbol;Acc:VGNC:73837]	-0.991530078	0.000455751	0.00344422
ENSMMUG00000019833	Fc receptor like 1 [Source:VGNC Symbol;Acc:VGNC:72528]	1.112666735	1.25E-05	0.000175696
ENSMMUG00000019830	Fc receptor like 2 [Source:VGNC Symbol;Acc:VGNC:72509]	0.949290048	0.007097457	0.031494211
ENSMMUG00000046969	Fc receptor like 3 [Source:NCBI gene;Acc:718801]	0.853108904	0.001930417	0.011284049
ENSMMUG00000042886	ETS variant transcription factor 3 [Source:NCBI gene;Acc:719741]	1.046121444	0.001847693	0.010892924
ENSMMUG00000064007	leucine rich repeat containing 71 [Source:NCBI gene;Acc:718714]	0.578328641	0.00728499	0.032161773
ENSMMUG00000055304	insulin receptor related receptor [Source:NCBI gene;Acc:718670]	-1.174640515	0.011434571	0.045647033
ENSMMUG00000000037	interferon stimulated exonuclease gene 20 like 2 [Source:NCBI gene;Acc:718621]	-2.114654527	0.000530711	0.003928528
ENSMMUG00000027037		2.063343507	4.58E-05	0.000519278
ENSMMUG00000017966	chromosome 1 C1orf61 homolog [Source:NCBI gene;Acc:106992893]	1.696824401	3.87E-06	6.56E-05
ENSMMUG00000016096	lamin A/C [Source:VGNC Symbol;Acc:VGNC:74427]	1.683348905	0.001828541	0.01081492
ENSMMUG00000004648	KH domain containing 4, pre-mRNA splicing factor [Source:VGNC Symbol;Acc:VGNC:73931]	-0.674010655	0.001508853	0.009287335
ENSMMUG00000062397		1.343505729	8.38E-06	0.000124139
ENSMMUG00000023145	RUN and SH3 domain containing 1 [Source:VGNC Symbol;Acc:VGNC:77036]	-1.240484846	0.003326445	0.017326289
ENSMMUG00000053423		4.022579987	2.38E-08	9.53E-07
ENSMMUG00000008506	metaxin-1 [Source:NCBI gene;Acc:717595]	2.692325071	0.010199825	0.041963764
ENSMMUG00000003902	thrombospondin 3 [Source:VGNC Symbol;Acc:VGNC:78345]	1.577654029	9.24E-10	6.61E-08
ENSMMUG00000010978	S100 calcium binding protein A2 [Source:VGNC Symbol;Acc:VGNC:76956]	-0.792722523	0.00019397	0.001714787
ENSMMUG00000008917	S100 calcium binding protein A6 [Source:VGNC Symbol;Acc:VGNC:76960]	-0.589661268	0.011766773	0.046715095
ENSMMUG00000051645		3.791381218	3.57E-08	1.29E-06
ENSMMUG00000061474		2.602612775	5.41E-10	4.32E-08
ENSMMUG00000063702		2.62124742	0.01141682	0.045604163
ENSMMUG00000042618	involucrin [Source:NCBI gene;Acc:713728]	2.397378783	2.07E-08	8.48E-07
ENSMMUG00000017740	sperm mitochondria associated cysteine rich protein [Source:VGNC Symbol;Acc:VGNC:77816]	3.099214299	3.62E-10	3.07E-08
ENSMMUG00000055038		2.798343939	1.48E-08	6.41E-07
ENSMMUG00000054634	late cornified envelope protein 1C-like [Source:NCBI gene;Acc:713600]	3.178888919	0.000659639	0.004700622
ENSMMUG00000051719	late cornified envelope 1C [Source:HGNC Symbol;Acc:HGNC:29464]	2.713058371	4.58E-05	0.00051945
ENSMMUG00000058472	late cornified envelope protein 1C [Source:NCBI gene;Acc:107000798]	2.466744157	4.96E-09	2.55E-07
ENSMMUG00000056940	late cornified envelope protein 1C [Source:NCBI gene;Acc:713408]	2.816921597	0.000390666	0.003044338
ENSMMUG00000055266	late cornified envelope protein 1C-like [Source:NCBI gene;Acc:107000794]	3.031271358	2.69E-07	7.06E-06
ENSMMUG00000051816	keratinocyte proline rich protein [Source:NCBI gene;Acc:100424312]	2.612809201	2.74E-09	1.58E-07
ENSMMUG00000049843	late cornified envelope protein 2D [Source:NCBI gene;Acc:100424118]	3.911836333	0.000213553	0.001859082
ENSMMUG00000062996		2.695712663	7.49E-06	0.000113209

ENSMMUG00000001967	late cornified envelope 5A [Source:VGNC Symbol;Acc:VGNC:74061]	3.839915284	3.64E-05	0.000430568
ENSMMUG00000009040	cornulin [Source:VGNC Symbol;Acc:VGNC:71397]	2.440924407	1.22E-05	0.00017232
ENSMMUG00000056511		2.545918664	3.11E-09	1.73E-07
ENSMMUG00000059472	filaggrin family member 2 [Source:HGNC Symbol;Acc:HGNC:33276]	2.048348709	4.33E-13	9.91E-11
ENSMMUG00000010067	MINDY lysine 48 deubiquitinase 1 [Source:VGNC Symbol;Acc:VGNC:74828]	-2.618185966	0.01230201	0.048250886
ENSMMUG00000021385	carbonic anhydrase 14 [Source:VGNC Symbol;Acc:VGNC:70489]	-0.654267391	0.00400396	0.020030915
ENSMMUG00000052282		-0.931641722	0.001835264	0.010839892
ENSMMUG00000007256	splicing factor 3b subunit 4 [Source:VGNC Symbol;Acc:VGNC:77182]	-0.734011253	0.012239139	0.048091312
ENSMMUG00000007257	synaptic vesicle glycoprotein 2A [Source:VGNC Symbol;Acc:VGNC:78181]	-0.671029078	0.00973447	0.040436449
ENSMMUG00000052382	histone H4 [Source:NCBI gene;Acc:107000818]	-0.666323515	0.002678844	0.014535271
ENSMMUG00000062140		-1.364494858	0.000156806	0.001435574
ENSMMUG00000024186	U1 spliceosomal RNA [Source:RFAM;Acc:RF00003]	-3.007757785	9.85E-06	0.000142145
ENSMMUG00000061239		-1.11927092	2.58E-05	0.000323581
ENSMMUG00000054590		-1.175862802	0.000103745	0.001023751
ENSMMUG00000056076	U1 spliceosomal RNA [Source:RFAM;Acc:RF00003]	-1.678621927	0.000443712	0.003372831
ENSMMUG00000056325	NBPF member 20 [Source:NCBI gene;Acc:708768]	-2.168782287	3.96E-06	6.66E-05
ENSMMUG00000055722		-1.745420663	0.000265628	0.002210337
ENSMMUG00000044046		-1.606406316	0.000167725	0.001513836
ENSMMUG00000062170		3.725130126	0.012416562	0.048584802
ENSMMUG00000041725	60S ribosomal protein L6-like [Source:NCBI gene;Acc:710287]	-2.892840797	0.008201877	0.035287334
ENSMMUG00000009781	synaptotagmin 6 [Source:VGNC Symbol;Acc:VGNC:78162]	0.974413423	0.002358009	0.013095663
ENSMMUG00000019528	adaptor related protein complex 4 subunit beta 1 [Source:VGNC Symbol;Acc:VGNC:69972]	2.362431216	2.34E-10	2.11E-08
ENSMMUG00000019527	DNA cross-link repair 1B [Source:VGNC Symbol;Acc:VGNC:71647]	1.321001234	0.004040233	0.020171697
ENSMMUG00000006787	BCL2 like 15 [Source:VGNC Symbol;Acc:VGNC:70162]	2.467345547	0.012153241	0.04781157
ENSMMUG00000006786	protein tyrosine phosphatase non-receptor type 22 [Source:VGNC Symbol;Acc:VGNC:76603]	1.377393994	7.83E-07	1.75E-05
ENSMMUG00000017819	putative homeodomain transcription factor 1 [Source:VGNC Symbol;Acc:VGNC:75982]	-1.275597123	3.78E-06	6.47E-05
ENSMMUG00000037458	endoplasmic reticulum resident protein 29 [Source:NCBI gene;Acc:720396]	-1.971601484	0.004812336	0.02315547
ENSMMUG00000014489	DEAD-box helicase 20 [Source:NCBI gene;Acc:704731]	1.002688714	0.000149277	0.001381844
ENSMMUG00000021544	solute carrier family 6 member 17 [Source:VGNC Symbol;Acc:VGNC:77805]	2.781085918	6.08E-06	9.54E-05
ENSMMUG00000051186	proteasome 20S subunit alpha 5 [Source:NCBI gene;Acc:698435]	0.977774423	1.27E-05	0.000177989
ENSMMUG00000017266	UPF0577 protein KIAA1324 [Source:NCBI gene;Acc:697670]	1.573011556	9.38E-07	2.03E-05
ENSMMUG00000042232	NBPF member 6 [Source:NCBI gene;Acc:698524]	1.301057469	0.000488729	0.003644733
ENSMMUG00000031785		1.008235364	0.001850303	0.010899025

ENSMMUG00000002789	solute carrier family 25 (mitochondrial carrier; phosphate carrier), member 24 [Source:NCBI gene;Acc:694803]	2.541558072	2.64E-05	0.000330031
ENSMMUG00000008197	netrin G1 [Source:VGNC Symbol;Acc:VGNC:75450]	2.06784088	0.001777539	0.010556304
ENSMMUG00000004103	collagen type XI alpha 1 chain [Source:VGNC Symbol;Acc:VGNC:71289]	1.497177876	1.36E-09	8.95E-08
ENSMMUG00000060790		-4.127266216	0.000821514	0.00559404
ENSMMUG00000022906	vascular cell adhesion molecule 1 [Source:VGNC Symbol;Acc:VGNC:78761]	-0.579525595	0.005549526	0.025897788
ENSMMUG00000055443	G protein-coupled receptor 88 [Source:NCBI gene;Acc:716520]	0.61916109	0.010430788	0.042540867
ENSMMUG00000019105	RNA 3'-terminal phosphate cyclase [Source:VGNC Symbol;Acc:VGNC:77043]	-2.577312722	1.44E-05	0.000198126
ENSMMUG00000007706	dihydrolipoamide branched chain transacylase E2 [Source:VGNC Symbol;Acc:VGNC:71744]	-3.658151236	0.00027395	0.002266538
ENSMMUG00000000931	solute carrier family 35 member A3 [Source:VGNC Symbol;Acc:VGNC:77461]	1.801025929	0.000286451	0.002352006
ENSMMUG00000038667		-1.968060536	0.002442246	0.013493565
ENSMMUG00000008518	phospholipid phosphatase related 5 [Source:NCBI gene;Acc:710549]	1.391192133	2.87E-10	2.52E-08
ENSMMUG00000008515	sorting nexin 7 [Source:NCBI gene;Acc:716015]	0.846197531	0.00322264	0.016907486
ENSMMUG00000020726	dihydropyrimidine dehydrogenase [Source:VGNC Symbol;Acc:VGNC:71982]	2.079200153	2.04E-08	8.44E-07
ENSMMUG00000049297		0.905485216	1.50E-05	0.000204082
ENSMMUG00000001690	calponin 3 [Source:VGNC Symbol;Acc:VGNC:71278]	0.745252046	0.0034703	0.017882217
ENSMMUG00000058244		-2.435202975	0.007553213	0.033121069
ENSMMUG00000045959		1.355138771	0.000280434	0.002311232
ENSMMUG00000015924	BCAR3 adaptor protein, NSP family member [Source:VGNC Symbol;Acc:VGNC:70235]	0.758303979	0.00553201	0.025843864
ENSMMUG00000015922	formin binding protein 1 like [Source:VGNC Symbol;Acc:VGNC:72543]	0.637559523	0.000341599	0.002725663
ENSMMUG00000016812	coiled-coil domain containing 18 [Source:VGNC Symbol;Acc:VGNC:70913]	-1.068624282	0.00057556	0.004200745
ENSMMUG00000051332		1.690160594	0.010797844	0.043600359
ENSMMUG00000005070	ecotropic viral integration site 5 [Source:NCBI gene;Acc:706592]	1.716381422	4.12E-10	3.48E-08
ENSMMUG00000014018	bromodomain testis associated [Source:VGNC Symbol;Acc:VGNC:70282]	-3.645352915	1.02E-09	7.27E-08
ENSMMUG00000001742	transforming growth factor beta receptor 3 [Source:VGNC Symbol;Acc:VGNC:78339]	-4.499358782	5.21E-39	6.77E-35
ENSMMUG00000051155	U2 spliceosomal RNA [Source:RFAM;Acc:RF00004]	-4.626327006	0.00035252	0.002802488
ENSMMUG00000006819	helicase for meiosis 1 [Source:NCBI gene;Acc:704516]	1.327215001	3.43E-05	0.000411277
ENSMMUG00000049678		1.521428503	0.000273011	0.002260205
ENSMMUG00000060600		-1.625140689	0.000271043	0.002246776
ENSMMUG00000017707	leucine rich repeat containing 8 VRAC subunit B [Source:VGNC Symbol;Acc:VGNC:81439]	1.75047913	1.87E-06	3.58E-05
ENSMMUG00000002794	general transcription factor IIB [Source:VGNC Symbol;Acc:VGNC:73306]	-1.921082378	8.03E-06	0.000120209
ENSMMUG00000020588	protein kinase N2 [Source:VGNC Symbol;Acc:VGNC:76015]	0.804354578	5.63E-06	9.03E-05
ENSMMUG00000065295		2.107901534	4.20E-10	3.52E-08
ENSMMUG00000000813	calcium-activated chloride channel regulator 1-like [Source:NCBI gene;Acc:715292]	0.965989515	0.004221139	0.02081101
ENSMMUG00000006836	chloride channel accessory 1 [Source:NCBI gene;Acc:574295]	2.320963019	8.11E-07	1.80E-05

ENSMMUG00000001380	outer dense fiber of sperm tails 2 like [Source:VGNC Symbol;Acc:VGNC:75482]	2.783446208	0.000104249	0.001027945
ENSMMUG00000014573	dimethylarginine dimethylaminohydrolase 1 [Source:VGNC Symbol;Acc:VGNC:71753]	1.686935583	9.63E-09	4.49E-07
ENSMMUG00000005720	BCL10 immune signaling adaptor [Source:VGNC Symbol;Acc:VGNC:70240]	0.612542767	0.000836508	0.005676943
ENSMMUG00000005361	mucolipin 3 [Source:VGNC Symbol;Acc:VGNC:74505]	1.347951122	5.93E-05	0.000639381
ENSMMUG00000063379		-0.797740643	0.003272916	0.017102269
ENSMMUG00000010127	chitobiase [Source:VGNC Symbol;Acc:VGNC:71540]	-2.164183162	2.62E-06	4.80E-05
ENSMMUG00000022183	spermatogenesis associated 1 [Source:NCBI gene;Acc:710462]	-1.260345711	3.59E-07	9.11E-06
ENSMMUG00000059938		-1.619160783	0.000461625	0.003480516
ENSMMUG00000003457	protein kinase cAMP-activated catalytic subunit beta [Source:NCBI gene;Acc:709728]	1.575269175	1.38E-06	2.78E-05
ENSMMUG00000016591	tubulin tyrosine ligase like 7 [Source:VGNC Symbol;Acc:VGNC:79591]	0.621561349	0.010366868	0.04238651
ENSMMUG00000057763		-1.56846433	0.000115076	0.001116928
ENSMMUG00000050832		2.880952959	0.010099712	0.041647701
ENSMMUG00000023345	adhesion G protein-coupled receptor L4 [Source:VGNC Symbol;Acc:VGNC:69652]	2.138940509	2.53E-05	0.000318566
ENSMMUG00000054277		0.62152942	0.007158414	0.031697707
ENSMMUG00000020217	phosphatidylinositol glycan anchor biosynthesis class K [Source:VGNC Symbol;Acc:VGNC:75988]	-0.644392227	0.002450556	0.013527984
ENSMMUG00000041837	Metazoan signal recognition particle RNA [Source:RFAM;Acc:RF00017]	-1.823291142	0.002317974	0.012955553
ENSMMUG00000056103	growth arrest and DNA damage inducible alpha [Source:NCBI gene;Acc:701054]	-1.126757328	8.79E-08	2.70E-06
ENSMMUG00000054553		3.18524155	0.004269194	0.02099249
ENSMMUG00000053254	adenylate kinase 4 [Source:NCBI gene;Acc:698560]	1.390268769	0.000791319	0.00543969
ENSMMUG00000053072		1.565096276	0.000187945	0.001669678
ENSMMUG00000018566	Janus kinase 1 [Source:VGNC Symbol;Acc:VGNC:73810]	3.004053659	1.72E-09	1.10E-07
ENSMMUG00000018565	ribonucleoprotein, PTB binding 2 [Source:VGNC Symbol;Acc:VGNC:76536]	-2.989064905	2.37E-05	0.000300922
ENSMMUG00000012538	ubiquitin conjugating enzyme E2 U [Source:VGNC Symbol;Acc:VGNC:79726]	0.814826357	0.008611825	0.036711044
ENSMMUG00000020049	phosphoglucomutase 1 [Source:NCBI gene;Acc:699401]	-0.824319854	8.79E-05	0.000899233
ENSMMUG00000010351	KN motif and ankyrin repeat domains 4 [Source:VGNC Symbol;Acc:VGNC:73916]	-0.821744136	0.000404695	0.003131153
ENSMMUG00000049952		0.832156034	0.000471219	0.003530345
ENSMMUG00000055834		1.887625132	0.000900588	0.006056387
ENSMMUG00000022762	OMA1 zinc metallopeptidase [Source:VGNC Symbol;Acc:VGNC:75485]	-1.019219957	0.001951919	0.011384151
ENSMMUG00000059612		0.268430888	0.006812519	0.030447981
ENSMMUG00000032084	prolyl-tRNA synthetase 2, mitochondrial [Source:VGNC Symbol;Acc:VGNC:75707]	-0.597138791	0.005299788	0.024983341
ENSMMUG00000006754	tetratricopeptide repeat domain 4 [Source:VGNC Symbol;Acc:VGNC:78668]	-0.940972824	7.70E-05	0.00080399
ENSMMUG00000009114	maestro heat like repeat family member 7 [Source:NCBI gene;Acc:715310]	1.672890421	5.98E-09	3.02E-07
ENSMMUG00000008828	BEN domain containing 5 [Source:NCBI gene;Acc:711150]	1.715546212	2.19E-06	4.09E-05
ENSMMUG00000005986	Kruppel like factor 17 [Source:NCBI gene;Acc:702810]	1.448688132	0.007125064	0.031595153

ENSMMUG00000015555	ST3 beta-galactoside alpha-2,3-sialyltransferase 3 [Source:VGNC Symbol;Acc:VGNC:77828]	1.84164437	0.000435467	0.003323756
ENSMMUG00000053498		2.352209833	1.06E-08	4.78E-07
ENSMMUG00000012477	protein tyrosine phosphatase receptor type F [Source:VGNC Symbol;Acc:VGNC:76615]	-0.337547629	0.010124898	0.041738328
ENSMMUG00000057076	claudin 19 [Source:NCBI gene;Acc:697807]	4.150949072	0.007450388	0.032791793
ENSMMUG00000014138	forkhead box J3 [Source:VGNC Symbol;Acc:VGNC:72596]	2.150062919	0.000134335	0.001273445
ENSMMUG00000043675	Cbp/p300 interacting transactivator with Glu/Asp rich carboxy-terminal domain 4 [Source:NCBI gene;Acc:114672840]	1.41851487	6.06E-08	1.99E-06
ENSMMUG00000019536	potassium voltage-gated channel subfamily Q member 4 [Source:VGNC Symbol;Acc:VGNC:74007]	0.646670997	0.007636318	0.033393226
ENSMMUG00000018664	collagen type IX alpha 2 chain [Source:VGNC Symbol;Acc:VGNC:71307]	1.681619186	3.34E-12	6.21E-10
ENSMMUG00000005992	zinc metallopeptidase STE24 [Source:VGNC Symbol;Acc:VGNC:78830]	1.676532912	0.000174608	0.001565094
ENSMMUG00000032180	RLF zinc finger [Source:VGNC Symbol;Acc:VGNC:76699]	1.407265825	0.008557276	0.036538388
ENSMMUG00000009562	microtubule actin crosslinking factor 1 [Source:NCBI gene;Acc:718651]	2.294471996	2.71E-05	0.000336753
ENSMMUG00000008419	G protein nucleolar 2 [Source:VGNC Symbol;Acc:VGNC:73037]	-1.544848494	0.000845609	0.005731106
ENSMMUG00000059855	Smad nuclear interacting protein 1 [Source:NCBI gene;Acc:713733]	-1.826483371	5.37E-07	1.27E-05
ENSMMUG00000010328	MYST/Esa1 associated factor 6 [Source:VGNC Symbol;Acc:VGNC:74580]	-1.453783233	0.003348854	0.01740021
ENSMMUG00000006252	argonaute RISC catalytic component 3 [Source:NCBI gene;Acc:712543]	-2.452725159	1.54E-17	1.05E-14
ENSMMUG00000006251	argonaute RISC component 1 [Source:VGNC Symbol;Acc:VGNC:81346]	-1.819978632	1.07E-09	7.52E-08
ENSMMUG00000019815	transcription factor AP-2 epsilon [Source:VGNC Symbol;Acc:VGNC:79878]	-1.987793997	5.58E-05	0.000610287
ENSMMUG00000003267	zinc finger protein 362 [Source:NCBI gene;Acc:710013]	0.755876364	0.000471027	0.003530345
ENSMMUG00000015097	tripartite motif containing 62 [Source:VGNC Symbol;Acc:VGNC:79513]	0.86244367	0.000254165	0.002132689
ENSMMUG00000006285	antizyme inhibitor 2 [Source:VGNC Symbol;Acc:VGNC:70153]	1.048746839	2.40E-08	9.53E-07
ENSMMUG00000006284	adenylate kinase 2 [Source:VGNC Symbol;Acc:VGNC:69761]	0.90686732	0.000131271	0.001248945
ENSMMUG00000044265	Metazoan signal recognition particle RNA [Source:RFAM;Acc:RF00017]	0.928453943	9.19E-05	0.000932724
ENSMMUG00000001264	BSD domain containing 1 [Source:VGNC Symbol;Acc:VGNC:70384]	-5.195487056	5.22E-06	8.47E-05
ENSMMUG00000053323		-3.978733537	0.002027861	0.011737587
ENSMMUG00000009951	MARCKS like 1 [Source:VGNC Symbol;Acc:VGNC:74723]	-3.501886092	0.008633453	0.036779133
ENSMMUG00000009948	histone deacetylase 1 [Source:NCBI gene;Acc:708441]	-2.188843146	1.39E-10	1.40E-08
ENSMMUG00000040694	LCK proto-oncogene, Src family tyrosine kinase [Source:NCBI gene;Acc:717704]	-1.69198772	7.56E-08	2.41E-06
ENSMMUG00000015650	transmembrane protein 39B [Source:VGNC Symbol;Acc:VGNC:79507]	-0.871639363	0.000651844	0.00465562
ENSMMUG00000041790	Metazoan signal recognition particle RNA [Source:RFAM;Acc:RF00017]	1.474883692	0.000275453	0.002277526
ENSMMUG00000023401	adhesion G protein-coupled receptor B2 [Source:VGNC Symbol;Acc:VGNC:69598]	-0.97802223	2.76E-05	0.000343424
ENSMMUG00000023398	collagen type XVI alpha 1 chain [Source:VGNC Symbol;Acc:VGNC:71293]	-0.786984546	0.001574695	0.009628813

ENSMMUG00000023397	penta-EF-hand domain containing 1 [Source:NCBI gene;Acc:706038]	-0.541613677	0.010689901	0.043245054
ENSMMUG00000013188	serine incorporator 2 [Source:VGNC Symbol;Acc:VGNC:77294]	-0.701123498	0.003672097	0.018721791
ENSMMUG00000049574	antizyme inhibitor 2-like [Source:NCBI gene;Acc:705256]	-0.857777667	5.21E-05	0.000578009
ENSMMUG00000063979		-0.789632786	1.81E-06	3.48E-05
ENSMMUG00000057452		1.384992188	2.08E-05	0.000270201
ENSMMUG00000015661	protein tyrosine phosphatase receptor type U [Source:VGNC Symbol;Acc:VGNC:76624]	-1.512517611	0.002058261	0.011881823
ENSMMUG00000007110	serine and arginine rich splicing factor 4 [Source:NCBI gene;Acc:717401]	-4.106338512	0.000126445	0.001210109
ENSMMUG00000016893	solute carrier family 9 member A1 [Source:VGNC Symbol;Acc:VGNC:82241]	-1.349052842	3.05E-08	1.15E-06
ENSMMUG00000021031	TMF1 regulated nuclear protein 1 [Source:VGNC Symbol;Acc:VGNC:79946]	0.780641499	0.000136178	0.001287158
ENSMMUG00000058432	zinc finger DHHC-type palmitoyltransferase 18 [Source:NCBI gene;Acc:719959]	1.783811646	7.64E-06	0.000115315
ENSMMUG00000000649	UBX domain protein 11 [Source:VGNC Symbol;Acc:VGNC:79859]	1.264218983	0.002219115	0.012543162
ENSMMUG00000000644	centrosomal protein 85 [Source:VGNC Symbol;Acc:VGNC:71096]	1.49451369	7.76E-05	0.000808257
ENSMMUG00000057953		1.514697927	6.80E-05	0.000721075
ENSMMUG00000057675	non-compact myelin associated protein [Source:NCBI gene;Acc:712192]	1.459832463	0.002118305	0.012126175
ENSMMUG00000053036		-1.00675714	0.007899271	0.034303147
ENSMMUG00000049609		-2.773839951	1.49E-05	0.000203164
ENSMMUG00000022927	RCAN family member 3 [Source:VGNC Symbol;Acc:VGNC:76875]	-1.166813895	2.94E-06	5.25E-05
ENSMMUG00000050770		-0.954416509	0.000441031	0.003356375
ENSMMUG00000059931		-0.698402973	0.004940827	0.023627373
ENSMMUG00000009773	lysine demethylase 1A [Source:VGNC Symbol;Acc:VGNC:73928]	-1.078498564	0.000262139	0.002182703
ENSMMUG00000041482	lactamase beta like 1 [Source:NCBI gene;Acc:718590]	-0.953866083	0.004944354	0.023627783
ENSMMUG00000002217	alkaline phosphatase, biomineralization associated [Source:VGNC Symbol;Acc:VGNC:69801]	2.008322993	3.02E-09	1.69E-07
ENSMMUG00000004149	eukaryotic translation initiation factor 4 gamma 3 [Source:NCBI gene;Acc:717555]	1.834152143	2.41E-09	1.44E-07
ENSMMUG00000002492	transmembrane and coiled-coil domains 4 [Source:VGNC Symbol;Acc:VGNC:79664]	4.105634633	1.71E-05	0.000228021
ENSMMUG00000050018		1.903556752	1.08E-06	2.26E-05
ENSMMUG00000004754	intermediate filament family orphan 2 [Source:NCBI gene;Acc:106995162]	-1.104272826	0.000132538	0.001260079
ENSMMUG00000032238	peptidyl arginine deiminase 4 [Source:NCBI gene;Acc:700806]	2.16377411	0.001968027	0.011446075
ENSMMUG00000051619	peptidyl arginine deiminase 3 [Source:NCBI gene;Acc:700560]	1.287867967	3.81E-06	6.51E-05
ENSMMUG00000007315	ciliary rootlet coiled-coil, rootletin [Source:NCBI gene;Acc:710793]	0.865588609	9.89E-05	0.000988693
ENSMMUG00000018245	EPH receptor A2 [Source:VGNC Symbol;Acc:VGNC:72235]	1.494456524	1.22E-13	3.23E-11
ENSMMUG00000058587	family with sequence similarity 131 member C [Source:NCBI gene;Acc:703604]	1.441039385	7.51E-09	3.62E-07
ENSMMUG00000018235	chloride voltage-gated channel Kb [Source:NCBI gene;Acc:703476]	1.606208316	2.08E-06	3.91E-05
ENSMMUG00000001910	heat shock protein family B (small) member 7 [Source:NCBI gene;Acc:696704]	1.701264433	0.000405876	0.003135329

ENSMMUG00000001911	steroid receptor associated and regulated protein [Source:VGNC Symbol;Acc:VGNC:78008]	1.455433132	0.000188924	0.001674942
ENSMMUG00000008031	zinc finger and BTB domain containing 17 [Source:VGNC Symbol;Acc:VGNC:78849]	1.332725594	1.41E-07	4.07E-06
ENSMMUG00000002802	agmatinase [Source:NCBI gene;Acc:713190]	-1.257068039	0.007043656	0.031298185
ENSMMUG00000032244	DnaJ heat shock protein family (Hsp40) member C16 [Source:NCBI gene;Acc:700894]	-1.486957214	0.003644432	0.018639198
ENSMMUG00000018163	caspase 9 [Source:NCBI gene;Acc:694544]	-2.526489814	0.010634238	0.04310031
ENSMMUG00000031821	chymotrypsin-like elastase family member 2B [Source:NCBI gene;Acc:106992406]	-2.18357738	0.000821213	0.00559404
ENSMMUG00000060541		-2.944862212	0.000691378	0.004884338
ENSMMUG00000003987	forkhead associated phosphopeptide binding domain 1 [Source:NCBI gene;Acc:699900]	-2.915578903	1.79E-07	5.03E-06
ENSMMUG00000055233	PRAME family member 17-like [Source:NCBI gene;Acc:100427847]	-0.972554622	2.45E-05	0.000309736
ENSMMUG00000054309	PRAME family member 5 [Source:NCBI gene;Acc:722781]	-1.885393747	0.001317951	0.008284812
ENSMMUG00000021449	vacuolar protein sorting 13 homolog D [Source:NCBI gene;Acc:722775]	1.089571293	3.00E-09	1.69E-07
ENSMMUG00000064242		0.921638993	0.003232699	0.016939761
ENSMMUG00000063313	Small nucleolar RNA U3 [Source:RFAM;Acc:RF00012]	1.010596641	0.007352512	0.032426849
ENSMMUG00000021447	TNF receptor superfamily member 1B [Source:VGNC Symbol;Acc:VGNC:78590]	5.083476179	0.000127008	0.001212828
ENSMMUG00000032249	procollagen-lysine,2-oxoglutarate 5-dioxygenase 1 [Source:NCBI gene;Acc:722763]	0.844196253	0.00088289	0.005955847
ENSMMUG00000012469	KIAA2013 [Source:VGNC Symbol;Acc:VGNC:73918]	0.472332619	0.009771275	0.040550478
ENSMMUG00000002261	F-box protein 2 [Source:VGNC Symbol;Acc:VGNC:72486]	-2.726422798	0.002198334	0.012447334
ENSMMUG00000049196	dispatched RND transporter family member 3 [Source:NCBI gene;Acc:722727]	-0.732250818	0.000631322	0.004546498
ENSMMUG00000032252	UbiA prenyltransferase domain containing 1 [Source:VGNC Symbol;Acc:VGNC:79881]	-1.225168199	5.07E-10	4.09E-08
ENSMMUG00000019325	mechanistic target of rapamycin kinase [Source:VGNC Symbol;Acc:VGNC:75062]	-1.324618851	4.75E-06	7.81E-05
ENSMMUG00000053062	U6 spliceosomal RNA [Source:RFAM;Acc:RF00026]	-2.083678031	0.008794179	0.037292826
ENSMMUG00000006187	angiopoietin like 7 [Source:VGNC Symbol;Acc:VGNC:69683]	-1.202637895	6.69E-05	0.000713438
ENSMMUG00000032281	zinc finger and BTB domain containing 48 [Source:VGNC Symbol;Acc:VGNC:79959]	-0.826017532	0.005111554	0.024316339
ENSMMUG00000023677	TNF receptor superfamily member 25 [Source:VGNC Symbol;Acc:VGNC:78584]	-3.550798385	0.004139298	0.02053231
ENSMMUG00000023676	espin [Source:NCBI gene;Acc:722532]	-0.912220381	0.005124228	0.024349914
ENSMMUG00000055126		1.477319379	0.006174703	0.028158551
ENSMMUG00000054719		-2.190122041	9.78E-09	4.54E-07
ENSMMUG00000050239		-1.238441171	0.000162457	0.001473691
ENSMMUG00000018911	protein kinase C zeta [Source:VGNC Symbol;Acc:VGNC:82212]	-2.003789264	0.002335201	0.01300155
ENSMMUG00000005578	solute carrier family 35 member E2 [Source:VGNC Symbol;Acc:VGNC:77590]	-0.995337689	0.007598414	0.033252009

ENSMMUG00000012961	mindbomb E3 ubiquitin protein ligase 2 [Source:VGNC Symbol;Acc:VGNC:74627]	-0.71492589	0.006116816	0.027953377
ENSMMUG00000048805		-1.442356612	3.42E-06	5.98E-05
ENSMMUG00000040063	fibronectin type III domain containing 10 [Source:NCBI gene;Acc:106996973]	-1.511366323	0.004617472	0.022408522
ENSMMUG00000060167		-1.105594111	0.000216764	0.001885773
ENSMMUG00000013862	ArfGAP with coiled-coil, ankyrin repeat and PH domains 3 [Source:VGNC Symbol;Acc:VGNC:69434]	-0.721127154	0.007160377	0.031697707
ENSMMUG00000006596	CCR4-NOT transcription complex subunit 10 [Source:VGNC Symbol;Acc:VGNC:71327]	1.341909107	0.002408804	0.013337124
ENSMMUG00000049551		1.306936737	0.00222606	0.012566029
ENSMMUG00000055585		-1.27023554	0.000375864	0.002948423
ENSMMUG00000051896		-3.438570316	0.006976888	0.03108647
ENSMMUG00000060958	leucine rich repeat containing 3B [Source:NCBI gene;Acc:701323]	-2.287857071	0.002061206	0.011893545
ENSMMUG00000051975		1.090873861	0.001578722	0.009639837
ENSMMUG00000019009	SATB homeobox 1 [Source:VGNC Symbol;Acc:VGNC:84086]	0.745234537	0.008219518	0.035328171
ENSMMUG00000020377	collagen type VI alpha 5 chain [Source:NCBI gene;Acc:719331]	-1.085691967	0.003416961	0.017663355
ENSMMUG00000005137	ATPase secretory pathway Ca2+ transporting 1 [Source:VGNC Symbol;Acc:VGNC:70054]	-3.806647573	5.83E-14	1.69E-11
ENSMMUG00000000220	mitochondrial ribosomal protein L3 [Source:VGNC Symbol;Acc:VGNC:74725]	-1.368346551	0.006933795	0.030915644
ENSMMUG00000003277	copine 4 [Source:VGNC Symbol;Acc:VGNC:71466]	-1.877257048	7.16E-10	5.48E-08
ENSMMUG00000054158	acyl-CoA dehydrogenase family member 11 [Source:NCBI gene;Acc:106997118]	2.327051542	4.17E-07	1.03E-05
ENSMMUG00000019854	transmembrane protein 108 [Source:VGNC Symbol;Acc:VGNC:79714]	-1.363012099	0.000148868	0.001380025
ENSMMUG00000019857	beaded filament structural protein 2 [Source:VGNC Symbol;Acc:VGNC:70245]	-1.75237996	3.86E-09	2.08E-07
ENSMMUG00000057769		-2.451235578	0.002034819	0.011758157
ENSMMUG00000000058	CDV3 homolog [Source:VGNC Symbol;Acc:VGNC:71114]	-2.126842012	2.02E-05	0.000263849
ENSMMUG00000000059	DNA topoisomerase II binding protein 1 [Source:VGNC Symbol;Acc:VGNC:79061]	-2.32108091	4.63E-22	8.60E-19
ENSMMUG00000053660	Metazoan signal recognition particle RNA [Source:RFAM;Acc:RF00017]	-2.213120794	5.76E-06	9.16E-05
ENSMMUG00000008916	transferrin [Source:VGNC Symbol;Acc:VGNC:82716]	-2.19021042	2.06E-14	7.07E-12
ENSMMUG00000008918	SRP receptor subunit beta [Source:VGNC Symbol;Acc:VGNC:77980]	-1.850996459	1.56E-07	4.46E-06
ENSMMUG00000008919	RAB6B, member RAS oncogene family [Source:VGNC Symbol;Acc:VGNC:81547]	-1.590536012	1.18E-05	0.000168398
ENSMMUG00000012605	solute carrier organic anion transporter family member 2A1 [Source:VGNC Symbol;Acc:VGNC:77600]	-1.088132157	1.36E-08	5.94E-07
ENSMMUG00000003263	stromal antigen 1 [Source:VGNC Symbol;Acc:VGNC:77901]	0.50710821	0.008126474	0.035063566
ENSMMUG00000007760	centrosomal protein 70 [Source:VGNC Symbol;Acc:VGNC:71140]	0.681697355	0.000539602	0.003983013
ENSMMUG00000053063	forkhead box L2 [Source:NCBI gene;Acc:717906]	0.818874638	0.001168846	0.007522026
ENSMMUG00000056024		1.930164906	6.72E-09	3.29E-07
ENSMMUG00000063351	Metazoan signal recognition particle RNA [Source:RFAM;Acc:RF00017]	1.593012906	0.008805172	0.037327273

ENSMMUG00000007387	G protein-coupled receptor kinase 7 [Source:VGNC Symbol;Acc:VGNC:73182]	1.861334096	1.07E-09	7.52E-08
ENSMMUG00000052474		1.845523228	5.90E-06	9.35E-05
ENSMMUG00000007927	ATR serine/threonine kinase [Source:VGNC Symbol;Acc:VGNC:70193]	2.089707758	0.001486502	0.009162771
ENSMMUG00000012405	transient receptor potential cation channel subfamily C member 1 [Source:VGNC Symbol;Acc:VGNC:78651]	2.332870401	0.000682496	0.004832085
ENSMMUG00000002775	U2 snRNP associated SURP domain containing [Source:VGNC Symbol;Acc:VGNC:79889]	0.891344675	0.000977856	0.006474706
ENSMMUG00000009694	solute carrier family 9 member A9 [Source:VGNC Symbol;Acc:VGNC:77747]	-1.563066057	0.012791031	0.049644925
ENSMMUG00000039078	phospholipid scramblase family member 5 [Source:VGNC Symbol;Acc:VGNC:84061]	2.150430665	0.000191514	0.001695597
ENSMMUG00000047621	transmembrane 4 L six family member 18 [Source:VGNC Symbol;Acc:VGNC:82713]	0.821640623	0.000147151	0.001368012
ENSMMUG00000031534	ankyrin repeat and ubiquitin domain containing 1 [Source:VGNC Symbol;Acc:VGNC:84239]	1.496954405	6.98E-07	1.60E-05
ENSMMUG00000035943	U6 spliceosomal RNA [Source:RFAM;Acc:RF00026]	2.231516629	5.30E-07	1.26E-05
ENSMMUG00000023335	ring finger protein 13 [Source:VGNC Symbol;Acc:VGNC:81557]	0.793794276	0.011697523	0.046482732
ENSMMUG00000057501		-1.967579153	5.50E-06	8.86E-05
ENSMMUG00000061194		3.145429099	3.89E-08	1.38E-06
ENSMMUG00000061684		1.178896025	0.009168394	0.038627838
ENSMMUG00000053540		1.851910368	0.012766752	0.049580285
ENSMMUG00000009036	Rho guanine nucleotide exchange factor 26 [Source:VGNC Symbol;Acc:VGNC:69897]	0.591113931	0.011147479	0.044748184
ENSMMUG00000011347	DEAH-box helicase 36 [Source:VGNC Symbol;Acc:VGNC:71623]	1.01170633	3.27E-08	1.21E-06
ENSMMUG00000056116		-1.323530317	9.61E-07	2.06E-05
ENSMMUG00000023045	zinc finger B-box domain containing [Source:VGNC Symbol;Acc:VGNC:79592]	1.728121266	0.003551056	0.018211764
ENSMMUG00000051641		-1.322113546	6.79E-05	0.000720465
ENSMMUG00000022199	polyhomeotic homolog 3 [Source:VGNC Symbol;Acc:VGNC:75804]	-1.88845721	8.13E-11	8.81E-09
ENSMMUG00000056664	U6 spliceosomal RNA [Source:RFAM;Acc:RF00026]	-2.306876991	0.001245477	0.007909216
ENSMMUG00000022880	eukaryotic translation initiation factor 5A2 [Source:VGNC Symbol;Acc:VGNC:72198]	-0.908007376	0.003038095	0.016127945
ENSMMUG00000023289	phospholipase D1 [Source:VGNC Symbol;Acc:VGNC:76171]	-3.719410978	0.00384751	0.019410375
ENSMMUG00000040869	spermatogenesis associated 16 [Source:VGNC Symbol;Acc:VGNC:84073]	1.023700124	0.004032537	0.020141004
ENSMMUG00000003594	ubiquitin specific peptidase 13 [Source:VGNC Symbol;Acc:VGNC:78735]	-0.636778723	0.004189673	0.02071897
ENSMMUG00000064088	U2 spliceosomal RNA [Source:RFAM;Acc:RF00004]	-0.750904256	0.006569002	0.029567217
ENSMMUG00000008070	YEATS domain containing 2 [Source:VGNC Symbol;Acc:VGNC:79502]	1.941934406	1.81E-10	1.76E-08
ENSMMUG00000005515	ATP binding cassette subfamily C member 5 [Source:VGNC Symbol;Acc:VGNC:69574]	-0.704124602	0.001438418	0.008917097
ENSMMUG00000006553	dishevelled segment polarity protein 3 [Source:VGNC Symbol;Acc:VGNC:72014]	-0.991364636	0.006470793	0.029221284
ENSMMUG00000000800	proteasome 26S subunit, non-ATPase 2 [Source:VGNC Symbol;Acc:VGNC:84144]	-0.414931848	0.010251898	0.042128338
ENSMMUG00000007503	family with sequence similarity 131 member A [Source:VGNC Symbol;Acc:VGNC:72385]	-0.472964901	0.007862813	0.034179061

ENSMMUG00000007525	thrombopoietin [Source:VGNC Symbol;Acc:VGNC:78348]	1.569090287	2.47E-07	6.60E-06
ENSMMUG00000007528	chordin [Source:VGNC Symbol;Acc:VGNC:71042]	1.458430501	0.007990134	0.034663005
ENSMMUG00000002953	MAGE family member F1 [Source:VGNC Symbol;Acc:VGNC:74399]	0.925828002	0.010948562	0.044099412
ENSMMUG00000013018	SUMO specific peptidase 2 [Source:VGNC Symbol;Acc:VGNC:77292]	-0.547046754	0.000669311	0.004751671
ENSMMUG00000021972	diacylglycerol kinase gamma [Source:VGNC Symbol;Acc:VGNC:71785]	0.993789762	1.39E-06	2.79E-05
ENSMMUG00000052418		-0.868642216	1.43E-05	0.000197129
ENSMMUG00000018910	histidine rich glycoprotein [Source:VGNC Symbol;Acc:VGNC:73521]	1.368723373	0.00516756	0.024491765
ENSMMUG00000054175		0.404632399	0.006442066	0.029140288
ENSMMUG00000004847	xylulokinase [Source:VGNC Symbol;Acc:VGNC:78815]	0.797542707	3.82E-06	6.52E-05
ENSMMUG00000018790	activin A receptor type 2B [Source:VGNC Symbol;Acc:VGNC:69450]	2.696691674	6.13E-11	7.00E-09
ENSMMUG00000020150	xin actin binding repeat containing 1 [Source:VGNC Symbol;Acc:VGNC:78952]	-0.554313172	0.008056435	0.034867049
ENSMMUG00000051408	myelin associated oligodendrocyte basic protein [Source:NCBI gene;Acc:717265]	1.324749096	8.66E-05	0.000888279
ENSMMUG00000009197	myelin associated oligodendrocyte basic protein [Source:VGNC Symbol;Acc:VGNC:74908]	1.636731547	9.99E-07	2.11E-05
ENSMMUG00000017701	myosin VIIA and Rab interacting protein [Source:VGNC Symbol;Acc:VGNC:74992]	2.144858119	1.15E-07	3.39E-06
ENSMMUG00000047891	Metazoan signal recognition particle RNA [Source:RFAM;Acc:RF00017]	2.047187348	0.010820956	0.043680123
ENSMMUG00000013330	ectonucleoside triphosphate diphosphohydrolase 3 [Source:VGNC Symbol;Acc:VGNC:72226]	2.133975643	3.67E-08	1.32E-06
ENSMMUG00000009437	zinc finger protein 619 [Source:VGNC Symbol;Acc:VGNC:82562]	2.046035243	0.003752255	0.019031349
ENSMMUG00000050991		-1.285529455	1.04E-06	2.18E-05
ENSMMUG00000052672		5.860535645	3.14E-08	1.18E-06
ENSMMUG00000021242	natural killer cell triggering receptor [Source:VGNC Symbol;Acc:VGNC:81486]	-5.316477645	1.38E-05	0.000192025
ENSMMUG00000021243	zinc finger and BTB domain containing 47 [Source:VGNC Symbol;Acc:VGNC:79645]	-3.609229299	0.003550537	0.018211764
ENSMMUG00000021244	kelch like family member 40 [Source:VGNC Symbol;Acc:VGNC:74165]	-3.389342211	0.001582282	0.009645286
ENSMMUG00000021246	hedgehog acyltransferase like [Source:NCBI gene;Acc:717333]	-3.675324857	3.17E-05	0.000384713
ENSMMUG00000056798		2.565872837	3.90E-06	6.58E-05
ENSMMUG00000054286		1.50958841	0.000321723	0.002582315
ENSMMUG00000001028	SNF related kinase [Source:VGNC Symbol;Acc:VGNC:84074]	0.469468905	0.000665128	0.004727136
ENSMMUG00000019130	exosome component 7 [Source:VGNC Symbol;Acc:VGNC:72383]	-2.429533247	0.00054416	0.004012046
ENSMMUG00000004576	C-C motif chemokine receptor 3 [Source:VGNC Symbol;Acc:VGNC:70738]	2.974452632	3.40E-09	1.87E-07
ENSMMUG00000062394	C-C motif chemokine receptor 2 [Source:NCBI gene;Acc:574098]	1.027547869	9.13E-12	1.40E-09
ENSMMUG00000014045	protein tyrosine phosphatase non-receptor type 23 [Source:VGNC Symbol;Acc:VGNC:76477]	1.131916195	0.00418157	0.020702511
ENSMMUG00000014046	SREBF chaperone [Source:VGNC Symbol;Acc:VGNC:77140]	1.625207263	7.34E-15	2.73E-12
ENSMMUG00000064458		-1.27604169	0.000712954	0.005012262
ENSMMUG00000010058	ATR interacting protein [Source:VGNC Symbol;Acc:VGNC:84235]	0.761007433	0.000590452	0.004280613
ENSMMUG00000010062	shisa family member 5 [Source:VGNC Symbol;Acc:VGNC:84082]	0.530654209	0.002980495	0.015853727
ENSMMUG00000000591	6-phosphofructo-2-kinase/fructose-2,6-biphosphatase 4 [Source:VGNC Symbol;Acc:VGNC:75960]	0.659196361	0.000681888	0.004830411

ENSMMUG00000059318	urocortin 2 [Source:NCBI gene;Acc:106996925]	2.188145558	3.65E-09	2.00E-07
ENSMMUG00000014699		1.35891317	2.07E-09	1.27E-07
ENSMMUG00000014707	cadherin EGF LAG seven-pass G-type receptor 3 [Source:VGNC Symbol;Acc:VGNC:71148]	1.629873041	0.00395	0.019812457
ENSMMUG00000064174		1.818011403	0.002760804	0.014923945
ENSMMUG00000014720	NCK interacting protein with SH3 domain [Source:VGNC Symbol;Acc:VGNC:74960]	2.175659058	2.39E-08	9.53E-07
ENSMMUG00000006130	prolyl 4-hydroxylase, transmembrane [Source:VGNC Symbol;Acc:VGNC:75693]	2.247670603	0.001008382	0.006637152
ENSMMUG00000005660	glutamine rich 1 [Source:VGNC Symbol;Acc:VGNC:76530]	2.640576097	0.003214216	0.016870093
ENSMMUG00000014564	ring finger protein 123 [Source:VGNC Symbol;Acc:VGNC:76790]	-0.743724937	0.000300143	0.002444766
ENSMMUG00000020979	inka box actin regulator 1 [Source:VGNC Symbol;Acc:VGNC:73692]	0.757616643	0.001928335	0.011276944
ENSMMUG00000020980	ubiquitin like modifier activating enzyme 7 [Source:VGNC Symbol;Acc:VGNC:78690]	0.644928764	0.012597302	0.049073031
ENSMMUG00000016476	TRAF interacting protein [Source:VGNC Symbol;Acc:VGNC:82261]	0.675895805	3.50E-05	0.000417558
ENSMMUG00000004273	dedicator of cytokinesis 3 [Source:VGNC Symbol;Acc:VGNC:71967]	1.112809609	0.000117173	0.001131365
ENSMMUG00000017351	stabilin 1 [Source:VGNC Symbol;Acc:VGNC:77956]	-0.651555747	0.003769946	0.019100862
ENSMMUG00000050505	small integral membrane protein 4 [Source:NCBI gene;Acc:696553]	1.070354772	0.000114483	0.001112028
ENSMMUG00000012526	glycosyltransferase 8 domain containing 1 [Source:VGNC Symbol;Acc:VGNC:73001]	1.643761403	6.35E-07	1.47E-05
ENSMMUG00000017370	signal peptidase complex subunit 1 [Source:VGNC Symbol;Acc:VGNC:84072]	1.811226739	5.71E-06	9.11E-05
ENSMMUG00000007230	protein kinase C delta [Source:VGNC Symbol;Acc:VGNC:76411]	-0.746750099	0.003799482	0.019213087
ENSMMUG00000039085	adaptor protein, phosphotyrosine interacting with PH domain and leucine zipper 1 [Source:NCBI gene;Acc:100425753]	-3.222301909	0.001229877	0.007833386
ENSMMUG00000021008	ankyrin repeat and SOCS box containing 14 [Source:VGNC Symbol;Acc:VGNC:84237]	-4.089212059	0.000173402	0.001558585
ENSMMUG00000004052	phosphodiesterase 12 [Source:VGNC Symbol;Acc:VGNC:75863]	-1.380997786	0.004637178	0.022485818
ENSMMUG00000018619	DENN domain containing 6A [Source:VGNC Symbol;Acc:VGNC:71726]	2.279587958	7.95E-06	0.000119189
ENSMMUG00000017510	membrane associated guanylate kinase, WW and PDZ domain containing 1 [Source:VGNC Symbol;Acc:VGNC:74499]	1.94741158	2.07E-08	8.48E-07
ENSMMUG00000057539		2.689569427	1.32E-05	0.000184461
ENSMMUG00000057486		3.039182718	1.21E-10	1.25E-08
ENSMMUG00000057543	MyoD family inhibitor domain containing 2 [Source:HGNC Symbol;Acc:HGNC:53442]	2.034947981	2.19E-07	6.00E-06
ENSMMUG00000018281	interleukin 17 receptor E [Source:VGNC Symbol;Acc:VGNC:73623]	-0.693643365	5.73E-05	0.000624121
ENSMMUG00000018282	interleukin 17 receptor C [Source:VGNC Symbol;Acc:VGNC:84164]	-0.673481339	0.000977106	0.006474706
ENSMMUG00000018285	cysteine rich with EGF like domains 1 [Source:VGNC Symbol;Acc:VGNC:71407]	-0.749747412	0.003768705	0.019100862
ENSMMUG00000018289	proline rich transmembrane protein 3 [Source:VGNC Symbol;Acc:VGNC:76324]	-0.483034404	0.011883596	0.047052905
ENSMMUG00000019933	TatD DNase domain containing 2 [Source:NCBI gene;Acc:701390]	1.235744177	0.005907206	0.027186527
ENSMMUG00000009536	makorin ring finger protein 2 [Source:VGNC Symbol;Acc:VGNC:74878]	-2.049797193	9.06E-10	6.55E-08
ENSMMUG00000023125	XPC complex subunit, DNA damage recognition and repair factor [Source:VGNC Symbol;Acc:VGNC:78809]	-0.913759376	0.006727363	0.030160662
ENSMMUG00000023124	transmembrane protein 43 [Source:VGNC Symbol;Acc:VGNC:82711]	-1.083378135	3.26E-06	5.76E-05

ENSMMUG00000044822	coiled-coil domain containing 174 [Source:VGNC Symbol;Acc:VGNC:84219]	1.428139548	1.64E-05	0.000220685
ENSMMUG00000017125	oxysterol binding protein like 11 [Source:VGNC Symbol;Acc:VGNC:75631]	-1.068088243	5.64E-06	9.03E-05
ENSMMUG00000003851	kalirin RhoGEF kinase [Source:VGNC Symbol;Acc:VGNC:84161]	-4.108114369	0.004218281	0.02081101
ENSMMUG00000032031		-1.317291714	0.004945012	0.023627783
ENSMMUG00000049323		1.583466735	0.000849243	0.005746747
ENSMMUG00000001943	GRAM domain containing 1C [Source:VGNC Symbol;Acc:VGNC:73007]	0.973008575	0.008128298	0.035063566
ENSMMUG00000052581	upstream transcription factor family member 3 [Source:NCBI gene;Acc:709660]	-0.608449334	0.012551391	0.048977916
ENSMMUG00000014874	GTP binding protein 8 (putative) [Source:VGNC Symbol;Acc:VGNC:84180]	1.567920753	5.90E-08	1.96E-06
ENSMMUG00000014876	nucleolus and neural progenitor protein [Source:VGNC Symbol;Acc:VGNC:75244]	1.443410535	2.37E-05	0.000300566
ENSMMUG00000007843	CD200 receptor 1 like [Source:VGNC Symbol;Acc:VGNC:84212]	-2.465117292	0.008636861	0.036781603
ENSMMUG00000003061	coiled-coil domain containing 80 [Source:VGNC Symbol;Acc:VGNC:70917]	2.934039358	4.02E-08	1.41E-06
ENSMMUG00000014236	solute carrier family 35 member A5 [Source:VGNC Symbol;Acc:VGNC:84077]	1.688810352	0.006860153	0.03063982
ENSMMUG00000000894	cellular inhibitor of PP2A [Source:VGNC Symbol;Acc:VGNC:71371]	2.366069182	0.011188861	0.04488659
ENSMMUG00000019977	myosin heavy chain 15 [Source:VGNC Symbol;Acc:VGNC:84148]	-1.464291763	9.35E-05	0.000945425
ENSMMUG00000008791	CD47 molecule [Source:VGNC Symbol;Acc:VGNC:70767]	1.623617639	0.002082385	0.011967964
ENSMMUG00000008787	BBX high mobility group box domain containing [Source:VGNC Symbol;Acc:VGNC:82110]	0.922785436	0.001666674	0.010063495
ENSMMUG00000055345	Y RNA [Source:RFAM;Acc:RF00019]	0.784269117	0.002281562	0.012812781
ENSMMUG00000007565	Cbl proto-oncogene B [Source:VGNC Symbol;Acc:VGNC:70555]	-0.602608427	0.000582884	0.004242301
ENSMMUG00000026103	U1 spliceosomal RNA [Source:RFAM;Acc:RF00003]	-1.856654265	0.002451929	0.013529822
ENSMMUG00000060744		-1.578740453	1.35E-05	0.000188378
ENSMMUG00000021859	zona pellucida like domain containing 1 [Source:VGNC Symbol;Acc:VGNC:79650]	1.271273969	0.000966919	0.006419476
ENSMMUG00000063181		0.512094276	0.00986951	0.040879886
ENSMMUG00000059753		-0.601994681	0.005451032	0.025557361
ENSMMUG00000057768		-1.39189737	3.61E-05	0.000427365
ENSMMUG00000024598	U6 spliceosomal RNA [Source:RFAM;Acc:RF00026]	-4.707794128	0.000157547	0.001439513
ENSMMUG00000038349	crystallin beta-gamma domain containing 3 [Source:NCBI gene;Acc:698282]	1.399206476	3.87E-06	6.56E-05
ENSMMUG00000033738	RNA, U6atac small nuclear 6, pseudogene [Source:HGNC Symbol;Acc:HGNC:46905]	-1.032339671	9.16E-05	0.000930962
ENSMMUG00000050994		-1.940174927	0.001764153	0.010491345
ENSMMUG00000055096		0.962537413	0.000188887	0.001674942
ENSMMUG00000049913		1.291621786	0.010299861	0.042238569
ENSMMUG00000056687		-1.657586048	1.26E-08	5.52E-07
ENSMMUG00000052831		0.665167415	0.005820979	0.026884819

ENSMMUG00000032295	zinc finger protein 595 [Source:VGNC Symbol;Acc:VGNC:79655]	-1.215182271	8.35E-05	0.000864019
ENSMMUG00000051381	Metazoan signal recognition particle RNA [Source:RFAM;Acc:RF00017]	-1.547486178	5.12E-06	8.34E-05
ENSMMUG00000039020	zinc finger protein 141 [Source:NCBI gene;Acc:714265]	-1.459883162	8.48E-09	4.01E-07
ENSMMUG00000012624	polycomb group ring finger 3 [Source:VGNC Symbol;Acc:VGNC:75593]	0.924690852	0.003198165	0.016806197
ENSMMUG00000017221	transmembrane protein 175 [Source:VGNC Symbol;Acc:VGNC:79736]	-0.692000608	0.004065119	0.020226065
ENSMMUG00000008323	C-terminal binding protein 1 [Source:VGNC Symbol;Acc:VGNC:71539]	-0.634238663	0.000661174	0.004706746
ENSMMUG00000019945	fibroblast growth factor receptor 3 [Source:VGNC Symbol;Acc:VGNC:72653]	1.089669947	0.000200588	0.001766315
ENSMMUG00000046942	nuclear receptor binding SET domain protein 2 [Source:NCBI gene;Acc:100429321]	1.511391083	5.95E-05	0.000640608
ENSMMUG00000008379	adducin 1 [Source:VGNC Symbol;Acc:VGNC:69528]	0.741612048	0.006024316	0.027627736
ENSMMUG00000008385	NOP14 nucleolar protein [Source:VGNC Symbol;Acc:VGNC:75194]	0.695816562	0.004856967	0.023309859
ENSMMUG00000008392	huntingtin [Source:VGNC Symbol;Acc:VGNC:73468]	-0.640836403	0.005122652	0.024349914
ENSMMUG00000015099	janus kinase and microtubule interacting protein 1 [Source:VGNC Symbol;Acc:VGNC:73662]	-0.648251219	0.004500191	0.02191675
ENSMMUG00000019563	collapsin response mediator protein 1 [Source:VGNC Symbol;Acc:VGNC:71494]	1.385005092	6.19E-08	2.02E-06
ENSMMUG00000057703		1.437904612	5.18E-08	1.75E-06
ENSMMUG00000020861	EvC ciliary complex subunit 1 [Source:VGNC Symbol;Acc:VGNC:72445]	1.402865443	1.54E-06	3.04E-05
ENSMMUG00000023242	transmembrane protein 128 [Source:VGNC Symbol;Acc:VGNC:82257]	-0.773821466	0.00616752	0.028145534
ENSMMUG00000049640		-0.860161555	0.002714205	0.014702602
ENSMMUG00000015298	CD38 molecule [Source:VGNC Symbol;Acc:VGNC:70762]	1.693796345	0.000451722	0.003422366
ENSMMUG00000008162	fibroblast growth factor binding protein 1 [Source:VGNC Symbol;Acc:VGNC:72536]	1.583836667	8.44E-08	2.62E-06
ENSMMUG00000008163	fibroblast growth factor binding protein 2 [Source:VGNC Symbol;Acc:VGNC:72599]	1.937825027	0.001782241	0.010574738
ENSMMUG00000023116	LIM domain binding 2 [Source:VGNC Symbol;Acc:VGNC:74246]	-0.609463946	0.005562612	0.025949544
ENSMMUG00000053953	mediator complex subunit 28 [Source:NCBI gene;Acc:722863]	2.072631227	0.00222455	0.012566029
ENSMMUG00000018073	ligand dependent nuclear receptor corepressor like [Source:NCBI gene;Acc:715285]	-0.535257312	0.00866311	0.036857181
ENSMMUG00000057338		0.808669664	0.000150435	0.001389604
ENSMMUG00000060440	leucine rich repeat LGI family member 2 [Source:NCBI gene;Acc:693308]	-0.60524807	0.002974921	0.015831353
ENSMMUG00000049298	cholecystokinin A receptor [Source:NCBI gene;Acc:694742]	4.852347432	0.001063282	0.006935327
ENSMMUG00000051768		-1.823578288	0.000244342	0.002074359
ENSMMUG00000060404		5.870143704	2.04E-07	5.62E-06
ENSMMUG00000050580		0.8929371	0.002141803	0.012209878
ENSMMUG00000055079		0.548831895	0.002954924	0.015770102
ENSMMUG00000002755	kelch like family member 5 [Source:VGNC Symbol;Acc:VGNC:74188]	-4.31207065	2.49E-05	0.000314597
ENSMMUG00000001892	UDP-glucose 6-dehydrogenase [Source:VGNC Symbol;Acc:VGNC:78711]	-0.798739179	0.003603101	0.018456848
ENSMMUG00000012036	RNA binding motif protein 47 [Source:VGNC Symbol;Acc:VGNC:76576]	-0.424904519	0.01016682	0.04187133

ENSMMUG00000012561	NOP2/Sun RNA methyltransferase family member 7 [Source:VGNC Symbol;Acc:VGNC:75463]	-1.334646725	1.16E-08	5.14E-07
ENSMMUG00000007805	LIM and calponin homology domains 1 [Source:VGNC Symbol;Acc:VGNC:74155]	-2.227419027	4.08E-07	1.01E-05
ENSMMUG00000051296	paired like homeobox 2B [Source:NCBI gene;Acc:701940]	-2.399480585	2.95E-05	0.000361604
ENSMMUG00000018625	NIPA like domain containing 1 [Source:NCBI gene;Acc:704998]	-0.713900306	0.012704079	0.04941066
ENSMMUG00000055221	Metazoan signal recognition particle RNA [Source:RFAM;Acc:RF00017]	-2.007279172	0.004742329	0.022903353
ENSMMUG00000006703	solute carrier family 10 member 4 [Source:VGNC Symbol;Acc:VGNC:77553]	-1.334562687	8.48E-05	0.000872353
ENSMMUG00000006704	zygote arrest 1 [Source:VGNC Symbol;Acc:VGNC:79251]	-0.999102322	0.000632896	0.004549137
ENSMMUG00000014868	OCIA domain containing 1 [Source:VGNC Symbol;Acc:VGNC:75409]	1.317880506	0.000591808	0.004285668
ENSMMUG00000014714	cell wall biogenesis 43 C-terminal homolog [Source:VGNC Symbol;Acc:VGNC:71583]	0.579901802	0.005757719	0.026639949
ENSMMUG00000058229	U6 spliceosomal RNA [Source:RFAM;Acc:RF00026]	2.321948127	0.005360628	0.02522443
ENSMMUG00000008514	lin-54 DREAM MuvB core complex component [Source:VGNC Symbol;Acc:VGNC:74123]	1.642653364	0.000806818	0.005514174
ENSMMUG00000052743	stearoyl-CoA desaturase 5 [Source:NCBI gene;Acc:694079]	-0.823771693	0.002330954	0.012983464
ENSMMUG00000048621	enolase-phosphatase 1 [Source:NCBI gene;Acc:106998330]	3.170431751	7.65E-08	2.43E-06
ENSMMUG00000060484	heterogeneous nuclear ribonucleoprotein D [Source:NCBI gene;Acc:694867]	1.301202839	1.32E-06	2.70E-05
ENSMMUG00000052348		1.616519516	2.31E-07	6.25E-06
ENSMMUG00000021988	N-alpha-acetyltransferase 11, NatA catalytic subunit [Source:NCBI gene;Acc:696758]	1.019888459	0.000218343	0.001897684
ENSMMUG00000007366		1.085319556	0.000758425	0.005255233
ENSMMUG00000002564	Fraser extracellular matrix complex subunit 1 [Source:VGNC Symbol;Acc:VGNC:72743]	1.871943267	1.13E-05	0.000161433
ENSMMUG00000062865		1.247183564	0.000123041	0.001180142
ENSMMUG00000045609	CCR4-NOT transcription complex subunit 6 like [Source:NCBI gene;Acc:697981]	2.705038623	2.06E-08	8.47E-07
ENSMMUG00000018186	sosondowah ankyrin repeat domain family member B [Source:VGNC Symbol;Acc:VGNC:78033]	-0.892805227	2.32E-05	0.000295888
ENSMMUG00000020919	shroom family member 3 [Source:VGNC Symbol;Acc:VGNC:77352]	2.006906765	0.006300559	0.028622099
ENSMMUG00000003833	Ras association domain family member 6 [Source:VGNC Symbol;Acc:VGNC:76515]	1.631892589	0.000267326	0.002221628
ENSMMUG00000003830	alpha fetoprotein [Source:VGNC Symbol;Acc:VGNC:69741]	1.561587111	0.001134911	0.007330775
ENSMMUG00000003827	albumin [Source:VGNC Symbol;Acc:VGNC:69782]	1.175809394	0.002740805	0.014828165
ENSMMUG00000000790	ankyrin repeat domain 17 [Source:VGNC Symbol;Acc:VGNC:84240]	1.932099366	0.000145259	0.001353319
ENSMMUG00000062248	follicular dendritic cell secreted protein [Source:NCBI gene;Acc:707784]	2.114328553	2.48E-08	9.77E-07
ENSMMUG00000006029	odontogenic, ameloblast associated [Source:NCBI gene;Acc:707906]	2.296128742	2.62E-08	1.02E-06
ENSMMUG00000004849	casein beta [Source:VGNC Symbol;Acc:VGNC:71523]	-0.942668438	0.003213346	0.016870093
ENSMMUG00000001919	clock circadian regulator [Source:VGNC Symbol;Acc:VGNC:71262]	-0.802190836	0.000554616	0.004077634
ENSMMUG00000001918	transmembrane protein 165 [Source:VGNC Symbol;Acc:VGNC:79875]	-1.251426412	0.000535556	0.003962142
ENSMMUG00000057204	Metazoan signal recognition particle RNA [Source:RFAM;Acc:RF00017]	1.666817515	0.007533215	0.033068159

ENSMMUG00000018943	spermatogenesis associated 18 [Source:VGNC Symbol;Acc:VGNC:78013]	-1.262937957	0.005072338	0.024156289
ENSMMUG00000018939	leucine rich repeat containing 66 [Source:VGNC Symbol;Acc:VGNC:74415]	-1.211538522	5.78E-05	0.000628586
ENSMMUG00000003726	protein tyrosine phosphatase non-receptor type 13 [Source:VGNC Symbol;Acc:VGNC:76599]	-0.859220166	0.003000957	0.015943809
ENSMMUG00000014076	AF4/FMR2 family member 1 [Source:VGNC Symbol;Acc:VGNC:69817]	1.048530628	0.001867676	0.010976502
ENSMMUG00000051677		1.125622663	1.80E-05	0.000238404
ENSMMUG00000019040	hydroxysteroid 17-beta dehydrogenase 13 [Source:VGNC Symbol;Acc:VGNC:73417]	0.727481206	0.002055239	0.011869644
ENSMMUG00000008795	polycystin 2, transient receptor potential cation channel [Source:NCBI gene;Acc:702179]	-0.615415081	0.010301456	0.042238569
ENSMMUG00000010316	HECT and RLD domain containing E3 ubiquitin protein ligase 5 [Source:VGNC Symbol;Acc:VGNC:73226]	2.703799577	1.11E-07	3.30E-06
ENSMMUG00000010318	phosphatidylinositol glycan anchor biosynthesis class Y [Source:NCBI gene;Acc:100426107]	2.90406696	2.65E-06	4.84E-05
ENSMMUG00000058544	nucleosome assembly protein 1 like 5 [Source:NCBI gene;Acc:703223]	0.505116265	0.007841344	0.034108533
ENSMMUG00000012123	synuclein alpha [Source:VGNC Symbol;Acc:VGNC:77668]	3.541181038	4.36E-06	7.24E-05
ENSMMUG00000022003	multimerin 1 [Source:VGNC Symbol;Acc:VGNC:74904]	2.577808559	1.83E-08	7.77E-07
ENSMMUG00000022246	PDZ and LIM domain 5 [Source:VGNC Symbol;Acc:VGNC:75816]	0.989972174	0.003398634	0.017589588
ENSMMUG00000060760		1.432774986	0.00648292	0.029252934
ENSMMUG00000006309	unc-5 netrin receptor C [Source:VGNC Symbol;Acc:VGNC:78723]	-4.210663739	2.21E-05	0.000283752
ENSMMUG00000011702	tetraspanin 5 [Source:VGNC Symbol;Acc:VGNC:79086]	-1.439735567	9.23E-05	0.000935885
ENSMMUG00000063972		-0.754615932	0.011960995	0.047247365
ENSMMUG00000018738	alcohol dehydrogenase 1B (class I), beta polypeptide [Source:NCBI gene;Acc:707908]	-0.581993085	0.001046054	0.006833237
ENSMMUG00000061366		0.527449664	0.006550626	0.029520941
ENSMMUG00000016880	solute carrier family 39 member 8 [Source:VGNC Symbol;Acc:VGNC:77541]	-3.060096684	6.62E-14	1.87E-11
ENSMMUG00000037551	Metazoan signal recognition particle RNA [Source:RFAM;Acc:RF00017]	-3.510366728	5.88E-15	2.25E-12
ENSMMUG00000003736	nuclear factor kappa B subunit 1 [Source:VGNC Symbol;Acc:VGNC:75339]	-3.3573236	5.09E-14	1.50E-11
ENSMMUG00000003739	mannosidase beta [Source:VGNC Symbol;Acc:VGNC:74486]	1.372591798	0.009441016	0.039546447
ENSMMUG00000019085	3-hydroxybutyrate dehydrogenase 2 [Source:VGNC Symbol;Acc:VGNC:70161]	1.048483701	0.000200756	0.001766596
ENSMMUG00000019089	centromere protein E [Source:VGNC Symbol;Acc:VGNC:71008]	0.863050266	0.000107762	0.001056981
ENSMMUG00000049998	tachykinin receptor 3 [Source:NCBI gene;Acc:653013]	1.593673602	9.44E-06	0.00013703
ENSMMUG00000010941	Rho guanine nucleotide exchange factor 38 [Source:VGNC Symbol;Acc:VGNC:82087]	-0.459153352	0.003748869	0.019031288
ENSMMUG00000018811	glutathione S-transferase C-terminal domain containing [Source:VGNC Symbol;Acc:VGNC:73234]	-1.144189054	0.000510018	0.003794792
ENSMMUG00000060432		-1.327782916	0.005169145	0.024491765
ENSMMUG00000052609	ribosomal protein L34 [Source:NCBI gene;Acc:696636]	-2.461787954	3.66E-06	6.31E-05
ENSMMUG00000011902	oligosaccharyltransferase complex non-catalytic subunit [Source:NCBI gene;Acc:697283]	-2.390627377	1.55E-06	3.05E-05
ENSMMUG00000055759	Metazoan signal recognition particle RNA [Source:RFAM;Acc:RF00017]	-1.999697785	1.80E-05	0.000238404
ENSMMUG00000005517	glutamyl aminopeptidase [Source:VGNC Symbol;Acc:VGNC:72221]	3.991156461	4.03E-05	0.000464276

ENSMMUG00000062794		2.730768464	4.46E-07	1.08E-05
ENSMMUG00000008705	ubiquitin specific peptidase 53 [Source:VGNC Symbol;Acc:VGNC:79793]	-3.862013477	0.000734566	0.005136436
ENSMMUG00000061098		1.527946329	2.51E-08	9.86E-07
ENSMMUG00000007422	spermatogenesis associated 5 [Source:VGNC Symbol;Acc:VGNC:77952]	0.97867135	0.000308079	0.002496498
ENSMMUG00000001848	sprouty RTK signaling antagonist 1 [Source:VGNC Symbol;Acc:VGNC:77867]	0.649012881	0.001840323	0.010861092
ENSMMUG00000011810	FAT atypical cadherin 4 [Source:VGNC Symbol;Acc:VGNC:72552]	-1.718139805	5.31E-05	0.000586082
ENSMMUG00000051329		1.694357208	0.000146787	0.001366583
ENSMMUG00000015244	uncoupling protein 1 [Source:VGNC Symbol;Acc:VGNC:78708]	3.431931333	9.68E-06	0.000140146
ENSMMUG00000015246	TBC1 domain family member 9 [Source:VGNC Symbol;Acc:VGNC:78193]	4.557390298	4.37E-06	7.24E-05
ENSMMUG00000060157	zinc finger protein 330 [Source:NCBI gene;Acc:699364]	0.939347606	0.000397648	0.003085808
ENSMMUG00000000827	ubiquitin specific peptidase 38 [Source:VGNC Symbol;Acc:VGNC:79224]	2.054912359	0.001869838	0.010984243
ENSMMUG00000044429	glycophorin A (MNS blood group) [Source:NCBI gene;Acc:574101]	2.087586165	1.56E-06	3.05E-05
ENSMMUG00000007968	hedgehog interacting protein [Source:VGNC Symbol;Acc:VGNC:73169]	-0.989465166	0.003481299	0.017910513
ENSMMUG00000005380	zinc finger protein 827 [Source:NCBI gene;Acc:706018]	1.316297963	1.28E-05	0.000179775
ENSMMUG00000014953	Rho GTPase activating protein 10 [Source:VGNC Symbol;Acc:VGNC:69914]	-1.261308058	0.007074971	0.031415867
ENSMMUG00000014955	nuclear receptor subfamily 3 group C member 2 [Source:VGNC Symbol;Acc:VGNC:75516]	-1.914224101	7.82E-05	0.000814571
ENSMMUG00000002211	doublecortin like kinase 2 [Source:VGNC Symbol;Acc:VGNC:71670]	0.928922886	0.003375851	0.01749953
ENSMMUG00000002212	LPS responsive beige-like anchor protein [Source:NCBI gene;Acc:693823]	-1.728358485	0.000234473	0.002006283
ENSMMUG00000039441	mab-21 like 2 [Source:NCBI gene;Acc:693948]	-2.207571932	0.001651743	0.009987245
ENSMMUG00000059101		-2.571312244	5.88E-06	9.33E-05
ENSMMUG00000052327		-1.528388028	0.002422312	0.013395075
ENSMMUG00000018490	ADP ribosylation factor interacting protein 1 [Source:VGNC Symbol;Acc:VGNC:69878]	-0.946663282	0.000363329	0.002870875
ENSMMUG00000039869		-0.5489932	0.001954689	0.011390094
ENSMMUG00000057643		0.605784845	0.012518464	0.048908123
ENSMMUG00000052999	lecithin retinol acyltransferase [Source:NCBI gene;Acc:698871]	0.620722694	0.009157755	0.038608027
ENSMMUG00000064074		0.732300512	0.008315665	0.035658931
ENSMMUG00000023244	guanylate cyclase 1 soluble subunit alpha 1 [Source:VGNC Symbol;Acc:VGNC:73327]	1.602649869	0.003464391	0.017865927
ENSMMUG00000064707		1.120913807	0.012904671	0.049936969
ENSMMUG00000053790		1.30146221	1.02E-08	4.67E-07
ENSMMUG00000010581	Rap guanine nucleotide exchange factor 2 [Source:VGNC Symbol;Acc:VGNC:76493]	2.033135775	0.001757341	0.010460404
ENSMMUG00000053881		2.202323934	0.000255782	0.002143489
ENSMMUG00000006851	SPARC (osteonectin), cwcv and kazal like domains proteoglycan 3 [Source:NCBI gene;Acc:705964]	1.681439371	0.010685464	0.043240554
ENSMMUG00000001879	annexin A10 [Source:VGNC Symbol;Acc:VGNC:69945]	0.953680675	0.000343861	0.002740275
ENSMMUG00000001880	DExD/H-box helicase 60 [Source:VGNC Symbol;Acc:VGNC:71711]	1.032558612	0.000433135	0.003309844
ENSMMUG00000003634	DExD/H-box 60 like [Source:VGNC Symbol;Acc:VGNC:71720]	-0.885898947	0.004779357	0.023039407
ENSMMUG00000052179		1.617892067	0.010378024	0.042400013
ENSMMUG00000013730	WD repeat domain 17 [Source:NCBI gene;Acc:700546]	0.65931348	0.000249968	0.002108354

ENSMMUG00000013733	spermatogenesis associated 4 [Source:VGNC Symbol;Acc:VGNC:77938]	1.669225653	7.15E-18	5.47E-15
ENSMMUG00000058974		-1.515534501	0.002113307	0.012113559
ENSMMUG00000011633	kallikrein B1 [Source:VGNC Symbol;Acc:VGNC:74195]	2.135795868	0.002144939	0.012209878
ENSMMUG00000018051	FAT atypical cadherin 1 [Source:VGNC Symbol;Acc:VGNC:72617]	-0.707215864	0.000371569	0.002918714
ENSMMUG00000050600		3.710801313	0.001566363	0.009591391
ENSMMUG00000058448		0.695831389	0.012698142	0.04941066
ENSMMUG00000050465	ZFP42 zinc finger protein [Source:NCBI gene;Acc:707621]	2.54653709	7.80E-10	5.83E-08
ENSMMUG00000064721		-2.694225473	0.001191521	0.007630196
ENSMMUG00000052287		-2.451610345	0.000762985	0.005284019
ENSMMUG00000044628		-2.617964345	0.000182087	0.00162096
ENSMMUG00000015797	disco interacting protein 2 homolog A [Source:VGNC Symbol;Acc:VGNC:71641]	0.484111634	0.003653323	0.018669988
ENSMMUG00000022324	formimidoyltransferase cyclodeaminase [Source:VGNC Symbol;Acc:VGNC:72825]	-2.176772391	0.002690708	0.014587473
ENSMMUG00000052777		1.347454792	0.009346525	0.039213195
ENSMMUG00000010695	adenosine deaminase RNA specific B1 [Source:VGNC Symbol;Acc:VGNC:69503]	-1.140618619	0.002259809	0.012728917
ENSMMUG00000000951	integrin subunit beta 2 [Source:VGNC Symbol;Acc:VGNC:73786]	1.44722932	2.65E-07	7.01E-06
ENSMMUG00000049755	leucine rich repeat containing 3 [Source:NCBI gene;Acc:712418]	3.118719801	9.25E-05	0.000937817
ENSMMUG00000004175	transient receptor potential cation channel subfamily M member 2 [Source:VGNC Symbol;Acc:VGNC:78655]	2.391532535	5.09E-05	0.000566542
ENSMMUG00000004173	phosphofructokinase, liver type [Source:VGNC Symbol;Acc:VGNC:81737]	-1.079739485	5.77E-06	9.17E-05
ENSMMUG00000010648	DNA methyltransferase 3 like [Source:NCBI gene;Acc:722184]	-2.321462074	3.22E-10	2.77E-08
ENSMMUG00000010647	inducible T cell costimulator ligand [Source:NCBI gene;Acc:722191]	1.814659676	0.000138863	0.001302131
ENSMMUG00000045179	1-acylglycerol-3-phosphate O-acyltransferase 3 [Source:NCBI gene;Acc:713301]	1.576803352	1.39E-09	8.98E-08
ENSMMUG00000000104	ribosomal RNA processing 1B [Source:VGNC Symbol;Acc:VGNC:77089]	-1.177943443	0.001055049	0.006888535
ENSMMUG00000020050	salt inducible kinase 1 [Source:NCBI gene;Acc:722303]	-2.128906958	0.002953112	0.015766903
ENSMMUG00000018081	ubiquitin associated and SH3 domain containing A [Source:VGNC Symbol;Acc:VGNC:78687]	-1.140874641	0.005472136	0.025619365
ENSMMUG00000018079	transmembrane serine protease 3 [Source:VGNC Symbol;Acc:VGNC:78576]	-1.25206804	0.000417961	0.003216572
ENSMMUG00000018058	ATP binding cassette subfamily G member 1 [Source:VGNC Symbol;Acc:VGNC:69584]	-2.183111065	4.46E-07	1.08E-05
ENSMMUG00000018057	uromodulin like 1 [Source:VGNC Symbol;Acc:VGNC:78717]	-1.491559005	2.59E-05	0.000325032
ENSMMUG00000018053	zinc finger and BTB domain containing 21 [Source:VGNC Symbol;Acc:VGNC:78892]	-0.824568546	0.000633088	0.004549137
ENSMMUG00000007030	C2 calcium dependent domain containing 2 [Source:VGNC Symbol;Acc:VGNC:70298]	-2.636003129	0.002984524	0.015862983
ENSMMUG00000015329	MX dynamin like GTPase 1 [Source:VGNC Symbol;Acc:VGNC:75086]	2.53788768	1.43E-05	0.000197181
ENSMMUG00000044257	MX dynamin like GTPase 2 [Source:VGNC Symbol;Acc:VGNC:81723]	3.018969356	3.40E-08	1.24E-06
ENSMMUG00000004257	beta-secretase 2 [Source:VGNC Symbol;Acc:VGNC:70216]	2.776838407	1.33E-15	5.98E-13

ENSMMUG00000010296	ETS transcription factor ERG [Source:VGNC Symbol;Acc:VGNC:72427]	3.974139972	0.003268748	0.01709928
ENSMMUG00000056855	potassium inwardly rectifying channel subfamily J member 15 [Source:NCBI gene;Acc:693427]	2.757661923	0.001283335	0.008106386
ENSMMUG00000056593		3.473031359	2.78E-05	0.000344757
ENSMMUG00000017505	tetratricopeptide repeat domain 3 [Source:VGNC Symbol;Acc:VGNC:81785]	0.676185639	3.53E-06	6.14E-05
ENSMMUG00000062048		0.725436965	4.10E-05	0.000471215
ENSMMUG00000028022	Y RNA [Source:RFAM;Acc:RF00019]	1.012708199	0.00201862	0.0117032
ENSMMUG00000010336	DOP1 leucine zipper like protein B [Source:VGNC Symbol;Acc:VGNC:71832]	0.591164776	0.000309236	0.002501198
ENSMMUG00000004605	chloride intracellular channel 6 [Source:VGNC Symbol;Acc:VGNC:71256]	0.832181551	0.009747962	0.040479564
ENSMMUG00000053087	mitochondrial ribosomal protein S6 [Source:NCBI gene;Acc:697852]	3.752304286	0.000191149	0.001693515
ENSMMUG00000065271		3.94737322	3.05E-06	5.41E-05
ENSMMUG00000003701	ATP synthase peripheral stalk subunit OSCP [Source:NCBI gene;Acc:698480]	2.121272668	0.000357467	0.002834891
ENSMMUG00000003703	crystallin zeta like 1 [Source:VGNC Symbol;Acc:VGNC:81297]	2.559059037	7.66E-05	0.000801017
ENSMMUG00000005511	SON DNA binding protein [Source:VGNC Symbol;Acc:VGNC:77689]	2.310362653	1.06E-07	3.17E-06
ENSMMUG00000022702	phosphoribosylglycinamide formyltransferase, phosphoribosylglycinamide synthetase, phosphoribosylaminoimidazole synthetase [Source:VGNC Symbol;Acc:VGNC:72882]	1.866357073	1.28E-05	0.000179304
ENSMMUG00000003956	interferon alpha and beta receptor subunit 2 [Source:VGNC Symbol;Acc:VGNC:81330]	0.466622573	0.00590246	0.0271743
ENSMMUG00000052780		1.712409868	2.18E-08	8.80E-07
ENSMMUG00000048824	eva-1 homolog C [Source:NCBI gene;Acc:708681]	1.152468894	8.32E-07	1.84E-05
ENSMMUG00000059707	MIS18 kinetochore protein A [Source:NCBI gene;Acc:709275]	-2.58814451	2.88E-05	0.000355024
ENSMMUG00000056363		-1.510005372	0.01108169	0.044524904
ENSMMUG00000061208	MAP3K7 C-terminal like [Source:NCBI gene;Acc:713717]	1.425740232	5.28E-07	1.26E-05
ENSMMUG00000017605	mitochondrial ribosomal protein L39 [Source:VGNC Symbol;Acc:VGNC:74737]	0.74276579	0.004957513	0.023670124
ENSMMUG00000060576		1.399255343	9.28E-06	0.000135223
ENSMMUG00000057702		0.944161193	0.001517669	0.009332768
ENSMMUG00000052328	FAM20C golgi associated secretory pathway kinase [Source:NCBI gene;Acc:695088]	1.670006088	0.001165192	0.007502219
ENSMMUG00000017056	Sad1 and UNC84 domain containing 1 [Source:VGNC Symbol;Acc:VGNC:78161]	3.618460472	1.87E-09	1.18E-07
ENSMMUG00000003283	extracellular leucine rich repeat and fibronectin type III domain containing 1 [Source:VGNC Symbol;Acc:VGNC:72204]	1.286188059	7.56E-07	1.70E-05
ENSMMUG00000021075	Rac family small GTPase 1 [Source:NCBI gene;Acc:717755]	1.003717893	3.36E-07	8.60E-06
ENSMMUG00000003167	cytohesin 3 [Source:VGNC Symbol;Acc:VGNC:71829]	1.172866455	9.56E-06	0.000138671
ENSMMUG00000030422	radial spoke head 10 homolog B [Source:NCBI gene;Acc:100424218]	-0.535679245	0.001032994	0.006751316
ENSMMUG00000015477	lemur tyrosine kinase 2 [Source:VGNC Symbol;Acc:VGNC:74309]	0.911103954	0.002174481	0.012350182
ENSMMUG00000015479	tectonin beta-propeller repeat containing 1 [Source:VGNC Symbol;Acc:VGNC:79348]	1.15951703	6.10E-06	9.56E-05
ENSMMUG00000011970	neuronal pentraxin 2 [Source:VGNC Symbol;Acc:VGNC:75392]	2.236004085	2.79E-06	5.05E-05
ENSMMUG00000023318	transformation/transcription domain associated protein [Source:VGNC Symbol;Acc:VGNC:78657]	0.684733796	1.93E-07	5.36E-06
ENSMMUG00000040393	zinc finger protein 655 [Source:NCBI gene;Acc:718811]	1.952260687	0.000425203	0.003260727
ENSMMUG00000022728	zinc finger and SCAN domain containing 25 [Source:VGNC Symbol;Acc:VGNC:79344]	1.575410005	8.84E-06	0.000130165

ENSMMUG00000022730	cytochrome P450, family 3, subfamily A, polypeptide 5 [Source:NCBI gene;Acc:678692]	1.226729909	1.22E-07	3.55E-06
ENSMMUG00000022504	alpha-2-glycoprotein 1, zinc-binding [Source:VGNC Symbol;Acc:VGNC:70201]	0.522707476	0.004250132	0.020906663
ENSMMUG00000004021	zinc finger protein 3 [Source:VGNC Symbol;Acc:VGNC:81889]	-2.19187247	0.010173403	0.041871925
ENSMMUG00000048302	ArfGAP with FG repeats 2 [Source:VGNC Symbol;Acc:VGNC:80329]	-0.614478162	0.000210154	0.001835635
ENSMMUG00000015311	leucine rich repeats and calponin homology domain containing 4 [Source:VGNC Symbol;Acc:VGNC:74441]	-0.458887737	0.00110511	0.007161464
ENSMMUG00000015309	F-box protein 24 [Source:VGNC Symbol;Acc:VGNC:72495]	-0.499304904	0.005865979	0.027054226
ENSMMUG00000002949	motile sperm domain containing 3 [Source:VGNC Symbol;Acc:VGNC:74823]	1.625638594	9.82E-09	4.54E-07
ENSMMUG00000002950	transferrin receptor 2 [Source:VGNC Symbol;Acc:VGNC:81770]	2.276471218	1.25E-08	5.52E-07
ENSMMUG00000004082	GRB10 interacting GYF protein 1 [Source:VGNC Symbol;Acc:VGNC:73158]	-1.393266522	0.002350358	0.01306622
ENSMMUG00000022840	zonadhesin [Source:VGNC Symbol;Acc:VGNC:81797]	-0.759245035	0.00749448	0.032952402
ENSMMUG00000060459		0.693926171	0.002350835	0.01306622
ENSMMUG00000063570	Metazoan signal recognition particle RNA [Source:RFAM;Acc:RF00017]	-3.035190366	0.008662408	0.036857181
ENSMMUG00000058787		3.156830491	0.007514256	0.033028188
ENSMMUG00000011367	BUD23 rRNA methyltransferase and ribosome maturation factor [Source:VGNC Symbol;Acc:VGNC:70325]	-3.164666682	0.007181049	0.031778402
ENSMMUG00000056841		1.150600754	0.009698276	0.040337631
ENSMMUG00000058227	Metazoan signal recognition particle RNA [Source:RFAM;Acc:RF00017]	1.667813594	0.007227855	0.031942059
ENSMMUG00000050132		1.175483583	9.38E-05	0.00094672
ENSMMUG00000015272	chaperonin containing TCP1 subunit 6A [Source:NCBI gene;Acc:703326]	0.888541855	1.29E-06	2.64E-05
ENSMMUG00000058499		4.426984379	0.007517754	0.033032403
ENSMMUG00000060184	SEC61 translocon subunit gamma [Source:NCBI gene;Acc:716898]	6.273872	1.47E-06	2.93E-05
ENSMMUG00000019991	dopa decarboxylase [Source:VGNC Symbol;Acc:VGNC:71755]	0.722654463	0.000235465	0.002012125
ENSMMUG00000049620		-1.260010991	0.006111071	0.027936937
ENSMMUG00000016191	ATP binding cassette subfamily A member 13 [Source:VGNC Symbol;Acc:VGNC:69402]	3.237062559	0.003339928	0.017361754
ENSMMUG00000019393	tensin 3 [Source:VGNC Symbol;Acc:VGNC:79324]	-4.129781309	0.000424319	0.003256352
ENSMMUG00000002433	calcium/calmodulin dependent protein kinase II beta [Source:VGNC Symbol;Acc:VGNC:70524]	2.403228275	0.000266759	0.002218333
ENSMMUG00000002430	YKT6 v-SNARE homolog [Source:VGNC Symbol;Acc:VGNC:81796]	2.674139869	2.02E-06	3.83E-05
ENSMMUG00000002427	glucokinase [Source:VGNC Symbol;Acc:VGNC:72901]	2.294406568	1.15E-11	1.69E-09
ENSMMUG00000002411	drebrin like [Source:NCBI gene;Acc:720621]	2.59182469	3.19E-15	1.26E-12
ENSMMUG00000002426	myosin light chain 7 [Source:VGNC Symbol;Acc:VGNC:75002]	2.129032741	2.41E-11	3.10E-09
ENSMMUG00000002423	DNA polymerase delta 2, accessory subunit [Source:VGNC Symbol;Acc:VGNC:76203]	2.35990867	2.71E-11	3.39E-09
ENSMMUG00000002422	AE binding protein 1 [Source:VGNC Symbol;Acc:VGNC:80328]	2.500391171	3.09E-09	1.73E-07
ENSMMUG00000019022	serine/threonine kinase 17a [Source:VGNC Symbol;Acc:VGNC:77913]	0.446616756	0.012010729	0.047423054
ENSMMUG00000057956		0.737333123	0.002769885	0.014960601
ENSMMUG00000053696	Metazoan signal recognition particle RNA [Source:RFAM;Acc:RF00017]	2.1467353	5.02E-05	0.000559578
ENSMMUG00000060273	mitochondrial ribosomal protein L32 [Source:NCBI gene;Acc:701820]	1.966318926	2.20E-07	6.02E-06

ENSMMUG00000022451	proteasome 20S subunit alpha 2 [Source:NCBI gene;Acc:701683]	2.258943674	3.68E-07	9.30E-06
ENSMMUG00000001702	succinyl-CoA:glutarate-CoA transferase [Source:VGNC Symbol;Acc:VGNC:81767]	1.562088822	3.18E-08	1.18E-06
ENSMMUG00000065307		-1.470354924	0.000650023	0.004650277
ENSMMUG00000001350	HERPUD family member 2 [Source:VGNC Symbol;Acc:VGNC:73216]	2.618928083	3.54E-10	3.03E-08
ENSMMUG00000058423		2.964156604	1.13E-07	3.32E-06
ENSMMUG00000002886	Bardet-Biedl syndrome 9 [Source:NCBI gene;Acc:708412]	1.485459231	0.000136538	0.001289626
ENSMMUG00000011695	5'-nucleotidase, cytosolic IIIA [Source:VGNC Symbol;Acc:VGNC:75423]	-0.50580495	0.012053712	0.047578325
ENSMMUG00000055809		-1.980681804	0.000105844	0.001042098
ENSMMUG00000062454		2.197567295	4.67E-08	1.61E-06
ENSMMUG00000039009		1.760644702	0.000352828	0.002803226
ENSMMUG00000012529	pleckstrin homology domain containing A8 [Source:VGNC Symbol;Acc:VGNC:81741]	1.250093512	0.000136744	0.001290205
ENSMMUG00000020184	carboxypeptidase vitellogenic like [Source:VGNC Symbol;Acc:VGNC:71406]	-2.81271879	4.34E-08	1.51E-06
ENSMMUG00000039317	cAMP responsive element binding protein 5 [Source:VGNC Symbol;Acc:VGNC:81295]	-2.168429387	0.001187419	0.007611423
ENSMMUG00000000517	family with sequence similarity 126 member A [Source:VGNC Symbol;Acc:VGNC:72327]	-1.765031299	0.000888991	0.005993894
ENSMMUG00000042472	Metazoan signal recognition particle RNA [Source:RFAM;Acc:RF00017]	0.77772082	0.010724975	0.043359972
ENSMMUG00000007821	ATP binding cassette subfamily B member 5 [Source:VGNC Symbol;Acc:VGNC:80321]	-2.788878118	1.27E-16	6.86E-14
ENSMMUG00000006898	integrin subunit beta 8 [Source:VGNC Symbol;Acc:VGNC:73791]	-3.174476585	2.70E-20	3.51E-17
ENSMMUG00000062260		4.517819765	0.007132216	0.031616089
ENSMMUG00000052574		-1.13067812	3.50E-06	6.09E-05
ENSMMUG00000012694	thrombospondin type 1 domain containing 7A [Source:VGNC Symbol;Acc:VGNC:79347]	-1.478818948	0.011826939	0.046923153
ENSMMUG00000041616	G protein subunit gamma 11 [Source:VGNC Symbol;Acc:VGNC:81319]	3.515081197	0.010430497	0.042540867
ENSMMUG00000045513		2.151331829	4.16E-08	1.46E-06
ENSMMUG00000019802	family with sequence similarity 133 member B [Source:NCBI gene;Acc:702054]	2.294697621	0.000396299	0.003079015
ENSMMUG00000015685	zinc finger protein 804B [Source:VGNC Symbol;Acc:VGNC:81890]	1.654086612	2.80E-10	2.48E-08
ENSMMUG00000022697	STEAP4 metalloreductase [Source:VGNC Symbol;Acc:VGNC:77974]	-0.759113776	0.003897827	0.01959611
ENSMMUG00000006766	RUN domain containing 3B [Source:VGNC Symbol;Acc:VGNC:77129]	1.408446035	0.000432079	0.003303718
ENSMMUG00000010788	ATP binding cassette subfamily B member 1 [Source:NCBI gene;Acc:574235]	-0.581302817	0.011901147	0.047083121
ENSMMUG00000000480	KIAA1324 like [Source:VGNC Symbol;Acc:VGNC:73882]	2.723473859	0.004746778	0.022916329
ENSMMUG00000053516		1.741475483	3.95E-05	0.000457578
ENSMMUG00000049939		-0.997762853	1.20E-06	2.47E-05
ENSMMUG00000009006	round spermatid basic protein 1 like [Source:VGNC Symbol;Acc:VGNC:77097]	-0.717865953	0.001683246	0.010135322
ENSMMUG00000012427	F-box and leucine rich repeat protein 13 [Source:VGNC Symbol;Acc:VGNC:81312]	-0.666433805	0.001270017	0.00803397
ENSMMUG00000022727	lysine methyltransferase 2E [Source:VGNC Symbol;Acc:VGNC:74073]	-4.477384671	0.000129149	0.001230561

ENSMMUG00000022732	SRSF protein kinase 2 [Source:VGNC Symbol;Acc:VGNC:81764]	-0.804261267	0.003021747	0.016047707
ENSMMUG00000008189	cadherin related family member 3 [Source:HGNC Symbol;Acc:HGNC:26308]	1.408148164	2.19E-06	4.09E-05
ENSMMUG00000003572	nicotinamide phosphoribosyltransferase [Source:VGNC Symbol;Acc:VGNC:75056]	1.072866684	0.00158258	0.009645286
ENSMMUG00000030218	B cell receptor associated protein 29 [Source:VGNC Symbol;Acc:VGNC:70119]	0.537038984	0.006740244	0.030207997
ENSMMUG00000015535	inner mitochondrial membrane peptidase subunit 2 [Source:VGNC Symbol;Acc:VGNC:73596]	0.874320945	0.000257375	0.002152678
ENSMMUG00000062092		3.403398818	0.011131234	0.044696766
ENSMMUG00000058817		3.124145574	6.46E-11	7.24E-09
ENSMMUG00000007097	testin LIM domain protein [Source:VGNC Symbol;Acc:VGNC:78383]	0.530872564	0.008211678	0.035317818
ENSMMUG00000011269	CF transmembrane conductance regulator [Source:VGNC Symbol;Acc:VGNC:71020]	-0.906085345	0.000341808	0.002725663
ENSMMUG00000023308	potassium voltage-gated channel subfamily D member 2 [Source:VGNC Symbol;Acc:VGNC:73976]	1.941652603	0.001335861	0.008385233
ENSMMUG00000023310	tetraspanin 12 [Source:VGNC Symbol;Acc:VGNC:79328]	2.975435343	0.001771189	0.010523565
ENSMMUG00000002434	inhibitor of growth family member 3 [Source:VGNC Symbol;Acc:VGNC:73595]	-2.66170681	1.08E-07	3.20E-06
ENSMMUG00000002428	zinc finger protein 800 [Source:VGNC Symbol;Acc:VGNC:79658]	1.305295394	1.11E-08	4.95E-07
ENSMMUG00000007250	RNA binding motif protein 28 [Source:VGNC Symbol;Acc:VGNC:76519]	-0.928970969	0.004929012	0.023594673
ENSMMUG00000012110	smoothened, frizzled class receptor [Source:VGNC Symbol;Acc:VGNC:77658]	-1.543126414	1.89E-07	5.28E-06
ENSMMUG00000041919	nuclear respiratory factor 1 [Source:VGNC Symbol;Acc:VGNC:81731]	2.790494101	2.54E-07	6.77E-06
ENSMMUG00000009494	carboxypeptidase A5 [Source:VGNC Symbol;Acc:VGNC:71410]	0.958146701	0.001968665	0.011446075
ENSMMUG00000009499	mesoderm specific transcript [Source:VGNC Symbol;Acc:VGNC:74707]	1.451480389	0.003802341	0.019220074
ENSMMUG00000004801	COPI coat complex subunit gamma 2 [Source:VGNC Symbol;Acc:VGNC:71315]	1.593427202	0.001589346	0.009672924
ENSMMUG00000025903		-3.050375783	0.010343391	0.042357091
ENSMMUG00000001671	coiled-coil-helix-coiled-coil-helix domain containing 3 [Source:VGNC Symbol;Acc:VGNC:80860]	-1.430670148	0.001383089	0.008639987
ENSMMUG00000000917	aldo-keto reductase family 1 member B [Source:NCBI gene;Acc:705957]	1.627931404	0.003232391	0.016939761
ENSMMUG00000013098	aldo-keto reductase family 1 member B10 [Source:NCBI gene;Acc:713150]	3.399011646	3.31E-06	5.83E-05
ENSMMUG00000037485		-3.150834268	0.005967297	0.027433959
ENSMMUG00000054821		-2.741940979	0.008773613	0.037220122
ENSMMUG00000006530	ATP/GTP binding protein like 3 [Source:VGNC Symbol;Acc:VGNC:69723]	0.967661211	1.79E-05	0.000237441
ENSMMUG00000050339	Metazoan signal recognition particle RNA [Source:RFAM;Acc:RF00017]	2.126700287	4.65E-09	2.42E-07
ENSMMUG00000002995	tripartite motif containing 24 [Source:VGNC Symbol;Acc:VGNC:78562]	0.720443227	0.012914649	0.049960715
ENSMMUG00000006547	ATPase H+ transporting V0 subunit a4 [Source:VGNC Symbol;Acc:VGNC:70189]	-3.024860408	0.002327104	0.012982861
ENSMMUG00000006548	KIAA1549 [Source:VGNC Symbol;Acc:VGNC:73885]	-1.842268285	0.00128904	0.008134526

ENSMMUG00000013645	tetratricopeptide repeat domain 26 [Source:VGNC Symbol;Acc:VGNC:79341]	-0.833878605	0.010881662	0.04385711
ENSMMUG00000004224	poly(ADP-ribose) polymerase family member 12 [Source:VGNC Symbol;Acc:VGNC:81734]	0.972449212	3.42E-05	0.000410181
ENSMMUG00000056175		0.789553822	0.004792318	0.023087405
ENSMMUG00000008126	lysine demethylase 7A [Source:VGNC Symbol;Acc:VGNC:73886]	1.088018059	8.65E-08	2.67E-06
ENSMMUG00000015870	solute carrier family 37 member 3 [Source:VGNC Symbol;Acc:VGNC:81574]	1.61761375	1.84E-05	0.000243341
ENSMMUG00000010420	makorin ring finger protein 1 [Source:VGNC Symbol;Acc:VGNC:81476]	0.733229157	0.010556204	0.042917783
ENSMMUG00000042299	DENN domain containing 2A [Source:VGNC Symbol;Acc:VGNC:81302]	-2.755422269	0.005195701	0.024581771
ENSMMUG00000060893		-1.634773626	0.001343156	0.008418836
ENSMMUG00000064529		-1.022170943	0.0001471	0.001368012
ENSMMUG00000021017	acylglycerol kinase [Source:VGNC Symbol;Acc:VGNC:69669]	1.110406434	1.62E-05	0.000218466
ENSMMUG00000042443	olfactory receptor 9A4 [Source:NCBI gene;Acc:695321]	3.281917153	7.13E-06	0.000108676
ENSMMUG00000055710	taste 2 receptor member 38 [Source:NCBI gene;Acc:695549]	2.307483787	0.000149448	0.001382449
ENSMMUG00000016273	maltase-glucoamylase [Source:VGNC Symbol;Acc:VGNC:74713]	2.933987394	9.39E-13	2.04E-10
ENSMMUG00000049575	T cell receptor beta variable 7-2 [Source:HGNC Symbol;Acc:HGNC:12236]	-1.378176391	8.25E-06	0.000122561
ENSMMUG00000061255		2.259765163	0.001672704	0.010085855
ENSMMUG00000049767		-3.187080436	0.007093222	0.03148616
ENSMMUG00000050269	T cell receptor beta variable 14 [Source:HGNC Symbol;Acc:HGNC:12189]	-1.538119447	1.52E-06	3.00E-05
ENSMMUG00000016286	protease, serine, 1 (trypsin 1) [Source:NCBI gene;Acc:698729]	-0.960467569	0.006245352	0.028410999
ENSMMUG00000051032	serine protease 2 [Source:NCBI gene;Acc:698352]	-1.816418137	5.26E-05	0.000581535
ENSMMUG00000052682		-1.841286131	0.001162709	0.007489945
ENSMMUG00000059019		-1.824820338	0.000612167	0.004420793
ENSMMUG00000058459		-3.007971781	0.002744168	0.014840184
ENSMMUG00000006571	Kell metallo-endopeptidase [Source:VGNC Symbol;Acc:VGNC:81628]	3.479734643	5.87E-08	1.95E-06
ENSMMUG00000012635	zyxin [Source:VGNC Symbol;Acc:VGNC:78917]	1.333499261	1.23E-09	8.32E-08
ENSMMUG00000055322	EPH receptor A1 [Source:NCBI gene;Acc:706295]	1.349136463	1.38E-09	8.98E-08
ENSMMUG00000058370	taste receptor type 2 member 62 [Source:NCBI gene;Acc:704388]	2.828195217	0.004072323	0.020233825
ENSMMUG00000062641		1.149016083	0.00015904	0.001447497
ENSMMUG00000042798		-1.141396084	0.000742516	0.005180878
ENSMMUG00000063823	U6 spliceosomal RNA [Source:RFAM;Acc:RF00026]	-1.843091612	0.012598405	0.049073031
ENSMMUG00000013955	TRPM8 channel associated factor 2 [Source:NCBI gene;Acc:707579]	0.893682713	7.94E-05	0.000824389
ENSMMUG00000057973		-2.394288254	0.000158132	0.001442258
ENSMMUG00000011788	zinc finger protein 212 [Source:VGNC Symbol;Acc:VGNC:78418]	-1.098966722	0.004587224	0.022295008
ENSMMUG00000002497	zinc finger protein 777 [Source:HGNC Symbol;Acc:HGNC:22213]	-1.492286594	1.10E-05	0.000158019

ENSMMUG00000051020	zinc finger protein 467 [Source:NCBI gene;Acc:712106]	-1.640909435	1.59E-06	3.09E-05
ENSMMUG00000048892	SCO-spondin [Source:VGNC Symbol;Acc:VGNC:81765]	-0.778722787	0.003714829	0.018895216
ENSMMUG00000022633	replication initiator 1 [Source:VGNC Symbol;Acc:VGNC:76758]	2.117372776	3.02E-08	1.14E-06
ENSMMUG00000062696		-1.591138884	0.000303914	0.002469855
ENSMMUG00000022636	GTPase, IMAP family member 8 [Source:VGNC Symbol;Acc:VGNC:72981]	-1.17065675	0.001683025	0.010135322
ENSMMUG00000011386	GTPase, IMAP family member 4 [Source:VGNC Symbol;Acc:VGNC:72982]	-2.030992435	8.97E-10	6.55E-08
ENSMMUG00000052830		-1.451179842	0.000516748	0.003836089
ENSMMUG00000047276	GTPase, IMAP family member 6 [Source:VGNC Symbol;Acc:VGNC:81315]	-0.776187145	0.005214709	0.024644805
ENSMMUG00000012238	polypeptide N-acetylgalactosaminyltransferase 11 [Source:VGNC Symbol;Acc:VGNC:72749]	-1.222486929	0.000364292	0.002875541
ENSMMUG00000062591	insulin induced gene 1 [Source:NCBI gene;Acc:718513]	-0.883736897	0.002418681	0.013386114
ENSMMUG00000010940	telomerase reverse transcriptase [Source:VGNC Symbol;Acc:VGNC:78313]	0.651107766	0.000867897	0.005866878
ENSMMUG00000019205	ADAM metallopeptidase with thrombospondin type 1 motif 16 [Source:VGNC Symbol;Acc:VGNC:69439]	-1.189703064	0.009395375	0.039392728
ENSMMUG00000018746	FAST kinase domains 3 [Source:VGNC Symbol;Acc:VGNC:72588]	-1.210059303	0.009014048	0.038100979
ENSMMUG00000018747	5-methyltetrahydrofolate-homocysteine methyltransferase reductase [Source:VGNC Symbol;Acc:VGNC:75079]	-0.969439943	0.001521624	0.009352664
ENSMMUG00000061922		0.703855083	4.12E-06	6.89E-05
ENSMMUG00000021942	ANKH inorganic pyrophosphate transport regulator [Source:VGNC Symbol;Acc:VGNC:69612]	-0.81756643	0.005231502	0.024696487
ENSMMUG00000062083		1.999375574	0.001755902	0.010456623
ENSMMUG00000059036		2.586075031	0.000173612	0.001559389
ENSMMUG00000059710		1.563895882	0.005860353	0.02703787
ENSMMUG00000060891	histone H3.Y [Source:NCBI gene;Acc:718280]	2.098260915	0.003541393	0.018176541
ENSMMUG00000013467	alpha-methylacyl-CoA racemase [Source:NCBI gene;Acc:698365]	1.843798178	8.67E-05	0.000888603
ENSMMUG00000018739	sperm flagellar 2 [Source:VGNC Symbol;Acc:VGNC:82243]	-5.439284728	4.32E-06	7.19E-05
ENSMMUG00000006107	WD repeat domain 70 [Source:VGNC Symbol;Acc:VGNC:79504]	1.426402144	5.55E-07	1.31E-05
ENSMMUG00000004774	maestro heat like repeat family member 2B [Source:VGNC Symbol;Acc:VGNC:74837]	2.374853867	0.00015147	0.001398171
ENSMMUG00000011878	poly(A) binding protein interacting protein 1 [Source:VGNC Symbol;Acc:VGNC:84476]	1.388858544	0.000873066	0.005895685
ENSMMUG00000011734	nicotinamide nucleotide transhydrogenase [Source:VGNC Symbol;Acc:VGNC:75363]	-1.845223842	0.00201714	0.0117032
ENSMMUG00000016149	embigin [Source:VGNC Symbol;Acc:VGNC:72144]	-3.396957952	0.004057237	0.020224222
ENSMMUG00000003984	ISL LIM homeobox 1 [Source:VGNC Symbol;Acc:VGNC:73770]	3.959457639	0.006052504	0.027737444
ENSMMUG00000001753	integrin subunit alpha 1 [Source:VGNC Symbol;Acc:VGNC:73772]	1.713921894	4.20E-08	1.46E-06
ENSMMUG00000039101	pelota mRNA surveillance and ribosome rescue factor [Source:VGNC Symbol;Acc:VGNC:84478]	1.976641329	4.09E-09	2.18E-07
ENSMMUG00000010379	Mtr4 exosome RNA helicase [Source:VGNC Symbol;Acc:VGNC:74989]	-1.067119012	0.000589285	0.004274535
ENSMMUG00000030099	phospholipid phosphatase 1 [Source:VGNC Symbol;Acc:VGNC:76226]	-2.535215212	9.66E-08	2.92E-06
ENSMMUG00000061973		-2.208136759	5.24E-12	9.20E-10

ENSMMUG00000051661		-1.104150826	0.008092744	0.034956568
ENSMMUG00000005181	RAB3C, member RAS oncogene family [Source:VGNC Symbol;Acc:VGNC:81541]	-1.29818527	0.001692315	0.010180503
ENSMMUG00000021812	ERCC excision repair 8, CSA ubiquitin ligase complex subunit [Source:VGNC Symbol;Acc:VGNC:72425]	0.84511215	2.93E-06	5.25E-05
ENSMMUG00000060505		-1.235901484	0.007689271	0.033559283
ENSMMUG00000015041	CWC27 spliceosome associated cyclophilin [Source:VGNC Symbol;Acc:VGNC:71396]	-2.882501242	9.66E-05	0.00096836
ENSMMUG00000051169		-1.850210536	0.007950486	0.034514027
ENSMMUG00000016522	neurolysin [Source:VGNC Symbol;Acc:VGNC:75183]	-0.738583093	0.000101433	0.001007051
ENSMMUG00000038355		-3.836058152	0.010762326	0.043497454
ENSMMUG00000046550	coiled-coil domain containing 125 [Source:NCBI gene;Acc:704391]	1.309750215	1.13E-05	0.000161905
ENSMMUG00000006217	general transcription factor IIH subunit 2 [Source:NCBI gene;Acc:700737]	-1.778827211	0.000172352	0.001550214
ENSMMUG00000011061	ankyrin repeat domain 31 [Source:VGNC Symbol;Acc:VGNC:84277]	0.696600872	0.000103699	0.001023751
ENSMMUG00000022982	IQ motif containing GTPase activating protein 2 [Source:VGNC Symbol;Acc:VGNC:73756]	-2.429788833	0.000114486	0.001112028
ENSMMUG00000006795	dimethylglycine dehydrogenase [Source:VGNC Symbol;Acc:VGNC:84292]	2.710178771	0.001632022	0.009890999
ENSMMUG00000006796	betaine--homocysteine S-methyltransferase 2 [Source:VGNC Symbol;Acc:VGNC:70248]	-1.088697065	0.01174746	0.046652662
ENSMMUG00000003143	thrombospondin 4 [Source:VGNC Symbol;Acc:VGNC:78346]	1.041901278	0.007139369	0.031637012
ENSMMUG00000036820	U7 small nuclear RNA [Source:RFAM;Acc:RF00066]	1.522509934	9.17E-06	0.000134079
ENSMMUG00000039944	Ras protein specific guanine nucleotide releasing factor 2 [Source:VGNC Symbol;Acc:VGNC:84486]	-2.845177714	6.91E-11	7.68E-09
ENSMMUG00000017583	single stranded DNA binding protein 2 [Source:NCBI gene;Acc:711752]	-0.975132243	0.005534222	0.025844917
ENSMMUG00000046560	transmembrane protein 167A [Source:VGNC Symbol;Acc:VGNC:84513]	0.891303483	0.009265966	0.038925439
ENSMMUG00000064265	X-ray repair cross complementing 4 [Source:NCBI gene;Acc:712291]	0.895356765	0.000477412	0.003568514
ENSMMUG00000011513	versican [Source:VGNC Symbol;Acc:VGNC:79445]	3.281916334	2.16E-15	9.05E-13
ENSMMUG00000061282	hyaluronan and proteoglycan link protein 1 [Source:NCBI gene;Acc:712501]	4.42640472	0.006548451	0.029520941
ENSMMUG00000057003	U6 spliceosomal RNA [Source:RFAM;Acc:RF00026]	-0.715934307	0.005806627	0.026847136
ENSMMUG00000009319	transmembrane protein 161B [Source:VGNC Symbol;Acc:VGNC:84508]	-1.264614896	0.001461691	0.009035526
ENSMMUG00000019836	myocyte enhancer factor 2C [Source:VGNC Symbol;Acc:VGNC:84438]	-1.459521896	0.000587464	0.004266083
ENSMMUG00000055392		-1.533983124	1.45E-05	0.000198948
ENSMMUG00000056790		-5.675702763	1.86E-12	3.65E-10
ENSMMUG00000018974	LysM domain containing 3 [Source:VGNC Symbol;Acc:VGNC:74373]	-1.436526608	3.15E-10	2.73E-08
ENSMMUG00000001397	multiple C2 and transmembrane domain containing 1 [Source:VGNC Symbol;Acc:VGNC:74589]	2.146635031	9.00E-11	9.44E-09
ENSMMUG00000010632	family with sequence similarity 81 member B [Source:VGNC Symbol;Acc:VGNC:72365]	3.132354634	0.007421851	0.032677248

ENSMMUG00000001212	Rieske Fe-S domain containing [Source:VGNC Symbol;Acc:VGNC:84487]	-0.544876828	0.00400111	0.020030189
ENSMMUG00000001213	spermatogenesis associated 9 [Source:VGNC Symbol;Acc:VGNC:77976]	-0.935111897	0.000574338	0.004196542
ENSMMUG00000005314	elongation factor for RNA polymerase II 2 [Source:VGNC Symbol;Acc:VGNC:84368]	1.035040926	0.000382766	0.002989945
ENSMMUG00000011043	endoplasmic reticulum aminopeptidase 2 [Source:VGNC Symbol;Acc:VGNC:72141]	0.413336912	0.003881927	0.019546396
ENSMMUG00000054789	family with sequence similarity 174 member A [Source:NCBI gene;Acc:702300]	0.537759278	0.000171213	0.001542104
ENSMMUG00000007773	solute carrier organic anion transporter family member 4C1 [Source:VGNC Symbol;Acc:VGNC:77764]	3.459575514	2.64E-06	4.83E-05
ENSMMUG00000013183	F-box and leucine rich repeat protein 17 [Source:VGNC Symbol;Acc:VGNC:82142]	-0.747754132	0.002126066	0.012154553
ENSMMUG00000038051	uncharacterized LOC106998885 [Source:NCBI gene;Acc:106998885]	-1.651017488	2.26E-09	1.37E-07
ENSMMUG00000011974	calcium/calmodulin dependent protein kinase IV [Source:VGNC Symbol;Acc:VGNC:70526]	-1.700601778	0.000282537	0.002324274
ENSMMUG00000063177	StAR related lipid transfer domain containing 4 [Source:NCBI gene;Acc:706654]	-1.489794578	3.27E-05	0.000395445
ENSMMUG00000012192	erythrocyte membrane protein band 4.1 like 4A [Source:VGNC Symbol;Acc:VGNC:72039]	-1.539737295	1.62E-06	3.15E-05
ENSMMUG00000005018	protein geranylgeranyltransferase type I subunit beta [Source:VGNC Symbol;Acc:VGNC:75965]	3.325606056	0.000157571	0.001439513
ENSMMUG00000015114	toll like receptor adaptor molecule 2 [Source:NCBI gene;Acc:100426353]	0.329112778	0.003473899	0.017893677
ENSMMUG00000060812	autophagy related 12 [Source:NCBI gene;Acc:696765]	0.989884638	6.04E-05	0.000650255
ENSMMUG00000022192		-1.546644395	0.001028699	0.006726624
ENSMMUG00000037127	U6 spliceosomal RNA [Source:RFAM;Acc:RF00026]	-1.64939309	0.010666458	0.043190519
ENSMMUG00000063474		-0.565615332	0.000112348	0.001095348
ENSMMUG00000053794		-1.20067248	0.007021962	0.031233802
ENSMMUG00000046428		-1.069456299	0.000836745	0.005676943
ENSMMUG00000023598	sorting nexin 2 [Source:VGNC Symbol;Acc:VGNC:77682]	2.178575294	0.002476255	0.01362936
ENSMMUG00000006646	casein kinase 1 gamma 3 [Source:VGNC Symbol;Acc:VGNC:81384]	-0.919199529	0.000299248	0.002441669
ENSMMUG00000063396		-5.335602796	0.007035362	0.031272016
ENSMMUG00000011214	solute carrier family 12 member 2 [Source:VGNC Symbol;Acc:VGNC:77386]	-1.457728528	5.64E-05	0.000615508
ENSMMUG00000010682	fibrillin 2 [Source:VGNC Symbol;Acc:VGNC:72624]	-0.951805465	1.39E-06	2.80E-05
ENSMMUG00000016909	meiotic kinetochore factor [Source:NCBI gene;Acc:709179]	0.916721989	7.18E-06	0.000109335
ENSMMUG00000010858	prolyl 4-hydroxylase subunit alpha 2 [Source:VGNC Symbol;Acc:VGNC:75742]	4.146803163	5.82E-12	9.82E-10
ENSMMUG00000051989	interleukin 5 [Source:NCBI gene;Acc:710622]	2.827035396	0.008563577	0.036553292
ENSMMUG00000003131	interleukin 13 [Source:VGNC Symbol;Acc:VGNC:84393]	0.884600258	0.003111733	0.016431669
ENSMMUG00000003133	interleukin 4 [Source:VGNC Symbol;Acc:VGNC:73729]	1.356668974	0.00237333	0.013163126
ENSMMUG00000010313	kinesin family member 3A [Source:VGNC Symbol;Acc:VGNC:74017]	1.111151556	2.83E-06	5.09E-05
ENSMMUG00000010308	cyclin I family member 2 [Source:VGNC Symbol;Acc:VGNC:70923]	1.285654247	1.65E-05	0.000221975
ENSMMUG00000010309	septin 8 [Source:VGNC Symbol;Acc:VGNC:77248]	1.610493997	0.000208426	0.001821769
ENSMMUG00000060221	growth differentiation factor 9 [Source:NCBI gene;Acc:712792]	-0.917533799	0.000381911	0.00298865

ENSMMUG00000015640	AF4/FMR2 family member 4 [Source:VGNC Symbol;Acc:VGNC:69629]	-0.954221149	0.000368457	0.002898977
ENSMMUG00000013047	S-phase kinase associated protein 1 [Source:NCBI gene;Acc:100423634]	-2.850281338	8.87E-25	2.38E-21
ENSMMUG00000016641	ubiquitin conjugating enzyme E2 B [Source:VGNC Symbol;Acc:VGNC:78692]	1.052657157	0.001809492	0.010716871
ENSMMUG00000053384		-2.173312776	4.62E-20	5.46E-17
ENSMMUG00000058096	CDKN2A interacting protein N-terminal like [Source:NCBI gene;Acc:710774]	1.196535191	0.000237124	0.002022318
ENSMMUG00000012463	DEAD-box helicase 46 [Source:VGNC Symbol;Acc:VGNC:71663]	-1.679649919	2.72E-06	4.94E-05
ENSMMUG00000060605		0.828952135	0.003728518	0.018950021
ENSMMUG00000016357	polycystin 2 like 2, transient receptor potential cation channel [Source:VGNC Symbol;Acc:VGNC:76009]	-2.33806641	0.001341962	0.008417207
ENSMMUG00000008009	family with sequence similarity 13 member B [Source:VGNC Symbol;Acc:VGNC:72268]	-2.337971713	2.90E-10	2.53E-08
ENSMMUG00000001507	bromodomain containing 8 [Source:VGNC Symbol;Acc:VGNC:70350]	-2.860380854	0.012480668	0.048789772
ENSMMUG00000016741	matrin 3 [Source:NCBI gene;Acc:100568450]	-1.232422161	0.010266991	0.042149592
ENSMMUG00000001941	CXXC finger protein 5 [Source:VGNC Symbol;Acc:VGNC:71594]	-0.477127527	1.68E-05	0.000224902
ENSMMUG00000028372	Y RNA [Source:RFAM;Acc:RF00019]	-2.729816251	0.005640644	0.026219518
ENSMMUG00000003279	prefoldin subunit 1 [Source:VGNC Symbol;Acc:VGNC:75956]	3.328882704	6.58E-10	5.10E-08
ENSMMUG00000019582	ankyrin repeat and KH domain-containing protein 1 [Source:NCBI gene;Acc:698045]	-0.674868645	7.55E-06	0.000114014
ENSMMUG00000001707	amyloid beta precursor protein binding family B member 3 [Source:VGNC Symbol;Acc:VGNC:69869]	2.389756837	6.45E-09	3.18E-07
ENSMMUG00000001725	solute carrier family 35 member A4 [Source:VGNC Symbol;Acc:VGNC:77532]	1.81936277	3.88E-06	6.56E-05
ENSMMUG00000062326		2.146925689	1.05E-07	3.16E-06
ENSMMUG00000024537		2.881677915	0.001249081	0.007920792
ENSMMUG00000010007	CD14 molecule [Source:VGNC Symbol;Acc:VGNC:70751]	2.1056584	9.19E-06	0.00013421
ENSMMUG00000060632		1.897808585	0.002671802	0.014515228
ENSMMUG00000018246	transmembrane and coiled-coil domains 6 [Source:VGNC Symbol;Acc:VGNC:84507]	2.086151504	6.51E-07	1.51E-05
ENSMMUG00000056796	NADH:ubiquinone oxidoreductase subunit A2 [Source:NCBI gene;Acc:697736]	2.037911154	0.001542715	0.009459946
ENSMMUG00000008710	protocadherin 1 [Source:VGNC Symbol;Acc:VGNC:75775]	-2.303695414	1.56E-05	0.000211482
ENSMMUG00000006287	DAP3 binding cell death enhancer 1 [Source:VGNC Symbol;Acc:VGNC:71806]	0.609355584	0.004153399	0.02058655
ENSMMUG00000006291	protocadherin 12 [Source:VGNC Symbol;Acc:VGNC:75776]	1.024917887	0.000137852	0.001297323
ENSMMUG00000009768	ring finger protein 14 [Source:VGNC Symbol;Acc:VGNC:84488]	0.724951812	0.00074237	0.005180878
ENSMMUG00000009770	glucosamine-6-phosphate deaminase 1 [Source:VGNC Symbol;Acc:VGNC:73101]	0.3461463	0.007537539	0.033074641
ENSMMUG00000060402		2.295485945	2.74E-08	1.05E-06
ENSMMUG00000000419	Rho GTPase activating protein 26 [Source:VGNC Symbol;Acc:VGNC:69846]	1.580122681	0.000141873	0.001327478
ENSMMUG00000050405		1.928031817	2.43E-06	4.49E-05
ENSMMUG00000000421	nuclear receptor subfamily 3 group C member 1 [Source:VGNC Symbol;Acc:VGNC:75515]	1.633617244	8.56E-07	1.88E-05
ENSMMUG00000019820	SH3 domain and tetratricopeptide repeats 2 [Source:VGNC Symbol;Acc:VGNC:77337]	0.934324915	0.000931823	0.006208649

ENSMMUG00000056140	U6 spliceosomal RNA [Source:RFAM;Acc:RF00026]	1.573415911	0.000915185	0.00612289
ENSMMUG00000063983		-2.668993672	0.008555567	0.036538388
ENSMMUG00000001758	casein kinase 1 alpha 1 [Source:VGNC Symbol;Acc:VGNC:84290]	-2.29066335	0.002843222	0.015280555
ENSMMUG00000029776	solute carrier family 26 member 2 [Source:VGNC Symbol;Acc:VGNC:84492]	-0.683595205	0.005229503	0.024696487
ENSMMUG00000011557	platelet derived growth factor receptor beta [Source:VGNC Symbol;Acc:VGNC:75929]	0.6058081	0.000461139	0.003478872
ENSMMUG00000011563	calcium/calmodulin dependent protein kinase II alpha [Source:VGNC Symbol;Acc:VGNC:70523]	4.081050193	0.000147602	0.001371222
ENSMMUG00000005414	N-deacetylase and N-sulfotransferase 1 [Source:VGNC Symbol;Acc:VGNC:75151]	-0.787025312	0.001026679	0.006720173
ENSMMUG00000020679	myozenin 3 [Source:VGNC Symbol;Acc:VGNC:74988]	1.419714958	1.50E-06	2.98E-05
ENSMMUG00000020683	RNA binding motif protein 22 [Source:VGNC Symbol;Acc:VGNC:76535]	1.699603492	2.52E-06	4.64E-05
ENSMMUG00000019764	dynactin subunit 4 [Source:VGNC Symbol;Acc:VGNC:71625]	1.539570173	0.000115199	0.001117288
ENSMMUG00000021051	TNFAIP3 interacting protein 1 [Source:VGNC Symbol;Acc:VGNC:79057]	-0.940596223	0.000909302	0.006092929
ENSMMUG00000021053	annexin A6 [Source:VGNC Symbol;Acc:VGNC:69951]	-1.175673066	2.78E-05	0.000344462
ENSMMUG00000003499	solute carrier family 36 member 1 [Source:VGNC Symbol;Acc:VGNC:77509]	0.912623924	0.012840052	0.049760942
ENSMMUG00000053367	secreted protein acidic and cysteine rich [Source:NCBI gene;Acc:712388]	0.703516057	0.003704331	0.018863953
ENSMMUG00000054302	antioxidant 1 copper chaperone [Source:NCBI gene;Acc:712464]	-1.007518467	0.005525019	0.02582048
ENSMMUG00000056369	SAP30 like [Source:NCBI gene;Acc:714400]	-3.522243734	0.011513463	0.04589154
ENSMMUG00000050350		-3.741554029	0.003854365	0.019430183
ENSMMUG00000002272	clathrin interactor 1 [Source:VGNC Symbol;Acc:VGNC:84289]	1.997401126	0.00157864	0.009639837
ENSMMUG00000059241		-5.024554044	4.71E-09	2.44E-07
ENSMMUG00000003067	endoplasmic reticulum-golgi intermediate compartment 1 [Source:VGNC Symbol;Acc:VGNC:82141]	0.798858194	2.48E-05	0.000313139
ENSMMUG00000004932	msh homeobox 2 [Source:VGNC Symbol;Acc:VGNC:84448]	3.269079033	0.000978421	0.006474706
ENSMMUG00000059262		1.408170432	0.009143321	0.038578877
ENSMMUG00000009059	Fas associated factor family member 2 [Source:VGNC Symbol;Acc:VGNC:72332]	-1.518040023	0.003418559	0.017664593
ENSMMUG00000013283	cadherin related family member 2 [Source:NCBI gene;Acc:697249]	2.039966598	6.13E-07	1.43E-05
ENSMMUG00000013284	G protein regulated inducer of neurite outgrowth 1 [Source:VGNC Symbol;Acc:VGNC:72998]	2.229345089	0.004239338	0.020861456
ENSMMUG00000000137	synuclein beta [Source:VGNC Symbol;Acc:VGNC:77670]	3.846255544	0.002678723	0.014535271
ENSMMUG00000002649	unc-5 netrin receptor A [Source:VGNC Symbol;Acc:VGNC:78721]	-4.430947517	2.99E-05	0.000365809
ENSMMUG00000002653	hexokinase 3 [Source:VGNC Symbol;Acc:VGNC:73476]	-0.913891217	2.49E-06	4.59E-05
ENSMMUG00000009045	RAB24, member RAS oncogene family [Source:VGNC Symbol;Acc:VGNC:81524]	-2.193994554	0.000599976	0.004342396
ENSMMUG00000016192	lectin, mannose binding 2 [Source:VGNC Symbol;Acc:VGNC:74302]	-0.731541304	0.000159279	0.001448661
ENSMMUG00000019807	regulator of G protein signaling 14 [Source:VGNC Symbol;Acc:VGNC:76918]	0.513528782	0.001726918	0.010321829
ENSMMUG00000016914	docking protein 3 [Source:VGNC Symbol;Acc:VGNC:71897]	-0.673928461	0.004369535	0.021388851

ENSMMUG00000016911	DEAD-box helicase 41 [Source:VGNC Symbol;Acc:VGNC:71660]	-1.175934539	0.009327778	0.039147168
ENSMMUG00000004002	CDC like kinase 4 [Source:VGNC Symbol;Acc:VGNC:71173]	1.33945157	3.61E-06	6.24E-05
ENSMMUG00000001728	mitogen-activated protein kinase 9 [Source:VGNC Symbol;Acc:VGNC:74652]	-0.889750224	0.000437321	0.003335948
ENSMMUG00000062522		1.597414375	3.85E-07	9.64E-06
ENSMMUG00000061151		1.588884521	0.000285477	0.002345489
ENSMMUG00000054414		1.314777031	0.000177814	0.001589449
ENSMMUG00000003598	tripartite motif containing 7 [Source:VGNC Symbol;Acc:VGNC:79022]	1.541344081	1.38E-07	3.98E-06
ENSMMUG00000021145	family with sequence similarity 120B [Source:VGNC Symbol;Acc:VGNC:72311]	-1.233940974	2.79E-05	0.000345013
ENSMMUG00000014474	SPARC related modular calcium binding 2 [Source:VGNC Symbol;Acc:VGNC:83470]	-1.008263068	8.41E-05	0.000868042
ENSMMUG00000021044	afadin, adherens junction formation factor [Source:VGNC Symbol;Acc:VGNC:69818]	1.34589715	7.26E-08	2.34E-06
ENSMMUG00000053990		2.039295143	9.16E-07	1.99E-05
ENSMMUG00000062443		-1.086168652	0.000639716	0.00458663
ENSMMUG00000057859		-2.048704515	1.82E-05	0.000240709
ENSMMUG00000057983		4.012098275	0.000236857	0.002021365
ENSMMUG00000054834		0.40200988	0.011651644	0.04634149
ENSMMUG00000050418	superoxide dismutase 2 [Source:NCBI gene;Acc:574097]	3.131347055	2.40E-08	9.53E-07
ENSMMUG00000022247	osteoclastogenesis associated transmembrane protein 1 [Source:VGNC Symbol;Acc:VGNC:75664]	2.373874477	1.69E-09	1.08E-07
ENSMMUG00000008184	methyltransferase like 24 [Source:VGNC Symbol;Acc:VGNC:74552]	1.657727978	4.58E-06	7.57E-05
ENSMMUG00000021534	solute carrier family 16 member 10 [Source:NCBI gene;Acc:696132]	-3.094329107	8.89E-05	0.000908041
ENSMMUG00000061031		4.800339963	2.61E-14	8.47E-12
ENSMMUG00000060903		1.167347224	7.81E-08	2.46E-06
ENSMMUG00000051427		1.055372563	0.0024674	0.013592125
ENSMMUG00000058557		1.657501987	3.40E-05	0.000408589
ENSMMUG00000017189	regulatory factor X6 [Source:VGNC Symbol;Acc:VGNC:76800]	1.779646902	2.55E-05	0.000320754
ENSMMUG00000023737	vestigial like family member 2 [Source:VGNC Symbol;Acc:VGNC:79237]	0.846948331	0.004052663	0.020218234
ENSMMUG00000059327	NUS1 dehydrodolichyl diphosphate synthase subunit [Source:NCBI gene;Acc:715015]	-1.227672194	2.27E-06	4.22E-05
ENSMMUG00000008525	minichromosome maintenance 9 homologous recombination repair factor [Source:VGNC Symbol;Acc:VGNC:83432]	-2.227816892	0.002384353	0.013218624
ENSMMUG00000013302	family with sequence similarity 184 member A [Source:VGNC Symbol;Acc:VGNC:72308]	-2.925951183	0.000445057	0.003381081
ENSMMUG00000060822		-0.87533167	0.001231782	0.007841685
ENSMMUG00000018176	clavesin 2 [Source:VGNC Symbol;Acc:VGNC:71322]	0.762992502	0.00262752	0.014316514
ENSMMUG00000009725	triadin [Source:HGNC Symbol;Acc:HGNC:12261]	0.619931478	0.004192947	0.020727276
ENSMMUG00000015317	HD domain containing 2 [Source:VGNC Symbol;Acc:VGNC:73209]	3.438689359	2.09E-08	8.54E-07
ENSMMUG00000060901		1.045919786	0.000279064	0.002301522
ENSMMUG00000015352	ethylmalonyl-CoA decarboxylase 1 [Source:VGNC Symbol;Acc:VGNC:81063]	4.028430505	1.19E-05	0.000169242
ENSMMUG00000048882	KIAA0408 ortholog [Source:NCBI gene;Acc:100429459]	-0.490917068	0.005745189	0.026614337
ENSMMUG00000022136	laminin subunit alpha 2 [Source:VGNC Symbol;Acc:VGNC:74225]	0.71504648	0.005341992	0.02515494
ENSMMUG00000021498	L3MBTL histone methyl-lysine binding protein 3 [Source:VGNC Symbol;Acc:VGNC:74106]	0.735694888	0.001322545	0.008309674

ENSMMUG00000021499	sterile alpha motif domain containing 3 [Source:VGNC Symbol;Acc:VGNC:77079]	-1.40789959	8.45E-06	0.000124932
ENSMMUG00000017544	mediator complex subunit 23 [Source:VGNC Symbol;Acc:VGNC:74562]	0.81414296	8.26E-05	0.000855962
ENSMMUG00000004996	monooxygenase DBH like 1 [Source:VGNC Symbol;Acc:VGNC:74800]	1.099308559	0.000187904	0.001669678
ENSMMUG00000004988	trace amine-associated receptor 3 [Source:NCBI gene;Acc:709130]	-0.931362046	0.006415589	0.029043211
ENSMMUG00000004987	trace amine associated receptor 2 [Source:VGNC Symbol;Acc:VGNC:83475]	-1.167515036	0.009457712	0.039584662
ENSMMUG00000015088	trace amine associated receptor 1 [Source:VGNC Symbol;Acc:VGNC:83474]	-2.862759628	0.009548165	0.039866272
ENSMMUG00000054573		1.149646115	0.000242202	0.002057532
ENSMMUG00000005745	EYA transcriptional coactivator and phosphatase 4 [Source:VGNC Symbol;Acc:VGNC:72456]	1.642486117	2.57E-07	6.83E-06
ENSMMUG00000022921	solute carrier family 2 member 12 [Source:VGNC Symbol;Acc:VGNC:77503]	1.532998315	5.88E-05	0.000636258
ENSMMUG00000061787		2.546825348	0.003179702	0.016729449
ENSMMUG00000018166	MYB proto-oncogene, transcription factor [Source:VGNC Symbol;Acc:VGNC:75089]	0.93926177	0.001091388	0.007086667
ENSMMUG00000009368	Abelson helper integration site 1 [Source:VGNC Symbol;Acc:VGNC:81010]	1.394441408	9.32E-08	2.83E-06
ENSMMUG00000044284	40S ribosomal protein S11-like [Source:NCBI gene;Acc:706532]	1.074927835	0.004221283	0.02081101
ENSMMUG00000016376	mitogen-activated protein kinase kinase kinase 5 [Source:VGNC Symbol;Acc:VGNC:74495]	1.145088674	0.00091916	0.006143161
ENSMMUG00000019136	taxilin beta [Source:VGNC Symbol;Acc:VGNC:79325]	0.407663221	0.005619187	0.026138464
ENSMMUG00000058187		1.563412134	2.67E-06	4.85E-05
ENSMMUG00000023012	vesicle trafficking 1 [Source:VGNC Symbol;Acc:VGNC:79296]	-2.095715044	0.000105104	0.001035589
ENSMMUG00000044958	adhesion G protein-coupled receptor G6 [Source:VGNC Symbol;Acc:VGNC:81009]	-1.911191705	2.58E-07	6.84E-06
ENSMMUG00000061117		-1.443633691	0.000118116	0.001138931
ENSMMUG00000003750	PLAG1 like zinc finger 1 [Source:VGNC Symbol;Acc:VGNC:83444]	1.669052414	3.63E-05	0.000429089
ENSMMUG00000023265	A-kinase anchoring protein 12 [Source:VGNC Symbol;Acc:VGNC:69766]	-2.167272867	2.89E-07	7.49E-06
ENSMMUG00000059202	Y RNA [Source:RFAM;Acc:RF00019]	-3.678051262	0.002653629	0.014428551
ENSMMUG00000029475	zinc finger and BTB domain containing 2 [Source:VGNC Symbol;Acc:VGNC:79291]	-2.238360754	3.86E-05	0.000448967
ENSMMUG00000018342	required for meiotic nuclear division 1 homolog [Source:VGNC Symbol;Acc:VGNC:76797]	-2.364281712	2.68E-08	1.03E-06
ENSMMUG00000060940	acidic residue methyltransferase 1 [Source:NCBI gene;Acc:704570]	-4.383493399	1.63E-05	0.000219692
ENSMMUG00000018346	coiled-coil domain containing 170 [Source:VGNC Symbol;Acc:VGNC:70832]	-2.339568817	0.000179162	0.001599304
ENSMMUG00000025949	U6 spliceosomal RNA [Source:RFAM;Acc:RF00026]	-3.212381595	0.010130175	0.04174685
ENSMMUG00000019045	estrogen receptor 1 [Source:VGNC Symbol;Acc:VGNC:72432]	-1.179297512	0.000203974	0.001786452
ENSMMUG00000057697		-1.761869179	8.32E-07	1.84E-05
ENSMMUG00000051522	Metazoan signal recognition particle RNA [Source:RFAM;Acc:RF00017]	-3.574523937	0.000833964	0.00566695
ENSMMUG00000051271		-3.054486121	1.15E-11	1.69E-09

ENSMMUG00000000808	melanin concentrating hormone receptor 2 [Source:VGNC Symbol;Acc:VGNC:74550]	0.467915526	0.002230353	0.012584805
ENSMMUG00000005925	PNN interacting serine and arginine rich protein [Source:VGNC Symbol;Acc:VGNC:76090]	-2.332673129	0.004186324	0.020710279
ENSMMUG00000005923	failed axon connections homolog, metaxin like GST domain containing [Source:VGNC Symbol;Acc:VGNC:72541]	-1.395003803	0.001750433	0.01044318
ENSMMUG00000009182	caspase 8 associated protein 2 [Source:VGNC Symbol;Acc:VGNC:70548]	4.470023174	0.000472379	0.003536997
ENSMMUG00000003462	ankyrin repeat domain 6 [Source:VGNC Symbol;Acc:VGNC:81023]	-0.716123555	0.001718559	0.010288517
ENSMMUG00000064235		0.621799396	0.000428777	0.003282329
ENSMMUG00000017039	arginyl-tRNA synthetase 2, mitochondrial [Source:VGNC Symbol;Acc:VGNC:76516]	0.77463338	0.000110732	0.001082032
ENSMMUG00000005446	sorting nexin 14 [Source:VGNC Symbol;Acc:VGNC:77708]	1.631026798	1.67E-05	0.000223476
ENSMMUG00000064804		-0.985608557	0.006251714	0.028429996
ENSMMUG00000005442	5'-nucleotidase ecto [Source:VGNC Symbol;Acc:VGNC:75537]	-0.88424562	0.002265399	0.012749364
ENSMMUG00000017186	pleckstrin homology domain interacting protein [Source:VGNC Symbol;Acc:VGNC:75803]	-1.895934806	3.92E-06	6.61E-05
ENSMMUG00000004491	SUMO specific peptidase 6 [Source:VGNC Symbol;Acc:VGNC:77287]	-0.455188362	0.003920183	0.019678079
ENSMMUG00000019261	collagen type XII alpha 1 chain [Source:VGNC Symbol;Acc:VGNC:71291]	-0.3071268	0.006347279	0.028774036
ENSMMUG00000062339	endogenous retrovirus group K member 25 Env polyprotein-like [Source:NCBI gene;Acc:114677579]	-0.774841952	0.006096772	0.027910812
ENSMMUG00000002777	protein tyrosine phosphatase 4A1 [Source:VGNC Symbol;Acc:VGNC:83452]	-0.716484903	0.006030277	0.027645324
ENSMMUG00000011858	bone morphogenetic protein 5 [Source:VGNC Symbol;Acc:VGNC:70264]	1.6388639	5.28E-05	0.000582951
ENSMMUG00000012781	muscular LMNA interacting protein [Source:VGNC Symbol;Acc:VGNC:83434]	-1.017646102	0.004821884	0.023184261
ENSMMUG00000011049	leucine rich repeat containing 1 [Source:VGNC Symbol;Acc:VGNC:81087]	-2.601824569	6.24E-08	2.03E-06
ENSMMUG00000013718	translocation associated membrane protein 2 [Source:VGNC Symbol;Acc:VGNC:83515]	-1.046649353	0.000157762	0.001439898
ENSMMUG00000052626	progestin and adipoQ receptor family member 8 [Source:NCBI gene;Acc:708418]	0.598110173	0.001668111	0.010067495
ENSMMUG00000016993	PKHD1 ciliary IPT domain containing fibrocystin/polyductin [Source:VGNC Symbol;Acc:VGNC:76010]	-1.978137509	2.77E-07	7.21E-06
ENSMMUG00000053984		1.885068486	0.009058623	0.038264517
ENSMMUG00000019254	phospholipase A2 group VII [Source:VGNC Symbol;Acc:VGNC:76158]	-0.92822127	0.000278271	0.002296439
ENSMMUG00000019250	solute carrier family 25 member 27 [Source:VGNC Symbol;Acc:VGNC:77543]	-0.526268012	0.004062912	0.020226065
ENSMMUG00000055199	cytochrome P450, family 39, subfamily A, polypeptide 1 [Source:NCBI gene;Acc:704242]	1.676001077	0.000438178	0.00334006
ENSMMUG00000019246	regulator of calcineurin 2 [Source:VGNC Symbol;Acc:VGNC:82092]	2.467145059	2.34E-07	6.32E-06
ENSMMUG00000062538	adenylate cyclase type 10 [Source:NCBI gene;Acc:719662]	-1.189456579	0.001461226	0.009035526
ENSMMUG00000021173	nuclear transcription factor Y subunit alpha [Source:VGNC Symbol;Acc:VGNC:75344]	-0.826177397	0.001419027	0.008822114
ENSMMUG00000021169	translocator protein 2 [Source:VGNC Symbol;Acc:VGNC:83960]	-1.197485353	2.53E-05	0.000318566
ENSMMUG00000021168	unc-5 family C-terminal like [Source:VGNC Symbol;Acc:VGNC:79314]	-1.149577478	8.20E-12	1.30E-09

ENSMMUG00000013648	potassium two pore domain channel subfamily K member 16 [Source:VGNC Symbol;Acc:VGNC:73827]	3.324398127	0.00610914	0.027936937
ENSMMUG00000013644	potassium two pore domain channel subfamily K member 17 [Source:VGNC Symbol;Acc:VGNC:73828]	2.780249806	5.96E-08	1.97E-06
ENSMMUG00000023381	dynein axonemal heavy chain 8 [Source:VGNC Symbol;Acc:VGNC:71943]	0.644411972	3.80E-05	0.000445262
ENSMMUG00000048502	Metazoan signal recognition particle RNA [Source:RFAM;Acc:RF00017]	1.274065122	0.000402635	0.003118925
ENSMMUG00000062055	glyoxalase I [Source:NCBI gene;Acc:719518]	0.962947333	1.35E-09	8.90E-08
ENSMMUG00000063198		2.904744981	0.001299249	0.008187029
ENSMMUG00000015416	bromodomain and PHD finger containing 3 [Source:VGNC Symbol;Acc:VGNC:70309]	0.997304372	9.59E-05	0.000962154
ENSMMUG00000023823	Y RNA [Source:RFAM;Acc:RF00019]	4.242169476	0.009459413	0.039584662
ENSMMUG00000000869	small nuclear ribonucleoprotein polypeptide C [Source:NCBI gene;Acc:718654]	4.956247641	1.57E-05	0.000212857
ENSMMUG00000063472		5.243707003	3.86E-05	0.000448967
ENSMMUG00000004287	death domain associated protein [Source:VGNC Symbol;Acc:VGNC:71737]	-1.013165873	0.004460262	0.021800139
ENSMMUG00000003864	VPS52 subunit of GARP complex [Source:VGNC Symbol;Acc:VGNC:78551]	-0.875088878	0.000144313	0.001345471
ENSMMUG00000003858	retinoid X receptor beta [Source:VGNC Symbol;Acc:VGNC:76952]	-0.900741592	0.005019351	0.023939009
ENSMMUG00000023138	bromodomain containing 2 [Source:VGNC Symbol;Acc:VGNC:83422]	-1.805265584	0.009172746	0.038633659
ENSMMUG00000022626	major histocompatibility complex, class II, DM alpha [Source:NCBI gene;Acc:717870]	-1.730071299	0.004880525	0.023405643
ENSMMUG00000006828	valyl-tRNA synthetase 1 [Source:VGNC Symbol;Acc:VGNC:78755]	-2.056836187	0.010263471	0.042149259
ENSMMUG00000009702	mutS homolog 5 [Source:NCBI gene;Acc:715494]	-1.878160771	0.006484408	0.029252934
ENSMMUG00000058534		3.162866287	5.04E-07	1.21E-05
ENSMMUG00000015693	lymphocyte antigen 6 family member G6C [Source:VGNC Symbol;Acc:VGNC:74285]	2.452569104	0.000169922	0.001532598
ENSMMUG00000053324	lymphocyte antigen 6 complex locus protein G6d [Source:NCBI gene;Acc:715304]	2.436098907	3.87E-08	1.38E-06
ENSMMUG00000015686	abhydrolase domain containing 16A [Source:VGNC Symbol;Acc:VGNC:69393]	2.412050414	1.99E-10	1.90E-08
ENSMMUG00000054159	lymphocyte antigen 6 family member G5C [Source:VGNC Symbol;Acc:VGNC:83431]	3.044397866	0.000320606	0.002575543
ENSMMUG00000005294	casein kinase 2 beta [Source:NCBI gene;Acc:715807]	2.563048117	2.97E-06	5.29E-05
ENSMMUG00000005293	G-patch domain and ankyrin repeats 1 [Source:VGNC Symbol;Acc:VGNC:81073]	2.766144604	3.43E-06	5.98E-05
ENSMMUG00000005302	BAG cochaperone 6 [Source:VGNC Symbol;Acc:VGNC:81044]	2.048739329	1.26E-08	5.52E-07
ENSMMUG00000005303	apolipoprotein M [Source:VGNC Symbol;Acc:VGNC:69825]	2.883297313	6.50E-05	0.000694869
ENSMMUG00000005297	proline rich coiled-coil 2A [Source:VGNC Symbol;Acc:VGNC:83448]	2.004872666	6.09E-08	1.99E-06
ENSMMUG00000025918	small nucleolar RNA, H/ACA box 38 [Source:HGNC Symbol;Acc:HGNC:32631]	2.750835382	1.46E-05	0.000199587
ENSMMUG00000049735	allograft inflammatory factor 1 [Source:NCBI gene;Acc:574115]	1.829605578	7.53E-08	2.41E-06
ENSMMUG00000008854	natural cytotoxicity triggering receptor 3 [Source:VGNC Symbol;Acc:VGNC:74990]	2.100014263	6.05E-07	1.42E-05
ENSMMUG00000052422	ATPase H+ transporting V1 subunit G2 [Source:NCBI gene;Acc:715331]	4.291982076	0.010173073	0.041871925
ENSMMUG00000008839	DExD-box helicase 39B [Source:VGNC Symbol;Acc:VGNC:71621]	1.791453654	0.010438844	0.042560378
ENSMMUG00000062264		-1.412649257	0.010380503	0.042400013

ENSMMUG00000037625		-1.400050062	0.001954395	0.011390094
ENSMMUG00000053561		4.460056689	7.86E-05	0.000817053
ENSMMUG00000029883	olfactory receptor, family 2, subfamily J, member 3 [Source:NCBI gene;Acc:708943]	4.095458434	9.77E-06	0.00014128
ENSMMUG00000059442		3.305726983	5.48E-05	0.000601309
ENSMMUG00000005415	olfactory receptor, family 2, subfamily B, member 3 [Source:NCBI gene;Acc:708747]	3.067929611	0.000211944	0.001847552
ENSMMUG00000004377	olfactory receptor, family 2, subfamily W, member 1 [Source:NCBI gene;Acc:708515]	4.19206824	0.011589169	0.046150867
ENSMMUG00000004617	glutathione peroxidase 6 [Source:VGNC Symbol;Acc:VGNC:73154]	-1.241375556	0.002733848	0.014796684
ENSMMUG00000060483		1.270663871	4.32E-06	7.19E-05
ENSMMUG00000012396	zinc finger and SCAN domain containing 23 [Source:VGNC Symbol;Acc:VGNC:79313]	-0.819239889	0.001448115	0.008964393
ENSMMUG00000011675	zinc finger and SCAN domain containing 12 [Source:VGNC Symbol;Acc:VGNC:78910]	-1.016727925	5.99E-06	9.43E-05
ENSMMUG00000005964	CMP-N-acetylneuraminic acid hydroxylase [Source:NCBI gene;Acc:574186]	1.662625784	1.96E-05	0.000256983
ENSMMUG00000049021	Metazoan signal recognition particle RNA [Source:RFAM;Acc:RF00017]	2.334458823	0.0001552	0.001424509
ENSMMUG00000058151	chromosome 4 C6orf62 homolog [Source:NCBI gene;Acc:708218]	-1.310838693	3.35E-06	5.88E-05
ENSMMUG00000029939	acyl-CoA thioesterase 13 [Source:VGNC Symbol;Acc:VGNC:69487]	-2.960195044	0.006313875	0.028662569
ENSMMUG00000051037		2.153247714	0.006200266	0.028265217
ENSMMUG00000052442		-0.742189218	0.009186534	0.038659351
ENSMMUG00000062089	ring finger protein 144B [Source:NCBI gene;Acc:705099]	-1.180853632	0.005708953	0.026461381
ENSMMUG00000052333		0.765856973	0.0031251	0.016488866
ENSMMUG00000052891		1.72148266	0.000995299	0.006566364
ENSMMUG00000020289	CD83 molecule [Source:VGNC Symbol;Acc:VGNC:70783]	-1.739932825	4.09E-06	6.85E-05
ENSMMUG00000020122	RAN binding protein 9 [Source:VGNC Symbol;Acc:VGNC:76475]	-0.77423923	0.002783039	0.015012945
ENSMMUG00000004347	androgen dependent TFPI regulating protein [Source:VGNC Symbol;Acc:VGNC:69661]	1.610982871	3.84E-05	0.000448418
ENSMMUG00000058800		1.101169154	0.000484482	0.00361513
ENSMMUG00000043161	endogenous retrovirus group FRD member 1, envelope [Source:VGNC Symbol;Acc:VGNC:81068]	-1.46903795	0.000136798	0.001290205
ENSMMUG00000049313	Metazoan signal recognition particle RNA [Source:RFAM;Acc:RF00017]	-1.801308678	0.003753299	0.019031349
ENSMMUG00000050417		-1.448676668	1.02E-08	4.67E-07
ENSMMUG00000018787	PAK1 interacting protein 1 [Source:VGNC Symbol;Acc:VGNC:75661]	1.252383315	1.12E-05	0.000159877
ENSMMUG00000061586	chromosome 4 C6orf52 homolog [Source:NCBI gene;Acc:702114]	0.984539623	0.001480703	0.009135684
ENSMMUG00000038560	eukaryotic translation elongation factor 1 epsilon 1 [Source:NCBI gene;Acc:695426]	-0.997583926	0.000366453	0.002890292
ENSMMUG00000060973		0.816183794	0.003298304	0.01722801
ENSMMUG00000055919		-2.012964865	0.009217587	0.038747232
ENSMMUG00000023558	exocyst complex component 2 [Source:VGNC Symbol;Acc:VGNC:72338]	-2.075904883	6.74E-05	0.000716812
ENSMMUG00000058002	zinc finger protein 669-like [Source:NCBI gene;Acc:696282]	1.196252383	4.10E-05	0.000471215

ENSMMUG00000065031		-0.851460837	0.005658004	0.026285197
ENSMMUG00000019483	putative golgin subfamily A member 8D [Source:NCBI gene;Acc:100430266]	-3.934901767	0.001291577	0.008146582
ENSMMUG00000055117		-2.196230459	2.91E-05	0.000357438
ENSMMUG00000005270	ryanodine receptor 3 [Source:NCBI gene;Acc:694996]	0.482124604	0.002403203	0.013311777
ENSMMUG00000018332	family with sequence similarity 98 member B [Source:VGNC Symbol;Acc:VGNC:72583]	-1.231641423	0.000397525	0.003085808
ENSMMUG00000020534	p21 (RAC1) activated kinase 6 [Source:NCBI gene;Acc:701589]	1.323567357	2.34E-06	4.33E-05
ENSMMUG00000020535	phospholipase C beta 2 [Source:VGNC Symbol;Acc:VGNC:76164]	1.446353555	6.32E-05	0.000677179
ENSMMUG00000012408	dispatched RND transporter family member 2 [Source:VGNC Symbol;Acc:VGNC:71676]	-1.182238659	3.73E-05	0.000439546
ENSMMUG00000058981	kinetochore localized astrin (SPAG5) binding protein [Source:NCBI gene;Acc:702753]	-0.963976802	0.000142179	0.001328432
ENSMMUG00000043229	carbohydrate sulfotransferase 14 [Source:NCBI gene;Acc:703115]	-1.200268623	0.005708596	0.026461381
ENSMMUG00000001482	coiled-coil domain containing 32 [Source:VGNC Symbol;Acc:VGNC:70891]	1.597259444	6.57E-06	0.000101468
ENSMMUG00000001483	RNA pseudouridine synthase domain containing 2 [Source:VGNC Symbol;Acc:VGNC:77091]	1.620326401	0.000201093	0.001768367
ENSMMUG00000048910	kinetochore scaffold 1 [Source:VGNC Symbol;Acc:VGNC:84366]	0.669729682	0.002589592	0.014139476
ENSMMUG00000010168	MAX dimerization protein MGA [Source:VGNC Symbol;Acc:VGNC:84390]	-0.396344665	0.00700743	0.031190499
ENSMMUG00000001532	glucosidase alpha, neutral C [Source:NCBI gene;Acc:708126]	0.739870646	0.000109794	0.001075286
ENSMMUG00000001534	calpain 3 [Source:VGNC Symbol;Acc:VGNC:70533]	0.743183308	0.008877997	0.037574756
[truncated: 423,796 more chars]
